# A Quantitative Structure-Activity Relationship for Human Plasma Protein Binding: Prediction, Validation and Applicability Domain

**DOI:** 10.34172/apb.2023.078

**Published:** 2023-04-29

**Authors:** Affaf Khaouane, Samira Ferhat, Salah Hanini

**Affiliations:** Laboratory of Biomaterial and transport Phenomena (LBMPT), University of Médéa, pole urbain, 26000, Médéa, Algeria.

**Keywords:** Quantitative structure-activity relationship, Artificial neural network, Prediction, Protein-binding

## Abstract

**Purpose:**

The purpose of this study was to develop a robust and externally predictive in silico QSAR-neural network model for predicting plasma protein binding of drugs. This model aims to enhance drug discovery processes by reducing the need for chemical synthesis and extensive laboratory testing.

**Methods:**

A dataset of 277 drugs was used to develop the QSAR-neural network model. The model was constructed using a Filter method to select 55 molecular descriptors. The validation set’s external accuracy was assessed through the predictive squared correlation coefficient Q2 and the root mean squared error (RMSE).

**Results:**

The developed QSAR-neural network model demonstrated robustness and good applicability domain. The external accuracy of the validation set was high, with a predictive squared correlation coefficient Q2 of 0.966 and a root mean squared error (RMSE) of 0.063. Comparatively, this model outperformed previously published models in the literature.

**Conclusion:**

The study successfully developed an advanced QSAR-neural network model capable of predicting plasma protein binding in human plasma for a diverse set of 277 drugs. This model’s accuracy and robustness make it a valuable tool in drug discovery, potentially reducing the need for resource-intensive chemical synthesis and laboratory testing.

## Introduction

 Many drugs interact with plasma or other molecules, such as DNA, to form a drug-molecule complex. The process is called protein binding, more specifically the binding of drugs to proteins. The bond drug remains in the bloodstream while the unbound component can be metabolized or excreted to become the active component.^1^ In short, protein-binding process is defined as the formation of complexes: hydrogen bonding, hydrophilic bonding, ionic bonding, Vander Walls bonding, and covalent bonding.

 The binding of drugs to proteins can be reversible or irreversible.^2,3^ Irreversible drug-protein binding is the result of chemical activation of a drug tightly binding to a protein or macromolecule through a covalent chemical bond. Irreversible drug binding is responsible for some types of drug toxicity that can occur over a long period of time.^4^ Reversible drug- protein binding means that the drug binds to weaker chemical bound, such as hydrogen bonds or Vander Waals forces. At low drug concentrations, most of the drug is bound to the protein, while at high drug concentrations, the protein is bound to the sites to saturate, leading to a rapid increase in the free drug concentration. Therefore plasma protein binding plays a key role in drug therapy as it affects the pharmacokinetics and pharmacodynamics of the drug as it is often directly related to the concentration of free drug in plasma.^5,6^

 The construction of in silico models that establish a mathematical relationship between the molecular structure and the properties of interest is an important step in drug discovery as it avoids chemical synthesis and expansive and lengthy ones laboratory tests reduced.^7,8^

 In recent years, several QSAR models have been developed to predict plasma protein binding and powerful plasma protein binding prediction algorithms are used, such as support vector machines and their derivatives,^9-11^ the random forest,^12^ neural networks,^13,14^ and gradient boosting decision trees.^15^ In 2017, Sun et al constructed QSAR models using six machine-learning algorithms with 26 molecular descriptors.^16^ Kumar et al presented in 2018 a systematic approach using support vector machine, artificial neural network, K-nearest neighbor, probabilistic neural network, partial least square, and linear discriminant analysis for a diverse dataset of 735 remdies.^17^ Yuan et al. published a global quantitative structure-activity relationships (QSAR) model for plasma protein-binding in 2020, and developed a novel strategy to construct a robust QSAR model for predicting plasma protein-binding.^18^ Altae-Tran et al introduced deep–learning healthcare techniques successfully predicting drug activity and structure.^19^ Wallach and his co-authors introduced AtomNet, known as the first structure-based deep convolutional neural network, to predict small molecule bioactivity for drug discovery applications.^20^

 This work uses a systematic methodology based on QSAR, Filter method, and feed-forward neural network (FFNN) to predict plasma protein binding for 277 molecules. Filter method, known as the most popular feature selection technique, was used to reduce the descriptors. A feed forward neural network was then used to predict plasma protein-binding from the extracted descriptors.

## Materials and Methods

**Figure 1:**
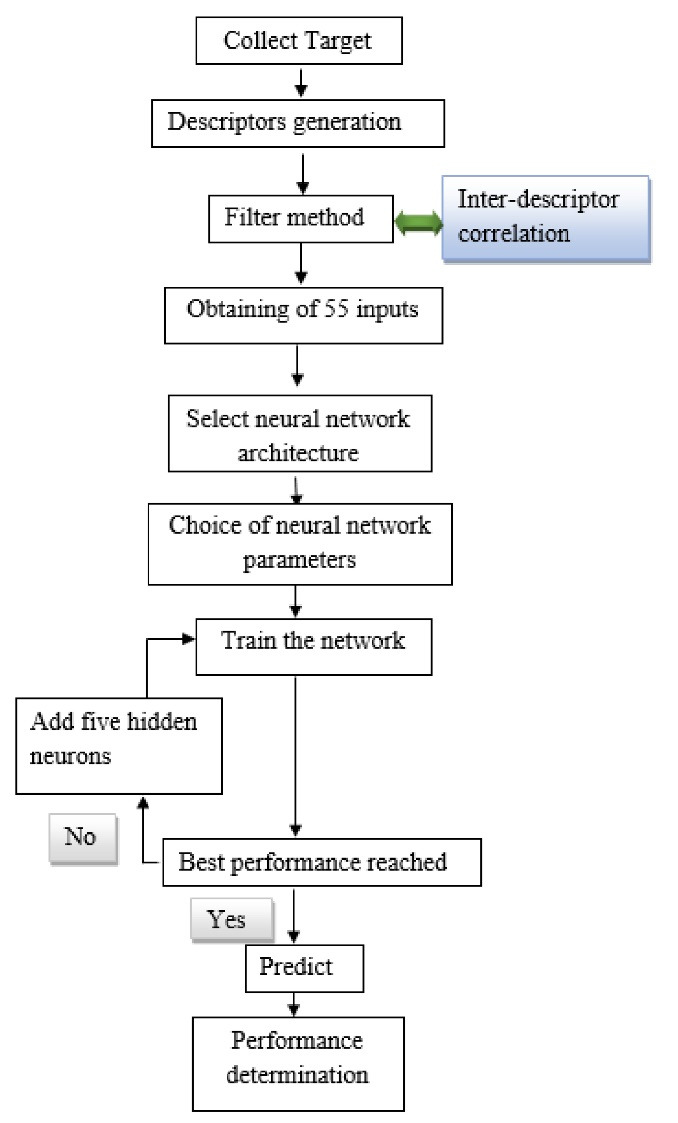
Flow sheet of the procedure followed

 A five-step process was employed to predict the plasma protein-binding, as shown in Figure 1: (1) data set collection, (2) molecular descriptors generation, (3) selection of relevant descriptors by a filter method, (4) FFNN modeling, (5) validation of models.

###  Data set collection

 The experimental data values of protein-binding of the 277 drugs used in this study were selected from the pharmacological basis of the therapeutics handbook^21^ and the handbook of clinical drug data.^22^ Chemical names and experimental protein-binding values are presented in Supplementary file 1. This dataset was divided into two parts. The first one with 235 plasma protein-binding values, dedicated to develop the QSAR model. The second included 42 elements left for the external validation. The data was partitioned using holdout cross-validation.

###  Molecular descriptors generation

 The numerical representation of molecular structure was assessed in terms of molecular descriptors; The SMILES script (simplified molecular input line-entry system) required to calculate descriptors was extracted from the open-access database PubChem.^23^ SMILES is a standard for specifying the structure of chemical species that takes the form of a line notation.^24^ Table 1 lists 1666 descriptors that were sorted into twenty categories using the SMILES scripts for the 277 drugs. The E-Dragon online programs,^25^ also known as the electronic remote version of the well-known software DRAGON created by the Milano Chemometrics and QSAR Research Group by Prof. R. Todeschini, were used to collect all descriptors. In Supplementary file 2, the name and number of calculated descriptors are presented.

**Table 1:** Number of calculated descriptors and their categories

**Descriptors category**	**Number**
Constitutional descriptors	48
Topological descriptors	119
Walk and pathcounts	47
Connectivity indices	33
Information indices	47
2D autocorrelations	96
Edgeadjacency indices	107
Burdeneigen value descriptors	64
Topological charge indices	21
Eigen value based indices	44
Randic molecular profiles	41
Geometrical descriptors	74
RDF descriptors	150
3D-morse descriptors	160
WHIM descriptors	99
GETAWAY descriptors	197
Functional group counts	154
Atomcentred fragments	120
Charge descriptors	14
Molecularproperties	31
**Total**	**1666**

###  Selection of relevant descriptors

 Feature selection techniques are applied to decrease the number of elements in the dataset by choosing features that will give us better accuracy with less data.^26-28^ It also reduces the overfitting and the overtraining risk.^29^ Feature selection methods are widely available in the literature. The characteristics, advantages, and disadvantages of the three main strategies that can be used for the selection of relevant descriptors are reported in Table 2.^30^

**Table 2:** Feature selection methods and their advantages and disadvantages

**Feature selection with filter methods**	**Feature selection with wrapper methods**	**Feature selection with embedded methods**
Relevance of the features is calculated by considering the intrinsic properties of the data.	Wrapper methods select a subset of relevant features using a learning algorithm.	Includes the classifier construction for the optimal feature selection.
Use feature relevance score to select the top rank features.	Conduct search in the space of possible parameters.	Like wrapper approaches, these methods are specific to a given learning algorithm.
**Examples**	**Examples**	**Examples**
Information gain Correlation coefficient scores Chi squared test T-test	Genetic search Sequential forward selection Sequential backward elimination	Decision tree Weighted Naive Bayes SVM
**Advantages**	**Advantages**	**Advantages**
Can scale to high-dimensional data sets Fast and computationally inexpensive in comparison to wrapper method	Considers features dependencies Interaction with classifier Simple to implement	Classifier interaction Considers feature dependencies
**Disadvantages**	**Disadvantages**	**Disadvantages**
No interaction with the classifier Univariate feature selection methods do not consider feature dependencies/ redundancy	Higher risk of overfitting Selection based on classifier Computationally intensive	Classifier dependencies

 The following procedure was used to reduce the number of molecular descriptors^31^:

1. Descriptors having constant values (min = max) were eliminated.
2. Quasi-constant descriptors (1^st^ quartile 25% = 2^nd^ quartile 75%) were removed.
3. Descriptors with standard relative deviation RSD < 0.05 were deleted.

The three steps above were performed using STATISTICA software.^32^

4. Matrices of the pairwise linear correlation between each pair of the column in the input matrices were calculated via MATLAB.^33^ Additionally, every variable that has a correlation coefficient *R* > 0.75 were removed. For more robustness of the model, the variance inflation factor *VIF *whose equation is as follows was calculated:

(1) $$VIF_{i}=\frac{1}{1-R_{i}^{2}}$$

Where $R_{i}^{2}$ is the squared correlation coefficient between the ith descriptor and the others. All descriptors with *VIF* > 5 were eliminated from the model.^34^

###  Model development

 For the purpose of predicting the plasma protein-binding, the selected descriptors were used as inputs in FFNN. There are different approaches to discover the number of hidden neurons required for a modeling task explained in detail in a review named methods of selecting the number of hidden nodes in Artificial Neural Networks review.^35^ In this work, the following steps were used to choose the number of neurons in the hidden layer^36^:

1. Initially, only five hidden neurons were taken.
2. The FFNN is trained until the mean square error does no longer seem to improve.
3. At this moment, five neurons are added to the hidden layer, each with randomly initialized weights, and resumed training.
4. The steps 2 and 3 are repeated until a termination criterion has been satisfied.

The mathematical equation of the model used for the prediction of protein binding is:


(2) $$fb=\sum_{j=1}^{k}w2j(\frac{\exp(\sum_{i=1}^{p}xi+wij+bj)-\exp(-\sum_{i=1}^{p}xi+wij+bj)}{\exp(\sum_{i=1}^{p}xi+wij+bj)+\exp(-\sum_{i=1}^{p}xi+wij+bj)})+b$$

*x*i (*i = 1…p*) is the input that corresponds to the number of data included in the training of the ANN, *i *from 1 to 15, *wij(i = 1…p, j = 1…k*) are weights from input to hidden layer*, b j (j = 1…k)* are biases of the neurons in the hidden layer, *k = 40* for filter method,* w2j(j = 1…k*) are weights from the hidden to the output layer*, b* is the bias of the output neuron and *fb* is the output.

###  Model validation

 We established internal and external validation criteria to assess the QSAR models’ generalizability and predictive power. The following statistical parameters were used in our investigation to evaluate the models’ efficacy: the mean squared error (MSE), correlation coefficient (*R*), predictive squared correlation coefficient (*Q*^2^), and coefficient of determination (*R*^2^) values.


(3) $$R^{2}=1-\frac{RSS}{SS}$$

(4) $$MSE=\sum_{i=1}^{n}\frac{{\left(y_{i}^{pred}-y_{i}\right)}^{2}}{n}$$

(5) $$Q=-\frac{PRESS}{SS}$$

The residual sum of squares (RSS) is the difference between the fitted values and the observed values. The sum of squares (SS) refers to the difference between the observation and their mean. The PREdictive residual SS (PRESS) is the difference between the predictions and the observations.

## Results and Discussion

**Figure 2:**
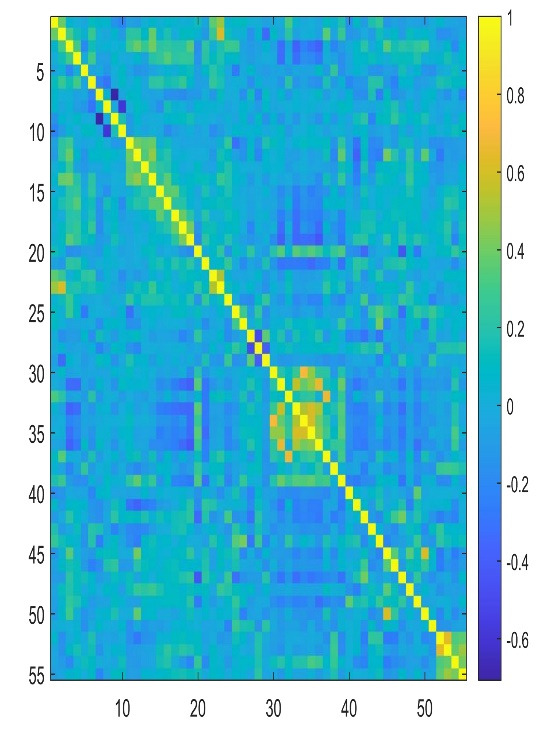
Heatmap of the correlation matrix for Filter method

 The results obtained from the selection of the most important descriptors using the correlation coefficient *R* and the variance inflation factor *VIF* showed that 55 descriptors seemed to be the most appropriate. The calculated *VIFs* among the values of the selected descriptors are less than five, indicating that multicollinearity between the selected descriptors is acceptable. To get an overview of the correlation structure we used a heatmap to highlight what is important (Figure 2). Table 3 shows the *VIF* values for the selected descriptors and their meanings.

**Table 3:** The VIF values for the selected descriptors by filter method

**Descriptor**	**Type**	**Description**	**VIF**
nX	Constitutional descriptors	number of halogen atoms maximal	2.6615
MAXDN	Topological descriptors	Electrotopological negative variation	4.4004
MAXDP	Topological descriptors	maximal electrotopological positive variation	3.5133
PJI2	Topological descriptors	2D Petitjean shape index	1.7543
Lop	Topological descriptors	Lopping centric index	1.9696
MATS1m	2D autocorrelations	Moran autocorrelation - lag 1 / weighted by atomic masses	2.9735
MATS2m	2D autocorrelations	Moran autocorrelation - lag 2 / weighted by atomic masses	4.0324
MATS4m	2D autocorrelations	Moran autocorrelation - lag 4 / weighted by atomic masses	2.6631
GATS2m	2D autocorrelations	Geary autocorrelation - lag 2 / weighted by atomic masses	3.3624
GATS4m	2D autocorrelations	Geary autocorrelation - lag 4 / weighted by atomic masses	2.7846
JGI2	Topological charge indices	Mean topological charge index of order2	2.4942
JGI3	Topological charge indices	Mean topological charge index of order3	3.5132
JGI4	Topological charge indices	Mean topological charge index of order4	1.8913
JGI5	Topological charge indices	Mean topological charge index of order5	2.3317
JGI6	Topological charge indices	Mean topological charge index of order6	1.9848
JGI7	Topological charge indices	Mean topological charge index of order7	2.0805
JGI8	Topological charge indices	Mean topological charge index of order8	1.6600
JGI9	Topological charge indices	Mean topological charge index of order9	2.0077
JGI10	Topological charge indices	Mean topological charge index of order10	1.8634
FDI	Geometrical descriptors	Folding degree index	2.7337
PJI3	Geometrical descriptors	3D Petitjean shape index	1.8725
DISPm	Geometrical descriptors	d COMMA2 value / weighted by atomic masses	2.3418
DISPe	Geometrical descriptors	d COMMA2 value / weighted by atomic Sanderson electronegativities	4.0247
Mor04m	3D-MoRSE descriptors	3D-MoRSE - signal 04 / weighted by atomic masses	2.4145
Mor12m	3D-MoRSE descriptors	3D-MoRSE - signal 12 / weighted by atomic masses	3.0770
Mor17m	3D-MoRSE descriptors	3D-MoRSE - signal 17 / weighted by atomic masses	1.8941
Mor26m	3D-MoRSE descriptors	3D-MoRSE - signal 26 / weighted by atomic masses	2.3693
Mor28m	3D-MoRSE descriptors	3D-MoRSE - signal 28 / weighted by atomic masses	2.5970
Mor31m	3D-MoRSE descriptors	3D-MoRSE - signal 31 / weighted by atomic masses	2.7935
G2u	WHIM descriptors	2st component symmetry directional WHIM index / unweighted	2.5765
G2m	WHIM descriptors	2st component symmetry directional WHIM index / weighted by atomic masses	2.7232
E2m	WHIM descriptors	2nd component accessibility directional WHIM index / weighted by atomic masses	2.9830
G2v	WHIM descriptors	2st component symmetry directional WHIM index / weighted by atomic van der Waals volumes	2.6210
G2e	WHIM descriptors	2st component symmetry directional WHIM index / weighted by atomic Sanderson electronegativities	3.5147
G2p	WHIM descriptors	2st component symmetry directional WHIM index / weighted by atomic polarizabilities	2.8308
G2s	WHIM descriptors	2st component symmetry directional WHIM index / weighted by atomic electrotopological states	2.9183
E2s	WHIM descriptors	2nd Component accessibility directional WHIM index / weighted by atomic electrotopological states	3.0706
ISH	GETAWAY descriptors	Standardized information content on the leverage equality	1.7718
HATS4m	GETAWAY descriptors	Leverage-weighted autocorrelation of lag 4 / weighted by atomic masses	3.2173
C-005	Atom-centred fragments	Atom-centred fragments	2.0372
C-006	Atom-centred fragments	Atom-centred fragments	2.3826
C-008	Atom-centred fragments	Atom-centred fragments	2.7744
C-025	Atom-centred fragments	Atom-centred fragments	2.4806
C-026	Atom-centred fragments	Atom-centred fragments	3.2520
C-040	Atom-centred fragments	Atom-centred fragments	3.5563
H-048	Atom-centred fragments	Atom-centred fragments	1.9890
H-052	Atom-centred fragments	Atom-centred fragments	2.3870
O-057	Atom-centred fragments	Atom-centred fragments	2.2844
O-060	Atom-centred fragments	Atom-centred fragments	2.7103
N-072	Atom-centred fragments	Atom-centred fragments	2.5844
N-075	Atom-centred fragments	Atom-centred fragments	2.0612
Inflammat-80	Molecularproperties	Ghose-Viswanadhan-Wendoloskiantiinflammatory at 80% (drug-like index)	2.6589
Hypertens-80	Molecularproperties	Ghose-Viswanadhan-Wendoloski antihypertensive at 80% (drug-like index)	2.8495
Hypnotic-80	Molecular properties	Ghose-Viswanadhan-Wendoloski hypnotic at 80% (drug-like index)	2.4199
Neoplastic-50	Molecular properties	Ghose-Viswanadhan-Wendoloski antineoplastic at 50% (drug-like index)	1.8707

**Figure 3:**
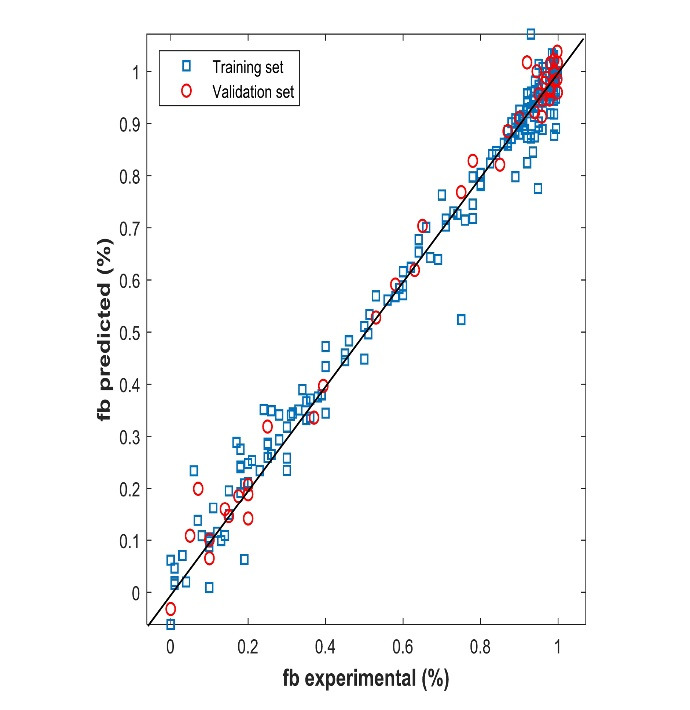
Comparison between experimental and predicted values for training and validation sets

 We followed the above-mentioned procedure to determine the required number of hidden neurons. The best model’s accuracy was assessed using the *R*(all), *MSE*(validation), $R_{train}^{2}$ , and *Q*^2^ criteria. The best model was chosen based on the maximum *R*(all), $R_{train}^{2}$ , and *Q*^2^ and the lowest MSE (validation).^31,37^ Table 4 shows 10 network models developed. The results obtained show that network eight with 40 neurons is the best model with *R* (all) = 0.990, $R_{train}^{2}$ = 0.981, *Q*^2^ = 0.989, and *MSE* (validation) = 0.002. The best performance of the model had a topology of (55-40-1): 55 input nodes, one hidden layer with 40 nodes having the hyperbolic tangent as a transfer function, and one output layer with an identity function. The neural networks were implemented using Neural Network Toolbox for MATLAB.^33^ Figure 3 shows the predicted protein-binding values versus the experimental ones for the training and validation sets. The results show a close correlation between predicted and observed plasma protein-binding. The network type used is a Feed-Forward Network with the Levenberg-Marquardt backpropagation training function and gradient descent with momentum weight and bias learning function and the data was partitioned using holdout cross-validation. The difference between $R_{train}^{2}$ and *Q*^2^ was equal to 0.008. this difference did not exceed 0.3 indicating the robustness of the model.^38^

**Table 4:** Selected criteria of the different multi-layer perceptron for Filter method

**Number of hidden neurons**	***R*** **(all)**	$R_{train}^{2}$	**Q**^2^	**MSE (validation)**
5	0.849	0.743	0.707	0.039
10	0.857	0.729	0.664	0.032
15	0.870	0.774	0.743	0.031
20	0.872	0.780	0.714	0.038
25	0.917	0.839	0.780	0.026
30	0.957	0.918	0.882	0.020
35	0.955	0.953	0.818	0.024
40	0.990	0.981	0.989	0.002
45	0.944	0.901	0.875	0.014
50	0.832	0.694	0.714	0.027

 In order to investigate the predictability and performance of the model developed in this work, a statistical evaluation is carried out, as shown in Table 5. The model’s robustness is demonstrated by the fact that the internal validation’s statistical coefficients are all acceptable and satisfactory (lowest *MSE*, *RMSE*, and *MAE*, as well as high $R_{train}^{2}$ , *Q*^2^, $R_{adjusted}^{2}$ ). External validation parameters were also used to evaluate the model’s quality. We can say that this model stands out due to its high predictive power. The excellent *Q*^2^ value is greater than 0.9.^38^

**Table 5:** External and internal criteria of the model

**Parameters**	**Value**
Internal validation	
R (all)	0.991
$R_{train}^{2}$	0.981
*Q*^2^	0.989
MSE	0.002
MAE	0.028
RMSE	0.039
$R_{adjusted}^{2}$	0.989
External validation	
R	0.983
*Q*^2^	0.966
MSE	0.004
MAE	0.042
RMSE	0.063

###  Comparison between models from literature

 We made a comparison between the few models reported in the literature with our developed model for the prediction of the binding of drugs to plasma proteins (Table 6). The evaluation of the advantages and disadvantages of these methods is quite difficult (each study used different data sets and different modeling approaches). We can see that the statistical parameters of our study exceed the models published previously. Our model gives a high *R*^2^, *Q*^2^, $R_{adjusted}^{2}$ and lowest MSE, RMSE, MAE. According to these results, our model can be used for predicting plasma protein binding for new drugs saving amounts of money and time.

**Table 6:** Comparison with literature

**Method**	***MAE***	***R***^2^	***R***	***MSE***
Suggested method (Filter method)	Train 0.0313 Validation 0.0284 Test 0.0423	Train 0.981 Validation 0.989 Test 0.966	Train 0.991 Validation 0.995 Test 0.983	Train 0.031 Validation 0.028 Test 0.042
Yuan et al^18^	Test 0.076			
Sun et al^16^	Test 0.126			
Kumar et al^17^				Train 0.869 Test 0.8881
Li et al^39^		Train 0.86		
Ghafourian et al^12^	Train 13.25 Validation 14.96	Train 0.717 Validation 0.646	Train 0.681 Validation 0.641	
Moda et al^40^		Test 0.91		

###  Applicability domain

 A clearly defined applicability domain is recommended as the principle in OECD^41^ guidelines. In this work, we analyzed the domain of applicability with different approaches reported in Table 7 with the results. The proposed approaches’ algorithm and method can be found in the literature.^42,43^

**Table 7:** Applicability domain for Filter method

**Approach**	**Test inside AD**	**Test outside AD**
Bounding box (PCA)	39	3
Euclidan distance (95 percentile)	40	2
Classical KNN (Euclidean distance, k = 5)	40	2
KNN (Euclidean distance, k = 25)	41	1

**Figure 4:**
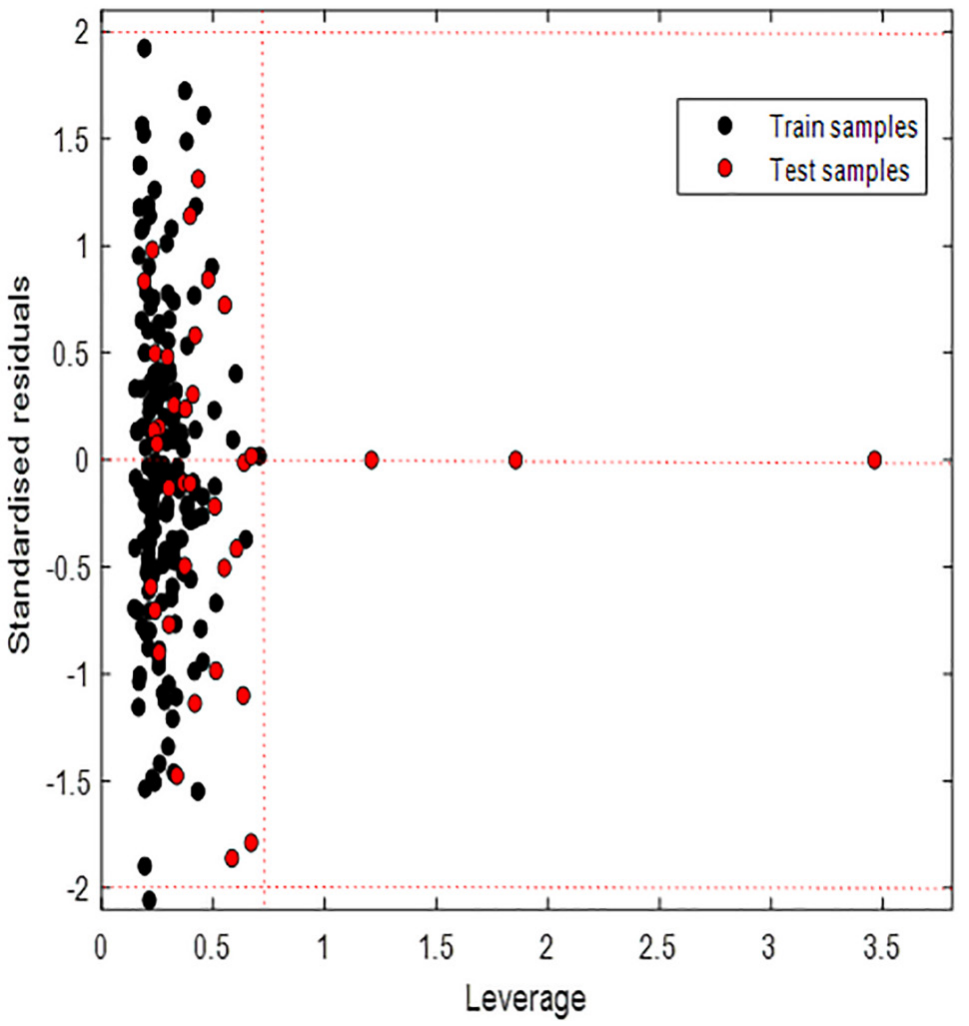
Plot of the residuals for calculated values of fb versus their experimental values for training and test sets

 The number of samples inside the applicability domain varied depending on the method used. Euclidean distance (95 percentile) and Classical KNN (Euclidean distance, k = 5) identified two test samples out of the domain of applicability. KNN (Euclidean distance k = 25) showed one of the test samples out of the applicability domain. Bounding box considered 03 test samples out of the applicability domain as shown in Figure 4. Although our points are far from the rest of the observations, they are close to the regression fitted line because they have a small residual, we speak of good leverage points. These results show that the model can be used to predict plasma protein binding for new compounds that have not been tested.

## Conclusion

 In this study, we constructed a QSAR model to predict 277 human plasma protein binding. The feature selection strategy by a Filter method has produced 55 inputs, which were used to train a FFNN for predictions. Examination of the estimates of external and internal criteria indicated that the QSAR model developed is robust, externally predictive, and distinguished by a good applicability domain. The external accuracy of the validation set was calculated by the *Q*^2^ and *RMSE* which are equal to 0.966 and 0.063 respectively. 98.30% of the external validation set is correctly predicted. According to the OECD principle, we can say that this QSAR model can be used to predict the fraction of human plasma protein binding for drugs that have not been tested to avoid chemical synthesis and reduce expansive laboratory tests.

## Competing Interests

 None to be declared.

## Ethical Approval

 Not applicable.

## Supplementary Files

Supplementary file 1. Chemical names and experimental protein-binding values.

Supplementary file 2. The name and number of calculated descriptors.
