# Supplementary material for: A Quantitative Structure-Activity Relationship for Human Plasma Protein Binding: Prediction, Validation and Applicability Domain
Source: Adv Pharm Bull. 2023 Apr 29;13(4):784–91. doi: 10.34172/apb.2023.078 (PMC10676552; doi:10.34172/apb.2023.078)
Supplement: Supplementary file 1 — Chemical names and experimental protein-binding values. [file apb-13-784-s001.pdf]

**Compound**	**fb**
3-Carboxy-4-methyl-5-propyl-2-furanpropionic acid	**0.99**
Acebutolol	**0.26**
Acenocoumarin	**0.99**
Acetaminophen	**0.2**
Acyclovir	**0.15**
Alendronate	**0.78**
Alfentanil	**0.92**
Allopurinol	**0.01**
Alprazolam	**0.71**
Alprenolol	**0.76**
Amikacin	**0.04**
Amiodarone	**0.999**
Amitriptyline	**0.95**
Amlodipine	**0.93**
Amoxicillin	**0.18**
Ampicillin	**0.18**
Antipyrine	**0.1**
Aprepitant	**0.95**
Atenolol	**0.03**
Atropine	**0.18**
Azapropazone	**0.99**
Azathioprine	**0.3**
Benoxaprofen	**0.99**
Benzylpenicillin / Penicillin G	**0.65**
Bepridil	**0.99**
Betamethasone	**0.64**
Betaxolol	**0.55**
Bilirubin	**0.999**
Binedaline	**0.96**
Bisoprolol	**0.35**
Bosentan	**0.98**
Bromocriptine	**0.93**
Bufuralol	**0.91**
Bupivacaine	**0.95**
Buprenorphine	**0.96**
Buspirone	**0.95**
Butorphanol	**0.8**
Caffeine	**0.36**
Calcitriol	**0.999**
Camptothecin	**0.983**
Candesartan	**0.998**
Carbamazepine	**0.8**
Carbenoxolone	**0.99**
Carprofen	**0.99**
Carvedilol	**0.95**
Cefaclor	**0.25**
Cefadroxil	**0.2**
Cefamandole	**0.73**
Cefazolin	**0.89**

Cefepime	**0.18**
Cefixime	**0.67**
Cefoperazone	**0.91**
Cefotaxime	**0.38**
Cefoxitin	**0.765**
Cefprozil	**0.4**
Cefsulodin	**0.45**
Ceftazidime	**0.21**
Ceftizoxime	**0.28**
Ceftriaxone	**0.938**
Cefuroxime	**0.315**
Celecoxib	**0.97**
Cephalexin	**0.14**
Cephaloridine	**0.2**
Cephalothin	**0.71**
Cephradine	**0.14**
Cetiedil	**0.74**
Cetirizine	**0.99**
Chlorothiazide	**0.95**
Chlorpheniramine	**0.7**
Chlorpromazine	**0.978**
Chlorpropamide	**0.96**
Chlorprothixene	**0.99**
Chlorthalidone	**0.75**
Cicletanine	**0.935**
Cimetidine	**0.19**
Cimoxatone	**0.945**
Cinacalcet	**0.95**
Cinoxacin	**0.63**
Ciprofloxacin	**0.4**
Clindamycin	**0.94**
Clofibric acid	**0.98**
Clometacin	**0.99**
Clonidine	**0.2**
Clozapine	**0.95**
Cyclophosphamide	**0.13**
Dapsone	**0.73**
Desipramine	**0.86**
Dexamethasone	**0.68**
Diazepam	**0.987**
Diclofenac	**0.995**
Dicloxacillin	**0.958**
Didanosine	**0.05**
Diflunisal	**0.99**
Digitoxigenin	**0.927**
Digitoxin	**0.935**
Diltiazem	**0.78**
Diphenhydramine	**0.78**
Disopyramide	**0.89**
Docetaxel	**0.94**

Dofetilide	**0.64**
Domperidone	**0.92**
Doxycycline	**0.88**
Entacapone	**0.98**
Ethambutol	**0.18**
Etodolac	**0.991**
Etoposide	**0.96**
Famotidine	**0.17**
Fenbufen	**0.985**
Fenoprofen	**0.99**
Fentanyl	**0.84**
Fentiazac	**0.995**
Finasteride	**0.9**
Flecainide	**0.61**
Fluconazole	**0.11**
Fluindione	**0.995**
Flumazenil	**0.4**
Fluorouracil	**0.1**
Fluphenazine	**0.92**
Flurbiprofen	**0.99**
Foscarnet	**0.15**
Fulvestrant	**0.99**
Furosemide	**0.986**
Fusidic acid	**0.948**
Gabapentin	**0.03**
Galantamine	**0.18**
Ganciclovir	**0.01**
Gemfibrozil	**0.97**
Gentamicin	**0.1**
Glimepiride	**0.995**
Glyburide	**0.998**
Granisetron	**0.65**
Halofenate	**0.995**
Hydrochlorothiazide	**0.58**
Hydromorphone	**0.071**
Ibuprofen	**0.99**
Imatinib	**0.95**
Indomethacin	**0.9**
Indoprofen	**0.98**
Irbesartan	**0.9**
Isosorbide dinitrate	**0.28**
Isosorbide-5-mononitrate	**0**
Isradipine	**0.97**
Itanoxone	**0.985**
Ketoprofen	**0.99**
Lamivudine	**0.36**
Lansoprazole	**0.97**
Letrozole	**0.6**
Levetiracetam	**0.1**
Levofloxacin	**0.3**

Lidocaine	0.67
Linezolid	**0.31**
Lomefloxacin	**0.1**
Lorazepam	**0.91**
Losartan	**0.987**
Meloxicam	**0.994**
Melphalan	**0.9**
Meperidine	**0.58**
Mepirzepine / mirtazapine	**0.85**
Mepivacaine	**0.84**
Mercaptopurine	**0.19**
Metformin	**0**
Methadone	**0.88**
Methicillin	**0.39**
Methotrexate	0.53
Methylprednisolone	**0.78**
Metoclopramide	**0.4**
Metoprolol	**0.08**
Metronidazole	**0.11**
Midazolam	**0.95**
Montelukast	**0.99**
Morphine	**0.35**
Moxifloxacin	**0.394**
Moxisylyte	**0.26**
Nadolol	**0.28**
Nafcillin	**0.89**
Nalidixic acid	**0.94**
Nalmefene	**0.34**
Naloxone	**0.3**
Naproxen	**0.994**
Neostigmine	**0**
Nicergoline	**0.95**
Nifedipine	**0.96**
Nimesulide	**0.99**
Nitrendipine	**0.94**
Nitrofurantoin	**0.62**
Norepinephrine	**0.5**
Norfloxacin	**0.18**
Nortriptyline	**0.92**
Novobiocin	**0.9**
Ofloxacin	**0.25**
Omeprazole	**0.95**
Ondansetron	**0.73**
Oxacillin	**0.92**
Oxazepam	**0.984**
Oxycodone	**0.45**
Oxyphenbutazone	**0.99**
Pancuronium	**0.07**
pentobarbital	**0.513**
Phenacetin	**0.33**

Phencyclidine	**0.65**
Phenobarbital	**0.51**
Phenylbutazone	**0.978**
Phenytoin	**0.91**
Pindolol	**0.59**
Pipotiazine	**0.45**
Pirenzepine	**0.12**
Piretanide	**0.94**
Pirprofen	**0.998**
Practolol	**0.3**
Pravastatin	**0.46**
Prazosin	**0.95**
Prednisone	**0.75**
Pregnenolone	**0.8**
Procainamide	**0.16**
Procaine	**0.06**
Propafenone	**0.95**
Propanolol	**0.94**
Propofol	**0.98**
Propranolol	**0.87**
Pyrimethamine	**0.87**
Quetiapine	**0.83**
Quinidine	**0.87**
Quinine	0.93
Ranitidine	**0.15**
Repaglinide	**0.974**
Riluzole	**0.98**
Risedronate	**0.24**
Risperidone	**0.89**
Rizatriptan	**0.14**
Rocuronium	**0.25**
Ropivacaine	**0.92**
Salicylic acid	**0.95**
Scopolamine	**0.1**
Sildenafil	**0.96**
Sotalol	**0.175**
Sufentanil	**0.93**
Sulfamethoxazole	**0.53**
Sulfaphenazole	**0.95**
Sulfinpyrazone	**0.99**
Sulfisoxazole	**0.914**
Sulindac	**0.935**
Sulpiride	**0**
Sumatriptan	**0.175**
Suprofen	**0.99**
Tamsulosin	**0.99**
Tegaserod	**0.98**
Temazepam	**0.968**
Tenofovir	**0.01**
Tenoxicam	**0.99**

Terazosin	**0.92**
Terbutaline	**0.23**
Testosterone	**0.6**
Tetracycline	0.5
Theophylline	**0.56**
Thiopental	**0.825**
Ticlopidine	**0.965**
Timolol	0.1
Tolamolol	**0.91**
Tolazamide	**0.97**
Tolbutamide	**0.96**
Tramadol	**0.2**
Trazodone	**0.93**
Triazolam	**0.9**
Trimethoprim	**0.37**
Urapidil	**0.8**
Valproic acid	**0.93**
Valsartan	**0.95**
Vecuronium	**0.69**
Verapamil	**0.9**
Vinorelbine	**0.87**
Vinpocetine	**0.66**
Warfarin	**0.99**
Zaleplon	**0.6**
Zidovudine	**0.25**
Ziprasidone	**0.999**
Zolpidem	**0.92**
Zomepirac	**0.985**
