# Supplementary material for: A Quantitative Structure-Activity Relationship for Human Plasma Protein Binding: Prediction, Validation and Applicability Domain
Source: Adv Pharm Bull. 2023 Apr 29;13(4):784–91. doi: 10.34172/apb.2023.078 (PMC10676552; doi:10.34172/apb.2023.078)
Supplement: Supplementary file 2 — The name and number of calculated descriptors. [file apb-13-784-s002.pdf]

**Compound**	fb	MOL_ID	MW
3-Carboxy-4-methyl-5-propyl-2-furanpropionic acid	0.99	C12H16O5	240.28
Acebutolol	0.26	C18H28O4N2	336.48
Acenocoumarin	0.99	C19H15O6N	353.35
Acetaminophen	0.2	C8H9O2N	151.18
Acyclovir	0.15	C8H11N5O3	225.24
Alendronate	0.78	C4H13O7NP2	249.12
Alfentanil	0.92	C21H32N6O3	416.59
Allopurinol	0.01	C5H4ON4	136.13
Alprazolam	0.71	C17H13N4Cl	308.79
Alprenolol	0.76	C15H23NO2	249.39
Amikacin	0.04	C22H43N5O13	585.7
Amiodarone	0.999	C25H29O3I2N	645.35
Amitriptyline	0.95	C20H23N	277.44
Amlodipine	0.93	C20H25N2O5Cl	408.92
Amoxicillin	0.18	C16H19SN3O5	365.45
Ampicillin	0.18	C16H19SN3O4	349.45
Antipyrine	0.1	C11H12N2O	188.25
Aprepitant	0.95	C23H21O3N4F7	534.48
Atenolol	0.03	C14H22N2O3	266.38
Atropine	0.18	C17H23O3N	289.41
Azapropazone	0.99	C16H20O2N4	300.4
Azathioprine	0.3	C9H7N7SO2	277.3
Benoxaprofen	0.99	C16H12O3NCl	301.74
Benzylpenicillin / Penicillin G	0.65	C16H18SN2O4	334.43
Bepridil	0.99	C24H34ON2	366.6
Betamethasone	0.64	C22H29O5F	392.51
Betaxolol	0.55	C18H29NO3	307.48
Bilirubin	0.999	C33H36N4O6	584.73
Binedaline	0.96	C19H23N3	293.45
Bisoprolol	0.35	C18H31NO4	325.5
Bosentan	0.98	C27H29O6N5S	551.68
Bromocriptine	0.93	C33H41N4O5Br	653.68
Bufuralol	0.91	C16H23O2N	261.4
Bupivacaine	0.95	C18H28N2O	288.48
Buprenorphine	0.96	C29H41O4N	467.71
Buspirone	0.95	C21H31O2N5	385.57
Butorphanol	0.8	C21H29O2N	327.51
Caffeine	0.36	C8H10N4O2	194.22
Calcitriol	0.999	C27H44O3	416.71
Camptothecin	0.983	C20H16O4N2	348.38
Candesartan	0.998	C24H20O3N6	440.5
Carbamazepine	0.8	C15H12N2O	236.29
Carbenoxolone	0.99	C34H50O7	570.84
Carprofen	0.99	C15H12NClO2	273.73
Carvedilol	0.95	C24H26O4N2	406.52
Cefaclor	0.25	C15H14N3O4SCl	367.84
Cefadroxil	0.2	C16H17N3SO5	363.43
Cefamandole	0.73	C18H18N6S2O5	462.56
Cefazolin	0.89	C14H14N8S3O4	454.57

Cefepime	**0.18**	C19H24N6O5S2	480.63
Cefixime	**0.67**	C16H15N5O7S2	453.5
Cefoperazone	**0.91**	C26H28N8O8S2	644.76
Cefotaxime	**0.38**	C16H17O7N5S2	455.52
Cefoxitin	**0.765**	C16H17O7N3S2	427.5
Cefprozil	**0.4**	C18H19N3O5S	389.47
Cefsulodin	**0.45**	C22H20N4S2O8	532.6
Ceftazidime	**0.21**	C22H22O7N6S2	546.64
Ceftizoxime	**0.28**	C13H13O5N5S2	383.45
Ceftriaxone	**0.938**	C18H18N8O7S3	554.65
Cefuroxime	**0.315**	C16H16O8N4S	424.43
Celecoxib	**0.97**	C17H14N3SO2F3	381.41
Cephalexin	**0.14**	C16H17N3SO4	347.43
Cephaloridine	**0.2**	C19H17N3S2O4	415.53
Cephalothin	**0.71**	C16H16O6N2S2	396.48
Cephradine	**0.14**	C16H19O4SN3	349.45
Cetiedil	**0.74**	C20H31NO2S	349.59
Cetirizine	**0.99**	C21H25O3N2Cl	388.93
Chlorothiazide	**0.95**	C7H6N3S2O4Cl	295.75
Chlorpheniramine	**0.7**	C16H19N2Cl	274.82
Chlorpromazine	**0.978**	C17H19N2SCl	318.9
Chlorpropamide	**0.96**	C10H13N2O3SCl	276.77
Chlorprothixene	**0.99**	C18H18NSCl	315.89
Chlorthalidone	**0.75**	C14H11O4N2ClS	338.79
Cicletanine	**0.935**	C14H12NO2Cl	261.72
Cimetidine	**0.19**	C10H16N6S	252.39
Cimoxatone	**0.945**	C19H18O4N2	338.39
Cinacalcet	**0.95**	C22H22NF3	357.45
Cinoxacin	**0.63**	C12H10O5N2	262.24
Ciprofloxacin	**0.4**	C17H18O3N3F	331.38
Clindamycin	**0.94**	C18H33O5N2ClS	425.05
Clofibric acid	**0.98**	C10H11O3Cl	214.66
Clometacin	**0.99**	C19H16NO4Cl	357.81
Clonidine	**0.2**	C9H9Cl2N3	230.11
Clozapine	**0.95**	C18H19N4Cl	326.86
Cyclophosphamide	**0.13**	C7H15N2O2PCl2	261.11
Dapsone	**0.73**	C12H12N2SO2	248.33
Desipramine	**0.86**	C18H22N2	266.42
Dexamethasone	**0.68**	C22H29O5F	392.51
Diazepam	**0.987**	C16H13N2ClO	284.76
Diclofenac	**0.995**	C14H11O2NCl2	296.16
Dicloxacillin	**0.958**	C19H17O5N3Cl2S	470.36
Didanosine	**0.05**	C10H12O3N4	236.26
Diflunisal	**0.99**	C13H8O3F2	250.21
Digitoxigenin	**0.927**	C23H34O4	374.57
Digitoxin	**0.935**	C41H64O13	765.05
Diltiazem	**0.78**	C22H26O4SN2	414.57
Diphenhydramine	**0.78**	C17H21NO	255.39
Disopyramide	**0.89**	C21H29N3O	339.53
Docetaxel	**0.94**	C43H53O14N	807.97

Dofetilide	0.64	C19H27N3O5S2	441.63
Domperidone	0.92	C22H24ClN5O2	425.96
Doxycycline	0.88	C22H24O8N2	444.48
Entacapone	0.98	C14H15N3O5	305.32
Ethambutol	0.18	C10H24O2N2	204.36
Etodolac	0.991	C17H21O3N	287.39
Etoposide	0.96	C29H32O13	588.61
Famotidine	0.17	C8H15O2S3N7	337.51
Fenbufen	0.985	C16H14O3	254.3
Fenoprofen	0.99	C15H14O3	242.29
Fentanyl	0.84	C22H28ON2	336.52
Fentiazac	0.995	C17H12NSO2Cl	329.82
Finasteride	0.9	C23H36N2O2	372.61
Flecainide	0.61	C17H20F6O3N2	414.39
Fluconazole	0.11	C13H12ON6F2	306.31
Fluindione	0.995	C15H9O2F	240.24
Flumazenil	0.4	C15H14O3N3F	303.32
Fluorouracil	0.1	C4H3N2FO2	130.09
Fluphenazine	0.92	C22H26ON3SF3	437.58
Flurbiprofen	0.99	C15H13O2F	244.28
Foscarnet	0.15	CH3O5P	126.01
Fulvestrant	0.99	C32H47SO3F5	606.86
Furosemide	0.986	C12H11O5ClN2S	330.77
Fusidic acid	0.948	C31H48O6	516.79
Gabapentin	0.03	C9H17NO2	171.27
Galantamine	0.18	C17H21O3N	287.39
Ganciclovir	0.01	C9H13N5O4	255.27
Gemfibrozil	0.97	C15H22O3	250.37
Gentamicin	0.1	C19H39O7N5	449.63
Glimepiride	0.995	C24H34N4O5S	490.69
Glyburide	0.998	C23H28O5ClN3S	494.06
Granisetron	0.65	C18H24N4O	312.46
Halofenate	0.995	C19H17O4NClF3	415.82
Hydrochlorothiazide	0.58	C7H8N3S2O4Cl	297.77
Hydromorphone	0.071	C17H19O3N	285.37
Ibuprofen	0.99	C13H18O2	206.31
Imatinib	0.95	C29H31N7O	493.67
Indomethacin	0.9	C19H16O4NCl	357.81
Indoprofen	0.98	C17H15O3N	281.33
Irbesartan	0.9	C25H28N6O	428.59
Isosorbide dinitrate	0.28	C6H8O8N2	236.16
Isosorbide-5-mononitrate	0	C6H9O6N	191.16
Isradipine	0.97	C19H23O5N3	373.45
Itanoxone	0.985	C17H13O3Cl	300.75
Ketoprofen	0.99	C16H14O3	254.3
Lamivudine	0.36	C8H11N3O3S	229.29
Lansoprazole	0.97	C16H14SO2N3F3	369.4
Letrozole	0.6	C17H11N5	285.33
Levetiracetam	0.1	C8H14N2O2	170.24
Levofloxacin	0.3	C18H20O4N3F	361.41

Lidocaine	0.67	C14H22N2O	234.38
Linezolid	**0.31**	C16H20O4N3F	337.39
Lomefloxacin	**0.1**	C17H19N3O3F2	351.39
Lorazepam	**0.91**	C15H10O2N2Cl2	321.17
Losartan	**0.987**	C22H23OClN6	422.96
Meloxicam	**0.994**	C14H13N3S2O4	351.44
Melphalan	**0.9**	C13H18N2Cl2O2	305.23
Meperidine	**0.58**	C15H21O2N	247.37
Mepirzepine / mirtazapine	**0.85**	C17H19N3	265.39
Mepivacaine	**0.84**	C15H22N2O	246.39
Mercaptopurine	**0.19**	C5H4SN4	152.2
Metformin	**0**	C4H11N5	129.2
Methadone	**0.88**	C21H27ON	309.49
Methicillin	**0.39**	C17H20N2SO6	380.46
Methotrexate	0.53	C20H22N8O5	454.5
Methylprednisolone	**0.78**	C22H30O5	374.52
Metoclopramide	**0.4**	C14H22N3O2Cl	299.84
Metoprolol	**0.08**	C15H25O3N	267.41
Metronidazole	**0.11**	C6H9N3O3	171.18
Midazolam	**0.95**	C18H13N3FCl	325.79
Montelukast	**0.99**	C35H36NClSO3	586.24
Morphine	**0.35**	C17H19NO3	285.37
Moxifloxacin	**0.394**	C21H24O4N3F	401.48
Moxisylyte	**0.26**	C16H25O3N	279.42
Nadolol	**0.28**	C17H27NO4	309.45
Nafcillin	**0.89**	C21H22O5N2S	414.52
Nalidixic acid	**0.94**	C12H12O3N2	232.26
Nalmefene	**0.34**	C21H25O3N	339.47
Naloxone	**0.3**	C19H21O3N	311.41
Naproxen	**0.994**	C14H14O3	230.28
Neostigmine	**0**	C12H19N2O2	223.33
Nicergoline	**0.95**	C24H26N3O3Br	484.43
Nifedipine	**0.96**	C17H18N2O6	346.37
Nimesulide	**0.99**	C13H12SO5N2	308.34
Nitrendipine	**0.94**	C18H20O6N2	360.4
Nitrofurantoin	**0.62**	C8H6O5N4	238.18
Norepinephrine	**0.5**	C8H11NO3	169.2
Norfloxacin	**0.18**	C16H18O3N3F	319.37
Nortriptyline	**0.92**	C19H21N	263.41
Novobiocin	**0.9**	C31H36O11N2	612.69
Ofloxacin	**0.25**	C18H20O4N3F	361.41
Omeprazole	**0.95**	C17H19O3N3S	345.46
Ondansetron	**0.73**	C17H17N3O	279.37
Oxacillin	**0.92**	C19H19O5N3S	401.48
Oxazepam	**0.984**	C15H11O2N2Cl	286.73
Oxycodone	**0.45**	C18H21O4N	315.4
Oxyphenbutazone	**0.99**	C19H20O3N2	324.41
Pancuronium	**0.07**	C35H60O4N2	572.97
pentobarbital	**0.513**	C11H18O3N2	226.31
Phenacetin	**0.33**	C10H13NO2	179.24

Phencyclidine	**0.65**	C17H25N	243.43
Phenobarbital	**0.51**	C12H12N2O3	232.26
Phenylbutazone	**0.978**	C19H20O2N2	308.41
Phenytoin	**0.91**	C15H12O2N2	252.29
Pindolol	**0.59**	C14H20N2O2	248.36
Pipotiazine	**0.45**	C24H33O3N3S2	475.74
Pirenzepine	**0.12**	C19H21N5O2	351.45
Piretanide	**0.94**	C17H18O5N2S	362.44
Pirprofen	**0.998**	C13H14O2ClN	251.73
Practolol	**0.3**	C14H22N2O3	266.38
Pravastatin	**0.46**	C23H36O7	424.59
Prazosin	**0.95**	C19H21O4N5	383.45
Prednisone	**0.75**	C21H26O5	358.47
Pregnenolone	**0.8**	C21H32O2	316.53
Procainamide	**0.16**	C13H21N3O	235.37
Procaine	**0.06**	C13H20N2O2	236.35
Propafenone	**0.95**	C21H27NO3	341.49
Propanolol	**0.94**	C16H21NO2	259.38
Propofol	**0.98**	C12H18O	178.3
Propranolol	**0.87**	C16H21NO2	259.38
Pyrimethamine	**0.87**	C12H13N4Cl	248.74
Quetiapine	**0.83**	C21H25O2N3S	383.56
Quinidine	**0.87**	C20H24O2N2	324.46
Quinine	0.93	C20H24O2N2	324.46
Ranitidine	**0.15**	C13H22N4O3S	314.46
Repaglinide	**0.974**	C27H36O4N2	452.65
Riluzole	**0.98**	C8H5N2OF3S	234.22
Risedronate	**0.24**	C7H11O7NP2	283.13
Risperidone	**0.89**	C23H27N4O2F	410.54
Rizatriptan	**0.14**	C15H19N5	269.39
Rocuronium	**0.25**	C32H53O4N2	529.87
Ropivacaine	**0.92**	C17H26N2O	274.45
Salicylic acid	**0.95**	C7H6O3	138.13
Scopolamine	**0.1**	C17H21NO4	303.39
Sildenafil	**0.96**	C22H30N6O4S	474.65
Sotalol	**0.175**	C12H20N2O3S	272.41
Sufentanil	**0.93**	C22H30O2N2S	386.61
Sulfamethoxazole	**0.53**	C10H11N3SO3	253.31
Sulfaphenazole	**0.95**	C15H14N4SO2	314.4
Sulfinpyrazone	**0.99**	C23H20N2O3S	404.52
Sulfisoxazole	**0.914**	C11H13N3SO3	267.34
Sulindac	**0.935**	C20H17SO3F	356.44
Sulpiride	**0**	C15H23N3O4S	341.48
Sumatriptan	**0.175**	C14H21N3SO2	295.45
Suprofen	**0.99**	C14H12O3S	260.33
Tamsulosin	**0.99**	C20H28O5N2S	408.57
Tegaserod	**0.98**	C16H23N5O	301.44
Temazepam	**0.968**	C16H13N2ClO2	300.76
Tenofovir	**0.01**	C9H14N5O4P	287.25
Tenoxicam	**0.99**	C13H11N3O4S2	337.41

Terazosin	**0.92**	C19H25N5O4	387.49
Terbutaline	**0.23**	C12H19NO3	225.32
Testosterone	**0.6**	C19H28O2	288.47
Tetracycline	0.5	C22H24O8N2	444.48
Theophylline	**0.56**	C7H8N4O2	180.19
Thiopental	**0.825**	C11H18O2N2S	242.38
Ticlopidine	**0.965**	C14H14SNCl	263.81
Timolol	0.1	C13H24N4O3S	316.48
Tolamolol	**0.91**	C19H24O4N2	344.45
Tolazamide	**0.97**	C14H21SO3N3	311.45
Tolbutamide	**0.96**	C12H18N2O3S	270.39
Tramadol	**0.2**	C16H25O2N	263.42
Trazodone	**0.93**	C19H22ClN5O	371.91
Triazolam	**0.9**	C17H12N4Cl2	343.23
Trimethoprim	**0.37**	C14H18O3N4	290.36
Urapidil	**0.8**	C20H29N5O3	387.54
Valproic acid	**0.93**	C8H16O2	144.24
Valsartan	**0.95**	C24H29O3N5	435.58
Vecuronium	**0.69**	C34H57N2O4	557.93
Verapamil	**0.9**	C27H38O4N2	454.67
Vinorelbine	**0.87**	C45H54O8N4	779.03
Vinpocetine	**0.66**	C22H26O2N2	350.5
Warfarin	**0.99**	C19H16O4	308.35
Zaleplon	**0.6**	C17H15N5O	305.37
Zidovudine	**0.25**	C10H14N5O4	268.29
Ziprasidone	**0.999**	C21H21N4OClS	412.98
Zolpidem	**0.92**	C19H21N3O	307.43
Zomepirac	**0.985**	C15H14O3NCl	291.75

AMW	Sv	Se	Sp	Ss	Mv	Me
7.28	19.34	33.71	20.36	49.5	0.59	1.02
6.47	29.81	52	31.73	59.5	0.57	1
8.62	27.25	42.25	28.06	73.67	0.66	1.03
7.56	12.41	20.29	12.96	30.5	0.62	1.01
8.34	16.3	28.14	16.68	44.17	0.6	1.04
9.23	14.53	28.53	16.88	55.36	0.54	1.06
6.72	36.27	62.08	38.3	67.25	0.58	1
9.72	9.49	14.73	9.48	27	0.68	1.05
8.82	24.67	35.15	25.69	46.28	0.7	1
6.08	23.59	40.48	25.29	39.5	0.58	0.99
7.06	44.98	85.55	47.4	118	0.54	1.03
10.76	39.35	59.48	44.11	63.74	0.66	0.99
6.31	27.57	42.82	29.38	38.33	0.63	0.97
7.72	32.42	53.77	34.28	69.78	0.61	1.01
8.31	27.41	45.09	29.03	70.25	0.62	1.02
8.13	26.9	43.76	28.57	64.58	0.63	1.02
7.24	16.49	25.95	17.27	32	0.63	1
9.22	36.46	61.58	37.09	118.67	0.63	1.06
6.5	23.5	41.02	24.99	47.17	0.57	1
6.58	26.11	43.8	27.74	46.67	0.59	1
7.15	25.78	42.13	27.02	51.33	0.61	1
10.67	18.07	27.44	18.6	50.67	0.69	1.06
9.14	22.82	33.71	23.8	52.61	0.69	1.02
8.16	25.91	41.66	27.57	60.75	0.63	1.02
6.01	36.06	59.67	38.65	49.5	0.59	0.98
6.89	33.64	57.4	35.63	74.83	0.59	1.01
6.03	28.9	50.45	31.03	45.33	0.57	0.99
7.4	49.61	79.51	51.93	109.5	0.63	1.01
6.52	27.96	44.14	29.63	41.67	0.62	0.98
6.03	30.01	53.67	32.24	49.83	0.56	0.99
8.11	43.3	69.15	45.54	95.17	0.64	1.02
7.78	51.98	84.06	55.11	96.08	0.62	1
6.22	24.59	41.48	26.29	40.25	0.59	0.99
5.89	28.27	48.02	30.36	42	0.58	0.98
6.24	44	74.08	47.05	66.75	0.59	0.99
6.54	34.76	58.65	36.84	59.25	0.59	0.99
6.18	31.39	52.13	33.57	46.67	0.59	0.98
8.09	14.79	24.71	15.22	37.67	0.62	1.03
5.63	41.69	72.42	45.11	62.17	0.56	0.98
8.29	28.22	42.7	29.16	63.25	0.67	1.02
8.31	35.68	53.78	36.73	76.67	0.67	1.01
7.88	20.49	29.95	21.27	41.33	0.68	1
6.27	52.53	90.38	56.22	97.17	0.58	0.99
8.83	21.31	31.38	22.34	46.61	0.69	1.01
7.26	35.21	56.12	36.97	64.5	0.63	1
9.68	25.4	39.32	26.91	66.94	0.67	1.03
8.65	26.81	43.2	28.27	70.5	0.64	1.03
9.44	32.29	50.7	34.17	82.83	0.66	1.03
10.57	29.06	45	31.09	76.83	0.68	1.05

8.58	35.08	57.35	37.45	82.17	0.63	1.02
10.08	29.72	47.37	31.31	89.33	0.66	1.05
8.95	46.2	74.42	48.59	120.5	0.64	1.03
9.69	30.31	49.25	32.07	85.83	0.64	1.05
9.5	28.93	46.93	30.82	79.92	0.64	1.04
8.47	29.41	47.09	31.03	74.5	0.64	1.02
9.51	37.03	58.25	39.05	106.58	0.66	1.04
9.27	38.5	61.12	40.6	102.58	0.65	1.04
10.09	25.1	39.83	26.64	70.5	0.66	1.05
10.27	35.79	56.75	37.98	102	0.66	1.05
9.43	28.74	47.4	29.87	84.83	0.64	1.05
9.54	26.61	41.76	27.72	79.17	0.67	1.04
8.47	26.3	41.88	27.81	64.83	0.64	1.02
9.23	30.39	45.95	32.46	71.33	0.68	1.02
9.44	27.42	43.51	29.36	72.5	0.65	1.04
8.13	26.9	43.76	28.57	63.83	0.63	1.02
6.36	32.07	54.09	34.98	45.83	0.58	0.98
7.48	32.4	52.11	34.37	61.11	0.62	1
12.86	16.1	24.86	17.51	56.11	0.7	1.08
7.23	24.07	37.48	25.72	38.44	0.63	0.99
7.97	26.16	39.56	28.37	40.78	0.65	0.99
9.23	18.9	30.89	20.45	49.03	0.63	1.03
8.1	26.16	38.45	28.36	40.94	0.67	0.99
10.27	22.81	34.33	24.14	63.78	0.69	1.04
8.72	20.31	30.38	21.34	41.44	0.68	1.01
7.65	20.04	33.11	21.49	39.33	0.61	1
7.87	27.82	43.58	28.92	60.17	0.65	1.01
7.45	30.5	48.24	31.96	65.92	0.64	1.01
9.04	18.94	30.37	19.33	52.67	0.65	1.05
7.89	26.41	42.87	27.41	62.5	0.63	1.02
7.08	33.9	60.38	36.97	66.61	0.57	1.01
8.59	15.82	25.61	16.79	38.86	0.63	1.02
8.73	27.52	41.8	28.77	64.11	0.67	1.02
10	15.77	23.49	16.78	31.89	0.69	1.02
7.78	27.46	41.8	28.97	45.61	0.65	1
9	17.08	29.55	19.41	34.53	0.59	1.02
8.56	19.09	29.35	20.38	45.58	0.66	1.01
6.34	25.97	41.04	27.63	36.67	0.62	0.98
6.89	33.64	57.4	35.63	74.83	0.59	1.01
8.63	22.79	33.16	23.89	45.61	0.69	1
9.87	21.01	30.71	22.2	49.22	0.7	1.02
10.01	31.81	48.73	33.74	80.31	0.68	1.04
8.15	17.9	29.92	18.43	43.17	0.62	1.03
9.62	17.75	27.43	18.05	58.67	0.68	1.05
6.14	35.21	60.33	37.76	57.25	0.58	0.99
6.48	66.78	118.53	71.27	122.75	0.57	1
7.54	34.3	55.19	36.61	66.5	0.62	1
6.38	24.48	39.27	26.07	37.17	0.61	0.98
6.29	32.26	53.12	34.37	53.92	0.6	0.98
7.28	66.7	112.66	70.17	151.42	0.6	1.01

7.89	33.89	56.7	36.72	75	0.61	1.01
7.89	34.67	54.32	36.41	66.61	0.64	1.01
7.94	34.66	57.54	36.02	93.58	0.62	1.03
8.25	23.13	38.24	23.86	68.5	0.63	1.03
5.38	19.59	37.58	21.3	32.67	0.52	0.99
6.84	25.51	41.92	26.98	47.75	0.61	1
7.95	45.22	76.39	47.09	103.33	0.61	1.03
9.64	21.64	36.13	23.94	56.75	0.62	1.03
7.71	21.72	33.17	22.69	49.33	0.66	1.01
7.57	20.72	32.17	21.69	44.5	0.65	1.01
6.35	32.27	52.02	34.36	49.83	0.61	0.98
9.7	24.39	34.46	25.99	53.11	0.72	1.01
5.91	36.17	61.88	38.86	55.75	0.57	0.98
8.63	28.36	50.86	29.14	94	0.59	1.06
9.01	22.09	35.5	22.41	61.25	0.65	1.04
8.9	19.12	27.59	19.65	49.33	0.71	1.02
8.43	23.21	37.1	23.89	59.17	0.64	1.03
10.84	7.72	13.25	7.62	34	0.64	1.1
7.81	34.69	56.73	36.83	74.92	0.62	1.01
7.88	20.32	31.35	21.18	48.67	0.66	1.01
12.6	5.64	11.38	6.48	34.47	0.56	1.14
6.9	50.72	88.6	54.5	109.14	0.58	1.01
10.34	21.32	33.66	22.6	63.53	0.67	1.05
6.08	48.42	84.17	52	84.92	0.57	0.99
5.91	15.8	28.83	17.01	30.42	0.54	0.99
6.84	25.51	41.92	26.98	43.58	0.61	1
8.23	18.41	32.35	18.89	51.5	0.59	1.04
6.26	23.11	39.7	24.74	42.92	0.58	0.99
6.42	37.71	70.82	40.15	77.92	0.54	1.01
7.22	40.59	68.37	43.36	85.75	0.6	1.01
8.1	38.1	61.83	40.69	83.53	0.62	1.01
6.65	28.47	46.57	30.09	46.67	0.61	0.99
9.24	29.05	47.11	30.11	85.19	0.65	1.05
11.91	16.7	26.74	18.27	55.11	0.67	1.07
7.13	24.91	40.04	26.22	45.58	0.62	1
6.25	19.4	32.61	20.76	36.17	0.59	0.99
7.26	43.64	67.64	45.63	77.5	0.64	0.99
8.73	27.52	41.8	28.77	64.11	0.67	1.02
7.81	23.71	36.27	24.7	52.83	0.66	1.01
7.14	38.05	59.66	39.86	66.42	0.63	0.99
9.84	13.88	26.47	13.93	54.33	0.58	1.1
8.69	12.46	23.6	12.78	40.83	0.57	1.07
7.47	30.52	50.78	31.9	65.67	0.61	1.02
8.85	23.42	34.49	24.55	56.28	0.69	1.01
7.71	21.72	33.17	22.69	49.67	0.66	1.01
8.82	16	26.9	17.07	40.33	0.62	1.03
9.47	25.61	40.76	26.72	72.47	0.66	1.05
8.65	23.76	33.16	24.31	53	0.72	1
6.55	14.6	26.16	15.49	32.67	0.56	1.01
7.86	28.52	47.08	29.63	67.67	0.62	1.02

6.01	22.48	38.37	24.08	36.67	0.58	0.98
7.67	26.52	45.08	27.63	62.17	0.6	1.02
7.99	27.12	45.27	28.11	71.17	0.62	1.03
10.36	22.4	31.92	23.44	53.72	0.72	1.03
7.98	34.56	53.21	36.2	65.44	0.65	1
9.76	24.19	37.19	25.94	62.92	0.67	1.03
8.25	22.79	37.46	24.49	49.06	0.62	1.01
6.34	23	38.59	24.53	38.58	0.59	0.99
6.8	24.76	38.37	26.11	37	0.63	0.98
6.16	23.48	39.37	25.08	37.5	0.59	0.98
10.87	10.06	14.48	10.67	23.83	0.72	1.03
6.46	10.76	20.16	11.31	25.33	0.54	1.01
6.19	30.28	48.92	32.36	47.58	0.61	0.98
8.27	28.53	47.2	30.24	69.58	0.62	1.03
8.26	34.69	56.64	35.65	92	0.63	1.03
6.57	33.53	56.89	35.69	66.92	0.59	1
7.14	24.68	42.12	26.4	47.44	0.59	1
6.08	24.7	43.69	26.51	41.5	0.56	0.99
8.15	12.31	21.94	12.66	37.33	0.59	1.04
9.05	25.38	36.44	26.38	52.94	0.71	1.01
7.61	50.08	76.39	53.58	89.78	0.65	0.99
7.13	24.91	40.04	26.22	45.25	0.62	1
7.58	32.72	53.85	34.15	71.83	0.62	1.02
6.21	25.7	44.69	27.51	44.67	0.57	0.99
6.32	27.81	48.9	29.72	52.25	0.57	1
8.13	32.61	51.75	34.55	73.25	0.64	1.01
8.01	18.51	29.6	19.18	46.5	0.64	1.02
6.79	30.7	49.69	32.51	51.33	0.61	0.99
7.08	27.51	43.92	28.98	50.33	0.63	1
7.43	19.72	31.17	20.69	42.17	0.64	1.01
6.38	20.09	34.87	21.39	37	0.57	1
8.5	36.78	57.12	38.87	65.33	0.65	1
8.06	26.84	44.24	27.83	70.17	0.62	1.03
9.34	21.62	34.33	22.74	61.58	0.66	1.04
7.83	28.44	47.12	29.59	71.67	0.62	1.02
10.36	15.13	24.93	15.06	55.17	0.66	1.08
7.36	13.52	23.5	14.18	35.83	0.59	1.02
7.79	25.41	41.87	26.41	61.67	0.62	1.02
6.42	25.97	39.94	27.62	36.83	0.63	0.97
7.66	48.78	81.83	50.95	116.75	0.61	1.02
7.86	28.52	47.08	29.63	67.67	0.62	1.02
8.03	27.39	43.43	29.12	54.56	0.64	1.01
7.35	24.68	37.82	25.8	44.17	0.65	1
8.54	30.41	48.09	32.03	72.75	0.65	1.02
9.25	21.7	31.6	22.59	49.94	0.7	1.02
7.17	27.02	44.25	28.44	51	0.61	1.01
7.37	27.9	44.14	29.23	58.17	0.63	1
5.67	56.37	99.14	60.91	78	0.56	0.98
6.66	19.31	34.25	20.47	44.08	0.57	1.01
6.89	15.6	26.06	16.48	31.5	0.6	1

5.66	25.17	41.71	27.14	29.92	0.59	0.97
8.01	18.51	29.6	19.18	47.42	0.64	1.02
7.17	27.39	42.81	28.77	52.5	0.64	1
8.14	21	31.28	21.73	46.92	0.68	1.01
6.54	22.39	37.81	23.77	39.17	0.59	1
7.32	39.66	64.69	43.1	69.92	0.61	1
7.48	29.78	47.23	31.03	59	0.63	1
8.43	27.42	43.99	29.02	67.42	0.64	1.02
8.12	19.9	31.27	21.1	42.11	0.64	1.01
6.5	23.5	41.02	24.99	46.17	0.57	1
6.43	37.34	66.2	39.89	78.17	0.57	1
7.83	30.8	49.89	31.94	64.83	0.63	1.02
6.89	31.33	52.12	33.17	66.42	0.6	1
5.76	31.59	53.79	34.09	45.5	0.57	0.98
6.19	21.87	37.59	23.32	38.5	0.58	0.99
6.39	21.39	36.81	22.77	39.5	0.58	0.99
6.57	31.3	51.57	33.27	56	0.6	0.99
6.48	24	39.59	25.53	40.67	0.6	0.99
5.75	17.89	30.28	19.31	27.67	0.58	0.98
6.48	24	39.59	25.53	40.67	0.6	0.99
8.29	19.67	30.15	20.69	39.61	0.66	1
7.38	32.67	51.76	34.95	54.67	0.63	1
6.76	29.59	47.58	31.3	49.5	0.62	0.99
6.76	29.59	47.58	31.3	49.5	0.62	0.99
7.31	24.98	43.42	26.89	51.33	0.58	1.01
6.56	41.2	68.53	43.77	74.33	0.6	0.99
11.71	13.71	21.8	14.21	50.25	0.69	1.09
10.11	16.93	29.64	19.12	61.03	0.6	1.06
7.2	35.28	57.18	37.01	66.17	0.62	1
6.91	24.15	38.69	25.36	39.67	0.62	0.99
5.82	51.28	89.54	55.25	73.33	0.56	0.98
5.97	26.67	45.13	28.6	40.5	0.58	0.98
8.63	10.33	16.63	10.65	32	0.65	1.04
7.06	26.02	43.25	27.44	49.83	0.61	1.01
7.53	38.27	63.6	40.64	77.75	0.61	1.01
7.17	21.99	38.21	23.88	47.42	0.58	1.01
6.78	34.47	56.31	37.23	54.58	0.6	0.99
9.05	18	28.9	19.07	46.58	0.64	1.03
8.73	24.08	36.56	25.39	55.08	0.67	1.02
8.26	32.99	49.21	34.88	68.89	0.67	1
8.62	19.59	31.78	20.84	48.25	0.63	1.03
8.49	28.11	42.52	29.8	65.72	0.67	1.01
7.42	27.09	46.53	29.1	59.42	0.59	1.01
7.21	24.47	40.99	26.43	47.08	0.6	1
8.68	20.21	30.36	21.58	47.5	0.67	1.01
7.3	33.41	56.4	35.83	67.58	0.6	1.01
6.7	26.86	44.79	28.34	48.83	0.6	1
8.85	23.3	34.48	24.35	51.44	0.69	1.01
8.7	19.89	34.21	21.34	53.64	0.6	1.04
10.22	22.6	34.3	24.18	61.25	0.68	1.04

7.31	31.99	53.65	33.46	63	0.6	1.01
6.44	19.91	35.04	21.22	41.58	0.57	1
5.89	28.39	48.03	30.57	42.17	0.58	0.98
7.94	34.66	57.54	36.02	93.67	0.62	1.03
8.58	13.19	21.83	13.45	36.17	0.63	1.04
7.13	19.88	34	21.66	40.75	0.58	1
8.51	20.97	30.69	22.84	32.61	0.68	0.99
7.03	25.58	45.3	27.65	46.25	0.57	1.01
7.03	29.61	49.23	31.2	60.17	0.6	1
7.42	24.99	42.32	26.88	52.92	0.59	1.01
7.51	21.39	36.33	23.11	48.42	0.59	1.01
5.99	25.19	43.36	27.05	38.92	0.57	0.99
7.75	30.56	48.11	32.19	55.28	0.64	1
9.81	25.37	35.47	26.55	50.06	0.72	1.01
7.45	23.7	39.57	24.72	49.67	0.61	1.01
6.8	33.68	57.09	35.53	62.83	0.59	1
5.55	13.81	25.72	15	26	0.53	0.99
7.14	37.68	61.09	39.53	75.83	0.62	1
5.75	54.47	95.31	58.77	76.5	0.56	0.98
6.4	41.8	70.42	44.53	70.58	0.59	0.99
7.02	68.01	111.12	71.69	125.33	0.61	1
6.74	32.18	51.46	34.06	51.58	0.62	0.99
7.91	25.83	39.38	26.91	58	0.66	1.01
8.04	25.47	38.25	26.29	55	0.67	1.01
8.13	19.71	34.29	20.27	55	0.6	1.04
8.43	32.66	49.09	34.84	58.28	0.67	1
6.99	27.87	43.59	29.32	49.17	0.63	0.99
8.58	22.41	34.59	23.56	53.28	0.66	1.02

Mp	Ms	nAT	nSK	nBT	nBO	nBM
0.62	2.91	33	17	33	17	7
0.61	2.48	52	24	52	24	8
0.68	2.83	41	26	43	28	17
0.65	2.77	20	11	20	11	7
0.62	2.76	27	16	28	17	7
0.63	3.95	27	14	26	13	2
0.62	2.24	62	30	64	32	9
0.68	2.7	14	10	15	11	4
0.73	2.1	35	22	38	25	18
0.62	2.19	41	18	41	18	7
0.57	2.95	83	40	85	42	1
0.74	2.06	60	31	62	33	17
0.67	1.83	44	21	46	23	13
0.65	2.49	53	28	54	29	10
0.66	2.81	44	25	46	27	9
0.66	2.69	43	24	45	26	9
0.66	2.29	26	14	27	15	8
0.64	3.21	58	37	61	40	14
0.61	2.48	41	19	41	19	7
0.63	2.22	44	21	46	23	7
0.64	2.33	42	22	44	24	9
0.72	2.67	26	19	28	21	17
0.72	2.51	33	21	35	23	17
0.67	2.64	41	23	43	25	9
0.63	1.83	61	27	63	29	12
0.63	2.67	57	28	60	31	4
0.61	2.06	51	22	52	23	6
0.66	2.55	79	43	82	46	20
0.66	1.89	45	22	47	24	16
0.6	2.17	54	23	54	23	6
0.67	2.44	68	39	71	42	26
0.66	2.23	84	43	90	49	14
0.63	2.12	42	19	43	20	10
0.62	2	49	21	50	22	7
0.63	1.96	75	34	81	40	6
0.62	2.12	59	28	62	31	8
0.63	1.94	53	24	57	28	6
0.63	2.69	24	14	25	15	7
0.61	2.07	74	30	76	32	3
0.69	2.43	42	26	46	30	15
0.69	2.32	53	33	57	37	28
0.71	2.3	30	18	32	20	14
0.62	2.37	91	41	95	45	5
0.72	2.45	31	19	33	21	16
0.66	2.15	56	30	59	33	21
0.71	2.79	38	24	40	26	10
0.67	2.82	42	25	44	27	10
0.7	2.67	49	31	52	34	15
0.72	2.65	43	29	46	32	14

0.67	2.57	56	32	59	35	10
0.7	2.98	45	30	47	32	12
0.67	2.74	72	44	76	48	18
0.68	2.86	47	30	49	32	11
0.68	2.85	45	28	47	30	10
0.67	2.76	46	27	48	29	11
0.7	2.96	56	36	59	39	19
0.69	2.77	59	37	62	40	17
0.7	2.82	38	25	40	27	10
0.7	2.83	54	36	57	39	13
0.66	2.93	45	29	47	31	11
0.69	3.04	40	26	42	28	19
0.68	2.7	41	24	43	26	10
0.72	2.55	45	28	48	31	15
0.7	2.79	42	26	44	28	10
0.66	2.66	43	24	45	26	6
0.64	1.91	55	24	57	26	6
0.66	2.26	52	27	54	29	13
0.76	3.3	23	17	24	18	11
0.68	2.02	38	19	39	20	12
0.71	1.94	40	21	42	23	12
0.68	2.88	30	17	30	17	9
0.73	1.95	39	21	41	23	13
0.73	2.9	33	22	35	24	15
0.71	2.3	30	18	32	20	12
0.65	2.31	33	17	33	17	7
0.67	2.41	43	25	45	27	14
0.67	2.54	48	26	50	28	17
0.67	2.77	29	19	31	21	9
0.65	2.6	42	24	45	27	9
0.62	2.47	60	27	61	28	1
0.67	2.78	25	14	25	14	7
0.7	2.56	41	25	43	27	18
0.73	2.28	23	14	24	15	7
0.69	1.98	42	23	45	26	14
0.67	2.47	29	14	29	14	1
0.7	2.68	29	17	30	18	14
0.66	1.83	42	20	44	22	12
0.63	2.67	57	28	60	31	4
0.72	2.28	33	20	35	22	14
0.74	2.59	30	19	31	20	13
0.72	2.68	47	30	50	33	14
0.64	2.54	29	17	31	19	7
0.69	3.26	26	18	27	19	13
0.62	2.12	61	27	65	31	2
0.6	2.27	118	54	125	61	2
0.67	2.29	55	29	57	31	14
0.65	1.96	40	19	41	20	12
0.64	2.16	54	25	55	26	13
0.63	2.61	111	58	116	63	18

0.66	2.59	56	29	57	30	16
0.67	2.22	54	30	58	34	14
0.64	2.92	56	32	59	35	11
0.64	3.11	37	22	37	22	11
0.56	2.33	38	14	37	13	0
0.64	2.27	42	21	44	23	11
0.64	2.46	74	42	80	48	13
0.68	2.84	35	20	35	20	9
0.69	2.6	33	19	34	20	14
0.68	2.47	32	18	33	19	13
0.65	1.99	53	25	55	27	13
0.76	2.41	34	22	36	24	18
0.62	2.06	63	27	66	30	3
0.61	3.36	48	28	49	29	7
0.66	2.78	34	22	36	24	16
0.73	2.74	27	18	29	20	14
0.66	2.69	36	22	38	24	13
0.63	3.78	12	9	12	9	3
0.66	2.5	56	30	59	33	12
0.68	2.7	31	18	32	19	13
0.65	4.92	10	7	9	6	2
0.62	2.66	88	41	91	44	7
0.71	3.03	32	21	33	22	14
0.61	2.3	85	37	88	40	4
0.59	2.53	29	12	29	12	1
0.64	2.08	42	21	45	24	7
0.61	2.86	31	18	32	19	7
0.62	2.38	40	18	40	18	7
0.57	2.51	70	31	72	33	0
0.64	2.52	68	34	70	36	12
0.67	2.53	61	33	63	35	16
0.64	2.03	47	23	50	26	11
0.67	3.04	45	28	46	29	14
0.73	3.24	25	17	26	18	10
0.66	2.17	40	21	44	25	7
0.63	2.41	33	15	33	15	7
0.67	2.09	68	37	72	41	25
0.7	2.56	41	25	43	27	18
0.69	2.52	36	21	38	23	14
0.66	2.08	60	32	64	36	19
0.58	3.4	24	16	25	17	4
0.58	3.14	22	13	23	14	2
0.64	2.43	50	27	52	29	10
0.72	2.68	34	21	35	22	15
0.69	2.61	33	19	34	20	14
0.66	2.69	26	15	27	16	3
0.69	2.9	39	25	41	27	17
0.74	2.41	33	22	35	24	19
0.6	2.72	26	12	26	12	2
0.64	2.6	46	26	49	29	9

0.62	2.16	39	17	39	17	7
0.63	2.59	44	24	46	26	8
0.64	2.85	44	25	46	27	9
0.76	2.56	31	21	33	23	14
0.68	2.18	53	30	56	33	22
0.72	2.74	36	23	38	25	15
0.66	2.58	37	19	37	19	7
0.63	2.14	39	18	40	19	7
0.67	1.85	39	20	42	23	12
0.63	2.08	40	18	41	19	7
0.76	2.38	14	10	15	11	4
0.57	2.81	20	9	19	8	2
0.65	2.07	50	23	51	24	13
0.66	2.68	46	26	48	28	9
0.65	2.79	55	33	57	35	20
0.63	2.48	57	27	60	30	4
0.63	2.37	42	20	42	20	7
0.6	2.18	44	19	44	19	6
0.6	3.11	21	12	21	12	7
0.73	2.3	36	23	39	26	18
0.7	2.19	77	41	81	45	25
0.66	2.15	40	21	44	25	7
0.64	2.48	53	29	57	33	9
0.61	2.23	45	20	45	20	7
0.61	2.38	49	22	50	23	6
0.68	2.53	51	29	54	32	14
0.66	2.74	29	17	30	18	9
0.65	2.05	50	25	55	30	7
0.66	2.19	44	23	48	27	8
0.67	2.48	31	17	32	18	12
0.61	2.31	35	16	35	16	7
0.68	2.11	57	31	61	35	17
0.65	2.81	43	25	44	26	12
0.69	2.93	33	21	34	22	16
0.64	2.76	46	26	47	27	12
0.65	3.25	23	17	24	18	10
0.62	2.99	23	12	23	12	6
0.64	2.68	41	23	43	25	9
0.67	1.84	41	20	43	22	13
0.64	2.65	80	44	83	47	17
0.64	2.6	46	26	49	29	9
0.68	2.27	43	24	45	26	17
0.68	2.1	38	21	41	24	16
0.68	2.6	47	28	50	31	14
0.73	2.5	31	20	33	22	14
0.65	2.22	44	23	48	27	7
0.66	2.42	44	24	46	26	14
0.6	1.9	101	41	106	46	2
0.6	2.76	34	16	34	16	3
0.63	2.42	26	13	26	13	7

0.63	1.66	43	18	45	20	6
0.66	2.79	29	17	30	18	9
0.67	2.28	43	23	45	25	14
0.7	2.47	31	19	33	21	14
0.63	2.18	38	18	39	19	10
0.66	2.18	65	32	68	35	14
0.66	2.27	47	26	50	29	14
0.67	2.7	43	25	45	27	15
0.68	2.48	31	17	32	18	8
0.61	2.43	41	19	41	19	7
0.6	2.61	66	30	67	31	4
0.65	2.32	49	28	52	31	17
0.64	2.55	52	26	55	29	5
0.62	1.98	55	23	58	26	2
0.61	2.26	38	17	38	17	7
0.62	2.32	37	17	37	17	7
0.64	2.24	52	25	53	26	13
0.64	2.14	40	19	41	20	11
0.62	2.13	31	13	31	13	6
0.64	2.14	40	19	41	20	11
0.69	2.33	30	17	31	18	12
0.67	2.02	52	27	55	30	13
0.65	2.06	48	24	51	27	12
0.65	2.06	48	24	51	27	12
0.63	2.44	43	21	43	21	8
0.63	2.25	69	33	71	35	14
0.71	3.35	20	15	21	16	10
0.68	3.59	28	17	28	17	8
0.65	2.21	57	30	61	34	13
0.65	1.98	39	20	41	22	15
0.61	1.93	91	38	96	43	2
0.62	2.03	46	20	47	21	7
0.67	3.2	16	10	16	10	7
0.64	2.27	43	22	46	25	7
0.65	2.36	63	33	66	36	15
0.63	2.63	38	18	38	18	8
0.65	2.02	57	27	59	29	12
0.68	2.74	28	17	29	18	13
0.71	2.5	36	22	38	24	19
0.71	2.38	49	29	52	32	21
0.67	2.68	31	18	32	19	13
0.71	2.63	42	25	44	27	16
0.63	2.58	46	23	47	24	9
0.64	2.35	41	20	42	21	12
0.72	2.64	30	18	31	19	13
0.64	2.41	56	28	57	29	14
0.63	2.22	45	22	46	23	9
0.72	2.45	34	21	36	23	14
0.65	2.82	33	19	34	20	11
0.73	2.78	33	22	35	24	15

0.63	2.25	53	28	56	31	12
0.61	2.6	35	16	35	16	6
0.62	2.01	49	21	52	24	2
0.64	2.93	56	32	59	35	11
0.64	2.78	21	13	22	14	7
0.64	2.55	34	16	34	16	3
0.74	1.92	31	17	33	19	11
0.61	2.2	45	21	46	22	5
0.64	2.41	49	25	50	26	13
0.64	2.52	42	21	43	22	9
0.64	2.69	36	18	36	18	9
0.61	2.05	44	19	45	20	6
0.67	2.13	48	26	51	29	10
0.76	2.18	35	23	38	26	18
0.63	2.37	39	21	40	22	12
0.62	2.24	57	28	59	30	9
0.58	2.6	26	10	25	9	1
0.65	2.37	61	32	63	34	19
0.61	1.91	97	40	102	45	2
0.63	2.14	71	33	72	34	13
0.65	2.2	111	57	119	65	21
0.65	1.98	52	26	56	30	12
0.69	2.52	39	23	41	25	15
0.69	2.39	38	23	40	25	18
0.61	2.89	33	19	34	20	5
0.71	2.08	49	28	53	32	17
0.67	2.14	44	23	46	25	17
0.69	2.66	34	20	35	21	13

SCBO	ARR	nCIC	nCIR	RBN	RBF	nDB
21.5	0.294	1	1	6	0.182	2
29	0.25	1	1	10	0.192	2
39	0.429	3	4	5	0.116	5
15	0.545	1	1	1	0.05	1
21.5	0.294	2	3	4	0.143	2
15	0	0	0	5	0.192	2
38	0.188	3	3	9	0.141	3
15	0	2	3	0	0	4
34.5	0.68	4	7	1	0.026	1
22	0.333	1	1	8	0.195	1
43	0	3	3	10	0.118	1
42	0.485	3	4	11	0.177	1
30	0.522	3	6	3	0.065	1
36	0.207	2	2	10	0.185	4
33	0.222	3	4	4	0.087	3
32	0.231	3	4	4	0.089	3
20	0.4	2	2	1	0.037	2
48	0.3	4	4	6	0.098	2
23	0.316	1	1	8	0.195	1
27	0.261	3	4	5	0.109	1
30	0.25	3	6	3	0.068	3
30.5	0.714	3	4	3	0.107	2
32	0.696	3	4	3	0.086	1
31	0.24	3	4	4	0.093	3
35	0.414	3	3	10	0.159	0
35	0	4	10	2	0.033	4
26	0.261	2	2	11	0.212	0
61	0.217	4	4	12	0.146	10
32	0.667	3	4	5	0.106	0
26	0.261	1	1	12	0.222	0
56	0.571	4	4	11	0.155	2
58	0.204	7	18	5	0.056	4
25	0.5	2	3	5	0.116	0
26	0.273	2	2	5	0.1	1
43	0.15	7	38	5	0.062	0
36	0.194	4	4	6	0.097	2
31	0.214	5	11	2	0.035	0
19.5	0.333	2	3	0	0	2
35	0	3	4	6	0.079	3
39.5	0.367	5	15	1	0.022	4
51.5	0.73	5	6	7	0.123	1
28	0.6	3	6	0	0	2
50	0	5	15	6	0.063	5
29.5	0.714	3	6	2	0.061	1
43.5	0.636	4	7	10	0.169	0
33	0.231	3	4	4	0.1	4
34	0.222	3	4	4	0.091	4
43.5	0.324	4	5	7	0.135	4
41	0.313	4	5	7	0.152	4

42.5	0.143	4	5	7	0.119	5
41.5	0.156	3	4	8	0.17	7
60.5	0.229	5	6	10	0.132	7
40.5	0.156	3	4	8	0.163	6
37.5	0.167	3	4	8	0.17	5
37	0.207	3	4	5	0.104	5
52	0.308	4	5	8	0.136	7
51.5	0.275	4	5	9	0.145	6
34.5	0.185	3	4	5	0.125	5
49.5	0.128	4	5	8	0.14	8
39.5	0.161	3	4	8	0.17	6
38.5	0.607	3	3	3	0.071	2
33	0.231	3	4	4	0.093	4
40.5	0.355	4	5	6	0.125	4
35.5	0.179	3	4	7	0.159	5
32	0	3	4	4	0.089	6
29.5	0.192	3	3	7	0.123	1
36	0.414	3	3	8	0.148	1
26	0.333	2	3	1	0.042	5
26	0.6	2	2	5	0.128	0
29	0.522	3	6	4	0.095	0
23	0.353	1	1	4	0.133	3
30	0.522	3	6	3	0.073	1
33	0.5	3	4	2	0.057	3
26	0.6	3	4	1	0.031	0
22.5	0.294	1	1	7	0.212	1
36	0.444	3	3	6	0.133	1
36.5	0.607	3	4	6	0.12	0
27	0.286	3	6	2	0.065	3
33	0.222	4	5	3	0.067	3
29	0	2	2	7	0.115	1
18	0.429	1	1	3	0.12	1
37	0.593	3	4	5	0.116	2
19	0.4	2	2	2	0.083	1
34	0.462	4	7	1	0.022	2
15	0	1	1	3	0.103	1
26	0.667	2	2	2	0.067	2
28	0.545	3	6	4	0.091	0
35	0	4	10	2	0.033	4
30	0.545	3	4	1	0.029	2
27	0.6	2	2	4	0.129	1
41.5	0.333	4	5	4	0.08	3
23.5	0.263	3	4	2	0.065	2
26	0.632	2	2	2	0.074	1
33	0	5	11	1	0.015	2
63	0	8	14	7	0.056	2
39	0.387	3	4	7	0.123	2
26	0.6	2	2	6	0.146	0
33	0.462	2	2	8	0.145	1
75	0.19	6	12	13	0.112	6

40	0.4	2	2	11	0.193	4
42	0.353	5	7	5	0.086	2
43	0.171	4	10	1	0.017	5
31	0.273	1	1	5	0.135	4
13	0	0	0	9	0.243	0
29	0.435	3	6	4	0.091	1
55	0.25	7	14	5	0.063	1
26.5	0.25	1	1	7	0.2	4
28	0.6	2	2	5	0.147	2
26	0.632	2	2	4	0.121	1
34	0.444	3	3	6	0.109	1
33.5	0.708	3	3	4	0.111	1
33	0	4	10	2	0.03	3
33	0.207	2	2	7	0.143	1
32	0.667	3	3	5	0.139	0
28	0.6	3	4	1	0.034	2
31.5	0.458	3	6	3	0.079	2
12	0	1	1	0	0	3
39	0.364	4	7	6	0.102	0
26	0.632	2	2	3	0.094	1
8	0	0	0	1	0.111	2
48	0.136	4	10	14	0.154	1
30.5	0.5	2	2	5	0.152	3
44	0	4	10	6	0.068	4
13	0	1	1	3	0.103	1
28	0.25	4	12	1	0.022	1
23.5	0.263	2	3	5	0.156	2
22	0.333	1	1	6	0.15	1
33	0	3	3	6	0.083	0
45	0.167	3	3	7	0.1	6
45	0.343	3	3	8	0.127	4
32	0.385	4	6	2	0.04	1
37	0.414	2	2	8	0.174	2
25	0.333	2	3	1	0.038	4
29	0.24	5	24	0	0	1
19	0.4	1	1	4	0.121	1
54	0.585	5	5	7	0.097	1
37	0.593	3	4	4	0.093	2
31	0.522	3	4	3	0.079	2
46.5	0.472	5	5	7	0.109	2
21	0	2	3	4	0.16	4
16	0	2	3	2	0.087	2
36	0.207	3	4	6	0.115	4
31	0.545	2	2	5	0.143	3
28	0.6	2	2	4	0.118	2
19	0	2	2	2	0.074	3
36	0.593	3	4	5	0.122	1
36.5	0.708	3	3	3	0.086	0
14	0	1	1	3	0.115	2
35	0.207	4	8	2	0.041	3

21	0.353	1	1	5	0.128	1
31	0.231	3	3	4	0.087	2
33	0.222	3	4	3	0.065	3
31	0.522	3	4	1	0.03	2
44	0.667	4	4	8	0.143	0
34.5	0.44	3	4	2	0.053	4
23	0.316	1	1	6	0.162	1
23	0.316	2	2	4	0.1	1
29	0.522	4	11	0	0	0
23	0.316	2	2	2	0.049	1
15	0	2	3	0	0	4
10	0	0	0	2	0.105	2
31	0.5	2	2	7	0.137	1
34	0.214	3	4	5	0.104	3
46.5	0.486	3	4	9	0.158	3
34	0	4	10	2	0.033	4
24	0.3	1	1	7	0.167	1
22	0.316	1	1	9	0.205	0
16.5	0.417	1	1	3	0.143	2
35.5	0.654	4	7	1	0.026	1
58.5	0.511	5	6	12	0.148	2
29	0.24	5	24	0	0	1
39	0.182	5	7	4	0.07	3
24	0.3	1	1	7	0.156	1
26	0.261	2	3	6	0.12	0
40.5	0.344	4	6	5	0.093	3
24	0.333	2	3	2	0.067	3
34	0.2	6	25	2	0.036	1
32	0.222	5	24	2	0.042	2
24.5	0.611	2	3	3	0.094	1
20	0.375	1	1	3	0.086	1
44	0.457	5	13	5	0.082	1
35	0.231	2	2	6	0.136	6
32	0.545	2	2	5	0.147	4
36	0.222	2	2	7	0.149	6
25.5	0.278	2	2	3	0.125	5
15	0.5	1	1	2	0.087	0
31	0.24	3	4	3	0.07	3
29	0.545	3	6	3	0.07	1
58	0.255	4	5	9	0.108	5
35	0.207	4	8	2	0.041	3
35	0.615	3	4	5	0.111	1
32.5	0.625	4	7	2	0.049	1
39.5	0.355	4	5	4	0.08	3
30	0.545	3	4	1	0.03	2
31	0.222	5	24	1	0.021	1
34	0.462	3	3	5	0.109	2
48	0	6	12	6	0.057	2
19	0	1	1	4	0.118	3
17	0.462	1	1	3	0.115	1

23	0.3	3	3	2	0.044	0
24	0.333	2	2	2	0.067	3
33	0.48	3	3	5	0.111	2
29	0.571	3	3	2	0.061	2
24	0.526	2	3	6	0.154	0
43	0.343	4	7	8	0.118	2
37	0.414	4	7	2	0.04	2
36	0.444	3	3	5	0.111	3
23	0.333	2	2	3	0.094	2
23	0.316	1	1	7	0.171	1
35	0	2	3	11	0.164	4
40	0.516	4	5	4	0.077	1
34	0	4	10	2	0.036	5
28	0	4	10	1	0.017	2
21	0.353	1	1	6	0.158	1
21	0.353	1	1	7	0.189	1
33	0.462	2	2	11	0.208	1
25.5	0.55	2	3	6	0.146	0
16	0.462	1	1	2	0.065	0
25.5	0.55	2	3	6	0.146	0
24	0.667	2	2	2	0.065	0
37	0.4	4	7	6	0.109	1
33.5	0.407	4	6	4	0.078	1
33.5	0.407	4	6	4	0.078	1
26.5	0.238	1	1	10	0.233	3
43	0.343	3	3	10	0.141	2
21	0.625	2	3	1	0.048	0
22	0.353	1	1	4	0.143	2
42	0.294	5	7	4	0.066	3
29.5	0.682	3	4	5	0.122	0
45	0	6	12	6	0.063	2
25	0.286	2	2	4	0.085	1
14	0.6	1	1	1	0.063	1
29	0.24	4	7	5	0.109	1
45.5	0.306	4	5	7	0.106	4
23	0.333	1	1	6	0.158	2
35.5	0.379	3	3	8	0.136	1
25.5	0.611	2	2	3	0.103	2
34.5	0.708	3	3	4	0.105	2
44	0.563	4	4	6	0.115	3
26.5	0.579	2	2	3	0.094	2
37	0.444	3	4	4	0.091	4
30	0.25	2	2	6	0.128	3
28	0.476	2	3	6	0.143	2
26.5	0.579	2	2	4	0.129	2
37	0.414	2	2	11	0.193	2
29	0.261	2	3	8	0.174	3
31	0.522	3	4	1	0.028	2
26	0.5	2	3	5	0.147	1
33.5	0.458	3	4	2	0.057	4

37.5	0.355	4	5	4	0.071	1
19	0.375	1	1	4	0.114	0
26	0	4	10	0	0	2
43	0.171	4	10	1	0.017	5
18.5	0.357	2	3	0	0	2
19	0	1	1	4	0.118	3
24.5	0.579	3	4	2	0.061	0
24.5	0.227	2	2	7	0.152	0
33	0.462	2	2	10	0.2	1
28	0.273	2	2	3	0.07	3
24	0.333	1	1	5	0.139	3
23	0.3	2	2	4	0.089	0
36	0.207	4	5	5	0.098	4
35.5	0.654	4	7	1	0.026	1
28	0.545	2	2	5	0.125	0
36	0.2	3	3	7	0.119	3
10	0	0	0	5	0.2	1
44.5	0.5	3	3	10	0.159	2
47	0	6	12	6	0.059	2
42	0.353	2	2	13	0.181	0
78	0.246	9	28	10	0.084	5
37	0.333	5	22	4	0.071	2
34	0.48	3	4	4	0.098	3
36	0.64	3	4	3	0.075	1
25	0	2	2	3	0.088	5
41	0.5	5	7	4	0.075	1
34	0.64	3	4	3	0.065	1
28.5	0.524	2	2	4	0.114	2

nTB	nAB	nH	nC	nN	nO	nP
0	5	16	12	0	5	0
0	6	28	18	2	4	0
0	12	15	19	1	6	0
0	6	9	8	1	2	0
0	5	11	8	5	3	0
0	0	13	4	1	7	2
0	6	32	21	6	3	0
0	0	4	5	4	1	0
0	17	13	17	4	0	0
0	6	23	15	1	2	0
0	0	43	22	5	13	0
0	16	29	25	1	3	0
0	12	23	20	1	0	0
0	6	25	20	2	5	0
0	6	19	16	3	5	0
0	6	19	16	3	4	0
0	6	12	11	2	1	0
0	12	21	23	4	3	0
0	6	22	14	2	3	0
0	6	23	17	1	3	0
0	6	20	16	4	2	0
0	15	7	9	7	2	0
0	16	12	16	1	3	0
0	6	18	16	2	4	0
0	12	34	24	2	1	0
0	0	29	22	0	5	0
0	6	29	18	1	3	0
0	10	36	33	4	6	0
0	16	23	19	3	0	0
0	6	31	18	1	4	0
0	24	29	27	5	6	0
0	10	41	33	4	5	0
0	10	23	16	1	2	0
0	6	28	18	2	1	0
0	6	41	29	1	4	0
0	6	31	21	5	2	0
0	6	29	21	1	2	0
0	5	10	8	4	2	0
0	0	44	27	0	3	0
0	11	16	20	2	4	0
0	27	20	24	6	3	0
0	12	12	15	2	1	0
0	0	50	34	0	7	0
0	15	12	15	1	2	0
0	21	26	24	2	4	0
0	6	14	15	3	4	0
0	6	17	16	3	5	0
0	11	18	18	6	5	0
0	10	14	14	8	4	0

0	5	24	19	6	5	0
0	5	15	16	5	7	0
0	11	28	26	8	8	0
0	5	17	16	5	7	0
0	5	17	16	3	7	0
0	6	19	18	3	5	0
0	12	20	22	4	8	0
0	11	22	22	6	7	0
0	5	13	13	5	5	0
0	5	18	18	8	7	0
0	5	16	16	4	8	0
0	17	14	17	3	2	0
0	6	17	16	3	4	0
0	11	17	19	3	4	0
0	5	16	16	2	6	0
0	0	19	16	3	4	0
0	5	31	20	1	2	0
0	12	25	21	2	3	0
0	6	6	7	3	4	0
0	12	19	16	2	0	0
0	12	19	17	2	0	0
0	6	13	10	2	3	0
0	12	18	18	1	0	0
0	12	11	14	2	4	0
0	12	12	14	1	2	0
1	5	16	10	6	0	0
1	12	18	19	2	4	0
0	17	22	22	1	0	0
0	6	10	12	2	5	0
0	6	18	17	3	3	0
0	0	33	18	2	5	0
0	6	11	10	0	3	0
0	16	16	19	1	4	0
0	6	9	9	3	0	0
0	12	19	18	4	0	0
0	0	15	7	2	2	1
0	12	12	12	2	2	0
0	12	22	18	2	0	0
0	0	29	22	0	5	0
0	12	13	16	2	1	0
0	12	11	14	1	2	0
0	11	17	19	3	5	0
0	5	12	10	4	3	0
0	12	8	13	0	3	0
0	0	34	23	0	4	0
0	0	64	41	0	13	0
0	12	26	22	2	4	0
0	12	21	17	1	1	0
0	12	29	21	3	1	0
0	12	53	43	1	14	0

0	12	27	19	3	5	0
0	12	24	22	5	2	0
0	6	24	22	2	8	0
1	6	15	14	3	5	0
0	0	24	10	2	2	0
0	10	21	17	1	3	0
0	12	32	29	0	13	0
0	5	15	8	7	2	0
0	12	14	16	0	3	0
0	12	14	15	0	3	0
0	12	28	22	2	1	0
0	17	12	17	1	2	0
0	0	36	23	2	2	0
0	6	20	17	2	3	0
0	16	12	13	6	1	0
0	12	9	15	0	2	0
0	11	14	15	3	3	0
0	0	3	4	2	2	0
0	12	26	22	3	1	0
0	12	13	15	0	2	0
0	0	3	1	0	5	1
0	6	47	32	0	3	0
0	11	11	12	2	5	0
0	0	48	31	0	6	0
0	0	17	9	1	2	0
0	6	21	17	1	3	0
0	5	13	9	5	4	0
0	6	22	15	0	3	0
0	0	39	19	5	7	0
0	6	34	24	4	5	0
0	12	28	23	3	5	0
0	10	24	18	4	1	0
0	12	17	19	1	4	0
0	6	8	7	3	4	0
0	6	19	17	1	3	0
0	6	18	13	0	2	0
0	24	31	29	7	1	0
0	16	16	19	1	4	0
0	12	15	17	1	3	0
0	17	28	25	6	1	0
0	0	8	6	2	8	0
0	0	9	6	1	6	0
0	6	23	19	3	5	0
0	12	13	17	0	3	0
0	12	14	16	0	3	0
0	0	11	8	3	3	0
0	16	14	16	3	2	0
2	17	11	17	5	0	0
0	0	14	8	2	2	0
0	6	20	18	3	4	0

0	6	22	14	2	1	0
0	6	20	16	3	4	0
0	6	19	17	3	3	0
0	12	10	15	2	2	0
0	22	23	22	6	1	0
0	11	13	14	3	4	0
0	6	18	13	2	2	0
0	6	21	15	1	2	0
0	12	19	17	3	0	0
0	6	22	15	2	1	0
0	0	4	5	4	0	0
0	0	11	4	5	0	0
0	12	27	21	1	1	0
0	6	20	17	2	6	0
0	17	22	20	8	5	0
0	0	30	22	0	5	0
0	6	22	14	3	2	0
0	6	25	15	1	3	0
0	5	9	6	3	3	0
0	17	13	18	3	0	0
0	23	36	35	1	3	0
0	6	19	17	1	3	0
0	6	24	21	3	4	0
0	6	25	16	1	3	0
0	6	27	17	1	4	0
0	11	22	21	2	5	0
0	6	12	12	2	3	0
0	6	25	21	1	3	0
0	6	21	19	1	3	0
0	11	14	14	0	3	0
0	6	19	12	2	2	0
0	16	26	24	3	3	0
0	6	18	17	2	6	0
0	12	12	13	2	5	0
0	6	20	18	2	6	0
0	5	6	8	4	5	0
0	6	11	8	1	3	0
0	6	18	16	3	3	0
0	12	21	19	1	0	0
0	12	36	31	2	11	0
0	6	20	18	3	4	0
0	16	19	17	3	3	0
0	15	17	17	3	1	0
0	11	19	19	3	5	0
0	12	11	15	2	2	0
0	6	21	18	1	4	0
0	12	20	19	2	3	0
0	0	60	35	2	4	0
0	0	18	11	2	3	0
0	6	13	10	1	2	0

0	6	25	17	1	0	0
0	6	12	12	2	3	0
0	12	20	19	2	2	0
0	12	12	15	2	2	0
0	10	20	14	2	2	0
0	12	33	24	3	3	0
0	12	21	19	5	2	0
0	12	18	17	2	5	0
0	6	14	13	1	2	0
0	6	22	14	2	3	0
0	0	36	23	0	7	0
0	16	21	19	5	4	0
0	0	26	21	0	5	0
0	0	32	21	0	2	0
0	6	21	13	3	1	0
0	6	20	13	2	2	0
0	12	27	21	1	3	0
0	11	21	16	1	2	0
0	6	18	12	0	1	0
0	11	21	16	1	2	0
0	12	13	12	4	0	0
0	12	25	21	3	2	0
0	11	24	20	2	2	0
0	11	24	20	2	2	0
0	5	22	13	4	3	0
0	12	36	27	2	4	0
0	10	5	8	2	1	0
0	6	11	7	1	7	2
0	10	27	23	4	2	0
0	15	19	15	5	0	0
0	0	53	32	2	4	0
0	6	26	17	2	1	0
0	6	6	7	0	3	0
0	6	21	17	1	4	0
0	11	30	22	6	4	0
0	6	20	12	2	3	0
0	11	30	22	2	2	0
0	11	11	10	3	3	0
0	17	14	15	4	2	0
0	18	20	23	2	3	0
0	11	13	11	3	3	0
0	12	17	20	0	3	0
0	6	23	15	3	4	0
0	10	21	14	3	2	0
0	11	12	14	0	3	0
0	12	28	20	2	5	0
0	6	23	16	5	1	0
0	12	13	16	2	2	0
0	10	14	9	5	4	1
0	11	11	13	3	4	0

0	11	25	19	5	4	0
0	6	19	12	1	3	0
0	0	28	19	0	2	0
0	6	24	22	2	8	0
0	5	8	7	4	2	0
0	0	18	11	2	2	0
0	11	14	14	1	0	0
0	5	24	13	4	3	0
0	12	24	19	2	4	0
0	6	21	14	3	3	0
0	6	18	12	2	3	0
0	6	25	16	1	2	0
0	6	22	19	5	1	0
0	17	12	17	4	0	0
0	12	18	14	4	3	0
0	6	29	20	5	3	0
0	0	16	8	0	2	0
0	17	29	24	5	3	0
0	0	57	34	2	4	0
1	12	38	27	2	4	0
0	16	54	45	4	8	0
0	10	26	22	2	2	0
0	12	16	19	0	4	0
1	16	15	17	5	1	0
0	0	14	10	5	4	0
0	16	21	21	4	1	0
0	16	21	19	3	1	0
0	11	14	15	1	3	0

nS	nF	nCL	nBR	nI	nB	nHM
0	0	0	0	0	0	0
0	0	0	0	0	0	0
0	0	0	0	0	0	0
0	0	0	0	0	0	0
0	0	0	0	0	0	0
0	0	0	0	0	0	0
0	0	0	0	0	0	0
0	0	0	0	0	0	0
0	0	1	0	0	0	0
0	0	0	0	0	0	0
0	0	0	0	0	0	0
0	0	0	0	2	0	0
0	0	0	0	0	0	0
0	0	1	0	0	0	0
1	0	0	0	0	0	0
1	0	0	0	0	0	0
0	0	0	0	0	0	0
0	7	0	0	0	0	0
0	0	0	0	0	0	0
0	0	0	0	0	0	0
0	0	0	0	0	0	0
1	0	0	0	0	0	0
0	0	1	0	0	0	0
1	0	0	0	0	0	0
0	0	0	0	0	0	0
0	1	0	0	0	0	0
0	0	0	0	0	0	0
0	0	0	0	0	0	0
0	0	0	0	0	0	0
0	0	0	0	0	0	0
1	0	0	0	0	0	0
0	0	0	1	0	0	0
0	0	0	0	0	0	0
0	0	0	0	0	0	0
0	0	0	0	0	0	0
0	0	0	0	0	0	0
0	0	0	0	0	0	0
0	0	0	0	0	0	0
0	0	0	0	0	0	0
0	0	0	0	0	0	0
0	0	0	0	0	0	0
0	0	0	0	0	0	0
0	0	0	0	0	0	0
0	0	1	0	0	0	0
0	0	0	0	0	0	0
1	0	1	0	0	0	0
1	0	0	0	0	0	0
2	0	0	0	0	0	0
3	0	0	0	0	0	0

2	0	0	0	0	0	0
2	0	0	0	0	0	0
2	0	0	0	0	0	0
2	0	0	0	0	0	0
2	0	0	0	0	0	0
1	0	0	0	0	0	0
2	0	0	0	0	0	0
2	0	0	0	0	0	0
2	0	0	0	0	0	0
3	0	0	0	0	0	0
1	0	0	0	0	0	0
1	3	0	0	0	0	0
1	0	0	0	0	0	0
2	0	0	0	0	0	0
2	0	0	0	0	0	0
1	0	0	0	0	0	0
1	0	0	0	0	0	0
0	0	1	0	0	0	0
2	0	1	0	0	0	0
0	0	1	0	0	0	0
1	0	1	0	0	0	0
1	0	1	0	0	0	0
1	0	1	0	0	0	0
1	0	1	0	0	0	0
0	0	1	0	0	0	0
1	0	0	0	0	0	0
0	0	0	0	0	0	0
0	3	0	0	0	0	0
0	0	0	0	0	0	0
0	1	0	0	0	0	0
1	0	1	0	0	0	0
0	0	1	0	0	0	0
0	0	1	0	0	0	0
0	0	2	0	0	0	0
0	0	1	0	0	0	0
0	0	2	0	0	0	0
1	0	0	0	0	0	0
0	0	0	0	0	0	0
0	1	0	0	0	0	0
0	0	1	0	0	0	0
0	0	2	0	0	0	0
1	0	2	0	0	0	0
0	0	0	0	0	0	0
0	2	0	0	0	0	0
0	0	0	0	0	0	0
0	0	0	0	0	0	0
1	0	0	0	0	0	0
0	0	0	0	0	0	0
0	0	0	0	0	0	0
0	0	0	0	0	0	0

2	0	0	0	0	0	0
0	0	1	0	0	0	0
0	0	0	0	0	0	0
0	0	0	0	0	0	0
0	0	0	0	0	0	0
0	0	0	0	0	0	0
0	0	0	0	0	0	0
3	0	0	0	0	0	0
0	0	0	0	0	0	0
0	0	0	0	0	0	0
0	0	0	0	0	0	0
1	0	1	0	0	0	0
0	0	0	0	0	0	0
0	6	0	0	0	0	0
0	2	0	0	0	0	0
0	1	0	0	0	0	0
0	1	0	0	0	0	0
0	1	0	0	0	0	0
1	3	0	0	0	0	0
0	1	0	0	0	0	0
0	0	0	0	0	0	0
1	5	0	0	0	0	0
1	0	1	0	0	0	0
0	0	0	0	0	0	0
0	0	0	0	0	0	0
0	0	0	0	0	0	0
0	0	0	0	0	0	0
0	0	0	0	0	0	0
0	0	0	0	0	0	0
1	0	0	0	0	0	0
1	0	1	0	0	0	0
0	0	0	0	0	0	0
0	3	1	0	0	0	0
2	0	1	0	0	0	0
0	0	0	0	0	0	0
0	0	0	0	0	0	0
0	0	0	0	0	0	0
0	0	1	0	0	0	0
0	0	0	0	0	0	0
0	0	0	0	0	0	0
0	0	0	0	0	0	0
0	0	0	0	0	0	0
0	0	0	0	0	0	0
0	0	1	0	0	0	0
0	0	0	0	0	0	0
1	0	0	0	0	0	0
1	3	0	0	0	0	0
0	0	0	0	0	0	0
0	0	0	0	0	0	0
0	1	0	0	0	0	0

0	0	0	0	0	0	0
0	1	0	0	0	0	0
0	2	0	0	0	0	0
0	0	2	0	0	0	0
0	0	1	0	0	0	0
2	0	0	0	0	0	0
0	0	2	0	0	0	0
0	0	0	0	0	0	0
0	0	0	0	0	0	0
0	0	0	0	0	0	0
1	0	0	0	0	0	0
0	0	0	0	0	0	0
0	0	0	0	0	0	0
1	0	0	0	0	0	0
0	0	0	0	0	0	0
0	0	0	0	0	0	0
0	0	1	0	0	0	0
0	0	0	0	0	0	0
0	0	0	0	0	0	0
0	1	1	0	0	0	0
1	0	1	0	0	0	0
0	0	0	0	0	0	0
0	1	0	0	0	0	0
0	0	0	0	0	0	0
0	0	0	0	0	0	0
1	0	0	0	0	0	0
0	0	0	0	0	0	0
0	0	0	0	0	0	0
0	0	0	0	0	0	0
0	0	0	0	0	0	0
0	0	0	0	0	0	0
0	0	0	1	0	0	0
0	0	0	0	0	0	0
1	0	0	0	0	0	0
0	0	0	0	0	0	0
0	0	0	0	0	0	0
0	0	0	0	0	0	0
0	1	0	0	0	0	0
0	0	0	0	0	0	0
0	0	0	0	0	0	0
0	1	0	0	0	0	0
1	0	0	0	0	0	0
0	0	0	0	0	0	0
1	0	0	0	0	0	0
0	0	1	0	0	0	0
0	0	0	0	0	0	0
0	0	0	0	0	0	0
0	0	0	0	0	0	0
0	0	0	0	0	0	0
0	0	0	0	0	0	0

0	0	0	0	0	0	0
0	0	0	0	0	0	0
0	0	0	0	0	0	0
0	0	0	0	0	0	0
0	0	0	0	0	0	0
2	0	0	0	0	0	0
0	0	0	0	0	0	0
1	0	0	0	0	0	0
0	0	1	0	0	0	0
0	0	0	0	0	0	0
0	0	0	0	0	0	0
0	0	0	0	0	0	0
0	0	0	0	0	0	0
0	0	0	0	0	0	0
0	0	0	0	0	0	0
0	0	0	0	0	0	0
0	0	0	0	0	0	0
0	0	0	0	0	0	0
0	0	0	0	0	0	0
0	0	0	0	0	0	0
0	0	1	0	0	0	0
1	0	0	0	0	0	0
0	0	0	0	0	0	0
0	0	0	0	0	0	0
1	0	0	0	0	0	0
0	0	0	0	0	0	0
1	3	0	0	0	0	0
0	0	0	0	0	0	0
0	1	0	0	0	0	0
0	0	0	0	0	0	0
0	0	0	0	0	0	0
0	0	0	0	0	0	0
0	0	0	0	0	0	0
0	0	0	0	0	0	0
1	0	0	0	0	0	0
1	0	0	0	0	0	0
1	0	0	0	0	0	0
1	0	0	0	0	0	0
1	0	0	0	0	0	0
1	0	0	0	0	0	0
1	0	0	0	0	0	0
1	1	0	0	0	0	0
1	0	0	0	0	0	0
1	0	0	0	0	0	0
1	0	0	0	0	0	0
1	0	0	0	0	0	0
0	0	0	0	0	0	0
0	0	1	0	0	0	0
0	0	0	0	0	0	0
2	0	0	0	0	0	0

0	0	0	0	0	0	0
0	0	0	0	0	0	0
0	0	0	0	0	0	0
0	0	0	0	0	0	0
0	0	0	0	0	0	0
1	0	0	0	0	0	0
1	0	1	0	0	0	0
1	0	0	0	0	0	0
0	0	0	0	0	0	0
1	0	0	0	0	0	0
1	0	0	0	0	0	0
0	0	0	0	0	0	0
0	0	1	0	0	0	0
0	0	2	0	0	0	0
0	0	0	0	0	0	0
0	0	0	0	0	0	0
0	0	0	0	0	0	0
0	0	0	0	0	0	0
0	0	0	0	0	0	0
0	0	0	0	0	0	0
0	0	0	0	0	0	0
0	0	0	0	0	0	0
0	0	0	0	0	0	0
0	0	0	0	0	0	0
0	0	0	0	0	0	0
1	0	1	0	0	0	0
0	0	0	0	0	0	0
0	0	1	0	0	0	0

nX	nR03	nR04	nR05	nR06	nR07	nR08
0	0	0	1	0	0	0
0	0	0	0	1	0	0
0	0	0	0	3	0	0
0	0	0	0	1	0	0
0	0	0	1	1	0	0
0	0	0	0	0	0	0
0	0	0	1	2	0	0
0	0	0	1	1	0	0
1	0	0	1	2	1	0
0	0	0	0	1	0	0
0	0	0	0	3	0	0
2	0	0	1	2	0	0
0	0	0	0	2	1	0
1	0	0	0	2	0	0
0	0	1	1	1	1	0
0	0	1	1	1	1	0
0	0	0	1	1	0	0
7	0	0	1	3	0	0
0	0	0	0	1	0	0
0	0	0	1	2	1	0
0	0	0	1	2	0	0
0	0	0	2	1	0	0
1	0	0	1	2	0	0
0	0	1	1	1	1	0
0	0	0	1	2	0	0
1	0	0	1	3	0	0
0	1	0	0	1	0	0
0	0	0	4	0	0	0
0	0	0	1	2	0	0
0	0	0	0	1	0	0
0	0	0	0	4	0	0
1	0	0	3	4	0	0
0	0	0	1	1	0	0
0	0	0	0	2	0	0
0	1	0	1	6	0	1
0	0	0	1	3	0	0
0	0	1	0	4	0	1
0	0	0	1	1	0	0
0	0	0	1	2	0	0
0	0	0	1	4	0	0
0	0	0	2	3	0	0
0	0	0	0	2	1	0
0	0	0	0	5	0	0
1	0	0	1	2	0	0
0	0	0	1	3	0	0
1	0	1	0	2	0	1
0	0	1	0	2	0	1
0	0	1	1	2	0	1
0	0	1	2	1	0	1

0	0	1	2	1	0	1
0	0	1	1	1	0	1
0	0	1	1	3	0	1
0	0	1	1	1	0	1
0	0	1	1	1	0	1
0	0	1	0	2	0	1
0	0	1	0	3	0	1
0	0	1	1	2	0	1
0	0	1	1	1	0	1
0	0	1	1	2	0	1
0	0	1	1	1	0	1
3	0	0	1	2	0	0
0	0	1	0	2	0	1
0	0	1	1	2	0	1
0	0	1	1	1	0	1
0	0	1	0	2	0	1
0	0	0	1	1	1	0
1	0	0	0	3	0	0
1	0	0	0	2	0	0
1	0	0	0	2	0	0
1	0	0	0	3	0	0
1	0	0	0	1	0	0
1	0	0	0	3	0	0
1	0	0	1	2	0	0
1	0	0	1	2	0	0
0	0	0	1	0	0	0
0	0	0	1	2	0	0
3	0	0	0	3	0	0
0	0	0	1	2	0	0
1	1	0	0	3	0	0
1	0	0	1	1	0	0
1	0	0	0	1	0	0
1	0	0	1	2	0	0
2	0	0	1	1	0	0
1	0	0	0	3	1	0
2	0	0	0	1	0	0
0	0	0	0	2	0	0
0	0	0	0	2	1	0
1	0	0	1	3	0	0
1	0	0	0	2	1	0
2	0	0	0	2	0	0
2	0	1	2	1	1	0
0	0	0	2	1	0	0
2	0	0	0	2	0	0
0	0	0	2	3	0	0
0	0	0	2	6	0	0
0	0	0	0	2	1	0
0	0	0	0	2	0	0
0	0	0	0	2	0	0
0	0	1	0	4	0	2

0	0	0	0	2	0	0
1	0	0	2	3	0	0
0	0	0	0	4	0	0
0	0	0	0	1	0	0
0	0	0	0	0	0	0
0	0	0	1	2	0	0
0	0	0	2	5	0	0
0	0	0	1	0	0	0
0	0	0	0	2	0	0
0	0	0	0	2	0	0
0	0	0	0	3	0	0
1	0	0	1	2	0	0
0	0	0	1	3	0	0
6	0	0	0	2	0	0
2	0	0	2	1	0	0
1	0	0	1	2	0	0
1	0	0	1	1	1	0
1	0	0	0	1	0	0
3	0	0	0	4	0	0
1	0	0	0	2	0	0
0	0	0	0	0	0	0
5	0	0	1	3	0	0
1	0	0	1	1	0	0
0	0	0	1	3	0	0
0	0	0	0	1	0	0
0	0	0	1	2	1	0
0	0	0	1	1	0	0
0	0	0	0	1	0	0
0	0	0	0	3	0	0
0	0	0	1	2	0	0
1	0	0	0	3	0	0
0	0	0	1	3	0	1
4	0	0	0	2	0	0
1	0	0	0	2	0	0
0	0	0	1	4	0	1
0	0	0	0	1	0	0
0	0	0	0	5	0	0
1	0	0	1	2	0	0
0	0	0	1	2	0	0
0	0	0	3	2	0	0
0	0	0	2	0	0	1
0	0	0	2	0	0	1
0	0	0	1	2	0	0
1	0	0	0	2	0	0
0	0	0	0	2	0	0
0	0	0	1	1	0	0
3	0	0	1	2	0	0
0	0	0	1	2	0	0
0	0	0	1	0	0	0
1	0	0	0	4	0	0

0	0	0	0	1	0	0
1	0	0	1	2	0	0
2	0	0	0	3	0	0
2	0	0	0	2	1	0
1	0	0	2	2	0	0
0	0	0	1	2	0	0
2	0	0	0	1	0	0
0	0	0	0	2	0	0
0	0	0	0	3	1	0
0	0	0	0	2	0	0
0	0	0	1	1	0	0
0	0	0	0	0	0	0
0	0	0	0	2	0	0
0	0	1	1	1	1	0
0	0	0	0	3	0	0
0	0	0	1	3	0	0
1	0	0	0	1	0	0
0	0	0	0	1	0	0
0	0	0	1	0	0	0
2	0	0	1	2	1	0
1	1	0	0	4	0	0
0	0	0	1	4	0	1
1	1	0	1	3	0	0
0	0	0	0	1	0	0
0	0	0	0	2	0	0
0	0	1	1	2	1	0
0	0	0	0	2	0	0
0	1	0	1	4	0	1
0	0	0	1	4	0	1
0	0	0	0	2	0	0
0	0	0	0	1	0	0
1	0	0	1	4	0	0
0	0	0	0	2	0	0
0	0	0	0	2	0	0
0	0	0	0	2	0	0
0	0	0	2	0	0	0
0	0	0	0	1	0	0
1	0	0	0	3	0	0
0	0	0	0	2	1	0
0	0	0	0	4	0	0
1	0	0	0	4	0	0
0	0	0	1	2	0	0
0	0	0	2	2	0	0
0	0	1	2	1	1	0
1	0	0	0	2	1	0
0	0	0	1	4	0	1
0	0	0	1	2	0	0
0	0	0	1	5	0	0
0	0	0	0	1	0	0
0	0	0	0	1	0	0

0	0	0	0	3	0	0
0	0	0	0	2	0	0
0	0	0	1	2	0	0
0	0	0	1	2	0	0
0	0	0	1	1	0	0
0	0	0	0	4	0	0
0	0	0	0	3	1	0
0	0	0	1	2	0	0
1	0	0	1	1	0	0
0	0	0	0	1	0	0
0	0	0	0	2	0	0
0	0	0	1	3	0	0
0	0	0	1	3	0	0
0	0	0	1	3	0	0
0	0	0	0	1	0	0
0	0	0	0	1	0	0
0	0	0	0	2	0	0
0	0	0	0	2	0	0
0	0	0	0	1	0	0
0	0	0	0	2	0	0
1	0	0	0	2	0	0
0	0	0	0	3	1	0
0	0	0	0	5	0	0
0	0	0	0	5	0	0
0	0	0	1	0	0	0
0	0	0	0	3	0	0
3	0	0	1	1	0	0
0	0	0	0	1	0	0
1	0	0	1	4	0	0
0	0	0	2	1	0	0
0	0	0	2	4	0	0
0	0	0	0	2	0	0
0	0	0	0	1	0	0
0	1	0	1	3	1	1
0	0	0	1	3	0	0
0	0	0	0	1	0	0
0	0	0	1	2	0	0
0	0	0	1	1	0	0
0	0	0	1	2	0	0
0	0	0	1	3	0	0
0	0	0	1	1	0	0
1	0	0	1	2	0	0
0	0	0	1	1	0	0
0	0	0	1	1	0	0
0	0	0	1	1	0	0
0	0	0	0	2	0	0
0	0	0	1	1	0	0
1	0	0	0	2	1	0
0	0	0	1	1	0	0
0	0	0	1	2	0	0

0	0	0	1	3	0	0
0	0	0	0	1	0	0
0	0	0	1	3	0	0
0	0	0	0	4	0	0
0	0	0	1	1	0	0
0	0	0	0	1	0	0
1	0	0	1	2	0	0
0	0	0	1	1	0	0
0	0	0	0	2	0	0
0	0	0	0	1	1	0
0	0	0	0	1	0	0
0	0	0	0	2	0	0
1	0	0	1	3	0	0
2	0	0	1	2	1	0
0	0	0	0	2	0	0
0	0	0	0	3	0	0
0	0	0	0	0	0	0
0	0	0	1	2	0	0
0	0	0	1	5	0	0
0	0	0	0	2	0	0
0	0	0	3	5	0	1
0	0	0	1	4	0	0
0	0	0	0	3	0	0
0	0	0	1	2	0	0
0	0	0	1	1	0	0
1	0	0	2	3	0	0
0	0	0	1	2	0	0
1	0	0	1	1	0	0

nR09	nR10	nR11	nR12	nBnz	ZM1	ZM1V
0	0	0	0	0	80	272
0	0	0	0	1	110	310
0	1	0	0	2	136	444
0	0	0	0	1	50	162
1	0	0	0	0	80	282
0	0	0	0	0	68	234.617
0	0	0	0	1	152	406
1	0	0	0	0	52	184
0	1	1	0	2	122	289.605
0	0	0	0	1	80	190
0	0	0	0	0	206	612
1	0	0	0	2	156	362.048
0	0	2	0	2	108	204
0	0	0	0	1	136	397.605
0	0	0	0	1	138	378.444
0	0	0	0	1	132	346.444
0	0	0	0	1	72	190
0	0	0	0	2	200	780
0	0	0	0	1	86	238
0	0	0	0	1	110	256
1	1	0	0	1	120	316
1	0	0	0	0	102	346.444
1	0	0	0	2	112	307.605
0	0	0	0	1	126	332.444
0	0	0	0	2	132	260
1	2	0	0	0	168	394
0	0	0	0	1	104	238
0	0	0	0	0	226	614
1	0	0	0	2	114	240
0	0	0	0	1	102	268
0	0	0	0	2	204	592.444
4	2	1	1	1	256	555.067
1	0	0	0	1	98	214
0	0	0	0	1	102	208
4	5	4	1	1	220	386
0	0	0	0	0	148	344
0	3	0	1	1	144	244
1	0	0	0	0	76	248
1	0	0	0	0	160	266
2	2	0	0	1	154	410
1	0	0	0	3	178	506
0	0	2	0	2	96	240
0	4	0	0	0	242	502
2	0	0	0	2	104	253.605
2	0	0	0	3	154	402
0	0	0	0	1	128	356.049
0	0	0	0	1	134	388.444
0	0	0	0	1	166	492.889
0	0	0	0	0	156	477.333

0	0	0	0	0	174	490.889
0	0	0	0	0	156	522.889
0	0	0	0	1	236	722.889
0	0	0	0	0	156	522.889
0	0	0	0	0	148	468.889
0	0	0	0	1	142	406.444
0	0	0	0	1	194	600.889
0	0	0	0	0	198	612.889
0	0	0	0	0	132	422.889
0	0	0	0	0	192	621.333
0	0	0	0	0	150	534.444
0	0	0	0	2	142	472.444
0	0	0	0	1	128	356.444
0	0	0	0	0	150	412.889
0	0	0	0	0	136	416.889
0	0	0	0	0	128	346.444
0	0	0	0	0	118	226.444
0	0	0	0	2	134	329.605
0	1	0	0	1	94	286.494
0	0	0	0	1	92	189.605
0	2	0	0	2	110	208.049
0	0	0	0	1	80	234.049
0	2	0	0	2	110	204.049
1	0	0	0	2	124	334.049
1	0	0	0	1	98	241.605
0	0	0	0	0	74	210.444
0	0	0	0	2	126	384
0	1	0	0	3	134	380
1	1	0	0	1	104	358
0	1	0	0	1	134	384
0	0	0	0	0	136	306.049
0	0	0	0	1	68	199.605
1	0	0	0	2	132	371.605
0	0	0	0	1	70	157.21
0	0	2	0	2	126	267.605
0	0	0	0	0	64	142.519
0	0	0	0	2	88	226.444
0	0	2	0	2	102	198
1	2	0	0	0	168	394
0	0	1	0	2	106	259.605
0	0	0	0	2	94	241.21
0	0	0	0	1	170	453.654
1	0	0	0	0	92	284
0	0	0	0	2	92	350
1	2	0	0	0	164	298
1	2	0	0	0	314	712
0	0	1	0	2	148	392.444
0	0	0	0	2	90	202
0	0	0	0	1	124	270
0	1	0	1	2	326	882

0	0	0	0	2	144	392.889
2	0	0	0	2	166	391.605
0	3	0	0	1	184	518
0	0	0	0	1	104	382
0	0	0	0	0	54	126
2	0	0	0	1	114	274
2	2	0	0	2	242	720
0	0	0	0	0	94	269.333
0	0	0	0	2	92	266
0	0	0	0	2	88	252
0	0	0	0	2	124	262
0	0	0	0	2	114	284.049
1	2	0	0	0	158	276
0	0	0	0	1	140	594
0	0	0	0	1	116	408
1	0	0	0	2	98	298
0	1	1	0	1	118	390
0	0	0	0	0	42	210
0	2	0	0	2	160	442.444
0	0	0	0	2	90	272
0	0	0	0	0	30	163.309
1	2	0	0	1	218	568.444
0	0	0	0	1	108	340.049
1	2	0	0	0	208	422
0	0	0	0	0	56	130
2	1	1	1	1	122	274
1	0	0	0	0	90	316
0	0	0	0	1	86	220
0	0	0	0	0	164	408
0	0	0	0	1	174	454.444
0	0	0	0	2	166	442.049
1	0	0	0	1	128	276
0	0	0	0	2	138	509.605
0	1	0	0	1	94	272.494
3	3	3	1	1	132	284
0	0	0	0	1	70	170
0	0	0	0	2	194	476
1	0	0	0	2	132	371.605
1	0	0	0	2	112	304
0	0	0	0	2	174	410
0	0	0	0	0	82	382
0	0	0	0	0	68	274
1	0	0	0	1	142	422
0	0	0	0	2	104	289.605
0	0	0	0	2	94	268
0	0	0	0	0	76	232.444
1	0	0	0	1	132	460.444
0	0	0	0	2	112	320
0	0	0	0	0	56	164
0	3	0	1	1	146	434

0	0	0	0	1	78	184
0	0	0	0	1	124	400
0	1	0	0	1	134	434
0	0	1	0	2	112	287.21
0	0	0	0	2	156	387.605
0	1	0	0	1	126	358.889
0	0	0	0	1	86	209.21
0	0	0	0	1	90	212
0	0	3	0	1	112	228
0	0	0	0	1	90	196
1	0	0	0	0	52	150.444
0	0	0	0	0	36	118
0	0	0	0	2	114	236
0	0	0	0	1	142	416.444
0	1	0	0	1	168	544
1	2	0	0	0	160	338
0	0	0	0	1	92	239.605
0	0	0	0	1	84	218
0	0	0	0	0	56	222
0	1	1	0	2	128	329.605
0	1	0	0	3	220	458.049
3	3	3	1	1	132	276
1	1	0	0	1	166	450
0	0	0	0	1	94	254
0	1	0	0	1	112	264
0	1	0	0	2	162	426.444
0	1	0	0	0	88	276
3	3	3	1	1	160	304
3	3	3	1	1	142	300
0	1	0	0	2	86	242
0	0	0	0	1	78	202
2	2	1	0	1	176	411.067
0	0	0	0	1	126	434
0	0	0	0	2	106	358.444
0	0	0	0	1	130	438
0	0	0	0	0	86	366
0	0	0	0	1	56	172
0	1	0	0	1	122	372
0	0	2	0	2	102	194
0	1	0	0	2	234	712
0	3	0	1	1	146	434
1	0	0	0	1	126	346.444
2	0	0	0	1	118	276
0	0	0	0	1	158	438.444
0	0	1	0	2	106	279.605
3	3	3	1	1	144	328
0	0	0	0	2	124	330
1	2	0	0	0	238	386
0	0	0	0	0	78	228
0	0	0	0	1	58	178

0	0	0	0	1	94	142
0	0	0	0	1	88	270
0	0	0	0	2	118	298
0	0	0	0	2	102	274
1	0	0	0	1	88	214
0	2	0	0	2	170	362.889
0	0	2	0	1	140	368
0	0	0	0	2	132	378.444
0	0	0	0	1	86	213.605
0	0	0	0	1	86	242
0	1	0	0	0	148	382
0	1	0	0	1	150	444
1	2	0	0	0	154	350
1	2	0	0	0	136	214
0	0	0	0	1	76	188
0	0	0	0	1	76	208
0	0	0	0	2	116	292
0	1	0	0	2	92	216
0	0	0	0	1	62	122
0	1	0	0	2	92	216
0	0	0	0	1	86	205.605
0	0	2	0	2	140	320.444
0	1	0	0	1	132	286
0	1	0	0	1	132	286
0	0	0	0	0	94	284.444
0	0	0	0	2	164	402
1	0	0	0	1	80	324.444
0	0	0	0	0	88	294.617
1	1	0	0	1	166	426
1	0	0	0	1	104	248
1	2	0	0	0	220	374
0	0	0	0	1	98	204
0	0	0	0	1	46	170
0	0	0	0	1	124	302
1	0	0	0	1	178	472.444
0	0	0	0	1	86	222.444
0	0	0	0	1	136	292.444
0	0	0	0	1	88	268.444
0	0	0	0	2	116	310.444
0	0	0	0	3	152	390.444
0	0	0	0	1	94	276.444
1	0	0	0	2	132	368.444
0	0	0	0	1	116	316.444
1	0	0	0	1	102	244.444
0	0	0	0	1	90	250.444
0	0	0	0	2	136	376.444
1	0	0	0	1	104	262
0	0	1	0	2	112	289.605
1	0	0	0	0	98	315.309
1	0	0	0	0	120	350.889

0	1	0	0	1	150	422
0	0	0	0	1	78	198
1	2	0	0	0	126	204
0	3	0	0	1	186	520
1	0	0	0	0	70	238
0	0	0	0	0	78	192.444
1	0	0	0	1	90	160.049
0	0	0	0	0	104	272.444
0	0	0	0	2	118	336
0	0	0	0	1	104	274.444
0	0	0	0	1	84	238.444
0	0	0	0	1	96	202
1	0	0	0	1	138	325.605
0	1	1	0	2	128	297.21
0	0	0	0	1	104	322
0	0	0	0	1	142	380
0	0	0	0	0	38	104
0	0	0	0	2	160	434
1	2	0	0	0	230	394
0	0	0	0	2	160	412
5	2	2	1	2	338	784
3	3	1	1	1	152	318
0	1	0	0	2	120	340
1	0	0	0	1	120	342
0	0	0	0	0	96	324
2	0	0	0	2	156	338.049
1	0	0	0	1	122	294
0	0	0	0	1	102	285.605

ZM2	ZM2V	Qindex	SNar	HNar	GNar	Xt
91	238	9	10.057	1.619	1.807	0.315
120	276	10	14.622	1.674	1.839	0.262
161	419	19	17.918	1.814	1.992	0.236
53	146	6	6.762	1.692	1.849	0.385
93	258	11	11.038	1.846	1.993	0.301
78	64.222	9	6.238	1.366	1.561	0.4
180	399	19	20.86	1.865	2.004	0.219
61	182	9	7.455	2	2.107	0.366
148	335.111	20	17.107	2.063	2.176	0.242
86	176	7	11.326	1.742	1.876	0.297
247	495	26	25.608	1.69	1.897	0.198
184	376.244	19	21.383	1.842	1.993	0.216
126	228	15	15.603	2	2.102	0.253
160	364.111	15	18.205	1.75	1.916	0.234
168	346.667	22	16.531	1.714	1.937	0.246
161	320.667	21	16.126	1.745	1.958	0.249
85	202	11	9.652	1.826	1.993	0.322
233	627	29	25.255	1.79	1.979	0.199
91	207	8	11.731	1.701	1.854	0.292
130	241	16	15.315	1.938	2.074	0.256
148	336	19	15.145	1.784	1.991	0.257
122	341.333	16	13.929	1.932	2.081	0.268
132	317.111	17	15.027	1.881	2.045	0.258
152	303.667	20	15.72	1.781	1.981	0.252
148	263	15	19.761	2	2.079	0.225
221	362	31	18.781	1.714	1.956	0.231
113	213	11	15.197	1.886	1.995	0.257
270	612	30	28.786	1.755	1.953	0.186
134	270	16	16.008	1.941	2.07	0.25
107	227	8	14.504	1.747	1.879	0.263
238	534.333	27	27.216	1.857	2.009	0.192
326	594.037	45	31.559	1.87	2.083	0.178
112	225	14	12.425	1.74	1.923	0.284
117	213	12	14.216	1.826	1.968	0.265
293	434	45	25.83	1.915	2.138	0.197
173	336	21	21.148	2.036	2.128	0.217
183	272	27	19.068	2.087	2.213	0.229
94	245	13	9.077	1.68	1.912	0.332
188	267	23	19.879	1.748	1.94	0.224
195	431	28	19.997	1.987	2.158	0.224
214	540	26	25.255	2.041	2.15	0.199
115	263	15	13.523	2	2.12	0.272
306	484	42	27.216	1.708	1.942	0.192
125	268.111	17	13.641	1.869	2.05	0.271
180	401	20	22.652	2.045	2.128	0.21
155	335.444	19	16.531	1.8	1.991	0.246
162	362.333	20	16.937	1.765	1.969	0.243
200	470.333	24	22.194	1.879	2.046	0.212
186	448.667	23	21.096	1.912	2.07	0.218

210	452	26	22.482	1.837	2.019	0.211
186	472	21	20.115	1.765	1.955	0.223
285	679.333	33	30.865	1.833	2.017	0.18
186	468	21	20.115	1.765	1.955	0.223
179	411	21	18.898	1.778	1.964	0.23
171	382.333	20	18.323	1.78	1.971	0.234
232	478.667	28	24.679	1.793	1.985	0.201
236	566	28	25.372	1.798	1.985	0.199
159	390	19	17.224	1.807	1.992	0.241
231	573	27	24.797	1.8	1.991	0.201
179	479.333	20	19.709	1.794	1.973	0.225
166	373.667	22	17.394	1.753	1.952	0.24
155	336.333	19	16.531	1.8	1.991	0.246
179	374	22	20.69	1.953	2.094	0.22
161	362	19	17.918	1.814	1.992	0.236
155	323.333	19	16.531	1.8	1.991	0.246
134	212	14	17.97	2.057	2.114	0.236
152	306.111	16	19.474	1.952	2.057	0.227
111	152.778	16	10.633	1.645	1.869	0.307
103	197.111	11	13.118	1.869	1.995	0.276
129	226.444	16	15.315	1.938	2.074	0.256
88	152.444	9	10.227	1.659	1.825	0.313
129	222.444	16	15.315	1.938	2.074	0.256
151	279.778	21	14.909	1.76	1.969	0.259
118	250.111	16	13.235	1.929	2.086	0.275
80	198.667	6	10.92	1.789	1.901	0.303
145	358	16	18.087	1.948	2.062	0.235
154	342	18	18.375	1.891	2.027	0.233
127	345	17	13.641	1.869	2.05	0.271
164	366	22	17.918	1.946	2.11	0.236
161	267	17	16.937	1.67	1.873	0.243
75	185.111	9	8.148	1.6	1.79	0.35
158	366.111	19	17.224	1.807	1.992	0.241
80	170.222	10	9.94	1.909	2.034	0.317
150	304.111	20	17.8	2.06	2.168	0.237
72	70.778	7	8.723	1.732	1.865	0.339
102	169.333	13	11.326	1.774	1.947	0.297
120	224	14	15.197	2.069	2.138	0.257
221	362	31	18.781	1.714	1.956	0.231
126	275.111	16	14.622	1.935	2.077	0.262
107	239.222	12	12.83	1.81	1.965	0.279
211	449.889	28	20.52	1.756	1.982	0.221
111	272	15	12.83	2	2.127	0.279
107	298	13	11.849	1.742	1.931	0.291
210	302	31	20.167	1.917	2.11	0.223
390	662	52	39.589	1.889	2.082	0.159
173	368.667	19	19.997	1.832	1.993	0.224
100	198	10	13.405	1.932	2.025	0.273
146	280	15	16.296	1.754	1.919	0.248
403	827	50	38.778	1.736	1.951	0.161

155	263.667	17	18.663	1.74	1.903	0.231
200	418.111	26	23.175	2.045	2.165	0.208
237	493	31	21.044	1.677	1.93	0.218
120	344	11	12.948	1.61	1.801	0.278
56	92	2	7.742	1.615	1.739	0.359
140	282	18	14.909	1.867	2.034	0.259
302	666	40	31.964	1.969	2.141	0.177
99	188.667	10	12.019	1.655	1.824	0.288
103	246	11	13.118	1.869	1.995	0.276
99	245	11	12.425	1.862	1.994	0.284
142	271	15	18.375	2	2.085	0.233
132	282.444	16	16.008	1.941	2.07	0.25
195	289	28	18.781	1.79	2.005	0.231
153	437	17	17.97	1.732	1.9	0.236
135	371	17	15.89	1.927	2.059	0.251
119	277	16	13.235	1.929	2.086	0.275
143	368	18	15.433	1.833	2.017	0.255
46	164	6	5.375	1.636	1.817	0.431
188	385.333	23	21.959	1.946	2.079	0.213
104	248	12	12.137	1.8	1.963	0.287
30	55.111	4	2.485	1.254	1.426	0.634
258	439.667	30	27.909	1.802	1.975	0.189
124	259.778	15	13.523	1.714	1.904	0.272
260	406	33	24.274	1.701	1.927	0.203
62	106	7	7.337	1.694	1.843	0.369
153	283	22	16.008	1.984	2.143	0.25
105	282	12	12.137	1.8	1.963	0.287
95	201	10	10.633	1.624	1.805	0.307
196	358	23	20.403	1.73	1.931	0.221
201	370.333	22	22.77	1.782	1.954	0.21
189	357.444	20	22.652	1.842	1.987	0.21
155	300	21	17.512	2	2.141	0.239
154	417.111	16	18.087	1.741	1.908	0.235
111	141.111	16	10.633	1.645	1.869	0.307
173	302	27	16.819	2.049	2.228	0.244
77	157	8	8.959	1.636	1.817	0.334
224	502	26	28.027	2.037	2.133	0.189
158	368.111	19	17.224	1.807	1.992	0.241
134	302	17	15.027	1.881	2.045	0.258
210	455	26	24.731	2.076	2.166	0.201
95	315	12	10.751	1.778	1.958	0.305
80	222	11	8.959	1.814	1.992	0.334
171	420	20	18.323	1.78	1.971	0.234
119	276.111	13	13.929	1.775	1.941	0.268
108	253	12	12.83	1.81	1.965	0.279
88	208.333	11	10.345	1.837	1.993	0.311
152	375	19	17.394	1.84	2.005	0.24
131	328	15	16.296	2	2.097	0.248
64	141	7	7.167	1.636	1.817	0.374
180	412	24	18.729	1.857	2.055	0.231

87	180	8	10.345	1.672	1.838	0.311
144	353	17	17.107	1.895	2.04	0.242
163	392	20	16.937	1.765	1.969	0.243
133	296.222	17	15.027	1.881	2.045	0.258
185	423.111	21	22.364	2	2.107	0.211
153	286.333	20	15.72	1.781	1.981	0.252
95	196.111	8	11.731	1.701	1.854	0.292
106	203	12	12.307	1.846	1.981	0.285
136	256	19	16.008	2.143	2.226	0.25
104	198	12	12.137	1.8	1.963	0.287
61	154.667	9	7.455	2	2.107	0.366
36	110	3	3.989	1.385	1.558	0.501
135	244	14	15.197	1.781	1.936	0.257
175	377.667	22	17.224	1.724	1.94	0.241
193	518	21	22.194	1.784	1.959	0.212
206	324	29	18.493	1.751	1.984	0.233
104	227.111	9	12.137	1.667	1.835	0.287
89	185	7	12.019	1.754	1.882	0.288
64	195	7	7.167	1.636	1.817	0.374
156	352.111	21	17.512	2	2.141	0.239
256	480.444	31	29.701	1.922	2.064	0.183
173	299	27	16.819	2.049	2.228	0.244
207	435	28	21.907	1.955	2.128	0.214
104	238	10	11.849	1.622	1.808	0.291
126	240	15	14.216	1.725	1.908	0.265
200	413.667	26	20.403	1.822	2.021	0.221
105	268	13	11.156	1.729	1.928	0.299
211	342	33	20.285	2.083	2.251	0.222
187	321	28	18.087	2.029	2.196	0.235
100	231	12	11.444	1.789	1.96	0.296
86	196	10	9.246	1.587	1.782	0.329
218	425.037	29	23.175	1.948	2.112	0.208
151	395	16	15.838	1.685	1.884	0.251
118	288.333	14	13.811	1.762	1.93	0.269
154	402	16	16.531	1.696	1.889	0.246
98	331	12	11.444	1.789	1.96	0.296
63	152	7	7.167	1.636	1.817	0.374
147	347	18	16.126	1.84	2.016	0.249
120	220	14	15.197	2.069	2.138	0.257
279	672	32	29.074	1.731	1.936	0.185
180	412	24	18.729	1.857	2.055	0.231
150	324	18	16.819	1.846	2.015	0.244
145	299	20	16.414	2.066	2.185	0.247
195	429.667	26	19.709	1.816	2.022	0.225
125	286.111	16	14.622	1.935	2.077	0.262
191	341	29	17.8	1.971	2.168	0.237
148	339	17	17.107	1.895	2.04	0.242
296	400	40	29.701	1.878	2.064	0.183
94	205	10	9.246	1.587	1.782	0.329
62	164	6	8.148	1.733	1.872	0.35

112	164	14	13.981	2.139	2.174	0.267
107	254	13	11.326	1.774	1.947	0.297
141	313	16	16.701	1.944	2.067	0.245
124	280	16	14.099	1.983	2.1	0.266
99	212	11	12.425	1.862	1.994	0.284
202	297.333	24	23.057	1.91	2.056	0.208
167	375	21	19.592	2	2.124	0.226
154	312.667	19	17.394	1.84	2.005	0.24
100	208.111	12	11.444	1.789	1.96	0.296
91	215	8	11.731	1.701	1.854	0.292
169	325	17	19.016	1.698	1.885	0.229
180	448	22	20.69	1.953	2.094	0.22
198	329	28	18.087	1.783	2.005	0.235
172	223	25	16.701	1.865	2.067	0.245
83	180	7	10.633	1.729	1.869	0.307
83	192	7	10.633	1.729	1.869	0.307
128	272	11	17.277	1.899	1.996	0.241
103	214	11	13.118	1.869	1.995	0.276
71	130	8	7.573	1.592	1.791	0.363
103	214	11	13.118	1.869	1.995	0.276
100	231.111	12	11.444	1.789	1.96	0.296
164	315.333	19	20.86	2.104	2.165	0.219
161	298	21	18.205	2	2.135	0.234
161	298	21	18.205	2	2.135	0.234
100	254.667	8	13.118	1.726	1.868	0.276
188	392	19	22.77	1.85	1.994	0.21
90	262.333	13	9.94	1.748	1.94	0.317
102	130.222	13	9.416	1.534	1.74	0.326
200	435	26	23.175	2.045	2.165	0.208
120	264	15	14.909	2	2.107	0.259
276	376	37	28.315	1.94	2.107	0.188
113	209	12	13.523	1.818	1.966	0.272
51	147	6	6.068	1.667	1.835	0.406
153	288	21	16.819	2	2.148	0.244
215	422	26	23.175	1.842	2.018	0.208
92	152.333	10	10.633	1.624	1.805	0.307
159	280.667	17	19.356	1.94	2.048	0.227
100	209.333	13	11.326	1.774	1.947	0.297
135	268.333	17	15.89	1.927	2.059	0.251
181	375	21	21.671	2	2.111	0.215
109	221.333	14	11.731	1.728	1.919	0.292
156	326.333	19	17.224	1.807	1.992	0.241
134	236.667	15	14.909	1.736	1.912	0.259
116	185	14	13.118	1.752	1.927	0.276
104	227.667	12	12.137	1.8	1.963	0.287
153	297.667	15	18.375	1.778	1.928	0.233
117	266	11	15.197	1.886	1.995	0.257
134	299.111	17	15.027	1.881	2.045	0.258
111	243	14	12.425	1.74	1.923	0.284
146	278.667	19	15.315	1.821	2.006	0.256

180	416	22	20.69	1.953	2.094	0.22
84	181	10	9.246	1.587	1.782	0.329
160	213	24	15.603	1.909	2.102	0.253
239	500	32	20.926	1.67	1.923	0.219
86	233	12	8.671	1.733	1.948	0.34
94	183.667	10	9.246	1.587	1.782	0.329
105	169.778	14	13.118	2.082	2.163	0.276
115	233.667	13	14.099	1.813	1.957	0.266
130	308	12	16.989	1.852	1.973	0.243
116	199.333	13	14.099	1.813	1.957	0.266
92	157.333	9	10.92	1.674	1.834	0.303
112	196	13	12.712	1.795	1.952	0.28
162	348.111	20	19.879	2.053	2.148	0.224
156	345.222	21	17.512	2	2.141	0.239
121	313	13	13.929	1.775	1.941	0.268
166	369	18	19.592	1.867	2.013	0.226
39	80	2	4.97	1.5	1.644	0.449
186	437	19	22.076	1.846	1.993	0.213
285	403	38	29.413	1.912	2.086	0.184
187	389	17	21.266	1.744	1.905	0.217
439	812	58	42.361	1.911	2.103	0.154
194	358	27	20.285	2.039	2.182	0.222
142	333	17	16.414	1.89	2.041	0.247
144	362	17	16.414	1.89	2.041	0.247
113	286	13	12.542	1.754	1.935	0.282
187	354.111	25	22.076	2.1	2.2	0.213
144	316	18	16.126	1.84	2.016	0.249
119	274.111	14	12.948	1.714	1.911	0.278

Dz	Ram	Pol	LPRS	VDA	MSD	SMTI
39	6	23	69.813	62.353	0.266	2048
53	7	31	116.313	130.667	0.281	6074
58.5	10	44	124.439	122.462	0.217	6702
24.5	3	11	37.263	30.182	0.328	679
37.5	5	22	64.183	56.625	0.28	1899
34.833	6	23	51.365	40.143	0.252	1010
66	9	49	153.983	173.267	0.234	10854
23	3	12	30.339	21	0.268	497
46.333	8	40	97.117	84.182	0.208	4183
38.5	4	21	78.264	79	0.306	2798
95.5	17	74	221.633	260.35	0.19	21098
64.3	10	50	161.37	186.903	0.237	11969
42.5	6	35	92.579	84	0.23	3814
62.333	9	47	135.837	131.143	0.195	7288
56.5	12	41	120.047	124.08	0.244	6539
53.5	11	39	113.169	113.75	0.242	5789
30	5	20	51.597	40.571	0.258	1230
89.5	15	59	200.697	231.081	0.199	18015
42	5	21	85.819	93.684	0.331	3495
45.5	7	32	94.459	91.333	0.255	4198
48	10	41	96.806	83.273	0.205	3866
43.5	7	28	80.742	71.158	0.24	2974
45.833	8	32	94.696	92.857	0.263	4202
51	10	35	107.323	108.435	0.253	5323
56	6	34	132.916	140.37	0.228	8026
62.5	15	70	133.312	119.286	0.182	7136
47.5	5	24	106.48	129.727	0.341	5956
94	18	70	245.615	308.884	0.196	27490
45.5	7	33	99.673	95.091	0.239	4477
50.5	5	25	113.627	143.478	0.345	6459
86.5	13	64	214.291	248.923	0.191	20358
92.75	22	92	241.098	276.744	0.178	26604
40.5	7	26	82.388	78	0.269	3058
44	6	31	94.371	91.238	0.252	3950
72.5	19	86	170.602	154.353	0.155	11944
60.5	8	42	144.853	179.571	0.287	11162
50.5	10	50	108.841	95.417	0.199	5296
32	7	25	50.292	36.857	0.229	1105
63	12	49	154.841	178.467	0.243	11099
57	12	54	122.627	113.769	0.203	6726
72	11	54	174.52	201.576	0.224	14693
38	6	33	72.774	57.889	0.214	2322
89	21	94	226.844	259.902	0.187	22483
40.833	8	31	81.001	72.526	0.25	3003
65	8	45	159.072	204.133	0.282	13434
53.833	10	39	113.483	115.25	0.245	5882
56.5	11	41	120.351	125.6	0.247	6637
70	12	49	161.616	188	0.239	12571
66	11	41	148.245	170.207	0.248	10736

72	13	50	167.133	189.313	0.223	12948
69.5	12	46	153.57	170.667	0.228	10576
100	18	75	254.955	335.591	0.207	31520
69.5	12	46	153.806	172.267	0.232	10666
64.5	11	44	139.187	147.929	0.229	8618
60.5	11	44	133.749	144.741	0.243	8175
82	15	60	196.408	239.389	0.224	18196
84	15	59	203.106	247.189	0.216	19281
57.5	10	38	119.737	122.48	0.238	6456
83	15	59	197.072	243.667	0.228	18532
68	11	44	146.89	162	0.234	9734
60	11	41	125.405	127.231	0.227	6922
53.5	10	39	113.483	115.25	0.245	5882
61.5	10	41	140.663	155.714	0.243	9530
59	10	37	127.399	137.692	0.25	7526
53.5	10	39	113.483	115.25	0.245	5882
50.5	5	34	114.717	121.333	0.257	6314
58.333	7	38	135.112	153.185	0.259	8695
39.833	8	29	67.587	54.118	0.228	1920
39.333	5	25	81.754	75.579	0.255	2990
43.333	7	33	92.895	85.333	0.235	3855
38.333	5	22	70.967	66.588	0.29	2231
42.833	7	33	92.895	85.333	0.235	3855
49.333	10	40	97.761	86.545	0.216	4069
38.833	7	29	74.876	65.333	0.251	2601
37	3	18	73.796	78.118	0.345	2648
55	7	35	123.586	143.28	0.287	7626
57	8	39	130.007	150.615	0.276	8384
44	8	33	79.318	66.211	0.222	2748
54	10	43	110.698	102.833	0.215	5464
60.333	11	45	132.212	136.519	0.225	7405
31.333	5	18	53.04	45	0.292	1258
54.833	10	42	117.786	113.92	0.219	5974
30.167	4	17	52.432	43	0.275	1316
48.333	8	41	104.068	94.087	0.213	4854
31.333	3	19	52.331	43	0.272	1196
37	6	26	69.451	60.706	0.263	2182
41	5	34	86.132	75.9	0.23	3306
62.5	15	70	133.312	119.286	0.182	7136
42.333	7	36	85.339	72.6	0.218	3173
41.167	6	27	81.518	74.316	0.251	2934
67.167	15	53	151.795	160.133	0.214	10281
39	6	25	69	59.059	0.255	2238
42	7	28	75.173	66.333	0.253	2484
58	13	58	128.91	121.111	0.202	7374
121	25	109	337.889	532.667	0.225	64029
63	10	50	143.257	143.241	0.201	8597
39.5	4	24	82.218	77.474	0.262	3074
52.5	8	42	116.96	109.76	0.207	5554
130.5	27	121	350.979	434.034	0.15	52970

64.5	9	35	154.771	212	0.317	12520
64.833	11	49	156.939	190.2	0.262	12880
73	18	76	158.913	146	0.167	9830
50.5	8	35	100.471	98	0.245	4164
31	2	15	55.758	54.714	0.359	1404
45.5	8	38	91.216	78.571	0.214	3516
97	19	83	232.7	260.19	0.173	24304
45.5	6	20	92.626	104.6	0.332	4112
41	5	25	83.982	85.053	0.299	3376
39	5	24	76.703	72.222	0.278	2734
52	6	36	121.97	134.4	0.266	7215
46.833	7	31	99.931	96.091	0.241	4546
57	13	53	130.359	127.852	0.214	7450
69	9	36	141.329	159.429	0.244	9036
51	7	30	98.761	90.909	0.224	4338
39.5	7	30	74.225	63	0.24	2516
50	9	41	97.746	86.909	0.217	4060
22.5	3	10	26.215	18.667	0.302	357
67	10	49	154.172	174.533	0.236	11272
39.5	6	27	75.98	69.556	0.269	2620
18.667	3	6	17.25	12	0.33	156
92.5	15	70	241.075	364.585	0.268	31436
48.333	8	30	93.92	89.429	0.248	3875
80	18	78	196.138	205.622	0.177	15748
26.5	3	15	41.404	32.167	0.282	780
45.5	9	44	89.674	72.952	0.197	3443
42.5	6	26	76.158	70.222	0.27	2617
39	6	24	78.221	78.333	0.305	2788
71.5	13	56	159.389	174.194	0.218	11189
75	12	52	188.154	258.235	0.277	18350
72.833	10	49	180.537	242.303	0.275	16799
49	9	39	106.453	103.913	0.241	5392
65.333	9	39	140.832	156.571	0.24	8836
39.833	8	29	67.587	54.118	0.228	1920
45.5	11	49	88.228	67.81	0.182	3359
32	5	19	59.455	53.867	0.307	1591
78.5	11	56	208.703	287.784	0.259	23439
54.833	10	42	117.786	113.92	0.219	5974
45.5	8	34	94.49	91.81	0.26	4170
68	10	51	168.194	195.313	0.232	13894
41	6	20	64.042	56.125	0.277	1868
32.5	5	16	46.593	36.769	0.277	1036
60.5	11	47	128.273	118	0.189	6617
45.333	7	31	95.912	98.286	0.279	4258
41	6	28	81.983	76.211	0.261	3018
34.5	5	20	58.131	49.2	0.276	1573
58	9	36	121.299	130.48	0.257	6957
46.5	6	32	99.921	96.182	0.242	4544
27	4	15	41.235	31.667	0.275	762
59	12	51	122.609	114.154	0.203	6444

36	5	23	70.758	65.412	0.281	2189
55	8	35	114.768	122.25	0.264	6250
57.5	11	47	116.753	108.96	0.21	5717
45.667	8	38	91.131	78	0.211	3544
64.333	9	46	154.82	177.667	0.24	11526
51.5	10	41	104.762	96.957	0.222	4784
41.667	5	25	83.874	84.632	0.294	3127
38.5	5	29	74.186	63.111	0.236	2366
41.5	7	38	84.276	68.7	0.205	3169
38	6	27	75.583	67.667	0.257	2566
22	3	12	30.339	21	0.268	497
20.5	3	8	27.36	21.333	0.349	348
47.5	7	39	103.208	90.783	0.201	4242
59	12	45	125.177	125.385	0.224	6799
75	12	51	179.117	232.242	0.263	15935
59	14	62	127.582	115.037	0.189	6674
43.833	6	29	89.62	90.2	0.28	3516
41.5	4	21	86.126	95.368	0.338	3559
28.5	4	15	41.373	32.167	0.282	768
49.333	9	43	103.089	90	0.202	4628
85.833	14	62	234.355	309.805	0.218	27456
45.5	11	49	88.228	67.81	0.182	3359
65	13	55	142.372	138.345	0.196	8954
43.5	7	27	88.683	86.5	0.266	3344
48.5	8	31	102.206	106.182	0.272	4786
64	13	51	144.785	149.862	0.214	9336
38	7	29	67.881	55.294	0.233	1952
53.5	13	59	113.341	95.12	0.181	5647
49.5	11	55	100.592	80.957	0.182	4282
37	6	26	69.882	62.353	0.272	2212
35	6	21	64.434	57.125	0.279	1794
66.25	13	60	158.463	169.355	0.214	11661
57	10	45	115.243	102.4	0.19	5104
48	7	28	93.853	89.143	0.244	3850
59	10	45	122.742	114.692	0.199	5938
41	6	20	71.493	68.235	0.299	2442
27.5	4	16	41.725	33	0.293	796
52	9	41	104.746	97.043	0.222	4751
40.5	5	34	86.132	75.9	0.23	3306
100	19	80	256.617	348.682	0.219	31802
59	12	51	122.609	114.154	0.203	6444
52.5	9	39	114.103	118.25	0.253	6042
44.5	8	35	92.486	83.429	0.232	4002
62.5	13	47	138.809	144.714	0.223	8771
43.333	7	35	85.368	72.7	0.218	3178
50.5	12	57	100.052	78.87	0.176	4166
52	8	39	111.071	104.917	0.218	5322
87	18	84	225.828	251.854	0.177	22471
36	6	29	62.145	49.75	0.237	1546
28.5	3	14	48.594	43.077	0.333	1117

36.5	4	29	73.642	61	0.227	2458
38	6	31	67.443	53.882	0.226	1922
49	7	37	104.518	96.435	0.218	4722
41	6	32	78.915	64.947	0.215	2714
39	5	22	77.686	76.333	0.297	2885
68.5	11	55	167.734	193.5	0.229	13212
56.5	9	47	123.616	118.462	0.21	6790
56	9	38	116.742	108.88	0.205	5758
36.833	6	24	69.877	62.235	0.27	2227
42	5	21	85.819	93.684	0.331	3495
67	11	46	153.946	173.333	0.232	10358
62.5	10	47	140.947	156.786	0.247	9580
57	13	59	121.584	109.615	0.196	6169
48	11	48	103.405	91.478	0.211	4660
36.5	4	21	72.532	72.706	0.319	2449
37	4	21	72.532	72.706	0.319	2449
53.5	5	32	123.434	142.88	0.282	7328
40.5	5	25	83.661	83.368	0.29	3306
27	5	19	46.348	36	0.265	929
40.5	5	25	83.661	83.368	0.29	3306
36.333	6	26	68.856	58.588	0.25	2088
57.5	7	45	133.608	144.889	0.246	8531
51	9	42	111.728	107.167	0.226	5696
51	9	42	111.728	107.167	0.226	5696
47	5	22	99.58	116.857	0.335	4822
71	10	50	174.908	204.121	0.224	13985
36.5	6	18	58.167	49.2	0.277	1575
40.833	7	28	68.197	56.235	0.237	1885
65.5	11	51	156.294	186.2	0.257	12615
42.5	6	25	88.889	87.1	0.271	3801
81	16	77	204.382	221.368	0.182	18517
42	6	30	87.829	82.2	0.251	3408
23	3	12	31.104	22.8	0.293	474
48.5	9	35	100.569	98.273	0.25	4922
73	13	59	171.944	187.03	0.204	13098
40	6	21	77.219	74.556	0.288	2622
57	7	41	132.795	140.222	0.233	8030
38.5	6	22	70.086	62.941	0.273	2267
48	7	32	100.388	97.727	0.248	4666
62	9	47	145.394	153.862	0.219	9733
40.5	7	26	75.677	68.222	0.262	2576
54.5	10	40	119.387	121.36	0.237	6372
51.5	8	34	107.43	108.783	0.251	5115
43.5	7	27	88.611	85.9	0.266	3511
39	6	25	76.753	72.556	0.282	2738
62	8	40	144.581	177.714	0.28	10142
47.5	5	28	104.574	118.727	0.309	5365
45.333	8	39	90.986	77.524	0.209	3524
44.167	7	25	82.926	80.105	0.278	3158
49.5	9	39	98.544	89.818	0.226	4277

62.5	10	47	140.947	156.786	0.247	9580
35.5	6	19	64.818	58.5	0.287	1848
44	10	44	90.674	76.381	0.211	3630
73	18	76	159.121	146.875	0.168	9888
30	6	22	45.065	32.462	0.236	922
35	6	29	62.145	49.75	0.237	1546
34.833	5	23	70.092	62.706	0.272	2423
47	6	25	96.562	101.238	0.286	4401
55	6	32	126.947	163.2	0.329	8462
46.5	6	31	95.59	96.571	0.272	4245
40	5	23	77.533	76.111	0.296	2698
40.5	6	30	80.463	70.526	0.237	2766
55.833	8	39	130.532	154.077	0.286	8941
48.667	9	43	103.089	90	0.202	4628
47	7	33	94.117	90.095	0.25	3878
61.5	9	45	144.753	178.714	0.284	10608
22	2	10	32.404	26.2	0.34	472
69.5	10	49	168.152	195.125	0.23	12990
85	17	80	219.093	244.45	0.181	21360
71	10	55	177.986	224.121	0.251	14914
124	27	136	336.863	374.947	0.132	47069
55	11	55	120.16	103.692	0.18	6061
50	8	38	104.284	94.957	0.214	4683
49.5	8	38	105.87	101.652	0.234	4961
44.5	7	29	81.314	73.789	0.251	2877
59.333	10	44	142.902	167.429	0.266	10723
48.5	9	36	105.142	98.87	0.224	4824
43.833	8	30	87.686	82	0.253	3370

SMTIV	GMTI	GMTIV	Xu	SPI	W	WA
4136	1804	6861	16.935	11.752	530	3.897
9990	5638	14707	23.869	16.841	1568	5.681
13172	6736	25305	24.507	15.662	1592	4.898
1385	589	2237	11.226	6.057	166	3.018
3893	1806	7147	16.105	8.087	453	3.775
2351.062	773	3900.951	13.646	10.838	281	3.088
17696	10985	28581	28.272	16.278	2599	5.975
1075	468	1882	9.576	2.973	105	2.333
6481.827	4437	10276	20.797	7.938	926	4.009
4240	2580	5678	18.51	10.473	711	4.647
39062	20876	70869	35.133	31.028	5207	6.676
16912.871	11976	23204.275	29.149	18.142	2897	6.23
5211	3867	7045	20.303	8.212	882	4.2
12489.605	6913	19753.111	25.743	17.64	1836	4.857
11749.444	6572	20693.333	24.063	17.594	1551	5.17
9952.778	5833	16743	23.162	15.767	1365	4.946
2065	1172	3049	13.806	6.811	284	3.121
38808	18499	83663	33.089	24.716	4275	6.419
5945	3245	8983	19.776	12.362	890	5.205
6328	4342	9496	20.711	9.505	959	4.567
6234	3793	9385	20.625	13.634	916	3.965
5906.444	3033	11342	18.538	8.851	676	3.953
7220.383	4262	12138.444	20.742	11.457	975	4.643
9070.778	5390	15148.667	22.472	14.23	1247	4.929
10836	8196	14634	25.742	9.869	1895	5.399
12152	7214	20564	25.2	19.109	1670	4.418
8428	5974	11637	22.965	10.722	1427	6.177
48010	27894	84394	37.571	31.952	6641	7.354
6397	4517	8956	21.374	10.115	1046	4.528
9796	6097	13596	23.864	14.538	1650	6.522
34181.778	20862	57454.667	34.491	22.615	4854	6.551
38321.401	29092	59620.37	36.89	27.711	5950	6.589
4430	2927	5839	18.916	11.743	741	4.333
5368	3846	6800	20.664	11.08	958	4.562
15909	12998	22649	29.336	19.363	2624	4.677
16572	12056	26041	27.59	9.61	2514	6.651
7002	5776	9845	22.397	8.153	1145	4.149
2179	1012	3560	13.321	8.605	258	2.835
14991	11121	19988	28.398	20.595	2677	6.154
11435	7267	20443	24.124	12.398	1479	4.551
25644	15803	47361	30.514	13.478	3326	6.299
3894	2366	6367	17.188	6.689	521	3.405
35102	23083	55550	35.633	30.9	5328	6.498
5008.605	3021	8123.444	18.609	10.45	689	4.029
21418	14361	36025	29.221	10.48	3062	7.039
10269.383	5960	17594.778	23.244	14.703	1383	5.011
12129.778	6705	21812.667	24.141	16.577	1570	5.233
22279.889	13173	40537.444	29.258	17.672	2914	6.267
19114	11314	34694	27.79	15.548	2468	6.079

21415.222	13425	35538.556	29.685	19.601	3029	6.107
20597.222	10549	39099.889	28.099	20.438	2560	5.885
56384.222	33077	104647.778	38.668	29.273	7383	7.804
20277.222	10633	37571.556	28.172	20.411	2584	5.94
16011.222	8615	28825.444	26.481	17.848	2071	5.479
14660.778	8219	25817	25.84	17.586	1954	5.567
33843.556	18747	63869.333	32.919	24.248	4309	6.84
34735.889	19839	63315.222	33.554	24.673	4573	6.866
12023.556	6498	21811.556	23.985	15.445	1531	5.103
34534.333	19120	65287.667	33.012	24.537	4386	6.962
19575.111	9726	38452.333	27.38	18.564	2349	5.786
13644.778	6909	25792.667	24.663	17.266	1654	5.089
10301.111	5960	17708.333	23.244	14.703	1383	5.011
15681.222	10068	26429.111	26.838	13.265	2180	5.767
13483.889	7589	23572.111	25.16	15.89	1790	5.508
10019.111	5960	16695.333	23.244	14.703	1383	5.011
7913.778	6584	10000.667	23.609	5.752	1456	5.275
14133.16	8819	22800	26.223	12.413	2068	5.892
3794.049	1792	6623.481	16.301	11.061	460	3.382
4242.494	2908	5654.444	18.85	9.253	718	4.199
5117.938	3883	6656.704	20.353	9.931	896	4.267
3819.494	2027	5549.593	17.301	10.637	566	4.162
5122.938	3883	6690.148	20.353	9.931	896	4.267
7306.272	4057	12690.444	20.831	13.518	952	4.121
4140.049	2649	6391	17.702	8.67	588	3.843
4568.444	2480	7060	17.961	8.859	664	4.882
13563	7830	24181	24.856	11.596	1791	5.97
14766	8668	25742	25.587	13.267	1958	6.025
5536	2754	10558	18.201	10.062	629	3.678
9590	5728	16908	22.696	11.858	1234	4.471
10818.938	7131	14707.111	25.48	19.654	1843	5.251
2297.605	1112	3406	14.156	9.076	315	3.462
10425.827	5956	17602.556	23.651	15.279	1424	4.747
1946.877	1284	2626.642	14.07	5.593	301	3.308
6907.605	5148	10095.778	21.805	8.553	1082	4.277
1633.407	1052	1761.864	14.024	7.106	301	3.308
3800.444	2114	6168.667	16.927	9.309	516	3.794
4592	3360	6315	19.373	5.747	759	3.995
12152	7214	20564	25.2	19.109	1670	4.418
5052.938	3225	7848.778	19.143	8.995	726	3.821
4958.877	2840	7760.531	18.725	10.35	706	4.129
17164.543	10597	28812.642	27.722	21.014	2402	5.522
4205	2279	7568	16.777	6.888	502	3.691
5590	2379	11432	17.711	11.069	597	3.902
10617	7919	16013	24.946	14.204	1635	4.658
97038	70459	160693	46.284	35.796	14382	10.05
13906.444	8544	21814.667	26.718	17.087	2077	5.116
4512	3008	6254	18.985	7.689	736	4.304
8323	5338	11549	23.445	15.023	1372	4.573
89502	54885	155605	46.303	43.503	12587	7.615

20936.889	12430	34220.889	28.881	19.824	3074	7.571
19877.16	14150	33146	28.819	12.734	2853	6.559
18242	9891	33397	28.062	24.064	2336	4.71
8762	3792	15907	21.372	16.274	1078	4.667
2200	1181	2773	14.9	8.846	383	4.209
5691	3473	8765	19.89	11.066	825	3.929
42714	26410	80593	36.056	23.465	5464	6.346
7387.333	3837	12002.667	20.755	13.949	1046	5.505
6176	3316	10700	19.402	9.336	808	4.725
4866	2679	8136	18.154	8.702	650	4.248
10436	7469	15358	24.614	8.968	1680	5.6
7383.16	4625	11821.519	21.433	10.184	1057	4.576
10303	7645	14390	25.162	17.295	1726	4.917
19850	8829	40403	26.874	18.392	2232	5.905
8774	4444	17437	21.199	9.494	1000	4.329
4796	2565	8871	17.571	8.31	567	3.706
7806	4030	14295	20.865	12.641	956	4.139
1026	291	1966	8.654	4.806	84	2.333
19087.778	11747	32483	28.314	14.746	2618	6.018
4894	2539	8452	17.984	9.888	626	4.092
565.753	90	953.333	6.367	4.711	42	2
53558.444	32407	90560.333	37.814	28.072	7474	9.115
7444.605	3757	13355.111	20.591	13.326	939	4.471
23230	15759	33849	32.352	27.618	3804	5.712
1332	670	1767	11.947	6.285	193	2.924
5282	3573	8114	19.575	9.531	766	3.648
5393	2500	10145	17.957	10.192	632	4.131
4588	2572	6686	18.43	12.432	705	4.608
18061	11215	28725	28.69	21.697	2700	5.806
28736.444	18789	45156	32.473	22.731	4390	7.825
26622.16	17281	42613.222	31.647	19.04	3998	7.572
7711	5785	11360	22.282	10.066	1195	4.723
17744.716	8589	33367.889	26.744	18.501	2192	5.799
3659.049	1792	6177.593	16.301	11.061	460	3.382
5165	3631	8232	19.22	9.179	712	3.39
2613	1417	3515	15.353	9.958	404	3.848
35874	25355	58418	34.613	14.663	5324	7.994
10417.827	5956	17583	23.651	15.279	1424	4.747
7344	4242	12794	20.694	11.245	964	4.59
21267	15031	34324	29.895	10.725	3125	6.3
4736	1753	10461	16.016	9.4	449	3.742
2408	966	4680	12.898	6.781	239	3.064
11675	6528	19701	24.7	16.962	1593	4.538
7600.827	4154	12777.667	21.011	12.695	1032	4.914
5474	2936	9300	18.869	10.249	724	4.234
2913.778	1507	4795.333	15.034	7.607	369	3.514
13752.778	7104	26615.333	24.38	14.188	1631	5.437
8386	4621	15348	21.436	8.631	1058	4.58
1420	645	2015	11.841	7.12	190	2.879
11456	6643	20121	24.114	15.497	1484	4.566

3247	1987	4121	17.164	10.702	556	4.088
11364	6367	20386	23.591	12.495	1467	5.315
10743	5687	19161	23.394	16.148	1362	4.54
5899.321	3578	9477.901	19.912	10.755	819	3.9
18287.605	12087	29658.778	28.395	12.825	2665	6.126
8244.556	4846	13823.333	21.946	13.755	1115	4.407
5023.988	2853	6890.407	19.281	12.147	804	4.702
3620	2263	5047	17.567	8.546	568	3.712
4517	3389	6660	18.909	5.297	687	3.616
3672	2507	4885	17.857	9.616	609	3.98
905.778	468	1340.333	9.576	2.973	105	2.333
718	248	910	9.029	6.386	96	2.667
6179	4052	8367	21.58	12.782	1044	4.126
12149.444	6755	20947	24.516	18.015	1630	5.015
30858	16151	59806	31.328	22.375	3832	7.258
10810	6781	17511	24.554	17.799	1553	4.425
5474.938	3226	7380	20.07	13.447	902	4.747
5520	3313	7660	19.878	11.157	906	5.298
1782	645	3072	11.891	7.237	193	2.924
7550.272	4872	12511.889	21.555	9.991	1035	4.091
40530.383	29152	63047.667	36.813	22.382	6351	7.745
5145	3631	8224	19.22	9.179	712	3.39
14866	9587	25441	26.56	15.062	2006	4.941
5364	3026	7372	19.883	14.472	865	4.553
7480	4641	10942	21.833	14.486	1168	5.056
15569.778	9639	26189.667	26.905	18.181	2173	5.352
3756	1828	6300	16.415	10.42	470	3.456
7953	6305	12138	22.811	10.154	1189	3.963
6551	4563	10387	20.996	9.784	931	3.68
4014	2112	6666	16.969	9.712	530	3.897
2884	1594	3784	16.05	11.146	457	3.808
17615.623	12518	28075.556	28.625	16.133	2625	5.645
10215	4798	18535	22.99	17.133	1280	4.267
7595.111	3722	13845.333	20.544	12.463	936	4.457
11626	5611	20794	24.059	17.876	1491	4.588
5691	2380	12164	17.356	9.856	580	4.265
1644	682	2707	11.992	7.249	198	3
8673	4773	15173	21.916	12.958	1116	4.411
4598	3360	6352	19.373	5.747	759	3.995
56376	32385	100446	38.857	33.714	7671	8.109
11456	6643	20121	24.114	15.497	1484	4.566
9920.778	6139	16075	23.354	13.812	1419	5.141
6249	4293	10136	20.327	7.388	876	4.171
15167.778	9119	26586	26.286	17.626	2026	5.36
5413.716	3231	8956.111	19.147	9.029	727	3.826
6536	4423	10524	20.825	11.201	907	3.585
8856	5335	14251	22.807	12.05	1259	4.562
28539	23853	38135	35.398	24.235	5163	6.296
2858	1334	4067	15.496	10.693	398	3.317
2017	991	2975	13.518	7.14	280	3.59

2974	2538	3611	17.496	0	549	3.588
3724	1822	6378	16.388	9.014	458	3.368
7454	4752	11372	21.936	9.945	1109	4.383
4796	2752	8196	18.2	6.926	617	3.608
4453	2820	6406	18.383	8.999	687	4.49
19054.222	13681	28075.222	29.824	17.071	3096	6.242
10930	7159	17884	24.388	10.947	1540	4.738
10176.111	5786	17469.667	23.443	13.554	1361	4.537
3704.827	2149	5680.222	17.005	9.443	529	3.89
5889	3245	8791	19.776	12.362	890	5.205
17722	9961	28641	28.202	21.805	2600	5.977
16637	10102	29819	26.801	13.814	2195	5.807
10339	6302	17373	23.87	16.283	1425	4.385
6180	4833	8411	21.594	12.889	1052	4.158
3813	2263	5147	17.65	9.977	618	4.544
3941	2263	5449	17.65	9.977	618	4.544
11293	7254	16777	24.908	11.252	1786	5.953
4993	3234	7087	19.279	9.373	792	4.632
1320	791	1478	12.763	8.42	234	3
4993	3234	7087	19.279	9.373	792	4.632
3326.716	1998	4811.556	16.724	9.482	498	3.662
12865.444	8950	20088.667	25.95	7.405	1956	5.573
8474	5995	12897	22.876	10.613	1286	4.659
8474	5995	12897	22.876	10.613	1286	4.659
8414.111	4532	13155.333	21.792	13.45	1227	5.843
21743	14137	33237	30.561	18.96	3368	6.379
3624.778	1497	7077.667	14.999	8.702	369	3.514
4071.728	1671	7410.173	16.545	11.643	478	3.515
20112	13857	34332	28.71	12.59	2793	6.421
5901	3902	9019	20.006	8.014	871	4.584
24515	19831	34489	33.306	19.919	4206	5.983
4706	3317	6047	19.684	10.606	822	4.326
1100	396	1862	9.788	5.248	114	2.533
7732	5293	12715	21.555	9.792	1081	4.68
20649.111	13475	32682	30.041	20.076	3086	5.845
4356.444	2376	6249	18.215	12.456	671	4.386
11379.778	8208	16128.333	25.811	11.419	1893	5.393
4192.778	2209	7146.333	17.076	9.377	535	3.934
7905.444	4801	13375.333	21.537	9.693	1075	4.654
15608.778	10267	25840.333	27.232	11.413	2231	5.495
4619.444	2496	7619.333	17.885	10.871	614	4.013
11272.111	6380	19354.667	23.96	15.661	1517	5.057
8448.111	4971	12950.333	22.442	14.574	1251	4.945
5508.778	3350	7966	19.838	12.361	859	4.521
4836.111	2668	7972.667	18.165	10.006	653	4.268
16807.111	10037	27015.667	27.41	17.25	2488	6.582
8439	5260	12516	22.436	10.672	1306	5.654
5873.383	3558	9474.667	19.9	10.633	814	3.876
6134.753	3047	11075.111	19.049	11.76	761	4.45
7573.889	4357	13138.667	21.125	11.937	988	4.277

15812	10102	26892	26.801	13.814	2195	5.807
3174	1658	4543	16.176	11.174	468	3.9
4928	3807	6883	19.813	10.674	802	3.819
18388	9947	33757	28.084	24.129	2350	4.738
1900	849	3237	12.404	7.224	211	2.705
2470.444	1334	3112.333	15.496	10.693	398	3.317
3126.272	2546	4107.37	17.099	4.723	533	3.919
6733.444	4313	9624.667	21.159	11.511	1063	5.062
14437	8522	24259	25.543	13.412	2040	6.8
6642.778	4219	9805	21.004	10.858	1014	4.829
4475.778	2476	6381.333	18.395	11.168	685	4.477
4005	2640	5301	18.489	10.229	670	3.918
13506.605	9662	21437.889	25.769	9.478	2003	6.163
7035.654	4872	10864.901	21.555	9.991	1035	4.091
7042	3742	11896	20.556	12.628	946	4.505
17350	10924	28590	27.459	15.888	2502	6.619
820	353	876	10.223	6.444	131	2.911
21825	13107	36228	29.846	18.428	3122	6.294
27750	22754	38024	34.791	22.402	4889	6.268
23762	14682	36716	31.106	22.112	3698	7.004
72160	50917	118762	44.676	34.542	10686	6.695
8669	6425	12759	23.596	10.908	1348	4.148
8204	4745	14068	21.841	11.38	1092	4.316
8803	4976	15154	22.083	12.112	1169	4.621
5850	2735	10834	18.653	11.652	701	4.099
15693.049	11898	25046.37	27.186	9.942	2344	6.201
7452	4828	11168	21.987	13.354	1137	4.494
5958.16	3234	9687.333	19.668	13.027	820	4.316

Har	Har2	QW	TI1	TI2	STN	HyDp
28.196	50.328	473.6	0	2.637	1.609	1573
40.414	79.508	1404	0	4.656	1.792	6784
49.757	99.378	1232.61	79.342	2.987	5.347	5784
16.586	25.652	134.667	0	2.349	1.792	408
27.458	46.951	362.345	19.08	2.927	3.367	1352
22.52	38.717	281	-18.088	2.081	0	665
56.773	117.932	2123	119.01	4.507	5.193	11658
16.521	24.25	68.448	5.667	0.973	3.367	198
43.547	81.47	623.401	69.219	2.008	7.085	2770
28.792	51.821	659	0	4.451	1.792	2544
79.329	182.844	4340.166	183.93	3.875	5.375	24607
58.408	122.498	2405.724	130.642	4.839	5.159	13598
39.094	72.709	671.833	53.086	2.827	5.481	2780
52.858	110.945	1576.667	48.057	2.248	3.584	6378
47.263	92.03	1259.333	84.183	4.308	4.736	6132
45.222	87.061	1115.763	77.573	4.097	4.736	5151
24.139	39.71	217.167	13.014	2.067	3.401	692
73.786	163.694	3572.533	241.92	4.746	6.985	19663
30.398	55.104	785.333	0	4.849	1.792	3650
38.497	70.836	784.692	62.004	3.919	5.05	3348
42.816	81.215	658.893	49.797	1.669	5.13	2702
35.086	62.771	540.497	46.986	3.12	4.977	2026
38.701	71.005	708	55.944	3.2	5.159	3540
42.774	80.995	1013.193	73.38	4.128	4.736	4728
49.121	99.33	1694	105.144	3.767	5.193	7339
60.222	122.143	1112.496	98.353	2.028	6.893	5555
36.017	66.932	1258.667	48.598	5.781	2.89	6930
85.37	198.803	5639.6	330.361	5.012	6.438	34554
40.732	76.632	832.385	62.909	3.129	5.159	3577
36.885	70.255	1478.667	0	6.184	1.792	8441
77.564	176.155	3996.333	257.241	4.008	7.167	22463
95.753	218.727	4352.022	493.76	4.822	11.784	28699
33.424	60.268	616.621	27.472	3.625	3.367	2489
36.976	69.406	831.667	33.605	3.837	3.584	3290
79.173	168.688	1735.148	244.937	1.867	11.011	8888
52.384	102.736	1974.167	174.531	6.27	6.985	13028
50.011	95.239	746.198	99.911	2.045	8.361	3594
25.063	41.35	184.207	11.039	1.097	3.367	565
57.043	117.414	2259.77	126.677	5.168	5.159	12495
54.328	105.864	913.946	113.6	2.3	8.649	5089
65.745	138.054	2552.08	253.614	4.859	8.56	15608
34.051	60.412	349.333	31.969	1.264	5.481	1332
89.832	203.945	3604.054	291.414	3.614	8.844	26277
35.433	63.146	478.357	41.584	2.482	5.124	2177
55.809	112.137	2675.214	221.469	7.617	6.916	16535
44.734	86.242	1115.37	77.545	4.11	4.927	5281
46.774	91.205	1258.986	84.16	4.32	4.927	6275
59.564	123.278	2344.29	188.093	4.768	6.537	13792
55.012	111.129	1980.209	169.316	4.516	6.354	11400

62.512	131.434	2450.809	190.761	4.562	6.354	13795
56.47	117.538	2177.017	122.038	4.442	4.745	11088
88.414	204.819	6064.284	458.321	5.891	8.328	41921
56.557	117.656	2141.017	120.02	4.432	4.745	11470
53.067	108.169	1694.348	101.536	3.763	4.745	8527
50.629	101.404	1567.217	97.275	4.389	4.927	8272
70.857	154.393	3401.196	236.465	5.069	6.719	22104
72.449	159.799	3803.446	257.556	5.174	6.537	23038
46.524	91.171	1278.539	85.467	4.082	4.745	5849
70.167	152.665	3544.558	246.433	5.351	6.537	22802
54.422	112.057	1948.783	112.888	4.277	4.745	10173
49.664	98.351	1268.433	81.648	3.084	5.193	6270
44.734	86.242	1115.37	77.545	4.11	4.927	5281
53.291	106.804	1713.399	151.477	4.183	6.537	9547
48.004	94.926	1460.478	94.01	4.053	4.745	7505
44.734	86.242	1115.37	77.545	4.11	4.927	5281
43.322	83.401	1264.5	87.913	4.815	5.347	5770
49.294	98.417	1663	103.22	4.378	5.375	9281
31.18	54.712	338.343	16.798	1.825	3.555	1189
32.891	59.845	598.667	26.672	2.417	3.584	2256
39.029	72.374	679.539	53.695	2.843	5.318	2875
27.864	49.406	506.667	0	3.634	1.792	1840
39.029	72.374	679.539	53.695	2.843	5.318	2875
42.775	80.568	716.713	54.167	2.71	5.159	2958
33.344	58.405	409.207	37.449	2.482	5.159	1774
26.364	46.157	632.8	0	5.303	1.609	2534
45.027	86.671	1405	93.921	5.004	5.193	8323
47.525	92.586	1676.5	107.915	6.189	5.347	9047
36.045	64.81	434.521	37.773	1.653	5.13	1754
47.023	90.587	877.357	89.758	2.378	6.446	4135
50.138	102.292	1604.467	50.683	4.243	3.401	7178
22.586	37.698	269.667	0	2.852	1.792	862
47.782	94.143	1104.851	73.857	2.867	5.159	5036
23.602	38.719	240.5	14.412	2.617	3.401	778
45.194	85.691	721.283	76.81	2.218	7.272	3453
22.469	37.836	266.167	0	2.101	1.792	765
29.944	52.29	397.333	19.726	2.731	3.584	1535
37.124	68.048	561.667	46.498	2.391	5.481	2282
60.222	122.143	1112.496	98.353	2.028	6.893	5555
37.877	69.49	511.337	42.331	2.053	5.505	2077
33.308	60.582	594.333	26.479	3.269	3.584	2195
59.937	123.443	1961.984	162.424	4.57	6.346	9877
31.135	53.508	364.207	35.186	2.524	4.977	1454
31.906	56.915	456.167	21.423	2.607	3.584	1793
57.311	112.938	1045.34	125.436	2.447	8.502	5848
116.305	276.048	9881.77	1046.204	7.56	13.877	110467
56.164	117.173	1618.325	93.745	2.563	5.505	7726
32.514	59.108	634.667	28.276	2.993	3.584	2371
46.528	93.592	1232.667	41.993	3.156	3.584	4528
130.343	337.788	10321.29	741.328	5.2	10.479	67580

50.492	101.324	2639.333	77.719	7.55	3.584	18090
58.737	117.315	2156.172	234.409	5.813	8.526	14454
69.319	148.401	1582.103	123.145	2.026	7.081	8006
38.185	73.944	969.333	0	3.8	1.792	3753
19.837	33.721	383	-24.653	4.425	0	1260
40.372	75.222	609.583	48.167	2.123	5.124	2436
91.853	209.851	3856.073	447.242	3.66	12.052	24949
32.312	59.175	975.8	0	5.796	1.609	4504
32.412	58.201	643.667	28.677	3.792	3.584	3013
30.866	54.798	531.667	24.968	3.473	3.584	2135
45.373	88.201	1339	89.509	4.433	5.375	7214
40.457	76.032	816.767	61.729	2.605	5.193	3619
55.16	109.501	1194.167	109.284	2.974	6.893	6527
50.172	102.573	1969.667	60.035	4.041	3.584	9610
41.002	77.459	837.833	63.321	2.33	5.011	3189
33.586	59.034	399.793	36.587	2.32	5.159	1630
42.357	80.117	687.915	51.991	2.278	5.293	3000
13.401	19.533	62.667	0	1.06	1.792	160
57.974	118.595	2042.142	169.059	4.785	7.11	11825
31.477	55.994	481.667	22.62	2.837	3.584	2003
8.917	12.5	42	-5.624	1.435	0	69
79.278	174.824	6701.434	411.05	10.206	6.893	52053
37.66	70.519	781.667	31.585	3.248	3.401	3181
78.537	175.631	2847.517	192.824	2.979	6.893	15874
18.855	30.183	164.5	0	1.977	1.792	442
42.706	78.646	473.159	54.879	1.197	7.002	2100
31.316	55.64	523.207	24.571	3.415	3.367	2022
29.498	52.765	647.667	0	4.77	1.792	2523
60.188	126.545	2183.333	118.565	4.492	5.375	11617
62.919	132.614	3729.567	185.162	7.422	5.193	26361
60.645	127.068	3413	174.432	7.201	5.375	23152
44.484	83.265	900.009	95.843	3.912	6.833	4319
50.408	103.41	1954.167	59.563	4.214	3.584	9292
31.18	54.712	338.343	16.798	1.825	3.555	1189
45.181	82.187	398.413	60.336	0.892	8.442	1815
24.051	40.952	336	0	2.928	1.792	1240
71.198	152.515	4189.667	373.569	6.914	8.959	32339
47.782	94.143	1104.851	73.857	2.867	5.159	5036
38.916	71.456	705.31	55.732	3.279	5.159	3461
63.336	131.121	2406.933	246.241	4.873	8.412	14751
27.581	47.142	365.333	19.238	2.697	3.178	1328
22.067	35.252	186.833	12.026	2.219	3.178	586
53.073	108.588	1315.184	81.631	2.395	5.159	5176
36.824	68.417	831.667	33.605	3.978	3.584	3953
33.277	60.35	596	26.553	3.343	3.584	2349
25.681	43.035	280.867	15.745	2.438	3.401	1026
46.215	89.639	1353.057	90.449	4.717	5.159	6763
40.408	76.058	814.667	61.57	2.668	5.193	3655
18.902	30.3	169.8	0	2.005	1.609	425
52.727	104.297	989.774	93.879	2.27	7.07	5100

27.765	49.396	508	0	3.736	1.792	1742
44.083	84.477	1125.067	78.219	3.901	5.193	6042
48.828	96.459	985.019	65.846	2.483	5.347	4635
40.136	74.961	588.13	46.472	2.122	5.505	2365
57.383	117.472	2107.667	174.484	4.802	6.802	12270
44.326	84.687	872.286	63.175	3.274	5.165	3723
31.137	57.103	690.333	0	3.624	1.792	2931
32.052	57.568	456.5	21.438	1.997	3.584	1590
39.674	71.918	415.265	50.411	1.156	7.247	1860
31.515	56.205	508.167	23.865	3.403	3.584	1851
16.521	24.25	68.448	5.667	0.973	3.367	198
11.924	17.967	96	-9.821	2.428	0	206
42.828	84.199	917.333	33.911	2.076	3.584	3102
49.863	99.101	1336.57	86.034	3.974	4.736	6116
61.437	129.379	3115.343	159.22	6.278	5.347	21228
57.008	114.14	1030.495	94.306	2.084	6.893	5185
33.379	62.269	818.667	0	4.383	1.792	3275
30.116	54.578	801.333	0	4.907	1.792	3788
18.855	30.183	169.2	0	1.909	1.609	442
45.912	87.308	709.296	75.534	2.13	7.085	3138
79.998	179.934	5463.238	441.743	5.787	8.237	35269
45.181	82.187	398.413	60.336	0.892	8.442	1815
59.733	121.88	1418.724	159.226	2.703	8.021	7331
33.71	63.141	755	0	3.233	1.792	2994
38.924	73.284	998.971	38.571	4.71	3.555	4542
57.505	117.35	1723.862	147.397	4.13	6.5	8612
30.573	53.845	343.886	17.073	1.519	3.555	1238
54.997	105.423	725.78	114.936	1.623	9.541	3548
49.695	93.401	562.527	78.344	1.337	8.442	2582
29.673	51.779	389.714	19.348	2.48	3.555	1635
26.389	45.962	399.833	0	3.14	1.792	1348
63.8	131.39	1942.768	204.792	4.37	8.634	11198
47.661	96.406	1084	36.928	2.243	3.584	3902
37.251	69.909	779.667	31.504	2.881	3.584	3094
49.119	100.287	1256.833	41.2	2.48	3.584	4924
28.873	49.917	489.6	24.307	3.929	3.219	1947
18.814	30.043	160.667	0	2.011	1.792	473
43.96	84.247	796.552	57.69	2.41	5.347	3722
37.124	68.048	561.667	46.498	2.391	5.481	2282
88.483	204.922	5936.638	340.112	6.162	7.139	46787
52.727	104.297	989.774	93.879	2.27	7.07	5100
44.672	85.722	1112.925	77.375	4.21	5.159	5599
40.882	74.614	614.048	71.22	2.748	6.733	2814
55.131	110.762	1636.844	144.709	4.383	6.346	8143
37.795	69.349	512.85	42.456	2.015	5.505	2076
50.19	94.397	532.482	74.16	1.086	8.442	2432
45.394	88.516	992.1	68.975	2.235	5.193	4241
89.433	203.267	3520.332	351.85	3.199	10.476	23725
27.486	48.427	342	0	1.968	1.792	1005
19.958	32.469	230.333	0	2.731	1.792	815

33.345	59.015	406.5	37.201	2.043	5.375	1485
30.832	54.404	352.667	17.509	1.861	3.584	1172
43.305	83.371	881.333	63.83	2.029	5.193	3588
35.956	64.899	469.867	40.846	1.935	5.193	1665
30.504	53.824	601.069	28.228	4.371	3.367	2409
62.342	130.488	2438.485	189.802	4.829	7.11	14410
51.419	101.736	1148.396	108.925	3.323	7.272	5421
47.826	94.818	1103.333	73.755	2.438	5.193	4469
29.587	51.654	414	20.554	2.705	3.401	1614
30.398	55.104	785.333	0	4.849	1.792	3650
55.165	116	2330.4	66.372	5.022	3.555	11488
54.112	107.941	1558.419	137.776	4.037	6.957	9815
54.439	107.41	939.276	89.09	2.111	6.893	4757
46.953	88.558	666.331	70.958	2.009	6.893	3379
27.009	47.591	558.667	0	4.447	1.792	2197
27.009	47.591	558.667	0	4.447	1.792	2197
42.92	84.211	1631.667	55.585	5.532	3.584	8059
32.45	58.376	680	30.296	4.378	3.555	2849
21.042	34.6	195.333	0	1.856	1.792	545
32.45	58.376	680	30.296	4.378	3.555	2849
29.955	52.596	377	18.717	2.248	3.584	1403
51.715	102.477	1431.083	130.966	3.973	7.272	8420
46.91	89.69	948.305	97.016	3.337	6.851	4539
46.91	89.69	948.305	97.016	3.337	6.851	4539
33.6	62.512	1151.6	0	6.162	1.609	5567
62.152	133.584	2924	149.44	5.369	5.375	15705
25.92	43.275	281.379	15.773	2.562	3.367	1029
29.66	52.99	433.667	0	2.607	1.792	1274
59.076	118.322	2031.308	220.834	5.362	8.714	13856
35.936	64.867	692.207	57.305	3.522	4.977	3086
82.278	182.152	2887.981	310.082	3.146	10.294	18479
35.239	65.156	704.167	29.842	3.638	3.584	2681
15.163	22.9	91.333	0	1.563	1.792	231
41.66	76.862	867.167	96.286	3.985	6.068	3869
65.97	140.909	2378.563	179.765	3.849	6.951	13191
29.652	53.429	600.167	0	3.755	1.792	2272
50.551	101.883	1545	95.896	3.727	5.193	7661
29.553	51.452	437.867	21.739	3.229	3.401	1646
40.671	76.169	879.967	66.505	3.92	5.193	3815
56.275	115.027	1811	154.848	4.03	6.985	9046
31.804	56.491	507.8	23.847	3.309	3.401	1911
47.13	92.194	1172.264	78.363	3.48	5.159	5806
41.194	79.242	1095.267	40.489	4.311	3.401	4644
35.203	64.595	715.207	30.309	3.467	3.367	2986
31.165	55.201	522	24.514	3.298	3.401	2194
49.572	100.07	2228.167	67.914	6.738	3.584	12466
37.339	69.81	1162	44.865	5.642	3.367	5761
40.272	75.243	577.683	45.647	1.975	5.505	2342
33.22	59.723	652.138	29.055	4.111	3.367	2634
42.114	79.263	778.592	58.844	3.269	5.159	3209

54.112	107.941	1558.419	137.776	4.037	6.957	9815
26.193	45.527	415.5	0	3.501	1.792	1416
42.726	78.11	489.413	56.764	1.746	6.893	2370
69.35	148.208	1588.585	123.649	2.077	7.081	8109
22.894	36.833	148.69	9.571	0.979	3.367	444
27.486	48.427	342	0	1.968	1.792	1005
30.502	52.187	392.069	37.878	3.109	5.159	1638
36.216	67.096	957	38.669	4.984	3.401	4138
42.323	81.348	1825.667	62.194	7.386	3.584	10791
36.833	68.556	866	34.992	4.468	3.738	3768
29.511	53.137	621	0	4.091	1.792	2397
34.034	62.11	548	24.415	2.654	3.584	1982
48.516	93.065	1540.787	146.143	5.606	6.951	9626
45.912	87.308	709.296	75.534	2.13	7.085	3138
37.415	70.181	754.667	30.494	3.254	3.584	3232
51.24	101.847	2032.5	121.801	6.307	5.375	12809
13.441	20.95	131	-11.988	2.02	0	300
60.354	128.173	2614.333	137.665	4.829	5.193	14532
86.473	194.814	3318.8	339.53	3.157	10.476	22418
60.493	129.585	3307.333	85.759	6.842	3.584	19459
138.009	352.732	7310.985	834.015	3.495	15.534	50023
55.204	108.775	882.712	109.717	1.67	8.553	4096
43.678	84.085	850.495	61.597	2.541	5.347	3477
43.188	82.485	913.839	66.184	3.341	5.159	4082
33.713	61.236	554.467	24.703	2.692	3.401	2197
54.572	106.523	1711.126	198.463	5.238	8.526	11279
43.298	82.869	887.494	64.276	2.595	5.159	3784
35.717	65.857	674.467	28.583	3.226	3.401	2721

RHyDp	w	ww	Rww	D/D	Wap	WhetZ
33.271	738	2781	22.059	101.493	1216	436
48.263	1964	9730	34.996	225.542	3330	1280.321
60.127	3044	18536	23.389	185.99	10254	1253.964
18.914	262	864	9.592	37.981	409	121.821
32.067	936	4665	11.916	63.227	2325	338.839
26.619	281	665	26.619	91	281	169.393
68.814	3583	20211	36.43	320.18	11757	2243.133
18.7	313	1287	2.632	16.157	692	75.977
52.098	2845	20715	7.363	80.358	14881	684.922
33.77	999	4776	22.836	117.047	1557	589.571
98.178	8013	54196	50.704	523.056	28786	4509.768
70.82	4878	34510	34.1	291.899	18978	2276.9
46.552	2581	18982	11.309	83.231	9194	716.905
64.65	3022	16077	34.811	247.994	6922	1499.886
56.706	2523	14427	26.678	190.439	9786	1270.214
54.155	2286	12885	24.977	171.311	8567	1123
28.055	555	2304	9.613	49.966	1320	215.242
90.54	7116	50946	41.488	415.747	30059	3451.452
35.634	1082	4898	25.247	143.39	1811	727.75
45.582	1649	8862	18.928	128.984	5716	823.857
51.507	2553	16477	13.954	91.745	9713	732.879
41.424	1490	8733	13.303	84.52	5496	375.071
45.829	1857	10532	15.463	115.538	7688	698.13
50.98	2117	12045	22.43	153.936	8128	1022.928
58.953	2667	13675	31.589	259.464	5506	1607.357
73.894	5473	44906	16.5	128.507	31795	1559
42.161	1696	8946	28.993	194.403	3633	1186.821
105.298	9771	70380	56.742	617.566	53858	5277.452
48.512	2160	13245	17.553	122.117	7342	788.286
43.44	1890	10169	32.897	221.14	3283	1344.071
95.5	7806	54371	44.511	479.059	30491	3234.984
118.948	15351	163046	29.619	367.267	377147	5075.472
39.462	1340	6979	19.106	104.689	3399	592.929
43.968	1672	9397	20.817	130.153	3900	823.595
98.054	9495	91594	22.849	176.043	217538	2408.524
62.397	3764	25705	22.775	248.323	18794	2235.012
60.177	3795	30223	8.894	87.458	28419	1024.167
29.314	664	2918	7.774	38.634	1538	190.988
68.984	4753	35237	31.833	257.841	19531	2441.75
65.608	5800	56391	7.716	89.84	54541	1151.488
79.731	6510	53618	24.923	275.798	58991	2475.456
40.364	1830	12488	5.92	48.814	6379	388.137
111.873	15890	173266	29.767	302.474	166629	4948.5
41.86	1805	11304	10.985	73.421	6983	488.984
66.674	5807	52817	25.167	246.659	31433	2409.143
53.514	2443	14607	23.021	164.42	8925	1111.368
56.063	2690	16303	24.711	183.123	10176	1274.464
71.943	4837	33632	31.028	285.015	25062	2156.439
66.052	4075	27203	28.233	248.489	20823	1734.505

75.906	4863	31669	35.711	316.357	23201	2409.059
68.392	3930	23861	37.263	296.006	13162	1983.446
108.837	11123	86813	51.215	633.121	63753	5900.423
68.521	4020	24758	36.666	289.092	14795	2014.017
64.187	3355	19739	32.338	242.466	12006	1615.339
60.957	3238	19996	28.301	219.462	12543	1569.179
86.552	6719	47667	41.444	411.783	36880	3278.407
88.559	6924	48249	46.409	454.379	32294	3522.203
55.782	2562	14923	27.382	190.3	8866	1203.726
85.536	6872	50052	40.702	407.798	37704	3178.696
65.809	3697	22399	34.882	267.665	13090	1833.035
59.823	2672	14833	27.03	209.908	10138	1125.455
53.514	2443	14607	23.021	164.42	8925	1126.25
63.981	3780	25009	24.988	222.176	19230	1686.333
57.54	2946	17745	27.859	203.892	10977	1399.625
53.514	2443	14607	23.021	164.42	8925	1157.75
51.543	2249	12718	22.533	185.449	5229	1286.173
59.047	2932	16253	27.378	249.52	8131	1719.355
36.936	1088	5345	12.42	64.87	2608	224.093
38.839	1102	4812	18.934	118.617	2235	579.115
46.413	2448	17185	12.52	88.11	8941	675.345
32.759	734	2872	22.462	110.754	1121	352.04
46.413	2448	17185	12.52	88.11	8941	660.63
51.33	2020	11577	17.38	118.62	7503	659.598
39.244	1347	7527	9.091	69.872	5185	443.024
30.627	813	3682	22.823	114.95	1226	476.298
53.476	2666	16221	23.076	200.505	10915	1372.619
56.658	3398	24661	24.055	198.095	12182	1624.857
42.802	1842	11526	9.788	64.975	6633	472.653
56.453	2699	16263	15.715	132.006	11660	972.976
60.783	2620	13495	35.727	254.975	6105	1623.784
26.282	447	1576	16.496	69.632	699	230.963
57.609	2823	17053	21.566	160.319	10064	1060.209
27.314	575	2507	9.629	51.051	1306	214.796
54.115	3284	25261	7.629	89.981	17362	808.765
26.205	433	1467	16.259	69.394	632	189.734
35.221	852	3647	14.764	87.636	1950	292.429
44.102	2328	16802	9.21	70.808	8253	620.714
73.894	5473	44906	16.5	128.507	31795	1559
45.137	1901	11608	8.869	77.249	6474	546.878
39.406	1324	7166	16.896	100.007	3176	525.527
72.817	4467	31591	29.66	244.09	27920	1904.542
36.463	1154	6297	9.239	62.333	4416	384.786
37.698	1059	4908	15.045	91.704	2645	437.542
69.493	5324	46345	10.685	113.919	44477	1564.75
143.843	26822	319972	39.774	762.004	1067756	12607.375
68.483	4517	31276	24.779	201.883	15257	1614.536
38.318	1120	5011	19.176	119.857	2148	591
56.418	1900	8272	35.304	231.955	3286	1178.238
165.758	28215	295638	63.516	802.115	243278	10923.487

60.17	3698	24030	38.773	335.202	9688	2272.536
70.373	5475	45966	18.571	225.84	51753	2423.843
85.956	7676	66282	19.655	166.544	45143	2002.411
45.82	1416	6068	32.367	184.485	2278	818.494
22.944	383	1260	22.944	91	383	349.929
48.329	2285	14926	14.021	87.411	8775	661.691
113.854	14924	157451	23.51	331.037	215686	4438.542
37.935	1230	5923	29.545	163.941	2022	653.707
38.063	1192	5725	18.478	120.431	2985	643
36.238	1080	5310	16.321	98.116	2607	484.542
54.094	2472	13994	24.066	207.967	8036	1441.857
48.109	1819	9557	18.333	139.383	6300	753.12
66.858	5117	43462	17.382	134.442	31401	1627.215
60.366	3192	18766	36.91	277.856	7360	1805.482
48.922	1598	7498	21.964	151.683	3824	773.804
39.613	1349	7548	9.074	68.419	4896	447.083
50.841	2688	18371	13.146	91.933	10314	719.405
15.133	162	481	5.636	20.895	243	59.69
69.947	5790	48767	22.194	212.698	33650	2151.732
37.094	1020	4602	16.28	99.48	2505	480.125
10	42	69	10	21	42	16.05
96.456	13801	154907	46.722	496.158	81342	6800.375
44.862	1441	6804	23.521	143.513	3332	639.255
97.588	10260	92995	33.182	279.613	62762	3487.5
21.762	301	964	12.073	47.171	442	181.804
51.162	2941	22741	6.276	58.272	17777	633.452
36.856	1209	6266	16.22	88.316	3040	481.339
34.658	945	4293	23.764	121.278	1474	593.875
73.336	4494	29316	31.795	288.007	17233	2326.785
75.898	5549	38907	43.649	439.562	19738	3401.942
73.055	5318	38112	40.111	399.369	18015	2993.652
52.938	2665	18314	13.276	116.372	15527	998.985
60.769	2936	15560	37.94	291.631	5877	1708.851
36.936	1088	5345	12.42	64.87	2608	230.111
54.159	3238	27065	4.625	48.33	36235	610.167
28.037	548	1984	17.568	81.219	905	335.75
85.963	8158	69248	31.821	432.544	68589	4121.119
57.609	2823	17053	21.566	160.319	10064	1047.637
46.14	1861	10826	15.767	116.142	7439	756.286
76.524	5323	38956	26.924	294.213	39703	2543.911
32.221	869	4146	14.368	67.595	2209	342.429
25.443	521	2247	9.059	39.626	1251	189
64.717	3238	20147	27.469	196.288	11584	1318.5
43.645	1520	7617	22.597	149.121	3884	810.392
39.338	1188	5733	18.59	111.267	2784	574.333
29.887	659	2797	11.233	61.848	1633	283.107
55.186	2856	18626	23.867	182.004	10795	1062.006
48.078	1670	7903	18.973	149.932	4467	792.694
21.833	284	887	13.693	48.33	427	163.964
63.887	3954	28819	14.702	128.622	24502	1169.012

32.658	798	3389	21.306	102.873	1219	459.619
52.526	2298	12866	21.817	178.118	8999	1182.536
59.124	2942	17811	18.422	146.446	11039	1073.048
48.036	2148	13571	10.323	86.455	7497	605.522
69.158	4493	32060	27.582	264.689	26249	2056.835
53.244	2404	14676	17.657	125.995	7907	706.021
36.758	996	4035	25.776	141.663	1679	646.67
37.979	928	3774	17.049	101.192	1875	485.268
47.233	2913	24350	2.599	47.372	16399	553.714
37.164	1095	5468	15.451	91.886	2477	508.899
18.7	313	1287	2.632	16.157	692	76.259
13.533	96	206	13.533	36	96	71.143
51.825	1524	6294	30.678	188.417	2714	895.393
60.196	2917	17755	27.171	190.362	11889	1301.5
74.247	5578	40455	38.973	376.601	23794	2990.285
69.613	5063	41405	15.284	119.888	29927	1455
39.599	1214	5531	26.699	148.52	1922	735.652
35.253	1098	5048	24.944	143.572	1841	744.571
21.762	308	1017	12.575	44.679	481	136.607
55.153	3151	23450	8.817	89.737	16942	770.86
97.511	10070	84687	46.474	540.687	55040	4920.547
54.159	3238	27065	4.625	48.33	36235	605.333
72.621	4668	33317	18.668	181.64	31641	1608.881
40.087	1193	5120	26.198	143.913	1978	674.577
46.25	2080	12530	22.784	143.292	5218	983.238
69.708	4589	34410	24.885	203.917	27815	1716.738
36.167	1118	5568	11.457	63.105	2626	356.762
66.513	4752	41855	8.941	80.046	64901	1045.714
59.983	3936	33703	7.681	64.795	44265	809.488
34.855	1094	5497	12.877	72.882	2699	393.167
30.981	661	2570	19.861	89.071	1056	340.732
77.546	6782	59816	18.997	192.251	62909	2150.774
58.106	2390	12943	28.626	179.456	5740	1030.714
44.32	1550	7937	22.062	137.587	3655	601.03
59.835	2583	13834	30.778	204.137	6316	1201.964
33.67	880	4035	16.786	91.517	2199	396.143
21.693	318	1045	11.174	44.883	496	154.179
52.797	2424	14326	15.504	122.778	8930	875.381
44.102	2328	16802	9.21	70.808	8253	613.643
109.266	12307	104815	48.63	590.591	86261	5979.922
63.887	3954	28819	14.702	128.622	24502	1169.012
53.352	2560	15756	20.186	159.344	10173	886.794
48.528	2440	16984	8.72	79.557	12691	691.119
66.567	3697	24826	26.519	214.774	21916	1627.548
45.024	1909	11732	8.859	77.432	6500	543.224
60.675	3936	33473	7.214	62.242	44191	767.441
54.586	2190	11585	21.694	166.515	7225	1006.969
111.039	13462	128274	27.231	333.37	157684	4849.428
32.594	616	2172	19.04	85.17	936	357.589
22.975	400	1415	13.08	57.6	652	208.071

39.371	1089	5031	10.11	81.799	2734	483.571
36.544	864	3677	13.756	78.966	1899	372.857
51.952	1982	10385	20.025	149.54	6332	893.929
42.733	1182	5346	13.226	94.223	3013	484.786
35.684	1207	6572	17.545	97.431	2852	548.548
75.575	6676	57016	26.558	249.824	39340	2404.475
62.069	4211	33628	13.38	132.065	21061	1244.893
57.751	2415	13080	24.72	181.313	7144	938.06
34.74	843	3547	16.029	89.84	2009	414.861
35.634	1082	4898	25.247	143.39	1811	714.893
66.98	4490	30739	37.99	274.276	12136	2407.5
65.057	3963	26501	20.531	211.082	23248	1654.839
66.225	4677	38213	14.079	110.638	27609	1321.625
56.459	3627	29062	9.204	79.571	21009	1003.25
31.551	786	3313	21.645	111.794	1215	516.286
31.551	786	3313	21.645	111.794	1215	501.071
51.047	2674	17327	29.742	213.076	6078	1511.571
38.127	1478	8634	17.529	103.45	3516	628.238
24.49	396	1368	12.903	50.943	611	193
38.127	1478	8634	17.529	103.45	3516	628.238
35.296	928	4244	13.009	78.519	2252	362.79
62.079	4675	38942	15.484	161.533	27756	1551.834
56.159	2930	20205	15.101	125.835	18832	1055.714
56.159	2930	20205	15.101	125.835	18832	1055.714
39.475	1423	7163	31.084	183.212	2326	876.071
75.61	5114	34632	39.959	367.047	16540	2830.572
30.162	759	3506	11.439	56.945	1849	234.857
35.262	646	2294	25.733	111.21	952	293.41
70.896	5577	46979	16.718	216.829	53500	2371.375
42.271	1675	9558	14.958	103.407	6380	671.163
101.649	11285	105248	23.655	278.849	128816	3963.838
41.825	1460	7882	18.972	115.849	3376	699.03
17.267	210	671	7.715	27.743	314	81.875
49.308	1985	11520	18.769	133.816	10304	930.553
80.611	5641	39450	30.847	302.379	32670	2158.607
34.935	921	3943	23.669	117.764	1490	496.859
60.948	2665	13861	31.144	256.181	7851	1607.893
34.654	830	3549	16.542	92.043	1867	309.417
48.361	1885	10882	19.356	140.301	6447	652.587
68.02	3716	22725	25.779	249.941	14236	1656.83
37.504	965	4260	18.166	102.356	2196	362.132
56.591	2909	18269	21.512	164.289	11060	1105.958
49.226	1927	10380	27.267	173.012	4562	961.771
41.673	1523	7964	20.759	116.56	4097	610.897
36.624	957	4132	18.534	108.163	2260	497.75
59.413	3476	22936	35.195	277.949	8404	1922.404
44.02	2040	12415	24.015	157.955	5290	1020.019
48.233	2115	12975	10.186	86.392	7110	613.654
39.15	1365	7431	19.45	106.594	3355	516.5
50.42	2093	12364	16.425	116.579	6905	636.502

65.057	3963	26501	20.531	211.082	23248	1699.589
30.695	658	2573	19.65	91.2	1026	391.702
51.071	2969	23141	5.956	60.329	16523	758.5
85.944	7658	66071	19.983	168.298	45481	2017.078
26.6	562	2437	6.462	32.134	1268	155.762
32.594	616	2172	19.04	85.17	936	354.777
35.562	1190	6795	8.119	64.064	4306	417.957
42.76	1607	9024	24.568	149.757	3859	879.19
49.982	2640	16935	29.512	234.992	5984	1646
43.67	1545	8070	21.643	146.099	3304	692.946
34.748	865	3567	24.419	126.805	1313	458.46
40.442	1242	6210	17.406	101.055	2948	577.833
57.573	3489	25301	17.589	187.421	20214	1671.96
55.153	3151	23450	8.817	89.737	16942	759.958
44.566	1544	7613	20.068	134.004	3874	712.595
61.269	3722	25435	26.648	254.19	14684	2116.809
15.348	131	300	15.348	45	131	123.125
73.328	4474	28614	42.649	367.942	16493	2537.214
107.079	12859	122828	25.353	313.616	151564	4593.32
73.499	4634	28819	48.149	426.65	10376	3166.572
176.219	34870	452486	36.503	521.25	2016472	9117.762
67.047	5243	47429	11.801	94.348	56686	1083.125
52.465	2328	13787	17.667	131.639	7191	882.5
51.677	2376	14885	19.408	135.84	9602	846.985
39.948	1154	5224	18.147	108.059	2971	557.388
65.086	4544	36025	15.41	193.443	42774	1904.666
51.835	2393	14753	18.726	131.747	8613	864.726
42.443	1271	5845	21.233	127.409	3113	629.872

Whetm	Whetv	Whete	Whetp	J	JhetZ	Jhetm
436.058	545.152	436.309	567.487	2.603	3.2	3.199
1280.561	1853.665	1283.079	1985.468	2.424	3.003	3.002
1254.109	1485.077	1255.114	1535.328	1.738	2.178	2.177
121.837	170.139	122.129	182.203	2.171	2.967	2.966
338.936	592.319	340.28	651.991	1.869	2.516	2.515
167.847	316.341	258.738	254.531	4.874	8.247	8.335
2243.379	3226.808	2250.504	3497.145	1.594	1.834	1.834
75.987	113.087	76.261	123.401	2.029	2.749	2.749
684.651	824.318	695.173	854.928	1.591	2.137	2.138
589.697	862.637	590.676	922.739	2.222	2.699	2.699
4511.343	7453.423	4520.925	8069.16	1.842	2.123	2.123
2276.076	2937.967	2332.06	3062.827	1.588	2.032	2.033
716.916	771.605	717.368	786.733	1.704	2.095	2.095
1499.782	2061.117	1513.868	2167.988	2.379	2.895	2.895
1270.275	1721.023	1332.828	1788.164	1.589	1.925	1.925
1123.042	1516.568	1181.463	1573.41	1.607	1.94	1.94
215.266	327.508	216.058	361.821	2.005	2.632	2.632
3443.352	5277.624	3462.963	5772.39	1.486	1.829	1.832
727.896	1047.868	729.07	1118.645	2.086	2.573	2.573
824.023	1157.907	825.018	1228.655	1.534	1.777	1.776
732.948	1031.921	735.195	1115.978	1.909	2.403	2.402
375.019	637.476	476.041	628.076	1.655	3.005	3.005
697.907	859.18	707.618	889.764	1.539	2.164	2.165
1022.966	1375.346	1077.214	1424.261	1.56	1.889	1.889
1607.566	2229.95	1611.257	2384.645	1.609	1.892	1.891
1558.136	1736.906	1559.931	1782.476	1.769	1.87	1.871
1187.097	1751.871	1188.845	1872.89	1.452	1.764	1.764
5277.692	6116.706	5283.418	6334.269	1.484	1.871	1.871
788.342	1093.655	790.572	1190.667	1.654	2.218	2.218
1344.482	2167.215	1346.848	2341.336	1.999	2.478	2.477
3235.394	5400.271	3581.157	5538.763	1.534	2.327	2.327
5075.101	6616.304	5113.741	6985.61	1.151	1.344	1.344
593.002	776.927	593.876	820.02	1.86	2.364	2.364
823.652	1062.803	825.649	1127.069	1.91	2.226	2.226
2408.751	2929.939	2411	3047.132	1.418	1.539	1.539
2235.175	2956.28	2240.974	3153.006	1.117	1.247	1.246
1024.223	1201.43	1025.392	1246.077	1.483	1.653	1.653
191.016	297.999	191.806	326.47	2.226	2.978	2.978
2441.806	2546.488	2442.047	2567.919	1.559	1.712	1.712
1151.57	1374.868	1153.036	1427.751	1.412	1.808	1.807
2475.665	3120.567	2479.438	3286.844	1.201	1.61	1.61
388.163	498.459	389.013	528.235	1.849	2.463	2.463
4948.922	5745.475	4950.757	5908.544	1.431	1.529	1.529
488.752	558.74	497.163	569.005	1.646	2.335	2.335
2409.791	3791.898	2414.554	4093.938	1.115	1.411	1.411
1111.082	1520.987	1165.656	1571.221	1.581	1.944	1.945
1274.522	1726.443	1321.578	1791.406	1.565	1.91	1.91
2156.292	3101.338	2425.757	3074.51	1.327	1.78	1.78
1734.334	2679.626	2023.444	2652.777	1.302	1.836	1.837

2409.141	3522.501	2521.441	3674.76	1.396	1.744	1.744
1983.668	3117.185	2065.261	3332.281	1.628	2.076	2.076
5900.32	8163.279	6348.778	8289.094	1.227	1.519	1.519
2014.207	3099.222	2109.123	3255.85	1.621	2.056	2.056
1615.482	2467.181	1715.43	2551.503	1.675	2.129	2.128
1569.218	2094.884	1630.986	2152.808	1.576	1.946	1.946
3278.338	4545.81	3508.079	4569.815	1.374	1.785	1.785
3522.512	5471.891	3667.081	5806.974	1.395	1.796	1.796
1203.829	1815.613	1256.965	1932.272	1.606	2.013	2.013
3178.522	4856.229	3603.246	4873.789	1.347	1.843	1.843
1833.252	2866.032	1910.919	3021.563	1.618	2.05	2.05
1122.848	1593.419	1199.984	1666.261	1.681	2.449	2.454
1126.289	1520.987	1170.481	1575.652	1.581	1.925	1.925
1686.321	2331.87	1788.926	2374.456	1.334	1.712	1.712
1399.712	2067.816	1487.759	2125.646	1.557	1.975	1.975
1157.789	1552.487	1201.981	1607.152	1.581	1.88	1.88
1286.375	1775.738	1297.531	1878.611	1.473	1.668	1.668
1719.222	2411.542	1734.807	2572.445	1.493	1.781	1.781
223.797	398.934	321.358	338.822	2.177	4.297	4.302
578.877	660.345	587.649	675.332	1.916	2.357	2.358
675.058	909.867	727.107	930.3	1.687	2.273	2.274
351.773	597.504	438.894	568.342	2.382	3.913	3.916
660.318	771.787	714.16	758.1	1.687	2.315	2.316
659.282	845.573	727.998	821.526	1.803	2.532	2.533
442.82	526.174	450.828	537.469	1.649	2.165	2.166
476.26	718.996	556.37	720.448	1.961	2.736	2.736
1372.92	2043.355	1375.476	2192.825	1.359	1.775	1.775
1622.307	1953.978	1628.082	2060.901	1.364	1.632	1.634
472.742	686.064	473.919	734.823	1.802	2.4	2.4
972.269	1315.764	975.952	1406.825	1.532	1.949	1.951
1623.625	2239.098	1680.592	2339.964	2.081	2.349	2.349
230.854	379.628	237.045	405.821	2.372	3.228	3.229
1059.964	1313.231	1071.913	1364.069	1.757	2.353	2.353
214.446	318.033	226.896	336.925	1.862	2.588	2.591
808.515	1116.284	820.867	1193.162	1.538	2.069	2.069
188.334	377.623	262.466	339.772	2.531	4.131	4.164
292.359	411.431	376.848	365.532	1.928	3.462	3.463
620.747	776.667	622.034	819.8	1.727	2.121	2.121
1558.136	1736.906	1559.931	1782.476	1.769	1.87	1.871
546.633	651.205	555.901	672.175	1.793	2.353	2.353
525.057	684.907	542.238	713.271	1.936	2.583	2.585
1903.793	2536.636	2012.853	2616.83	1.461	1.827	1.828
384.879	630.061	386.189	687.557	1.648	2.161	2.16
436.394	549.008	438.396	583.36	1.966	2.67	2.676
1564.811	1679.119	1565.074	1702.521	1.432	1.484	1.484
12611.652	18637.438	12630.207	19762.305	0.822	0.936	0.935
1614.689	2311.084	1709.682	2402.01	1.839	2.384	2.384
591.105	818.056	591.918	868.033	1.864	2.314	2.313
1178.286	1370.871	1179.892	1422.003	2.245	2.599	2.599
10925.896	15737.909	10939.616	16754.303	1.415	1.616	1.616

2272.761	3471.15	2427.648	3567.063	1.501	1.987	1.987
2423.617	3266.584	2443.316	3486.22	1.061	1.234	1.234
2002.561	2349.738	2003.984	2428.344	1.833	2.104	2.104
818.569	1060.503	819.94	1125.172	2.698	3.548	3.548
349.968	489.466	350.933	525.806	3.348	3.672	3.671
661.752	782.913	662.331	810.481	1.847	2.327	2.327
4439.866	6687.997	4445.614	7134.965	1.193	1.461	1.461
653.594	1045.318	823.781	983.81	2.049	3.249	3.249
643.023	686.34	643.123	695.208	1.689	2.126	2.126
484.653	694.62	485.137	737.605	1.784	2.402	2.401
1441.943	1825.808	1445.041	1930.802	1.448	1.674	1.673
752.823	889.227	796.457	888.28	1.626	2.29	2.291
1627.26	1798.175	1628.485	1843.206	1.536	1.614	1.614
1800.26	2931.754	1812.699	3231.725	1.913	2.37	2.376
772.411	1100.49	776.533	1203.824	1.708	2.175	2.178
446.507	497.7	447.496	513.829	1.713	2.127	2.129
718.786	1051.169	721.536	1137.156	1.812	2.409	2.41
59.424	104.459	60.062	117.843	2.346	3.207	3.218
2148.816	3002.59	2214.017	3204.359	1.344	1.647	1.649
479.554	540.974	480.567	559.198	1.873	2.448	2.45
15.776	52.554	32.208	41.622	3.541	9.373	9.554
6793.176	7639.599	7177.739	7585.271	1.126	1.227	1.228
638.992	928.346	704.788	933.559	1.959	2.839	2.84
3487.741	3942.57	3488.789	4035.683	1.722	1.863	1.863
181.815	208.069	181.914	213.904	2.476	2.594	2.594
633.549	839.159	634.258	884.097	1.716	2.072	2.072
481.487	849.337	483.283	934.406	1.866	2.469	2.468
593.994	818.4	594.511	864.341	2.209	2.628	2.627
2327.688	3922.635	2332.495	4248.261	1.683	1.951	1.951
3401.921	4842.885	3700.193	4919.686	1.331	1.731	1.731
2993.18	4199.495	3280.983	4217.331	1.337	1.788	1.788
999.046	1270.284	1001.189	1346.522	1.372	1.637	1.637
1706.12	2779.703	1725.483	3024.704	1.949	2.493	2.496
229.815	406.619	327.179	348.422	2.177	4.2	4.205
610.227	746.107	610.791	776.285	1.652	1.911	1.911
335.763	361.032	335.822	366.205	2.291	2.792	2.792
4121.404	5421.109	4132.206	5776.937	1.029	1.333	1.333
1047.404	1351.868	1059.775	1416.869	1.757	2.39	2.391
756.339	933.626	757.516	979.209	1.548	1.97	1.97
2544.009	2997.807	2547.529	3127.024	1.172	1.443	1.442
342.595	759.66	343.663	873.631	1.9	2.407	2.406
189.086	390.851	189.592	443.275	1.943	2.393	2.392
1318.707	1768.003	1320.351	1870.428	1.952	2.35	2.35
810.135	871.489	819.274	877.49	1.768	2.243	2.244
574.356	617.673	574.456	626.541	1.885	2.368	2.368
283.175	492.572	295.358	532.558	1.881	2.439	2.438
1059.528	1747.617	1235.45	1784.802	1.492	2.314	2.318
792.718	914.298	793.653	950.88	1.625	2.145	2.145
163.982	227.252	164.425	243.802	2.523	2.897	2.897
1168.286	1635.141	1172.711	1754.655	1.605	2.05	2.051

459.656	622.875	460.947	667.335	2.399	2.917	2.917
1181.882	1709.968	1186.481	1852.369	1.495	1.863	1.864
1071.482	1474.93	1076.705	1586.459	1.827	2.332	2.335
604.992	744.625	623.768	765.635	1.827	2.432	2.433
2056.51	2441.612	2072.306	2539.446	1.307	1.698	1.698
705.986	1105.429	825.793	1108.676	1.741	2.736	2.736
646.202	817.688	663.479	849.271	2.34	2.915	2.917
485.329	618.449	485.83	647.719	2.1	2.434	2.434
553.744	688.479	554.92	725.067	1.637	2.02	2.02
508.936	659.014	510.208	698.768	1.902	2.276	2.276
76.264	112.405	79.043	119.531	2.029	2.711	2.711
71.153	119.442	71.552	132.8	3.377	4.582	4.582
895.413	969.155	895.943	988.602	2.333	2.704	2.704
1301.638	1912.983	1368.475	2010.54	1.694	2.107	2.106
2990.539	3987.305	2997.991	4253.091	1.407	1.806	1.806
1455.066	1580.204	1455.355	1605.823	1.712	1.804	1.804
735.468	1031.013	745.919	1100.054	2.418	2.967	2.967
744.743	1105.983	745.949	1184.454	2.053	2.523	2.523
136.633	229.571	137.191	255.526	2.51	3.576	3.576
769.813	942.533	781.941	986.501	1.612	2.148	2.15
4919.802	5497.279	5245.782	5372.863	1.158	1.493	1.494
605.401	753.313	605.992	785.954	1.652	1.929	1.929
1608.094	2218.234	1613.669	2373.292	1.415	1.773	1.773
674.769	1067.67	675.982	1151.829	2.568	3.337	3.336
983.449	1435.765	985.034	1534.932	1.789	2.144	2.144
1716.874	2394.666	1795.858	2501.45	1.47	1.852	1.851
356.803	467.02	357.695	491.64	2.145	2.822	2.821
1045.805	1281.747	1047.044	1337.623	1.461	1.652	1.652
809.557	987.492	810.496	1029.484	1.635	1.86	1.86
393.211	477.439	393.405	494.682	1.886	2.562	2.562
340.83	582.478	341.948	638.727	2.449	3.313	3.312
2150.503	2909.965	2174.452	3062.69	1.291	1.554	1.554
1030.851	1342.947	1032.112	1416.125	2.434	2.986	2.985
601.139	1057.947	654.52	1100.658	1.968	3.011	3.011
1202.135	1580.663	1203.582	1667.756	2.344	2.879	2.879
396.24	720.778	397.962	813.743	1.692	2.45	2.45
154.201	200.308	154.345	210.207	2.395	3.11	3.109
874.701	1183.146	878.068	1264.482	1.75	2.241	2.242
613.65	645.648	613.914	654.5	1.727	2.133	2.133
5981.333	8758.605	5991.289	9354.538	1.367	1.76	1.759
1168.286	1635.142	1172.711	1754.655	1.605	2.05	2.051
886.763	1352.499	1051.787	1325.628	1.53	2.483	2.483
691.15	818.765	692.132	853.202	1.478	1.857	1.857
1627.613	2162.135	1702.347	2243.678	1.426	1.765	1.765
542.988	662.568	552.245	686.046	1.789	2.364	2.365
767.552	999.857	768.367	1049.968	1.675	1.96	1.959
1007.061	1387.913	1009.638	1499.57	1.771	2.215	2.215
4849.901	6064.926	4855.922	6351.924	1.299	1.371	1.37
357.614	431.209	358.09	449.305	2.903	3.198	3.198
208.117	316.303	208.594	341.213	2.147	2.917	2.917

483.589	565.029	484.276	587.4	1.766	2.002	2.002
372.884	451.774	373.392	471.205	2.204	2.655	2.655
893.999	1215.332	896.281	1311.525	1.779	2.207	2.207
484.809	562.319	485.32	582.204	1.849	2.309	2.309
548.675	830.536	549.727	893.106	1.686	2.131	2.131
2404.438	3311.31	2612.881	3349.263	1.368	1.775	1.775
1245.012	1756.166	1249.145	1894.406	1.523	1.881	1.881
938.203	1484.627	1008.01	1536.861	1.821	2.639	2.639
414.664	520.446	422.501	541.623	1.879	2.41	2.411
715.05	1087.383	716.656	1172.645	2.086	2.618	2.617
2407.787	2949.249	2409.034	3060.099	2.026	2.18	2.179
1655.103	2498.346	1660.512	2712.083	1.311	1.738	1.738
1321.681	1426.965	1321.923	1448.519	1.677	1.782	1.782
1003.271	1042.978	1003.363	1051.107	1.603	1.671	1.671
516.325	687.689	517.682	734.402	2.137	2.557	2.557
501.184	760.795	502.253	819.633	2.137	2.642	2.641
1511.791	1979.287	1513.388	2081.269	1.7	2.014	2.013
628.376	927.047	629.438	992.71	1.715	2.184	2.183
193.008	207.447	193.041	210.403	2.6	3.228	3.228
628.376	927.047	629.438	992.71	1.715	2.184	2.183
362.575	425.304	370.252	436.384	2.008	2.759	2.76
1552.009	2276.234	1600.63	2423.128	1.346	1.706	1.706
1055.802	1245.158	1057.238	1284.12	1.46	1.77	1.77
1055.802	1245.158	1057.238	1284.12	1.46	1.77	1.77
876.086	1396.927	1000.44	1432.749	2.017	2.837	2.837
2830.813	3583.482	2835.495	3772.848	1.603	1.904	1.904
233.454	457.426	253.198	505.558	1.871	2.952	2.965
291.441	476.891	407.267	398.17	2.841	4.513	4.545
2370.491	2919.133	2375.916	3075.862	1.085	1.267	1.268
671.196	833.623	672.476	881.187	1.496	1.959	1.959
3964.159	4860.472	3969.146	5079.273	1.291	1.359	1.358
699.08	908.54	700.836	964.635	1.925	2.267	2.266
81.889	108.962	81.952	114.505	2.396	3.321	3.321
930.749	1321.127	931.884	1403.564	1.341	1.548	1.547
2158.618	3173.707	2391.136	3191.447	1.498	2.158	2.158
496.857	704.968	541.12	703.067	2.378	3.202	3.202
1608.01	2142.076	1638.198	2259.751	1.624	1.894	1.894
309.372	524.908	389.023	494.893	1.848	3.242	3.242
652.526	1066.726	778.774	1040.933	1.576	2.608	2.608
1656.766	2263.884	1835.365	2284.734	1.433	1.898	1.898
362.089	609.422	450.874	576.59	1.903	3.286	3.287
1105.104	1258.99	1161.468	1251.111	1.638	2.22	2.221
961.815	1371.029	1025.773	1409.415	1.903	2.455	2.455
610.857	789.045	678.278	764.314	1.874	2.64	2.64
497.756	555.001	514.487	554.015	1.786	2.336	2.336
1922.743	2938.3	2003.645	3085.925	1.671	2.137	2.137
1020.106	1437.309	1023.076	1564.891	1.601	2.071	2.071
613.41	758.728	623.314	788.82	1.841	2.415	2.415
515.492	921.43	583.216	947.528	1.795	2.606	2.61
636.494	993.811	724.417	1006.614	1.726	2.663	2.663

1699.869	2573.193	1705.348	2793.087	1.311	1.701	1.701
391.744	505.814	392.363	533.477	2.379	2.852	2.852
758.519	794.617	758.602	802.007	1.623	1.7	1.7
2017.228	2364.405	2018.651	2443.011	1.82	2.087	2.087
155.785	243.142	156.42	266.269	2.185	2.915	2.915
354.793	423.447	359.407	437.355	2.903	3.218	3.218
417.741	521.247	433.798	537.086	1.511	1.897	1.898
879.397	1416.8	888.876	1533.632	1.708	2.088	2.088
1646.419	2554.225	1649.673	2754.13	1.446	1.782	1.782
692.915	1210.026	810.745	1231.881	1.778	2.635	2.635
458.416	731.618	547.598	705.618	2.331	3.585	3.586
577.888	710.986	578.486	741.749	2.073	2.393	2.393
1671.754	2387.641	1688.545	2583.42	1.142	1.353	1.353
759.364	921.733	780.331	949.897	1.612	2.173	2.174
712.72	987.817	713.76	1048.705	1.939	2.564	2.564
2117.042	3025.931	2123.787	3268.19	1.325	1.552	1.552
123.134	139.377	123.171	142.703	3.5	3.696	3.696
2537.343	3051.456	2541.028	3193.334	1.585	1.957	1.956
4593.774	5737.224	4599.311	6005.721	1.282	1.352	1.352
3166.88	3945.493	3170.615	4128.349	1.835	2.122	2.122
9118.517	10807.198	9125.621	11184.491	1.213	1.418	1.418
1083.246	1385.8	1085.095	1455.428	1.574	1.969	1.969
882.603	1002.403	883.052	1021.617	1.778	2.182	2.182
847.042	1098.478	849.051	1168.922	1.66	2.302	2.302
557.498	882.997	559.297	966.285	1.981	2.456	2.456
1904.384	2435.808	1931.561	2558.68	1.065	1.298	1.298
864.765	1027.891	866.057	1071.802	1.727	2.292	2.292
629.641	751.731	638.85	775.082	1.963	2.541	2.542

Jhetv	Jhete	Jhetp	MAXDN	MAXDP	DELS	TIE
2.594	3.198	2.5	2.728	4.097	30.427	53.718
2.069	2.996	1.932	2.004	4.822	32.635	91.841
1.883	2.176	1.834	2.536	5.881	48.858	77.209
2.186	2.96	2.05	1.782	3.524	12.969	15.007
1.472	2.505	1.34	2.146	4.375	23.213	23.814
4.457	5.2	5.624	6.108	3.717	44.362	72.47
1.288	1.828	1.191	1.841	5.953	30.633	71.658
1.914	2.739	1.769	1.928	3.849	10.922	8.79
1.78	2.113	1.715	1.003	2.117	12.959	23.913
1.82	2.694	1.7	1.829	3.773	14.853	39.185
1.291	2.118	1.193	3.089	5.578	111.034	210.858
1.608	1.989	1.549	1.651	6.588	25.682	68.252
1.982	2.094	1.954	0.412	0.42	5.347	23.435
2.196	2.873	2.101	2.223	5.881	42.289	89.296
1.426	1.83	1.376	2.723	5.348	47.698	80.439
1.434	1.838	1.386	2.7	5.334	41.204	71.187
1.711	2.621	1.547	1.656	4.603	9.207	15.947
1.213	1.824	1.117	6.259	5.584	94.998	131.362
1.777	2.569	1.665	2.024	3.747	22.323	50.047
1.258	1.774	1.186	1.961	5.362	23.365	40.829
1.673	2.394	1.542	1.943	5.772	26.079	44.905
1.795	2.318	1.861	2.52	3.902	24.165	26.565
1.758	2.143	1.693	2.532	4.038	24.887	34.885
1.4	1.786	1.355	2.674	5.268	36.824	63.171
1.365	1.887	1.276	0.893	2.588	11.478	40.664
1.724	1.869	1.692	3.23	8.859	66.406	110.395
1.197	1.761	1.121	1.821	3.803	17.952	43.07
1.623	1.869	1.569	2.588	5.334	61.273	137.192
1.618	2.212	1.491	0.514	0.273	5.105	23.723
1.545	2.473	1.431	1.846	3.8	21.235	61.941
1.385	2.088	1.357	5.056	6.5	56.394	100.252
1.026	1.337	0.97	3.187	7.452	66.807	122.813
1.807	2.361	1.716	1.948	4.192	15.502	34.927
1.707	2.22	1.607	1.497	5.693	14.387	40.656
1.295	1.537	1.252	2.159	6.195	38.184	75.963
0.944	1.243	0.886	1.602	5.51	28.676	55.787
1.43	1.651	1.384	1.853	5.95	21.528	42.964
1.957	2.966	1.793	2.027	4.672	20.309	23.368
1.662	1.712	1.652	1.948	4.127	29.285	88.669
1.529	1.805	1.475	3.04	6.01	38.646	50.56
1.3	1.607	1.239	2.668	4.819	30.255	51.422
1.93	2.457	1.825	2.14	4.753	12.557	21.613
1.349	1.529	1.318	2.676	7.451	66.801	157.773
2.087	2.307	2.052	2.487	4.025	19.531	29.331
0.902	1.408	0.836	2.239	4.192	24.36	44.574
1.424	1.853	1.381	2.926	5.276	43.593	68.332
1.416	1.838	1.369	2.818	5.324	47.139	74.66
1.242	1.58	1.258	2.876	5.714	52.35	85.453
1.2	1.573	1.222	2.828	5.598	45.64	70.518

1.208	1.663	1.165	3.034	5.893	52.975	91.413
1.348	1.992	1.269	2.967	5.718	60.518	100.605
1.109	1.415	1.093	2.949	6.623	90.641	166.656
1.365	1.96	1.307	2.986	5.659	58.841	98.858
1.415	2	1.374	3.017	5.831	54.713	88.1
1.457	1.867	1.421	2.84	5.502	48.705	83.46
1.291	1.669	1.283	5.775	5.875	80.984	128.491
1.171	1.722	1.109	3.153	6.114	69.356	125.138
1.364	1.928	1.29	2.844	5.473	44.929	67.176
1.224	1.623	1.229	2.975	5.915	69.657	116.831
1.342	1.96	1.282	3.02	5.57	57.119	90.7
1.773	2.32	1.696	5.856	5.125	51.518	67.338
1.424	1.847	1.378	2.795	5.311	40.655	65.602
1.237	1.612	1.218	3.034	5.615	41.221	67.178
1.35	1.855	1.318	2.922	5.428	48.268	76.564
1.399	1.806	1.354	2.785	5.336	40.956	66.911
1.202	1.654	1.137	1.657	5.814	17.693	44.989
1.295	1.768	1.216	2.589	3.505	22.627	45.87
2.569	3.075	2.996	5.01	4.547	40.474	46.826
2.11	2.331	2.07	1.013	1.855	8.145	26.18
1.683	2.089	1.661	0.869	2.098	7.501	24.122
2.271	3.071	2.416	4.744	4.702	30.33	51.147
2	2.119	2.059	0.868	2.098	7.331	24.428
2.053	2.327	2.104	4.976	4.963	43.538	59.447
1.861	2.139	1.825	1.503	3.964	18.307	25.66
1.816	2.314	1.83	1.163	2.427	11.249	24.963
1.196	1.771	1.115	2.038	4.907	26.614	42.816
1.379	1.629	1.316	5.531	4.765	31.877	53.128
1.715	2.394	1.614	3.008	5.085	32.837	34.554
1.474	1.944	1.386	2.942	6.608	40.696	54.517
1.711	2.281	1.634	2.695	5.901	47.284	108.986
1.948	3.165	1.817	2.674	3.741	19.74	29.305
1.96	2.334	1.895	2.645	6.011	33.986	54.726
1.771	2.475	1.667	1.075	1.867	12.116	15.379
1.508	2.045	1.411	0.949	2.072	12.418	25.427
1.994	2.895	2.245	3.648	5.189	20.815	32.903
2.445	2.617	2.799	4.4	5.175	26.768	35.001
1.694	2.117	1.605	0.444	0.744	5.854	21.223
1.724	1.869	1.692	3.23	8.859	66.406	110.395
2.007	2.324	1.947	1.695	5.024	16.992	28.276
1.997	2.519	1.911	2.556	3.827	22.455	35.721
1.38	1.735	1.338	2.737	6.064	53.98	91.247
1.332	2.153	1.222	2.019	4.44	23.259	25.352
2.258	2.667	2.159	2.989	5.52	40.557	52.893
1.408	1.484	1.394	1.883	6.116	34.718	63.925
0.635	0.934	0.599	2.329	6.558	96.666	172.756
1.693	2.218	1.652	2.25	6.555	30.545	65.684
1.703	2.31	1.612	1.317	2.583	7.195	24.314
2.263	2.596	2.189	2.127	5.751	20.152	62.626
1.145	1.614	1.079	3.603	7.945	141.96	274.852

1.321	1.885	1.275	4.203	4.218	38.316	89.163
0.918	1.226	0.86	1.732	5.489	27.777	48.491
1.859	2.103	1.812	4.216	6.635	88.052	135.776
2.846	3.542	2.705	2.87	5.101	45.335	101.992
2.617	3.661	2.433	1.312	2.904	14.712	48.52
1.993	2.325	1.931	2.487	4.286	22.897	38.608
0.997	1.459	0.939	2.77	6.385	81.853	104.978
2.076	2.569	2.221	4.833	3.649	28.236	53.91
2.027	2.125	2.008	2.616	4.734	21.416	37.669
1.672	2.399	1.575	2.508	3.929	18.119	30.045
1.314	1.67	1.241	1.436	5.516	13.79	40.667
1.946	2.16	1.954	2.529	4.11	21.353	35.176
1.484	1.613	1.453	1.601	6.038	27.037	63.893
1.506	2.362	1.378	5.884	5.408	68.98	101.087
1.583	2.168	1.461	2.945	6.112	39.14	51.051
1.969	2.126	1.925	2.15	5.178	30.434	38.913
1.694	2.402	1.573	2.206	5.468	36.803	48.482
1.931	3.189	1.731	2.668	3.985	20.678	15.935
1.196	1.592	1.13	5.601	5.311	39.418	60.843
2.236	2.446	2.182	2.623	5.967	25.401	40.038
2.923	4.481	3.783	5.627	2.426	18.767	10.057
1.118	1.167	1.132	6.792	4.963	73.851	149.32
2.017	2.597	2.003	5.023	4.349	41.462	58.993
1.681	1.862	1.65	2.619	5.893	58.372	144.325
2.337	2.593	2.288	2.373	3.576	15.811	26.722
1.631	2.07	1.56	1.745	3.973	19.156	32.03
1.412	2.459	1.285	2.194	4.456	29.832	34.459
1.886	2.625	1.783	2.422	3.943	18.549	48.152
1.16	1.947	1.073	2.543	5.019	63.973	116.522
1.214	1.583	1.199	4.899	5.536	55.037	119.621
1.286	1.627	1.285	4.884	5.492	51.158	102.947
1.291	1.633	1.22	1.7	5.741	17.354	34.418
1.551	2.474	1.431	5.798	5.444	58.645	97.474
2.53	3.028	2.929	4.986	4.634	40.912	47.946
1.615	1.91	1.564	1.699	5.493	25.831	36.574
2.643	2.791	2.615	2.438	3.762	14.013	32.257
1.013	1.329	0.95	1.809	5.93	24.81	55.749
1.882	2.37	1.797	2.621	5.987	33.988	54.68
1.598	1.967	1.523	2.521	5.356	24.691	39.01
1.226	1.44	1.176	1.729	6.361	23.68	53.504
1.15	2.399	1.01	2.943	3.136	34.868	31.333
1.217	2.386	1.083	2.871	3.276	26.754	23.001
1.826	2.347	1.739	2.183	5.904	35.385	64.816
2.127	2.225	2.117	2.823	4.924	27.407	48.886
2.243	2.367	2.22	2.565	5.244	24.413	41.579
1.412	2.341	1.309	2.098	4.442	22.403	24.987
1.44	1.963	1.435	5.674	5.521	45.147	54.645
1.868	2.142	1.801	1.483	2.918	16.148	30.69
2.08	2.889	1.937	2.062	4.197	18.49	27.14
1.495	2.044	1.4	2.982	6.984	45.653	64.289

2.143	2.908	1.999	1.605	4.892	11.361	32.893
1.305	1.857	1.208	2.205	6.448	37.839	53.964
1.74	2.324	1.628	3.088	7.193	54.524	83.43
2.028	2.379	1.974	2.831	4.789	27.694	37.484
1.443	1.688	1.39	1.631	3.765	21.622	46.325
1.761	2.326	1.76	4.814	5.571	43.849	60.64
2.356	2.86	2.269	2.657	3.687	22.619	50.017
1.976	2.432	1.9	1.732	5.386	15.305	33.455
1.635	2.016	1.557	0.902	1.679	7.92	19.841
1.748	2.27	1.647	1.533	5.33	12.566	30.585
1.883	2.636	1.774	1.458	1.244	8.821	7.648
2.729	4.556	2.455	1.756	2.052	8.987	10.956
2.536	2.703	2.497	1.832	6.174	14.273	52.975
1.459	1.999	1.395	2.713	5.751	45.727	79.346
1.365	1.801	1.281	2.967	5.37	54.313	94.24
1.699	1.804	1.68	2.822	5.415	48.948	89.177
2.16	2.932	2.03	1.879	5.137	22.196	53.261
1.699	2.519	1.588	1.824	3.726	16.204	42.041
2.164	3.56	1.95	2.518	3.373	20.22	22.692
1.78	2.125	1.707	1.96	6.302	22.875	33.59
1.346	1.399	1.38	2.379	4.736	34.739	93.183
1.611	1.927	1.556	1.928	4.441	24.162	34.89
1.309	1.767	1.228	2.968	7.32	48.612	69.637
2.142	3.331	1.992	1.968	4.119	17.049	46.277
1.472	2.14	1.378	2.093	3.995	29.321	60.713
1.344	1.769	1.291	2.698	6.181	45.074	78.044
2.198	2.814	2.098	2.875	4.915	26.251	35.283
1.38	1.65	1.329	2.03	6.08	30.319	46.481
1.565	1.858	1.51	2.132	5.818	30.723	46.136
2.212	2.561	2.153	2.474	3.916	17.06	27.865
1.967	3.302	1.8	2.029	4.372	12.509	28.048
1.171	1.542	1.116	2.013	5.543	27.663	53.58
2.403	2.982	2.3	2.55	5.341	45.346	84.237
1.744	2.792	1.673	4.442	4.324	36.578	54.264
2.291	2.876	2.191	2.528	5.546	45.409	87.214
1.37	2.439	1.217	2.691	4.061	32.874	35.754
2.53	3.107	2.435	2.128	3.227	20.806	26.664
1.701	2.234	1.601	2.967	6.495	39.715	57.611
2.056	2.132	2.036	0.478	0.726	5.611	21.441
1.206	1.756	1.13	2.851	6.015	84.274	165.569
1.495	2.044	1.4	2.982	6.984	45.653	64.289
1.645	2.059	1.699	2.551	5.68	25.351	42.339
1.595	1.855	1.537	1.387	5.923	14.814	28.576
1.329	1.686	1.283	2.709	5.927	45.092	75.271
1.979	2.336	1.916	2.767	4.737	23.396	31.787
1.576	1.958	1.514	2.15	5.749	32.886	47.072
1.571	2.208	1.448	1.987	5.892	30.603	54.443
1.128	1.369	1.083	1.767	5.528	36.969	98.697
2.673	3.193	2.571	2.386	4.892	27.582	56.49
1.975	2.91	1.838	1.732	3.681	11.228	16.951

1.708	1.999	1.642	0.875	0.811	4.235	21.008
2.237	2.652	2.155	2.56	4.976	28.013	41.448
1.578	2.2	1.455	1.921	5.869	23.604	46.205
2.019	2.307	1.959	2.389	5.313	20.272	32.555
1.398	2.127	1.3	1.832	3.787	14.716	28.148
1.29	1.628	1.277	4.404	5.742	31.623	67.06
1.335	1.875	1.238	1.898	6.224	28.037	45.082
1.685	2.457	1.63	5.093	5.072	43.086	62.03
1.959	2.372	1.887	2.503	3.889	18.457	29.839
1.714	2.612	1.59	1.877	3.858	20.716	46.357
1.812	2.178	1.754	2.762	5.43	53.383	138.949
1.166	1.732	1.075	1.777	5.401	32.078	45.749
1.685	1.782	1.667	2.873	6.286	49.792	84.685
1.628	1.671	1.619	1.451	5.154	21.186	47.504
1.931	2.551	1.811	1.711	4.744	14.408	33.611
1.732	2.636	1.609	1.961	4.633	16.478	33.746
1.547	2.011	1.474	1.931	5.54	24.901	61.207
1.473	2.18	1.376	1.832	3.844	14.023	30.779
3.04	3.227	3.006	1.188	3.93	8.667	22.958
1.473	2.18	1.376	1.832	3.844	14.023	30.779
2.394	2.718	2.336	1.461	1.904	14.857	22.785
1.18	1.648	1.114	1.41	2.795	17.061	34.779
1.532	1.768	1.491	1.837	5.152	19.858	38.492
1.532	1.768	1.491	1.837	5.152	19.858	38.492
1.796	2.454	1.772	2.485	3.349	19.327	48.799
1.519	1.9	1.445	2.708	6.071	35.611	86.384
1.614	2.738	1.487	5.927	3.887	28.484	25.286
2.912	3.27	3.464	6.216	4.036	47.6	53.162
1.037	1.265	0.986	1.97	5.922	33.039	54.821
1.611	1.955	1.532	0.742	1.152	8.053	20.491
1.136	1.357	1.093	1.773	5.491	37.32	87.416
1.729	2.26	1.627	1.504	5.604	14.007	37.104
2.654	3.319	2.552	2.78	3.262	17.135	16.479
1.089	1.545	1.025	1.989	5.424	27.7	40.662
1.488	1.944	1.485	4.615	6.329	48.838	83.415
2.316	2.959	2.318	4.209	4.111	25.594	54.753
1.426	1.864	1.351	1.511	5.913	18.251	51.839
1.885	2.524	2.024	4.561	4.889	26.166	33.091
1.566	2.161	1.61	4.614	5.401	27.121	39.412
1.382	1.722	1.366	2.484	6.214	36.013	66.117
1.912	2.584	2.042	4.595	5.035	27.346	38.085
2.017	2.136	2.031	2.611	5.656	35.003	60.008
1.764	2.314	1.717	4.788	5.41	35.714	63.709
2.114	2.394	2.184	4.148	4.617	20.375	40.429
2.14	2.263	2.152	2.535	5.017	23.511	37.891
1.427	2.06	1.359	4.758	4.726	35.012	74.561
1.462	2.065	1.341	1.483	3.204	16.847	36.496
1.993	2.386	1.922	2.753	5.105	24.497	35.731
1.501	2.355	1.442	4.964	3.708	30.74	34.09
1.72	2.321	1.706	4.769	5.434	42.66	55.821

1.138	1.695	1.049	1.61	5.509	33.157	49.224
2.267	2.847	2.159	2.086	3.848	22.105	44.666
1.647	1.7	1.636	1.416	4.787	20	40.951
1.847	2.086	1.801	4.066	6.632	88.018	139.278
1.916	2.904	1.756	2.037	4.441	19.764	20.535
2.709	3.184	2.623	2.21	5.025	22.657	51.042
1.52	1.84	1.469	0.788	2.087	6.453	17.047
1.279	2.062	1.181	1.913	3.947	22.701	39.745
1.142	1.778	1.059	2.13	3.975	27.65	56.19
1.476	2.22	1.456	4.742	5.073	30.165	52.267
2.184	2.926	2.289	4.692	4.815	28.003	54.806
2.022	2.391	1.954	1.961	5.189	14.613	38.149
0.947	1.342	0.875	1.73	5.27	19.153	36.06
1.805	2.131	1.749	1.062	2.274	17.083	28.308
1.954	2.561	1.858	1.513	1.848	21.329	33.017
1.095	1.547	1.015	1.984	4.948	26.699	54.597
3.323	3.695	3.258	2.302	3.479	12.307	28.673
1.64	1.954	1.57	2.643	5.862	34.368	84.476
1.114	1.35	1.07	1.777	5.495	36.843	92.66
1.748	2.12	1.678	1.83	4.242	25.457	94.24
1.227	1.417	1.192	3.524	8.172	83.772	154.399
1.561	1.965	1.49	1.856	5.921	19.678	37.447
1.938	2.181	1.907	2.094	5.814	33.29	53.963
1.792	2.296	1.688	1.671	4.74	20.617	35.908
1.562	2.448	1.431	2.249	4.713	33.012	48.858
1.015	1.283	0.964	1.617	4.566	20.71	38.048
1.933	2.289	1.855	1.596	5.261	15.02	34.291
2.171	2.515	2.109	2.587	5.487	27.702	46.047

S0K	S1K	S2K	S3K	PHI	BLI	PW2
69.487	13.778	5.869	3.333	4.757	0.976	0.568
108.039	20.46	10.316	7.701	8.794	1.04	0.563
116.211	17.836	7.103	3.538	4.873	0.843	0.588
34.054	7.92	3.28	2.495	2.362	0.886	0.565
64	11.069	4.609	2.24	3.189	0.814	0.564
41.303	14.234	4.393	3.115	4.466	1.574	0.625
139.207	22.667	10.338	5.018	7.811	0.998	0.567
33.219	5.471	1.836	0.729	1.004	0.749	0.576
94.107	13.382	5.166	2.21	3.143	0.891	0.586
73.059	14.904	8.035	5.846	6.653	1.06	0.546
212.877	33.514	14.459	7.335	12.115	0.933	0.593
143.58	24.83	11.882	5.959	9.517	1.186	0.566
72.239	14.096	6.278	3.086	4.214	1.013	0.567
134.606	22.432	10.623	5.157	8.51	0.964	0.557
110.096	18.201	6.287	3.038	4.577	0.954	0.614
104.039	17.271	6.063	2.844	4.363	0.976	0.607
46.548	9.127	3.368	1.511	2.196	0.902	0.584
165.24	27.185	10.487	6.533	7.705	0.825	0.607
74.711	15.797	8.02	7.034	6.668	1.008	0.563
82.239	14.705	6.266	3.029	4.388	1.013	0.578
96.107	15.042	5.398	2.241	3.691	0.937	0.592
78.711	12.08	4.607	2.07	2.929	0.851	0.589
86.239	13.959	5.413	2.741	3.598	0.889	0.595
98.042	16.342	5.84	2.857	4.15	0.99	0.601
114.382	20.069	10.605	6.459	7.883	1.074	0.556
134.606	19.944	5.842	2.104	4.161	0.982	0.633
90.107	17.419	9.259	8.336	7.331	1.088	0.561
231.329	31.798	13.024	6.608	9.631	0.909	0.592
92.107	14.98	6.447	3.147	4.39	0.96	0.572
96.042	20.071	11.545	10.42	10.075	1.082	0.554
191.376	28.399	12.057	6.885	8.78	0.963	0.584
229.329	29.672	10.248	4.213	7.072	1.01	0.616
75.956	14.257	5.454	3.441	4.093	1.027	0.592
82.239	16.193	7.721	4.149	5.953	1.124	0.563
166.219	22.241	6.47	2.432	4.233	1.016	0.637
114.606	19.633	8.774	4.676	6.152	1.038	0.577
108.039	15.366	5.299	2.049	3.392	1.062	0.605
53.303	9.214	2.819	1.092	1.855	0.822	0.615
145.207	23.76	9.305	5.49	7.37	1.142	0.605
120.211	15.594	5.18	1.986	3.107	0.859	0.609
162.465	21.013	8.946	4.023	5.696	0.846	0.574
61.059	10.903	4.222	1.794	2.558	0.859	0.577
217.66	30.772	9.665	4.39	7.254	1.05	0.634
80.711	12.363	4.458	2.06	2.901	0.911	0.6
147.207	20.691	9.957	4.961	6.867	0.91	0.563
106.039	17.296	6.762	3.079	4.873	0.983	0.594
112.096	17.95	6.804	3.181	4.885	0.954	0.601
149.58	21.99	9.104	4.267	6.458	0.988	0.591
140.881	20.567	8.577	4.271	6.083	1.042	0.588

154	23.331	9.196	4.506	6.705	1.043	0.592
147.207	22.426	9.26	4.593	6.922	0.951	0.586
236.215	32.131	13.382	6.441	9.772	0.971	0.595
147.207	22.68	9.428	4.694	7.127	0.969	0.584
134.606	21.107	8.405	3.945	6.336	0.994	0.587
124.382	19.639	7.862	3.662	5.719	0.96	0.588
174.117	26.011	10.127	5.241	7.317	1.008	0.6
184.75	27.198	10.872	5.698	7.991	0.963	0.6
116.096	18.164	7.311	3.382	5.312	0.987	0.584
186.117	26.718	10.959	5.369	8.133	0.99	0.592
140.881	21.386	9.001	4.326	6.638	0.888	0.578
107.457	18.34	6.366	3.885	4.491	0.962	0.616
106.039	17.019	6.59	2.982	4.673	0.975	0.594
128.606	19.31	8.155	3.933	5.624	1.035	0.582
122.211	19.235	8.006	4.091	5.923	1.035	0.585
110.039	17.271	6.747	3.07	4.855	1.008	0.594
100.039	18.227	9.793	5.465	7.437	1.234	0.553
116.382	19.993	9.973	5.862	7.385	0.99	0.564
65.487	12.482	3.763	2.202	2.763	1.25	0.628
70.711	14.041	6.692	4.051	4.946	1.028	0.564
90.239	14.917	6.406	3.267	4.55	1.087	0.576
63.487	14.127	6.121	4.44	5.087	1.214	0.573
90.239	14.705	6.266	3.179	4.388	1.078	0.576
96.107	15.154	4.877	2.368	3.36	1.016	0.618
71.059	11.662	4.357	1.941	2.823	0.913	0.595
69.487	13.881	7.99	5.115	6.524	1.121	0.534
112.096	17.283	8.02	4.3	5.545	0.876	0.564
113.457	18.479	7.929	4.662	5.635	0.945	0.583
80.711	12.142	4.33	1.803	2.767	0.813	0.593
104.039	15.609	5.867	2.84	3.816	0.904	0.596
128.382	23.358	10.21	5.366	8.833	1.211	0.586
47.303	11.18	4.089	3.082	3.266	0.959	0.603
112.096	17.522	6.904	3.277	4.839	0.888	0.587
47.303	9.926	4.275	2.266	3.031	1.004	0.573
100.042	14.851	6.06	2.909	3.913	0.954	0.586
47.303	12.756	6.31	3.656	5.749	1.552	0.541
45.487	11.8	4.298	2.366	2.984	1.074	0.599
72.439	13.351	6.209	2.795	4.145	1.02	0.553
134.606	19.944	5.842	2.104	4.161	0.982	0.633
82.439	13.042	5.198	2.413	3.39	0.915	0.584
74.711	14.029	6.171	3.548	4.557	0.951	0.575
139.207	21.653	7.534	3.386	5.437	0.966	0.614
69.487	10.745	4.126	1.733	2.608	0.862	0.581
75.059	12.368	4.649	2.453	3.194	0.79	0.594
128.382	18.335	5.904	2.359	4.009	1.07	0.625
308.764	39.759	14.957	7.015	11.013	1.025	0.616
134.881	21.69	9.658	5.13	7.224	0.998	0.575
58.711	13.787	7.076	4.196	5.135	0.995	0.55
102.096	19.301	8.375	4.148	6.466	1.028	0.579
325.008	43.74	15.646	7.511	11.799	0.921	0.613

128.881	23.486	10.301	9.991	8.343	1.274	0.589
143.207	19.823	8.194	3.841	5.414	0.951	0.589
158	22.641	7.233	2.731	5.118	0.858	0.621
92.107	17.635	7.552	4.05	6.054	0.882	0.575
39.303	13.844	9.401	6.904	9.296	1.261	0.514
90.239	14.456	5.375	2.238	3.7	0.991	0.581
218.477	28.422	10.976	4.654	7.428	0.861	0.599
82.439	17.325	7.898	8.24	6.841	1.318	0.577
72.711	13.191	6.077	3.604	4.219	0.911	0.567
71.059	12.495	5.584	3.269	3.876	0.904	0.571
104.096	17.849	8.937	4.778	6.381	1.046	0.559
87.353	15.042	6.489	3.496	4.436	0.982	0.575
123.627	19.335	6.257	3.04	4.48	1.086	0.624
125.096	22.638	9.707	8.504	7.848	0.888	0.592
86.107	14.606	5.815	3.149	3.861	0.799	0.584
61.059	10.855	3.885	1.599	2.343	0.842	0.591
98.107	14.73	5.54	2.432	3.709	0.852	0.588
28.529	6.074	2.051	1.192	1.384	0.725	0.585
138.452	21.665	9.291	5.034	6.71	1.008	0.583
71.059	12.469	5.113	2.657	3.542	0.895	0.582
17.651	6.792	1.721	2.483	1.67	1.423	0.631
210.905	32.879	13.732	7.739	11.012	1.167	0.599
90.239	15.849	6.07	3.766	4.581	1.032	0.599
190.75	28.677	10.01	4.503	7.759	1.072	0.613
39.02	9.684	4.012	2.557	3.238	1.151	0.561
92.239	13.467	4.65	1.834	2.982	0.946	0.598
71.059	12.976	5.451	2.7	3.929	0.82	0.572
73.059	14.878	6.123	4.552	5.06	1.044	0.594
153.58	25.161	10.207	5.376	8.285	0.99	0.6
162.974	26.398	11.48	6.915	8.913	1.108	0.59
156.465	25.505	11.842	7.44	9.152	1.087	0.579
98.042	15.01	5.825	2.631	3.802	0.99	0.594
125.851	22.06	9.808	7.031	7.727	0.884	0.584
65.487	12.76	3.913	2.305	2.937	1.287	0.628
92.239	12.336	3.751	1.26	2.204	0.945	0.619
52.603	11.929	5.083	3.484	4.043	1.064	0.58
184.75	24.883	11.508	6.507	7.739	0.911	0.579
112.096	17.522	6.904	3.277	4.839	0.889	0.587
88.239	13.686	5.246	2.398	3.419	0.887	0.592
150	20.878	8.859	3.981	5.78	0.961	0.575
44	11.472	4.465	2.406	3.201	0.782	0.581
44.106	8.99	3.285	1.567	2.272	0.837	0.588
126.382	19.576	7.822	3.639	5.671	0.893	0.585
88.239	15.173	6.487	3.66	4.687	0.908	0.584
76.711	13.191	5.596	2.955	3.885	0.903	0.578
58.603	10.792	4.426	2.273	3.184	1.027	0.578
111.342	17.748	7.047	4.317	5.003	0.958	0.595
74.107	13.711	6.019	2.793	3.751	0.832	0.563
43.02	9.361	3.787	1.915	2.954	1.063	0.571
118.211	17.465	6.339	2.747	4.258	0.889	0.604

55.487	13.881	6.528	3.953	5.331	1.088	0.562
106.039	17.145	7.468	4.058	5.335	0.911	0.58
116.096	17.836	6.734	3.026	4.805	0.902	0.595
92.239	14.233	5.582	2.712	3.783	0.904	0.591
143.207	20.579	9.416	4.428	6.459	0.941	0.565
102.042	16.117	5.709	2.67	4	1.06	0.607
70.711	16.405	8.507	5.466	7.345	1.12	0.551
67.059	13.255	5.639	2.653	4.152	1.042	0.566
86.439	12.071	4.691	1.981	2.831	0.952	0.582
65.059	13.255	5.639	2.997	4.152	1.053	0.582
33.219	5.832	2.042	0.837	1.191	0.875	0.576
24.529	8.234	3.297	3.787	3.016	0.897	0.572
80.042	17.432	7.566	3.536	5.735	1.041	0.571
112.211	19.172	6.852	3.044	5.052	0.948	0.599
162.465	24.104	10.381	6.248	7.582	0.836	0.589
126.382	19.049	5.857	2.284	4.132	1.008	0.625
82.439	17.079	8.343	4.826	7.125	1.056	0.561
74.711	16.12	9.046	7.28	7.675	1.064	0.546
41.02	9.001	3.538	1.756	2.654	0.86	0.565
104.042	14.316	5.413	2.313	3.369	0.883	0.592
215.66	29.541	12.507	8.082	9.012	1.034	0.592
92.239	12.359	3.763	1.265	2.214	0.926	0.619
138.881	19.042	7.081	3.161	4.649	0.924	0.597
82.439	16.832	7.532	5.364	6.339	1.037	0.577
93.353	17.381	7.068	5.028	5.584	1.016	0.596
138.881	20.205	7.313	3.215	5.095	0.968	0.598
69.487	11.648	4.206	1.907	2.882	0.881	0.589
114.096	14.921	4.552	1.673	2.717	0.971	0.623
104.042	13.913	4.324	1.415	2.616	0.945	0.606
69.487	11.774	4.659	2.356	3.227	0.903	0.583
57.245	12.847	4.744	3.707	3.809	0.994	0.603
153.58	20.988	7.913	3.399	5.357	0.971	0.597
100.096	18.84	7.595	3.478	5.724	0.856	0.581
84.239	15.288	6.119	4.445	4.454	1.054	0.588
120.211	19.826	8.257	4.031	6.297	0.889	0.577
67.487	11.447	4.449	2.555	2.995	0.743	0.589
43.02	9.155	3.644	2.031	2.78	0.887	0.578
100.042	15.967	6.301	2.843	4.374	0.91	0.586
72.439	13.141	6.064	2.712	3.984	1.011	0.553
236.215	33.451	13.276	7.12	10.093	0.875	0.6
118.211	17.465	6.339	2.747	4.258	0.889	0.604
110.039	16.932	6.914	3.332	4.877	1.011	0.585
92.239	12.695	4.763	1.912	2.879	0.915	0.588
128.606	19.186	6.728	3	4.61	0.934	0.607
82.439	13.005	5.175	2.525	3.365	0.879	0.585
104.042	14.115	4.224	1.377	2.592	0.928	0.618
102.039	16.53	7.058	3.181	4.861	0.941	0.57
206.905	30.231	10.97	5.092	8.089	1.157	0.608
58	13.015	4.854	2.091	3.949	1.06	0.58
44.106	9.903	4.749	3.362	3.618	0.975	0.545

63.059	12.228	5.441	2.364	3.696	1.159	0.56
59.487	11.648	4.206	1.696	2.882	0.889	0.585
78.042	15.604	6.865	2.96	4.657	0.962	0.561
60.711	11.785	4.43	1.783	2.748	0.854	0.577
73.059	13.242	6.113	3.647	4.497	0.99	0.568
152	23.745	10.249	5.245	7.605	1.166	0.586
118.211	17.009	7.06	3.335	4.618	0.903	0.583
106.096	17.736	7.039	3.952	4.994	1.036	0.591
65.487	12.305	5.004	2.574	3.622	0.982	0.587
74.711	15.797	8.02	7.034	6.668	1.009	0.563
147.207	24.902	11.311	7.094	9.388	1.063	0.582
130.606	18.838	7.852	3.629	5.283	0.859	0.582
122.211	17.823	5.467	2.113	3.748	0.984	0.623
104.042	15.878	5.184	2.131	3.579	1.124	0.62
61.487	13.843	7.173	4.651	5.841	1.064	0.553
61.487	13.843	7.173	4.651	5.841	1.045	0.553
112.096	19.326	10.78	6.79	8.334	1.025	0.547
76.711	14.003	6.664	4.03	4.912	1.003	0.564
34.106	10.264	3.927	2.101	3.1	1.12	0.589
76.711	14.003	6.664	4.03	4.912	1.003	0.564
65.487	11.989	4.798	2.443	3.383	0.945	0.579
124.382	18.665	9.001	4.423	6.222	1.027	0.559
110.039	15.768	6.286	2.685	4.13	0.965	0.583
110.039	15.768	6.286	2.685	4.13	0.965	0.583
86.239	18.037	9.839	8.113	8.451	1.105	0.551
160.465	25.263	12.198	7.057	9.338	1.029	0.566
53.848	10.49	3.524	2.505	2.465	0.876	0.605
57.487	14.489	4.599	3.005	3.919	1.329	0.627
143.207	19.75	8.148	3.814	5.364	0.967	0.591
84.439	13.191	5.673	3.042	3.741	0.925	0.574
191.421	27.394	10.246	4.4	7.386	1.116	0.599
76.439	15.212	7.007	3.691	5.33	1.106	0.566
33.219	6.932	2.602	1.417	1.804	0.819	0.573
88.107	14.34	5.427	2.518	3.538	0.951	0.589
160.465	23.896	9.549	4.578	6.915	1.051	0.588
71.059	15.124	6.296	6	5.29	1.277	0.586
120.382	20.347	9.699	4.912	7.309	1.105	0.56
63.487	11.951	4.391	2.781	3.087	1.045	0.598
88.107	14.631	5.831	3.159	3.878	1.003	0.586
105.372	19.409	8.624	3.964	5.772	1.003	0.573
69.059	12.925	4.629	2.679	3.324	1.057	0.606
112.096	17.472	6.873	3.532	4.803	1.033	0.592
102.042	18.046	7.514	4.37	5.895	1.143	0.586
82.439	15.199	6.06	3.906	4.605	1.173	0.588
71.059	12.811	5.34	2.803	3.801	1.008	0.585
132.606	22.496	10.67	6.959	8.573	1.104	0.568
98.107	16.525	8.563	4.847	6.432	0.984	0.547
88.239	13.959	5.413	2.492	3.598	0.893	0.59
78.711	14.219	5.43	3.641	4.064	1.086	0.597
96.107	15.154	5.464	2.467	3.764	1.059	0.601

130.606	19.335	8.171	3.81	5.642	0.915	0.582
53.245	13.131	4.931	4.641	4.047	0.982	0.599
90.239	14	4.443	1.777	2.962	1.109	0.624
158	22.641	6.974	2.731	4.935	0.855	0.627
48.106	8.257	2.568	0.971	1.631	0.797	0.608
58	13.429	5.129	2.245	4.305	1.137	0.58
69.487	11.393	4.887	2.44	3.275	1.12	0.577
83.484	16.87	7.673	5.658	6.164	1.077	0.579
110.096	19.25	10.036	6.743	7.727	0.941	0.564
80.239	16.117	7.14	4.899	5.48	1.174	0.579
69.059	14.839	6.642	4.902	5.476	1.218	0.569
78.711	14.512	6.038	3.166	4.612	1.062	0.576
118.211	17.588	7.824	3.979	5.293	0.968	0.579
104.042	14.583	5.571	2.396	3.532	0.913	0.592
84.239	15.505	6.714	3.416	4.957	0.851	0.569
128.606	20.803	9.521	4.933	7.074	0.962	0.578
27.219	9.636	5.423	4.168	5.226	1.316	0.517
154	23.621	11.012	5.816	8.129	0.959	0.569
202.877	29.275	11.036	5.089	8.077	1.142	0.602
164.465	26.988	12.84	6.633	10.501	1.004	0.567
332.475	38.834	13.701	5.032	9.335	0.939	0.599
120.211	16.435	5.883	2.134	3.719	0.997	0.586
96.042	15.354	6.29	2.999	4.199	0.884	0.577
104.042	15.055	6.099	2.642	3.992	0.872	0.575
80.711	13.775	5.549	2.695	4.023	0.865	0.576
130.606	18.347	7.619	3.665	4.993	1.008	0.59
93.287	15.604	6.074	3.094	4.121	0.924	0.594
82.439	14.664	5.714	3.068	4.189	0.921	0.592

PW3	PW4	PW5	PJI2	CSI	ECC	AECC
0.316	0.179	0.098	0.8	222	117	6.882
0.293	0.147	0.084	0.875	522	270	11.25
0.34	0.189	0.11	1	500	238	9.154
0.273	0.137	0.081	0.75	115	60	5.455
0.326	0.183	0.099	0.8	239	116	7.25
0.35	0.132	0.045	1	127	73	5.214
0.356	0.201	0.106	0.875	739	354	11.8
0.349	0.19	0.079	0.333	79	37	3.7
0.353	0.207	0.118	0.8	346	156	7.091
0.287	0.148	0.088	0.833	305	157	8.722
0.374	0.184	0.096	0.875	958	470	11.75
0.346	0.188	0.099	1	792	381	12.29
0.321	0.184	0.106	0.8	331	155	7.381
0.339	0.199	0.12	0.833	485	243	8.679
0.363	0.19	0.103	0.857	537	255	10.2
0.364	0.197	0.103	1	481	228	9.5
0.355	0.197	0.096	0.75	161	78	5.571
0.328	0.163	0.1	1	879	418	11.297
0.269	0.129	0.08	0.857	374	193	10.158
0.354	0.193	0.092	0.833	393	182	8.667
0.364	0.217	0.137	0.8	322	154	7
0.355	0.195	0.094	1	273	127	6.684
0.342	0.165	0.092	1	409	193	9.19
0.348	0.198	0.106	1	464	219	9.522
0.302	0.164	0.087	1	538	256	9.481
0.424	0.222	0.115	1	530	248	8.857
0.267	0.122	0.072	1	556	270	12.273
0.351	0.185	0.094	0.889	1203	577	13.419
0.325	0.184	0.112	0.833	391	185	8.409
0.266	0.121	0.073	0.889	583	300	13.043
0.332	0.171	0.097	1	936	445	11.41
0.384	0.224	0.133	1	1223	545	12.674
0.314	0.158	0.093	1	306	151	7.947
0.334	0.18	0.097	0.833	367	180	8.571
0.393	0.21	0.132	1	624	276	8.118
0.34	0.186	0.093	0.889	829	376	13.429
0.379	0.206	0.122	0.833	441	193	8.042
0.399	0.203	0.097	0.5	137	67	4.786
0.336	0.17	0.083	0.875	724	350	11.667
0.391	0.21	0.115	0.833	497	222	8.538
0.348	0.208	0.116	1	835	379	11.485
0.35	0.211	0.122	0.75	223	103	5.722
0.381	0.187	0.112	1	1183	554	13.512
0.352	0.18	0.102	1	309	145	7.632
0.335	0.19	0.103	0.889	899	410	13.667
0.364	0.196	0.103	1	484	229	9.542
0.364	0.189	0.103	0.857	540	256	10.24
0.359	0.19	0.096	1	817	380	12.258
0.343	0.193	0.096	1	756	349	12.034

0.354	0.199	0.104	0.875	801	375	11.719
0.343	0.189	0.102	1	685	331	11.033
0.365	0.192	0.098	1	1451	677	15.386
0.339	0.192	0.109	0.875	717	347	11.567
0.349	0.197	0.104	1	615	296	10.571
0.356	0.193	0.11	0.875	644	308	11.407
0.347	0.19	0.099	1	1043	493	13.694
0.35	0.172	0.094	0.889	1035	491	13.27
0.355	0.201	0.108	1	500	238	9.52
0.358	0.195	0.106	1	1048	495	13.75
0.34	0.194	0.106	1	654	316	10.897
0.327	0.151	0.091	1	503	242	9.308
0.364	0.196	0.103	1	484	229	9.542
0.342	0.195	0.102	0.875	691	318	11.357
0.331	0.185	0.1	1	579	277	10.654
0.364	0.196	0.103	1	484	229	9.542
0.32	0.183	0.091	1	500	233	9.708
0.308	0.165	0.097	0.875	663	315	11.667
0.341	0.168	0.088	0.75	194	96	5.647
0.301	0.159	0.099	0.8	279	137	7.211
0.32	0.181	0.112	0.8	332	156	7.429
0.304	0.145	0.078	0.833	274	141	8.294
0.32	0.181	0.112	0.8	332	156	7.429
0.362	0.194	0.102	0.8	336	160	7.273
0.359	0.185	0.102	1	295	137	7.611
0.31	0.155	0.061	0.833	301	153	9
0.33	0.18	0.096	1	646	305	12.2
0.329	0.172	0.094	1	630	296	11.385
0.368	0.205	0.113	1	256	120	6.316
0.343	0.191	0.109	0.833	440	201	8.375
0.361	0.191	0.101	0.857	554	275	10.185
0.322	0.115	0.069	1	170	89	6.357
0.345	0.191	0.119	1	476	228	9.12
0.333	0.174	0.078	0.75	167	80	5.714
0.34	0.189	0.117	1	397	180	7.826
0.326	0.186	0.102	0.75	149	78	5.571
0.339	0.154	0.088	1	262	127	7.471
0.33	0.195	0.114	0.8	314	146	7.3
0.424	0.222	0.115	1	530	248	8.857
0.349	0.19	0.113	1	279	131	6.55
0.32	0.174	0.097	0.8	281	138	7.263
0.376	0.216	0.108	0.857	666	311	10.367
0.36	0.198	0.099	0.8	260	120	7.059
0.343	0.176	0.091	0.8	262	129	7.167
0.391	0.208	0.112	0.857	581	259	9.593
0.377	0.198	0.103	1	2583	1155	21.389
0.324	0.18	0.114	0.857	584	282	9.724
0.297	0.164	0.092	0.8	285	139	7.316
0.349	0.19	0.087	0.8	371	185	7.4
0.362	0.197	0.112	1	1759	827	14.259

0.269	0.135	0.077	0.9	885	435	15
0.353	0.203	0.111	0.889	894	398	13.267
0.406	0.238	0.133	0.833	578	274	8.563
0.361	0.18	0.082	1	346	180	8.182
0.316	0.13	0.041	0.8	186	104	7.429
0.364	0.218	0.121	0.8	310	148	7.048
0.377	0.212	0.12	1	1152	517	12.31
0.257	0.138	0.059	0.857	406	209	10.45
0.308	0.152	0.087	1	360	175	9.211
0.321	0.158	0.084	1	291	142	7.889
0.329	0.18	0.1	1	583	274	10.96
0.316	0.176	0.106	0.833	391	184	8.364
0.355	0.192	0.103	0.857	569	265	9.815
0.278	0.132	0.084	0.857	584	290	10.357
0.333	0.186	0.083	0.8	341	160	7.273
0.363	0.205	0.112	0.8	275	127	7.056
0.358	0.199	0.114	0.833	377	179	8.136
0.322	0.135	0.071	0.667	67	35	3.889
0.331	0.177	0.103	0.875	782	362	12.067
0.341	0.168	0.087	1	283	138	7.667
0.286	0	0	0.5	29	19	2.714
0.343	0.157	0.086	0.909	1538	721	17.585
0.326	0.166	0.079	1	341	168	8
0.362	0.199	0.117	0.875	866	414	11.189
0.314	0.174	0.082	1	108	57	4.75
0.37	0.218	0.137	0.6	299	136	6.476
0.346	0.179	0.087	0.8	271	133	7.389
0.328	0.121	0.063	1	290	149	8.278
0.368	0.187	0.097	1	703	339	10.935
0.342	0.173	0.084	0.909	1145	548	16.118
0.329	0.171	0.089	0.9	1033	493	14.939
0.363	0.204	0.11	0.833	452	203	8.826
0.304	0.145	0.084	0.857	582	289	10.321
0.341	0.168	0.088	0.75	194	96	5.647
0.398	0.238	0.149	1	289	126	6
0.305	0.14	0.071	0.8	203	106	7.067
0.33	0.172	0.1	1	1361	619	16.73
0.345	0.191	0.119	1	476	228	9.12
0.358	0.182	0.093	0.833	391	183	8.714
0.351	0.202	0.107	0.875	849	383	11.969
0.304	0.181	0.099	0.8	222	110	6.875
0.329	0.185	0.092	0.75	146	71	5.462
0.352	0.209	0.114	0.8	414	200	7.407
0.338	0.156	0.081	1	402	197	9.381
0.34	0.164	0.085	1	304	149	7.842
0.34	0.179	0.09	0.8	212	103	6.867
0.319	0.162	0.092	0.857	544	257	10.28
0.342	0.185	0.096	1	413	195	8.864
0.352	0.187	0.069	0.667	98	52	4.333
0.366	0.204	0.125	1	501	232	8.923

0.335	0.165	0.079	0.8	240	124	7.294
0.325	0.176	0.097	1	541	256	10.667
0.367	0.209	0.118	0.833	439	210	8.4
0.349	0.187	0.115	1	290	137	6.524
0.345	0.201	0.107	0.875	767	355	11.833
0.37	0.2	0.102	0.833	417	196	8.522
0.315	0.156	0.089	0.833	317	165	8.684
0.345	0.192	0.111	1	227	112	6.222
0.351	0.21	0.128	0.6	280	125	6.25
0.347	0.178	0.086	0.8	268	130	7.222
0.349	0.19	0.079	0.333	79	37	3.7
0.266	0.093	0.043	0.667	63	38	4.222
0.354	0.194	0.116	1	294	147	6.391
0.376	0.218	0.112	0.833	486	234	9
0.327	0.164	0.093	0.9	1009	485	14.697
0.405	0.219	0.118	1	513	239	8.852
0.346	0.164	0.082	0.833	341	176	8.8
0.276	0.13	0.084	1	393	203	10.684
0.338	0.188	0.094	1	109	58	4.833
0.36	0.208	0.117	0.8	361	164	7.13
0.318	0.168	0.091	0.889	1234	572	13.951
0.398	0.238	0.149	1	289	126	6
0.358	0.211	0.12	1	590	265	9.138
0.293	0.152	0.092	0.833	322	168	8.4
0.306	0.146	0.094	1	427	210	9.545
0.365	0.217	0.12	1	605	282	9.724
0.359	0.197	0.117	1	204	101	5.941
0.381	0.222	0.136	1	432	184	7.36
0.396	0.245	0.149	0.8	363	159	6.913
0.345	0.163	0.092	1	260	128	7.529
0.311	0.123	0.072	1	200	105	6.563
0.372	0.213	0.119	1	758	341	11
0.364	0.208	0.121	1	326	165	6.6
0.292	0.151	0.094	0.8	317	157	7.476
0.352	0.199	0.114	1	403	203	7.808
0.329	0.163	0.075	1	280	136	8
0.345	0.143	0.077	0.75	124	65	5.417
0.36	0.201	0.116	0.833	405	192	8.348
0.33	0.195	0.114	0.8	314	146	7.3
0.35	0.185	0.108	0.909	1491	715	16.25
0.366	0.204	0.125	1	501	232	8.923
0.363	0.189	0.097	0.857	513	243	10.125
0.366	0.212	0.11	1	366	163	7.762
0.368	0.212	0.109	0.857	620	287	10.25
0.342	0.185	0.117	1	277	130	6.5
0.41	0.243	0.147	0.8	341	151	6.565
0.343	0.198	0.121	0.833	415	198	8.25
0.357	0.199	0.124	0.875	1056	484	11.805
0.385	0.213	0.084	1	182	96	6
0.278	0.153	0.083	0.8	171	89	6.846

0.346	0.208	0.116	1	240	111	6.167
0.381	0.217	0.098	1	210	103	6.059
0.343	0.206	0.123	0.667	380	180	7.826
0.358	0.22	0.121	1	250	117	6.158
0.306	0.158	0.085	1	308	148	8.222
0.349	0.175	0.098	1	865	404	12.625
0.347	0.2	0.118	0.833	511	233	8.962
0.326	0.173	0.105	1	412	197	7.88
0.346	0.169	0.076	0.8	247	120	7.059
0.269	0.129	0.08	0.857	374	193	10.158
0.321	0.167	0.087	0.875	694	347	11.567
0.362	0.196	0.101	0.875	705	325	11.607
0.405	0.215	0.114	1	496	230	8.846
0.385	0.209	0.111	0.833	416	190	8.261
0.321	0.141	0.066	0.833	286	147	8.647
0.321	0.141	0.066	0.833	286	147	8.647
0.304	0.157	0.084	1	612	300	12
0.304	0.159	0.093	0.833	345	167	8.789
0.346	0.173	0.074	1	119	63	4.846
0.304	0.159	0.093	0.833	345	167	8.789
0.342	0.193	0.108	0.8	241	118	6.941
0.326	0.183	0.107	0.875	691	315	11.667
0.368	0.205	0.118	1	483	220	9.167
0.368	0.205	0.118	1	483	220	9.167
0.276	0.149	0.072	1	458	235	11.19
0.319	0.188	0.108	0.875	818	393	11.909
0.287	0.161	0.086	1	195	95	6.333
0.348	0.144	0.061	0.75	194	102	6
0.356	0.197	0.107	0.889	887	395	13.167
0.314	0.17	0.088	0.833	368	172	8.6
0.367	0.214	0.126	0.875	972	441	11.605
0.341	0.188	0.098	1	323	158	7.9
0.327	0.16	0.062	0.667	76	40	4
0.354	0.199	0.099	0.833	435	193	8.773
0.355	0.196	0.111	0.875	803	377	11.424
0.266	0.131	0.078	1	274	143	7.944
0.34	0.197	0.105	0.857	582	276	10.222
0.322	0.156	0.079	1	267	129	7.588
0.334	0.174	0.093	1	433	202	9.182
0.348	0.198	0.11	0.857	652	300	10.345
0.344	0.155	0.08	1	284	138	7.667
0.334	0.181	0.112	1	500	239	9.56
0.343	0.169	0.082	0.833	418	206	8.957
0.31	0.147	0.085	0.833	344	171	8.55
0.346	0.161	0.069	1	292	142	7.889
0.319	0.173	0.095	1	729	358	12.786
0.311	0.16	0.085	0.875	527	256	11.636
0.356	0.191	0.114	1	292	138	6.571
0.305	0.155	0.088	1	315	154	8.105
0.371	0.2	0.1	1	377	176	8

0.362	0.196	0.101	0.875	705	325	11.607
0.278	0.133	0.08	1	204	106	6.625
0.393	0.203	0.11	1	349	157	7.476
0.403	0.228	0.127	0.833	582	276	8.625
0.396	0.198	0.089	0.25	122	59	4.538
0.385	0.213	0.084	1	182	96	6
0.338	0.18	0.09	0.8	271	123	7.235
0.292	0.148	0.084	1	409	200	9.524
0.311	0.145	0.076	0.889	695	337	13.48
0.311	0.156	0.081	1	405	196	9.333
0.3	0.142	0.074	1	318	163	9.056
0.336	0.183	0.108	0.8	271	134	7.053
0.34	0.185	0.099	0.875	690	312	12
0.36	0.208	0.117	0.8	361	164	7.13
0.353	0.195	0.103	0.833	367	181	8.619
0.355	0.181	0.093	1	760	360	12.857
0.293	0.151	0.069	1	79	47	4.7
0.335	0.191	0.098	0.875	793	381	11.906
0.352	0.2	0.125	0.875	1031	471	11.775
0.363	0.186	0.088	1	940	464	14.061
0.392	0.24	0.149	1	1583	712	12.491
0.378	0.233	0.143	0.8	421	188	7.231
0.336	0.201	0.12	1	379	179	7.783
0.363	0.205	0.097	0.833	417	199	8.652
0.352	0.19	0.106	1	297	147	7.737
0.349	0.195	0.106	1	794	350	12.5
0.337	0.168	0.108	1	383	183	7.957
0.336	0.18	0.098	0.833	341	168	8.4

DECC	MDDD	UNIP	CENT	VAR	BAC	Lop
1.08	12.374	43	329	45	51	1.816
2.021	25.389	92	928	98	73	2.363
1.278	20.876	82	1052	94	42	1.134
1.041	5.14	22	90	18	12	1.455
1.156	10.656	40	266	51	14	1.703
0.786	6.694	25	212	36	78	1.611
1.72	30.551	121	1568	142	39	1.187
0.42	2.4	16	50	10	2	0.664
1.017	13.727	59	554	60	5	0.517
1.309	13.556	57	396	52	29	2.345
1.75	44.95	171	3574	204	183	1.472
2.087	36.01	132	1702	147	56	1.774
1.029	14.571	56	588	67	9	1.369
1.273	23.857	84	1320	122	73	1.806
1.504	20.33	90	852	96	66	0.898
1.417	18.896	82	762	80	51	0.901
0.776	6.367	28	176	26	10	0.748
1.765	38.299	159	2667	163	86	0.864
1.762	17.878	69	469	59	38	2.308
1.238	13.873	66	532	57	11	0.819
1	14.727	58	556	66	42	1.231
0.925	10.238	51	383	39	11	0.868
1.551	17.156	69	501	67	19	1.083
1.456	18.665	78	700	79	38	0.899
1.278	23.764	93	1279	101	9	1.159
1.378	19.704	86	932	94	84	1.041
2.364	26.066	96	742	85	16	1.811
1.944	55.049	211	4209	220	169	1.371
1.355	17.289	67	618	70	14	1.405
2.48	29.062	107	839	96	50	2.514
1.682	44.126	161	3429	186	61	1.136
1.744	41.366	197	3429	190	106	0.948
1.324	13.684	56	418	47	33	1.743
1.306	15.107	64	572	72	20	1.292
1.19	28.159	107	1610	117	55	1.009
2.041	28.755	137	1192	118	5	0.444
1.302	17.521	69	634	78	5	0.49
0.561	5.714	28	124	19	26	0.802
2.267	33.564	127	1544	124	70	1.49
1.385	18.308	83	800	77	18	0.777
1.776	32.836	147	1801	122	15	0.865
0.753	8.765	41	304	32	6	0.816
2.403	52.266	184	3112	240	178	1.391
1.44	13.241	54	352	45	19	1.144
2.067	31.2	153	1534	128	6	0.588
1.458	18.771	83	774	80	38	0.882
1.549	20.272	91	865	96	51	0.886
2.154	34.968	135	1643	133	38	0.778
2.245	33.042	121	1427	124	27	0.772

1.879	33.457	137	1674	126	55	1.042
1.571	29.556	121	1490	116	77	1.495
2.222	58.917	235	4426	242	105	0.813
1.862	31.436	123	1478	122	79	1.428
1.857	29.066	104	1230	106	61	1.36
1.682	25.342	104	1100	112	55	1.135
2.395	42.389	173	2390	184	91	0.942
2.115	43.88	176	2634	181	89	1.287
1.379	19.779	88	862	78	42	1.157
2.444	43.63	175	2472	170	87	1.019
1.914	29.655	116	1334	114	64	1.435
1.408	23.556	86	1072	93	54	0.975
1.458	18.771	83	774	80	38	0.882
2.026	29.908	111	1252	108	18	0.744
1.911	27.053	99	1006	92	43	1.315
1.458	18.771	83	774	80	38	0.882
1.365	19.861	85	872	79	2	0.382
1.877	30.118	105	1301	132	15	1.409
0.886	8.699	40	240	26	38	1.011
1.086	13.285	49	505	55	13	1.315
1.075	15.302	57	595	67	14	1.447
1.287	11.799	46	350	56	32	2.202
1.075	15.302	57	595	67	14	1.447
1.116	13.504	61	562	51	38	0.917
1.167	10.926	47	330	50	10	0.662
1.412	12.464	59	325	48	21	2.728
2.032	25.389	108	882	100	15	1.03
1.663	21.586	114	952	78	18	0.777
0.997	11.169	48	346	38	21	1.039
1.375	18.5	73	716	74	18	0.813
1.405	21.684	94	1148	117	92	1.233
1.071	6.857	33	168	30	29	1.618
1.344	20.784	78	898	92	42	1.157
0.796	6.429	30	182	24	5	0.673
1.081	15.577	65	669	75	5	0.503
0.857	7.714	29	196	30	19	1.822
1.204	9.924	42	318	45	17	0.732
1	13.19	51	498	70	6	1.297
1.378	19.704	86	932	94	84	1.041
0.995	11.4	51	432	50	10	0.627
1.014	11.983	52	424	46	19	1.144
1.458	23.933	115	1354	96	83	0.848
1.017	9.599	42	290	47	6	0.844
1.093	10.667	47	348	46	27	0.977
1.578	21.77	87	921	94	26	0.627
3.352	94.173	399	7218	384	122	0.574
1.389	26.818	99	1283	122	51	1.41
1.069	13.762	50	522	57	9	1.46
1.056	18.029	69	1019	70	49	1.604
1.939	75.969	293	8180	342	300	1.157

2.483	37.103	159	1537	122	58	1.195
2.031	29.787	144	1386	119	10	0.496
1.277	23.375	106	1280	86	149	0.937
1.198	16	70	616	60	85	1.991
1.224	9.469	41	192	28	44	2.236
1.011	13.374	55	495	55	27	1.275
1.768	44.277	181	3326	214	54	0.768
1.75	17.96	79	512	60	50	2.482
1.695	15.945	64	400	55	14	1.538
1.247	12	52	364	46	12	1.126
1.811	24.256	97	935	99	7	0.849
1.273	17.628	66	662	70	12	1
1.674	23.602	93	941	93	53	1.209
1.643	31.102	104	1552	116	62	1.488
1.116	15.372	58	724	66	10	0.595
0.963	10.222	44	342	50	10	0.662
1.252	15.62	63	526	73	29	1.297
0.593	2.889	15	33	9	10	0.881
1.671	30.24	118	1696	168	22	0.975
1.481	12.444	52	316	42	19	1.177
0.408	2	8	28	6	29	0.863
2.554	58.706	270	3878	249	97	2.571
1.238	16.027	63	555	59	41	1.049
1.718	39.246	142	2354	170	161	1.611
0.792	5.5	21	134	20	15	1.528
0.834	11.66	52	440	52	11	0.819
1.167	12.383	50	364	48	24	1.761
1.247	12.074	59	348	40	43	2.15
1.752	27.954	123	1587	126	86	0.933
2.706	44.765	195	2150	180	66	0.79
2.615	41.767	182	1990	159	38	0.753
1.255	15.293	76	642	58	10	0.58
1.63	28.367	102	1528	126	59	1.802
0.886	8.699	40	240	26	38	1.011
0.667	9.819	48	416	44	10	0.61
1.404	10.524	41	193	32	34	1.564
2.71	52.079	209	2915	228	10	0.435
1.344	20.784	78	898	92	42	1.157
1.469	16.372	67	521	60	19	1.083
1.729	32.438	143	1674	129	8	0.875
1.375	11.406	41	242	35	25	1.5
0.959	6.331	27	127	23	12	1.342
1.015	18.963	75	1161	87	59	1.413
1.696	17.347	74	510	59	30	1.539
1.235	12.327	54	422	49	19	1.144
0.96	7.973	35	213	37	11	0.985
1.674	21.738	94	912	80	29	1.207
1.24	17.322	63	730	80	9	0.832
0.667	5.333	22	116	18	22	1.557
1.473	20.166	82	836	94	38	0.85

1.1	10.623	47	313	41	34	2.085
1.806	23.292	88	822	89	20	1.195
1.456	18.918	78	774	72	54	0.991
0.925	12.19	53	525	50	17	0.665
1.744	29.311	131	1400	126	16	1.083
1.195	15.429	67	689	78	37	0.702
1.629	16.731	63	411	50	48	1.913
0.938	11.235	40	416	50	13	1.357
0.8	10.5	51	354	42	2	0.432
1	10.222	49	336	38	17	0.714
0.42	2.4	16	50	10	2	0.664
0.691	3.852	15	57	11	33	1.436
0.93	15.27	55	823	62	35	1.485
1.308	19.953	91	894	70	74	1.063
2.391	40.567	172	1988	152	74	1.658
1.361	19.007	83	865	86	67	1.048
1.34	15.82	64	524	62	51	2.228
1.911	18.598	70	482	71	32	2.393
0.722	6.028	22	122	24	22	1.557
1.017	14.348	63	621	59	10	0.58
2.157	52.063	208	4174	227	42	0.88
0.667	9.819	48	416	44	10	0.61
1.532	23.829	97	1199	94	30	0.871
1.48	17.1	61	510	62	65	1.921
1.455	17.306	76	664	68	43	1.93
1.358	22.813	107	1243	95	55	1.096
1.01	9.453	41	243	34	30	1.123
1.283	16.304	67	703	75	10	0.553
1.055	13.168	57	551	74	12	0.973
1.502	11.592	47	261	45	22	1.335
1.117	9.641	41	258	34	43	1.656
1.548	28.776	120	1530	129	27	0.744
0.88	16.592	65	935	70	78	1.37
1.12	15.66	59	633	61	31	1.234
1.006	19.408	73	1084	90	79	1.529
1.176	10.983	51	293	39	18	0.972
0.917	5.833	24	108	22	19	1.425
1.406	17.437	70	622	68	30	0.982
1	13.19	51	498	70	6	1.297
2.875	62.562	255	4122	264	184	1.235
1.473	20.166	82	836	94	38	0.85
1.552	18.938	86	774	88	30	0.961
1.32	14.49	60	492	56	5	0.532
1.429	22.531	105	1112	97	51	0.843
0.95	11.44	50	454	50	10	0.627
0.938	11.864	55	549	64	18	0.832
1.208	19.569	70	838	87	20	1.195
1.877	45.941	179	2987	167	73	1.016
0.75	8.5	32	284	42	43	1.656
1.396	8.71	33	131	30	18	1.604

0.87	10	39	396	42	0	0
0.789	8.699	35	321	38	18	0.972
1.153	17.66	65	723	81	13	1.17
0.831	10.571	41	455	46	5	0.565
1.333	12.111	56	366	52	16	2.052
1.711	34.344	130	2032	188	34	1.075
1.204	19.308	79	1026	106	10	0.54
1.034	18.464	72	922	81	30	0.942
1.356	11.308	47	259	35	19	1.212
1.762	17.878	69	469	59	38	2.308
1.738	31.778	118	1660	138	101	2.217
1.893	28.383	114	1198	104	21	0.845
1.408	18.296	80	770	84	52	1.052
1.338	15.497	67	563	68	27	0.872
1.433	12.394	53	335	45	26	2.297
1.433	12.394	53	335	45	26	2.297
2	26.816	99	1097	123	17	1.853
1.296	13.651	60	444	59	16	1.985
0.805	5.692	26	130	19	30	1.23
1.296	13.651	60	444	59	16	1.985
0.9	9.446	40	316	45	18	0.972
1.877	27.868	100	1212	150	7	1.233
1.278	16.667	76	748	82	14	0.865
1.278	16.667	76	748	82	14	0.865
1.819	20.041	88	606	67	45	2.681
1.625	32.973	143	2017	137	50	1.16
1.156	8.24	37	183	29	19	1.29
0.824	8.457	37	327	41	56	1.612
1.956	29.08	140	1386	128	10	0.496
1.46	16.32	62	502	59	8	1.197
1.837	40.019	157	2446	170	45	1.006
1.13	12.82	58	484	58	19	1.113
0.6	3.6	16	68	12	11	1.185
1.269	15.182	72	578	62	11	0.797
1.589	31.912	127	1981	152	58	1.118
1.395	13.284	53	388	50	47	1.928
1.621	26.23	94	1248	110	18	1.085
1.26	9.938	44	322	45	17	0.732
1.306	15.43	70	610	72	10	0.595
1.505	27.572	107	1359	119	10	0.506
1.259	10.802	48	364	50	26	0.745
1.462	21.974	84	934	93	42	1.157
1.274	17.773	76	754	76	46	1.086
1.405	16.08	61	498	59	38	1.713
1.469	13.222	54	334	42	19	1.177
1.903	28	132	1280	118	47	1.165
1.76	21.132	87	698	102	22	2.531
0.966	12.404	54	494	54	17	0.665
1.28	13.175	58	420	48	31	1.848
1.091	14	62	612	70	26	0.688

1.893	28.383	114	1198	104	21	0.845
1.172	9.563	42	264	32	41	1.781
1.261	12.209	56	428	57	17	0.665
1.234	23.25	108	1244	84	149	0.937
0.497	5.041	26	84	13	17	0.808
0.75	8.5	32	284	42	43	1.656
1.073	9.571	46	284	35	2	0.481
1.501	16.39	73	593	61	23	1.92
2.141	26.208	124	980	97	18	0.795
1.492	15.361	70	558	66	17	0.665
1.401	13.679	53	416	66	33	2.367
1.014	12.028	47	447	53	22	1.262
1.923	25.077	117	964	90	5	0.465
1.017	14.348	63	621	59	10	0.58
1.197	15.138	64	548	64	35	1.103
2.031	28.571	136	1196	98	27	0.787
0.76	4.96	17	92	18	30	1.846
1.686	32.125	141	1732	134	54	1.804
1.897	45.645	174	2818	162	58	1.005
1.888	35.662	163	2017	163	101	1.096
1.588	55.736	260	6552	276	167	1.126
0.959	17.154	74	772	85	16	1.186
1.115	15.259	64	712	63	19	1.029
1.319	16.216	72	682	72	27	1.216
1.147	13.147	52	414	55	31	1.296
1.929	26.5	126	1160	105	5	0.444
1.183	17.864	66	756	77	29	1.265
1.28	14.4	57	500	62	39	1.169

ICR	D/Dr03	D/Dr04	D/Dr05	D/Dr06	D/Dr07	D/Dr08
2.257	0	0	52.314	0	0	0
2.959	0	0	0	94.204	0	0
2.599	0	0	0	224.465	0	0
1.981	0	0	0	35.181	0	0
2.281	0	0	31.319	37.716	0	0
1.689	0	0	0	0	0	0
2.844	0	0	103.071	227.07	0	0
0.881	0	0	14.765	18.569	0	0
2.254	0	0	35.281	87.884	43.282	0
2.53	0	0	0	68.161	0	0
2.915	0	0	0	437.302	0	0
3.127	0	0	75.38	197.763	0	0
2.201	0	0	0	74.586	44.424	0
2.481	0	0	0	187.689	0	0
2.759	0	50.636	63.579	88.922	91.681	0
2.669	0	47.581	59.766	85.055	86.225	0
1.842	0	0	35.453	40.583	0	0
2.868	0	0	107.841	376.372	0	0
2.76	0	0	0	72.59	0	0
2.489	0	0	52.195	135.081	72.022	0
2.267	0	0	33.632	84.768	0	0
2.076	0	0	83.897	44.556	0	0
2.725	0	0	43.486	118.148	0	0
2.682	0	44.915	56.455	80.865	81.414	0
2.61	0	0	88.985	208.481	0	0
2.697	0	0	40.545	138.214	0	0
3.118	52.331	0	0	86.285	0	0
3.095	0	0	549.128	0	0	0
2.556	0	0	44.314	115.007	0	0
3.099	0	0	0	92.34	0	0
2.836	0	0	0	556.489	0	0
2.992	0	0	241.291	349.528	0	0
2.484	0	0	39.23	52.117	0	0
2.506	0	0	0	143.833	0	0
2.412	38.072	0	35.751	283.029	0	63.536
3.115	0	0	89.767	311.379	0	0
2.519	0	39.503	0	150.313	0	49.286
1.432	0	0	24.769	29.659	0	0
2.906	0	0	68.486	184.198	0	0
2.537	0	0	25.952	153.71	0	0
2.815	0	0	155.023	297.103	0	0
1.772	0	0	0	58.887	32.762	0
3.26	0	0	0	347.097	0	0
2.55	0	0	26.781	75.738	0	0
3.061	0	0	54.436	262.096	0	0
2.685	0	44.808	0	153.26	0	95.429
2.759	0	47.722	0	161.592	0	101.495
3.127	0	62.757	93.787	206.289	0	127.999
3.125	0	58.664	176.09	86.829	0	119.26

2.957	0	65.718	195.301	98.125	0	134.699
2.867	0	62.021	94.64	95.907	0	130.613
3.338	0	96.743	143.743	485.929	0	199.343
2.978	0	62.102	93.982	94.062	0	128.679
2.937	0	55.723	87.569	84.221	0	115.52
2.904	0	53.234	0	177.712	0	111.529
3.253	0	76.722	0	377.906	0	155.965
3.129	0	79.471	120.041	255.66	0	163.512
2.661	0	48.202	74.67	76.222	0	103.311
3.229	0	76.694	114.74	245.174	0	155.269
2.945	0	59.517	90.682	90.021	0	123.23
2.643	0	0	76.74	168.629	0	0
2.685	0	44.808	0	153.26	0	95.429
2.985	0	54.663	84.762	175.468	0	111.534
2.936	0	51.628	79.308	77.88	0	106.595
2.685	0	44.808	0	153.26	0	95.429
2.615	0	0	74.226	87.579	101.039	0
2.922	0	0	0	298.905	0	0
1.954	0	0	0	76.228	0	0
2.268	0	0	0	133.279	0	0
2.226	0	0	0	119.362	0	0
2.514	0	0	0	62.954	0	0
2.226	0	0	0	119.362	0	0
2.258	0	0	42.569	118.891	0	0
2.441	0	0	32.971	89.776	0	0
2.514	0	0	56.919	0	0	0
3.099	0	0	77.883	179.463	0	0
2.855	0	0	0	237.512	0	0
2.168	0	0	34.22	66.801	0	0
2.542	32.87	0	0	182.617	0	0
2.712	0	0	87.659	101.973	0	0
2.217	0	0	0	48.832	0	0
2.649	0	0	53.099	139.867	0	0
1.877	0	0	35.31	43.995	0	0
2.403	0	0	0	136.985	45.178	0
1.924	0	0	0	48.594	0	0
2.44	0	0	0	113.298	0	0
2.183	0	0	0	69.393	40.562	0
2.697	0	0	40.545	138.214	0	0
2.148	0	0	0	90.369	48.391	0
2.215	0	0	0	122.315	0	0
2.68	0	56.735	156.168	93.957	103.523	0
2.257	0	0	68.651	38.571	0	0
2.261	0	0	0	114.828	0	0
2.756	0	0	88.203	132.211	0	0
3.825	0	0	262.845	950.719	0	0
2.666	0	0	0	154.616	80.726	0
2.249	0	0	0	134.519	0	0
2.217	0	0	0	189.616	0	0
3.139	0	99.822	0	603.569	0	351.028

3.194	0	0	0	239.954	0	0
3.039	0	0	138.451	261.292	0	0
2.505	0	0	0	221.13	0	0
2.422	0	0	0	84.385	0	0
2.236	0	0	0	0	0	0
2.267	0	0	26.923	81.861	0	0
3.028	0	0	134.71	440.016	0	0
2.728	0	0	68.008	0	0	0
2.786	0	0	0	133.3	0	0
2.489	0	0	0	120.183	0	0
2.859	0	0	0	279.484	0	0
2.504	0	0	60.542	143.319	0	0
2.769	0	0	40.806	138.816	0	0
2.753	0	0	0	211.37	0	0
2.258	0	0	134.102	77.064	0	0
2.224	0	0	32.28	90.913	0	0
2.495	0	0	34.467	42.054	44.776	0
1.53	0	0	0	26	0	0
2.911	0	0	0	290.111	0	0
2.53	0	0	0	119.754	0	0
0.863	0	0	0	0	0	0
3.234	0	0	81.598	292.432	0	0
2.453	0	0	64.2	74.256	0	0
2.912	0	0	59.701	211.373	0	0
1.855	0	0	0	39.371	0	0
1.904	0	0	20.81	60.139	33.487	0
2.261	0	0	37.441	45.063	0	0
2.377	0	0	0	68.543	0	0
2.905	0	0	0	322.123	0	0
3.385	0	0	122.36	280.209	0	0
3.253	0	0	0	404.899	0	0
2.496	0	0	45.224	172.14	0	77.305
2.764	0	0	0	217.857	0	0
1.954	0	0	0	76.228	0	0
1.936	0	0	18.099	98.815	0	32.557
2.257	0	0	0	52.419	0	0
3.506	0	0	0	678.363	0	0
2.649	0	0	53.099	139.867	0	0
2.57	0	0	43.199	120.852	0	0
2.889	0	0	269.166	214.361	0	0
2.25	0	0	65.953	0	0	54.539
1.988	0	0	48.974	0	0	40.515
2.193	0	0	51.463	151.345	0	0
2.743	0	0	0	149.433	0	0
2.484	0	0	0	128.564	0	0
2.2	0	0	38.692	46.741	0	0
2.744	0	0	54.66	160.462	0	0
2.561	0	0	65.321	150.846	0	0
1.459	0	0	34.395	0	0	0
2.716	0	0	0	210.135	0	0

2.218	0	0	0	62.915	0	0
2.892	0	0	71.571	163.964	0	0
2.565	0	0	0	192.499	0	0
2.089	0	0	0	96.773	50.631	0
2.906	0	0	169.392	199.892	0	0
2.475	0	0	59.609	114.953	0	0
2.524	0	0	0	70.863	0	0
2.155	0	0	0	120.061	0	0
1.883	0	0	0	83.512	28.129	0
2.147	0	0	0	119.975	0	0
0.881	0	0	14.765	18.569	0	0
1.53	0	0	0	0	0	0
2.119	0	0	0	169.079	0	0
2.484	0	49.24	62.691	89.987	89.759	0
3.248	0	0	0	334.355	0	0
2.68	0	0	39.224	130.963	0	0
2.528	0	0	0	75.918	0	0
2.931	0	0	0	72.772	0	0
1.825	0	0	33.676	0	0	0
2.234	0	0	37.314	93.549	45.469	0
3.049	86.894	0	0	567.965	0	0
1.936	0	0	18.099	98.815	0	32.557
2.738	39.096	0	52.535	209.616	0	0
2.504	0	0	0	74.037	0	0
2.681	0	0	0	115.754	0	0
2.625	0	50.899	65.588	148.438	93.211	0
2.207	0	0	0	75.472	0	0
2.469	27.707	0	24.513	129.801	0	42.08
2.246	0	0	21.803	115.282	0	37.413
2.542	0	0	0	80.158	0	0
2.18	0	0	0	56.975	0	0
2.87	0	0	53.186	266.774	0	0
2.04	0	0	0	159.737	0	0
2.226	0	0	0	140.559	0	0
2.333	0	0	0	170.429	0	0
2.469	0	0	99.977	0	0	0
1.959	0	0	0	38.81	0	0
2.562	0	0	0	175.52	0	0
2.183	0	0	0	69.393	40.562	0
3.411	0	0	0	582.44	0	0
2.716	0	0	0	210.135	0	0
2.757	0	0	51.725	148.794	0	0
2.453	0	0	76.095	81.344	0	0
2.708	0	53.187	147.109	86.223	96.976	0
2.121	0	0	0	90.783	48.206	0
2.104	0	0	20.286	114.11	0	36.675
2.492	0	0	67.297	151.194	0	0
2.937	0	0	71.741	442.026	0	0
2.022	0	0	0	55.344	0	0
2.258	0	0	0	43.8	0	0

2.108	0	0	0	163.598	0	0
2.064	0	0	0	105.978	0	0
2.298	0	0	63.213	143.506	0	0
2.076	0	0	48.54	117.901	0	0
2.441	0	0	38.287	49.304	0	0
3.007	0	0	0	315.078	0	0
2.473	0	0	0	166.982	58.625	0
2.375	0	0	63.962	165.543	0	0
2.278	0	0	48.657	55.758	0	0
2.76	0	0	0	72.59	0	0
2.915	0	0	0	168.32	0	0
2.968	0	0	84.542	238.997	0	0
2.705	0	0	37.415	125.669	0	0
2.525	0	0	32.366	106.385	0	0
2.571	0	0	0	63.994	0	0
2.571	0	0	0	63.994	0	0
3.083	0	0	0	186.447	0	0
2.524	0	0	0	96.043	0	0
1.854	0	0	0	42.762	0	0
2.524	0	0	0	96.043	0	0
2.19	0	0	0	105.02	0	0
2.893	0	0	0	184.749	63.78	0
2.642	0	0	0	293.219	0	0
2.642	0	0	0	293.219	0	0
2.915	0	0	72.51	0	0	0
2.912	0	0	0	357.937	0	0
2.206	0	0	30.82	34.919	0	0
1.851	0	0	0	63.41	0	0
3.043	0	0	66.898	337.745	0	0
2.504	0	0	96.184	48.83	0	0
2.909	0	0	142.675	312.187	0	0
2.39	0	0	0	135.513	0	0
1.571	0	0	0	30.514	0	0
2.517	30.502	0	48.842	194.277	68.315	79.454
2.893	0	0	77.981	305.432	0	0
2.441	0	0	0	66.541	0	0
2.732	0	0	92.871	200.681	0	0
2.469	0	0	50.486	58.329	0	0
2.642	0	0	63.573	140.876	0	0
2.715	0	0	83.465	300.085	0	0
2.461	0	0	53.786	61.771	0	0
2.649	0	0	54.247	142.116	0	0
2.496	0	0	71.193	83.292	0	0
2.504	0	0	45.631	51.544	0	0
2.53	0	0	55.385	61.557	0	0
2.999	0	0	0	221.893	0	0
2.959	0	0	52.997	62.717	0	0
2.124	0	0	0	96.158	51.243	0
2.439	0	0	41.091	50.114	0	0
2.401	0	0	48.956	122.894	0	0

2.968	0	0	84.542	238.997	0	0
2.139	0	0	0	57.687	0	0
2.506	0	0	28.728	92.232	0	0
2.491	0	0	0	221.13	0	0
0.996	0	0	22.521	26.992	0	0
2.022	0	0	0	55.344	0	0
2.257	0	0	35.261	88.033	0	0
2.684	0	0	65.216	67.71	0	0
3.133	0	0	0	203.118	0	0
2.684	0	0	0	76.274	86.16	0
2.663	0	0	0	68.005	0	0
2.249	0	0	0	119.929	0	0
2.936	0	0	60.653	256.442	0	0
2.234	0	0	37.314	93.549	45.469	0
2.475	0	0	0	144.113	0	0
3.017	0	0	0	313.878	0	0
1.846	0	0	0	0	0	0
2.858	0	0	88.886	241.907	0	0
2.95	0	0	68.993	427.77	0	0
3.153	0	0	0	274.664	0	0
2.938	0	0	215.986	493.703	0	126.331
2.19	0	0	25.219	143.086	0	0
2.376	0	0	0	187.34	0	0
2.525	0	0	45.858	126.329	0	0
2.439	0	0	52.402	63.208	0	0
3.04	0	0	132.92	237.832	0	0
2.455	0	0	46.957	122.662	0	0
2.471	0	0	59.359	68.331	0	0

D/Dr09	D/Dr10	D/Dr11	D/Dr12	T(N..N)	T(N..O)	T(N..S)
0	0	0	0	0	0	0
0	0	0	0	9	44	0
0	120.203	0	0	0	32	0
0	0	0	0	0	5	0
58.373	0	0	0	19	72	0
0	0	0	0	0	41	0
0	0	0	0	54	112	0
28.103	0	0	0	11	12	0
0	65.815	69.678	0	10	0	0
0	0	0	0	0	7	0
0	0	0	0	92	514	0
142.67	0	0	0	0	23	0
0	0	141.873	0	0	0	0
0	0	0	0	6	58	0
0	0	0	0	12	76	9
0	0	0	0	12	53	9
0	0	0	0	0	3	0
0	0	0	0	10	52	0
0	0	0	0	11	35	0
0	0	0	0	0	18	0
61.619	71.864	0	0	6	24	0
67.173	0	0	0	84	72	17
81.373	0	0	0	0	12	0
0	0	0	0	3	26	3
0	0	0	0	3	7	0
67.71	153.146	0	0	0	0	0
0	0	0	0	0	18	0
0	0	0	0	40	200	0
83.963	0	0	0	7	0	0
0	0	0	0	0	30	0
0	0	0	0	26	158	20
564.535	284.748	167.151	195.755	38	111	0
77.125	0	0	0	0	7	0
0	0	0	0	3	3	0
274.895	390.398	342.582	101.174	0	24	0
0	0	0	0	46	82	0
0	188.02	0	77.701	0	10	0
45.931	0	0	0	10	20	0
128.214	0	0	0	0	0	0
97.719	129.98	0	0	3	40	0
140.952	0	0	0	84	172	0
0	0	107.792	0	0	0	0
0	452.324	0	0	0	0	0
107.984	0	0	0	0	12	0
217.373	0	0	0	8	52	0
0	0	0	0	12	53	9
0	0	0	0	12	76	9
0	0	0	0	67	225	46
0	0	0	0	191	224	172

0	0	0	0	91	180	58
0	0	0	0	46	218	42
0	0	0	0	230	604	92
0	0	0	0	46	244	42
0	0	0	0	18	97	33
0	0	0	0	12	76	9
0	0	0	0	40	236	50
0	0	0	0	91	282	58
0	0	0	0	46	144	42
0	0	0	0	219	435	152
0	0	0	0	39	185	15
0	0	0	0	13	26	11
0	0	0	0	12	53	9
0	0	0	0	14	51	29
0	0	0	0	3	52	14
0	0	0	0	12	53	9
0	0	0	0	0	8	8
0	0	0	0	3	39	0
0	65.545	0	0	12	44	18
0	0	0	0	5	0	0
0	135.884	0	0	4	0	10
0	0	0	0	0	8	3
0	135.884	0	0	0	0	7
81.153	0	0	0	6	26	5
62.476	0	0	0	0	7	0
0	0	0	0	76	0	27
0	0	0	0	11	56	0
0	122.983	0	0	0	0	0
53.858	57.343	0	0	0	41	0
0	96.621	0	0	14	65	0
0	0	0	0	3	51	13
0	0	0	0	0	0	0
99.336	0	0	0	0	14	0
0	0	0	0	0	0	0
0	0	149.978	0	22	0	0
0	0	0	0	0	0	0
0	0	0	0	10	24	10
0	0	130.587	0	4	0	0
67.71	153.146	0	0	0	0	0
0	0	77.125	0	3	3	0
0	0	0	0	0	10	0
0	0	0	0	14	68	10
58.191	0	0	0	10	53	0
0	0	0	0	0	0	0
63.008	146.307	0	0	0	0	0
212.004	427.064	0	0	0	0	0
0	0	135.496	0	3	46	9
0	0	0	0	0	3	0
0	0	0	0	16	10	0
0	209.379	0	259.121	0	106	0

0	0	0	0	30	124	49
259.813	0	0	0	60	56	0
0	269.926	0	0	5	86	0
0	0	0	0	18	80	0
0	0	0	0	3	18	0
114.1	0	0	0	0	13	0
234.184	284.952	0	0	0	0	0
0	0	0	0	134	100	115
0	0	0	0	0	0	0
0	0	0	0	0	0	0
0	0	0	0	4	6	0
0	0	0	0	0	10	0
67.296	153.825	0	0	9	20	0
0	0	0	0	3	29	0
0	0	0	0	54	24	0
63.836	0	0	0	0	0	0
0	67.714	74.62	0	7	34	0
0	0	0	0	0	4	0
0	227.091	0	0	14	19	20
0	0	0	0	0	0	0
0	0	0	0	0	0	0
141.167	330.996	0	0	0	0	0
0	0	0	0	6	44	5
101.45	233.852	0	0	0	0	0
0	0	0	0	0	10	0
85.364	46.668	54.866	60.14	0	18	0
69.726	0	0	0	19	109	0
0	0	0	0	0	0	0
0	0	0	0	82	231	0
0	0	0	0	40	118	19
0	0	0	0	18	86	10
85.712	0	0	0	26	13	0
0	0	0	0	0	14	0
0	65.545	0	0	12	44	18
107.483	125.421	135.163	51.798	0	18	0
0	0	0	0	0	0	0
0	0	0	0	182	51	0
99.336	0	0	0	0	15	0
85.083	0	0	0	0	14	0
0	0	0	0	84	49	0
0	0	0	0	7	60	0
0	0	0	0	0	14	0
95.564	0	0	0	13	75	0
0	0	0	0	0	0	0
0	0	0	0	0	0	0
0	0	0	0	4	35	15
107.422	0	0	0	10	28	3
0	0	0	0	60	0	0
0	0	0	0	3	8	0
0	250.17	0	104.165	14	77	0

0	0	0	0	3	3	0
0	0	0	0	18	62	0
0	102.68	0	0	14	65	0
0	0	81.872	0	3	6	0
0	0	0	0	84	57	0
0	98.336	0	0	8	44	15
0	0	0	0	7	22	0
0	0	0	0	0	10	0
0	0	144.178	0	8	0	0
0	0	0	0	3	3	0
28.103	0	0	0	10	0	11
0	0	0	0	16	0	0
0	0	0	0	0	5	0
0	0	0	0	3	48	3
0	179.493	0	0	145	503	0
64.957	145.587	0	0	0	0	0
0	0	0	0	18	26	0
0	0	0	0	0	18	0
0	0	0	0	3	27	0
0	69.273	73.13	0	7	0	0
0	216.278	0	0	0	39	8
107.483	125.421	135.163	51.798	0	18	0
105.457	116.256	0	0	14	77	0
0	0	0	0	0	21	0
0	99.161	0	0	0	26	0
0	126.77	0	0	3	37	3
0	64.972	0	0	0	28	0
142.38	162.92	176.527	67.199	0	15	0
126.548	144.723	157.077	59.609	0	14	0
0	68.623	0	0	0	0	0
0	0	0	0	6	10	0
183.06	194.94	112.75	0	25	53	0
0	0	0	0	6	54	0
0	0	0	0	5	33	6
0	0	0	0	7	60	0
0	0	0	0	22	84	0
0	0	0	0	0	16	0
0	93.032	0	0	14	65	0
0	0	130.587	0	0	0	0
0	220.412	0	0	13	167	0
0	250.17	0	104.165	14	77	0
100.089	0	0	0	10	46	3
118.121	0	0	0	14	14	0
0	0	0	0	14	68	10
0	0	77.944	0	3	6	0
122.854	143.937	154.096	58.659	0	21	0
0	0	0	0	0	17	0
119.27	256.532	0	0	9	58	0
0	0	0	0	0	8	0
0	0	0	0	0	5	0

0	0	0	0	0	0	0
0	0	0	0	0	8	0
0	0	0	0	0	6	0
0	0	0	0	0	3	0
73.377	0	0	0	8	18	0
0	247.404	0	0	18	59	28
0	0	184.76	0	46	44	0
0	0	0	0	5	39	4
0	0	0	0	0	14	0
0	0	0	0	9	31	0
0	144.277	0	0	0	0	0
0	128.854	0	0	28	126	0
61.884	139.176	0	0	0	0	0
52.896	117.771	0	0	0	0	0
0	0	0	0	18	11	0
0	0	0	0	9	20	0
0	0	0	0	0	15	0
0	82.309	0	0	0	7	0
0	0	0	0	0	0	0
0	82.309	0	0	0	7	0
0	0	0	0	8	0	0
0	0	205.604	0	8	43	14
0	94.138	0	0	6	20	0
0	94.138	0	0	6	20	0
0	0	0	0	38	68	20
0	0	0	0	4	62	0
54.963	0	0	0	0	11	0
0	0	0	0	0	41	0
125.248	141.831	0	0	35	47	0
77.431	0	0	0	53	0	0
104.604	234.607	0	0	9	56	0
0	0	0	0	3	3	0
0	0	0	0	0	0	0
0	0	0	0	0	21	0
134.097	0	0	0	88	137	30
0	0	0	0	6	24	7
0	0	0	0	4	14	12
0	0	0	0	14	32	8
0	0	0	0	26	30	12
0	0	0	0	0	18	10
0	0	0	0	18	30	9
101.932	0	0	0	0	0	0
0	0	0	0	18	58	13
81.501	0	0	0	21	32	14
0	0	0	0	0	0	0
0	0	0	0	7	57	6
97.254	0	0	0	29	41	0
0	0	82.589	0	3	6	0
77.07	0	0	0	21	153	0
86.28	0	0	0	8	44	26

0	128.854	0	0	28	126	0
0	0	0	0	0	15	0
46.808	102.038	0	0	0	0	0
0	269.926	0	0	5	90	0
41.667	0	0	0	10	20	0
0	0	0	0	0	8	0
60.648	0	0	0	0	0	4
0	0	0	0	23	54	10
0	0	0	0	9	50	0
0	0	0	0	3	21	7
0	0	0	0	0	8	3
0	0	0	0	0	11	0
117.748	0	0	0	42	18	0
0	69.273	73.13	0	10	0	0
0	0	0	0	8	94	0
0	0	0	0	52	95	0
0	0	0	0	0	0	0
0	0	0	0	38	152	0
114.908	248.909	0	0	9	58	0
0	0	0	0	6	68	0
707.589	332.231	356.198	185.021	42	233	0
151.812	177.315	64.275	73.253	3	18	0
0	99.218	0	0	0	0	0
86.012	0	0	0	42	35	0
0	0	0	0	36	95	0
239.169	0	0	0	37	32	22
90.489	0	0	0	9	9	0
0	0	0	0	0	11	0

T(N..P)	T(N..F)	T(N..Cl)	T(N..Br)	T(N..I)	T(O..O)	T(O..S)
0	0	0	0	0	46	0
0	0	0	0	0	38	0
0	0	0	0	0	98	0
0	0	0	0	0	7	0
0	0	0	0	0	18	0
10	0	0	0	0	54	0
0	0	0	0	0	26	0
0	0	0	0	0	0	0
0	0	24	0	0	0	0
0	0	0	0	0	3	0
0	0	0	0	0	576	0
0	0	0	0	12	18	0
0	0	0	0	0	0	0
0	0	16	0	0	48	0
0	0	0	0	0	74	27
0	0	0	0	0	31	17
0	0	0	0	0	0	0
0	310	0	0	0	16	0
0	0	0	0	0	20	0
0	0	0	0	0	8	0
0	0	0	0	0	4	0
0	0	0	0	0	0	8
0	0	6	0	0	14	0
0	0	0	0	0	31	17
0	0	0	0	0	0	0
0	0	0	0	0	68	0
0	0	0	0	0	20	0
0	0	0	0	0	164	0
0	0	0	0	0	0	0
0	0	0	0	0	42	0
0	0	0	0	0	86	24
0	0	0	26	0	45	0
0	0	0	0	0	3	0
0	0	0	0	0	0	0
0	0	0	0	0	29	0
0	0	0	0	0	4	0
0	0	0	0	0	6	0
0	0	0	0	0	4	0
0	0	0	0	0	34	0
0	0	0	0	0	21	0
0	0	0	0	0	12	0
0	0	0	0	0	0	0
0	0	0	0	0	204	0
0	0	5	0	0	0	0
0	0	0	0	0	33	0
0	0	18	0	0	31	19
0	0	0	0	0	74	29
0	0	0	0	0	58	60
0	0	0	0	0	31	77

0	0	0	0	0	62	66
0	0	0	0	0	154	102
0	0	0	0	0	240	138
0	0	0	0	0	144	102
0	0	0	0	0	124	93
0	0	0	0	0	74	29
0	0	0	0	0	217	91
0	0	0	0	0	154	102
0	0	0	0	0	62	66
0	0	0	0	0	182	161
0	0	0	0	0	203	43
0	51	0	0	0	0	0
0	0	0	0	0	31	19
0	0	0	0	0	31	50
0	0	0	0	0	89	84
0	0	0	0	0	31	19
0	0	0	0	0	0	10
0	0	15	0	0	6	0
0	0	14	0	0	24	20
0	0	15	0	0	0	0
0	0	12	0	0	0	0
0	0	14	0	0	8	3
0	0	8	0	0	0	0
0	0	10	0	0	32	12
0	0	8	0	0	4	0
0	0	0	0	0	0	0
0	0	0	0	0	31	0
0	24	0	0	0	0	0
0	0	0	0	0	49	0
0	14	0	0	0	8	0
0	0	9	0	0	46	19
0	0	0	0	0	6	0
0	0	8	0	0	35	0
0	0	26	0	0	0	0
0	0	24	0	0	0	0
0	0	16	0	0	0	0
0	0	0	0	0	0	0
0	0	0	0	0	0	0
0	0	0	0	0	68	0
0	0	10	0	0	0	0
0	0	6	0	0	0	0
0	0	38	0	0	62	24
0	0	0	0	0	18	0
0	0	0	0	0	8	0
0	0	0	0	0	46	0
0	0	0	0	0	877	0
0	0	0	0	0	32	18
0	0	0	0	0	0	0
0	0	0	0	0	0	0
0	0	0	0	0	680	0

0	0	0	0	0	114	89
0	0	46	0	0	12	0
0	0	0	0	0	158	0
0	0	0	0	0	52	0
0	0	0	0	0	9	0
0	0	0	0	0	8	0
0	0	0	0	0	590	0
0	0	0	0	0	0	32
0	0	0	0	0	10	0
0	0	0	0	0	12	0
0	0	0	0	0	0	0
0	0	6	0	0	0	8
0	0	0	0	0	11	0
0	108	0	0	0	14	0
0	84	0	0	0	0	0
0	0	0	0	0	4	0
0	17	0	0	0	14	0
0	6	0	0	0	4	0
0	78	0	0	0	0	13
0	0	0	0	0	0	0
0	0	0	0	0	18	0
0	0	0	0	0	42	30
0	0	8	0	0	56	18
0	0	0	0	0	105	0
0	0	0	0	0	0	0
0	0	0	0	0	14	0
0	0	0	0	0	34	0
0	0	0	0	0	12	0
0	0	0	0	0	108	0
0	0	0	0	0	82	23
0	0	33	0	0	82	23
0	0	0	0	0	0	0
0	33	10	0	0	26	0
0	0	14	0	0	24	20
0	0	0	0	0	12	0
0	0	0	0	0	0	0
0	0	0	0	0	0	0
0	0	6	0	0	35	0
0	0	0	0	0	18	0
0	0	0	0	0	0	0
0	0	0	0	0	124	0
0	0	0	0	0	54	0
0	0	0	0	0	52	0
0	0	0	0	0	10	0
0	0	0	0	0	14	0
0	0	0	0	0	14	8
0	81	0	0	0	6	5
0	0	0	0	0	0	0
0	0	0	0	0	5	0
0	14	0	0	0	27	0

0	0	0	0	0	0	0
0	15	0	0	0	46	0
0	26	0	0	0	8	0
0	0	20	0	0	3	0
0	0	53	0	0	0	0
0	0	0	0	0	24	32
0	0	26	0	0	0	0
0	0	0	0	0	0	0
0	0	0	0	0	0	0
0	0	0	0	0	0	0
0	0	0	0	0	0	0
0	0	0	0	0	0	0
0	0	0	0	0	0	0
0	0	0	0	0	97	31
0	0	0	0	0	48	0
0	0	0	0	0	68	0
0	0	16	0	0	4	0
0	0	0	0	0	20	0
0	0	0	0	0	12	0
0	18	17	0	0	0	0
0	0	4	0	0	24	17
0	0	0	0	0	12	0
0	14	0	0	0	27	0
0	0	0	0	0	12	0
0	0	0	0	0	34	0
0	0	0	0	0	62	24
0	0	0	0	0	8	0
0	0	0	0	0	13	0
0	0	0	0	0	12	0
0	0	0	0	0	18	0
0	0	0	0	0	0	0
0	0	0	25	0	12	0
0	0	0	0	0	80	0
0	0	0	0	0	52	18
0	0	0	0	0	88	0
0	0	0	0	0	61	0
0	0	0	0	0	14	0
0	14	0	0	0	8	0
0	0	0	0	0	0	0
0	0	0	0	0	438	0
0	14	0	0	0	27	0
0	0	0	0	0	24	11
0	0	0	0	0	0	0
0	0	0	0	0	62	24
0	0	10	0	0	3	0
0	0	0	0	0	27	0
0	0	0	0	0	19	0
0	0	0	0	0	48	0
0	0	0	0	0	12	0
0	0	0	0	0	7	0

0	0	0	0	0	0	0
0	0	0	0	0	12	0
0	0	0	0	0	4	0
0	0	0	0	0	4	0
0	0	0	0	0	3	0
0	0	0	0	0	30	39
0	0	0	0	0	6	0
0	0	0	0	0	44	13
0	0	3	0	0	0	0
0	0	0	0	0	20	0
0	0	0	0	0	164	0
0	0	0	0	0	54	0
0	0	0	0	0	68	0
0	0	0	0	0	11	0
0	0	0	0	0	0	0
0	0	0	0	0	0	0
0	0	0	0	0	14	0
0	0	0	0	0	3	0
0	0	0	0	0	0	0
0	0	0	0	0	3	0
0	0	30	0	0	0	0
0	0	0	0	0	3	23
0	0	0	0	0	6	0
0	0	0	0	0	6	0
0	0	0	0	0	20	17
0	0	0	0	0	30	0
0	45	0	0	0	0	4
10	0	0	0	0	54	0
0	42	0	0	0	11	0
0	0	0	0	0	0	0
0	0	0	0	0	54	0
0	0	0	0	0	0	0
0	0	0	0	0	8	0
0	0	0	0	0	29	0
0	0	0	0	0	34	12
0	0	0	0	0	14	6
0	0	0	0	0	5	19
0	0	0	0	0	10	4
0	0	0	0	0	0	0
0	0	0	0	0	16	10
0	0	0	0	0	8	3
0	0	0	0	0	24	22
0	0	0	0	0	28	10
0	0	0	0	0	0	0
0	0	0	0	0	16	21
0	0	0	0	0	80	24
0	0	0	0	0	0	0
0	0	10	0	0	3	0
37	0	0	0	0	9	0
0	0	0	0	0	24	24

0	0	0	0	0	54	0
0	0	0	0	0	14	0
0	0	0	0	0	10	0
0	0	0	0	0	164	0
0	0	0	0	0	4	0
0	0	0	0	0	4	8
0	0	4	0	0	0	0
0	0	0	0	0	18	15
0	0	0	0	0	47	0
0	0	0	0	0	8	3
0	0	0	0	0	8	3
0	0	0	0	0	5	0
0	0	47	0	0	0	0
0	0	49	0	0	0	0
0	0	0	0	0	10	0
0	0	0	0	0	32	0
0	0	0	0	0	0	0
0	0	0	0	0	10	0
0	0	0	0	0	48	0
0	0	0	0	0	66	0
0	0	0	0	0	187	0
0	0	0	0	0	0	0
0	0	0	0	0	26	0
0	0	0	0	0	0	0
0	0	0	0	0	33	0
0	0	27	0	0	0	14
0	0	0	0	0	0	0
0	0	7	0	0	14	0

T(O..P)	T(O..F)	T(O..Cl)	T(O..Br)	T(O..I)	T(S..S)	T(S..P)
0	0	0	0	0	0	0
0	0	0	0	0	0	0
0	0	0	0	0	0	0
0	0	0	0	0	0	0
0	0	0	0	0	0	0
18	0	0	0	0	0	0
0	0	0	0	0	0	0
0	0	0	0	0	0	0
0	0	0	0	0	0	0
0	0	0	0	0	0	0
0	0	0	0	0	0	0
0	0	0	0	30	0	0
0	0	0	0	0	0	0
0	0	31	0	0	0	0
0	0	0	0	0	0	0
0	0	0	0	0	0	0
0	0	0	0	0	0	0
0	193	0	0	0	0	0
0	0	0	0	0	0	0
0	0	0	0	0	0	0
0	0	0	0	0	0	0
0	0	0	0	0	0	0
0	0	30	0	0	0	0
0	0	0	0	0	0	0
0	0	0	0	0	0	0
0	30	0	0	0	0	0
0	0	0	0	0	0	0
0	0	0	0	0	0	0
0	0	0	0	0	0	0
0	0	0	0	0	0	0
0	0	0	0	0	0	0
0	0	0	60	0	0	0
0	0	0	0	0	0	0
0	0	0	0	0	0	0
0	0	0	0	0	0	0
0	0	0	0	0	0	0
0	0	0	0	0	0	0
0	0	0	0	0	0	0
0	0	0	0	0	0	0
0	0	0	0	0	0	0
0	0	0	0	0	0	0
0	0	0	0	0	0	0
0	0	0	0	0	0	0
0	0	20	0	0	0	0
0	0	0	0	0	0	0
0	0	21	0	0	0	0
0	0	0	0	0	0	0
0	0	0	0	0	4	0
0	0	0	0	0	10	0

0	0	0	0	0	8	0
0	0	0	0	0	8	0
0	0	0	0	0	4	0
0	0	0	0	0	8	0
0	0	0	0	0	7	0
0	0	0	0	0	0	0
0	0	0	0	0	6	0
0	0	0	0	0	8	0
0	0	0	0	0	8	0
0	0	0	0	0	24	0
0	0	0	0	0	0	0
0	60	0	0	0	0	0
0	0	0	0	0	0	0
0	0	0	0	0	7	0
0	0	0	0	0	7	0
0	0	0	0	0	0	0
0	0	0	0	0	0	0
0	0	42	0	0	0	0
0	0	20	0	0	4	0
0	0	0	0	0	0	0
0	0	0	0	0	0	0
0	0	20	0	0	0	0
0	0	0	0	0	0	0
0	0	22	0	0	0	0
0	0	15	0	0	0	0
0	0	0	0	0	0	0
0	0	0	0	0	0	0
0	0	0	0	0	0	0
0	0	0	0	0	0	0
0	19	0	0	0	0	0
0	0	27	0	0	0	0
0	0	21	0	0	0	0
0	0	39	0	0	0	0
0	0	0	0	0	0	0
0	0	0	0	0	0	0
0	0	20	0	0	0	0
0	0	0	0	0	0	0
0	0	0	0	0	0	0
0	30	0	0	0	0	0
0	0	7	0	0	0	0
0	0	32	0	0	0	0
0	0	88	0	0	0	0
0	0	0	0	0	0	0
0	48	0	0	0	0	0
0	0	0	0	0	0	0
0	0	0	0	0	0	0
0	0	0	0	0	0	0
0	0	0	0	0	0	0
0	0	0	0	0	0	0
0	0	0	0	0	0	0

0	0	0	0	0	17	0
0	0	21	0	0	0	0
0	0	0	0	0	0	0
0	0	0	0	0	0	0
0	0	0	0	0	0	0
0	0	0	0	0	0	0
0	0	0	0	0	0	0
0	0	0	0	0	18	0
0	0	0	0	0	0	0
0	0	0	0	0	0	0
0	0	0	0	0	0	0
0	0	18	0	0	0	0
0	0	0	0	0	0	0
0	111	0	0	0	0	0
0	10	0	0	0	0	0
0	14	0	0	0	0	0
0	23	0	0	0	0	0
0	8	0	0	0	0	0
0	45	0	0	0	0	0
0	12	0	0	0	0	0
0	0	0	0	0	0	0
0	239	0	0	0	0	0
0	0	27	0	0	0	0
0	0	0	0	0	0	0
0	0	0	0	0	0	0
0	0	0	0	0	0	0
0	0	0	0	0	0	0
0	0	0	0	0	0	0
0	0	0	0	0	0	0
0	0	0	0	0	0	0
0	0	51	0	0	0	0
0	0	0	0	0	0	0
0	102	32	0	0	0	0
0	0	20	0	0	4	0
0	0	0	0	0	0	0
0	0	0	0	0	0	0
0	0	0	0	0	0	0
0	0	39	0	0	0	0
0	0	0	0	0	0	0
0	0	0	0	0	0	0
0	0	0	0	0	0	0
0	0	0	0	0	0	0
0	0	0	0	0	0	0
0	0	30	0	0	0	0
0	0	0	0	0	0	0
0	0	0	0	0	0	0
0	36	0	0	0	0	0
0	0	0	0	0	0	0
0	0	0	0	0	0	0
0	23	0	0	0	0	0

0	0	0	0	0	0	0
0	28	0	0	0	0	0
0	38	0	0	0	0	0
0	0	27	0	0	0	0
0	0	4	0	0	0	0
0	0	0	0	0	6	0
0	0	44	0	0	0	0
0	0	0	0	0	0	0
0	0	0	0	0	0	0
0	0	0	0	0	0	0
0	0	0	0	0	0	0
0	0	0	0	0	0	0
0	0	0	0	0	0	0
0	0	0	0	0	0	0
0	0	0	0	0	0	0
0	0	0	0	0	0	0
0	0	10	0	0	0	0
0	0	0	0	0	0	0
0	0	0	0	0	0	0
0	0	0	0	0	0	0
0	0	51	0	0	0	0
0	0	0	0	0	0	0
0	23	0	0	0	0	0
0	0	0	0	0	0	0
0	0	0	0	0	0	0
0	0	0	0	0	0	0
0	0	0	0	0	0	0
0	0	0	0	0	0	0
0	0	0	0	0	0	0
0	0	0	0	0	0	0
0	0	0	0	0	0	0
0	0	0	20	0	0	0
0	0	0	0	0	0	0
0	0	0	0	0	0	0
0	0	0	0	0	0	0
0	0	0	0	0	0	0
0	0	0	0	0	0	0
0	19	0	0	0	0	0
0	0	0	0	0	0	0
0	0	0	0	0	0	0
0	23	0	0	0	0	0
0	0	0	0	0	0	0
0	0	0	0	0	0	0
0	0	0	0	0	0	0
0	0	14	0	0	0	0
0	0	0	0	0	0	0
0	0	0	0	0	0	0
0	0	0	0	0	0	0
0	0	0	0	0	0	0
0	0	0	0	0	0	0

0	0	0	0	0	0	0
0	0	0	0	0	0	0
0	0	0	0	0	0	0
0	0	0	0	0	0	0
0	0	0	0	0	0	0
0	0	0	0	0	5	0
0	0	0	0	0	0	0
0	0	0	0	0	0	0
0	0	12	0	0	0	0
0	0	0	0	0	0	0
0	0	0	0	0	0	0
0	0	0	0	0	0	0
0	0	0	0	0	0	0
0	0	0	0	0	0	0
0	0	0	0	0	0	0
0	0	0	0	0	0	0
0	0	0	0	0	0	0
0	0	0	0	0	0	0
0	0	0	0	0	0	0
0	0	0	0	0	0	0
0	0	0	0	0	0	0
0	0	0	0	0	0	0
0	0	0	0	0	0	0
0	0	0	0	0	0	0
0	0	0	0	0	0	0
0	0	0	0	0	0	0
0	0	0	0	0	0	0
18	0	0	0	0	0	0
0	18	0	0	0	0	0
0	0	0	0	0	0	0
0	0	0	0	0	0	0
0	0	0	0	0	0	0
0	0	0	0	0	0	0
0	0	0	0	0	0	0
0	0	0	0	0	0	0
0	0	0	0	0	0	0
0	0	0	0	0	0	0
0	0	0	0	0	0	0
0	0	0	0	0	0	0
0	0	0	0	0	0	0
0	0	0	0	0	0	0
0	26	0	0	0	0	0
0	0	0	0	0	0	0
0	0	0	0	0	0	0
0	0	0	0	0	0	0
0	0	0	0	0	0	0
0	0	0	0	0	0	0
0	0	14	0	0	0	0
0	0	0	0	0	0	0
0	0	0	0	0	3	0

0	0	0	0	0	0	0
0	0	0	0	0	0	0
0	0	0	0	0	0	0
0	0	0	0	0	0	0
0	0	0	0	0	0	0
0	0	0	0	0	0	0
0	0	0	0	0	0	0
0	0	0	0	0	0	0
0	0	0	0	0	0	0
0	0	0	0	0	0	0
0	0	0	0	0	0	0
0	0	0	0	0	0	0
0	0	13	0	0	0	0
0	0	0	0	0	0	0
0	0	0	0	0	0	0
0	0	0	0	0	0	0
0	0	0	0	0	0	0
0	0	0	0	0	0	0
0	0	0	0	0	0	0
0	0	0	0	0	0	0
0	0	0	0	0	0	0
0	0	0	0	0	0	0
0	0	0	0	0	0	0
0	0	0	0	0	0	0
0	0	0	0	0	0	0
0	0	6	0	0	0	0
0	0	0	0	0	0	0
0	0	28	0	0	0	0

T(S..F)	T(S..Cl)	T(S..Br)	T(S..I)	T(P..P)	T(P..F)	T(P..Cl)
0	0	0	0	0	0	0
0	0	0	0	0	0	0
0	0	0	0	0	0	0
0	0	0	0	0	0	0
0	0	0	0	0	0	0
0	0	0	0	0	0	0
0	0	0	0	0	0	0
0	0	0	0	0	0	0
0	0	0	0	0	0	0
0	0	0	0	0	0	0
0	0	0	0	0	0	0
0	0	0	0	0	0	0
0	0	0	0	0	0	0
0	0	0	0	0	0	0
0	0	0	0	0	0	0
0	0	0	0	0	0	0
0	0	0	0	0	0	0
0	0	0	0	0	0	0
0	0	0	0	0	0	0
0	0	0	0	0	0	0
0	0	0	0	0	0	0
0	0	0	0	0	0	0
0	0	0	0	0	0	0
0	0	0	0	0	0	0
0	0	0	0	0	0	0
0	0	0	0	0	0	0
0	0	0	0	0	0	0
0	0	0	0	0	0	0
0	0	0	0	0	0	0
0	0	0	0	0	0	0
0	0	0	0	0	0	0
0	0	0	0	0	0	0
0	0	0	0	0	0	0
0	0	0	0	0	0	0
0	0	0	0	0	0	0
0	0	0	0	0	0	0
0	0	0	0	0	0	0
0	0	0	0	0	0	0
0	0	0	0	0	0	0
0	0	0	0	0	0	0
0	0	0	0	0	0	0
0	0	0	0	0	0	0
0	0	0	0	0	0	0
0	0	0	0	0	0	0
0	0	0	0	0	0	0
0	3	0	0	0	0	0
0	0	0	0	0	0	0
0	0	0	0	0	0	0
0	0	0	0	0	0	0

0	0	0	0	0	0	0
0	0	0	0	0	0	0
0	0	0	0	0	0	0
0	0	0	0	0	0	0
0	0	0	0	0	0	0
0	0	0	0	0	0	0
0	0	0	0	0	0	0
0	0	0	0	0	0	0
0	0	0	0	0	0	0
0	0	0	0	0	0	0
0	0	0	0	0	0	0
27	0	0	0	0	0	0
0	0	0	0	0	0	0
0	0	0	0	0	0	0
0	0	0	0	0	0	0
0	0	0	0	0	0	0
0	0	0	0	0	0	0
0	0	0	0	0	0	0
0	8	0	0	0	0	0
0	0	0	0	0	0	0
0	5	0	0	0	0	0
0	5	0	0	0	0	0
0	5	0	0	0	0	0
0	3	0	0	0	0	0
0	0	0	0	0	0	0
0	0	0	0	0	0	0
0	0	0	0	0	0	0
0	0	0	0	0	0	0
0	0	0	0	0	0	0
0	0	0	0	0	0	0
0	6	0	0	0	0	0
0	0	0	0	0	0	0
0	0	0	0	0	0	0
0	0	0	0	0	0	0
0	0	0	0	0	0	0
0	0	0	0	0	0	8
0	0	0	0	0	0	0
0	0	0	0	0	0	0
0	0	0	0	0	0	0
0	0	0	0	0	0	0
0	0	0	0	0	0	0
0	18	0	0	0	0	0
0	0	0	0	0	0	0
0	0	0	0	0	0	0
0	0	0	0	0	0	0
0	0	0	0	0	0	0
0	0	0	0	0	0	0
0	0	0	0	0	0	0
0	0	0	0	0	0	0
0	0	0	0	0	0	0

0	0	0	0	0	0	0
0	0	0	0	0	0	0
0	0	0	0	0	0	0
0	0	0	0	0	0	0
0	0	0	0	0	0	0
0	0	0	0	0	0	0
0	0	0	0	0	0	0
0	0	0	0	0	0	0
0	0	0	0	0	0	0
0	0	0	0	0	0	0
0	0	0	0	0	0	0
0	7	0	0	0	0	0
0	0	0	0	0	0	0
0	0	0	0	0	0	0
0	0	0	0	0	0	0
0	0	0	0	0	0	0
0	0	0	0	0	0	0
0	0	0	0	0	0	0
18	0	0	0	0	0	0
0	0	0	0	0	0	0
0	0	0	0	0	0	0
28	0	0	0	0	0	0
0	3	0	0	0	0	0
0	0	0	0	0	0	0
0	0	0	0	0	0	0
0	0	0	0	0	0	0
0	0	0	0	0	0	0
0	0	0	0	0	0	0
0	0	0	0	0	0	0
0	0	0	0	0	0	0
0	12	0	0	0	0	0
0	0	0	0	0	0	0
0	0	0	0	0	0	0
0	8	0	0	0	0	0
0	0	0	0	0	0	0
0	0	0	0	0	0	0
0	0	0	0	0	0	0
0	0	0	0	0	0	0
0	0	0	0	0	0	0
0	0	0	0	0	0	0
0	0	0	0	0	0	0
0	0	0	0	0	0	0
0	0	0	0	0	0	0
0	0	0	0	0	0	0
0	0	0	0	0	0	0
0	0	0	0	0	0	0
24	0	0	0	0	0	0
0	0	0	0	0	0	0
0	0	0	0	0	0	0
0	0	0	0	0	0	0

0	0	0	0	0	0	0
0	0	0	0	0	0	0
0	0	0	0	0	0	0
0	0	0	0	0	0	0
0	0	0	0	0	0	0
0	0	0	0	0	0	0
0	0	0	0	0	0	0
0	0	0	0	0	0	0
0	0	0	0	0	0	0
0	0	0	0	0	0	0
0	0	0	0	0	0	0
0	0	0	0	0	0	0
0	0	0	0	0	0	0
0	0	0	0	0	0	0
0	0	0	0	0	0	0
0	0	0	0	0	0	0
0	0	0	0	0	0	0
0	0	0	0	0	0	0
0	0	0	0	0	0	0
0	0	0	0	0	0	0
0	12	0	0	0	0	0
0	0	0	0	0	0	0
0	0	0	0	0	0	0
0	0	0	0	0	0	0
0	0	0	0	0	0	0
0	0	0	0	0	0	0
0	0	0	0	0	0	0
0	0	0	0	0	0	0
0	0	0	0	0	0	0
0	0	0	0	0	0	0
0	0	0	0	0	0	0
0	0	0	0	0	0	0
0	0	0	0	0	0	0
0	0	0	0	0	0	0
0	0	0	0	0	0	0
0	0	0	0	0	0	0
0	0	0	0	0	0	0
0	0	0	0	0	0	0
0	0	0	0	0	0	0
0	0	0	0	0	0	0
0	0	0	0	0	0	0
0	0	0	0	0	0	0
0	0	0	0	0	0	0
0	0	0	0	0	0	0
0	0	0	0	0	0	0
0	0	0	0	0	0	0
0	0	0	0	0	0	0
0	0	0	0	0	0	0
0	0	0	0	0	0	0
0	0	0	0	0	0	0

0	0	0	0	0	0	0
0	0	0	0	0	0	0
0	0	0	0	0	0	0
0	0	0	0	0	0	0
0	0	0	0	0	0	0
0	0	0	0	0	0	0
0	0	0	0	0	0	0
0	0	0	0	0	0	0
0	0	0	0	0	0	0
0	0	0	0	0	0	0
0	0	0	0	0	0	0
0	0	0	0	0	0	0
0	0	0	0	0	0	0
0	0	0	0	0	0	0
0	0	0	0	0	0	0
0	0	0	0	0	0	0
0	0	0	0	0	0	0
0	0	0	0	0	0	0
0	0	0	0	0	0	0
0	0	0	0	0	0	0
0	0	0	0	0	0	0
0	0	0	0	0	0	0
0	0	0	0	0	0	0
0	0	0	0	0	0	0
0	0	0	0	0	0	0
0	0	0	0	0	0	0
18	0	0	0	0	0	0
0	0	0	0	0	0	0
0	0	0	0	0	0	0
0	0	0	0	0	0	0
0	0	0	0	0	0	0
0	0	0	0	0	0	0
0	0	0	0	0	0	0
0	0	0	0	0	0	0
0	0	0	0	0	0	0
0	0	0	0	0	0	0
0	0	0	0	0	0	0
0	0	0	0	0	0	0
0	0	0	0	0	0	0
0	0	0	0	0	0	0
0	0	0	0	0	0	0
11	0	0	0	0	0	0
0	0	0	0	0	0	0
0	0	0	0	0	0	0
0	0	0	0	0	0	0
0	0	0	0	0	0	0
0	0	0	0	0	0	0
0	0	0	0	0	0	0
0	0	0	0	0	0	0
0	0	0	0	0	0	0

0	0	0	0	0	0	0
0	0	0	0	0	0	0
0	0	0	0	0	0	0
0	0	0	0	0	0	0
0	0	0	0	0	0	0
0	0	0	0	0	0	0
0	8	0	0	0	0	0
0	0	0	0	0	0	0
0	0	0	0	0	0	0
0	0	0	0	0	0	0
0	0	0	0	0	0	0
0	0	0	0	0	0	0
0	0	0	0	0	0	0
0	0	0	0	0	0	0
0	0	0	0	0	0	0
0	0	0	0	0	0	0
0	0	0	0	0	0	0
0	0	0	0	0	0	0
0	0	0	0	0	0	0
0	0	0	0	0	0	0
0	0	0	0	0	0	0
0	0	0	0	0	0	0
0	0	0	0	0	0	0
0	0	0	0	0	0	0
0	0	0	0	0	0	0
0	11	0	0	0	0	0
0	0	0	0	0	0	0
0	0	0	0	0	0	0

T(P..Br)	T(P..I)	T(F..F)	T(F..Cl)	T(F..Br)	T(F..I)	T(Cl..Cl)
0	0	0	0	0	0	0
0	0	0	0	0	0	0
0	0	0	0	0	0	0
0	0	0	0	0	0	0
0	0	0	0	0	0	0
0	0	0	0	0	0	0
0	0	0	0	0	0	0
0	0	0	0	0	0	0
0	0	0	0	0	0	0
0	0	0	0	0	0	0
0	0	0	0	0	0	0
0	0	0	0	0	0	0
0	0	0	0	0	0	0
0	0	0	0	0	0	0
0	0	0	0	0	0	0
0	0	0	0	0	0	0
0	0	0	0	0	0	0
0	0	132	0	0	0	0
0	0	0	0	0	0	0
0	0	0	0	0	0	0
0	0	0	0	0	0	0
0	0	0	0	0	0	0
0	0	0	0	0	0	0
0	0	0	0	0	0	0
0	0	0	0	0	0	0
0	0	0	0	0	0	0
0	0	0	0	0	0	0
0	0	0	0	0	0	0
0	0	0	0	0	0	0
0	0	0	0	0	0	0
0	0	0	0	0	0	0
0	0	0	0	0	0	0
0	0	0	0	0	0	0
0	0	0	0	0	0	0
0	0	0	0	0	0	0
0	0	0	0	0	0	0
0	0	0	0	0	0	0
0	0	0	0	0	0	0
0	0	0	0	0	0	0
0	0	0	0	0	0	0
0	0	0	0	0	0	0
0	0	0	0	0	0	0
0	0	0	0	0	0	0
0	0	0	0	0	0	0
0	0	0	0	0	0	0
0	0	0	0	0	0	0
0	0	0	0	0	0	0
0	0	0	0	0	0	0
0	0	0	0	0	0	0

0	0	0	0	0	0	0
0	0	0	0	0	0	0
0	0	0	0	0	0	0
0	0	0	0	0	0	0
0	0	0	0	0	0	0
0	0	0	0	0	0	0
0	0	0	0	0	0	0
0	0	0	0	0	0	0
0	0	0	0	0	0	0
0	0	0	0	0	0	0
0	0	0	0	0	0	0
0	0	0	0	0	0	0
0	0	0	0	0	0	0
0	0	0	0	0	0	0
0	0	0	0	0	0	0
0	0	0	0	0	0	0
0	0	0	0	0	0	0
0	0	0	0	0	0	0
0	0	0	0	0	0	0
0	0	0	0	0	0	0
0	0	0	0	0	0	0
0	0	0	0	0	0	0
0	0	0	0	0	0	0
0	0	0	0	0	0	0
0	0	0	0	0	0	0
0	0	0	0	0	0	0
0	0	0	0	0	0	0
0	0	0	0	0	0	0
0	0	0	0	0	0	0
0	0	0	0	0	0	0
0	0	0	0	0	0	0
0	0	0	0	0	0	0
0	0	0	0	0	0	0
0	0	0	0	0	0	4
0	0	0	0	0	0	0
0	0	0	0	0	0	6
0	0	0	0	0	0	0
0	0	0	0	0	0	0
0	0	0	0	0	0	0
0	0	0	0	0	0	0
0	0	0	0	0	0	4
0	0	0	0	0	0	4
0	0	0	0	0	0	0
0	0	4	0	0	0	0
0	0	0	0	0	0	0
0	0	0	0	0	0	0
0	0	0	0	0	0	0
0	0	0	0	0	0	0
0	0	0	0	0	0	0
0	0	0	0	0	0	0

0	0	0	0	0	0	0
0	0	0	0	0	0	0
0	0	0	0	0	0	0
0	0	0	0	0	0	0
0	0	0	0	0	0	0
0	0	0	0	0	0	0
0	0	0	0	0	0	0
0	0	0	0	0	0	0
0	0	0	0	0	0	0
0	0	0	0	0	0	0
0	0	0	0	0	0	0
0	0	0	0	0	0	0
0	0	0	0	0	0	0
0	0	99	0	0	0	0
0	0	4	0	0	0	0
0	0	0	0	0	0	0
0	0	0	0	0	0	0
0	0	0	0	0	0	0
0	0	0	0	0	0	0
0	0	0	0	0	0	0
0	0	0	0	0	0	0
0	0	18	0	0	0	0
0	0	0	0	0	0	0
0	0	0	0	0	0	0
0	0	0	0	0	0	0
0	0	0	0	0	0	0
0	0	0	0	0	0	0
0	0	0	0	0	0	0
0	0	0	0	0	0	0
0	0	0	0	0	0	0
0	0	0	0	0	0	0
0	0	0	0	0	0	0
0	0	0	33	0	0	0
0	0	0	0	0	0	0
0	0	0	0	0	0	0
0	0	0	0	0	0	0
0	0	0	0	0	0	0
0	0	0	0	0	0	0
0	0	0	0	0	0	0
0	0	0	0	0	0	0
0	0	0	0	0	0	0
0	0	0	0	0	0	0
0	0	0	0	0	0	0
0	0	0	0	0	0	0
0	0	0	0	0	0	0
0	0	0	0	0	0	0
0	0	0	0	0	0	0
0	0	0	0	0	0	0
0	0	0	0	0	0	0
0	0	0	0	0	0	0

0	0	0	0	0	0	0
0	0	0	0	0	0	0
0	0	4	0	0	0	0
0	0	0	0	0	0	7
0	0	0	0	0	0	0
0	0	0	0	0	0	0
0	0	0	0	0	0	6
0	0	0	0	0	0	0
0	0	0	0	0	0	0
0	0	0	0	0	0	0
0	0	0	0	0	0	0
0	0	0	0	0	0	0
0	0	0	0	0	0	0
0	0	0	0	0	0	0
0	0	0	0	0	0	0
0	0	0	0	0	0	0
0	0	0	0	0	0	0
0	0	0	0	0	0	0
0	0	0	0	0	0	0
0	0	0	7	0	0	0
0	0	0	0	0	0	0
0	0	0	0	0	0	0
0	0	0	0	0	0	0
0	0	0	0	0	0	0
0	0	0	0	0	0	0
0	0	0	0	0	0	0
0	0	0	0	0	0	0
0	0	0	0	0	0	0
0	0	0	0	0	0	0
0	0	0	0	0	0	0
0	0	0	0	0	0	0
0	0	0	0	0	0	0
0	0	0	0	0	0	0
0	0	0	0	0	0	0
0	0	0	0	0	0	0
0	0	0	0	0	0	0
0	0	0	0	0	0	0
0	0	0	0	0	0	0
0	0	0	0	0	0	0
0	0	0	0	0	0	0
0	0	0	0	0	0	0
0	0	0	0	0	0	0
0	0	0	0	0	0	0
0	0	0	0	0	0	0
0	0	0	0	0	0	0
0	0	0	0	0	0	0
0	0	0	0	0	0	0
0	0	0	0	0	0	0
0	0	0	0	0	0	0
0	0	0	0	0	0	0

0	0	0	0	0	0	0
0	0	0	0	0	0	0
0	0	0	0	0	0	0
0	0	0	0	0	0	0
0	0	0	0	0	0	0
0	0	0	0	0	0	0
0	0	0	0	0	0	0
0	0	0	0	0	0	0
0	0	0	0	0	0	0
0	0	0	0	0	0	0
0	0	0	0	0	0	0
0	0	0	0	0	0	0
0	0	0	0	0	0	0
0	0	0	0	0	0	0
0	0	0	0	0	0	0
0	0	0	0	0	0	0
0	0	0	0	0	0	0
0	0	0	0	0	0	0
0	0	0	0	0	0	0
0	0	0	0	0	0	0
0	0	0	0	0	0	0
0	0	0	0	0	0	0
0	0	0	0	0	0	0
0	0	0	0	0	0	0
0	0	0	0	0	0	0
0	0	0	0	0	0	0
0	0	0	0	0	0	0
0	0	0	0	0	0	0
0	0	0	0	0	0	0
0	0	0	0	0	0	0
0	0	0	0	0	0	0
0	0	0	0	0	0	0
0	0	0	0	0	0	0
0	0	0	0	0	0	0
0	0	0	0	0	0	0
0	0	0	0	0	0	0
0	0	0	0	0	0	0
0	0	0	0	0	0	0
0	0	0	0	0	0	0
0	0	0	0	0	0	0
0	0	0	0	0	0	0
0	0	0	0	0	0	0
0	0	0	0	0	0	0
0	0	0	0	0	0	0
0	0	0	0	0	0	0
0	0	0	0	0	0	0
0	0	0	0	0	0	0
0	0	0	0	0	0	0
0	0	0	0	0	0	0
0	0	0	0	0	0	0

0	0	0	0	0	0	0
0	0	0	0	0	0	0
0	0	0	0	0	0	0
0	0	0	0	0	0	0
0	0	0	0	0	0	0
0	0	0	0	0	0	0
0	0	0	0	0	0	0
0	0	0	0	0	0	0
0	0	0	0	0	0	0
0	0	0	0	0	0	0
0	0	0	0	0	0	0
0	0	0	0	0	0	0
0	0	0	0	0	0	0
0	0	0	0	0	0	7
0	0	0	0	0	0	0
0	0	0	0	0	0	0
0	0	0	0	0	0	0
0	0	0	0	0	0	0
0	0	0	0	0	0	0
0	0	0	0	0	0	0
0	0	0	0	0	0	0
0	0	0	0	0	0	0
0	0	0	0	0	0	0
0	0	0	0	0	0	0
0	0	0	0	0	0	0
0	0	0	0	0	0	0
0	0	0	0	0	0	0
0	0	0	0	0	0	0

T(Cl..Br)	T(Cl..I)	T(Br..Br)	T(Br..I)	T(I..I)	MWC01	MWC02
0	0	0	0	0	17	4.394
0	0	0	0	0	24	4.71
0	0	0	0	0	28	4.92
0	0	0	0	0	11	3.932
0	0	0	0	0	17	4.394
0	0	0	0	0	13	4.234
0	0	0	0	0	32	5.03
0	0	0	0	0	11	3.97
0	0	0	0	0	25	4.812
0	0	0	0	0	18	4.394
0	0	0	0	0	42	5.333
0	0	0	0	4	33	5.056
0	0	0	0	0	23	4.691
0	0	0	0	0	29	4.92
0	0	0	0	0	27	4.934
0	0	0	0	0	26	4.89
0	0	0	0	0	15	4.29
0	0	0	0	0	40	5.303
0	0	0	0	0	19	4.466
0	0	0	0	0	23	4.71
0	0	0	0	0	24	4.796
0	0	0	0	0	21	4.635
0	0	0	0	0	23	4.727
0	0	0	0	0	25	4.844
0	0	0	0	0	29	4.89
0	0	0	0	0	31	5.13
0	0	0	0	0	23	4.654
0	0	0	0	0	46	5.425
0	0	0	0	0	24	4.745
0	0	0	0	0	23	4.635
0	0	0	0	0	42	5.323
0	0	0	0	0	49	5.549
0	0	0	0	0	20	4.595
0	0	0	0	0	22	4.635
0	0	0	0	0	40	5.398
0	0	0	0	0	31	5.004
0	0	0	0	0	28	4.977
0	0	0	0	0	15	4.344
0	0	0	0	0	32	5.081
0	0	0	0	0	30	5.043
0	0	0	0	0	37	5.187
0	0	0	0	0	20	4.575
0	0	0	0	0	45	5.493
0	0	0	0	0	21	4.654
0	0	0	0	0	33	5.043
0	0	0	0	0	26	4.86
0	0	0	0	0	27	4.905
0	0	0	0	0	34	5.118
0	0	0	0	0	32	5.056

0	0	0	0	0	35	5.165
0	0	0	0	0	32	5.056
0	0	0	0	0	48	5.468
0	0	0	0	0	32	5.056
0	0	0	0	0	30	5.004
0	0	0	0	0	29	4.963
0	0	0	0	0	39	5.273
0	0	0	0	0	40	5.293
0	0	0	0	0	27	4.89
0	0	0	0	0	39	5.263
0	0	0	0	0	31	5.017
0	0	0	0	0	28	4.963
0	0	0	0	0	26	4.86
0	0	0	0	0	31	5.017
0	0	0	0	0	28	4.92
0	0	0	0	0	26	4.86
0	0	0	0	0	26	4.779
0	0	0	0	0	29	4.905
0	0	0	0	0	18	4.554
0	0	0	0	0	20	4.533
0	0	0	0	0	23	4.71
0	0	0	0	0	17	4.394
0	0	0	0	0	23	4.71
0	0	0	0	0	24	4.828
0	0	0	0	0	20	4.595
0	0	0	0	0	17	4.317
0	0	0	0	0	27	4.844
0	0	0	0	0	28	4.905
0	0	0	0	0	21	4.654
0	0	0	0	0	27	4.905
0	0	0	0	0	28	4.92
0	0	0	0	0	14	4.234
0	0	0	0	0	27	4.89
0	0	0	0	0	15	4.263
0	0	0	0	0	26	4.844
0	0	0	0	0	14	4.174
0	0	0	0	0	18	4.489
0	0	0	0	0	22	4.635
0	0	0	0	0	31	5.13
0	0	0	0	0	22	4.673
0	0	0	0	0	20	4.554
0	0	0	0	0	33	5.142
0	0	0	0	0	19	4.533
0	0	0	0	0	19	4.533
0	0	0	0	0	31	5.106
0	0	0	0	0	61	5.753
0	0	0	0	0	31	5.004
0	0	0	0	0	20	4.511
0	0	0	0	0	26	4.828
0	0	0	0	0	63	5.79

0	0	0	0	0	30	4.977
0	0	0	0	0	34	5.118
0	0	0	0	0	35	5.22
0	0	0	0	0	22	4.654
0	0	0	0	0	13	4.007
0	0	0	0	0	23	4.745
0	0	0	0	0	48	5.493
0	0	0	0	0	20	4.554
0	0	0	0	0	20	4.533
0	0	0	0	0	19	4.489
0	0	0	0	0	27	4.828
0	0	0	0	0	24	4.745
0	0	0	0	0	30	5.069
0	0	0	0	0	29	4.949
0	0	0	0	0	24	4.762
0	0	0	0	0	20	4.595
0	0	0	0	0	24	4.779
0	0	0	0	0	9	3.761
0	0	0	0	0	33	5.081
0	0	0	0	0	19	4.511
0	0	0	0	0	6	3.434
0	0	0	0	0	44	5.389
0	0	0	0	0	22	4.691
0	0	0	0	0	40	5.342
0	0	0	0	0	12	4.043
0	0	0	0	0	24	4.812
0	0	0	0	0	19	4.511
0	0	0	0	0	18	4.466
0	0	0	0	0	33	5.106
0	0	0	0	0	36	5.165
0	0	0	0	0	35	5.118
0	0	0	0	0	26	4.86
0	0	0	0	0	29	4.934
0	0	0	0	0	18	4.554
0	0	0	0	0	25	4.89
0	0	0	0	0	15	4.263
0	0	0	0	0	41	5.273
0	0	0	0	0	27	4.89
0	0	0	0	0	23	4.727
0	0	0	0	0	36	5.165
0	0	0	0	0	17	4.419
0	0	0	0	0	14	4.234
0	0	0	0	0	29	4.963
0	0	0	0	0	22	4.654
0	0	0	0	0	20	4.554
0	0	0	0	0	16	4.344
0	0	0	0	0	27	4.89
0	0	0	0	0	24	4.727
0	0	0	0	0	12	4.043
0	0	0	0	0	29	4.99

0	0	0	0	0	17	4.369
0	0	0	0	0	26	4.828
0	0	0	0	0	27	4.905
0	0	0	0	0	23	4.727
0	0	0	0	0	33	5.056
0	0	0	0	0	25	4.844
0	0	0	0	0	19	4.466
0	0	0	0	0	19	4.511
0	0	0	0	0	23	4.727
0	0	0	0	0	19	4.511
0	0	0	0	0	11	3.97
0	0	0	0	0	8	3.611
0	0	0	0	0	24	4.745
0	0	0	0	0	28	4.963
0	0	0	0	0	35	5.13
0	0	0	0	0	30	5.081
0	0	0	0	0	20	4.533
0	0	0	0	0	19	4.443
0	0	0	0	0	12	4.043
0	0	0	0	0	26	4.86
0	0	0	0	0	45	5.398
0	0	0	0	0	25	4.89
0	0	0	0	0	33	5.118
0	0	0	0	0	20	4.554
0	0	0	0	0	23	4.727
0	0	0	0	0	32	5.094
0	0	0	0	0	18	4.489
0	0	0	0	0	30	5.081
0	0	0	0	0	27	4.963
0	0	0	0	0	18	4.466
0	0	0	0	0	16	4.369
0	0	0	0	0	35	5.176
0	0	0	0	0	26	4.844
0	0	0	0	0	22	4.673
0	0	0	0	0	27	4.875
0	0	0	0	0	18	4.466
0	0	0	0	0	12	4.043
0	0	0	0	0	25	4.812
0	0	0	0	0	22	4.635
0	0	0	0	0	47	5.46
0	0	0	0	0	29	4.99
0	0	0	0	0	26	4.844
0	0	0	0	0	24	4.779
0	0	0	0	0	31	5.069
0	0	0	0	0	22	4.673
0	0	0	0	0	27	4.977
0	0	0	0	0	26	4.828
0	0	0	0	0	46	5.476
0	0	0	0	0	16	4.369
0	0	0	0	0	13	4.078

0	0	0	0	0	20	4.554
0	0	0	0	0	18	4.489
0	0	0	0	0	25	4.779
0	0	0	0	0	21	4.635
0	0	0	0	0	19	4.489
0	0	0	0	0	35	5.142
0	0	0	0	0	29	4.949
0	0	0	0	0	27	4.89
0	0	0	0	0	18	4.466
0	0	0	0	0	19	4.466
0	0	0	0	0	31	5.004
0	0	0	0	0	31	5.017
0	0	0	0	0	29	5.043
0	0	0	0	0	26	4.92
0	0	0	0	0	17	4.344
0	0	0	0	0	17	4.344
0	0	0	0	0	26	4.762
0	0	0	0	0	20	4.533
0	0	0	0	0	13	4.143
0	0	0	0	0	20	4.533
0	0	0	0	0	18	4.466
0	0	0	0	0	30	4.949
0	0	0	0	0	27	4.89
0	0	0	0	0	27	4.89
0	0	0	0	0	21	4.554
0	0	0	0	0	35	5.106
0	0	0	0	0	16	4.394
0	0	0	0	0	17	4.489
0	0	0	0	0	34	5.118
0	0	0	0	0	22	4.654
0	0	0	0	0	43	5.398
0	0	0	0	0	21	4.595
0	0	0	0	0	10	3.85
0	0	0	0	0	25	4.828
0	0	0	0	0	36	5.187
0	0	0	0	0	18	4.466
0	0	0	0	0	29	4.92
0	0	0	0	0	18	4.489
0	0	0	0	0	24	4.762
0	0	0	0	0	32	5.03
0	0	0	0	0	19	4.554
0	0	0	0	0	27	4.89
0	0	0	0	0	24	4.762
0	0	0	0	0	21	4.635
0	0	0	0	0	19	4.511
0	0	0	0	0	29	4.92
0	0	0	0	0	23	4.654
0	0	0	0	0	23	4.727
0	0	0	0	0	20	4.595
0	0	0	0	0	24	4.796

0	0	0	0	0	31	5.017
0	0	0	0	0	16	4.369
0	0	0	0	0	24	4.844
0	0	0	0	0	35	5.231
0	0	0	0	0	14	4.263
0	0	0	0	0	16	4.369
0	0	0	0	0	19	4.511
0	0	0	0	0	22	4.654
0	0	0	0	0	26	4.779
0	0	0	0	0	22	4.654
0	0	0	0	0	18	4.443
0	0	0	0	0	20	4.575
0	0	0	0	0	29	4.934
0	0	0	0	0	26	4.86
0	0	0	0	0	22	4.654
0	0	0	0	0	30	4.963
0	0	0	0	0	9	3.664
0	0	0	0	0	34	5.081
0	0	0	0	0	45	5.442
0	0	0	0	0	34	5.081
0	0	0	0	0	65	5.826
0	0	0	0	0	30	5.03
0	0	0	0	0	25	4.796
0	0	0	0	0	25	4.796
0	0	0	0	0	20	4.575
0	0	0	0	0	32	5.056
0	0	0	0	0	25	4.812
0	0	0	0	0	21	4.635

MWC03	MWC04	MWC05	MWC06	MWC07	MWC08	MWC09
5.209	6.084	6.938	7.825	8.696	9.587	10.466
5.485	6.319	7.125	7.966	8.789	9.633	10.466
5.778	6.673	7.556	8.459	9.352	10.258	11.156
4.673	5.485	6.252	7.064	7.843	8.652	9.437
5.231	6.107	6.974	7.864	8.744	9.639	10.525
5.056	5.984	6.855	7.784	8.666	9.595	10.481
5.889	6.777	7.658	8.556	9.445	10.347	11.239
4.812	5.673	6.529	7.394	8.253	9.119	9.979
5.694	6.6	7.503	8.417	9.329	10.246	11.161
5.153	5.958	6.746	7.556	8.357	9.169	9.976
6.205	7.098	7.988	8.886	9.783	10.684	11.585
5.911	6.792	7.673	8.568	9.461	10.364	11.265
5.533	6.405	7.274	8.159	9.04	9.929	10.817
5.771	6.663	7.555	8.468	9.376	10.297	11.211
5.82	6.76	7.675	8.622	9.55	10.502	11.44
5.778	6.718	7.634	8.582	9.513	10.466	11.406
5.142	6.023	6.895	7.783	8.661	9.551	10.432
6.146	7.052	7.921	8.825	9.705	10.608	11.493
5.209	6.019	6.788	7.596	8.379	9.187	9.978
5.565	6.441	7.309	8.19	9.061	9.944	10.819
5.694	6.629	7.561	8.505	9.448	10.396	11.343
5.501	6.392	7.276	8.169	9.058	9.952	10.842
5.58	6.46	7.334	8.219	9.101	9.99	10.877
5.72	6.661	7.569	8.519	9.445	10.401	11.339
5.694	6.526	7.352	8.195	9.031	9.879	10.722
6.094	7.084	8.072	9.068	10.063	11.062	12.06
5.425	6.232	7.022	7.829	8.627	9.435	10.238
6.293	7.195	8.085	8.993	9.893	10.804	11.709
5.595	6.479	7.362	8.261	9.16	10.066	10.973
5.371	6.159	6.919	7.708	8.481	9.273	10.054
6.168	7.059	7.927	8.825	9.704	10.605	11.491
6.482	7.451	8.408	9.381	10.348	11.323	12.294
5.416	6.308	7.159	8.052	8.916	9.809	10.681
5.46	6.312	7.159	8.018	8.872	9.734	10.591
6.375	7.39	8.4	9.423	10.446	11.476	12.506
5.849	6.727	7.586	8.471	9.335	10.223	11.091
5.905	6.864	7.82	8.788	9.755	10.729	11.702
5.242	6.159	7.072	7.992	8.908	9.828	10.744
5.932	6.845	7.717	8.634	9.52	10.44	11.334
5.969	6.911	7.853	8.8	9.748	10.699	11.649
6.061	6.959	7.856	8.762	9.666	10.574	11.481
5.442	6.337	7.228	8.128	9.025	9.927	10.826
6.418	7.401	8.359	9.347	10.32	11.312	12.293
5.525	6.428	7.323	8.232	9.136	10.048	10.957
5.889	6.756	7.623	8.502	9.381	10.267	11.153
5.74	6.65	7.555	8.474	9.391	10.316	11.239
5.784	6.696	7.599	8.518	9.433	10.357	11.278
5.994	6.895	7.793	8.702	9.612	10.528	11.445
5.922	6.825	7.716	8.627	9.532	10.45	11.364

6.043	6.967	7.87	8.8	9.713	10.646	11.567
5.922	6.83	7.726	8.644	9.556	10.48	11.4
6.347	7.251	8.148	9.057	9.963	10.876	11.789
5.922	6.832	7.731	8.65	9.564	10.49	11.412
5.883	6.812	7.73	8.674	9.611	10.566	11.515
5.838	6.746	7.65	8.568	9.484	10.409	11.332
6.142	7.062	7.955	8.881	9.785	10.715	11.627
6.159	7.066	7.958	8.871	9.776	10.695	11.608
5.765	6.676	7.578	8.496	9.408	10.329	11.248
6.138	7.047	7.946	8.859	9.769	10.686	11.602
5.883	6.79	7.689	8.607	9.522	10.446	11.37
5.808	6.72	7.594	8.507	9.393	10.306	11.2
5.74	6.65	7.555	8.474	9.391	10.316	11.239
5.883	6.786	7.678	8.592	9.5	10.422	11.34
5.778	6.683	7.575	8.492	9.402	10.327	11.248
5.74	6.65	7.555	8.474	9.391	10.316	11.239
5.595	6.431	7.265	8.112	8.954	9.807	10.653
5.72	6.569	7.41	8.271	9.126	9.994	10.858
5.407	6.347	7.226	8.165	9.051	9.989	10.877
5.333	6.172	7	7.849	8.69	9.542	10.389
5.557	6.444	7.323	8.222	9.116	10.021	10.922
5.176	6.033	6.845	7.711	8.535	9.407	10.236
5.557	6.444	7.323	8.222	9.116	10.021	10.922
5.714	6.658	7.569	8.515	9.435	10.381	11.306
5.468	6.361	7.253	8.153	9.053	9.956	10.859
5.081	5.866	6.648	7.439	8.227	9.021	9.813
5.673	6.529	7.376	8.238	9.091	9.955	10.813
5.733	6.605	7.453	8.325	9.18	10.05	10.909
5.541	6.454	7.363	8.281	9.197	10.117	11.035
5.796	6.711	7.623	8.546	9.467	10.394	11.319
5.778	6.668	7.549	8.444	9.332	10.227	11.118
5.017	5.872	6.686	7.536	8.36	9.209	10.036
5.759	6.666	7.566	8.484	9.398	10.322	11.244
5.081	5.927	6.763	7.612	8.453	9.304	10.147
5.707	6.597	7.482	8.378	9.271	10.171	11.068
4.977	5.832	6.671	7.536	8.386	9.254	10.107
5.323	6.201	7.06	7.943	8.811	9.698	10.569
5.485	6.354	7.232	8.117	9.005	9.896	10.788
6.094	7.084	8.072	9.068	10.063	11.062	12.06
5.533	6.418	7.302	8.196	9.087	9.984	10.878
5.371	6.229	7.069	7.931	8.781	9.644	10.497
6.047	7.002	7.929	8.887	9.823	10.781	11.723
5.407	6.301	7.194	8.095	8.994	9.898	10.8
5.371	6.248	7.106	7.986	8.85	9.731	10.598
6.043	7.011	7.971	8.944	9.913	10.89	11.865
6.661	7.6	8.525	9.47	10.404	11.353	12.296
5.849	6.735	7.612	8.509	9.401	10.304	11.204
5.303	6.129	6.948	7.786	8.618	9.461	10.3
5.68	6.567	7.448	8.349	9.243	10.15	11.051
6.693	7.653	8.591	9.562	10.52	11.499	12.469

5.74	6.605	7.383	8.245	9.032	9.889	10.682
5.994	6.895	7.786	8.691	9.589	10.495	11.396
6.163	7.14	8.105	9.085	10.06	11.042	12.021
5.485	6.347	7.198	8.064	8.922	9.788	10.649
4.727	5.46	6.192	6.929	7.664	8.402	9.139
5.638	6.572	7.494	8.437	9.369	10.315	11.251
6.405	7.338	8.268	9.208	10.147	11.09	12.034
5.293	6.146	6.903	7.757	8.523	9.375	10.147
5.333	6.163	6.986	7.822	8.654	9.494	10.331
5.293	6.129	6.952	7.793	8.621	9.464	10.295
5.652	6.497	7.339	8.192	9.041	9.898	10.751
5.58	6.454	7.317	8.201	9.077	9.966	10.85
5.969	6.932	7.857	8.822	9.759	10.726	11.67
5.727	6.597	7.401	8.268	9.087	9.953	10.782
5.602	6.494	7.353	8.255	9.123	10.031	10.903
5.476	6.382	7.284	8.198	9.109	10.026	10.942
5.659	6.569	7.476	8.395	9.31	10.232	11.151
4.533	5.352	6.146	6.963	7.764	8.579	9.383
5.932	6.823	7.698	8.596	9.483	10.385	11.278
5.342	6.201	7.052	7.916	8.774	9.641	10.502
4.111	4.934	5.666	6.475	7.222	8.026	8.777
6.248	7.152	8.045	8.96	9.871	10.795	11.718
5.517	6.409	7.259	8.153	9.012	9.909	10.772
6.256	7.226	8.179	9.163	10.134	11.125	12.107
4.828	5.694	6.506	7.377	8.197	9.068	9.891
5.727	6.678	7.624	8.586	9.543	10.509	11.471
5.352	6.225	7.089	7.972	8.848	9.736	10.618
5.252	6.094	6.905	7.74	8.558	9.391	10.212
5.974	6.878	7.763	8.668	9.558	10.463	11.356
5.999	6.876	7.724	8.603	9.457	10.337	11.194
5.938	6.801	7.636	8.502	9.341	10.208	11.051
5.74	6.642	7.536	8.441	9.339	10.243	11.143
5.733	6.594	7.421	8.284	9.125	9.99	10.839
5.407	6.347	7.226	8.165	9.051	9.989	10.877
5.849	6.834	7.818	8.811	9.802	10.798	11.791
5.043	5.878	6.683	7.52	8.336	9.173	9.994
6.107	6.965	7.812	8.671	9.522	10.381	11.235
5.759	6.666	7.566	8.484	9.398	10.322	11.244
5.595	6.482	7.367	8.26	9.152	10.049	10.945
6.043	6.944	7.844	8.755	9.664	10.578	11.491
5.252	6.146	7.015	7.917	8.802	9.706	10.6
5.081	5.974	6.849	7.747	8.633	9.532	10.424
5.838	6.756	7.66	8.588	9.503	10.436	11.356
5.476	6.326	7.168	8.02	8.868	9.723	10.574
5.38	6.229	7.074	7.928	8.78	9.637	10.491
5.176	6.043	6.895	7.767	8.625	9.499	10.359
5.72	6.605	7.456	8.341	9.201	10.084	10.95
5.572	6.435	7.299	8.172	9.045	9.923	10.8
4.86	5.714	6.553	7.408	8.253	9.107	9.955
5.889	6.819	7.743	8.681	9.615	10.557	11.496

5.165	5.999	6.815	7.652	8.476	9.315	10.144
5.666	6.541	7.402	8.283	9.154	10.039	10.916
5.79	6.711	7.618	8.546	9.462	10.393	11.313
5.587	6.479	7.363	8.26	9.154	10.053	10.95
5.916	6.797	7.677	8.566	9.453	10.344	11.234
5.727	6.65	7.554	8.481	9.395	10.322	11.242
5.252	6.075	6.891	7.719	8.544	9.375	10.204
5.361	6.244	7.13	8.026	8.924	9.825	10.726
5.609	6.515	7.419	8.333	9.244	10.16	11.074
5.342	6.201	7.047	7.907	8.758	9.619	10.472
4.812	5.673	6.529	7.394	8.253	9.119	9.979
4.29	5.069	5.784	6.555	7.284	8.05	8.785
5.602	6.494	7.392	8.3	9.212	10.127	11.043
5.861	6.81	7.733	8.687	9.621	10.578	11.519
5.958	6.828	7.676	8.547	9.404	10.276	11.138
6.023	7.004	7.969	8.955	9.927	10.915	11.891
5.342	6.18	7.015	7.86	8.704	9.554	10.403
5.187	5.979	6.751	7.546	8.331	9.128	9.92
4.86	5.72	6.569	7.434	8.291	9.156	10.016
5.746	6.658	7.565	8.483	9.398	10.318	11.237
6.24	7.133	7.993	8.885	9.752	10.644	11.515
5.849	6.834	7.818	8.811	9.802	10.798	11.791
6.028	6.963	7.894	8.836	9.774	10.718	11.659
5.342	6.201	7.027	7.892	8.737	9.605	10.46
5.533	6.412	7.246	8.124	8.972	9.848	10.705
5.994	6.942	7.868	8.822	9.759	10.714	11.657
5.352	6.252	7.143	8.048	8.946	9.852	10.753
6.047	7.04	8.031	9.032	10.033	11.039	12.044
5.927	6.921	7.915	8.919	9.923	10.931	11.938
5.303	6.168	7.027	7.897	8.764	9.636	10.506
5.153	6.019	6.834	7.695	8.523	9.381	10.216
6.08	7.017	7.944	8.889	9.828	10.779	11.725
5.714	6.624	7.527	8.449	9.362	10.287	11.204
5.468	6.344	7.166	8.043	8.88	9.756	10.602
5.733	6.635	7.526	8.438	9.341	10.259	11.167
5.283	6.142	6.978	7.835	8.677	9.532	10.378
4.844	5.673	6.5	7.332	8.164	8.998	9.831
5.687	6.589	7.488	8.398	9.306	10.219	11.131
5.485	6.354	7.232	8.117	9.005	9.896	10.788
6.326	7.238	8.128	9.042	9.942	10.857	11.761
5.889	6.819	7.743	8.681	9.615	10.557	11.496
5.707	6.592	7.47	8.357	9.241	10.129	11.015
5.673	6.586	7.501	8.423	9.345	10.272	11.2
5.969	6.917	7.84	8.793	9.728	10.683	11.625
5.525	6.409	7.287	8.177	9.064	9.957	10.848
5.948	6.946	7.944	8.951	9.958	10.967	11.977
5.694	6.589	7.487	8.399	9.312	10.232	11.152
6.385	7.345	8.287	9.255	10.211	11.183	12.146
5.242	6.151	7.047	7.964	8.867	9.786	10.692
4.828	5.638	6.415	7.231	8.019	8.835	9.63

5.416	6.301	7.192	8.092	8.993	9.899	10.803
5.371	6.282	7.186	8.106	9.017	9.94	10.854
5.645	6.538	7.438	8.35	9.264	10.184	11.105
5.517	6.435	7.349	8.279	9.206	10.139	11.071
5.293	6.138	6.969	7.82	8.664	9.519	10.371
6.004	6.893	7.779	8.675	9.571	10.473	11.374
5.814	6.708	7.595	8.497	9.393	10.298	11.2
5.733	6.632	7.508	8.415	9.305	10.217	11.113
5.303	6.168	7.024	7.892	8.754	9.625	10.489
5.209	6.019	6.788	7.596	8.379	9.187	9.978
5.826	6.703	7.553	8.441	9.306	10.2	11.076
5.889	6.777	7.662	8.555	9.445	10.339	11.232
5.984	6.959	7.923	8.903	9.874	10.856	11.83
5.844	6.804	7.747	8.711	9.662	10.627	11.581
5.118	5.916	6.708	7.512	8.31	9.116	9.918
5.118	5.916	6.708	7.512	8.31	9.116	9.918
5.549	6.361	7.169	7.991	8.809	9.637	10.462
5.333	6.172	6.999	7.844	8.684	9.534	10.38
4.963	5.826	6.668	7.533	8.382	9.247	10.097
5.333	6.172	6.999	7.844	8.684	9.534	10.38
5.303	6.184	7.043	7.929	8.796	9.683	10.552
5.796	6.663	7.535	8.416	9.3	10.189	11.08
5.778	6.681	7.585	8.494	9.404	10.315	11.226
5.778	6.681	7.585	8.494	9.404	10.315	11.226
5.303	6.116	6.887	7.703	8.484	9.302	10.09
5.932	6.801	7.652	8.529	9.388	10.266	11.13
5.198	6.107	6.934	7.84	8.679	9.58	10.428
5.323	6.244	7.119	8.04	8.928	9.85	10.744
5.994	6.889	7.777	8.675	9.568	10.467	11.361
5.485	6.351	7.206	8.079	8.946	9.824	10.697
6.315	7.272	8.219	9.184	10.143	11.113	12.078
5.425	6.282	7.132	7.994	8.849	9.713	10.57
4.635	5.46	6.263	7.091	7.899	8.727	9.536
5.727	6.648	7.566	8.497	9.426	10.364	11.3
6.066	6.98	7.883	8.804	9.715	10.639	11.554
5.22	6.084	6.866	7.731	8.525	9.389	10.192
5.765	6.645	7.517	8.41	9.293	10.193	11.081
5.303	6.184	7.02	7.903	8.746	9.63	10.477
5.602	6.485	7.345	8.233	9.101	9.991	10.862
5.894	6.779	7.667	8.566	9.467	10.375	11.283
5.389	6.275	7.132	8.019	8.882	9.77	10.635
5.746	6.645	7.532	8.439	9.339	10.251	11.159
5.595	6.472	7.326	8.204	9.063	9.941	10.803
5.451	6.337	7.182	8.072	8.931	9.824	10.691
5.342	6.192	7.04	7.893	8.745	9.6	10.454
5.727	6.589	7.419	8.283	9.123	9.987	10.832
5.46	6.293	7.128	7.979	8.83	9.691	10.552
5.595	6.488	7.378	8.28	9.178	10.083	10.985
5.407	6.297	7.136	8.025	8.879	9.767	10.631
5.68	6.6	7.506	8.431	9.348	10.277	11.201

5.889	6.777	7.662	8.555	9.445	10.339	11.232
5.13	6.004	6.79	7.658	8.456	9.319	10.125
5.771	6.727	7.674	8.632	9.584	10.544	11.498
6.172	7.151	8.115	9.097	10.071	11.054	12.032
5.153	6.057	6.961	7.867	8.774	9.681	10.587
5.242	6.151	7.047	7.964	8.867	9.786	10.692
5.352	6.213	7.067	7.931	8.79	9.654	10.515
5.442	6.301	7.114	7.973	8.799	9.657	10.491
5.565	6.375	7.175	7.988	8.793	9.607	10.414
5.451	6.308	7.124	7.987	8.809	9.676	10.503
5.22	6.071	6.878	7.74	8.56	9.43	10.256
5.416	6.304	7.18	8.076	8.965	9.864	10.759
5.784	6.653	7.516	8.389	9.257	10.132	11.003
5.746	6.658	7.565	8.483	9.398	10.318	11.237
5.493	6.365	7.222	8.096	8.959	9.833	10.698
5.808	6.668	7.527	8.39	9.252	10.117	10.98
4.369	5.13	5.872	6.635	7.387	8.148	8.904
5.922	6.795	7.658	8.537	9.408	10.29	11.164
6.347	7.299	8.237	9.199	10.152	11.12	12.081
5.927	6.795	7.66	8.534	9.406	10.283	11.16
6.779	7.769	8.754	9.758	10.757	11.767	12.772
5.964	6.927	7.89	8.867	9.842	10.824	11.802
5.652	6.547	7.431	8.334	9.229	10.136	11.036
5.666	6.558	7.446	8.345	9.238	10.139	11.036
5.425	6.308	7.185	8.074	8.958	9.85	10.737
5.927	6.819	7.703	8.597	9.486	10.38	11.271
5.666	6.558	7.445	8.346	9.247	10.155	11.063
5.476	6.365	7.232	8.125	9.004	9.9	10.786

MWC10	TWC	SRW01	SRW02	SRW03	SRW04	SRW05
11.357	104.557	17	34	0	126	10
11.314	119.806	24	48	0	172	0
12.064	130.217	26	56	0	216	0
10.243	85.579	11	22	0	78	0
11.422	103.9	16	34	0	126	10
11.41	97.066	14	26	0	110	0
12.143	139.082	30	64	0	240	10
10.844	87.575	10	22	0	82	10
12.081	122.843	22	50	0	194	10
10.79	104.101	18	36	0	124	0
12.488	162.049	40	84	0	328	0
12.172	141.263	31	66	0	246	10
11.709	117.559	21	46	0	170	0
12.136	133.399	28	58	0	214	0
12.395	129.698	25	54	0	230	10
12.364	127.352	24	52	0	220	10
11.322	99.098	14	30	0	114	10
12.396	156.45	37	80	0	320	10
10.786	106.409	19	38	0	134	0
11.703	117.74	21	46	0	174	10
12.292	122.663	22	48	0	192	10
11.735	113.559	19	42	0	162	20
11.769	118.057	21	46	0	178	10
12.299	124.797	23	50	0	210	10
11.574	129.863	27	58	0	206	10
13.061	140.693	28	62	0	274	10
11.046	115.51	22	46	6	162	40
12.622	170.02	43	92	0	360	40
11.885	120.525	22	48	0	180	10
10.849	115.449	23	46	0	158	0
12.393	160.494	39	84	0	324	0
13.27	176.505	43	98	0	414	30
11.574	111.509	19	40	0	156	10
11.454	115.233	21	44	0	160	0
13.539	158.952	34	80	6	360	50
11.981	135.268	28	62	0	234	10
12.679	131.218	24	56	0	240	0
11.664	100.953	14	30	0	122	10
12.257	139.761	30	64	0	256	10
12.602	135.274	26	60	0	248	10
12.391	148.938	33	74	0	282	20
11.728	111.216	18	40	0	152	0
13.289	170.232	41	90	0	394	0
11.872	114.176	19	42	0	166	10
12.045	139.66	30	66	0	242	10
12.168	126.393	24	52	0	212	0
12.206	128.776	25	54	0	222	0
12.368	143.455	31	68	0	272	10
12.288	138.781	29	64	0	256	20

12.502	146.273	32	70	0	286	20
12.328	139.941	30	64	0	256	10
12.706	173.605	44	96	0	384	10
12.342	140	30	64	0	256	10
12.477	136.273	28	60	0	244	10
12.261	133.252	27	58	0	234	0
12.561	155.001	36	78	0	318	0
12.532	156.96	37	80	0	324	10
12.172	128.563	25	54	0	218	10
12.524	154.832	36	78	0	314	10
12.3	137.625	29	62	0	246	10
12.113	130.605	26	56	0	228	10
12.168	126.393	24	52	0	212	0
12.268	136.485	28	62	0	246	10
12.178	130.604	26	56	0	224	10
12.168	126.393	24	52	0	212	0
11.509	123.104	24	52	0	184	10
11.73	130.585	27	58	0	210	0
11.815	108.433	17	36	0	152	0
11.243	109.751	19	40	0	144	0
11.829	118.144	21	46	0	174	0
11.111	103.448	17	34	0	126	0
11.829	118.144	21	46	0	174	0
12.252	122.658	22	48	0	200	10
11.765	111.465	18	40	0	156	10
10.61	101.022	17	34	0	114	10
11.678	126.199	25	54	0	198	10
11.778	128.939	26	56	0	212	0
11.955	114.596	19	42	0	166	10
12.248	128.008	24	54	6	214	40
12.014	131.05	27	56	0	216	10
10.884	95.835	14	28	0	108	0
12.172	128.501	25	54	0	210	10
10.997	97.547	14	30	0	110	10
11.97	124.488	23	52	0	200	0
10.975	95.912	14	28	0	100	0
11.458	106.553	17	36	0	140	0
11.681	115.193	20	44	0	160	0
13.061	140.693	28	62	0	274	10
11.777	115.848	20	44	0	168	0
11.36	110.435	19	40	0	148	0
12.681	143.015	30	66	0	282	20
11.705	108.925	17	38	0	146	20
11.478	108.9	18	38	0	146	0
12.844	138.587	27	62	0	266	20
13.249	200.311	54	122	0	506	20
12.109	136.727	29	62	0	234	0
11.146	109.203	19	40	0	140	0
11.96	126.276	25	52	0	196	0
13.454	207.231	58	126	0	534	0

11.535	133.087	29	60	0	228	0
12.302	142.265	30	68	0	264	20
13.004	148.839	32	70	0	298	0
11.515	116.622	22	44	0	164	0
9.876	89.396	14	26	0	82	0
12.198	120.02	21	46	0	182	10
12.98	172.962	42	96	0	388	20
10.997	109.694	20	40	0	148	10
11.173	109.488	19	40	0	144	0
11.14	107.176	18	38	0	138	0
11.611	125.81	25	54	0	194	0
11.742	119.93	22	48	0	180	10
12.638	136.443	27	60	0	256	10
11.647	131.41	28	58	0	222	0
11.814	120.336	22	48	0	184	20
11.861	111.874	18	40	0	156	10
12.075	121.647	22	48	0	188	10
10.197	80.679	9	18	0	66	0
12.184	140.461	30	66	0	254	0
11.371	108.309	18	38	0	142	0
9.58	71.226	7	12	0	48	0
12.649	165.828	41	88	0	348	10
11.67	116.392	21	44	0	172	10
13.102	159.633	37	80	0	336	10
10.762	90.366	12	24	0	88	0
12.438	122.389	21	48	0	196	10
11.51	108.862	18	38	0	142	10
11.044	105.662	18	36	0	136	0
12.262	142.028	31	66	0	262	0
12.074	147.43	34	72	0	276	10
11.919	144.514	33	70	0	262	0
12.047	124.992	23	52	0	204	10
11.704	131.626	28	58	0	218	0
11.815	108.433	17	36	0	152	0
12.788	125.382	21	50	0	214	10
10.831	97.722	15	30	0	110	0
12.095	156.06	37	82	0	306	0
12.172	128.501	25	54	0	210	10
11.844	118.421	21	46	0	178	10
12.408	146.891	32	72	0	276	30
11.505	104.362	16	34	0	130	20
11.323	96.798	13	28	0	108	20
12.29	133.389	27	58	0	226	10
11.43	115.239	21	44	0	164	0
11.349	110.421	19	40	0	148	0
11.234	100.941	15	32	0	120	10
11.833	127.081	25	54	0	210	10
11.681	119.655	22	48	0	176	10
10.808	90.7	12	24	0	88	10
12.439	133.228	26	58	0	234	0

10.983	102.918	17	34	0	122	0
11.803	124.632	24	52	0	196	10
12.245	128.983	25	54	0	214	0
11.849	118.422	21	46	0	178	0
12.126	140.17	30	66	0	246	20
12.172	124.387	23	50	0	202	10
11.037	107.563	19	38	0	134	0
11.629	109.377	18	38	0	142	0
11.991	118.071	20	46	0	178	0
11.333	108.189	18	38	0	142	0
10.844	87.575	10	22	0	82	10
9.547	75.975	9	16	0	56	0
11.961	121.876	23	48	0	180	0
12.477	132.249	26	56	0	236	10
12.01	144.967	33	70	0	266	0
12.879	137.644	27	60	0	260	10
11.256	110.846	20	40	0	144	0
10.719	106.005	19	38	0	130	0
10.88	90.971	12	24	0	88	10
12.158	125.423	23	52	0	204	10
12.406	165.966	41	90	6	350	50
12.788	125.382	21	50	0	214	10
12.605	141.595	29	66	6	266	50
11.33	111.148	20	40	0	148	0
11.582	118.15	22	46	0	178	0
12.614	140.465	29	64	0	268	10
11.659	107.493	17	36	0	140	0
13.052	136.4	25	60	6	260	50
12.947	130.384	23	54	0	230	10
11.379	106.145	17	36	0	136	0
11.071	101.261	16	32	0	124	0
12.68	146.117	31	70	0	282	10
12.13	127.141	25	52	0	200	0
11.477	115.409	21	44	0	168	0
12.086	129.06	26	54	0	206	0
11.231	105.522	17	36	0	136	20
10.665	90.05	12	24	0	88	0
12.046	123.676	23	50	0	194	0
11.681	115.193	20	44	0	160	0
12.679	172.433	44	94	0	374	0
12.439	133.228	26	58	0	234	0
11.904	125.259	24	52	0	200	10
12.13	120.909	21	48	0	188	20
12.581	138.205	28	62	0	262	20
11.741	115.681	20	44	0	168	0
12.988	130.655	23	54	0	234	10
12.076	125.77	24	52	0	196	10
13.121	170.409	41	92	0	384	10
11.611	103.728	16	32	0	124	0
10.444	91.118	13	26	0	90	0

11.71	110.96	18	40	0	148	0
11.778	108.023	17	36	0	140	0
12.03	123.332	23	50	0	186	10
12.006	114.637	19	42	0	162	10
11.23	107.492	18	38	0	138	10
12.28	145.189	32	70	0	270	0
12.107	131.561	26	58	0	222	0
12.027	127.841	25	54	0	210	10
11.361	106.082	17	36	0	136	10
10.786	106.409	19	38	0	134	0
11.974	137.084	30	62	0	234	0
12.128	136.044	28	62	0	238	10
12.813	135.184	26	58	0	250	10
12.547	127.441	23	52	0	220	10
10.727	101.669	17	34	0	118	0
10.727	101.669	17	34	0	118	0
11.293	123.035	25	52	0	180	0
11.234	109.711	19	40	0	144	0
10.962	93.821	13	26	0	98	0
11.234	109.711	19	40	0	144	0
11.44	106.396	17	36	0	136	0
11.973	132.902	27	60	0	220	0
12.138	127.511	24	54	0	210	0
12.138	127.511	24	54	0	210	0
10.908	111.346	21	42	0	146	10
12.009	144.813	33	70	0	258	0
11.325	101.487	15	32	0	128	10
11.668	106.405	17	34	0	142	0
12.261	142.109	30	68	0	264	10
11.578	114.819	20	44	0	164	20
13.051	163.773	38	86	0	354	20
11.434	112.993	20	42	0	154	0
10.363	83.824	10	20	0	72	0
12.244	123.598	22	50	6	198	60
12.479	148.306	33	72	0	284	10
11.055	105.528	18	36	0	136	0
11.984	131.808	27	58	0	214	10
11.361	106.113	17	36	0	140	10
11.754	120.135	22	48	0	184	10
12.197	138.259	29	64	0	240	10
11.524	109.179	18	38	0	150	10
12.074	128.076	25	54	0	210	10
11.681	120.848	23	48	0	184	10
11.585	113.708	20	42	0	162	10
11.31	108.088	18	38	0	142	10
11.696	131.576	28	58	0	214	0
11.42	117.007	22	46	0	162	10
11.891	118.605	21	46	0	178	0
11.518	111.256	19	40	0	156	10
12.133	121.972	22	48	0	192	10

12.128	136.044	28	62	0	238	10
10.985	100.837	16	32	0	124	0
12.458	122.734	21	48	0	204	10
13.016	148.938	32	70	0	302	0
11.494	97.837	13	28	0	112	10
11.611	103.728	16	32	0	124	0
11.38	107.412	17	38	0	142	10
11.349	114.779	21	44	0	164	10
11.229	122.925	25	52	0	184	0
11.373	114.885	21	44	0	164	0
11.13	105.728	18	36	0	132	0
11.659	111.798	19	40	0	152	0
11.88	130.549	26	58	0	218	10
12.158	125.423	23	52	0	204	10
11.573	115.892	21	44	0	164	0
11.845	133.549	28	60	0	224	0
9.664	78.773	10	18	0	58	0
12.046	142.9	32	68	0	252	10
13.052	167.932	40	90	0	370	10
12.039	143.885	33	68	0	252	0
13.787	209.969	57	130	0	546	30
12.786	135.932	26	60	0	244	10
11.945	123.106	23	50	0	190	0
11.938	123.162	23	50	0	190	10
11.63	111.741	19	40	0	152	10
12.165	137.403	28	64	0	248	20
11.975	123.268	23	50	0	194	10
11.683	114.207	20	42	0	162	10

SRW06	SRW07	SRW08	SRW09	SRW10	MPC01	MPC02
550	126	2630	1116	13324	17	23
720	0	3276	0	15738	24	31
1010	0	5200	0	28276	28	40
322	0	1430	0	6602	11	14
562	112	2782	936	14654	17	23
560	0	3134	0	18416	13	21
1108	112	5632	882	30214	32	44
370	98	1834	738	9522	11	15
908	126	4642	1116	24970	25	36
504	0	2228	0	10346	18	22
1554	0	8080	0	44164	42	61
1116	126	5582	1152	29506	33	45
760	14	3738	216	19406	23	31
976	0	5006	0	27578	29	39
1176	154	6534	1656	38134	27	42
1126	154	6276	1656	36772	26	40
522	126	2594	1080	13480	15	21
1502	98	7592	684	40080	40	60
542	0	2342	0	10538	19	24
796	126	3966	1062	20646	23	32
936	140	5056	1368	29018	24	36
744	210	3722	1638	19512	21	30
820	112	4074	936	21076	23	33
1064	154	5898	1656	34510	25	38
868	84	3958	540	18868	29	37
1514	154	9314	1638	60652	31	53
664	224	2914	1194	13296	23	29
1652	504	8176	4356	42342	46	67
816	126	4052	1170	21248	24	33
622	0	2614	0	11446	23	28
1512	0	7676	0	40714	42	60
2120	378	11998	3600	72028	49	79
712	112	3492	954	17850	20	29
710	0	3472	0	17824	22	29
2024	364	12768	2760	86290	40	70
1070	98	5362	720	28252	31	43
1262	0	7424	0	46756	28	44
600	112	3242	936	18480	15	23
1216	126	6312	1170	34484	32	48
1266	126	7112	1170	41970	30	47
1304	210	6626	1692	35504	37	52
706	14	3608	216	19380	20	28
2112	0	12594	0	79980	45	76
786	126	4046	1152	21812	21	31
1080	126	5314	1170	27626	33	44
1066	0	5908	0	34602	26	38
1116	0	6166	0	35964	27	40
1328	98	7136	684	40628	34	49
1228	182	6512	1206	36914	32	46

1408	196	7622	1404	43700	35	52
1240	98	6656	702	38124	32	46
1866	98	9952	684	56006	48	70
1240	98	6664	702	38234	32	46
1230	84	6996	522	42900	30	44
1162	0	6378	0	37088	29	42
1572	0	8526	0	48788	39	58
1580	98	8420	702	47340	40	59
1074	98	5850	702	33784	27	39
1530	98	8218	702	46748	39	57
1190	84	6414	540	36962	31	44
1088	112	5548	972	29316	28	43
1066	0	5908	0	34602	26	38
1190	84	6382	522	36502	31	44
1082	84	5816	522	33526	28	40
1066	0	5908	0	34602	26	38
772	98	3536	684	17102	26	33
916	0	4362	0	21848	29	38
762	0	4152	0	23646	18	29
622	0	2920	0	14360	20	26
802	0	4118	0	22566	23	32
556	0	2670	0	13454	17	23
802	0	4118	0	22566	23	32
1002	140	5456	1368	31098	24	38
736	112	3788	954	20410	20	29
448	98	1898	684	8384	17	20
870	112	4142	900	20574	27	36
968	0	4820	0	25136	28	39
798	98	4238	720	23842	21	31
1038	238	5534	1410	31104	27	40
998	112	5040	900	26776	28	40
490	0	2372	0	11828	14	20
984	140	5042	1368	27244	27	39
480	84	2286	522	11410	15	20
928	14	4688	216	24742	26	37
448	0	2244	0	11868	14	18
660	0	3372	0	17956	18	26
716	14	3552	216	18634	22	29
1514	154	9314	1638	60652	31	53
776	14	3904	216	20594	22	31
658	0	3204	0	16330	20	27
1446	266	8090	2574	47516	33	52
674	210	3394	1674	17938	19	27
674	0	3394	0	17858	19	27
1400	238	8170	2106	50542	31	51
2540	238	14058	2106	82232	61	96
1058	14	5226	234	27202	31	43
592	0	2732	0	13240	20	25
916	0	4748	0	26052	26	36
2742	0	15590	0	94776	63	100

990	0	4556	0	21640	30	42
1232	252	6264	2250	33358	34	49
1570	0	9258	0	58230	35	57
740	0	3644	0	18784	22	30
308	0	1234	0	5076	13	14
892	126	4854	1206	27906	23	34
1908	210	10348	1656	59366	48	73
610	98	2660	702	12020	20	27
622	0	2904	0	14140	20	26
602	0	2834	0	13848	19	25
852	0	4074	0	20354	27	35
804	112	3892	936	19718	24	33
1314	126	7416	1170	44180	30	49
982	0	4622	0	22548	29	41
834	168	4136	1044	21688	24	34
742	140	3852	1350	20950	20	29
882	126	4516	1170	24338	24	35
288	0	1362	0	6678	9	12
1188	0	6102	0	33036	33	47
644	0	3174	0	16318	19	26
216	0	1008	0	4752	6	9
1660	126	8756	1134	49108	44	65
788	84	3924	522	20574	22	32
1724	140	9944	1422	61710	40	64
396	0	1960	0	10164	12	16
978	154	5412	1656	31848	24	37
638	112	3142	936	16328	19	26
606	0	2904	0	14396	18	25
1260	0	6638	0	36626	33	49
1254	126	6140	1098	31272	36	51
1174	0	5702	0	28890	35	48
970	126	5060	1134	27652	26	38
964	0	4602	0	22958	29	40
762	0	4152	0	23646	18	29
1142	140	6854	1494	43970	25	41
474	0	2198	0	10610	15	20
1372	0	6642	0	33372	41	56
984	140	5042	1368	27244	27	39
832	126	4210	1134	22196	23	33
1284	322	6556	2628	35332	36	51
574	224	2770	1836	14224	17	24
484	224	2364	1818	12238	14	20
1066	98	5586	738	31178	29	42
734	0	3548	0	17824	22	30
664	0	3220	0	16240	20	27
536	98	2600	720	13192	16	22
960	112	4714	936	24024	27	39
786	84	3832	540	19608	24	32
384	98	1824	702	9074	12	16
1156	0	6330	0	36808	29	44

526	0	2474	0	12174	17	22
880	112	4292	900	21952	26	36
1038	0	5582	0	31814	27	40
826	14	4170	216	22036	23	33
1110	210	5486	1638	28526	33	45
986	98	5314	684	30470	25	38
566	0	2598	0	12458	19	24
668	0	3502	0	19468	19	26
844	14	4394	234	24006	23	33
644	0	3190	0	16448	19	26
370	98	1834	738	9522	11	15
220	0	912	0	3896	8	10
846	0	4428	0	24698	24	33
1214	154	6844	1656	40556	28	43
1198	0	5858	0	30020	35	49
1392	140	8276	1404	52080	30	50
628	0	3000	0	15100	20	26
518	0	2218	0	9918	19	23
384	112	1832	900	9194	12	16
964	126	4980	1116	27042	26	38
1626	350	8206	2328	43280	45	65
1142	140	6854	1494	43970	25	41
1308	350	7090	2346	40606	33	50
640	0	3036	0	15310	20	27
808	0	3970	0	20466	23	33
1372	154	7724	1656	45794	32	49
666	0	3508	0	19576	18	26
1404	364	8548	2724	55860	30	50
1242	140	7646	1494	50694	27	44
624	0	3120	0	16326	18	25
560	0	2700	0	13492	16	23
1396	126	7682	1152	45060	35	53
946	0	4976	0	27852	26	37
752	0	3616	0	18094	22	31
960	0	4966	0	27334	27	38
588	210	2704	1584	12896	18	25
390	0	1872	0	9344	12	16
926	0	4882	0	27210	25	36
716	14	3552	216	18634	22	29
1786	0	9382	0	52084	47	70
1156	0	6330	0	36808	29	44
928	112	4688	936	24732	26	37
894	224	4676	1908	25848	24	35
1334	266	7406	2574	43232	31	48
770	14	3832	216	19964	22	31
1278	140	7906	1512	52484	27	45
904	140	4588	1350	24602	26	36
1964	140	11112	1422	66512	46	73
608	0	3364	0	19732	16	23
368	0	1634	0	7586	13	16

700	0	3692	0	20620	20	27
690	0	3812	0	22296	18	26
854	140	4330	1350	23240	25	34
780	126	4178	1134	23792	21	30
590	98	2754	738	13588	19	25
1276	0	6630	0	36260	35	50
1030	14	5222	234	27768	29	41
972	84	4906	540	26144	27	39
612	84	2992	540	15316	18	25
542	0	2342	0	10538	19	24
1046	0	5138	0	26822	31	43
1112	84	5686	540	30532	31	44
1336	140	7922	1404	49628	29	48
1144	126	6588	1170	40032	26	42
490	0	2190	0	10154	17	21
490	0	2190	0	10154	17	21
748	0	3388	0	16102	26	32
622	0	2952	0	14830	20	26
446	0	2226	0	11646	13	18
622	0	2952	0	14830	20	26
624	0	3152	0	16756	18	25
984	14	4836	216	25000	30	40
1026	0	5538	0	31364	27	39
1026	0	5538	0	31364	27	39
576	98	2426	702	10652	21	26
1144	0	5562	0	28480	35	47
584	112	2848	900	14452	16	24
712	0	3894	0	22334	17	27
1244	112	6416	936	34698	34	49
716	196	3396	1440	16994	22	30
1796	238	10122	2160	60496	43	67
690	0	3402	0	17562	21	28
314	0	1496	0	7420	10	13
968	518	5262	4128	30430	25	37
1362	126	7164	1134	39612	36	53
588	0	2712	0	13006	18	25
970	84	4870	522	25988	29	39
636	98	3100	684	15736	18	26
846	98	4208	720	21818	24	34
1102	140	5528	1350	29134	32	44
698	112	3478	864	17988	19	28
972	140	4898	1350	25964	27	39
840	98	4144	702	21328	24	34
732	112	3562	918	18132	21	30
632	84	3030	540	15118	19	26
946	0	4574	0	23258	29	39
682	112	3154	918	15496	23	29
832	14	4226	234	22476	23	33
706	112	3436	936	17470	20	29
936	98	5048	774	28908	24	36

1112	84	5686	540	30532	31	44
548	0	2580	0	12592	16	23
1068	126	6172	1134	37548	24	39
1606	0	9518	0	59980	35	58
544	98	2912	756	16448	14	21
608	0	3364	0	19732	16	23
638	98	3118	720	15878	19	26
710	98	3284	720	15794	22	30
772	0	3496	0	16462	26	33
716	14	3380	144	16704	22	30
576	0	2740	0	13706	18	24
712	0	3696	0	20300	20	28
988	126	4858	1116	24898	29	40
964	126	4980	1116	27042	26	38
746	0	3732	0	19584	22	30
1020	0	5088	0	26600	30	41
222	0	930	0	4078	9	10
1124	84	5500	540	28358	34	46
1866	140	10434	1422	61900	45	70
1154	0	5820	0	30848	34	46
2854	406	16714	4104	105010	65	104
1248	140	7148	1440	43680	30	46
884	0	4558	0	24910	25	35
884	112	4510	936	24130	25	35
694	112	3464	918	18140	20	28
1150	224	5792	1836	30494	32	46
896	126	4490	1152	23600	25	36
738	126	3642	1098	18832	21	30

MPC03	MPC04	MPC05	MPC06	MPC07	MPC08	MPC09
28	36	37	36	33	19	9
34	40	47	46	50	47	44
53	71	92	101	109	123	129
14	16	18	12	10	4	0
30	40	46	48	45	44	32
23	19	9	6	0	0	0
60	77	89	89	94	98	96
20	25	24	20	16	14	3
51	72	94	110	132	149	159
24	27	31	25	23	22	19
83	101	128	146	160	184	211
61	80	98	110	128	139	140
41	55	72	81	92	100	102
53	73	96	110	110	110	97
57	76	92	103	100	95	94
55	74	88	97	96	93	90
28	36	37	29	28	24	16
73	94	124	139	153	170	178
24	27	32	27	28	28	22
43	55	62	63	64	64	62
52	76	105	122	142	154	145
41	54	59	62	65	71	66
43	54	69	78	89	103	104
51	71	84	91	89	84	77
45	56	65	68	77	83	84
84	116	155	194	243	288	321
29	31	37	33	34	38	36
90	118	135	158	190	231	271
44	60	78	85	96	103	97
28	30	36	32	32	36	34
76	96	125	147	180	187	219
119	179	251	315	391	482	537
34	43	53	56	55	57	52
37	45	52	47	46	44	44
110	171	279	409	586	809	1048
56	72	83	85	86	84	80
67	94	136	167	209	253	283
33	42	47	46	38	30	17
60	77	88	97	105	120	126
71	99	139	175	227	288	340
73	102	129	143	166	181	196
39	54	70	73	78	84	82
109	152	221	285	354	459	576
42	56	73	83	101	117	102
59	78	96	98	109	120	112
53	68	83	87	96	93	89
55	70	87	93	100	95	93
68	87	104	115	137	150	164
62	83	95	106	127	138	151

71	96	113	130	154	169	186
62	82	98	111	127	125	133
97	124	151	170	201	220	253
62	83	101	114	132	135	144
61	83	100	117	135	134	137
58	75	95	104	115	114	114
77	103	125	141	169	188	209
78	98	122	143	167	185	213
54	71	83	88	97	93	97
78	102	125	137	156	171	196
60	80	96	109	127	131	138
52	65	86	105	114	122	142
53	68	83	87	96	93	89
60	81	98	109	130	142	153
53	71	85	96	112	113	115
53	68	83	87	96	93	89
42	53	57	59	56	54	50
47	60	77	78	94	100	94
35	45	55	65	53	51	40
31	38	48	46	46	42	36
42	58	80	88	102	113	116
25	28	30	28	21	16	12
42	58	80	88	102	113	116
51	69	85	92	100	110	103
40	52	65	71	77	80	73
23	24	19	16	15	15	16
46	58	68	68	73	74	81
48	59	72	65	69	63	65
44	60	78	92	103	110	104
54	74	101	120	141	149	155
53	66	76	80	77	69	68
21	20	24	18	18	14	4
53	73	97	109	126	134	122
25	30	30	27	24	22	16
50	68	95	109	128	152	167
22	25	26	17	12	8	4
32	36	44	44	36	28	24
40	54	71	79	88	94	96
84	116	155	194	243	288	321
42	55	73	82	86	85	82
33	41	49	45	43	44	37
74	105	123	142	144	151	172
38	51	58	65	69	74	65
34	42	50	51	50	45	44
77	108	144	179	227	277	321
137	187	243	297	369	452	551
56	75	102	121	141	157	169
30	37	44	40	44	40	32
48	59	64	68	76	60	44
140	195	269	340	426	508	608

41	50	58	54	61	58	48
68	94	115	120	139	150	139
88	131	182	225	278	328	392
38	43	46	43	46	42	36
15	12	9	8	8	8	4
49	70	90	105	123	133	119
108	155	217	278	369	481	592
25	31	26	26	26	21	23
31	36	44	42	46	42	42
30	35	41	37	39	40	34
45	56	68	62	70	74	70
42	56	71	78	87	90	96
67	95	124	155	190	235	272
42	50	65	64	71	79	76
43	57	58	67	67	67	62
41	56	69	69	77	77	69
49	67	87	107	119	130	142
13	13	14	7	1	0	0
61	80	109	124	146	167	189
33	39	46	45	49	46	34
6	0	0	0	0	0	0
84	103	136	168	206	244	277
38	47	51	59	52	49	45
92	135	187	242	309	368	436
18	21	20	12	8	4	0
55	82	118	149	175	201	200
34	43	49	53	52	55	44
27	25	28	22	20	22	21
65	81	100	107	109	128	134
63	76	82	81	78	73	81
58	70	80	76	74	72	76
53	72	90	92	101	107	107
45	53	66	65	75	75	74
35	45	55	65	53	51	40
66	104	158	207	264	323	364
22	24	26	24	26	20	8
71	89	115	113	129	147	158
53	73	97	109	126	134	122
45	57	69	74	87	94	91
72	98	118	130	147	161	170
30	42	45	50	53	42	31
26	35	35	36	34	21	10
58	83	104	124	132	141	131
37	42	50	48	53	54	52
34	39	46	43	46	42	36
28	35	38	39	33	29	26
47	59	73	75	77	86	87
43	55	66	72	84	84	72
20	23	17	11	8	3	0
63	90	129	158	195	221	258

26	29	30	23	22	20	14
46	60	72	75	88	97	94
56	78	102	118	131	140	150
44	58	79	88	94	95	92
62	83	98	104	115	125	137
52	69	82	84	91	89	96
28	31	36	33	34	36	28
35	43	53	50	44	34	22
47	67	94	109	128	144	156
33	39	43	37	35	32	31
20	25	24	20	16	14	3
8	6	4	0	0	0	0
45	57	71	66	62	52	36
61	85	100	109	112	114	122
60	74	93	98	104	107	126
76	108	144	181	222	274	311
32	35	40	37	30	24	24
24	26	32	27	26	28	24
20	24	21	14	6	4	2
54	76	99	116	140	160	173
78	98	119	120	135	155	175
66	104	158	207	264	323	364
71	103	140	174	211	231	254
30	37	45	46	43	35	26
37	45	60	58	58	61	64
70	100	125	141	151	162	179
35	46	60	62	57	52	39
78	122	186	248	326	425	507
72	114	170	221	287	360	411
32	38	49	51	55	55	51
24	25	30	26	28	19	12
77	111	151	178	225	268	301
51	70	90	100	97	79	70
34	43	55	54	62	60	44
51	69	88	97	101	99	87
30	36	34	31	29	34	36
19	19	22	18	11	3	0
50	67	90	101	113	120	129
40	54	71	79	88	94	96
92	121	159	177	187	224	260
63	90	129	158	195	221	258
50	63	76	80	85	94	89
51	72	90	101	119	137	136
68	96	113	127	134	141	154
41	54	74	81	87	88	82
74	116	175	232	298	371	429
50	69	89	95	106	114	108
104	150	216	275	347	440	562
32	38	34	32	16	12	4
17	20	22	18	18	12	4

38	50	62	56	60	52	40
37	47	50	47	38	32	20
48	67	85	89	98	102	94
43	60	71	66	68	60	44
30	37	41	39	40	41	33
67	84	113	130	158	183	209
56	77	103	113	130	144	157
49	65	85	99	106	109	97
32	38	40	43	45	40	30
24	27	32	27	28	28	22
52	67	82	95	103	107	114
61	79	99	114	130	145	168
73	102	135	166	208	252	285
62	87	114	139	172	208	231
24	24	26	24	20	16	14
24	24	26	24	20	16	14
38	44	51	46	48	48	50
31	38	47	42	44	45	46
22	25	24	19	14	8	0
31	38	47	42	44	45	46
32	42	50	47	44	37	32
54	72	98	113	135	159	178
56	76	105	112	126	132	147
56	76	105	112	126	132	147
27	32	29	27	26	22	22
59	78	95	98	101	104	99
26	36	40	40	45	46	26
31	31	28	22	20	18	12
68	91	115	125	137	152	163
38	50	57	63	73	82	75
99	144	201	255	324	411	513
36	44	50	44	42	41	40
15	16	14	8	5	2	0
51	70	86	88	98	102	100
73	98	126	150	175	196	216
24	29	33	31	31	26	19
52	68	79	79	85	88	80
30	36	38	38	33	26	24
43	54	63	65	63	61	64
61	82	103	107	122	134	142
34	39	43	42	36	31	30
51	69	91	100	112	121	122
42	49	54	58	55	46	47
35	44	53	58	68	73	56
33	37	38	40	44	40	32
46	56	64	56	59	51	47
36	44	51	54	56	56	49
45	59	79	90	97	96	91
33	42	49	47	51	51	42
50	66	78	80	83	84	76

61	79	99	114	130	145	168
22	26	30	25	20	18	10
58	79	106	127	154	180	199
88	128	179	225	276	327	395
30	37	40	39	30	24	12
32	38	34	32	16	12	4
34	43	50	48	53	60	53
33	40	45	41	43	44	41
38	41	47	43	38	34	33
34	40	44	47	36	34	32
26	29	31	30	22	19	14
36	45	56	51	50	45	38
53	68	82	80	86	91	88
54	76	99	116	140	160	173
39	49	58	55	57	58	58
54	64	76	71	70	70	71
10	9	6	1	0	0	0
60	78	89	91	100	110	120
100	145	209	265	338	431	546
61	70	78	82	86	70	56
166	264	394	545	754	1001	1293
72	112	168	222	296	368	427
47	65	84	87	91	99	99
49	65	76	84	98	108	104
37	47	56	59	57	55	51
63	85	105	112	127	141	138
47	62	85	97	111	123	118
38	49	55	53	53	50	42

MPC10	piPC01	piPC02	piPC03	piPC04	piPC05	piPC06
6	3.114	3.644	4.101	4.64	4.841	4.973
37	3.401	3.839	4.193	4.658	5.115	5.227
100	3.689	4.29	4.804	5.341	5.862	6.116
0	2.773	3.277	3.59	4.067	4.541	4.227
20	3.114	3.644	4.18	4.739	5.024	5.327
0	2.773	3.332	3.401	3.367	2.485	2.197
102	3.664	4.086	4.519	4.935	5.227	5.209
0	2.773	3.258	3.807	4.19	4.443	4.543
160	3.57	4.223	4.87	5.513	6.044	6.401
15	3.135	3.512	3.866	4.289	4.717	4.547
232	3.784	4.159	4.466	4.654	4.89	5.024
149	3.761	4.353	4.986	5.606	6.136	6.476
100	3.434	3.989	4.57	5.146	5.692	5.896
82	3.611	4.119	4.632	5.147	5.639	5.892
94	3.526	4.069	4.48	4.904	5.276	5.303
86	3.497	4.016	4.428	4.853	5.186	5.149
10	3.045	3.57	4.056	4.545	4.903	4.776
202	3.892	4.454	4.898	5.376	5.865	6.002
20	3.178	3.597	3.855	4.289	4.763	4.674
62	3.332	3.773	4.186	4.596	4.907	4.812
123	3.434	3.998	4.557	5.129	5.677	5.986
63	3.45	4.135	4.758	5.375	5.743	6.072
98	3.497	4.155	4.768	5.393	5.981	6.392
79	3.466	3.98	4.353	4.809	5.135	5.057
76	3.584	4.007	4.413	4.842	5.266	5.137
332	3.584	4.143	4.673	5.043	5.403	5.673
34	3.296	3.676	3.937	4.289	4.754	4.655
301	4.127	4.723	5.277	5.82	6.182	6.579
89	3.497	4.107	4.759	5.429	6.006	6.363
30	3.296	3.651	3.917	4.275	4.745	4.646
236	4.043	4.673	5.204	5.754	6.321	6.61
586	4.078	4.684	5.272	5.871	6.385	6.752
40	3.258	3.876	4.438	5.063	5.564	5.895
40	3.296	3.75	4.171	4.579	4.95	4.86
1259	3.784	4.413	4.954	5.501	6.09	6.514
78	3.611	4.034	4.404	4.805	5.064	5.017
275	3.466	4.016	4.546	5.036	5.564	5.811
4	3.02	3.618	4.21	4.712	4.913	5.13
140	3.584	4.043	4.369	4.691	4.963	5.252
387	3.701	4.363	5.032	5.637	6.268	6.701
223	3.961	4.61	5.279	5.95	6.489	6.849
80	3.367	3.951	4.549	5.169	5.745	6.008
675	3.932	4.477	4.86	5.236	5.663	5.958
95	3.418	4.131	4.814	5.529	6.185	6.66
111	3.795	4.404	5.061	5.727	6.313	6.644
86	3.526	4.052	4.519	4.935	5.282	5.221
94	3.555	4.103	4.567	4.983	5.364	5.365
177	3.795	4.341	4.837	5.273	5.54	5.573
164	3.738	4.284	4.747	5.221	5.321	5.433

200	3.773	4.307	4.786	5.245	5.485	5.697
136	3.75	4.266	4.744	5.191	5.46	5.645
294	4.119	4.652	5.134	5.536	5.826	5.922
148	3.726	4.252	4.717	5.168	5.43	5.609
127	3.651	4.163	4.589	5.034	5.228	5.416
113	3.638	4.167	4.647	5.087	5.504	5.56
241	3.97	4.564	4.984	5.501	5.895	6.027
228	3.961	4.519	4.995	5.435	5.778	5.982
96	3.57	4.099	4.604	5.034	5.253	5.398
224	3.922	4.457	4.955	5.364	5.642	5.799
139	3.701	4.208	4.676	5.115	5.337	5.528
140	3.676	4.373	4.893	5.471	6.023	6.441
86	3.526	4.052	4.519	4.935	5.282	5.221
164	3.726	4.259	4.728	5.216	5.498	5.591
113	3.597	4.099	4.493	4.939	5.12	5.262
86	3.497	3.989	4.407	4.779	5.056	5.063
48	3.418	3.767	4.133	4.544	4.566	4.758
94	3.611	4.078	4.489	4.97	5.478	5.494
33	3.296	4.052	4.491	5.03	5.534	6.016
24	3.296	3.807	4.277	4.779	5.31	5.277
93	3.401	3.97	4.511	5.092	5.685	5.896
8	3.178	3.75	4.065	4.504	4.866	4.977
93	3.434	4.025	4.605	5.214	5.815	6.072
87	3.526	4.22	4.745	5.319	5.838	6.076
68	3.296	3.932	4.511	5.045	5.575	5.74
15	3.157	3.534	3.879	4.231	3.989	3.736
86	3.611	4.094	4.6	5.078	5.555	5.606
61	3.624	4.22	4.794	5.364	5.907	6.014
89	3.332	3.922	4.535	5.081	5.619	5.988
139	3.526	4.086	4.637	5.175	5.735	6.12
65	3.401	3.761	4.043	4.277	4.431	4.477
0	2.944	3.481	3.812	4.117	4.633	4.407
117	3.638	4.301	4.943	5.61	6.204	6.58
12	2.996	3.541	4.003	4.447	4.797	4.758
174	3.555	4.205	4.84	5.469	6.104	6.532
0	2.773	3.091	3.296	3.401	3.434	2.996
24	3.296	3.97	4.494	4.961	5.483	5.674
94	3.367	3.892	4.46	5.009	5.55	5.704
332	3.584	4.143	4.673	5.043	5.403	5.673
81	3.434	4.025	4.605	5.173	5.739	5.973
32	3.332	3.912	4.388	4.907	5.407	5.426
190	3.75	4.366	4.906	5.471	5.824	6.089
54	3.199	3.72	4.296	4.823	5.124	5.475
31	3.296	3.932	4.494	5.051	5.59	5.825
349	3.526	4.043	4.477	4.844	5.136	5.357
633	4.159	4.625	4.997	5.328	5.595	5.799
165	3.689	4.205	4.673	5.178	5.712	5.883
24	3.296	3.761	4.227	4.73	5.222	5.107
36	3.526	4.025	4.516	5.019	5.459	5.505
726	4.331	4.883	5.321	5.752	6.17	6.326

50	3.714	4.331	4.483	5.002	5.481	5.459
156	3.761	4.304	4.812	5.36	5.818	5.911
427	3.784	4.413	5.022	5.57	5.999	6.332
26	3.466	3.998	4.525	4.971	5.444	5.671
0	2.639	2.708	2.773	2.565	2.303	2.197
110	3.401	4.03	4.672	5.341	5.886	6.303
700	4.025	4.595	5.15	5.694	6.244	6.608
22	3.314	3.897	4.092	4.58	4.534	4.732
40	3.367	3.892	4.382	4.879	5.424	5.558
32	3.296	3.829	4.29	4.79	5.282	5.253
70	3.555	4.007	4.449	4.888	5.311	5.092
84	3.541	4.147	4.708	5.326	5.848	6.16
288	3.526	4.025	4.382	4.762	5.056	5.288
70	3.526	3.998	4.294	4.722	5.228	5.341
48	3.497	4.094	4.605	5.136	5.302	5.551
58	3.367	3.97	4.575	5.152	5.657	5.76
132	3.481	4.09	4.715	5.309	5.805	6.209
0	2.565	2.996	3.219	3.401	3.555	3.332
193	3.689	4.22	4.727	5.259	5.833	6.099
30	3.296	3.871	4.407	4.945	5.474	5.633
0	2.197	2.708	2.565	0	0	0
283	3.892	4.363	4.721	5.074	5.51	5.753
40	3.45	4.107	4.55	5.072	5.405	5.766
491	3.807	4.331	4.71	5.118	5.497	5.817
0	2.639	2.944	2.996	3.219	3.178	2.708
204	3.367	3.942	4.541	5.129	5.685	6.024
31	3.199	3.72	4.24	4.765	5.05	5.369
16	3.135	3.651	3.994	4.261	4.688	4.451
145	3.526	3.912	4.19	4.407	4.615	4.682
90	3.829	4.363	4.73	5.123	5.38	5.542
83	3.829	4.382	4.8	5.267	5.673	5.754
121	3.497	4.082	4.681	5.277	5.739	5.991
73	3.638	4.159	4.549	5.004	5.509	5.533
33	3.258	4.016	4.41	4.948	5.438	5.891
387	3.401	4.016	4.613	5.203	5.78	6.143
0	2.996	3.481	3.855	4.296	4.756	4.751
174	4.007	4.575	5.103	5.635	6.188	6.321
117	3.638	4.301	4.943	5.61	6.204	6.58
93	3.466	4.043	4.595	5.116	5.62	5.786
187	3.861	4.422	5.013	5.577	6.023	6.307
22	3.091	3.555	3.555	3.932	4.06	4.205
7	2.833	3.258	3.367	3.689	3.738	3.807
119	3.611	4.182	4.716	5.27	5.734	6.049
50	3.466	4.025	4.543	5.002	5.52	5.682
32	3.367	3.912	4.443	4.929	5.43	5.483
15	2.996	3.401	3.784	4.143	4.369	4.489
84	3.611	4.245	4.801	5.381	5.898	6.143
64	3.624	4.147	4.761	5.307	5.763	6.071
0	2.708	3.045	3.258	3.434	3.296	2.708
261	3.584	4.167	4.76	5.331	5.906	6.32

10	3.091	3.57	3.966	4.387	4.764	4.58
99	3.466	3.942	4.347	4.819	5.258	5.447
137	3.526	4.103	4.689	5.237	5.765	6.134
84	3.466	4.078	4.654	5.224	5.799	6.044
151	3.807	4.413	5.035	5.64	6.07	6.35
84	3.57	4.245	4.793	5.335	5.715	5.895
16	3.178	3.597	3.975	4.362	4.858	4.887
14	3.178	3.624	4.09	4.507	4.909	4.874
166	3.401	3.97	4.538	5.104	5.667	5.879
28	3.178	3.676	4.107	4.515	4.877	4.771
0	2.773	3.219	3.689	4.159	4.248	4.511
0	2.398	2.773	2.833	2.833	2.197	0
20	3.466	3.97	4.494	5.006	5.509	5.537
132	3.555	4.103	4.593	5.084	5.419	5.478
120	3.861	4.454	4.968	5.518	6.096	6.364
318	3.555	4.094	4.605	4.977	5.366	5.609
21	3.219	3.701	4.178	4.594	5.057	5.117
18	3.135	3.512	3.834	4.218	4.71	4.586
0	2.862	3.442	3.919	4.423	4.511	4.477
172	3.597	4.266	4.915	5.557	6.084	6.445
189	4.086	4.687	5.203	5.758	6.313	6.53
387	3.401	4.016	4.632	5.236	5.82	6.188
274	3.689	4.256	4.826	5.387	5.931	6.344
17	3.219	3.726	4.115	4.624	5.127	5.272
51	3.296	3.795	4.155	4.609	5.104	5.183
196	3.726	4.324	4.894	5.492	6.01	6.276
26	3.219	3.795	4.373	4.889	5.402	5.664
556	3.555	4.167	4.725	5.303	5.881	6.25
433	3.497	4.069	4.652	5.238	5.792	6.137
30	3.239	3.861	4.451	5.044	5.68	6
6	3.045	3.57	3.937	4.332	4.807	4.785
329	3.807	4.44	5.068	5.69	6.224	6.528
57	3.584	4.167	4.68	5.209	5.688	5.975
39	3.497	4.143	4.466	5.007	5.564	5.705
71	3.611	4.182	4.675	5.188	5.673	5.938
32	3.277	3.812	4.218	4.683	4.88	5.182
0	2.773	3.314	3.79	4.188	4.665	4.547
113	3.466	4.016	4.598	5.123	5.668	6.015
94	3.401	3.951	4.559	5.14	5.686	5.885
287	4.078	4.663	5.136	5.634	6.165	6.446
261	3.584	4.167	4.76	5.331	5.906	6.32
92	3.584	4.245	4.876	5.465	5.995	6.294
138	3.512	4.167	4.842	5.493	5.941	6.279
168	3.701	4.287	4.813	5.361	5.704	5.9
76	3.434	4.025	4.595	5.162	5.744	5.97
456	3.466	4.086	4.698	5.288	5.869	6.252
88	3.555	4.078	4.595	5.124	5.621	5.701
671	3.892	4.357	4.673	5.043	5.416	5.666
2	2.996	3.401	3.761	4.094	3.85	4.025
0	2.89	3.35	3.695	4.188	4.655	4.523

28	3.178	3.597	4.073	4.518	4.934	4.849
16	3.219	3.726	4.216	4.724	4.987	5.042
76	3.526	4.025	4.549	5.083	5.558	5.6
28	3.401	3.951	4.494	5.077	5.56	5.602
29	3.219	3.767	4.332	4.924	5.325	5.572
219	3.784	4.357	4.86	5.334	5.909	6.227
172	3.638	4.174	4.705	5.277	5.81	6.023
82	3.611	4.234	4.7	5.256	5.835	6.13
22	3.178	3.701	4.163	4.634	5.054	5.276
20	3.178	3.597	3.855	4.289	4.763	4.674
102	3.584	3.97	4.248	4.575	4.883	5.112
176	3.714	4.327	4.955	5.534	6.047	6.44
291	3.555	4.094	4.595	4.984	5.361	5.602
230	3.367	3.871	4.277	4.654	4.949	5.198
14	3.091	3.512	3.917	4.254	4.685	4.647
14	3.091	3.512	3.917	4.254	4.685	4.647
49	3.526	3.97	4.425	4.864	5.3	5.141
34	3.277	3.818	4.376	4.96	5.525	5.695
0	2.833	3.384	3.907	4.355	4.727	4.616
34	3.277	3.818	4.376	4.96	5.525	5.695
22	3.219	3.85	4.425	5.017	5.547	5.725
191	3.638	4.143	4.696	5.251	5.821	6.119
153	3.541	4.103	4.71	5.288	5.878	6.159
153	3.541	4.103	4.71	5.288	5.878	6.159
23	3.314	3.767	4.041	4.467	4.42	4.379
105	3.784	4.277	4.745	5.269	5.729	5.865
14	3.091	3.767	4.296	4.973	5.448	5.732
0	3.135	3.75	4.021	4.326	4.615	4.414
173	3.761	4.347	4.932	5.491	5.938	6.244
77	3.418	4.034	4.635	5.261	5.66	6.024
600	3.829	4.263	4.625	5.011	5.352	5.591
35	3.258	3.726	4.155	4.568	4.936	4.836
0	2.708	3.239	3.707	4.141	4.467	4.123
104	3.401	3.882	4.301	4.737	5.07	4.997
244	3.839	4.463	5.028	5.598	6.083	6.493
16	3.178	3.75	3.897	4.414	4.851	4.911
80	3.597	4.056	4.501	4.961	5.185	5.169
22	3.277	3.946	4.371	4.875	5.17	5.338
64	3.57	4.223	4.739	5.292	5.722	5.938
140	3.807	4.337	4.869	5.379	5.849	5.844
26	3.314	4.003	4.462	4.936	5.266	5.405
119	3.638	4.263	4.832	5.435	6.028	6.374
48	3.434	3.998	4.398	4.815	5.268	5.547
53	3.367	4.03	4.484	5.095	5.594	5.959
28	3.314	3.866	4.402	4.875	5.183	5.426
42	3.638	4.205	4.625	5.119	5.575	5.529
44	3.401	3.882	4.398	4.908	5.404	5.671
85	3.466	4.06	4.654	5.207	5.786	6.04
38	3.296	3.937	4.429	5.037	5.484	5.752
71	3.541	4.201	4.755	5.29	5.662	5.837

176	3.651	4.227	4.824	5.387	5.975	6.372
12	2.996	3.541	3.845	4.322	4.727	4.67
190	3.296	3.807	4.248	4.585	4.934	5.124
431	3.784	4.425	5.029	5.57	6.003	6.343
3	2.97	3.534	4.117	4.583	4.745	4.983
2	2.996	3.401	3.761	4.094	3.85	4.025
50	3.239	3.801	4.319	4.801	5.068	5.031
39	3.239	3.67	3.996	4.417	4.505	4.622
31	3.526	4.007	4.443	4.842	5.291	5.195
30	3.367	3.902	4.208	4.638	4.975	5.124
10	3.219	3.773	4.082	4.515	4.874	4.997
30	3.178	3.651	4.099	4.545	4.993	4.998
95	3.611	4.086	4.567	5.031	5.424	5.479
172	3.597	4.266	4.915	5.557	6.084	6.445
52	3.367	3.97	4.527	5.056	5.554	5.657
73	3.611	4.069	4.53	4.897	5.231	5.2
0	2.398	2.565	2.565	2.485	2.197	0.693
128	3.818	4.347	4.87	5.426	5.877	6.146
648	3.871	4.317	4.635	5.011	5.384	5.628
52	3.761	4.248	4.792	5.246	5.668	5.794
1634	4.369	4.982	5.607	6.233	6.817	7.292
473	3.638	4.266	4.965	5.66	6.292	6.784
78	3.555	4.127	4.659	5.215	5.728	5.877
108	3.611	4.216	4.891	5.555	6.091	6.491
37	3.258	3.689	4.025	4.369	4.635	4.796
151	3.738	4.353	4.944	5.546	6.029	6.267
108	3.555	4.216	4.835	5.477	6.101	6.504
36	3.384	4.003	4.539	5.099	5.467	5.621

piPC07	piPC08	piPC09	piPC10	TPC	piID	PCR
4.992	4.633	3.944	4.038	48.066	59.919	1.247
5.506	5.477	5.446	5.246	74.255	93.377	1.258
6.444	6.759	7.104	7.051	95.829	136.598	1.425
4.202	3.841	0	0	31.251	41.518	1.329
5.471	5.625	5.399	4.872	58.512	79.292	1.355
0	0	0	0	30.152	31.555	1.047
5.405	5.564	5.46	5.493	104.537	125.568	1.201
4.615	4.635	3.892	0	34.751	46.155	1.328
6.885	7.299	7.685	8.012	101.372	159.296	1.571
4.604	4.684	4.555	4.428	58.293	79.622	1.366
5.118	5.268	5.398	5.501	132.882	133.477	1.004
6.874	7.242	7.44	7.774	124.639	191.945	1.54
6.295	6.641	6.951	7.199	93.241	141.802	1.521
6.101	6.384	6.543	6.582	88.365	117.262	1.327
5.383	5.363	5.309	5.589	93.216	111.851	1.2
5.286	5.331	5.24	5.454	90.346	109.069	1.207
5.08	5.273	5.183	5.03	47.307	63.983	1.352
6.245	6.323	6.442	6.741	131.978	165.492	1.254
4.857	4.94	4.582	4.541	59.104	74.522	1.261
4.967	5.026	4.82	4.995	79.954	95.414	1.193
6.351	6.689	6.907	6.889	86.507	114.113	1.319
6.404	6.83	6.947	7.28	77.283	121.644	1.574
6.869	7.353	7.632	7.92	80.296	119.781	1.492
5.178	5.282	5.157	5.419	88.57	107.447	1.213
5.445	5.741	6.049	6.213	81.24	100.23	1.234
5.911	6.153	6.4	6.551	120.968	137.275	1.135
4.737	4.902	4.981	4.961	77.29	95.266	1.233
7.069	7.531	7.937	8.259	155.283	226.222	1.457
6.825	7.233	7.412	7.679	87.259	138.383	1.586
4.711	4.868	4.933	4.835	76.802	94.849	1.235
7.057	7.326	7.752	8.11	139.058	206.565	1.485
7.182	7.592	7.839	8.135	225.749	290.481	1.287
6.183	6.496	6.326	6.217	66.953	93.491	1.396
4.834	4.975	5.112	5.063	77.7	99.665	1.283
6.959	7.405	7.772	8.069	176.696	203.226	1.15
5.107	5.128	5.17	5.23	118.037	137.469	1.165
6.131	6.452	6.67	6.76	119.421	143.762	1.204
5.152	5.104	4.875	3.596	46.557	58.329	1.253
5.501	5.771	5.971	6.172	124.526	149.741	1.202
7.195	7.697	8.156	8.477	136.037	203.17	1.493
7.316	7.713	8.03	8.421	160.831	277.651	1.726
6.414	6.821	7.156	7.494	78.114	120.048	1.537
6.203	6.518	6.773	6.987	193.335	210.866	1.091
7.21	7.719	7.886	8.166	76.674	119.335	1.556
7.096	7.539	7.658	7.921	148.289	249.015	1.679
5.466	5.52	5.5	5.659	93.397	119.03	1.274
5.548	5.547	5.554	5.77	96.461	121.882	1.264
5.889	6.116	6.289	6.517	129.446	171.976	1.329
5.778	5.984	6.242	6.477	119.9	153.338	1.279

5.915	5.953	6.099	6.255	121.515	142.789	1.175
5.879	5.886	6.092	6.224	103.13	124.849	1.211
6.227	6.447	6.635	6.891	169.791	208.183	1.226
5.833	5.82	5.934	6.092	107.057	128.586	1.201
5.703	5.722	5.859	5.912	101.824	122.771	1.206
5.733	5.77	5.824	5.962	101.942	127.928	1.255
6.358	6.592	6.777	7.109	140.705	184.161	1.309
6.273	6.481	6.78	6.945	136.099	173.16	1.272
5.578	5.509	5.629	5.764	92.244	113.816	1.234
6.008	6.109	6.336	6.569	142.798	174.33	1.221
5.762	5.776	5.885	6.027	103.715	125.382	1.209
6.865	7.236	7.691	7.97	93.047	136.054	1.462
5.466	5.52	5.5	5.659	93.397	119.03	1.274
5.94	6.171	6.428	6.665	116.814	155.251	1.329
5.561	5.569	5.682	5.8	98.304	119.583	1.216
5.298	5.323	5.328	5.389	93.397	112.271	1.202
4.855	4.692	4.641	4.619	84.362	94.043	1.115
5.859	6.172	6.315	6.473	91.46	113.887	1.245
6.105	6.324	6.222	6.481	57.619	79.215	1.375
5.568	5.839	6.002	6.081	58.827	80.7	1.372
6.292	6.708	7.036	7.123	90.485	130.976	1.447
4.841	4.395	4.313	3.684	50.226	65.855	1.311
6.494	6.883	7.203	7.333	90.485	136.525	1.509
6.448	6.746	6.994	7.185	82.475	115.457	1.4
6.024	6.36	6.593	6.873	72.33	103.577	1.432
3.712	3.783	3.969	4.15	53.376	70.28	1.317
5.9	6.049	6.341	6.679	99.125	140.089	1.413
6.322	6.492	6.76	6.384	108.601	165.823	1.527
6.314	6.589	6.749	6.735	76.163	101.14	1.328
6.489	6.746	6.994	6.974	92.22	116.519	1.263
4.443	4.331	4.29	4.22	86.634	87.266	1.007
4.467	4.577	3.841	0	39.313	50.279	1.279
7.016	7.379	7.531	7.808	93.383	140.552	1.505
4.732	4.904	4.961	4.808	48.835	66.311	1.358
6.998	7.493	7.9	8.255	107.819	167.998	1.558
2.565	2.197	1.609	0	38.604	39.362	1.02
5.752	5.668	5.901	6.196	55.82	80.083	1.435
6.07	6.433	6.766	7.02	89.684	133.245	1.486
5.911	6.153	6.4	6.551	120.968	137.275	1.135
6.315	6.569	6.804	7.092	80.495	117.481	1.459
5.622	5.977	6.188	6.347	70.01	108.13	1.544
6.313	6.572	6.879	7.052	128.053	174.662	1.364
5.69	5.903	5.859	5.708	68.818	90.723	1.318
6.135	6.386	6.683	6.771	60.783	89.485	1.472
5.606	5.826	5.999	6.133	133.234	140.799	1.057
6.026	6.242	6.45	6.613	299.485	309.765	1.034
6.215	6.547	6.86	7.086	109.454	148.907	1.36
5.472	5.74	5.864	5.939	58.573	79.714	1.361
5.892	6.072	5.99	6.11	69.002	88.624	1.284
6.619	6.84	7.109	7.331	243.698	297.716	1.222

5.764	5.858	5.377	5.49	103.333	139.09	1.346
6.166	6.37	6.131	6.234	151.397	204.505	1.351
6.683	7.021	7.375	7.637	132.683	168.176	1.268
6.079	6.304	6.281	6.106	62.344	84.461	1.355
2.197	2.197	1.609	0	35.188	35.188	1
6.704	7.05	7.069	7.176	85.419	121.269	1.42
7.042	7.456	7.809	8.124	204.645	260.172	1.271
4.814	4.463	4.738	4.992	62.91	85.062	1.352
5.998	6.181	6.446	6.683	66.034	95.617	1.448
5.558	5.915	6.055	6.326	63.148	92.645	1.467
5.323	5.492	5.429	5.523	93.736	123.518	1.318
6.6	6.965	7.358	7.592	79.247	117.691	1.485
5.497	5.746	5.976	6.078	123.596	133.56	1.081
5.58	5.72	5.766	5.654	95.354	117.86	1.236
5.781	6.084	6.386	6.529	69.443	92.872	1.337
6.143	6.389	6.614	6.759	72.285	105.84	1.464
6.583	6.905	7.232	7.421	90.094	126.75	1.407
1.609	0	0	0	24.626	29.678	1.205
6.505	6.893	7.266	7.451	137.287	186.412	1.358
6.025	6.306	6.386	6.601	59.47	83.57	1.405
0	0	0	0	13.194	14.47	1.097
6.032	6.342	6.622	6.743	199.082	237.435	1.193
5.849	5.944	6.043	6.292	68.908	97.381	1.413
6.125	6.292	6.532	6.712	156.759	175.251	1.118
2.398	1.946	0	0	32.85	34.028	1.036
6.348	6.658	6.799	6.999	98.742	125.005	1.266
5.538	5.744	5.593	5.21	64.282	84.684	1.317
4.189	4.317	4.43	4.457	55.509	71.783	1.293
4.7	4.86	4.905	4.984	115.95	115.95	1
5.651	5.495	5.72	5.981	131.077	164.353	1.254
5.857	5.711	5.914	6.159	132.449	184.493	1.393
6.29	6.585	6.453	6.644	103.662	138.378	1.335
5.834	5.978	6.214	6.445	85.222	110.345	1.295
5.887	5.952	5.603	5.789	57.619	75.5	1.31
6.508	6.861	7.122	7.282	110.687	133.161	1.203
4.973	4.822	3.726	0	42.224	52.656	1.247
6.701	7.07	7.413	7.753	177.204	280.344	1.582
7.016	7.379	7.531	7.808	93.383	140.552	1.505
6.188	6.486	6.698	6.984	82.649	117.512	1.422
6.718	7.094	7.436	7.744	143.219	215.291	1.503
4.357	4.263	4.159	4.078	56.427	62.588	1.109
3.85	3.526	2.833	2.639	45.049	48.15	1.069
6.313	6.615	6.785	6.879	100.551	135.204	1.345
6.107	6.386	6.614	6.847	72.819	106.179	1.458
5.839	5.968	6.124	6.326	65.021	94.874	1.459
4.369	4.431	4.443	4.205	50.907	61.541	1.209
6.425	6.817	6.859	7.086	100.05	151.214	1.511
6.521	6.865	7.116	7.368	69.095	94.237	1.364
2.565	2.197	0	0	32.58	35.211	1.081
6.721	7.023	7.349	7.448	112.962	144.871	1.282

4.494	4.609	4.559	4.202	51.428	66.79	1.299
5.756	5.99	6.008	6.118	91.531	111.593	1.219
6.476	6.728	7.007	6.981	92.226	117.401	1.273
6.387	6.668	6.915	7.119	85.531	126.279	1.476
6.776	7.17	7.55	7.907	132.588	217.436	1.64
6.276	6.459	6.846	6.993	89.058	129.546	1.455
5.083	5.229	5.062	4.227	54.749	67.184	1.227
5.062	5.05	4.725	4.577	54.395	66.041	1.214
6.284	6.673	7.014	7.307	100.115	141.337	1.412
4.725	4.854	4.962	4.857	64.128	82.68	1.289
4.489	4.654	3.714	0	34.751	45.455	1.308
0	0	0	0	19.348	22.034	1.139
5.872	6.082	6.006	5.912	64.691	86.032	1.33
5.633	5.695	5.851	6.038	99.257	122.512	1.234
6.696	6.971	7.398	7.44	129.747	193.835	1.494
5.849	6.116	6.384	6.515	118.34	134.748	1.139
4.96	4.575	4.62	4.513	61.578	79.432	1.29
4.621	4.725	4.665	4.298	60.408	75.445	1.249
3.726	3.446	3.056	0	34.115	45.863	1.344
6.922	7.348	7.742	8.063	106.399	169.594	1.594
6.914	7.32	7.771	7.996	168.624	276.114	1.637
6.583	6.966	7.256	7.471	110.687	137.714	1.244
6.732	7.001	7.302	7.438	122.116	152.818	1.251
5.342	5.249	5.062	4.585	58.855	74.042	1.258
5.345	5.536	5.676	5.381	80.473	99.539	1.237
6.634	6.951	7.237	7.219	128.243	182.112	1.42
5.85	6.079	6.135	6.015	58.038	78.334	1.35
6.631	7.031	7.325	7.52	135.583	161.944	1.194
6.532	6.918	7.199	7.37	120.953	147.526	1.22
6.398	6.731	6.993	6.565	58.441	83.914	1.436
5.015	4.707	4.467	3.446	46.092	58.109	1.261
6.962	7.312	7.503	7.743	150.884	210.918	1.398
6.232	6.301	6.536	6.654	83.775	118.873	1.419
6.108	6.405	6.394	6.624	72.174	107.671	1.492
6.277	6.512	6.676	6.781	85.353	118.31	1.386
5.198	5.537	5.872	5.938	58.135	80.681	1.388
4.189	3.056	0	0	33.341	42.522	1.275
6.354	6.619	6.881	6.821	86.484	111.746	1.292
6.273	6.609	6.919	7.163	89.684	138.926	1.549
6.729	7.073	7.421	7.642	180.067	247.248	1.373
6.721	7.023	7.349	7.448	112.962	144.871	1.282
6.596	6.972	7.014	7.248	92.932	137.111	1.475
6.667	7.044	7.126	7.393	96.414	147.142	1.526
6.198	6.469	6.7	6.816	119.007	161.213	1.355
6.331	6.605	6.83	7.034	80.782	117.566	1.455
6.629	6.993	7.267	7.43	118.9	142.877	1.202
6.043	6.371	6.545	6.598	85.29	115.225	1.351
5.9	6.138	6.387	6.565	182.571	184.552	1.011
3.135	3.045	1.946	1.609	44.329	47.863	1.08
4.66	4.486	3.056	0	37.606	48.503	1.29

5.112	5.131	5.012	4.818	59.763	71.124	1.19
5.159	5.263	4.817	4.937	55.397	71.487	1.29
5.959	6.274	6.437	6.484	82.354	112.568	1.367
5.983	6.221	6.222	6.154	61.595	83.023	1.348
5.87	6.159	5.658	5.644	66.338	95.43	1.439
6.663	7.047	7.431	7.669	144.764	198.712	1.373
6.405	6.738	7.026	7.349	120.784	170.666	1.413
6.449	6.686	6.811	7.027	88.482	127.537	1.441
5.488	5.596	5.476	5.426	54.37	69.714	1.282
4.857	4.94	4.582	4.541	59.104	74.522	1.261
5.283	5.434	5.583	5.537	110.76	129.875	1.173
6.792	7.146	7.512	7.545	115.157	161.691	1.404
5.855	6.107	6.37	6.485	116.038	133.671	1.152
5.468	5.727	5.911	5.961	106.493	116.132	1.091
4.462	4.004	4.018	4.15	51.402	65.361	1.272
4.462	4.004	4.018	4.15	51.402	65.361	1.272
5.347	5.461	5.73	5.91	96.038	145.121	1.511
6.033	6.33	6.632	6.042	71.698	106.666	1.488
4.577	3.944	0	0	36.097	45.344	1.256
6.033	6.33	6.632	6.042	71.698	106.666	1.488
5.968	6.193	6.405	6.477	58.343	86.759	1.487
6.561	6.99	7.37	7.688	131.372	193.534	1.473
6.524	6.847	7.199	7.167	109.255	152.886	1.399
6.524	6.847	7.199	7.167	109.255	152.886	1.399
4.408	4.172	4.261	4.563	66.959	86.225	1.288
6.049	6.191	6.163	6.337	124.808	174.213	1.396
6.122	6.44	5.853	5.481	51.015	71.245	1.397
4.611	4.849	4.808	0	45.211	55.527	1.228
6.518	6.794	6.725	6.872	150.82	209.639	1.39
6.476	6.903	6.995	7.3	80.174	119.024	1.485
5.835	6.078	6.293	6.449	171.634	173.736	1.012
4.794	4.954	5.082	5.008	73.027	93.899	1.286
3.992	3.169	0	0	28.438	39.546	1.391
5.191	5.27	5.124	5.331	91.021	106.304	1.168
6.88	7.23	7.568	7.944	131.13	179.926	1.372
5.107	5.16	4.727	4.547	54.143	70.082	1.294
5.404	5.604	5.48	5.575	93.249	117.807	1.263
5.433	5.183	5.497	5.744	55.679	77.063	1.384
6.188	6.338	6.662	6.93	87.128	136.709	1.569
6.184	6.443	6.668	6.824	106.137	141.43	1.333
5.483	5.364	5.651	5.88	59.5	83.785	1.408
6.813	7.21	7.592	7.9	95.548	142.588	1.492
5.654	5.323	5.188	5.276	79.952	103.06	1.289
6.414	6.779	6.603	6.731	70.04	97.274	1.389
5.736	5.887	5.922	6.124	58.458	80.122	1.371
5.723	5.525	5.478	5.254	103.531	147.683	1.426
5.979	6.224	6.292	6.335	84.355	125.195	1.484
6.4	6.66	6.902	7.119	82.972	119.477	1.44
6.077	6.361	6.065	6.015	68.879	99.665	1.447
6.085	6.417	6.619	6.856	85.778	126.572	1.476

6.752	7.11	7.476	7.481	115.157	153.795	1.336
4.46	4.251	4.038	4.343	46.086	57.193	1.241
5.342	5.557	5.753	5.756	97.852	105.538	1.079
6.694	7.021	7.381	7.65	132.697	168.386	1.269
4.941	4.949	4.627	3.446	43.877	55.896	1.274
3.135	3.045	1.946	1.609	44.329	47.863	1.08
5.249	5.576	5.734	6.043	71.068	101.062	1.422
4.75	4.86	4.853	4.878	76.982	94.134	1.223
5.151	4.878	4.919	4.872	93.496	129.507	1.385
4.987	4.687	4.7	4.505	73.318	91.735	1.251
4.849	4.455	4.353	3.791	54.239	70.874	1.307
5.182	5.124	5.049	4.952	67.032	83.6	1.247
5.645	5.819	5.746	5.798	118.697	158.873	1.338
6.922	7.348	7.742	8.063	106.399	169.594	1.594
5.856	6.165	6.499	6.738	70.058	98.371	1.404
5.22	5.223	5.339	5.439	114.613	145.587	1.27
0	0	0	0	22.04	22.903	1.039
6.581	6.961	7.303	7.577	118.815	178.438	1.502
5.875	6.118	6.36	6.531	180.81	182.816	1.011
5.961	5.839	5.411	5.234	109.747	144.296	1.315
7.807	8.277	8.678	9.11	316.118	437.72	1.385
7.296	7.737	8.004	8.323	134.348	184.904	1.376
6.189	6.532	6.816	6.844	86.645	127.121	1.467
6.977	7.423	7.666	8.031	92.132	149.079	1.618
4.796	4.934	5.017	4.905	62.367	74.755	1.199
6.573	6.857	6.66	6.841	139.75	201.593	1.443
6.972	7.425	7.685	7.92	90.969	144.792	1.592
5.864	6.019	6.204	6.373	65.458	90.264	1.379

PCD	CID	BID	X0	X1	X2	X3
11.853	32.928	17.315	13	7.968	7.196	5.045
19.122	46.798	24.204	18.113	11.329	10.13	6.797
40.769	52.534	26.252	18.845	12.38	11.57	9.185
10.267	21.393	11.414	8.268	5.182	4.755	2.94
20.78	32.178	16.34	11.544	7.72	6.715	5.384
1.403	25.626	14.396	11.621	6.061	6.936	4.341
21.03	60.522	30.201	21.562	14.541	12.307	10.836
11.404	20.41	10.597	6.975	4.877	4.263	3.683
57.924	45.956	22.325	15.104	10.737	9.777	8.582
21.329	35.256	18.251	13.38	8.63	7.247	4.993
0.596	79.412	40.177	29.886	18.852	17.093	15.453
67.305	62.522	31.194	22.38	14.994	12.746	11.007
48.561	43.271	21.302	14.656	10.254	8.983	7.263
28.898	55.628	28.249	20.681	13.455	11.278	9.318
18.636	49.895	25.239	18.516	11.631	11.819	9.469
18.724	48.038	24.251	17.646	11.237	11.197	9.059
16.676	28.09	14.418	10.129	6.698	6.025	5.099
33.514	74.597	37.187	26.957	17.308	17.874	12.955
15.417	37.087	19.223	14.251	8.969	8.17	5.076
15.46	42.859	21.271	14.82	10.186	8.977	7.83
27.606	44.592	22.325	16.016	10.452	9.811	8.362
44.361	38.847	19.323	13.405	9.165	8.408	7.141
39.485	42.784	21.272	14.983	10.042	9.448	7.914
18.877	46.22	23.254	16.776	10.809	10.807	8.259
18.99	54.939	27.224	18.899	13.22	11.263	8.706
16.307	56.562	28.29	20.698	12.963	13.318	13.127
17.976	43.555	22.192	15.786	10.631	9.268	6.154
70.94	86.243	43.162	31.585	20.353	19.016	15.851
51.124	44.952	22.281	15.527	10.665	9.522	7.705
18.047	45.095	23.176	17.079	10.969	9.572	6.165
67.507	79.016	39.184	28.045	18.619	17.512	13.678
64.732	88.932	43.189	30.585	20.369	20.501	18.221
26.539	37.783	19.285	14.044	8.904	8.831	6.22
21.965	42.009	21.265	15.242	10.13	8.613	7.052
26.531	70.911	34.287	23.889	16.017	17.124	15.608
19.431	57.696	28.182	19.399	13.651	12.197	10.244
24.341	50.416	24.324	16.364	11.623	11.239	10.438
11.773	27.8	14.468	10.456	6.537	6.232	5.877
25.214	60.013	30.197	22.104	14.01	14.129	10.644
67.133	54.477	26.287	18.052	12.525	11.966	11.588
116.82	68.481	33.196	22.786	16.152	14.298	12.355
41.935	37.243	18.381	12.535	8.771	7.816	6.603
17.531	82.663	41.192	30.389	18.839	20.372	17.312
42.661	38.857	19.321	13.569	9.059	8.678	7.343
100.726	61.946	30.17	20.761	14.758	12.506	10.699
25.633	48.32	24.247	17.43	11.414	10.61	9.088
25.421	50.177	25.235	18.301	11.807	11.232	9.499
42.53	63.027	31.197	22.121	14.88	13.639	11.838
33.438	59.097	29.205	20.543	13.936	13.012	10.59

21.274	64.726	32.201	23.043	15.237	14.489	11.86
21.718	59.999	30.206	21.999	14.239	13.211	10.515
38.393	89.204	44.154	31.706	21.01	19.406	16.963
21.529	60.012	30.205	21.999	14.239	13.238	10.409
20.947	56.073	28.226	20.474	13.297	12.385	10.006
25.986	54.22	27.22	19.715	12.845	11.804	9.961
43.457	72.615	36.177	26.198	16.985	16.686	12.841
37.062	74.501	37.176	26.905	17.491	16.9	13.718
21.572	50.283	25.241	18.137	11.935	10.947	9.09
31.532	72.616	36.174	26.146	17.116	16.007	13.396
21.666	58.128	29.211	21.129	13.845	12.604	10.073
43.007	52.157	26.244	19.113	12.054	12.86	9.156
25.633	48.32	24.247	17.43	11.414	10.61	9.088
38.436	57.331	28.218	19.673	13.542	12.39	10.202
21.279	52.273	26.225	18.845	12.38	11.621	8.956
18.874	48.32	24.247	17.43	11.414	10.61	9.088
9.681	49.116	24.229	16.615	11.843	9.786	8.042
22.427	54.958	27.208	19.062	13.131	11.455	8.879
21.596	33.606	17.375	12.845	7.716	8.669	5.95
21.873	38.155	19.293	13.665	9.165	8.053	5.999
40.492	43.098	21.299	14.82	10.148	9.275	7.302
15.629	32.962	17.287	12.889	7.954	7.397	5.046
46.04	43.098	21.299	14.82	10.148	9.275	7.302
32.982	44.32	22.307	16.121	10.232	10.702	8.4
31.247	36.931	18.338	12.698	8.665	8.003	7.127
16.904	33.314	17.235	12.51	8.274	6.369	5.001
40.963	50.929	25.203	17.648	12.19	10.432	8.687
57.222	52.749	26.197	18.571	12.449	11.66	8.89
24.977	38.831	19.352	13.569	9.113	8.421	7.428
24.299	49.016	24.289	16.845	11.559	10.842	8.965
0.632	53.164	27.226	20.301	12.705	11.533	9.696
10.965	26.984	14.352	10.768	6.416	6.473	4.513
47.17	50.513	25.264	18.137	11.918	11.024	9.123
17.476	28.18	14.386	9.966	6.771	5.936	4.861
60.179	48.039	23.301	15.811	11.204	10.302	8.766
0.758	27.314	14.376	10.441	6.726	5.489	4.256
24.264	33.875	17.331	12.466	7.998	7.735	6.136
43.561	41.437	20.321	13.786	9.898	8.154	7.085
16.307	56.562	28.29	20.698	12.963	13.318	13.127
36.986	41.01	20.334	14.113	9.665	8.722	7.551
38.12	38.026	19.297	13.828	9.075	8.219	6.196
46.609	60.493	30.224	21.955	14.041	14.198	11.659
21.904	35.016	17.357	11.828	8.292	7.325	6.626
28.702	35.867	18.318	13.284	8.486	8.018	6.258
7.565	55.98	27.282	19.027	12.777	13.07	12.149
10.28	111.411	54.122	38.319	25.59	25.6	22.823
39.453	58.629	29.24	20.966	13.901	12.585	9.889
21.141	38.3	19.285	13.502	9.271	7.785	5.83
19.622	49.574	25.263	18.449	11.892	10.628	8.429
54.019	116.488	58.158	42.75	26.99	27.641	22.626

35.757	57.399	29.151	21.493	13.5	13.916	8.112
53.109	62.514	30.189	20.665	14.597	13.429	11.638
35.493	64.349	32.265	23.859	14.867	14.852	13.467
22.117	42.613	22.249	16.861	10.349	8.921	7.436
0	26.497	14.264	10.812	6.778	4.65	3.784
35.85	42.848	21.329	15.035	10.109	9.139	7.903
55.527	87.623	42.2	29.283	20.282	19.006	17.692
22.151	38.767	20.208	15.173	9.26	9.329	4.975
29.583	38.147	19.258	13.665	9.165	8.021	6.137
29.497	36.144	18.288	12.958	8.665	7.636	6.01
29.782	51.067	25.217	17.485	12.275	10.223	8.639
38.444	44.905	22.276	15.527	10.631	9.703	7.543
9.964	54.987	27.26	19.613	12.544	13.507	10.623
22.506	55.41	28.2	20.786	13.034	13.36	8.21
23.428	44.55	22.29	15.579	10.566	9.865	7.786
33.555	36.934	18.352	12.698	8.682	7.928	7.045
36.656	44.698	22.308	15.853	10.524	9.653	8.392
5.051	17.382	9.566	6.853	4.198	3.873	2.86
49.125	61.68	30.206	21.14	14.453	13.514	10.759
24.099	35.993	18.303	13.121	8.592	7.715	6.327
1.276	12.293	7.689	6.077	2.943	3.521	1.732
38.353	82.687	41.129	29.838	19.332	18.892	15.163
28.474	41.518	21.272	15.621	9.787	9.817	6.882
18.492	74.229	37.217	27.499	17.169	17.562	14.346
1.178	23.32	12.435	9.027	5.684	5.012	3.505
26.264	43.887	21.367	14.613	10.137	9.62	8.638
20.402	36.015	18.302	13.121	8.651	7.479	6.306
16.273	34.835	18.25	13.759	8.326	8.045	5.834
0	61.962	31.206	22.922	14.584	13.834	11.736
33.276	67.951	34.149	24.88	16.101	15.093	11.897
52.044	66.308	33.154	23.847	15.78	14.414	11.124
34.716	47.744	23.268	15.974	11.131	10.35	9.145
25.123	55.461	28.204	20.734	13.164	12.671	8.701
17.881	33.606	17.375	12.845	7.716	8.669	5.95
22.474	44.729	21.408	14.353	10.126	10.036	9.695
10.432	29.083	15.314	11.422	7.003	6.513	4.491
103.139	76.475	37.151	25.614	18.03	16.198	13.45
47.17	50.513	25.264	18.137	11.918	11.024	9.123
34.863	42.783	21.274	14.983	10.075	9.245	8.111
72.073	66.395	32.197	21.968	15.699	13.802	12.393
6.161	31.959	16.346	11.707	7.575	7.262	5.051
3.101	26.082	13.436	9.422	6.182	5.849	4.487
34.653	54.313	27.273	19.715	12.845	11.864	9.622
33.361	41.828	21.245	15.405	9.986	9.035	7.286
29.853	37.956	19.289	13.828	9.092	8.047	6.687
10.634	30.089	15.365	10.836	7.203	6.407	5.262
51.165	50.526	25.224	18.027	11.832	11.659	8.659
25.141	45.008	22.275	15.364	10.813	9	7.97
2.631	23.144	12.443	9.138	5.664	4.888	3.805
31.909	53.424	26.281	18.585	12.38	11.937	10.321

15.362	33.065	17.289	12.836	8.079	6.808	5.346
20.063	48.74	24.233	17.104	11.542	10.562	8.331
25.176	50.396	25.275	18.301	11.845	11.094	9.382
40.748	42.867	21.325	14.983	10.059	9.358	7.878
84.848	61.634	30.2	20.924	14.707	12.524	10.943
40.488	46.291	23.284	16.776	10.819	10.599	8.974
12.435	37.112	19.251	14.251	9.079	7.575	5.72
11.646	36.077	18.341	13.01	8.688	7.44	6.327
41.222	42.229	20.365	13.527	9.826	8.898	7.858
18.552	35.965	18.307	13.121	8.592	7.688	6.333
10.704	20.41	10.597	6.975	4.877	4.263	3.683
2.686	16.328	9.453	7.439	4.037	3.851	1.981
21.34	45.728	23.297	16.872	11.019	9.517	8.074
23.255	51.976	26.246	19.223	12.224	11.785	9.712
64.088	66.177	33.162	24.121	15.634	14.733	11.23
16.408	54.802	27.29	19.776	12.581	12.865	11.906
17.854	38.933	20.246	15.121	9.528	7.849	6.577
15.037	37.257	19.219	14.088	9.113	7.683	5.237
11.748	23.197	12.441	9.138	5.664	4.915	3.715
63.195	47.811	23.316	15.974	11.148	10.305	9.054
107.49	83.381	41.155	29.037	19.6	18.979	13.981
27.027	44.729	21.408	14.353	10.126	10.036	9.695
30.702	59.665	29.261	20.284	13.99	13.144	11.262
15.187	38.819	20.262	15.284	9.307	8.831	5.647
19.066	43.684	22.237	16.328	10.26	10.323	7.068
53.87	58.965	29.232	20.922	13.758	13.352	10.999
20.297	33.928	17.369	12.577	8.041	7.417	6.129
26.361	52.828	25.348	16.974	12.043	12.14	11.262
26.573	48.686	23.373	15.82	11.115	10.722	10.38
25.473	34.086	17.323	12.414	8.113	7.284	6.135
12.017	30.851	16.315	12.345	7.304	7.553	4.851
60.034	64.264	31.224	21.75	14.925	14.05	12.526
35.098	49.437	25.286	18.723	11.828	10.55	8.774
35.497	41.807	21.273	15.458	9.849	9.842	6.359
32.956	51.436	26.265	19.43	12.311	11.005	8.891
22.547	33.853	17.289	12.414	8.059	7.574	5.754
9.182	23.234	12.419	9.138	5.647	4.913	4.087
25.262	46.71	23.285	16.56	11.024	10.009	8.636
49.242	41.437	20.321	13.786	9.898	8.154	7.085
67.182	88.169	44.146	32.507	20.638	20.111	16.048
31.909	53.424	26.281	18.585	12.38	11.937	10.321
44.178	48.626	24.239	17.267	11.528	10.298	9.13
50.729	43.812	21.314	14.397	10.254	9.295	8.547
42.206	56.79	28.233	20.215	13.22	13.134	10.787
36.784	41.011	20.333	14.113	9.648	8.83	7.405
23.977	48.51	23.382	15.983	11.026	10.87	10.734
29.935	48.778	24.277	17.104	11.63	10.097	8.699
1.981	84.368	41.199	29.209	19.395	19.645	16.441
3.534	30.847	16.377	12.345	7.484	6.534	5.598
10.897	25.423	13.342	9.682	6.22	5.305	3.468

11.361	37.3	18.363	12.261	8.917	7.464	6.621
16.091	33.914	17.38	12.466	8.108	7.18	6.324
30.215	46.92	23.29	16.234	11.236	9.475	8.289
21.428	38.935	19.362	13.295	9.232	8.228	7.106
29.093	36.118	18.271	12.958	8.665	7.7	5.74
53.948	65.507	32.197	22.717	15.396	14.124	12.075
49.882	53.816	26.266	18.096	12.631	11.45	9.735
39.055	50.471	25.274	18.027	11.859	11.56	8.589
15.344	33.876	17.321	12.414	8.092	7.362	6.077
15.417	37.087	19.223	14.251	8.969	8.17	5.076
19.115	59.28	30.198	22.422	14.116	13.144	9.678
46.534	57.526	28.214	19.673	13.601	12.019	10.882
17.633	52.972	26.294	18.905	12.171	12.32	11.54
9.639	47.379	23.315	16.405	10.86	11.009	9.806
13.959	33.218	17.256	12.673	8.168	6.611	5.292
13.959	33.218	17.256	12.673	8.168	6.611	5.292
49.083	50.131	25.198	17.908	12.203	9.945	7.785
34.968	38.218	19.262	13.665	9.165	8.053	5.99
9.247	25.089	13.419	10.008	6.037	5.606	4.138
34.968	38.218	19.262	13.665	9.165	8.053	5.99
28.415	34.026	17.344	12.414	8.113	7.319	5.81
62.162	56.234	27.227	18.476	13.348	11.278	9.728
43.63	49.818	24.275	16.681	11.707	10.366	9.632
43.63	49.818	24.275	16.681	11.707	10.366	9.632
19.266	41.013	21.194	15.665	10.007	8.716	5.561
49.405	66.505	33.185	23.794	15.884	14.112	10.616
20.231	29.929	15.363	11.052	6.938	7.541	4.56
10.316	32.503	17.346	13.32	7.578	8.54	5.732
58.819	62.54	30.193	20.665	14.597	13.386	11.853
38.851	40.989	20.277	13.949	9.72	8.798	6.927
2.102	78.67	38.212	26.709	18.216	17.578	15.521
20.872	40.003	20.28	14.535	9.63	8.259	6.783
11.107	19.385	10.514	7.56	4.715	4.17	3.034
15.282	45.17	22.273	15.267	10.686	9.771	8.48
48.796	67.076	33.21	23.75	15.756	14.712	12.431
15.939	34.855	18.27	13.759	8.277	8.506	4.729
24.558	54.751	27.227	19.115	13.182	11.093	9.429
21.384	33.788	17.316	12.466	7.971	7.934	5.739
49.581	44.718	22.267	15.579	10.56	9.902	7.883
35.293	59.623	29.226	20.217	14.165	12.286	10.808
24.285	35.658	18.308	13.336	8.399	8.323	6.543
47.04	50.533	25.246	18.137	11.863	11.323	8.851
23.108	45.559	23.241	17.035	10.863	10.122	7.862
27.234	39.803	20.273	14.751	9.41	9.134	6.584
21.663	35.842	18.288	13.121	8.592	7.682	6.499
44.152	55.664	28.174	20.571	13.346	12.039	8.722
40.839	44.185	22.211	15.786	10.741	8.734	7.07
36.505	42.837	21.327	14.983	10.075	9.239	8.069
30.785	37.759	19.275	14.044	8.866	9.014	6.046
40.794	44.427	22.293	15.905	10.426	9.965	8.645

38.638	57.526	28.214	19.673	13.601	12.019	10.882
11.107	30.836	16.306	12.345	7.277	7.754	4.369
7.686	43.515	21.347	14.828	9.949	10.059	9.402
35.689	64.285	32.263	23.911	14.773	15.244	13.529
12.019	25.929	13.495	9.585	6.126	5.695	5.464
3.534	30.847	16.377	12.345	7.484	6.534	5.598
29.994	35.201	17.326	11.665	8.326	7.388	6.323
17.152	41.832	21.236	15.295	9.955	9.535	6.391
36.011	49.98	25.168	18.071	12.059	10.367	7.963
18.417	41.906	21.246	15.295	9.971	9.348	6.706
16.635	34.961	18.265	13.596	8.454	7.75	5.296
16.568	37.952	19.319	13.88	9.059	8.216	6.499
40.175	53.87	26.199	17.933	12.704	11.307	9.715
63.195	47.811	23.316	15.974	11.148	10.305	9.054
28.313	41.921	21.269	15.405	10.083	8.637	7.249
30.974	56.648	28.178	20.096	13.507	11.732	10.401
0.863	18.501	10.411	7.983	4.719	3.581	2.262
59.623	64.37	32.188	23.087	15.418	13.522	10.761
2.006	82.617	40.201	28.286	19.049	18.886	15.858
34.548	65.364	33.163	24.433	15.842	13.179	11.514
121.602	118.596	57.187	40.233	27.385	25.868	24.48
50.556	54.769	26.321	17.889	12.699	11.536	10.924
40.475	46.816	23.29	16.397	11.075	10.049	8.075
56.947	46.832	23.27	16.397	11.151	9.671	8.47
12.388	37.84	19.304	13.991	9.062	8.016	6.757
61.842	58.632	28.202	19.087	13.67	12.613	10.993
53.823	46.666	23.281	16.56	10.952	10.383	8.433
24.806	39.622	20.286	14.861	9.397	8.918	6.868

X4	X5	X0A	X1A	X2A	X3A	X4A
4.265	2.895	0.765	0.469	0.313	0.18	0.118
5.189	4.029	0.755	0.472	0.327	0.2	0.13
7.919	6.405	0.725	0.442	0.289	0.173	0.112
2.231	1.746	0.752	0.471	0.34	0.21	0.139
4.643	3.369	0.721	0.454	0.292	0.179	0.116
2.405	0.884	0.83	0.466	0.33	0.189	0.127
8.876	6.72	0.719	0.454	0.28	0.181	0.115
3.089	1.979	0.697	0.443	0.284	0.184	0.124
7.622	6.435	0.687	0.429	0.272	0.168	0.106
3.676	3.026	0.743	0.479	0.329	0.208	0.136
11.376	8.674	0.747	0.449	0.28	0.186	0.113
9.141	6.991	0.722	0.454	0.283	0.18	0.114
6.252	5.295	0.698	0.446	0.29	0.177	0.114
7.901	6.331	0.739	0.464	0.289	0.176	0.108
7.896	6.032	0.741	0.431	0.281	0.166	0.104
7.744	5.815	0.735	0.432	0.28	0.165	0.105
4.269	2.951	0.724	0.447	0.287	0.182	0.119
10.282	8.804	0.729	0.433	0.298	0.177	0.109
3.705	3.079	0.75	0.472	0.34	0.211	0.137
6.612	4.745	0.706	0.443	0.281	0.182	0.12
7.436	6.612	0.728	0.435	0.273	0.161	0.098
6.083	4.378	0.706	0.436	0.28	0.174	0.113
6	4.91	0.713	0.437	0.286	0.184	0.111
7.528	5.66	0.729	0.432	0.284	0.162	0.106
7.09	5.288	0.7	0.456	0.304	0.193	0.127
10.56	8.529	0.739	0.418	0.251	0.156	0.091
4.246	3.538	0.718	0.462	0.32	0.212	0.137
13.008	9.365	0.735	0.442	0.284	0.176	0.11
6.484	5.583	0.706	0.444	0.289	0.175	0.108
4.254	3.539	0.743	0.477	0.342	0.22	0.142
11.082	8.953	0.719	0.443	0.292	0.18	0.115
16.718	14.592	0.711	0.416	0.26	0.153	0.093
4.835	4.003	0.739	0.445	0.305	0.183	0.112
5.63	4.321	0.726	0.46	0.297	0.191	0.125
13.506	13.191	0.703	0.4	0.245	0.142	0.079
8.782	6.425	0.693	0.44	0.284	0.183	0.122
8.807	8.018	0.682	0.415	0.255	0.156	0.094
4.482	3.124	0.747	0.436	0.271	0.178	0.107
8.771	6.224	0.737	0.438	0.294	0.177	0.114
9.725	8.386	0.694	0.418	0.255	0.163	0.098
10.982	8.989	0.69	0.437	0.275	0.169	0.108
5.937	5.114	0.696	0.439	0.279	0.169	0.11
14.044	12.417	0.741	0.419	0.268	0.159	0.092
5.874	4.95	0.714	0.431	0.28	0.175	0.105
9.069	7.372	0.692	0.447	0.284	0.181	0.116
7.423	5.822	0.726	0.439	0.279	0.171	0.109
7.575	6.039	0.732	0.437	0.281	0.173	0.108
9.613	7.364	0.714	0.438	0.278	0.174	0.11
9.178	6.745	0.708	0.435	0.283	0.171	0.111

10.294	7.757	0.72	0.435	0.279	0.167	0.107
8.895	6.841	0.733	0.445	0.287	0.17	0.108
13.786	10.586	0.721	0.438	0.277	0.175	0.111
8.874	7.084	0.733	0.445	0.288	0.168	0.107
8.706	6.574	0.731	0.443	0.281	0.164	0.105
8.056	6.613	0.73	0.443	0.281	0.172	0.107
11.316	8.647	0.728	0.436	0.288	0.167	0.11
10.61	8.402	0.727	0.437	0.286	0.176	0.108
7.719	5.985	0.725	0.442	0.281	0.168	0.109
11.111	8.863	0.726	0.439	0.281	0.172	0.109
8.62	6.709	0.729	0.447	0.286	0.168	0.108
7.021	5.798	0.735	0.43	0.299	0.176	0.108
7.423	5.822	0.726	0.439	0.279	0.171	0.109
8.93	6.895	0.703	0.437	0.282	0.17	0.11
7.688	5.914	0.725	0.442	0.291	0.169	0.108
7.423	5.822	0.726	0.439	0.279	0.171	0.109
6.749	4.802	0.692	0.456	0.297	0.191	0.127
7.192	6.079	0.706	0.453	0.301	0.189	0.12
4.993	3.559	0.756	0.429	0.299	0.17	0.111
4.658	3.928	0.719	0.458	0.31	0.194	0.123
6.294	5.578	0.706	0.441	0.29	0.174	0.109
3.761	2.557	0.758	0.468	0.322	0.202	0.134
6.294	5.578	0.706	0.441	0.29	0.174	0.109
7.253	5.59	0.733	0.426	0.282	0.165	0.105
5.651	4.543	0.705	0.433	0.276	0.178	0.109
3.628	2.08	0.736	0.487	0.318	0.217	0.151
7.127	5.483	0.706	0.451	0.29	0.189	0.123
7.148	5.86	0.714	0.445	0.299	0.185	0.121
6.335	5.036	0.714	0.434	0.272	0.169	0.106
7.687	6.438	0.702	0.428	0.271	0.166	0.104
7.577	5.497	0.752	0.454	0.288	0.183	0.115
2.513	2.115	0.769	0.458	0.324	0.215	0.126
7.654	6.52	0.725	0.441	0.283	0.172	0.105
3.846	2.439	0.712	0.451	0.297	0.194	0.128
7.422	6.705	0.687	0.431	0.278	0.175	0.109
3.306	2.488	0.746	0.48	0.305	0.193	0.132
4.342	3.371	0.733	0.444	0.297	0.192	0.121
6.121	5.234	0.689	0.45	0.281	0.177	0.113
10.56	8.529	0.739	0.418	0.251	0.156	0.091
6.095	5.192	0.706	0.439	0.281	0.18	0.111
5.076	3.944	0.728	0.454	0.304	0.188	0.124
10.808	7.768	0.732	0.425	0.273	0.158	0.103
5.613	4.059	0.696	0.436	0.271	0.174	0.11
5.098	3.709	0.738	0.447	0.297	0.184	0.121
10.296	8.495	0.705	0.412	0.256	0.158	0.095
19.386	15.243	0.71	0.42	0.267	0.167	0.104
8.218	7.166	0.723	0.448	0.293	0.177	0.11
4.743	3.746	0.711	0.464	0.311	0.194	0.128
7.106	4.725	0.738	0.457	0.295	0.176	0.12
19.097	15.674	0.737	0.428	0.276	0.162	0.098

6.6	5.12	0.741	0.45	0.331	0.198	0.132
10.34	8.367	0.689	0.429	0.274	0.171	0.11
12.533	10.071	0.746	0.425	0.261	0.153	0.096
5.528	3.607	0.766	0.47	0.297	0.196	0.129
2.26	1.069	0.772	0.521	0.332	0.252	0.188
7.21	5.883	0.716	0.44	0.269	0.161	0.103
15.347	13.195	0.697	0.423	0.26	0.164	0.099
4.395	2.397	0.759	0.463	0.346	0.199	0.142
4.623	3.758	0.719	0.458	0.309	0.198	0.128
4.463	3.468	0.72	0.456	0.305	0.2	0.128
6.914	5.653	0.699	0.455	0.292	0.192	0.123
6.36	5.223	0.706	0.443	0.294	0.18	0.114
9.556	7.474	0.726	0.418	0.276	0.159	0.101
6.121	5.364	0.742	0.449	0.326	0.195	0.122
6.835	4.082	0.708	0.44	0.29	0.181	0.12
6.073	4.869	0.705	0.434	0.273	0.172	0.108
7.038	5.703	0.721	0.439	0.276	0.171	0.105
1.817	1.332	0.761	0.466	0.323	0.22	0.14
8.958	7.703	0.705	0.438	0.288	0.176	0.112
4.746	3.622	0.729	0.452	0.297	0.192	0.122
0	0	0.868	0.491	0.391	0.289	0
11.073	8.987	0.728	0.439	0.291	0.181	0.108
5.639	3.652	0.744	0.445	0.307	0.181	0.12
12.642	10.436	0.743	0.429	0.274	0.156	0.094
2.87	1.975	0.752	0.474	0.313	0.195	0.137
7.772	7.088	0.696	0.422	0.26	0.157	0.095
5.133	3.532	0.729	0.455	0.288	0.185	0.119
3.328	2.622	0.764	0.463	0.322	0.216	0.133
9.398	6.951	0.739	0.442	0.282	0.181	0.116
9.636	6.513	0.732	0.447	0.296	0.189	0.127
9.007	6.65	0.723	0.451	0.3	0.192	0.129
7.923	6.517	0.695	0.428	0.272	0.173	0.11
6.513	5.369	0.74	0.454	0.317	0.193	0.123
4.993	3.559	0.756	0.429	0.299	0.17	0.111
8.996	8.573	0.683	0.405	0.245	0.147	0.086
3.161	2.235	0.761	0.467	0.326	0.204	0.132
10.811	9.213	0.692	0.44	0.289	0.189	0.121
7.654	6.52	0.725	0.441	0.283	0.172	0.105
6.368	4.96	0.713	0.438	0.28	0.18	0.112
10.621	8.259	0.687	0.436	0.271	0.172	0.108
4.636	3.292	0.732	0.446	0.303	0.168	0.11
3.908	2.628	0.725	0.442	0.292	0.173	0.112
8.835	6.503	0.73	0.443	0.282	0.166	0.106
5.238	4.039	0.734	0.454	0.301	0.197	0.125
4.811	3.715	0.728	0.455	0.298	0.197	0.123
4.262	2.884	0.722	0.45	0.291	0.188	0.122
6.825	5.589	0.721	0.438	0.299	0.184	0.116
6.46	4.865	0.698	0.451	0.281	0.185	0.117
3.079	1.569	0.761	0.472	0.306	0.19	0.134
8.974	7.953	0.715	0.427	0.271	0.164	0.1

3.967	2.749	0.755	0.475	0.309	0.206	0.137
6.989	5.508	0.713	0.444	0.293	0.181	0.116
8.334	6.625	0.732	0.439	0.277	0.168	0.107
6.324	5.599	0.713	0.437	0.284	0.179	0.109
9.451	7.224	0.697	0.446	0.278	0.177	0.114
7.52	5.551	0.729	0.433	0.279	0.173	0.109
4.051	3.197	0.75	0.478	0.316	0.204	0.131
5.001	4.012	0.723	0.457	0.286	0.181	0.116
7.046	6.382	0.676	0.427	0.27	0.167	0.105
5.01	3.555	0.729	0.452	0.296	0.192	0.128
3.089	1.979	0.697	0.443	0.284	0.184	0.124
1.016	0.544	0.827	0.505	0.385	0.248	0.169
6.126	5.218	0.734	0.459	0.288	0.179	0.107
8.856	6.695	0.739	0.437	0.274	0.159	0.104
8.927	7.114	0.731	0.447	0.301	0.187	0.121
10.185	8.181	0.732	0.419	0.257	0.157	0.094
4.545	3.346	0.756	0.476	0.302	0.206	0.13
3.584	3.131	0.741	0.48	0.334	0.218	0.138
2.965	1.965	0.761	0.472	0.307	0.186	0.124
7.987	6.701	0.695	0.429	0.271	0.168	0.105
11.633	9.289	0.708	0.436	0.292	0.179	0.119
8.996	8.573	0.683	0.405	0.245	0.147	0.086
10.209	8.562	0.699	0.424	0.263	0.159	0.099
4.49	3.548	0.764	0.465	0.327	0.188	0.121
5.185	4.728	0.742	0.446	0.313	0.191	0.115
10.228	8.131	0.721	0.43	0.272	0.157	0.102
5.048	4.151	0.74	0.447	0.285	0.175	0.11
10.315	9.804	0.679	0.401	0.243	0.144	0.085
9.855	9.157	0.688	0.412	0.244	0.144	0.086
4.415	3.646	0.73	0.451	0.291	0.192	0.116
3.09	2.513	0.772	0.456	0.328	0.202	0.124
11.201	9.623	0.702	0.426	0.265	0.163	0.101
7.405	5.861	0.749	0.455	0.285	0.172	0.106
5.163	4.335	0.736	0.448	0.317	0.187	0.12
7.567	5.851	0.747	0.456	0.29	0.174	0.11
4.506	2.892	0.73	0.448	0.303	0.192	0.125
2.439	1.867	0.761	0.471	0.307	0.215	0.128
7.271	6.1	0.72	0.441	0.278	0.173	0.109
6.121	5.234	0.689	0.45	0.281	0.177	0.113
13.199	11.037	0.739	0.439	0.287	0.174	0.109
8.974	7.953	0.715	0.427	0.271	0.164	0.1
7.331	5.543	0.719	0.443	0.278	0.183	0.116
7.53	5.94	0.686	0.427	0.266	0.168	0.105
9.919	7.376	0.722	0.426	0.274	0.159	0.103
5.955	5.364	0.706	0.439	0.285	0.181	0.11
9.916	9.187	0.695	0.408	0.242	0.145	0.085
7.393	6.166	0.713	0.447	0.28	0.174	0.107
14.413	12.851	0.712	0.422	0.269	0.158	0.096
4.946	2.324	0.772	0.468	0.284	0.175	0.13
2.831	2.063	0.745	0.478	0.332	0.204	0.142

5.715	4.772	0.681	0.446	0.276	0.174	0.114
5.658	3.539	0.733	0.45	0.276	0.171	0.12
7.242	5.931	0.706	0.449	0.279	0.173	0.108
6.463	5.091	0.7	0.44	0.274	0.165	0.108
4.52	3.484	0.72	0.456	0.308	0.191	0.122
9.319	7.913	0.71	0.44	0.282	0.18	0.111
8.47	7.358	0.696	0.436	0.279	0.174	0.11
7.034	5.743	0.721	0.439	0.296	0.175	0.108
4.569	3.01	0.73	0.45	0.294	0.19	0.12
3.705	3.079	0.75	0.472	0.34	0.211	0.137
7.934	5.713	0.747	0.455	0.306	0.186	0.118
8.991	6.943	0.703	0.439	0.273	0.178	0.114
9.716	7.819	0.727	0.42	0.257	0.158	0.095
8.608	6.942	0.713	0.418	0.262	0.158	0.099
3.516	2.367	0.745	0.48	0.315	0.22	0.147
3.516	2.367	0.745	0.48	0.315	0.22	0.147
5.918	4.602	0.716	0.469	0.311	0.205	0.135
4.691	4.018	0.719	0.458	0.31	0.193	0.123
3.313	1.933	0.77	0.464	0.311	0.188	0.133
4.691	4.018	0.719	0.458	0.31	0.193	0.123
4.986	3.876	0.73	0.451	0.293	0.182	0.119
8.218	7.158	0.684	0.445	0.282	0.18	0.114
8.161	7.117	0.695	0.434	0.266	0.172	0.107
8.161	7.117	0.695	0.434	0.266	0.172	0.107
4.52	2.893	0.746	0.477	0.335	0.206	0.141
9.334	7.352	0.721	0.454	0.3	0.18	0.12
4.204	3.071	0.737	0.434	0.314	0.175	0.117
3.706	2.235	0.784	0.446	0.316	0.185	0.12
10.213	8.198	0.689	0.429	0.273	0.174	0.112
5.774	4.282	0.697	0.442	0.293	0.182	0.115
13.917	12.06	0.703	0.424	0.262	0.157	0.097
5.534	4.163	0.727	0.459	0.295	0.188	0.126
2.335	1.342	0.756	0.472	0.321	0.202	0.146
7.461	5.73	0.694	0.427	0.264	0.166	0.107
10.599	8.449	0.72	0.438	0.278	0.17	0.108
3.733	2.881	0.764	0.46	0.34	0.197	0.129
7.875	6.012	0.708	0.455	0.284	0.181	0.116
4.463	3.024	0.733	0.443	0.305	0.191	0.124
6.386	4.81	0.708	0.44	0.291	0.183	0.118
9.196	7.415	0.697	0.443	0.279	0.177	0.112
4.639	3.359	0.741	0.442	0.297	0.192	0.119
7.368	6.349	0.725	0.439	0.29	0.174	0.107
5.981	4.219	0.741	0.453	0.298	0.187	0.122
4.954	3.957	0.738	0.448	0.304	0.188	0.113
4.609	2.958	0.729	0.452	0.295	0.197	0.125
7.085	5.548	0.735	0.46	0.309	0.19	0.127
5.459	4.106	0.718	0.467	0.301	0.196	0.124
6.425	5.452	0.713	0.438	0.28	0.179	0.109
4.837	3.899	0.739	0.443	0.311	0.183	0.115
7.233	5.287	0.723	0.434	0.277	0.173	0.11

8.991	6.943	0.703	0.439	0.273	0.178	0.114
3.37	2.634	0.772	0.455	0.337	0.199	0.13
7.735	6.534	0.706	0.415	0.258	0.162	0.098
12.206	9.796	0.747	0.422	0.263	0.154	0.095
4.06	2.704	0.737	0.438	0.271	0.182	0.11
4.946	2.324	0.772	0.468	0.284	0.175	0.13
5.185	3.979	0.686	0.438	0.284	0.186	0.121
4.929	3.915	0.728	0.453	0.318	0.194	0.123
5.711	4.281	0.723	0.464	0.314	0.21	0.139
5.358	3.717	0.728	0.453	0.312	0.197	0.134
3.951	2.6	0.755	0.47	0.323	0.204	0.136
5.185	4.329	0.731	0.453	0.293	0.181	0.115
8.081	6.437	0.69	0.438	0.283	0.183	0.119
7.987	6.701	0.695	0.429	0.271	0.168	0.105
5.991	4.639	0.734	0.458	0.288	0.186	0.122
7.822	6.079	0.718	0.45	0.286	0.193	0.122
1.553	0.955	0.798	0.524	0.358	0.226	0.173
9.249	6.798	0.721	0.453	0.294	0.179	0.119
14.037	12.578	0.707	0.423	0.27	0.159	0.097
8.892	6.245	0.74	0.466	0.286	0.189	0.127
22.805	20.882	0.706	0.421	0.249	0.147	0.086
10.235	9.4	0.688	0.423	0.251	0.152	0.091
7.242	5.971	0.713	0.443	0.287	0.172	0.111
7.455	5.394	0.713	0.446	0.276	0.173	0.115
5.377	4.126	0.736	0.453	0.286	0.183	0.114
9.505	7.677	0.682	0.427	0.274	0.174	0.112
6.454	5.808	0.72	0.438	0.288	0.179	0.104
5.721	4.128	0.743	0.447	0.297	0.181	0.117

X5A	X0v	X1v	X2v	X3v	X4v	X5v
0.078	9.948	5.531	3.897	2.575	1.767	1.072
0.086	14.887	8.321	6.093	3.313	2.076	1.353
0.07	13.839	7.867	5.954	4.16	2.954	2.063
0.097	6.165	3.249	2.228	1.188	0.748	0.454
0.073	8.381	4.613	3.1	1.975	1.307	0.777
0.098	8.935	6.822	6.763	5.526	3.057	0.937
0.076	18.159	10.643	7.718	5.865	4.193	3.03
0.082	4.957	2.745	1.855	1.254	0.786	0.372
0.068	12.749	7.427	5.628	4.007	2.965	2.093
0.098	11.225	6.362	4.628	2.53	1.585	1.077
0.068	21.701	13.065	10.334	7.509	5.067	3.447
0.071	22.657	13.046	9.798	7.064	5.728	3.083
0.074	12.972	7.769	6.024	4.204	3.104	2.265
0.066	16.968	9.32	6.471	4.615	3.363	2.365
0.066	14.487	8.589	8.424	6.079	4.772	3.378
0.066	14.117	8.455	8.244	5.985	4.751	3.342
0.08	8.267	4.51	3.295	2.352	1.508	0.872
0.071	19.16	11.006	8.437	5.844	4.072	2.927
0.096	11.426	6.386	4.815	2.506	1.528	1.047
0.077	12.443	7.77	6.227	5.007	3.706	2.52
0.063	13.329	7.499	5.94	3.96	2.89	2.104
0.074	10.457	5.958	4.601	3.586	2.506	1.554
0.071	11.964	6.814	5.249	3.625	2.198	1.523
0.067	13.67	8.251	8.077	5.77	4.702	3.32
0.081	16.888	10.378	8.03	5.187	3.778	2.386
0.055	16.613	10.142	9.749	8.791	7.261	5.534
0.096	13.755	8.341	6.44	3.424	1.982	1.384
0.069	24.787	13.934	10.716	8.114	5.611	3.44
0.072	13.529	7.683	5.955	4.016	2.745	2.015
0.098	14.749	8.296	6.144	3.075	1.838	1.232
0.072	22.767	13.478	10.99	6.828	4.484	3.011
0.058	27.032	16.497	14.834	11.368	9.164	6.998
0.076	12.156	6.849	5.985	3.18	2.089	1.434
0.083	13.615	8.243	6.157	4.445	3.135	2.168
0.047	21.486	13.553	13.795	11.568	9.462	8.702
0.077	16.684	10.728	8.584	6.632	4.993	3.334
0.059	14.507	9.912	8.817	7.798	6.115	5.13
0.066	8.183	4.108	3.233	2.317	1.471	0.867
0.071	19.661	12.183	11.346	8.437	6.737	4.528
0.06	14.152	8.585	6.792	5.417	3.925	2.813
0.07	17.765	10.429	7.37	5.354	3.901	2.698
0.073	9.706	5.729	4.125	3.018	2.211	1.558
0.056	26.101	15.747	16.291	13.846	10.987	9.218
0.068	11.031	6.375	5.116	3.69	2.478	1.779
0.077	16.929	10.005	6.979	4.972	3.539	2.394
0.07	13.881	8.517	6.904	5.614	4.112	3.014
0.069	14.117	8.584	7.004	5.618	4.07	2.993
0.071	17.838	11.201	8.888	7.305	5.569	4.093
0.071	17.33	11.119	9.604	7.539	6.125	4.553

0.069	19.354	12.167	10.469	8.147	6.44	4.452
0.07	16.735	10.146	7.917	6.033	4.576	3.142
0.07	25.208	15.528	12.158	9.923	7.371	5.294
0.07	17.411	10.338	8.087	6.162	4.685	3.326
0.066	16.301	9.94	8.048	6.513	5.094	3.385
0.07	15.272	9.284	7.249	5.703	4.292	3.194
0.069	20.091	13.108	10.772	8.247	6.258	4.434
0.069	21.268	12.844	10.661	7.654	5.469	3.959
0.072	14.465	8.881	7.011	5.372	4.108	2.813
0.071	21.031	12.869	10.237	8.151	6.315	4.57
0.07	15.802	9.178	6.931	5.417	4.118	2.945
0.067	14.343	8.977	7.291	4.686	2.939	2.017
0.07	13.747	8.45	6.824	5.525	4.048	2.957
0.07	16.394	10.694	8.737	6.941	5.412	3.737
0.07	15.393	9.661	7.886	6.147	4.735	3.193
0.07	14.007	8.74	7.103	5.802	4.277	3.129
0.084	15.568	10.698	8.136	6.329	4.942	3.397
0.079	16.015	9.574	7.125	5.102	3.619	2.713
0.065	10.473	7.498	6.716	4.682	3.442	2.459
0.082	12.139	6.855	5.387	3.434	2.276	1.659
0.07	13.916	8.331	7.011	4.94	3.831	3.094
0.085	10.807	6.878	5.434	3.485	2.124	1.489
0.07	13.839	8.265	6.969	4.941	3.789	3.098
0.066	12.649	8.13	6.837	4.904	3.541	2.202
0.07	10.608	6.088	4.805	3.476	2.334	1.607
0.109	10.765	6.354	4.368	3.08	2.05	1.236
0.081	13.845	7.884	5.666	3.837	2.625	1.629
0.081	14.683	8.821	6.546	4.637	3.252	2.237
0.065	10.044	5.693	4.021	2.952	2.055	1.381
0.064	13.088	8.134	6.373	4.44	3.239	2.331
0.072	17.989	11.303	9.359	6.757	4.999	3.317
0.088	8.707	4.474	3.808	1.954	0.967	0.647
0.067	14.502	7.988	6.096	4.411	3.081	2.204
0.081	8.861	5.021	3.693	2.51	1.709	0.839
0.071	13.845	8.265	6.491	4.666	3.253	2.488
0.096	10.323	7.245	5.884	4.595	3.921	2.869
0.077	9.815	6.445	5.591	4.166	2.691	1.925
0.074	12.102	7.479	5.366	4.037	3.025	2.219
0.055	16.613	10.142	9.749	8.791	7.261	5.534
0.071	11.762	6.708	5.082	3.604	2.478	1.759
0.08	11.372	6.34	4.764	3.211	2.203	1.344
0.063	18.431	10.622	10.194	7.452	6.02	3.971
0.07	9.036	5.459	4.029	2.978	2.118	1.232
0.074	9.023	5.005	3.661	2.469	1.598	1.031
0.059	16.746	11.055	10.72	9.957	8.387	6.698
0.063	32.587	20.839	18.738	15.858	12.783	9.627
0.07	17.94	10.313	8.286	6.075	4.557	3.545
0.085	11.621	6.634	4.875	3.028	1.994	1.343
0.074	15.645	8.909	7.053	4.913	3.61	1.88
0.058	33.885	19.335	17.574	12.567	9.476	6.951

0.088	18.385	12.743	10.368	5.152	3.666	2.302
0.073	17.361	10.775	8.423	6.374	4.82	3.442
0.055	17.604	10.016	8.792	6.979	5.565	4.196
0.078	12.107	6.466	4.312	3.001	1.838	0.941
0.119	9.292	5.464	3.484	2.417	1.229	0.541
0.065	12.531	7.597	5.689	4.602	3.486	2.44
0.061	23.128	13.777	10.988	8.746	6.735	5.074
0.092	12.84	8.786	6.713	4.026	3.171	1.941
0.085	10.374	6.071	4.26	2.88	1.844	1.217
0.085	10.037	5.723	4.048	2.707	1.615	1.027
0.083	15.103	9.41	6.761	5.087	3.65	2.714
0.074	13.065	7.856	6.254	4.504	3.352	2.351
0.06	17.601	10.862	11.128	8.501	7.221	5.582
0.083	14.752	8.581	6.323	3.911	2.636	1.876
0.07	11.342	6.395	4.842	3.213	2.252	1.272
0.071	9.391	5.612	4.259	3.186	2.301	1.554
0.066	12.168	6.813	4.953	3.517	2.494	1.678
0.095	4.272	2.175	1.42	0.774	0.393	0.213
0.071	17.553	11.084	8.746	6.82	5.153	4.012
0.079	9.93	5.67	4.165	2.948	1.795	1.151
0	4	2.846	2.297	0.748	0	0
0.066	25.057	17.115	14.889	12.048	9.48	7.475
0.072	12.11	7.571	6.048	4.018	2.789	1.541
0.056	23.842	14.3	13.646	11.568	9.789	7.584
0.099	7.383	4.604	3.835	2.873	2.076	1.366
0.06	12.503	7.568	6.428	5.147	4.077	3.303
0.072	9.406	5.193	3.622	2.365	1.428	0.827
0.094	11.617	6.262	5.41	3.126	1.666	1.079
0.07	18.17	10.892	9.152	6.546	4.694	3.071
0.079	20.834	13.292	10.848	7.861	5.327	3.543
0.083	19.968	12.684	10.027	7.088	4.839	3.344
0.072	13.827	8.578	7.035	5.648	4.369	3.278
0.081	15.511	8.548	6.162	3.774	2.352	1.568
0.065	10.656	7.723	6.998	4.988	3.702	2.709
0.054	12.133	7.876	7.1	6.386	5.52	4.752
0.086	9.527	5.32	4.399	2.494	1.556	0.925
0.08	21.185	12.457	9.392	6.573	4.278	3.137
0.067	14.502	7.999	6.101	4.416	3.073	2.195
0.072	11.614	6.802	5.2	3.897	2.548	1.657
0.07	18.42	11.532	8.754	6.616	4.885	3.213
0.073	7.884	4.432	3.331	2.405	1.831	0.988
0.075	6.659	3.907	3.031	2.203	1.645	0.858
0.063	15.928	8.633	6.657	4.45	3.375	2.238
0.081	11.931	6.658	4.973	3.398	2.147	1.376
0.081	10.537	6.019	4.381	3.055	1.848	1.206
0.076	8.684	5.479	4.271	3.154	2.261	1.313
0.077	13.948	8.624	6.7	4.661	3.35	2.414
0.074	11.587	6.658	4.746	3.412	2.329	1.549
0.092	7.247	4.253	3.017	2.201	1.574	0.672
0.062	14.659	8.591	6.967	5.192	3.689	2.868

0.092	11.209	6.165	4.276	3.007	1.841	0.932
0.076	13.457	7.892	5.789	4.047	2.821	1.857
0.065	13.975	8.115	6.181	4.486	3.304	2.294
0.071	12.189	6.932	5.354	3.674	2.534	1.848
0.074	17.472	10.355	7.449	5.428	3.806	2.569
0.068	13.903	8.831	8.069	6.015	4.305	2.764
0.089	12.07	7.093	4.936	3.121	2.166	1.447
0.076	11.186	6.601	4.972	3.833	2.637	2.015
0.068	11.789	7.296	5.833	4.518	3.491	2.75
0.083	11.493	6.667	5.248	3.802	2.625	1.642
0.082	5.798	3.208	2.34	1.638	1.058	0.526
0.136	5.549	2.393	1.755	0.659	0.222	0.115
0.073	14.621	8.328	6.431	4.813	3.211	2.395
0.067	15.625	8.852	8.358	6.188	4.985	3.48
0.076	17.595	9.75	7.2	4.721	3.027	1.917
0.057	16.312	10.081	9.72	8.574	7.343	5.484
0.084	12.958	7.043	4.764	3.499	2.042	1.141
0.098	12.056	6.736	4.915	2.722	1.595	1.142
0.094	6.597	3.439	2.296	1.545	0.978	0.458
0.068	13.179	7.656	5.875	4.229	3.126	2.202
0.078	25.089	15.515	13.329	9.505	6.917	4.965
0.054	11.99	7.713	6.93	6.15	5.238	4.469
0.061	16.281	10.162	8.32	6.2	4.879	3.591
0.079	13.318	6.914	5.556	2.979	1.856	1.25
0.079	13.542	7.788	7.032	3.816	2.545	1.967
0.065	17.155	10.321	9.413	6.951	5.671	4.05
0.069	9.597	5.284	3.787	2.632	1.753	1.26
0.053	14.501	9.705	8.908	7.423	6.274	5.408
0.054	13.125	8.507	7.404	6.631	5.767	4.802
0.074	9.805	5.421	4.032	2.942	1.781	1.247
0.084	10.573	5.302	5.04	2.27	1.218	0.823
0.064	19.555	11.325	9.694	7.359	5.991	4.511
0.065	14.283	7.415	5.434	4.015	2.964	2.03
0.079	11.832	7.733	5.975	3.033	2.177	1.457
0.066	14.991	7.997	5.7	4.075	3.005	2.036
0.085	8.322	4.456	3.089	1.891	1.161	0.534
0.085	6.435	3.549	2.5	1.608	0.884	0.533
0.068	12.804	7.583	5.521	4.178	2.961	2.116
0.074	12.025	7.412	5.324	4.022	2.997	2.216
0.069	25.166	13.716	11.218	7.326	5.063	3.38
0.062	14.659	8.591	6.967	5.192	3.689	2.868
0.073	14.86	8.762	6.827	5.061	3.709	2.485
0.066	11.99	7.317	5.757	4.565	3.483	2.488
0.065	16.318	9.654	9.149	6.67	5.342	3.683
0.072	11.133	6.448	4.833	3.263	2.267	1.675
0.052	13.464	8.351	7.441	6.73	5.685	4.768
0.069	13.553	8.151	5.847	4.155	2.902	2.043
0.059	27.273	17.741	17.197	15.044	12.836	11.187
0.068	9.923	5.654	4.252	3.385	2.36	1.007
0.094	7.833	4.225	2.639	1.486	1.004	0.591

0.077	11.405	7.724	6.006	4.953	3.994	3.136
0.071	9.319	5.334	3.862	3.026	2.064	1.149
0.07	13.183	8.017	5.666	4.062	2.877	2.01
0.072	10.09	5.98	4.39	3.282	2.405	1.565
0.085	10.811	6.269	4.723	2.748	1.799	1.162
0.07	20.538	13.598	12.025	9.418	6.553	5.086
0.071	14.682	8.725	6.654	4.84	3.358	2.497
0.068	14.199	9.327	7.312	4.915	3.52	2.353
0.075	10.315	5.893	4.554	3.304	2.175	1.231
0.096	11.642	6.39	4.733	2.379	1.461	0.997
0.07	18.107	10.982	8.881	6.443	4.52	3.281
0.07	15.714	8.874	6.39	4.8	3.274	2.181
0.058	15.326	9.507	9.015	8.014	6.622	4.957
0.061	14.976	9.741	9.441	8.569	7.309	5.657
0.091	10.571	6.031	3.905	2.781	1.604	0.813
0.091	10.479	5.92	3.785	2.681	1.51	0.729
0.09	14.78	8.881	6.074	3.909	2.55	1.673
0.085	11.466	6.686	5.005	2.943	1.938	1.38
0.081	8.834	4.854	4.094	2.342	1.512	0.847
0.085	11.466	6.686	5.005	2.943	1.938	1.38
0.078	10.2	5.671	4.086	2.749	1.859	1.254
0.073	16.198	10.275	7.75	6.04	4.471	3.412
0.068	14.059	8.683	6.974	5.819	4.444	3.521
0.068	14.059	8.683	6.974	5.819	4.444	3.521
0.1	13.404	7.734	5.768	3.481	2.378	1.505
0.077	19.972	12.009	9.102	5.992	4.499	3.197
0.077	8.024	4.674	3.792	2.457	1.883	1.134
0.08	10.2	7.528	7.409	5.843	3.287	1.311
0.071	17.364	10.963	8.58	6.695	4.944	3.639
0.075	11.874	6.782	5.34	3.471	2.376	1.598
0.06	24.336	15.997	14.859	13.39	11.618	9.579
0.083	12.908	7.743	5.804	4.156	3.061	2.043
0.096	5.112	2.729	1.822	1.134	0.654	0.342
0.067	12.591	7.924	6.542	5.191	3.954	2.855
0.067	20.276	12.617	10.168	7.75	5.542	4.08
0.087	11.66	7.661	6.327	2.901	2.044	1.3
0.076	17.211	10.681	8.279	6.431	4.733	3.283
0.08	9.861	6.272	5.18	3.428	2.242	1.454
0.076	12.364	8.023	6.321	4.443	3.041	2.066
0.072	16.496	10.699	8.246	6.218	4.53	3.255
0.078	10.783	6.695	5.579	3.922	2.385	1.641
0.07	14.692	9.298	7.325	5.241	3.574	2.515
0.078	14.227	9.142	7.103	5.059	3.43	2.05
0.075	12.919	8.212	7.137	4.663	2.921	2.049
0.078	10.607	6.383	4.936	3.657	2.302	1.175
0.087	17.291	10.671	7.848	5.01	3.554	2.316
0.081	12.841	7.541	5.111	3.369	2.138	1.292
0.069	12.08	6.843	5.213	3.66	2.528	1.775
0.08	11.065	7.239	5.687	2.896	2.035	1.379
0.068	12.98	8.471	7.307	5.981	4.605	3.19

0.07	16.181	9.45	6.947	5.298	3.711	2.388
0.088	9.858	5.238	4.929	2.135	1.331	0.872
0.062	13.399	8.87	8.607	7.912	6.532	5.174
0.055	17.657	9.97	8.962	7.006	5.489	4.176
0.068	7.235	3.718	2.807	2.029	1.269	0.663
0.068	10.74	6.063	4.66	3.589	2.546	1.189
0.08	11.098	7.095	5.829	4.63	3.395	2.356
0.087	13.65	7.897	6.617	3.535	2.413	1.619
0.091	14.274	8.159	5.672	3.591	2.188	1.374
0.084	12.949	8.606	6.943	4.771	3.149	2.195
0.084	11.38	7.311	5.71	3.721	2.242	1.504
0.077	12.225	7.077	5.861	4.191	3.184	2.423
0.079	15.347	9.355	7.033	5.113	3.645	2.538
0.068	13.805	7.911	6.149	4.419	3.231	2.276
0.08	12.213	6.244	4.349	3.036	2.028	1.358
0.08	16.85	9.622	7.049	5.281	3.525	2.423
0.159	6.761	3.947	2.613	1.624	1.088	0.536
0.076	18.654	10.867	8.145	5.43	3.793	2.386
0.06	26.22	17.135	16.04	13.982	11.991	10.407
0.08	21.104	11.379	8.654	6.425	4.225	2.651
0.053	34.016	20.35	17.179	14.76	12.845	10.488
0.056	15.625	9.965	7.845	6.733	5.634	4.617
0.071	12.653	7.367	5.516	3.855	2.811	1.969
0.071	12.893	7.268	5.049	3.672	2.609	1.506
0.074	10.29	5.764	4.334	3.071	2.069	1.295
0.073	17.022	10.752	8.619	6.794	5.107	3.617
0.068	13.998	7.703	6.173	4.008	2.612	1.959
0.075	11.939	6.449	5.069	3.429	2.343	1.479

X0Av	X1Av	X2Av	X3Av	X4Av	X5Av	X0sol
0.585	0.325	0.169	0.092	0.049	0.029	13
0.62	0.347	0.197	0.097	0.052	0.029	18.113
0.532	0.281	0.149	0.078	0.042	0.022	18.845
0.56	0.295	0.159	0.085	0.047	0.025	8.268
0.524	0.271	0.135	0.066	0.033	0.017	11.544
0.638	0.525	0.322	0.24	0.161	0.104	12.121
0.605	0.333	0.175	0.098	0.054	0.034	21.562
0.496	0.25	0.124	0.063	0.031	0.015	6.975
0.579	0.297	0.156	0.079	0.041	0.022	15.604
0.624	0.353	0.21	0.105	0.059	0.035	13.38
0.543	0.311	0.169	0.09	0.05	0.027	29.886
0.731	0.395	0.218	0.116	0.072	0.031	25.38
0.618	0.338	0.194	0.103	0.056	0.031	14.656
0.606	0.321	0.166	0.087	0.046	0.025	21.181
0.579	0.318	0.201	0.107	0.063	0.037	18.87
0.588	0.325	0.206	0.109	0.064	0.038	17.999
0.59	0.301	0.157	0.084	0.042	0.024	10.129
0.518	0.275	0.141	0.08	0.043	0.024	21.087
0.601	0.336	0.201	0.104	0.057	0.033	14.251
0.593	0.338	0.195	0.116	0.067	0.041	14.82
0.606	0.312	0.165	0.076	0.038	0.02	16.016
0.55	0.284	0.153	0.087	0.046	0.026	13.759
0.57	0.296	0.159	0.084	0.041	0.022	15.483
0.594	0.33	0.213	0.113	0.066	0.04	17.129
0.625	0.358	0.217	0.115	0.067	0.037	18.899
0.593	0.327	0.184	0.105	0.063	0.036	19.776
0.625	0.363	0.222	0.118	0.064	0.037	15.786
0.576	0.303	0.16	0.09	0.048	0.025	31.585
0.615	0.32	0.18	0.091	0.046	0.026	15.527
0.641	0.361	0.219	0.11	0.061	0.034	17.079
0.584	0.321	0.183	0.09	0.047	0.024	28.295
0.629	0.337	0.188	0.096	0.051	0.028	31.585
0.64	0.342	0.206	0.094	0.049	0.027	14.044
0.648	0.375	0.212	0.12	0.07	0.042	15.242
0.632	0.339	0.197	0.105	0.055	0.031	23.889
0.596	0.346	0.2	0.118	0.069	0.04	19.399
0.604	0.354	0.2	0.116	0.065	0.038	16.364
0.584	0.274	0.141	0.07	0.035	0.018	10.456
0.655	0.381	0.236	0.141	0.087	0.051	22.104
0.544	0.286	0.145	0.076	0.04	0.02	18.052
0.538	0.282	0.142	0.073	0.038	0.021	22.786
0.539	0.286	0.147	0.077	0.041	0.022	12.535
0.637	0.35	0.214	0.127	0.072	0.042	30.389
0.581	0.304	0.165	0.088	0.044	0.024	14.069
0.564	0.303	0.159	0.084	0.045	0.025	20.761
0.578	0.328	0.182	0.106	0.06	0.036	18.284
0.565	0.318	0.175	0.102	0.058	0.034	18.654
0.575	0.329	0.181	0.107	0.064	0.039	22.828
0.598	0.347	0.209	0.122	0.074	0.048	21.604

0.605	0.348	0.201	0.115	0.067	0.039	23.75
0.558	0.317	0.172	0.097	0.056	0.032	22.706
0.573	0.324	0.174	0.102	0.059	0.035	32.413
0.58	0.323	0.176	0.099	0.056	0.033	22.706
0.582	0.331	0.183	0.107	0.061	0.034	21.181
0.566	0.32	0.173	0.098	0.057	0.034	20.068
0.558	0.336	0.186	0.107	0.061	0.035	26.801
0.575	0.321	0.181	0.098	0.056	0.032	27.612
0.579	0.329	0.18	0.099	0.058	0.034	18.845
0.584	0.33	0.18	0.105	0.062	0.037	27.206
0.545	0.296	0.158	0.09	0.051	0.031	21.483
0.552	0.321	0.17	0.09	0.045	0.023	16.863
0.573	0.325	0.18	0.104	0.06	0.036	17.784
0.586	0.345	0.199	0.116	0.067	0.038	20.38
0.592	0.345	0.197	0.116	0.067	0.038	19.552
0.584	0.336	0.187	0.109	0.063	0.038	17.784
0.649	0.411	0.247	0.151	0.093	0.06	16.968
0.593	0.33	0.188	0.109	0.06	0.035	19.562
0.616	0.417	0.232	0.134	0.076	0.045	13.845
0.639	0.343	0.207	0.111	0.06	0.035	14.165
0.663	0.362	0.219	0.118	0.066	0.039	15.673
0.636	0.405	0.236	0.139	0.076	0.05	13.639
0.659	0.359	0.218	0.118	0.065	0.039	15.673
0.575	0.339	0.18	0.096	0.051	0.026	16.871
0.589	0.304	0.166	0.087	0.045	0.025	13.198
0.633	0.374	0.218	0.134	0.085	0.065	12.864
0.554	0.292	0.157	0.083	0.045	0.024	17.648
0.565	0.315	0.168	0.097	0.055	0.031	16.071
0.529	0.271	0.13	0.067	0.034	0.018	13.569
0.545	0.301	0.159	0.082	0.044	0.023	15.974
0.666	0.404	0.234	0.127	0.076	0.044	21.154
0.622	0.32	0.19	0.093	0.048	0.027	11.268
0.58	0.296	0.156	0.083	0.042	0.023	18.637
0.633	0.335	0.185	0.1	0.057	0.028	10.966
0.602	0.318	0.175	0.093	0.048	0.026	16.311
0.737	0.517	0.327	0.209	0.157	0.11	11.691
0.577	0.358	0.215	0.13	0.075	0.044	12.716
0.605	0.34	0.185	0.101	0.056	0.031	13.786
0.593	0.327	0.184	0.105	0.063	0.036	19.776
0.588	0.305	0.164	0.086	0.045	0.024	14.613
0.599	0.317	0.176	0.097	0.054	0.027	14.828
0.614	0.322	0.196	0.101	0.057	0.032	23.309
0.532	0.287	0.149	0.078	0.042	0.021	11.828
0.501	0.263	0.136	0.073	0.038	0.021	11.544
0.62	0.357	0.21	0.129	0.078	0.047	19.027
0.603	0.342	0.195	0.116	0.068	0.04	38.319
0.619	0.333	0.193	0.108	0.061	0.035	21.319
0.612	0.332	0.195	0.101	0.054	0.031	13.502
0.626	0.343	0.196	0.102	0.061	0.029	18.449
0.584	0.307	0.176	0.09	0.049	0.026	42.75

0.634	0.425	0.247	0.126	0.073	0.04	21.993
0.579	0.317	0.172	0.094	0.051	0.03	21.165
0.55	0.286	0.154	0.079	0.042	0.023	23.859
0.55	0.294	0.144	0.079	0.043	0.02	16.861
0.664	0.42	0.249	0.161	0.102	0.06	10.812
0.597	0.33	0.167	0.094	0.05	0.027	15.035
0.551	0.287	0.151	0.081	0.043	0.023	29.283
0.642	0.439	0.249	0.161	0.102	0.075	16.13
0.546	0.304	0.164	0.093	0.051	0.028	13.665
0.558	0.301	0.162	0.09	0.046	0.025	12.958
0.604	0.349	0.193	0.113	0.065	0.04	17.485
0.594	0.327	0.19	0.107	0.06	0.033	16.38
0.652	0.362	0.227	0.127	0.076	0.045	19.613
0.527	0.296	0.154	0.093	0.053	0.029	15.786
0.516	0.266	0.142	0.075	0.04	0.022	13.839
0.522	0.281	0.147	0.078	0.041	0.023	11.828
0.553	0.284	0.142	0.072	0.037	0.019	14.983
0.475	0.242	0.118	0.06	0.03	0.015	5.983
0.585	0.336	0.186	0.112	0.064	0.037	18.993
0.552	0.298	0.16	0.089	0.046	0.025	12.251
0.571	0.474	0.255	0.125	0	0	6.327
0.611	0.389	0.229	0.143	0.092	0.055	25.834
0.577	0.344	0.189	0.106	0.059	0.03	16.371
0.644	0.357	0.213	0.126	0.073	0.041	27.499
0.615	0.384	0.24	0.16	0.099	0.068	9.027
0.595	0.315	0.174	0.094	0.05	0.028	14.613
0.523	0.273	0.139	0.07	0.033	0.017	13.121
0.645	0.348	0.216	0.116	0.067	0.039	13.759
0.586	0.33	0.187	0.101	0.058	0.031	22.922
0.613	0.369	0.213	0.125	0.07	0.043	25.13
0.605	0.362	0.209	0.122	0.069	0.042	24.597
0.601	0.33	0.185	0.107	0.061	0.036	15.974
0.554	0.295	0.154	0.084	0.044	0.024	18.734
0.627	0.429	0.241	0.143	0.082	0.049	13.845
0.578	0.315	0.173	0.097	0.053	0.03	14.353
0.635	0.355	0.22	0.113	0.065	0.036	11.422
0.573	0.304	0.168	0.093	0.048	0.027	25.614
0.58	0.296	0.156	0.083	0.042	0.023	18.637
0.553	0.296	0.158	0.087	0.045	0.024	14.983
0.576	0.32	0.172	0.092	0.05	0.027	21.968
0.493	0.261	0.139	0.08	0.044	0.022	11.707
0.512	0.279	0.152	0.085	0.047	0.025	9.422
0.59	0.298	0.158	0.077	0.041	0.022	19.715
0.568	0.303	0.166	0.092	0.051	0.028	15.905
0.555	0.301	0.162	0.09	0.047	0.026	13.828
0.579	0.342	0.194	0.113	0.065	0.035	11.19
0.558	0.319	0.172	0.099	0.057	0.033	15.815
0.527	0.277	0.148	0.079	0.042	0.023	15.364
0.604	0.354	0.189	0.11	0.068	0.04	9.138
0.564	0.296	0.158	0.082	0.041	0.022	17.715

0.659	0.363	0.194	0.116	0.063	0.031	12.836
0.561	0.304	0.161	0.088	0.047	0.026	16.234
0.559	0.301	0.155	0.08	0.042	0.022	16.56
0.58	0.301	0.162	0.084	0.044	0.023	15.983
0.582	0.314	0.166	0.088	0.046	0.026	21.424
0.604	0.353	0.212	0.116	0.062	0.034	17.379
0.635	0.373	0.206	0.111	0.07	0.04	15.251
0.621	0.347	0.191	0.11	0.061	0.038	13.01
0.589	0.317	0.177	0.096	0.052	0.029	13.527
0.639	0.351	0.202	0.115	0.067	0.038	13.121
0.58	0.292	0.156	0.082	0.042	0.022	7.475
0.617	0.299	0.175	0.082	0.037	0.029	7.439
0.636	0.347	0.195	0.107	0.056	0.034	16.872
0.601	0.316	0.194	0.101	0.059	0.035	19.577
0.533	0.279	0.147	0.079	0.041	0.021	24.121
0.604	0.336	0.194	0.113	0.068	0.038	19.776
0.648	0.352	0.183	0.109	0.058	0.029	15.621
0.635	0.355	0.214	0.113	0.061	0.036	14.088
0.55	0.287	0.143	0.077	0.041	0.022	9.138
0.573	0.294	0.155	0.078	0.041	0.022	15.604
0.612	0.345	0.205	0.122	0.071	0.042	29.891
0.571	0.309	0.169	0.093	0.05	0.028	14.353
0.561	0.308	0.166	0.087	0.047	0.026	19.413
0.666	0.346	0.206	0.099	0.05	0.028	15.284
0.616	0.339	0.213	0.103	0.057	0.033	16.328
0.592	0.323	0.192	0.099	0.057	0.032	21.275
0.565	0.294	0.146	0.075	0.038	0.021	12.577
0.58	0.324	0.178	0.095	0.051	0.029	16.974
0.571	0.315	0.168	0.092	0.051	0.028	15.82
0.577	0.301	0.161	0.092	0.047	0.025	12.414
0.661	0.331	0.219	0.095	0.049	0.027	12.345
0.631	0.324	0.183	0.096	0.054	0.03	22.75
0.571	0.285	0.147	0.079	0.042	0.023	18.723
0.563	0.351	0.193	0.089	0.051	0.026	15.708
0.577	0.296	0.15	0.08	0.044	0.023	19.43
0.49	0.248	0.124	0.063	0.032	0.016	12.414
0.536	0.296	0.156	0.085	0.047	0.024	9.138
0.557	0.303	0.153	0.084	0.044	0.024	15.69
0.601	0.337	0.184	0.101	0.056	0.031	13.786
0.572	0.292	0.16	0.08	0.042	0.021	32.507
0.564	0.296	0.158	0.082	0.041	0.022	17.715
0.619	0.337	0.185	0.101	0.059	0.033	17.556
0.571	0.305	0.164	0.09	0.048	0.028	14.397
0.583	0.311	0.191	0.098	0.056	0.033	20.568
0.557	0.293	0.156	0.08	0.042	0.023	14.613
0.585	0.309	0.165	0.091	0.049	0.027	15.983
0.565	0.314	0.162	0.083	0.042	0.023	17.104
0.665	0.386	0.236	0.145	0.086	0.052	29.209
0.62	0.353	0.185	0.106	0.062	0.03	12.345
0.603	0.325	0.165	0.087	0.05	0.027	9.682

0.634	0.386	0.222	0.13	0.08	0.051	12.261
0.548	0.296	0.149	0.082	0.044	0.023	12.466
0.573	0.321	0.167	0.085	0.043	0.024	16.234
0.531	0.285	0.146	0.076	0.04	0.022	13.295
0.601	0.33	0.189	0.092	0.049	0.028	12.958
0.642	0.389	0.24	0.141	0.078	0.045	23.321
0.565	0.301	0.162	0.086	0.044	0.024	18.096
0.568	0.345	0.187	0.1	0.054	0.028	18.277
0.607	0.327	0.182	0.103	0.057	0.031	12.914
0.613	0.336	0.197	0.099	0.054	0.031	14.251
0.604	0.354	0.207	0.124	0.067	0.04	22.422
0.561	0.286	0.145	0.079	0.041	0.022	19.673
0.589	0.328	0.188	0.11	0.065	0.037	18.905
0.651	0.375	0.225	0.138	0.084	0.05	16.405
0.622	0.355	0.186	0.116	0.067	0.031	12.673
0.616	0.348	0.18	0.112	0.063	0.028	12.673
0.591	0.342	0.19	0.103	0.058	0.033	17.908
0.603	0.334	0.193	0.095	0.051	0.029	13.665
0.68	0.373	0.227	0.106	0.06	0.035	10.008
0.603	0.334	0.193	0.095	0.051	0.029	13.665
0.6	0.315	0.163	0.086	0.044	0.025	12.914
0.6	0.342	0.194	0.112	0.062	0.035	18.83
0.586	0.322	0.179	0.104	0.058	0.034	16.681
0.586	0.322	0.179	0.104	0.058	0.034	16.681
0.638	0.368	0.222	0.129	0.074	0.052	16.018
0.605	0.343	0.194	0.102	0.058	0.034	23.794
0.535	0.292	0.158	0.095	0.052	0.028	8.906
0.6	0.443	0.274	0.188	0.106	0.047	13.82
0.579	0.322	0.175	0.098	0.054	0.032	19.794
0.594	0.308	0.178	0.091	0.048	0.028	13.949
0.64	0.372	0.222	0.135	0.081	0.048	26.709
0.645	0.369	0.207	0.115	0.07	0.041	14.535
0.511	0.273	0.14	0.076	0.041	0.024	7.56
0.572	0.317	0.177	0.102	0.056	0.033	15.267
0.614	0.35	0.192	0.106	0.057	0.032	24
0.648	0.426	0.253	0.121	0.07	0.039	14.009
0.637	0.368	0.212	0.124	0.07	0.042	19.468
0.58	0.348	0.199	0.114	0.062	0.038	12.716
0.562	0.334	0.186	0.103	0.056	0.033	15.829
0.569	0.334	0.187	0.102	0.055	0.032	20.506
0.599	0.352	0.199	0.115	0.061	0.038	13.586
0.588	0.344	0.188	0.103	0.052	0.028	17.556
0.619	0.381	0.209	0.12	0.07	0.038	17.285
0.646	0.391	0.238	0.133	0.066	0.039	15.001
0.589	0.336	0.19	0.111	0.062	0.031	13.475
0.618	0.368	0.201	0.109	0.063	0.036	20.821
0.584	0.328	0.176	0.094	0.049	0.025	15.786
0.575	0.298	0.158	0.081	0.043	0.022	15.483
0.582	0.362	0.196	0.088	0.048	0.028	14.294
0.59	0.353	0.203	0.12	0.07	0.041	16.509

0.578	0.305	0.158	0.087	0.047	0.024	19.673
0.616	0.327	0.214	0.097	0.051	0.029	12.345
0.638	0.37	0.221	0.136	0.083	0.049	14.828
0.552	0.285	0.155	0.08	0.043	0.023	23.911
0.557	0.266	0.134	0.068	0.034	0.017	9.585
0.671	0.379	0.203	0.112	0.067	0.035	12.845
0.653	0.373	0.224	0.136	0.079	0.047	12.518
0.65	0.359	0.221	0.107	0.06	0.036	15.648
0.571	0.314	0.172	0.095	0.053	0.029	18.071
0.617	0.391	0.231	0.14	0.079	0.05	15.545
0.632	0.406	0.238	0.143	0.077	0.049	13.846
0.643	0.354	0.209	0.116	0.071	0.043	13.88
0.59	0.323	0.176	0.096	0.054	0.031	18.433
0.6	0.304	0.162	0.082	0.043	0.023	16.974
0.582	0.284	0.145	0.078	0.041	0.023	15.405
0.602	0.321	0.172	0.098	0.055	0.032	20.096
0.676	0.439	0.261	0.162	0.121	0.089	7.983
0.583	0.32	0.177	0.091	0.049	0.027	23.087
0.655	0.381	0.229	0.14	0.083	0.05	28.286
0.64	0.335	0.188	0.105	0.06	0.034	24.433
0.597	0.313	0.165	0.089	0.049	0.027	40.233
0.601	0.332	0.171	0.094	0.05	0.027	17.889
0.55	0.295	0.158	0.082	0.043	0.023	16.397
0.561	0.291	0.144	0.075	0.04	0.02	16.397
0.542	0.288	0.155	0.083	0.044	0.023	13.991
0.608	0.336	0.187	0.108	0.06	0.034	19.941
0.609	0.308	0.171	0.085	0.042	0.023	16.56
0.597	0.307	0.169	0.09	0.048	0.027	15.361

X1sol	X2sol	X3sol	X4sol	X5sol	XMOD	RDCHI
7.968	7.196	5.045	4.265	2.895	50.937	2.747
11.329	10.13	6.797	5.189	4.029	71.474	3.514
12.38	11.57	9.185	7.919	6.405	78.727	3.561
5.182	4.755	2.94	2.231	1.746	32.652	2.272
7.72	6.715	5.384	4.643	3.369	50.736	2.823
7.811	9.769	6.684	4.275	1.237	55.968	2.164
14.541	12.307	10.836	8.876	6.72	92.757	3.982
4.877	4.263	3.683	3.089	1.979	31.702	2.218
11.026	10.186	8.844	7.841	6.706	69.461	3.323
8.63	7.247	4.993	3.676	3.026	53.721	3.045
18.852	17.093	15.453	11.376	8.674	123.396	4.437
16.726	14.971	12.998	11.967	8.513	120.014	4.09
10.254	8.983	7.263	6.252	5.295	62.306	3.273
13.744	11.649	9.676	8.197	6.591	88.846	3.557
12.012	12.612	10.256	8.933	7.027	77.677	3.517
11.618	11.99	9.845	8.782	6.81	74.737	3.449
6.698	6.025	5.099	4.269	2.951	41.89	2.561
14.491	13.337	10.566	9.362	7.232	113.406	4.393
8.969	8.17	5.076	3.705	3.079	56.621	3.178
10.186	8.977	7.83	6.612	4.745	63.838	3.339
10.452	9.811	8.362	7.436	6.612	66.017	3.129
9.573	9.024	8.034	7.089	5.306	63.9	3.096
10.33	9.856	8.202	6.167	5.124	65.805	3.311
11.19	11.601	9.045	8.566	6.661	71.883	3.432
13.22	11.263	8.706	7.09	5.288	81.472	3.915
12.591	12.783	12.059	10.242	8.258	81.469	3.385
10.631	9.268	6.154	4.246	3.538	66.726	3.746
20.353	19.016	15.851	13.008	9.365	127.213	4.829
10.665	9.522	7.705	6.484	5.583	65.967	3.384
10.969	9.572	6.165	4.254	3.539	69.663	3.684
19.44	18.812	14.769	12.095	9.873	126.576	4.56
20.946	21.243	18.885	17.379	15.324	135.449	4.678
8.904	8.831	6.22	4.835	4.003	55.245	3.031
10.13	8.613	7.052	5.63	4.321	62.342	3.25
16.017	17.124	15.608	13.506	13.191	99.629	3.909
13.651	12.197	10.244	8.782	6.425	85.696	4.176
11.623	11.239	10.438	8.807	8.018	71.389	3.48
6.537	6.232	5.877	4.482	3.124	42.732	2.405
14.01	14.129	10.644	8.771	6.224	85.713	3.949
12.525	11.966	11.588	9.725	8.386	78.659	3.594
16.152	14.298	12.355	10.982	8.989	101.83	4.359
8.771	7.816	6.603	5.937	5.114	53.991	2.919
18.839	20.372	17.312	14.044	12.417	117.316	4.361
9.347	9.086	7.605	6.093	5.241	59.09	3.061
14.758	12.506	10.699	9.069	7.372	92.968	4.365
12.156	11.506	10.262	8.72	7.134	79.705	3.503
12.262	11.757	10.264	8.443	7.028	79.47	3.57
15.788	14.714	13.319	11.293	9.167	104.225	4.189
15.252	14.941	12.738	11.624	9.187	102.971	4.106

16.145	15.65	13.181	11.77	9.341	106.861	4.146
15.148	14.373	11.837	10.422	8.332	101.042	3.955
21.918	20.481	18.443	15.466	12.389	143.851	5.044
15.148	14.4	11.73	10.372	8.599	101.58	3.952
14.205	13.503	11.386	10.332	7.939	94.887	3.773
13.3	12.328	10.684	8.972	7.677	85.698	3.739
18.334	18.56	14.324	13.117	10.165	121.591	4.42
18.399	18.061	15.039	12.095	9.951	121.017	4.51
12.843	12.141	10.347	9.148	7.419	86.267	3.583
18.479	17.72	15.456	13.408	11.249	124.528	4.468
14.3	13.129	10.796	9.536	7.743	95.175	3.898
11.737	12.289	8.924	7.103	5.706	85.711	3.566
11.868	11.135	9.853	8.292	6.812	76.529	3.503
14.45	13.524	11.541	10.467	8.248	94.163	4.009
13.288	12.755	10.295	9.239	7.232	88.065	3.739
11.868	11.135	9.853	8.292	6.812	76.529	3.503
12.343	10.284	8.556	7.324	5.172	78.158	3.724
13.42	11.863	9.167	7.359	6.293	85.304	3.936
9.72	11.712	8.158	7.292	5.313	69.758	2.645
9.453	8.461	6.287	4.825	4.142	59.399	3.112
10.845	10.325	8.374	7.413	6.981	69.464	3.27
9.063	9.106	6.469	4.985	3.537	61.52	2.824
10.845	10.325	8.374	7.413	6.981	68.927	3.27
11.415	12.441	9.545	8.496	6.232	76.219	3.142
8.953	8.411	7.416	5.818	4.757	57.103	3.001
8.774	6.867	5.538	4.206	2.621	57.374	3.06
12.19	10.432	8.687	7.127	5.483	77.541	3.83
11.237	9.703	7.928	6.63	5.231	77.395	3.837
9.113	8.421	7.428	6.335	5.036	59.212	2.981
11.148	10.303	8.552	7.312	6.022	73.562	3.501
13.551	12.455	10.442	8.241	6.134	89.175	3.552
6.704	6.882	4.802	2.68	2.351	43.585	2.472
12.207	11.432	9.411	7.821	6.734	78.068	3.481
7.348	6.677	5.578	4.563	2.846	48.292	2.654
11.492	10.71	9.028	7.657	6.956	72.485	3.441
8.181	7.27	5.929	5.015	4.254	57.201	2.509
8.787	9.054	7.477	5.419	4.321	57.452	2.832
9.898	8.154	7.085	6.121	5.234	60.53	3.203
12.591	12.783	12.059	10.242	8.258	81.469	3.385
9.953	9.13	7.813	6.315	5.463	62.817	3.097
9.653	8.961	6.913	5.792	4.333	62.366	3.047
15	15.733	13.119	12.673	9.202	98.988	3.848
8.292	7.325	6.626	5.613	4.059	53.753	2.952
7.682	6.856	5.471	4.354	3.347	54.381	2.89
12.777	13.07	12.149	10.296	8.495	79.228	3.602
25.59	25.6	22.823	19.386	15.243	162.734	5.831
14.309	13.2	10.747	9.214	8.259	91.894	3.749
9.271	7.785	5.83	4.743	3.746	57.315	3.172
11.892	10.628	8.429	7.106	4.725	73.253	3.366
26.99	27.641	22.626	19.097	15.674	171.534	5.277

15.353	16.681	9.013	7.67	5.85	103.902	4.156
14.886	13.838	11.9	10.575	8.599	94.381	4.28
14.867	14.852	13.467	12.532	10.071	94.774	3.596
10.349	8.921	7.436	5.528	3.607	66.653	3.121
6.778	4.65	3.784	2.26	1.069	42.99	2.608
10.109	9.139	7.903	7.21	5.883	63.072	3.103
20.282	19.006	17.692	15.347	13.195	132.317	4.708
11.141	11.787	6.647	6.368	3.686	77.681	3.249
9.165	8.021	6.137	4.623	3.758	56.72	3.202
8.665	7.636	6.01	4.463	3.468	53.959	3.051
12.275	10.223	8.639	6.914	5.653	75.341	3.794
11.328	10.753	8.619	7.526	6.264	72.607	3.412
12.544	13.507	10.623	9.556	7.474	77.21	3.557
10.741	8.739	6.999	5.644	4.506	86.004	3.85
9.762	8.698	7.05	5.964	3.822	68.763	3.353
8.288	7.306	6.634	5.921	4.633	54.11	2.974
10.13	9.019	8.05	6.826	5.383	67.724	3.192
3.788	3.377	2.199	1.737	0.971	28.073	1.975
13.65	12.199	10.606	9.291	8.394	95.519	4.105
8.182	7.176	5.928	4.292	3.29	53.574	2.965
3.838	4.992	2.598	0	0	28.364	1.509
18.133	16.203	13.631	11.113	9.155	128.444	5.01
10.97	11.556	8	6.939	4.343	74.606	3.163
17.169	17.562	14.346	12.642	10.436	106.718	4.058
5.684	5.012	3.505	2.87	1.975	35.614	2.291
10.137	9.62	8.638	7.772	7.088	64.028	3.117
8.651	7.479	6.306	5.133	3.532	56.942	2.975
8.326	8.045	5.834	3.328	2.622	52.02	2.933
14.584	13.834	11.736	9.398	6.951	94.292	3.896
16.923	16.394	13.032	10.684	7.224	109.332	4.492
16.89	16.123	12.521	10.274	7.68	110.58	4.456
11.131	10.35	9.145	7.923	6.517	69.462	3.519
12.241	11.121	8.027	6.162	4.943	87.741	3.82
9.72	11.712	8.158	7.292	5.313	69.758	2.645
10.126	10.036	9.695	8.996	8.573	63.386	3.101
7.003	6.513	4.491	3.161	2.235	43.172	2.63
18.03	16.198	13.45	10.811	9.213	112.246	4.937
12.207	11.432	9.411	7.821	6.734	78.03	3.481
10.075	9.245	8.111	6.368	4.96	62.722	3.288
15.699	13.802	12.393	10.621	8.259	97.601	4.345
7.575	7.262	5.051	4.636	3.292	52.774	2.781
6.182	5.849	4.487	3.908	2.628	42.236	2.488
12.845	11.864	9.622	8.835	6.503	82.476	3.485
10.275	9.406	7.645	5.56	4.267	64.824	3.274
9.092	8.047	6.687	4.811	3.715	56.285	3.061
7.657	6.984	5.963	4.921	3.375	51.093	2.706
11.345	10.19	8.929	7.485	6.253	82.595	3.666
10.813	9	7.97	6.46	4.865	67.115	3.424
5.664	4.888	3.805	3.079	1.569	35.964	2.263
11.969	11.387	9.97	8.513	7.51	79.595	3.505

8.079	6.808	5.346	3.967	2.749	50.071	2.817
11.131	10.023	7.932	6.526	5.217	74.654	3.622
11.007	10.104	8.551	7.2	5.915	76.141	3.373
10.636	10.137	8.498	6.853	6.131	68.673	3.135
14.996	12.895	11.275	9.843	7.488	94.979	4.153
12.016	12.677	10.969	9.425	7.195	80.502	3.286
9.786	8.075	6.009	4.421	3.577	64.27	3.058
8.688	7.44	6.327	5.001	4.012	54.308	2.895
9.827	8.898	7.858	7.046	6.382	60.648	3.158
8.592	7.688	6.333	5.01	3.555	53.198	2.953
5.166	4.634	4.042	3.411	2.193	33.965	2.218
4.037	3.851	1.981	1.016	0.544	26.238	1.868
11.019	9.517	8.074	6.126	5.218	67.435	3.181
12.604	12.578	10.498	9.893	7.689	82.599	3.513
15.634	14.733	11.23	8.927	7.114	100.014	4.341
12.581	12.865	11.906	10.185	8.181	78.426	3.391
9.816	8.22	6.98	4.749	3.646	63.389	3.095
9.113	7.683	5.237	3.584	3.131	57.827	3.234
5.664	4.915	3.715	2.965	1.965	37.581	2.274
11.026	10.186	8.844	7.841	6.706	72.337	3.354
20.343	19.918	15.034	12.646	10.338	127.379	5.037
10.126	10.036	9.695	8.996	8.573	63.386	3.101
13.58	12.595	10.911	9.749	8.123	89.222	3.841
9.307	8.831	5.647	4.49	3.548	58.928	3.042
10.26	10.323	7.068	5.185	4.728	64.626	3.325
14.139	14.145	11.785	11.265	9.125	90.483	3.821
8.041	7.417	6.129	5.048	4.151	50.961	2.721
12.043	12.14	11.262	10.315	9.804	74.73	3.492
11.115	10.722	10.38	9.855	9.157	69.16	3.234
8.113	7.284	6.135	4.415	3.646	50.95	2.856
7.304	7.553	4.851	3.09	2.513	46.854	2.641
15.502	14.867	13.05	11.708	10.091	102.285	4.04
11.828	10.55	8.774	7.405	5.861	76.659	3.228
10.776	11.224	6.796	5.691	4.816	72.459	3.204
12.311	11.005	8.891	7.567	5.851	79.351	3.365
8.059	7.574	5.754	4.506	2.892	53.659	2.964
5.647	4.913	4.087	2.439	1.867	35.968	2.286
10.613	9.47	8.224	6.897	5.688	70.392	3.315
9.898	8.154	7.085	6.121	5.234	59.993	3.203
20.638	20.111	16.048	13.199	11.037	132.316	4.888
11.969	11.387	9.97	8.513	7.51	79.595	3.505
12.188	11.14	10.006	8.239	6.54	79.844	3.536
10.254	9.295	8.547	7.53	5.94	63.836	3.331
13.601	13.927	11.573	10.957	8.371	87.709	3.787
9.937	9.238	7.668	6.175	5.655	63.034	3.104
11.026	10.87	10.734	9.916	9.187	69.824	3.177
11.63	10.097	8.699	7.393	6.166	72.513	3.457
19.395	19.645	16.441	14.413	12.851	120.654	4.525
7.484	6.534	5.598	4.946	2.324	47.455	2.502
6.22	5.305	3.468	2.831	2.063	39.211	2.526

8.917	7.464	6.621	5.715	4.772	54.057	3.026
8.108	7.18	6.324	5.658	3.539	51.198	2.709
11.236	9.475	8.289	7.242	5.931	69.573	3.395
9.232	8.228	7.106	6.463	5.091	57.336	3.013
8.665	7.7	5.74	4.52	3.484	54.382	3.104
16.593	16.17	14.286	10.902	9.524	107.925	4.214
12.631	11.45	9.735	8.47	7.358	79.613	3.651
12.754	12.928	9.311	7.704	6.456	83.895	3.473
8.381	7.733	6.409	4.94	3.32	53.458	2.868
8.969	8.17	5.076	3.705	3.079	56.741	3.178
14.116	13.144	9.678	7.934	5.713	88.978	3.879
13.601	12.019	10.882	8.991	6.943	87.577	3.946
12.171	12.32	11.54	9.716	7.819	75.962	3.364
10.86	11.009	9.806	8.608	6.942	66.314	3.256
8.168	6.611	5.292	3.516	2.367	50.942	2.948
8.168	6.611	5.292	3.516	2.367	51.396	2.948
12.203	9.945	7.785	5.918	4.602	75.779	3.825
9.165	8.053	5.99	4.691	4.018	56.928	3.186
6.037	5.606	4.138	3.313	1.933	36.797	2.312
9.165	8.053	5.99	4.691	4.018	56.928	3.186
8.402	7.728	6.099	5.152	4.09	53.25	2.813
13.756	11.921	10.552	9.111	8.099	87.471	3.942
11.707	10.366	9.632	8.161	7.117	72.964	3.538
11.707	10.366	9.632	8.161	7.117	72.964	3.538
10.507	9.214	6.118	5.028	3.417	69.589	3.426
15.884	14.112	10.616	9.334	7.352	98.929	4.233
6.2	5.913	4.922	4.306	3.322	49.422	2.66
9.328	11.373	8.052	5.563	2.77	65.22	2.575
14.204	12.752	11.518	9.954	7.951	91.948	4.245
9.72	8.798	6.927	5.774	4.282	61.124	3.344
18.216	17.578	15.521	13.917	12.06	113.516	4.39
9.63	8.259	6.783	5.534	4.163	59.342	3.138
4.715	4.17	3.034	2.335	1.342	30.023	2.09
10.686	9.771	8.48	7.461	5.73	67.655	3.507
16.544	16.031	13.758	11.668	9.463	108.04	4.106
9.203	9.888	5.179	4.258	3.249	61.34	2.903
13.636	11.703	10.064	8.439	6.29	86.507	3.798
8.792	9.234	6.844	5.485	3.908	59.07	2.88
11.382	11.203	8.974	7.408	5.784	74.235	3.401
14.824	13.154	11.688	9.944	8.154	94.315	3.998
9.22	9.624	7.634	5.679	4.352	61.635	2.914
12.213	11.547	9.147	7.577	6.401	81.217	3.552
11.757	11.509	8.605	6.484	4.708	78.091	3.361
10.263	10.494	7.483	5.51	4.388	67.76	3.136
9.046	8.265	7.199	5.256	3.27	57.827	3.007
14.24	13.407	9.455	7.8	6.177	93.655	3.946
10.741	8.734	7.07	5.459	4.106	67.302	3.569
10.364	9.647	8.331	6.644	5.723	65.813	3.126
9.793	10.419	6.493	5.177	4.298	66.171	3.06
11.668	11.845	10.714	9.369	7.389	78.598	3.237

13.601	12.019	10.882	8.991	6.943	87.577	3.946
7.277	7.754	4.369	3.37	2.634	45.819	2.67
9.949	10.059	9.402	7.735	6.534	60.85	3.117
14.773	15.244	13.529	12.206	9.796	94.132	3.595
6.126	5.695	5.464	4.06	2.704	40.062	2.355
7.773	6.943	5.834	5.3	2.544	49.765	2.502
9.069	8.342	7.362	6.116	4.693	58.288	3.057
10.455	10	6.947	5.652	4.435	69.127	3.351
12.059	10.367	7.963	5.711	4.281	76.111	3.916
10.792	10.648	7.84	6.406	4.452	71.017	3.295
9.275	9.051	6.43	5.009	3.336	61.345	2.956
9.059	8.216	6.499	5.185	4.329	56.749	2.964
12.992	11.715	9.977	8.335	6.651	82.682	4
11.725	11.084	9.675	8.515	7.223	75.1	3.354
10.083	8.637	7.249	5.991	4.639	65.285	3.183
13.507	11.732	10.401	7.822	6.079	86.199	4.02
4.719	3.581	2.262	1.553	0.955	29.467	2.048
15.418	13.522	10.761	9.249	6.798	96.685	4.153
19.049	18.886	15.858	14.037	12.578	118.405	4.523
15.842	13.179	11.514	8.892	6.245	100.567	4.199
27.385	25.868	24.48	22.805	20.882	172.921	5.111
12.699	11.536	10.924	10.235	9.4	78.752	3.503
11.075	10.049	8.075	7.242	5.971	69.001	3.344
11.151	9.671	8.47	7.455	5.394	69.822	3.386
9.062	8.016	6.757	5.377	4.126	59.408	2.991
14.413	13.535	12.035	10.633	8.658	92.363	4.154
10.952	10.383	8.433	6.454	5.808	67.981	3.372
9.685	9.326	7.156	5.888	4.343	61.91	3.072

RDSQ	ISIZ	IAC	AAC	IDE	IDM	IDDE
107.715	166.465	47.836	1.45	2.933	6.888	3.572
167.496	296.423	76.758	1.476	3.627	7.853	3.887
224.24	219.66	64.836	1.581	3.307	8.135	4.21
53.686	86.439	31.909	1.595	2.587	5.569	2.664
104.573	128.382	49.964	1.851	2.957	6.679	3.75
79.986	128.382	50.625	1.875	2.451	6.352	2.36
262.706	369.16	96.657	1.559	3.652	8.517	4.457
54.942	53.303	25.693	1.835	1.951	5.352	2.922
191.153	179.525	53.932	1.541	2.935	7.668	4.061
108.164	219.66	55.014	1.342	3.279	7.015	3.614
405.803	529.128	137.976	1.662	3.715	9.4	4.972
273.223	354.413	90.681	1.511	3.747	8.607	4.503
164.766	240.215	49.735	1.13	3.028	7.516	3.63
243.925	303.58	87.435	1.65	3.181	8.389	4.593
210.357	240.215	79.14	1.799	3.456	7.981	4.324
199.254	233.329	75.864	1.764	3.368	7.869	4.252
89.058	122.211	39.138	1.505	2.554	6.329	3.325
369.922	339.763	111.076	1.915	3.678	9.165	4.196
115.017	219.66	61.494	1.5	3.508	7.138	3.722
160.35	240.215	61.931	1.408	3.228	7.482	3.916
187.954	226.477	66.039	1.572	2.909	7.674	4.096
144.092	122.211	52.379	2.015	2.909	7.218	3.682
161.824	166.465	54.691	1.657	3.316	7.462	3.821
185.383	219.66	70.601	1.722	3.374	7.736	4.002
219.235	361.775	76.762	1.258	3.368	8.258	4.134
289.398	332.475	81.876	1.436	3.152	8.361	4.423
143.316	289.294	68.599	1.345	3.794	7.551	3.971
447.347	497.999	121.907	1.543	3.892	9.601	4.589
173.978	247.133	57.626	1.281	3.208	7.636	3.88
145.682	310.764	74.125	1.373	3.878	7.679	4.176
395.586	413.947	117.565	1.729	3.681	9.33	4.734
521.296	536.955	131.22	1.562	3.777	9.584	5.24
133.682	226.477	56.435	1.344	3.156	7.183	3.471
151.706	275.121	63.456	1.295	3.207	7.499	3.749
419.388	467.161	98.62	1.315	3.143	8.955	4.139
232.66	347.076	87.648	1.486	3.879	8.259	3.852
230.717	303.58	68.46	1.292	3.055	7.91	3.72
94.474	110.039	42.82	1.784	2.294	6.363	3.807
263.462	459.5	86.148	1.164	3.72	8.503	4.457
254.139	226.477	66.039	1.572	3.21	8.129	4.624
319.431	303.58	86.837	1.638	3.695	8.794	4.234
139.001	147.207	43.584	1.453	2.602	7.098	3.281
476.908	592.209	117.391	1.29	3.836	9.419	5.162
145.872	153.58	49.957	1.612	3.063	7.187	3.787
252.755	325.212	82.961	1.481	3.923	8.469	4.64
196.035	199.421	74.76	1.967	3.39	7.868	4.002
207.119	226.477	76.626	1.824	3.476	7.981	4.324
281.307	275.121	95.884	1.957	3.768	8.602	4.632
254.467	233.329	89.969	2.092	3.731	8.404	4.513

301.077	325.212	105.343	1.881	3.694	8.71	4.688
264.167	247.133	91.269	2.028	3.606	8.535	4.64
465.529	444.235	137.417	1.909	4.07	9.639	5.124
264.677	261.066	94.318	2.007	3.662	8.523	4.482
244.82	247.133	87.24	1.939	3.536	8.327	4.209
229.176	254.084	81.95	1.782	3.585	8.204	4.236
350.516	325.212	106.666	1.905	3.904	9.038	4.292
361.6	347.076	113.7	1.927	3.867	9.137	4.939
206.839	199.421	77.991	2.052	3.389	8.002	4.404
346.342	310.764	112.24	2.079	3.916	9.039	4.892
252.15	247.133	87.133	1.936	3.614	8.425	4.582
223.009	212.877	78.578	1.964	3.369	8.122	4.027
196.035	219.66	73.418	1.791	3.39	7.868	4.002
244.97	247.133	82.181	1.826	3.642	8.309	4.138
214.374	226.477	78.968	1.88	3.565	8.094	3.825
196.035	233.329	75.864	1.764	3.39	7.868	4.002
184.521	317.975	75.956	1.381	3.432	7.871	3.918
218.499	296.423	81.333	1.564	3.67	8.195	4.134
123.656	104.042	54.126	2.353	2.634	6.913	3.41
130.751	199.421	52.711	1.387	3.013	7.219	3.432
164.919	212.877	60.68	1.517	3.059	7.51	4.297
104.436	147.207	59.127	1.971	3.13	6.854	3.617
164.919	206.131	56.013	1.436	3.059	7.51	4.297
185.824	166.465	65.108	1.973	3.005	7.654	3.823
134.907	147.207	48.884	1.629	2.987	7.034	3.642
95.556	166.465	53.736	1.628	3.345	6.819	3.735
193.225	233.329	67.56	1.571	3.727	7.943	4.484
205.911	268.078	67.108	1.398	3.665	8.067	4.027
150.144	140.881	51.033	1.76	2.808	7.235	4.037
212.17	226.477	72.422	1.724	3.17	7.904	3.887
224.982	354.413	99.28	1.655	3.435	8.236	4.236
80.054	116.096	40.068	1.603	2.789	6.295	3.093
214.034	219.66	66.949	1.633	3.266	8.02	4.324
85.734	104.042	40.228	1.749	2.649	6.313	2.896
199.461	226.477	62.708	1.493	3.064	7.788	4.176
80.014	140.881	56.627	1.953	2.623	6.331	2.664
116.002	140.881	50.842	1.753	2.964	6.868	2.676
154.558	226.477	51.311	1.222	2.954	7.378	3.422
289.398	332.475	81.876	1.436	3.152	8.361	4.423
158.702	166.465	52.359	1.587	2.839	7.391	3.922
133.126	147.207	51.85	1.728	3.001	7.217	3.827
286.7	261.066	92.504	1.968	3.506	8.533	4.107
124.047	140.881	51.888	1.789	2.907	6.871	3.69
126.374	122.211	43.351	1.667	2.962	7.049	3.837
271.743	361.775	76.759	1.258	3.294	8.226	4.459
647.259	812.152	160.387	1.359	4.532	10.165	5.245
262.724	317.975	87.656	1.594	3.341	8.47	4.418
128.425	212.877	51.152	1.279	3.056	7.214	2.945
205.333	310.764	72.889	1.35	3.06	8.053	3.364
774.797	754.18	163.968	1.477	3.945	10.48	5.431

217.72	325.212	97.755	1.746	4.082	8.351	4.254
272.8	310.764	89.008	1.648	3.836	8.472	4.548
346.888	325.212	91.065	1.626	3.177	8.773	4.414
157.689	192.75	64.479	1.743	3.206	7.642	3.88
65.766	199.421	52.163	1.373	3.084	6.265	2.236
173.897	226.477	59.997	1.428	2.922	7.528	4.011
499.087	459.5	110.512	1.493	3.7	9.537	4.916
124.048	179.525	70.515	2.015	3.565	7.286	3.784
126.79	166.465	44.407	1.346	3.354	7.152	3.722
119.777	160	43.339	1.354	3.128	7.027	3.614
195.635	303.58	68.866	1.299	3.576	7.967	4.054
171.98	172.974	58.467	1.72	3.192	7.64	3.914
257.061	376.569	82.409	1.308	3.386	8.215	4.431
222.134	268.078	89.888	1.873	3.562	8.338	4.012
175.16	172.974	64.339	1.892	3.021	7.664	3.459
136.537	128.382	39.249	1.454	2.892	7.052	3.17
183.931	186.117	64.701	1.797	3.055	7.651	4.096
41.235	43.02	26.265	2.189	2.037	5.021	2.725
270.52	325.212	95.383	1.703	3.689	8.514	4.015
123.505	153.58	44.871	1.447	3.083	7.026	3.503
24.029	33.219	16.855	1.685	1.557	4.284	1.842
392.545	568.43	131	1.489	4.292	9.363	5.144
155.53	160	65.317	2.041	3.174	7.496	3.554
405.661	544.798	107.63	1.266	3.553	9.168	4.919
63.915	140.881	40.865	1.409	2.412	5.876	2.918
188.024	226.477	59.997	1.428	2.768	7.543	4.107
123.404	153.58	57.335	1.85	3.057	7.031	3.684
111.287	212.877	51.411	1.285	3.21	7.005	3.197
283.691	429.05	110.947	1.585	3.598	8.629	4.825
291.87	413.947	111.325	1.637	4.142	8.829	4.396
278.599	361.775	106.763	1.75	4.074	8.748	4.741
194.077	261.066	67.968	1.446	3.273	7.745	3.936
224.308	247.133	84.181	1.871	3.557	8.339	4.209
123.656	116.096	57.69	2.308	2.634	6.913	3.41
204.214	212.877	57.925	1.448	2.61	7.557	3.88
86.615	166.465	41.301	1.252	2.986	6.474	3.374
345.14	413.947	99.834	1.468	4.179	9.086	4.703
214.034	219.66	66.949	1.633	3.266	8.02	4.324
162.989	186.117	53.272	1.48	3.273	7.463	4.107
303.906	354.413	88.201	1.47	3.726	8.698	4.563
105.683	110.039	44.529	1.855	2.932	6.682	2.75
79.777	98.107	38.559	1.753	2.582	6.087	3.085
246.45	282.193	81.076	1.622	3.023	8.29	4.459
149.697	172.974	50.626	1.489	3.386	7.455	3.916
132.478	166.465	44.407	1.346	3.11	7.198	3.827
95.885	122.211	50.648	1.948	2.837	6.499	3.774
201.187	206.131	77.318	1.983	3.533	7.975	4.023
171.669	166.465	47.315	1.434	3.228	7.635	3.187
64.572	122.211	40.908	1.573	2.295	5.887	3.252
244.222	254.084	79.832	1.735	3.219	8.135	3.844

104.205	206.131	52.72	1.352	3.015	6.872	3.382
190.048	240.215	77.022	1.75	3.535	7.846	4.252
220.092	240.215	78.508	1.784	3.194	8.024	4.404
171.142	153.58	55.757	1.799	2.831	7.541	4.011
267.17	303.58	85.921	1.621	3.692	8.51	4.231
193.482	186.117	69.954	1.943	3.16	7.77	4.176
120.286	192.75	63.585	1.719	3.297	7.171	3.616
127.6	206.131	53.289	1.366	2.81	7.077	3.419
169.949	206.131	51.178	1.312	2.728	7.401	4.322
123.705	212.877	54.166	1.354	2.959	7.05	3.461
54.942	53.303	25.693	1.835	1.951	5.352	2.922
34.603	86.439	28.775	1.439	2.261	4.997	2.725
185.569	282.193	61.572	1.231	2.837	7.827	3.116
226.708	254.084	80.649	1.753	3.332	8.121	3.825
285.813	317.975	97.819	1.779	4.012	8.756	4.597
270.102	332.475	75.551	1.325	3.163	8.251	4.236
131.694	226.477	68.312	1.626	3.289	7.333	3.584
113.464	240.215	60.76	1.381	3.555	7.135	3.827
64.488	92.239	38.69	1.842	2.412	5.876	3.252
204.647	186.117	58.198	1.617	2.949	7.802	4.437
407.414	482.543	112.145	1.456	3.993	9.442	5.095
204.214	212.877	57.925	1.448	2.61	7.557	3.88
289.087	303.58	88.547	1.671	3.319	8.459	4.237
134.233	247.133	62.282	1.384	3.225	7.348	3.584
160.824	275.121	69.252	1.413	3.413	7.599	3.971
272.452	289.294	85.338	1.673	3.433	8.443	4.582
120.892	140.881	48.087	1.658	2.701	6.912	3.572
263.48	282.193	69.103	1.382	2.947	8.044	4.404
230.203	240.215	62.511	1.421	2.8	7.81	4.524
115.008	153.58	42.219	1.362	3.025	6.855	3.97
97.892	179.525	51.795	1.48	2.902	6.694	3.203
308.889	332.475	90.714	1.591	3.576	8.614	4.89
213.993	233.329	71.274	1.658	2.865	8.075	3.654
153.375	166.465	61.73	1.871	3.076	7.514	3.785
221.726	254.084	75.077	1.632	3.079	8.172	4.162
110.216	104.042	44.923	1.953	3.145	6.837	3.617
63.747	104.042	37.233	1.619	2.531	5.856	3.022
192.071	219.66	71.091	1.734	3.166	7.773	4.089
154.558	219.66	46.71	1.139	2.954	7.378	3.422
460.387	505.754	126.003	1.575	4.175	9.605	5.141
244.222	254.084	79.832	1.735	3.219	8.135	3.844
193.737	233.329	73.622	1.712	3.455	7.857	4.168
175.982	199.421	55.693	1.466	3.089	7.494	4.202
257.678	261.066	83.28	1.772	3.478	8.328	4.307
158.262	153.58	52.923	1.707	2.826	7.393	3.922
233.343	240.215	64.918	1.475	2.72	7.822	4.35
200.718	240.215	66.311	1.507	3.163	7.913	4.137
478.877	672.479	128.541	1.273	3.698	9.455	4.967
104.697	172.974	53.106	1.562	2.607	6.743	3.078
67.912	122.211	38.886	1.496	2.909	6.043	3.239

134.881	233.329	47.746	1.11	2.734	7.083	2.837
121.887	140.881	48.087	1.658	2.641	6.921	3.146
189.151	233.329	62.18	1.446	3.081	7.794	3.154
149.481	153.58	47.957	1.547	2.718	7.25	3.09
117.807	199.421	55.68	1.465	3.216	7.006	3.837
296.852	391.454	103.44	1.591	3.742	8.706	4.5
234.52	261.066	74.507	1.585	3.246	8.138	4.47
214.277	233.329	75.174	1.748	3.067	8.051	3.974
114.484	153.58	50.171	1.618	2.984	6.862	3.337
115.017	219.66	61.494	1.5	3.508	7.138	3.722
253.166	398.93	89.119	1.35	3.654	8.532	4.282
247.474	275.121	82.562	1.685	3.664	8.293	4.423
254.43	296.423	70.363	1.353	3.165	8.133	4.393
210.676	317.975	63.736	1.159	3.096	7.765	4.263
99.329	199.421	54.322	1.43	3.263	6.833	3.617
99.329	192.75	54.205	1.465	3.263	6.833	3.617
180.546	296.423	71.047	1.366	3.699	7.97	4.324
127.328	212.877	54.638	1.366	3.28	7.168	3.932
74.02	153.58	35.502	1.145	2.441	6.121	2.316
127.328	212.877	54.638	1.366	3.28	7.168	3.932
116.893	147.207	48.081	1.603	2.853	6.892	3.69
234.283	296.423	81.333	1.564	3.601	8.192	4.357
209.308	268.078	67.601	1.408	3.267	7.886	4.252
209.308	268.078	67.601	1.408	3.267	7.886	4.252
130.155	233.329	74.361	1.729	3.665	7.429	3.821
294.745	421.488	96.989	1.406	3.724	8.812	4.741
96.95	86.439	44.074	2.204	2.819	6.495	3.457
114.221	134.606	55.249	1.973	2.695	6.913	2.822
274.95	332.475	90.051	1.58	3.817	8.476	4.707
147.087	206.131	55.207	1.416	3.263	7.328	3.922
431.588	592.209	118.63	1.304	3.633	9.233	4.596
142.833	254.084	60.386	1.313	3.101	7.361	3.684
48.306	64	24.084	1.505	2.151	5.339	2.722
180.397	233.329	63.604	1.479	3.267	7.615	3.914
322.205	376.569	107.745	1.71	3.591	8.823	4.802
113.056	199.421	63.208	1.663	3.17	7.024	3.308
227.105	332.475	83.161	1.459	3.479	8.222	4.162
113.875	134.606	53.823	1.922	3.021	6.861	3.617
172.008	186.117	64.211	1.784	3.281	7.622	3.789
262.204	275.121	77.885	1.589	3.493	8.443	3.729
125.368	153.58	57.911	1.868	3.038	7.036	3.837
209.007	226.477	65.797	1.567	3.4	7.995	4.054
173.872	254.084	78.684	1.711	3.32	7.756	3.882
142.875	219.66	67.363	1.643	3.231	7.336	4.022
121.556	147.207	46.129	1.538	3.152	7.011	3.461
216.021	325.212	90.558	1.617	3.815	8.283	4.182
151.425	247.133	67.482	1.5	3.645	7.569	4.369
171.901	172.974	56.868	1.673	2.837	7.541	4.011
132.056	166.465	65.023	1.97	3.197	7.174	3.682
181.224	166.465	65.551	1.986	3.099	7.641	4.061

247.474	303.58	87.163	1.645	3.664	8.293	4.423
96.657	179.525	51.04	1.458	2.929	6.688	3.031
187.053	275.121	57.804	1.18	2.943	7.509	4.297
346.653	325.212	91.065	1.626	3.19	8.771	3.976
84.028	92.239	38.587	1.837	2.192	6.145	2.565
104.697	172.974	55.861	1.643	2.607	6.743	3.078
119.152	153.58	46.974	1.515	2.989	6.856	3.97
146.144	247.133	76.234	1.694	3.417	7.46	3.749
173.798	275.121	74.371	1.518	3.911	7.923	4.244
148.866	226.477	71.426	1.701	3.351	7.469	3.594
112.009	186.117	61.284	1.702	3.26	7.011	3.419
137.655	240.215	58.119	1.321	2.929	7.227	4.037
212.114	268.078	77.65	1.618	3.749	8.049	4.258
204.647	179.525	57.018	1.629	2.949	7.802	4.437
154.489	206.131	65.014	1.667	3.198	7.494	3.916
225.843	332.475	88.79	1.558	3.833	8.266	4.253
40.361	122.211	32.211	1.239	2.403	5.318	2.246
283.733	361.775	94.49	1.549	3.699	8.712	4.351
459.039	640.192	124.743	1.286	3.697	9.381	4.703
280.195	436.632	98.829	1.392	3.925	8.775	4.863
846.695	754.18	164.283	1.48	3.706	10.451	5.693
262.751	296.423	72.104	1.387	2.943	8.173	4.7
190.509	206.131	53.42	1.37	3.048	7.797	4.089
187.49	199.421	59.721	1.572	3.238	7.764	4.35
136.195	166.465	60.333	1.828	3.049	7.207	3.892
248.593	275.121	82.643	1.687	3.761	8.272	4.522
188.65	240.215	62.511	1.421	3.124	7.785	3.969
145.995	172.974	56.313	1.656	3.162	7.346	3.784

IDDM	IDET	IDMT	IVDE	IVDM	HVcpx	HDcpx
4.05	398.846	3650.561	1.58	3.954	2.785	1.577
4.547	1000.943	12313.719	1.563	4.475	3.441	1.669
4.669	1074.698	12950.483	1.549	4.601	3.111	1.698
3.431	142.3	924.463	1.539	3.356	2.548	1.42
3.963	354.79	3025.555	1.477	3.918	2.837	1.551
3.774	223.073	1784.866	1.414	3.547	2.302	1.521
4.875	1588.652	22134.589	1.549	4.824	3.426	1.73
3.307	87.805	561.937	1.295	3.266	2.093	1.397
4.433	678.032	7100.265	1.322	4.403	2.825	1.655
4.139	501.739	4987.937	1.436	4.086	3.13	1.584
5.292	2897.478	48944.195	1.552	5.192	3.499	1.81
4.917	1742.427	24933.171	1.492	4.869	3.522	1.739
4.36	635.908	6628.727	1.268	4.338	2.871	1.64
4.772	1202.36	15402.047	1.55	4.706	2.991	1.726
4.616	1036.702	12378.612	1.735	4.504	3.303	1.68
4.558	929.537	10741.171	1.759	4.454	3.222	1.67
3.783	232.399	1797.405	1.531	3.714	2.526	1.514
5.183	2449.305	39178.517	1.772	5.093	3.435	1.789
4.214	599.872	6352.711	1.524	4.149	3.329	1.594
4.369	677.843	7175.522	1.418	4.322	3.102	1.632
4.428	672.042	7029.509	1.539	4.344	2.795	1.658
4.226	497.39	4879.113	1.462	4.171	2.802	1.608
4.36	696.268	7275.108	1.51	4.305	3.201	1.629
4.495	853.56	9646.319	1.762	4.403	3.205	1.656
4.725	1182.228	15649.597	1.117	4.711	3.195	1.711
4.777	1191.543	13962.73	1.944	4.643	3.052	1.72
4.422	876.395	10775.774	1.293	4.398	3.58	1.635
5.397	3514.606	63759.403	1.562	5.311	3.676	1.825
4.424	741.142	7986.855	1.395	4.392	3.071	1.651
4.486	981.184	12669.968	1.422	4.442	3.666	1.647
5.253	2727.862	45288.489	1.634	5.192	3.417	1.804
5.403	3410.748	57024.148	1.743	5.313	3.59	1.821
4.218	539.655	5322.291	1.763	4.128	2.982	1.602
4.364	673.546	7183.715	1.461	4.311	3.071	1.636
5.056	1763.291	23496.767	1.914	4.962	2.975	1.775
4.782	1466.242	20762.503	1.299	4.752	3.659	1.7
4.551	843.261	9056.417	1.551	4.512	2.919	1.679
3.786	208.757	1641.638	1.432	3.664	2.329	1.521
4.873	1618.187	22763.766	1.806	4.781	3.455	1.728
4.675	1043.367	12022.478	1.651	4.614	3.095	1.696
5.019	1951.213	29250.49	1.301	4.989	3.455	1.754
4.148	398.1	3698.164	1.352	4.109	2.552	1.599
5.316	3145.831	50187.052	1.937	5.194	3.628	1.809
4.218	523.823	4952.118	1.529	4.153	2.957	1.603
4.883	1706.41	25932.297	1.159	4.862	3.676	1.72
4.558	935.645	10882.133	1.555	4.478	3.238	1.67
4.616	1042.686	12529.844	1.55	4.527	3.319	1.68
4.921	1752.076	25066.6	1.514	4.866	3.551	1.737
4.822	1514.73	20741.311	1.487	4.777	3.524	1.72

4.971	1832.39	26382.956	1.693	4.896	3.494	1.747
4.877	1568.532	21850.787	1.566	4.796	3.425	1.733
5.428	3850.086	71161.394	1.544	5.36	3.841	1.826
4.875	1593.183	22023.223	1.566	4.796	3.473	1.731
4.77	1336.646	17245.144	1.728	4.694	3.34	1.715
4.724	1258.168	16031.58	1.561	4.646	3.446	1.701
5.138	2459.52	38946.093	1.705	5.057	3.657	1.775
5.18	2575.743	41783.862	1.698	5.099	3.659	1.785
4.617	1016.747	12251.283	1.554	4.541	3.246	1.684
5.139	2467.201	39646.219	1.555	5.063	3.701	1.775
4.826	1467.319	19790.649	1.556	4.756	3.403	1.723
4.668	1094.855	13434.256	1.834	4.57	3.146	1.696
4.558	935.645	10882.133	1.555	4.478	3.238	1.67
4.772	1376.849	18112.754	1.432	4.736	3.449	1.711
4.664	1158.64	14488.926	1.549	4.601	3.361	1.692
4.558	935.645	10882.133	1.555	4.478	3.238	1.67
4.558	947.097	11460.6	0.974	4.551	3.239	1.67
4.714	1288.143	16947.755	1.277	4.698	3.438	1.701
4.066	358.222	3180.169	1.904	3.919	2.524	1.579
4.216	515.287	5183.258	1.384	4.178	2.876	1.61
4.359	642.382	6728.969	1.418	4.322	2.911	1.639
4.052	425.646	3879.253	1.713	3.962	3.021	1.569
4.359	642.382	6728.969	1.418	4.322	2.911	1.639
4.435	694.108	7286.2	1.868	4.324	2.868	1.652
4.141	457.029	4136.041	1.481	4.09	2.929	1.588
4.063	454.901	4527.652	1.29	4.021	3.161	1.559
4.613	1117.975	14225.158	1.323	4.583	3.558	1.673
4.68	1191.043	15795.56	1.542	4.619	3.46	1.684
4.222	480.155	4550.71	1.529	4.153	2.71	1.612
4.555	874.944	9753.562	1.483	4.504	3.033	1.677
4.726	1205.745	15179.029	1.561	4.623	3.286	1.707
3.781	253.76	1983.011	1.809	3.655	2.713	1.507
4.609	979.934	11420.358	1.554	4.541	3.137	1.688
3.785	241.053	1900.16	1.379	3.74	2.586	1.51
4.495	775.255	8426.113	1.302	4.469	2.958	1.666
3.774	238.696	1905.57	1.43	3.709	2.544	1.517
4.057	403.062	3543.785	1.735	3.975	2.893	1.571
4.288	561.282	5599.806	1.076	4.283	2.831	1.627
4.777	1191.543	13962.73	1.944	4.643	3.052	1.72
4.296	539.439	5365.728	1.441	4.248	2.738	1.628
4.223	513.242	5095.189	1.509	4.159	2.887	1.608
4.884	1525.087	20497	1.697	4.774	3.322	1.732
4.059	395.36	3449.149	1.379	4.023	2.845	1.571
4.143	453.114	4207.963	1.572	4.056	2.865	1.59
4.722	1156.131	13448.862	1.828	4.643	3.179	1.705
5.724	6484.864	146187.621	1.759	5.649	4.283	1.863
4.822	1356.42	17591.589	1.519	4.768	3.164	1.731
4.216	522.578	5309.534	1.19	4.196	2.918	1.609
4.616	918.092	11049.361	1.682	4.536	2.878	1.693
5.826	6520.924	131905.886	1.87	5.717	3.699	1.895

4.829	1657.244	25670.647	1.69	4.744	3.811	1.709
4.883	1668.646	24171.304	1.347	4.848	3.62	1.721
4.975	1575.663	20493.639	1.507	4.842	3.033	1.758
4.434	740.52	8238.057	1.573	4.322	3.064	1.65
3.78	280.648	2399.382	1.379	3.719	2.953	1.501
4.363	613.582	6210.927	1.691	4.295	2.828	1.642
5.362	3185.701	52111.357	1.479	5.311	3.513	1.82
4.295	677.429	7620.903	1.72	4.196	3.345	1.608
4.214	573.568	5779.186	1.384	4.178	3.201	1.597
4.143	478.636	4567.503	1.415	4.096	3.031	1.586
4.612	1072.796	13384.307	1.164	4.597	3.373	1.678
4.427	737.317	8075.621	1.395	4.392	3.053	1.651
4.721	1188.43	14179.171	1.893	4.619	3.215	1.704
4.773	1346.419	18609.69	1.716	4.689	3.323	1.716
4.43	697.891	7663.531	1.529	4.381	2.837	1.655
4.142	442.524	3998.525	1.481	4.09	2.818	1.591
4.427	705.817	7314.485	1.544	4.36	2.957	1.653
3.149	73.349	421.796	1.585	3.044	2.138	1.354
4.873	1604.589	22290.153	1.524	4.832	3.482	1.73
4.14	471.65	4398.482	1.53	4.076	2.953	1.587
2.78	32.69	179.948	1.149	2.522	1.72	1.254
5.33	3519.251	69975.329	1.742	5.246	3.93	1.797
4.362	666.489	7039.189	1.772	4.266	2.971	1.636
5.173	2366.21	34873.537	1.858	5.059	3.347	1.791
3.556	159.201	1134.124	1.551	3.47	2.363	1.463
4.364	581.283	5777.724	1.648	4.308	2.69	1.645
4.14	467.701	4443.316	1.53	4.076	2.913	1.587
4.148	491.135	4938.875	1.772	4.03	3.041	1.581
4.928	1673.215	23297.32	1.735	4.828	3.414	1.741
5.059	2323.461	38759.441	1.681	4.982	3.915	1.753
5.017	2151.048	34975.08	1.573	4.957	3.817	1.746
4.502	827.976	9255.379	1.422	4.454	3.142	1.657
4.773	1344.496	18279.609	1.686	4.698	3.344	1.717
4.066	358.222	3180.169	1.904	3.919	2.524	1.579
4.37	548.205	5380.923	1.664	4.308	2.573	1.646
3.874	313.553	2615.664	1.585	3.781	2.858	1.526
5.178	2783.155	48375.028	1.241	5.159	4.006	1.776
4.609	979.934	11420.358	1.554	4.541	3.137	1.688
4.363	687.385	7194.187	1.51	4.305	3.124	1.629
4.973	1848.075	27182.168	1.305	4.947	3.514	1.745
3.963	351.836	3000.013	1.561	3.895	2.798	1.551
3.67	201.361	1454.89	1.549	3.601	2.509	1.485
4.727	1061.023	13205.183	1.561	4.646	2.852	1.716
4.363	710.969	7693.511	1.545	4.294	3.214	1.628
4.221	531.789	5211.428	1.509	4.159	3.028	1.605
3.877	297.904	2398.199	1.506	3.82	2.791	1.531
4.616	1060.05	13007.624	1.673	4.546	3.342	1.679
4.424	745.604	8077.83	1.241	4.407	3.07	1.651
3.559	151.475	1118.462	1.585	3.459	2.27	1.465
4.668	1046.323	12073.048	1.526	4.598	3.091	1.699

4.061	409.989	3820.76	1.566	3.976	2.879	1.571
4.55	975.661	11509.493	1.459	4.507	3.372	1.667
4.614	958.223	10928.543	1.55	4.527	3.05	1.688
4.368	594.48	6176.432	1.51	4.305	2.731	1.644
4.879	1605.955	22679.365	1.295	4.851	3.492	1.728
4.495	799.537	8663.703	1.762	4.403	3.026	1.663
4.214	563.799	5765.228	1.524	4.149	3.124	1.601
4.136	429.914	4019.655	1.528	4.083	2.688	1.597
4.299	518.381	5084.801	1.188	4.278	2.678	1.63
4.148	452.772	4293.457	1.53	4.076	2.856	1.59
3.307	87.805	561.937	1.295	3.266	2.093	1.397
3.142	81.388	479.679	1.352	2.983	2.259	1.349
4.494	717.687	8171.4	1.644	4.423	2.668	1.674
4.676	1082.832	13237.858	1.744	4.565	3.156	1.695
5.015	2118.48	33553.056	1.556	4.943	3.801	1.748
4.726	1110.301	12813.493	1.912	4.606	3.055	1.709
4.292	624.907	6614.044	1.571	4.209	3.126	1.618
4.211	607.914	6463.918	1.403	4.168	3.401	1.594
3.552	159.201	1134.124	1.585	3.459	2.368	1.464
4.498	746.104	8074.873	1.422	4.454	2.831	1.668
5.329	3274.569	59964.648	1.583	5.275	3.718	1.81
4.37	548.205	5380.923	1.664	4.308	2.573	1.646
4.829	1347.641	16969.581	1.487	4.774	3.147	1.728
4.284	612.665	6355.628	1.581	4.19	3.043	1.622
4.431	788.406	8875.657	1.753	4.338	3.213	1.642
4.834	1393.917	18345.588	1.723	4.745	3.263	1.724
4.059	367.319	3248.807	1.566	3.968	2.6	1.58
4.612	884.078	9563.955	1.71	4.56	2.796	1.692
4.493	708.341	7271.471	1.721	4.435	2.698	1.671
4.055	411.461	3633.153	1.549	3.989	2.932	1.569
3.974	348.277	3059.346	1.805	3.843	2.775	1.552
4.925	1662.748	22612.063	1.633	4.868	3.387	1.739
4.617	859.597	10335.919	1.569	4.517	2.713	1.697
4.363	645.979	7032.818	1.705	4.283	2.886	1.639
4.67	1000.682	12184.719	1.577	4.578	2.918	1.705
4.062	427.692	3965.226	1.549	3.989	3.011	1.563
3.557	167.04	1159.576	1.585	3.459	2.525	1.459
4.493	800.879	8674.96	1.538	4.428	3.043	1.664
4.288	561.282	5599.806	1.076	4.283	2.831	1.627
5.427	3949.121	73682.372	1.694	5.333	3.928	1.821
4.668	1046.323	12073.048	1.526	4.598	3.091	1.699
4.558	953.515	11149.613	1.528	4.493	3.325	1.668
4.363	648.673	6564.843	1.342	4.334	2.951	1.635
4.783	1314.711	16871.926	1.728	4.691	3.32	1.713
4.296	537	5374.701	1.441	4.248	2.732	1.629
4.498	688.034	7094.244	1.805	4.421	2.646	1.672
4.549	873.096	9962.31	1.459	4.507	3.018	1.68
5.328	3031.983	48814.864	1.798	5.246	3.5	1.813
3.967	312.886	2683.669	1.805	3.843	2.498	1.564
3.663	226.939	1691.931	1.457	3.613	2.82	1.477

4.142	418.351	3888.456	0.803	4.134	2.656	1.597
4.06	359.131	3169.928	1.735	3.975	2.555	1.581
4.488	779.543	8643.458	1.371	4.458	2.945	1.669
4.22	464.767	4473.521	1.457	4.178	2.627	1.616
4.142	492.088	4813.355	1.415	4.096	3.034	1.582
4.965	1856.053	26953.245	1.574	4.918	3.551	1.748
4.671	1055.017	12532.716	1.37	4.637	3.082	1.699
4.615	920.24	10958.012	1.673	4.546	2.904	1.693
4.058	405.887	3630.02	1.549	3.989	2.854	1.569
4.214	599.872	6352.711	1.524	4.149	3.329	1.594
4.872	1589.703	22182.107	1.58	4.788	3.459	1.733
4.777	1384.981	18203.275	1.432	4.736	3.501	1.708
4.67	1028.602	11589.443	1.909	4.56	3.067	1.698
4.494	783.182	8169.229	1.837	4.407	2.996	1.662
4.059	443.829	4222.851	1.468	3.999	3.129	1.563
4.059	443.829	4222.851	1.468	3.999	3.129	1.563
4.608	1109.812	14234.578	1.21	4.589	3.535	1.679
4.219	560.96	5677.292	1.384	4.178	3.12	1.599
3.674	190.386	1432.406	1.549	3.555	2.383	1.492
4.219	560.96	5677.292	1.384	4.178	3.12	1.599
4.058	388.004	3432.012	1.549	3.989	2.778	1.576
4.713	1263.801	16023.27	1.038	4.719	3.399	1.701
4.557	901.74	10141.024	1.406	4.518	3.18	1.673
4.557	901.74	10141.024	1.406	4.518	3.18	1.673
4.365	769.606	9115.779	1.475	4.302	3.466	1.622
5.018	1966.407	29679.171	1.462	4.964	3.513	1.757
3.881	295.943	2396.587	1.806	3.781	2.693	1.529
4.062	366.557	3304.339	1.739	3.889	2.551	1.579
4.882	1660.307	23673.335	1.347	4.848	3.629	1.722
4.289	619.927	6382.266	1.295	4.266	3.117	1.617
5.217	2553.822	38834.554	1.716	5.152	3.455	1.794
4.297	589.235	6050.665	1.485	4.237	2.98	1.622
3.299	96.789	608.672	1.571	3.209	2.168	1.396
4.436	754.792	8231.97	1.437	4.388	3.131	1.645
5.014	1895.981	27229.229	1.681	4.943	3.45	1.759
4.139	485.084	4713.17	1.772	4.03	2.995	1.586
4.719	1221.26	15563.919	1.369	4.689	3.249	1.705
4.059	410.923	3670.628	1.735	3.975	2.921	1.569
4.432	757.8	8193.451	1.529	4.381	3.172	1.647
4.826	1418.311	18836.341	1.314	4.8	3.328	1.726
4.143	464.783	4319.937	1.788	4.043	2.921	1.588
4.61	1019.915	12128.342	1.554	4.541	3.217	1.684
4.497	840.053	9703.176	1.731	4.407	3.144	1.66
4.289	613.852	6301.602	1.734	4.207	3.079	1.619
4.141	482.309	4578.183	1.53	4.076	3.005	1.583
4.784	1441.976	20609.072	1.607	4.711	3.59	1.705
4.424	841.94	9885.688	1.293	4.398	3.485	1.638
4.367	595.786	6138.377	1.51	4.305	2.729	1.644
4.221	546.696	5459.189	1.763	4.128	3	1.6
4.433	715.789	7549.168	1.742	4.35	2.963	1.65

4.777	1384.981	18203.275	1.432	4.736	3.501	1.708
3.975	351.475	3129.763	1.805	3.843	2.747	1.55
4.366	618.069	6022.314	1.819	4.282	2.862	1.638
4.976	1582.169	20611.164	1.677	4.835	3.046	1.757
3.681	171.003	1296.496	1.526	3.574	2.276	1.496
3.967	312.886	2683.669	1.805	3.843	2.498	1.564
4.066	406.518	3654.389	1.166	4.043	2.901	1.567
4.365	717.592	7930.404	1.582	4.3	3.221	1.628
4.619	1173.422	16161.991	1.359	4.575	3.689	1.668
4.366	703.627	7573.319	1.582	4.3	3.203	1.63
4.133	498.732	4802.444	1.676	4.051	3.132	1.585
4.218	500.94	4841.954	1.657	4.146	2.844	1.611
4.676	1218.327	16122.174	1.239	4.65	3.549	1.681
4.498	746.104	8074.873	1.422	4.454	2.831	1.668
4.365	671.503	7089.796	1.545	4.294	3.08	1.635
4.784	1448.902	20682.396	1.47	4.727	3.608	1.701
3.286	108.126	696.662	1.522	3.197	2.366	1.394
4.973	1834.885	27200.291	1.477	4.918	3.466	1.747
5.291	2883.527	45863.118	1.743	5.222	3.501	1.807
5.017	2072.411	32449.094	1.65	4.94	3.705	1.75
5.809	5914.983	111675.369	1.784	5.727	3.528	1.893
4.672	956.607	11017.673	1.57	4.627	2.823	1.705
4.496	771.146	8514.259	1.478	4.443	2.904	1.668
4.497	819.199	9075.648	1.478	4.443	3.116	1.661
4.217	521.317	5051.806	1.568	4.14	2.973	1.607
4.783	1421.834	19389.769	1.264	4.757	3.586	1.703
4.492	790.369	8851.255	1.538	4.428	2.973	1.666
4.289	600.858	6024.028	1.571	4.201	3.034	1.621

Uindex	Vindex	Xindex	Yindex	IC0	TIC0	SIC0
37.744	0.458	0.68	1.369	1.45	47.836	0.287
67.017	0.356	0.564	0.957	1.476	76.758	0.259
43.961	0.258	0.39	0.757	1.581	64.836	0.295
19.301	0.466	0.692	1.351	1.595	31.909	0.369
25.98	0.335	0.504	0.972	1.851	49.964	0.389
47.429	0.981	1.368	3.298	1.875	50.625	0.394
55.266	0.22	0.344	0.606	1.559	96.657	0.262
13.24	0.49	0.666	1.66	1.835	25.693	0.482
29.526	0.256	0.376	0.794	1.541	53.932	0.3
41.87	0.364	0.574	0.982	1.342	55.014	0.25
86.479	0.235	0.361	0.672	1.662	137.976	0.261
58.198	0.216	0.339	0.591	1.511	90.681	0.256
31.848	0.276	0.41	0.826	1.13	49.735	0.207
60.827	0.35	0.516	1.078	1.65	87.435	0.288
41.862	0.235	0.365	0.652	1.799	79.14	0.329
39.298	0.242	0.373	0.681	1.764	75.864	0.325
21.161	0.394	0.565	1.241	1.505	39.138	0.32
64.323	0.193	0.299	0.546	1.915	111.076	0.327
46.068	0.328	0.532	0.848	1.5	61.494	0.28
32.189	0.242	0.373	0.678	1.408	61.931	0.258
33.972	0.311	0.45	0.981	1.572	66.039	0.292
27.649	0.277	0.415	0.82	2.015	52.379	0.429
32.271	0.243	0.375	0.677	1.657	54.691	0.329
36.931	0.237	0.368	0.661	1.722	70.601	0.321
46.396	0.231	0.355	0.661	1.258	76.762	0.212
42.044	0.264	0.387	0.827	1.436	81.876	0.246
42.721	0.212	0.352	0.533	1.345	68.599	0.237
81.452	0.183	0.285	0.511	1.543	121.907	0.245
34.228	0.26	0.393	0.762	1.281	57.626	0.233
63.128	0.287	0.477	0.715	1.373	74.125	0.239
69.574	0.197	0.302	0.566	1.729	117.565	0.284
58.038	0.144	0.222	0.41	1.562	131.22	0.244
33.702	0.306	0.47	0.865	1.344	56.435	0.249
39.081	0.302	0.462	0.862	1.295	63.456	0.231
40.9	0.2	0.29	0.645	1.315	98.62	0.211
42.97	0.152	0.249	0.385	1.486	87.648	0.253
30.014	0.233	0.341	0.725	1.292	68.46	0.226
21.021	0.45	0.624	1.511	1.784	42.82	0.389
55.375	0.214	0.337	0.583	1.164	86.148	0.187
33.709	0.212	0.317	0.633	1.572	66.039	0.292
47.84	0.16	0.252	0.437	1.638	86.837	0.286
25.321	0.327	0.468	1.053	1.453	43.584	0.296
66.326	0.183	0.281	0.523	1.29	117.391	0.198
27.805	0.275	0.415	0.804	1.612	49.957	0.325
47.568	0.147	0.243	0.374	1.481	82.961	0.255
39.28	0.237	0.367	0.664	1.967	74.76	0.375
41.841	0.23	0.359	0.638	1.824	76.626	0.338
49.443	0.18	0.284	0.491	1.957	95.884	0.349
44.74	0.18	0.284	0.492	2.092	89.969	0.386

51.776	0.189	0.295	0.524	1.881	105.343	0.324
55.073	0.225	0.35	0.628	2.028	91.269	0.369
73.799	0.149	0.236	0.405	1.909	137.417	0.309
55.189	0.224	0.349	0.622	2.007	94.318	0.361
49.48	0.24	0.368	0.682	1.939	87.24	0.353
47.022	0.225	0.352	0.623	1.782	81.95	0.323
62.16	0.178	0.281	0.485	1.905	106.666	0.328
64.643	0.179	0.282	0.492	1.927	113.7	0.328
41.648	0.238	0.367	0.67	2.052	77.991	0.391
62.159	0.174	0.275	0.471	2.079	112.24	0.361
52.334	0.226	0.352	0.631	1.936	87.133	0.353
44.054	0.248	0.378	0.715	1.964	78.578	0.369
39.28	0.237	0.367	0.664	1.791	73.418	0.334
42.41	0.188	0.294	0.52	1.826	82.181	0.333
44.314	0.226	0.352	0.626	1.88	78.968	0.349
39.28	0.237	0.367	0.664	1.764	75.864	0.325
39.209	0.218	0.342	0.596	1.381	75.956	0.239
46.995	0.211	0.334	0.574	1.564	81.333	0.274
28.136	0.393	0.567	1.243	2.353	54.126	0.52
33.319	0.318	0.478	0.937	1.387	52.711	0.264
31.893	0.272	0.407	0.809	1.517	60.68	0.285
38.115	0.409	0.628	1.152	1.971	59.127	0.402
31.893	0.272	0.407	0.809	1.436	56.013	0.272
34.201	0.289	0.428	0.877	1.973	65.108	0.391
25.702	0.283	0.423	0.835	1.629	48.884	0.332
38.547	0.32	0.522	0.817	1.628	53.736	0.323
42.135	0.194	0.314	0.506	1.571	67.56	0.29
44.729	0.192	0.311	0.498	1.398	67.108	0.25
27.515	0.309	0.449	0.96	1.76	51.033	0.362
33.562	0.236	0.352	0.705	1.724	72.422	0.32
58.105	0.301	0.463	0.859	1.655	99.28	0.28
27.96	0.451	0.676	1.312	1.603	40.068	0.345
41.385	0.266	0.399	0.788	1.633	66.949	0.305
21.26	0.357	0.527	1.071	1.749	40.228	0.387
31.504	0.241	0.358	0.729	1.493	62.708	0.277
27.588	0.493	0.712	1.54	1.953	56.627	0.402
28.378	0.338	0.506	0.996	1.753	50.842	0.361
29.607	0.286	0.423	0.871	1.222	51.311	0.227
42.044	0.264	0.387	0.827	1.436	81.876	0.246
29.563	0.3	0.438	0.932	1.587	52.359	0.315
33.415	0.322	0.485	0.941	1.728	51.85	0.352
46.94	0.204	0.315	0.577	1.968	92.504	0.354
23.687	0.29	0.432	0.863	1.789	51.888	0.368
30.863	0.337	0.504	0.992	1.667	43.351	0.355
35.621	0.213	0.318	0.637	1.258	76.759	0.212
76.089	0.092	0.151	0.236	1.359	160.387	0.197
51.773	0.265	0.396	0.79	1.594	87.656	0.276
33.353	0.308	0.466	0.892	1.279	51.152	0.24
50.856	0.342	0.507	1.044	1.35	72.889	0.235
100.536	0.165	0.251	0.479	1.477	163.968	0.217

66.405	0.198	0.332	0.489	1.746	97.755	0.301
42.012	0.142	0.231	0.371	1.648	89.008	0.286
51.183	0.262	0.383	0.823	1.626	91.065	0.28
57.671	0.421	0.643	1.204	1.743	64.479	0.335
48.622	0.602	0.962	1.574	1.373	52.163	0.262
31.798	0.304	0.444	0.947	1.428	59.997	0.265
55.702	0.152	0.231	0.444	1.493	110.512	0.241
50.198	0.313	0.513	0.796	2.015	70.515	0.393
33.888	0.272	0.429	0.733	1.346	44.407	0.267
31.003	0.298	0.459	0.835	1.354	43.339	0.271
41.887	0.21	0.333	0.566	1.299	68.866	0.227
34.191	0.255	0.386	0.744	1.72	58.467	0.338
40.132	0.226	0.342	0.66	1.308	82.409	0.219
61.474	0.269	0.419	0.746	1.873	89.888	0.335
34.031	0.271	0.403	0.818	1.892	64.339	0.372
25.594	0.297	0.438	0.903	1.454	39.249	0.306
34.15	0.291	0.43	0.885	1.797	64.701	0.348
14.143	0.597	0.819	1.921	2.189	26.265	0.611
47.003	0.185	0.29	0.507	1.703	95.383	0.293
30.999	0.317	0.482	0.909	1.447	44.871	0.292
14.382	1.159	1.415	4.656	1.685	16.855	0.507
77.182	0.134	0.224	0.335	1.489	131	0.23
39.099	0.312	0.475	0.902	2.041	65.317	0.408
64.244	0.23	0.346	0.689	1.266	107.63	0.198
21.698	0.527	0.746	1.694	1.409	40.865	0.29
27.586	0.287	0.411	0.933	1.428	59.997	0.265
30.998	0.315	0.48	0.899	1.85	57.335	0.373
42.11	0.361	0.574	0.961	1.285	51.411	0.242
58.014	0.231	0.358	0.65	1.585	110.947	0.259
67.99	0.169	0.279	0.429	1.637	111.325	0.269
64.779	0.172	0.283	0.438	1.75	106.763	0.295
31.891	0.209	0.323	0.589	1.446	67.968	0.26
61.482	0.275	0.427	0.766	1.871	84.181	0.341
28.136	0.393	0.567	1.243	2.308	57.69	0.497
24.894	0.281	0.395	0.944	1.448	57.925	0.272
31.312	0.416	0.637	1.165	1.252	41.301	0.248
57.104	0.128	0.21	0.329	1.468	99.834	0.241
41.385	0.266	0.399	0.788	1.633	66.949	0.305
32.303	0.244	0.378	0.684	1.48	53.272	0.286
45.774	0.157	0.248	0.426	1.47	88.201	0.249
25.945	0.342	0.511	1.003	1.855	44.529	0.405
19.146	0.393	0.569	1.211	1.753	38.559	0.393
46.145	0.293	0.428	0.916	1.622	81.076	0.287
39.48	0.275	0.432	0.748	1.489	50.626	0.293
33.563	0.311	0.474	0.892	1.346	44.407	0.267
23.582	0.349	0.519	1.03	1.948	50.648	0.414
41.858	0.217	0.342	0.592	1.983	77.318	0.375
34.214	0.255	0.386	0.741	1.434	47.315	0.284
21.661	0.539	0.759	1.749	1.573	40.908	0.335
37.706	0.242	0.36	0.731	1.735	79.832	0.314

38.017	0.412	0.631	1.167	1.352	52.72	0.256
39.392	0.222	0.348	0.605	1.75	77.022	0.321
41.403	0.278	0.415	0.837	1.784	78.508	0.327
31.707	0.3	0.438	0.937	1.799	55.757	0.363
47.099	0.179	0.282	0.484	1.621	85.921	0.283
36.634	0.272	0.408	0.805	1.943	69.954	0.376
45.704	0.379	0.592	1.036	1.719	63.585	0.33
30.559	0.367	0.533	1.153	1.366	53.289	0.259
25.787	0.277	0.399	0.88	1.312	51.178	0.248
30.88	0.323	0.488	0.937	1.354	54.166	0.254
13.24	0.49	0.666	1.66	1.835	25.693	0.482
22.491	0.832	1.186	2.527	1.439	28.775	0.333
44.35	0.372	0.539	1.194	1.231	61.572	0.218
44.207	0.25	0.382	0.714	1.753	80.649	0.317
64.688	0.182	0.297	0.473	1.779	97.819	0.308
39.871	0.258	0.379	0.795	1.325	75.551	0.227
49.645	0.385	0.6	1.058	1.626	68.312	0.302
46.071	0.322	0.523	0.827	1.381	60.76	0.253
21.691	0.535	0.756	1.721	1.842	38.69	0.419
31.434	0.256	0.375	0.793	1.617	58.198	0.313
66.055	0.142	0.226	0.384	1.456	112.145	0.232
24.894	0.281	0.395	0.944	1.448	57.925	0.272
39.245	0.204	0.306	0.61	1.671	88.547	0.292
49.34	0.415	0.633	1.185	1.384	62.282	0.252
42.401	0.273	0.43	0.742	1.413	69.252	0.252
44.524	0.208	0.319	0.594	1.673	85.338	0.295
28.082	0.387	0.558	1.219	1.658	48.087	0.341
29.053	0.229	0.33	0.739	1.382	69.103	0.245
28.189	0.267	0.379	0.88	1.421	62.511	0.26
28.462	0.328	0.496	0.947	1.362	42.219	0.275
34.493	0.437	0.659	1.258	1.48	51.795	0.289
43.631	0.178	0.276	0.502	1.591	90.714	0.273
50.628	0.377	0.547	1.204	1.658	71.274	0.305
38.898	0.314	0.474	0.919	1.871	61.73	0.371
54.068	0.354	0.522	1.095	1.632	75.077	0.295
28.66	0.286	0.449	0.778	1.953	44.923	0.432
21.875	0.502	0.727	1.526	1.619	37.233	0.358
36.564	0.274	0.409	0.814	1.734	71.091	0.324
29.607	0.286	0.423	0.871	1.139	46.71	0.213
85.465	0.165	0.264	0.44	1.575	126.003	0.249
37.706	0.242	0.36	0.731	1.735	79.832	0.314
39.37	0.228	0.356	0.626	1.712	73.622	0.316
27.913	0.239	0.358	0.705	1.466	55.693	0.279
42.368	0.203	0.314	0.573	1.772	83.28	0.319
29.545	0.299	0.437	0.93	1.707	52.923	0.345
28.136	0.275	0.388	0.922	1.475	64.918	0.27
38.766	0.273	0.406	0.826	1.507	66.311	0.276
59.056	0.166	0.254	0.483	1.273	128.541	0.191
33.986	0.544	0.771	1.783	1.562	53.106	0.307
25.042	0.415	0.636	1.151	1.496	38.886	0.318

25.355	0.31	0.448	0.984	1.11	47.746	0.205
27.999	0.401	0.571	1.309	1.658	48.087	0.341
36.321	0.28	0.413	0.854	1.446	62.18	0.266
27.35	0.32	0.459	1.035	1.547	47.957	0.312
31.204	0.278	0.437	0.75	1.465	55.68	0.279
51.764	0.184	0.289	0.507	1.591	103.44	0.264
37.688	0.227	0.341	0.673	1.585	74.507	0.285
41.116	0.278	0.411	0.848	1.748	75.174	0.322
28.436	0.327	0.494	0.947	1.618	50.171	0.327
46.068	0.328	0.532	0.848	1.5	61.494	0.28
68.906	0.28	0.436	0.779	1.35	89.119	0.223
42.605	0.184	0.29	0.499	1.685	82.562	0.3
37.772	0.255	0.376	0.782	1.353	70.363	0.237
31.704	0.254	0.376	0.77	1.159	63.736	0.2
38.361	0.357	0.567	0.948	1.43	54.322	0.272
38.361	0.357	0.567	0.948	1.465	54.205	0.281
51.71	0.244	0.39	0.647	1.366	71.047	0.24
33.819	0.277	0.435	0.751	1.366	54.638	0.257
24.567	0.532	0.756	1.696	1.145	35.502	0.231
33.819	0.277	0.435	0.751	1.366	54.638	0.257
28.208	0.356	0.524	1.08	1.603	48.081	0.327
40.267	0.193	0.301	0.531	1.564	81.333	0.274
33.718	0.222	0.337	0.645	1.408	67.601	0.252
33.718	0.222	0.337	0.645	1.408	67.601	0.252
54.338	0.301	0.496	0.757	1.729	74.361	0.319
63.988	0.213	0.335	0.585	1.406	96.989	0.23
23.652	0.346	0.518	1.01	2.204	44.074	0.51
37.635	0.512	0.74	1.613	1.973	55.249	0.41
41.966	0.146	0.236	0.384	1.58	90.051	0.271
29.976	0.239	0.37	0.668	1.416	55.207	0.268
52.745	0.17	0.258	0.494	1.304	118.63	0.2
36.224	0.312	0.474	0.9	1.313	60.386	0.238
16.456	0.57	0.788	1.869	1.505	24.084	0.376
29.961	0.207	0.321	0.58	1.479	63.604	0.273
53.993	0.203	0.312	0.581	1.71	107.745	0.286
41.774	0.396	0.613	1.103	1.663	63.208	0.317
46.77	0.234	0.361	0.663	1.459	83.161	0.25
28.447	0.32	0.486	0.921	1.922	53.823	0.4
34.397	0.245	0.376	0.695	1.784	64.211	0.345
44.404	0.202	0.311	0.578	1.589	77.885	0.283
30.972	0.323	0.489	0.935	1.868	57.911	0.377
41.636	0.244	0.374	0.693	1.567	65.797	0.291
45.173	0.289	0.447	0.81	1.711	78.684	0.31
36.381	0.302	0.463	0.852	1.643	67.363	0.307
31.136	0.299	0.461	0.832	1.538	46.129	0.313
62.419	0.228	0.372	0.586	1.617	90.558	0.278
42.658	0.239	0.387	0.616	1.5	67.482	0.273
31.706	0.303	0.441	0.95	1.673	56.868	0.329
33.803	0.293	0.455	0.81	1.97	65.023	0.391
34.295	0.274	0.411	0.813	1.986	65.551	0.394

42.605	0.184	0.29	0.499	1.645	87.163	0.287
34.558	0.421	0.641	1.197	1.458	51.04	0.284
27.845	0.268	0.392	0.828	1.18	57.804	0.21
51.219	0.26	0.38	0.811	1.626	91.065	0.28
18.906	0.458	0.632	1.543	1.837	38.587	0.418
33.986	0.544	0.771	1.783	1.643	55.861	0.323
23.806	0.26	0.398	0.737	1.515	46.974	0.306
39.443	0.263	0.417	0.707	1.694	76.234	0.308
52.398	0.2	0.336	0.494	1.518	74.371	0.27
39.368	0.277	0.433	0.759	1.701	71.426	0.315
41.901	0.387	0.603	1.065	1.702	61.284	0.329
33.298	0.351	0.518	1.07	1.321	58.119	0.242
38.477	0.16	0.261	0.411	1.618	77.65	0.29
31.434	0.256	0.375	0.793	1.629	57.018	0.318
39.145	0.308	0.47	0.879	1.667	65.014	0.315
50.253	0.18	0.295	0.46	1.558	88.79	0.267
26.744	0.804	1.155	2.461	1.239	32.211	0.264
61.081	0.213	0.335	0.582	1.549	94.49	0.261
56.924	0.165	0.252	0.478	1.286	124.743	0.195
81.198	0.24	0.387	0.629	1.392	98.829	0.226
75.011	0.144	0.215	0.438	1.48	164.283	0.218
33.364	0.242	0.35	0.776	1.387	72.104	0.243
36.368	0.279	0.414	0.849	1.37	53.42	0.259
36.672	0.256	0.389	0.739	1.572	59.721	0.299
33.488	0.331	0.497	0.973	1.828	60.333	0.362
38.032	0.147	0.237	0.383	1.687	82.643	0.3
36.426	0.269	0.402	0.805	1.421	62.511	0.26
36.304	0.32	0.484	0.927	1.656	56.313	0.326

CIC0	BIC0	IC1	TIC1	SIC1	CIC1	BIC1
3.595	0.277	3.043	100.407	0.603	2.002	0.582
4.224	0.253	3.463	180.051	0.607	2.238	0.594
3.776	0.275	3.711	152.15	0.693	1.647	0.645
2.726	0.348	3.284	65.684	0.76	1.038	0.716
2.904	0.368	3.93	106.117	0.827	0.825	0.783
2.88	0.39	3.158	85.263	0.664	1.597	0.657
4.395	0.254	3.16	195.949	0.531	2.794	0.516
1.972	0.432	3.236	45.303	0.85	0.571	0.762
3.588	0.277	3.255	113.915	0.635	1.875	0.584
4.016	0.244	3.334	136.705	0.622	2.023	0.607
4.713	0.259	3.258	270.413	0.511	3.117	0.507
4.396	0.246	3.364	201.842	0.57	2.543	0.547
4.329	0.197	2.81	123.628	0.515	2.65	0.491
4.078	0.278	3.694	195.766	0.645	2.034	0.623
3.661	0.316	3.897	171.486	0.714	1.562	0.684
3.662	0.311	3.753	161.381	0.692	1.673	0.662
3.195	0.301	3.061	79.582	0.651	1.64	0.612
3.943	0.314	3.895	225.931	0.665	1.963	0.638
3.858	0.273	3.502	143.602	0.654	1.855	0.638
4.052	0.249	3.125	137.509	0.572	2.334	0.554
3.82	0.279	3.273	137.481	0.607	2.119	0.58
2.686	0.385	3.873	100.702	0.824	0.827	0.741
3.387	0.304	3.611	119.162	0.716	1.433	0.661
3.636	0.307	3.601	147.632	0.672	1.757	0.641
4.672	0.206	2.861	174.542	0.482	3.069	0.468
4.396	0.239	3.437	195.903	0.589	2.396	0.573
4.327	0.233	3.147	160.497	0.555	2.525	0.544
4.761	0.234	3.847	303.891	0.61	2.457	0.583
4.211	0.222	2.856	128.524	0.52	2.636	0.494
4.382	0.235	2.998	161.913	0.521	2.757	0.514
4.359	0.27	3.763	255.855	0.618	2.325	0.587
4.83	0.236	3.806	319.716	0.595	2.586	0.574
4.049	0.241	3.166	132.99	0.587	2.226	0.567
4.32	0.225	2.816	137.962	0.501	2.799	0.489
4.914	0.206	3.105	232.902	0.499	3.123	0.486
4.397	0.245	2.825	166.696	0.48	3.057	0.466
4.436	0.219	2.906	154.031	0.507	2.822	0.492
2.801	0.365	3.031	72.755	0.661	1.554	0.621
5.045	0.185	2.828	209.24	0.455	3.382	0.449
3.82	0.271	3.972	166.829	0.737	1.42	0.686
4.089	0.266	3.77	199.821	0.658	1.958	0.612
3.454	0.273	2.733	81.987	0.557	2.174	0.514
5.218	0.194	2.772	252.208	0.426	3.736	0.417
3.343	0.3	3.566	110.561	0.72	1.388	0.664
4.326	0.242	3.466	194.085	0.597	2.342	0.566
3.281	0.354	4.028	153.083	0.768	1.219	0.725
3.568	0.322	4.071	170.968	0.755	1.322	0.718
3.658	0.329	4.243	207.927	0.756	1.371	0.714
3.334	0.362	4.227	181.766	0.779	1.199	0.731

3.926	0.311	4.023	225.264	0.693	1.785	0.664
3.464	0.348	4.597	206.869	0.837	0.895	0.79
4.261	0.295	4.18	300.976	0.678	1.99	0.646
3.548	0.343	4.368	205.291	0.786	1.187	0.747
3.553	0.336	4.276	192.404	0.779	1.216	0.741
3.742	0.307	4.154	191.064	0.752	1.37	0.715
3.903	0.309	4.261	238.625	0.734	1.546	0.691
3.956	0.311	4.526	267.037	0.769	1.357	0.73
3.196	0.368	4.428	168.26	0.844	0.82	0.795
3.676	0.342	4.521	244.135	0.786	1.234	0.744
3.556	0.334	4.424	199.094	0.806	1.068	0.764
3.357	0.344	3.558	142.309	0.669	1.764	0.623
3.567	0.317	3.925	160.931	0.733	1.432	0.695
3.666	0.312	3.973	178.769	0.723	1.519	0.68
3.512	0.331	4.087	171.669	0.758	1.305	0.719
3.662	0.311	3.844	165.29	0.708	1.582	0.678
4.4	0.233	2.606	143.33	0.451	3.175	0.44
4.136	0.264	3.176	165.151	0.557	2.524	0.536
2.17	0.471	3.588	82.532	0.793	0.935	0.718
3.861	0.253	3.045	115.716	0.58	2.203	0.554
3.805	0.272	3.064	122.555	0.576	2.258	0.549
2.936	0.381	3.585	107.555	0.731	1.322	0.693
3.849	0.257	3.161	123.26	0.598	2.125	0.566
3.071	0.361	3.588	118.407	0.711	1.456	0.657
3.277	0.31	3.578	107.332	0.729	1.329	0.682
3.416	0.309	3.597	118.691	0.713	1.448	0.683
3.855	0.273	3.491	150.11	0.643	1.935	0.607
4.187	0.238	3.068	147.242	0.549	2.517	0.523
3.098	0.338	3.836	111.23	0.79	1.022	0.736
3.668	0.304	3.753	157.617	0.696	1.64	0.662
4.252	0.278	3.154	189.267	0.534	2.752	0.53
3.041	0.33	3.303	82.587	0.711	1.34	0.68
3.725	0.285	3.716	152.357	0.694	1.642	0.649
2.775	0.364	3.501	80.532	0.774	1.022	0.728
3.899	0.261	3.36	141.121	0.623	2.032	0.587
2.905	0.398	2.718	78.824	0.56	2.14	0.554
3.105	0.334	2.651	76.881	0.546	2.207	0.505
4.171	0.216	2.914	122.372	0.54	2.479	0.516
4.396	0.239	3.437	195.903	0.589	2.396	0.573
3.458	0.292	3.177	104.855	0.63	1.867	0.586
3.179	0.329	3.33	99.904	0.679	1.577	0.635
3.586	0.335	4.123	193.781	0.742	1.432	0.702
3.069	0.347	3.892	112.881	0.801	0.966	0.756
3.033	0.328	3.017	78.437	0.642	1.684	0.593
4.672	0.207	2.736	166.911	0.461	3.194	0.451
5.523	0.194	2.833	334.302	0.412	4.05	0.405
4.188	0.265	3.398	186.916	0.588	2.383	0.564
4.043	0.23	2.61	104.385	0.49	2.712	0.47
4.405	0.227	3.176	171.524	0.552	2.579	0.533
5.317	0.211	3.745	415.691	0.551	3.049	0.535

4.062	0.288	3.352	187.725	0.577	2.455	0.553
4.107	0.273	3.372	182.103	0.586	2.383	0.558
4.181	0.268	4.081	228.525	0.703	1.727	0.673
3.467	0.315	3.805	140.776	0.73	1.405	0.689
3.875	0.264	2.5	94.983	0.476	2.748	0.48
3.964	0.253	3.434	144.213	0.637	1.959	0.608
4.716	0.232	3.658	270.674	0.589	2.552	0.568
3.115	0.375	3.807	133.26	0.742	1.322	0.708
3.699	0.25	2.808	92.651	0.557	2.237	0.521
3.646	0.254	2.904	92.941	0.581	2.096	0.546
4.429	0.218	2.893	153.328	0.505	2.835	0.486
3.368	0.312	3.292	111.915	0.647	1.796	0.598
4.669	0.214	2.805	176.694	0.469	3.173	0.459
3.712	0.327	3.494	167.7	0.626	2.091	0.61
3.195	0.347	3.664	124.567	0.72	1.424	0.671
3.301	0.279	2.755	74.382	0.579	2	0.529
3.373	0.326	3.837	138.143	0.742	1.333	0.697
1.396	0.56	3.085	37.02	0.861	0.5	0.79
4.104	0.283	3.222	180.444	0.555	2.585	0.535
3.507	0.274	3.083	95.58	0.622	1.871	0.583
1.636	0.487	2.646	26.464	0.797	0.675	0.765
4.971	0.227	2.918	256.775	0.452	3.542	0.444
2.959	0.38	4.114	131.635	0.823	0.886	0.765
5.143	0.194	2.965	252.054	0.463	3.444	0.455
3.449	0.287	2.551	73.969	0.525	2.307	0.52
3.964	0.254	3.392	142.477	0.629	2	0.604
3.105	0.356	3.973	123.174	0.802	0.981	0.766
4.037	0.235	3.008	120.309	0.565	2.314	0.551
4.544	0.257	3.147	220.264	0.513	2.983	0.51
4.45	0.26	3.56	242.056	0.585	2.528	0.565
4.181	0.283	3.689	225.049	0.622	2.241	0.596
4.108	0.249	3.106	145.991	0.559	2.448	0.535
3.621	0.325	3.653	164.379	0.665	1.839	0.635
2.336	0.457	3.604	90.096	0.776	1.04	0.714
3.874	0.259	3.422	136.877	0.643	1.9	0.613
3.793	0.24	2.897	95.604	0.574	2.147	0.556
4.619	0.229	3.516	239.113	0.578	2.571	0.549
3.725	0.285	3.765	154.357	0.703	1.593	0.657
3.69	0.268	3.295	118.608	0.637	1.875	0.596
4.437	0.236	3.424	205.438	0.58	2.483	0.551
2.73	0.382	2.585	62.039	0.564	2	0.532
2.707	0.377	2.732	60.107	0.613	1.727	0.588
4.022	0.276	3.609	180.441	0.639	2.035	0.613
3.598	0.273	3.147	107	0.619	1.94	0.576
3.699	0.25	2.952	97.406	0.585	2.093	0.547
2.752	0.397	3.95	102.702	0.84	0.75	0.805
3.303	0.351	3.913	152.605	0.74	1.372	0.693
3.611	0.257	3.068	101.246	0.608	1.976	0.551
3.127	0.327	2.738	71.192	0.583	1.962	0.57
3.788	0.3	3.709	170.597	0.671	1.815	0.641

3.934	0.249	2.912	113.562	0.551	2.374	0.537
3.709	0.309	3.471	152.705	0.636	1.989	0.612
3.675	0.313	3.673	161.612	0.673	1.786	0.644
3.156	0.336	3.493	108.277	0.705	1.461	0.652
4.107	0.267	3.787	200.719	0.661	1.941	0.624
3.227	0.349	4.139	148.998	0.801	1.031	0.743
3.491	0.321	3.532	130.696	0.678	1.677	0.659
3.919	0.25	2.946	114.911	0.557	2.339	0.54
3.973	0.235	3.037	118.44	0.575	2.248	0.544
3.968	0.247	2.989	119.554	0.562	2.333	0.544
1.972	0.432	3.522	49.303	0.925	0.286	0.829
2.883	0.328	2.946	58.929	0.682	1.375	0.671
4.412	0.21	2.805	140.267	0.497	2.839	0.479
3.77	0.305	3.722	171.216	0.674	1.801	0.647
4.003	0.292	4.232	232.781	0.732	1.549	0.694
4.507	0.221	3.275	186.661	0.561	2.558	0.546
3.766	0.294	3.291	138.236	0.61	2.101	0.596
4.079	0.249	3.171	139.504	0.581	2.289	0.571
2.55	0.394	3.361	70.587	0.765	1.031	0.719
3.553	0.289	3.579	128.856	0.692	1.591	0.639
4.81	0.222	3.605	277.613	0.575	2.661	0.549
3.874	0.259	3.669	146.771	0.689	1.653	0.657
4.057	0.28	3.705	196.386	0.647	2.023	0.62
4.108	0.247	2.869	129.091	0.522	2.623	0.511
4.201	0.247	3.187	156.153	0.568	2.428	0.556
3.999	0.28	3.754	191.461	0.662	1.918	0.629
3.2	0.321	3.729	108.127	0.768	1.129	0.721
4.262	0.235	3.47	173.482	0.615	2.174	0.59
4.039	0.248	3.651	160.647	0.669	1.808	0.637
3.592	0.259	3.255	100.909	0.657	1.699	0.618
3.649	0.28	2.805	98.167	0.547	2.325	0.531
4.241	0.26	3.827	218.113	0.656	2.006	0.624
3.769	0.289	3.563	153.224	0.657	1.863	0.622
3.174	0.343	3.203	105.71	0.635	1.841	0.587
3.891	0.281	3.597	165.48	0.651	1.926	0.619
2.57	0.392	3.969	91.287	0.877	0.555	0.797
2.905	0.344	3.436	79.022	0.76	1.088	0.731
3.624	0.309	3.639	149.189	0.679	1.719	0.648
4.218	0.202	2.935	120.322	0.548	2.423	0.52
4.747	0.24	4.101	328.066	0.649	2.221	0.626
3.788	0.3	3.709	170.597	0.671	1.815	0.641
3.714	0.298	3.611	155.272	0.665	1.815	0.627
3.782	0.26	3.593	136.55	0.685	1.654	0.638
3.783	0.302	3.916	184.072	0.705	1.638	0.667
3.247	0.319	3.341	103.58	0.674	1.613	0.624
3.984	0.259	3.435	151.156	0.629	2.024	0.603
3.952	0.262	3.118	137.182	0.571	2.342	0.542
5.386	0.188	2.316	233.96	0.348	4.342	0.343
3.526	0.3	2.609	88.709	0.513	2.478	0.501
3.205	0.305	3.162	82.211	0.673	1.538	0.644

4.316	0.199	2.271	97.672	0.419	3.155	0.407
3.2	0.321	3.286	95.298	0.676	1.572	0.636
3.98	0.252	2.812	120.917	0.518	2.614	0.491
3.407	0.289	2.617	81.142	0.528	2.337	0.489
3.783	0.268	3.495	132.792	0.666	1.753	0.64
4.431	0.255	3.262	212.055	0.542	2.76	0.522
3.969	0.271	3.485	163.799	0.627	2.07	0.595
3.678	0.304	3.722	160.026	0.686	1.705	0.647
3.336	0.311	3.523	109.216	0.711	1.431	0.676
3.858	0.273	3.44	141.056	0.642	1.917	0.626
4.694	0.22	3.162	208.664	0.523	2.883	0.514
3.93	0.284	3.719	182.208	0.662	1.896	0.627
4.347	0.229	3.383	175.92	0.593	2.317	0.573
4.623	0.196	2.664	146.503	0.461	3.118	0.451
3.818	0.265	3.036	115.363	0.578	2.212	0.563
3.744	0.273	3.1	114.692	0.595	2.11	0.579
4.334	0.231	3.295	171.364	0.578	2.405	0.558
3.956	0.247	3.164	126.555	0.594	2.158	0.571
3.809	0.225	2.541	78.767	0.513	2.413	0.499
3.956	0.247	3.164	126.555	0.594	2.158	0.571
3.304	0.308	3.52	105.597	0.717	1.387	0.676
4.136	0.263	3.193	166.058	0.56	2.507	0.536
4.177	0.241	3.614	173.475	0.647	1.971	0.618
4.177	0.241	3.614	173.475	0.647	1.971	0.618
3.697	0.309	3.357	144.333	0.619	2.07	0.599
4.703	0.223	3.406	235.048	0.558	2.702	0.54
2.118	0.469	3.509	70.174	0.812	0.813	0.746
2.834	0.391	3.464	96.996	0.721	1.343	0.687
4.253	0.259	3.42	194.926	0.586	2.413	0.56
3.87	0.253	3.56	138.846	0.674	1.725	0.636
5.204	0.197	2.899	263.804	0.445	3.609	0.438
4.211	0.231	2.88	132.467	0.521	2.644	0.508
2.495	0.348	2.75	44	0.688	1.25	0.636
3.947	0.262	3.32	142.752	0.612	2.106	0.588
4.267	0.274	3.664	230.847	0.613	2.313	0.587
3.585	0.307	3.403	129.316	0.648	1.845	0.627
4.374	0.242	3.192	181.961	0.547	2.641	0.529
2.885	0.37	3.567	99.877	0.742	1.24	0.687
3.386	0.319	2.947	106.089	0.57	2.223	0.526
4.025	0.265	2.823	138.304	0.503	2.792	0.47
3.086	0.352	3.655	113.315	0.738	1.299	0.689
3.826	0.272	3.621	152.071	0.671	1.772	0.629
3.813	0.299	3.513	161.577	0.636	2.011	0.613
3.715	0.293	3.355	137.546	0.626	2.003	0.598
3.369	0.292	3.402	102.045	0.693	1.505	0.646
4.19	0.269	3.403	190.586	0.586	2.404	0.565
3.992	0.263	3.731	167.908	0.679	1.761	0.655
3.415	0.306	3.382	114.974	0.665	1.706	0.619
3.074	0.37	3.931	129.71	0.779	1.114	0.739
3.058	0.363	4.127	136.2	0.818	0.917	0.754

4.083	0.276	3.497	185.341	0.611	2.231	0.586
3.671	0.278	3.242	113.486	0.632	1.887	0.618
4.435	0.205	2.625	128.645	0.468	2.989	0.456
4.181	0.268	4.117	230.525	0.709	1.691	0.679
2.555	0.389	3.368	70.729	0.767	1.024	0.712
3.444	0.315	2.69	91.464	0.529	2.397	0.516
3.439	0.288	3.176	98.461	0.641	1.778	0.603
3.798	0.303	2.95	132.761	0.537	2.542	0.527
4.097	0.26	3.497	171.346	0.623	2.118	0.6
3.692	0.303	3.283	137.874	0.609	2.11	0.585
3.468	0.316	3.364	121.098	0.651	1.806	0.624
4.139	0.237	2.904	127.776	0.532	2.555	0.52
3.967	0.276	3.386	162.511	0.606	2.199	0.578
3.5	0.292	3.389	118.612	0.661	1.74	0.608
3.618	0.302	3.412	133.057	0.646	1.874	0.618
4.275	0.259	3.286	187.313	0.563	2.547	0.546
3.462	0.264	2.062	53.608	0.439	2.639	0.439
4.382	0.25	3.599	219.555	0.607	2.331	0.581
5.314	0.192	2.408	233.611	0.365	4.192	0.359
4.758	0.22	3.009	213.612	0.489	3.141	0.476
5.314	0.21	3.834	425.527	0.564	2.961	0.544
4.314	0.232	3.381	175.82	0.593	2.319	0.566
3.916	0.243	3.373	131.562	0.638	1.912	0.598
3.676	0.277	3.741	142.16	0.713	1.507	0.66
3.216	0.346	3.977	131.246	0.788	1.067	0.752
3.928	0.283	3.616	177.189	0.644	1.999	0.607
4.039	0.246	3.313	145.751	0.607	2.147	0.573
3.431	0.306	3.64	123.754	0.715	1.448	0.673

IC2	TIC2	SIC2	CIC2	BIC2	IC3	TIC3
4.165	137.445	0.826	0.879	0.797	4.514	148.955
4.777	248.384	0.838	0.924	0.819	5.027	261.403
4.412	180.905	0.824	0.945	0.767	4.9	200.905
3.484	69.684	0.806	0.838	0.76	3.684	73.684
4.31	116.382	0.907	0.444	0.858	4.459	120.382
3.57	96.382	0.751	1.185	0.743	3.718	100.382
4.473	277.312	0.751	1.481	0.73	5.035	312.141
3.664	51.303	0.962	0.143	0.863	3.807	53.303
4	140.015	0.78	1.129	0.718	4.629	162.015
4.491	184.15	0.838	0.866	0.818	4.687	192.15
4.661	386.889	0.731	1.714	0.725	5.688	472.109
4.514	270.865	0.764	1.392	0.734	5.036	302.149
3.709	163.195	0.679	1.75	0.648	4.198	184.705
4.893	259.315	0.854	0.835	0.825	5.195	275.315
4.607	202.705	0.844	0.852	0.808	4.834	212.705
4.452	191.455	0.821	0.974	0.785	4.722	203.065
3.628	94.337	0.772	1.072	0.726	3.921	101.947
4.812	279.092	0.821	1.046	0.788	5.082	294.743
4.443	182.15	0.829	0.915	0.809	4.54	186.15
4.161	183.096	0.762	1.298	0.737	4.516	198.705
4.35	182.703	0.807	1.042	0.771	4.606	193.458
4.364	113.457	0.928	0.337	0.835	4.441	115.457
4.184	138.059	0.829	0.861	0.766	4.658	153.71
4.336	177.785	0.809	1.021	0.772	4.619	189.395
4.164	254.026	0.702	1.766	0.682	4.701	286.755
4.788	272.926	0.821	1.045	0.798	5.407	308.21
4.515	230.274	0.796	1.157	0.781	4.78	243.784
4.721	372.979	0.749	1.583	0.715	5.126	404.979
3.699	166.465	0.674	1.793	0.64	4.517	203.259
4.227	228.235	0.734	1.528	0.725	4.699	253.744
4.726	321.379	0.776	1.361	0.737	5.146	349.908
5.234	439.67	0.819	1.158	0.79	5.633	473.18
3.969	166.703	0.736	1.423	0.711	4.391	184.438
4.059	198.882	0.723	1.556	0.705	4.548	222.856
4.742	355.613	0.761	1.487	0.742	5.338	400.367
3.916	231.037	0.666	1.967	0.646	4.662	275.076
4.115	218.07	0.718	1.613	0.697	5.02	266.07
3.313	79.51	0.723	1.272	0.679	3.741	89.774
4.456	329.725	0.718	1.754	0.707	5.493	406.48
4.812	202.113	0.892	0.58	0.83	5.089	213.722
4.498	238.407	0.785	1.23	0.73	5.103	270.46
3.374	101.207	0.688	1.533	0.634	3.907	117.207
4.182	380.547	0.643	2.326	0.629	5.122	466.096
4.171	129.315	0.842	0.783	0.776	4.801	148.825
4.478	250.794	0.771	1.329	0.732	5.194	290.847
4.607	175.057	0.878	0.641	0.829	4.86	184.666
4.803	201.722	0.891	0.589	0.847	4.993	209.722
4.939	242.001	0.88	0.676	0.831	5.217	255.611
5.037	216.574	0.928	0.39	0.871	5.176	222.574

5.173	289.702	0.891	0.634	0.854	5.173	289.702
5.27	237.133	0.96	0.222	0.905	5.314	239.133
5.395	388.46	0.874	0.775	0.834	5.76	414.725
5.225	245.556	0.941	0.33	0.894	5.225	245.556
5.12	230.379	0.932	0.372	0.888	5.208	234.379
4.985	229.329	0.903	0.538	0.858	5.159	237.329
5.05	282.806	0.87	0.757	0.819	5.33	298.457
5.302	312.811	0.901	0.581	0.855	5.349	315.566
5.018	190.666	0.956	0.23	0.901	5.018	190.666
5.357	289.254	0.931	0.398	0.881	5.468	295.254
5.208	234.379	0.948	0.283	0.899	5.253	236.379
4.071	162.838	0.765	1.251	0.712	4.584	183.367
4.647	190.54	0.867	0.71	0.823	4.882	200.15
4.923	221.524	0.896	0.569	0.842	5.181	233.133
5.041	211.722	0.935	0.351	0.886	5.136	215.722
4.833	207.82	0.891	0.593	0.852	5.083	218.574
3.742	205.797	0.647	2.04	0.632	4.486	246.756
3.96	205.919	0.695	1.74	0.668	4.571	237.668
4.176	96.042	0.923	0.348	0.835	4.263	98.042
4.059	154.26	0.774	1.188	0.739	4.471	169.911
3.943	157.716	0.741	1.379	0.706	4.584	183.367
4.148	124.452	0.845	0.758	0.802	4.282	128.452
4.025	156.969	0.762	1.261	0.721	4.58	178.621
4.206	138.814	0.834	0.838	0.77	4.802	158.465
4.282	128.452	0.873	0.625	0.816	4.415	132.452
4.574	150.955	0.907	0.47	0.869	4.574	150.955
4.525	194.574	0.834	0.901	0.786	4.99	214.574
4.104	197.005	0.735	1.481	0.699	4.814	231.059
4.349	126.127	0.895	0.509	0.835	4.487	130.127
4.44	186.477	0.823	0.952	0.783	4.678	196.477
4.873	292.374	0.825	1.034	0.818	5.311	318.639
3.463	86.587	0.746	1.18	0.713	3.623	90.587
4.5	184.498	0.84	0.858	0.786	4.882	200.15
3.882	89.287	0.858	0.642	0.808	4.002	92.042
4.097	172.071	0.76	1.295	0.715	4.755	199.722
3.702	107.372	0.762	1.156	0.755	3.892	112.881
2.927	84.881	0.603	1.931	0.558	3.203	92.881
3.851	161.722	0.714	1.542	0.682	4.327	181.722
4.788	272.926	0.821	1.045	0.798	5.407	308.21
3.908	128.955	0.775	1.137	0.72	4.514	148.955
3.827	114.8	0.78	1.08	0.729	4.415	132.452
4.809	226.046	0.866	0.745	0.819	4.911	230.801
4.513	130.881	0.929	0.345	0.876	4.651	134.881
3.873	100.702	0.824	0.827	0.761	4.624	120.211
4.126	251.697	0.696	1.805	0.68	5.062	308.755
4.467	527.132	0.649	2.415	0.639	5.585	659.054
4.599	252.955	0.796	1.182	0.764	5.036	276.955
3.316	132.638	0.623	2.006	0.597	3.696	147.858
4.048	218.583	0.703	1.707	0.68	4.375	236.235
5.177	574.618	0.762	1.618	0.74	5.814	645.347

4.267	238.947	0.735	1.54	0.703	4.624	258.947
4.187	226.093	0.728	1.568	0.693	4.898	264.499
5.048	282.683	0.869	0.759	0.832	5.374	300.947
4.25	157.24	0.816	0.96	0.769	4.358	161.24
3.682	139.911	0.702	1.566	0.707	3.682	139.911
4.445	186.703	0.824	0.947	0.788	4.928	206.968
5.179	383.235	0.834	1.031	0.804	5.557	411.235
4.343	152.015	0.847	0.786	0.808	4.501	157.525
3.263	107.671	0.647	1.782	0.605	4.006	132.2
3.499	111.961	0.7	1.501	0.657	4.242	135.735
3.973	210.586	0.694	1.755	0.667	4.591	243.315
3.756	127.69	0.738	1.332	0.682	4.359	148.219
4.106	258.681	0.687	1.871	0.672	4.957	312.265
4.34	208.304	0.777	1.245	0.758	4.762	228.568
4.183	142.219	0.822	0.905	0.766	4.382	148.974
3.347	90.382	0.704	1.407	0.643	3.94	106.382
4.663	167.853	0.902	0.507	0.847	4.795	172.608
3.418	41.02	0.954	0.167	0.875	3.585	43.02
4.027	225.503	0.693	1.781	0.669	4.865	272.457
3.72	115.315	0.751	1.234	0.704	4.365	135.315
2.922	29.219	0.88	0.4	0.845	2.922	29.219
4.333	381.295	0.671	2.127	0.66	5.34	469.901
4.563	146	0.913	0.438	0.849	4.813	154
4.714	400.72	0.736	1.695	0.723	5.45	463.249
3.273	94.907	0.674	1.585	0.667	3.702	107.372
4.785	200.968	0.887	0.607	0.852	4.975	208.968
4.374	135.58	0.883	0.581	0.843	4.374	135.58
4.028	161.103	0.757	1.294	0.738	4.296	171.858
4.774	334.156	0.779	1.356	0.774	5.429	380.03
4.937	335.683	0.811	1.151	0.783	5.319	361.683
4.679	285.394	0.789	1.252	0.756	5.172	315.51
4.076	191.582	0.734	1.478	0.702	4.757	223.556
4.508	202.869	0.821	0.984	0.783	4.953	222.869
4.084	102.096	0.879	0.56	0.81	4.324	108.096
4.703	188.122	0.884	0.619	0.842	4.953	198.122
3.574	117.936	0.708	1.471	0.686	3.945	130.2
4.69	318.954	0.771	1.397	0.732	5.278	358.928
4.5	184.498	0.84	0.858	0.786	4.882	200.15
4.072	146.608	0.788	1.097	0.737	4.628	166.608
4.377	262.639	0.741	1.53	0.704	5.061	303.659
3.085	74.039	0.673	1.5	0.635	3.252	78.039
3.369	74.107	0.755	1.091	0.725	3.823	84.107
4.513	225.663	0.8	1.131	0.767	4.688	234.418
3.864	131.364	0.759	1.224	0.708	4.452	151.364
3.589	118.426	0.711	1.456	0.666	4.309	142.2
4.47	116.211	0.951	0.231	0.911	4.47	116.211
4.59	179.011	0.868	0.695	0.813	4.888	190.621
3.59	118.465	0.712	1.455	0.645	3.832	126.465
3.825	99.457	0.814	0.875	0.796	3.902	101.457
4.665	214.574	0.845	0.859	0.807	4.839	222.574

3.855	150.356	0.729	1.43	0.71	3.926	153.111
4.38	192.705	0.802	1.08	0.772	4.76	209.46
4.516	198.705	0.827	0.943	0.792	5.061	222.705
4.127	127.929	0.833	0.827	0.77	4.825	149.58
4.695	248.825	0.82	1.033	0.774	5.11	270.825
4.572	164.608	0.884	0.597	0.821	4.795	172.608
4.182	154.75	0.803	1.027	0.781	4.291	158.75
4.007	156.256	0.758	1.279	0.734	4.202	163.866
4.144	161.601	0.784	1.142	0.742	4.734	184.621
4.109	164.348	0.772	1.213	0.748	4.415	176.612
3.807	53.303	1	0	0.896	3.807	53.303
2.946	58.929	0.682	1.375	0.671	2.946	58.929
3.729	186.444	0.661	1.915	0.637	4.153	207.663
4.485	206.309	0.812	1.039	0.779	4.588	211.064
5.113	281.22	0.884	0.668	0.838	5.331	293.22
4.674	266.436	0.801	1.159	0.779	5.407	308.21
4.148	174.213	0.769	1.244	0.751	4.481	188.213
4.408	193.95	0.807	1.051	0.794	4.59	201.95
3.88	81.484	0.883	0.512	0.83	3.88	81.484
4.381	157.711	0.847	0.789	0.782	4.927	177.362
4.882	375.908	0.779	1.385	0.744	5.63	433.523
4.953	198.122	0.931	0.369	0.887	5.053	202.122
4.817	255.315	0.841	0.911	0.806	5.261	278.825
4.191	188.604	0.763	1.301	0.746	4.369	196.604
4.221	206.817	0.752	1.394	0.737	4.471	219.082
4.559	232.5	0.804	1.114	0.764	5.025	256.274
4.392	127.372	0.904	0.466	0.85	4.461	129.372
4.724	236.193	0.837	0.92	0.803	5.164	258.193
4.851	213.46	0.889	0.608	0.847	5.141	226.215
4.018	124.561	0.811	0.936	0.763	4.583	142.07
3.449	120.731	0.673	1.68	0.653	3.678	128.731
5.162	294.21	0.885	0.671	0.842	5.442	310.21
4.1	176.31	0.756	1.326	0.716	4.286	184.31
3.787	124.955	0.751	1.258	0.694	4.393	144.955
4.501	207.064	0.815	1.022	0.775	4.719	217.064
4.263	98.042	0.942	0.261	0.856	4.35	100.042
3.882	89.287	0.858	0.642	0.826	4.176	96.042
4.285	175.685	0.8	1.073	0.763	4.705	192.905
3.79	155.395	0.707	1.567	0.672	4.315	176.905
5.332	426.596	0.843	0.989	0.814	5.665	453.225
4.665	214.574	0.845	0.859	0.807	4.839	222.574
4.408	189.555	0.812	1.018	0.766	4.612	198.31
4.702	178.666	0.896	0.546	0.835	4.912	186.666
4.648	218.436	0.837	0.907	0.792	4.852	228.046
3.962	122.825	0.8	0.992	0.74	4.607	142.825
4.789	210.705	0.877	0.671	0.84	5.016	220.705
3.833	168.666	0.702	1.626	0.666	4.516	198.705
4.208	424.984	0.632	2.45	0.623	5.054	510.44
3.498	118.935	0.688	1.589	0.671	4.08	138.709
3.796	98.702	0.808	0.904	0.774	3.95	102.702

2.999	128.965	0.553	2.427	0.537	3.874	166.574
3.716	107.762	0.765	1.142	0.719	3.978	115.372
3.45	148.336	0.636	1.977	0.602	4.025	173.065
3.06	94.851	0.618	1.894	0.571	3.551	110.07
4.419	167.911	0.842	0.829	0.809	4.629	175.911
4.424	287.538	0.735	1.599	0.708	5.084	330.434
4.315	202.801	0.777	1.24	0.737	4.73	222.311
4.254	182.923	0.784	1.172	0.739	4.618	198.574
4.196	130.07	0.847	0.758	0.805	4.349	134.825
4.424	181.395	0.826	0.933	0.806	4.522	185.395
4.741	312.917	0.784	1.303	0.771	5.525	364.665
4.319	211.611	0.769	1.296	0.728	4.604	225.611
4.758	247.403	0.835	0.943	0.805	5.287	274.913
4.362	239.936	0.755	1.419	0.739	5.158	283.71
3.735	141.911	0.712	1.513	0.693	4.156	157.911
3.831	141.73	0.735	1.379	0.715	4.088	151.24
4.414	229.529	0.774	1.286	0.747	5.04	262.058
4.055	162.206	0.762	1.267	0.732	4.446	177.858
2.897	89.806	0.585	2.057	0.569	2.986	92.561
4.055	162.206	0.762	1.267	0.732	4.446	177.858
4.015	120.452	0.818	0.892	0.771	4.282	128.452
4.033	209.694	0.707	1.668	0.677	4.931	256.423
4.944	237.323	0.885	0.641	0.846	5.278	253.323
4.944	237.323	0.885	0.641	0.846	5.278	253.323
4.344	186.8	0.801	1.082	0.776	4.629	199.065
4.744	327.307	0.777	1.365	0.752	5.322	367.224
3.746	74.929	0.867	0.575	0.797	3.984	79.684
3.879	108.606	0.807	0.929	0.769	4.022	112.606
4.613	262.965	0.791	1.219	0.755	5.188	295.72
4.561	177.866	0.863	0.725	0.814	4.683	182.621
4.837	440.131	0.743	1.671	0.731	5.477	498.435
4.11	189.064	0.744	1.413	0.725	4.561	209.819
3.25	52	0.813	0.75	0.752	3.875	62
4.359	187.455	0.803	1.067	0.772	4.6	197.82
4.838	304.794	0.809	1.139	0.776	5.231	329.549
4.346	165.157	0.828	0.902	0.801	4.557	173.157
4.545	259.049	0.779	1.288	0.753	4.986	284.21
3.937	110.241	0.819	0.87	0.759	4.209	117.851
3.726	134.143	0.721	1.444	0.665	4.427	159.362
3.388	165.988	0.603	2.227	0.565	4.078	199.837
3.938	122.07	0.795	1.016	0.742	4.26	132.07
4.49	188.561	0.833	0.903	0.78	4.862	204.213
4.615	212.309	0.836	0.908	0.806	5.012	230.574
4.302	176.375	0.803	1.056	0.766	4.619	189.395
4.098	122.942	0.835	0.809	0.778	4.482	134.452
4.617	258.541	0.795	1.191	0.767	5.111	286.192
4.758	214.114	0.866	0.734	0.835	5.12	230.379
4.043	137.464	0.795	1.044	0.741	4.631	157.464
4.597	151.71	0.911	0.447	0.864	4.597	151.71
4.597	151.71	0.911	0.447	0.84	4.84	159.71

4.454	236.07	0.778	1.274	0.747	4.681	248.07
3.757	131.486	0.732	1.373	0.716	3.835	134.241
4.119	201.837	0.734	1.496	0.716	5.012	245.611
5.048	282.683	0.869	0.759	0.832	5.338	298.947
3.559	74.729	0.81	0.834	0.753	3.654	76.729
3.498	118.935	0.688	1.589	0.671	4.08	138.709
4.064	125.97	0.82	0.891	0.772	4.503	139.58
3.974	178.83	0.724	1.518	0.71	4.219	189.849
4.441	217.611	0.791	1.174	0.761	5.053	247.611
4.041	169.722	0.749	1.351	0.72	4.327	181.722
4.239	152.608	0.82	0.931	0.786	4.461	160.608
4.135	181.95	0.757	1.324	0.74	4.635	203.95
4.298	206.304	0.77	1.287	0.734	5.043	242.078
4.146	145.119	0.808	0.983	0.744	4.822	168.77
3.9	152.092	0.738	1.386	0.706	4.073	158.846
4.407	251.191	0.756	1.426	0.732	4.986	284.21
2.95	76.702	0.628	1.75	0.628	3.258	84.702
4.643	283.246	0.783	1.287	0.749	5.14	313.51
4.407	427.451	0.668	2.193	0.658	5.37	520.907
4.336	307.838	0.705	1.814	0.686	4.695	333.348
5.62	623.837	0.827	1.174	0.798	6.168	684.631
4.681	243.403	0.821	1.02	0.783	5.21	270.913
4.032	157.237	0.763	1.254	0.714	4.661	181.766
4.587	174.302	0.874	0.661	0.809	4.945	187.911
4.779	157.71	0.947	0.265	0.904	4.779	157.71
4.416	216.392	0.787	1.199	0.742	5.043	247.121
4.112	180.931	0.753	1.347	0.711	4.419	194.441
4.29	145.854	0.843	0.798	0.793	4.514	153.464

SIC3	CIC3	BIC3	IC4	TIC4	SIC4	CIC4
0.895	0.531	0.863	4.514	148.955	0.895	0.531
0.882	0.673	0.862	5.027	261.403	0.882	0.673
0.915	0.457	0.851	4.949	202.905	0.924	0.409
0.852	0.638	0.804	3.684	73.684	0.852	0.638
0.938	0.296	0.888	4.459	120.382	0.938	0.296
0.782	1.037	0.773	3.718	100.382	0.782	1.037
0.846	0.92	0.821	5.111	316.896	0.858	0.843
1	0	0.896	3.807	53.303	1	0
0.902	0.5	0.831	4.708	164.77	0.918	0.422
0.875	0.671	0.853	4.735	194.15	0.884	0.622
0.892	0.687	0.885	6.101	506.373	0.957	0.274
0.853	0.871	0.819	5.069	304.149	0.858	0.838
0.769	1.262	0.733	4.289	188.705	0.786	1.171
0.907	0.533	0.876	5.27	279.315	0.92	0.458
0.885	0.625	0.848	4.834	212.705	0.885	0.625
0.87	0.704	0.833	4.787	205.82	0.882	0.64
0.834	0.779	0.784	4.027	104.702	0.857	0.673
0.867	0.776	0.832	5.129	297.498	0.876	0.729
0.847	0.817	0.827	4.54	186.15	0.847	0.817
0.827	0.943	0.8	4.579	201.46	0.839	0.881
0.854	0.786	0.816	4.654	195.458	0.863	0.739
0.945	0.26	0.849	4.441	115.457	0.945	0.26
0.923	0.387	0.853	4.658	153.71	0.923	0.387
0.862	0.738	0.823	4.687	192.15	0.875	0.671
0.793	1.23	0.77	4.89	298.265	0.824	1.041
0.927	0.426	0.901	5.407	308.21	0.927	0.426
0.843	0.892	0.827	4.78	243.784	0.843	0.892
0.813	1.177	0.777	5.304	418.979	0.841	1
0.822	0.975	0.781	4.73	212.869	0.861	0.761
0.817	1.056	0.806	4.773	257.744	0.829	0.982
0.845	0.942	0.803	5.205	353.908	0.855	0.883
0.881	0.759	0.85	5.776	485.18	0.904	0.616
0.814	1.001	0.786	4.391	184.438	0.814	1.001
0.81	1.067	0.79	4.671	228.856	0.832	0.944
0.857	0.891	0.835	5.338	400.367	0.857	0.891
0.793	1.22	0.769	4.73	279.076	0.804	1.153
0.876	0.708	0.85	5.2	275.58	0.908	0.528
0.816	0.844	0.766	3.991	95.774	0.87	0.594
0.885	0.716	0.871	5.547	410.48	0.893	0.662
0.944	0.304	0.878	5.136	215.722	0.953	0.256
0.891	0.625	0.828	5.374	284.825	0.938	0.354
0.796	1	0.734	4.04	121.207	0.823	0.867
0.787	1.386	0.771	5.768	524.925	0.886	0.739
0.969	0.153	0.893	4.801	148.825	0.969	0.153
0.894	0.614	0.849	5.437	304.457	0.936	0.371
0.926	0.388	0.875	4.932	187.421	0.94	0.316
0.926	0.399	0.88	4.993	209.722	0.926	0.399
0.929	0.398	0.878	5.273	258.366	0.939	0.342
0.954	0.25	0.895	5.176	222.574	0.954	0.25

0.891	0.634	0.854	5.173	289.702	0.891	0.634
0.968	0.178	0.913	5.314	239.133	0.968	0.178
0.934	0.41	0.891	5.76	414.725	0.934	0.41
0.941	0.33	0.894	5.225	245.556	0.941	0.33
0.948	0.283	0.903	5.208	234.379	0.948	0.283
0.934	0.364	0.888	5.159	237.329	0.934	0.364
0.918	0.478	0.864	5.379	301.212	0.926	0.429
0.909	0.534	0.863	5.349	315.566	0.909	0.534
0.956	0.23	0.901	5.018	190.666	0.956	0.23
0.95	0.287	0.9	5.468	295.254	0.95	0.287
0.956	0.239	0.907	5.253	236.379	0.956	0.239
0.861	0.738	0.802	4.584	183.367	0.861	0.738
0.911	0.476	0.865	4.949	202.905	0.924	0.409
0.943	0.311	0.886	5.181	233.133	0.943	0.311
0.953	0.256	0.903	5.136	215.722	0.953	0.256
0.937	0.343	0.896	5.13	220.574	0.945	0.297
0.776	1.295	0.758	4.763	261.975	0.824	1.018
0.802	1.13	0.771	4.893	254.423	0.858	0.808
0.942	0.261	0.853	4.263	98.042	0.942	0.261
0.852	0.777	0.814	4.471	169.911	0.852	0.777
0.861	0.738	0.821	4.734	189.367	0.89	0.588
0.873	0.625	0.828	4.282	128.452	0.873	0.625
0.867	0.705	0.82	4.734	184.621	0.896	0.552
0.952	0.242	0.88	4.923	162.465	0.976	0.121
0.9	0.492	0.841	4.415	132.452	0.9	0.492
0.907	0.47	0.869	4.574	150.955	0.907	0.47
0.92	0.436	0.867	4.99	214.574	0.92	0.436
0.862	0.771	0.82	5.179	248.568	0.927	0.406
0.924	0.371	0.861	4.556	132.127	0.938	0.302
0.868	0.714	0.825	4.678	196.477	0.868	0.714
0.899	0.596	0.892	5.423	325.394	0.918	0.484
0.78	1.02	0.746	3.623	90.587	0.78	1.02
0.911	0.476	0.852	4.882	200.15	0.911	0.476
0.885	0.522	0.832	4.002	92.042	0.885	0.522
0.882	0.637	0.83	4.803	201.722	0.891	0.589
0.801	0.966	0.793	3.892	112.881	0.801	0.966
0.659	1.655	0.61	3.203	92.881	0.659	1.655
0.802	1.066	0.767	4.422	185.722	0.82	0.97
0.927	0.426	0.901	5.407	308.21	0.927	0.426
0.895	0.531	0.832	4.597	151.71	0.911	0.447
0.9	0.492	0.841	4.574	137.207	0.932	0.333
0.884	0.644	0.837	4.911	230.801	0.884	0.644
0.957	0.207	0.903	4.651	134.881	0.957	0.207
0.984	0.077	0.909	4.7	122.211	1	0
0.853	0.869	0.834	5.447	332.265	0.918	0.484
0.811	1.297	0.799	5.975	705.093	0.868	0.907
0.871	0.746	0.836	5.072	278.955	0.877	0.709
0.695	1.625	0.665	3.834	153.367	0.72	1.488
0.76	1.38	0.735	4.477	241.744	0.778	1.278
0.856	0.98	0.831	5.992	665.122	0.882	0.802

0.796	1.183	0.762	4.731	264.947	0.815	1.076
0.851	0.857	0.81	5.199	280.764	0.903	0.556
0.925	0.433	0.886	5.374	300.947	0.925	0.433
0.837	0.852	0.789	4.412	163.24	0.847	0.798
0.702	1.566	0.707	3.682	139.911	0.702	1.566
0.914	0.465	0.873	4.928	206.968	0.914	0.465
0.895	0.652	0.863	5.692	421.235	0.917	0.517
0.877	0.629	0.837	4.501	157.525	0.877	0.629
0.794	1.038	0.743	4.438	146.465	0.88	0.606
0.848	0.758	0.797	4.515	144.49	0.903	0.485
0.801	1.137	0.771	4.808	254.825	0.839	0.92
0.857	0.728	0.791	4.558	154.974	0.896	0.529
0.829	1.021	0.811	5.108	321.775	0.854	0.87
0.853	0.823	0.831	4.845	232.568	0.868	0.74
0.861	0.706	0.803	4.382	148.974	0.861	0.706
0.829	0.815	0.756	3.94	106.382	0.829	0.815
0.927	0.375	0.871	4.795	172.608	0.927	0.375
1	0	0.918	3.585	43.02	1	0
0.838	0.942	0.808	5.044	282.457	0.869	0.763
0.881	0.589	0.826	4.543	140.825	0.917	0.411
0.88	0.4	0.845	2.5	25	0.753	0.822
0.827	1.12	0.813	5.692	500.92	0.881	0.767
0.963	0.188	0.895	4.813	154	0.963	0.188
0.85	0.959	0.835	5.712	485.514	0.891	0.697
0.762	1.156	0.755	3.892	112.881	0.801	0.966
0.923	0.417	0.886	4.975	208.968	0.923	0.417
0.883	0.581	0.843	4.374	135.58	0.883	0.581
0.807	1.025	0.787	4.496	179.858	0.845	0.825
0.886	0.7	0.88	5.565	389.54	0.908	0.564
0.874	0.769	0.844	5.319	361.683	0.874	0.769
0.872	0.758	0.836	5.263	321.02	0.887	0.668
0.856	0.798	0.819	4.799	225.556	0.864	0.756
0.902	0.539	0.861	5.014	225.624	0.913	0.478
0.931	0.32	0.857	4.324	108.096	0.931	0.32
0.931	0.369	0.887	4.953	198.122	0.931	0.369
0.782	1.099	0.757	4.067	134.2	0.806	0.978
0.867	0.809	0.824	5.506	374.438	0.905	0.581
0.911	0.476	0.852	4.882	200.15	0.911	0.476
0.895	0.542	0.838	4.76	171.362	0.921	0.41
0.857	0.846	0.814	5.194	311.659	0.879	0.713
0.709	1.333	0.669	3.252	78.039	0.709	1.333
0.857	0.636	0.823	4.096	90.107	0.918	0.364
0.831	0.955	0.797	4.808	240.418	0.852	0.835
0.875	0.636	0.815	4.735	160.974	0.931	0.353
0.854	0.735	0.799	4.574	150.955	0.907	0.47
0.951	0.231	0.911	4.47	116.211	0.951	0.231
0.925	0.398	0.866	4.939	192.621	0.934	0.346
0.76	1.212	0.688	3.832	126.465	0.76	1.212
0.83	0.798	0.812	3.979	103.457	0.847	0.721
0.876	0.685	0.837	4.839	222.574	0.876	0.685

0.743	1.359	0.724	3.926	153.111	0.743	1.359
0.872	0.699	0.839	4.806	211.46	0.88	0.654
0.927	0.398	0.888	5.061	222.705	0.927	0.398
0.974	0.129	0.901	4.954	153.58	1	0
0.892	0.618	0.842	5.261	278.825	0.918	0.467
0.927	0.375	0.861	4.85	174.608	0.938	0.32
0.824	0.919	0.801	4.291	158.75	0.824	0.919
0.795	1.084	0.77	4.272	166.621	0.808	1.013
0.896	0.552	0.848	4.907	191.376	0.928	0.378
0.83	0.907	0.804	4.415	176.612	0.83	0.907
1	0	0.896	3.807	53.303	1	0
0.682	1.375	0.671	2.946	58.929	0.682	1.375
0.736	1.491	0.709	4.263	213.173	0.755	1.38
0.831	0.935	0.797	4.588	211.064	0.831	0.935
0.922	0.45	0.874	5.368	295.22	0.928	0.414
0.927	0.426	0.901	5.407	308.21	0.927	0.426
0.831	0.911	0.811	4.481	188.213	0.831	0.911
0.841	0.87	0.826	4.59	201.95	0.841	0.87
0.883	0.512	0.83	3.88	81.484	0.883	0.512
0.953	0.243	0.88	4.982	179.362	0.964	0.188
0.898	0.637	0.858	5.78	445.033	0.922	0.487
0.949	0.269	0.905	5.053	202.122	0.949	0.269
0.918	0.467	0.88	5.261	278.825	0.918	0.467
0.796	1.123	0.778	4.413	198.604	0.804	1.078
0.796	1.144	0.781	4.65	227.837	0.828	0.965
0.886	0.647	0.842	5.157	263.029	0.909	0.515
0.918	0.397	0.863	4.461	129.372	0.918	0.397
0.915	0.48	0.878	5.164	258.193	0.915	0.48
0.942	0.318	0.898	5.141	226.215	0.942	0.318
0.925	0.371	0.87	4.647	144.07	0.938	0.307
0.717	1.451	0.696	3.678	128.731	0.717	1.451
0.933	0.391	0.888	5.442	310.21	0.933	0.391
0.79	1.14	0.748	4.333	186.31	0.798	1.093
0.871	0.652	0.805	4.537	149.71	0.899	0.508
0.854	0.805	0.813	4.806	221.064	0.87	0.718
0.962	0.174	0.874	4.35	100.042	0.962	0.174
0.923	0.348	0.888	4.263	98.042	0.942	0.261
0.878	0.653	0.838	4.705	192.905	0.878	0.653
0.805	1.043	0.765	4.412	180.905	0.824	0.945
0.896	0.657	0.864	5.715	457.225	0.904	0.607
0.876	0.685	0.837	4.839	222.574	0.876	0.685
0.85	0.814	0.801	4.798	206.31	0.884	0.628
0.936	0.336	0.873	4.965	188.666	0.946	0.283
0.874	0.703	0.827	4.911	230.801	0.884	0.644
0.93	0.347	0.86	4.696	145.58	0.948	0.258
0.919	0.443	0.88	5.016	220.705	0.919	0.443
0.827	0.943	0.785	4.715	207.46	0.864	0.744
0.759	1.604	0.748	5.287	533.95	0.794	1.372
0.802	1.008	0.783	4.256	144.709	0.837	0.831
0.84	0.75	0.805	3.95	102.702	0.84	0.75

0.714	1.552	0.694	4.217	181.329	0.777	1.209
0.819	0.88	0.77	4.073	118.127	0.838	0.785
0.742	1.402	0.703	4.153	178.574	0.765	1.273
0.717	1.404	0.663	3.728	115.58	0.753	1.226
0.882	0.619	0.848	4.682	177.911	0.892	0.566
0.844	0.939	0.814	5.261	341.944	0.874	0.762
0.852	0.825	0.807	4.985	234.311	0.898	0.569
0.851	0.808	0.802	4.682	201.329	0.863	0.744
0.878	0.605	0.835	4.349	134.825	0.878	0.605
0.844	0.836	0.823	4.522	185.395	0.844	0.836
0.914	0.519	0.898	5.646	372.665	0.934	0.398
0.82	1.01	0.776	4.808	235.611	0.856	0.806
0.927	0.414	0.895	5.287	274.913	0.927	0.414
0.892	0.623	0.873	5.231	287.71	0.905	0.55
0.792	1.092	0.771	4.156	157.911	0.792	1.092
0.785	1.122	0.763	4.088	151.24	0.785	1.122
0.884	0.661	0.853	5.224	271.668	0.916	0.476
0.835	0.875	0.803	4.684	187.367	0.88	0.638
0.603	1.968	0.587	2.986	92.561	0.603	1.968
0.835	0.875	0.803	4.684	187.367	0.88	0.638
0.873	0.625	0.822	4.282	128.452	0.873	0.625
0.865	0.769	0.828	5.124	266.423	0.899	0.577
0.945	0.307	0.903	5.278	253.323	0.945	0.307
0.945	0.307	0.903	5.278	253.323	0.945	0.307
0.853	0.797	0.827	4.676	201.065	0.862	0.75
0.871	0.786	0.844	5.38	371.224	0.881	0.728
0.922	0.338	0.848	3.984	79.684	0.922	0.338
0.837	0.786	0.797	4.022	112.606	0.837	0.786
0.889	0.645	0.849	5.188	295.72	0.889	0.645
0.886	0.603	0.836	4.683	182.621	0.886	0.603
0.842	1.03	0.828	5.67	515.945	0.871	0.838
0.826	0.962	0.804	4.648	213.819	0.842	0.875
0.969	0.125	0.897	4	64	1	0
0.848	0.826	0.815	4.665	200.574	0.86	0.762
0.875	0.746	0.839	5.231	329.549	0.875	0.746
0.868	0.691	0.84	4.557	173.157	0.868	0.691
0.855	0.847	0.826	5.034	286.965	0.863	0.798
0.876	0.598	0.811	4.209	117.851	0.876	0.598
0.856	0.743	0.791	4.614	166.117	0.893	0.556
0.726	1.536	0.68	4.416	216.366	0.786	1.199
0.86	0.694	0.803	4.26	132.07	0.86	0.694
0.902	0.53	0.845	4.928	206.968	0.914	0.465
0.907	0.511	0.875	5.012	230.574	0.907	0.511
0.862	0.738	0.823	4.619	189.395	0.862	0.738
0.913	0.425	0.851	4.482	134.452	0.913	0.425
0.88	0.697	0.849	5.195	290.947	0.895	0.612
0.932	0.372	0.898	5.12	230.379	0.932	0.372
0.91	0.456	0.848	4.712	160.219	0.926	0.375
0.911	0.447	0.864	4.597	151.71	0.911	0.447
0.959	0.205	0.884	4.84	159.71	0.959	0.205

0.817	1.047	0.785	4.869	258.07	0.85	0.859
0.748	1.294	0.731	3.835	134.241	0.748	1.294
0.893	0.602	0.871	5.094	249.611	0.907	0.521
0.919	0.469	0.88	5.338	298.947	0.919	0.469
0.832	0.739	0.773	3.939	82.729	0.897	0.453
0.802	1.008	0.783	4.256	144.709	0.837	0.831
0.909	0.452	0.855	4.696	145.58	0.948	0.258
0.768	1.273	0.753	4.219	189.849	0.768	1.273
0.9	0.561	0.866	5.15	252.366	0.917	0.464
0.802	1.066	0.771	4.327	181.722	0.802	1.066
0.863	0.709	0.827	4.461	160.608	0.863	0.709
0.849	0.824	0.83	4.726	207.95	0.866	0.733
0.903	0.542	0.861	5.043	242.078	0.903	0.542
0.94	0.307	0.866	4.936	172.77	0.962	0.193
0.771	1.212	0.737	4.144	161.601	0.784	1.142
0.855	0.847	0.828	5.126	292.21	0.879	0.706
0.693	1.443	0.693	3.258	84.702	0.693	1.443
0.867	0.791	0.829	5.271	321.51	0.889	0.66
0.814	1.23	0.801	5.613	544.417	0.85	0.987
0.763	1.455	0.743	4.864	345.348	0.791	1.286
0.908	0.627	0.876	6.278	696.896	0.924	0.516
0.914	0.491	0.872	5.248	272.913	0.921	0.452
0.882	0.625	0.826	4.907	191.376	0.928	0.378
0.942	0.303	0.872	4.945	187.911	0.942	0.303
0.947	0.265	0.904	4.779	157.71	0.947	0.265
0.898	0.571	0.847	5.084	249.121	0.905	0.531
0.809	1.04	0.764	4.618	203.195	0.846	0.841
0.887	0.574	0.834	4.514	153.464	0.887	0.574

BIC4	IC5	TIC5	SIC5	CIC5	BIC5	ATS1m
0.863	4.514	148.955	0.895	0.531	0.863	2.995
0.862	5.027	261.403	0.882	0.673	0.862	3.308
0.86	4.949	202.905	0.924	0.409	0.86	3.464
0.804	3.684	73.684	0.852	0.638	0.804	2.565
0.888	4.459	120.382	0.938	0.296	0.888	3.044
0.773	3.624	97.835	0.762	1.131	0.754	3.474
0.834	5.111	316.896	0.858	0.843	0.834	3.613
0.896	3.807	53.303	1	0	0.896	2.617
0.845	4.708	164.77	0.918	0.422	0.845	3.384
0.862	4.735	194.15	0.884	0.622	0.862	3.012
0.949	6.158	511.128	0.966	0.217	0.958	3.905
0.824	5.069	304.149	0.858	0.838	0.824	4.013
0.749	4.289	188.705	0.786	1.171	0.749	3.199
0.889	5.27	279.315	0.92	0.458	0.889	3.558
0.848	4.834	212.705	0.885	0.625	0.848	3.526
0.844	4.787	205.82	0.882	0.64	0.844	3.486
0.805	4.027	104.702	0.857	0.673	0.805	2.854
0.84	5.129	297.498	0.876	0.729	0.84	3.877
0.827	4.54	186.15	0.847	0.817	0.827	3.083
0.811	4.579	201.46	0.839	0.881	0.811	3.251
0.825	4.654	195.458	0.863	0.739	0.825	3.315
0.849	4.441	115.457	0.945	0.26	0.849	3.36
0.853	4.658	153.71	0.923	0.387	0.853	3.318
0.835	4.687	192.15	0.875	0.671	0.835	3.45
0.8	4.922	300.265	0.83	1.008	0.806	3.455
0.901	5.407	308.21	0.927	0.426	0.901	3.533
0.827	4.78	243.784	0.843	0.892	0.827	3.258
0.804	5.405	426.979	0.857	0.899	0.819	3.919
0.818	4.73	212.869	0.861	0.761	0.818	3.278
0.818	4.773	257.744	0.829	0.982	0.818	3.283
0.812	5.234	355.908	0.86	0.854	0.817	4.02
0.871	5.776	485.18	0.904	0.616	0.871	4.083
0.786	4.391	184.438	0.814	1.001	0.786	3.106
0.812	4.752	232.856	0.846	0.863	0.826	3.185
0.835	5.338	400.367	0.857	0.891	0.835	3.773
0.78	4.73	279.076	0.804	1.153	0.78	3.55
0.88	5.2	275.58	0.908	0.528	0.88	3.407
0.817	3.991	95.774	0.87	0.594	0.817	2.918
0.88	5.547	410.48	0.893	0.662	0.88	3.526
0.886	5.136	215.722	0.953	0.256	0.886	3.511
0.872	5.412	286.825	0.945	0.316	0.879	3.728
0.759	4.04	121.207	0.823	0.867	0.759	3.091
0.868	5.812	528.925	0.893	0.695	0.875	3.885
0.893	4.801	148.825	0.969	0.153	0.893	3.217
0.889	5.544	310.457	0.955	0.263	0.906	3.611
0.888	4.932	187.421	0.94	0.316	0.888	3.544
0.88	4.993	209.722	0.926	0.399	0.88	3.526
0.887	5.273	258.366	0.939	0.342	0.887	3.823
0.895	5.176	222.574	0.954	0.25	0.895	3.86

0.854	5.173	289.702	0.891	0.634	0.854	3.851
0.913	5.314	239.133	0.968	0.178	0.913	3.786
0.891	5.76	414.725	0.934	0.41	0.891	4.123
0.894	5.225	245.556	0.941	0.33	0.894	3.793
0.903	5.208	234.379	0.948	0.283	0.903	3.73
0.888	5.159	237.329	0.934	0.364	0.888	3.584
0.872	5.379	301.212	0.926	0.429	0.872	4.023
0.863	5.349	315.566	0.909	0.534	0.863	3.962
0.901	5.018	190.666	0.956	0.23	0.901	3.648
0.9	5.468	295.254	0.95	0.287	0.9	4.018
0.907	5.253	236.379	0.956	0.239	0.907	3.698
0.802	4.584	183.367	0.861	0.738	0.802	3.701
0.877	4.949	202.905	0.924	0.409	0.877	3.486
0.886	5.181	233.133	0.943	0.311	0.886	3.722
0.903	5.136	215.722	0.953	0.256	0.903	3.659
0.904	5.13	220.574	0.945	0.297	0.904	3.486
0.805	4.763	261.975	0.824	1.018	0.805	3.461
0.825	4.893	254.423	0.858	0.808	0.825	3.535
0.853	4.263	98.042	0.942	0.261	0.853	3.67
0.814	4.471	169.911	0.852	0.777	0.814	3.169
0.848	4.734	189.367	0.89	0.588	0.848	3.411
0.828	4.282	128.452	0.873	0.625	0.828	3.391
0.848	4.734	184.621	0.896	0.552	0.848	3.394
0.902	4.923	162.465	0.976	0.121	0.902	3.607
0.841	4.415	132.452	0.9	0.492	0.841	3.19
0.869	4.574	150.955	0.907	0.47	0.869	3.143
0.867	4.99	214.574	0.92	0.436	0.867	3.434
0.882	5.262	252.568	0.942	0.323	0.896	3.437
0.875	4.556	132.127	0.938	0.302	0.875	3.226
0.825	4.678	196.477	0.868	0.714	0.825	3.431
0.911	5.457	327.394	0.924	0.45	0.916	3.614
0.746	3.623	90.587	0.78	1.02	0.746	2.906
0.852	4.882	200.15	0.911	0.476	0.852	3.469
0.832	4.002	92.042	0.885	0.522	0.832	3.04
0.839	4.803	201.722	0.891	0.589	0.839	3.422
0.793	3.892	112.881	0.801	0.966	0.793	3.354
0.61	3.203	92.881	0.659	1.655	0.61	3.325
0.784	4.422	185.722	0.82	0.97	0.784	3.171
0.901	5.407	308.21	0.927	0.426	0.901	3.533
0.847	4.597	151.71	0.911	0.447	0.847	3.263
0.871	4.574	137.207	0.932	0.333	0.871	3.254
0.837	4.911	230.801	0.884	0.644	0.837	3.795
0.903	4.651	134.881	0.957	0.207	0.903	3.128
0.924	4.7	122.211	1	0	0.924	3.098
0.898	5.447	332.265	0.918	0.484	0.898	3.516
0.855	6.19	730.377	0.899	0.693	0.886	4.229
0.842	5.072	278.955	0.877	0.709	0.842	3.646
0.69	3.834	153.367	0.72	1.488	0.69	3.098
0.752	4.477	241.744	0.778	1.278	0.752	3.344
0.856	6.01	667.122	0.885	0.784	0.859	4.258

0.78	4.767	266.947	0.821	1.04	0.786	3.918
0.86	5.273	284.764	0.916	0.481	0.872	3.683
0.886	5.374	300.947	0.925	0.433	0.886	3.672
0.799	4.412	163.24	0.847	0.798	0.799	3.256
0.707	3.682	139.911	0.702	1.566	0.707	2.73
0.873	4.928	206.968	0.914	0.465	0.873	3.245
0.884	5.719	423.235	0.921	0.49	0.888	4.031
0.837	4.501	157.525	0.877	0.629	0.837	3.647
0.823	4.438	146.465	0.88	0.606	0.823	3.091
0.848	4.601	147.245	0.92	0.399	0.865	3.06
0.808	4.846	256.825	0.846	0.882	0.814	3.379
0.828	4.558	154.974	0.896	0.529	0.828	3.443
0.836	5.108	321.775	0.854	0.87	0.836	3.476
0.846	4.97	238.568	0.89	0.615	0.868	3.578
0.803	4.382	148.974	0.861	0.706	0.803	3.363
0.756	3.94	106.382	0.829	0.815	0.756	3.102
0.871	4.795	172.608	0.927	0.375	0.871	3.341
0.918	3.585	43.02	1	0	0.918	2.478
0.838	5.187	290.457	0.893	0.621	0.861	3.712
0.859	4.543	140.825	0.917	0.411	0.859	3.056
0.723	0.918	9.183	0.276	2.404	0.265	2.806
0.866	5.897	518.92	0.913	0.563	0.898	3.998
0.895	4.813	154	0.963	0.188	0.895	3.57
0.876	5.735	487.514	0.895	0.674	0.879	3.769
0.793	3.892	112.881	0.801	0.966	0.793	2.627
0.886	4.975	208.968	0.923	0.417	0.886	3.302
0.843	4.374	135.58	0.883	0.581	0.843	3.15
0.824	4.496	179.858	0.845	0.825	0.824	3.012
0.902	5.622	393.54	0.917	0.507	0.911	3.655
0.844	5.319	361.683	0.874	0.769	0.844	3.876
0.85	5.263	321.02	0.887	0.668	0.85	3.892
0.826	4.799	225.556	0.864	0.756	0.826	3.368
0.871	5.014	225.624	0.913	0.478	0.871	3.584
0.857	4.324	108.096	0.931	0.32	0.857	3.67
0.887	4.953	198.122	0.931	0.369	0.887	3.326
0.781	4.067	134.2	0.806	0.978	0.781	2.813
0.859	5.506	374.438	0.905	0.581	0.859	3.807
0.852	4.882	200.15	0.911	0.476	0.852	3.469
0.862	4.76	171.362	0.921	0.41	0.862	3.239
0.835	5.228	313.659	0.885	0.679	0.841	3.678
0.669	3.252	78.039	0.709	1.333	0.669	3.149
0.882	4.187	92.107	0.939	0.273	0.902	2.926
0.817	4.848	242.418	0.859	0.795	0.824	3.519
0.867	4.735	160.974	0.931	0.353	0.867	3.256
0.848	4.658	153.71	0.923	0.387	0.864	3.091
0.911	4.47	116.211	0.951	0.231	0.911	3.121
0.875	4.939	192.621	0.934	0.346	0.875	3.619
0.688	3.832	126.465	0.76	1.212	0.688	3.278
0.828	4.133	107.457	0.879	0.567	0.86	2.662
0.837	4.839	222.574	0.876	0.685	0.837	3.519

0.724	3.926	153.111	0.743	1.359	0.724	2.953
0.847	4.806	211.46	0.88	0.654	0.847	3.431
0.888	5.061	222.705	0.927	0.398	0.888	3.45
0.925	4.954	153.58	1	0	0.925	3.375
0.867	5.299	280.825	0.925	0.429	0.873	3.651
0.871	4.85	174.608	0.938	0.32	0.871	3.686
0.801	4.291	158.75	0.824	0.919	0.801	3.228
0.783	4.272	166.621	0.808	1.013	0.783	3.068
0.879	4.958	193.376	0.938	0.327	0.888	3.232
0.804	4.415	176.612	0.83	0.907	0.804	3.052
0.896	3.807	53.303	1	0	0.896	2.708
0.671	2.642	52.832	0.611	1.68	0.601	2.335
0.728	4.263	213.173	0.755	1.38	0.728	3.252
0.797	4.588	211.064	0.831	0.935	0.797	3.579
0.88	5.368	295.22	0.928	0.414	0.88	3.693
0.901	5.407	308.21	0.927	0.426	0.901	3.486
0.811	4.481	188.213	0.831	0.911	0.811	3.217
0.826	4.59	201.95	0.841	0.87	0.826	3.091
0.83	3.88	81.484	0.883	0.512	0.83	2.737
0.89	4.982	179.362	0.964	0.188	0.89	3.424
0.881	5.806	447.033	0.926	0.461	0.885	3.963
0.905	5.053	202.122	0.949	0.269	0.905	3.326
0.88	5.261	278.825	0.918	0.467	0.88	3.626
0.786	4.413	198.604	0.804	1.078	0.786	3.142
0.812	4.731	231.837	0.843	0.883	0.826	3.258
0.865	5.197	265.029	0.916	0.476	0.871	3.668
0.863	4.461	129.372	0.918	0.397	0.863	3.036
0.878	5.164	258.193	0.915	0.48	0.878	3.491
0.898	5.141	226.215	0.942	0.318	0.898	3.395
0.882	4.647	144.07	0.938	0.307	0.882	3.012
0.696	3.678	128.731	0.717	1.451	0.696	2.953
0.888	5.442	310.21	0.933	0.391	0.888	3.799
0.756	4.333	186.31	0.798	1.093	0.756	3.421
0.831	4.537	149.71	0.899	0.508	0.831	3.527
0.828	4.849	223.064	0.878	0.674	0.835	3.453
0.874	4.35	100.042	0.962	0.174	0.874	3.127
0.907	4.35	100.042	0.962	0.174	0.925	2.651
0.838	4.705	192.905	0.878	0.653	0.838	3.364
0.782	4.412	180.905	0.824	0.945	0.782	3.15
0.872	5.715	457.225	0.904	0.607	0.872	3.986
0.837	4.839	222.574	0.876	0.685	0.837	3.519
0.834	4.937	212.31	0.91	0.489	0.858	3.562
0.882	4.965	188.666	0.946	0.283	0.882	3.283
0.837	4.911	230.801	0.884	0.644	0.837	3.652
0.877	4.696	145.58	0.948	0.258	0.877	3.269
0.88	5.016	220.705	0.919	0.443	0.88	3.417
0.819	4.76	209.46	0.872	0.699	0.827	3.368
0.783	5.425	547.95	0.815	1.233	0.803	3.919
0.817	4.256	144.709	0.837	0.831	0.817	2.927
0.805	3.95	102.702	0.84	0.75	0.805	2.73

0.755	4.217	181.329	0.777	1.209	0.755	3.068
0.788	4.073	118.127	0.838	0.785	0.788	3.028
0.725	4.153	178.574	0.765	1.273	0.725	3.321
0.696	3.728	115.58	0.753	1.226	0.696	3.15
0.858	4.682	177.911	0.892	0.566	0.858	3.076
0.842	5.261	341.944	0.874	0.762	0.842	3.91
0.851	4.985	234.311	0.898	0.569	0.851	3.491
0.814	4.682	201.329	0.863	0.744	0.814	3.657
0.835	4.349	134.825	0.878	0.605	0.835	3.096
0.823	4.522	185.395	0.844	0.836	0.823	3.091
0.918	5.646	372.665	0.934	0.398	0.918	3.546
0.811	4.849	237.611	0.864	0.766	0.818	3.588
0.895	5.287	274.913	0.927	0.414	0.895	3.455
0.886	5.231	287.71	0.905	0.55	0.886	3.32
0.771	4.156	157.911	0.792	1.092	0.771	2.962
0.763	4.088	151.24	0.785	1.122	0.763	2.979
0.884	5.224	271.668	0.916	0.476	0.884	3.356
0.846	4.784	191.367	0.899	0.538	0.864	3.106
0.587	2.986	92.561	0.603	1.968	0.587	2.663
0.846	4.784	191.367	0.899	0.538	0.864	3.106
0.822	4.282	128.452	0.873	0.625	0.822	3.089
0.86	5.162	268.423	0.906	0.538	0.867	3.602
0.903	5.278	253.323	0.945	0.307	0.903	3.395
0.903	5.278	253.323	0.945	0.307	0.903	3.395
0.835	4.676	201.065	0.862	0.75	0.835	3.348
0.853	5.38	371.224	0.881	0.728	0.853	3.65
0.848	3.984	79.684	0.922	0.338	0.848	3.146
0.797	4.022	112.606	0.837	0.786	0.797	3.596
0.849	5.188	295.72	0.889	0.645	0.849	3.645
0.836	4.683	182.621	0.886	0.603	0.836	3.22
0.857	5.714	519.945	0.878	0.794	0.864	3.854
0.819	4.648	213.819	0.842	0.875	0.819	3.143
0.926	4	64	1	0	0.926	2.485
0.826	4.665	200.574	0.86	0.762	0.826	3.35
0.839	5.231	329.549	0.875	0.746	0.839	3.897
0.84	4.557	173.157	0.868	0.691	0.84	3.358
0.834	5.034	286.965	0.863	0.798	0.834	3.565
0.811	4.209	117.851	0.876	0.598	0.811	3.377
0.824	4.614	166.117	0.893	0.556	0.824	3.558
0.736	4.472	219.121	0.796	1.143	0.745	3.703
0.803	4.26	132.07	0.86	0.694	0.803	3.411
0.856	4.928	206.968	0.914	0.465	0.856	3.559
0.875	5.012	230.574	0.907	0.511	0.875	3.576
0.823	4.619	189.395	0.862	0.738	0.823	3.463
0.851	4.482	134.452	0.913	0.425	0.851	3.192
0.863	5.231	292.947	0.901	0.576	0.869	3.719
0.898	5.12	230.379	0.932	0.372	0.898	3.253
0.863	4.712	160.219	0.926	0.375	0.863	3.312
0.864	4.597	151.71	0.911	0.447	0.864	3.472
0.884	4.84	159.71	0.959	0.205	0.884	3.661

0.816	4.907	260.07	0.857	0.821	0.823	3.588
0.731	3.835	134.241	0.748	1.294	0.731	2.909
0.885	5.094	249.611	0.907	0.521	0.885	3.245
0.88	5.338	298.947	0.919	0.469	0.88	3.672
0.833	3.939	82.729	0.897	0.453	0.833	2.852
0.817	4.256	144.709	0.837	0.831	0.817	2.996
0.892	4.696	145.58	0.948	0.258	0.892	3.25
0.753	4.219	189.849	0.768	1.273	0.753	3.403
0.883	5.15	252.366	0.917	0.464	0.883	3.384
0.771	4.327	181.722	0.802	1.066	0.771	3.504
0.827	4.461	160.608	0.863	0.709	0.827	3.358
0.846	4.726	207.95	0.866	0.733	0.846	3.113
0.861	5.043	242.078	0.903	0.542	0.861	3.545
0.886	4.936	172.77	0.962	0.193	0.886	3.479
0.75	4.356	169.866	0.824	0.93	0.789	3.258
0.851	5.126	292.21	0.879	0.706	0.851	3.546
0.693	3.143	81.73	0.669	1.557	0.669	2.367
0.85	5.303	323.51	0.894	0.627	0.855	3.635
0.838	5.695	552.417	0.863	0.905	0.85	3.895
0.769	5.005	355.348	0.814	1.145	0.792	3.646
0.891	6.332	702.896	0.932	0.462	0.899	4.274
0.878	5.248	272.913	0.921	0.452	0.878	3.496
0.869	4.907	191.376	0.928	0.378	0.869	3.32
0.872	4.945	187.911	0.942	0.303	0.872	3.339
0.904	4.779	157.71	0.947	0.265	0.904	3.194
0.854	5.084	249.121	0.905	0.531	0.854	3.703
0.799	4.618	203.195	0.846	0.841	0.799	3.32
0.834	4.514	153.464	0.887	0.574	0.834	3.237

ATS2m	ATS3m	ATS4m	ATS5m	ATS6m	ATS7m	ATS8m
3.34	3.308	3.363	3.115	2.961	2.895	1.54
3.589	3.601	3.606	3.568	3.428	3.433	3.161
3.833	3.925	3.958	3.895	3.864	3.521	3.441
2.836	2.59	2.319	2.106	1.54	1.413	0
3.358	3.334	3.225	2.818	2.63	2.407	1.992
3.757	3.89	3.485	2.807	2.334	0	0
3.93	4.032	4.044	4.045	3.899	3.575	3.323
2.924	2.742	2.356	0	0	0	0
3.772	3.86	3.812	3.821	3.662	3.452	2.438
3.225	3.212	3.086	3.098	2.89	2.651	2.539
4.316	4.582	4.475	4.449	4.519	4.556	4.52
4.475	4.782	5.406	4.746	4.698	5.14	4.905
3.471	3.588	3.502	3.412	3.205	3.044	2.73
3.884	4.08	4.232	4.339	4.479	4.014	3.541
4.007	3.987	4.025	3.744	3.537	3.516	3.446
3.958	3.936	4.001	3.712	3.449	3.412	3.403
3.194	3.138	2.935	2.627	1.992	0.693	0
4.385	4.295	4.294	4.455	4.633	4.238	4.292
3.34	3.218	3.116	3.142	2.862	2.708	2.639
3.586	3.588	3.448	3.314	3.26	3.06	2.973
3.726	3.878	3.879	3.79	3.384	2.843	2.197
3.765	3.837	3.71	3.255	3.322	3.173	2.291
3.723	3.695	3.464	3.348	3.395	3.141	2.866
3.912	3.828	3.935	3.622	3.236	3.332	3.279
3.689	3.631	3.652	3.776	3.895	3.895	3.693
4.079	4.404	4.313	4.096	3.824	3.59	3.223
3.395	3.349	3.187	3.251	3.106	3.017	2.953
4.317	4.339	4.287	4.369	4.534	4.384	4.368
3.569	3.57	3.551	3.512	3.367	3.121	2.741
3.491	3.399	3.253	3.314	3.182	3.044	3.068
4.315	4.419	4.404	4.475	4.643	4.67	4.515
4.613	4.8	4.857	4.855	4.711	4.413	4.336
3.486	3.406	3.273	3.15	3.012	2.697	2.413
3.467	3.548	3.455	3.39	3.277	3.164	2.708
4.286	4.556	4.577	4.496	4.419	4.058	3.756
3.882	3.854	3.767	3.543	3.264	3.236	3.303
3.814	4.002	3.915	3.757	3.534	3.055	2.59
3.329	3.437	3.211	2.34	0.847	0	0
3.938	3.951	3.859	3.62	3.697	3.761	3.836
3.97	4.136	3.976	3.819	3.727	3.453	3.205
4.074	4.094	4.166	4.178	3.971	3.78	3.546
3.438	3.583	3.536	3.434	2.565	1.386	0
4.415	4.615	4.598	4.555	4.442	4.327	4.193
3.638	3.635	3.457	3.368	3.076	2.727	2.586
3.901	3.94	3.817	3.665	3.572	3.472	3.385
3.93	4.132	4.083	3.959	3.524	3.432	3.549
3.905	4.017	3.942	3.896	3.542	3.476	3.452
4.187	4.298	4.267	4.3	4.116	4.124	4.014
4.287	4.154	4.252	4.288	4.085	4.149	4.025

4.228	4.208	4.334	4.471	4.239	4.113	4.037
4.157	4.169	4.271	4.288	4.012	4.122	4.158
4.505	4.639	4.624	4.653	4.546	4.68	4.649
4.157	4.158	4.296	4.372	4.127	4.064	4.013
4.113	4.157	4.287	4.24	4.079	4.121	3.743
3.945	4.07	4.067	4.073	3.726	3.579	3.518
4.316	4.433	4.516	4.482	4.365	4.348	4.157
4.357	4.379	4.399	4.524	4.454	4.5	4.408
4.009	3.972	4.06	4.113	3.732	3.748	3.807
4.406	4.483	4.548	4.562	4.319	4.395	4.447
4.038	4.102	4.24	4.262	4.013	3.992	3.808
4.057	3.963	3.867	3.764	3.923	3.901	3.611
3.85	3.968	3.916	3.869	3.454	3.367	3.408
4.063	4.022	4.136	4.177	3.915	3.998	3.739
4.015	3.957	4.105	4.107	3.818	3.893	3.615
3.85	3.968	3.916	3.869	3.454	3.367	3.408
3.679	3.669	3.693	3.803	3.536	3.284	3.23
3.832	3.829	3.748	3.813	3.778	3.721	3.535
3.879	3.987	3.966	3.786	3.53	1.575	0
3.452	3.42	3.288	3.372	3.403	3.206	2.731
3.797	3.865	3.716	3.741	3.502	3.389	3.138
3.618	3.635	3.453	3.351	3.282	2.647	2.593
3.779	3.838	3.691	3.733	3.502	3.389	3.138
3.943	4.096	4.044	3.708	3.793	3.572	3.306
3.585	3.614	3.377	3.058	3.039	2.928	2.462
3.295	3.273	3.199	2.948	2.805	2.661	2.517
3.727	3.704	3.594	3.501	3.334	3.253	3.091
3.845	3.784	3.668	3.536	3.188	3.139	3.159
3.625	3.684	3.627	3.462	3.238	2.475	1.976
3.764	3.913	3.926	3.861	3.729	3.381	2.905
3.989	4.172	4.174	4.178	4.175	4.022	3.599
3.332	3.275	2.769	2.848	2.508	2.527	2.182
3.861	3.922	3.886	3.901	3.804	3.679	3.505
3.42	3.456	3.445	3.196	2.952	1.099	0
3.79	3.891	3.782	3.782	3.702	3.491	3.156
3.509	3.49	3.619	3.57	3.158	1.932	0
3.596	3.611	3.308	3.116	2.865	2.269	1.897
3.434	3.593	3.487	3.384	3.121	2.833	2.234
4.079	4.404	4.313	4.096	3.824	3.59	3.223
3.632	3.776	3.665	3.651	3.558	3.076	1.986
3.635	3.742	3.746	3.562	3.585	3.224	3.18
4.295	4.35	4.562	4.269	4.16	3.976	3.899
3.492	3.441	3.323	2.911	2.823	2.521	1.819
3.479	3.533	3.467	3.109	3.06	3.019	2.099
4.015	4.138	4.007	3.905	3.7	3.371	3.168
4.707	4.838	4.753	4.676	4.626	4.606	4.368
4.01	4.227	4.24	4.269	4.185	4.021	3.603
3.302	3.291	3.264	3.258	3.164	2.979	2.512
3.669	3.807	3.902	3.861	3.833	3.734	3.121
4.729	4.947	5.126	5.226	5.237	5.195	5.068

3.985	3.83	3.832	3.707	3.518	3.473	3.383
4.08	4.067	3.963	3.815	3.634	3.595	3.529
4.165	4.491	4.647	4.529	4.274	3.997	3.694
3.574	3.745	3.714	3.537	3.32	3.395	3.283
2.813	2.916	2.685	2.427	2.346	2.268	2.335
3.649	3.743	3.77	3.549	3.295	2.813	2.397
4.459	4.603	4.593	4.64	4.694	4.754	4.652
3.801	3.572	3.791	3.398	3.341	3.204	3.165
3.371	3.32	3.164	3.116	2.871	2.772	2.512
3.359	3.32	3.15	3.091	2.956	2.615	2.512
3.643	3.668	3.616	3.642	3.457	3.135	2.881
3.849	3.786	3.834	3.707	3.585	3.675	3.174
3.969	4.037	4.007	3.745	3.689	3.338	3.192
4.064	3.857	3.753	3.944	3.952	3.954	3.934
3.716	3.611	3.793	3.748	3.75	3.524	2.826
3.481	3.55	3.402	3.117	2.823	2.666	1.819
3.726	3.881	3.878	3.707	3.446	3.019	2.532
2.824	2.738	1.98	1.134	0	0	0
4.145	4.162	4.014	3.937	4.035	3.844	3.848
3.388	3.436	3.32	3.164	2.86	2.367	2.427
2.943	2.455	0	0	0	0	0
4.4	4.488	4.223	4.256	4.282	4.098	3.869
3.826	3.899	3.951	3.708	3.78	3.365	3.19
4.247	4.427	4.525	4.502	4.416	4.413	4.045
2.923	2.843	2.803	2.451	1.299	0	0
3.713	3.897	3.816	3.566	3.18	2.696	1.54
3.478	3.511	3.382	3.011	2.99	2.865	2.614
3.348	3.344	2.926	2.813	2.678	2.685	2.852
4.078	4.275	4.134	4.036	4.071	3.989	3.921
4.165	4.212	4.116	3.909	3.905	3.835	3.764
4.164	4.213	4.152	4.091	3.946	3.815	3.744
3.743	3.767	3.582	3.338	3.279	3.253	3.151
4.016	3.954	3.877	3.955	3.871	4.006	4.027
3.879	3.987	3.966	3.786	3.53	1.575	0
3.81	4.008	3.905	3.518	3.142	1.586	0.847
3.111	3.06	2.908	2.662	2.159	2.037	2.037
4.118	4.119	4.005	4.028	3.992	3.9	3.866
3.865	3.92	3.884	3.907	3.811	3.675	3.49
3.611	3.646	3.471	3.238	2.962	2.859	2.833
4.028	4.032	4.028	4.022	3.895	3.668	3.455
3.506	3.339	3.278	3.295	2.692	2.247	1.976
3.289	3.144	2.829	2.699	2.065	1.515	0
3.89	3.976	4.157	4.137	4.258	3.959	3.121
3.611	3.693	3.551	3.416	3.119	2.993	3.006
3.405	3.455	3.32	3.232	3.044	2.846	2.512
3.495	3.371	3.242	3.039	2.607	2.393	1.252
4.014	3.936	3.815	3.827	3.773	3.528	3.507
3.552	3.551	3.565	3.555	3.356	3.123	2.998
2.976	2.922	2.843	2.538	0	0	0
3.934	4.097	4.124	4.041	3.836	3.568	2.967

3.214	3.255	3.135	3.028	2.639	2.651	2.398
3.779	3.732	3.692	3.664	3.515	3.231	3.208
3.856	4.044	4.123	3.988	3.786	3.472	2.959
3.797	3.967	3.876	3.926	3.945	3.702	1.838
3.986	4.029	4.07	4.015	3.932	3.673	3.366
4.07	4.048	4.103	3.836	3.746	3.626	3.219
3.445	3.501	3.496	3.598	3.532	2.779	2.794
3.371	3.481	3.444	3.324	2.987	2.234	1.386
3.575	3.714	3.633	3.542	3.091	2.216	0.693
3.363	3.42	3.238	3.052	2.944	2.843	2.079
3.077	2.996	2.681	0	0	0	0
2.578	2.335	2.133	1.734	0	0	0
3.555	3.726	3.832	3.971	3.765	3.06	1.792
4.031	4.068	4.208	3.877	3.568	3.802	3.639
4.062	4.093	4.016	3.97	3.917	3.819	3.625
3.995	4.232	4.196	4.003	3.7	3.462	3.149
3.515	3.673	3.53	3.497	3.184	2.979	2.924
3.277	3.212	3.055	3.128	2.908	2.67	2.651
3.02	2.954	2.868	2.493	1.515	0	0
3.831	3.945	3.907	3.91	3.807	3.693	2.689
4.318	4.368	4.323	4.286	4.247	4.346	4.355
3.81	4.008	3.905	3.518	3.142	1.586	0.847
3.988	4.164	4.256	4.213	4.09	3.874	3.41
3.443	3.43	3.45	3.359	3.171	3.081	2.631
3.628	3.609	3.419	3.453	3.223	3.004	3.009
4.127	4.151	4.272	4.033	3.809	3.913	3.862
3.417	3.526	3.479	3.171	2.779	1.54	1.299
3.945	4.197	4.155	3.844	3.614	2.826	2.565
3.885	4.127	4.079	3.693	3.247	2.457	2.037
3.336	3.367	3.135	2.944	2.685	2.367	1.992
3.291	3.212	2.998	2.953	2.596	2.552	1.946
4.24	4.371	4.337	4.28	3.909	3.797	3.855
3.769	3.944	4.109	4.121	4.143	3.824	2.233
3.684	3.608	3.645	3.679	3.562	3.635	3.341
3.799	3.941	4.084	4.071	4.036	3.815	3.545
3.482	3.298	3.062	2.825	2.942	2.806	2.571
2.953	3.02	2.564	2.313	1.921	0.938	0
3.736	3.872	3.866	3.773	3.569	3.251	2.789
3.407	3.56	3.471	3.379	3.121	2.833	2.234
4.416	4.573	4.564	4.558	4.507	4.295	4.332
3.934	4.097	4.124	4.041	3.836	3.568	2.967
3.859	3.952	3.84	3.676	3.57	3.375	3.192
3.643	3.651	3.582	3.418	3.146	2.783	2.71
4.119	4.091	4.225	3.947	3.771	3.859	3.626
3.655	3.784	3.661	3.734	3.569	3.216	1.838
3.915	4.178	4.118	3.717	3.41	2.447	1.252
3.716	3.816	3.809	3.84	3.711	3.382	3.034
4.376	4.497	4.532	4.599	4.566	4.4	4.177
3.317	3.536	3.548	2.751	2.484	1.204	0.847
2.964	2.793	2.651	2.418	1.921	1.836	1.466

3.362	3.429	3.384	3.302	2.833	1.946	0.693
3.42	3.592	3.604	3.075	2.564	1.54	0.847
3.649	3.756	3.779	3.806	3.637	3.164	2.708
3.527	3.589	3.64	3.545	3.06	2.037	0.693
3.356	3.264	3.118	2.944	2.843	2.765	2.459
4.23	4.285	4.111	4.11	4.118	4.063	3.979
3.839	3.979	3.985	3.96	3.706	3.508	3.291
3.902	3.914	4.026	4.203	4.211	3.741	2.953
3.466	3.497	3.475	3.307	2.883	2.313	2.335
3.352	3.225	3.108	3.137	2.862	2.704	2.655
3.903	3.957	4.027	3.912	3.995	3.887	3.663
3.935	4.013	3.959	3.798	3.63	3.451	3.371
3.957	4.188	4.118	3.9	3.558	3.386	3.044
3.792	3.919	3.807	3.455	3.232	2.962	2.751
3.18	3.179	2.944	2.755	2.767	2.651	2.428
3.203	3.201	2.979	2.786	2.779	2.651	2.428
3.574	3.611	3.516	3.501	3.506	3.484	3.292
3.373	3.361	3.187	3.098	2.89	2.803	2.627
2.979	3.06	2.926	2.197	1.609	0	0
3.373	3.361	3.187	3.098	2.89	2.803	2.627
3.466	3.483	3.379	3.162	2.876	2.924	2.088
3.923	4.089	3.921	3.889	3.744	3.469	3.173
3.749	3.833	3.701	3.631	3.53	3.375	3.068
3.749	3.833	3.701	3.631	3.53	3.375	3.068
3.535	3.459	3.503	3.354	3.155	3.204	2.846
3.961	4.013	4.12	4.086	4.075	3.857	3.846
3.732	3.235	3.241	2.964	3.097	2.522	1.877
3.892	3.99	3.716	3.467	3.211	2.196	0
4.015	4.054	3.94	3.723	3.592	3.516	3.356
3.498	3.308	3.239	3.253	3.179	2.98	2.734
4.29	4.417	4.456	4.5	4.402	4.251	4.046
3.436	3.519	3.418	3.32	3.157	3.004	2.639
2.779	2.73	2.502	1.299	0	0	0
3.658	3.698	3.529	3.376	3.31	3.196	3.101
4.224	4.317	4.298	4.228	4.243	4.205	4.14
3.462	3.322	3.344	3.322	3.139	2.935	2.508
3.866	3.851	3.954	4.022	3.844	3.412	3.21
3.609	3.517	3.396	3.238	2.951	2.398	2.062
3.801	3.787	3.755	3.682	3.496	3.205	2.91
3.991	4.074	4.057	4.132	4.007	3.871	3.761
3.662	3.657	3.486	3.363	3.051	2.552	2.185
3.842	3.847	3.757	3.793	3.631	3.52	3.461
3.775	3.758	3.705	3.675	3.655	3.363	3.294
3.63	3.512	3.423	3.397	3.394	3.184	2.716
3.53	3.483	3.367	3.136	2.752	2.639	2.807
3.896	3.952	3.937	3.725	3.476	3.502	3.35
3.484	3.462	3.327	3.213	3.187	3.081	2.865
3.706	3.878	3.793	3.78	3.699	3.293	1.986
3.664	3.503	3.398	3.262	3.052	3.22	3.082
3.995	4.127	4.111	3.747	3.466	3.431	3.178

3.935	4.013	3.959	3.798	3.63	3.451	3.371
3.283	3.123	3.065	3.039	2.586	2.367	2.484
3.722	3.85	3.593	3.32	3.06	2.751	2.233
4.185	4.491	4.601	4.48	4.301	4.017	3.726
3.255	3.33	3.051	1.999	0	0	0
3.424	3.611	3.68	2.909	2.773	1.541	1.3
3.595	3.522	3.219	3.079	3.024	3.008	3.053
3.666	3.556	3.508	3.577	3.437	2.996	3.037
3.639	3.639	3.38	3.258	3.312	3.118	3.052
3.714	3.782	3.582	3.438	3.428	3.157	2.995
3.537	3.555	3.428	3.192	3.076	2.697	2.498
3.428	3.516	3.457	3.432	3.076	2.569	2.058
3.893	3.864	3.666	3.499	3.406	3.319	3.257
3.899	4.031	4	3.999	3.936	3.905	2.882
3.554	3.665	3.588	3.361	3.171	3.251	3.028
3.855	3.949	3.769	3.531	3.323	3.246	3.276
2.521	2.512	2.427	2.12	0.693	0	0
3.946	4	4.13	4.141	3.961	3.729	3.491
4.338	4.452	4.485	4.558	4.509	4.363	4.146
3.915	4.112	4.054	3.878	3.846	3.809	3.542
4.737	5.018	5.218	5.322	5.264	5.152	5.019
3.921	4.091	4.127	4.06	3.845	3.439	2.651
3.675	3.761	3.826	3.814	3.753	3.219	2.685
3.678	3.746	3.724	3.507	3.385	3.248	3.108
3.544	3.592	3.52	3.333	3.161	2.948	2.562
4.077	4.075	3.948	3.8	3.7	3.505	3.356
3.678	3.68	3.567	3.674	3.629	3.439	2.918
3.629	3.627	3.557	3.432	3.322	3.073	3.051

ATS1v	ATS2v	ATS3v	ATS4v	ATS5v	ATS6v	ATS7v
2.713	2.924	2.951	2.87	2.681	2.409	1.963
3.061	3.243	3.238	3.231	3.207	3.054	2.977
3.217	3.527	3.599	3.553	3.524	3.396	3.186
2.343	2.483	2.295	1.978	1.683	1.106	0.573
2.565	2.841	2.746	2.569	2.287	2	1.812
2.416	2.626	2.628	2.302	1.803	1.142	0
3.275	3.582	3.701	3.727	3.747	3.569	3.258
2.215	2.485	2.184	1.553	0	0	0
3.15	3.485	3.6	3.55	3.498	3.253	2.883
2.829	2.978	2.91	2.857	2.819	2.561	2.408
3.493	3.772	3.847	3.79	3.821	3.926	3.916
3.469	3.775	3.874	3.911	3.788	3.818	3.938
3.139	3.456	3.575	3.487	3.381	3.126	2.9
3.226	3.514	3.699	3.777	3.859	3.79	3.48
3.174	3.518	3.493	3.35	3.051	2.981	3.004
3.152	3.487	3.462	3.332	3.026	2.936	2.972
2.623	2.897	2.882	2.648	2.461	1.874	0.693
3.459	3.769	3.794	3.773	3.88	3.843	3.748
2.841	3.006	2.893	2.791	2.72	2.412	2.353
3.051	3.353	3.343	2.944	2.935	2.925	2.886
3.033	3.406	3.471	3.48	3.436	3.142	2.647
2.81	3.176	3.094	2.943	2.757	2.768	2.473
3.065	3.376	3.328	3.107	3.003	2.888	2.677
3.122	3.444	3.389	3.255	2.92	2.847	2.899
3.303	3.543	3.429	3.464	3.6	3.77	3.773
3.366	3.858	4.067	3.961	3.666	3.372	3.125
3.042	3.121	3.015	2.94	2.933	2.801	2.843
3.729	4.061	4.125	4.061	4.103	4.224	4.165
3.107	3.442	3.445	3.392	3.376	3.265	3.034
2.994	3.144	3.051	2.941	2.935	2.817	2.769
3.578	3.884	3.916	3.882	3.926	4.014	4.08
3.793	4.227	4.343	4.373	4.325	4.249	4.049
2.94	3.252	3.13	3.026	2.86	2.671	2.533
3.044	3.286	3.322	3.208	3.129	3.113	3.087
3.615	4.11	4.323	4.343	4.264	4.155	3.876
3.298	3.614	3.589	3.401	3.243	3.01	2.959
3.3	3.672	3.816	3.739	3.574	3.279	2.911
2.457	2.918	2.899	2.604	1.956	0.413	0
3.451	3.82	3.852	3.733	3.528	3.612	3.639
3.297	3.708	3.789	3.636	3.473	3.341	3.135
3.477	3.8	3.844	3.867	3.845	3.704	3.522
2.96	3.257	3.42	3.34	3.146	2.343	1.386
3.74	4.247	4.457	4.428	4.308	4.216	4.043
3.016	3.37	3.361	3.141	2.898	2.62	2.307
3.38	3.651	3.656	3.529	3.369	3.215	3.074
3.152	3.418	3.464	3.331	3.093	2.919	2.946
3.174	3.451	3.496	3.349	3.116	2.965	2.978
3.365	3.645	3.687	3.59	3.488	3.448	3.459
3.276	3.557	3.525	3.502	3.389	3.326	3.349

3.378	3.72	3.697	3.71	3.695	3.632	3.501
3.279	3.526	3.56	3.567	3.461	3.352	3.361
3.679	3.99	4.098	4.018	3.954	4.014	4.074
3.26	3.526	3.569	3.58	3.501	3.405	3.35
3.224	3.504	3.539	3.547	3.429	3.338	3.323
3.254	3.513	3.583	3.504	3.345	3.175	3.084
3.519	3.813	3.887	3.831	3.746	3.711	3.66
3.515	3.809	3.8	3.775	3.818	3.861	3.842
3.114	3.374	3.373	3.329	3.19	3.064	3.048
3.456	3.749	3.81	3.786	3.687	3.639	3.678
3.199	3.458	3.508	3.538	3.429	3.315	3.242
3.209	3.522	3.504	3.417	3.392	3.344	3.21
3.152	3.418	3.464	3.331	3.093	2.919	2.946
3.331	3.597	3.552	3.524	3.457	3.42	3.373
3.195	3.44	3.413	3.399	3.273	3.174	3.148
3.152	3.418	3.464	3.331	3.093	2.919	2.946
3.211	3.436	3.447	3.314	3.312	3.167	2.995
3.266	3.531	3.559	3.524	3.617	3.513	3.297
2.773	3.122	3.156	2.893	2.487	2.074	1
2.969	3.25	3.21	3.112	3.176	3.13	2.838
3.107	3.451	3.474	3.396	3.331	3.173	2.972
2.744	2.936	2.882	2.658	2.529	2.42	2.222
3.147	3.498	3.53	3.433	3.352	3.173	2.972
3.108	3.472	3.514	3.366	3.139	3.01	2.797
2.94	3.283	3.232	2.974	2.768	2.559	2.372
2.696	2.837	2.763	2.622	2.484	2.316	2.165
3.151	3.428	3.379	3.241	3.148	3.027	2.968
3.282	3.55	3.571	3.409	3.216	2.961	2.924
2.842	3.185	3.24	3.119	2.89	2.522	1.718
3.157	3.427	3.579	3.522	3.424	3.172	2.899
3.208	3.505	3.543	3.53	3.5	3.46	3.349
2.569	2.824	2.681	2.445	2.357	1.953	1.614
3.205	3.55	3.634	3.562	3.519	3.385	3.16
2.651	2.903	2.745	2.601	2.447	2.341	1.099
3.176	3.521	3.603	3.472	3.557	3.42	3.103
2.522	2.673	2.707	2.708	2.521	2.079	1.099
2.872	3.155	3.101	2.813	2.678	2.469	2.052
3.067	3.338	3.483	3.43	3.335	3.034	2.651
3.366	3.858	4.067	3.961	3.666	3.372	3.125
3.044	3.369	3.488	3.381	3.285	3.088	2.504
2.966	3.221	3.214	3.097	3.08	3.047	2.642
3.376	3.759	3.799	3.742	3.541	3.468	3.337
2.728	3.039	2.912	2.799	2.515	2.326	1.893
2.854	3.133	3.119	2.884	2.694	2.46	2.018
3.386	3.858	3.982	3.863	3.642	3.398	3.084
3.956	4.362	4.483	4.4	4.313	4.286	4.146
3.311	3.638	3.796	3.796	3.852	3.813	3.674
2.95	3.188	3.109	3.033	3.153	3.081	2.878
3.206	3.509	3.681	3.694	3.588	3.632	3.535
3.991	4.382	4.569	4.654	4.699	4.76	4.744

3.274	3.504	3.442	3.348	3.281	3.121	3.002
3.403	3.74	3.749	3.614	3.409	3.221	3.25
3.43	3.884	4.089	4.091	3.958	3.727	3.48
2.93	3.193	3.293	3.203	3.067	2.898	2.789
2.468	2.501	2.534	2.345	2.055	1.96	2.082
3.065	3.405	3.529	3.499	3.251	2.976	2.53
3.644	4.05	4.158	4.162	4.208	4.217	4.184
2.864	3.007	2.864	2.871	2.682	2.51	2.439
2.972	3.191	3.16	2.973	2.837	2.607	2.404
2.893	3.106	3.049	2.867	2.837	2.677	2.487
3.246	3.479	3.509	3.454	3.482	3.308	3.01
3.161	3.447	3.396	3.331	3.311	3.213	3.099
3.36	3.82	3.907	3.802	3.593	3.55	3.114
3.127	3.328	3.281	3.243	3.295	3.259	3.165
2.957	3.283	3.15	3.187	3.216	3.161	2.938
2.967	3.291	3.255	3.033	2.802	2.553	2.109
2.997	3.351	3.492	3.425	3.255	2.815	2.394
1.976	2.113	1.827	0.932	0.19	0	0
3.373	3.695	3.775	3.656	3.568	3.469	3.408
2.914	3.182	3.19	2.98	2.753	2.384	2.2
1.613	1.567	0.945	0	0	0	0
3.709	4.022	4.083	3.994	3.939	3.845	3.722
2.965	3.249	3.214	3.108	2.964	2.869	2.666
3.627	4.071	4.279	4.308	4.187	4.12	3.982
2.461	2.707	2.647	2.318	1.696	0.705	0
3.075	3.511	3.662	3.545	3.297	2.811	2.123
2.675	2.953	2.883	2.676	2.502	2.38	2.235
2.836	3.128	3.002	2.678	2.53	2.444	2.567
3.288	3.611	3.631	3.561	3.468	3.576	3.474
3.467	3.754	3.745	3.541	3.403	3.417	3.432
3.449	3.709	3.706	3.544	3.446	3.43	3.413
3.159	3.514	3.55	3.255	2.986	2.999	3.044
3.206	3.429	3.416	3.308	3.36	3.43	3.415
2.773	3.122	3.156	2.893	2.487	2.074	1
3.141	3.616	3.766	3.636	3.2	2.596	1.053
2.71	2.959	2.893	2.711	2.402	1.949	1.796
3.601	3.907	3.896	3.734	3.801	3.774	3.701
3.205	3.545	3.626	3.553	3.524	3.385	3.173
3.074	3.399	3.397	3.185	2.959	2.768	2.655
3.491	3.816	3.803	3.804	3.814	3.703	3.505
2.416	2.713	2.5	2.394	2.102	1.759	0.928
2.316	2.61	2.279	2.015	1.552	1.225	0.421
3.201	3.546	3.681	3.745	3.765	3.716	3.364
3.07	3.343	3.353	3.181	3.047	2.807	2.57
2.972	3.231	3.223	3.049	2.975	2.777	2.53
2.595	2.838	2.728	2.532	2.319	1.997	1.522
3.142	3.409	3.366	3.254	3.173	3.106	2.954
3.107	3.396	3.389	3.358	3.379	3.176	2.898
2.38	2.581	2.508	2.455	1.878	0	0
3.187	3.592	3.716	3.638	3.552	3.297	2.99

2.772	2.998	3.051	2.881	2.628	2.424	2.617
3.046	3.331	3.351	3.265	3.145	2.852	2.734
3.132	3.484	3.609	3.569	3.419	3.184	2.883
3.082	3.398	3.507	3.399	3.393	3.226	2.622
3.395	3.691	3.735	3.721	3.734	3.599	3.288
3.106	3.462	3.471	3.336	3.138	2.984	2.917
2.879	3.066	3.101	3.096	3.002	2.67	2.219
2.869	3.186	3.269	3.135	3.052	2.717	2.127
3.071	3.435	3.568	3.486	3.413	2.953	2.163
2.89	3.18	3.179	2.946	2.75	2.791	2.815
2.267	2.547	2.339	1.868	0	0	0
1.881	2.067	1.881	1.596	1.33	0	0
3.161	3.479	3.631	3.705	3.815	3.625	3.015
3.167	3.536	3.581	3.509	3.211	3.108	3.157
3.367	3.645	3.685	3.593	3.513	3.447	3.379
3.352	3.831	4.011	3.931	3.65	3.398	3.027
2.874	3.125	3.22	3.005	2.802	2.692	2.6
2.83	3.003	2.91	2.767	2.759	2.523	2.413
2.24	2.479	2.456	2.182	1.565	0.421	0
3.189	3.534	3.655	3.607	3.56	3.343	3.016
3.786	4.075	4.1	4.026	3.987	4.03	4.109
3.141	3.616	3.766	3.636	3.2	2.596	1.053
3.351	3.676	3.799	3.805	3.782	3.615	3.364
2.87	3.159	3.175	3.127	3.101	2.951	2.714
3.042	3.346	3.245	3.089	3.025	2.725	2.684
3.358	3.71	3.77	3.708	3.481	3.41	3.454
2.773	3.089	3.164	3.009	2.821	2.173	1.106
3.337	3.747	3.927	3.906	3.625	3.305	2.699
3.224	3.673	3.862	3.789	3.411	2.951	2.305
2.836	3.149	3.181	2.894	2.644	2.357	1.881
2.595	3.015	2.872	2.706	2.613	2.307	2.081
3.45	3.885	3.962	3.928	3.752	3.523	3.391
3.085	3.435	3.62	3.674	3.684	3.496	2.997
2.949	3.174	3.11	3.058	3.108	3.02	2.883
3.13	3.451	3.628	3.669	3.674	3.529	3.226
2.596	2.839	2.573	2.399	2.266	2.307	2.115
2.419	2.622	2.516	2.073	1.646	1.038	0.304
3.068	3.404	3.522	3.429	3.284	3.026	2.757
3.109	3.391	3.547	3.456	3.346	3.034	2.651
3.671	4.011	4.094	4.071	4.048	4	3.927
3.187	3.592	3.716	3.638	3.552	3.297	2.99
3.133	3.466	3.483	3.351	3.091	3.01	2.964
3.094	3.477	3.463	3.358	3.179	2.87	2.554
3.305	3.661	3.654	3.577	3.383	3.32	3.263
3.035	3.329	3.413	3.305	3.287	3.043	2.421
3.185	3.682	3.874	3.767	3.361	2.9	2.066
3.17	3.425	3.458	3.479	3.563	3.445	3.171
3.729	4.192	4.356	4.353	4.394	4.39	4.214
2.661	2.961	3.148	2.957	2.437	1.939	0.921
2.479	2.602	2.559	2.419	2.126	1.551	1.244

3	3.276	3.349	3.335	3.284	2.833	1.946
2.792	3.105	3.23	3.056	2.698	2.073	1.106
3.148	3.391	3.425	3.463	3.552	3.417	3.092
2.986	3.29	3.335	3.328	3.229	2.738	1.796
2.852	3.079	2.931	2.801	2.635	2.455	2.412
3.472	3.817	3.895	3.787	3.71	3.626	3.646
3.221	3.554	3.676	3.596	3.591	3.374	3.3
3.182	3.465	3.434	3.482	3.624	3.609	3.286
2.84	3.138	3.097	3.032	2.756	2.133	2.043
2.823	2.975	2.876	2.81	2.749	2.412	2.339
3.336	3.602	3.668	3.63	3.629	3.623	3.554
3.228	3.573	3.621	3.508	3.434	3.262	3.013
3.316	3.787	3.955	3.828	3.525	3.234	2.951
3.259	3.715	3.851	3.691	3.316	3.093	2.776
2.752	2.93	2.934	2.618	2.429	2.421	2.408
2.729	2.905	2.907	2.563	2.379	2.409	2.408
3.196	3.366	3.317	3.227	3.231	3.219	3.175
2.94	3.163	3.107	2.94	2.819	2.561	2.535
2.604	2.892	2.893	2.678	2.197	1.609	0
2.94	3.163	3.107	2.94	2.819	2.561	2.535
2.843	3.123	3.176	3.04	2.83	2.44	2.011
3.306	3.601	3.727	3.606	3.665	3.537	3.197
3.219	3.581	3.64	3.452	3.352	3.28	3.164
3.219	3.581	3.64	3.452	3.352	3.28	3.164
2.861	3.066	2.936	2.912	2.867	2.593	2.508
3.467	3.727	3.788	3.822	3.871	3.801	3.66
2.604	2.875	2.722	2.46	2.055	1.726	1.329
2.701	2.971	2.92	2.699	2.566	2.206	1.404
3.403	3.739	3.771	3.589	3.448	3.334	3.203
2.967	3.315	3.16	3.057	3.032	2.914	2.711
3.662	4.104	4.264	4.291	4.282	4.218	4.045
2.995	3.248	3.286	3.179	3.037	2.97	2.98
2.255	2.426	2.26	1.626	0.705	0	0
3.098	3.389	3.39	2.997	2.948	2.952	2.928
3.412	3.762	3.864	3.816	3.78	3.753	3.699
2.807	3.01	2.927	2.864	2.785	2.63	2.352
3.291	3.581	3.607	3.679	3.734	3.558	3.195
2.767	3.018	2.869	2.638	2.566	2.454	2.13
3.087	3.346	3.278	3.18	3.16	3.039	2.886
3.402	3.663	3.677	3.656	3.729	3.661	3.503
2.828	3.114	3.009	2.702	2.711	2.612	2.361
3.264	3.592	3.639	3.526	3.463	3.292	3.071
3.043	3.33	3.341	3.197	3.02	2.974	2.96
2.951	3.283	3.207	3.063	3.005	2.899	2.647
2.929	3.202	3.101	2.959	2.756	2.453	2.212
3.235	3.467	3.513	3.457	3.317	3.092	3.035
3.038	3.251	3.188	3.003	2.842	2.755	2.679
3.068	3.398	3.522	3.448	3.359	3.177	2.584
2.771	3.032	2.93	2.864	2.631	2.419	2.397
3.06	3.396	3.411	3.262	2.989	2.864	2.843

3.228	3.573	3.621	3.508	3.434	3.262	3.013
2.703	2.988	2.78	2.583	2.484	2.342	2.2
3.179	3.639	3.739	3.498	3.16	2.893	2.447
3.43	3.895	4.089	4.072	3.933	3.729	3.501
2.395	2.803	2.738	2.452	1.405	0	0
2.701	3.002	3.196	3.016	2.533	2.161	1.128
2.958	3.271	3.134	2.806	2.759	2.714	2.578
2.887	3.171	2.984	2.998	2.957	2.613	2.561
3.142	3.334	3.264	3.046	2.91	2.846	2.789
2.982	3.204	3.241	2.864	2.786	2.782	2.696
2.807	2.988	2.937	2.705	2.606	2.522	2.334
2.924	3.268	3.295	3.219	3.174	2.859	2.357
3.232	3.538	3.527	3.306	3.151	3.015	2.948
3.192	3.544	3.679	3.625	3.584	3.386	3.071
2.904	3.222	3.29	3.162	3.064	2.799	2.683
3.21	3.533	3.605	3.428	3.251	3.031	2.922
2.2	2.228	2.202	2.085	1.619	0.693	0
3.416	3.693	3.759	3.832	3.826	3.707	3.47
3.712	4.146	4.304	4.307	4.343	4.333	4.169
3.397	3.742	3.894	3.822	3.68	3.554	3.554
4.039	4.52	4.755	4.94	4.969	4.954	4.861
3.322	3.732	3.918	3.942	3.814	3.562	3.249
3.16	3.445	3.501	3.471	3.472	3.312	2.999
3.102	3.404	3.504	3.483	3.298	3.125	2.923
2.753	3.05	3.023	2.899	2.759	2.547	2.248
3.388	3.744	3.706	3.515	3.386	3.283	3.146
3.139	3.494	3.481	3.375	3.422	3.421	3.24
2.977	3.299	3.296	3.144	3.067	2.877	2.505

ATS8v	ATS1e	ATS2e	ATS3e	ATS4e	ATS5e	ATS6e
1.106	2.994	3.337	3.306	3.36	3.113	2.959
2.853	3.306	3.587	3.599	3.604	3.565	3.426
2.94	3.462	3.831	3.923	3.956	3.893	3.861
0	2.563	2.833	2.588	2.317	2.103	1.538
1.367	3.04	3.353	3.329	3.219	2.813	2.626
0	2.748	3.372	3.47	3.372	2.536	2.326
3.031	3.609	3.926	4.028	4.041	4.042	3.897
0	2.612	2.919	2.737	2.349	0	0
2.09	3.323	3.688	3.784	3.733	3.688	3.416
2.389	3.011	3.224	3.21	3.084	3.097	2.888
3.792	3.902	4.313	4.578	4.471	4.445	4.516
3.72	3.587	3.901	4.017	4	3.938	3.894
2.667	3.198	3.471	3.588	3.501	3.412	3.204
3.085	3.507	3.81	3.988	4.154	4.245	4.299
2.93	3.426	3.842	3.885	3.869	3.648	3.427
2.903	3.382	3.784	3.829	3.841	3.613	3.329
0	2.851	3.191	3.135	2.933	2.626	1.992
3.709	3.856	4.341	4.269	4.269	4.425	4.575
2.435	3.081	3.337	3.216	3.113	3.14	2.859
2.758	3.25	3.585	3.586	3.445	3.312	3.258
2.197	3.312	3.723	3.874	3.875	3.787	3.382
1.237	3.238	3.586	3.534	3.438	3.25	3.316
2.487	3.254	3.636	3.605	3.408	3.285	3.24
2.781	3.342	3.73	3.709	3.763	3.514	3.169
3.617	3.454	3.687	3.629	3.65	3.775	3.894
2.761	3.529	4.071	4.392	4.303	4.085	3.81
2.787	3.256	3.394	3.347	3.185	3.25	3.104
4.089	3.917	4.315	4.337	4.285	4.367	4.532
2.645	3.276	3.568	3.569	3.549	3.51	3.366
2.876	3.281	3.489	3.397	3.252	3.312	3.18
4.05	3.869	4.246	4.332	4.313	4.366	4.503
3.958	3.984	4.485	4.652	4.717	4.657	4.548
2.37	3.105	3.484	3.404	3.271	3.148	3.01
2.708	3.183	3.465	3.546	3.453	3.388	3.275
3.522	3.771	4.285	4.554	4.576	4.494	4.417
3.073	3.548	3.879	3.852	3.764	3.54	3.262
2.527	3.406	3.813	4.001	3.914	3.756	3.533
0	2.913	3.324	3.433	3.206	2.337	0.845
3.641	3.526	3.938	3.951	3.859	3.619	3.697
2.896	3.51	3.968	4.134	3.974	3.816	3.724
3.225	3.725	4.072	4.092	4.163	4.176	3.969
0	3.09	3.436	3.582	3.534	3.432	2.563
3.845	3.884	4.414	4.614	4.597	4.553	4.441
2.085	3.146	3.543	3.54	3.344	3.162	2.905
3.028	3.609	3.899	3.938	3.816	3.663	3.57
2.893	3.391	3.746	3.849	3.829	3.698	3.346
2.921	3.426	3.795	3.887	3.831	3.716	3.432
3.36	3.67	4.012	4.064	4.032	4.034	3.912
3.195	3.631	3.965	3.884	3.954	3.96	3.747

3.264	3.703	4.061	4.074	4.181	4.224	4.082
3.303	3.626	3.976	4.028	4.111	4.069	3.88
4.026	4.011	4.38	4.478	4.465	4.473	4.423
3.171	3.635	3.976	4.016	4.132	4.147	3.94
3.039	3.561	3.934	3.976	4.087	4.061	3.871
3.008	3.489	3.839	3.946	3.938	3.891	3.636
3.562	3.798	4.196	4.25	4.373	4.28	4.154
3.682	3.83	4.211	4.267	4.256	4.314	4.329
2.935	3.463	3.797	3.834	3.892	3.847	3.555
3.627	3.826	4.19	4.273	4.281	4.253	4.107
3.064	3.613	3.941	3.981	4.123	4.113	3.865
2.938	3.477	3.97	3.881	3.812	3.7	3.818
2.893	3.382	3.734	3.831	3.802	3.683	3.335
3.134	3.552	3.876	3.86	3.936	3.956	3.74
2.908	3.478	3.817	3.783	3.894	3.9	3.608
2.893	3.382	3.734	3.831	3.802	3.683	3.335
2.989	3.354	3.594	3.626	3.609	3.62	3.385
3.003	3.482	3.755	3.751	3.706	3.774	3.689
0	3.096	3.608	3.575	3.508	3.078	3.253
2.163	3.094	3.338	3.302	3.221	3.311	3.283
2.658	3.234	3.55	3.585	3.485	3.421	3.278
1.975	3.008	3.367	3.307	3.282	3.014	2.92
2.658	3.215	3.526	3.557	3.463	3.411	3.278
2.294	3.311	3.804	3.849	3.819	3.51	3.515
1.826	3.117	3.486	3.517	3.315	2.974	2.828
2.169	2.991	3.165	3.045	2.88	2.728	2.588
2.823	3.432	3.724	3.701	3.592	3.499	3.332
2.937	3.424	3.811	3.766	3.647	3.512	3.172
0.935	3.224	3.622	3.681	3.623	3.458	3.234
2.551	3.425	3.755	3.902	3.914	3.844	3.713
3.079	3.471	3.846	4.02	3.987	3.911	3.863
0.705	2.808	3.202	3.136	2.657	2.707	2.358
2.851	3.414	3.785	3.851	3.849	3.864	3.71
0	2.862	3.167	3.043	2.86	2.804	2.516
2.623	3.363	3.709	3.817	3.693	3.694	3.559
0	2.827	3.147	3.169	3.043	2.954	2.268
1.564	3.012	3.402	3.42	3.182	2.934	2.861
2.127	3.17	3.433	3.591	3.486	3.384	3.12
2.761	3.529	4.071	4.392	4.303	4.085	3.81
1.507	3.194	3.537	3.694	3.573	3.491	3.282
1.959	3.114	3.438	3.448	3.348	3.348	3.254
3.352	3.633	4.064	4.13	4.246	4.031	3.891
1.173	3.125	3.488	3.436	3.318	2.908	2.819
1.21	3.086	3.462	3.513	3.437	3.091	3.034
2.853	3.516	4.014	4.137	4.006	3.904	3.698
3.943	4.228	4.705	4.836	4.751	4.674	4.624
3.244	3.558	3.886	4.034	4.081	4.165	4.121
2.433	3.097	3.301	3.29	3.263	3.257	3.163
3.034	3.342	3.667	3.805	3.9	3.858	3.831
4.66	4.256	4.727	4.945	5.123	5.224	5.234

2.933	3.55	3.92	3.679	3.681	3.623	3.399
3.198	3.638	4.019	4.005	3.888	3.729	3.522
3.159	3.67	4.163	4.489	4.644	4.525	4.271
2.512	3.253	3.571	3.742	3.71	3.534	3.317
1.953	2.727	2.81	2.912	2.683	2.424	2.342
1.803	3.244	3.647	3.741	3.768	3.547	3.294
4.014	4.029	4.457	4.601	4.591	4.637	4.691
2.356	3.176	3.539	3.17	3.316	3.016	2.85
2.202	3.09	3.37	3.319	3.163	3.114	2.87
2.202	3.059	3.358	3.318	3.148	3.089	2.954
2.747	3.377	3.641	3.666	3.615	3.64	3.455
2.713	3.273	3.608	3.535	3.523	3.532	3.427
2.909	3.475	3.968	4.036	4.006	3.744	3.688
3.056	3.555	4.008	3.833	3.733	3.912	3.914
2.158	3.351	3.7	3.591	3.766	3.733	3.723
1.34	3.096	3.473	3.542	3.396	3.11	2.807
2.191	3.334	3.717	3.873	3.87	3.692	3.427
0	2.464	2.805	2.704	1.958	1.075	0
3.386	3.619	4.009	4.01	3.907	3.854	3.832
2.085	3.05	3.378	3.423	3.301	3.141	2.831
0	2.107	2.671	2.448	0	0	0
3.547	3.882	4.328	4.416	4.165	4.121	4.13
2.343	3.261	3.669	3.588	3.652	3.441	3.58
3.748	3.768	4.246	4.426	4.524	4.5	4.414
0	2.626	2.922	2.841	2.8	2.447	1.296
1.106	3.3	3.712	3.896	3.814	3.564	3.178
1.924	3.146	3.473	3.506	3.376	3.006	2.985
2.642	3.011	3.346	3.342	2.925	2.812	2.676
3.331	3.652	4.075	4.271	4.13	4.032	4.068
3.429	3.7	4.084	4.097	4.059	3.805	3.801
3.403	3.678	4.025	4.041	4.039	3.88	3.772
2.978	3.366	3.74	3.764	3.579	3.335	3.276
3.288	3.524	3.922	3.87	3.823	3.902	3.776
0	3.096	3.608	3.575	3.508	3.078	3.253
0.413	3.325	3.809	4.006	3.903	3.516	3.139
1.796	2.813	3.11	3.059	2.907	2.661	2.158
3.64	3.804	4.116	4.117	4.002	4.026	3.989
2.868	3.414	3.79	3.849	3.847	3.871	3.711
2.491	3.237	3.609	3.644	3.469	3.237	2.96
3.298	3.676	4.026	4.03	4.026	4.02	3.893
0.885	3.144	3.501	3.335	3.274	3.289	2.688
0	2.922	3.285	3.14	2.825	2.693	2.06
2.763	3.517	3.887	3.974	4.154	4.135	4.254
2.447	3.188	3.515	3.558	3.393	3.297	3.04
2.202	3.09	3.404	3.454	3.318	3.23	3.043
0.791	2.966	3.315	3.22	3.104	2.726	2.478
2.871	3.452	3.879	3.783	3.609	3.643	3.706
2.676	3.276	3.55	3.548	3.562	3.553	3.353
0	2.66	2.972	2.919	2.84	2.534	0
2.604	3.512	3.926	4.087	4.111	4.024	3.822

2.398	2.951	3.212	3.252	3.133	3.025	2.637
2.779	3.424	3.77	3.72	3.676	3.648	3.493
2.626	3.439	3.843	4.025	4.093	3.96	3.759
1.256	3.251	3.631	3.791	3.675	3.687	3.573
3.072	3.604	3.913	3.928	3.967	3.949	3.826
2.722	3.366	3.809	3.896	3.89	3.717	3.372
2.208	3.083	3.329	3.372	3.266	3.273	3.01
1.386	3.066	3.369	3.48	3.443	3.322	2.985
0.693	3.23	3.573	3.712	3.631	3.54	3.089
2.079	3.051	3.361	3.418	3.236	3.05	2.943
0	2.592	2.899	2.701	2.223	0	0
0	2.33	2.571	2.33	2.126	1.73	0
1.792	3.251	3.555	3.725	3.831	3.97	3.764
3.067	3.483	3.871	3.975	4.079	3.793	3.471
3.247	3.69	4.057	4.089	4.012	3.967	3.913
2.716	3.485	3.994	4.231	4.195	4.001	3.698
2.55	3.144	3.407	3.525	3.422	3.276	3.031
2.419	3.089	3.275	3.21	3.053	3.126	2.906
0	2.732	3.015	2.95	2.864	2.488	1.509
2.207	3.362	3.747	3.868	3.827	3.781	3.584
4.161	3.865	4.202	4.189	4.179	4.072	4.097
0.413	3.325	3.809	4.006	3.903	3.516	3.139
3.102	3.621	3.981	4.155	4.243	4.198	4.077
2.474	3.141	3.441	3.429	3.448	3.358	3.169
2.7	3.256	3.627	3.607	3.417	3.451	3.22
3.393	3.581	3.982	4.065	4.151	3.962	3.733
0.705	3.034	3.414	3.523	3.475	3.169	2.775
2.355	3.49	3.943	4.195	4.154	3.842	3.612
1.796	3.394	3.883	4.125	4.077	3.691	3.245
1.398	3.011	3.335	3.366	3.134	2.943	2.684
1.946	2.95	3.289	3.21	2.996	2.951	2.593
3.299	3.666	4.066	4.206	4.18	4.052	3.791
1.8	3.418	3.766	3.942	4.106	4.119	4.139
2.481	3.267	3.64	3.515	3.493	3.592	3.404
2.658	3.451	3.796	3.939	4.081	4.069	4.033
1.724	3.122	3.477	3.293	3.058	2.821	2.937
0	2.649	2.951	3.017	2.562	2.309	1.915
2.381	3.357	3.728	3.861	3.852	3.755	3.551
2.127	3.149	3.407	3.56	3.471	3.378	3.12
3.852	3.984	4.414	4.57	4.561	4.555	4.503
2.604	3.512	3.926	4.087	4.111	4.024	3.822
2.902	3.396	3.738	3.813	3.649	3.481	3.404
2.507	3.281	3.641	3.649	3.58	3.416	3.143
3.201	3.564	3.973	4	4.098	3.869	3.692
1.256	3.201	3.562	3.701	3.568	3.587	3.356
0.791	3.416	3.914	4.176	4.116	3.715	3.407
2.821	3.366	3.713	3.813	3.806	3.838	3.709
4	3.917	4.375	4.496	4.531	4.598	4.565
0.413	2.924	3.313	3.533	3.544	2.749	2.481
1.256	2.728	2.962	2.791	2.649	2.415	1.919

0.693	3.067	3.36	3.428	3.384	3.302	2.833
0.413	3.026	3.417	3.59	3.6	3.073	2.562
2.708	3.319	3.646	3.753	3.776	3.805	3.636
0.693	3.148	3.524	3.587	3.638	3.543	3.058
2.29	3.074	3.353	3.262	3.116	2.942	2.84
3.632	3.662	4.023	4.112	3.964	3.914	3.889
3.09	3.488	3.836	3.976	3.981	3.957	3.703
2.622	3.433	3.832	3.8	3.897	4.027	4.108
1.953	3.016	3.353	3.319	3.24	3.022	2.59
2.421	3.089	3.349	3.222	3.106	3.135	2.859
3.372	3.544	3.901	3.956	4.025	3.91	3.993
2.942	3.585	3.931	4.009	3.956	3.794	3.626
2.611	3.454	3.956	4.186	4.116	3.898	3.556
2.447	3.32	3.791	3.918	3.806	3.454	3.231
2.341	2.959	3.178	3.177	2.941	2.752	2.764
2.341	2.977	3.2	3.199	2.976	2.783	2.776
3.104	3.354	3.573	3.609	3.514	3.5	3.505
2.451	3.105	3.371	3.359	3.185	3.097	2.888
0	2.662	2.978	3.059	2.925	2.197	1.609
2.451	3.105	3.371	3.359	3.185	3.097	2.888
1.307	3.007	3.352	3.372	3.316	3.085	2.662
2.819	3.509	3.786	3.906	3.813	3.82	3.664
2.869	3.394	3.748	3.832	3.699	3.629	3.528
2.869	3.394	3.748	3.832	3.699	3.629	3.528
2.457	3.226	3.435	3.263	3.344	3.158	2.895
3.58	3.649	3.959	4.011	4.118	4.084	4.073
0.618	2.978	3.5	3.082	3.046	2.833	2.622
0	2.985	3.564	3.619	3.535	3.209	3.062
3.105	3.64	4.008	4.048	3.931	3.714	3.586
2.463	3.217	3.496	3.306	3.236	3.25	3.176
3.841	3.852	4.288	4.415	4.455	4.499	4.4
2.639	3.141	3.434	3.516	3.416	3.318	3.155
0	2.483	2.777	2.727	2.498	1.296	0
2.821	3.348	3.656	3.696	3.526	3.374	3.308
3.615	3.724	4.123	4.225	4.223	4.11	4.169
1.929	3.044	3.407	3.198	3.222	3.197	2.961
2.905	3.469	3.758	3.815	3.916	3.962	3.772
1.739	3.069	3.466	3.294	3.196	3.089	2.947
2.701	3.308	3.685	3.621	3.582	3.543	3.391
3.34	3.562	3.896	3.986	3.968	3.994	3.945
1.909	3.115	3.528	3.458	3.255	3.232	3.047
2.904	3.391	3.764	3.77	3.712	3.747	3.576
2.862	3.331	3.696	3.678	3.62	3.503	3.565
2.319	3.184	3.539	3.41	3.311	3.281	3.206
2.166	3.051	3.378	3.36	3.249	2.985	2.643
2.983	3.511	3.827	3.842	3.837	3.684	3.361
2.605	3.25	3.481	3.459	3.324	3.209	3.183
1.507	3.247	3.617	3.803	3.712	3.641	3.464
2.131	3.171	3.601	3.446	3.276	3.181	2.876
2.654	3.33	3.762	3.858	3.851	3.558	3.291

2.942	3.585	3.931	4.009	3.956	3.794	3.626
1.956	2.907	3.281	3.121	3.062	3.035	2.583
1.8	3.245	3.721	3.849	3.592	3.319	3.059
3.181	3.67	4.183	4.489	4.598	4.476	4.298
0	2.848	3.25	3.325	3.046	1.995	0
0.736	2.911	3.292	3.518	3.517	2.717	2.416
2.226	3.04	3.341	3.242	2.968	2.819	2.748
2.602	3.268	3.578	3.431	3.351	3.43	3.224
2.75	3.382	3.637	3.636	3.378	3.255	3.308
2.705	3.238	3.587	3.601	3.476	3.267	3.316
2.222	3.044	3.383	3.324	3.315	3.049	2.996
1.744	3.112	3.427	3.515	3.455	3.43	3.074
2.927	3.492	3.819	3.788	3.565	3.39	3.285
2.28	3.367	3.75	3.867	3.824	3.779	3.575
2.455	3.255	3.55	3.662	3.585	3.359	3.168
3.02	3.542	3.851	3.946	3.765	3.529	3.32
0	2.366	2.519	2.51	2.426	2.117	0.693
3.236	3.633	3.944	3.998	4.127	4.139	3.958
3.963	3.894	4.336	4.45	4.483	4.557	4.508
3.339	3.644	3.914	4.11	4.053	3.876	3.843
4.737	4.272	4.736	5.016	5.216	5.319	5.261
2.617	3.495	3.919	4.089	4.125	4.059	3.843
2.567	3.319	3.673	3.76	3.824	3.812	3.75
2.788	3.336	3.675	3.743	3.721	3.505	3.382
1.76	3.19	3.539	3.588	3.515	3.328	3.156
3.011	3.566	3.927	3.884	3.729	3.587	3.494
2.845	3.318	3.676	3.678	3.565	3.671	3.627
2.307	3.167	3.533	3.532	3.505	3.374	3.167

ATS7e	ATS8e	ATS1p	ATS2p	ATS3p	ATS4p	ATS5p
2.891	1.538	2.69	2.893	2.92	2.831	2.649
3.43	3.159	3.034	3.206	3.204	3.197	3.174
3.519	3.437	3.197	3.503	3.57	3.519	3.491
1.408	0	2.318	2.444	2.263	1.944	1.647
2.403	1.988	2.491	2.773	2.663	2.49	2.215
0	0	2.697	2.907	2.821	2.379	1.885
3.572	3.32	3.228	3.535	3.662	3.69	3.716
0	0	2.151	2.422	2.095	1.449	0
3.115	2.277	3.136	3.47	3.587	3.537	3.486
2.649	2.537	2.81	2.952	2.887	2.839	2.794
4.552	4.515	3.449	3.715	3.776	3.719	3.762
3.904	3.672	3.533	3.874	3.984	4.148	3.906
3.043	2.729	3.13	3.454	3.573	3.485	3.376
3.905	3.436	3.209	3.502	3.693	3.761	3.845
3.414	3.336	3.191	3.559	3.497	3.338	3.023
3.299	3.288	3.172	3.533	3.469	3.322	3.001
0.693	0	2.591	2.854	2.852	2.617	2.445
4.216	4.259	3.41	3.713	3.741	3.713	3.811
2.705	2.637	2.815	2.971	2.866	2.76	2.675
3.059	2.971	3.03	3.331	3.322	2.904	2.9
2.841	2.197	2.992	3.364	3.416	3.43	3.395
3.167	2.284	2.81	3.196	3.176	2.969	2.682
2.981	2.652	3.059	3.371	3.318	3.087	2.988
3.209	3.149	3.146	3.495	3.407	3.252	2.903
3.894	3.692	3.283	3.524	3.407	3.44	3.578
3.579	3.214	3.353	3.839	4.04	3.931	3.629
3.016	2.952	3.022	3.094	2.988	2.921	2.905
4.382	4.366	3.707	4.034	4.099	4.034	4.069
3.12	2.74	3.08	3.422	3.426	3.368	3.354
3.042	3.067	2.967	3.11	3.025	2.916	2.9
4.567	4.434	3.583	3.88	3.914	3.872	3.922
4.342	4.259	3.778	4.208	4.324	4.35	4.309
2.695	2.412	2.924	3.228	3.109	3.005	2.832
3.163	2.708	3.024	3.262	3.294	3.179	3.101
4.057	3.754	3.6	4.094	4.303	4.323	4.244
3.233	3.301	3.259	3.581	3.555	3.358	3.21
3.054	2.59	3.288	3.658	3.798	3.721	3.557
0	0	2.378	2.868	2.826	2.531	1.92
3.76	3.835	3.446	3.811	3.844	3.725	3.521
3.451	3.203	3.273	3.681	3.754	3.603	3.439
3.778	3.543	3.446	3.769	3.811	3.829	3.805
1.386	0	2.942	3.237	3.398	3.317	3.112
4.326	4.191	3.729	4.236	4.445	4.414	4.291
2.608	2.449	3.015	3.37	3.36	3.143	2.908
3.469	3.382	3.356	3.627	3.631	3.5	3.341
3.259	3.317	3.182	3.447	3.512	3.352	3.106
3.369	3.342	3.191	3.46	3.516	3.343	3.111
3.936	3.92	3.402	3.672	3.755	3.644	3.535
3.849	3.81	3.35	3.646	3.615	3.594	3.478

3.954	3.77	3.41	3.743	3.718	3.724	3.73
3.934	3.838	3.321	3.553	3.583	3.589	3.462
4.559	4.558	3.693	3.994	4.132	4.037	3.967
3.896	3.737	3.301	3.553	3.592	3.592	3.513
3.877	3.576	3.275	3.556	3.577	3.571	3.444
3.483	3.416	3.27	3.521	3.601	3.514	3.354
4.14	4.021	3.558	3.833	3.925	3.849	3.788
4.313	4.167	3.543	3.822	3.805	3.784	3.836
3.512	3.458	3.169	3.413	3.39	3.348	3.203
4.176	4.199	3.511	3.785	3.87	3.855	3.735
3.873	3.691	3.199	3.453	3.519	3.526	3.416
3.763	3.447	3.235	3.511	3.494	3.389	3.366
3.249	3.294	3.172	3.431	3.489	3.327	3.09
3.72	3.572	3.381	3.65	3.593	3.548	3.5
3.622	3.424	3.254	3.502	3.46	3.422	3.305
3.249	3.294	3.172	3.431	3.489	3.327	3.09
3.155	3.152	3.24	3.458	3.449	3.329	3.344
3.541	3.314	3.251	3.523	3.557	3.51	3.606
1.572	0	2.916	3.218	3.275	3.008	2.669
2.961	2.302	2.963	3.257	3.218	3.107	3.168
3.122	2.785	3.148	3.521	3.551	3.453	3.39
2.518	2.214	2.818	3.011	2.957	2.691	2.593
3.122	2.785	3.195	3.575	3.62	3.498	3.415
3.225	2.995	3.16	3.493	3.556	3.381	3.163
2.611	2.087	2.937	3.282	3.227	2.958	2.763
2.517	2.512	2.719	2.855	2.793	2.725	2.5
3.251	3.089	3.122	3.401	3.347	3.207	3.116
3.122	3.138	3.266	3.53	3.549	3.381	3.179
2.471	1.971	2.8	3.138	3.191	3.073	2.835
3.358	2.902	3.122	3.386	3.544	3.485	3.378
3.761	3.445	3.234	3.51	3.552	3.544	3.526
2.008	1.472	2.569	2.825	2.682	2.451	2.34
3.587	3.266	3.194	3.542	3.628	3.544	3.494
1.099	0	2.655	2.925	2.788	2.682	2.451
3.26	2.816	3.157	3.508	3.584	3.451	3.549
1.262	0	2.657	2.879	2.836	2.875	2.586
2.266	1.893	2.947	3.223	3.167	2.844	2.7
2.831	2.232	3.05	3.323	3.466	3.422	3.328
3.579	3.214	3.353	3.839	4.04	3.931	3.629
2.774	1.721	3.036	3.366	3.479	3.375	3.283
2.911	2.71	2.977	3.241	3.24	3.143	3.106
3.753	3.672	3.404	3.81	3.831	3.766	3.553
2.516	1.815	2.67	2.984	2.843	2.739	2.466
2.979	2.066	2.833	3.104	3.081	2.837	2.655
3.369	3.167	3.377	3.847	3.97	3.852	3.621
4.603	4.365	3.934	4.336	4.456	4.374	4.287
3.93	3.6	3.324	3.673	3.849	3.835	3.864
2.978	2.511	2.933	3.178	3.096	3.013	3.142
3.732	3.12	3.187	3.489	3.666	3.667	3.554
5.193	5.066	3.968	4.354	4.537	4.611	4.655

3.367	3.256	3.346	3.497	3.488	3.392	3.288
3.495	3.423	3.377	3.717	3.728	3.593	3.382
3.993	3.691	3.406	3.859	4.053	4.041	3.907
3.391	3.277	2.894	3.154	3.246	3.153	3.017
2.267	2.333	2.434	2.456	2.495	2.309	2.003
2.812	2.395	3.048	3.382	3.506	3.474	3.227
4.751	4.649	3.611	4.017	4.124	4.128	4.173
2.87	2.8	3.003	3.084	3.006	3.028	2.791
2.77	2.51	2.963	3.179	3.147	2.959	2.82
2.614	2.51	2.88	3.088	3.027	2.844	2.817
3.134	2.88	3.227	3.458	3.489	3.437	3.464
3.385	3.02	3.207	3.51	3.469	3.413	3.361
3.336	3.19	3.347	3.803	3.892	3.781	3.577
3.911	3.884	3.076	3.262	3.235	3.188	3.224
3.495	2.789	2.897	3.227	3.094	3.116	3.143
2.623	1.776	2.956	3.275	3.23	3.004	2.775
3.002	2.521	2.952	3.308	3.447	3.372	3.209
0	0	1.906	2.026	1.725	0.817	0.135
3.776	3.75	3.379	3.718	3.806	3.671	3.57
2.366	2.426	2.903	3.168	3.169	2.949	2.714
0	0	1.915	1.622	0.806	0	0
4.051	3.821	3.727	4.019	4.08	4	3.952
3.15	2.843	3.02	3.275	3.277	3.135	2.986
4.411	4.043	3.616	4.06	4.268	4.29	4.162
0	0	2.445	2.691	2.624	2.262	1.612
2.693	1.538	3.052	3.493	3.642	3.52	3.271
2.86	2.61	2.604	2.886	2.801	2.592	2.438
2.685	2.851	2.822	3.113	2.973	2.658	2.507
3.985	3.917	3.248	3.562	3.566	3.5	3.412
3.755	3.685	3.482	3.762	3.745	3.518	3.41
3.693	3.661	3.477	3.734	3.725	3.541	3.463
3.25	3.149	3.129	3.481	3.521	3.214	2.939
3.869	3.895	3.185	3.405	3.392	3.263	3.323
1.572	0	2.916	3.218	3.275	3.008	2.669
1.582	0.845	3.122	3.598	3.747	3.61	3.169
2.035	2.035	2.702	2.951	2.88	2.696	2.381
3.898	3.864	3.568	3.877	3.863	3.694	3.767
3.583	3.258	3.194	3.537	3.617	3.532	3.502
2.857	2.831	3.056	3.379	3.372	3.157	2.934
3.666	3.453	3.466	3.788	3.772	3.771	3.784
2.24	1.968	2.345	2.642	2.413	2.286	1.971
1.509	0	2.26	2.549	2.198	1.921	1.433
3.954	3.118	3.169	3.51	3.649	3.704	3.724
2.903	2.788	3.073	3.35	3.364	3.196	3.057
2.844	2.51	2.963	3.22	3.204	3.027	2.954
2.201	1.249	2.629	2.865	2.709	2.564	2.386
3.452	3.232	3.164	3.401	3.394	3.322	3.2
3.12	2.994	3.08	3.373	3.363	3.323	3.35
0	0	2.343	2.534	2.463	2.411	1.809
3.548	2.964	3.145	3.552	3.677	3.592	3.502

2.65	2.398	2.746	2.97	3.027	2.852	2.582
3.222	3.199	2.998	3.281	3.311	3.221	3.093
3.444	2.956	3.091	3.44	3.563	3.515	3.358
3.072	1.524	3.086	3.407	3.516	3.408	3.407
3.533	3.303	3.376	3.671	3.725	3.704	3.714
3.291	3.005	3.183	3.549	3.511	3.354	3.146
2.541	2.56	2.884	3.061	3.087	3.124	3.05
2.232	1.386	2.847	3.171	3.251	3.106	3.027
2.215	0.693	3.045	3.414	3.545	3.462	3.393
2.843	2.079	2.867	3.157	3.15	2.912	2.718
0	0	2.267	2.563	2.419	1.994	0
0	0	1.792	1.994	1.792	1.518	1.253
3.06	1.792	3.15	3.471	3.623	3.693	3.8
3.617	3.502	3.18	3.577	3.583	3.494	3.183
3.815	3.622	3.32	3.592	3.63	3.538	3.458
3.459	3.147	3.342	3.819	3.995	3.911	3.621
2.887	2.81	2.854	3.114	3.214	2.987	2.768
2.668	2.649	2.805	2.976	2.887	2.744	2.725
0	0	2.172	2.407	2.4	2.1	1.457
3.34	2.544	3.175	3.519	3.639	3.593	3.548
4.204	4.286	3.81	4.101	4.154	4.059	4.038
1.582	0.845	3.122	3.598	3.747	3.61	3.169
3.853	3.407	3.317	3.639	3.76	3.761	3.74
3.078	2.63	2.842	3.136	3.156	3.099	3.079
3.002	3.007	3.022	3.317	3.217	3.062	2.987
3.763	3.716	3.372	3.745	3.775	3.697	3.458
1.538	1.296	2.74	3.052	3.12	2.966	2.789
2.825	2.564	3.321	3.728	3.902	3.884	3.605
2.456	2.035	3.207	3.653	3.838	3.763	3.386
2.364	1.989	2.822	3.137	3.167	2.875	2.619
2.548	1.946	2.545	2.992	2.837	2.676	2.576
3.664	3.588	3.434	3.879	3.946	3.917	3.73
3.819	2.231	3.053	3.404	3.587	3.633	3.638
3.413	3.279	2.991	3.152	3.119	3.08	3.117
3.812	3.54	3.099	3.419	3.597	3.631	3.633
2.801	2.566	2.528	2.762	2.489	2.32	2.196
0.932	0	2.397	2.592	2.472	2.018	1.573
3.23	2.786	3.029	3.364	3.485	3.389	3.231
2.831	2.232	3.102	3.389	3.545	3.454	3.341
4.293	4.33	3.641	3.977	4.053	4.027	4.003
3.548	2.964	3.145	3.552	3.677	3.592	3.502
3.316	3.189	3.162	3.472	3.507	3.417	3.135
2.78	2.707	3.066	3.456	3.44	3.334	3.153
3.692	3.535	3.318	3.694	3.655	3.56	3.362
2.835	1.524	3.028	3.322	3.399	3.294	3.276
2.444	1.249	3.161	3.66	3.849	3.735	3.329
3.381	3.032	3.146	3.389	3.416	3.444	3.536
4.399	4.176	3.707	4.175	4.343	4.335	4.374
1.202	0.845	2.629	2.928	3.104	2.897	2.404
1.832	1.465	2.452	2.563	2.535	2.397	2.103

1.946	0.693	2.989	3.263	3.337	3.328	3.282
1.538	0.845	2.764	3.077	3.19	3.002	2.655
3.164	2.708	3.127	3.358	3.386	3.429	3.528
2.035	0.693	2.966	3.266	3.307	3.293	3.194
2.762	2.457	2.825	3.046	2.901	2.773	2.604
3.875	3.844	3.524	3.891	3.944	3.826	3.765
3.506	3.288	3.179	3.515	3.637	3.546	3.548
3.7	2.951	3.214	3.467	3.443	3.504	3.657
2.31	2.333	2.835	3.14	3.105	3.063	2.797
2.701	2.652	2.792	2.932	2.844	2.784	2.714
3.885	3.662	3.319	3.58	3.645	3.601	3.606
3.446	3.367	3.18	3.53	3.574	3.448	3.392
3.383	3.042	3.306	3.774	3.938	3.805	3.494
2.961	2.75	3.255	3.709	3.846	3.682	3.306
2.649	2.427	2.722	2.897	2.904	2.574	2.385
2.649	2.427	2.699	2.873	2.88	2.52	2.337
3.482	3.29	3.181	3.347	3.293	3.206	3.207
2.801	2.625	2.924	3.142	3.088	2.921	2.794
0	0	2.599	2.885	2.88	2.658	2.197
2.801	2.625	2.924	3.142	3.088	2.921	2.794
2.397	1.546	2.833	3.115	3.167	3.009	2.803
3.408	3.102	3.32	3.637	3.779	3.616	3.674
3.373	3.066	3.198	3.561	3.619	3.426	3.325
3.373	3.066	3.198	3.561	3.619	3.426	3.325
2.841	2.843	2.878	3.079	2.959	2.955	2.916
3.856	3.844	3.447	3.704	3.763	3.792	3.848
2.45	1.802	2.641	2.925	2.751	2.454	2.021
2.193	0	2.915	3.175	3.064	2.855	2.701
3.506	3.345	3.372	3.704	3.736	3.55	3.421
2.977	2.73	2.925	3.288	3.135	3.027	2.997
4.249	4.044	3.64	4.085	4.251	4.274	4.262
3.004	2.639	2.974	3.223	3.257	3.153	3.007
0	0	2.237	2.4	2.217	1.545	0.647
3.194	3.099	3.073	3.363	3.364	2.952	2.911
4.093	4.049	3.415	3.77	3.868	3.8	3.775
2.828	2.364	2.862	2.995	2.959	2.889	2.812
3.357	3.059	3.311	3.604	3.599	3.664	3.74
2.396	2.058	2.821	3.051	2.93	2.672	2.583
3.137	2.908	3.122	3.364	3.308	3.215	3.178
3.727	3.599	3.429	3.678	3.685	3.667	3.733
2.55	2.182	2.879	3.144	3.049	2.777	2.717
3.405	3.226	3.306	3.61	3.657	3.527	3.456
3.304	3.155	3.076	3.332	3.345	3.195	3.006
3.111	2.582	2.992	3.3	3.228	3.085	3.027
2.517	2.59	2.979	3.257	3.117	2.992	2.807
3.45	3.29	3.264	3.469	3.525	3.475	3.297
3.076	2.862	3.007	3.219	3.149	2.954	2.783
2.946	1.721	3.057	3.391	3.508	3.435	3.349
2.966	2.911	2.835	2.984	2.912	2.908	2.606
3.186	2.954	3.141	3.485	3.492	3.284	3.023

3.446	3.367	3.18	3.53	3.574	3.448	3.392
2.366	2.482	2.682	2.956	2.75	2.537	2.433
2.75	2.231	3.174	3.633	3.731	3.491	3.147
4.014	3.723	3.406	3.869	4.053	4.024	3.884
0	0	2.318	2.747	2.657	2.385	1.34
1.124	0.731	2.712	3.005	3.206	3.015	2.599
2.616	2.28	3.016	3.343	3.199	2.828	2.818
2.895	2.954	2.874	3.166	2.969	3.039	2.991
3.115	3.05	3.118	3.305	3.23	3.013	2.873
3.009	2.994	3.021	3.232	3.267	2.846	2.833
2.582	2.497	2.862	3.037	2.983	2.717	2.637
2.567	2.056	2.904	3.254	3.275	3.196	3.151
3.242	3.187	3.202	3.514	3.508	3.287	3.142
3.337	2.524	3.188	3.544	3.685	3.628	3.594
3.246	3.023	2.861	3.185	3.249	3.117	3.028
3.243	3.273	3.16	3.493	3.563	3.394	3.215
0	0	2.187	2.21	2.177	2.056	1.572
3.727	3.489	3.39	3.664	3.731	3.797	3.79
4.362	4.144	3.691	4.128	4.29	4.289	4.323
3.807	3.541	3.372	3.726	3.875	3.796	3.657
5.15	5.017	4.013	4.499	4.728	4.914	4.935
3.438	2.65	3.3	3.709	3.898	3.921	3.789
3.217	2.685	3.147	3.429	3.48	3.444	3.443
3.245	3.105	3.067	3.363	3.467	3.451	3.27
2.943	2.556	2.695	2.993	2.949	2.831	2.689
3.341	3.221	3.401	3.771	3.737	3.555	3.421
3.438	2.917	3.111	3.47	3.454	3.35	3.39
2.885	2.665	2.969	3.296	3.292	3.122	3.05

ATS6p	ATS7p	ATS8p	MATS1m	MATS2m	MATS3m	MATS4m
2.356	1.88	1.068	-0.083	0.096	-0.199	-0.027
3.017	2.936	2.822	-0.121	-0.17	0.007	-0.016
3.356	3.154	2.892	0.126	0.105	-0.192	-0.08
1.068	0.507	0	-0.068	-0.066	-0.203	-0.313
1.936	1.745	1.285	-0.434	0.097	-0.119	0.094
0.995	0	0	-0.258	0.175	-0.167	-0.127
3.528	3.215	2.994	-0.105	0.126	0.002	-0.2
0	0	0	-0.215	0.061	-0.697	0.689
3.28	2.912	2.094	-0.007	-0.033	-0.021	-0.028
2.528	2.378	2.371	-0.11	-0.206	0.358	0.052
3.867	3.848	3.716	-0.167	-0.244	0.099	-0.091
3.945	4.175	3.99	0.001	-0.033	-0.046	0.222
3.114	2.876	2.657	-0.087	0.016	0.02	0.017
3.77	3.461	3.075	-0.036	-0.035	-0.078	-0.085
2.982	3.019	2.95	-0.093	-0.139	-0.047	0.087
2.944	2.995	2.926	-0.09	-0.127	-0.038	0.097
1.865	0.693	0	-0.012	-0.04	0.029	-0.331
3.777	3.696	3.652	-0.064	0.36	-0.129	-0.197
2.361	2.322	2.412	-0.119	-0.13	0.218	-0.089
2.895	2.869	2.737	-0.095	0.096	-0.171	0.193
3.118	2.624	2.197	-0.129	0.155	-0.12	-0.002
2.685	2.37	1.114	-0.143	-0.066	-0.274	0.084
2.875	2.695	2.517	-0.007	-0.031	-0.065	-0.035
2.85	2.93	2.82	-0.089	-0.127	-0.04	0.104
3.755	3.76	3.611	-0.146	-0.131	0.203	0.078
3.337	3.09	2.724	0.024	-0.049	0.018	-0.15
2.771	2.828	2.772	-0.132	-0.218	0.176	-0.001
4.193	4.143	4.063	-0.046	0.186	-0.083	-0.015
3.248	3.02	2.63	0.077	-0.158	0.099	0.025
2.784	2.742	2.859	-0.203	-0.287	0.356	-0.099
4.036	4.076	4.046	0.193	-0.084	-0.048	-0.064
4.233	4.024	3.935	-0.001	0.008	-0.027	-0.027
2.637	2.512	2.363	-0.089	-0.188	0.292	-0.009
3.093	3.078	2.708	-0.098	0.106	0.14	-0.291
4.133	3.859	3.505	-0.068	-0.094	0.036	-0.074
2.972	2.921	3.048	-0.203	0.199	-0.178	-0.161
3.259	2.901	2.522	-0.026	-0.079	-0.025	-0.129
0.375	0	0	-0.463	0.323	-0.375	0.176
3.606	3.63	3.626	0.007	-0.051	-0.025	0.187
3.302	3.108	2.87	-0.082	0.07	0.002	-0.057
3.673	3.491	3.186	0.017	0.32	-0.169	-0.097
2.318	1.386	0	-0.061	0.468	-0.111	-0.18
4.198	4.021	3.818	-0.008	0.231	-0.025	-0.043
2.636	2.308	2.086	0.005	-0.015	-0.033	-0.063
3.182	3.029	2.991	-0.145	0.01	0.212	-0.233
2.932	2.984	2.935	-0.061	-0.078	0.211	-0.073
2.963	2.996	2.941	-0.093	-0.039	-0.102	-0.09
3.467	3.486	3.36	-0.126	-0.042	-0.16	0.202
3.411	3.415	3.229	-0.187	0.1	-0.183	0.073

3.667	3.522	3.325	-0.137	-0.031	-0.074	-0.043
3.365	3.402	3.378	-0.152	-0.076	-0.088	-0.103
4.012	4.075	4.015	-0.117	-0.02	-0.108	0.191
3.443	3.376	3.242	-0.166	-0.076	-0.102	-0.045
3.378	3.387	3.095	-0.164	-0.132	-0.071	-0.038
3.166	3.095	3.023	-0.086	-0.032	-0.086	-0.118
3.758	3.708	3.594	0.065	-0.041	-0.077	-0.116
3.874	3.893	3.72	-0.125	-0.043	-0.083	-0.038
3.106	3.104	3.041	-0.167	-0.088	-0.067	-0.087
3.676	3.722	3.683	-0.176	-0.077	-0.168	0.091
3.313	3.225	3.076	-0.147	-0.064	-0.144	-0.002
3.322	3.214	2.968	0.143	0.065	-0.049	-0.005
2.924	2.972	2.917	-0.09	-0.029	-0.091	-0.083
3.478	3.448	3.187	-0.117	-0.101	-0.079	0.006
3.237	3.219	2.982	-0.149	-0.14	-0.109	0.006
2.924	2.972	2.917	-0.09	-0.029	-0.091	-0.083
3.216	3.03	2.986	-0.074	-0.027	-0.002	-0.108
3.508	3.309	3.023	-0.023	-0.035	0.009	0.001
2.062	0.93	0	-0.189	-0.164	0.008	0.005
3.129	2.862	2.225	-0.012	-0.035	-0.04	-0.001
3.213	3.008	2.745	-0.057	-0.116	-0.116	-0.05
2.452	2.222	1.993	-0.03	-0.193	-0.195	-0.148
3.213	3.008	2.745	-0.046	-0.099	-0.125	-0.078
3.074	2.88	2.347	0.041	-0.041	0.166	-0.029
2.562	2.42	1.867	-0.007	-0.042	-0.059	-0.016
2.381	2.145	2.114	-0.155	-0.095	-0.028	-0.26
3	2.946	2.792	-0.191	0.167	-0.061	-0.198
2.935	2.902	2.923	-0.001	0.594	-0.061	-0.103
2.448	1.655	0.842	-0.165	0.006	-0.092	0.202
3.107	2.856	2.514	-0.077	-0.05	-0.062	0.096
3.512	3.38	3.101	-0.076	-0.027	-0.021	-0.047
1.954	1.727	0.754	-0.02	-0.09	-0.111	-0.029
3.369	3.153	2.867	-0.013	-0.028	-0.017	-0.003
2.416	1.099	0	-0.009	-0.102	-0.231	0.198
3.422	3.129	2.671	-0.018	-0.035	-0.031	0.013
2.25	1.246	0	-0.136	-0.24	-0.089	0.416
2.432	2.015	1.504	0.124	-0.177	-0.23	-0.161
3.02	2.621	2.11	-0.141	-0.119	-0.15	0.326
3.337	3.09	2.724	0.024	-0.049	0.018	-0.15
3.127	2.532	1.546	-0.008	-0.029	-0.024	-0.042
3.102	2.693	1.956	0.003	-0.069	-0.11	0.228
3.492	3.375	3.398	-0.044	-0.119	-0.122	0.103
2.275	1.822	1.095	-0.304	-0.008	-0.067	0.056
2.398	1.917	1.099	0.018	-0.017	-0.193	0.127
3.374	3.063	2.829	-0.015	0.099	-0.049	-0.033
4.258	4.111	3.911	-0.115	-0.004	0.05	0.079
3.797	3.694	3.212	-0.103	-0.123	-0.042	-0.111
3.068	2.862	2.42	-0.127	-0.081	0.151	-0.27
3.606	3.51	3.02	-0.09	0.097	-0.071	-0.222
4.722	4.708	4.627	-0.092	0.049	-0.025	-0.215

3.127	3.026	2.947	0.135	-0.001	-0.093	-0.148
3.2	3.237	3.193	-0.023	-0.016	-0.052	0.001
3.674	3.433	3.109	-0.007	-0.031	-0.114	0.011
2.844	2.726	2.441	0.107	0.025	-0.15	0.039
1.92	2.068	1.92	-0.151	-0.231	0.221	-0.327
2.949	2.507	1.745	-0.067	0.064	-0.006	0.201
4.175	4.137	3.96	-0.216	-0.01	0.114	-0.02
2.655	2.529	2.433	-0.211	-0.132	-0.295	0.112
2.586	2.373	2.177	0.009	0.203	-0.05	-0.095
2.662	2.477	2.177	-0.053	0.104	-0.2	-0.22
3.291	2.993	2.73	-0.102	0.109	-0.15	-0.028
3.234	3.142	2.722	-0.045	-0.09	-0.14	-0.064
3.536	3.088	2.878	-0.031	0.168	-0.055	-0.179
3.174	3.081	2.983	-0.086	0.374	0.333	-0.059
3.079	2.86	2.07	-0.095	-0.126	-0.189	0.113
2.529	2.07	1.291	0.018	-0.044	-0.153	-0.061
2.742	2.339	2.161	-0.152	0.003	-0.089	-0.123
0	0	0	-0.173	-0.175	-0.052	-0.504
3.459	3.375	3.38	-0.085	-0.007	-0.111	-0.111
2.353	2.187	2.056	0.01	0.091	-0.118	-0.236
0	0	0	-0.629	-0.021	0.077	0
3.846	3.71	3.539	0.036	0.1	0.168	-0.005
2.87	2.692	2.418	-0.002	-0.085	0.177	-0.105
4.096	3.948	3.725	-0.015	0.162	-0.021	-0.119
0.647	0	0	0	0.328	-0.036	-0.533
2.778	2.071	1.068	-0.137	-0.101	0.025	-0.011
2.312	2.169	1.829	-0.371	0.011	-0.07	0.104
2.435	2.558	2.626	-0.067	0.152	-0.3	-0.175
3.522	3.416	3.263	-0.21	-0.18	0.092	-0.135
3.429	3.428	3.441	0.231	-0.03	-0.012	-0.087
3.466	3.425	3.408	0.064	-0.08	-0.064	-0.104
2.965	3.017	2.952	-0.05	-0.035	0.064	0.013
3.411	3.386	3.262	-0.027	0.015	-0.097	-0.11
2.062	0.93	0	-0.189	-0.164	0.008	0.005
2.546	1.007	0.375	-0.07	-0.085	0.152	-0.199
1.933	1.776	1.776	0	0.356	-0.089	-0.135
3.747	3.675	3.61	-0.2	0.149	-0.169	-0.185
3.366	3.169	2.893	-0.013	-0.023	-0.035	-0.021
2.748	2.641	2.462	-0.038	0.239	-0.167	-0.227
3.676	3.482	3.275	0.167	0.25	-0.103	-0.24
1.649	0.796	0.759	-0.015	0.127	0.018	-0.655
1.132	0.346	0	-0.077	-0.054	0.135	-0.632
3.66	3.307	2.723	0.007	0.091	-0.155	-0.135
2.804	2.562	2.453	0.005	-0.024	-0.098	-0.128
2.755	2.511	2.177	0.009	0.202	-0.152	-0.208
2.021	1.526	0.732	-0.179	-0.048	0.095	-0.188
3.052	2.93	2.859	-0.04	0.077	-0.053	-0.315
3.146	2.864	2.623	0.046	0.205	-0.151	-0.272
0	0	0	-0.143	0.054	-0.085	-0.367
3.238	2.938	2.563	-0.137	-0.061	-0.047	0.073

2.394	2.612	2.398	-0.14	0.096	0.247	-0.288
2.781	2.68	2.738	-0.193	-0.013	0.001	-0.168
3.115	2.832	2.592	-0.076	-0.071	-0.052	0.015
3.275	2.703	1.306	0.004	-0.05	-0.07	-0.077
3.589	3.285	3.052	-0.002	0.03	-0.007	0.032
3.095	3.021	2.801	-0.03	-0.174	-0.136	0.002
2.744	2.241	2.23	-0.013	0.012	0.089	-0.098
2.687	2.11	1.386	-0.129	0.243	-0.157	-0.304
2.931	2.155	0.693	-0.233	0.058	0.011	-0.193
2.77	2.811	2.079	-0.115	0.114	0.131	-0.36
0	0	0	-0.039	-0.019	-0.202	-0.266
0	0	0	-1	0.98	-1	0.95
3.609	3.008	1.792	-0.051	-0.033	-0.065	-0.112
3.125	3.187	3.106	-0.125	-0.144	-0.062	0.111
3.401	3.325	3.197	-0.168	0.166	-0.033	-0.155
3.374	2.994	2.677	0.023	-0.018	0.105	-0.056
2.692	2.588	2.53	-0.036	-0.071	-0.066	-0.089
2.483	2.39	2.397	-0.177	-0.191	0.257	-0.035
0.346	0	0	0.03	-0.001	0.189	-0.496
3.362	3.042	2.198	-0.008	-0.038	-0.033	-0.025
4.072	4.145	4.169	-0.023	-0.045	-0.105	-0.053
2.546	1.007	0.375	-0.07	-0.085	0.152	-0.199
3.565	3.318	3.069	-0.092	-0.045	-0.044	0.078
2.928	2.676	2.463	-0.199	0.046	-0.045	-0.284
2.677	2.65	2.67	-0.076	-0.174	0.199	-0.142
3.416	3.487	3.438	-0.083	-0.096	-0.02	0.126
2.116	1.068	0.647	-0.11	0.086	-0.279	0.488
3.279	2.69	2.338	-0.055	-0.119	-0.001	-0.042
2.925	2.293	1.776	-0.066	-0.126	0.017	-0.087
2.329	1.836	1.34	-0.056	0.198	-0.066	-0.15
2.278	2.013	1.946	-0.339	0.47	-0.336	-0.013
3.503	3.376	3.297	-0.004	-0.011	0.004	-0.013
3.432	2.92	1.761	0.021	0.253	-0.151	-0.138
3.05	2.918	2.455	0.176	-0.008	-0.125	-0.072
3.48	3.164	2.58	0.027	0.214	-0.148	-0.109
2.245	2.033	1.615	-0.069	-0.028	0.038	-0.148
0.944	0.25	0	0	-0.18	-0.019	-0.484
2.964	2.712	2.337	-0.096	-0.036	-0.073	0.075
3.02	2.621	2.11	-0.043	0.016	0.022	0.019
3.955	3.896	3.807	-0.145	0.033	0.003	-0.045
3.238	2.938	2.563	-0.137	-0.061	-0.047	0.073
3.03	2.959	2.866	-0.002	0.014	-0.104	-0.232
2.838	2.516	2.476	-0.154	-0.011	-0.164	0.2
3.322	3.287	3.216	-0.079	-0.117	-0.041	0.113
3.063	2.45	1.306	-0.001	-0.032	-0.02	-0.061
2.857	2.034	0.732	-0.11	-0.11	0.069	-0.164
3.422	3.156	2.808	-0.009	0	-0.127	-0.091
4.374	4.197	3.983	-0.123	0.164	0.068	-0.112
1.875	0.898	0.375	-0.1	0.331	-0.317	0.222
1.512	1.19	1.24	-0.163	-0.117	-0.132	-0.14

2.833	1.946	0.693	-0.1	-0.137	-0.124	-0.055
2.018	1.068	0.375	-0.067	0.342	-0.288	0.26
3.397	3.087	2.708	0.01	0.088	-0.058	-0.052
2.707	1.776	0.693	-0.048	0.362	-0.059	-0.021
2.411	2.37	2.266	-0.145	-0.23	0.25	0.132
3.676	3.7	3.661	0.051	-0.156	-0.103	-0.046
3.335	3.272	3.065	-0.219	0.058	0.031	-0.248
3.627	3.263	2.592	0.194	-0.039	-0.026	-0.116
2.114	2.024	1.92	-0.007	-0.035	-0.057	-0.2
2.361	2.302	2.397	-0.159	-0.193	0.182	-0.049
3.596	3.527	3.348	-0.032	0.079	-0.149	0.142
3.216	2.957	2.893	-0.298	-0.052	0.065	-0.295
3.209	2.918	2.572	0.025	-0.02	0.1	-0.078
3.083	2.761	2.422	0.011	-0.009	0.004	-0.061
2.383	2.378	2.327	-0.16	0.042	-0.104	-0.413
2.373	2.378	2.327	-0.179	0.135	-0.069	-0.493
3.192	3.147	3.086	-0.066	-0.14	0.147	0.187
2.528	2.505	2.427	-0.089	-0.154	0.348	0.004
1.609	0	0	0	-0.037	-0.145	-0.255
2.528	2.505	2.427	-0.089	-0.154	0.348	0.004
2.451	2.056	1.412	-0.007	-0.04	-0.045	-0.007
3.555	3.184	2.821	-0.085	-0.128	-0.089	-0.03
3.255	3.142	2.847	-0.111	-0.095	-0.008	-0.235
3.255	3.142	2.847	-0.111	-0.095	-0.008	-0.235
2.65	2.593	2.411	-0.135	-0.103	0.09	-0.212
3.775	3.641	3.559	-0.084	0.114	-0.145	0.1
1.693	1.156	0.468	-0.169	-0.002	-0.12	-0.143
2.304	1.316	0	-0.014	0.259	-0.026	-0.25
3.301	3.162	3.076	-0.021	-0.064	-0.164	0.044
2.87	2.667	2.423	-0.152	0.156	-0.093	-0.188
4.201	4.026	3.821	-0.116	0.037	0.128	-0.081
2.946	2.977	2.639	-0.103	0.099	0.129	-0.274
0	0	0	0	0.136	-0.405	0.012
2.92	2.906	2.794	-0.129	0.033	-0.131	0.012
3.751	3.7	3.598	0.198	-0.131	-0.147	-0.062
2.655	2.345	1.959	0.186	-0.008	-0.098	-0.151
3.568	3.199	2.936	-0.081	-0.08	-0.009	0.036
2.4	2.099	1.699	0.152	-0.162	-0.245	-0.131
3.054	2.888	2.678	0.229	-0.099	-0.146	-0.162
3.668	3.548	3.392	0.063	-0.08	-0.105	-0.11
2.567	2.335	1.877	0.17	-0.135	-0.151	-0.288
3.288	3.099	2.993	0.047	-0.016	-0.017	-0.001
2.981	2.953	2.881	0.18	-0.05	-0.091	-0.109
2.939	2.634	2.321	0.213	-0.024	-0.065	-0.099
2.486	2.248	2.264	-0.078	-0.091	0.057	-0.122
3.104	3.02	2.981	0.198	-0.071	0.013	-0.122
2.687	2.617	2.568	-0.024	0.131	-0.066	-0.268
3.204	2.609	1.546	-0.008	-0.028	-0.017	-0.052
2.517	2.5	2.245	0.183	0.141	0.037	-0.15
2.895	2.924	2.739	-0.043	-0.213	0.149	0.041

3.216	2.957	2.893	-0.298	-0.052	0.065	-0.295
2.322	2.187	1.906	-0.044	-0.182	-0.115	-0.142
2.88	2.422	1.761	0.013	-0.008	-0.045	-0.025
3.675	3.453	3.129	-0.007	-0.05	-0.114	-0.02
0	0	0	-0.436	0.299	-0.461	0.334
2.313	1.294	0.974	-0.021	0.088	-0.076	0.081
2.78	2.693	2.449	-0.054	-0.091	-0.135	0.003
2.627	2.549	2.594	0.028	-0.104	0.18	-0.215
2.802	2.753	2.718	-0.126	-0.125	0.085	-0.143
2.786	2.737	2.676	0.204	-0.107	-0.01	-0.07
2.512	2.364	2.196	0.186	-0.133	-0.065	-0.16
2.837	2.338	1.711	-0.122	-0.084	-0.176	-0.024
3.003	2.915	2.906	-0.026	-0.036	-0.048	-0.002
3.436	3.143	2.315	-0.002	-0.044	-0.053	-0.038
2.75	2.62	2.376	-0.305	-0.009	0.094	-0.092
2.994	2.877	2.991	-0.296	0.137	-0.187	0.354
0.693	0	0	-0.028	0.375	-0.25	-0.306
3.677	3.44	3.205	0.084	0.271	0.025	-0.274
4.317	4.151	3.945	-0.111	0.161	0.065	-0.106
3.523	3.528	3.318	-0.191	-0.074	0.171	-0.157
4.925	4.834	4.71	-0.13	0.117	0.023	0.077
3.537	3.233	2.612	-0.118	0.12	0.157	-0.133
3.278	2.982	2.558	-0.032	0.064	-0.172	0.04
3.088	2.877	2.745	-0.086	-0.015	-0.138	0.031
2.474	2.158	1.648	-0.182	0.069	-0.311	0.192
3.292	3.166	3.018	-0.008	-0.063	-0.077	-0.112
3.395	3.217	2.838	-0.155	0.156	-0.192	0.179
2.874	2.512	2.377	-0.007	-0.033	-0.043	-0.026

MATS5m	MATS6m	MATS7m	MATS8m	MATS1v	MATS2v	MATS3v
0.165	-0.528	0.369	-0.528	-0.083	0.096	-0.199
-0.094	-0.338	0.324	0.106	-0.135	-0.174	0.004
-0.268	-0.009	-0.206	-0.042	0.159	0.098	-0.193
0.017	-0.653	1.377	0	-0.085	-0.057	-0.22
-0.242	0.293	-0.099	-1.064	-0.517	0.158	-0.178
-0.163	0.068	0	0	-0.346	0.145	-0.257
0.182	-0.207	-0.208	0.042	-0.088	0.138	0.017
0	0	0	0	-0.246	0.069	-0.722
0.035	-0.207	-0.122	-0.136	0.051	0.086	0.1
-0.267	-0.343	-0.201	-0.057	-0.118	-0.218	0.371
0.082	0.026	-0.084	0.006	-0.173	-0.255	0.091
-0.057	-0.069	-0.201	-0.246	-0.008	-0.007	-0.193
-0.022	-0.133	-0.282	-0.1	-0.087	0.016	0.02
-0.114	0.131	-0.014	-0.116	-0.167	0.129	-0.001
0.019	-0.03	-0.139	-0.129	-0.065	0.103	0.096
0.03	-0.022	-0.162	-0.12	-0.069	0.158	0.167
-0.108	-0.039	0.236	0	0.01	-0.074	0.028
-0.269	0.585	0.005	0.153	-0.108	0.293	-0.096
-0.436	-0.49	0.521	-0.06	-0.128	-0.123	0.221
-0.38	-0.139	-0.058	0.103	-0.109	0.074	-0.168
-0.143	-0.188	-0.091	0.32	-0.146	0.187	-0.117
0.032	0.047	-0.011	-0.08	-0.103	0.178	0.023
-0.038	0.148	-0.107	-0.195	-0.073	0.183	-0.204
0.035	-0.04	-0.167	-0.129	-0.069	0.187	0.133
-0.186	-0.1	-0.099	-0.027	-0.162	-0.149	0.25
-0.227	0.158	0.182	0.018	0.026	-0.043	0.044
-0.295	-0.272	0.372	-0.061	-0.139	-0.229	0.193
-0.16	0.034	-0.098	0.072	-0.06	0.166	-0.101
-0.178	-0.099	-0.063	-0.09	0.077	-0.158	0.099
-0.348	0.001	-0.184	-0.055	-0.211	-0.298	0.365
-0.018	-0.137	-0.145	-0.03	-0.25	0.102	0.208
-0.029	-0.032	-0.002	-0.001	-0.085	0.143	-0.034
-0.284	-0.365	-0.079	0.106	-0.096	-0.201	0.304
-0.324	-0.141	0.007	0.146	-0.117	0.098	0.167
-0.007	0.024	-0.016	0.051	-0.075	-0.099	0.024
-0.059	-0.217	0.223	-0.08	-0.244	0.231	-0.174
-0.143	0.135	0.217	0.026	-0.036	-0.086	-0.014
0.01	-1.538	0	0	-0.575	0.381	-0.369
-0.016	-0.006	-0.056	-0.153	0.007	-0.051	-0.025
0.069	0.046	-0.005	-0.266	-0.097	0.057	-0.013
-0.174	0.188	-0.199	-0.325	0.054	0.341	-0.188
-0.348	-0.253	0.174	0	-0.075	0.516	-0.131
0.169	-0.123	-0.103	-0.054	-0.008	0.231	-0.025
-0.097	-0.133	-0.116	-0.166	-0.021	0.21	-0.093
-0.134	0.318	-0.253	-0.264	-0.153	-0.01	0.239
0.036	-0.015	-0.124	-0.087	-0.069	0.132	0.174
0.174	-0.039	-0.135	-0.139	-0.065	0.073	0.103
0.014	0.026	-0.106	-0.094	0.024	0.094	0.134
-0.109	0.215	-0.114	-0.087	0.029	0.053	0.192

0.033	-0.14	-0.054	0.339	-0.114	0.027	0.099
0.101	-0.111	-0.111	0.341	-0.062	0.011	0.185
0.075	-0.028	-0.076	0.011	-0.067	0.092	0.066
0.056	-0.114	-0.069	0.313	-0.115	0.011	0.02
-0.025	-0.036	0.274	-0.213	-0.146	0.245	0.061
0.113	-0.038	-0.131	-0.127	-0.061	0.081	0.123
-0.093	0.222	-0.046	-0.189	-0.134	0.251	-0.061
0.086	-0.125	-0.142	0.328	-0.078	-0.008	0.062
0.085	-0.128	-0.106	0.339	-0.071	-0.012	0.06
0.024	-0.07	-0.103	0.124	-0.087	-0.01	0.182
0.023	0.019	0.123	-0.28	-0.16	0.086	0.026
0.036	-0.123	-0.308	-0.366	-0.05	0.359	0.019
0.192	-0.031	-0.158	-0.129	-0.069	0.132	0.174
-0.013	-0.152	0.327	-0.183	-0.093	0.149	0.11
-0.023	-0.124	0.383	-0.237	-0.097	0.158	0.045
0.192	-0.031	-0.158	-0.129	-0.069	0.132	0.174
0.108	-0.16	-0.106	0.08	-0.081	0.227	0.063
0.013	0.052	-0.192	-0.282	-0.15	0.07	0.567
0.065	-0.136	0.146	0	-0.246	0.393	-0.233
0.003	-0.058	-0.06	-0.188	-0.162	-0.054	-0.061
0.304	-0.102	-0.104	-0.162	-0.136	-0.062	-0.171
0.423	-0.024	-0.018	0.125	-0.227	0.579	-0.414
0.309	-0.103	-0.102	-0.156	-0.048	0.026	0.032
-0.025	-0.139	-0.313	-0.268	-0.097	0.304	-0.208
-0.029	0.062	-0.348	-0.175	-0.087	-0.119	-0.061
0.087	-0.249	0.054	0.062	-0.38	0.492	-0.442
0.056	-0.101	0.415	-0.166	-0.202	0.179	-0.082
-0.16	-0.071	-0.064	0.336	-0.011	0.546	-0.074
-0.184	-0.46	0.179	0.663	-0.162	-0.039	-0.086
-0.047	-0.315	0.282	0.1	-0.13	-0.079	-0.063
-0.031	0.213	-0.121	-0.056	-0.12	-0.172	0.252
0.139	-0.167	-0.916	0.842	-0.091	-0.003	0.226
-0.039	0.034	-0.033	-0.246	-0.091	0.05	0.106
-0.049	-0.348	0.219	0	-0.236	0.527	-0.326
0.034	-0.016	-0.187	-0.355	-0.255	-0.059	0.019
-0.237	-0.03	-1.306	0	-0.674	0.789	-0.414
0.066	0.092	0.074	0.024	-0.096	0.128	-0.266
-0.008	-0.101	-0.333	-0.167	-0.141	-0.119	-0.15
-0.227	0.158	0.182	0.018	0.026	-0.043	0.044
0.023	-0.253	-0.02	-0.317	-0.11	0.127	0.157
-0.109	-0.243	-0.255	0.172	-0.032	0.214	-0.127
-0.055	-0.059	-0.074	-0.149	0.007	0.089	0.034
-0.117	0.14	-0.209	-1.148	-0.362	0.034	-0.097
-0.2	-0.433	0.109	-0.9	0.02	0.029	-0.208
-0.2	-0.036	0.068	0.101	-0.015	0.099	-0.049
0.146	-0.154	0.049	0.053	-0.115	-0.004	0.05
-0.01	0.087	-0.09	0.037	-0.165	0.114	0.116
-0.061	-0.016	-0.043	-0.016	-0.141	-0.077	0.202
0.246	-0.111	-0.206	0.008	-0.106	0.085	-0.071
-0.009	0.052	0.038	0.018	-0.095	0.056	-0.026

-0.043	0.069	-0.05	-0.018	-0.292	0.238	0.024
-0.028	0.106	-0.097	-0.092	-0.211	0.334	-0.238
0.076	-0.122	-0.014	0.044	-0.017	-0.023	-0.114
-0.028	-0.311	0.034	0.708	0.117	0.011	-0.158
-0.372	0.482	-0.057	-0.461	-0.183	-0.286	0.299
0.001	-0.295	-0.25	-0.766	-0.073	0.045	-0.007
-0.032	-0.209	-0.03	-0.019	-0.216	-0.01	0.114
0.371	-0.228	-0.185	-0.16	-0.278	0.584	-0.179
0.542	-0.208	-0.361	-0.288	0.009	0.203	-0.05
-0.179	0.65	0	-0.28	-0.053	0.104	-0.2
-0.158	0.184	-0.094	-0.114	-0.122	0.101	-0.164
-0.075	-0.018	0.329	-0.108	-0.011	0.202	-0.035
-0.095	-0.071	-0.206	-0.308	-0.04	0.185	-0.061
-0.207	-0.415	-0.12	0.35	-0.144	0.222	0.343
-0.004	-0.02	0.052	-0.107	-0.148	-0.105	-0.163
-0.175	-0.162	0.865	-0.584	0.02	-0.048	-0.188
0.243	-0.539	0.155	-0.237	-0.229	0.03	-0.085
1.698	0	0	0	-0.304	-0.05	-0.15
-0.035	0.414	-0.034	-0.167	-0.107	0.324	0.025
-0.325	0.691	-0.012	-0.207	0.01	0.163	-0.116
0	0	0	0	-0.458	-0.342	0.387
0.254	0.447	0.036	-0.05	0.004	0.28	0.413
-0.06	-0.017	-0.055	-0.317	-0.165	0.192	-0.1
-0.073	-0.131	0.104	-0.089	-0.015	0.162	-0.021
-0.186	-1.145	0	0	0	0.311	-0.057
-0.077	-0.175	0.338	-0.656	-0.149	-0.101	0.015
-0.036	-0.007	0.041	-0.983	-0.446	0.066	-0.129
-0.229	1	0.015	-0.12	-0.067	0.152	-0.3
0.069	0.07	-0.077	-0.003	-0.216	-0.201	0.096
-0.224	-0.218	-0.046	-0.065	-0.176	0.5	-0.335
-0.021	-0.182	-0.037	-0.01	-0.168	0.375	-0.293
-0.495	-0.054	-0.047	0.058	-0.048	-0.067	0.066
-0.005	0.044	0.041	-0.101	-0.105	0.274	-0.016
0.065	-0.136	0.146	0	-0.246	0.393	-0.233
-0.143	0	0.44	-1.094	-0.082	-0.089	0.138
-0.231	-0.176	-0.231	-0.231	0	0.356	-0.089
-0.252	0.263	0.074	0.116	-0.224	0.155	-0.172
-0.016	0.095	-0.028	-0.341	-0.091	0.143	-0.105
-0.229	-0.09	0.395	-0.344	-0.052	0.238	-0.179
-0.201	-0.172	-0.111	-0.095	0.206	0.276	-0.114
0.24	-0.941	0.716	-0.125	0.089	0.113	-0.039
0.36	-0.866	1.083	0	-0.01	-0.06	0.098
-0.288	0.1	0.338	-0.28	0.029	0.068	-0.166
-0.049	-0.048	-0.097	0.053	0.008	0.206	-0.097
-0.19	-0.208	0.753	-0.288	0.009	0.202	-0.152
-0.028	-0.185	-0.027	-0.048	-0.255	0.114	-0.3
-0.195	-0.172	-0.05	0.757	-0.127	0.164	0.3
-0.191	-0.204	0.216	0.051	0.046	0.205	-0.151
0.143	0	0	0	-0.178	0.061	-0.036
-0.073	-0.252	0.354	0.082	-0.204	-0.09	-0.024

-0.558	-0.21	0.125	0.186	-0.167	0.087	0.286
-0.222	0.288	0.026	-0.01	-0.272	0.019	0.039
-0.114	-0.276	0.505	0.119	-0.138	-0.096	-0.036
-0.092	-0.245	0.482	-0.383	-0.058	0.181	0.378
-0.042	-0.097	-0.149	-0.049	0.194	0.168	0.019
-0.048	0.352	-0.141	-0.196	-0.185	0.213	-0.148
-0.264	0.255	-0.176	-0.165	-0.096	0.134	0.187
0.139	-0.243	0.018	0.183	-0.145	0.221	-0.153
0.091	-0.176	0.029	0.176	-0.233	0.058	0.011
-0.391	-0.103	0.125	0.174	-0.138	0.108	0.16
0	0	0	0	-0.452	0.133	-0.008
-1	0	0	0	-1	0.98	-1
0.05	-0.127	0.02	0.085	-0.063	-0.035	-0.061
0.007	-0.126	0.076	-0.217	-0.163	0.081	-0.05
0.041	0.403	-0.069	-0.239	-0.211	0.148	-0.018
-0.154	-0.039	0.076	-0.419	0.023	-0.018	0.105
0.192	-0.146	-0.053	0.06	-0.23	-0.045	-0.151
-0.354	-0.38	0.407	-0.144	-0.186	-0.204	0.273
-0.908	2.27	0	0	-0.009	-0.051	0.248
0.016	-0.207	-0.02	-0.124	-0.112	-0.07	-0.079
0.005	-0.069	-0.006	0.019	-0.003	0.145	-0.004
-0.143	0	0.44	-1.094	-0.082	-0.089	0.138
-0.043	-0.222	0.222	0.047	-0.138	-0.072	-0.03
0.111	-0.115	0.063	0.272	-0.215	0.039	-0.014
-0.171	-0.204	-0.241	0.148	-0.083	-0.187	0.207
0.041	-0.089	-0.032	-0.184	-0.086	0.123	0.059
-0.296	-0.25	-0.674	-1.195	-0.141	0.064	-0.31
-0.047	-0.015	0.269	-0.168	-0.064	-0.126	0
0.117	-0.08	-0.062	-0.233	-0.077	-0.133	0.02
-0.241	-0.292	-0.545	-0.757	-0.056	0.198	-0.066
-0.339	0.291	-0.562	0.291	-0.395	0.483	-0.336
0.053	-0.003	-0.008	-0.057	-0.126	0.057	-0.134
-0.339	-0.03	0.415	-0.449	0.043	0.223	-0.162
-0.064	-0.119	-0.027	-0.138	0.043	0.097	-0.187
-0.289	-0.009	-0.271	0.878	0.05	0.185	-0.155
-0.439	0.232	-0.038	-0.866	-0.076	-0.026	0.028
-0.119	0.514	0.798	0	0	-0.177	-0.004
-0.09	-0.276	0.366	0.067	-0.157	-0.057	-0.076
-0.023	-0.148	-0.368	-0.211	-0.043	0.016	0.022
-0.001	0.063	-0.111	-0.17	-0.149	0.047	0.011
-0.073	-0.252	0.354	0.082	-0.204	-0.09	-0.024
-0.094	-0.022	-0.014	0.024	-0.222	-0.164	-0.086
-0.273	0.188	-0.226	0.086	-0.183	0.001	-0.166
0.047	-0.111	0.004	-0.08	0.001	0.074	0.001
-0.005	-0.233	0.067	-0.496	-0.061	0.174	0.373
-0.018	0.138	-0.027	-0.741	-0.121	-0.114	0.071
-0.126	-0.098	0.181	0.314	0.003	-0.003	-0.127
-0.038	-0.11	-0.126	-0.111	-0.142	0.139	0.084
-0.215	-0.667	-0.4	-1.2	-0.125	0.417	-0.334
0.131	-0.435	0.871	-0.199	-0.176	-0.104	-0.143

0.018	0.059	0.059	0.059	-0.1	-0.137	-0.124
-0.327	-0.57	-0.674	-1.195	-0.087	0.425	-0.303
-0.241	-0.191	0.056	0.186	0.031	0.089	-0.054
-0.309	-0.342	-0.26	0.234	-0.063	0.426	-0.057
-0.313	-0.447	-0.038	0.175	-0.161	-0.251	0.234
0.289	0.001	-0.118	-0.043	-0.214	0.265	-0.16
-0.161	0.214	0.065	0.008	-0.265	0.049	0.069
-0.037	-0.11	-0.082	0.015	-0.164	0.174	-0.203
-0.296	0.309	0.095	-0.002	-0.08	0.203	-0.119
-0.208	-0.49	0.222	0.176	-0.178	-0.201	0.176
-0.14	0.073	-0.091	-0.156	-0.032	0.079	-0.149
0.165	0.138	0.039	-0.153	-0.332	-0.018	0.021
-0.169	-0.043	0.104	-0.417	0.025	-0.02	0.1
-0.094	-0.095	-0.162	-0.326	0.011	-0.009	0.004
0.043	0.211	-0.262	0.085	-0.188	0.02	-0.072
-0.16	0.23	-0.176	0.116	-0.199	0.08	-0.042
-0.238	-0.26	0.025	0.076	-0.072	-0.143	0.157
-0.274	-0.349	-0.247	-0.1	-0.096	-0.163	0.361
0.083	0.083	0	0	0	-0.037	-0.145
-0.274	-0.349	-0.247	-0.1	-0.096	-0.163	0.361
-0.027	-0.179	-0.1	-0.895	-0.128	0.569	-0.195
-0.028	-0.01	0.062	-0.07	-0.161	-0.028	0.305
0.049	0.223	0.041	-0.158	-0.127	-0.11	-0.002
0.049	0.223	0.041	-0.158	-0.127	-0.11	-0.002
-0.149	-0.05	0.062	0.036	0.029	0.004	0.066
-0.132	0.105	-0.108	0.408	-0.097	0.105	-0.161
-0.34	0.666	-0.241	-0.172	-0.094	0.69	-0.073
-0.259	-0.24	0	0	-0.324	0.221	-0.132
0.177	-0.037	-0.218	-0.149	-0.03	-0.063	-0.223
-0.072	-0.175	-0.137	0.333	-0.152	0.156	-0.093
-0.102	0.017	-0.144	-0.142	-0.133	0.017	0.155
-0.359	-0.176	0.113	0.154	-0.124	0.09	0.155
-1	0	0	0	0	0.136	-0.405
-0.152	-0.167	0.14	-0.012	-0.142	0.016	-0.113
0.082	-0.05	-0.071	-0.009	-0.261	0.073	-0.092
-0.168	0.046	0.072	-0.164	-0.219	0.157	0.025
-0.027	-0.055	-0.028	0.009	-0.133	0.039	-0.02
-0.082	0.044	0.091	0.088	-0.079	0.126	-0.246
-0.096	-0.107	-0.052	0.073	-0.13	0.245	-0.28
0.205	-0.019	-0.175	-0.219	-0.009	0.061	-0.066
-0.106	0.055	0.12	0.122	-0.072	0.204	-0.383
-0.046	-0.059	-0.051	-0.362	-0.017	0.119	0.01
0.168	-0.063	-0.053	-0.05	-0.25	0.183	-0.177
-0.109	-0.129	-0.086	0.066	-0.303	0.376	-0.096
-0.143	-0.072	-0.148	-0.231	0.019	0.209	-0.212
-0.075	-0.009	-0.054	-0.081	-0.235	0.05	-0.021
-0.353	-0.329	0.132	0.141	-0.019	0.167	-0.048
0.002	-0.257	0.091	-0.346	-0.097	0.181	0.337
-0.008	-0.273	-0.236	-0.398	-0.53	0.069	0.375
-0.025	-0.1	-0.167	-0.214	-0.189	0.231	-0.179

0.165	0.138	0.039	-0.153	-0.332	-0.018	0.021
0.144	0.446	-0.005	-0.631	-0.055	-0.202	-0.088
-0.116	-0.127	-0.32	-0.526	0.013	-0.008	-0.045
0.07	-0.04	-0.08	0.01	-0.017	-0.041	-0.114
-0.378	0	0	0	-0.546	0.355	-0.454
-0.17	-0.51	-0.68	-1.53	-0.108	0.189	-0.277
-0.105	-0.115	-0.249	0.614	-0.058	-0.027	-0.072
-0.247	0.147	0.067	-0.1	-0.416	-0.122	0.394
-0.299	0.285	0.097	-0.23	-0.133	-0.112	0.103
-0.282	-0.238	-0.201	0.039	-0.143	0.454	-0.162
-0.231	-0.14	-0.102	0.056	-0.219	0.596	-0.397
0.128	-0.171	0.267	-0.371	-0.137	-0.081	-0.177
-0.088	-0.096	0.05	-0.048	-0.166	0.209	-0.007
-0.049	-0.24	0.227	-0.194	0.059	0.099	0.112
-0.182	-0.333	0.345	-0.043	-0.319	0.056	0.035
-0.209	0.039	-0.19	0.293	-0.35	0.16	-0.184
-0.583	0.25	0	0	-0.028	0.375	-0.25
0.032	-0.192	-0.209	-0.202	0.131	0.283	0.026
-0.046	-0.108	-0.134	-0.117	-0.127	0.136	0.081
-0.101	0.015	0.022	0.022	-0.199	-0.074	0.15
-0.145	-0.028	-0.062	0.035	-0.146	0.094	0.004
-0.241	-0.02	-0.163	0.107	-0.137	0.074	0.187
-0.286	0.15	-0.12	0.024	-0.032	0.064	-0.172
-0.054	-0.149	0.025	-0.088	-0.09	-0.036	-0.147
-0.181	0.056	-0.021	-0.421	-0.181	0.116	-0.368
-0.094	0.023	-0.09	0.022	-0.191	0.125	-0.048
0.034	-0.214	-0.209	0.035	-0.182	0.165	-0.209
-0.035	0.051	-0.023	-0.571	-0.067	0.109	0.017

MATS4v	MATS5v	MATS6v	MATS7v	MATS8v	MATS1e	MATS2e
-0.027	0.165	-0.528	0.369	-0.528	-0.083	0.096
-0.014	-0.059	-0.344	0.271	0.142	-0.119	-0.169
-0.082	-0.266	-0.019	-0.218	0.024	0.122	0.106
-0.319	0.106	-0.637	1.241	0	-0.066	-0.067
0.167	-0.248	0.283	-0.051	-1.097	-0.424	0.091
0.234	-0.604	0.222	0	0	-0.281	0.141
-0.177	0.198	-0.217	-0.216	0.049	-0.106	0.124
0.762	0	0	0	0	-0.211	0.06
-0.051	-0.219	-0.095	-0.228	-0.236	-0.015	-0.038
0.098	-0.265	-0.354	-0.232	-0.077	-0.109	-0.204
-0.086	0.111	0.016	-0.093	0.008	-0.166	-0.243
0.259	-0.263	-0.075	-0.237	0.131	-0.105	-0.152
0.017	-0.022	-0.133	-0.282	-0.1	-0.087	0.016
-0.03	-0.112	-0.037	-0.083	0.015	-0.169	0.102
-0.578	-0.05	0.17	0	0.123	-0.073	0.083
-0.605	0.026	0.186	-0.237	0.194	-0.079	0.144
-0.327	-0.092	-0.009	0.255	0	-0.013	-0.036
-0.265	-0.327	0.538	-0.019	0.21	-0.084	0.327
-0.066	-0.445	-0.509	0.51	-0.071	-0.118	-0.131
0.204	-0.379	-0.109	-0.067	0.13	-0.094	0.098
-0.051	-0.134	-0.168	-0.092	0.348	-0.127	0.152
-0.227	-0.315	-0.008	-0.189	0.422	-0.099	0.218
-0.253	-0.223	0.124	0.684	0.008	-0.067	0.106
-0.593	0.124	0.121	-0.213	0.316	-0.078	0.165
0.072	-0.2	-0.109	-0.101	-0.017	-0.144	-0.128
-0.14	-0.226	0.125	0.189	-0.081	0.026	-0.046
0.039	-0.295	-0.281	0.345	-0.063	-0.132	-0.217
-0.001	-0.193	0.058	-0.103	0.117	-0.044	0.188
0.025	-0.178	-0.099	-0.063	-0.09	0.077	-0.158
-0.066	-0.349	-0.017	-0.195	-0.057	-0.202	-0.286
-0.162	-0.03	0.17	-0.238	-0.073	-0.111	0.045
-0.019	0.088	-0.03	-0.116	0.062	-0.089	0.186
0.025	-0.29	-0.373	-0.107	0.098	-0.088	-0.186
-0.3	-0.321	-0.155	0.007	0.157	-0.096	0.106
-0.081	-0.002	0.041	-0.007	0.049	-0.067	-0.094
-0.213	0.015	-0.252	0.201	-0.042	-0.198	0.195
-0.138	-0.147	0.116	0.274	0.032	-0.025	-0.078
0.156	0.071	-1.443	0	0	-0.45	0.316
0.187	-0.016	-0.006	-0.056	-0.153	0.007	-0.051
-0.063	0.073	0.049	0.04	-0.253	-0.08	0.071
-0.111	-0.175	0.118	-0.203	-0.333	0.014	0.318
-0.187	-0.354	-0.256	0.187	0	-0.059	0.462
-0.043	0.169	-0.123	-0.103	-0.054	-0.008	0.231
-0.125	-0.201	0.507	-0.292	-0.396	-0.01	0.163
-0.242	-0.119	0.295	-0.256	-0.246	-0.144	0.012
-0.497	-0.074	0.146	-0.196	0.169	-0.079	0.088
-0.477	-0.134	0.132	0.046	0.098	-0.073	0.085
-0.425	-0.227	-0.029	-0.085	0.173	-0.053	0.116
-0.305	0.027	-0.008	-0.22	0.135	-0.113	0.073

-0.119	-0.108	0.083	-0.07	-0.241	-0.151	0.085
-0.102	-0.302	-0.04	0.241	-0.295	-0.109	0.05
-0.334	0.014	-0.209	-0.047	0.308	-0.105	0.099
-0.197	-0.192	0.103	0.086	-0.152	-0.171	0.05
-0.375	-0.105	0.11	0.057	0.109	-0.197	0.189
-0.41	-0.122	0.048	-0.008	0.088	-0.068	0.093
-0.127	-0.037	-0.196	0.057	-0.164	-0.004	0.261
-0.041	-0.095	-0.063	0.207	-0.306	-0.107	0.033
-0.196	-0.311	0.211	0.037	-0.147	-0.134	0.033
-0.148	-0.33	0.016	0.02	-0.141	-0.161	0.035
-0.143	-0.244	-0.045	0.163	-0.126	-0.187	0.08
0.006	-0.127	-0.167	-0.246	-0.4	0.052	0.41
-0.497	-0.074	0.146	-0.196	0.169	-0.079	0.152
-0.404	0.159	0.093	-0.246	0.021	-0.122	0.139
-0.418	0.035	0.104	-0.005	0.145	-0.141	0.127
-0.497	-0.074	0.146	-0.196	0.169	-0.079	0.152
-0.281	-0.2	-0.176	-0.107	-0.176	-0.105	0.234
-0.094	-0.05	0.363	-0.155	-0.253	-0.131	0.051
-0.309	-0.776	0.879	-0.88	0	0.126	0.199
-0.125	0.229	-0.208	-0.08	-0.022	-0.127	-0.08
0.311	-0.036	-0.064	-0.292	-0.081	-0.176	-0.153
0.213	-0.475	-0.445	-0.176	0.01	0.031	0.272
0.02	-0.011	-0.081	-0.422	-0.093	-0.085	-0.04
-0.231	-0.337	0.464	-0.383	0.29	0.105	0.188
0.263	-0.136	-0.206	-0.121	-0.163	-0.081	-0.138
0.209	-0.398	0.031	-0.306	0.18	-0.555	0.412
-0.185	0.057	-0.073	0.382	-0.189	-0.189	0.165
-0.129	-0.203	-0.103	-0.084	0.606	-0.004	0.582
0.233	-0.164	-0.447	0.149	0.585	-0.164	0.011
0.235	-0.092	-0.345	0.231	0.159	-0.088	-0.039
-0.083	-0.123	0.033	-0.072	0.055	-0.14	-0.174
-0.045	-0.258	-0.364	-0.236	-1	-0.104	-0.109
-0.005	-0.296	-0.253	0.756	-0.358	-0.081	0.033
-0.182	-0.51	0.018	0.273	0	-0.19	-0.054
-0.134	0.071	-0.092	0.197	-0.135	-0.214	-0.118
-0.192	-0.21	0.254	0.254	0	-0.513	0.373
-0.358	-0.276	0.837	-0.179	-0.434	0.182	0.084
0.326	-0.008	-0.101	-0.333	-0.167	-0.141	-0.119
-0.14	-0.226	0.125	0.189	-0.081	0.026	-0.046
-0.174	-0.177	-0.106	-0.245	-0.202	-0.086	0.04
-0.138	0.276	-0.057	-0.166	-0.857	-0.021	0.072
-0.241	0.007	-0.155	0.043	0.18	-0.031	0.005
0.106	-0.127	0.111	-0.159	-1.152	-0.297	-0.012
0.13	-0.225	-0.475	0.089	-0.988	0.02	0.01
-0.033	-0.2	-0.036	0.068	0.101	-0.015	0.099
0.079	0.146	-0.154	0.049	0.053	-0.115	-0.004
-0.229	0.008	-0.207	-0.011	0.173	-0.179	0.072
-0.263	-0.065	-0.036	-0.069	-0.036	-0.126	-0.081
-0.232	0.279	-0.098	-0.216	-0.006	-0.088	0.098
-0.213	-0.013	0.049	0.034	0.018	-0.092	0.048

-0.231	-0.146	-0.196	0.278	-0.16	0.041	0.208
-0.1	-0.357	0.328	-0.211	-0.029	-0.169	0.223
0.016	0.089	-0.137	-0.011	0.032	-0.006	-0.032
0.05	-0.075	-0.358	0.094	0.768	0.106	0.026
-0.329	-0.429	0.55	-0.029	-0.43	-0.148	-0.225
0.173	0.051	-0.287	-0.242	-0.757	-0.066	0.066
-0.02	-0.032	-0.209	-0.03	-0.019	-0.216	-0.01
0.115	-0.243	-0.377	-0.205	-0.612	-0.057	0.6
-0.095	0.542	-0.208	-0.361	-0.287	0.009	0.203
-0.22	-0.179	0.65	0	-0.28	-0.053	0.104
0.012	-0.162	0.187	-0.109	-0.128	-0.1	0.109
-0.226	0.251	-0.145	-0.229	-0.275	-0.025	0.167
-0.183	-0.098	-0.075	-0.224	-0.322	-0.03	0.166
-0.09	-0.163	-0.475	-0.057	0.545	-0.112	0.297
0.005	-0.023	-0.028	0.115	-0.014	-0.114	-0.134
-0.051	-0.223	-0.152	0.945	-0.473	0.019	-0.047
-0.112	0.232	-0.541	0.251	-0.222	-0.174	0.027
-0.314	1.593	0	0	0	-0.207	-0.137
-0.054	0.143	-0.249	-0.226	-0.257	-0.086	0.396
-0.229	-0.318	0.705	-0.049	-0.296	0.01	0.136
0	0	0	0	0	-0.446	-0.354
0.019	-0.023	0.041	0.169	-0.111	0.028	0.307
-0.263	-0.463	0.505	0.139	-0.475	-0.01	0.09
-0.119	-0.073	-0.131	0.104	-0.089	-0.015	0.162
-0.559	-0.074	-1.129	0	0	0	0.33
-0.026	-0.065	-0.143	0.365	-0.648	-0.136	-0.1
0.14	-0.021	-0.019	0.124	-1.033	-0.362	0.006
-0.175	-0.229	1	0.015	-0.12	-0.067	0.152
-0.124	0.078	0.054	-0.07	-0.001	-0.209	-0.178
0.085	-0.39	-0.356	-0.274	0.157	0.001	0.406
0.168	-0.4	-0.264	-0.196	0.164	-0.007	0.229
0.046	-0.501	-0.058	-0.023	0.063	-0.05	-0.032
-0.386	0.127	-0.065	-0.175	0.419	-0.094	0.267
-0.309	-0.776	0.879	-0.88	0	0.126	0.199
-0.206	-0.13	0.02	0.661	-1.085	-0.069	-0.085
-0.135	-0.231	-0.176	-0.231	-0.231	0	0.356
-0.172	-0.266	0.294	0.072	0.089	-0.197	0.148
-0.221	0.007	-0.253	0.785	-0.319	-0.081	0.099
-0.236	-0.228	-0.101	0.488	-0.336	-0.037	0.239
-0.249	-0.214	-0.178	-0.114	-0.105	0.163	0.247
-0.63	0.142	-0.891	0.666	0.092	-0.026	0.129
-0.606	0.28	-0.813	1.037	0	-0.083	-0.054
-0.106	-0.302	0.098	0.359	-0.3	0.005	0.093
-0.103	0.522	-0.157	-0.3	-0.282	0.011	0.143
-0.208	-0.19	-0.208	0.753	-0.288	0.009	0.202
0.138	-0.164	-0.168	0.29	-0.857	-0.305	0.071
0.011	-0.251	-0.401	0.195	-0.299	-0.075	0.284
-0.272	-0.191	-0.204	0.216	0.051	0.046	0.205
-0.377	0.107	0	0	0	-0.14	0.052
0.133	-0.093	-0.245	0.337	0.113	-0.154	-0.057

-0.292	-0.558	-0.242	0.128	0.201	-0.137	0.096
-0.14	-0.15	0.122	0.073	-0.01	-0.218	0.011
0.096	-0.138	-0.261	0.474	0.168	-0.091	-0.063
-0.215	-0.23	-0.265	-0.37	-0.255	-0.039	0.027
-0.102	-0.202	-0.207	-0.237	-0.244	0.097	0.169
-0.331	0.337	-0.214	-0.044	-0.135	-0.009	0.163
0.004	-0.14	-0.229	0.017	0.66	-0.088	0.099
-0.306	0.195	-0.261	-0.014	0.191	-0.127	0.245
-0.193	0.091	-0.176	0.029	0.176	-0.233	0.058
-0.366	-0.385	-0.121	0.129	0.187	-0.112	0.114
-0.274	0	0	0	0	-0.65	0.205
0.95	-1	0	0	0	-1	0.98
-0.114	0.066	-0.137	0.01	0.09	-0.05	-0.033
-0.161	-0.101	-0.011	0.097	0.019	-0.179	0.052
-0.126	0.004	0.387	-0.136	-0.254	-0.163	0.168
-0.056	-0.154	-0.039	0.076	-0.419	0.023	-0.018
0.221	-0.33	0.133	0.067	-0.204	-0.214	-0.113
0.006	-0.353	-0.393	0.367	-0.149	-0.176	-0.189
-0.473	-0.813	1.931	0	0	0.033	0.005
0.099	-0.166	0.082	-0.257	0.373	-0.077	-0.096
-0.094	-0.077	0.024	-0.056	-0.089	-0.008	0.098
-0.206	-0.13	0.02	0.661	-1.085	-0.069	-0.085
0.125	-0.063	-0.21	0.227	0.061	-0.103	-0.038
-0.281	0.095	-0.131	0.021	0.331	-0.197	0.047
-0.114	-0.177	-0.222	-0.263	0.116	-0.075	-0.173
-0.287	0.006	-0.033	0.031	0.118	-0.095	0.103
0.511	-0.283	-0.163	-0.651	-1.172	-0.107	0.088
-0.052	-0.033	-0.028	0.326	-0.162	-0.054	-0.119
-0.098	0.122	-0.048	-0.055	-0.226	-0.065	-0.125
-0.15	-0.241	-0.292	-0.545	-0.757	-0.056	0.198
0.003	-0.347	0.381	-0.627	0.311	-0.333	0.468
0.069	-0.144	-0.073	0.021	0.038	-0.17	0.053
-0.109	-0.355	-0.009	0.392	-0.436	0.019	0.256
-0.256	0.299	-0.04	-0.312	0.07	0.183	0.086
-0.075	-0.299	-0.04	-0.221	0.848	0.025	0.217
-0.175	-0.444	0.301	-0.068	-0.827	-0.068	-0.029
-0.51	-0.186	0.528	1.178	0	0	-0.18
0.217	-0.145	-0.281	0.286	0.121	-0.109	-0.022
0.019	-0.023	-0.148	-0.368	-0.211	-0.043	0.016
-0.059	-0.005	0.065	-0.104	-0.181	-0.145	0.032
0.133	-0.093	-0.245	0.337	0.113	-0.154	-0.057
0.171	-0.072	-0.172	0.337	-0.248	-0.149	-0.109
0.209	-0.265	0.225	-0.284	0.11	-0.151	-0.013
-0.287	0.017	-0.098	0.019	0.158	-0.022	0.06
-0.219	-0.266	-0.261	-0.39	-0.246	-0.043	0.076
-0.173	-0.015	0.146	0.043	-0.817	-0.109	-0.109
-0.124	-0.114	-0.077	0.175	0.299	-0.01	0
-0.119	-0.026	-0.111	-0.128	-0.116	-0.121	0.166
0.188	-0.221	-0.687	-0.376	-1.177	-0.098	0.322
-0.141	0.202	-0.456	0.753	-0.185	-0.162	-0.118

-0.055	0.018	0.059	0.059	0.059	-0.1	-0.137
0.229	-0.347	-0.586	-0.651	-1.172	-0.065	0.333
-0.094	-0.24	-0.183	0.071	0.198	0.009	0.087
-0.067	-0.322	-0.338	-0.237	0.25	-0.047	0.355
0.193	-0.311	-0.46	-0.036	0.218	-0.143	-0.228
-0.035	0.191	-0.178	-0.183	-0.165	0.017	0.203
-0.259	-0.138	0.198	0.048	0.024	-0.214	0.059
-0.012	-0.029	0.328	-0.357	-0.24	0.009	0.151
-0.074	-0.156	-0.49	1.005	-0.457	-0.064	0.142
-0.016	-0.174	-0.509	0.16	0.201	-0.157	-0.192
0.142	-0.14	0.073	-0.091	-0.156	-0.032	0.079
-0.304	0.207	0.139	0.051	-0.142	-0.294	-0.055
-0.078	-0.169	-0.043	0.104	-0.417	0.025	-0.02
-0.061	-0.094	-0.095	-0.162	-0.326	0.011	-0.009
-0.431	0.019	0.273	-0.262	0.075	-0.157	0.044
-0.488	-0.13	0.292	-0.198	0.1	-0.177	0.141
0.209	-0.236	-0.264	0.004	0.101	-0.066	-0.139
0.049	-0.273	-0.36	-0.272	-0.131	-0.088	-0.153
-0.255	0.083	0.083	0	0	0	-0.037
0.049	-0.273	-0.36	-0.272	-0.131	-0.088	-0.153
0.008	-0.381	-0.237	-0.51	-0.128	-0.108	0.233
-0.194	0.011	0.306	-0.219	0.016	-0.178	-0.085
-0.241	0.082	0.212	0.054	-0.168	-0.11	-0.094
-0.241	0.082	0.212	0.054	-0.168	-0.11	-0.094
0.213	-0.191	-0.023	-0.384	-0.13	-0.019	-0.033
0.083	-0.143	0.113	-0.113	0.416	-0.083	0.115
-0.456	-0.558	-0.882	-0.227	0.756	-0.112	0.77
0.4	-0.468	-0.063	-0.839	0	-0.245	0.239
-0.03	0.306	-0.049	-0.276	-0.162	-0.019	-0.064
-0.188	-0.072	-0.175	-0.137	0.333	-0.152	0.156
-0.086	-0.097	0.008	-0.147	-0.148	-0.115	0.039
-0.277	-0.356	-0.194	0.117	0.166	-0.1	0.099
0.012	-1	0	0	0	0	0.136
0.027	-0.159	-0.144	0.124	0.007	-0.128	0.035
-0.107	-0.168	0.416	-0.253	-0.136	-0.085	0.044
-0.294	-0.201	-0.173	0.348	0.421	0.074	0.143
-0.156	0.166	-0.015	-0.113	-0.154	-0.152	0.006
-0.316	0.037	0.036	-0.137	0.053	0.155	0.045
-0.162	-0.121	0.126	-0.188	0.062	0.16	0.227
-0.106	-0.32	0.372	-0.156	-0.207	0.057	0.018
-0.075	-0.251	0.09	-0.001	0.303	0.163	0.143
-0.064	-0.184	-0.184	0.367	-0.215	0.048	0.097
-0.098	-0.454	0.771	-0.113	-0.198	-0.041	0.151
-0.18	-0.105	-0.318	0.113	-0.303	0.057	0.375
-0.207	-0.126	-0.085	-0.245	0.946	-0.004	0.178
0.082	-0.31	-0.033	0.118	-0.218	-0.07	0.006
-0.279	-0.37	-0.382	0.118	0.097	-0.025	0.127
-0.23	-0.244	-0.215	-0.314	-0.136	-0.074	0.086
-0.053	-0.326	0.212	-0.377	0.269	-0.449	0.114
-0.407	0.467	-0.254	0.082	-0.152	-0.015	0.114

-0.304	0.207	0.139	0.051	-0.142	-0.294	-0.055
-0.165	0.106	0.53	0.006	-0.621	-0.043	-0.18
-0.025	-0.116	-0.127	-0.32	-0.526	0.013	-0.008
-0.018	0.086	-0.05	-0.087	-0.002	-0.006	-0.05
0.329	-0.32	0	0	0	-0.424	0.293
-0.073	-0.042	-0.15	0.389	0.514	-0.104	0.254
-0.433	-0.004	0.042	0.061	0.101	-0.102	-0.105
-0.13	-0.209	0.157	-0.014	-0.322	-0.303	-0.189
-0.13	-0.317	0.259	0.047	-0.245	-0.126	-0.127
0.044	-0.154	-0.615	-0.237	-0.214	0.097	0.37
0.208	-0.438	-0.501	-0.122	-0.192	0.074	0.459
-0.005	0.1	-0.179	0.329	-0.387	-0.12	-0.085
-0.144	-0.072	0.17	-0.187	-0.103	-0.167	0.101
-0.063	-0.193	-0.102	-0.211	-0.274	-0.035	-0.087
-0.079	-0.199	-0.364	0.412	-0.058	-0.302	-0.015
0.381	-0.23	0.045	-0.204	0.334	-0.29	0.134
-0.306	-0.583	0.25	0	0	-0.028	0.375
-0.283	-0.023	-0.203	-0.215	-0.214	0.079	0.27
-0.113	-0.034	-0.108	-0.137	-0.122	-0.11	0.164
-0.172	-0.121	0.051	0.041	0.032	-0.19	-0.073
0.1	-0.153	-0.016	-0.048	0.03	-0.128	0.119
-0.147	-0.243	0.021	-0.153	0.105	-0.116	0.125
0.04	-0.286	0.15	-0.12	0.024	-0.032	0.064
0.072	-0.043	-0.146	0.009	-0.109	-0.085	-0.012
0.191	-0.156	0.07	-0.052	-0.392	-0.182	0.063
-0.186	-0.008	-0.225	0.026	0.141	-0.107	0.021
0.192	0.045	-0.222	-0.205	0.047	-0.151	0.154
-0.025	-0.25	-0.199	0.658	-0.242	-0.053	0.073

MATS3e	MATS4e	MATS5e	MATS6e	MATS7e	MATS8e	MATS1p
-0.199	-0.027	0.165	-0.528	0.369	-0.528	-0.083
0.008	-0.016	-0.097	-0.338	0.329	0.102	-0.142
-0.191	-0.08	-0.268	-0.008	-0.205	-0.048	0.175
-0.201	-0.312	0.007	-0.654	1.39	0	-0.092
-0.113	0.085	-0.241	0.293	-0.105	-1.058	-0.551
-0.235	0.241	-0.596	-0.017	0	0	-0.66
0	-0.202	0.18	-0.206	-0.207	0.041	-0.077
-0.693	0.678	0	0	0	0	-0.256
-0.007	-0.103	0.124	-0.33	0.14	-0.347	0.069
0.356	0.047	-0.267	-0.342	-0.198	-0.055	-0.122
0.099	-0.091	0.079	0.027	-0.083	0.006	-0.176
-0.078	0.015	-0.2	0.14	-0.055	0.21	0.013
0.02	0.017	-0.022	-0.133	-0.282	-0.1	-0.087
-0.079	-0.132	-0.17	0.197	-0.031	-0.101	-0.146
0.039	-0.466	0.032	0.098	-0.044	0.126	-0.014
0.105	-0.477	0.127	0.127	-0.287	0.207	-0.015
0.029	-0.331	-0.109	-0.042	0.233	0	0.021
-0.129	-0.234	-0.298	0.574	-0.002	0.215	-0.109
0.218	-0.091	-0.434	-0.487	0.521	-0.058	-0.132
-0.171	0.191	-0.38	-0.142	-0.057	0.1	-0.115
-0.12	0.003	-0.144	-0.189	-0.09	0.317	-0.151
-0.023	-0.036	-0.352	-0.031	-0.302	0.184	0.013
-0.224	-0.244	-0.221	0.332	0.483	-0.138	-0.062
0.083	-0.46	0.205	0.051	-0.273	0.302	-0.015
0.197	0.078	-0.185	-0.098	-0.099	-0.028	-0.169
0.035	-0.145	-0.228	0.14	0.189	-0.043	0.026
0.174	-0.005	-0.295	-0.271	0.374	-0.061	-0.142
-0.081	-0.016	-0.156	0.031	-0.097	0.068	-0.067
0.099	0.025	-0.178	-0.099	-0.063	-0.09	0.077
0.355	-0.103	-0.348	0.003	-0.183	-0.055	-0.214
0.177	-0.175	-0.011	0.149	-0.277	-0.064	-0.424
-0.109	-0.094	0.093	-0.102	-0.176	0.015	-0.071
0.29	-0.013	-0.283	-0.364	-0.076	0.107	-0.099
0.136	-0.29	-0.324	-0.139	0.007	0.144	-0.126
0.037	-0.073	-0.007	0.022	-0.017	0.052	-0.078
-0.178	-0.155	-0.067	-0.213	0.224	-0.084	-0.261
-0.026	-0.128	-0.142	0.137	0.211	0.026	-0.041
-0.375	0.177	0.003	-1.544	0	0	-0.622
-0.025	0.187	-0.016	-0.006	-0.056	-0.153	0.007
0.004	-0.056	0.068	0.045	-0.01	-0.268	-0.104
-0.167	-0.096	-0.174	0.196	-0.198	-0.324	0.073
-0.109	-0.178	-0.347	-0.252	0.172	0	-0.081
-0.025	-0.043	0.169	-0.123	-0.103	-0.054	-0.008
-0.1	-0.156	-0.152	0.155	-0.374	-0.512	-0.02
0.209	-0.232	-0.135	0.32	-0.252	-0.265	-0.157
0.087	-0.335	0.165	0.134	-0.264	0.265	-0.014
0.029	-0.468	0.046	0.065	0.005	0.088	-0.014
0.097	-0.473	-0.047	0.064	-0.12	0.099	0.087
0.103	-0.449	0.172	0.028	-0.181	0.164	0.112

0.031	-0.128	0.058	-0.036	-0.093	-0.189	-0.005
0.138	-0.154	-0.134	-0.135	0.208	-0.205	0.026
0.022	-0.349	0.129	-0.196	-0.063	0.298	0.015
-0.057	-0.209	-0.032	0.035	0.083	-0.125	0.001
0.036	-0.337	-0.042	0.137	0.027	0.031	-0.028
0.052	-0.407	0.021	-0.019	-0.042	0.088	-0.014
-0.067	-0.161	-0.038	-0.235	0.073	-0.219	-0.265
0.022	-0.057	0.048	-0.143	0.145	-0.213	0.004
-0.023	-0.242	-0.073	0.087	0.046	-0.108	0.032
0.088	-0.189	-0.119	-0.006	0.01	-0.166	0.032
-0.05	-0.117	-0.156	-0.042	0.207	-0.169	-0.069
-0.001	-0.007	-0.092	-0.24	-0.374	-0.455	-0.262
0.095	-0.489	0.144	0.091	-0.242	0.169	-0.015
0.05	-0.32	0.269	-0.024	-0.257	-0.046	-0.01
-0.028	-0.348	0.118	0.051	0.003	0.066	-0.006
0.095	-0.489	0.144	0.091	-0.242	0.169	-0.015
0.019	-0.357	-0.037	-0.249	-0.133	-0.052	-0.016
0.463	-0.066	-0.024	0.338	-0.261	-0.42	-0.132
-0.413	-0.267	-0.584	0.912	-1.04	0	-0.635
-0.091	-0.088	0.049	-0.215	0.208	0.472	-0.087
-0.114	0.32	-0.014	-0.196	-0.203	0.277	0.007
-0.449	-0.021	-0.564	0.111	-0.25	0.807	-0.556
-0.048	-0.038	0.05	-0.218	-0.191	0.378	0.006
-0.273	-0.045	-0.275	0.426	-0.533	0.213	-0.41
-0.154	0.129	-0.142	0.143	-0.38	0.036	-0.068
-0.294	0.15	-0.066	-0.178	-0.136	0.182	-0.104
-0.059	-0.199	0.056	-0.104	0.418	-0.163	-0.207
-0.065	-0.111	-0.173	-0.08	-0.07	0.418	-0.012
-0.093	0.199	-0.186	-0.461	0.182	0.671	-0.158
-0.075	0.164	-0.066	-0.342	0.27	0.113	-0.143
0.231	-0.037	0.031	0.039	-0.075	0.011	-0.043
0.05	-0.066	0.053	-0.479	-0.894	2.096	-0.067
0.048	-0.05	-0.294	-0.135	0.611	-0.429	-0.081
-0.193	-0.202	0.51	-0.461	0.504	0	-0.092
-0.08	0.01	0.188	0.054	-0.134	-0.542	-0.184
-0.257	-0.535	0.728	-0.142	-0.867	0	-0.91
-0.383	-0.472	-0.127	0.722	-0.093	-0.337	-0.44
-0.15	0.326	-0.008	-0.101	-0.333	-0.167	-0.141
0.035	-0.145	-0.228	0.14	0.189	-0.043	0.026
0.032	-0.179	0.075	-0.335	0.21	-0.5	-0.084
-0.045	-0.135	-0.033	-0.288	-0.411	0.912	-0.02
-0.103	-0.202	0.078	0.005	-0.026	0.037	0.042
-0.064	0.051	-0.116	0.143	-0.214	-1.146	-0.386
-0.204	0.126	-0.217	-0.463	0.107	-0.964	0.02
-0.049	-0.033	-0.2	-0.036	0.068	0.101	-0.015
0.05	0.079	0.146	-0.154	0.049	0.053	-0.115
0.13	-0.228	0.014	-0.148	-0.032	0.146	-0.077
0.145	-0.27	-0.061	-0.014	-0.04	-0.014	-0.146
-0.071	-0.221	0.242	-0.112	-0.205	0.009	-0.112
-0.025	-0.216	-0.009	0.053	0.039	0.018	-0.096

-0.022	-0.306	-0.193	-0.11	0.255	-0.164	-0.662
-0.233	-0.082	-0.27	0.435	-0.244	-0.129	-0.191
-0.114	0.011	0.074	-0.12	-0.014	0.046	-0.021
-0.149	0.038	-0.023	-0.306	0.028	0.701	0.119
0.213	-0.326	-0.366	0.473	-0.06	-0.464	-0.197
-0.006	0.204	-0.005	-0.295	-0.251	-0.766	-0.076
0.114	-0.02	-0.032	-0.209	-0.03	-0.019	-0.216
-0.258	0.105	-0.29	-0.142	-0.274	-0.537	-0.375
-0.05	-0.095	0.542	-0.208	-0.361	-0.288	0.009
-0.2	-0.22	-0.179	0.65	0	-0.28	-0.053
-0.149	-0.032	-0.157	0.183	-0.092	-0.112	-0.131
-0.097	-0.046	0.071	-0.084	-0.324	-0.427	0.007
-0.055	-0.178	-0.094	-0.07	-0.204	-0.306	-0.045
0.357	-0.073	-0.186	-0.447	-0.095	0.464	-0.146
-0.183	0.078	-0.015	-0.003	0.077	-0.075	-0.153
-0.176	-0.056	-0.207	-0.158	0.935	-0.521	0.02
-0.093	-0.128	0.227	-0.554	0.22	-0.247	-0.244
-0.116	-0.414	1.83	0	0	0	-0.332
-0.012	-0.087	0.086	-0.089	-0.221	-0.276	-0.054
-0.118	-0.234	-0.324	0.716	-0.035	-0.264	0.01
0.393	0	0	0	0	0	-0.545
0.456	0.021	0.047	0.146	0.155	-0.106	-0.053
-0.213	-0.106	-0.416	0.525	0.211	-0.637	-0.401
-0.021	-0.119	-0.073	-0.131	0.104	-0.089	-0.015
-0.034	-0.53	-0.197	-1.145	0	0	0
0.026	-0.01	-0.079	-0.178	0.335	-0.657	-0.155
-0.063	0.1	-0.038	-0.006	0.031	-0.976	-0.479
-0.3	-0.175	-0.229	1	0.015	-0.12	-0.067
0.091	-0.137	0.068	0.072	-0.078	-0.003	-0.217
-0.282	0.077	-0.423	-0.403	-0.261	0.08	-0.415
-0.269	0.057	-0.161	-0.359	-0.194	0.075	-0.404
0.063	0.009	-0.493	-0.054	-0.049	0.058	-0.047
-0.094	-0.38	0.091	-0.014	-0.074	0.31	-0.094
-0.413	-0.267	-0.584	0.912	-1.04	0	-0.635
0.153	-0.198	-0.145	-0.002	0.416	-1.094	-0.087
-0.089	-0.135	-0.231	-0.176	-0.231	-0.231	0
-0.168	-0.186	-0.25	0.259	0.074	0.119	-0.232
-0.106	-0.208	-0.074	-0.06	0.635	-0.521	-0.081
-0.166	-0.226	-0.229	-0.089	0.385	-0.344	-0.058
-0.102	-0.239	-0.199	-0.171	-0.111	-0.094	0.221
0.023	-0.657	0.249	-0.946	0.722	-0.147	0.14
0.139	-0.634	0.368	-0.871	1.087	0	0.023
-0.154	-0.138	-0.286	0.101	0.335	-0.278	0.038
-0.158	-0.184	0.367	-0.182	-0.35	0.02	0.005
-0.152	-0.208	-0.19	-0.208	0.753	-0.287	0.009
-0.135	0	-0.039	-0.3	0.442	-0.881	-0.081
0.308	-0.079	-0.278	-0.4	0.077	-0.008	-0.147
-0.151	-0.272	-0.191	-0.204	0.216	0.051	0.046
-0.09	-0.366	0.146	0	0	0	-0.194
-0.053	0.104	-0.076	-0.265	0.375	0.088	-0.219

0.242	-0.288	-0.557	-0.206	0.125	0.184	-0.179
0.01	-0.171	-0.193	0.232	0.06	-0.04	-0.286
-0.06	0.049	-0.117	-0.283	0.527	0.13	-0.151
0.131	-0.205	-0.181	-0.279	0.479	-0.635	-0.041
0.07	0.06	-0.194	-0.262	-0.344	-0.247	0.204
-0.275	-0.25	0.327	-0.2	-0.005	-0.243	-0.339
0.297	-0.085	-0.391	-0.349	-0.288	0.151	-0.06
-0.158	-0.304	0.133	-0.241	0.021	0.182	-0.152
0.011	-0.193	0.091	-0.176	0.029	0.176	-0.233
0.127	-0.358	-0.391	-0.1	0.124	0.172	-0.148
-0.088	0.02	0	0	0	0	-0.121
-1	0.95	-1	0	0	0	-1
-0.066	-0.112	0.048	-0.126	0.021	0.084	-0.068
-0.09	-0.083	-0.057	-0.083	0.151	-0.028	-0.077
-0.034	-0.157	0.045	0.405	-0.061	-0.237	-0.23
0.105	-0.056	-0.154	-0.039	0.076	-0.419	0.023
-0.161	0.074	0.28	-0.105	-0.032	0.01	-0.188
0.255	-0.039	-0.353	-0.378	0.411	-0.144	-0.189
0.182	-0.498	-0.917	2.302	0	0	-0.033
-0.104	0.021	-0.01	-0.124	0.049	0.108	-0.093
-0.045	-0.061	0.055	-0.005	0.003	-0.052	0.006
0.153	-0.198	-0.145	-0.002	0.416	-1.094	-0.087
-0.049	0.105	-0.049	-0.229	0.249	0.045	-0.148
-0.048	-0.284	0.112	-0.114	0.068	0.266	-0.223
0.198	-0.145	-0.171	-0.202	-0.238	0.151	-0.086
0.024	-0.189	0.059	-0.087	0.043	0.082	-0.034
-0.276	0.485	-0.297	-0.259	-0.676	-1.196	-0.156
-0.001	-0.041	-0.048	-0.014	0.263	-0.169	-0.069
0.017	-0.086	0.117	-0.083	-0.062	-0.234	-0.082
-0.066	-0.15	-0.241	-0.292	-0.545	-0.757	-0.056
-0.336	-0.015	-0.338	0.282	-0.555	0.288	-0.419
-0.108	-0.013	0.198	-0.145	-0.013	-0.15	-0.091
-0.15	-0.141	-0.338	-0.032	0.417	-0.45	0.053
-0.239	-0.263	0.29	-0.07	-0.272	0.037	-0.256
-0.147	-0.113	-0.288	-0.006	-0.276	0.88	0.06
0.039	-0.146	-0.437	0.225	-0.035	-0.868	-0.079
-0.02	-0.482	-0.113	0.512	0.758	0	0
-0.087	0.145	-0.114	-0.289	0.34	0.077	-0.172
0.022	0.019	-0.023	-0.148	-0.368	-0.211	-0.043
0.002	-0.044	-0.001	0.062	-0.112	-0.168	-0.151
-0.053	0.104	-0.076	-0.265	0.375	0.088	-0.219
-0.109	0.044	-0.117	-0.06	0.318	-0.221	-0.25
-0.163	0.198	-0.274	0.184	-0.219	0.084	-0.194
-0.024	-0.21	0.083	-0.149	0.037	0.159	0.039
0.239	-0.237	-0.117	-0.436	0.276	-0.649	-0.053
0.068	-0.163	-0.019	0.137	-0.034	-0.733	-0.126
-0.127	-0.087	-0.127	-0.1	0.181	0.315	0.011
0.066	-0.112	-0.039	-0.11	-0.126	-0.111	-0.151
-0.315	0.225	-0.215	-0.664	-0.402	-1.201	-0.137
-0.131	-0.14	0.124	-0.433	0.883	-0.2	-0.182

-0.124	-0.055	0.018	0.059	0.059	0.059	-0.1
-0.286	0.262	-0.325	-0.568	-0.676	-1.196	-0.096
-0.059	-0.047	-0.241	-0.192	0.055	0.184	0.042
-0.06	-0.016	-0.308	-0.342	-0.261	0.232	-0.07
0.251	0.125	-0.312	-0.445	-0.039	0.171	-0.168
-0.184	-0.047	0.112	-0.035	-0.203	-0.159	-0.406
0.028	-0.247	-0.164	0.216	0.067	0.006	-0.283
-0.197	-0.026	-0.046	0.299	-0.377	-0.245	-0.422
-0.073	-0.23	-0.381	0.541	0.66	-0.447	-0.066
0.183	-0.053	-0.211	-0.487	0.228	0.173	-0.186
-0.149	0.142	-0.14	0.073	-0.091	-0.156	-0.032
0.069	-0.294	0.16	0.137	0.037	-0.155	-0.346
0.1	-0.078	-0.169	-0.043	0.104	-0.417	0.025
0.004	-0.061	-0.094	-0.095	-0.162	-0.326	0.011
-0.107	-0.41	0.045	0.204	-0.261	0.086	-0.199
-0.072	-0.492	-0.164	0.223	-0.173	0.118	-0.207
0.145	0.185	-0.238	-0.259	0.027	0.073	-0.075
0.347	0	-0.274	-0.348	-0.245	-0.097	-0.099
-0.145	-0.255	0.083	0.083	0	0	0
0.347	0	-0.274	-0.348	-0.245	-0.097	-0.099
-0.208	-0.08	-0.326	-0.419	0.869	-1.286	-0.09
0.321	-0.144	-0.014	0.28	-0.128	-0.044	-0.062
-0.009	-0.234	0.045	0.225	0.039	-0.157	-0.134
-0.009	-0.234	0.045	0.225	0.039	-0.157	-0.134
0.143	0.083	-0.214	0.059	-0.14	-0.137	0.098
-0.143	0.101	-0.131	0.104	-0.108	0.407	-0.103
-0.072	-0.425	-0.667	-0.576	-0.499	0.2	-0.008
-0.086	0.407	-0.494	-0.219	-0.913	0	-0.65
-0.188	0.023	0.201	-0.035	-0.235	-0.15	-0.037
-0.093	-0.188	-0.072	-0.175	-0.137	0.333	-0.152
0.125	-0.08	-0.102	0.018	-0.144	-0.141	-0.141
0.125	-0.273	-0.359	-0.174	0.113	0.153	-0.132
-0.405	0.012	-1	0	0	0	0
-0.133	0.011	-0.151	-0.17	0.142	-0.014	-0.148
-0.177	-0.133	-0.09	0.388	-0.254	-0.06	-0.452
-0.033	-0.355	-0.302	-0.121	0.492	0.284	-0.585
-0.042	-0.144	0.15	-0.063	-0.12	-0.056	-0.043
-0.309	-0.349	0.103	-0.049	-0.103	0.018	-0.417
-0.316	-0.212	-0.152	0.021	-0.225	0.041	-0.492
-0.123	-0.127	-0.124	0.34	-0.215	-0.281	-0.13
-0.407	-0.098	-0.324	0.018	0.068	0.326	-0.418
0.004	-0.066	-0.198	-0.205	0.359	-0.311	-0.143
-0.225	-0.119	-0.289	0.711	-0.148	-0.17	-0.503
-0.126	-0.219	-0.181	-0.306	-0.023	-0.169	-0.659
-0.14	-0.268	-0.186	-0.117	-0.319	0.845	0.029
-0.001	0.047	-0.313	-0.008	0.018	-0.234	-0.48
-0.068	-0.266	-0.351	-0.323	0.133	0.145	-0.016
0.211	-0.238	-0.094	-0.41	0.305	-0.421	-0.084
0.458	-0.086	-0.337	0.157	-0.387	0.123	-0.593
-0.279	-0.244	0.55	-0.288	0.006	-0.272	-0.337

0.069	-0.294	0.16	0.137	0.037	-0.155	-0.346
-0.118	-0.14	0.148	0.437	-0.006	-0.632	-0.06
-0.045	-0.025	-0.116	-0.127	-0.32	-0.526	0.013
-0.114	-0.02	0.069	-0.039	-0.08	0.011	-0.021
-0.46	0.333	-0.384	0	0	0	-0.592
-0.327	0.062	-0.109	-0.354	0.143	-0.056	-0.046
-0.228	0.321	-0.125	-0.08	-0.158	0.117	0.007
0.507	-0.214	-0.341	0.293	-0.008	-0.321	-0.451
0.083	-0.144	-0.297	0.288	0.102	-0.229	-0.135
-0.116	0.073	-0.292	-0.704	-0.318	-0.221	-0.473
-0.324	0.138	-0.53	-0.563	-0.173	-0.19	-0.585
-0.176	-0.026	0.13	-0.17	0.26	-0.369	-0.143
-0.083	-0.066	-0.175	0.018	-0.019	-0.171	-0.125
-0.079	-0.047	-0.003	-0.258	0.311	-0.059	0.089
0.1	-0.093	-0.179	-0.329	0.335	-0.043	-0.323
-0.187	0.351	-0.206	0.038	-0.188	0.288	-0.372
-0.25	-0.306	-0.583	0.25	0	0	-0.028
0.025	-0.272	0.038	-0.191	-0.208	-0.2	0.155
0.063	-0.106	-0.048	-0.107	-0.134	-0.116	-0.135
0.173	-0.156	-0.099	0.012	0.02	0.02	-0.202
0.025	0.074	-0.144	-0.029	-0.064	0.035	-0.154
0.153	-0.131	-0.241	-0.025	-0.163	0.107	-0.145
-0.172	0.04	-0.286	0.15	-0.12	0.024	-0.032
-0.137	0.027	-0.055	-0.149	0.027	-0.086	-0.091
-0.305	0.191	-0.184	0.054	-0.018	-0.424	-0.176
-0.097	-0.151	-0.017	0.134	-0.116	0.294	-0.205
-0.19	0.177	0.033	-0.213	-0.209	0.033	-0.193
-0.041	-0.114	-0.255	-0.003	0.625	-0.642	-0.06

MATS2p	MATS3p	MATS4p	MATS5p	MATS6p	MATS7p	MATS8p
0.096	-0.199	-0.027	0.165	-0.528	0.369	-0.528
-0.176	0.002	-0.013	-0.043	-0.345	0.244	0.157
0.095	-0.194	-0.083	-0.264	-0.025	-0.223	0.056
-0.055	-0.227	-0.321	0.146	-0.625	1.17	0
0.187	-0.202	0.195	-0.247	0.273	-0.033	-1.097
0.225	-0.292	0.236	-0.303	0.303	0	0
0.141	0.022	-0.164	0.204	-0.221	-0.216	0.051
0.07	-0.721	0.774	0	0	0	0
0.117	0.118	-0.009	-0.334	0.01	-0.42	-0.123
-0.223	0.375	0.12	-0.263	-0.358	-0.246	-0.086
-0.259	0.086	-0.083	0.124	0.011	-0.097	0.009
0.004	-0.142	0.28	-0.172	-0.112	-0.247	-0.062
0.016	0.02	0.017	-0.022	-0.133	-0.282	-0.1
0.129	0.034	0.021	-0.071	-0.161	-0.1	0.067
0.1	0.085	-0.602	-0.085	0.12	0.073	0.153
0.127	0.129	-0.628	-0.037	0.109	-0.098	0.199
-0.092	0.025	-0.322	-0.084	0.005	0.261	0
0.293	-0.087	-0.262	-0.328	0.532	-0.023	0.183
-0.119	0.22	-0.055	-0.446	-0.515	0.5	-0.076
0.064	-0.166	0.209	-0.377	-0.095	-0.071	0.141
0.2	-0.115	-0.074	-0.13	-0.156	-0.091	0.358
0.064	0.001	-0.417	-0.175	0.024	-0.04	0.43
0.191	-0.158	-0.209	-0.182	-0.04	0.66	0.063
0.143	0.09	-0.639	0.03	0.136	-0.078	0.293
-0.157	0.269	0.067	-0.205	-0.112	-0.101	-0.012
-0.044	0.042	-0.141	-0.226	0.129	0.189	-0.072
-0.234	0.2	0.057	-0.294	-0.285	0.331	-0.064
0.154	-0.11	0.009	-0.208	0.068	-0.105	0.139
-0.158	0.099	0.025	-0.178	-0.099	-0.063	-0.09
-0.303	0.368	-0.05	-0.348	-0.026	-0.2	-0.057
0.143	0.19	-0.121	-0.058	0.165	-0.145	-0.066
0.105	-0.008	0.005	0.067	-0.007	-0.081	0.068
-0.206	0.308	0.041	-0.292	-0.376	-0.121	0.093
0.091	0.178	-0.301	-0.317	-0.161	0.007	0.16
-0.101	0.018	-0.084	0	0.05	-0.003	0.048
0.243	-0.17	-0.232	0.051	-0.266	0.185	-0.024
-0.09	-0.01	-0.142	-0.149	0.107	0.298	0.035
0.402	-0.361	0.143	0.095	-1.377	0	0
-0.051	-0.025	0.187	-0.016	-0.006	-0.056	-0.153
0.05	-0.019	-0.065	0.074	0.049	0.061	-0.244
0.348	-0.195	-0.119	-0.175	0.087	-0.203	-0.333
0.53	-0.139	-0.189	-0.354	-0.254	0.192	0
0.231	-0.025	-0.043	0.169	-0.123	-0.103	-0.054
0.189	-0.067	-0.084	-0.231	0.556	-0.193	-0.26
-0.02	0.25	-0.245	-0.112	0.281	-0.257	-0.233
0.079	0.191	-0.397	-0.312	0.048	-0.061	0.131
0.037	0.111	-0.361	-0.324	0.095	0.105	0.14
-0.003	0.068	-0.157	-0.339	-0.167	-0.083	0.204
0.064	0.114	-0.034	-0.119	0.002	-0.229	0.046

-0.057	0.097	-0.116	-0.262	0.123	-0.068	-0.037
-0.041	0.16	-0.048	-0.413	0.031	0.169	-0.197
0.026	0.048	-0.154	-0.126	-0.191	-0.037	0.213
-0.041	0.059	-0.165	-0.31	0.084	0.041	0.026
0.202	0.026	-0.332	-0.13	-0.003	0.148	0.184
0.042	0.121	-0.303	-0.273	0.038	0.062	0.128
0.179	-0.079	-0.06	-0.05	-0.038	-0.02	-0.021
-0.057	0.084	-0.062	-0.255	0.02	0.177	-0.224
-0.068	0.075	-0.111	-0.45	0.182	-0.023	0.05
-0.078	0.163	-0.037	-0.439	-0.039	-0.02	-0.002
0.076	0.074	-0.156	-0.284	-0.123	0.039	0.018
0.306	0.052	0.028	-0.163	-0.136	-0.091	-0.191
0.069	0.155	-0.367	-0.31	0.084	-0.072	0.188
0.093	0.083	-0.401	-0.023	0.143	-0.063	0.12
0.128	0.058	-0.405	-0.065	0.082	0.086	0.211
0.069	0.155	-0.367	-0.31	0.084	-0.072	0.188
0.16	0.073	-0.09	-0.404	-0.033	-0.02	-0.289
0.072	0.532	-0.089	-0.051	0.298	-0.085	-0.142
0.299	-0.034	0.006	-0.857	0.606	-0.293	0
-0.02	-0.022	-0.07	0.221	-0.099	-0.304	-0.545
-0.003	-0.209	0.045	0.151	0.002	-0.331	-0.185
0.487	-0.359	0.291	-0.038	-0.407	-0.088	-0.327
-0.051	-0.065	-0.037	0.174	0.025	-0.38	-0.241
0.254	-0.022	-0.179	-0.357	0.361	-0.278	0.273
-0.083	-0.002	0.277	-0.101	-0.354	0	-0.388
0.361	-0.451	0.1	-0.583	0.157	-0.359	0.133
0.184	-0.092	-0.178	0.057	-0.06	0.365	-0.199
0.538	-0.075	-0.132	-0.208	-0.107	-0.086	0.64
-0.061	-0.084	0.246	-0.153	-0.438	0.135	0.546
-0.1	-0.053	0.236	-0.093	-0.338	0.229	0.169
-0.166	0.156	-0.094	-0.235	0.11	-0.115	0.008
0.047	0.267	-0.029	-0.373	-0.247	0.043	-2.607
0.054	0.12	0.02	-0.254	-0.281	0.726	-0.315
0.464	-0.35	0.131	-0.904	0.085	0.059	0
-0.011	0.062	-0.167	-0.019	-0.155	0.252	0.036
0.446	-0.08	0.194	-0.338	0.086	0.037	0
0.147	-0.055	-0.119	-0.457	0.577	-0.115	-0.226
-0.119	-0.15	0.326	-0.008	-0.101	-0.333	-0.167
-0.044	0.042	-0.141	-0.226	0.129	0.189	-0.072
0.133	0.178	-0.119	-0.316	0.002	-0.431	-0.044
0.202	-0.198	0.024	0.312	0.022	-0.037	-1.597
0.083	0.047	-0.268	-0.059	-0.149	-0.038	0.182
0.055	-0.108	0.125	-0.133	0.096	-0.139	-1.139
0.024	-0.207	0.129	-0.223	-0.473	0.095	-0.983
0.099	-0.049	-0.033	-0.2	-0.036	0.068	0.101
-0.004	0.05	0.079	0.146	-0.154	0.049	0.053
0.144	0.012	-0.174	-0.019	-0.266	0.036	0.152
-0.075	0.222	-0.258	-0.066	-0.046	-0.082	-0.046
0.076	-0.071	-0.235	0.289	-0.091	-0.218	-0.013
0.06	-0.026	-0.212	-0.014	0.047	0.032	0.018

0.201	0.056	-0.068	-0.04	-0.217	0.226	-0.142
0.331	-0.199	-0.104	-0.354	0.235	-0.161	0.02
-0.019	-0.113	0.018	0.096	-0.144	-0.01	0.025
0.004	-0.161	0.056	-0.097	-0.378	0.124	0.788
-0.311	0.333	-0.327	-0.454	0.57	-0.014	-0.41
0.035	-0.008	0.159	0.075	-0.282	-0.237	-0.75
-0.01	0.114	-0.02	-0.032	-0.209	-0.03	-0.019
0.279	-0.133	0.074	0.063	-0.439	-0.157	-0.455
0.203	-0.05	-0.095	0.542	-0.208	-0.361	-0.288
0.104	-0.2	-0.22	-0.179	0.65	0	-0.28
0.094	-0.169	0.031	-0.162	0.184	-0.115	-0.133
0.034	0.014	-0.419	0.222	-0.093	0.068	-0.067
0.189	-0.063	-0.184	-0.099	-0.077	-0.231	-0.326
0.227	0.336	-0.094	-0.17	-0.476	-0.051	0.538
-0.091	-0.162	-0.008	-0.023	-0.042	0.121	-0.003
-0.048	-0.185	-0.052	-0.22	-0.154	0.945	-0.485
0.022	-0.082	-0.106	0.243	-0.534	0.242	-0.209
-0.038	-0.127	-0.324	1.511	0	0	0
0.301	0.024	0	0.149	-0.498	-0.219	-0.152
0.157	-0.116	-0.23	-0.32	0.709	-0.046	-0.288
-0.225	0.298	0	0	0	0	0
0.259	0.374	0.022	-0.157	-0.187	0.153	-0.08
0.19	0.078	-0.203	-0.531	0.376	0.096	-0.381
0.162	-0.021	-0.119	-0.073	-0.131	0.104	-0.089
0.302	-0.067	-0.569	-0.024	-1.115	0	0
-0.102	0.011	-0.034	-0.059	-0.127	0.376	-0.641
0.096	-0.155	0.153	-0.015	-0.027	0.157	-1.044
0.152	-0.3	-0.175	-0.229	1	0.015	-0.12
-0.209	0.096	-0.117	0.08	0.047	-0.067	0
0.452	-0.338	0.126	-0.213	-0.187	-0.248	0.175
0.348	-0.291	0.209	-0.316	-0.101	-0.184	0.157
-0.082	0.065	0.06	-0.499	-0.061	-0.012	0.064
0.292	0.007	-0.342	0.138	-0.086	-0.223	0.425
0.299	-0.034	0.006	-0.857	0.606	-0.293	0
-0.091	0.131	-0.208	-0.123	0.029	0.763	-1.076
0.356	-0.089	-0.135	-0.231	-0.176	-0.231	-0.231
0.155	-0.172	-0.165	-0.27	0.305	0.07	0.075
0.147	-0.088	-0.193	0.047	-0.336	0.752	-0.189
0.236	-0.183	-0.24	-0.227	-0.107	0.53	-0.33
0.284	-0.118	-0.251	-0.217	-0.179	-0.114	-0.109
0.105	-0.068	-0.614	0.091	-0.861	0.645	0.195
-0.063	0.079	-0.591	0.239	-0.785	1.011	0
0.058	-0.171	-0.093	-0.308	0.095	0.366	-0.307
0.2	-0.055	-0.052	0.506	-0.117	-0.221	-0.419
0.202	-0.152	-0.208	-0.19	-0.208	0.753	-0.288
0.063	-0.368	0.22	-0.347	0.01	0.006	-0.47
0.074	0.243	0.132	-0.172	-0.267	0.206	-0.698
0.205	-0.151	-0.272	-0.191	-0.204	0.216	0.051
0.061	-0.011	-0.377	0.088	0	0	0
-0.106	-0.01	0.134	-0.097	-0.234	0.321	0.117

0.079	0.301	-0.291	-0.552	-0.254	0.128	0.206
0.012	0.047	-0.126	-0.144	0.103	0.064	0.013
-0.114	-0.021	0.095	-0.144	-0.252	0.46	0.175
0.193	0.371	-0.172	-0.231	-0.252	-0.552	-0.057
0.127	-0.02	-0.144	-0.166	-0.146	-0.151	-0.196
0.117	0.017	-0.321	0.239	-0.019	-0.147	-0.002
0.122	0.041	0.026	-0.021	0.042	0.143	0.658
0.21	-0.15	-0.305	0.219	-0.269	-0.029	0.194
0.058	0.011	-0.193	0.091	-0.176	0.029	0.176
0.102	0.171	-0.366	-0.379	-0.129	0.129	0.192
0.011	-0.026	-0.503	0	0	0	0
0.98	-1	0.95	-1	0	0	0
-0.036	-0.059	-0.114	0.073	-0.141	0.006	0.092
0.089	-0.028	-0.258	-0.11	0.061	-0.05	0.111
0.141	-0.01	-0.108	-0.012	0.381	-0.165	-0.258
-0.018	0.105	-0.056	-0.154	-0.039	0.076	-0.419
-0.006	-0.14	0.246	-0.571	0.211	0.098	-0.301
-0.209	0.28	0.026	-0.352	-0.398	0.346	-0.151
-0.071	0.272	-0.46	-0.76	1.758	0	0
-0.036	-0.047	0.116	-0.242	0.148	-0.401	0.409
0.118	0.029	-0.058	-0.316	0.03	-0.141	-0.124
-0.091	0.131	-0.208	-0.123	0.029	0.763	-1.076
-0.089	-0.02	0.124	-0.065	-0.201	0.213	0.066
0.035	0.001	-0.279	0.088	-0.138	-0.001	0.358
-0.193	0.211	-0.1	-0.179	-0.231	-0.273	0.099
0.101	0.038	-0.382	-0.04	0.031	-0.013	0.172
0.055	-0.323	0.516	-0.275	-0.122	-0.635	-1.152
-0.129	0	-0.057	-0.027	-0.034	0.351	-0.158
-0.137	0.021	-0.103	0.124	-0.033	-0.051	-0.221
0.198	-0.066	-0.15	-0.241	-0.292	-0.545	-0.757
0.484	-0.333	0.008	-0.348	0.422	-0.654	0.319
0.047	-0.125	0.077	-0.196	-0.04	0.025	0.076
0.207	-0.167	-0.095	-0.362	0.002	0.378	-0.427
0.102	-0.072	-0.207	0.271	-0.028	-0.338	0.152
0.169	-0.158	-0.059	-0.302	-0.055	-0.195	0.83
-0.026	0.022	-0.189	-0.439	0.332	-0.086	-0.796
-0.174	0.003	-0.52	-0.218	0.53	1.351	0
-0.076	-0.066	0.218	-0.147	-0.275	0.284	0.13
0.016	0.022	0.019	-0.023	-0.148	-0.368	-0.211
0.054	0.015	-0.066	-0.006	0.065	-0.101	-0.187
-0.106	-0.01	0.134	-0.097	-0.234	0.321	0.117
-0.226	-0.052	0.238	-0.064	-0.201	0.275	-0.168
0.007	-0.165	0.208	-0.259	0.238	-0.307	0.121
0.073	0	-0.362	-0.039	-0.011	-0.076	0.161
0.185	0.367	-0.165	-0.328	-0.14	-0.667	-0.044
-0.115	0.071	-0.177	-0.013	0.15	0.077	-0.853
-0.006	-0.128	-0.139	-0.108	-0.067	0.172	0.291
0.127	0.091	-0.121	-0.02	-0.111	-0.129	-0.118
0.455	-0.34	0.17	-0.223	-0.692	-0.361	-1.156
-0.098	-0.148	-0.141	0.233	-0.464	0.693	-0.177

-0.137	-0.124	-0.055	0.018	0.059	0.059	0.059
0.46	-0.308	0.212	-0.355	-0.59	-0.635	-1.152
0.085	-0.054	-0.113	-0.237	-0.178	0.077	0.202
0.451	-0.057	-0.088	-0.326	-0.334	-0.224	0.256
-0.259	0.224	0.218	-0.308	-0.463	-0.037	0.24
0.21	-0.088	-0.045	0.303	-0.295	-0.108	-0.121
0.043	0.086	-0.263	-0.127	0.191	0.039	0.033
0.168	-0.183	0.009	-0.063	0.32	-0.223	-0.174
0.19	-0.152	-0.001	-0.038	-0.931	0.959	-0.35
-0.205	0.171	-0.001	-0.158	-0.515	0.13	0.209
0.079	-0.149	0.142	-0.14	0.073	-0.091	-0.156
0.001	0	-0.305	0.223	0.135	0.054	-0.138
-0.02	0.1	-0.078	-0.169	-0.043	0.104	-0.417
-0.009	0.004	-0.061	-0.094	-0.095	-0.162	-0.326
0.008	-0.056	-0.435	0.004	0.296	-0.26	0.07
0.054	-0.03	-0.482	-0.116	0.315	-0.208	0.091
-0.144	0.161	0.218	-0.235	-0.265	-0.007	0.112
-0.167	0.364	0.07	-0.27	-0.364	-0.283	-0.146
-0.037	-0.145	-0.255	0.083	0.083	0	0
-0.167	0.364	0.07	-0.27	-0.364	-0.283	-0.146
0.519	-0.124	0.056	-0.265	-0.101	-1.183	0.26
0.046	0.178	-0.181	0.032	0.217	-0.269	0.075
-0.117	-0.001	-0.242	0.095	0.208	0.058	-0.172
-0.117	-0.001	-0.242	0.095	0.208	0.058	-0.172
0.066	-0.139	0.274	-0.155	-0.227	-0.588	-0.076
0.099	-0.168	0.076	-0.147	0.116	-0.115	0.418
0.461	-0.009	-0.362	-0.278	-1.264	-0.012	0.771
0.215	-0.286	0.298	-0.182	0.153	-0.207	0
-0.064	-0.228	-0.04	0.339	-0.057	-0.286	-0.166
0.156	-0.093	-0.188	-0.072	-0.175	-0.137	0.333
0.007	0.166	-0.088	-0.095	0.004	-0.148	-0.15
0.082	0.165	-0.277	-0.351	-0.2	0.118	0.17
0.136	-0.405	0.012	-1	0	0	0
0.007	-0.104	0.034	-0.162	-0.132	0.115	0.015
0.127	0.017	-0.069	-0.27	0.372	-0.193	-0.177
0.147	0.071	-0.116	-0.032	-0.245	0.092	0.41
0.072	0.019	-0.154	0.144	0.039	-0.049	-0.282
0.184	-0.118	-0.21	-0.014	0.073	-0.115	0.049
0.229	-0.168	-0.08	-0.082	0.17	-0.088	0.036
0.096	0.027	-0.011	-0.56	0.301	-0.033	-0.049
0.22	-0.244	0.005	-0.161	0.097	-0.049	0.182
0.096	0.011	-0.041	-0.104	-0.102	0.33	-0.031
0.177	-0.074	-0.008	-0.593	0.644	-0.041	-0.21
0.269	-0.028	-0.062	0.004	-0.245	0.186	-0.413
0.167	-0.256	-0.066	-0.023	-0.022	-0.077	0.739
0.089	-0.062	0.128	-0.199	-0.091	0.162	-0.127
0.182	-0.039	-0.281	-0.373	-0.404	0.11	0.075
0.19	0.335	-0.177	-0.31	-0.096	-0.582	-0.001
-0.04	0.209	0.056	-0.211	0.241	-0.276	0.366
0.231	0.058	-0.474	0.21	-0.132	0.048	-0.004

0.001	0	-0.305	0.223	0.135	0.054	-0.138
-0.211	-0.075	-0.175	0.087	0.567	0.012	-0.613
-0.008	-0.045	-0.025	-0.116	-0.127	-0.32	-0.526
-0.037	-0.113	-0.017	0.094	-0.055	-0.09	-0.008
0.375	-0.444	0.322	-0.294	0	0	0
-0.056	-0.108	-0.205	0.032	0.085	0.394	0.714
-0.017	0.007	-0.489	-0.017	-0.043	-0.1	0.314
-0.006	0.084	0.016	-0.006	-0.112	-0.119	-0.169
-0.106	0.11	-0.124	-0.324	0.245	0.022	-0.252
0.397	-0.219	0.03	0.05	-0.289	-0.069	-0.144
0.503	-0.403	0.264	-0.171	-0.238	-0.033	-0.128
-0.079	-0.177	0.003	0.087	-0.182	0.355	-0.394
0.216	0.028	-0.167	-0.013	0.201	-0.243	-0.049
0.143	0.143	-0.058	-0.238	-0.078	-0.306	-0.381
0.09	0.008	-0.068	-0.206	-0.376	0.432	-0.07
0.167	-0.181	0.389	-0.237	0.048	-0.208	0.348
0.375	-0.25	-0.306	-0.583	0.25	0	0
0.288	0.024	-0.284	-0.047	-0.206	-0.216	-0.217
0.123	0.087	-0.116	-0.029	-0.108	-0.137	-0.124
-0.074	0.139	-0.179	-0.13	0.069	0.048	0.035
0.082	-0.006	0.111	-0.157	-0.011	-0.041	0.028
0.053	0.199	-0.153	-0.243	0.038	-0.147	0.104
0.064	-0.172	0.04	-0.286	0.15	-0.12	0.024
-0.047	-0.15	0.089	-0.038	-0.143	0.001	-0.118
0.137	-0.392	0.188	-0.144	0.078	-0.067	-0.376
0.124	-0.073	-0.087	0.003	-0.327	0.072	0.042
0.167	-0.215	0.195	0.049	-0.223	-0.202	0.052
0.109	0.043	0.026	-0.202	-0.272	0.512	-0.044

GATS1m	GATS2m	GATS3m	GATS4m	GATS5m	GATS6m	GATS7m
0.8	0.788	0.986	0.986	0.756	1.511	1.133
0.832	1.046	0.992	1.073	1.108	1.375	0.931
0.555	0.71	1.014	1.06	1.174	1.074	1.002
0.676	0.85	0.946	1.208	1.114	1.981	1.486
1.217	0.656	0.976	0.877	1.101	0.953	1.191
2.061	0.785	1.218	0.565	0.964	0.051	0
0.893	0.65	0.964	1.186	0.812	1.175	1.063
0.931	0.75	1.619	0.582	0	0	0
0.466	0.64	0.576	0.609	0.848	2.073	2.747
0.869	1.168	0.957	0.998	1.469	1.638	1.025
1.042	1.183	0.951	1.07	0.891	0.932	1.025
0.491	0.719	0.948	0.99	1.031	1.065	2.082
1.37	0.339	0.3	0.328	0.724	1.826	3.316
0.576	0.765	0.951	0.867	1.037	1.387	1.005
0.986	1.518	0.925	1.1	0.809	0.976	1.098
0.976	1.526	0.92	1.083	0.796	1.004	1.152
0.714	0.893	0.937	1.588	1.003	0.891	0
0.729	0.552	0.901	0.944	1.098	0.519	0.677
0.847	1.023	0.766	1.024	1.559	1.633	0.494
0.775	0.703	1.084	1.537	1.671	1.278	0.691
0.819	0.642	1.155	1.155	1.306	1.198	0.847
1.008	1.236	2.319	1.602	0.116	0.084	0.129
0.505	0.677	0.726	0.487	0.534	0.81	1.322
0.967	1.535	0.972	1.116	0.837	0.655	1.197
1.161	1.041	1.272	1.168	1.443	0.993	1.097
0.546	0.76	0.988	1.092	1.269	1.035	0.913
0.932	1.162	1.056	0.974	1.462	1.334	0.325
0.663	0.569	0.735	0.664	0.859	0.871	0.848
1.182	1.105	0.86	1.14	1.177	0.901	0.772
1.065	1.253	0.718	1.075	1.455	1.061	0.998
1.422	1.025	1.187	1.214	1.355	1.968	1.483
0.453	0.542	0.723	0.688	0.953	0.799	0.337
0.82	1.131	0.991	1.12	1.522	1.791	0.632
0.782	0.791	0.986	1.772	2.054	1.034	0.521
0.762	0.876	0.971	1.09	1.017	1.113	0.788
0.906	0.621	1.113	1.552	1.201	0.988	0.608
0.568	0.826	0.983	1.037	1.149	1.396	0.684
1.11	0.456	1.33	0.967	1.093	3.496	0
0.503	0.783	0.658	0.61	0.614	0.565	0.806
0.736	0.696	0.981	1.056	0.906	1.009	1.013
0.729	0.519	0.956	0.988	1.255	0.725	1.113
0.666	0.356	0.807	1.18	1.847	1.511	0
0.613	0.453	0.66	0.712	0.73	0.94	1.08
0.48	0.632	0.65	0.803	1.393	1.298	0.953
0.959	0.854	0.769	1.211	1.163	0.857	1.428
0.751	0.878	0.792	1.156	0.977	0.749	0.902
0.986	0.977	1.213	1.041	1.228	0.98	1.124
1.025	1.002	1.473	0.911	1.4	1.045	1.148
1.091	0.957	1.214	0.922	1.297	0.851	1.188

1.036	0.968	0.879	0.924	1.3	1.088	0.971
1.088	1.063	0.908	1.003	0.976	0.889	1.163
1.019	0.966	1.347	0.851	1.225	0.952	1.03
1.098	1.063	0.926	0.935	1.081	1.149	1.005
1.092	1.094	1.001	1.019	0.924	1.075	0.778
0.98	0.99	1.206	1.278	1.427	0.892	1.089
1.222	0.858	1.208	1.069	1.369	0.908	1.346
1.04	0.992	0.873	0.974	1.18	0.994	1.406
1.085	1.061	0.758	0.886	1.032	0.996	1.192
1.105	1.04	1.155	0.991	1.264	1.014	1.09
1.067	1.048	1.331	1.108	1.341	1.324	0.974
1.315	0.629	0.723	0.499	0.503	0.944	1.421
0.976	0.975	1.214	1.025	1.215	1.009	1.184
1	1.058	0.916	0.937	1.101	1.088	0.816
1.064	1.118	0.973	0.933	0.984	1.143	0.659
0.976	0.975	1.214	1.025	1.215	1.009	1.184
0.954	0.752	0.417	0.883	1.307	1.447	1.165
0.521	0.744	0.699	0.411	0.378	0.684	1.818
1.357	0.885	0.891	1.02	1.322	0.821	0.096
0.495	0.745	0.775	0.43	0.396	0.816	1.602
0.703	1.002	1.087	0.87	0.64	1.01	1.168
1.073	1.027	1.19	0.737	0.338	1.208	0.602
0.693	0.989	1.101	0.898	0.634	1.005	1.161
0.955	0.662	0.665	0.935	0.755	1.285	1.771
0.492	0.667	0.685	0.453	0.578	1.123	2.487
1.092	0.902	1.325	2.258	1.131	1.639	0.757
1.016	0.714	1.004	1.216	0.979	1.071	0.546
0.53	0.39	0.751	0.872	1.036	0.724	0.742
0.921	0.806	0.87	0.728	1.198	1.642	1.248
0.663	0.772	0.886	0.937	1.198	1.529	1.134
0.784	0.726	0.748	0.937	1.296	1.118	1.448
0.561	0.775	0.85	0.637	0.472	0.981	4.303
0.519	0.681	0.632	0.387	0.427	0.694	0.892
0.534	0.79	1.318	1.13	1.018	1.574	0
0.477	0.653	0.593	0.621	0.59	1.044	2.026
0.937	0.874	0.684	0.442	1.539	1.915	3.351
1.547	1.361	1.412	0.985	0.938	0.036	0.042
1.199	1.092	1.242	0.341	0.565	1.005	2.111
0.546	0.76	0.988	1.092	1.269	1.035	0.913
0.482	0.668	0.586	0.666	0.958	2.21	2.11
0.531	0.776	1.095	0.884	0.882	1.425	1.407
0.677	0.953	0.967	0.913	0.941	1.043	0.836
1.041	0.82	0.949	0.898	0.949	0.973	1.405
0.602	0.755	0.999	0.901	0.988	1.43	1.333
0.615	0.598	0.724	0.673	1.214	1.199	1.068
0.88	0.895	0.862	0.832	0.788	1.026	1.035
0.997	1.356	1.821	1.61	1.008	0.515	1.027
1.094	1.033	1.407	2.16	0.829	0.363	0.444
0.77	0.723	0.857	1.377	1.142	1.19	1.316
0.83	0.821	0.937	1.251	1.174	1.04	1.008

1.59	0.431	0.927	0.996	0.57	0.549	0.698
0.496	0.637	0.682	0.712	0.859	0.886	1.087
0.668	0.774	1.008	1.066	1.018	1.164	1.033
0.592	0.8	1.077	0.955	0.955	1.074	0.983
0.808	0.876	0.699	1.312	1.168	0.437	0.874
0.734	0.772	0.667	0.716	1.062	1.467	1.339
1.047	0.876	0.798	0.908	0.919	1.129	1.056
1.436	0.868	1.394	1.004	0.476	1.514	1.099
0.534	0.548	0.713	0.848	0.563	1.188	1.644
0.716	0.816	1.133	1.19	1.074	0.425	0.567
0.83	0.832	1.182	1.25	1.343	0.772	0.854
0.711	0.993	1.09	1.113	0.749	0.683	0.606
0.6	0.521	0.736	1.26	0.923	0.811	1.26
0.79	0.616	0.323	0.715	1.018	1.324	1.04
0.694	0.878	1.143	1.008	0.671	1.02	0.99
0.503	0.693	0.934	0.878	0.946	1.153	1.441
0.787	0.737	0.807	0.892	0.691	1.932	1.233
0.882	0.914	1.071	1.411	0.739	0	0
0.899	1.143	1.363	1.044	0.781	0.941	0.638
0.529	0.621	0.97	1.403	1.699	0.946	0.441
2.387	0.742	0	0	0	0	0
1.041	0.653	0.575	0.663	0.848	0.733	0.548
1.031	0.774	0.839	1.297	1.025	0.843	0.955
0.627	0.559	0.643	0.938	1.178	1.177	1.349
0.596	0.447	0.584	1.652	1.887	3.179	0
0.899	0.861	0.831	0.938	0.968	1.406	1.534
1.157	0.76	0.986	0.865	0.869	1.074	1.012
0.756	0.68	1.417	1.063	1.214	0	0.523
1.064	1.161	1.053	1.18	0.999	0.834	0.99
1.411	0.983	1.221	0.861	1.456	1.46	0.957
0.957	0.901	0.992	0.826	1.182	1.213	0.736
0.79	0.856	0.833	1.377	2.064	1.411	0.966
0.596	0.748	0.844	0.625	0.502	0.706	0.954
1.357	0.885	0.891	1.02	1.322	0.821	0.096
0.713	0.8	0.746	1.108	1.164	1.77	1.561
0.538	0.404	0.85	1.01	1.346	1.154	1.346
0.998	0.73	1.131	1.375	1.242	0.75	0.908
0.519	0.674	0.649	0.404	0.404	0.636	0.889
0.612	0.483	0.993	1.172	1.236	0.772	0.374
0.592	0.608	1.215	1.253	1.102	1.076	0.915
0.962	0.955	0.982	1.55	0.642	1.854	0
0.964	1.025	0.813	1.458	0.556	1.7	0
0.803	0.74	0.925	1.121	1.179	1.063	0.947
0.505	0.721	1.056	1.248	0.928	0.65	0.8
0.534	0.528	1.018	1.188	1.134	1.188	0.509
1.068	0.991	0.658	1.014	1.644	0.983	1.117
1.192	0.854	1.176	1.846	1.518	0.749	0.675
0.793	0.594	0.934	1.189	1.019	1.045	0.815
0.786	0.737	0.995	1.291	1.309	0	0
0.768	0.779	0.875	1.028	1.273	1.385	1.04

0.838	0.791	0.619	1.581	2.603	1.055	0.122
0.87	0.803	0.858	1.209	1.386	1.044	0.799
0.676	0.78	0.931	1.235	1.323	1.459	0.688
0.512	0.704	0.761	0.839	0.98	1.701	1.82
0.485	0.638	0.954	0.903	0.688	1.15	1.557
1.3	1.29	0.849	0.85	0.618	0.962	1.541
0.568	0.453	0.366	0.917	1.443	1.335	1.068
0.882	0.601	1.116	1.571	0.93	1.249	0.28
1.296	0.903	0.98	1.171	0.815	1.118	0.466
0.789	0.704	0.924	1.889	2.123	0.783	0.104
0.523	0.661	1.271	2.011	0	0	0
1.8	0	1.8	0	1.8	0	0
0.697	0.723	1.04	1.276	1.266	1.253	0.24
1.019	1.531	0.909	0.94	0.756	1.052	1.446
0.881	0.647	0.794	0.974	0.96	0.729	1.066
0.532	0.638	0.669	0.919	1.222	1.003	1.227
0.564	0.823	1.046	0.867	1.12	1.237	0.723
1	1.014	0.74	1.007	1.5	1.497	0.432
0.667	0.751	0.622	1.487	2.165	0	0
0.479	0.648	0.595	0.611	0.841	1.884	2.671
0.679	0.839	1.256	1	1.286	1.051	0.862
0.713	0.8	0.746	1.108	1.164	1.77	1.561
0.712	0.751	0.867	1.018	1.214	1.384	1.252
1.036	0.967	0.967	1.367	0.793	0.815	1.001
0.754	0.956	0.865	1.122	1.364	1.498	1.067
0.951	1.463	0.855	0.931	0.683	0.893	1.438
0.739	0.662	1.079	0.601	1.289	1.692	2.083
0.689	0.925	1.069	1.068	0.932	1.291	0.362
0.712	0.92	0.975	1.168	0.925	1.196	0.809
0.72	0.518	0.747	0.971	1.214	1.349	2.024
1.15	0.505	1.428	0.911	1.391	0.527	1.646
0.457	0.597	0.517	0.55	0.564	0.413	0.472
0.737	0.536	0.911	1.036	1.22	1.186	1.061
1.477	0.452	0.883	1.153	0.805	1.257	1.543
0.727	0.606	0.918	1.015	1.165	1.045	1.335
0.856	0.898	0.898	1.083	1.333	0.733	0.963
0.668	0.964	1.002	1.357	1.234	0.925	0.614
0.694	0.755	0.902	0.979	1.295	1.498	0.935
0.909	0.345	0.294	0.323	0.714	1.905	4
0.931	0.877	1.004	1.075	1.039	0.962	0.985
0.768	0.779	0.875	1.028	1.273	1.385	1.04
1.266	0.911	1.239	1.841	1.675	1.331	0.59
0.862	0.738	1.131	1.021	1.592	1.118	0.863
0.95	1.467	0.905	0.977	0.708	0.93	1.502
0.49	0.677	0.601	0.709	0.929	1.742	2.647
0.816	0.852	0.859	1.17	1.026	1.292	1.178
0.625	0.79	1.145	1.145	1.113	1.099	0.78
0.895	0.705	0.612	0.957	0.989	0.996	1.045
0.751	0.456	1.19	1	1.039	1.833	1.499
0.913	1.06	0.848	0.918	0.847	1.526	1.06

1.35	1.667	1.552	0.964	0.346	0	0
0.695	0.42	1.16	0.97	1.216	1.719	2.083
0.626	0.768	1.269	1.373	1.434	1.303	0.474
0.635	0.407	0.937	1.077	1.406	1.603	1.479
0.895	1.105	0.966	1.061	1.46	1.745	1.063
1.224	1.352	1.084	0.873	0.591	1.216	1.178
0.917	0.741	0.861	1.484	1.46	0.903	0.591
1.423	0.716	0.975	1.24	1.446	1.088	0.552
0.507	0.706	1.046	1.586	2.052	1.226	0.015
0.891	1.094	0.806	0.98	1.336	1.633	0.777
0.697	0.754	0.94	0.862	0.921	0.881	0.965
1.136	0.861	0.845	1.231	0.665	0.649	0.833
0.534	0.645	0.682	0.974	1.253	1.032	1.161
0.463	0.574	0.502	0.861	1.039	1.048	1.417
0.852	0.827	1.102	1.627	1.113	1.223	1.326
0.956	0.822	1.111	1.902	1.483	0.936	0.931
0.753	1.019	1.053	1.086	1.387	1.424	1.204
0.82	1.036	0.843	1.17	1.541	1.718	1.256
0.5	0.722	1.368	2.031	0	0	0
0.82	1.036	0.843	1.17	1.541	1.718	1.256
0.502	0.703	0.691	0.387	0.489	1.47	3.817
0.95	1.365	1.703	0.964	0.676	0.746	0.475
0.852	0.798	0.87	1.39	1.229	0.902	0.885
0.852	0.798	0.87	1.39	1.229	0.902	0.885
1.071	0.908	1.206	1.444	1.568	1.879	2.487
0.764	0.728	0.941	0.95	0.931	0.904	0.895
1.055	1.077	0.942	1.041	1.023	1.257	0.546
1.743	0.676	1.107	0.928	1.124	0.808	0.226
0.65	0.798	0.939	0.905	0.68	0.691	1.147
1.152	0.676	0.811	0.991	0.991	1.206	1.192
0.868	0.737	0.563	0.828	1.057	0.851	1.072
0.784	0.784	0.975	1.691	2.13	1.131	0.096
0.643	0.659	1.25	1.339	2.143	0	0
0.852	0.742	1.036	1.43	1.347	1.165	0.676
1.418	1.282	1.206	0.918	1.086	0.969	1.284
1.557	0.439	0.927	0.977	1.026	1.002	0.64
0.972	1.055	0.403	0.347	0.643	0.762	0.577
1.505	1.076	1.598	1.325	1.024	0.113	0.094
1.415	1.019	1.4	1.497	1.168	0.962	0.647
1.176	1.015	1.008	1.041	0.954	0.713	1.604
1.496	1.042	1.388	1.849	0.97	0.104	0.056
1.16	0.679	0.662	0.441	0.512	0.583	1.042
1.499	0.745	0.793	0.856	1.013	0.906	0.617
1.479	0.705	0.805	0.91	0.945	1.42	0.666
0.97	1.057	0.668	0.916	1.125	0.811	1.008
1.492	0.813	0.993	1.144	0.571	1.042	0.644
0.742	0.588	0.941	1.228	1.403	1.628	0.863
0.494	0.665	0.573	0.65	0.913	2.005	2.401
1.474	0.305	0.431	0.999	0.595	1.486	1.863
1.306	1.214	0.8	0.93	0.812	0.849	1.201

1.136	0.861	0.845	1.231	0.665	0.649	0.833
0.661	0.919	1.032	1.332	1.227	0.205	0.671
0.461	0.567	0.754	0.65	1.105	1.163	2.126
0.668	0.806	1.008	1.066	0.987	1.111	1.111
1.092	0.484	1.426	0.794	1.694	0	0
0.558	0.604	0.654	0.769	1.289	2.749	3.991
0.69	0.855	1.03	0.76	0.963	1.029	1.522
0.886	0.804	0.649	1.409	1.402	1.337	0.646
0.89	1.039	0.991	1.097	1.247	0.974	0.768
1.476	1.084	1.241	0.891	1.616	1.146	1.341
1.557	1.159	1.452	0.973	1.207	0.729	0.909
0.879	0.878	1.171	1.004	0.998	1.11	0.639
0.497	0.684	0.72	0.826	1.048	1.16	0.555
0.485	0.66	0.726	0.738	0.906	1.761	2.015
1.213	0.801	0.89	1.186	0.961	1.202	0.844
1.066	0.675	1.159	0.69	1.03	0.71	0.969
0.625	0.563	1.125	1.25	1.875	0	0
0.586	0.536	0.79	1.3	1.066	1.073	1.141
0.867	0.719	0.629	0.921	1.012	0.973	1.066
1.132	0.813	0.719	0.987	0.778	0.939	0.855
0.877	0.623	0.817	0.831	1.274	1.051	1.035
0.871	0.695	0.581	0.968	1.506	1.556	1.295
0.666	0.761	1.051	1.11	1.366	1.236	0.908
0.833	0.87	0.928	0.865	0.854	1.047	1.019
0.967	0.796	1.257	0.858	1.115	0.909	1.009
0.642	0.796	1	1.187	1.168	0.984	0.952
0.894	0.672	1.137	0.698	1.169	1.313	1.394
0.519	0.703	0.712	0.442	0.49	0.904	1.33

GATS8m	GATS1v	GATS2v	GATS3v	GATS4v	GATS5v	GATS6v
1.511	0.8	0.788	0.986	0.986	0.756	1.511
0.707	0.866	1.071	0.991	1.053	1.062	1.36
1.027	0.538	0.716	1.014	1.051	1.164	1.076
0	0.717	0.873	0.974	1.201	1.011	1.889
2.225	1.337	0.654	1.052	0.797	1.127	0.881
0	1.512	0.826	1.25	0.612	1.37	0.213
0.733	0.937	0.713	0.937	1.13	0.759	1.165
0	1.023	0.782	1.608	0.391	0	0
1.416	0.898	0.98	0.882	1.013	1.159	0.832
0.61	0.889	1.188	0.913	0.923	1.424	1.632
0.971	1.062	1.197	0.946	1.064	0.864	0.944
2.243	0.617	0.812	1.206	0.968	1.337	1.007
1.5	1.37	0.339	0.3	0.328	0.724	1.826
1.132	0.973	0.707	0.816	1.011	1.113	1.25
1.153	0.746	0.693	0.745	1.628	1.186	0.764
1.148	0.736	0.643	0.706	1.754	1.243	0.764
0	0.782	1.015	0.937	1.48	0.912	0.763
0.664	0.849	0.633	0.931	1.093	1.201	0.557
0.706	0.865	1.021	0.745	0.991	1.535	1.629
0.607	0.813	0.742	1.061	1.467	1.634	1.238
0	0.913	0.683	1.177	1.177	1.234	1.096
0.194	0.907	0.708	0.921	1.248	1.172	0.863
1.763	0.752	0.692	1.081	1.191	1.128	0.977
1.255	0.716	0.612	0.706	1.8	1.249	0.758
0.822	1.222	1.13	1.222	1.227	1.453	1.017
1.002	0.553	0.721	0.865	1.05	1.266	1.005
0.745	0.945	1.181	1.023	0.92	1.434	1.342
0.789	0.708	0.626	0.789	0.698	0.946	0.867
0.868	1.182	1.105	0.86	1.14	1.177	0.901
0.73	1.075	1.268	0.703	1.032	1.436	1.078
1.084	1.066	0.823	0.835	1.184	1.088	1.027
0.366	0.736	0.711	1.057	1.143	1.079	1.056
0.118	0.839	1.157	0.944	1.067	1.505	1.77
0	0.872	0.868	1.021	1.703	1.889	1.069
1.069	0.786	0.888	0.987	1.094	1.013	1.094
1.057	1.022	0.644	1.098	1.488	1.07	1.074
0.481	0.619	0.854	0.994	1.064	1.146	1.347
0	1.289	0.451	1.309	0.932	0.972	3.072
1.279	0.503	0.783	0.658	0.61	0.614	0.565
1.345	0.779	0.734	1.003	1.058	0.915	1.02
1.408	0.735	0.528	0.991	1.014	1.214	0.78
0	0.726	0.373	0.88	1.16	1.751	1.433
1.209	0.613	0.453	0.66	0.712	0.73	0.94
1.165	0.588	0.51	0.796	0.918	1.148	0.675
1.384	0.974	0.883	0.75	1.216	1.139	0.888
1.116	0.736	0.673	0.709	1.58	1.394	0.814
1.168	0.746	0.724	0.747	1.473	1.316	0.807
0.714	0.709	0.735	0.801	1.469	1.422	1.003
0.892	0.745	0.809	0.705	1.36	1.184	0.921

0.938	0.88	0.761	0.765	1.176	1.246	0.934
1.131	0.837	0.825	0.673	1.064	1.333	0.942
0.739	0.806	0.736	0.848	1.348	1.078	1.094
0.95	0.914	0.825	0.814	1.183	1.273	0.846
1.167	0.914	0.618	0.798	1.365	1.197	0.889
1.152	0.731	0.724	0.732	1.397	1.313	0.961
1.143	0.848	0.605	0.879	1.177	1.09	1.136
0.974	0.829	0.866	0.866	1.033	1.118	1.001
1.028	0.848	0.818	0.799	1.222	1.425	0.709
0.865	0.874	0.832	0.714	1.16	1.393	0.868
1.532	0.987	0.776	0.827	1.129	1.292	0.962
1.687	0.729	0.559	0.738	0.771	0.864	1.179
1.164	0.736	0.673	0.709	1.58	1.394	0.814
1.111	0.761	0.667	0.757	1.52	1.181	0.862
1.248	0.808	0.684	0.765	1.406	1.164	0.84
1.164	0.736	0.673	0.709	1.58	1.394	0.814
0.506	0.862	0.679	0.926	1.893	1.882	1.399
2.379	0.914	0.74	0.187	0.76	0.662	0.276
0	1.045	0.503	1.052	1.214	1.789	0.474
3.386	1.257	0.774	0.805	1.093	1.006	1.467
1.527	1.379	1.018	1.246	0.557	0.765	0.875
1.198	0.935	0.422	1.366	1.114	1.519	1.45
1.518	1.3	0.394	0.406	0.393	0.731	1.606
1.745	0.75	0.545	1.04	1.23	1.26	0.698
2.775	0.8	0.901	1.156	1.09	0.942	1.143
0.035	1.262	0.496	1.324	0.757	1.23	0.902
0.903	1.034	0.711	1.021	1.19	0.968	1.024
0.358	0.565	0.436	0.781	0.931	1.133	0.811
1.248	0.94	0.867	0.89	0.703	1.166	1.593
0.48	0.79	0.852	0.89	0.766	1.117	1.499
0.802	0.837	1.016	0.889	1.151	1.081	0.924
4.844	0.788	0.965	0.919	0.689	1.149	1.379
2.345	0.776	0.692	0.602	0.916	1.339	1.24
0	1.103	0.552	1.298	0.985	1.697	0.552
3.329	1.28	1.08	1.137	1.295	0.72	0.832
0	1.58	0.51	1.492	1.059	0.954	0
0.067	0.715	0.775	1.32	1.566	1.172	0.34
1.319	1.199	1.092	1.242	0.341	0.565	1.005
1.002	0.553	0.721	0.865	1.05	1.266	1.005
2.563	0.834	0.699	0.842	1.114	1.146	1.022
1.471	0.617	0.586	0.778	0.956	0.792	0.807
1.048	0.704	0.713	0.77	1.375	1.142	1.114
2.569	1.137	0.812	0.995	0.851	0.976	0.954
2.288	0.617	0.722	1.012	0.895	1.032	1.466
1.145	0.615	0.598	0.724	0.673	1.214	1.199
0.976	0.88	0.895	0.862	0.832	0.788	1.026
0.142	0.967	0.765	0.75	1.185	1.026	1.197
0.363	1.14	0.975	1.241	1.998	0.833	0.511
0.382	0.838	0.772	0.829	1.346	1.115	1.166
0.954	0.836	0.817	0.94	1.251	1.17	1.038

0.71	1.065	0.751	0.689	1.128	1.023	0.945
1.152	0.981	0.58	1.14	1.109	1.478	0.652
0.994	0.693	0.771	1.005	1.055	0.999	1.176
0.576	0.621	0.825	1.08	0.937	1	1.149
1.749	0.879	0.995	0.655	1.278	1.269	0.426
2.812	0.752	0.804	0.712	0.757	0.998	1.415
1.114	1.047	0.876	0.798	0.908	0.919	1.129
1.13	1.114	0.399	0.999	0.843	1.036	1.274
1.425	0.534	0.548	0.713	0.848	0.563	1.188
1.36	0.716	0.816	1.133	1.19	1.074	0.425
0.98	0.941	0.926	1.211	1.156	1.293	0.811
0.86	0.579	0.579	0.643	1.118	0.839	1.161
1.714	0.646	0.557	0.754	1.234	0.915	0.818
0.627	0.912	0.752	0.471	0.856	1.034	1.393
1.478	0.871	0.94	1.132	1.106	0.856	0.974
3.043	0.509	0.702	1.071	1.113	1.155	1.034
1.263	0.951	0.774	0.889	0.987	0.704	1.789
0	1.098	0.84	1.083	1.28	0.086	0
1.056	0.749	0.576	0.677	0.804	0.66	1.235
0.881	0.536	0.557	0.914	1.259	1.524	0.803
0	1.873	1.085	0	0	0	0
0.648	0.581	0.595	0.521	0.538	0.607	0.74
1.738	0.924	0.686	0.869	1.156	1.401	0.72
1.146	0.627	0.559	0.643	0.938	1.178	1.177
0	0.603	0.452	0.64	1.67	1.768	3.028
2.499	0.93	0.858	0.836	0.955	0.97	1.362
2.05	1.272	0.755	1.048	0.821	0.888	1.057
0.907	0.756	0.68	1.417	1.063	1.214	0
0.948	1.074	1.167	1.02	1.152	0.97	0.862
1.004	0.876	0.414	1.29	1.201	1.418	1.403
0.556	0.889	0.533	1.228	1.105	1.486	1.254
0.49	0.86	0.943	0.84	1.27	1.923	1.292
1.193	0.819	0.708	0.923	1.332	0.876	0.8
0	1.045	0.503	1.052	1.214	1.789	0.474
3.749	0.748	0.809	0.751	1.115	1.161	1.714
1.346	0.538	0.404	0.85	1.01	1.346	1.154
1.044	1.078	0.762	1.134	1.323	1.245	0.704
2.439	0.776	0.617	0.804	1.123	1.061	1.24
1.607	0.656	0.515	1.017	1.185	1.215	0.804
0.802	0.609	0.626	1.152	1.226	1.11	1.055
0.543	0.879	0.981	1.044	1.534	0.715	1.809
0	0.913	1.034	0.844	1.438	0.615	1.648
1.145	0.795	0.779	0.946	1.084	1.193	1.056
1.419	0.53	0.519	0.878	0.897	0.507	1.08
1.425	0.534	0.528	1.018	1.188	1.134	1.188
0.191	1.025	0.75	1.201	0.922	1.066	1.124
0.698	0.832	0.76	0.507	0.767	1.168	1.426
1.279	0.793	0.594	0.934	1.189	1.019	1.045
0	0.859	0.766	0.949	1.275	1.261	0
0.472	0.914	0.859	0.88	0.918	1.154	1.318

0	0.937	0.854	0.634	1.5	2.399	1.152
0.849	1.031	0.834	0.788	1.066	1.206	1.06
0.333	0.81	0.854	0.905	1.017	1.23	1.345
1.935	0.68	0.615	0.579	1.128	1.206	1.388
0.689	0.633	0.659	0.756	1.071	1.029	1.102
1.175	0.885	0.656	1.109	1.506	0.927	1.123
1.049	0.758	0.732	0.685	0.558	0.951	1.209
0	0.93	0.616	1.079	1.534	0.893	1.288
0	1.296	0.903	0.98	1.171	0.815	1.118
0	0.881	0.759	0.941	1.791	1.943	0.854
0	1.222	0.748	0.946	1.284	0	0
0	1.8	0	1.8	0	1.8	0
0	0.78	0.727	0.971	1.238	1.248	1.283
1.512	0.914	0.711	0.891	1.275	1.19	0.864
1.073	0.974	0.705	0.833	0.981	0.981	0.697
1.679	0.532	0.638	0.669	0.919	1.222	1.003
0.653	1.048	0.887	1.076	1.04	1.544	0.905
0.936	1.015	1.04	0.72	0.952	1.473	1.507
0	0.758	0.838	0.601	1.439	1.971	0
1.256	0.8	0.891	0.955	0.819	1.053	1.005
0.454	0.545	0.686	0.593	1.096	0.661	0.438
3.749	0.748	0.809	0.751	1.115	1.161	1.714
0.516	0.829	0.827	0.886	0.938	1.12	1.294
0.375	1.072	0.951	0.919	1.337	0.804	0.856
0.696	0.772	0.983	0.845	1.08	1.346	1.502
1.657	0.772	0.682	0.767	1.436	1.196	0.934
3.125	0.81	0.718	1.129	0.58	1.235	1.54
1.185	0.722	0.941	1.072	1.07	0.929	1.28
1.349	0.747	0.937	0.972	1.168	0.927	1.158
2.59	0.72	0.518	0.747	0.971	1.214	1.349
0	1.267	0.471	1.376	0.897	1.364	0.492
1.022	0.882	0.69	1.016	0.876	1.233	0.985
1.51	0.735	0.578	0.932	1.012	1.239	1.158
0.748	0.731	0.816	1.035	1.165	0.7	0.843
0.79	0.725	0.646	0.931	0.984	1.174	1.07
1.853	0.905	0.914	0.907	1.112	1.356	0.67
0	0.677	0.957	0.978	1.385	1.294	0.917
0.537	0.829	0.822	0.907	0.802	1.211	1.387
2.5	0.909	0.345	0.294	0.323	0.714	1.905
1.172	0.938	0.866	0.995	1.084	1.041	0.964
0.472	0.914	0.859	0.88	0.918	1.154	1.318
0.119	1.05	0.952	1.036	0.799	1.192	1.256
0.531	0.966	0.761	1.082	0.92	1.448	1.041
1.02	0.714	0.738	0.827	1.45	1.105	1.008
3.4	0.683	0.629	0.604	1.135	1.271	1.367
2.042	0.844	0.86	0.854	1.173	1.027	1.262
0.676	0.666	0.864	1.187	1.18	1.084	1.047
0.964	0.966	0.742	0.616	0.969	0.985	0.982
2.999	0.806	0.411	1.204	1.008	1.04	1.816
1.13	0.938	1.071	0.871	0.911	0.767	1.542

0	1.35	1.667	1.552	0.964	0.346	0
3.125	0.746	0.378	1.172	0.977	1.236	1.701
0	0.675	0.862	1.304	1.387	1.369	1.224
0	0.686	0.402	0.946	1.118	1.394	1.524
0.213	0.93	1.138	0.935	0.952	1.4	1.708
0.811	0.942	0.608	1.046	0.783	0.643	1.163
0.685	1.037	0.803	0.853	1.434	1.339	0.882
0.181	0.885	0.73	1.103	1.001	0.967	0.833
0.123	0.71	0.574	0.868	0.804	1.041	1.927
0.494	0.931	1.124	0.804	0.926	1.26	1.629
0.954	0.697	0.754	0.94	0.862	0.921	0.881
1.203	1.188	0.864	0.902	1.26	0.66	0.699
1.639	0.534	0.645	0.682	0.974	1.253	1.032
2.317	0.463	0.574	0.502	0.861	1.039	1.048
0.29	0.933	0.876	1.031	1.585	1.129	1.067
0.204	0.994	0.874	1.044	1.801	1.402	0.873
0.679	0.77	1.018	1.037	1.031	1.356	1.413
0.684	0.839	1.053	0.804	1.086	1.493	1.71
0	0.5	0.722	1.368	2.031	0	0
0.684	0.839	1.053	0.804	1.086	1.493	1.71
5.797	0.872	0.418	1.006	1.09	1.377	1.09
0.798	0.994	0.8	0.478	0.987	0.665	0.308
1.068	0.901	0.848	0.875	1.358	1.168	0.911
1.068	0.901	0.848	0.875	1.358	1.168	0.911
0.124	0.802	0.95	0.754	0.804	1.06	0.873
0.652	0.805	0.766	0.983	0.986	0.956	0.906
0.422	0.826	0.309	0.772	1.341	1.55	2.064
0	1.286	0.718	1.132	0.611	1.329	0.887
1.096	0.771	0.883	1.083	1.083	0.586	0.819
0.779	1.152	0.676	0.811	0.991	0.991	1.206
1.042	0.926	0.784	0.555	0.84	1.05	0.849
0	0.874	0.86	1.01	1.601	1.959	1.17
0	0.643	0.659	1.25	1.339	2.143	0
0.788	0.881	0.774	1.012	1.381	1.33	1.139
1.02	1.069	0.858	1.05	1.138	1.086	0.723
1.162	0.921	0.782	0.752	1.207	1.23	1.068
1.301	0.984	0.778	1.017	1.314	1.028	1.103
0.07	0.884	0.817	1.154	1.403	1.109	0.928
0.067	0.864	0.75	1.272	1.343	1.282	0.963
1.855	0.677	0.813	1.22	1.335	1.427	0.876
0.038	0.868	0.739	1.341	1.328	1.39	0.849
2.351	0.569	0.559	0.545	0.755	1.132	1.149
1.124	1.017	0.701	1.025	1.06	1.495	0.581
0.814	1.043	0.54	0.84	1.006	0.986	1.266
1.809	0.529	0.517	1.106	1.203	0.977	0.829
0.733	1.061	0.916	0.958	0.938	1.215	0.766
0.724	0.782	0.62	0.949	1.264	1.444	1.645
2.669	0.754	0.615	0.677	1.187	1.243	1.265
1.641	1.425	0.809	0.408	0.845	1.263	0.742
1.205	0.894	0.634	1.155	1.585	0.763	1.139

1.203	1.188	0.864	0.902	1.26	0.66	0.699
2.012	0.69	0.96	1.003	1.336	1.237	0.178
3.158	0.461	0.567	0.754	0.65	1.105	1.163
1.043	0.693	0.801	1.005	1.058	0.97	1.119
0	1.263	0.481	1.397	0.745	1.515	0
7.982	0.757	0.639	1.21	1.429	0.738	1.078
1.637	1.265	0.949	1.375	2.528	0.548	0.045
0.767	1.264	0.998	0.637	1.023	1.321	1.202
1.095	0.9	1.019	0.964	1.085	1.27	0.994
0.131	0.869	0.529	1.083	1.356	1.317	1.844
0.114	0.921	0.421	1.363	1.161	1.491	1.578
1.757	0.926	0.854	1.151	1.001	1.014	1.117
0.678	1.066	0.782	1.037	1.143	0.99	0.772
1.756	0.896	0.964	0.852	1.056	1.11	0.859
1.057	1.215	0.766	0.924	1.128	0.991	1.239
0.463	1.179	0.703	1.148	0.644	1.061	0.759
0	0.625	0.563	1.125	1.25	1.875	0
1.092	0.597	0.558	0.801	1.273	1.078	1.073
0.976	0.925	0.757	0.633	0.937	1.008	0.962
0.724	1.14	0.808	0.734	1.023	0.841	0.952
0.88	0.92	0.666	0.856	0.82	1.269	1.032
0.112	0.937	0.79	0.613	1.007	1.452	1.42
0.512	0.666	0.761	1.051	1.11	1.366	1.236
1.348	0.898	0.938	0.977	0.823	0.849	1.054
1.404	1.007	0.774	1.308	0.834	1.093	0.89
0.625	0.996	0.738	0.868	1.124	0.92	1.138
0.547	1.001	0.719	1.164	0.735	1.154	1.272
3.677	0.69	0.644	0.731	1.125	1.221	1.11

GATS7v	GATS8v	GATS1e	GATS2e	GATS3e	GATS4e	GATS5e
1.133	1.511	0.8	0.788	0.986	0.986	0.756
0.958	0.705	0.828	1.044	0.992	1.074	1.113
1.024	0.993	0.557	0.709	1.013	1.06	1.175
1.417	0	0.672	0.848	0.943	1.208	1.124
1.101	2.174	1.203	0.657	0.968	0.887	1.098
0	0	1.391	0.837	1.222	0.648	1.368
1.089	0.77	0.888	0.644	0.967	1.191	0.818
0	0	0.92	0.747	1.619	0.607	0
1.182	1.203	0.773	0.909	0.818	0.913	0.805
1.119	0.685	0.867	1.166	0.961	1.006	1.473
1.039	0.97	1.04	1.181	0.951	1.07	0.894
1.668	1.418	0.917	1.144	1.285	1.215	1.421
3.316	1.5	1.37	0.339	0.3	0.328	0.724
1.168	0.882	0.972	0.733	0.909	1.099	1.175
0.954	0.83	0.731	0.658	0.767	1.527	1.112
1.119	0.816	0.717	0.595	0.726	1.658	1.167
0	0	0.707	0.881	0.937	1.599	1.012
0.809	0.678	0.78	0.59	0.931	1.022	1.15
0.509	0.737	0.845	1.023	0.768	1.028	1.561
0.72	0.622	0.771	0.7	1.086	1.543	1.674
0.84	0	0.809	0.638	1.153	1.153	1.314
1.206	1.399	0.834	0.609	0.8	1.021	1.29
0.452	0.603	0.74	0.733	1.064	1.098	1.063
1.112	0.759	0.696	0.569	0.72	1.689	1.179
1.056	0.728	1.155	1.031	1.278	1.163	1.441
0.962	1.201	0.552	0.735	0.907	1.066	1.269
0.372	0.75	0.931	1.16	1.059	0.979	1.464
0.857	0.76	0.659	0.564	0.73	0.661	0.851
0.772	0.868	1.182	1.105	0.86	1.14	1.177
1.021	0.735	1.063	1.252	0.72	1.08	1.456
1.394	1.115	0.888	0.854	0.846	1.169	1.038
1.041	0.919	0.753	0.684	1.166	1.281	1.121
0.741	0.173	0.818	1.128	0.995	1.125	1.524
0.526	0	0.772	0.783	0.982	1.779	2.071
0.803	1.039	0.76	0.875	0.97	1.09	1.018
0.693	0.965	0.893	0.62	1.114	1.558	1.214
0.616	0.456	0.563	0.823	0.982	1.034	1.149
0	0	1.091	0.458	1.331	0.971	1.105
0.806	1.279	0.503	0.783	0.658	0.61	0.614
0.955	1.28	0.732	0.692	0.979	1.056	0.905
1.089	1.363	0.729	0.518	0.952	0.986	1.259
0	0	0.659	0.356	0.799	1.182	1.856
1.08	1.209	0.613	0.453	0.66	0.712	0.73
1.614	1.972	0.578	0.534	0.784	0.935	1.142
1.458	1.377	0.957	0.851	0.771	1.21	1.165
1.035	0.899	0.749	0.691	0.746	1.442	1.082
0.88	0.902	0.731	0.684	0.771	1.478	1.131
1.051	0.982	0.737	0.676	0.806	1.568	1.272
1.207	1.01	0.835	0.733	0.799	1.604	1.11

1.133	1.343	0.877	0.659	0.82	1.229	1.078
0.767	1.302	0.868	0.754	0.704	1.123	1.173
1.028	0.775	0.792	0.689	0.876	1.404	0.974
0.924	1.175	0.958	0.754	0.877	1.218	1.118
0.988	0.925	0.963	0.651	0.808	1.339	1.152
0.97	0.917	0.714	0.681	0.753	1.391	1.159
0.979	1.14	0.674	0.592	0.853	1.214	1.062
0.839	1.327	0.829	0.799	0.892	1.055	0.961
0.904	1.17	0.892	0.726	0.89	1.318	1.202
0.906	1.192	0.92	0.741	0.78	1.225	1.177
0.861	1.142	1.002	0.759	0.863	1.104	1.194
1.377	1.451	0.564	0.509	0.726	0.768	0.857
1.035	0.899	0.717	0.618	0.731	1.601	1.193
1.291	1.243	0.748	0.631	0.784	1.461	1.073
1.046	0.965	0.843	0.686	0.818	1.343	1.08
1.035	0.899	0.717	0.618	0.731	1.601	1.193
0.914	0.962	0.853	0.672	0.987	2.055	1.772
0.968	1.302	0.831	0.727	0.264	0.677	0.602
1.848	0	0.628	0.746	1.313	1.227	1.427
0.888	0.629	0.862	0.813	0.845	0.749	0.689
1.456	1.164	0.961	0.931	0.923	0.47	0.954
0.955	0.56	0.638	0.643	1.348	1.219	1.541
2.672	2.135	0.769	0.66	0.66	0.682	0.928
1.387	1.225	0.547	0.657	1.126	1.072	1.173
0.92	0.981	0.767	0.899	1.158	1.032	0.902
1.28	0.768	1.434	0.58	1.155	0.703	0.908
0.561	0.955	1.014	0.715	1.002	1.219	0.981
0.791	0.157	0.539	0.401	0.759	0.887	1.06
1.208	1.208	0.919	0.8	0.869	0.731	1.201
0.952	0.647	0.691	0.773	0.894	0.858	1.148
1.063	0.923	0.847	1.017	0.856	1.082	0.96
1.103	2.758	0.825	1.031	1.008	0.756	0.834
0.631	1.518	0.751	0.694	0.648	0.904	1.27
0	0	0.882	0.863	1.209	1.315	0.507
0.579	0.951	1.006	0.971	0.976	0.923	0.565
0	0	1.349	0.624	1.171	1.417	0.308
0.691	1.106	0.407	0.827	1.466	1.783	1.072
2.111	1.319	1.199	1.092	1.242	0.341	0.565
0.962	1.201	0.552	0.735	0.907	1.066	1.269
1.622	1.554	0.705	0.704	0.793	1.018	0.904
1.183	3.55	0.641	0.699	0.755	1.034	1.007
1.017	0.711	0.747	0.764	0.906	1.292	1.046
1.298	2.437	1.031	0.821	0.944	0.904	0.946
1.379	2.346	0.613	0.737	1.01	0.9	1.018
1.068	1.145	0.615	0.598	0.724	0.673	1.214
1.035	0.976	0.88	0.895	0.862	0.832	0.788
0.924	0.972	0.947	0.785	0.693	1.163	1.022
0.624	0.511	1.089	1.039	1.424	2.175	0.828
1.289	0.485	0.762	0.718	0.86	1.381	1.146
1.008	0.953	0.83	0.822	0.936	1.251	1.174

0.714	0.995	0.648	0.805	0.736	1.226	1.109
1.059	0.864	0.838	0.576	1.1	1.085	1.394
1.029	1.007	0.666	0.775	1.008	1.068	1.019
0.931	0.498	0.59	0.797	1.077	0.957	0.951
0.802	1.604	0.801	0.864	0.705	1.315	1.157
1.292	2.713	0.732	0.769	0.663	0.712	1.068
1.056	1.114	1.047	0.876	0.798	0.908	0.919
1.068	1.476	0.832	0.421	1.044	0.794	1.148
1.644	1.425	0.534	0.548	0.713	0.848	0.563
0.567	1.36	0.716	0.816	1.133	1.19	1.074
0.927	1.032	0.819	0.823	1.179	1.26	1.348
1.568	1.722	0.596	0.569	0.672	0.77	0.855
1.295	1.7	0.596	0.518	0.734	1.262	0.924
1.008	0.493	0.847	0.684	0.384	0.784	1.028
0.842	1.15	0.747	0.919	1.161	1.075	0.758
0.886	2.15	0.507	0.7	1.021	1.026	1.078
1.097	1.186	0.839	0.736	0.848	0.95	0.698
0	0	0.949	0.891	1.092	1.394	0.264
1.22	1.269	0.664	0.496	0.67	0.822	0.695
0.654	1.307	0.534	0.582	0.936	1.316	1.593
0	0	1.837	1.109	0	0	0
0.701	0.851	0.551	0.593	0.499	0.525	0.511
0.879	1.481	0.761	0.805	0.994	1.021	1.357
1.349	1.146	0.627	0.559	0.643	0.938	1.178
0	0	0.596	0.447	0.578	1.65	1.9
1.49	2.411	0.896	0.862	0.83	0.936	0.968
0.907	2.052	1.145	0.76	0.979	0.87	0.868
0.523	0.907	0.756	0.68	1.417	1.063	1.214
0.994	0.961	1.063	1.16	1.057	1.182	1.002
1.211	0.669	0.634	0.463	1.229	1.232	1.43
1.081	0.651	0.692	0.634	1.175	1.15	1.228
0.931	0.605	0.782	0.846	0.832	1.39	2.079
1.034	0.724	0.804	0.715	0.978	1.291	0.851
1.848	0	0.628	0.746	1.313	1.227	1.427
1.375	3.617	0.709	0.799	0.746	1.107	1.164
1.346	1.346	0.538	0.404	0.85	1.01	1.346
0.872	0.988	0.988	0.726	1.13	1.381	1.241
0.585	1.455	0.751	0.634	0.796	1.056	1.061
0.309	1.55	0.608	0.481	0.99	1.171	1.237
0.9	0.836	0.59	0.606	1.223	1.256	1.101
0	0.307	0.97	0.952	0.976	1.552	0.635
0	0	0.969	1.024	0.81	1.46	0.551
0.908	1.17	0.804	0.736	0.923	1.125	1.178
1.556	1.496	0.561	0.597	0.976	1.034	0.633
0.509	1.425	0.534	0.528	1.018	1.188	1.134
0.93	1.779	1.055	0.752	1.079	1.094	0.875
0.73	1.087	0.725	0.64	0.39	0.749	1.148
0.815	1.279	0.793	0.594	0.934	1.189	1.019
0	0	0.779	0.735	0.999	1.291	1.314
0.904	0.638	0.809	0.792	0.893	0.974	1.199

0.162	0	0.828	0.785	0.618	1.589	2.623
0.848	0.919	0.929	0.804	0.815	1.138	1.302
0.685	0.521	0.712	0.786	0.924	1.132	1.267
1.753	1.463	0.667	0.717	0.707	1.073	1.14
1.215	1.195	0.625	0.571	0.747	0.948	0.972
0.991	0.972	0.646	0.653	1.259	1.466	1.007
0.967	0.43	0.755	0.665	0.463	0.781	1.323
0.413	0	0.878	0.6	1.12	1.575	0.935
0.466	0	1.296	0.903	0.98	1.171	0.815
0.138	0	0.779	0.699	0.923	1.899	2.141
0	0	1.514	0.742	0.966	0.802	0
0	0	1.8	0	1.8	0	1.8
0.337	0	0.689	0.723	1.046	1.28	1.268
0.93	0.972	0.916	0.689	0.914	1.205	1.149
1.113	1.109	0.871	0.641	0.79	0.972	0.958
1.227	1.679	0.532	0.638	0.669	0.919	1.222
0.867	0.998	0.983	0.928	1.115	1.123	0.94
0.495	0.942	0.999	1.012	0.742	1.012	1.502
0	0	0.66	0.742	0.625	1.491	2.183
1.596	1.28	0.663	0.805	0.893	0.803	0.888
0.74	0.945	0.547	0.695	0.617	1.01	0.547
1.375	3.617	0.709	0.799	0.746	1.107	1.164
1.014	0.685	0.746	0.754	0.878	0.976	1.162
1.074	0.33	1.032	0.968	0.971	1.37	0.791
1.115	0.754	0.753	0.953	0.866	1.126	1.366
0.9	0.853	0.76	0.655	0.778	1.352	1.148
1.962	2.943	0.732	0.656	1.073	0.604	1.294
0.319	1.142	0.686	0.924	1.068	1.068	0.932
0.78	1.301	0.709	0.919	0.976	1.168	0.925
2.024	2.59	0.72	0.518	0.747	0.971	1.214
1.751	0	1.139	0.509	1.433	0.912	1.393
0.994	1.156	0.966	0.751	1.074	1.055	1.027
1.046	1.466	0.738	0.532	0.908	1.039	1.218
1.303	1.351	0.528	0.853	1.095	1.17	0.73
1.294	0.773	0.728	0.602	0.917	1.018	1.164
0.992	1.784	0.851	0.897	0.898	1.08	1.329
0.337	0	0.667	0.964	1.004	1.354	1.228
0.865	0.726	0.724	0.754	0.911	0.897	1.242
4	2.5	0.909	0.345	0.294	0.323	0.714
0.975	1.18	0.931	0.878	1.005	1.074	1.038
0.904	0.638	0.809	0.792	0.893	0.974	1.199
0.742	1.057	0.951	0.857	1.024	0.886	1.19
1.098	0.676	0.85	0.735	1.136	1.033	1.608
1.027	0.771	0.711	0.701	0.818	1.389	1.064
1.748	1.406	0.645	0.668	0.619	1.109	1.118
1.109	2.21	0.814	0.852	0.86	1.17	1.026
0.73	0.633	0.621	0.783	1.142	1.142	1.116
1.035	0.97	0.888	0.701	0.612	0.955	0.989
1.414	2.828	0.745	0.462	1.188	1	1.038
1.121	1.075	0.911	1.059	0.846	0.919	0.855

0	0	1.35	1.667	1.552	0.964	0.346
1.962	2.943	0.69	0.426	1.158	0.97	1.214
0.431	0	0.622	0.76	1.266	1.371	1.44
1.348	0	0.63	0.409	0.936	1.073	1.407
1.103	0.303	0.891	1.102	0.969	1.073	1.466
1.095	1.034	0.624	0.618	1.061	0.788	0.688
0.659	0.7	0.904	0.735	0.862	1.489	1.473
1.3	1.104	0.67	0.758	1.101	1.004	0.937
0.142	2.015	0.665	0.606	0.871	1.107	1.433
0.84	0.509	0.887	1.091	0.807	0.985	1.343
0.965	0.954	0.697	0.754	0.94	0.862	0.921
0.87	1.172	1.13	0.861	0.839	1.227	0.666
1.161	1.639	0.534	0.645	0.682	0.974	1.253
1.417	2.317	0.463	0.574	0.502	0.861	1.039
1.254	0.371	0.843	0.822	1.109	1.631	1.112
0.979	0.289	0.952	0.817	1.118	1.911	1.491
1.222	0.684	0.751	1.019	1.055	1.092	1.389
1.322	0.803	0.818	1.034	0.847	1.179	1.545
0	0	0.5	0.722	1.368	2.031	0
1.322	0.803	0.818	1.034	0.847	1.179	1.545
1.635	0.872	0.799	0.625	0.98	0.873	1.103
1.069	1.201	0.961	0.771	0.351	0.846	0.654
0.889	1.079	0.847	0.793	0.869	1.393	1.236
0.889	1.079	0.847	0.793	0.869	1.393	1.236
1.277	1.034	0.793	1.03	0.614	0.899	1.08
0.902	0.638	0.76	0.725	0.937	0.947	0.928
1.437	0.592	0.818	0.201	0.747	1.323	1.692
1.639	0	1.156	0.708	1.082	0.629	1.355
1.199	1.013	0.688	0.827	1.01	1.003	0.663
1.192	0.779	1.152	0.676	0.811	0.991	0.991
1.066	1.048	0.863	0.733	0.564	0.826	1.058
0.128	0	0.774	0.776	0.971	1.7	2.147
0	0	0.643	0.659	1.25	1.339	2.143
0.699	0.796	0.849	0.739	1.038	1.435	1.348
1.301	1.261	0.81	0.849	1.127	1.179	1.011
0.913	0.545	0.558	0.802	0.808	1.282	1.349
0.932	0.961	0.947	0.709	1.03	1.346	1.102
0.85	0.638	0.588	0.886	1.166	1.433	1.111
1.069	0.664	0.492	0.742	1.278	1.408	1.369
1.131	1.308	0.547	0.771	1.231	1.362	1.277
0.563	0.376	0.576	0.79	1.339	1.367	1.524
0.826	1.345	0.498	0.594	0.579	0.779	1.166
0.975	0.993	0.749	0.729	1.089	1.086	1.326
1.23	1.175	0.592	0.581	0.894	1.051	1.05
1.323	0.678	0.562	0.55	1.05	1.267	1.033
0.88	1.071	0.871	0.965	0.948	0.968	1.216
0.978	0.798	0.737	0.585	0.94	1.223	1.398
1.577	1.097	0.683	0.651	0.681	1.131	1.09
1.493	0.919	1.274	0.789	0.301	0.828	1.319
0.791	1.001	0.656	0.709	1.252	1.451	0.724

0.87	1.172	1.13	0.861	0.839	1.227	0.666
0.649	1.946	0.658	0.915	1.035	1.331	1.225
2.126	3.158	0.461	0.567	0.754	0.65	1.105
1.111	1.054	0.666	0.806	1.008	1.067	0.989
0	0	1.074	0.486	1.428	0.8	1.712
0.055	0.11	0.747	0.541	1.307	1.352	0.791
0.106	0.238	0.768	0.831	1.3	0.894	1.056
0.829	1.11	1.133	1.041	0.536	1.084	1.484
0.85	1.118	0.889	1.041	0.994	1.098	1.245
1.163	1.078	0.543	0.569	1.003	1.37	1.422
0.914	0.996	0.558	0.513	1.265	1.244	1.583
0.618	1.768	0.874	0.88	1.173	1.005	0.996
1.267	1.125	0.905	0.751	1.071	1.061	1.112
1.158	1.332	0.737	0.888	0.883	0.876	0.899
0.768	1.11	1.213	0.803	0.886	1.191	0.958
1.04	0.462	1.054	0.673	1.16	0.696	1.026
0	0	0.625	0.563	1.125	1.25	1.875
1.122	1.093	0.585	0.533	0.789	1.302	1.064
1.056	0.982	0.862	0.716	0.629	0.919	1.013
0.874	0.746	1.131	0.813	0.718	0.983	0.772
1.016	0.895	0.873	0.619	0.814	0.833	1.274
1.201	0.158	0.865	0.685	0.578	0.964	1.511
0.908	0.512	0.666	0.761	1.051	1.11	1.366
1.052	1.275	0.826	0.862	0.922	0.87	0.854
1.026	1.357	0.962	0.799	1.251	0.86	1.117
0.828	0.81	0.79	0.751	0.922	1.081	0.998
1.29	0.496	0.882	0.668	1.134	0.694	1.171
0.593	1.264	0.666	0.668	0.777	1.102	1.168

GATS6e	GATS7e	GATS8e	GATS1p	GATS2p	GATS3p	GATS4p
1.511	1.133	1.511	0.8	0.788	0.986	0.986
1.376	0.929	0.707	0.882	1.084	0.99	1.043
1.074	1	1.031	0.532	0.72	1.013	1.044
1.989	1.492	0	0.737	0.888	0.987	1.195
0.961	1.201	2.227	1.388	0.654	1.085	0.766
0.436	0	0	2.18	0.715	1.353	0.367
1.176	1.06	0.729	0.954	0.746	0.926	1.101
0	0	0	1.057	0.795	1.594	0.332
1.524	1.474	1.425	0.799	0.885	0.797	0.91
1.638	1.016	0.603	0.899	1.197	0.893	0.885
0.93	1.024	0.972	1.07	1.201	0.944	1.061
0.694	0.819	0.625	0.505	0.714	1.075	0.943
1.826	3.316	1.5	1.37	0.339	0.3	0.328
1.046	1.123	0.989	0.917	0.692	0.777	0.942
0.802	1.005	0.827	0.735	0.89	0.776	1.696
0.787	1.18	0.811	0.732	0.888	0.762	1.791
0.904	0	0	0.812	1.074	0.94	1.427
0.532	0.736	0.639	0.854	0.635	0.92	1.085
1.632	0.493	0.703	0.874	1.02	0.736	0.974
1.282	0.688	0.606	0.834	0.762	1.048	1.43
1.208	0.847	0	0.955	0.705	1.188	1.188
0.935	1.457	2.235	0.817	0.928	1.44	1.709
0.736	0.687	0.916	0.707	0.661	1.016	1.105
0.829	1.186	0.792	0.722	0.884	0.789	1.862
0.991	1.101	0.831	1.248	1.17	1.203	1.258
1.011	0.943	1.129	0.553	0.724	0.875	1.054
1.333	0.321	0.745	0.951	1.191	1.007	0.893
0.872	0.847	0.792	0.731	0.657	0.816	0.715
0.901	0.772	0.868	1.182	1.105	0.86	1.14
1.059	0.995	0.73	1.08	1.275	0.697	1.009
1.019	1.428	1.136	1.451	0.799	0.898	1.201
1.165	1.186	1.063	0.694	0.706	0.978	1.04
1.793	0.621	0.112	0.848	1.17	0.921	1.042
1.031	0.52	0	0.912	0.906	1.04	1.669
1.115	0.787	1.072	0.8	0.894	0.995	1.096
0.979	0.6	1.066	1.072	0.657	1.088	1.456
1.401	0.691	0.484	0.646	0.869	1.002	1.078
3.536	0	0	1.369	0.453	1.295	0.92
0.565	0.806	1.279	0.503	0.783	0.658	0.61
1.009	1.019	1.351	0.8	0.755	1.013	1.058
0.719	1.115	1.412	0.735	0.533	1.004	1.026
1.519	0	0	0.753	0.388	0.912	1.149
0.94	1.08	1.209	0.613	0.453	0.66	0.712
0.991	1.64	2.004	0.561	0.517	0.76	0.871
0.854	1.426	1.384	0.981	0.897	0.743	1.216
0.749	1.102	0.822	0.703	0.757	0.791	1.449
0.831	0.921	0.927	0.735	0.799	0.84	1.341
0.887	1.051	1.132	0.693	0.876	1.023	1.209
0.863	1.152	1.062	0.696	0.885	0.81	1.016

1.063	1.208	1.289	0.81	0.893	0.732	1.092
1.033	0.784	1.176	0.769	0.927	0.671	0.981
1.076	1.042	0.825	0.77	0.841	0.967	1.153
0.888	0.946	1.128	0.814	0.927	0.751	1.101
0.85	1.018	1.011	0.816	0.696	0.85	1.314
1.009	1.004	0.924	0.729	0.809	0.834	1.351
1.161	0.952	1.207	1.161	0.644	1.001	1.09
1.068	0.895	1.226	0.783	0.942	0.798	1.035
0.818	0.881	1.095	0.765	0.937	0.688	1.029
0.868	0.898	1.246	0.778	0.949	0.77	1.061
0.918	0.817	1.172	0.899	0.817	0.882	1.174
1.327	1.566	1.494	1.175	0.536	0.669	0.643
0.822	1.081	0.928	0.732	0.783	0.833	1.405
0.972	1.323	1.358	0.754	0.788	0.777	1.449
0.87	1.048	1.044	0.761	0.77	0.762	1.373
0.822	1.081	0.928	0.732	0.783	0.833	1.405
1.48	0.846	0.768	0.845	0.666	0.665	1.356
0.275	1.164	1.608	0.884	0.739	0.228	0.74
0.32	2.126	0	1.742	0.485	0.81	0.829
1.158	1.038	1.651	1.054	0.722	0.751	0.917
1.249	1.504	0.968	0.999	1.088	1.474	0.869
0.994	1.152	0.059	1.578	0.542	1.454	0.87
1.481	1.784	1.148	0.948	0.999	1.179	0.834
0.682	1.54	1.301	1.306	0.49	0.827	1.069
0.84	1.42	1.089	0.738	0.837	1.036	0.967
1.055	1.122	0.851	1.009	0.555	1.509	1.375
1.076	0.544	0.898	1.043	0.711	1.028	1.176
0.746	0.754	0.292	0.571	0.444	0.786	0.941
1.646	1.251	1.251	0.948	0.899	0.903	0.694
1.531	1.024	0.589	0.823	0.887	0.884	0.758
0.945	1.131	0.948	0.787	0.945	0.91	1.121
1.444	1.971	0.088	0.715	0.881	0.847	0.635
1.106	0.719	1.678	0.745	0.683	0.58	0.841
1.496	0	0	0.82	0.508	1.379	0.855
0.83	1.339	2.2	1.125	0.973	1.015	1.256
0.973	1.703	0	2.228	0.934	1.122	0.612
0.452	0.496	0.794	1.455	0.871	1.173	1.155
1.005	2.111	1.319	1.199	1.092	1.242	0.341
1.011	0.943	1.129	0.553	0.724	0.875	1.054
1.6	1.502	2.254	0.788	0.679	0.784	1.019
1.22	1.493	1.399	0.558	0.579	0.952	0.864
0.96	0.981	0.892	0.663	0.84	0.762	1.389
0.975	1.416	2.579	1.18	0.808	1.016	0.834
1.456	1.348	2.33	0.617	0.726	1.012	0.896
1.199	1.068	1.145	0.615	0.598	0.724	0.673
1.026	1.035	0.976	0.88	0.895	0.862	0.832
1.161	0.962	1.035	0.915	0.855	1.162	1.318
0.348	0.426	0.348	1.163	0.945	1.162	1.915
1.193	1.318	0.371	0.867	0.796	0.816	1.331
1.04	1.008	0.954	0.839	0.815	0.942	1.251

0.883	0.782	1.007	1.835	0.55	0.665	0.923
0.493	1.103	1.009	0.955	0.588	1.071	1.066
1.163	1.033	0.993	0.707	0.768	1.002	1.049
1.066	0.988	0.586	0.639	0.838	1.079	0.926
0.441	0.881	1.762	0.913	1.053	0.638	1.257
1.471	1.343	2.82	0.761	0.821	0.739	0.779
1.129	1.056	1.114	1.047	0.876	0.798	0.908
0.981	1.105	1.372	1.372	0.584	1.047	0.982
1.188	1.644	1.425	0.534	0.548	0.713	0.848
0.425	0.567	1.36	0.716	0.816	1.133	1.19
0.769	0.846	0.974	0.991	0.973	1.223	1.112
1.112	1.7	1.895	0.648	0.913	0.874	1.72
0.81	1.257	1.715	0.668	0.579	0.762	1.22
1.361	1.03	0.549	0.914	0.749	0.465	0.855
0.962	0.903	1.326	0.897	0.932	1.118	1.09
1.081	1.074	2.491	0.509	0.702	1.059	1.093
1.894	1.195	1.266	0.98	0.792	0.895	0.987
0	0	0	1.137	0.839	1.059	1.248
1.091	1.269	1.339	0.752	0.683	0.851	0.794
0.841	0.574	1.148	0.536	0.563	0.919	1.273
0	0	0	2.135	0.91	0	0
0.591	0.688	0.821	0.744	0.572	0.5	0.53
0.684	0.866	1.641	1.379	0.593	0.757	1.094
1.177	1.349	1.146	0.627	0.559	0.643	0.938
3.193	0	0	0.606	0.454	0.67	1.678
1.41	1.538	2.507	0.947	0.856	0.838	0.964
1.075	1.024	2.047	1.322	0.751	1.076	0.805
0	0.523	0.907	0.756	0.68	1.417	1.063
0.832	0.99	0.947	1.075	1.165	1.005	1.137
1.458	1.223	0.746	1.386	0.494	1.368	1.037
1.344	1.09	0.745	1.351	0.583	1.297	0.939
1.425	0.969	0.477	0.887	0.98	0.844	1.227
0.73	0.926	0.849	0.779	0.674	0.875	1.241
0.32	2.126	0	1.742	0.485	0.81	0.829
1.776	1.581	3.76	0.767	0.813	0.753	1.117
1.154	1.346	1.346	0.538	0.404	0.85	1.01
0.756	0.912	1.052	1.107	0.775	1.134	1.302
1.034	0.693	1.762	0.745	0.612	0.779	1.046
0.769	0.382	1.612	0.68	0.535	1.03	1.191
1.078	0.916	0.798	0.615	0.635	1.128	1.213
1.858	0	0.57	0.842	0.991	1.074	1.525
1.704	0	0	0.889	1.036	0.859	1.429
1.063	0.951	1.142	0.793	0.798	0.955	1.065
1.06	1.496	1.217	0.504	0.507	0.849	0.878
1.188	0.509	1.425	0.534	0.528	1.018	1.188
1.284	0.795	1.886	0.875	0.843	1.17	0.764
1.453	0.848	0.748	0.981	0.85	0.683	0.913
1.045	0.815	1.279	0.793	0.594	0.934	1.189
0	0	0	0.893	0.785	0.926	1.263
1.365	0.945	0.562	0.947	0.884	0.87	0.912

1.045	0.118	0	0.981	0.886	0.644	1.46
1.012	0.815	0.926	1.056	0.852	0.793	1.058
1.411	0.662	0.444	0.838	0.882	0.899	1.015
1.513	1.193	1.984	0.613	0.58	0.561	1.055
1.22	1.471	1.179	0.6	0.702	0.805	1.09
1.037	0.891	1.108	1.275	0.876	0.826	1.372
1.492	1.194	0.778	0.675	0.653	0.738	0.546
1.245	0.268	0	0.954	0.623	1.057	1.512
1.118	0.466	0	1.296	0.903	0.98	1.171
0.776	0.101	0	0.922	0.788	0.952	1.743
0	0	0	0.695	0.715	1.067	1.974
0	0	0	1.8	0	1.8	0
1.25	0.23	0	0.822	0.728	0.935	1.217
0.93	0.854	1.009	0.855	0.894	0.868	1.365
0.732	1.06	1.069	1.016	0.731	0.851	0.981
1.003	1.227	1.679	0.532	0.638	0.669	0.919
1.148	0.936	0.778	0.97	0.84	1.058	0.954
1.496	0.427	0.935	1.021	1.053	0.71	0.925
0	0	0	0.81	0.879	0.596	1.411
1.415	1.684	1.567	0.762	0.848	0.891	0.771
0.51	0.639	0.854	0.622	0.747	0.956	1.143
1.776	1.581	3.76	0.767	0.813	0.753	1.117
1.349	1.098	0.613	0.854	0.858	0.887	0.936
0.811	0.994	0.38	1.092	0.941	0.894	1.319
1.497	1.063	0.691	0.781	0.997	0.836	1.058
0.98	0.876	0.873	0.761	0.889	0.791	1.508
1.707	2.094	3.141	0.845	0.746	1.153	0.576
1.291	0.367	1.189	0.74	0.949	1.074	1.07
1.199	0.812	1.353	0.766	0.945	0.971	1.167
1.349	2.024	2.59	0.72	0.518	0.747	0.971
0.532	1.635	0	1.324	0.459	1.346	0.892
1.14	1.172	1.41	0.785	0.656	0.921	0.771
1.189	1.063	1.514	0.737	0.601	0.941	1.001
0.828	1.214	1.466	1.32	0.636	0.871	1.166
1.043	1.339	0.792	0.726	0.667	0.937	0.969
0.739	0.96	1.858	0.931	0.924	0.912	1.126
0.927	0.646	0	0.68	0.951	0.965	1.398
1.446	0.888	0.66	0.864	0.852	0.902	0.794
1.905	4	2.5	0.909	0.345	0.294	0.323
0.962	0.986	1.171	0.941	0.86	0.99	1.088
1.365	0.945	0.562	0.947	0.884	0.87	0.912
1.105	0.795	1.044	1.228	1.06	1.088	0.985
1.127	0.837	0.515	1.011	0.769	1.059	0.881
1.054	0.982	0.789	0.71	0.917	0.845	1.515
1.655	1.398	2.171	0.652	0.613	0.591	1.044
1.295	1.185	2.026	0.859	0.863	0.851	1.174
1.104	0.785	0.68	0.687	0.905	1.21	1.197
0.998	1.046	0.963	1.003	0.761	0.619	0.975
1.834	1.507	3.013	0.834	0.396	1.208	1.014
1.524	1.054	1.135	0.95	1.079	0.882	0.906

0	0	0	1.35	1.667	1.552	0.964
1.72	2.094	3.141	0.771	0.364	1.175	0.981
1.311	0.478	0	0.699	0.914	1.325	1.392
1.61	1.492	0	0.711	0.407	0.951	1.137
1.748	1.06	0.205	0.946	1.152	0.922	0.903
0.954	1.088	1.043	1.395	0.828	1.014	0.807
0.905	0.584	0.683	1.088	0.833	0.849	1.412
0.87	1.349	1.173	1.409	0.656	1.086	1.035
0.904	0.232	1.773	0.678	0.578	0.929	0.805
1.632	0.77	0.493	0.95	1.14	0.805	0.898
0.881	0.965	0.954	0.697	0.754	0.94	0.862
0.645	0.83	1.206	1.211	0.864	0.929	1.272
1.032	1.161	1.639	0.534	0.645	0.682	0.974
1.048	1.417	2.317	0.463	0.574	0.502	0.861
1.24	1.333	0.281	0.967	0.899	0.998	1.564
0.944	0.926	0.195	1.011	0.898	1.012	1.746
1.425	1.202	0.679	0.779	1.017	1.03	1.003
1.718	1.249	0.673	0.848	1.062	0.786	1.044
0	0	0	0.5	0.722	1.368	2.031
1.718	1.249	0.673	0.848	1.062	0.786	1.044
1.438	1.244	3.563	0.761	0.412	0.889	0.928
0.291	0.957	1.291	0.92	0.927	0.953	1.052
0.901	0.885	1.067	0.925	0.872	0.879	1.34
0.901	0.885	1.067	0.925	0.872	0.879	1.34
0.715	0.911	1.104	0.789	0.81	1.132	0.856
0.905	0.894	0.653	0.827	0.787	1.005	1.003
1.737	1.835	1.23	0.745	0.59	0.691	1.226
1.03	1.776	0	2.042	0.686	1.36	0.555
0.731	1.145	1.04	0.794	0.897	1.088	1.079
1.206	1.192	0.779	1.152	0.676	0.811	0.991
0.851	1.072	1.041	0.957	0.808	0.554	0.846
1.127	0.093	0	0.915	0.899	1.029	1.558
0	0	0	0.643	0.659	1.25	1.339
1.168	0.674	0.788	0.897	0.79	0.999	1.354
0.8	1.326	1.234	1.517	0.888	0.975	1.049
1.018	0.848	0.54	1.667	0.599	0.704	0.989
1.173	0.932	0.826	0.929	0.844	0.745	0.996
1.079	0.891	0.668	1.529	0.763	1.227	1.376
1.106	1.189	0.735	1.561	0.76	1.283	1.348
0.97	1.188	1.374	1.005	0.836	1.08	1.156
0.983	0.521	0.353	1.524	0.719	1.331	1.416
1.204	0.853	1.395	0.887	0.547	0.533	0.61
0.683	1.035	0.956	1.571	0.638	0.848	0.9
1.216	1.399	1.052	1.776	0.529	0.732	0.866
0.875	1.393	0.725	0.616	0.636	1.061	0.989
0.694	0.944	1.098	1.561	0.807	1.009	0.904
1.626	0.85	0.717	0.797	0.632	0.95	1.276
1.626	1.258	1.69	0.726	0.602	0.648	1.09
0.767	1.512	1.105	1.921	0.615	0.362	0.736
1.164	0.851	1.148	1.272	0.707	0.916	1.587

0.645	0.83	1.206	1.211	0.864	0.929	1.272
0.21	0.673	2.018	0.705	0.981	0.989	1.336
1.163	2.126	3.158	0.461	0.567	0.754	0.65
1.111	1.111	1.042	0.707	0.798	1.002	1.054
0	0	0	1.338	0.485	1.377	0.728
1.166	0.108	0.216	0.613	0.818	0.871	1.37
0.963	1.168	1.354	0.922	0.918	0.909	1.487
1.105	0.807	1.083	1.313	0.827	0.873	0.992
0.973	0.76	1.093	0.903	1.009	0.951	1.079
1.969	1.26	1.185	1.546	0.617	1.265	1.207
1.671	0.999	1.081	1.667	0.559	1.544	0.961
1.109	0.642	1.756	0.95	0.841	1.139	1.001
0.879	0.995	1.129	1.006	0.765	0.946	1.13
1.456	1.337	1.37	0.736	0.814	0.743	0.975
1.197	0.855	1.053	1.211	0.746	0.937	1.095
0.706	0.961	0.464	1.23	0.72	1.141	0.625
0	0	0	0.625	0.563	1.125	1.25
1.072	1.142	1.091	0.601	0.57	0.808	1.257
0.974	1.067	0.975	0.955	0.777	0.637	0.945
0.938	0.854	0.722	1.144	0.805	0.741	1.042
1.053	1.037	0.879	0.942	0.688	0.877	0.815
1.569	1.303	0.108	0.97	0.837	0.632	1.026
1.236	0.908	0.512	0.666	0.761	1.051	1.11
1.046	1.016	1.356	0.922	0.967	0.995	0.804
0.91	1.008	1.409	1.022	0.766	1.328	0.825
0.908	0.98	0.542	1.01	0.739	0.933	1.102
1.317	1.405	0.552	1.047	0.742	1.174	0.753
0.948	0.631	1.927	0.665	0.633	0.697	1.01

GATS5p	GATS6p	GATS7p	GATS8p	EPS0	EPS1	EEig01x
0.756	1.511	1.133	1.511	10.836	8.317	4.579
1.039	1.35	0.97	0.709	15.422	11.793	4.371
1.157	1.075	1.036	0.982	17.052	13.784	4.601
0.963	1.836	1.377	0	7	5.433	4.036
1.136	0.849	1.062	2.134	10.784	8.364	4.524
1.029	0.045	0	0	7.772	6.31	5.098
0.735	1.159	1.098	0.788	20.499	15.546	4.616
0	0	0	0	6.845	5.422	4.386
1.256	0.902	1.643	1.149	15.137	12.353	4.648
1.398	1.626	1.169	0.726	11.905	8.853	4.199
0.85	0.949	1.046	0.97	25.938	20.329	4.249
1.177	1.088	2.003	1.941	21.077	16.098	4.704
0.724	1.826	3.316	1.5	14.397	11.386	4.533
1.06	1.398	1.171	0.851	18.82	14.148	4.669
1.151	0.838	0.879	0.824	15.8	13.24	4.689
1.192	0.871	1.003	0.822	15.353	12.774	4.689
0.868	0.702	0	0	9.259	7.369	4.415
1.2	0.557	0.809	0.7	23.562	19.758	4.575
1.518	1.623	0.522	0.752	12.137	9.382	4.06
1.612	1.218	0.735	0.634	14.301	11.277	4.209
1.201	1.046	0.835	0	14.491	11.762	4.652
0.7	0.522	0.696	0.71	12.776	10.344	4.558
1.047	1.134	0.471	0.628	13.878	11.317	4.541
1.22	0.644	1.008	0.804	14.8	12.338	4.689
1.456	1.027	1.036	0.684	18.535	14.392	4.132
1.267	1.006	0.957	1.184	17.748	15.02	5.1
1.417	1.345	0.398	0.751	14.706	11.365	4.041
0.99	0.869	0.859	0.748	28.032	22.457	4.604
1.177	0.901	0.772	0.868	14.897	11.87	4.703
1.424	1.086	1.033	0.736	14.966	11.365	4.041
1.216	1.239	1.373	1.098	25.874	20.664	4.864
1.053	0.988	0.903	0.801	28.219	24.089	4.918
1.494	1.757	0.8	0.203	12.179	9.798	4.574
1.81	1.084	0.527	0	14.113	10.799	4.354
1.011	1.085	0.813	1.021	22.35	19.55	5.211
1.009	1.11	0.738	0.922	19.173	15.3	4.472
1.143	1.319	0.585	0.441	16.388	13.795	5.02
0.916	2.852	0	0	8.897	7.273	4.621
0.614	0.565	0.806	1.279	19.048	15.712	4.666
0.922	1.028	0.926	1.242	17.643	14.67	4.817
1.194	0.804	1.074	1.338	22.853	18.274	4.729
1.705	1.395	0	0	12.328	9.883	4.522
0.73	0.94	1.08	1.209	25.502	21.983	5.076
1.238	0.645	1.43	1.748	12.516	10.334	4.721
1.126	0.906	1.474	1.372	20.905	16.274	4.759
1.67	0.922	0.919	0.925	15.742	12.754	4.602
1.608	0.874	0.838	0.867	16.19	13.22	4.602
1.645	1.193	1.105	0.738	20.673	16.708	4.61
1.341	0.972	1.271	0.956	19.361	15.795	4.609

1.51	0.872	1.058	1.237	21.129	17.195	4.624
1.469	0.819	0.882	1.406	19.629	15.678	4.614
1.298	1.074	1.011	0.753	29.129	23.475	4.611
1.435	0.914	0.935	1.113	19.629	15.716	4.611
1.166	1.033	0.915	0.832	18.369	14.664	4.87
1.604	0.943	0.903	0.888	17.767	14.196	4.614
1.218	1.047	1.167	0.984	23.3	19.213	4.817
1.382	0.899	0.964	1.357	24.007	19.662	4.615
1.594	0.721	1.025	1.151	16.56	13.25	4.543
1.615	0.937	0.972	1.028	23.646	19.128	4.612
1.443	1.177	1.014	1.088	19.181	15.233	4.611
0.787	1.071	1.227	1.343	16.322	13.807	4.803
1.693	0.911	0.959	0.87	15.742	12.754	4.602
1.308	0.808	1.148	1.021	18.913	15.329	4.612
1.215	0.906	0.96	0.891	17.052	13.812	4.609
1.693	0.911	0.959	0.87	15.742	12.754	4.602
1.988	1.305	0.97	1.002	16.725	12.888	4.288
0.641	0.369	0.989	1.298	18.328	14.347	4.331
2.266	0.473	0.845	0	10.289	8.856	4.951
0.843	1.269	1.259	1.7	12.69	9.881	4.263
0.596	0.635	1.175	1.466	14.137	11.386	4.513
1.065	1.316	0.575	0.746	10.809	8.337	4.854
0.58	0.794	1.471	1.836	14.137	11.386	4.58
1.29	0.856	1.475	1.13	13.935	11.801	4.879
0.868	1.326	0.988	1.461	12.069	9.816	4.538
1.546	1.008	1.258	0.405	11.621	8.287	4.187
0.963	0.999	0.568	0.982	17.187	13.247	4.27
1.149	0.825	0.8	0.135	17.231	13.84	4.527
1.148	1.564	1.182	1.182	12.732	10.272	4.604
1.118	1.485	0.948	0.641	16.137	13.306	4.626
1.223	0.903	1.082	0.945	17.43	13.598	4.248
1.207	1.251	1.229	4.933	8.524	6.855	4.438
1.233	1.235	0.615	1.571	16.508	13.217	4.804
2.045	0.654	0	0	9.414	7.395	4.347
0.766	0.921	0.687	1.128	15.74	12.863	4.678
0.731	0.1	0.175	0	9.422	6.82	4.641
1.354	0.187	0.464	0.742	10.911	8.831	4.969
0.565	1.005	2.111	1.319	14.113	10.868	4.455
1.267	1.006	0.957	1.184	17.748	15.02	5.1
1.28	1.039	1.845	1.452	13.483	10.833	4.541
0.694	0.785	1.066	4.115	12.483	9.866	4.375
1.091	1.076	1.186	0.684	19.214	16.201	4.694
0.989	0.943	1.249	2.358	11.732	9.303	4.538
1.029	1.464	1.37	2.343	11.569	9.323	4.456
1.214	1.199	1.068	1.145	17.73	15.195	4.98
0.788	1.026	1.035	0.976	35.365	29.959	4.98
1.045	1.116	0.872	0.676	19.284	15.231	4.458
0.835	0.585	0.715	0.585	12.949	9.915	4.246
1.108	1.154	1.276	0.529	16.335	12.739	4.82
1.167	1.035	1.008	0.952	36.753	30.914	5.062

0.737	0.861	0.603	0.891	18.204	14.858	4.643
1.427	0.774	1.026	0.829	20.516	16.797	4.49
0.989	1.182	1.028	1.015	20.266	17.046	4.953
1.022	1.186	0.906	0.47	14.232	10.553	4.504
1.318	0.435	0.762	1.525	9.585	6.126	3.473
0.967	1.385	1.264	2.655	14.231	11.178	4.814
0.919	1.129	1.056	1.114	28.566	23.521	4.639
0.664	1.518	1.035	1.362	12.378	9.921	4.673
0.563	1.188	1.644	1.425	12.69	9.865	4.283
1.074	0.425	0.567	1.36	11.983	9.375	4.227
1.269	0.833	0.96	1.055	17.336	13.282	4.281
0.832	0.857	0.934	0.98	14.792	11.886	4.505
0.91	0.82	1.311	1.69	17.114	14.738	4.736
1.038	1.393	1	0.491	17.602	14.327	4.386
0.859	0.999	0.853	1.145	14.662	11.864	4.633
1.137	1.046	0.928	2.231	12.121	9.829	4.629
0.702	1.759	1.066	1.149	14.646	11.76	4.641
0.117	0	0	0	5.638	4.401	4.147
0.63	1.595	1.075	1.118	20.203	16.292	4.568
1.541	0.81	0.635	1.27	11.828	9.33	4.409
0	0	0	0	3.593	2.907	4.562
0.852	1.061	0.63	0.763	26.509	21.664	4.682
1.509	0.661	0.785	1.551	13.248	10.84	4.855
1.178	1.177	1.349	1.146	23.318	19.608	5.043
1.716	2.94	0	0	7.82	5.812	4.357
0.972	1.34	1.471	2.36	14.281	11.757	4.868
0.898	1.048	0.862	2.039	12.102	9.22	4.524
1.214	0	0.523	0.907	11.146	8.806	4.406
0.957	0.876	0.998	0.969	20.057	16.029	4.457
1.323	1.297	1.159	0.668	22.118	17.628	4.854
1.453	1.152	1.031	0.546	21.825	17.204	4.854
1.861	1.245	0.916	0.652	15.585	12.828	4.666
0.815	0.801	1.084	0.734	17.886	14.312	4.455
2.266	0.473	0.845	0	10.289	8.856	4.94
1.161	1.685	1.295	3.54	14.28	12.287	4.954
1.346	1.154	1.346	1.346	9.414	7.358	4.193
1.245	0.687	0.86	0.97	25.256	20.276	4.315
0.962	1.288	0.565	1.416	16.508	13.217	4.804
1.202	0.821	0.282	1.517	13.983	11.291	4.497
1.112	1.045	0.893	0.848	22.275	17.739	4.716
0.753	1.781	0	0.212	10.361	8.409	4.214
0.647	1.622	0	0	8.5	6.899	4.186
1.197	1.054	0.892	1.181	17.767	14.211	4.68
0.511	1.002	1.434	1.672	13.69	10.79	4.377
1.134	1.188	0.509	1.425	12.535	9.82	4.368
1.541	0.879	1.229	1.068	10.025	7.815	4.227
1.238	1.147	0.627	1.673	16.24	13.322	4.502
1.019	1.045	0.815	1.279	15.378	11.719	4.403
1.238	0	0	0	7.768	5.806	4.181
1.155	1.306	0.909	0.648	17.137	14.251	4.727

2.301	1.194	0.18	0	11.154	8.216	4.342
1.194	1.08	0.862	0.89	15.999	12.843	4.381
1.233	1.339	0.688	0.516	16.353	13.192	4.7
1.183	1.436	2.113	1.311	13.93	11.327	4.552
0.975	1.043	1.148	1.095	20.861	16.217	4.531
0.738	1.142	1.327	0.861	14.825	12.264	5.023
0.896	1.071	0.857	0.457	12.569	9.276	4.149
0.879	1.308	0.485	0	12.145	9.282	4.705
0.815	1.118	0.466	0	13.93	11.397	4.482
1.857	0.886	0.154	0	11.828	9.323	4.353
0	0	0	0	6.845	5.422	4.285
1.8	0	0	0	5.19	3.907	4
1.242	1.297	0.385	0	15.291	11.71	4.834
1.121	0.805	1.243	0.985	16.886	13.632	4.691
0.99	0.679	1.131	1.121	21.378	17.228	4.542
1.222	1.003	1.227	1.679	17.291	14.593	4.979
1.809	0.838	0.795	1.076	13.232	9.605	4.437
1.456	1.511	0.529	0.945	12.56	9.348	4.04
1.866	0	0	0	7.768	5.844	4.496
1.122	0.991	1.868	1.179	15.637	12.82	4.659
1.356	0.704	1.058	0.863	27.126	22.221	4.535
1.161	1.685	1.295	3.54	14.28	12.287	4.953
1.116	1.283	1.023	0.688	19.662	16.211	4.724
0.809	0.878	1.113	0.311	12.483	9.856	4.421
1.334	1.503	1.141	0.785	13.878	11.327	4.404
1.118	0.841	1.091	0.967	19.085	15.736	4.694
1.202	1.465	1.892	2.838	11.077	8.767	4.568
0.928	1.273	0.3	1.117	16.998	14.754	5.124
0.929	1.14	0.764	1.273	15.766	13.252	5.117
1.214	1.349	2.024	2.59	11.232	8.773	4.462
1.347	0.479	1.801	0	9.679	7.865	4.454
1.166	0.844	0.853	1.06	20.919	17.157	4.891
1.247	1.144	1.038	1.438	16.328	12.618	4.679
0.638	0.95	1.542	1.03	13.378	10.894	4.656
1.176	1.081	1.274	0.766	16.983	13.156	4.648
1.36	0.641	1.01	1.739	11.016	8.862	4.314
1.325	0.923	0.23	0	7.715	5.789	4.257
1.211	1.381	0.859	0.72	15.353	12.239	4.608
0.714	1.905	4	2.5	14.113	10.868	4.533
1.043	0.965	0.969	1.184	28.187	23.011	4.631
1.155	1.306	0.909	0.648	17.137	14.251	4.727
1.392	1.402	0.624	0.72	16.118	12.649	4.527
1.383	1.012	1.199	0.738	14.483	11.84	4.795
1.054	0.894	1.286	0.756	18.214	15.261	4.692
1.316	1.275	2.158	1.395	13.43	10.86	4.515
1.029	1.247	1.076	2.303	15.559	13.181	5.124
1.068	1.019	0.703	0.609	16.32	12.778	4.56
0.983	0.973	1.029	0.972	26.82	22.658	4.778
1.038	1.802	1.364	2.728	10.264	7.651	4.774
0.73	1.548	1.152	1.043	8.577	6.398	4.058

0.346	0	0	0	12.689	9.884	4.671
1.244	1.687	1.892	2.838	11.255	8.719	4.824
1.335	1.181	0.408	0	15.872	12.311	4.558
1.386	1.48	1.279	0	12.954	10.358	4.855
1.366	1.685	1.128	0.347	11.983	9.381	4.51
0.553	1.416	1.097	0.925	21.488	17.216	4.925
1.285	0.872	0.691	0.704	17.706	14.326	4.564
1.178	0.806	0.999	0.773	16.317	13.361	4.876
1.042	2.393	0.083	1.691	11.121	8.83	4.363
1.221	1.623	0.873	0.522	12.137	9.382	4.06
0.921	0.881	0.965	0.954	19.231	15.193	4.363
0.662	0.73	0.893	1.158	19.187	15.147	4.597
1.253	1.032	1.161	1.639	16.791	14.105	4.982
1.039	1.048	1.417	2.317	15.012	12.74	4.75
1.141	1.003	1.221	0.405	11.361	8.215	4.179
1.364	0.849	1.001	0.332	11.361	8.215	4.179
1.338	1.405	1.232	0.69	17.096	12.82	4.335
1.465	1.705	1.357	0.867	12.69	9.881	4.493
0	0	0	0	8.104	6.343	4.422
1.465	1.705	1.357	0.867	12.69	9.881	4.493
1.172	1.036	2.791	1.291	11.232	8.799	4.515
0.625	0.43	0.996	0.968	18.991	14.845	4.501
1.141	0.914	0.893	1.082	16.618	13.186	4.568
1.141	0.914	0.893	1.082	16.618	13.186	4.568
1.201	1.442	2.119	0.671	13.715	10.343	4.124
0.969	0.91	0.904	0.634	22.06	17.286	4.378
1.166	2.797	0.952	0.345	9.342	7.942	4.487
1.024	0.569	0.563	0	9.918	8.354	5.103
0.561	0.843	1.22	1.021	20.516	16.765	4.58
0.991	1.206	1.192	0.779	13.585	10.898	4.552
1.046	0.847	1.061	1.051	25.511	21.141	4.782
1.878	1.187	0.142	0	13.405	10.298	4.354
2.143	0	0	0	6.397	4.901	4.291
1.319	1.126	0.712	0.802	15.093	12.282	4.45
1.249	0.697	1.286	1.261	21.944	17.667	4.938
1.006	1.183	0.954	0.67	11.016	8.899	4.645
0.813	0.9	0.754	1.289	18.629	14.19	4.616
1.08	0.555	0.529	0.397	10.834	8.872	4.856
1.214	0.834	0.811	0.409	14.662	11.854	4.861
1.664	0.744	1.174	1.358	19.933	15.79	4.558
1.197	0.515	0.374	0.254	11.386	9.307	4.861
0.875	0.906	0.815	1.507	16.292	13.3	4.705
1.689	0.573	0.776	1.077	14.988	11.672	4.813
0.859	1.35	0.896	1.264	12.886	10.275	4.7
0.925	0.739	1.088	0.969	11.828	9.303	4.35
0.976	0.922	0.741	0.902	18.471	14.236	4.849
1.458	1.653	1.028	0.832	15.137	11.274	4.471
1.291	1.21	1.954	1.093	13.983	11.307	4.564
0.886	0.883	1.738	0.979	12.016	9.86	4.601
0.854	0.956	0.888	0.876	14.377	11.781	5.027

0.662	0.73	0.893	1.158	19.187	15.147	4.597
1.241	0.174	0.635	1.906	9.602	7.889	4.23
1.105	1.163	2.126	3.158	13.805	11.745	4.738
0.961	1.123	1.111	1.06	20.056	17.068	4.962
1.431	0	0	0	8.397	6.8	4.545
0.82	1.474	1.303	2.607	10.264	7.651	4.774
0.597	0.507	1.056	1.767	11.723	9.399	4.263
1.15	1.555	0.877	0.909	13.637	10.876	4.317
1.28	1.006	0.894	1.129	16.673	12.811	4.165
1.235	1.434	1.063	0.684	13.662	10.872	4.854
1.222	1.142	0.788	0.616	11.516	8.837	4.854
1.022	1.119	0.614	1.772	12.54	9.791	4.642
0.931	0.788	1.271	1.018	17.861	14.326	4.398
1.13	1.022	1.605	1.591	15.637	12.82	4.659
1.002	1.253	0.746	1.145	14.127	10.642	4.429
1.072	0.783	1.072	0.466	18.836	14.658	4.365
1.875	0	0	0	6.483	4.349	3.879
1.082	1.07	1.11	1.092	21.457	16.733	4.475
1.007	0.954	1.049	0.984	26.451	22.202	4.774
0.874	0.962	0.888	0.759	22.212	16.307	4.714
1.265	1.023	1.007	0.903	38.709	31.641	5.142
1.422	1.355	1.153	0.181	18.146	14.687	4.922
1.366	1.236	0.908	0.512	15.397	12.35	4.599
0.846	1.055	1.067	1.248	15.723	12.186	4.663
1.082	0.882	1.035	1.336	12.602	9.737	4.307
0.906	1.104	0.702	0.795	19.256	15.824	4.563
1.149	1.252	1.244	0.474	15.085	12.31	4.686
1.107	1.171	0.748	1.312	12.776	10.302	4.611

EEig02x	EEig03x	EEig04x	EEig05x	EEig06x	EEig07x	EEig08x
3.614	3.556	3.207	2.699	2.099	1.767	1.002
3.796	3.455	3.42	3.26	3.06	2.532	2.127
4.27	4.075	3.727	3.51	3.338	3.012	2.881
3.592	3.23	2.5	1.405	0.722	0.5	0.329
3.792	3.435	2.833	2.585	2.49	1.767	1.245
4.125	2.974	2.348	1.187	0.637	0.612	0
4.199	3.871	3.535	3.387	2.939	2.869	2.832
3.426	3.125	2.359	2.015	1.153	0.241	0.097
4.26	3.919	3.46	3.319	3.101	2.772	2.545
3.422	3.107	2.992	2.89	2.582	1.874	1.438
4.155	4.1	3.889	3.512	3.37	3.269	3.069
4.399	3.922	3.526	3.439	3.232	3.166	2.874
4.045	3.523	3.205	2.974	2.867	2.736	2.598
4.122	4.103	3.413	3.18	3.069	2.858	2.654
4.214	4.081	3.746	3.477	3.276	2.752	2.511
4.195	4.067	3.558	3.472	2.913	2.747	2.5
3.809	3.248	2.972	2.522	2.5	1.291	0.794
4.292	4.286	4.04	3.913	3.767	3.291	3.236
3.647	3.411	3.256	3.066	2.5	1.891	1.444
4.077	3.613	3.151	2.879	2.851	2.5	2.24
4.138	3.917	3.48	3.227	3.079	2.637	2.377
4.448	3.75	3.461	3.048	2.717	2.608	2.5
4.147	3.961	3.594	3.293	3.03	2.723	2.5
4.114	3.945	3.517	3.463	2.865	2.747	2.5
3.858	3.812	3.226	3.174	2.883	2.827	2.5
4.92	4.093	3.753	3.434	3.267	3.132	3
3.66	3.532	3.371	3.071	2.533	2.5	1.911
4.557	4.474	4.425	3.661	3.605	3.535	3.511
3.99	3.617	3.244	3.205	2.834	2.775	2.5
3.534	3.371	3.232	3.071	2.594	2.5	1.952
4.47	4.414	4.19	4	3.486	3.313	3.273
4.766	4.398	4.206	3.874	3.789	3.68	3.562
4.106	3.822	3.373	2.967	2.477	2.172	1.53
4.042	3.279	3.232	2.864	2.619	2.29	2.161
4.803	4.348	4.304	3.792	3.628	3.548	3.253
4.081	3.929	3.518	3.303	3.118	2.657	2.5
4.13	3.778	3.537	3.442	3.135	2.636	2.473
3.898	3.576	3.029	2.409	1.921	1.746	0.715
4.256	4.099	3.809	3.34	3.2	3.164	2.607
4.567	4.246	3.922	3.391	3.309	3.13	2.922
4.474	4.026	3.928	3.713	3.501	3.291	3.037
4.045	3.683	3.132	2.919	2.867	2.736	2.341
4.723	4.552	4.303	4.121	3.71	3.69	3.496
4.145	3.807	3.451	3.294	3.131	2.596	2.026
4.192	3.911	3.493	3.333	3.061	2.969	2.917
4.183	4.028	3.552	3.223	2.915	2.845	2.5
4.206	4.039	3.743	3.276	3.223	2.852	2.5
4.199	4.176	4.04	3.553	3.28	2.915	2.858
4.11	4.062	3.96	3.503	3.299	3.118	2.857

4.377	4.294	4.016	3.8	3.232	3.125	2.853
4.373	4.073	3.804	3.545	3.312	3.124	2.865
4.351	4.227	4.189	4.053	3.804	3.682	3.281
4.372	4.068	3.801	3.549	3.252	3.126	2.856
4.29	3.97	3.555	3.501	3.252	3.007	2.799
4.21	4.068	3.745	3.359	3.276	2.89	2.74
4.611	4.179	4.073	3.913	3.681	3.51	3.224
4.442	4.336	4.095	3.86	3.792	3.252	3.124
4.365	3.926	3.79	3.129	2.989	2.799	2.708
4.372	4.258	4.065	3.801	3.663	3.27	3.126
4.341	4.061	3.597	3.548	3.252	2.976	2.849
4.618	4.235	3.971	3.649	3.283	3.007	2.5
4.183	4.028	3.552	3.223	2.915	2.845	2.5
4.113	3.967	3.853	3.501	3.252	2.895	2.831
4.089	3.961	3.551	3.5	3.252	2.858	2.594
4.179	4.026	3.518	3.223	2.98	2.851	2.465
3.63	3.507	3.31	2.702	2.64	2.371	2.247
3.87	3.622	3.548	3.436	3.097	2.83	2.5
4.79	3.965	3.377	3.006	2.694	1.565	1.06
3.861	3.455	3.202	2.912	2.5	2.5	2.278
4.028	3.461	3.351	3.202	2.882	2.712	2.417
3.846	3.573	3.057	2.5	2.443	1.305	1.12
4.029	3.584	3.416	3.206	2.884	2.712	2.597
4.765	4.037	3.707	3.34	3.008	2.658	2.645
4.091	3.719	3.345	3.05	2.5	2.389	1.922
3.846	3.38	2.839	2.568	2.525	1.907	1.542
4.134	3.877	3.587	3.398	3.094	2.948	2.617
4.452	3.787	3.688	3.118	2.978	2.891	2.751
4.182	3.775	3.476	2.958	2.817	2.253	2.114
4.22	3.919	3.719	3.25	3.173	2.858	2.651
4.11	3.753	3.397	3.227	2.881	2.854	2.093
3.967	3.38	2.762	2.5	1.363	0.554	0.5
4.186	3.847	3.731	3.516	3.299	3.213	2.682
3.814	3.232	2.888	2.254	1.794	1.51	1
4.196	3.829	3.469	3.383	3.016	2.859	2.815
3.031	2.578	2.247	2	1.674	1	0.555
3.929	3.787	3.239	2.895	2.5	2.5	1.125
4.045	3.331	2.971	2.867	2.736	2.694	2.233
4.92	4.093	3.753	3.434	3.267	3.132	3
4.04	3.793	3.422	3.299	2.772	2.554	2.5
4.154	3.495	3.232	2.983	2.849	2.769	1.717
4.63	4.2	4.08	3.482	3.397	3.232	3.124
3.882	3.491	3.016	2.871	2.485	2.224	1.712
4.206	3.656	3.315	3.104	2.703	2.532	1.455
4.568	4.098	3.613	3.337	3.157	2.911	2.771
4.569	4.099	4.094	4.019	3.93	3.627	3.518
4.133	3.9	3.639	3.501	3.222	3.048	2.914
3.736	3.242	3.117	2.781	2.5	2.5	2.405
3.871	3.736	3.515	2.974	2.781	2.732	2.5
4.823	4.4	4.248	4.18	4.135	4.083	3.807

4.643	4.007	4.004	3.486	3.434	3.275	2.5
4.428	3.838	3.571	3.536	3.256	3.165	3.012
4.621	4.254	3.93	3.862	3.666	3.268	3.057
4.28	3.81	3.593	3.425	2.884	2.869	2.342
3.414	2.463	1.618	1.618	1.618	1	0.586
4.357	3.714	3.427	3.067	2.836	2.583	2.158
4.385	4.2	4.171	3.785	3.739	3.527	3.355
4.164	3.636	3.567	3.038	2.468	2.199	1.547
4.033	3.547	3.377	2.915	2.549	2.5	2.5
3.938	3.669	3.188	2.864	2.663	2.5	1.925
3.904	3.634	3.507	3.104	2.908	2.813	2.5
4.071	3.823	3.524	3.326	3.035	2.535	2.5
4.56	4.143	3.721	3.554	3.253	2.974	2.685
4.106	4.089	3.66	3.509	3.327	2.612	2.465
3.998	3.897	3.724	3.223	2.741	2.619	2.502
4.029	3.601	3.441	3.156	2.689	2.5	2.48
4.269	3.761	3.669	3.31	2.799	2.575	2.503
3.465	2.908	1.473	1	0.419	-0.107	-0.4
4.351	3.902	3.658	3.356	3.208	2.975	2.842
4.055	3.615	3.231	2.908	2.608	2.5	1.969
2.879	0.653	0.438	0	-0.532	0	0
4.663	4.36	3.784	3.658	3.421	3.181	3.11
4.306	3.916	3.642	3.216	2.718	2.656	2.118
4.583	4.31	3.744	3.655	3.621	3.477	3.251
3.422	2.601	2	1.491	1	0.452	0.144
4.091	3.679	3.348	3.185	2.939	2.796	2.123
3.797	3.468	3.386	2.745	2.524	1.808	1.618
4.231	3.446	2.942	2.842	2.415	1.628	1.252
4.189	3.861	3.617	3.335	3.178	2.976	2.804
4.471	3.905	3.767	3.734	3.436	3.135	3.093
4.371	3.902	3.711	3.598	3.34	3.289	3.099
4.067	3.821	3.342	3.208	3	2.88	2.621
4.233	3.824	3.7	3.549	3.29	2.93	2.572
4.783	3.964	3.376	2.881	2.324	1.464	1
4.148	3.863	3.539	3.443	2.954	2.781	2.337
3.809	3.321	2.967	2.5	2.011	1.131	0.5
4.24	4.165	3.82	3.74	3.544	3.434	3.203
4.186	3.847	3.731	3.516	3.299	3.213	2.682
4.182	3.859	3.426	3.209	2.92	2.637	2.5
4.474	4.018	3.778	3.632	3.311	3.153	2.876
3.88	3.763	3.184	2.414	2.252	1.606	1
3.819	3.198	2.414	2.216	1.584	1	0.849
4.313	4.11	3.446	3.434	3.206	2.937	2.886
4.155	3.926	3.505	2.93	2.858	2.632	2.5
4.092	3.675	3.305	2.93	2.675	2.5	2.328
3.728	3.369	3.02	2.589	1.807	1.441	1.25
4.347	4.092	3.86	3.325	3.032	2.98	2.867
4.06	3.977	3.653	3.5	3.032	2.892	2.589
3.616	2.676	2.294	1.868	1	0.678	0.379
4.29	3.878	3.681	3.434	3.226	3.021	2.763

3.712	3.232	3.21	2.85	1.618	1.603	1.243
4.11	3.595	3.499	3.165	3.033	2.772	2.338
4.285	3.832	3.566	3.435	3.05	2.976	2.673
4.121	3.856	3.499	3.37	2.865	2.742	2.608
4.472	4.002	3.71	3.393	3.293	2.953	2.876
4.291	4.078	3.942	3.241	3.034	2.743	2.541
3.872	3.586	2.856	2.5	2.353	2.247	1.731
3.631	3.348	3.007	2.551	2.5	2.061	2
4.09	3.674	3.153	2.971	2.933	2.754	2.476
4.02	3.232	3.205	2.852	2.414	2.165	1.58
3.491	2.977	2.598	2.01	1.204	0.16	0.124
3.377	2.274	1	0	0	0	-0.651
3.736	3.627	3.529	3.021	2.781	2.5	2.5
4.471	4.092	3.486	3.426	3.355	2.771	2.703
4.234	3.974	3.825	3.705	3.524	3.428	3.369
4.74	3.966	3.688	3.494	3.302	3.085	2.692
3.855	3.451	3.33	2.833	2.2	1.847	1.618
3.533	3.371	3.071	2.5	2.48	1.888	1.49
3.742	2.776	2.669	2.063	1	0.975	0.275
4.338	4.007	3.557	3.338	3.107	2.864	2.748
4.476	4.441	4.179	3.806	3.666	3.583	3.44
4.151	3.808	3.533	3.351	3.022	2.846	2.477
4.309	3.98	3.864	3.656	3.409	3.025	2.807
3.85	3.457	3.256	3.131	2.576	2.151	1.59
4.103	3.657	3.368	3.218	2.883	2.376	1.89
4.624	4.105	3.874	3.482	3.428	3.073	2.903
4.024	3.513	3.457	2.931	2.545	2.159	1.271
4.312	3.95	3.705	3.556	3.46	3.179	2.834
4.213	3.927	3.521	3.317	3.24	2.73	2.65
4.039	3.613	3.251	3.118	2.256	1.997	1.609
3.957	3.635	2.907	2.571	2.303	1.179	0.67
4.418	4.202	3.646	3.594	3.459	3.254	3.122
4.313	4.103	3.574	3.368	3.129	3.029	2.616
4.34	3.997	3.799	2.983	2.922	2.76	2.5
4.255	4.105	3.727	3.378	3.14	3.024	2.6
4.116	3.731	3.315	2.981	2.609	2.184	1.978
3.716	2.821	2.628	1.428	1.132	0.709	0.023
4.218	3.811	3.498	3.226	2.935	2.712	2.259
4.045	3.517	2.975	2.867	2.741	2.736	2.388
4.501	4.323	4.174	3.853	3.758	3.624	3.573
4.29	3.878	3.681	3.434	3.226	3.021	2.763
4.439	3.947	3.614	3.467	3.067	3.026	2.635
4.048	3.852	3.572	3.233	2.87	2.713	2.483
4.558	4.1	3.866	3.481	3.381	3.089	2.753
3.975	3.85	3.434	3.325	2.771	2.612	2.5
4.323	3.929	3.609	3.483	3.204	2.852	2.339
4	3.859	3.508	3.24	2.952	2.66	2.5
4.635	4.409	4.29	3.671	3.585	3.405	3.177
3.629	3.303	3.016	2.363	1.851	1.137	1
3.635	3.268	2.5	2.19	1.356	0.759	0.5

3.594	3.101	2.761	2.506	2.5	2.091	2
3.763	3.469	3.303	2.832	2.5	2.304	1.241
3.929	3.826	3.286	3.113	2.825	2.636	2.5
3.736	3.718	3.306	2.987	2.781	2.5	2.5
3.537	3.372	3.074	3.047	2.5	2.184	1.74
4.486	3.963	3.658	3.36	3.208	3.186	2.876
4.096	3.853	3.581	3.336	3.055	2.882	2.859
4.306	3.952	3.83	3.388	2.913	2.811	2.732
4.001	3.486	3.203	2.856	2.397	1.932	1.618
3.647	3.411	3.256	3.066	2.5	1.891	1.444
3.902	3.697	3.616	3.373	3.31	2.99	2.861
4.25	4.072	3.792	3.497	3.398	2.941	2.778
4.717	3.996	3.689	3.449	3.277	3.147	2.695
4.574	3.687	3.422	3.228	3.123	2.631	2.235
3.66	3.439	2.865	2.5	2.171	1.618	1.185
3.66	3.439	2.865	2.5	2.171	1.618	1.185
3.897	3.472	3.331	2.92	2.91	2.598	2.575
3.545	3.379	3.118	3.076	2.769	2.397	1.809
3.659	2.879	2.719	2.268	1.108	0.732	0
3.545	3.379	3.118	3.076	2.769	2.397	1.809
3.921	3.546	3.299	3.086	2.5	1.742	1.353
4.083	3.792	3.468	2.99	2.932	2.85	2.739
4.136	3.781	3.582	3.324	2.92	2.782	2.715
4.136	3.781	3.582	3.324	2.92	2.782	2.715
4.05	3.462	3.37	2.918	2.618	2.214	1.877
4.362	3.839	3.605	3.397	3.234	3.156	2.944
4.143	3.608	3.191	3.005	1.958	1.124	0.718
4.125	3.881	2.997	2.691	2.5	1.068	0.637
4.494	3.827	3.763	3.523	3.405	3.275	2.966
3.979	3.617	3.367	3.145	2.73	2.581	2.211
4.606	4.354	3.906	3.588	3.391	3.17	2.937
4.042	3.274	3.232	2.861	2.555	2.177	2
3.339	2.861	2.532	1.093	0.638	0.408	-0.107
4.199	3.646	3.358	3.053	3	2.844	2.5
4.646	4.307	3.667	3.569	3.457	3.169	3.077
4.126	3.513	3.192	2.749	2.443	1.399	1.195
3.928	3.868	3.533	3.364	2.871	2.654	2.5
4.061	3.829	3.145	3.005	2.5	2.208	1.12
4.363	3.837	3.651	3.079	2.916	2.589	2.5
4.141	3.929	3.824	3.29	3.286	3.095	2.825
4.326	3.833	3.146	3.005	2.565	2.5	1.32
4.171	3.861	3.784	3.54	3.316	3.173	2.774
4.302	3.86	3.445	3.104	2.644	2.476	2.202
4.537	3.65	3.366	3.145	2.717	2.223	1.613
4.116	3.696	3.327	2.821	2.5	2.218	2.118
4.196	3.998	3.571	3.168	2.966	2.93	2.665
3.767	3.714	3.359	2.808	2.723	2.532	2.146
4.047	3.908	3.439	3.41	2.79	2.632	2.5
4.379	3.727	3.245	3.048	2.692	2.284	1.669
4.282	4.007	3.841	3.127	2.867	2.53	2.5

4.132	4.057	3.776	3.401	3.255	2.886	2.725
4.094	3.551	3.232	2.547	1.481	1	0.847
4.545	3.727	3.389	3.103	2.697	2.512	2.13
4.75	4.267	4.07	3.861	3.831	3.089	3.031
3.775	3.571	2.659	2.318	1.896	1.686	0.358
3.629	3.303	3.016	2.363	1.851	1.137	1
4.161	3.336	2.874	2.805	2.768	2.517	1.711
4.103	3.401	3.273	2.672	2.582	2.058	2
4.162	3.699	3.367	2.911	2.845	2.815	2.586
3.851	3.701	3.275	3.048	2.59	2.5	2.247
3.846	3.574	3.058	2.622	2.5	1.761	1.12
3.876	3.315	3.024	2.696	2.452	2.382	1.583
4.157	3.623	3.516	3.367	3.158	2.819	2.749
4.338	4.007	3.557	3.338	3.107	2.864	2.748
4.228	3.599	3.362	3.355	2.823	2.204	1.618
4.294	3.59	3.562	3.446	3.187	2.917	2.583
2.732	2.247	1.653	0.555	0.468	0	-0.732
4.246	3.993	3.721	3.616	3.296	2.982	2.877
4.599	4.303	3.954	3.587	3.543	3.388	3.123
4.264	4.161	3.587	3.315	3.307	3.064	2.919
4.94	4.565	4.442	4.385	4.011	3.944	3.599
4.462	4.072	3.652	3.384	3.267	2.841	2.75
4.135	3.789	3.526	3.338	3.017	2.874	2.528
4.25	3.974	3.586	3.3	3.048	2.948	2.662
3.988	3.659	3.237	3.077	2.707	2.317	1.701
4.42	3.877	3.726	3.408	3.282	3.06	3.046
4.081	3.872	3.699	3.44	3.249	2.765	2.5
4.099	3.703	3.508	3.184	2.944	2.5	2.464

EEig09x	EEig10x	EEig11x	EEig12x	EEig13x	EEig14x	EEig15x
0.659	0.457	0.365	0.138	-0.202	-0.308	-0.598
1.917	1.466	1.153	0.787	0.667	0.528	0.407
2.534	2.5	2.337	1.435	1.062	1	0.965
-0.101	-0.5	-0.713	0	0	0	0
0.855	0.552	0.207	0.024	-0.14	-0.37	-0.628
0	0	-0.361	-0.762	-0.858	0	0
2.5	2.182	2.052	2	1.702	1.475	1.371
-0.302	-0.634	-0.866	0	0	0	0
2.5	2.386	1.434	1.284	0.75	0.683	0.509
1.152	0.664	0.467	0	0	-0.059	-0.5
3.001	2.957	2.62	2.02	1.96	1.845	1.683
2.731	2.431	2.322	1.948	1.618	1.571	1.368
1.986	1.711	1.102	0.901	0.616	0.549	0.336
2.445	2.096	1.825	1.49	1.268	1.205	0.828
2.5	2.044	1.751	1.019	0.813	0.636	0.5
2.499	1.968	1.739	0.813	0.665	0.622	0.5
0.539	0.5	-0.092	-0.431	-0.5	-0.756	-0.81
3.061	2.874	2.65	2.5	2.354	2.266	1.898
1.152	0.641	0.5	0.306	0	0	-0.056
2.153	1.187	1.096	0.653	0.615	0.513	0.5
2.199	1.547	1.202	1.088	0.554	0.354	0.25
2.073	1.309	1	0.824	0.5	0.255	0.079
2.103	1.77	1.141	0.75	0.562	0.5	0.436
2.487	1.79	1.179	0.813	0.651	0.5	0.418
2.5	2.37	2.112	1.673	1.618	1.085	0.806
2.422	2.296	1.781	1.593	1.444	1.129	0.513
1.661	1.366	1	0.628	0.5	0.054	0
3.322	3.216	3.117	3.025	2.917	2.835	2.398
2.477	2.065	1.369	0.829	0.674	0.556	0.5
1.767	1.388	1.061	0.67	0.5	0.055	0
2.917	2.901	2.767	2.623	2.5	2.5	2.482
3.331	3.136	3.009	2.935	2.83	2.583	2.426
1.451	0.774	0.5	0.083	0	0	0
1.581	1.322	1	0.648	0.523	0.173	0.045
3.104	2.837	2.386	2.087	1.978	1.666	1.58
2.478	2.383	2	1.954	1.618	1.263	1
2.06	1.846	1.507	1.156	1.019	1	0.605
0.106	0	-0.106	-0.33	-0.475	-0.664	-0.945
2.339	2.251	2.062	1.842	1.382	0.936	0.758
2.888	2.427	2.005	1.852	1.338	1.309	1.007
2.876	2.838	2.625	2.5	2.337	2.29	2.118
1.663	1.102	0.656	0.616	0.532	0.332	-0.107
3.445	2.922	2.862	2.756	2.283	2.099	1.756
1.854	1.049	0.846	0.559	0.441	0.43	-0.038
2.913	2.634	2.575	2.027	1.894	1.685	1.535
2.452	2.097	1.876	1	0.88	0.652	0.521
2.464	2.128	1.933	1.019	1	0.88	0.523
2.719	2.559	2.5	2.469	2.097	1.881	1.753
2.594	2.463	2.22	2.118	2.05	1.773	0.972

2.725	2.465	2.35	2.198	2.049	1.702	1.618
2.725	2.537	2.202	2.159	2.053	1.833	0.913
3.276	3.122	2.863	2.721	2.655	2.559	2.5
2.725	2.463	2.198	2.049	1.788	1.7	0.915
2.566	2.213	2.118	1.796	1.421	0.972	0.912
2.5	2.386	2.126	1.932	1.019	1	0.885
2.887	2.851	2.759	2.5	2.5	2.449	2.063
2.895	2.861	2.828	2.599	2.5	2.414	2.184
2.268	2.198	2.049	1.701	0.901	0.649	0.553
2.984	2.857	2.726	2.477	2.24	2.198	2.049
2.464	2.44	2.118	2.048	1.787	1.681	0.915
2.5	2.489	2.309	1.141	1	0.952	0.5
2.452	2.097	1.876	1	0.88	0.652	0.521
2.589	2.5	2.432	2.118	2.05	1.329	0.972
2.463	2.118	2.05	1.786	0.972	0.912	0.826
2.202	2.048	1.82	1	0.88	0.599	0.524
2.141	2.118	2	1.383	1.154	0.608	0.555
2.5	2.438	2	1.782	1.454	1.012	0.746
1	1	0.669	0.303	0.051	-0.05	-0.441
1.385	0.974	0.563	0.5	0.5	0	-0.039
1.897	1.386	1.029	0.733	0.645	0.349	0.331
1	1	0.5	0.185	-0.015	-0.332	-0.5
1.909	1.496	1.049	0.784	0.645	0.351	0.343
2.453	1.236	1	0.951	0.708	0.634	0.579
1.377	1.113	0.611	0.5	0.01	-0.077	-0.166
1.096	0.671	0.412	0.026	-0.142	-0.324	-0.416
2.5	2.239	2.016	1.806	1.038	0.862	0.789
2.573	2.419	2.102	1.432	0.984	0.882	0.774
1.734	1.169	0.727	0.615	0.448	0.236	-0.219
2.232	2	1.881	1.256	0.764	0.629	0.42
2.031	1.911	1.653	1.289	1	0.597	0.504
0.442	0	-0.103	-0.492	-0.5	-0.811	0
2.5	2.253	1.675	1.278	1.114	0.841	0.675
0.521	0.045	-0.232	-0.301	-0.474	-0.5	-0.898
2.187	2	1.621	1.37	0.9	0.794	0.56
0.167	0	-0.229	-0.802	-0.862	-1	0
1.114	1	0.5	0.5	0.04	-0.047	-0.306
1.877	1.469	1.102	0.73	0.616	0.547	0.295
2.422	2.296	1.781	1.593	1.444	1.129	0.513
2.034	1.434	1.038	0.708	0.663	0.5	0.14
1.278	1	0.788	0.543	0.258	0.218	0.044
2.753	2.628	2.523	2.281	1.773	1.258	1
1.329	1.182	0.344	0.172	-0.019	-0.205	-0.407
1.056	0.795	0.659	0.409	0.126	-0.061	-0.229
2.342	2.086	1.897	1.643	1.158	0.973	0.82
3.292	3.166	3.11	3	2.879	2.879	2.879
2.611	2.5	2.194	1.84	1.613	1.329	0.968
1.725	0.792	0.719	0.5	0.5	0.315	0
2.5	2.416	1.886	1.005	0.719	0.561	0.5
3.544	3.471	3.457	3.334	3.229	3.067	3.007

2.5	2.254	1.78	1.409	1.217	1	1
2.839	2.821	2.458	2	1.963	1.788	1.253
2.924	2.809	2.696	2.217	1.756	1.504	1.359
1.618	1.524	1	0.917	0.788	0.664	0.063
0	-0.618	-0.618	-0.618	-0.935	0	0
1.615	1.439	1.062	0.772	0.642	0.443	0.229
3.252	3.049	3.044	2.968	2.58	2.24	2.162
1.015	1	0.876	0.407	0.284	0.05	0
1.706	1.044	0.605	0.538	0.5	0.5	0.204
1.292	0.741	0.612	0.5	0.436	0.226	-0.336
2.5	2	1.867	1.59	1.413	0.844	0.616
2.5	2.378	1.176	0.991	0.735	0.5	0.5
2.356	2.143	1.85	1.382	0.884	0.834	0.675
2.005	2	1.687	1.63	0.992	0.669	0.518
2.118	2.118	1.247	0.961	0.796	0.617	0.104
1.613	0.938	0.926	0.604	0.5	-0.039	-0.049
2.133	2.058	1.264	0.862	0.688	0.661	0.17
-0.905	0	0	0	0	0	0
2.707	2.461	2.128	2	1.909	1.361	1.003
1.19	0.74	0.564	0.5	0.436	-0.056	-0.347
0	0	0	0	0	0	0
2.892	2.715	2.692	2.532	2.48	2.185	2.034
1.836	1.297	1	0.771	0.47	0.335	0.246
3.067	2.822	2.699	2.359	2.044	1.828	1.358
0	-0.658	-0.809	-1	0	0	0
1.875	1.464	1.11	0.956	0.537	0.388	0
1.239	0.879	0.616	0.215	0.081	-0.139	-0.319
0.718	0.45	0.272	0.111	0	-0.246	-0.441
2.477	1.888	1.785	1.625	1.536	1.12	0.92
2.956	2.515	2.5	2.189	2	1.759	1.231
2.613	2.5	2.427	2.186	2	1.769	1.231
2.414	2.18	1.336	1	0.794	0.763	0.688
2.5	2.492	2.1	1.474	1.156	0.853	0.647
1	0.98	0.656	0.302	0.048	-0.194	-0.445
1.903	1.56	1.375	1.025	0.677	0.361	0.133
0.45	0.395	0	-0.335	-0.5	-0.67	-0.77
3.033	2.857	2.744	2.709	2.5	2.5	2.5
2.5	2.253	1.675	1.278	1.114	0.841	0.675
2.177	1.91	1.118	0.781	0.609	0.5	0.437
2.627	2.5	2.475	2.393	2.293	2.118	1.873
1	1	0.59	-0.184	-0.414	-0.526	-0.88
0.177	-0.392	-0.414	-0.744	-0.892	-1	0
2.554	2.158	1.684	1.494	1.27	1.108	0.819
2.488	1.222	1	0.836	0.567	0.5	0.483
1.922	0.788	0.735	0.605	0.5	0.435	-0.074
0.861	0	-0.085	-0.233	-0.433	-0.756	-0.843
2.565	1.909	1.847	1.402	0.949	0.73	0.558
2.5	2.5	2.118	1.699	1.02	0.71	0.5
-0.452	-0.645	-0.656	-0.938	0	0	0
2.706	2.031	2	1.493	1.14	0.963	0.672

1	0.612	0.321	0.045	-0.232	-0.5	-0.537
2.081	2	1.644	1.148	0.849	0.691	0.498
2.198	2.139	1.442	1.229	1	0.592	0.497
2.046	1.504	1.114	0.74	0.7	0.627	0.182
2.614	2.5	2.462	2.293	2.118	1.854	1.321
2.337	2.201	1.293	1	0.922	0.718	0.603
1.11	0.555	0.5	0.47	0.374	-0.035	-0.403
1.055	0.673	0.601	0.5	0	-0.111	-0.385
2.167	1.401	1.17	0.708	0.623	0.529	0.214
1	0.838	0.579	0.19	0.045	-0.232	-0.414
-0.314	-0.64	-0.895	0	0	0	0
0	0	0	0	0	0	0
2.363	1.92	1.459	0.719	0.69	0.5	0.5
2.469	1.819	1.698	1.576	0.82	0.811	0.65
2.966	2.534	2.5	2.454	2.374	1.571	1.497
2.566	2.202	1.885	1.466	1.071	0.996	0.503
1.253	1.179	0.765	0.541	0.203	-0.161	-0.181
1.274	0.803	0.5	0.066	0	0	-0.127
0.083	-0.327	-0.53	-0.722	0	0	0
2.541	2.386	1.493	1.289	1.025	0.709	0.628
3.427	3.066	2.96	2.846	2.586	2.548	2.425
1.87	1.574	1.336	1.073	0.672	0.278	0.089
2.727	2.532	2.34	1.959	1.614	1.347	1.237
0.917	0.874	0.527	0.285	0.006	0	0
1.47	1.172	0.907	0.431	0.267	0	0
2.747	2.493	2.276	2.144	1.776	1.263	0.977
1.082	0.66	0.461	0.235	0.056	-0.298	-0.5
2.341	1.947	1.664	1.514	1.305	1.035	0.627
2.327	1.834	1.587	1.449	0.996	0.821	0.463
0.969	0.882	0.449	0.42	0.03	-0.348	-0.5
0.484	0	0	0	-0.227	-0.5	-0.605
2.835	2.69	2.568	2.535	1.867	1.629	1.277
2.435	1.516	1.481	1.26	1.181	1	0.718
1.604	1.114	1	1	0.683	0.584	0.5
2.445	2.089	1.497	1.267	1.107	1	0.728
1	1	0.708	0.303	0.081	-0.206	-0.528
-0.172	-0.345	-0.5	-0.696	0	0	0
2.154	2	1.411	1.069	0.764	0.497	0.277
1.912	1.498	1.102	0.845	0.616	0.548	0.304
3.465	3.3	3.071	2.886	2.859	2.767	2.165
2.706	2.031	2	1.493	1.14	0.963	0.672
2.002	1.884	1.567	1.524	0.88	0.764	0.708
2.225	2.118	1.624	0.978	0.755	0.607	0.403
2.531	2.522	2.5	2.272	1.772	0.908	0.813
2.023	1.504	0.825	0.702	0.683	0.5	0.181
2.025	1.689	1.504	1.425	0.947	0.707	0.347
2.5	2.33	1.802	1.313	1	0.865	0.532
2.937	2.875	2.584	2.513	2.481	2.128	2
1	0.406	-0.129	-0.303	-0.497	-0.684	-0.864
0.409	0.207	-0.5	-0.64	-0.743	0	0

2	0.777	0.5	0.495	0.096	0	0
1	1	0.625	0.5	-0.138	-0.303	-0.5
2.5	2.286	1.793	1.177	1	0.635	0.5
2.25	1	0.86	0.719	0.5	0.5	0.485
1.343	0.832	0.5	0	0	-0.004	-0.124
2.711	2.465	2.461	2.128	2	1.911	1.373
2.746	2.529	2	1.749	1.231	1.2	0.829
2.5	2.008	1.618	1.487	1	0.879	0.776
1.096	0.69	0.438	0.014	-0.106	-0.351	-0.5
1.152	0.641	0.5	0.306	0	0	-0.056
2.709	2.465	1.891	1.529	1.195	0.973	0.863
2.493	2.123	2.118	2	1.89	1.516	1.187
2.296	2.267	1.643	1.466	1.201	0.839	0.704
1.995	1.68	1.136	0.914	0.682	0.409	0.285
1.112	0.539	0.5	0.033	-0.184	-0.5	-0.579
1.112	0.539	0.5	0.033	-0.184	-0.5	-0.579
2.5	1.882	1.756	1.535	0.892	0.787	0.631
1.377	0.96	0.882	0.49	0	0	-0.005
0	-0.099	-0.5	-0.529	-0.659	0	0
1.377	0.96	0.882	0.49	0	0	-0.005
0.949	0.822	0.5	0.233	-0.066	-0.229	-0.5
2.699	2.38	2.037	2	1.41	1.275	1.141
2.329	2	1.865	1.603	0.981	0.89	0.633
2.329	2	1.865	1.603	0.981	0.89	0.633
1.591	1.097	1	0.799	0.442	0.066	0
2.748	2.665	2.339	2.178	2.143	2	1.342
0.408	0.328	0	0	-0.331	-0.363	-0.5
0.615	0.5	0.307	0	0	-0.324	-0.5
2.574	2.535	2.188	2.08	2	1.977	1.412
2.118	1.602	1.153	0.633	0.581	0.142	0
2.876	2.706	2.549	2.396	2.28	2.06	2
1.579	1	0.876	0.59	0.258	0.045	0
-0.5	-0.556	0	0	0	0	0
2.24	1.347	1.097	1	0.762	0.538	0.5
2.791	2.655	2.551	2.181	2.094	2	1.299
1	0.633	0.473	0.216	0	0	-0.481
2.172	2.118	2	1.815	1.587	1.409	0.916
1.025	1	0.5	0.406	0.016	-0.073	-0.334
2.5	2.269	1.121	1.061	1	0.698	0.5
2.634	2.5	2.5	2.5	2.426	1.986	1.278
1.119	1	0.62	0.5	0.086	-0.025	-0.221
2.516	2.5	1.724	1.25	0.985	0.76	0.537
1.726	1.52	1.286	1	0.87	0.658	0.361
1.543	1.181	1	0.632	0.567	0.13	0
1.944	0.87	0.5	0.438	0.229	-0.118	-0.195
2.407	2.155	1.803	1.541	1.097	1	0.961
1.881	1.639	1.23	0.995	0.663	0.522	0.376
2.133	1.932	1.068	0.819	0.683	0.5	0.182
1.105	0.853	0.637	0.379	0	0	-0.006
2.304	2.208	1.346	1	0.897	0.5	0.425

2.371	2	1.905	1.826	1.618	1.515	1.187
0.163	0	0	0	-0.232	-0.5	-0.528
1.699	1.496	0.869	0.798	0.415	0.172	-0.141
2.934	2.794	2.764	2.193	1.816	1.4	1.333
0	-0.057	-0.17	-0.475	-0.661	-0.944	0
1	0.406	-0.129	-0.303	-0.497	-0.684	-0.864
1.365	0.814	0.573	0.258	0.103	-0.112	-0.137
1.815	1.351	0.517	0.162	0	0	0
2.5	1.958	1.669	1.16	0.993	0.685	0.602
1.212	1.12	1	1	0.555	0.5	0.479
1	1	0.751	0.5	0.007	-0.093	-0.387
1.208	0.701	0.578	0.427	0	0	-0.306
2.532	2.464	2.053	2	1.438	1.012	0.896
2.541	2.386	1.493	1.289	1.025	0.709	0.628
1.576	1.394	0.957	0.796	0.65	0.454	0.051
2.411	2	1.972	1.775	1.667	1.517	0.933
-0.802	0	0	0	0	0	0
2.649	2.532	2.5	2.296	2.254	2.118	1.385
2.923	2.869	2.56	2.483	2.399	2.079	2
2.919	2.427	2.051	1.938	1.807	1.594	1.445
3.525	3.505	3.266	3.217	3.182	3.157	3.018
2.418	2.303	2.075	1.864	1.478	0.863	0.821
2.5	2.287	1.429	1.061	0.797	0.657	0.5
2.562	2.242	2.107	1.107	1	0.678	0.592
1.317	1.169	1	0.58	0.071	-0.041	-0.287
2.853	2.504	2.141	2	1.956	1.591	1.042
2.377	2.15	1.36	1.05	0.804	0.593	0.5
1.203	1.113	0.85	0.5	0.459	0.161	0.058

EEig01d	EEig02d	EEig03d	EEig04d	EEig05d	EEig06d	EEig07d
3.679	3.594	3.077	2.405	1.862	1.096	1.015
3.611	3.573	2.939	2.666	2.409	2.264	1.502
3.82	3.776	3.57	3.113	2.952	2.561	2.104
3.533	2.725	2.285	1	0.731	0.282	-0.235
3.672	3.005	2.713	2.341	1.766	1.334	1.273
4.064	2.791	1.939	1.493	0.43	-0.646	-1
3.941	3.776	3.314	2.782	2.527	2.195	2.135
3.644	2.894	2.059	1.498	1.121	0.514	-0.444
3.469	3.055	2.88	2.406	2.232	1.941	1.338
3.169	2.714	2.351	2.123	1.627	1.211	1.084
3.753	3.606	3.546	3.348	2.937	2.739	2.59
3.673	3.358	3.009	2.79	2.461	2.367	2.16
3.274	2.763	2.6	2.349	1.687	1.513	1.482
3.955	3.654	3.328	3.021	2.649	2.443	2.108
4.952	3.963	3.761	3.714	3.173	2.828	2.486
4.952	3.963	3.761	3.714	3.173	2.765	2.225
3.745	2.857	2.588	1.746	1.29	1	0.667
4.483	4.439	3.65	3.575	3.19	2.92	2.865
3.559	2.956	2.668	2.46	2.269	1.297	1
3.881	3.299	2.832	2.47	1.96	1.848	1.346
4.159	3.425	3.317	2.86	2.373	2.185	1.834
4.611	3.788	3.282	2.651	2.05	1.619	1.548
3.789	3.098	2.922	2.693	2.389	1.537	1.422
4.952	3.952	3.715	3.594	3.173	2.612	2.225
3.252	3.027	2.593	2.495	2.04	1.861	1.547
4.394	4.286	3.464	3.337	3.081	2.49	2.324
2.972	2.782	2.696	2.532	2.281	2.051	1.3
3.67	3.661	3.606	3.602	3.413	3.257	2.782
3.305	2.767	2.55	2.344	1.762	1.587	1.313
2.987	2.808	2.714	2.575	2.288	2.042	1.373
4.686	3.534	3.412	3.082	2.743	2.492	2.164
4.485	3.947	3.751	3.582	3.531	3.171	3.009
3.315	3.209	2.917	2.208	1.594	1.304	0.949
3.786	3.108	2.822	2.147	1.72	1.558	1.433
4.337	3.93	3.538	3.308	2.941	2.67	2.564
3.978	3.364	3.22	3.139	2.736	1.996	1.934
4.179	3.131	2.895	2.599	2.445	2.303	1.551
3.975	3.4	2.928	2.205	1.663	1.19	1.031
3.722	3.331	3.322	2.852	2.467	2.318	2.247
4.302	3.789	3.211	3.034	2.751	2.44	2.159
3.638	3.301	3.117	2.722	2.541	2.437	1.953
3.741	3.008	2.67	2.067	1.541	1.414	1.331
4.217	4.015	3.822	3.703	3.582	3.487	3.381
3.79	3.252	2.865	2.656	2.061	1.742	1.262
3.332	3.184	2.991	2.558	2.318	2.109	1.859
4.616	3.845	3.74	3.62	3.08	2.765	2.58
4.61	3.843	3.74	3.594	2.945	2.827	2.508
4.622	4.45	3.86	3.754	3.601	3.134	2.801
4.621	4.431	3.808	3.628	3.556	3.103	2.747

4.612	4.017	3.801	3.714	3.572	3.469	2.935
4.608	4.058	3.8	3.619	3.584	3.441	2.915
4.621	4.45	4.036	3.847	3.753	3.607	3.563
4.612	4.017	3.799	3.653	3.595	3.47	2.999
4.756	4.015	3.658	3.592	3.566	3.025	2.761
4.608	3.843	3.74	3.588	2.922	2.826	2.497
5.252	4.611	3.807	3.637	3.575	3.5	3.042
4.611	4.319	3.825	3.765	3.613	3.444	3.042
4.595	4.017	3.797	3.572	3.468	2.784	2.74
4.629	4.538	4.017	3.824	3.797	3.613	3.471
4.612	4.017	3.799	3.633	3.57	3.47	2.998
4.661	4.462	3.203	2.668	2.38	2.009	1.724
4.61	3.843	3.74	3.594	2.945	2.764	2.507
4.611	3.809	3.636	3.566	3.043	2.654	2.629
4.612	3.809	3.657	3.616	3.56	2.999	2.629
4.61	3.852	3.75	3.595	2.976	2.94	2.508
3.891	2.906	2.817	2.319	1.876	1.638	1.612
3.623	3.555	2.922	2.852	2.367	2.26	2.15
4.757	4.643	2.871	2.374	2.088	1.417	0.85
3.354	2.895	2.556	2.278	1.618	1.126	1
4.606	3.246	3.124	2.803	2.569	1.762	1.48
4.689	3.497	2.897	1.789	1.688	1	0.79
4.597	3.232	2.954	2.742	2.569	1.774	1.584
4.694	4.186	3.412	2.815	2.403	1.871	1.339
3.66	3.075	2.883	2.36	1.94	1.731	1.321
4.253	3.92	3.024	2.941	2.352	1.42	1.289
4.058	3.851	3.01	2.76	2.677	2.562	2.191
4.46	3.269	2.746	2.595	1.963	1.778	1.618
3.836	3.417	3.247	2.904	2.198	2.007	1.772
3.795	3.438	3.263	3.171	2.625	2.342	1.883
4.444	3.795	3.401	3.037	2.868	2.583	2.476
4.099	3.02	2.837	2.331	1	0.759	0.454
3.712	3.601	3.179	2.904	2.767	2.161	2.025
3.393	2.995	2.623	1.693	1.342	1.132	1.043
3.418	3.052	2.889	2.621	2.551	1.991	1.507
3.524	2.4	2.128	1.886	1.514	1.406	0.306
5.351	3.122	2.474	2.381	1.365	1	1
3.427	2.789	2.471	1.877	1.608	1.519	1.343
4.394	4.286	3.464	3.337	3.081	2.49	2.324
3.771	3.224	2.881	2.619	2.092	1.569	1.366
3.644	3.375	2.913	2.623	1.897	1.546	1.363
4.952	3.951	3.715	3.632	3.344	3.173	2.736
3.647	3.31	2.801	2.23	1.993	1.34	1.283
3.614	3.244	2.79	2.649	2.221	1.404	1.31
4.086	3.644	3.565	3.034	2.574	2.407	2.004
4.086	3.644	3.572	3.531	3.437	3.295	3.056
4.667	3.856	3.599	3.424	2.835	2.616	2.435
3.502	2.595	2.361	2.03	1.588	1.38	1
4.29	3.159	3.025	2.361	1.886	1.825	1.38
4.36	4.08	3.937	3.897	3.755	3.677	3.514

4.311	4.311	2.926	2.832	2.64	2.428	2.323
3.821	3.781	3.002	2.903	2.807	2.68	2.374
4.457	3.792	3.591	3.281	3.035	2.854	2.564
4.109	3.816	3.608	3.034	2.421	2.238	1.919
2.685	2.614	1.692	1.207	1.052	0.798	0.252
3.989	3.53	3.171	2.61	1.958	1.741	1.479
3.959	3.633	3.504	3.338	3.238	3.122	2.945
4.252	3.277	3.023	2.79	2.596	2.242	1.668
3.651	3.539	2.844	2.13	1.716	1.304	1.126
3.789	3.085	2.716	2.261	1.561	1.33	1.007
3.786	2.951	2.796	2.626	2.201	1.597	1.512
3.646	3.151	2.91	2.651	2.167	1.757	1.244
3.914	3.783	3.435	3.35	3.062	2.429	2.062
4.271	4.271	3.605	2.941	2.684	2.656	1.653
3.874	3.032	2.642	2.593	2.442	1.519	1.296
4.091	3.287	2.94	2.658	2.269	1.351	1.277
3.77	3.665	3.12	2.906	2.527	1.989	1.808
3.747	3.361	2.443	1.252	0.809	-0.046	-0.889
4.608	4.458	3.222	3.104	2.98	2.578	2.075
3.79	3.212	2.658	2.372	1.667	1.315	1.005
3.776	2.461	0.435	-1	-1	-1.412	0
4.649	4.61	3.701	3.343	3.335	2.7	2.455
4.693	3.604	2.927	2.759	2.422	1.643	1.41
4.09	3.752	3.622	3.52	3.137	2.808	2.674
3.681	3.211	1.62	1	0.862	0.65	0.093
4.066	3.097	2.911	2.735	2.37	2.28	1.759
3.672	3.051	2.83	2.681	1.929	1.335	1.296
3.979	3.133	2.578	2.39	2.078	1.435	0.748
3.727	3.515	3.298	3.049	2.65	2.435	2.302
4.689	3.933	3.516	3.406	2.741	2.709	2.625
4.689	3.606	3.515	2.975	2.712	2.683	2.533
3.623	3.262	3.039	2.339	2.073	2.016	1.749
4.461	3.933	3.514	3.03	2.887	2.585	1.998
4.755	4.642	2.871	2.365	1.906	1.385	0.811
4.176	3.569	3.121	2.86	2.537	2.123	1.912
3.788	2.867	2.606	1.923	1.247	1	0.437
3.573	3.072	3.043	2.87	2.687	2.462	2.335
3.777	3.626	3.194	2.906	2.773	2.191	2.053
3.803	3.785	2.828	2.679	2.578	1.728	1.386
4.199	3.125	3.053	2.849	2.667	2.418	1.905
4.697	4.69	3.487	2.634	2.175	2.111	1.658
4.694	3.498	2.6	2.175	1.962	1.387	1.182
4.524	3.946	3.663	3.278	2.877	2.721	2.48
3.698	3.507	3.196	2.454	2.085	1.909	1.583
3.79	3.579	2.754	2.21	1.944	1.324	1.256
4.519	3.747	2.877	2.715	2.606	1.701	1.307
4.799	4.271	3.275	2.978	2.645	2.171	1.533
4.057	4.057	3.509	2.558	2.481	2.232	2.031
3.865	3.548	2.422	1.603	1.077	0.469	0.359
3.819	3.551	3.302	3.143	2.787	2.493	2.364

3.634	3.079	2.68	2.147	1.558	0.955	0.76
3.853	3.515	3.329	2.76	2.602	2.512	2.155
3.812	3.552	3.306	3.015	2.724	2.475	2.196
3.797	3.327	3.011	2.716	2.51	2.002	1.624
3.462	3.119	2.788	2.553	2.407	2.162	1.889
4.759	3.591	2.997	2.747	2.362	1.992	1.798
3.735	3.117	2.697	2.138	2.128	1.862	1.469
4.203	3.057	2.656	2.002	1.801	1.254	1.077
3.57	2.889	2.852	2.482	1.756	1.625	1.379
3.781	3.103	2.75	2.147	1.56	1.521	1.431
4.03	3.162	2.156	1.743	1.613	0.677	-0.346
3.224	2.596	1.525	0.175	-0.6	-0.6	-0.81
4.323	3.117	2.865	2.361	1.876	1.38	1.331
4.952	3.951	3.715	3.629	3.173	2.957	2.555
3.777	3.592	3.521	3.176	3.038	2.529	2.197
4.304	3.997	3.403	3.322	2.769	2.606	2.269
3.614	3.14	2.784	2.711	1.789	1.56	1.459
2.971	2.725	2.544	2.282	2.063	1.291	1
3.813	2.977	1.858	1.655	1.15	1	0.3
3.49	3.198	2.94	2.662	2.394	2.077	1.642
4.507	3.748	3.709	3.371	3.07	2.82	2.692
4.1	3.158	3.034	2.749	2.473	2.232	1.876
3.817	3.549	3.324	3.192	2.983	2.618	2.367
3.669	3.307	2.801	2.617	2.373	1.745	1.395
3.381	3.243	2.912	2.631	2.327	1.934	1.427
4.952	3.951	3.715	3.624	3.173	2.986	2.616
3.782	3.378	3.01	2.503	1.965	1.473	1.163
4.306	3.403	3.074	2.878	2.628	2.534	2.356
4.336	3.666	3.256	2.876	2.558	2.354	1.834
3.789	3.039	2.743	2.422	1.618	1.255	1.074
3.765	3.652	2.675	1.987	1.739	1.156	0.669
4.046	3.663	3.197	2.878	2.831	2.425	2.223
3.964	3.78	3.648	3.21	2.76	2.388	2.125
4.312	3.782	3.169	2.609	1.916	1.799	1.421
3.941	3.778	3.652	3.223	2.749	2.391	2.149
3.845	3.781	3.327	2.662	2.206	1.715	1.108
3.215	2.873	1.732	1.611	0.722	0.468	-0.287
3.787	3.424	3.263	2.952	2.616	2.003	1.88
3.274	2.763	2.39	1.95	1.595	1.513	1.301
3.861	3.802	3.577	3.545	3.291	3.068	3.034
3.819	3.551	3.302	3.143	2.787	2.493	2.364
4.799	3.353	3.072	2.843	2.515	2.222	1.982
3.806	3.207	2.661	2.38	1.98	1.936	1.449
4.952	3.951	3.715	3.614	3.173	2.924	2.437
3.796	3.225	2.887	2.533	2.269	1.82	1.33
4.339	3.664	3.339	2.899	2.671	2.487	2.084
4.137	3.393	2.934	2.663	2.376	1.904	1.692
3.941	3.86	3.645	3.629	3.598	3.428	2.658
4.349	3.469	3.343	2.568	1.684	0.921	0.863
3.533	2.893	2.413	1.519	1	0.691	0.248

3.901	2.476	2.036	1.615	1.4	1.077	1.055
4.434	3.486	3.343	2.746	1.934	1.24	1
4.137	3.393	2.918	2.524	1.932	1.784	1.519
4.397	3.428	3.058	2.361	1.893	1.38	1.142
3.185	2.733	2.404	2.224	1.548	1.315	1
4.726	4.6	3.217	3.099	2.875	2.355	2.3
3.849	3.57	2.993	2.853	2.654	2.424	1.854
4.692	3.579	3.264	2.855	2.327	1.918	1.594
3.791	3.355	2.803	2.542	1.805	1.315	1.227
3.533	2.952	2.654	2.406	2.257	1.278	1
3.817	3.613	3.428	2.831	2.729	2.483	2.271
3.696	3.306	3.105	2.778	2.359	2.234	2.12
4.321	4.068	3.594	3.324	3.258	2.576	2.173
3.906	3.804	3.322	2.65	2.403	2.134	1.665
3.556	2.782	2.523	1.856	1.557	1	0.744
3.659	2.789	2.529	1.903	1.781	1	0.744
3.641	2.937	2.699	2.591	1.893	1.702	1.61
3.176	2.73	2.4	2.223	1.618	1.39	1.247
3.355	2.779	1.904	1.497	1	0.235	-0.533
3.176	2.73	2.4	2.223	1.618	1.39	1.247
3.239	2.906	2.385	2.226	1.905	1	0.775
4.592	3.361	3.221	2.833	2.385	2.252	1.88
3.488	3.153	2.855	2.602	2.355	1.816	1.652
3.488	3.153	2.855	2.602	2.355	1.816	1.652
4.25	3.687	2.998	2.728	2.625	2.241	1.725
3.656	3.613	3.33	2.929	2.795	2.424	2.168
4.437	3.041	2.558	1.978	1.52	0.718	0.51
4.078	2.791	2.668	1.826	1.472	1	0.141
3.841	3.306	3.031	2.931	2.822	2.711	2.337
3.186	2.846	2.576	2.298	2.04	1.333	1.225
3.972	3.695	3.643	3.468	3.156	2.576	2.442
3.786	3.108	2.82	2.147	1.672	1.557	1.432
3.609	2.682	1.543	1.311	0.471	0.106	-0.762
3.884	3.746	2.876	2.726	2.425	2.119	1.848
4.727	3.709	3.194	3.047	2.826	2.615	2.336
4.311	3.173	2.509	2.352	1.583	1.16	0.748
3.941	3.314	2.851	2.637	2.515	1.937	1.567
4.686	2.809	2.485	2.098	1.601	1	0.78
4.686	3.145	2.491	2.304	1.739	1.512	1.216
4.773	4.136	3.393	2.928	2.783	2.524	1.864
4.686	3.146	2.488	2.1	1.625	1.364	1
4.698	3.67	3.364	2.855	2.66	2.408	2.176
4.662	3.596	3.11	2.824	1.811	1.779	1.402
4.502	3.163	2.599	2.413	2.047	1.324	1.177
3.789	3.58	2.749	2.24	1.979	1.178	1.031
4.688	3.197	2.874	2.675	2.382	2.213	1.757
3.205	2.913	2.655	2.111	1.782	1.642	1.442
3.901	3.226	2.917	2.704	2.27	1.889	1.468
3.222	3.157	2.713	2.331	1.558	1.483	1.044
4.76	3.591	2.974	2.601	2.225	1.853	1.465

3.867	3.307	3.111	2.795	2.655	2.246	2.21
3.284	3.152	2.671	2.232	1.518	0.863	-0.047
3.869	3.631	3.435	2.535	2.156	1.859	1.604
4.45	3.965	3.757	3.366	3.048	2.905	2.626
3.965	3.382	2.827	1.776	1.662	1.032	0.775
4.358	3.714	3.343	2.579	1.684	1.024	0.868
3.296	3.132	2.578	1.901	1.839	1.501	1.189
3.306	3.241	2.765	2.519	2.146	1.597	1.256
3.5	3.111	2.872	2.657	2.083	1.957	1.78
4.689	3.508	2.653	2.558	1.723	1.64	1.298
4.689	3.497	2.558	1.787	1.72	1.048	1
3.891	2.83	2.598	2.033	1.619	1.413	1.176
3.87	3.184	2.881	2.823	2.665	2.274	1.71
3.491	3.206	2.942	2.671	2.395	2.079	1.65
3.387	3.077	2.555	2.537	2.182	1.949	1.549
3.926	3.333	3.288	2.845	2.773	2.453	1.752
3.722	2.137	1.247	0.859	0.384	-0.445	-0.687
3.911	3.591	3.119	2.798	2.536	2.389	1.914
3.94	3.711	3.644	3.616	3.449	3.13	2.541
4.443	3.436	3.209	3.145	2.763	2.483	2.196
4.415	4.363	3.931	3.701	3.532	3.357	3.169
3.949	3.633	3.311	2.804	2.405	2.223	1.857
3.818	3.57	3.113	2.952	2.556	1.922	1.555
4.072	3.706	3.179	2.787	2.509	1.918	1.553
3.84	3.452	3.237	2.583	2.071	1.93	1.377
3.69	3.292	3.231	2.852	2.641	2.514	2.094
3.734	3.275	2.729	2.514	2.222	2.058	1.94
3.692	3.606	3.022	2.892	2.221	1.968	1.694

EEig08d	EEig09d	EEig10d	EEig11d	EEig12d	EEig13d	EEig14d
0.6	0.39	0.146	-0.165	-0.685	-1.174	-1.367
1.353	1.174	0.782	0.58	0.345	0.144	-0.185
1.636	1.462	1.311	1	1	0.882	0.467
-1	-1.01	-1.57	-2	0	0	0
0.629	0.33	-0.099	-0.629	-0.794	-1.092	-1.299
-1	-1	-1	-1.172	-1.791	-1.848	0
1.936	1.562	1.077	1.025	1	0.986	0.75
-0.727	-1.37	-1.626	-1.765	0	0	0
1.256	1	0.998	0.596	0.3	-0.043	-0.612
0.749	0.167	-0.405	-0.667	-0.872	-1	-1.064
2.532	2.444	2.265	2.17	1.602	1.554	1.301
1.74	1.665	1.492	1.304	1.034	0.744	0.72
1.301	0.789	0.516	0.069	-0.067	-0.6	-0.788
1.81	1.654	1.461	1.346	1.244	0.804	0.493
2.225	1.86	1.353	1	0.796	0.459	0.366
1.869	1.754	1.067	1	0.795	0.459	0.348
0.072	-0.674	-0.853	-1	-1.363	-1.628	-1.767
2.566	2.282	2.192	1.7	1.634	1.324	1.079
0.803	0.688	0.109	-0.332	-0.655	-0.911	-1
1.303	1	0.839	0.601	-0.017	-0.322	-0.54
1.764	1.088	0.989	0.511	0.257	-0.386	-0.464
1.109	1	1	0.495	0.155	-0.666	-0.959
1.158	1	0.476	0.342	0.325	-0.557	-0.814
1.861	1.561	1	0.799	0.653	0.456	0.249
1.486	1.153	1	1	0.851	0.744	0.306
2.078	1.753	1.425	1.361	0.894	0.774	0.336
1	0.887	0.748	0.265	-0.257	-0.538	-0.68
2.743	2.483	2.221	2.107	1.91	1.863	1.715
1.072	1	0.72	0.454	-0.593	-0.6	-0.6
1.196	1	0.767	0.512	-0.06	-0.546	-0.661
1.991	1.818	1.791	1.574	1.437	1.224	1.084
2.676	2.557	2.218	2.21	2.196	1.89	1.85
0.837	0.25	-0.456	-0.634	-0.885	-1	-1
1.33	0.928	0.466	0.075	-0.11	-0.315	-0.656
2.407	2.243	1.952	1.61	1.335	1.127	1.006
1.43	1.383	1.21	1.146	1	0.737	0.618
1.237	1.138	0.887	0.606	0.335	0.137	0
-0.116	-0.446	-0.689	-1.025	-1.217	-1.64	-1.773
1.661	1.365	1.131	0.958	0.791	0.553	-0.07
1.836	1.604	1.419	1.393	0.912	0.557	0.415
1.814	1.748	1.414	1.283	1.2	1.021	1
0.806	0.543	0.03	-0.126	-0.802	-0.804	-0.932
3.036	2.612	2.592	2.135	1.959	1.405	1.305
1.004	0.633	0.4	0.083	-0.513	-0.782	-0.907
1.651	1.576	1.446	1.279	1.151	1.001	0.939
1.962	1.759	1.629	1.067	1	0.694	0.365
2.485	1.899	1.468	1.353	1	0.659	0.373
2.757	2.431	1.996	1.862	1.804	1.334	1.189
2.558	2.481	2.278	1.866	1.353	1.335	0.883

2.741	2.514	2.117	1.906	1.576	1.353	1.203
2.742	2.489	2.123	1.887	1.468	1.331	1.015
3.134	2.993	2.818	2.76	2.478	2.431	2.307
2.742	2.559	2.12	1.987	1.75	1.205	1.203
2.613	2.558	1.852	1.385	1.211	1.193	0.667
2.484	1.91	1.644	1.353	1.197	1	0.637
2.77	2.656	2.423	2.038	1.958	1.833	1.445
2.757	2.653	2.513	2.408	2.068	1.899	1.619
2.314	2.113	1.822	1.203	0.95	0.697	0.532
3.178	2.888	2.781	2.74	2.433	2.117	1.96
2.586	2.558	1.994	1.753	1.635	1.205	0.849
1.248	1	1	0.898	0.51	0.51	0.241
1.906	1.756	1.468	1.067	1	0.658	0.359
2.408	1.907	1.619	1.385	1.331	1	0.68
2.559	1.995	1.752	1.385	1.205	0.669	0.618
2.113	1.896	1.616	1.468	1.233	0.658	0.365
1.298	1.225	1	0.678	0.618	0.47	-0.083
1.531	1.146	1.062	1	1	0.823	0.49
0.326	0.091	-0.213	-0.867	-1	-1	-1
1	0.598	0.162	-0.6	-0.607	-0.752	-1
1.332	1.044	0.722	0.541	0.032	-0.2	-0.6
0.628	0.238	-0.193	-0.622	-1	-1	-1.253
1.366	1.044	0.685	0.489	0.026	-0.11	-0.6
1.313	1.222	0.676	0.514	-0.066	-0.182	-0.652
1	0.626	0.423	-0.247	-0.674	-0.877	-0.979
1.108	0.943	0.152	-0.185	-0.622	-0.895	-1.101
1.874	1.482	1.273	1	0.996	0.705	0.134
1.356	1.249	1.176	0.783	0.51	0.51	0.45
1.549	0.959	0.483	0.257	-0.298	-0.346	-0.674
1.827	1.371	1.171	1.146	0.553	0.385	-0.177
2.103	1.718	1.397	1.363	1.075	1.037	0.411
-0.039	-0.852	-0.962	-1	-1	-1.427	-2
1.908	1.135	1.091	1	0.702	0.469	0.391
0.173	-0.169	-0.912	-1.055	-1.235	-1.358	-1.644
1.432	1.146	0.973	0.67	0.164	0.12	-0.161
-0.063	-0.653	-1	-1.237	-1.474	-1.727	-1.83
0.674	0.056	-0.457	-0.532	-1	-1	-1
1.17	0.761	0.484	0.108	-0.2	-0.76	-0.771
2.078	1.753	1.425	1.361	0.894	0.774	0.336
1.186	1	0.708	0.359	-0.193	-0.612	-0.749
1.094	0.74	0.315	0.173	-0.141	-0.664	-0.847
2.623	2.225	1.955	1.859	1.311	1.039	0.796
0.963	0.937	0.462	-0.465	-0.621	-0.95	-1.07
0.574	0.434	0.235	0.123	-0.743	-0.882	-1.131
1.811	1.695	1.16	1.096	0.667	0.468	0.305
3.02	2.811	2.574	2.438	2.388	2.268	2.253
2.078	2.031	1.65	1.202	1.104	1	0.726
1	0.798	0.045	-0.529	-0.6	-0.634	-1
1.314	1	1	0.94	0.222	0.009	-0.529
3.301	3.218	3.072	2.954	2.765	2.432	2.222

1.541	1	1	0.943	0.801	0.683	0.347
2.185	1.678	1.525	1.359	1.077	1.045	0.86
2.496	2.463	2.206	1.761	1.359	1.234	0.921
1.799	1	0.906	0.744	0.061	-0.045	-0.255
-0.097	-0.814	-1.311	-1.532	-1.551	-1.676	0
1.122	0.786	0.654	0.356	0.126	-0.216	-0.394
2.597	2.555	2.503	2.353	2.059	1.927	1.772
0.753	0.499	0.101	-0.167	-0.577	-0.6	-1
1	1	0.559	0.023	-0.315	-0.833	-1
1	0.392	0.299	-0.452	-0.646	-0.97	-1
1.077	1.018	1	1	0.866	0.605	-0.103
1	1	0.98	0.648	0.32	0.092	-0.655
1.681	1.49	1.116	0.891	0.666	0.449	-0.098
1.621	1.296	1.169	1.149	1.083	0.51	0.51
1.198	0.618	0.618	0.484	0.235	-0.129	-0.133
1.261	1	0.35	0.014	-0.665	-0.804	-0.872
1.491	1.287	1.155	0.47	0.246	0.034	-0.263
-0.997	-1.769	0	0	0	0	0
1.763	1.578	1.428	1.353	1.146	0.917	0.735
1	0.404	0.324	-0.418	-0.832	-0.97	-1
0	0	0	0	0	0	0
2.437	2.262	1.904	1.859	1.68	1.478	1.253
1.01	0.618	0.597	0.414	-0.037	-0.355	-0.862
2.489	2.308	2.214	1.908	1.696	1.265	1.037
-0.577	-1	-1.546	-1.656	-2	0	0
1.165	1.009	0.667	0.363	0.176	-0.209	-0.409
1.267	0.645	0.437	-0.062	-0.59	-0.751	-1.007
0.481	0.404	-0.224	-0.646	-0.877	-0.962	-1
2.195	1.849	1.439	1.409	1.191	0.852	0.747
2.114	2.002	1.803	1.476	1.452	1	1
1.804	1.519	1.508	1.472	1.119	1	1
1.498	1.203	0.907	0.657	0.13	0	-0.417
1.958	1.468	1.252	1.174	1	0.51	0.51
0.265	0.049	-0.232	-0.869	-1	-1	-1
1.644	1.201	0.773	0.705	0.252	-0.131	-0.393
0.138	-0.716	-0.97	-1	-1	-1.36	-1.66
2.27	1.869	1.829	1.543	1.262	1.146	1
1.923	1.161	1.118	1	0.751	0.49	0.414
1.31	1.025	1	0.409	0.306	-0.334	-0.758
1.614	1.471	1.414	1.268	1.121	1	0.805
1.303	1.117	1	1	0.081	-0.688	-1.143
1	0.165	-0.633	-1.116	-1.315	-1.403	-1.837
2.415	2.336	1.601	1.461	1.379	0.886	0.58
1.113	1	0.555	0.378	0.289	-0.349	-0.832
1.007	1	0.389	-0.306	-0.594	-0.752	-0.97
0.814	0.37	-0.035	-0.664	-0.736	-0.976	-1.281
1.532	1.452	1.34	0.625	0.51	0.51	0.405
1.283	1	1	0.654	0.618	0.211	-0.17
-0.154	-1.051	-1.245	-1.445	-1.688	0	0
2.176	1.712	1.379	1.146	0.905	0.507	0.325

0.744	-0.011	-0.11	-0.421	-1.015	-1.28	-1.344
1.504	1.29	1.256	1.187	0.651	0.394	0.302
1.862	1.437	1.39	0.723	0.682	0.461	0.188
1.326	1.032	0.806	0.379	0.193	-0.221	-0.413
1.553	1.414	1.25	1.111	1	0.806	0.618
1.325	1.137	0.776	0.478	0.214	0.07	-0.456
1	0.469	0.306	0.24	-0.461	-0.789	-0.936
1	0.384	0.134	-0.569	-0.673	-0.784	-0.996
1.207	1.068	0.39	0.229	-0.225	-0.505	-0.755
0.928	0.242	-0.11	-0.131	-0.582	-0.967	-1.142
-0.487	-0.958	-1.284	-1.445	0	0	0
-1.31	0	0	0	0	0	0
1.026	1	1	0.895	-0.366	-0.471	-0.529
2.225	1.86	1.439	1.279	0.916	0.796	0.46
2.149	2.064	1.979	1.359	1.104	1.058	1
2.101	1.425	1.324	1.119	0.831	0.321	0.07
0.744	0.666	0.522	-0.104	-0.336	-0.442	-0.835
0.792	0.665	0.086	-0.437	-0.674	-1	-1
-0.484	-0.922	-1.466	-1.711	-1.831	0	0
1.334	1.252	0.998	0.63	0.407	0.208	-0.064
2.647	2.454	2.194	1.798	1.623	1.577	1.238
1.569	1.095	0.703	0.524	0.189	-0.181	-0.518
2.068	1.818	1.683	1.487	1.309	1.037	0.545
0.907	0.473	0.139	-0.092	-0.407	-0.6	-0.729
1.272	0.916	0.628	0.005	-0.344	-0.643	-0.83
2.225	1.868	1.754	1.59	1.348	0.833	0.794
0.491	0.371	0.09	-0.494	-0.746	-0.997	-1.297
1.895	1.558	1.069	0.818	0.499	0.486	0.167
1.573	1.358	1.152	0.823	0.614	0.073	-0.08
0.677	0.397	-0.152	-0.618	-0.724	-0.97	-1.176
-0.348	-0.6	-0.6	-0.6	-0.645	-1.019	-1.316
2.073	1.907	1.472	1.387	1.301	1.191	1.188
1.461	1.461	1.359	1.283	1	0.866	0.244
1	1	0.813	0.451	0.051	-0.529	-0.651
1.81	1.565	1.461	1.157	1	0.874	0.303
1	0.785	0.687	0.408	-0.678	-0.918	-1.171
-0.718	-1.171	-1.209	-1.577	-2	0	0
1.44	1.285	1.146	0.702	0.47	0.236	-0.274
1.297	0.772	0.448	0.069	-0.152	-0.785	-0.811
2.813	2.701	2.628	2.337	1.911	1.887	1.865
2.176	1.712	1.379	1.146	0.905	0.507	0.325
1.527	1.365	1.124	0.607	0.423	0.351	0.322
1.225	0.955	0.83	0.618	-0.011	-0.293	-0.648
2.225	1.86	1.73	1.139	1.036	1	0.795
1.035	1	0.805	0.194	-0.168	-0.404	-0.713
1.724	1.249	1.127	0.829	0.697	0.236	-0.019
1.321	1.202	1	1	0.579	0.234	0.063
2.454	2.062	1.943	1.701	1.634	1.537	1.262
0.694	0.386	-0.558	-0.634	-0.659	-1.392	-1.523
-0.191	-0.732	-1	-1.183	-1.591	-2	0

1	1	-0.273	-0.576	-0.784	-0.977	-1
0.911	0.694	0.413	-0.424	-0.634	-0.792	-1
1.321	1.056	1	1	0.489	0.23	-0.433
1	1	0.789	0.411	-0.494	-0.529	-1
0.79	0.54	-0.213	-0.656	-1	-1	-1.015
1.766	1.603	1.527	1.346	1.317	1.077	0.883
1.522	1.358	1.291	1.146	0.839	0.686	0.405
1.504	1.085	1	0.744	0.69	0.424	0.057
0.972	0.403	0.333	-0.43	-0.969	-1.041	-1.138
0.78	0.641	0.181	-0.308	-0.658	-0.966	-1
2.232	1.939	1.703	0.888	0.741	0.638	0.347
1.954	1.56	1.146	1.037	1.027	0.965	0.618
2.11	1.434	1.425	1.079	0.769	0.633	0.2
1.482	1.249	0.776	0.498	0.308	-0.137	-0.256
0.536	0.063	-0.262	-0.781	-1	-1.128	-1.344
0.643	0.102	-0.148	-0.669	-1	-1.079	-1.344
1.32	1.075	1	0.991	0.84	0.103	-0.099
0.854	0.693	-0.111	-0.618	-0.642	-0.822	-1
-1	-1	-1.121	-1.33	-1.566	-2	0
0.854	0.693	-0.111	-0.618	-0.642	-0.822	-1
0.381	-0.016	-0.305	-0.673	-1	-1.022	-1.391
1.688	1.528	1.447	1.146	1.067	0.767	0.71
1.467	1.19	1.072	0.749	0.614	-0.088	-0.328
1.467	1.19	1.072	0.749	0.614	-0.088	-0.328
1.239	1	0.76	0.698	0.053	0.003	-0.6
1.856	1.763	1.538	1.234	1.183	1.077	1.076
0.51	0.267	-0.374	-0.664	-0.956	-1.285	-1.713
-0.731	-1	-1	-1	-1	-1	-1.175
1.803	1.591	1.521	1.433	1.263	1.077	0.696
0.915	0.618	0.494	0.078	-0.454	-0.6	-0.799
2.144	2.081	1.859	1.685	1.647	1.394	1.256
1.065	0.928	0.212	-0.11	-0.111	-0.516	-0.9
-1.223	-1.617	-2	0	0	0	0
1.346	1	0.912	0.662	0.33	0.293	-0.387
2.193	1.845	1.636	1.317	1.174	1.146	1.017
0.725	-0.076	-0.435	-0.744	-1	-1	-1
1.429	1.077	1.018	1	0.815	0.618	0.613
0.437	-0.101	-0.478	-0.563	-1	-1	-1.364
1	1	0.785	0.425	-0.168	-0.488	-0.665
1.756	1.661	1.519	1.059	1	1	1
0.592	0.306	-0.441	-0.532	-0.818	-1	-1
2.035	1.158	1.031	1	0.752	0.493	0.282
1.312	1.058	0.942	0.415	0.301	-0.142	-0.473
0.904	0.505	0.349	-0.215	-0.6	-0.607	-0.905
1	0.618	0.409	-0.233	-0.824	-0.97	-1
1.434	1.366	1.251	1.097	0.804	0.335	0.219
1.148	1.055	0.77	0.343	-0.007	-0.32	-0.496
1.209	1.035	1	0.361	-0.166	-0.249	-0.62
0.867	0.005	-0.321	-0.711	-1	-1	-1
1.037	1	0.971	0.449	0.124	-0.068	-0.728

1.954	1.561	1.355	1.146	1.03	0.965	0.963
-0.07	-0.461	-0.746	-1	-1	-1.325	-1.355
1.149	0.972	0.615	0.07	-0.064	-0.354	-0.565
2.494	2.401	2.074	1.618	1.482	1.136	0.944
-0.436	-0.473	-0.938	-1.172	-1.55	-1.77	-1.882
0.694	0.393	-0.551	-0.634	-0.647	-1.391	-1.523
0.629	0.475	0.019	-0.471	-0.577	-1.006	-1.155
1.024	0.934	0.769	-0.275	-0.36	-0.589	-0.712
1.49	1.276	1.008	1	0.359	0.205	0.124
1	0.73	0.489	0.112	-0.287	-0.332	-0.413
0.639	0.26	-0.139	-0.4	-0.889	-1	-1
0.935	0.353	-0.271	-0.362	-0.514	-0.6	-0.899
1.632	1.411	1.199	1.146	1.059	0.437	0.21
1.334	1.252	0.998	0.631	0.414	0.214	-0.063
1.029	0.916	0.626	0.16	-0.305	-0.314	-0.339
1.681	1.65	1.284	1.165	1.146	0.939	0.656
-1.476	-1.802	0	0	0	0	0
1.685	1.563	1.414	1.163	1	0.813	0.783
2.419	2.062	1.936	1.699	1.615	1.5	1.206
2.175	1.971	1.49	1.234	1.127	1.008	0.936
3.104	3.038	2.776	2.616	2.54	2.498	2.335
1.781	1.404	1.282	1.225	0.664	0.462	0.307
1.321	1.272	1	0.876	0.444	0.252	-0.168
1.498	1.198	1.096	0.754	0.442	0.018	-0.18
0.989	0.946	0.811	-0.188	-0.21	-0.587	-0.971
1.641	1.573	1.544	1.146	1.099	0.793	0.634
1.347	1.052	1	0.459	0.098	-0.11	-0.6
1	0.746	0.479	0.347	0.076	-0.56	-0.686

EEig15d	EEig01r	EEig02r	EEig03r	EEig04r	EEig05r	EEig06r
-1.582	4.219	3.313	3.231	2.858	2.412	1.915
-0.643	3.999	3.496	3.361	3.073	3.044	2.855
0.285	4.291	3.857	3.656	3.276	3.151	3.021
0	3.631	3.222	2.809	2	1.066	0.313
-1.606	4.115	3.326	3.026	2.587	2.274	1.968
0	4.935	3.791	2.798	2.251	1.109	0
0.556	4.528	3.969	3.602	3.304	3.138	2.875
0	3.999	2.979	2.468	1.949	1.599	0.65
-0.68	4.249	3.843	3.453	2.963	2.901	2.696
-1.356	3.817	3.353	3.04	2.638	2.453	2
1.259	4.198	4.107	4.048	3.703	3.454	3.325
0.313	4.301	3.918	3.525	3.352	3.174	2.744
-0.811	4.188	3.639	3.204	3.078	2.556	2.414
0.294	4.367	3.706	3.662	3.12	2.779	2.747
0.15	4.576	4.015	3.88	3.359	3.183	2.885
0.108	4.576	4.013	3.87	3.206	3.122	2.526
-2	4.172	3.372	2.888	2.57	2.127	2
1	4.288	4.131	4.059	3.682	3.636	3.4
-1	3.677	3.397	3.256	3.026	2.832	2
-0.784	4.053	3.952	3.275	2.967	2.861	2.429
-0.522	4.425	3.802	3.59	3.144	2.923	2.834
-1	4.054	3.918	3.139	2.928	2.451	2.149
-0.965	4.09	3.854	3.563	3.13	2.843	2.506
0.061	4.576	3.981	3.61	3.204	3.093	2.452
0.003	3.907	3.682	3.431	3.196	2.896	2.68
-0.126	5.059	4.859	3.917	3.562	3.244	3.196
-1	3.656	3.633	3.37	3.16	2.995	2.367
1.255	4.222	4.168	4.102	4.044	3.309	3.266
-0.775	4.261	3.641	3.314	3.06	2.757	2.448
-0.844	3.636	3.369	3.163	3.128	2.992	2.452
1	4.356	4.276	4.015	3.767	3.569	3.069
1.61	4.836	4.371	4.319	3.921	3.746	3.567
-1	4.136	4.065	3.57	3.015	2.522	2.169
-0.968	4.03	3.856	3.053	2.879	2.608	2.423
0.805	5.163	4.767	4.272	4.059	3.727	3.547
0.558	4.423	3.78	3.751	3.355	2.932	2.782
-0.348	4.961	3.86	3.669	3.485	3.128	3.01
-1.886	4.289	3.538	3.288	2.59	1.946	1.472
-0.136	4.646	4.055	3.986	3.592	3.223	3.046
0.092	4.594	4.231	3.857	3.544	2.973	2.9
0.804	4.254	4.106	3.635	3.52	3.302	3.03
-1.414	4.202	3.633	3.319	2.645	2.451	2.414
1.04	5.043	4.702	4.525	4.251	4.095	3.456
-0.97	4.216	3.87	3.344	2.954	2.795	2.628
0.424	4.262	3.775	3.483	3.312	2.979	2.681
0.187	4.374	3.924	3.675	3.139	2.823	2.528
0.168	4.38	3.936	3.709	3.353	2.894	2.884
1.15	4.384	3.921	3.754	3.684	3.142	2.973
0.658	4.383	3.774	3.572	3.508	3.106	2.982

0.744	4.399	4.201	3.971	3.696	3.288	2.918
0.693	4.385	3.976	3.738	3.295	3.2	2.973
1.995	4.385	4.125	3.979	3.762	3.687	3.485
0.697	4.385	3.975	3.743	3.292	3.195	2.92
0.618	4.724	3.941	3.56	3.191	3.08	2.915
0.371	4.385	3.939	3.74	3.354	3.005	2.885
1.384	4.403	4.334	3.817	3.735	3.456	3.296
1.331	4.387	4.295	3.967	3.769	3.411	3.284
0.472	4.349	3.967	3.571	3.283	2.712	2.62
1.84	4.385	3.975	3.915	3.737	3.312	3.287
0.655	4.385	3.974	3.741	3.187	3.148	2.914
0.029	4.28	4.221	3.931	3.553	3.233	2.823
0.127	4.38	3.927	3.705	3.141	2.892	2.528
0.618	4.384	3.798	3.561	3.405	3.076	2.905
0.539	4.383	3.775	3.554	3.199	3.075	2.92
0.133	4.38	3.927	3.705	3.141	2.892	2.528
-0.287	4.113	3.437	3.262	3.085	2.652	2.458
0.27	4.113	3.484	3.383	3.206	3.084	2.82
-1.199	4.412	4.211	3.442	2.888	2.469	2.056
-1	4.004	3.377	3.126	2.972	2.478	2.03
-0.707	4.165	3.56	3.179	3.033	2.933	2.403
-1.392	4.308	3.361	3.182	2.532	2.34	2
-0.777	4.203	3.567	3.191	3.055	2.936	2.405
-0.789	4.617	4.268	3.657	3.215	2.906	2.572
-1	4.211	3.717	3.297	2.937	2.613	2.071
-1.441	3.768	3.368	2.581	2.276	2.048	1.997
0.009	4.003	3.691	3.493	3.172	3.023	2.762
-0.221	4.18	4.101	3.46	3.393	2.693	2.618
-0.914	4.261	3.797	3.471	3.177	2.515	2.413
-0.336	4.304	3.889	3.777	3.42	3.001	2.89
0.021	4.167	4.006	3.674	3.262	3.103	2.744
0	4.304	3.5	2.915	2.335	2	0.983
-0.141	4.373	3.798	3.423	3.315	3.092	2.859
-2	3.902	3.368	2.707	2.465	2.1	1.518
-0.6	4.138	3.748	3.513	3.163	2.977	2.624
0	4.415	2.921	2.47	2.063	1.902	1.381
-1.364	4.375	3.478	3.33	2.809	2.3	2
-0.902	4.149	3.633	3.129	2.632	2.52	2.414
-0.126	5.059	4.859	3.917	3.562	3.244	3.196
-0.915	4.189	3.697	3.39	2.938	2.846	2.325
-1.182	3.966	3.74	3.119	2.707	2.56	2.387
0.727	4.577	4.258	3.973	3.75	3.194	3.005
-1.303	4.149	3.669	3.237	2.679	2.451	2.011
-1.234	4.072	3.812	3.24	2.874	2.665	2.219
0.162	4.96	4.563	3.848	3.472	3.288	2.924
2.203	4.96	4.564	4.043	3.967	3.878	3.848
0.618	4.207	3.804	3.534	3.32	3.144	3.074
-1	3.979	3.278	3.135	2.716	2.317	2.154
-0.735	4.694	3.801	3.27	3.066	2.711	2.53
2.157	5.002	4.764	4.306	4.091	4.017	3.925

0.089	4.075	4.074	3.601	3.553	3.276	3.051
0.556	4.211	4.123	3.584	3.329	3.162	2.98
0.83	4.816	4.323	3.939	3.781	3.539	3.401
-0.699	4.061	3.916	3.325	3.155	2.88	2.239
0	3.427	3.372	2.416	1.581	1.553	1.497
-0.678	4.55	4.042	3.387	3.046	2.592	2.535
1.76	4.442	4.141	3.98	3.88	3.608	3.487
-1	4.123	3.645	3.177	3.128	2.61	2.15
-1	3.917	3.709	3.205	2.903	2.475	2.077
-1.121	3.937	3.562	3.243	2.753	2.396	2.182
-0.561	4.07	3.571	3.406	3.172	2.935	2.496
-0.752	4.078	3.671	3.383	3.123	2.814	2.565
-0.113	4.719	4.526	4.094	3.564	3.301	3.116
0.51	4.024	3.896	3.868	3.488	3.243	2.966
-0.712	4.463	3.572	3.424	3.274	2.813	2.279
-1	4.374	3.623	3.208	3.079	2.695	2.242
-0.673	4.267	3.893	3.45	3.264	2.95	2.411
0	3.775	3.064	2.373	1.144	0.537	-0.056
0.556	4.248	4.065	3.578	3.466	3.153	3.032
-1.12	4.048	3.801	3.17	2.782	2.457	2.125
0	4.263	2.352	0	0	0	-0.614
1.067	4.645	4.522	4.15	3.578	3.23	3.219
-1	4.309	3.898	3.527	3.147	2.801	2.266
0.837	5.031	4.535	4.069	3.674	3.464	3.262
0	4.345	3.063	2.587	2	1.419	0.856
-0.573	4.712	3.774	3.438	3.216	2.988	2.649
-1.286	4.116	3.405	3.252	3.024	2.369	1.975
-1.283	4.332	3.827	3.092	2.661	2.451	2.194
0.39	4.414	4.142	3.829	3.579	3.282	3.133
0.757	4.31	4.17	3.641	3.518	3.407	3.243
0.736	4.31	3.992	3.578	3.494	3.16	3.052
-0.637	4.207	4.009	3.49	3.072	2.984	2.868
0.462	4.181	3.965	3.384	3.287	3.226	2.859
-1.202	4.412	4.211	3.442	2.888	2.469	2.056
-0.527	4.844	3.833	3.725	3.325	3.13	2.837
-2	3.922	3.546	3.042	2.735	2	1.727
1	3.93	3.858	3.787	3.604	3.344	3.267
-0.111	4.363	3.8	3.431	3.313	3.091	2.862
-0.891	4.205	3.91	3.517	3.005	2.806	2.515
0.618	4.567	4.107	3.675	3.501	3.252	2.873
-1.315	4.12	3.446	3.135	2.841	2.245	2.083
0	4.101	3.353	2.932	2.245	2.088	1.407
0.344	4.383	3.934	3.701	3.181	3.17	2.883
-1	3.992	3.828	3.539	3.048	2.49	2.35
-1	4.045	3.833	3.259	2.855	2.471	2.199
-1.441	3.956	3.43	3.061	2.652	2.146	1.497
0.347	4.001	3.939	3.857	3.358	2.923	2.495
-0.852	4.122	3.559	3.464	3.122	2.928	2.147
0	4.025	3.349	2.367	2.146	1.832	0.862
0.142	4.386	3.959	3.667	3.42	3.238	2.898

-1.357	3.97	3.5	2.984	2.879	2.389	1.591
-0.277	4.06	3.86	3.351	3.181	2.951	2.702
-0.185	4.345	3.956	3.569	3.315	3.191	2.715
-0.766	4.178	3.702	3.544	3.01	2.925	2.362
0.514	4.114	4.082	3.585	3.306	3.059	2.858
-0.593	4.571	3.813	3.601	3.471	2.875	2.554
-1	3.834	3.67	3.266	2.588	2.063	2.035
-1	4.599	3.264	3.158	2.653	2.193	2
-0.826	4.224	3.689	3.496	3.011	2.562	2.516
-1.251	4.021	3.834	2.967	2.879	2.423	2.389
0	3.953	2.885	2.451	1.917	1.494	0.517
0	3.591	2.944	1.9	0.409	-0.1	-0.1
-0.6	4.713	3.532	3.278	3.145	2.641	2.317
0.38	4.577	4.115	3.946	3.197	3.039	2.972
0.548	4.026	3.905	3.734	3.553	3.277	3.177
-0.126	4.946	4.648	3.834	3.478	3.323	3.156
-1.147	4.042	3.468	3.351	2.948	2.433	2.037
-1.03	3.64	3.371	3.16	2.995	2.303	2
0	4.018	3.151	2.461	2.062	1.748	0.717
-0.62	4.265	3.923	3.545	3.061	2.921	2.721
1.208	4.461	4.347	3.934	3.851	3.444	3.362
-0.664	4.842	3.833	3.708	3.282	3.11	2.837
0.521	4.388	4.05	3.806	3.759	3.344	3.073
-1	4.054	3.544	3.152	3.091	2.849	2.307
-0.92	4.086	4.071	3.508	3.255	2.952	2.515
0.462	4.577	4.208	3.963	3.457	3.19	3.041
-1.548	4.228	3.624	3.214	3.114	2.425	2.065
-0.379	5.047	4.042	3.776	3.635	3.294	3.202
-0.273	5.045	3.988	3.701	3.341	3.158	2.931
-1.3	4.03	3.753	3.204	2.809	2.618	1.803
-1.595	4.24	3.649	3.281	2.525	2.112	1.967
0.51	4.686	4.065	3.812	3.457	3.265	3.086
-0.009	4.375	3.845	3.683	3.095	2.987	2.664
-1	4.115	3.842	3.515	3.306	2.534	2.292
0.078	4.348	3.758	3.687	3.296	3.044	2.687
-1.246	3.894	3.814	3.216	2.92	2.411	2.125
0	3.882	3.439	2.377	2.364	1.196	0.884
-0.567	4.267	3.884	3.511	3.228	2.977	2.459
-0.928	4.188	3.639	3.16	2.635	2.523	2.414
1.735	4.448	4.238	3.949	3.795	3.654	3.503
0.142	4.386	3.959	3.667	3.42	3.238	2.898
-0.396	4.061	3.981	3.478	3.223	3.024	2.638
-0.807	4.382	3.762	3.385	3.163	2.893	2.465
0.459	4.577	4.203	3.962	3.487	3.191	2.99
-0.934	4.148	3.598	3.516	2.965	2.854	2.322
-0.25	5.047	4.039	3.746	3.341	3.245	3.079
-0.5	4.375	3.598	3.528	3.171	2.839	2.612
1.078	4.761	4.607	4.339	4.229	3.537	3.387
-1.529	4.692	3.345	2.905	2.861	2.11	1.832
0	3.653	3.286	2.86	2.014	2	1.057

-1	4.58	3.249	2.988	2.588	2.302	2
-1.418	4.702	3.391	3.061	2.905	2.355	2
-0.664	4.375	3.535	3.524	2.85	2.818	2.48
-1	4.71	3.34	3.278	2.866	2.544	2.317
-1.306	4.018	3.368	3.089	2.947	2.544	1.972
0.718	4.417	4.137	3.62	3.504	3.188	3.042
-0.053	4.252	3.709	3.594	3.418	3.048	2.775
-0.29	4.357	3.891	3.603	3.459	3.077	2.504
-1.147	4.019	3.778	3.141	2.755	2.343	1.935
-1	3.658	3.39	3.249	3.026	2.832	2
0.238	4.231	3.705	3.57	3.483	3.181	3.032
0.205	4.12	3.961	3.708	3.469	3.157	2.916
0.03	4.946	4.622	3.838	3.426	3.21	3.164
-0.465	4.719	4.526	3.581	3.276	3.123	2.851
-1.531	3.817	3.365	3.258	2.502	2	1.974
-1.492	3.805	3.362	3.251	2.468	2	1.958
-0.112	3.981	3.52	3.313	3.044	2.569	2.501
-1.103	4.014	3.369	3.102	2.969	2.618	2.273
0	4.088	3.491	2.629	2.391	2	0.812
-1.103	4.014	3.369	3.102	2.969	2.618	2.273
-1.494	4.129	3.436	3.084	2.929	2.645	2
0.55	4.115	3.687	3.528	3.241	2.683	2.561
-0.475	4.212	3.991	3.564	3.239	3.016	2.659
-0.475	4.212	3.991	3.564	3.239	3.016	2.659
-0.611	3.75	3.499	3.177	2.895	2.694	2.408
0.747	4.092	3.984	3.594	3.423	3.109	3.053
-1.897	3.97	3.817	3.15	2.701	2.442	1.397
-1.738	4.94	3.791	3.478	2.634	2.293	1.956
0.584	4.156	4.15	3.604	3.45	3.268	3.002
-0.962	4.083	3.579	3.224	3.085	2.843	2.25
1.122	4.762	4.588	4.294	3.851	3.456	3.177
-0.999	4.03	3.855	3.043	2.879	2.543	2.423
0	3.909	2.895	2.392	2.005	0.697	0.157
-0.592	4.388	3.971	3.403	3.119	2.976	2.901
0.756	4.444	4.251	3.912	3.36	3.325	3.093
-1.281	4.082	3.791	3.237	3.073	2.379	2.087
0.199	4.528	3.61	3.478	3.234	3.135	2.437
-1.419	4.312	3.623	3.372	2.778	2.504	2
-1	4.323	3.93	3.386	3.175	2.607	2.45
0.617	4.375	3.67	3.535	3.509	2.85	2.836
-1.366	4.325	3.91	3.379	2.778	2.51	2.146
-0.13	4.338	3.709	3.412	3.38	3.163	2.861
-0.804	4.258	3.918	3.79	3.059	2.599	2.497
-1	4.158	4.041	3.284	3.109	2.843	2.241
-1.323	4.003	3.85	3.226	2.868	2.322	2
0.071	4.3	3.781	3.603	3.291	2.944	2.643
-0.829	4.078	3.358	3.28	2.931	2.707	2.305
-0.819	4.223	3.735	3.517	3.036	2.928	2.362
-1.211	4.099	4.078	3.384	3.008	2.454	2.225
-0.852	4.567	3.806	3.551	3.379	2.703	2.368

0.207	4.12	3.941	3.704	3.464	3.123	2.915
-1.657	4.077	3.856	3.274	2.816	2.282	1.337
-0.931	4.701	4.501	3.515	3.274	2.879	2.658
0.734	4.812	4.563	3.969	3.798	3.631	3.488
0	4.236	3.389	3.284	2.158	1.807	1.452
-1.529	4.691	3.264	2.905	2.82	2.08	1.828
-1.488	3.907	3.732	3.111	2.498	2.415	2.334
-1	4.072	3.95	3.285	3.108	2.385	2.202
-0.232	3.785	3.763	3.327	3.282	2.578	2.457
-1	4.309	3.551	3.395	3.027	2.601	2.497
-1.326	4.309	3.425	3.215	2.62	2.511	2
-1.117	4.54	3.504	3.176	2.802	2.427	2.37
0.149	4.105	3.839	3.414	3.177	3.063	2.701
-0.62	4.261	3.911	3.533	3.049	2.911	2.717
-0.886	4.024	3.809	3.23	2.972	2.939	2.353
0.097	4.084	3.961	3.446	3.302	3.034	2.968
0	3.718	2.49	2.247	1.568	0.555	0.056
0.667	4.117	4.091	3.597	3.413	3.258	2.852
1.077	4.758	4.583	4.239	3.885	3.455	3.296
0.933	4.583	3.854	3.764	3.359	3.118	2.856
2.038	5.08	4.764	4.399	4.298	4.056	3.765
0.006	4.671	4.202	3.734	3.394	3.006	2.972
-0.374	4.29	3.822	3.323	3.198	3.023	2.624
-0.483	4.173	3.916	3.603	3.056	2.685	2.488
-1.045	4.076	3.803	3.394	2.81	2.553	2.338
0.507	4.099	4.048	3.582	3.48	3.165	2.871
-0.601	4.22	3.651	3.605	3.312	3.048	2.809
-1	4.232	3.703	3.312	3.089	2.789	2.518

EEig07r	EEig08r	EEig09r	EEig10r	EEig11r	EEig12r	EEig13r
1.552	0.829	0.435	0.047	-0.009	-0.056	-0.453
2.037	2.028	1.719	1.287	0.878	0.483	0.368
2.478	2.355	2.093	2	1.915	1.037	0.816
0.092	0	-0.426	-0.906	-1	0	0
1.514	0.8	0.533	0.334	-0.131	-0.333	-0.569
0	0	0	-0.07	-0.456	-0.791	-0.868
2.436	2.212	2.011	2	1.984	1.831	1.549
-0.064	-0.312	-0.651	-0.945	-1.071	0	0
2.317	2	1.976	1.871	1.169	0.897	0.336
1.671	1.232	0.784	0.266	0	0	-0.085
3.212	3.013	2.944	2.907	2.437	1.923	1.864
2.639	2.488	2.311	2.27	2.072	1.591	1.463
2.289	2.228	1.739	1.457	0.816	0.655	0.162
2.48	2.351	2.139	1.902	1.634	1.244	0.966
2.417	2.318	2	1.758	1.39	0.661	0.551
2.404	2.267	2	1.672	1.37	0.551	0.338
0.93	0.497	0.125	0	-0.354	-0.696	-0.834
3.044	2.933	2.712	2.599	2.211	2.163	2.028
1.712	1.27	0.894	0.253	0.034	0	0
2.119	2	1.965	1.049	0.815	0.648	0.371
2.322	2.142	1.812	1.226	0.89	0.62	0.228
2.005	1.917	1.506	0.844	0.371	0	-0.081
2.215	2	1.736	1.321	0.674	0.296	0.073
2.4	2.264	2	1.476	0.861	0.55	0.337
2.43	2.052	2.006	2	2	1.591	1.571
2.927	2.709	2.308	1.936	1.518	1.25	1.074
2	1.744	1.556	1.213	0.824	0.431	0
3.201	3.125	2.995	2.85	2.71	2.584	2.526
2.309	2.02	2	1.664	1.243	0.395	0.195
2	1.777	1.605	1.223	0.854	0.421	0
2.883	2.756	2.51	2.446	2.35	2.293	2.087
3.492	3.356	3.066	2.959	2.723	2.502	2.434
1.837	1.316	1.236	0.408	0	0	0
2.264	2.035	1.365	1.248	0.653	0.512	0.224
3.257	3.167	3.046	2.476	2.28	2.068	1.829
2.556	2.388	2.118	1.967	1.917	1.767	1.618
2.516	2.185	1.994	1.827	1.404	1.013	1
1.295	0.395	-0.26	-0.267	-0.473	-0.618	-0.802
2.928	2.598	2.314	2.092	1.872	1.589	1.223
2.729	2.511	2.397	2.058	1.706	1.44	1.15
2.845	2.569	2.414	2.333	2.155	2	1.998
2.28	1.711	1.195	0.804	0.155	0.138	0.056
3.421	3.323	3.107	2.82	2.72	2.442	2.234
2.108	1.671	1.368	0.545	0.363	0.091	0
2.579	2.466	2.418	2.369	2.142	1.778	1.542
2.453	2.239	2	1.746	1.504	0.663	0.501
2.487	2.306	2	1.803	1.556	0.81	0.66
2.526	2.504	2.31	2.228	2.09	2	1.737
2.669	2.494	2.32	2.091	1.704	1.655	1.567

2.707	2.5	2.364	2.308	2.209	1.692	1.591
2.703	2.503	2.319	2.21	1.704	1.568	1.473
3.346	2.976	2.889	2.845	2.511	2.432	2.327
2.707	2.498	2.326	2.221	1.699	1.56	1.492
2.721	2.492	2.093	1.802	1.527	1.464	1.213
2.518	2.348	2.033	2	1.803	1.55	0.855
3.103	2.859	2.499	2.454	2.288	2.213	2
2.906	2.702	2.515	2.438	2.384	2.256	1.998
2.383	2.269	2.061	1.683	1.536	1.259	0.54
2.967	2.707	2.607	2.497	2.327	2.223	1.831
2.546	2.492	2.229	2.071	1.684	1.618	1.501
2.524	2.007	2	2	1.825	0.806	0.508
2.473	2.253	2	1.746	1.508	0.807	0.516
2.515	2.398	2.278	2.075	1.984	1.665	1.463
2.5	2.322	2.077	1.678	1.499	1.463	0.703
2.473	2.253	2	1.746	1.508	0.807	0.516
2.237	2.079	2	1.918	1.504	1.168	0.958
2.42	2.247	2	2	1.967	1.539	1.261
1.011	0.641	0.205	0	0	0	-0.312
2	1.957	1.203	0.547	0.166	0	-0.025
2.239	2.216	1.636	1.058	0.624	0.486	0.176
1.187	0.64	0.524	0.018	0	0	-0.532
2.246	2.226	1.651	1.071	0.637	0.499	0.185
2.201	2.141	1.963	0.771	0.49	0.384	0.153
2	1.695	1.078	0.686	0.208	0	-0.33
1.582	1.143	0.723	0.333	0.049	-0.282	-0.657
2.235	2	1.973	1.809	1.521	1.37	0.701
2.53	2.28	2.203	2.089	1.717	1.275	0.581
2.085	1.682	1.386	0.99	0.357	0.281	0
2.43	2.327	1.967	1.967	1.339	0.961	0.454
2.694	2.042	1.947	1.734	1.394	1.109	0.772
0.189	0	0	0	-0.538	-0.65	-1
2.765	2.176	2	1.772	1.396	0.988	0.604
1.169	0.473	0.17	-0.501	-0.617	-0.718	-0.78
2.432	2.318	1.967	1.82	1.207	0.855	0.704
0.579	0.175	0	-0.213	-0.479	-0.924	-0.939
2	0.758	0.688	0	0	0	-0.311
2.28	1.955	1.68	1.311	0.804	0.553	0.155
2.927	2.709	2.308	1.936	1.518	1.25	1.074
2.036	2	1.617	1.16	0.581	0.253	0.143
2.304	1.369	0.93	0.473	0.373	0.052	-0.068
2.728	2.707	2.416	2.316	2.134	1.811	1.426
1.765	1.535	1.158	0.688	0.117	-0.259	-0.316
2.005	1.092	0.642	0.361	0.18	-0.018	-0.227
2.886	2.762	2.187	2.069	1.649	1.564	1.097
3.517	3.431	3.263	3.1	2.987	2.922	2.825
2.733	2.443	2.182	2	1.941	1.51	1.301
2	2	1.506	0.579	0.295	0	0
2.301	2.21	2	1.956	1.661	0.911	0.278
3.753	3.577	3.32	3.214	3.112	3.075	2.89

2.994	2.103	2	2	1.61	1.13	0.947
2.765	2.703	2.492	2.355	2.25	1.984	1.714
3.027	2.667	2.588	2.52	2.262	1.88	1.501
1.984	1.591	1.402	1.034	0.648	0.481	0.252
0.909	0.493	-0.051	-0.643	-0.649	-0.7	-1.006
2.257	1.89	1.492	1.289	0.829	0.422	0.286
3.435	3.044	2.987	2.972	2.875	2.607	2.298
1.549	1.228	0.623	0.508	0.162	0	0
2	2	1.417	0.631	0.126	0.1	0
2	1.618	0.918	0.307	0.179	0	0
2.407	2	2	1.984	1.72	1.475	1.081
2.018	2	2	1.792	0.728	0.573	0.29
2.861	2.592	2.162	1.939	1.626	1.137	0.817
2.49	2.108	1.958	1.74	1.457	1.385	0.657
2.126	2.001	1.532	1.532	0.884	0.732	0.365
2	1.948	1.144	0.506	0.464	0.126	0
2.097	2.093	1.741	1.634	0.913	0.534	0.392
-0.417	-0.65	-1.069	0	0	0	0
2.536	2.374	2.298	2.234	2.013	1.967	1.665
2	1.658	0.825	0.3	0.109	0	0
0	0	0	0	0	0	0
2.977	2.903	2.849	2.674	2.6	2.477	2.23
2.182	1.618	1.519	0.866	0.333	0.011	0
3.217	3.199	2.848	2.785	2.47	2.316	2.001
0.178	0	-0.036	-0.677	-0.836	-1	0
2.453	1.74	1.626	1.285	0.884	0.697	0.307
1.521	1.477	0.8	0.565	0.392	-0.082	-0.301
1.419	1.047	0.447	0	0	0	-0.031
2.929	2.741	2.453	1.782	1.677	1.555	1.43
2.873	2.737	2.615	2.399	2	2	1.975
2.92	2.638	2.38	2.238	2	2	1.872
2.427	2.39	2.131	1.706	1.224	1	0.538
2.504	2.319	2.095	2	1.751	1.17	0.751
1.011	0.641	0.205	0	0	0	-0.312
2.471	2.217	1.66	1.46	1.105	0.834	0.462
0.919	0.165	0	0	0	-0.41	-0.735
3.087	2.861	2.731	2.412	2.282	2.207	2
2.762	2.17	2	1.779	1.399	0.986	0.604
2.208	2	1.776	1.603	0.733	0.369	0.163
2.785	2.414	2.384	2.316	2.14	2	1.782
1.422	0.725	0.371	0	0	-0.384	-0.445
0.605	0.006	0	-0.445	-0.539	-0.828	-1.009
2.573	2.533	2.311	2	1.485	1.284	0.983
2.111	2	1.889	0.804	0.512	0.338	0.084
2	1.745	1.609	0.342	0.279	0.169	0
1.164	0.868	0.465	-0.17	-0.322	-0.505	-0.665
2.45	2.375	1.99	1.643	1.295	1.08	0.563
2	2	1.61	1.531	1.467	1.119	0.459
0.323	0.042	-0.516	-0.673	-0.763	-0.995	0
2.745	2.511	2.286	1.967	1.562	1.149	0.935

1.415	1.076	0.653	0.229	0.215	-0.254	-0.532
2.398	2.08	1.952	1.823	1.445	0.825	0.439
2.67	2.328	2.096	1.651	1.141	0.955	0.701
2.287	2.125	1.583	1.094	0.607	0.295	0.234
2.509	2.414	2.365	2.136	2	1.783	1.685
2.336	2.061	1.845	1.582	0.925	0.459	0.411
2	1.424	0.829	0.175	0.139	0	-0.002
1.984	1.833	0.936	0.394	0.223	0	-0.049
2.288	2.067	1.983	1.131	0.967	0.457	0.204
2.035	1.331	0.723	0.653	0.224	0.131	-0.252
-0.082	-0.485	-0.688	-0.988	-1.072	0	0
-0.528	-0.917	0	0	0	0	0
2.144	2	2	1.751	1.202	0.48	0.295
2.424	2.354	2.158	1.549	1.351	1.27	0.555
2.964	2.881	2.555	2.227	2.017	2	1.912
2.998	2.503	2.215	1.971	1.316	1.129	1.015
1.591	1.514	1.056	0.902	0.399	0.299	-0.089
1.713	1.332	1.106	0.52	0	0	-0.067
0.063	0	-0.338	-0.754	-0.932	-0.996	0
2.383	2.295	1.981	1.844	1.237	0.886	0.608
3.214	3.065	2.954	2.589	2.541	2.42	2.155
2.471	2.21	1.619	1.459	1.056	0.805	0.446
2.747	2.523	2.495	2.384	2.24	1.459	1.305
1.994	1.342	0.707	0.504	0.112	0.062	0
2.192	1.73	1.304	0.944	0.638	0.205	0
2.61	2.439	2.401	2.26	2.014	1.731	1.438
1.664	0.986	0.793	0.231	-0.003	-0.122	-0.172
3.066	2.474	2.221	1.727	1.585	1.251	1.107
2.329	2.26	2.121	1.574	1.392	1.247	0.667
1.666	1.307	0.571	0.382	0	-0.021	-0.325
0.741	0.237	-0.001	-0.1	-0.1	-0.1	-0.492
2.983	2.834	2.324	2.224	2.186	2.035	1.662
2.618	2.103	2	1.358	1.173	0.903	0.741
2.234	2	1.245	0.743	0.235	0.149	0
2.536	2.135	2	1.886	1.284	0.972	0.69
1.663	1.381	0.531	0.405	0	-0.063	-0.325
0.289	-0.286	-0.488	-0.506	-0.85	-1	0
2.425	2.014	1.967	1.645	1.092	0.868	0.35
2.289	1.97	1.682	1.326	0.816	0.571	0.162
3.299	3.179	3.148	2.94	2.816	2.493	2.416
2.745	2.511	2.286	1.967	1.562	1.149	0.935
2.491	2.038	1.653	1.536	1.203	1.155	0.62
2.283	2.084	1.823	1.554	1.284	0.737	0.322
2.656	2.416	2.316	2.045	2	1.803	1.419
2.126	2	1.546	1.091	0.377	0.257	0.225
2.508	2.218	1.79	1.473	1.373	1.237	0.679
2.442	2.109	2	2	1.51	0.945	0.806
3.163	3.006	2.893	2.838	2.559	2.504	2.425
1.022	0.658	0.443	0.398	-0.4	-0.494	-0.508
0.464	0.099	0	-0.044	-0.815	-0.975	-1

2	1.984	1.982	0.607	0.285	0	0
1.976	1.089	0.658	0.445	0.195	0	-0.426
2.347	2.04	2	2	1.495	0.806	0.789
2	2	1.875	0.585	0.453	0.295	0.03
1.823	1.477	1.115	0.443	0	-0.017	-0.083
2.79	2.408	2.3	2.241	2.079	2.026	1.984
2.45	2.395	2.268	2.008	1.967	1.38	1.025
2.424	2.212	2	1.84	1.591	1.144	0.45
1.609	1.591	0.658	0.228	0	-0.3	-0.458
1.71	1.263	0.869	0.233	0.09	0	0
2.934	2.678	2.461	2.199	1.656	1.198	1.116
2.557	2.41	2.163	1.967	1.636	1.618	1.536
2.751	2.504	2.214	1.79	1.235	1.109	0.966
2.595	2.159	1.936	1.618	1.087	0.816	0.562
1.591	1.062	0.729	0.396	0	-0.221	-0.43
1.591	1.037	0.728	0.376	0	-0.227	-0.46
2.441	2.125	2	1.751	1.499	1.326	0.619
1.979	1.598	1.176	0.562	0.382	0.055	0
0.343	0	0	-0.478	-0.642	-0.834	-1
1.979	1.598	1.176	0.562	0.382	0.055	0
1.544	1.025	0.47	0.428	0.017	0	-0.537
2.476	2.408	2.263	1.998	1.967	1.783	1.15
2.486	2.387	1.996	1.634	1.446	1.298	0.585
2.486	2.387	1.996	1.634	1.446	1.298	0.585
1.784	1.605	1.247	0.834	0.599	0.208	0
2.806	2.661	2.492	2.172	1.995	1.984	1.975
0.712	0.3	-0.058	-0.095	-0.3	-0.3	-0.85
0.787	0	0	0	0	-0.022	-0.063
2.973	2.533	2.428	2.207	2.093	1.984	1.858
2.08	1.785	1.532	1.374	0.833	0.235	0.145
3.12	2.886	2.824	2.518	2.413	2.296	2.069
2.039	1.983	1.328	0.792	0.653	0.225	0.194
-0.018	-0.482	-0.755	-1	0	0	0
2.429	2	1.967	1.22	0.955	0.838	0.492
2.835	2.692	2.487	2.312	2.043	1.967	1.965
1.267	0.86	0.227	0	0	0	-0.089
2.266	2	1.99	1.984	1.642	1.47	1.441
1.72	0.785	0.661	0.142	0	0	-0.325
2.086	2	2	1.767	0.774	0.657	0.258
2.711	2.347	2.23	2.011	2	2	2
2	1.12	0.726	0.33	0	0	-0.194
2.78	2.2	2	1.964	1.408	0.922	0.527
2.216	2.05	1.461	1.313	1.221	0.466	0.207
1.805	1.416	1.33	0.82	0.201	0.122	0
1.742	1.511	1.455	0.468	0	0	-0.189
2.488	2.225	2.152	1.939	1.535	1.327	0.867
2.117	1.911	1.677	1.302	1.093	0.718	0.27
2.184	2	1.646	1.483	0.614	0.375	0.238
1.758	1.46	0.732	0.477	0	0	-0.051
2.143	1.958	1.673	1.637	0.947	0.574	0

2.548	2.405	2.152	1.967	1.62	1.53	1.508
0.588	0.457	0	0	-0.069	-0.119	-0.604
2.477	2.051	1.662	1.172	0.85	0.693	0.269
2.801	2.657	2.587	2.537	2.338	1.862	1.513
1.197	-0.023	-0.267	-0.47	-0.562	-0.713	-1.017
1.006	0.658	0.401	0.237	-0.461	-0.494	-0.533
1.972	1.421	1.036	0.435	0.172	0.035	-0.219
1.952	1.703	1.642	1.138	0.334	0	0
2.414	2.262	2	1.76	1.419	0.913	0.709
2.237	2	1.144	0.779	0.578	0.521	0.296
1.662	0.793	0.678	0.504	0	0	-0.238
2.104	1.294	1.155	0.431	0.25	0.121	-0.028
2.439	2.357	2.146	2.016	1.967	1.741	1.046
2.36	2.287	1.977	1.871	1.225	0.898	0.543
1.97	1.335	1.27	1.131	0.628	0.392	0.24
2.466	2.438	2.112	1.967	1.718	1.382	1.33
-0.064	-0.769	-0.802	0	0	0	0
2.738	2.514	2.414	2.171	2.001	2	1.779
3.163	3.004	2.871	2.835	2.542	2.43	2.357
2.521	2.521	2.433	2.089	1.719	1.573	1.463
3.662	3.326	3.179	3.163	3.098	3.021	2.867
2.472	2.298	2.217	2.126	1.821	1.568	1.36
2.397	2.105	2	1.905	1.033	0.816	0.44
2.304	2.097	1.987	1.725	1.219	0.904	0.546
1.895	1.406	1.16	0.547	0.523	0.227	-0.218
2.665	2.516	2.471	2.066	1.967	1.934	1.533
2.261	2.096	2	1.683	1.018	0.7	0.404
2	1.959	0.918	0.637	0.556	0.038	0

EEig14r	EEig15r	ESpm01u	ESpm02u	ESpm03u	ESpm04u	ESpm05u
-0.577	-0.858	0	3.85	3.611	5.442	5.994
0.133	0.091	0	4.143	3.761	5.645	5.994
0.587	0.22	0	4.394	4.111	6.023	6.524
0	0	0	3.367	2.944	4.796	5.017
-0.708	-0.994	0	3.85	3.434	5.425	5.861
0	0	0	3.761	4.29	5.861	7.013
1.365	1.07	0	4.489	4.205	6.184	6.804
0	0	0	3.434	2.944	4.99	5.398
0.24	0.072	0	4.29	3.892	5.922	6.382
-0.298	-0.769	0	3.807	3.219	5.242	5.442
1.717	1.527	0	4.812	4.635	6.491	7.074
1.268	1.236	0	4.511	4.111	6.12	6.581
0.123	-0.013	0	4.143	3.611	5.7	6.043
0.82	0.575	0	4.369	4.007	5.989	6.494
0.337	0	0	4.443	4.443	6.24	7.004
0.237	0	0	4.394	4.369	6.192	6.957
-0.998	-1	0	3.761	3.434	5.371	5.889
2	1.701	0	4.796	4.745	6.535	7.216
-0.085	-0.269	0	3.892	3.434	5.323	5.525
0.126	0	0	4.174	3.761	5.771	6.196
0.162	-0.005	0	4.29	4.111	5.994	6.608
-0.151	-0.407	0	4.111	3.761	5.733	6.236
0	0	0	4.205	3.892	5.814	6.275
0.035	0	0	4.344	4.29	6.125	6.868
0.892	0.587	0	4.317	3.611	5.79	5.969
0.972	0.416	0	4.673	4.934	6.703	7.728
0	0	0	4.078	3.611	5.541	5.771
2.427	1.839	0	4.905	4.691	6.567	7.14
0.09	0	0	4.205	3.761	5.79	6.217
0	0	0	4.043	3.434	5.434	5.525
2	1.917	0	4.796	4.635	6.518	7.163
2.317	2.28	0	5.069	5.056	6.903	7.683
-0.086	-0.248	0	4.078	4.007	5.79	6.447
0.123	-0.248	0	4.078	3.611	5.631	6.019
1.607	1.428	0	4.949	5.198	6.974	7.959
1.129	0.718	0	4.466	4.111	6.12	6.648
0.882	0.283	0	4.489	4.443	6.365	7.179
-1.018	-1.073	0	3.85	3.761	5.572	6.256
0.761	0.654	0	4.575	4.575	6.358	7.116
0.891	0.612	0	4.554	4.443	6.347	7.057
1.783	1.732	0	4.654	4.205	6.271	6.711
-0.024	-0.414	0	4.043	3.611	5.652	6.089
1.932	1.613	0	5.03	5.293	7.029	8.003
-0.086	-0.407	0	4.143	3.892	5.79	6.312
1.372	1.234	0	4.489	3.892	6.043	6.365
0.237	0.139	0	4.344	4.111	6.033	6.635
0.514	0.167	0	4.394	4.205	6.089	6.698
1.516	1.379	0	4.595	4.29	6.26	6.815
1.384	0.701	0	4.533	4.205	6.176	6.698

1.542	1.271	0	4.654	4.511	6.399	7.074
1.315	0.587	0	4.533	4.29	6.201	6.781
2.239	2.09	0	4.949	4.691	6.624	7.186
1.258	0.616	0	4.533	4.29	6.201	6.781
0.705	0.559	0	4.489	4.369	6.256	7.004
0.661	0.521	0	4.443	4.205	6.125	6.711
1.984	1.686	0	4.762	4.635	6.494	7.155
1.984	1.655	0	4.779	4.635	6.503	7.163
0.329	0.227	0	4.369	4.111	6.047	6.635
1.689	1.542	0	4.745	4.511	6.422	6.995
1.257	0.614	0	4.489	4.205	6.151	6.723
0	0	0	4.466	4.511	6.26	7.022
0.237	0.167	0	4.344	4.111	6.033	6.635
1.075	0.669	0	4.489	4.111	6.125	6.621
0.559	0.485	0	4.394	4.111	6.043	6.594
0.237	0.167	0	4.344	4.111	6.033	6.635
0.521	0.451	0	4.205	3.434	5.687	5.889
0.642	0.48	0	4.344	3.761	5.855	6.111
-0.543	-0.859	0	4.078	4.29	5.969	6.847
-0.1	-0.319	0	3.97	3.434	5.468	5.74
-0.02	-0.1	0	4.174	3.761	5.759	6.155
-0.629	-0.881	0	3.85	3.761	5.541	6.236
-0.012	-0.1	0	4.174	3.761	5.759	6.155
0.109	0	0	4.344	4.443	6.209	7.057
-0.406	-0.504	0	4.078	3.761	5.714	6.217
-0.757	-0.832	0	3.714	2.944	5.13	5.303
0.504	0.366	0	4.29	3.761	5.832	6.133
0.467	0.382	0	4.369	4.111	6.028	6.594
-0.11	-0.453	0	4.143	3.892	5.814	6.365
0.347	0.137	0	4.394	4.205	6.08	6.648
0.58	0.292	0	4.394	4.205	6.052	6.635
-1.038	0	0	3.714	3.761	5.451	6.217
0.425	0.239	0	4.369	4.111	6.019	6.553
-1	-1.057	0	3.714	3.219	5.242	5.602
0.317	0.114	0	4.317	3.892	5.916	6.293
-1.053	0	0	3.611	3.434	5.342	6.089
-0.342	-0.782	0	3.97	3.892	5.707	6.431
0.119	-0.024	0	4.078	3.434	5.631	5.943
0.972	0.416	0	4.673	4.934	6.703	7.728
0	-0.232	0	4.143	3.761	5.753	6.196
-0.203	-0.507	0	4.007	3.611	5.557	5.943
0.879	0.551	0	4.654	4.635	6.457	7.216
-0.426	-0.688	0	4.007	3.611	5.631	6.111
-0.428	-0.633	0	4.007	3.761	5.617	6.111
0.843	0.812	0	4.635	4.745	6.567	7.479
2.806	2.805	0	5.263	5.231	7.077	7.844
1.115	0.639	0	4.466	4.111	6.066	6.509
-0.054	-0.1	0	3.932	3.219	5.389	5.565
0.11	0.04	0	4.29	4.111	6.014	6.746
2.806	2.654	0	5.303	5.407	7.195	8.068

0.605	0.264	0	4.443	4.369	6.116	6.723
1.563	1.001	0	4.595	4.205	6.221	6.673
1.282	0.96	0	4.745	4.796	6.594	7.409
0	-0.369	0	4.111	3.892	5.733	6.275
0	0	0	3.367	2.565	4.727	4.796
0.092	-0.031	0	4.234	4.111	6.014	6.735
2.132	2.044	0	4.99	4.745	6.693	7.252
-0.1	-0.403	0	4.007	3.892	5.617	6.196
0	-0.059	0	3.97	3.434	5.468	5.74
-0.215	-0.427	0	3.932	3.434	5.442	5.74
0.609	0.254	0	4.263	3.611	5.778	6.019
0	0	0	4.205	3.761	5.765	6.133
0.635	0.507	0	4.595	4.745	6.503	7.378
0.388	0.213	0	4.419	4.369	6.12	6.77
0.338	-0.261	0	4.234	4.007	5.922	6.553
-0.396	-0.406	0	4.078	3.761	5.727	6.256
0.227	-0.125	0	4.263	4.007	5.927	6.479
0	0	0	3.219	2.944	4.727	5.142
1.116	0.817	0	4.554	4.29	6.229	6.781
-0.354	-0.51	0	3.97	3.611	5.549	5.994
0	0	0	2.944	3.434	4.844	5.889
2.108	1.925	0	4.875	4.844	6.673	7.462
0	-0.041	0	4.174	4.111	5.889	6.581
1.662	1.311	0	4.86	4.977	6.753	7.645
0	0	0	3.497	3.434	5.242	5.994
0.115	-0.067	0	4.317	4.205	6.111	6.836
-0.565	-0.677	0	3.97	3.611	5.565	6.019
-0.509	-0.561	0	3.932	3.892	5.631	6.347
1.018	0.836	0	4.595	4.511	6.333	7.004
1.527	1.123	0	4.635	4.443	6.304	6.899
1.452	0.943	0	4.575	4.29	6.209	6.735
0.383	0.201	0	4.344	4.007	5.984	6.463
0.401	0.262	0	4.394	4.205	6.033	6.608
-0.543	-0.859	0	4.078	4.29	5.969	6.847
0.093	-0.101	0	4.419	4.369	6.267	7.048
-0.91	-1	0	3.714	3.434	5.242	5.673
1.967	1.961	0	4.727	4.205	6.271	6.538
0.433	0.237	0	4.369	4.111	6.019	6.553
0	0	0	4.205	3.892	5.838	6.347
1.618	1.591	0	4.635	4.29	6.326	6.919
-0.63	-0.985	0	3.892	3.611	5.485	5.994
-1.116	0	0	3.714	3.434	5.323	5.861
0.711	0.474	0	4.443	4.205	6.107	6.673
0	0	0	4.111	3.761	5.68	6.111
0	-0.393	0	4.007	3.611	5.572	5.994
-0.988	-1.068	0	3.807	3.434	5.38	5.802
0.202	0.051	0	4.369	4.205	6.047	6.635
0.194	0	0	4.174	3.611	5.733	6.066
0	0	0	3.497	3.219	5.081	5.638
0.697	0.452	0	4.489	4.29	6.184	6.758

-0.691	-0.721	0	3.807	3.434	5.342	5.74
0.277	0.15	0	4.29	3.892	5.866	6.275
0.535	0.12	0	4.394	4.205	6.08	6.661
0.153	-0.171	0	4.205	3.892	5.826	6.293
1.591	1.146	0	4.511	4.007	6.103	6.509
0.11	0.091	0	4.344	4.29	6.116	6.858
-0.349	-0.507	0	3.892	3.434	5.398	5.74
-0.245	-0.571	0	3.97	3.761	5.707	6.431
0.097	-0.053	0	4.205	3.761	5.826	6.236
-0.478	-0.532	0	3.97	3.611	5.549	5.994
0	0	0	3.434	2.944	4.99	5.398
0	0	0	3.045	2.944	4.489	5.017
0.022	0	0	4.205	4.007	5.938	6.673
0.431	0.329	0	4.466	4.443	6.275	7.04
1.342	1.093	0	4.595	4.29	6.18	6.608
0.901	0.4	0	4.615	4.796	6.575	7.518
-0.409	-0.604	0	3.97	3.611	5.533	5.969
-0.122	-0.376	0	3.85	3.219	5.252	5.352
0	0	0	3.497	3.219	5.081	5.638
0.293	0.174	0	4.344	4.007	5.994	6.494
2.119	1.84	0	4.875	4.745	6.6	7.245
0.069	-0.141	0	4.419	4.369	6.267	7.048
1.289	0.936	0	4.615	4.443	6.33	6.929
-0.1	-0.186	0	4.007	3.761	5.557	6.019
0	-0.054	0	4.205	4.111	5.883	6.494
0.92	0.554	0	4.595	4.511	6.382	7.107
-0.565	-0.833	0	3.97	3.761	5.624	6.176
0.898	0.392	0	4.615	4.691	6.529	7.403
0.501	0.148	0	4.489	4.511	6.405	7.287
-0.487	-0.785	0	3.932	3.611	5.525	5.969
-0.81	-1	0	3.85	3.892	5.572	6.312
1.389	1.077	0	4.673	4.511	6.428	7.099
0.399	0.191	0	4.317	4.111	5.989	6.581
0	0	0	4.143	4.007	5.802	6.382
0.395	0.368	0	4.344	4.111	5.994	6.553
-0.57	-0.817	0	3.932	3.611	5.493	5.943
0	0	0	3.497	3.219	5.056	5.525
0.139	0.018	0	4.29	4.007	5.943	6.463
0.121	-0.016	0	4.078	3.434	5.631	5.943
2.265	2.02	0	4.949	4.844	6.666	7.307
0.697	0.452	0	4.489	4.29	6.184	6.758
0.282	0.238	0	4.317	4.007	5.948	6.431
0.23	0.052	0	4.263	3.892	5.916	6.431
0.623	0.545	0	4.575	4.511	6.365	7.107
0	-0.175	0	4.143	3.761	5.74	6.155
0.456	0.046	0	4.511	4.575	6.441	7.334
0.47	0.079	0	4.29	3.892	5.911	6.399
2.103	1.984	0	4.99	5.056	6.878	7.728
-0.717	-0.878	0	3.85	3.892	5.687	6.567
0	0	0	3.497	2.944	4.92	5.081

-0.006	-0.049	0	4.007	3.611	5.714	6.365
-0.494	-0.707	0	3.97	3.892	5.771	6.581
0.189	0.02	0	4.234	3.761	5.844	6.312
0	0	0	4.111	3.892	5.866	6.608
-0.26	-0.63	0	3.932	3.434	5.442	5.771
1.666	1.117	0	4.615	4.369	6.308	6.909
0.882	0.353	0	4.419	4.007	6.028	6.447
0.335	0.006	0	4.369	4.205	6.066	6.698
-0.615	-0.691	0	3.932	3.611	5.525	6.019
-0.085	-0.274	0	3.892	3.434	5.323	5.525
0.805	0.645	0	4.466	4.205	6.057	6.524
1.225	0.794	0	4.489	4.111	6.116	6.567
0.763	0.475	0	4.575	4.745	6.535	7.479
0.214	0.097	0	4.443	4.511	6.33	7.186
-0.691	-0.747	0	3.761	3.219	5.231	5.485
-0.691	-0.75	0	3.761	3.219	5.231	5.485
0.445	0.4	0	4.174	3.434	5.624	5.771
-0.087	-0.299	0	3.97	3.434	5.468	5.74
0	0	0	3.611	3.434	5.22	5.802
-0.087	-0.299	0	3.97	3.434	5.468	5.74
-0.632	-0.745	0	3.932	3.611	5.525	5.969
0.962	0.81	0	4.394	3.761	5.943	6.217
0.439	0.381	0	4.369	4.007	6.019	6.479
0.439	0.381	0	4.369	4.007	6.019	6.479
-0.1	-0.161	0	3.97	3.434	5.398	5.602
1.937	1.033	0	4.554	4.111	6.111	6.479
-0.967	-1	0	3.892	3.892	5.609	6.275
-0.455	-0.791	0	4.007	4.369	6.009	7.066
1.571	1.099	0	4.595	4.205	6.221	6.661
-0.1	-0.17	0	4.111	3.611	5.652	5.994
1.952	1.946	0	4.905	4.89	6.765	7.581
-0.016	-0.252	0	4.043	3.611	5.609	6.019
0	0	0	3.296	2.944	4.812	5.252
0.126	0.071	0	4.317	4.111	6.028	6.621
1.907	0.961	0	4.673	4.511	6.402	7.057
-0.211	-0.646	0	3.932	3.892	5.587	6.217
1.065	0.787	0	4.369	4.007	6.038	6.621
-0.468	-0.775	0	3.97	3.892	5.68	6.365
0	0	0	4.234	4.007	5.922	6.538
1.587	0.861	0	4.489	4.007	6.089	6.524
-0.327	-0.576	0	4.043	4.007	5.784	6.509
0.393	0.042	0	4.369	4.111	5.999	6.509
0.177	0	0	4.234	4.111	5.943	6.608
-0.1	-0.207	0	4.111	4.007	5.808	6.447
-0.391	-0.604	0	3.97	3.611	5.549	6.019
0.722	0.349	0	4.369	4.111	6.009	6.567
0.202	0.1	0	4.078	3.434	5.572	5.832
0	-0.174	0	4.205	3.892	5.838	6.33
-0.074	-0.405	0	4.078	4.007	5.778	6.431
-0.023	-0.056	0	4.29	4.205	6.061	6.804

1.225	0.794	0	4.489	4.111	6.116	6.567
-0.724	-0.97	0	3.85	3.892	5.541	6.217
0.045	-0.167	0	4.369	4.443	6.267	7.132
1.161	0.921	0	4.762	4.89	6.661	7.534
-1.073	0	0	3.761	3.611	5.46	6.111
-0.745	-0.888	0	3.85	3.892	5.687	6.567
-0.656	-0.678	0	3.97	3.434	5.517	5.832
-0.006	-0.1	0	4.111	3.892	5.746	6.312
0.379	0.19	0	4.205	3.611	5.673	5.889
0	0	0	4.111	3.892	5.759	6.347
-0.289	-0.646	0	3.892	3.761	5.565	6.236
-0.1	-0.442	0	4.043	3.892	5.771	6.479
0.657	0.43	0	4.394	3.892	5.964	6.312
0.287	0.162	0	4.344	4.007	5.994	6.494
0.151	-0.279	0	4.111	3.761	5.707	6.133
1.231	0.725	0	4.419	4.007	6.009	6.431
0	0	0	3.045	2.565	4.443	4.796
1.591	1.159	0	4.533	4.111	6.116	6.553
2.052	1.984	0	4.949	4.934	6.79	7.586
1.237	1.183	0	4.533	4.29	6.225	6.858
2.846	2.627	0	5.342	5.352	7.236	8.084
0.713	0.533	0	4.533	4.369	6.33	7.031
0.219	0.099	0	4.263	3.892	5.872	6.33
0.133	0.094	0	4.263	3.892	5.894	6.382
-0.305	-0.458	0	4.043	3.761	5.666	6.176
1.375	0.705	0	4.533	4.111	6.142	6.553
0.12	0	0	4.29	4.007	5.911	6.399
-0.086	-0.352	0	4.111	3.892	5.733	6.275

ESpm06u	ESpm07u	ESpm08u	ESpm09u	ESpm10u	ESpm11u	ESpm12u
7.382	8.288	9.524	10.584	11.77	12.894	14.066
7.407	8.107	9.325	10.212	11.347	12.334	13.436
7.958	8.815	10.07	11.093	12.283	13.384	14.554
6.444	6.984	8.204	8.924	10.032	10.857	11.904
7.328	8.165	9.43	10.447	11.636	12.731	13.894
8.396	9.704	11.066	12.417	13.782	15.146	16.516
8.262	9.258	10.556	11.706	12.961	14.174	15.424
6.88	7.689	8.947	9.928	11.095	12.149	13.28
7.87	8.724	10.005	11.038	12.244	13.356	14.538
6.942	7.537	8.794	9.6	10.739	11.657	12.741
8.456	9.357	10.58	11.62	12.79	13.891	15.045
8.048	8.901	10.168	11.198	12.394	13.503	14.677
7.571	8.34	9.633	10.609	11.808	12.88	14.045
7.953	8.852	10.127	11.198	12.414	13.561	14.762
8.401	9.467	10.753	11.939	13.202	14.439	15.704
8.368	9.439	10.732	11.923	13.19	14.43	15.697
7.334	8.23	9.492	10.548	11.744	12.865	14.039
8.594	9.57	10.813	11.911	13.111	14.261	15.45
6.957	7.476	8.695	9.394	10.499	11.308	12.35
7.666	8.474	9.729	10.709	11.878	12.934	14.069
8.034	8.999	10.274	11.39	12.622	13.803	15.027
7.678	8.545	9.79	10.81	11.982	13.066	14.211
7.703	8.506	9.74	10.703	11.858	12.896	14.021
8.29	9.357	10.659	11.855	13.13	14.376	15.65
7.536	8.145	9.439	10.269	11.435	12.381	13.489
9.176	10.45	11.837	13.187	14.568	15.942	17.325
7.216	7.769	8.992	9.721	10.828	11.658	12.702
8.54	9.453	10.689	11.744	12.924	14.037	15.198
7.714	8.55	9.839	10.868	12.08	13.195	14.383
7.026	7.46	8.72	9.355	10.482	11.241	12.294
8.585	9.557	10.823	11.926	13.14	14.297	15.496
9.111	10.21	11.527	12.752	14.051	15.329	16.635
7.805	8.742	9.955	11.005	12.165	13.263	14.405
7.498	8.281	9.539	10.5	11.665	12.704	13.83
9.403	10.646	12.025	13.361	14.739	16.112	17.5
8.121	9.033	10.317	11.397	12.62	13.773	14.982
8.668	9.837	11.22	12.523	13.891	15.242	16.613
7.626	8.616	9.855	10.968	12.172	13.335	14.532
8.502	9.56	10.838	12.015	13.271	14.499	15.757
8.5	9.542	10.843	12.016	13.279	14.503	15.76
8.208	9.049	10.331	11.356	12.559	13.665	14.842
7.589	8.429	9.715	10.739	11.943	13.048	14.226
9.413	10.626	11.968	13.264	14.6	15.929	17.273
7.738	8.608	9.858	10.886	12.07	13.168	14.331
7.916	8.689	9.995	10.984	12.196	13.284	14.464
8.074	9.029	10.305	11.409	12.634	13.801	15.015
8.119	9.071	10.337	11.436	12.655	13.819	15.029
8.265	9.187	10.459	11.54	12.755	13.906	15.11
8.149	9.054	10.327	11.407	12.627	13.784	14.995

8.488	9.486	10.75	11.874	13.093	14.266	15.475
8.208	9.146	10.412	11.506	12.723	13.884	15.092
8.616	9.529	10.786	11.852	13.054	14.19	15.379
8.209	9.148	10.415	11.508	12.726	13.887	15.095
8.451	9.558	10.892	12.136	13.451	14.745	16.062
8.146	9.088	10.36	11.456	12.677	13.84	15.051
8.572	9.562	10.836	11.963	13.194	14.378	15.601
8.574	9.558	10.821	11.936	13.154	14.323	15.53
8.072	9.018	10.286	11.381	12.597	13.755	14.958
8.419	9.341	10.599	11.675	12.881	14.025	15.22
8.163	9.104	10.376	11.474	12.695	13.859	15.07
8.387	9.412	10.658	11.785	12.994	14.162	15.361
8.074	9.029	10.305	11.409	12.634	13.801	15.015
8.101	8.999	10.29	11.369	12.6	13.76	14.978
8.035	8.964	10.24	11.336	12.564	13.733	14.952
8.074	9.029	10.305	11.409	12.634	13.801	15.015
7.476	8.161	9.467	10.39	11.583	12.619	13.769
7.652	8.333	9.631	10.539	11.733	12.759	13.912
8.2	9.306	10.569	11.755	12.994	14.208	15.442
7.251	7.923	9.2	10.083	11.255	12.247	13.374
7.646	8.444	9.729	10.727	11.932	13.024	14.202
7.62	8.636	9.898	11.039	12.266	13.456	14.675
7.646	8.444	9.729	10.727	11.932	13.024	14.202
8.456	9.576	10.875	12.092	13.367	14.619	15.891
7.661	8.525	9.783	10.811	12.001	13.103	14.271
6.834	7.452	8.697	9.525	10.644	11.573	12.637
7.639	8.336	9.602	10.5	11.662	12.662	13.784
8.034	8.955	10.22	11.29	12.494	13.627	14.812
7.789	8.684	9.932	10.978	12.166	13.279	14.45
8.067	8.965	10.218	11.265	12.459	13.575	14.751
8.013	8.918	10.137	11.179	12.346	13.447	14.597
7.556	8.59	9.828	10.965	12.175	13.353	14.557
7.987	8.887	10.156	11.228	12.445	13.595	14.801
7.064	7.816	9.054	9.986	11.133	12.148	13.26
7.789	8.553	9.828	10.784	11.968	13.018	14.169
7.532	8.611	9.908	11.093	12.347	13.565	14.804
7.843	8.893	10.185	11.362	12.622	13.847	15.1
7.515	8.285	9.596	10.579	11.786	12.864	14.033
9.176	10.45	11.837	13.187	14.568	15.942	17.325
7.665	8.489	9.755	10.757	11.946	13.031	14.195
7.398	8.157	9.404	10.339	11.495	12.514	13.631
8.621	9.685	10.97	12.151	13.407	14.635	15.89
7.573	8.432	9.691	10.716	11.902	12.999	14.162
7.513	8.34	9.563	10.542	11.694	12.742	13.866
8.924	10.126	11.485	12.786	14.134	15.469	16.82
9.278	10.382	11.713	12.957	14.277	15.579	16.909
7.96	8.775	10.033	11.03	12.216	13.301	14.466
7.155	7.787	9.105	9.975	11.167	12.157	13.294
8.204	9.298	10.629	11.855	13.155	14.428	15.725
9.487	10.65	11.987	13.26	14.591	15.911	17.251

8.069	8.956	10.162	11.179	12.333	13.412	14.55
8.159	9.007	10.288	11.317	12.52	13.628	14.805
8.815	9.928	11.237	12.468	13.764	15.043	16.346
7.642	8.503	9.702	10.703	11.843	12.904	14.023
6.301	6.767	7.977	8.642	9.707	10.475	11.465
8.178	9.235	10.539	11.722	12.989	14.22	15.48
8.693	9.603	10.869	11.936	13.145	14.283	15.478
7.522	8.398	9.586	10.595	11.735	12.805	13.931
7.242	7.897	9.159	10.008	11.156	12.105	13.2
7.228	7.902	9.159	10.023	11.172	12.135	13.235
7.584	8.263	9.569	10.473	11.666	12.685	13.832
7.618	8.373	9.641	10.588	11.766	12.808	13.952
8.776	9.913	11.218	12.448	13.732	14.997	16.28
8.116	9.034	10.24	11.273	12.426	13.51	14.644
7.979	8.974	10.256	11.402	12.648	13.855	15.098
7.721	8.636	9.921	11.005	12.23	13.386	14.594
7.903	8.803	10.053	11.106	12.299	13.421	14.598
6.503	7.235	8.418	9.302	10.406	11.364	12.43
8.231	9.14	10.413	11.48	12.69	13.826	15.018
7.433	8.234	9.473	10.436	11.591	12.628	13.75
7.116	8.271	9.463	10.639	11.824	13.005	14.188
8.886	9.995	11.31	12.534	13.824	15.09	16.377
7.944	8.934	10.173	11.285	12.49	13.654	14.854
9.071	10.261	11.615	12.914	14.264	15.606	16.965
7.392	8.444	9.713	10.869	12.096	13.287	14.501
8.297	9.381	10.716	11.943	13.251	14.53	15.837
7.454	8.281	9.525	10.527	11.702	12.784	13.938
7.698	8.703	9.935	11.049	12.246	13.404	14.592
8.39	9.37	10.621	11.732	12.944	14.111	15.317
8.304	9.239	10.491	11.575	12.78	13.927	15.122
8.172	9.066	10.332	11.393	12.603	13.741	14.937
7.929	8.783	10.052	11.076	12.269	13.366	14.532
8.003	8.912	10.156	11.215	12.408	13.533	14.713
8.2	9.306	10.569	11.755	12.994	14.208	15.442
8.515	9.643	10.998	12.261	13.595	14.909	16.246
7.043	7.793	8.985	9.89	11.004	11.986	13.068
8.057	8.703	9.976	10.818	11.974	12.919	14.02
7.987	8.887	10.156	11.228	12.445	13.595	14.801
7.789	8.655	9.908	10.932	12.114	13.208	14.366
8.4	9.386	10.693	11.839	13.099	14.311	15.563
7.405	8.274	9.516	10.547	11.728	12.83	13.992
7.272	8.165	9.404	10.448	11.628	12.734	13.894
8.097	9.019	10.278	11.357	12.565	13.711	14.907
7.535	8.309	9.532	10.464	11.601	12.608	13.709
7.441	8.223	9.462	10.409	11.56	12.584	13.699
7.232	8.015	9.243	10.195	11.344	12.376	13.495
8.023	8.914	10.136	11.155	12.312	13.386	14.518
7.578	8.323	9.597	10.544	11.725	12.771	13.919
7.009	7.902	9.102	10.127	11.268	12.347	13.47
8.187	9.107	10.374	11.451	12.666	13.815	15.018

7.151	7.907	9.12	10.049	11.181	12.191	13.292
7.738	8.523	9.778	10.742	11.912	12.96	14.096
8.075	8.997	10.253	11.327	12.531	13.673	14.866
7.735	8.561	9.811	10.807	11.984	13.06	14.214
8.011	8.827	10.102	11.103	12.292	13.373	14.53
8.262	9.323	10.612	11.8	13.068	14.309	15.578
7.182	7.881	9.099	9.969	11.087	12.04	13.113
7.905	8.995	10.328	11.546	12.84	14.101	15.384
7.753	8.569	9.863	10.87	12.078	13.174	14.353
7.433	8.241	9.48	10.449	11.604	12.644	13.764
6.88	7.689	8.947	9.928	11.095	12.149	13.28
6.217	6.997	8.069	8.959	9.98	10.922	11.923
8.145	9.253	10.596	11.837	13.15	14.436	15.744
8.45	9.523	10.814	12.003	13.266	14.502	15.765
8.023	8.788	10.011	10.941	12.083	13.096	14.207
8.949	10.162	11.518	12.822	14.167	15.503	16.851
7.375	8.165	9.377	10.334	11.474	12.511	13.627
6.866	7.322	8.587	9.245	10.375	11.156	12.21
7.026	7.928	9.145	10.185	11.343	12.437	13.575
7.959	8.836	10.107	11.151	12.353	13.47	14.65
8.661	9.633	10.903	12.016	13.242	14.414	15.63
8.515	9.643	10.998	12.261	13.595	14.909	16.246
8.351	9.283	10.544	11.622	12.829	13.972	15.167
7.391	8.185	9.392	10.351	11.495	12.535	13.656
7.851	8.751	9.961	10.987	12.143	13.225	14.362
8.538	9.589	10.886	12.065	13.329	14.559	15.82
7.581	8.471	9.713	10.757	11.943	13.056	14.226
8.864	10.06	11.442	12.763	14.141	15.509	16.896
8.774	9.989	11.385	12.719	14.105	15.48	16.871
7.392	8.177	9.404	10.35	11.495	12.517	13.629
7.641	8.643	9.865	10.974	12.165	13.319	14.505
8.554	9.59	10.907	12.096	13.382	14.634	15.921
7.991	8.929	10.183	11.27	12.476	13.626	14.823
7.757	8.633	9.851	10.861	12.019	13.093	14.23
7.965	8.874	10.123	11.189	12.388	13.523	14.711
7.331	8.116	9.311	10.247	11.363	12.366	13.45
6.895	7.683	8.869	9.799	10.909	11.904	12.983
7.904	8.779	10.037	11.073	12.265	13.374	14.545
7.515	8.285	9.596	10.579	11.786	12.864	14.033
8.696	9.656	10.907	12.008	13.221	14.385	15.593
8.187	9.107	10.374	11.451	12.666	13.815	15.018
7.859	8.691	9.926	10.917	12.077	13.139	14.272
7.909	8.821	10.109	11.195	12.423	13.582	14.795
8.528	9.587	10.88	12.062	13.324	14.555	15.815
7.622	8.411	9.672	10.643	11.822	12.883	14.033
8.807	10.021	11.411	12.742	14.125	15.498	16.887
7.886	8.783	10.079	11.161	12.394	13.555	14.773
9.156	10.292	11.612	12.848	14.142	15.414	16.705
7.986	9.163	10.491	11.758	13.066	14.36	15.665
6.575	7.094	8.347	9.064	10.188	11.019	12.075

7.908	8.978	10.338	11.554	12.859	14.121	15.41
8.038	9.185	10.527	11.785	13.099	14.391	15.699
7.828	8.728	10.038	11.126	12.367	13.533	14.754
8.109	9.235	10.597	11.853	13.178	14.475	15.792
7.245	7.972	9.226	10.153	11.316	12.34	13.468
8.361	9.325	10.613	11.731	12.969	14.151	15.378
7.938	8.751	10.031	11.035	12.236	13.332	14.509
8.106	9.066	10.325	11.426	12.642	13.806	15.015
7.421	8.256	9.471	10.454	11.597	12.644	13.76
6.957	7.476	8.695	9.394	10.499	11.308	12.35
7.922	8.737	9.966	10.956	12.125	13.202	14.355
8.022	8.836	10.082	11.061	12.226	13.282	14.417
8.915	10.13	11.488	12.792	14.138	15.473	16.822
8.613	9.758	11.082	12.325	13.624	14.901	16.195
6.946	7.567	8.789	9.595	10.707	11.611	12.673
6.946	7.567	8.789	9.595	10.707	11.611	12.673
7.356	7.953	9.258	10.092	11.269	12.226	13.346
7.251	7.933	9.211	10.108	11.282	12.287	13.418
7.163	8.06	9.267	10.294	11.446	12.529	13.663
7.251	7.933	9.211	10.108	11.282	12.287	13.418
7.407	8.219	9.47	10.462	11.641	12.717	13.87
7.787	8.503	9.81	10.744	11.941	12.981	14.135
7.96	8.796	10.069	11.08	12.273	13.362	14.526
7.96	8.796	10.069	11.08	12.273	13.362	14.526
7.054	7.623	8.842	9.608	10.712	11.585	12.634
7.961	8.717	9.981	10.927	12.098	13.136	14.271
7.615	8.55	9.757	10.805	11.962	13.057	14.196
8.467	9.737	11.101	12.439	13.803	15.164	16.533
8.14	8.962	10.232	11.233	12.423	13.505	14.666
7.476	8.215	9.472	10.408	11.575	12.607	13.74
9.025	10.15	11.476	12.711	14.009	15.283	16.579
7.487	8.281	9.533	10.499	11.661	12.703	13.828
6.663	7.46	8.678	9.631	10.767	11.787	12.888
8.069	9.021	10.307	11.415	12.649	13.823	15.045
8.489	9.486	10.768	11.905	13.143	14.335	15.564
7.54	8.441	9.634	10.658	11.804	12.882	14.013
8.104	9.093	10.407	11.564	12.834	14.059	15.323
7.745	8.742	9.995	11.121	12.339	13.517	14.73
7.973	8.936	10.209	11.315	12.537	13.701	14.909
8.039	8.899	10.202	11.257	12.487	13.629	14.84
7.88	8.897	10.144	11.274	12.486	13.662	14.867
7.936	8.807	10.066	11.111	12.314	13.44	14.629
7.987	8.956	10.189	11.278	12.465	13.603	14.779
7.818	8.757	9.984	11.048	12.224	13.34	14.5
7.426	8.238	9.449	10.41	11.544	12.572	13.675
7.993	8.91	10.172	11.249	12.459	13.606	14.805
7.367	8.073	9.357	10.285	11.466	12.5	13.643
7.774	8.626	9.882	10.902	12.09	13.188	14.355
7.788	8.726	9.941	10.995	12.16	13.262	14.409
8.222	9.288	10.587	11.781	13.055	14.301	15.575

8.022	8.836	10.082	11.061	12.226	13.282	14.417
7.515	8.438	9.617	10.646	11.781	12.856	13.976
8.56	9.709	11.032	12.275	13.572	14.846	16.137
8.937	10.083	11.398	12.645	13.946	15.232	16.538
7.489	8.456	9.692	10.787	11.983	13.13	14.316
7.986	9.163	10.491	11.758	13.066	14.36	15.665
7.344	8.055	9.321	10.22	11.375	12.363	13.47
7.7	8.582	9.803	10.82	11.977	13.053	14.186
7.398	8.001	9.258	10.058	11.194	12.097	13.172
7.769	8.721	9.991	11.1	12.324	13.496	14.711
7.63	8.636	9.901	11.039	12.268	13.456	14.676
7.924	8.987	10.3	11.497	12.775	14.019	15.29
7.826	8.582	9.863	10.817	12.002	13.05	14.198
7.959	8.836	10.107	11.151	12.353	13.47	14.65
7.57	8.356	9.591	10.548	11.701	12.737	13.859
7.892	8.691	9.943	10.919	12.089	13.146	14.286
6.18	6.903	8.07	8.951	10.024	10.972	12.003
8.013	8.834	10.087	11.081	12.256	13.328	14.474
9.026	10.136	11.46	12.686	13.981	15.25	16.543
8.295	9.297	10.587	11.74	12.994	14.21	15.462
9.546	10.717	12.079	13.369	14.719	16.056	17.414
8.503	9.559	10.882	12.077	13.362	14.611	15.892
7.805	8.653	9.932	10.962	12.167	13.279	14.461
7.843	8.703	9.96	10.981	12.162	13.251	14.406
7.586	8.438	9.665	10.667	11.824	12.893	14.027
8.039	8.834	10.101	11.076	12.255	13.313	14.459
7.828	8.67	9.921	10.935	12.123	13.219	14.389
7.67	8.555	9.792	10.834	12.017	13.129	14.298

ESpm13u	ESpm14u	ESpm15u	ESpm01x	ESpm02x	ESpm03x	ESpm04x
15.216	16.384	17.545	3.114	4.334	5.549	6.868
14.476	15.568	16.635	3.401	4.61	5.769	7.036
15.693	16.86	18.018	3.689	4.934	6.166	7.494
12.791	13.804	14.728	2.773	3.922	5.107	6.372
15.022	16.177	17.32	3.114	4.334	5.525	6.832
17.886	19.258	20.63	2.773	4.127	5.497	6.983
16.664	17.917	19.17	3.664	4.924	6.081	7.379
14.363	15.479	16.575	2.773	3.989	5.182	6.483
15.686	16.862	18.028	3.57	4.806	6.036	7.36
13.718	14.776	15.785	3.135	4.297	5.408	6.629
16.174	17.325	18.468	3.784	5.124	6.261	7.548
15.821	16.992	18.153	3.761	4.99	6.194	7.512
15.161	16.315	17.452	3.434	4.644	5.825	7.111
15.942	17.144	18.34	3.611	4.848	6.041	7.359
16.964	18.236	19.509	3.526	4.856	6.09	7.44
16.959	18.232	19.505	3.497	4.816	6.038	7.383
15.187	16.354	17.512	3.045	4.27	5.465	6.762
16.623	17.813	18.998	3.892	5.204	6.435	7.771
13.225	14.234	15.151	3.178	4.363	5.502	6.74
15.158	16.281	17.385	3.332	4.59	5.724	6.992
16.237	17.464	18.687	3.434	4.732	5.951	7.285
15.322	16.458	17.581	3.45	4.67	5.918	7.255
15.097	16.211	17.306	3.497	4.727	5.966	7.288
16.918	18.197	19.476	3.466	4.775	5.992	7.331
14.494	15.578	16.614	3.584	4.779	5.882	7.104
18.709	20.095	21.482	3.584	5.011	6.297	7.724
13.592	14.602	15.527	3.296	4.494	5.607	6.833
16.334	17.489	18.636	4.127	5.409	6.655	7.998
15.537	16.719	17.89	3.497	4.71	5.911	7.212
13.132	14.143	15.031	3.296	4.472	5.562	6.764
16.677	17.875	19.068	4.043	5.293	6.541	7.894
17.938	19.254	20.573	4.078	5.449	6.693	8.064
15.523	16.659	17.787	3.258	4.516	5.761	7.102
14.904	16.015	17.104	3.296	4.516	5.66	6.936
18.89	20.287	21.688	3.784	5.239	6.53	7.963
16.169	17.378	18.582	3.611	4.879	6.019	7.305
17.981	19.357	20.732	3.466	4.824	6.036	7.411
15.717	16.915	18.11	3.02	4.307	5.552	6.901
17.01	18.275	19.541	3.584	4.927	6.136	7.476
17.007	18.267	19.526	3.701	5.016	6.277	7.65
15.985	17.157	18.318	3.961	5.186	6.409	7.727
15.367	16.538	17.696	3.367	4.585	5.791	7.093
18.619	19.972	21.328	3.932	5.361	6.644	8.061
15.461	16.618	17.764	3.418	4.661	5.923	7.269
15.598	16.77	17.926	3.795	4.999	6.186	7.483
16.211	17.425	18.635	3.526	4.808	6.012	7.337
16.222	17.434	18.643	3.555	4.848	6.064	7.397
16.294	17.5	18.703	3.795	5.067	6.267	7.585
16.189	17.404	18.615	3.738	5.007	6.206	7.521

16.671	17.88	19.087	3.773	5.083	6.301	7.645
16.283	17.493	18.7	3.75	5.026	6.236	7.561
16.548	17.74	18.928	4.119	5.411	6.614	7.936
16.286	17.496	18.703	3.726	5.006	6.216	7.544
17.377	18.699	20.022	3.651	4.943	6.161	7.508
16.244	17.457	18.666	3.638	4.916	6.125	7.45
16.81	18.034	19.255	3.97	5.257	6.502	7.86
16.723	17.931	19.136	3.961	5.251	6.48	7.818
16.144	17.348	18.548	3.57	4.846	6.054	7.38
16.397	17.596	18.791	3.922	5.216	6.431	7.762
16.264	17.476	18.685	3.701	4.972	6.171	7.493
16.546	17.744	18.938	3.676	4.958	6.256	7.642
16.211	17.425	18.635	3.526	4.808	6.012	7.337
16.175	17.395	18.609	3.726	4.982	6.172	7.479
16.154	17.375	18.592	3.597	4.869	6.063	7.379
16.211	17.425	18.635	3.497	4.796	5.996	7.324
14.858	15.995	17.108	3.418	4.627	5.697	6.917
14.997	16.137	17.251	3.611	4.82	5.957	7.209
16.668	17.901	19.133	3.296	4.6	5.956	7.409
14.421	15.53	16.605	3.296	4.477	5.637	6.891
15.337	16.507	17.662	3.401	4.635	5.822	7.115
15.883	17.101	18.316	3.178	4.388	5.639	7.01
15.337	16.507	17.662	3.434	4.663	5.868	7.171
17.158	18.432	19.707	3.526	4.828	6.134	7.542
15.407	16.571	17.724	3.296	4.543	5.767	7.09
13.614	14.655	15.656	3.157	4.321	5.468	6.719
14.836	15.943	17.022	3.611	4.836	6.01	7.286
15.972	17.152	18.324	3.624	4.854	6.069	7.384
15.594	16.763	17.923	3.332	4.61	5.836	7.173
15.9	17.074	18.24	3.526	4.824	6.041	7.372
15.725	16.873	18.014	3.401	4.718	5.852	7.132
15.752	16.957	18.158	2.944	4.182	5.43	6.767
15.986	17.193	18.394	3.638	4.883	6.133	7.474
14.314	15.412	16.486	2.996	4.197	5.381	6.674
15.265	16.406	17.526	3.555	4.804	6.02	7.343
16.033	17.268	18.5	2.773	3.989	5.13	6.471
16.344	17.597	18.848	3.296	4.522	5.804	7.184
15.152	16.309	17.447	3.367	4.564	5.715	6.99
18.709	20.095	21.482	3.584	5.011	6.297	7.724
15.319	16.476	17.619	3.434	4.663	5.876	7.185
14.688	15.79	16.866	3.332	4.533	5.741	7.039
17.14	18.4	19.662	3.75	5.08	6.335	7.7
15.291	16.448	17.593	3.199	4.457	5.634	6.937
14.947	16.061	17.158	3.296	4.522	5.767	7.092
18.171	19.527	20.885	3.526	4.942	6.176	7.568
18.239	19.582	20.928	4.159	5.561	6.749	8.092
15.594	16.756	17.906	3.689	4.934	6.115	7.405
14.342	15.456	16.531	3.296	4.454	5.582	6.811
17.015	18.314	19.612	3.526	4.762	5.957	7.269
18.594	19.946	21.301	4.331	5.694	6.947	8.325

15.659	16.793	17.917	3.714	4.956	6.218	7.587
15.947	17.116	18.274	3.761	5.037	6.216	7.516
17.649	18.963	20.278	3.784	5.15	6.424	7.81
15.113	16.226	17.328	3.466	4.705	5.946	7.276
12.288	13.238	14.092	2.639	3.738	4.7	5.835
16.731	17.993	19.253	3.401	4.677	5.921	7.287
16.65	17.846	19.037	4.025	5.361	6.555	7.881
15.031	16.154	17.269	3.314	4.535	5.793	7.147
14.199	15.27	16.296	3.367	4.543	5.725	6.997
14.248	15.327	16.367	3.296	4.477	5.658	6.927
14.908	16.041	17.145	3.555	4.754	5.879	7.124
15.041	16.175	17.287	3.541	4.747	5.959	7.257
17.56	18.846	20.133	3.526	4.927	6.17	7.549
15.75	16.878	17.995	3.526	4.808	6.012	7.329
16.33	17.575	18.818	3.497	4.727	5.951	7.27
15.779	16.985	18.183	3.367	4.605	5.827	7.147
15.751	16.927	18.096	3.481	4.743	5.972	7.304
13.424	14.473	15.486	2.565	3.761	4.956	6.252
16.184	17.373	18.554	3.689	4.963	6.147	7.456
14.822	15.931	17.019	3.296	4.5	5.709	7.006
15.37	16.552	17.734	2.197	3.434	4.787	6.221
17.659	18.948	20.236	3.892	5.223	6.422	7.764
16.042	17.245	18.445	3.45	4.698	5.977	7.352
18.326	19.694	21.065	3.807	5.198	6.446	7.831
15.703	16.913	18.119	2.639	3.871	5.024	6.342
17.137	18.446	19.755	3.367	4.696	5.914	7.267
15.058	16.207	17.344	3.199	4.434	5.624	6.931
15.768	16.957	18.143	3.135	4.376	5.603	6.932
16.51	17.719	18.926	3.526	4.883	6.035	7.342
16.298	17.493	18.683	3.829	5.103	6.319	7.663
16.11	17.306	18.496	3.829	5.069	6.279	7.612
15.662	16.819	17.963	3.497	4.775	5.968	7.282
15.869	17.047	18.219	3.638	4.875	6.083	7.388
16.668	17.901	19.133	3.258	4.57	5.922	7.38
17.579	18.92	20.262	3.401	4.775	6.009	7.386
14.089	15.158	16.199	2.996	4.197	5.381	6.656
15.019	16.094	17.122	4.007	5.231	6.413	7.694
15.986	17.193	18.394	3.638	4.883	6.133	7.474
15.492	16.644	17.786	3.466	4.71	5.92	7.23
16.804	18.06	19.315	3.861	5.113	6.311	7.628
15.125	16.282	17.429	3.091	4.357	5.521	6.822
15.027	16.181	17.325	2.833	4.111	5.252	6.541
16.084	17.282	18.474	3.611	4.894	6.116	7.454
14.753	15.841	16.904	3.466	4.673	5.887	7.189
14.759	15.859	16.936	3.367	4.564	5.764	7.053
14.565	15.673	16.762	2.996	4.248	5.398	6.672
15.617	16.74	17.85	3.611	4.86	6.105	7.438
15.013	16.152	17.271	3.624	4.838	6.048	7.336
14.571	15.689	16.799	2.708	3.932	5.069	6.347
16.2	17.405	18.605	3.584	4.902	6.121	7.459

14.341	15.431	16.5	3.091	4.284	5.456	6.74
15.183	16.308	17.414	3.466	4.723	5.881	7.165
16.038	17.232	18.42	3.526	4.824	6.048	7.386
15.329	16.477	17.612	3.466	4.71	5.936	7.255
15.647	16.794	17.926	3.807	5.021	6.221	7.525
16.843	18.119	19.396	3.57	4.85	6.134	7.522
14.108	15.16	16.177	3.178	4.363	5.502	6.759
16.659	17.94	19.219	3.178	4.413	5.584	6.899
15.49	16.662	17.819	3.401	4.654	5.83	7.125
14.835	15.941	17.027	3.178	4.413	5.584	6.875
14.363	15.479	16.575	2.773	3.989	5.165	6.446
12.889	13.883	14.861	2.398	3.555	4.754	6.028
17.046	18.354	19.661	3.466	4.691	5.887	7.202
17.022	18.29	19.558	3.555	4.879	6.112	7.471
15.261	16.36	17.437	3.861	5.101	6.321	7.631
18.199	19.552	20.905	3.555	4.963	6.227	7.628
14.703	15.814	16.91	3.219	4.425	5.599	6.894
13.065	14.078	14.979	3.135	4.297	5.395	6.607
14.69	15.821	16.944	2.862	4.047	5.272	6.602
15.801	16.976	18.143	3.597	4.846	6.088	7.421
16.829	18.045	19.258	4.086	5.351	6.588	7.922
17.579	18.92	20.262	3.401	4.775	6.009	7.381
16.341	17.537	18.728	3.689	5.014	6.229	7.568
14.736	15.852	16.952	3.219	4.449	5.643	6.936
15.469	16.6	17.719	3.296	4.58	5.778	7.089
17.073	18.338	19.603	3.726	5.035	6.273	7.63
15.371	16.539	17.698	3.219	4.472	5.705	7.036
18.282	19.676	21.071	3.555	4.952	6.207	7.603
18.261	19.657	21.054	3.497	4.864	6.101	7.488
14.688	15.787	16.864	3.239	4.451	5.677	6.995
15.68	16.868	18.054	3.045	4.297	5.538	6.867
17.2	18.493	19.787	3.807	5.106	6.34	7.691
16.003	17.201	18.395	3.584	4.84	6.081	7.427
15.334	16.467	17.588	3.497	4.718	5.985	7.346
15.88	17.07	18.256	3.611	4.864	6.096	7.436
14.485	15.555	16.604	3.277	4.503	5.713	7.017
14.01	15.077	16.121	2.773	3.961	5.16	6.457
15.688	16.856	18.015	3.466	4.741	5.946	7.271
15.152	16.309	17.447	3.401	4.595	5.767	7.053
16.788	18.001	19.212	4.078	5.384	6.625	7.969
16.2	17.405	18.605	3.584	4.902	6.121	7.459
15.366	16.49	17.599	3.584	4.82	6.061	7.396
15.986	17.198	18.404	3.512	4.76	5.986	7.317
17.069	18.334	19.6	3.701	5.016	6.254	7.606
15.138	16.281	17.407	3.434	4.663	5.876	7.182
18.276	19.67	21.066	3.466	4.856	6.112	7.515
15.968	17.184	18.394	3.555	4.787	5.967	7.261
17.991	19.285	20.579	3.892	5.293	6.503	7.874
16.966	18.27	19.574	2.996	4.277	5.489	6.85
12.97	13.99	14.923	2.89	4.034	5.189	6.442

16.687	17.972	19.254	3.178	4.413	5.538	6.832
17.002	18.309	19.615	3.219	4.472	5.695	7.043
15.952	17.171	18.383	3.526	4.745	5.909	7.192
17.102	18.417	19.73	3.401	4.635	5.848	7.177
14.538	15.654	16.746	3.219	4.413	5.583	6.858
16.587	17.814	19.037	3.784	5.056	6.264	7.607
15.649	16.823	17.985	3.638	4.89	6.07	7.364
16.208	17.42	18.628	3.611	4.868	6.115	7.474
14.837	15.944	17.036	3.178	4.413	5.607	6.903
13.225	14.234	15.151	3.178	4.363	5.502	6.74
15.475	16.629	17.772	3.584	4.868	6.019	7.29
15.508	16.634	17.744	3.714	4.963	6.172	7.491
18.169	19.521	20.875	3.555	4.949	6.215	7.617
17.484	18.779	20.073	3.367	4.762	5.986	7.363
13.63	14.669	15.656	3.091	4.256	5.39	6.64
13.63	14.669	15.656	3.091	4.256	5.39	6.64
14.366	15.463	16.517	3.526	4.691	5.816	7.053
14.477	15.591	16.675	3.277	4.463	5.628	6.897
14.77	15.898	17.017	2.833	4.052	5.264	6.571
14.477	15.591	16.675	3.277	4.463	5.628	6.897
14.984	16.131	17.262	3.219	4.431	5.661	6.979
15.227	16.368	17.486	3.638	4.86	6.005	7.277
15.651	16.808	17.95	3.541	4.81	5.992	7.297
15.651	16.808	17.95	3.541	4.81	5.992	7.297
13.565	14.587	15.549	3.314	4.491	5.645	6.906
15.351	16.475	17.576	3.784	5.017	6.181	7.461
15.311	16.445	17.57	3.091	4.35	5.627	6.978
17.903	19.276	20.649	3.135	4.449	5.785	7.223
15.786	16.938	18.075	3.761	5.04	6.238	7.557
14.819	15.942	17.043	3.418	4.623	5.822	7.116
17.869	19.167	20.466	3.829	5.215	6.399	7.753
14.903	16.013	17.103	3.258	4.483	5.636	6.917
13.937	15.022	16.084	2.708	3.861	5.064	6.353
16.248	17.469	18.686	3.401	4.705	5.876	7.182
16.78	18.01	19.237	3.839	5.128	6.374	7.742
15.119	16.247	17.366	3.178	4.413	5.66	7.015
16.575	17.84	19.103	3.597	4.826	5.982	7.273
15.93	17.144	18.355	3.277	4.508	5.787	7.163
16.099	17.306	18.508	3.57	4.79	6.044	7.398
16.022	17.231	18.432	3.807	5.027	6.206	7.491
16.062	17.27	18.475	3.314	4.562	5.853	7.24
15.793	16.983	18.165	3.638	4.89	6.137	7.469
15.939	17.116	18.289	3.434	4.696	5.928	7.291
15.637	16.792	17.939	3.367	4.61	5.877	7.252
14.733	15.825	16.897	3.314	4.519	5.724	7.019
15.985	17.185	18.381	3.638	4.86	6.076	7.415
14.727	15.856	16.963	3.401	4.59	5.75	7.024
15.49	16.654	17.808	3.466	4.71	5.928	7.242
15.532	16.674	17.806	3.296	4.549	5.81	7.168
16.844	18.123	19.403	3.541	4.81	6.085	7.469

15.508	16.634	17.744	3.651	4.918	6.107	7.423
15.073	16.189	17.297	2.996	4.256	5.498	6.824
17.423	18.714	20.004	3.296	4.691	5.919	7.3
17.842	19.154	20.468	3.784	5.162	6.452	7.854
15.487	16.672	17.852	2.97	4.238	5.463	6.796
16.966	18.27	19.574	2.996	4.277	5.489	6.85
14.5	15.586	16.637	3.239	4.451	5.624	6.905
15.287	16.413	17.526	3.239	4.491	5.649	6.934
14.131	15.177	16.165	3.526	4.71	5.859	7.114
15.91	17.124	18.334	3.367	4.6	5.812	7.156
15.883	17.102	18.317	3.219	4.425	5.664	7.027
16.551	17.821	19.089	3.178	4.437	5.618	6.935
15.291	16.427	17.541	3.611	4.864	6.019	7.292
15.801	16.976	18.143	3.597	4.846	6.088	7.421
14.932	16.042	17.133	3.367	4.585	5.801	7.118
15.379	16.509	17.621	3.611	4.864	6.019	7.303
12.981	13.994	14.984	2.398	3.497	4.554	5.759
15.581	16.719	17.842	3.818	5.039	6.221	7.508
17.83	19.126	20.422	3.871	5.257	6.446	7.798
16.706	17.964	19.221	3.761	5.011	6.21	7.528
18.774	20.142	21.515	4.369	5.733	6.986	8.374
17.165	18.449	19.733	3.638	4.952	6.186	7.553
15.61	16.787	17.953	3.555	4.787	5.998	7.308
15.527	16.674	17.81	3.611	4.844	6.078	7.401
15.126	16.253	17.367	3.258	4.522	5.69	6.981
15.556	16.69	17.806	3.738	4.997	6.198	7.511
15.526	16.694	17.851	3.555	4.796	6.038	7.366
15.442	16.609	17.769	3.384	4.623	5.867	7.196

ESpm05x	ESpm06x	ESpm07x	ESpm08x	ESpm09x	ESpm10x	ESpm11x
8.227	9.62	11.038	12.478	13.934	15.403	16.884
8.334	9.662	11.012	12.381	13.765	15.163	16.573
8.853	10.242	11.652	13.079	14.519	15.97	17.431
7.662	8.97	10.291	11.622	12.962	14.309	15.662
8.181	9.567	10.978	12.41	13.858	15.317	16.786
8.506	10.059	11.629	13.213	14.806	16.407	18.014
8.717	10.095	11.501	12.928	14.372	15.828	17.296
7.824	9.201	10.602	12.021	13.454	14.897	16.349
8.722	10.118	11.539	12.98	14.436	15.904	17.382
7.883	9.174	10.491	11.832	13.192	14.567	15.955
8.852	10.188	11.54	12.907	14.285	15.672	17.066
8.872	10.271	11.698	13.146	14.611	16.089	17.578
8.439	9.805	11.199	12.615	14.049	15.498	16.957
8.719	10.116	11.54	12.983	14.442	15.914	17.395
8.818	10.227	11.657	13.103	14.562	16.032	17.512
8.758	10.166	11.597	13.046	14.508	15.983	17.466
8.094	9.46	10.848	12.256	13.679	15.113	16.557
9.133	10.523	11.932	13.356	14.79	16.234	17.685
7.999	9.281	10.579	11.889	13.21	14.541	15.88
8.285	9.612	10.96	12.325	13.703	15.091	16.486
8.652	10.051	11.473	12.914	14.37	15.838	17.315
8.634	10.046	11.481	12.933	14.398	15.872	17.353
8.645	10.03	11.434	12.855	14.288	15.731	17.183
8.699	10.101	11.526	12.97	14.429	15.902	17.385
8.355	9.64	10.948	12.274	13.613	14.964	16.323
9.19	10.697	12.23	13.784	15.354	16.934	18.523
8.082	9.358	10.649	11.954	13.27	14.594	15.926
9.375	10.783	12.214	13.662	15.125	16.598	18.079
8.558	9.946	11.367	12.813	14.281	15.764	17.26
7.992	9.249	10.525	11.819	13.126	14.445	15.774
9.289	10.72	12.175	13.65	15.139	16.639	18.149
9.468	10.908	12.374	13.86	15.362	16.877	18.403
8.478	9.883	11.306	12.745	14.195	15.655	17.123
8.247	9.596	10.969	12.361	13.768	15.186	16.612
9.426	10.927	12.451	13.996	15.556	17.129	18.713
8.623	9.979	11.359	12.758	14.171	15.596	17.03
8.822	10.28	11.769	13.283	14.818	16.369	17.934
8.286	9.704	11.143	12.599	14.069	15.549	17.038
8.846	10.249	11.674	13.117	14.572	16.039	17.514
9.059	10.501	11.967	13.45	14.946	16.453	17.969
9.088	10.486	11.91	13.356	14.817	16.293	17.778
8.433	9.807	11.205	12.623	14.057	15.503	16.959
9.506	10.984	12.482	13.998	15.528	17.069	18.619
8.652	10.065	11.501	12.956	14.427	15.911	17.405
8.828	10.215	11.635	13.08	14.547	16.03	17.526
8.694	10.085	11.5	12.933	14.38	15.838	17.305
8.758	10.152	11.566	12.997	14.441	15.896	17.359
8.937	10.325	11.735	13.164	14.606	16.058	17.519
8.867	10.247	11.65	13.07	14.505	15.951	17.406

9.022	10.432	11.863	13.309	14.765	16.231	17.703
8.918	10.31	11.725	13.158	14.605	16.063	17.53
9.288	10.674	12.08	13.503	14.938	16.383	17.836
8.905	10.299	11.715	13.149	14.596	16.055	17.522
8.894	10.319	11.772	13.248	14.742	16.252	17.773
8.806	10.195	11.606	13.036	14.479	15.933	17.397
9.253	10.679	12.129	13.597	15.08	16.574	18.079
9.187	10.589	12.011	13.45	14.901	16.362	17.83
8.74	10.135	11.552	12.986	14.432	15.888	17.351
9.123	10.517	11.932	13.363	14.806	16.26	17.721
8.849	10.241	11.656	13.09	14.538	15.998	17.466
9.064	10.516	11.989	13.476	14.976	16.485	18.002
8.694	10.085	11.5	12.933	14.38	15.838	17.305
8.818	10.192	11.591	13.009	14.442	15.888	17.345
8.724	10.104	11.506	12.928	14.364	15.814	17.274
8.683	10.077	11.493	12.926	14.374	15.832	17.299
8.171	9.471	10.8	12.154	13.525	14.911	16.309
8.489	9.802	11.136	12.49	13.858	15.239	16.631
8.898	10.414	11.948	13.495	15.052	16.616	18.185
8.179	9.498	10.84	12.199	13.574	14.959	16.355
8.444	9.807	11.195	12.605	14.031	15.471	16.923
8.429	9.887	11.374	12.882	14.406	15.943	17.49
8.512	9.886	11.286	12.707	14.146	15.599	17.063
8.989	10.468	11.97	13.489	15.021	16.562	18.111
8.449	9.839	11.251	12.678	14.119	15.57	17.03
8.008	9.328	10.67	12.028	13.398	14.777	16.164
8.591	9.925	11.278	12.647	14.027	15.416	16.812
8.74	10.13	11.546	12.981	14.431	15.892	17.362
8.546	9.95	11.376	12.819	14.274	15.74	17.214
8.734	10.128	11.545	12.979	14.427	15.886	17.355
8.435	9.77	11.124	12.492	13.873	15.262	16.659
8.134	9.527	10.937	12.362	13.797	15.24	16.69
8.853	10.264	11.7	13.157	14.631	16.121	17.623
8.006	9.369	10.753	12.153	13.565	14.987	16.416
8.704	10.1	11.521	12.962	14.42	15.892	17.374
7.873	9.331	10.819	12.325	13.843	15.368	16.896
8.613	10.083	11.582	13.104	14.645	16.2	17.766
8.308	9.668	11.056	12.467	13.894	15.333	16.783
9.19	10.697	12.23	13.784	15.354	16.934	18.523
8.53	9.906	11.304	12.72	14.151	15.594	17.047
8.371	9.731	11.112	12.51	13.922	15.344	16.774
9.097	10.525	11.975	13.442	14.923	16.413	17.912
8.279	9.659	11.067	12.495	13.94	15.397	16.864
8.449	9.833	11.236	12.654	14.085	15.524	16.972
8.996	10.465	11.96	13.476	15.007	16.55	18.103
9.463	10.875	12.317	13.783	15.27	16.773	18.291
8.726	10.077	11.449	12.839	14.242	15.658	17.083
8.076	9.378	10.707	12.058	13.427	14.809	16.203
8.629	10.035	11.478	12.949	14.442	15.953	17.477
9.735	11.181	12.652	14.144	15.653	17.176	18.712

8.991	10.423	11.875	13.342	14.821	16.309	17.805
8.848	10.215	11.606	13.018	14.446	15.887	17.338
9.229	10.682	12.158	13.653	15.163	16.685	18.218
8.639	10.028	11.435	12.857	14.29	15.733	17.184
6.998	8.194	9.404	10.624	11.85	13.079	14.311
8.694	10.139	11.609	13.098	14.602	16.117	17.642
9.235	10.623	12.03	13.455	14.893	16.342	17.801
8.539	9.959	11.4	12.858	14.329	15.81	17.301
8.304	9.639	10.996	12.369	13.754	15.15	16.554
8.229	9.556	10.903	12.265	13.638	15.02	16.409
8.4	9.71	11.042	12.393	13.759	15.137	16.525
8.59	9.953	11.339	12.744	14.163	15.594	17.035
8.953	10.391	11.85	13.324	14.811	16.308	17.813
8.671	10.04	11.425	12.822	14.228	15.642	17.061
8.628	10.019	11.433	12.866	14.314	15.774	17.245
8.504	9.894	11.311	12.748	14.201	15.669	17.147
8.675	10.079	11.506	12.95	14.409	15.879	17.358
7.572	8.914	10.271	11.639	13.017	14.402	15.794
8.8	10.18	11.585	13.009	14.449	15.901	17.363
8.34	9.704	11.089	12.491	13.905	15.33	16.763
7.684	9.167	10.663	12.166	13.675	15.187	16.701
9.143	10.562	12.009	13.476	14.958	16.451	17.954
8.769	10.219	11.692	13.182	14.687	16.204	17.73
9.248	10.703	12.185	13.69	15.213	16.75	18.3
7.696	9.087	10.499	11.926	13.364	14.81	16.261
8.66	10.094	11.558	13.047	14.554	16.077	17.612
8.275	9.653	11.055	12.476	13.913	15.363	16.824
8.296	9.688	11.101	12.527	13.965	15.41	16.86
8.67	10.032	11.414	12.813	14.225	15.648	17.08
9.044	10.464	11.912	13.381	14.868	16.37	17.884
8.987	10.401	11.844	13.31	14.794	16.295	17.807
8.629	10.016	11.429	12.865	14.32	15.79	17.271
8.724	10.087	11.471	12.871	14.283	15.706	17.137
8.873	10.392	11.928	13.476	15.032	16.595	18.163
8.797	10.247	11.726	13.227	14.747	16.282	17.83
7.96	9.289	10.637	11.998	13.37	14.751	16.139
9.003	10.342	11.701	13.075	14.462	15.859	17.264
8.853	10.264	11.7	13.157	14.631	16.121	17.623
8.575	9.952	11.35	12.766	14.195	15.634	17.082
8.99	10.392	11.822	13.274	14.741	16.222	17.712
8.144	9.491	10.852	12.223	13.601	14.986	16.376
7.854	9.199	10.559	11.932	13.315	14.704	16.098
8.827	10.233	11.663	13.112	14.575	16.049	17.533
8.524	9.887	11.271	12.669	14.079	15.498	16.923
8.378	9.733	11.11	12.503	13.91	15.326	16.75
7.972	9.302	10.65	12.013	13.389	14.774	16.167
8.804	10.198	11.61	13.037	14.475	15.922	17.375
8.658	10.008	11.378	12.764	14.163	15.571	16.987
7.659	9.004	10.367	11.745	13.132	14.527	15.927
8.829	10.233	11.661	13.11	14.575	16.053	17.542

8.057	9.403	10.77	12.154	13.553	14.964	16.384
8.477	9.824	11.192	12.577	13.977	15.388	16.809
8.758	10.163	11.593	13.042	14.506	15.984	17.471
8.607	9.988	11.39	12.809	14.243	15.687	17.141
8.872	10.257	11.667	13.097	14.542	15.999	17.464
8.954	10.422	11.916	13.431	14.962	16.507	18.063
8.048	9.369	10.709	12.063	13.428	14.8	16.178
8.263	9.678	11.126	12.601	14.095	15.604	17.123
8.453	9.816	11.205	12.614	14.039	15.476	16.923
8.197	9.554	10.933	12.329	13.738	15.157	16.585
7.763	9.113	10.487	11.879	13.285	14.702	16.127
7.33	8.652	9.986	11.329	12.679	14.035	15.395
8.568	9.982	11.432	12.911	14.413	15.933	17.466
8.862	10.286	11.732	13.194	14.67	16.156	17.65
8.97	10.336	11.72	13.12	14.534	15.958	17.393
9.065	10.54	12.042	13.565	15.103	16.652	18.212
8.227	9.593	10.98	12.384	13.803	15.232	16.671
7.849	9.121	10.412	11.72	13.041	14.373	15.713
7.976	9.385	10.816	12.264	13.724	15.192	16.668
8.792	10.196	11.623	13.069	14.53	16.002	17.483
9.288	10.683	12.098	13.528	14.972	16.425	17.886
8.788	10.236	11.713	13.214	14.734	16.27	17.819
8.937	10.341	11.767	13.212	14.671	16.144	17.626
8.26	9.614	10.99	12.384	13.793	15.214	16.647
8.425	9.789	11.172	12.57	13.98	15.4	16.828
9.021	10.446	11.895	13.362	14.843	16.335	17.835
8.4	9.794	11.21	12.643	14.09	15.549	17.017
9.033	10.504	12.005	13.53	15.076	16.639	18.215
8.917	10.392	11.9	13.434	14.989	16.561	18.146
8.348	9.727	11.126	12.54	13.966	15.402	16.845
8.228	9.616	11.022	12.442	13.874	15.314	16.762
9.079	10.505	11.957	13.433	14.926	16.433	17.953
8.81	10.223	11.657	13.108	14.572	16.047	17.531
8.742	10.165	11.608	13.064	14.532	16.008	17.492
8.811	10.215	11.641	13.082	14.537	16.002	17.475
8.352	9.712	11.09	12.482	13.884	15.293	16.709
7.791	9.151	10.53	11.921	13.323	14.733	16.149
8.63	10.025	11.443	12.879	14.33	15.792	17.264
8.386	9.758	11.16	12.583	14.024	15.478	16.942
9.34	10.738	12.154	13.586	15.03	16.485	17.948
8.829	10.233	11.661	13.11	14.575	16.053	17.542
8.768	10.169	11.59	13.026	14.474	15.931	17.396
8.691	10.105	11.548	13.014	14.498	15.997	17.508
8.992	10.411	11.854	13.316	14.791	16.277	17.771
8.522	9.892	11.283	12.692	14.115	15.55	16.996
8.957	10.44	11.952	13.489	15.044	16.614	18.197
8.591	9.958	11.35	12.763	14.192	15.635	17.089
9.272	10.71	12.171	13.649	15.141	16.642	18.151
8.256	9.705	11.182	12.682	14.199	15.727	17.265
7.724	9.031	10.353	11.688	13.033	14.384	15.742

8.18	9.587	11.033	12.508	14.001	15.509	17.025
8.436	9.873	11.341	12.834	14.347	15.875	17.414
8.514	9.876	11.267	12.681	14.113	15.56	17.018
8.555	9.979	11.439	12.927	14.437	15.965	17.505
8.177	9.537	10.929	12.345	13.781	15.234	16.699
8.997	10.431	11.897	13.389	14.899	16.424	17.96
8.691	10.053	11.441	12.849	14.274	15.713	17.163
8.876	10.314	11.778	13.262	14.762	16.275	17.799
8.234	9.594	10.974	12.37	13.777	15.194	16.619
7.999	9.281	10.579	11.889	13.21	14.541	15.88
8.581	9.901	11.241	12.597	13.969	15.353	16.748
8.847	10.236	11.646	13.073	14.514	15.964	17.423
9.055	10.532	12.034	13.555	15.093	16.641	18.199
8.771	10.217	11.687	13.176	14.679	16.192	17.713
7.925	9.241	10.576	11.927	13.291	14.664	16.045
7.925	9.241	10.576	11.927	13.291	14.664	16.045
8.329	9.643	10.984	12.347	13.728	15.124	16.53
8.208	9.558	10.938	12.344	13.772	15.216	16.674
7.921	9.304	10.711	12.137	13.576	15.025	16.482
8.208	9.558	10.938	12.344	13.772	15.216	16.674
8.332	9.713	11.115	12.534	13.967	15.412	16.867
8.587	9.937	11.315	12.716	14.134	15.565	17.008
8.635	10.01	11.408	12.827	14.26	15.707	17.164
8.635	10.01	11.408	12.827	14.26	15.707	17.164
8.199	9.521	10.86	12.214	13.578	14.95	16.329
8.774	10.121	11.494	12.886	14.293	15.713	17.143
8.357	9.757	11.174	12.603	14.043	15.491	16.947
8.703	10.217	11.756	13.315	14.888	16.474	18.068
8.91	10.3	11.714	13.147	14.595	16.056	17.526
8.452	9.824	11.221	12.64	14.075	15.524	16.984
9.139	10.57	12.027	13.503	14.995	16.497	18.009
8.233	9.586	10.962	12.357	13.765	15.184	16.61
7.682	9.042	10.425	11.825	13.24	14.665	16.098
8.512	9.877	11.265	12.671	14.092	15.525	16.967
9.154	10.606	12.086	13.587	15.105	16.635	18.175
8.407	9.829	11.274	12.734	14.208	15.692	17.185
8.605	9.979	11.38	12.803	14.244	15.698	17.165
8.582	10.036	11.514	13.012	14.526	16.052	17.588
8.801	10.242	11.711	13.201	14.707	16.226	17.755
8.811	10.169	11.552	12.956	14.377	15.812	17.257
8.67	10.133	11.62	13.124	14.641	16.169	17.705
8.834	10.228	11.645	13.081	14.533	15.999	17.476
8.698	10.142	11.61	13.097	14.598	16.111	17.632
8.67	10.12	11.591	13.079	14.577	16.085	17.599
8.352	9.715	11.098	12.496	13.905	15.322	16.746
8.801	10.227	11.682	13.16	14.655	16.165	17.686
8.341	9.695	11.075	12.476	13.893	15.323	16.765
8.591	9.972	11.375	12.795	14.23	15.677	17.133
8.563	9.987	11.431	12.891	14.362	15.842	17.328
8.9	10.371	11.869	13.389	14.925	16.475	18.036

8.775	10.16	11.568	12.992	14.43	15.879	17.337
8.171	9.537	10.916	12.303	13.698	15.1	16.506
8.714	10.165	11.639	13.131	14.634	16.147	17.666
9.288	10.755	12.243	13.75	15.27	16.802	18.343
8.168	9.572	10.999	12.443	13.899	15.366	16.84
8.256	9.705	11.182	12.682	14.199	15.727	17.265
8.221	9.572	10.945	12.336	13.74	15.154	16.574
8.252	9.607	10.984	12.377	13.783	15.198	16.62
8.402	9.723	11.066	12.426	13.8	15.184	16.576
8.547	9.982	11.448	12.94	14.452	15.979	17.518
8.44	9.895	11.378	12.885	14.408	15.945	17.491
8.295	9.698	11.132	12.589	14.063	15.55	17.048
8.594	9.932	11.293	12.675	14.072	15.482	16.902
8.792	10.196	11.623	13.069	14.53	16.002	17.483
8.471	9.851	11.249	12.662	14.086	15.52	16.961
8.619	9.971	11.345	12.738	14.145	15.563	16.99
7.004	8.288	9.594	10.917	12.25	13.591	14.936
8.832	10.19	11.571	12.971	14.384	15.809	17.243
9.179	10.601	12.048	13.516	14.999	16.494	17.998
8.89	10.289	11.715	13.161	14.623	16.099	17.584
9.8	11.267	12.762	14.279	15.814	17.363	18.923
8.962	10.41	11.886	13.383	14.896	16.422	17.958
8.652	10.03	11.433	12.856	14.295	15.747	17.21
8.764	10.16	11.581	13.021	14.478	15.946	17.425
8.297	9.642	11.004	12.382	13.771	15.17	16.576
8.858	10.24	11.646	13.072	14.513	15.966	17.428
8.73	10.125	11.543	12.98	14.432	15.899	17.376
8.559	9.951	11.365	12.798	14.246	15.707	17.177

ESpm12x	ESpm13x	ESpm14x	ESpm15x	ESpm01d	ESpm02d	ESpm03d
18.373	19.87	21.371	22.877	2.326	4.125	4.858
17.994	19.423	20.859	22.301	2.428	4.361	4.968
18.899	20.373	21.853	23.337	2.741	4.652	5.36
17.021	18.384	19.75	21.121	1.747	3.591	4.17
18.263	19.746	21.233	22.725	2.184	4.036	4.605
19.624	21.239	22.856	24.475	0.815	3.782	4.414
18.772	20.254	21.743	23.237	2.501	4.645	5.208
17.807	19.27	20.736	22.205	1.917	3.664	4.31
18.868	20.36	21.858	23.361	1.726	4.35	4.49
17.353	18.759	20.172	21.589	1.747	3.881	4.126
18.465	19.87	21.278	22.69	3.008	4.956	5.581
19.076	20.581	22.091	23.606	2.26	4.63	4.946
18.425	19.899	21.38	22.864	1.445	4.172	4.141
18.884	20.379	21.881	23.387	2.84	4.607	5.332
18.999	20.493	21.993	23.499	3.042	4.82	5.866
18.957	20.455	21.959	23.467	3	4.781	5.832
18.007	19.464	20.925	22.39	1.737	3.909	4.39
19.142	20.604	22.071	23.542	3.183	5.009	5.901
17.226	18.579	19.937	21.301	2.145	4.067	4.612
17.887	19.292	20.701	22.113	2.145	4.309	4.79
18.801	20.294	21.793	23.297	2.48	4.477	5.196
18.84	20.331	21.825	23.322	2.46	4.463	5.272
18.642	20.107	21.577	23.052	1.971	4.342	4.728
18.877	20.376	21.882	23.393	2.98	4.746	5.795
17.689	19.06	20.436	21.816	1.758	4.355	4.375
20.119	21.719	23.323	24.93	2.587	4.831	5.607
17.264	18.609	19.958	21.313	1.914	4.151	4.396
19.567	21.061	22.558	24.059	3.031	5.105	5.718
18.767	20.281	21.802	23.328	1.099	4.217	4.056
17.113	18.459	19.812	21.172	2.14	4.142	4.492
19.666	21.19	22.719	24.252	2.402	4.909	5.45
19.937	21.479	23.026	24.578	3.007	5.221	5.931
18.597	20.078	21.563	23.052	1.391	4.111	4.419
18.045	19.483	20.925	22.37	2.007	4.204	4.625
20.305	21.905	23.511	25.123	2.166	4.989	5.542
18.472	19.92	21.374	22.833	2.376	4.614	5.117
19.509	21.092	22.681	24.276	1.664	4.521	4.854
18.534	20.036	21.543	23.054	2.152	4.088	4.839
18.997	20.486	21.981	23.481	1.841	4.616	4.989
19.491	21.019	22.552	24.088	2.51	4.71	5.353
19.273	20.775	22.283	23.796	1.954	4.733	4.831
18.423	19.894	21.369	22.849	1.609	4.149	4.41
20.178	21.743	23.315	24.891	2.897	5.203	5.969
18.909	20.42	21.937	23.459	1.971	4.289	4.728
19.033	20.548	22.071	23.599	2.14	4.553	4.664
18.779	20.259	21.745	23.234	3.075	4.768	5.757
18.829	20.305	21.787	23.274	3.074	4.791	5.767
18.986	20.459	21.937	23.419	3.315	5.049	6.048
18.869	20.34	21.817	23.299	3.283	5.005	6.007

19.181	20.663	22.149	23.638	3.257	5.045	6.004
19.004	20.484	21.97	23.459	3.319	5.004	6.005
19.295	20.76	22.23	23.704	3.632	5.378	6.356
18.996	20.476	21.962	23.451	3.373	5.012	6.03
19.304	20.844	22.39	23.942	3.281	4.916	5.949
18.868	20.346	21.829	23.317	3.074	4.824	5.76
19.591	21.111	22.636	24.166	3.381	5.186	6.223
19.304	20.783	22.266	23.752	3.357	5.169	6.12
18.821	20.295	21.773	23.255	3.165	4.853	5.853
19.189	20.662	22.14	23.623	3.626	5.273	6.332
18.941	20.423	21.909	23.4	3.349	4.982	6.004
19.524	21.051	22.583	24.118	2.326	4.641	5.446
18.779	20.259	21.745	23.234	3.033	4.751	5.729
18.81	20.283	21.763	23.249	3.033	4.85	5.726
18.743	20.219	21.703	23.192	3.225	4.846	5.858
18.773	20.254	21.739	23.229	3.156	4.767	5.791
17.716	19.132	20.554	21.982	2.039	4.326	4.618
18.033	19.443	20.86	22.283	2.483	4.497	4.999
19.759	21.335	22.914	24.495	2.318	4.382	5.473
17.758	19.167	20.582	22.001	1.633	4.04	4.244
18.383	19.851	21.325	22.803	2.385	4.452	5.207
19.045	20.605	22.17	23.738	2.209	4.165	5.1
18.537	20.019	21.507	23.001	2.336	4.452	5.157
19.665	21.224	22.786	24.351	2.392	4.563	5.456
18.497	19.969	21.447	22.929	2.062	4.182	4.643
17.557	18.954	20.356	21.76	2.692	4.303	5.28
18.215	19.623	21.035	22.451	2.721	4.589	5.3
18.838	20.319	21.805	23.293	2.125	4.472	4.967
18.696	20.183	21.675	23.171	2.468	4.374	5.056
18.832	20.315	21.805	23.299	2.431	4.573	5.149
18.062	19.469	20.881	22.296	2.881	4.715	5.545
18.146	19.606	21.07	22.537	2.107	3.949	4.788
19.137	20.659	22.189	23.726	2.46	4.567	5.091
17.852	19.292	20.737	22.185	1.949	3.86	4.332
18.866	20.365	21.87	23.38	1.954	4.378	4.644
18.427	19.959	21.493	23.027	1.821	3.763	4.152
19.34	20.921	22.507	24.097	2.041	4.259	5.267
18.239	19.701	21.167	22.637	1.472	4.106	4.111
20.119	21.719	23.323	24.93	2.587	4.831	5.607
18.508	19.977	21.451	22.931	2.097	4.292	4.739
18.211	19.654	21.1	22.55	2.255	4.214	4.767
19.417	20.927	22.441	23.959	3.096	4.994	5.93
18.339	19.821	21.308	22.799	2.138	4.166	4.663
18.425	19.884	21.347	22.813	2.097	4.202	4.68
19.663	21.229	22.799	24.374	2.104	4.724	5.228
19.821	21.362	22.911	24.467	3.049	5.365	5.931
18.516	19.956	21.402	22.853	2.975	4.798	5.682
17.606	19.015	20.431	21.851	1.664	3.987	4.17
19.012	20.555	22.105	23.659	1.952	4.392	4.917
20.257	21.812	23.374	24.943	3.464	5.5	6.32

19.307	20.814	22.326	23.842	2.425	4.655	5.399
18.798	20.264	21.736	23.212	2.531	4.745	5.248
19.759	21.308	22.862	24.422	2.862	4.934	5.69
18.641	20.104	21.572	23.043	2.739	4.513	5.372
15.545	16.78	18.015	19.251	1.463	3.438	3.456
19.173	20.71	22.252	23.797	2.163	4.367	4.953
19.267	20.739	22.218	23.701	3.186	5.137	5.783
18.799	20.303	21.812	23.326	2.456	4.325	5.178
17.964	19.379	20.799	22.223	2.082	4.192	4.665
17.805	19.205	20.609	22.017	1.993	4.093	4.592
17.921	19.325	20.734	22.149	1.969	4.365	4.641
18.485	19.942	21.404	22.872	1.971	4.342	4.635
19.324	20.84	22.361	23.885	2.17	4.719	5.319
18.486	19.916	21.349	22.786	2.862	4.664	5.522
18.724	20.211	21.704	23.202	1.85	4.319	4.666
18.635	20.13	21.631	23.137	2.235	4.307	4.923
18.844	20.336	21.834	23.336	2.426	4.47	5.078
17.192	18.593	19.999	21.407	2.187	3.675	4.619
18.833	20.308	21.789	23.274	2.808	4.803	5.626
18.203	19.649	21.098	22.552	1.964	4.138	4.61
18.216	19.732	21.249	22.766	1.449	3.239	4.175
19.463	20.977	22.496	24.017	2.864	5.089	5.838
19.264	20.804	22.35	23.901	2.327	4.424	5.25
19.859	21.427	23.001	24.581	2.699	4.994	5.622
17.718	19.177	20.64	22.104	1.675	3.692	4.287
19.157	20.709	22.267	23.83	2.221	4.395	4.961
18.293	19.77	21.253	22.741	2.276	4.148	4.717
18.316	19.774	21.235	22.699	2.082	4.1	4.758
18.52	19.966	21.418	22.874	2.554	4.683	5.242
19.407	20.938	22.477	24.02	2.837	4.884	5.683
19.331	20.863	22.402	23.947	2.689	4.807	5.512
18.762	20.261	21.767	23.277	1.758	4.427	4.656
18.576	20.021	21.471	22.926	2.937	4.672	5.548
19.735	21.309	22.886	24.465	2.268	4.373	5.458
19.388	20.954	22.526	24.104	2.293	4.536	5.151
17.533	18.933	20.336	21.743	1.841	3.89	4.482
18.676	20.093	21.515	22.941	2.17	4.798	4.952
19.137	20.659	22.189	23.726	2.516	4.572	5.137
18.536	19.997	21.462	22.931	2.293	4.396	4.997
19.21	20.715	22.226	23.741	2.072	4.715	5.022
17.77	19.168	20.569	21.973	3.023	4.499	5.653
17.497	18.9	20.306	21.714	2.592	4.154	5.165
19.024	20.521	22.023	23.53	3.027	4.811	5.719
18.354	19.79	21.229	22.671	2.26	4.339	4.877
18.18	19.616	21.055	22.499	2.082	4.222	4.755
17.567	18.973	20.384	21.799	2.693	4.277	5.314
18.834	20.297	21.764	23.234	2.675	4.66	5.547
18.411	19.84	21.274	22.712	2.506	4.55	5.286
17.332	18.74	20.151	21.565	2.17	3.83	4.72
19.041	20.547	22.06	23.578	2.602	4.668	5.285

17.813	19.249	20.691	22.137	2.007	3.969	4.53
18.236	19.671	21.11	22.554	2.683	4.516	5.206
18.968	20.471	21.981	23.495	2.556	4.596	5.219
18.603	20.072	21.546	23.026	2.318	4.384	4.925
18.936	20.414	21.897	23.383	1.768	4.561	4.547
19.628	21.201	22.78	24.365	2.25	4.536	5.278
17.561	18.947	20.337	21.73	2.336	4.126	4.714
18.649	20.182	21.718	23.258	2.039	4.121	4.755
18.377	19.838	21.303	22.773	1.694	4.239	4.472
18.018	19.457	20.899	22.345	2.007	4.11	4.613
17.558	18.994	20.434	21.877	2.288	3.761	4.716
16.76	18.127	19.498	20.871	1.649	3.16	3.958
19.009	20.56	22.117	23.679	1.991	4.319	4.875
19.151	20.657	22.167	23.68	3.08	4.845	5.86
18.835	20.286	21.743	23.206	2.692	4.79	5.365
19.778	21.349	22.925	24.505	2.466	4.763	5.478
18.119	19.573	21.032	22.496	2.206	4.153	4.696
17.061	18.416	19.777	21.142	1.914	3.941	4.198
18.148	19.633	21.121	22.612	1.993	3.75	4.396
18.972	20.467	21.966	23.47	1.964	4.428	4.679
19.353	20.826	22.303	23.784	2.728	5.075	5.697
19.378	20.944	22.518	24.096	2.123	4.481	4.998
19.117	20.616	22.122	23.633	2.572	4.773	5.313
18.087	19.536	20.99	22.449	2.311	4.183	4.852
18.263	19.704	21.149	22.599	2.01	4.276	4.725
19.342	20.855	22.372	23.893	2.998	4.925	5.852
18.493	19.975	21.463	22.956	2.145	4.193	4.803
19.803	21.4	23.005	24.615	2.123	4.666	5.215
19.74	21.343	22.952	24.566	2.359	4.603	5.262
18.296	19.753	21.214	22.68	1.993	4.093	4.601
18.215	19.674	21.137	22.604	2.069	4.014	4.794
19.482	21.019	22.563	24.113	2.423	4.783	5.248
19.022	20.519	22.021	23.527	2.881	4.608	5.425
18.982	20.476	21.975	23.477	2.349	4.401	5.15
18.956	20.443	21.935	23.432	2.881	4.628	5.436
18.13	19.556	20.985	22.417	2.594	4.285	5.116
17.569	18.994	20.422	21.852	1.539	3.579	3.954
18.743	20.229	21.719	23.215	2.431	4.487	5.069
18.413	19.891	21.373	22.859	1.345	4.106	3.951
19.419	20.896	22.378	23.865	3.177	5.14	5.841
19.041	20.547	22.06	23.578	2.602	4.668	5.285
18.868	20.345	21.825	23.31	2.516	4.572	5.331
19.03	20.559	22.095	23.637	1.716	4.357	4.588
19.272	20.779	22.29	23.805	2.944	4.902	5.823
18.449	19.91	21.377	22.849	2.152	4.3	4.775
19.79	21.391	22.998	24.61	2.453	4.633	5.322
18.551	20.022	21.499	22.982	2.264	4.464	4.995
19.666	21.186	22.709	24.236	2.625	5.098	5.686
18.81	20.36	21.913	23.468	2.412	4.187	5.132
17.106	18.473	19.845	21.22	1.887	3.714	4.292

18.549	20.077	21.609	23.143	1.058	4.024	4.109
18.962	20.516	22.074	23.637	2.471	4.281	5.192
18.485	19.96	21.442	22.929	2.17	4.408	4.912
19.055	20.613	22.175	23.743	2.245	4.311	5.029
18.174	19.657	21.145	22.637	1.477	3.98	4.045
19.506	21.058	22.617	24.18	2.633	4.861	5.656
18.623	20.091	21.566	23.047	2.387	4.572	5.079
19.332	20.872	22.419	23.971	2.316	4.566	5.298
18.051	19.487	20.928	22.373	2.276	4.131	4.805
17.226	18.579	19.937	21.301	2.111	4.062	4.575
18.153	19.567	20.988	22.416	2.664	4.657	5.296
18.889	20.362	21.84	23.322	2.266	4.593	4.936
19.765	21.335	22.91	24.489	2.686	4.779	5.593
19.24	20.771	22.306	23.843	2.036	4.541	5.07
17.433	18.827	20.225	21.628	1.758	3.906	4.288
17.433	18.827	20.225	21.628	1.905	3.929	4.393
17.946	19.37	20.799	22.234	2.097	4.307	4.575
18.142	19.618	21.101	22.588	1.477	4.017	4.045
17.944	19.412	20.883	22.357	1.169	3.655	3.984
18.142	19.618	21.101	22.588	1.477	4.017	4.045
18.331	19.802	21.278	22.76	1.396	3.993	4.119
18.459	19.918	21.382	22.852	2.579	4.63	5.288
18.629	20.101	21.58	23.063	1.815	4.414	4.6
18.629	20.101	21.58	23.063	1.815	4.414	4.6
17.713	19.101	20.493	21.888	2.738	4.39	5.267
18.581	20.026	21.475	22.929	2.519	4.709	5.183
18.408	19.874	21.344	22.817	2.035	4.051	4.803
19.67	21.277	22.889	24.504	0.932	4.029	4.541
19.005	20.489	21.98	23.474	2.427	4.708	5.138
18.453	19.929	21.412	22.899	1.376	4.136	4.129
19.526	21.049	22.576	24.107	2.473	4.987	5.468
18.043	19.482	20.924	22.369	2.007	4.174	4.625
17.537	18.98	20.426	21.875	1.633	3.533	4.074
18.416	19.871	21.331	22.794	2.328	4.453	5.044
19.722	21.276	22.835	24.397	2.584	4.834	5.518
18.683	20.188	21.696	23.209	1.901	4.117	4.831
18.641	20.125	21.616	23.114	2.184	4.479	4.882
19.132	20.683	22.239	23.799	1.692	4.135	4.863
19.291	20.835	22.383	23.936	1.639	4.359	4.912
18.711	20.173	21.642	23.116	2.685	4.783	5.565
19.248	20.796	22.349	23.905	1.81	4.204	4.94
18.964	20.461	21.964	23.475	2.618	4.669	5.435
19.16	20.694	22.234	23.777	2.31	4.451	5.242
19.118	20.641	22.167	23.695	1.933	4.266	4.933
18.176	19.609	21.047	22.487	2.082	4.192	4.755
19.217	20.755	22.299	23.849	2.36	4.532	5.212
18.215	19.673	21.138	22.607	1.847	4.137	4.253
18.597	20.068	21.545	23.028	2.197	4.355	4.862
18.82	20.316	21.815	23.317	1.258	4.108	4.321
19.605	21.183	22.766	24.354	2.176	4.487	5.224

18.802	20.274	21.752	23.235	2.488	4.613	5.087
17.916	19.33	20.746	22.165	1.621	3.912	4.409
19.191	20.72	22.252	23.786	1.841	4.465	4.959
19.891	21.446	23.006	24.57	2.862	4.948	5.745
18.322	19.809	21.301	22.797	2.104	4.016	4.769
18.81	20.36	21.913	23.468	2.448	4.218	5.192
17.999	19.429	20.861	22.295	1.758	4.048	4.31
18.047	19.479	20.914	22.352	1.988	4.182	4.606
17.974	19.377	20.784	22.194	2.26	4.349	4.711
19.066	20.622	22.183	23.748	2.155	4.338	5.113
19.045	20.605	22.17	23.738	2.108	4.165	5.046
18.553	20.064	21.58	23.099	1.697	4.097	4.478
18.331	19.766	21.208	22.654	2.328	4.519	4.972
18.972	20.467	21.966	23.47	1.971	4.43	4.685
18.408	19.861	21.318	22.778	2.119	4.2	4.56
18.424	19.864	21.309	22.758	2.574	4.587	5.175
16.284	17.635	18.987	20.341	1.597	3.331	4.016
18.684	20.131	21.583	23.04	2.293	4.674	5.062
19.51	21.027	22.55	24.077	2.595	5.06	5.596
19.079	20.58	22.087	23.599	2.742	4.749	5.414
20.493	22.071	23.655	25.244	3.321	5.48	6.187
19.502	21.054	22.61	24.172	2.17	4.628	5.087
18.683	20.163	21.649	23.141	2.407	4.461	5.048
18.911	20.405	21.904	23.407	2.306	4.522	5.102
17.989	19.407	20.83	22.257	2.439	4.279	4.999
18.899	20.377	21.859	23.346	2.322	4.643	5.025
18.864	20.36	21.863	23.372	1.991	4.396	4.742
18.657	20.144	21.636	23.134	2.366	4.347	4.971

ESpm04d	ESpm05d	ESpm06d	ESpm07d	ESpm08d	ESpm09d	ESpm10d
6.259	7.377	8.669	9.896	11.17	12.432	13.708
6.355	7.395	8.663	9.844	11.095	12.328	13.584
6.797	7.915	9.237	10.479	11.781	13.065	14.37
5.578	6.581	7.855	9.006	10.252	11.463	12.708
5.987	7.001	8.267	9.44	10.691	11.926	13.188
5.945	7.145	8.542	9.884	11.272	12.653	14.047
6.695	7.783	9.127	10.38	11.706	13.015	14.345
5.679	6.756	8.031	9.241	10.505	11.759	13.03
6.135	6.902	8.237	9.261	10.497	11.627	12.837
5.53	6.292	7.496	8.463	9.607	10.663	11.792
6.962	8.013	9.283	10.474	11.732	12.974	14.241
6.493	7.378	8.695	9.804	11.063	12.261	13.513
5.833	6.453	7.801	8.716	9.933	10.983	12.157
6.7	7.825	9.126	10.378	11.682	12.98	14.297
7.332	8.703	10.171	11.645	13.153	14.676	16.216
7.307	8.686	10.16	11.638	13.149	14.674	16.215
5.887	6.906	8.232	9.434	10.729	11.997	13.295
7.355	8.637	10.054	11.453	12.89	14.334	15.792
5.966	6.938	8.168	9.291	10.506	11.695	12.92
6.289	7.318	8.656	9.874	11.186	12.472	13.789
6.625	7.779	9.124	10.421	11.774	13.125	14.498
6.819	8.112	9.579	11.009	12.483	13.958	15.449
6.293	7.235	8.577	9.73	11.021	12.261	13.55
7.271	8.65	10.126	11.606	13.12	14.648	16.193
5.985	6.667	7.961	8.913	10.102	11.165	12.323
7.139	8.378	9.807	11.186	12.614	14.04	15.482
5.794	6.51	7.678	8.571	9.659	10.63	11.69
7.171	8.231	9.533	10.735	12.007	13.255	14.525
5.868	6.429	7.836	8.73	9.978	11.026	12.219
5.8	6.577	7.709	8.636	9.712	10.702	11.76
7.043	8.229	9.697	11.099	12.579	14.059	15.564
7.425	8.61	9.999	11.336	12.73	14.125	15.542
5.957	6.834	8.113	9.178	10.381	11.512	12.693
6.13	7.121	8.449	9.641	10.935	12.199	13.496
7.132	8.255	9.663	10.974	12.365	13.744	15.149
6.634	7.683	9.022	10.247	11.557	12.846	14.165
6.534	7.544	8.972	10.239	11.631	12.995	14.396
6.264	7.423	8.757	10.038	11.368	12.691	14.031
6.547	7.495	8.814	9.961	11.226	12.447	13.707
6.872	8.019	9.415	10.734	12.125	13.508	14.915
6.54	7.323	8.697	9.759	11.03	12.203	13.451
6.052	6.962	8.337	9.491	10.797	12.043	13.339
7.477	8.665	10.038	11.344	12.701	14.047	15.411
6.278	7.249	8.592	9.761	11.056	12.304	13.596
6.27	6.987	8.31	9.29	10.509	11.6	12.784
7.179	8.482	9.89	11.29	12.724	14.169	15.63
7.184	8.478	9.881	11.278	12.71	14.154	15.614
7.494	8.819	10.247	11.668	13.118	14.576	16.046
7.45	8.774	10.198	11.617	13.066	14.525	15.996

7.439	8.727	10.125	11.509	12.927	14.354	15.797
7.422	8.718	10.113	11.5	12.918	14.346	15.79
7.781	9.079	10.483	11.88	13.308	14.746	16.198
7.435	8.732	10.122	11.506	12.922	14.348	15.79
7.361	8.685	10.099	11.519	12.971	14.439	15.925
7.189	8.473	9.879	11.274	12.706	14.149	15.609
7.727	9.131	10.648	12.173	13.735	15.314	16.911
7.556	8.847	10.254	11.647	13.077	14.514	15.967
7.279	8.583	9.987	11.382	12.809	14.246	15.697
7.749	9.077	10.49	11.902	13.341	14.791	16.254
7.412	8.714	10.106	11.494	12.911	14.339	15.782
7.059	8.397	9.91	11.38	12.889	14.394	15.908
7.155	8.458	9.867	11.269	12.704	14.15	15.611
7.176	8.443	9.851	11.241	12.675	14.118	15.58
7.257	8.557	9.947	11.337	12.758	14.192	15.643
7.177	8.489	9.885	11.288	12.718	14.162	15.621
6.199	7.157	8.522	9.729	11.057	12.353	13.685
6.437	7.425	8.711	9.868	11.123	12.345	13.6
7.033	8.488	10.023	11.549	13.091	14.633	16.18
5.798	6.579	7.874	8.883	10.093	11.2	12.386
6.731	7.988	9.446	10.869	12.348	13.832	15.333
6.628	7.998	9.488	10.966	12.474	13.988	15.512
6.706	7.944	9.412	10.833	12.316	13.801	15.305
7.005	8.347	9.83	11.286	12.777	14.27	15.775
6.101	7.061	8.354	9.497	10.755	11.97	13.228
6.667	7.951	9.324	10.691	12.082	13.478	14.885
6.72	7.859	9.202	10.487	11.834	13.174	14.534
6.593	7.759	9.231	10.617	12.08	13.534	15.009
6.462	7.545	8.843	10.06	11.342	12.608	13.897
6.607	7.642	8.945	10.134	11.406	12.65	13.923
6.965	8.192	9.574	10.935	12.342	13.757	15.191
6.185	7.391	8.742	10.062	11.428	12.796	14.18
6.587	7.586	8.906	10.076	11.353	12.588	13.86
5.726	6.664	7.902	8.996	10.193	11.344	12.533
6.179	6.99	8.269	9.294	10.495	11.609	12.792
5.557	6.489	7.759	8.907	10.155	11.376	12.627
6.94	8.473	10.125	11.769	13.436	15.104	16.778
5.796	6.51	7.866	8.88	10.129	11.264	12.484
7.139	8.378	9.807	11.186	12.614	14.04	15.482
6.253	7.23	8.558	9.727	11.014	12.258	13.543
6.212	7.22	8.515	9.678	10.936	12.156	13.41
7.421	8.745	10.207	11.662	13.162	14.678	16.215
6.116	7.117	8.409	9.579	10.836	12.064	13.32
6.173	7.12	8.421	9.546	10.796	11.993	13.237
6.825	7.898	9.286	10.546	11.897	13.22	14.573
7.413	8.459	9.783	10.997	12.3	13.581	14.893
7.135	8.416	9.842	11.252	12.706	14.17	15.652
5.718	6.531	7.855	8.929	10.188	11.368	12.613
6.473	7.623	9.043	10.387	11.803	13.211	14.641
7.77	8.983	10.35	11.674	13.042	14.408	15.79

6.926	8.174	9.61	11.002	12.442	13.879	15.329
6.742	7.762	9.087	10.291	11.59	12.863	14.169
7.155	8.341	9.723	11.061	12.459	13.86	15.285
6.782	7.992	9.345	10.658	12.012	13.361	14.723
4.899	5.505	6.656	7.496	8.535	9.462	10.461
6.473	7.575	8.934	10.195	11.528	12.843	14.181
7.174	8.236	9.522	10.728	12.006	13.271	14.561
6.543	7.755	9.095	10.428	11.804	13.192	14.597
6.201	7.21	8.555	9.74	11.031	12.275	13.553
6.074	7.092	8.419	9.617	10.915	12.181	13.482
6.218	7.128	8.474	9.626	10.922	12.169	13.466
6.235	7.111	8.454	9.555	10.823	12.016	13.269
6.857	7.955	9.307	10.551	11.868	13.16	14.476
6.994	8.263	9.676	11.057	12.474	13.891	15.319
6.25	7.216	8.565	9.76	11.072	12.354	13.674
6.419	7.526	8.894	10.172	11.529	12.872	14.243
6.532	7.593	8.908	10.12	11.409	12.675	13.966
5.929	7.123	8.403	9.66	10.942	12.222	13.511
7.162	8.47	9.943	11.387	12.871	14.357	15.856
6.132	7.135	8.478	9.669	10.971	12.234	13.535
5.51	6.747	8.05	9.349	10.663	11.98	13.302
7.421	8.702	10.186	11.625	13.115	14.605	16.111
6.783	8.095	9.57	11.025	12.522	14.026	15.544
7.124	8.224	9.574	10.83	12.16	13.473	14.811
5.798	6.884	8.217	9.435	10.723	11.981	13.261
6.433	7.504	8.847	10.106	11.449	12.784	14.147
6.1	7.113	8.371	9.536	10.777	12.002	13.253
6.181	7.296	8.629	9.899	11.236	12.565	13.917
6.658	7.679	8.954	10.129	11.383	12.615	13.874
7.171	8.436	9.871	11.283	12.744	14.213	15.701
7.043	8.277	9.729	11.139	12.613	14.091	15.592
6.29	7.145	8.497	9.598	10.868	12.062	13.313
6.984	8.242	9.643	11.019	12.438	13.861	15.3
7.027	8.482	10.019	11.545	13.087	14.63	16.176
6.665	7.784	9.159	10.45	11.816	13.172	14.552
5.908	6.978	8.288	9.513	10.812	12.095	13.404
6.564	7.323	8.646	9.661	10.895	12.026	13.241
6.614	7.641	8.961	10.151	11.437	12.689	13.974
6.478	7.565	8.905	10.148	11.465	12.761	14.081
6.667	7.669	9.078	10.338	11.719	13.077	14.473
7.126	8.571	10.077	11.589	13.115	14.646	16.183
6.614	7.998	9.477	10.962	12.472	13.99	15.517
7.13	8.394	9.784	11.164	12.583	14.012	15.457
6.363	7.388	8.709	9.896	11.176	12.419	13.693
6.271	7.325	8.679	9.902	11.217	12.496	13.804
6.701	8.033	9.444	10.866	12.313	13.773	15.244
7.1	8.449	9.948	11.424	12.937	14.453	15.981
6.786	7.979	9.368	10.694	12.071	13.438	14.819
6.089	7.311	8.628	9.925	11.242	12.558	13.881
6.719	7.767	9.061	10.257	11.529	12.781	14.059

5.935	6.957	8.227	9.397	10.645	11.871	13.125
6.585	7.674	8.961	10.181	11.461	12.731	14.022
6.654	7.708	9.006	10.206	11.481	12.735	14.016
6.391	7.397	8.708	9.885	11.165	12.412	13.695
6.274	6.96	8.321	9.306	10.547	11.66	12.869
6.851	8.147	9.642	11.106	12.619	14.138	15.672
6.082	7.108	8.382	9.57	10.839	12.09	13.37
6.26	7.435	8.842	10.186	11.593	12.992	14.409
6.051	6.896	8.214	9.297	10.544	11.722	12.959
6.079	7.1	8.417	9.617	10.909	12.175	13.472
6.017	7.274	8.602	9.938	11.292	12.654	14.024
5.097	6.155	7.278	8.395	9.53	10.669	11.814
6.434	7.604	9.038	10.399	11.83	13.253	14.696
7.33	8.689	10.156	11.627	13.135	14.659	16.2
6.832	7.866	9.179	10.37	11.65	12.898	14.176
7.003	8.19	9.59	10.924	12.317	13.703	15.109
6.098	7.09	8.35	9.495	10.728	11.933	13.17
5.566	6.278	7.439	8.329	9.418	10.388	11.452
5.824	6.943	8.271	9.531	10.845	12.149	13.467
6.264	7.088	8.401	9.452	10.682	11.825	13.034
7.243	8.394	9.805	11.139	12.551	13.959	15.395
6.518	7.574	8.935	10.193	11.544	12.882	14.252
6.792	7.812	9.12	10.305	11.579	12.824	14.1
6.193	7.242	8.49	9.666	10.908	12.134	13.386
6.134	7.056	8.286	9.367	10.554	11.695	12.875
7.356	8.689	10.164	11.628	13.137	14.659	16.201
6.265	7.321	8.635	9.84	11.125	12.384	13.671
6.759	7.862	9.253	10.558	11.95	13.336	14.751
6.785	7.955	9.356	10.692	12.093	13.489	14.907
6.09	7.097	8.426	9.615	10.912	12.174	13.474
6.184	7.343	8.66	9.922	11.229	12.523	13.831
6.797	7.823	9.188	10.421	11.757	13.066	14.41
6.814	7.994	9.318	10.601	11.923	13.239	14.568
6.662	7.888	9.301	10.661	12.07	13.474	14.894
6.819	7.992	9.312	10.59	11.907	13.22	14.545
6.499	7.684	9	10.279	11.591	12.898	14.216
5.387	6.254	7.495	8.534	9.711	10.814	11.97
6.523	7.561	8.864	10.055	11.328	12.574	13.849
5.73	6.3	7.693	8.6	9.842	10.898	12.088
7.239	8.333	9.627	10.847	12.129	13.397	14.687
6.719	7.767	9.061	10.257	11.529	12.781	14.059
6.874	8.164	9.66	11.129	12.653	14.183	15.73
6.245	7.15	8.527	9.692	11.004	12.263	13.568
7.339	8.675	10.154	11.621	13.132	14.655	16.198
6.274	7.258	8.584	9.759	11.049	12.301	13.593
6.828	8.001	9.394	10.726	12.121	13.514	14.929
6.519	7.61	8.989	10.272	11.639	12.992	14.373
7.247	8.351	9.724	10.98	12.31	13.613	14.937
6.562	7.849	9.239	10.617	12.025	13.439	14.867
5.68	6.684	7.947	9.094	10.33	11.534	12.771

5.896	6.847	8.31	9.554	10.936	12.263	13.627
6.645	7.93	9.34	10.731	12.159	13.592	15.041
6.463	7.559	8.954	10.245	11.621	12.979	14.364
6.553	7.785	9.217	10.598	12.033	13.466	14.916
5.625	6.297	7.577	8.504	9.68	10.72	11.863
7.238	8.571	10.08	11.557	13.075	14.594	16.124
6.57	7.591	8.914	10.114	11.408	12.675	13.974
6.859	8.138	9.614	11.054	12.546	14.042	15.555
6.19	7.285	8.574	9.796	11.078	12.347	13.637
5.944	6.898	8.13	9.242	10.453	11.633	12.852
6.709	7.772	9.064	10.273	11.553	12.817	14.104
6.473	7.377	8.692	9.814	11.075	12.282	13.537
7.086	8.314	9.71	11.058	12.451	13.842	15.248
6.638	7.717	9.089	10.342	11.677	12.986	14.319
5.763	6.692	7.979	9.108	10.355	11.559	12.805
5.833	6.816	8.108	9.278	10.547	11.788	13.063
6.102	6.972	8.284	9.392	10.652	11.856	13.113
5.646	6.278	7.581	8.481	9.67	10.695	11.843
5.461	6.356	7.619	8.701	9.909	11.059	12.255
5.646	6.278	7.581	8.481	9.67	10.695	11.843
5.734	6.431	7.742	8.686	9.889	10.946	12.112
6.81	8.029	9.479	10.886	12.356	13.83	15.323
6.215	7.004	8.333	9.368	10.605	11.743	12.956
6.215	7.004	8.333	9.368	10.605	11.743	12.956
6.638	7.869	9.221	10.563	11.941	13.326	14.727
6.663	7.652	8.956	10.121	11.386	12.615	13.876
6.362	7.627	9.095	10.514	11.983	13.448	14.927
6.105	7.235	8.633	9.946	11.33	12.702	14.093
6.638	7.595	8.903	10.058	11.329	12.565	13.844
5.798	6.433	7.743	8.651	9.833	10.862	12.003
7.038	8.086	9.452	10.685	12.009	13.302	14.623
6.117	7.121	8.445	9.641	10.934	12.198	13.495
5.547	6.577	7.896	9.086	10.367	11.612	12.887
6.525	7.608	8.951	10.198	11.52	12.824	14.151
7.052	8.288	9.744	11.167	12.65	14.142	15.655
6.336	7.566	8.986	10.366	11.794	13.222	14.664
6.426	7.432	8.78	9.997	11.315	12.609	13.937
6.486	7.825	9.355	10.849	12.382	13.914	15.453
6.601	7.876	9.411	10.882	12.41	13.932	15.467
7.121	8.422	9.908	11.36	12.86	14.363	15.882
6.548	7.878	9.397	10.882	12.407	13.932	15.466
6.973	8.226	9.691	11.115	12.599	14.087	15.596
6.778	8.082	9.553	10.998	12.486	13.98	15.489
6.501	7.75	9.218	10.641	12.117	13.592	15.082
6.26	7.332	8.675	9.905	11.214	12.496	13.802
6.738	8.005	9.484	10.937	12.445	13.957	15.485
5.795	6.5	7.772	8.726	9.901	10.958	12.101
6.356	7.373	8.71	9.916	11.226	12.507	13.825
5.882	6.686	7.961	8.976	10.164	11.259	12.419
6.815	8.122	9.626	11.096	12.614	14.135	15.672

6.554	7.538	8.847	10.025	11.309	12.565	13.859
5.799	6.732	7.95	9.024	10.198	11.325	12.49
6.552	7.612	8.984	10.22	11.543	12.834	14.149
7.217	8.431	9.823	11.174	12.577	13.981	15.405
6.2	7.367	8.706	9.992	11.324	12.649	13.99
6.63	7.932	9.329	10.714	12.124	13.539	14.966
5.829	6.655	7.928	8.968	10.165	11.285	12.461
6	6.918	8.131	9.211	10.382	11.519	12.686
6.164	7.038	8.303	9.377	10.591	11.745	12.953
6.683	8.001	9.498	10.965	12.475	13.987	15.512
6.598	7.964	9.466	10.948	12.463	13.98	15.507
6.048	7.049	8.428	9.656	10.998	12.306	13.649
6.447	7.43	8.74	9.922	11.208	12.471	13.771
6.269	7.095	8.408	9.46	10.69	11.835	13.045
5.998	6.838	8.086	9.122	10.316	11.438	12.621
6.598	7.656	8.958	10.174	11.465	12.742	14.046
5.449	6.603	7.939	9.214	10.531	11.835	13.149
6.633	7.628	8.985	10.19	11.505	12.786	14.102
7.16	8.229	9.594	10.828	12.148	13.436	14.75
6.883	8.049	9.442	10.787	12.201	13.619	15.062
7.689	8.873	10.269	11.609	13.01	14.409	15.829
6.657	7.693	9.058	10.289	11.614	12.911	14.237
6.519	7.575	8.896	10.105	11.396	12.66	13.952
6.647	7.749	9.131	10.405	11.76	13.093	14.452
6.378	7.5	8.792	10.031	11.318	12.597	13.891
6.548	7.472	8.778	9.902	11.154	12.356	13.603
6.295	7.203	8.542	9.678	10.964	12.193	13.473
6.417	7.468	8.774	9.969	11.245	12.493	13.768

ESpm11d	ESpm12d	ESpm13d	ESpm14d	ESpm15d	ESpm01r	ESpm02r
14.982	16.263	17.544	18.828	20.113	2.89	4.161
14.838	16.102	17.367	18.636	19.907	3.195	4.455
15.672	16.982	18.293	19.608	20.925	3.371	4.694
13.944	15.194	16.443	17.697	18.952	2.468	3.681
14.45	15.724	17.002	18.285	19.571	2.809	4.118
15.441	16.838	18.237	19.637	21.037	2.617	4.015
15.676	17.014	18.355	19.699	21.046	3.466	4.781
14.301	15.579	16.859	18.142	19.427	2.451	3.721
14.015	15.224	16.424	17.638	18.851	3.207	4.563
12.89	14.02	15.14	16.274	17.406	2.901	4.12
15.506	16.781	18.059	19.342	20.626	3.694	5.075
14.751	16.009	17.267	18.533	19.802	3.456	4.797
13.269	14.433	15.576	16.741	17.901	3.165	4.448
15.618	16.947	18.281	19.619	20.961	3.343	4.657
17.769	19.331	20.901	22.478	24.06	3.296	4.704
17.768	19.331	20.901	22.478	24.06	3.266	4.662
14.59	15.896	17.203	18.514	19.828	2.76	4.055
17.257	18.73	20.207	21.688	23.172	3.638	5.049
14.142	15.38	16.62	17.868	19.119	2.96	4.202
15.104	16.432	17.762	19.097	20.436	3.148	4.463
15.876	17.265	18.66	20.061	21.466	3.199	4.566
16.945	18.449	19.957	21.469	22.984	2.996	4.361
14.831	16.131	17.432	18.743	20.056	3.144	4.487
17.749	19.315	20.888	22.467	24.051	3.231	4.615
13.435	14.586	15.722	16.874	18.022	3.367	4.626
16.927	18.379	19.835	21.295	22.756	3.45	4.922
12.7	13.751	14.782	15.833	16.877	3.127	4.38
15.791	17.065	18.338	19.616	20.894	3.842	5.196
13.339	14.518	15.671	16.85	18.021	3.19	4.496
12.782	13.836	14.875	15.93	16.98	3.109	4.345
17.076	18.598	20.126	21.658	23.194	3.699	5.064
16.967	18.405	19.85	21.303	22.763	3.886	5.329
13.849	15.025	16.193	17.368	18.542	3.025	4.36
14.789	16.095	17.403	18.717	20.033	3.122	4.388
16.557	17.977	19.402	20.833	22.268	3.676	5.183
15.485	16.818	18.157	19.504	20.855	3.437	4.757
15.796	17.21	18.626	20.046	21.469	3.343	4.751
15.374	16.725	18.079	19.438	20.8	2.728	4.107
14.958	16.225	17.492	18.767	20.044	3.478	4.851
16.326	17.747	19.174	20.606	22.041	3.411	4.819
14.672	15.92	17.163	18.417	19.672	3.608	4.942
14.622	15.923	17.222	18.528	19.835	3.035	4.34
16.777	18.15	19.529	20.912	22.3	3.829	5.29
14.878	16.178	17.478	18.787	20.098	3.054	4.417
13.922	15.097	16.256	17.43	18.6	3.484	4.783
17.101	18.582	20.072	21.568	23.07	3.243	4.615
17.084	18.565	20.054	21.549	23.05	3.296	4.668
17.524	19.009	20.5	21.996	23.495	3.503	4.868
17.475	18.962	20.454	21.951	23.451	3.421	4.796

17.251	18.715	20.189	21.67	23.159	3.517	4.913
17.245	18.71	20.184	21.666	23.155	3.437	4.803
17.66	19.13	20.608	22.091	23.58	3.854	5.227
17.244	18.709	20.182	21.664	23.152	3.431	4.8
17.423	18.933	20.452	21.978	23.511	3.367	4.752
17.079	18.56	20.048	21.544	23.044	3.367	4.722
18.519	20.136	21.761	23.392	25.027	3.658	5.038
17.428	18.899	20.377	21.86	23.349	3.658	5.046
17.157	18.627	20.105	21.59	23.08	3.262	4.633
17.726	19.206	20.694	22.187	23.685	3.622	5.009
17.237	18.702	20.177	21.659	23.148	3.424	4.77
17.424	18.944	20.464	21.986	23.509	3.314	4.722
17.082	18.564	20.053	21.549	23.05	3.266	4.623
17.053	18.537	20.028	21.527	23.031	3.414	4.766
17.107	18.581	20.066	21.558	23.056	3.314	4.67
17.091	18.571	20.059	21.554	23.055	3.266	4.623
15.014	16.356	17.7	19.049	20.401	3.254	4.512
14.849	16.111	17.373	18.64	19.909	3.346	4.638
17.727	19.275	20.824	22.373	23.922	2.862	4.312
13.538	14.723	15.897	17.085	18.272	2.991	4.266
16.841	18.356	19.875	21.397	22.92	3.1	4.443
17.041	18.575	20.112	21.651	23.192	2.833	4.135
16.813	18.328	19.846	21.367	22.889	3.114	4.449
17.285	18.8	20.319	21.841	23.366	3.174	4.599
14.477	15.743	17.009	18.284	19.561	2.976	4.34
16.296	17.713	19.132	20.555	21.981	2.791	4.005
15.897	17.268	18.641	20.019	21.398	3.281	4.58
16.487	17.971	19.458	20.948	22.439	3.329	4.655
15.186	16.485	17.787	19.096	20.407	3.068	4.422
15.193	16.475	17.759	19.049	20.343	3.3	4.668
16.635	18.088	19.548	21.013	22.483	3.277	4.652
15.57	16.966	18.366	19.769	21.174	2.639	3.98
15.123	16.4	17.677	18.96	20.245	3.292	4.648
13.709	14.902	16.092	17.29	18.488	2.653	3.974
13.948	15.132	16.31	17.501	18.693	3.235	4.588
13.873	15.127	16.382	17.639	18.896	2.588	3.879
18.453	20.129	21.805	23.482	25.159	2.901	4.243
13.669	14.885	16.091	17.311	18.528	3.114	4.383
16.927	18.379	19.835	21.295	22.756	3.45	4.922
14.821	16.116	17.411	18.714	20.02	3.096	4.427
14.656	15.914	17.172	18.436	19.702	2.976	4.291
17.764	19.326	20.896	22.473	24.055	3.466	4.906
14.571	15.833	17.096	18.364	19.634	2.929	4.272
14.468	15.717	16.965	18.221	19.479	2.955	4.286
15.923	17.284	18.648	20.018	21.392	3.447	4.89
16.207	17.536	18.87	20.214	21.563	4.088	5.521
17.144	18.646	20.156	21.671	23.192	3.414	4.747
13.835	15.081	16.32	17.568	18.815	3.011	4.244
16.075	17.516	18.961	20.409	21.86	3.281	4.588
17.177	18.573	19.974	21.382	22.794	4.127	5.564

16.781	18.237	19.695	21.154	22.613	3.384	4.721
15.472	16.788	18.107	19.43	20.756	3.512	4.872
16.72	18.166	19.621	21.083	22.55	3.561	5.002
16.088	17.459	18.833	20.211	21.591	3.105	4.404
11.419	12.404	13.374	14.353	15.328	2.58	3.702
15.519	16.866	18.216	19.571	20.93	3.153	4.511
15.854	17.16	18.471	19.79	21.114	3.833	5.247
16.013	17.437	18.867	20.302	21.74	2.944	4.269
14.819	16.096	17.37	18.649	19.927	3.054	4.298
14.778	16.087	17.398	18.715	20.034	2.976	4.239
14.755	16.062	17.371	18.687	20.006	3.318	4.578
14.504	15.761	17.017	18.282	19.55	3.161	4.487
15.788	17.109	18.432	19.76	21.09	3.434	4.861
16.751	18.188	19.627	21.069	22.512	3.303	4.675
14.993	16.326	17.662	19.003	20.348	3.144	4.497
15.616	17	18.388	19.782	21.178	3.049	4.374
15.253	16.551	17.849	19.152	20.457	3.174	4.534
14.801	16.096	17.393	18.693	19.994	2.272	3.515
17.358	18.865	20.374	21.885	23.398	3.447	4.815
14.828	16.135	17.443	18.757	20.073	2.981	4.271
14.626	15.951	17.278	18.605	19.933	1.946	3.222
17.621	19.138	20.658	22.181	23.707	3.75	5.14
17.069	18.6	20.134	21.672	23.212	3.086	4.447
16.15	17.5	18.853	20.213	21.577	3.699	5.124
14.534	15.816	17.097	18.382	19.668	2.557	3.804
15.516	16.896	18.281	19.671	21.064	3.165	4.571
14.507	15.772	17.042	18.319	19.6	2.912	4.236
15.271	16.634	18.001	19.371	20.743	2.923	4.225
15.131	16.399	17.669	18.945	20.224	3.466	4.855
17.199	18.707	20.222	21.743	23.268	3.597	4.929
17.102	18.622	20.147	21.678	23.213	3.544	4.866
14.544	15.796	17.044	18.3	19.558	3.273	4.624
16.746	18.201	19.662	21.128	22.598	3.329	4.67
17.722	19.27	20.818	22.367	23.916	2.862	4.312
15.936	17.331	18.731	20.136	21.546	3.231	4.671
14.712	16.029	17.348	18.672	19.997	2.773	4.027
14.427	15.647	16.862	18.093	19.326	3.704	5.02
15.253	16.544	17.837	19.135	20.436	3.292	4.648
15.397	16.722	18.047	19.375	20.705	3.174	4.499
15.871	17.284	18.7	20.123	21.548	3.597	4.928
17.721	19.262	20.805	22.348	23.892	2.821	4.156
17.049	18.584	20.123	21.663	23.205	2.625	3.967
16.912	18.376	19.847	21.324	22.806	3.374	4.725
14.958	16.233	17.507	18.787	20.067	3.118	4.415
15.104	16.413	17.721	19.033	20.346	3.054	4.324
16.722	18.207	19.696	21.188	22.684	2.734	4.064
17.514	19.053	20.596	22.142	23.69	3.239	4.621
16.2	17.587	18.975	20.365	21.756	3.19	4.474
15.205	16.533	17.862	19.193	20.525	2.565	3.809
15.336	16.624	17.916	19.213	20.515	3.353	4.75

14.376	15.639	16.903	18.174	19.447	2.874	4.119
15.315	16.617	17.923	19.235	20.55	3.239	4.569
15.295	16.585	17.878	19.177	20.479	3.288	4.663
14.974	16.269	17.566	18.87	20.178	3.109	4.473
14.038	15.244	16.439	17.65	18.86	3.481	4.8
17.212	18.758	20.308	21.861	23.415	3.199	4.599
14.65	15.941	17.235	18.534	19.837	2.912	4.184
15.829	17.255	18.683	20.113	21.545	2.97	4.265
14.178	15.421	16.662	17.913	19.166	3.144	4.483
14.766	16.072	17.38	18.694	20.01	2.981	4.27
15.399	16.778	18.16	19.545	20.932	2.389	3.688
12.964	14.118	15.275	16.434	17.596	2.104	3.313
16.142	17.595	19.051	20.509	21.969	3.215	4.509
17.754	19.319	20.891	22.469	24.052	3.314	4.723
15.448	16.731	18.015	19.303	20.594	3.547	4.881
16.518	17.936	19.359	20.786	22.217	3.428	4.874
14.402	15.649	16.898	18.155	19.414	2.976	4.259
12.464	13.521	14.556	15.615	16.664	2.934	4.156
14.787	16.112	17.439	18.77	20.101	2.501	3.773
14.218	15.428	16.631	17.847	19.062	3.235	4.608
16.838	18.296	19.761	21.233	22.712	3.793	5.156
15.625	17.011	18.402	19.799	21.198	3.215	4.663
15.372	16.657	17.944	19.237	20.535	3.487	4.881
14.637	15.9	17.166	18.437	19.711	3.001	4.296
14.043	15.226	16.407	17.595	18.783	3.127	4.475
17.754	19.319	20.891	22.469	24.052	3.463	4.862
14.954	16.248	17.544	18.846	20.15	2.929	4.257
16.172	17.605	19.044	20.489	21.937	3.398	4.86
16.331	17.764	19.203	20.648	22.096	3.307	4.743
14.769	16.079	17.39	18.707	20.027	2.923	4.225
15.137	16.447	17.757	19.068	20.381	2.779	4.117
15.752	17.108	18.467	19.833	21.203	3.523	4.931
15.899	17.235	18.574	19.916	21.26	3.27	4.604
16.317	17.748	19.181	20.62	22.061	3.096	4.424
15.871	17.203	18.538	19.875	21.215	3.307	4.633
15.536	16.859	18.185	19.513	20.843	2.934	4.23
13.098	14.249	15.39	16.54	17.687	2.51	3.778
15.12	16.403	17.689	18.981	20.277	3.223	4.569
13.21	14.384	15.535	16.708	17.873	3.127	4.39
15.978	17.278	18.582	19.891	21.203	3.831	5.219
15.336	16.624	17.916	19.213	20.515	3.353	4.75
17.283	18.842	20.403	21.967	23.533	3.219	4.579
14.864	16.176	17.488	18.806	20.127	3.195	4.542
17.752	19.317	20.89	22.468	24.052	3.44	4.843
14.879	16.18	17.484	18.795	20.108	3.091	4.425
16.351	17.783	19.22	20.663	22.111	3.292	4.754
15.756	17.149	18.547	19.951	21.357	3.288	4.593
16.256	17.582	18.908	20.238	21.57	3.824	5.252
16.303	17.745	19.193	20.646	22.102	2.845	4.152
14	15.243	16.485	17.733	18.983	2.61	3.814

14.979	16.34	17.698	19.058	20.419	3.03	4.31
16.498	17.962	19.431	20.905	22.383	2.955	4.271
15.749	17.145	18.544	19.948	21.355	3.258	4.545
16.371	17.834	19.3	20.77	22.243	3.091	4.408
12.957	14.094	15.214	16.353	17.487	2.944	4.222
17.656	19.193	20.731	22.27	23.811	3.529	4.887
15.27	16.578	17.889	19.207	20.527	3.371	4.704
17.076	18.603	20.135	21.671	23.209	3.296	4.647
14.927	16.227	17.53	18.838	20.149	2.896	4.215
14.066	15.297	16.529	17.77	19.013	2.955	4.199
15.391	16.688	17.988	19.291	20.598	3.44	4.76
14.782	16.044	17.307	18.579	19.853	3.411	4.761
16.659	18.077	19.5	20.928	22.359	3.408	4.841
15.647	16.984	18.322	19.663	21.005	3.296	4.71
14.041	15.293	16.545	17.803	19.062	2.868	4.083
14.333	15.615	16.899	18.187	19.477	2.856	4.077
14.358	15.624	16.89	18.164	19.441	3.273	4.5
12.928	14.066	15.18	16.318	17.448	3.006	4.27
13.432	14.627	15.818	17.016	18.214	2.625	3.905
12.928	14.066	15.18	16.318	17.448	3.006	4.27
13.222	14.378	15.513	16.668	17.817	2.879	4.205
16.824	18.332	19.844	21.361	22.879	3.367	4.676
14.138	15.35	16.555	17.772	18.989	3.292	4.644
14.138	15.35	16.555	17.772	18.989	3.292	4.644
16.135	17.55	18.97	20.394	21.822	3.025	4.269
15.13	16.396	17.662	18.933	20.206	3.564	4.859
16.407	17.891	19.377	20.864	22.352	2.695	4.113
15.485	16.883	18.283	19.685	21.088	2.868	4.266
15.121	16.415	17.715	19.025	20.339	3.526	4.877
13.087	14.218	15.33	16.462	17.589	3.091	4.396
15.939	17.266	18.594	19.927	21.263	3.75	5.167
14.788	16.094	17.402	18.716	20.033	3.077	4.35
14.153	15.429	16.704	17.982	19.261	2.38	3.603
15.477	16.81	18.145	19.483	20.822	3.215	4.586
17.177	18.708	20.244	21.785	23.329	3.564	4.941
16.11	17.561	19.015	20.471	21.928	2.901	4.213
15.264	16.605	17.949	19.3	20.654	3.353	4.659
16.994	18.537	20.08	21.624	23.168	2.912	4.247
17.004	18.544	20.085	21.628	23.171	3.186	4.517
17.407	18.94	20.477	22.018	23.562	3.478	4.788
17.004	18.544	20.085	21.628	23.171	2.965	4.316
17.112	18.636	20.166	21.7	23.238	3.3	4.65
17.005	18.527	20.053	21.583	23.115	3.178	4.514
16.574	18.071	19.571	21.072	22.574	3.049	4.386
15.102	16.41	17.718	19.03	20.342	2.976	4.27
17.017	18.555	20.095	21.637	23.18	3.343	4.653
13.202	14.338	15.461	16.598	17.732	3.14	4.386
15.141	16.471	17.805	19.145	20.489	3.131	4.481
13.548	14.701	15.844	16.998	18.149	2.976	4.336
17.213	18.76	20.31	21.863	23.418	3.157	4.548

15.152	16.461	17.775	19.096	20.422	3.398	4.755
13.639	14.804	15.965	17.132	18.299	2.785	4.12
15.458	16.776	18.093	19.415	20.737	3.219	4.636
16.836	18.277	19.725	21.179	22.637	3.561	5.016
15.333	16.684	18.038	19.396	20.757	2.667	4.025
16.399	17.839	19.283	20.732	22.185	2.815	4.137
13.612	14.784	15.948	17.12	18.29	2.918	4.242
13.846	15.017	16.187	17.363	18.54	3.059	4.38
14.143	15.355	16.565	17.785	19.006	3.254	4.512
17.041	18.575	20.112	21.651	23.192	3.114	4.409
17.038	18.573	20.11	21.65	23.191	2.923	4.195
14.986	16.335	17.684	19.037	20.391	3.001	4.322
15.072	16.389	17.71	19.04	20.374	3.346	4.676
14.229	15.439	16.643	17.859	19.074	3.223	4.605
13.782	14.969	16.149	17.341	18.533	3.054	4.378
15.354	16.674	18	19.333	20.672	3.388	4.705
14.46	15.774	17.088	18.402	19.716	2.303	3.404
15.414	16.739	18.065	19.398	20.734	3.547	4.841
16.058	17.375	18.693	20.015	21.339	3.804	5.216
16.515	17.978	19.448	20.924	22.404	3.497	4.815
17.256	18.694	20.138	21.588	23.042	4.154	5.598
15.559	16.893	18.228	19.568	20.912	3.408	4.801
15.24	16.54	17.841	19.148	20.458	3.266	4.57
15.81	17.178	18.548	19.924	21.302	3.231	4.551
15.189	16.493	17.801	19.113	20.429	3.006	4.326
14.84	16.095	17.351	18.615	19.882	3.447	4.807
14.741	16.027	17.313	18.607	19.902	3.235	4.574
15.037	16.317	17.597	18.882	20.169	3.059	4.396

ESpm03r	ESpm04r	ESpm05r	ESpm06r	ESpm07r	ESpm08r	ESpm09r
5.26	6.507	7.781	9.095	10.435	11.796	13.174
5.509	6.716	7.932	9.181	10.448	11.732	13.028
5.796	7.057	8.334	9.649	10.986	12.341	13.712
4.718	5.897	7.078	8.286	9.506	10.736	11.974
5.148	6.381	7.627	8.922	10.241	11.583	12.941
5.343	6.792	8.28	9.8	11.341	12.897	14.463
5.86	7.137	8.446	9.805	11.192	12.604	14.032
4.752	5.981	7.234	8.535	9.859	11.201	12.556
5.623	6.876	8.143	9.456	10.793	12.151	13.524
5.12	6.29	7.471	8.691	9.931	11.189	12.46
6.185	7.464	8.754	10.076	11.414	12.768	14.132
5.845	7.091	8.354	9.663	10.997	12.354	13.727
5.493	6.717	7.962	9.253	10.571	11.912	13.27
5.716	6.97	8.246	9.57	10.922	12.297	13.69
5.861	7.188	8.534	9.919	11.326	12.752	14.193
5.811	7.137	8.484	9.874	11.287	12.719	14.165
5.128	6.374	7.644	8.959	10.299	11.661	13.039
6.178	7.468	8.77	10.108	11.463	12.833	14.215
5.233	6.411	7.588	8.792	10.005	11.229	12.46
5.523	6.765	8.02	9.317	10.635	11.971	13.319
5.682	6.97	8.274	9.621	10.991	12.383	13.791
5.403	6.654	7.914	9.219	10.545	11.888	13.242
5.574	6.825	8.089	9.388	10.705	12.038	13.381
5.755	7.073	8.412	9.796	11.204	12.635	14.081
5.624	6.805	7.997	9.232	10.487	11.761	13.048
6.181	7.606	9.068	10.573	12.105	13.657	15.224
5.407	6.596	7.786	9.007	10.238	11.479	12.727
6.313	7.588	8.88	10.208	11.557	12.924	14.304
5.551	6.785	8.04	9.344	10.675	12.031	13.404
5.332	6.487	7.637	8.82	10.013	11.218	12.431
6.167	7.46	8.77	10.123	11.499	12.892	14.298
6.499	7.843	9.209	10.617	12.051	13.507	14.98
5.501	6.796	8.109	9.453	10.813	12.184	13.564
5.433	6.654	7.894	9.179	10.486	11.814	13.154
6.437	7.863	9.315	10.808	12.326	13.865	15.421
5.824	7.087	8.372	9.707	11.069	12.454	13.856
5.911	7.271	8.664	10.111	11.592	13.101	14.632
5.229	6.526	7.841	9.192	10.563	11.95	13.35
6.019	7.342	8.691	10.078	11.49	12.923	14.371
5.961	7.289	8.637	10.029	11.445	12.88	14.329
6	7.248	8.513	9.825	11.16	12.516	13.887
5.401	6.646	7.909	9.217	10.55	11.904	13.273
6.551	7.965	9.407	10.884	12.384	13.902	15.433
5.519	6.787	8.069	9.39	10.731	12.089	13.462
5.808	7.029	8.267	9.555	10.871	12.212	13.571
5.708	6.99	8.29	9.635	11.004	12.392	13.794
5.778	7.065	8.367	9.71	11.074	12.458	13.856
5.944	7.215	8.502	9.835	11.191	12.566	13.957
5.853	7.116	8.388	9.707	11.051	12.418	13.802

6.023	7.327	8.647	10.01	11.393	12.793	14.205
5.889	7.164	8.453	9.787	11.144	12.521	13.914
6.32	7.596	8.885	10.216	11.568	12.937	14.32
5.884	7.16	8.449	9.783	11.141	12.519	13.913
5.862	7.178	8.519	9.914	11.342	12.797	14.273
5.82	7.1	8.397	9.737	11.1	12.482	13.879
6.161	7.459	8.773	10.128	11.505	12.901	14.311
6.157	7.454	8.768	10.124	11.502	12.899	14.308
5.711	6.991	8.285	9.626	10.99	12.374	13.772
6.097	7.377	8.666	9.997	11.35	12.721	14.106
5.853	7.125	8.416	9.753	11.114	12.496	13.893
5.88	7.193	8.523	9.886	11.265	12.658	14.06
5.722	7.006	8.308	9.654	11.024	12.413	13.816
5.824	7.082	8.354	9.675	11.022	12.392	13.781
5.745	7.013	8.294	9.62	10.971	12.345	13.737
5.722	7.006	8.308	9.654	11.024	12.413	13.816
5.499	6.688	7.896	9.155	10.443	11.757	13.089
5.652	6.852	8.061	9.316	10.595	11.896	13.215
5.51	6.872	8.244	9.649	11.069	12.501	13.943
5.286	6.482	7.691	8.943	10.22	11.517	12.831
5.479	6.71	7.945	9.227	10.533	11.862	13.209
5.231	6.504	7.802	9.145	10.515	11.907	13.315
5.496	6.731	7.974	9.264	10.579	11.918	13.275
5.782	7.133	8.508	9.923	11.361	12.817	14.285
5.415	6.677	7.953	9.271	10.61	11.968	13.339
4.946	6.104	7.277	8.5	9.746	11.01	12.286
5.605	6.821	8.043	9.305	10.585	11.881	13.189
5.739	6.996	8.273	9.594	10.938	12.3	13.676
5.521	6.797	8.089	9.419	10.769	12.136	13.515
5.781	7.063	8.357	9.688	11.039	12.406	13.785
5.743	7.012	8.293	9.61	10.944	12.294	13.656
5.121	6.423	7.747	9.109	10.494	11.896	13.31
5.743	7.011	8.293	9.617	10.965	12.336	13.724
4.973	6.178	7.392	8.65	9.928	11.224	12.533
5.637	6.876	8.12	9.406	10.712	12.037	13.376
4.96	6.27	7.624	9.034	10.472	11.928	13.395
5.352	6.638	7.945	9.298	10.677	12.077	13.494
5.4	6.616	7.854	9.143	10.46	11.801	13.158
6.181	7.606	9.068	10.573	12.105	13.657	15.224
5.491	6.738	7.999	9.303	10.628	11.973	13.333
5.337	6.557	7.787	9.056	10.346	11.653	12.973
6.058	7.388	8.736	10.125	11.537	12.966	14.409
5.318	6.571	7.837	9.147	10.479	11.828	13.189
5.383	6.635	7.9	9.2	10.518	11.852	13.197
6.1	7.487	8.908	10.375	11.87	13.387	14.92
6.684	8.021	9.382	10.789	12.226	13.69	15.176
5.806	7.047	8.296	9.586	10.896	12.226	13.571
5.237	6.413	7.605	8.848	10.118	11.411	12.722
5.698	6.992	8.332	9.731	11.168	12.633	14.119
6.76	8.127	9.521	10.959	12.424	13.912	15.418

5.843	7.113	8.393	9.702	11.025	12.362	13.709
5.934	7.184	8.444	9.75	11.081	12.432	13.8
6.196	7.548	8.926	10.344	11.786	13.249	14.73
5.496	6.749	8.021	9.325	10.646	11.98	13.326
4.642	5.774	6.926	8.112	9.311	10.519	11.733
5.639	6.959	8.306	9.7	11.118	12.558	14.011
6.353	7.644	8.945	10.284	11.642	13.019	14.409
5.356	6.612	7.876	9.176	10.493	11.827	13.173
5.343	6.548	7.769	9.03	10.309	11.605	12.913
5.276	6.482	7.699	8.956	10.231	11.524	12.829
5.591	6.789	8.002	9.26	10.541	11.843	13.16
5.528	6.752	7.984	9.258	10.553	11.866	13.194
6.082	7.459	8.86	10.297	11.756	13.231	14.719
5.78	7.047	8.322	9.627	10.946	12.275	13.613
5.583	6.862	8.168	9.521	10.903	12.308	13.731
5.474	6.744	8.039	9.379	10.747	12.138	13.546
5.623	6.895	8.182	9.509	10.857	12.222	13.6
4.575	5.791	7.015	8.27	9.539	10.821	12.112
5.883	7.144	8.416	9.732	11.071	12.429	13.801
5.346	6.584	7.841	9.138	10.454	11.787	13.132
4.513	5.892	7.3	8.728	10.165	11.608	13.054
6.281	7.598	8.947	10.345	11.772	13.222	14.69
5.574	6.864	8.172	9.516	10.879	12.259	13.652
6.334	7.705	9.109	10.559	12.04	13.546	15.073
4.932	6.239	7.586	8.978	10.394	11.828	13.273
5.704	7.034	8.389	9.794	11.231	12.693	14.176
5.28	6.522	7.772	9.064	10.375	11.706	13.051
5.363	6.654	7.975	9.334	10.716	12.115	13.527
5.989	7.292	8.61	9.964	11.337	12.727	14.13
6.035	7.306	8.593	9.919	11.267	12.635	14.016
5.943	7.197	8.466	9.777	11.113	12.469	13.841
5.708	6.968	8.241	9.556	10.892	12.247	13.617
5.751	7.001	8.263	9.565	10.887	12.229	13.584
5.51	6.872	8.244	9.649	11.069	12.501	13.943
5.835	7.189	8.569	9.997	11.456	12.941	14.447
5.113	6.337	7.575	8.844	10.129	11.428	12.736
6.06	7.277	8.498	9.757	11.032	12.322	13.623
5.742	7.009	8.29	9.612	10.958	12.326	13.712
5.591	6.851	8.13	9.45	10.791	12.149	13.52
6.011	7.289	8.599	9.96	11.352	12.767	14.201
5.223	6.469	7.724	9.02	10.338	11.675	13.027
5.031	6.284	7.55	8.859	10.189	11.537	12.899
5.827	7.101	8.394	9.728	11.086	12.464	13.858
5.492	6.727	7.978	9.266	10.571	11.89	13.22
5.399	6.633	7.889	9.185	10.501	11.833	13.177
5.088	6.318	7.548	8.817	10.103	11.405	12.719
5.702	6.966	8.235	9.541	10.863	12.197	13.541
5.508	6.732	7.974	9.259	10.564	11.887	13.223
4.882	6.121	7.389	8.696	10.023	11.365	12.718
5.86	7.146	8.443	9.778	11.135	12.512	13.903

5.171	6.388	7.621	8.888	10.174	11.476	12.791
5.615	6.846	8.084	9.364	10.664	11.982	13.313
5.773	7.055	8.35	9.683	11.038	12.411	13.799
5.547	6.8	8.063	9.365	10.687	12.027	13.381
5.839	7.073	8.326	9.625	10.947	12.29	13.647
5.729	7.043	8.378	9.756	11.16	12.586	14.028
5.215	6.424	7.645	8.904	10.178	11.467	12.763
5.36	6.661	8.008	9.417	10.86	12.33	13.818
5.54	6.79	8.051	9.359	10.689	12.04	13.407
5.338	6.574	7.826	9.119	10.432	11.762	13.105
4.695	5.918	7.159	8.449	9.763	11.096	12.441
4.367	5.546	6.737	7.953	9.18	10.418	11.663
5.619	6.917	8.264	9.672	11.118	12.594	14.091
5.869	7.202	8.553	9.946	11.361	12.795	14.242
5.96	7.199	8.443	9.72	11.012	12.319	13.636
6.109	7.507	8.938	10.41	11.908	13.427	14.961
5.301	6.527	7.768	9.046	10.343	11.656	12.981
5.145	6.304	7.464	8.658	9.864	11.082	12.308
4.823	6.056	7.314	8.616	9.941	11.283	12.638
5.682	6.945	8.222	9.542	10.885	12.249	13.626
6.283	7.58	8.896	10.253	11.633	13.033	14.448
5.825	7.178	8.557	9.986	11.445	12.931	14.437
6.002	7.298	8.604	9.949	11.312	12.693	14.087
5.373	6.601	7.837	9.108	10.398	11.706	13.028
5.601	6.882	8.174	9.497	10.836	12.189	13.552
5.998	7.321	8.663	10.049	11.459	12.888	14.332
5.361	6.632	7.917	9.239	10.582	11.942	13.316
6.056	7.435	8.843	10.298	11.787	13.303	14.842
5.922	7.301	8.716	10.186	11.689	13.221	14.774
5.308	6.554	7.815	9.11	10.421	11.747	13.084
5.257	6.545	7.852	9.193	10.553	11.929	13.316
6.05	7.361	8.692	10.072	11.48	12.914	14.367
5.711	6.99	8.289	9.628	10.99	12.372	13.769
5.531	6.796	8.072	9.382	10.708	12.047	13.396
5.732	7.001	8.289	9.617	10.967	12.338	13.723
5.306	6.538	7.783	9.061	10.355	11.662	12.978
4.847	6.076	7.323	8.601	9.895	11.2	12.515
5.657	6.924	8.206	9.529	10.872	12.235	13.611
5.419	6.64	7.886	9.184	10.51	11.86	13.227
6.352	7.648	8.955	10.299	11.661	13.042	14.437
5.86	7.146	8.443	9.778	11.135	12.512	13.903
5.642	6.897	8.159	9.458	10.775	12.108	13.452
5.62	6.887	8.176	9.511	10.874	12.259	13.661
5.988	7.312	8.657	10.044	11.456	12.886	14.33
5.489	6.732	7.985	9.279	10.593	11.925	13.272
5.946	7.333	8.752	10.221	11.723	13.252	14.801
5.66	6.909	8.182	9.504	10.855	12.232	13.627
6.436	7.798	9.187	10.619	12.076	13.552	15.041
5.319	6.664	8.056	9.499	10.973	12.47	13.983
4.817	5.988	7.163	8.371	9.592	10.826	12.069

5.37	6.65	7.979	9.378	10.816	12.284	13.769
5.412	6.743	8.118	9.55	11.016	12.508	14.017
5.598	6.841	8.112	9.436	10.792	12.175	13.577
5.517	6.825	8.179	9.596	11.053	12.539	14.045
5.248	6.454	7.672	8.933	10.216	11.52	12.839
5.97	7.246	8.54	9.882	11.249	12.638	14.044
5.763	7.008	8.263	9.561	10.883	12.224	13.582
5.752	7.031	8.327	9.665	11.024	12.401	13.792
5.283	6.523	7.783	9.079	10.394	11.724	13.064
5.225	6.402	7.575	8.775	9.985	11.204	12.431
5.854	7.097	8.352	9.64	10.947	12.274	13.615
5.823	7.076	8.339	9.642	10.962	12.298	13.644
6.074	7.472	8.904	10.378	11.879	13.399	14.935
5.911	7.281	8.681	10.124	11.592	13.078	14.579
5.097	6.283	7.484	8.721	9.975	11.242	12.52
5.085	6.27	7.469	8.704	9.955	11.22	12.495
5.495	6.671	7.862	9.1	10.361	11.644	12.943
5.295	6.494	7.704	8.958	10.234	11.531	12.845
5.013	6.268	7.547	8.862	10.197	11.548	12.911
5.295	6.494	7.704	8.958	10.234	11.531	12.845
5.268	6.508	7.759	9.05	10.363	11.697	13.047
5.677	6.89	8.109	9.379	10.673	11.989	13.32
5.721	6.985	8.262	9.579	10.917	12.272	13.641
5.721	6.985	8.262	9.579	10.917	12.272	13.641
5.274	6.447	7.624	8.835	10.061	11.302	12.553
5.914	7.139	8.376	9.655	10.955	12.275	13.609
5.214	6.487	7.758	9.059	10.373	11.7	13.035
5.537	6.949	8.401	9.894	11.414	12.953	14.507
5.949	7.202	8.467	9.774	11.103	12.451	13.813
5.434	6.653	7.886	9.161	10.457	11.771	13.101
6.328	7.682	9.064	10.494	11.95	13.427	14.918
5.402	6.629	7.875	9.164	10.475	11.805	13.148
4.659	5.873	7.112	8.392	9.695	11.015	12.348
5.69	6.979	8.281	9.626	10.995	12.384	13.789
6.05	7.344	8.658	10.016	11.4	12.803	14.222
5.341	6.608	7.887	9.196	10.52	11.857	13.204
5.714	6.981	8.279	9.633	11.02	12.433	13.866
5.369	6.654	7.959	9.302	10.667	12.051	13.449
5.606	6.878	8.172	9.512	10.876	12.26	13.657
5.833	7.069	8.326	9.636	10.975	12.34	13.724
5.454	6.753	8.074	9.432	10.811	12.206	13.612
5.746	7.004	8.277	9.589	10.924	12.282	13.657
5.622	6.907	8.213	9.556	10.917	12.293	13.68
5.506	6.79	8.09	9.425	10.776	12.143	13.52
5.339	6.581	7.842	9.141	10.458	11.789	13.13
5.722	6.976	8.248	9.563	10.903	12.263	13.639
5.393	6.588	7.802	9.063	10.35	11.66	12.988
5.559	6.816	8.087	9.399	10.732	12.083	13.448
5.456	6.748	8.052	9.39	10.745	12.113	13.49
5.668	6.982	8.32	9.703	11.112	12.543	13.99

5.811	7.064	8.326	9.627	10.946	12.28	13.625
5.276	6.564	7.862	9.186	10.523	11.872	13.228
5.838	7.212	8.618	10.064	11.534	13.023	14.523
6.231	7.602	9	10.436	11.896	13.374	14.868
5.131	6.418	7.726	9.071	10.435	11.815	13.207
5.297	6.642	8.035	9.479	10.956	12.456	13.971
5.258	6.476	7.7	8.967	10.254	11.557	12.871
5.453	6.711	7.985	9.298	10.63	11.977	13.334
5.524	6.711	7.907	9.142	10.395	11.664	12.944
5.493	6.75	8.026	9.347	10.693	12.063	13.451
5.291	6.557	7.85	9.188	10.553	11.94	13.343
5.431	6.726	8.057	9.441	10.858	12.301	13.762
5.704	6.925	8.154	9.429	10.727	12.046	13.379
5.674	6.936	8.21	9.529	10.871	12.233	13.61
5.433	6.676	7.926	9.212	10.515	11.833	13.163
5.751	6.984	8.229	9.515	10.822	12.147	13.485
4.41	5.572	6.773	8.015	9.281	10.564	11.857
5.905	7.139	8.395	9.693	11.013	12.353	13.706
6.378	7.723	9.095	10.513	11.959	13.427	14.91
5.902	7.184	8.498	9.861	11.253	12.671	14.107
6.775	8.145	9.545	10.996	12.476	13.98	15.504
5.926	7.254	8.606	10.008	11.436	12.887	14.354
5.654	6.906	8.178	9.492	10.831	12.192	13.569
5.618	6.873	8.145	9.46	10.796	12.149	13.514
5.401	6.655	7.92	9.223	10.544	11.879	13.226
5.864	7.109	8.363	9.658	10.974	12.308	13.654
5.664	6.917	8.182	9.483	10.804	12.144	13.497
5.496	6.753	8.027	9.338	10.671	12.024	13.392

ESpm10r	ESpm11r	ESpm12r	ESpm13r	ESpm14r	ESpm15r	BEHm1
14.565	15.967	17.377	18.794	20.216	21.642	3.949
14.337	15.656	16.984	18.321	19.664	21.013	3.888
15.094	16.487	17.888	19.296	20.71	22.13	3.974
13.219	14.469	15.725	16.985	18.249	19.515	3.816
14.311	15.691	17.079	18.472	19.87	21.272	3.964
16.037	17.616	19.2	20.786	22.375	23.966	4.569
15.475	16.93	18.393	19.864	21.341	22.823	3.884
13.92	15.289	16.664	18.041	19.421	20.803	3.848
14.909	16.303	17.705	19.113	20.526	21.944	4.072
13.742	15.032	16.331	17.635	18.944	20.258	3.84
15.505	16.886	18.274	19.666	21.063	22.463	3.847
15.113	16.51	17.916	19.329	20.748	22.172	10.676
14.641	16.023	17.414	18.812	20.215	21.623	3.979
15.097	16.516	17.943	19.378	20.819	22.265	4.011
15.646	17.108	18.579	20.056	21.54	23.028	4.111
15.622	17.089	18.563	20.043	21.529	23.019	4.111
14.428	15.826	17.231	18.641	20.055	21.472	3.88
15.605	17.003	18.406	19.814	21.226	22.642	3.932
13.698	14.941	16.19	17.443	18.701	19.963	3.837
14.677	16.042	17.412	18.787	20.165	21.545	3.857
15.213	16.646	18.088	19.537	20.993	22.454	3.987
14.605	15.974	17.347	18.723	20.103	21.484	4.172
14.733	16.092	17.456	18.825	20.197	21.573	4.078
15.541	17.011	18.489	19.974	21.465	22.961	4.11
14.344	15.649	16.96	18.276	19.598	20.923	3.87
16.802	18.387	19.978	21.573	23.172	24.773	4.013
13.981	15.239	16.502	17.767	19.036	20.307	3.836
15.695	17.092	18.496	19.904	21.316	22.731	4.05
14.792	16.191	17.599	19.014	20.436	21.862	4.105
13.652	14.878	16.11	17.347	18.589	19.835	3.831
15.714	17.136	18.565	19.997	21.434	22.873	4.633
16.467	17.965	19.473	20.989	22.513	24.042	6.843
14.95	16.34	17.735	19.132	20.532	21.933	4.062
14.505	15.863	17.227	18.595	19.966	21.339	3.876
16.99	18.569	20.157	21.752	23.353	24.96	4.125
15.272	16.699	18.135	19.579	21.029	22.484	3.852
16.18	17.742	19.313	20.892	22.476	24.065	4.018
14.761	16.179	17.605	19.036	20.472	21.911	3.969
15.832	17.304	18.785	20.274	21.769	23.269	3.92
15.79	17.26	18.737	20.22	21.707	23.199	4.082
15.27	16.661	18.06	19.466	20.876	22.29	4.103
14.654	16.044	17.442	18.846	20.254	21.667	3.975
16.974	18.524	20.082	21.645	23.213	24.786	4.035
14.845	16.236	17.634	19.037	20.445	21.857	4.184
14.946	16.332	17.729	19.133	20.544	21.961	4.166
15.208	16.63	18.06	19.495	20.936	22.38	4.106
15.265	16.683	18.109	19.542	20.98	22.423	4.068
15.359	16.77	18.189	19.615	21.047	22.485	4.109
15.2	16.612	18.033	19.464	20.901	22.345	4.296

15.625	17.053	18.487	19.924	21.366	22.811	4.208
15.32	16.736	18.16	19.591	21.029	22.471	4.208
15.714	17.118	18.528	19.945	21.368	22.795	4.111
15.319	16.735	18.16	19.592	21.029	22.471	4.208
15.766	17.272	18.787	20.31	21.839	23.373	4.2
15.288	16.706	18.133	19.565	21.004	22.447	4.074
15.732	17.163	18.601	20.044	21.493	22.946	4.609
15.728	17.155	18.589	20.027	21.47	22.916	4.208
15.181	16.598	18.022	19.453	20.888	22.327	4.207
15.503	16.909	18.323	19.743	21.17	22.602	4.208
15.302	16.721	18.148	19.582	21.021	22.465	4.072
15.47	16.885	18.305	19.728	21.155	22.584	4.62
15.23	16.653	18.084	19.52	20.961	22.406	4.068
15.184	16.599	18.024	19.458	20.898	22.345	4.2
15.143	16.562	17.99	19.427	20.87	22.319	4.2
15.23	16.653	18.084	19.52	20.961	22.406	4.067
14.436	15.796	17.166	18.544	19.929	21.319	4.188
14.549	15.895	17.253	18.619	19.993	21.374	3.968
15.391	16.844	18.3	19.76	21.222	22.685	4.687
14.157	15.495	16.841	18.195	19.554	20.919	3.958
14.571	15.945	17.328	18.719	20.117	21.52	4.15
14.735	16.165	17.602	19.045	20.492	21.942	4.631
14.647	16.031	17.424	18.826	20.234	21.647	4.161
15.764	17.249	18.741	20.238	21.738	23.242	4.628
14.72	16.111	17.509	18.912	20.321	21.734	3.994
13.572	14.864	16.16	17.461	18.765	20.071	3.947
14.506	15.832	17.165	18.503	19.847	21.195	3.894
15.062	16.455	17.854	19.257	20.664	22.074	4.02
14.905	16.302	17.707	19.118	20.534	21.954	3.983
15.175	16.574	17.979	19.39	20.807	22.228	4.001
15.027	16.405	17.789	19.177	20.57	21.966	3.932
14.735	16.166	17.604	19.046	20.491	21.94	3.929
15.127	16.542	17.968	19.402	20.843	22.29	4.125
13.851	15.178	16.509	17.846	19.187	20.53	4.03
14.727	16.087	17.455	18.829	20.209	21.594	4.094
14.869	16.347	17.827	19.309	20.792	22.276	4.414
14.924	16.364	17.812	19.265	20.724	22.186	4.654
14.528	15.907	17.294	18.686	20.083	21.484	3.957
16.802	18.387	19.978	21.573	23.172	24.773	4.013
14.704	16.084	17.473	18.868	20.268	21.672	4.011
14.303	15.641	16.984	18.332	19.683	21.036	4.038
15.863	17.325	18.794	20.268	21.748	23.231	4.124
14.561	15.94	17.326	18.717	20.112	21.511	3.979
14.552	15.914	17.281	18.652	20.028	21.406	3.95
16.465	18.02	19.581	21.149	22.72	24.295	3.985
16.68	18.198	19.73	21.271	22.821	24.378	3.995
14.929	16.297	17.674	19.06	20.451	21.849	4.09
14.047	15.383	16.728	18.08	19.437	20.799	3.885
15.619	17.131	18.651	20.176	21.707	23.241	3.936
16.939	18.473	20.016	21.568	23.127	24.692	4.025

15.064	16.425	17.793	19.166	20.543	21.924	4.604
15.18	16.57	17.968	19.371	20.78	22.192	4.004
16.224	17.729	19.245	20.768	22.298	23.834	4.04
14.68	16.04	17.406	18.776	20.149	21.526	3.95
12.95	14.169	15.39	16.612	17.835	19.059	3.59
15.476	16.949	18.429	19.914	21.403	22.896	4.116
15.811	17.222	18.642	20.069	21.502	22.941	4.042
14.531	15.897	17.271	18.651	20.037	21.427	4.599
14.229	15.552	16.88	18.212	19.548	20.887	3.922
14.143	15.465	16.793	18.127	19.465	20.806	3.882
14.49	15.831	17.181	18.538	19.902	21.271	3.863
14.533	15.881	17.237	18.599	19.967	21.339	4.268
16.217	17.722	19.233	20.748	22.268	23.791	3.963
14.956	16.305	17.657	19.013	20.372	21.733	3.888
15.17	16.62	18.08	19.548	21.021	22.5	3.94
14.969	16.402	17.844	19.293	20.748	22.206	3.985
14.989	16.387	17.791	19.202	20.617	22.037	4.036
13.41	14.715	16.024	17.336	18.652	19.971	3.712
15.184	16.576	17.975	19.379	20.789	22.203	4.139
14.485	15.845	17.209	18.578	19.95	21.324	3.938
14.502	15.951	17.4	18.85	20.3	21.749	4.375
16.17	17.659	19.155	20.656	22.162	23.67	4.233
15.054	16.464	17.88	19.303	20.73	22.161	4.628
16.616	18.171	19.736	21.309	22.888	24.472	4.017
14.725	16.183	17.645	19.108	20.574	22.04	3.74
15.675	17.185	18.705	20.232	21.764	23.301	4.027
14.408	15.775	17.151	18.534	19.923	21.316	3.965
14.949	16.378	17.813	19.252	20.694	22.14	3.851
15.544	16.966	18.396	19.833	21.275	22.721	3.829
15.41	16.813	18.224	19.642	21.065	22.492	4.631
15.226	16.621	18.026	19.437	20.855	22.278	4.63
14.997	16.386	17.782	19.184	20.589	21.998	4.074
14.951	16.327	17.71	19.1	20.494	21.893	3.952
15.391	16.844	18.3	19.76	21.222	22.685	4.683
15.969	17.503	19.047	20.599	22.157	23.719	4.065
14.054	15.377	16.707	18.041	19.379	20.721	3.861
14.932	16.248	17.57	18.896	20.225	21.558	3.946
15.112	16.525	17.947	19.378	20.816	22.26	4.127
14.9	16.288	17.683	19.082	20.485	21.891	3.965
15.649	17.108	18.576	20.051	21.533	23.019	3.992
14.391	15.764	17.146	18.534	19.927	21.324	3.838
14.271	15.651	17.037	18.428	19.823	21.222	3.815
15.265	16.683	18.108	19.541	20.98	22.424	3.985
14.556	15.899	17.245	18.595	19.948	21.304	3.999
14.529	15.887	17.251	18.618	19.989	21.362	3.914
14.043	15.376	16.714	18.059	19.408	20.761	3.969
14.891	16.246	17.604	18.966	20.329	21.695	4.329
14.572	15.929	17.295	18.669	20.048	21.432	3.971
14.079	15.446	16.817	18.192	19.57	20.95	3.744
15.307	16.722	18.145	19.575	21.012	22.454	4.024

14.116	15.45	16.79	18.136	19.487	20.843	3.866
14.655	16.005	17.362	18.725	20.092	21.463	3.911
15.199	16.609	18.027	19.451	20.882	22.317	3.998
14.745	16.119	17.5	18.888	20.281	21.678	4.037
15.014	16.39	17.772	19.159	20.55	21.945	4.051
15.484	16.952	18.429	19.913	21.404	22.9	4.652
14.066	15.374	16.686	18	19.317	20.636	3.877
15.317	16.825	18.338	19.855	21.375	22.897	3.89
14.785	16.173	17.569	18.971	20.379	21.791	3.974
14.456	15.814	17.177	18.544	19.913	21.284	3.874
13.795	15.155	16.519	17.887	19.256	20.627	3.968
12.914	14.17	15.429	16.692	17.957	19.225	3.608
15.603	17.125	18.656	20.192	21.732	23.274	3.945
15.7	17.167	18.641	20.12	21.604	23.092	4.112
14.962	16.296	17.635	18.98	20.328	21.681	4.01
16.507	18.062	19.623	21.19	22.76	24.334	3.999
14.317	15.662	17.015	18.374	19.738	21.108	3.981
13.541	14.78	16.023	17.271	18.523	19.778	3.832
14.001	15.371	16.746	18.125	19.506	20.889	3.962
15.015	16.413	17.818	19.228	20.644	22.063	4.078
15.874	17.31	18.753	20.203	21.659	23.118	4.072
15.959	17.494	19.038	20.59	22.148	23.711	4.069
15.491	16.903	18.323	19.749	21.18	22.616	4.026
14.362	15.707	17.06	18.42	19.787	21.159	3.905
14.923	16.3	17.682	19.068	20.458	21.85	3.918
15.787	17.251	18.722	20.199	21.68	23.166	4.119
14.702	16.097	17.5	18.909	20.324	21.743	3.948
16.398	17.969	19.551	21.141	22.738	24.34	4.081
16.342	17.923	19.513	21.11	22.712	24.318	4.07
14.429	15.78	17.137	18.498	19.863	21.231	4.023
14.713	16.118	17.528	18.944	20.364	21.788	3.88
15.836	17.319	18.811	20.313	21.822	23.336	6.842
15.179	16.599	18.026	19.461	20.901	22.346	3.986
14.754	16.117	17.486	18.86	20.237	21.618	4.598
15.122	16.531	17.948	19.373	20.805	22.242	3.975
14.302	15.631	16.964	18.301	19.64	20.982	3.931
13.836	15.161	16.491	17.824	19.16	20.498	3.852
14.998	16.394	17.797	19.206	20.621	22.04	3.982
14.606	15.995	17.392	18.794	20.201	21.612	3.978
15.844	17.261	18.687	20.12	21.559	23.004	3.985
15.307	16.722	18.145	19.575	21.012	22.454	4.024
14.805	16.166	17.532	18.903	20.278	21.656	4.33
15.077	16.504	17.94	19.383	20.832	22.286	4.121
15.785	17.249	18.719	20.196	21.677	23.163	4.115
14.629	15.996	17.371	18.753	20.14	21.532	4.007
16.367	17.945	19.532	21.127	22.728	24.333	4.078
15.038	16.462	17.895	19.337	20.785	22.238	3.939
16.541	18.048	19.561	21.079	22.6	24.125	4.005
15.506	17.037	18.572	20.111	21.652	23.194	3.827
13.319	14.575	15.836	17.1	18.369	19.64	3.827

15.267	16.772	18.283	19.798	21.314	22.833	3.895
15.538	17.068	18.603	20.142	21.684	23.228	3.904
14.995	16.424	17.863	19.309	20.761	22.218	3.934
15.564	17.093	18.629	20.169	21.712	23.257	3.958
14.171	15.514	16.866	18.224	19.589	20.959	4.042
15.461	16.889	18.325	19.767	21.215	22.667	4.639
14.953	16.334	17.724	19.123	20.528	21.938	3.969
15.195	16.607	18.027	19.453	20.885	22.323	4.623
14.412	15.766	17.124	18.486	19.851	21.219	3.991
13.664	14.903	16.147	17.395	18.648	19.904	3.832
14.971	16.338	17.715	19.101	20.495	21.896	3.897
15	16.362	17.73	19.102	20.479	21.859	4.04
16.482	18.037	19.599	21.165	22.736	24.309	3.994
16.089	17.606	19.129	20.655	22.185	23.717	3.967
13.805	15.097	16.394	17.697	19.003	20.313	3.839
13.778	15.067	16.361	17.66	18.963	20.269	3.837
14.255	15.578	16.909	18.247	19.591	20.941	3.874
14.173	15.513	16.862	18.218	19.581	20.948	4.008
14.282	15.659	17.042	18.428	19.818	21.211	3.884
14.173	15.513	16.862	18.218	19.581	20.948	4.008
14.41	15.784	17.166	18.556	19.951	21.351	3.986
14.664	16.018	17.38	18.748	20.122	21.501	4.122
15.02	16.407	17.801	19.2	20.603	22.01	4.033
15.02	16.407	17.801	19.2	20.603	22.01	4.033
13.814	15.082	16.357	17.636	18.921	20.209	3.953
14.954	16.308	17.669	19.036	20.408	21.785	3.92
14.376	15.723	17.075	18.429	19.785	21.144	4.307
16.072	17.645	19.224	20.808	22.395	23.985	4.57
15.186	16.569	17.958	19.353	20.753	22.157	4.056
14.443	15.795	17.155	18.523	19.896	21.275	4.072
16.419	17.929	19.445	20.966	22.491	24.02	4
14.5	15.86	17.224	18.592	19.963	21.337	3.876
13.69	15.038	16.39	17.745	19.102	20.461	3.83
15.207	16.635	18.07	19.513	20.96	22.411	3.944
15.651	17.09	18.536	19.987	21.442	22.902	4.641
14.56	15.922	17.289	18.661	20.037	21.416	4.598
15.316	16.779	18.252	19.734	21.221	22.714	4.197
14.859	16.278	17.704	19.136	20.574	22.015	4.632
15.065	16.482	17.905	19.334	20.767	22.204	4.633
15.125	16.538	17.962	19.395	20.835	22.281	4.285
15.028	16.45	17.878	19.311	20.747	22.187	4.633
15.048	16.452	17.866	19.29	20.721	22.159	4.27
15.074	16.474	17.879	19.288	20.7	22.116	4.62
14.906	16.297	17.694	19.095	20.498	21.905	4.608
14.478	15.831	17.189	18.55	19.914	21.28	4.211
15.027	16.426	17.833	19.248	20.668	22.094	4.622
14.33	15.684	17.048	18.42	19.798	21.181	3.96
14.825	16.21	17.604	19.003	20.409	21.818	4.014
14.874	16.263	17.656	19.052	20.451	21.851	4.36
15.45	16.92	18.4	19.886	21.379	22.876	4.68

14.979	16.34	17.707	19.078	20.454	21.833	4.04
14.592	15.96	17.333	18.709	20.088	21.469	3.856
16.033	17.549	19.069	20.593	22.12	23.648	3.958
16.373	17.887	19.41	20.938	22.472	24.01	4.043
14.609	16.018	17.434	18.856	20.282	21.711	3.941
15.496	17.028	18.565	20.104	21.646	23.189	3.93
14.194	15.523	16.856	18.192	19.531	20.873	4.225
14.699	16.069	17.444	18.822	20.204	21.588	4.176
14.232	15.527	16.827	18.13	19.438	20.747	3.848
14.853	16.267	17.69	19.12	20.556	21.997	4.63
14.76	16.186	17.62	19.06	20.505	21.953	4.63
15.237	16.722	18.215	19.712	21.214	22.718	3.898
14.725	16.08	17.442	18.811	20.185	21.564	3.947
14.998	16.395	17.799	19.209	20.624	22.042	4.087
14.502	15.849	17.202	18.56	19.921	21.287	3.923
14.834	16.191	17.556	18.925	20.3	21.678	3.868
13.157	14.462	15.77	17.079	18.39	19.702	3.611
15.07	16.442	17.821	19.206	20.595	21.987	3.983
16.406	17.911	19.424	20.942	22.464	23.991	3.997
15.559	17.024	18.499	19.982	21.472	22.967	3.92
17.042	18.592	20.151	21.719	23.293	24.873	4.158
15.833	17.322	18.819	20.322	21.83	23.343	4.17
14.96	16.361	17.772	19.189	20.612	22.04	3.957
14.889	16.27	17.656	19.047	20.442	21.84	4.096
14.58	15.941	17.307	18.678	20.052	21.429	3.836
15.011	16.376	17.748	19.125	20.506	21.891	4.301
14.862	16.238	17.622	19.014	20.412	21.817	4.11
14.773	16.164	17.563	18.969	20.381	21.798	4.009

BEHm2	BEHm3	BEHm4	BEHm5	BEHm6	BEHm7	BEHm8
3.405	3.263	3.031	2.825	2.529	2.252	1.803
3.527	3.472	3.255	3.211	2.978	2.755	2.519
3.852	3.568	3.319	3.295	2.894	2.746	2.617
3.337	2.799	2.617	2.147	1.865	1.491	0.94
3.412	3.254	2.921	2.668	2.341	2.134	1.856
4.094	3.316	2.721	2.253	1.885	1.885	1.727
3.737	3.638	3.391	3.229	3.05	2.906	2.766
3.137	2.863	2.201	2.105	1.162	0.718	0.397
3.772	3.766	3.295	3.11	2.78	2.617	2.519
3.52	3.273	3.24	2.81	2.651	2.467	2.331
3.719	3.669	3.526	3.215	3.162	3.13	3.066
10.67	4.081	3.719	3.516	3.417	3.231	3.088
3.689	3.466	3.179	3.098	2.712	2.651	2.515
3.775	3.453	3.329	3.235	3.183	2.996	2.873
3.835	3.488	3.279	3.242	2.802	2.742	2.617
3.815	3.479	3.261	3.242	2.745	2.728	2.617
3.613	2.874	2.795	2.617	2.546	1.981	1.717
3.854	3.698	3.529	3.333	3.297	2.958	2.913
3.525	3.355	3.225	2.813	2.617	2.489	2.331
3.779	3.409	3.076	2.794	2.75	2.74	2.617
3.648	3.413	3.167	2.963	2.843	2.672	2.459
3.923	3.448	3.275	3.061	2.564	2.424	2.142
3.879	3.537	3.282	2.994	2.744	2.617	2.598
3.797	3.47	3.243	3.214	2.743	2.67	2.617
3.731	3.668	3.446	3.271	3.025	2.703	2.617
3.795	3.453	3.213	3.18	3.011	2.835	2.812
3.686	3.523	3.288	3.17	2.811	2.617	2.541
3.925	3.804	3.755	3.401	3.387	3.127	3.085
3.714	3.484	3.275	3.042	2.743	2.617	2.526
3.523	3.459	3.274	3.166	2.9	2.617	2.582
3.922	3.788	3.757	3.61	3.259	3.238	2.984
4.091	3.953	3.704	3.591	3.451	3.261	3.201
3.57	3.383	3.101	2.957	2.565	2.388	2.331
3.691	3.323	3.184	2.976	2.855	2.779	2.584
3.753	3.706	3.556	3.39	3.154	2.965	2.953
3.795	3.569	3.459	3.227	2.981	2.894	2.747
3.687	3.592	3.288	3.07	2.917	2.812	2.575
3.411	3.068	2.689	2.547	2.426	1.978	1.626
3.756	3.572	3.411	3.199	3.096	3.066	2.901
3.865	3.561	3.245	3.03	2.935	2.797	2.765
3.958	3.738	3.548	3.416	3.165	3.094	2.881
3.68	3.306	3.134	2.717	2.613	2.5	2.28
3.818	3.656	3.513	3.395	3.246	3.212	3.12
3.768	3.509	3.2	2.842	2.795	2.605	2.363
3.821	3.594	3.465	3.216	3.064	2.982	2.901
3.817	3.61	3.392	3.281	3.109	2.729	2.617
3.834	3.484	3.369	3.263	2.869	2.799	2.617
3.963	3.812	3.502	3.383	3.293	3.194	2.72
4.064	3.797	3.573	3.377	3.317	3.148	2.722

4.072	3.744	3.548	3.35	3.312	2.966	2.93
4.067	3.577	3.45	3.361	3.282	3.067	2.801
3.963	3.861	3.741	3.515	3.426	3.336	3.257
4.066	3.564	3.455	3.33	3.292	2.959	2.865
4.08	3.545	3.393	3.312	3.203	2.853	2.84
3.834	3.525	3.394	3.29	3.169	2.8	2.64
4.076	3.853	3.744	3.455	3.346	3.235	3.12
4.073	3.804	3.639	3.484	3.351	3.289	2.94
4.05	3.548	3.359	3.309	2.957	2.687	2.632
4.093	3.908	3.568	3.479	3.335	3.291	2.966
3.852	3.509	3.401	3.319	3.261	2.959	2.853
4.009	3.722	3.55	3.152	2.818	2.617	2.617
3.813	3.475	3.366	3.248	2.869	2.727	2.617
4.072	3.804	3.46	3.343	3.197	2.936	2.633
4.065	3.513	3.389	3.324	3.187	2.865	2.633
3.76	3.468	3.364	3.251	2.869	2.799	2.603
3.693	3.617	3.377	3.142	2.943	2.838	2.747
3.782	3.624	3.436	3.312	3.093	2.816	2.747
4.577	3.754	3.202	2.969	2.46	2.077	2.001
3.761	3.45	3.283	2.918	2.617	2.584	2.533
3.79	3.475	3.425	3.168	2.922	2.744	2.535
3.845	3.433	3.132	3.03	2.617	2.403	2.071
3.791	3.493	3.425	3.218	2.917	2.71	2.535
3.972	3.769	3.363	3.139	2.803	2.514	2.408
3.846	3.368	3.087	2.817	2.617	2.527	2.315
3.672	3.52	3.053	2.851	2.66	2.42	2.183
3.808	3.588	3.183	3.112	2.921	2.678	2.653
3.852	3.463	3.311	3.201	3.053	2.776	2.647
3.589	3.228	3.095	2.798	2.658	2.39	2.202
3.675	3.555	3.43	2.998	2.838	2.747	2.626
3.784	3.704	3.447	3.307	3.112	2.921	2.751
3.585	3.24	2.779	2.617	2.331	2.318	1.735
3.911	3.485	3.401	3.214	2.896	2.837	2.638
3.622	3.499	3.02	2.776	2.235	2.172	2.127
3.852	3.603	3.318	3.142	2.852	2.763	2.747
3.657	3.599	3.216	2.798	2.628	2.349	1.677
3.724	3.613	2.741	2.628	2.617	2.617	1.893
3.689	3.398	3.101	3.05	2.704	2.651	2.515
3.795	3.453	3.213	3.18	3.011	2.835	2.812
3.768	3.548	3.136	3.116	2.666	2.617	2.517
3.806	3.499	3.3	3.021	2.88	2.646	2.354
4.064	3.78	3.499	3.426	3.243	3.039	3.019
3.598	3.254	2.916	2.778	2.358	2.202	2.146
3.702	3.164	2.919	2.737	2.505	2.432	1.773
3.767	3.588	3.269	3.136	2.955	2.878	2.68
3.797	3.765	3.688	3.612	3.572	3.293	3.137
3.781	3.519	3.476	3.328	3.203	2.97	2.82
3.682	3.439	3.099	2.756	2.617	2.617	2.502
3.673	3.595	3.345	3.109	2.928	2.674	2.617
3.836	3.795	3.793	3.567	3.496	3.455	3.355

4.591	3.808	3.773	3.45	3.078	2.863	2.617
3.91	3.683	3.492	3.336	3.132	2.983	2.803
3.84	3.533	3.364	3.219	3.119	2.935	2.82
3.664	3.396	3.272	2.899	2.869	2.744	2.552
3.46	3.098	2.83	2.818	2.504	2.123	1.857
3.625	3.314	3.11	3.032	3.013	2.607	2.466
3.826	3.772	3.648	3.366	3.209	3.108	3.076
4.22	3.783	3.328	3.224	2.694	2.327	2.234
3.671	3.441	3.108	2.689	2.617	2.617	2.345
3.724	3.374	2.878	2.723	2.617	2.522	2.432
3.786	3.608	3.388	3.112	2.84	2.747	2.617
3.896	3.698	3.299	3.273	2.67	2.617	2.617
3.741	3.533	3.315	3.131	3.02	2.921	2.765
3.633	3.451	3.401	3.249	2.92	2.835	2.708
3.757	3.752	3.244	2.768	2.623	2.532	2.231
3.746	3.282	2.894	2.741	2.617	2.411	2.237
3.697	3.362	3.279	2.908	2.805	2.648	2.438
3.001	2.774	1.926	1.575	1.249	0.347	0
3.707	3.676	3.385	3.35	3.159	2.987	2.855
3.674	3.364	2.821	2.725	2.617	2.595	2.265
2.84	1.885	1.725	1.66	0.419	0	0
3.99	3.746	3.598	3.518	3.383	3.223	3.112
3.879	3.777	3.434	3.047	2.772	2.469	2.181
3.779	3.579	3.465	3.359	3.217	3.193	3.01
3.29	3.029	2.747	2.526	1.987	1.694	1.565
3.591	3.399	3.069	2.981	2.86	2.609	2.526
3.486	3.337	2.93	2.73	2.659	2.341	2.008
3.637	3.211	3.017	2.808	2.659	2.409	2.331
3.711	3.629	3.211	3.151	3.042	3.019	2.937
3.841	3.704	3.681	3.347	3.261	3.017	2.891
3.961	3.708	3.651	3.45	3.238	3.201	2.918
3.793	3.387	3.22	3.012	2.946	2.867	2.641
3.831	3.548	3.435	3.207	3.178	2.931	2.714
4.573	3.756	3.212	2.952	2.462	2.084	2.01
3.596	3.428	3.113	2.947	2.798	2.693	2.375
3.461	3.268	2.764	2.617	2.526	2.331	1.982
3.857	3.797	3.614	3.578	3.27	3.013	2.911
3.91	3.487	3.396	3.219	2.89	2.837	2.635
3.785	3.41	3.172	2.797	2.723	2.617	2.475
3.884	3.733	3.541	3.419	3.238	2.921	2.806
3.579	3.443	3.008	2.538	2.208	2.005	1.409
3.514	3.07	2.591	2.238	2.13	1.562	1.331
3.752	3.471	3.283	3.094	3.031	2.867	2.777
3.762	3.548	3.239	3.115	2.725	2.617	2.575
3.729	3.391	3.054	2.727	2.617	2.523	2.435
3.696	3.017	2.889	2.815	2.467	2.144	1.831
3.898	3.773	3.406	3.1	2.864	2.769	2.74
3.753	3.699	3.197	3.016	2.621	2.617	2.617
3.37	2.952	2.671	2.445	2.018	1.838	1.447
3.652	3.477	3.375	3.043	2.818	2.757	2.747

3.542	3.181	2.976	2.899	2.765	2.387	2.159
3.651	3.479	3.356	2.914	2.765	2.747	2.717
3.656	3.474	3.206	3.023	2.901	2.814	2.65
3.88	3.567	3.24	3.177	3.058	2.575	2.537
3.949	3.733	3.545	3.39	3.213	2.941	2.884
4.214	3.679	3.489	2.935	2.77	2.64	2.554
3.668	3.599	3.461	3.07	2.736	2.617	2.461
3.569	3.318	3.023	2.747	2.656	2.617	2.501
3.683	3.522	2.979	2.919	2.834	2.53	2.489
3.669	3.189	2.976	2.905	2.779	2.568	2.242
3.458	3.051	2.356	2.026	1.543	0.7	0.37
3.189	2.67	2.331	2.137	1.725	1.019	0.198
3.682	3.511	3.359	2.977	2.761	2.617	2.617
3.87	3.436	3.243	3.104	3.076	2.857	2.743
3.855	3.601	3.442	3.314	3.189	2.958	2.941
3.786	3.428	3.267	3.168	2.979	2.836	2.72
3.555	3.417	3.2	2.899	2.895	2.844	2.511
3.523	3.309	3.205	2.864	2.617	2.576	2.362
3.348	3.108	2.59	2.324	1.625	1.592	1.331
3.797	3.775	3.301	3.114	2.793	2.736	2.529
4.004	3.852	3.794	3.625	3.532	3.297	3.218
3.607	3.417	3.127	2.943	2.755	2.691	2.375
3.766	3.615	3.508	3.166	3.039	2.948	2.793
3.44	3.361	3.226	2.973	2.707	2.589	2.331
3.584	3.41	3.294	2.943	2.677	2.652	2.423
4.021	3.49	3.387	3.243	3.144	3.073	2.744
3.517	3.199	3.018	2.682	2.586	2.401	1.945
3.71	3.594	3.408	3.123	2.935	2.886	2.675
3.599	3.475	3.228	3.11	2.839	2.793	2.613
3.554	3.243	2.93	2.801	2.607	2.386	2.183
3.493	3.336	2.824	2.539	2.461	2.331	2.331
4.137	3.756	3.674	3.329	3.269	3.072	2.994
3.75	3.421	3.328	3.137	2.924	2.82	2.551
3.906	3.741	3.365	2.854	2.617	2.533	2.501
3.769	3.437	3.335	3.201	2.994	2.828	2.723
3.698	3.347	2.956	2.763	2.31	2.089	1.815
3.301	2.775	2.717	2.36	1.985	1.612	1.513
3.634	3.433	3.204	2.985	2.747	2.743	2.618
3.689	3.415	3.106	3.088	2.665	2.651	2.515
3.865	3.773	3.534	3.467	3.321	3.193	3.124
3.652	3.477	3.375	3.043	2.818	2.757	2.747
3.917	3.801	3.236	2.992	2.964	2.836	2.645
3.824	3.511	3.217	2.913	2.762	2.551	2.434
3.951	3.648	3.424	3.243	3.039	2.787	2.743
3.769	3.544	3.177	3.058	2.617	2.574	2.514
3.622	3.429	3.128	3.034	2.913	2.706	2.614
3.746	3.604	3.293	2.905	2.845	2.623	2.617
3.84	3.702	3.572	3.416	3.31	3.179	3.144
3.377	3.139	2.94	2.864	2.722	2.233	1.878
3.359	3.14	2.683	2.617	2.135	1.929	1.593

3.598	3.45	2.945	2.747	2.747	2.617	2.555
3.593	3.161	2.94	2.783	2.617	2.527	1.811
3.73	3.594	3.289	2.853	2.835	2.617	2.617
3.682	3.524	2.893	2.694	2.617	2.617	2.502
3.533	3.306	3.193	2.87	2.514	2.331	2.239
4.106	3.692	3.591	3.369	3.209	3.115	2.948
3.682	3.652	3.325	3.17	2.919	2.747	2.663
3.853	3.726	3.514	3.155	2.761	2.617	2.597
3.599	3.473	3.15	2.754	2.529	2.331	2.325
3.524	3.353	3.228	2.82	2.617	2.488	2.331
3.591	3.554	3.406	3.299	3.096	2.958	2.838
3.847	3.612	3.403	3.071	2.991	2.835	2.749
3.78	3.435	3.197	3.17	2.956	2.821	2.726
3.729	3.36	3.154	3.114	2.942	2.793	2.641
3.533	3.278	2.906	2.899	2.617	2.554	2.175
3.528	3.258	2.902	2.899	2.617	2.544	2.157
3.769	3.5	3.289	3.239	2.863	2.825	2.617
3.533	3.337	3.225	2.932	2.582	2.489	2.331
3.337	3.099	2.73	2.423	2.331	2.331	1.775
3.533	3.337	3.225	2.932	2.582	2.489	2.331
3.774	3.246	3.108	2.853	2.617	2.489	2.157
3.694	3.649	3.382	3.266	3.16	2.938	2.747
3.808	3.423	3.235	3.063	2.865	2.755	2.721
3.808	3.423	3.235	3.063	2.865	2.755	2.721
3.733	3.651	3.286	3.198	2.874	2.548	2.405
3.856	3.548	3.46	3.356	3.176	3.112	2.878
3.548	3.23	2.833	2.53	2.166	1.778	1.581
4.094	3.751	2.911	2.566	2.407	1.891	1.885
3.873	3.622	3.405	3.262	3.017	2.968	2.779
3.787	3.436	3.268	2.898	2.608	2.535	2.331
3.818	3.666	3.533	3.349	3.223	3.211	3.123
3.688	3.242	3.174	2.976	2.78	2.72	2.53
3.149	2.765	2.432	1.774	1.675	0.814	0.738
3.818	3.439	3.187	2.923	2.765	2.741	2.617
4.006	3.733	3.521	3.316	3.181	3.116	2.903
3.811	3.448	3.123	2.697	2.546	2.331	2.244
3.876	3.651	3.393	3.22	2.962	2.798	2.747
3.783	3.659	2.741	2.661	2.617	2.212	1.903
3.92	3.673	3.574	2.697	2.68	2.617	2.617
3.924	3.73	3.605	3.53	2.908	2.853	2.617
3.824	3.661	2.749	2.674	2.649	2.617	2.097
4.008	3.627	3.455	3.236	2.864	2.623	2.617
3.787	3.676	3.228	3.015	2.911	2.77	2.452
4.047	3.434	3.252	2.885	2.768	2.588	2.331
3.835	3.406	2.986	2.652	2.633	2.617	2.284
3.828	3.733	3.431	3.168	3.139	2.949	2.677
3.572	3.416	3.297	3.018	2.846	2.688	2.572
3.769	3.571	3.18	3.118	2.716	2.617	2.517
4.048	3.415	3.142	2.841	2.449	2.363	2.158
4.073	3.783	3.481	2.894	2.738	2.581	2.532

3.735	3.561	3.388	3.076	2.995	2.835	2.766
3.54	3.187	2.856	2.593	2.331	2.331	2.249
3.72	3.302	3.133	2.956	2.856	2.657	2.556
3.842	3.526	3.359	3.268	3.112	2.894	2.785
3.373	3.061	2.622	2.43	2.143	1.899	1.083
3.604	3.255	2.94	2.897	2.849	2.235	2.034
3.94	3.392	3.23	2.857	2.797	2.421	2.208
3.59	3.533	3.328	2.883	2.747	2.657	2.372
3.813	3.489	3.288	3.117	2.883	2.795	2.617
3.679	3.627	3.249	2.943	2.849	2.739	2.617
3.678	3.462	3.159	2.756	2.656	2.617	2.016
3.606	3.298	3.035	2.872	2.785	2.494	2.395
3.864	3.624	3.366	3.244	3.063	2.847	2.749
3.904	3.768	3.33	3.222	3.11	2.777	2.538
3.763	3.144	3.076	2.901	2.837	2.699	2.446
3.788	3.576	3.298	3.041	3.016	2.981	2.793
3.155	3.074	2.528	2.181	1.742	1.579	1.33
3.782	3.661	3.528	3.416	3.138	3.042	2.908
3.81	3.661	3.546	3.414	3.31	3.167	3.141
3.853	3.578	3.434	3.134	3.029	2.965	2.964
4.044	3.809	3.73	3.549	3.512	3.358	3.274
3.723	3.494	3.304	3.204	3.018	2.869	2.735
3.778	3.485	3.319	2.895	2.841	2.617	2.586
3.799	3.432	3.266	2.975	2.926	2.747	2.617
3.639	3.27	3.076	2.949	2.722	2.373	2.175
4.012	3.623	3.531	3.392	3.189	2.971	2.807
3.769	3.51	3.302	2.892	2.875	2.679	2.617
3.871	3.391	3.261	2.87	2.795	2.617	2.479

BELm1	BELm2	BELm3	BELm4	BELm5	BELm6	BELm7
1.833	1.768	1.6	1.296	1.206	0.975	0.75
1.948	1.821	1.783	1.635	1.52	1.323	1.249
2.017	1.933	1.712	1.492	1.228	1.082	1.074
1.925	1.559	1.082	1.06	0.692	0.444	0.311
1.724	1.615	1.472	1.173	0.985	0.802	0.677
1.786	1.355	0.977	0.74	0.736	0.579	0.531
1.994	1.893	1.705	1.686	1.629	1.467	1.372
1.696	1.332	0.989	0.582	0.304	0.193	0
1.993	1.88	1.628	1.519	1.181	1.082	0.994
1.969	1.821	1.651	1.626	1.249	1.188	1.136
1.94	1.847	1.831	1.76	1.503	1.461	1.399
1.971	1.862	1.834	1.798	1.562	1.541	1.5
2.017	1.969	1.776	1.529	1.519	1.249	1.209
2	1.805	1.666	1.652	1.489	1.433	1.262
1.956	1.808	1.708	1.452	1.249	1.218	1.082
1.969	1.809	1.709	1.464	1.249	1.218	1.082
1.937	1.705	1.444	1.207	1.082	0.999	0.594
1.981	1.953	1.745	1.572	1.464	1.273	1.214
1.96	1.821	1.641	1.476	1.249	1.173	1.082
1.978	1.878	1.734	1.57	1.434	1.082	1.079
1.972	1.831	1.666	1.459	1.423	1.249	1.183
1.768	1.629	1.425	1.42	1.035	0.927	0.571
1.976	1.914	1.6	1.235	1.082	1.043	0.966
1.971	1.809	1.709	1.442	1.249	1.214	1.082
1.996	1.92	1.839	1.75	1.723	1.458	1.359
2.082	1.86	1.802	1.577	1.478	1.422	1.344
1.967	1.822	1.652	1.606	1.449	1.372	1.249
1.875	1.847	1.832	1.776	1.7	1.643	1.542
2.013	1.914	1.781	1.592	1.405	1.301	1.249
1.951	1.821	1.768	1.639	1.566	1.28	1.249
1.999	1.919	1.833	1.71	1.669	1.554	1.418
2.074	1.922	1.843	1.801	1.76	1.723	1.584
1.991	1.867	1.681	1.58	1.26	1.249	1.249
1.993	1.939	1.789	1.493	1.429	1.408	1.312
2.151	1.941	1.833	1.695	1.51	1.482	1.45
1.933	1.898	1.786	1.723	1.697	1.414	1.372
2.127	1.991	1.828	1.651	1.494	1.293	1.26
1.761	1.593	1.448	1.215	1.077	0.682	0.45
2.031	1.954	1.903	1.799	1.612	1.534	1.471
2.065	1.925	1.648	1.592	1.378	1.144	1.101
2.047	1.959	1.842	1.663	1.467	1.252	1.192
1.958	1.954	1.441	1.333	1.053	0.993	0.974
2.162	2.047	1.87	1.718	1.687	1.624	1.564
2.027	1.913	1.617	1.222	1.113	0.995	0.869
2.032	1.918	1.917	1.719	1.494	1.418	1.339
1.97	1.825	1.516	1.387	1.082	1.07	0.912
1.957	1.871	1.62	1.437	1.166	1.082	1.069
1.964	1.859	1.596	1.554	1.374	1.084	1.082
1.865	1.654	1.532	1.519	1.152	1.077	1.066

1.913	1.771	1.723	1.592	1.47	1.393	1.239
1.88	1.664	1.555	1.442	1.288	1.237	1.074
1.976	1.858	1.822	1.645	1.586	1.556	1.503
1.87	1.643	1.557	1.491	1.394	1.24	1.11
1.86	1.684	1.573	1.427	1.362	1.264	1.067
1.957	1.887	1.702	1.489	1.386	1.082	1.07
1.974	1.96	1.831	1.578	1.381	1.267	1.082
1.969	1.834	1.756	1.601	1.494	1.249	1.241
1.852	1.606	1.479	1.393	1.239	1.075	1.013
1.864	1.643	1.611	1.51	1.394	1.3	1.239
1.869	1.654	1.57	1.472	1.376	1.279	1.083
1.995	1.913	1.542	1.375	1.224	1.082	1.082
1.97	1.872	1.622	1.448	1.166	1.082	1.069
1.969	1.837	1.675	1.548	1.414	1.147	1.082
1.872	1.687	1.593	1.508	1.411	1.099	1.068
1.963	1.872	1.625	1.47	1.22	1.168	1.07
2	1.839	1.801	1.675	1.499	1.372	1.342
2.024	1.867	1.846	1.601	1.403	1.372	1.237
1.88	1.342	1.094	0.99	0.625	0.513	0.261
2.005	1.828	1.741	1.477	1.249	1.109	1.082
1.978	1.808	1.784	1.537	1.249	1.169	1.025
1.885	1.781	1.361	1.1	1.082	0.86	0.684
2.017	1.808	1.771	1.525	1.249	1.203	1.023
1.966	1.857	1.343	1.154	1.1	0.972	0.932
1.957	1.845	1.424	1.363	1.082	0.977	0.825
1.719	1.674	1.504	1.428	1.232	1.196	0.883
1.971	1.916	1.68	1.415	1.349	1.21	1.134
2.118	1.983	1.753	1.62	1.509	1.217	1.151
1.941	1.642	1.4	1.323	1.008	0.911	0.782
1.985	1.813	1.529	1.372	1.372	1.283	1.088
1.879	1.856	1.695	1.619	1.578	1.377	1.324
1.9	1.728	1.249	1.082	0.949	0.851	0.311
1.978	1.909	1.733	1.453	1.386	1.173	1.082
1.867	1.599	1.387	0.943	0.911	0.571	0.414
2.058	1.901	1.831	1.42	1.372	1.26	1.138
1.77	1.667	1.435	1.154	0.863	0.739	0.695
1.952	1.883	1.194	1.094	1.082	1.082	0.751
1.978	1.969	1.763	1.519	1.475	1.181	1.065
2.082	1.86	1.802	1.577	1.478	1.422	1.344
1.99	1.869	1.604	1.384	1.141	1.082	0.971
1.978	1.842	1.451	1.162	0.968	0.911	0.773
1.892	1.811	1.719	1.623	1.321	1.249	1.212
1.775	1.687	1.557	1.286	1.046	0.901	0.674
2.001	1.774	1.166	1.049	1.007	0.733	0.674
2.115	1.9	1.788	1.607	1.548	1.474	1.384
2.115	1.909	1.893	1.857	1.814	1.783	1.607
1.949	1.92	1.752	1.599	1.491	1.427	1.354
2.014	1.886	1.749	1.438	1.249	1.248	1.082
2.029	1.875	1.838	1.666	1.592	1.286	1.249
2.078	1.975	1.942	1.938	1.814	1.622	1.608

1.977	1.923	1.741	1.493	1.444	1.423	1.336
1.955	1.918	1.855	1.71	1.372	1.354	1.256
2.079	1.897	1.664	1.585	1.476	1.31	1.249
1.949	1.785	1.562	1.513	1.148	1.01	0.917
1.864	1.807	1.6	1.406	1.363	1.01	0.897
2.011	1.904	1.651	1.561	1.44	1.274	1.072
2.056	1.868	1.841	1.618	1.583	1.437	1.431
1.693	1.542	1.463	1.194	1.061	0.932	0.889
2.035	1.849	1.67	1.163	1.082	1.082	0.988
1.982	1.906	1.597	1.082	1.076	1.064	0.944
2	1.964	1.872	1.699	1.542	1.372	1.208
1.947	1.92	1.574	1.196	1.082	1.082	1.016
2.097	1.874	1.821	1.73	1.572	1.469	1.359
1.947	1.91	1.54	1.453	1.398	1.299	1.16
1.946	1.674	1.462	1.309	1.296	1.082	1.017
1.944	1.925	1.44	1.082	1.065	1.011	0.744
1.93	1.722	1.648	1.404	1.349	1.107	1.023
1.664	0.968	0.823	0.235	0.062	0	0
1.99	1.899	1.831	1.697	1.533	1.372	1.221
2.029	1.859	1.568	1.085	1.082	1.011	0.975
1.092	0.649	0.471	0.201	0	0	0
2.114	1.917	1.857	1.795	1.753	1.713	1.599
1.9	1.639	1.473	1.158	1.095	0.986	0.857
2.129	1.922	1.879	1.755	1.613	1.605	1.547
2.011	1.601	1.372	1.333	1.082	0.697	0.666
1.986	1.88	1.686	1.451	1.372	1.259	1.184
1.745	1.664	1.489	1.173	1.173	0.973	0.677
1.979	1.89	1.578	1.509	1.249	1.21	1.105
1.942	1.887	1.863	1.536	1.501	1.433	1.358
1.989	1.964	1.827	1.631	1.557	1.541	1.372
1.971	1.965	1.897	1.592	1.432	1.41	1.372
2.028	1.974	1.631	1.554	1.437	1.437	1.302
1.952	1.899	1.702	1.498	1.324	1.107	1.082
1.886	1.452	1.115	1.001	0.701	0.666	0.346
2.092	1.819	1.642	1.463	1.356	1.205	1.058
2.016	1.802	1.588	1.249	1.178	1.082	1.003
1.997	1.963	1.921	1.847	1.667	1.465	1.41
1.975	1.913	1.737	1.452	1.387	1.156	1.082
1.98	1.936	1.622	1.391	1.121	1.082	1.037
2.042	1.865	1.833	1.723	1.695	1.514	1.34
1.651	1.612	1.313	1.008	0.984	0.618	0.405
1.649	1.609	1.312	0.997	0.703	0.513	0.357
2.007	1.81	1.741	1.542	1.434	1.307	1.249
2.02	1.834	1.698	1.221	1.082	1.04	0.95
2.013	1.91	1.606	1.197	1.082	1.065	0.946
1.772	1.518	1.435	1.075	0.877	0.753	0.545
1.952	1.906	1.448	1.402	1.371	1.235	1.06
2.025	1.888	1.478	1.303	1.095	1.082	1.082
1.806	1.693	1.442	1.26	0.942	0.632	0.603
2.005	1.848	1.661	1.515	1.372	1.371	1.202

1.987	1.807	1.562	1.493	1.441	1.31	1.002
1.944	1.756	1.707	1.524	1.372	1.264	1.035
1.979	1.851	1.642	1.514	1.464	1.183	0.987
1.968	1.848	1.514	1.18	1.016	0.999	0.851
2.043	1.88	1.826	1.625	1.477	1.293	1.249
1.951	1.612	1.596	1.37	1.182	1.054	1.004
1.986	1.704	1.565	1.435	1.082	1.076	0.864
2.026	1.822	1.65	1.437	1.372	1.115	1.082
2.003	1.927	1.738	1.387	1.342	1.326	1.007
1.992	1.924	1.493	1.46	1.413	1.312	1.162
1.65	1.316	1.027	0.632	0.125	0.05	0
1.688	1.449	1.249	1.04	0.889	0.304	0
2.054	1.886	1.802	1.698	1.493	1.332	1.249
1.925	1.803	1.7	1.437	1.394	1.255	1.249
1.974	1.912	1.785	1.591	1.392	1.356	1.159
2.105	1.861	1.746	1.647	1.475	1.434	1.35
1.903	1.824	1.57	1.562	1.411	1.215	1.011
1.966	1.822	1.647	1.573	1.349	1.249	1.169
1.767	1.541	1.421	0.95	0.807	0.461	0.383
1.978	1.871	1.649	1.571	1.226	1.029	1.003
2.062	2.021	1.963	1.693	1.664	1.62	1.557
2.093	1.82	1.635	1.458	1.347	1.185	1.024
1.96	1.92	1.622	1.462	1.429	1.397	1.372
1.988	1.748	1.7	1.534	1.422	1.31	1.249
2.029	1.868	1.668	1.602	1.326	1.249	1.249
2.1	1.809	1.708	1.663	1.527	1.277	1.249
1.96	1.688	1.543	1.317	0.972	0.814	0.719
2.089	1.846	1.696	1.499	1.45	1.372	1.315
2.086	1.832	1.725	1.558	1.441	1.234	1.102
2.109	1.729	1.478	1.363	1.169	1.003	0.85
1.982	1.699	1.637	1.249	1.249	1.249	1.057
2.077	1.86	1.819	1.703	1.505	1.447	1.394
2.023	1.824	1.552	1.439	1.43	1.294	1.177
1.957	1.886	1.448	1.166	1.082	1.076	0.933
2.022	1.826	1.652	1.539	1.434	1.31	1.165
1.64	1.533	1.436	1.245	0.921	0.545	0.334
1.941	1.567	1.063	1.036	0.776	0.597	0.525
1.993	1.822	1.641	1.491	1.372	1.094	1.016
2.016	1.969	1.745	1.519	1.471	1.213	1.065
1.987	1.965	1.876	1.788	1.556	1.523	1.422
2.005	1.848	1.661	1.515	1.372	1.371	1.202
1.939	1.931	1.515	1.424	1.417	1.397	1.278
1.988	1.925	1.643	1.475	1.387	1.206	1.152
1.959	1.813	1.721	1.625	1.322	1.249	1.214
1.988	1.861	1.515	1.183	1.082	1.001	0.918
2.082	1.819	1.643	1.472	1.422	1.319	1.193
1.949	1.91	1.858	1.568	1.206	1.181	1.082
2.128	2.001	1.992	1.853	1.699	1.61	1.567
1.95	1.725	1.525	1.436	1.14	0.958	0.884
1.931	1.639	1.553	1.082	1.044	0.846	0.623

2.057	1.889	1.844	1.372	1.372	1.341	1.115
1.996	1.731	1.465	1.082	1.069	0.884	0.842
1.955	1.921	1.859	1.569	1.219	1.183	1.082
2.016	1.886	1.371	1.283	1.082	1.082	0.918
1.958	1.821	1.638	1.568	1.249	1.178	1.147
1.99	1.96	1.821	1.721	1.599	1.534	1.372
1.948	1.882	1.855	1.507	1.372	1.322	1.252
1.952	1.883	1.723	1.517	1.174	1.082	1.059
1.955	1.653	1.62	1.417	1.04	0.984	0.734
1.932	1.82	1.637	1.555	1.249	1.185	1.082
2.06	1.846	1.83	1.716	1.599	1.444	1.376
2.02	1.843	1.581	1.486	1.419	1.372	1.372
2.089	1.85	1.746	1.561	1.449	1.424	1.314
2.114	1.89	1.775	1.57	1.546	1.473	1.387
1.948	1.827	1.572	1.562	1.225	1.082	1.016
1.942	1.821	1.562	1.526	1.205	1.082	0.971
1.993	1.929	1.813	1.658	1.591	1.293	1.126
2.096	1.823	1.647	1.563	1.249	1.218	1.116
2.013	1.765	1.586	1.249	1.249	0.968	0.931
2.096	1.823	1.647	1.563	1.249	1.218	1.116
1.98	1.76	1.523	1.161	1.082	1.006	0.839
1.947	1.899	1.848	1.649	1.486	1.372	1.226
2.088	2.012	1.628	1.557	1.416	1.357	1.245
2.088	2.012	1.628	1.557	1.416	1.357	1.245
1.751	1.743	1.61	1.521	1.421	1.361	1.249
2.019	1.959	1.886	1.806	1.642	1.517	1.372
1.902	1.296	1.023	0.962	0.7	0.368	0.175
1.909	1.444	1	0.898	0.744	0.736	0.565
1.996	1.949	1.871	1.744	1.496	1.372	1.328
2.003	1.793	1.58	1.537	1.311	1.269	1.249
2.119	1.907	1.894	1.779	1.723	1.645	1.571
1.993	1.936	1.729	1.493	1.418	1.314	1.295
1.926	1.171	1.051	0.726	0.459	0.254	0.22
1.978	1.881	1.57	1.52	1.434	1.082	1.045
1.941	1.847	1.81	1.677	1.629	1.483	1.39
1.966	1.794	1.511	1.436	1.249	1.083	1.022
1.994	1.895	1.718	1.681	1.515	1.446	1.372
1.92	1.658	1.346	1.139	1.082	0.857	0.704
1.949	1.919	1.545	1.305	1.149	1.082	1.082
1.955	1.922	1.916	1.734	1.229	1.219	1.082
1.92	1.739	1.429	1.205	1.082	1.072	0.705
1.969	1.9	1.75	1.422	1.385	1.249	1.082
1.919	1.784	1.733	1.513	1.416	1.248	1.118
1.995	1.791	1.579	1.528	1.377	1.249	1.213
1.986	1.641	1.551	1.215	1.082	1.025	0.832
1.965	1.918	1.739	1.634	1.54	1.414	1.197
1.944	1.864	1.634	1.563	1.355	1.331	1.086
1.99	1.868	1.574	1.366	1.128	1.082	0.971
1.846	1.715	1.419	1.294	1.072	1.019	0.837
1.878	1.704	1.452	1.345	1.136	0.992	0.804

2.02	1.849	1.714	1.498	1.461	1.419	1.372
1.948	1.842	1.501	1.249	1.249	1.024	1.004
2.108	1.863	1.728	1.582	1.499	1.379	1.262
2.076	1.899	1.664	1.581	1.47	1.321	1.249
1.738	1.475	1.422	1.214	0.725	0.489	0.223
1.949	1.723	1.524	1.407	1.139	0.929	0.884
1.954	1.826	1.465	1.341	1.112	1.013	0.913
1.869	1.81	1.665	1.372	1.296	1.249	1.249
1.956	1.936	1.757	1.558	1.411	1.273	1.168
1.951	1.839	1.74	1.405	1.263	1.114	1.082
1.951	1.831	1.485	1.394	1.178	1.082	0.916
2.036	1.854	1.68	1.421	1.382	1.308	1.249
1.956	1.829	1.819	1.672	1.372	1.279	1.123
1.974	1.865	1.627	1.519	1.179	1.016	0.988
1.963	1.762	1.437	1.428	1.401	1.228	1.099
1.966	1.853	1.78	1.647	1.446	1.418	1.372
1.905	1.707	1.434	1.091	0.95	0.678	0.622
2.044	1.894	1.827	1.81	1.508	1.354	1.337
2.121	2.001	1.943	1.85	1.698	1.598	1.567
2.01	1.96	1.802	1.733	1.565	1.434	1.419
2.047	2.009	1.967	1.901	1.757	1.704	1.607
2.054	1.985	1.696	1.668	1.619	1.504	1.161
2.016	1.931	1.708	1.489	1.216	1.082	1.064
2.006	1.793	1.665	1.507	1.492	1.061	1.049
1.815	1.722	1.516	1.274	1.159	0.949	0.87
1.969	1.939	1.827	1.532	1.463	1.372	1.216
1.99	1.961	1.713	1.538	1.417	1.347	1.249
1.928	1.749	1.696	1.392	1.122	1.082	1.02

BELm8	BEHv1	BEHv2	BEHv3	BEHv4	BEHv5	BEHv6
0.645	3.855	3.331	3.214	2.813	2.684	2.501
1.178	3.843	3.454	3.407	3.213	3.142	2.888
1.064	3.924	3.797	3.483	3.223	2.987	2.741
0.105	3.783	3.188	2.697	2.647	2.021	1.509
0.508	3.786	3.217	3.096	2.717	2.431	2.188
0.471	3.711	3.217	2.959	2.536	1.927	1.608
1.262	3.832	3.637	3.454	3.303	3.161	2.965
0	3.702	2.922	2.628	2.028	1.857	0.94
0.908	3.962	3.741	3.527	3.13	2.801	2.667
1	3.824	3.454	3.291	3.211	2.723	2.678
1.34	3.741	3.597	3.58	3.433	3.1	3.06
1.236	4.026	3.83	3.439	3.438	3.229	3.016
1.065	3.988	3.701	3.431	3.146	3.124	2.741
1.034	3.921	3.648	3.316	3.202	3.096	2.895
0.899	3.841	3.776	3.349	3.163	2.894	2.647
0.913	3.838	3.774	3.347	3.156	2.893	2.647
0.311	3.811	3.559	2.803	2.718	2.647	2.534
1.128	3.884	3.819	3.527	3.348	3.186	3.027
1.014	3.817	3.456	3.245	3.156	2.666	2.647
1.048	3.834	3.755	3.296	3.026	2.833	2.743
1.021	3.889	3.584	3.306	3.114	2.934	2.693
0.354	3.888	3.703	2.998	2.911	2.654	2.447
0.831	3.98	3.759	3.387	2.993	2.681	2.647
0.912	3.832	3.771	3.34	3.113	2.892	2.647
1.249	3.848	3.74	3.615	3.431	3.256	2.982
1.297	3.994	3.771	3.369	3.195	3.096	3.003
1.171	3.82	3.693	3.456	3.234	3.126	2.682
1.489	3.998	3.873	3.752	3.703	3.283	3.271
1.098	4.057	3.717	3.392	3.212	2.989	2.736
1.249	3.808	3.456	3.365	3.221	3.108	2.867
1.249	3.932	3.799	3.711	3.553	3.453	3.155
1.44	4.098	3.855	3.651	3.534	3.469	3.307
1.037	4	3.527	3.359	3.117	2.907	2.55
1.196	3.855	3.645	3.349	3.078	3.01	2.844
1.382	4.094	3.732	3.706	3.548	3.321	3.147
1.239	3.808	3.672	3.506	3.382	3.202	2.835
1.111	4.002	3.69	3.584	3.265	3.089	2.867
0	3.794	3.196	2.901	2.601	2.461	2.325
1.418	3.934	3.75	3.57	3.411	3.203	3.125
1.047	4.033	3.808	3.493	3.192	2.955	2.836
1.132	4.017	3.921	3.679	3.325	3.277	3.076
0.643	3.947	3.692	3.161	3.081	2.638	2.629
1.464	4.036	3.82	3.626	3.415	3.355	3.226
0.785	4.129	3.653	3.294	2.947	2.771	2.587
1.214	4.13	3.776	3.59	3.348	3.105	2.993
0.894	3.829	3.754	3.332	3.145	2.824	2.647
0.894	3.838	3.771	3.373	3.17	2.844	2.647
1.067	3.836	3.774	3.527	3.356	3.136	2.856
0.86	3.81	3.616	3.522	3.341	2.975	2.876

1.226	3.837	3.724	3.629	3.334	3.133	2.87
1.011	3.819	3.721	3.411	3.217	3.084	2.939
1.149	3.862	3.782	3.584	3.528	3.385	3.282
1.069	3.81	3.717	3.38	3.169	3.105	2.839
1.051	3.825	3.758	3.395	3.079	2.988	2.828
0.979	3.845	3.785	3.453	3.216	3.066	2.814
1.082	3.888	3.826	3.722	3.48	3.329	3.005
1.153	3.848	3.734	3.686	3.459	3.344	3.104
0.667	3.787	3.708	3.306	3.118	2.821	2.75
1.09	3.816	3.719	3.512	3.359	3.14	2.918
1.058	3.809	3.713	3.376	3.145	2.99	2.827
1.064	3.964	3.818	3.591	3.267	2.95	2.647
0.912	3.834	3.769	3.371	3.162	2.844	2.647
1.017	3.85	3.771	3.691	3.346	3.063	2.835
0.941	3.818	3.756	3.38	3.149	3.05	2.839
1.024	3.811	3.735	3.37	3.178	2.844	2.717
1.158	3.85	3.605	3.587	3.233	3.069	2.987
1.082	3.902	3.703	3.532	3.247	3.063	2.9
0	3.97	3.527	3.139	2.804	2.439	2.346
0.976	3.879	3.661	3.359	3.053	2.647	2.637
0.988	3.931	3.665	3.404	3.056	2.877	2.738
0.497	3.873	3.407	3.287	2.86	2.647	2.448
0.973	3.986	3.665	3.426	3.139	2.871	2.736
0.693	3.974	3.798	3.333	3.103	2.834	2.581
0.635	3.917	3.714	3.142	2.783	2.681	2.647
0.698	3.729	3.404	3.157	2.842	2.74	2.656
1.082	3.854	3.782	3.45	3.073	2.943	2.831
1.105	4.025	3.838	3.432	3.295	3.131	2.91
0.464	3.895	3.398	3.136	2.915	2.7	2.473
0.967	3.936	3.63	3.493	3.341	2.797	2.785
1.238	3.736	3.635	3.365	3.243	3.008	2.909
0.301	3.781	3.437	2.7	2.647	2.525	2.414
1.041	4.065	3.782	3.38	3.156	2.882	2.804
0.193	3.804	3.467	2.798	2.721	2.384	2.07
1.035	4.027	3.721	3.513	3.083	2.913	2.797
0.488	3.621	3.288	3.012	2.858	2.504	2.093
0.628	3.967	3.718	3.261	2.66	2.647	2.647
0.999	3.936	3.701	3.365	3.13	2.961	2.708
1.297	3.994	3.771	3.369	3.195	3.096	3.003
0.823	3.924	3.718	3.298	3.018	2.755	2.647
0.615	3.871	3.734	3.149	2.856	2.721	2.588
0.974	3.906	3.79	3.585	3.301	2.9	2.808
0.519	3.814	3.506	3.063	2.819	2.554	2.23
0.413	3.924	3.648	2.987	2.796	2.652	2.357
1.306	3.983	3.754	3.512	3.243	3.165	2.976
1.553	3.989	3.758	3.67	3.583	3.526	3.5
1.249	3.895	3.76	3.408	3.305	3.092	2.978
1.082	3.881	3.696	3.341	3.027	2.761	2.647
1.249	3.918	3.656	3.536	3.271	3.153	2.84
1.56	3.975	3.818	3.784	3.742	3.467	3.371

1.082	3.851	3.803	3.509	3.483	3.356	3.023
1.151	3.878	3.836	3.594	3.321	3.11	3.004
1.153	4.002	3.776	3.463	3.241	3.153	3.019
0.77	3.873	3.531	3.264	2.966	2.955	2.735
0.827	3.529	3.422	3.095	2.799	2.76	2.306
0.947	4.072	3.58	3.268	3.071	2.991	2.944
1.391	4.002	3.759	3.636	3.531	3.207	3.043
0.85	3.728	3.569	3.253	3.113	2.808	2.458
0.749	3.925	3.664	3.364	2.953	2.647	2.647
0.867	3.854	3.727	3.275	2.77	2.647	2.573
1.082	3.834	3.786	3.581	3.308	3.121	2.807
0.719	3.944	3.764	3.542	3.063	2.779	2.647
1.265	3.963	3.708	3.49	3.283	3.155	2.923
0.991	3.828	3.602	3.188	3.141	3.085	2.867
0.84	3.854	3.583	3.514	3.132	2.652	2.514
0.352	3.958	3.734	3.223	2.775	2.648	2.647
0.874	3.932	3.606	3.198	3.139	2.787	2.728
0	3.523	2.724	2.511	1.337	1.187	0.886
1.058	3.941	3.684	3.582	3.299	3.079	2.999
0.674	3.929	3.671	3.266	2.733	2.652	2.647
0	3.409	2.284	1.409	1.233	1.094	0
1.469	3.998	3.74	3.612	3.515	3.409	3.298
0.588	3.915	3.687	3.362	2.941	2.849	2.431
1.509	4.016	3.769	3.587	3.436	3.231	3.185
0.65	3.742	3.205	2.883	2.797	2.436	1.905
1.013	3.988	3.574	3.303	3.007	2.868	2.828
0.56	3.787	3.352	3.132	2.724	2.529	2.425
0.982	3.837	3.605	3.138	3.042	2.667	2.527
1.309	3.731	3.617	3.572	3.109	3.044	2.941
1.354	3.902	3.753	3.681	3.44	3.195	3.089
1.202	3.904	3.814	3.645	3.435	3.169	2.996
1.033	3.993	3.757	3.286	3.146	2.982	2.952
0.988	3.849	3.766	3.356	3.191	2.97	2.906
0.184	3.965	3.515	3.141	2.843	2.457	2.364
0.9	4.03	3.566	3.324	3.113	2.885	2.72
0.706	3.866	3.446	3.221	2.647	2.647	2.493
1.372	3.894	3.816	3.779	3.524	3.475	3.153
1.045	4.063	3.785	3.39	3.14	2.887	2.805
0.948	3.927	3.785	3.314	3.072	2.767	2.647
1.327	3.961	3.798	3.677	3.437	3.33	3.198
0.328	3.676	3.133	2.851	2.75	2.255	2.254
0.267	3.67	3.097	2.794	2.298	2.254	1.79
1.069	3.937	3.673	3.35	3.22	2.912	2.88
0.867	3.931	3.679	3.484	2.995	2.69	2.647
0.87	3.904	3.734	3.301	2.964	2.647	2.574
0.398	3.645	3.399	2.903	2.659	2.547	1.982
0.892	3.966	3.784	3.34	3.123	2.958	2.84
1.032	3.93	3.749	3.51	3.113	2.905	2.647
0.344	3.642	3.312	2.853	2.541	2.352	1.986
0.964	3.942	3.547	3.377	3.308	2.865	2.797

0.851	3.85	3.444	3.065	3.01	2.955	2.781
0.997	3.837	3.501	3.379	3.183	2.81	2.797
0.958	3.919	3.559	3.387	3.135	2.966	2.787
0.567	3.923	3.721	3.29	2.955	2.709	2.687
1.132	3.963	3.797	3.646	3.348	3.322	2.953
0.747	3.96	3.705	3.426	3.204	2.813	2.707
0.833	3.842	3.404	3.255	3.012	2.735	2.647
0.911	3.877	3.534	3.172	2.992	2.797	2.647
0.96	3.941	3.655	3.451	2.97	2.883	2.821
0.941	3.854	3.621	3.078	3.01	2.869	2.799
0	3.685	3.014	2.678	2.202	1.649	1.197
0	3.403	3.026	2.528	2.414	1.923	1.478
1.082	3.944	3.696	3.473	3.298	2.989	2.79
1.115	3.85	3.767	3.316	2.965	2.906	2.886
1.099	3.881	3.805	3.504	3.327	3.12	3.004
1.294	3.995	3.762	3.355	3.266	3.078	2.986
0.913	3.836	3.453	3.154	2.955	2.915	2.828
1.082	3.816	3.456	3.24	3.148	2.763	2.647
0	3.765	3.061	2.922	2.484	2.103	1.547
0.983	3.976	3.751	3.582	3.131	2.828	2.679
1.372	3.994	3.897	3.83	3.785	3.442	3.303
0.873	4.036	3.583	3.329	3.122	2.884	2.687
1.276	3.952	3.716	3.604	3.41	3.12	2.882
1.249	3.871	3.335	3.311	3.129	2.875	2.742
1.168	3.899	3.532	3.339	3.252	2.907	2.603
1.188	4.026	3.794	3.398	3.3	3.086	3.025
0.624	3.866	3.436	3.131	2.907	2.588	2.39
1.114	4.044	3.708	3.586	3.361	3.118	2.876
1.003	4.032	3.572	3.41	3.196	3.113	2.805
0.695	4.021	3.502	3.135	2.905	2.697	2.501
0.947	3.825	3.309	3.225	2.714	2.53	2.414
1.223	4.097	3.754	3.621	3.301	3.188	2.974
0.988	3.933	3.661	3.279	2.971	2.925	2.839
0.758	3.864	3.727	3.495	2.977	2.759	2.647
1.018	3.928	3.669	3.293	3.11	2.998	2.882
0.117	3.749	3.468	2.994	2.66	2.501	2.126
0.398	3.819	3.202	2.682	2.622	2.074	1.596
0.885	3.917	3.529	3.352	3.135	2.797	2.784
0.999	3.987	3.701	3.404	3.13	3.048	2.687
1.38	3.92	3.843	3.655	3.507	3.392	3.147
0.964	3.942	3.547	3.377	3.308	2.865	2.797
1.07	3.972	3.812	3.367	3.08	2.912	2.794
1.003	4.07	3.693	3.484	3.147	2.883	2.754
1.082	3.894	3.787	3.577	3.301	2.9	2.805
0.577	3.919	3.714	3.29	2.955	2.709	2.647
1.038	4.034	3.575	3.326	3.117	2.931	2.781
1.082	3.857	3.739	3.561	3.266	2.818	2.71
1.534	3.991	3.782	3.656	3.552	3.31	3.175
0.701	3.743	3.319	3.058	2.915	2.616	2.541
0.311	3.79	3.207	3.097	2.65	2.647	2.029

1.082	3.886	3.592	3.474	2.943	2.797	2.797
0.574	3.869	3.491	3.053	2.718	2.647	2.616
1.082	3.855	3.735	3.56	3.265	2.808	2.694
0.837	3.925	3.696	3.366	2.778	2.647	2.647
0.871	3.993	3.459	3.241	3.177	2.788	2.465
1.249	3.971	3.743	3.664	3.408	3.314	3.069
1.07	3.893	3.653	3.553	3.177	3.022	2.81
0.946	3.936	3.739	3.535	3.323	2.941	2.659
0.677	3.871	3.561	3.259	2.686	2.593	2.487
1.005	3.792	3.454	3.232	3.166	2.719	2.647
1.334	3.89	3.538	3.5	3.382	3.237	3.027
1.263	3.958	3.719	3.501	3.24	2.914	2.835
1.187	3.985	3.756	3.348	3.181	3.073	2.961
1.285	3.979	3.731	3.331	3.169	3.075	2.971
0.869	3.815	3.452	3.15	2.955	2.879	2.647
0.826	3.81	3.442	3.093	2.955	2.878	2.647
1.083	3.853	3.771	3.431	3.253	3.222	2.825
0.871	4.002	3.461	3.323	3.205	2.904	2.526
0.516	3.877	3.367	3.137	2.617	2.459	2.414
0.871	4.002	3.461	3.323	3.205	2.904	2.526
0.659	3.895	3.581	3.08	2.841	2.647	2.547
1.046	3.928	3.692	3.543	3.244	3.071	2.912
1.194	3.998	3.78	3.382	3.211	3	2.809
1.194	3.998	3.78	3.382	3.211	3	2.809
1.048	3.758	3.505	3.257	3.152	2.849	2.779
1.351	3.889	3.829	3.529	3.458	3.265	3.11
0	3.958	3.222	2.809	2.644	2.372	1.626
0.471	3.806	3.567	2.959	2.812	2.479	2.179
1.268	3.97	3.778	3.579	3.415	3.132	2.946
1.046	4.032	3.576	3.378	3.189	2.873	2.633
1.54	3.986	3.772	3.62	3.457	3.256	3.237
1.12	3.855	3.64	3.254	3.077	3.01	2.799
0	3.799	2.961	2.681	2.264	1.452	1.228
0.988	3.862	3.806	3.31	3.112	2.804	2.803
1.372	3.95	3.801	3.56	3.298	3.248	3.094
0.748	3.847	3.503	3.394	3.087	2.669	2.537
1.259	3.842	3.772	3.62	3.306	3.139	2.88
0.536	3.886	3.635	3.286	2.695	2.647	2.484
0.914	3.911	3.813	3.559	3.27	2.668	2.647
1.082	3.87	3.8	3.735	3.534	3.185	2.788
0.664	3.89	3.691	3.296	2.736	2.647	2.574
1.029	4.022	3.81	3.419	3.253	3.05	2.797
1.044	3.9	3.646	3.365	3.085	3.028	2.766
1.022	4.037	3.603	3.356	3.147	2.853	2.671
0.515	3.897	3.713	3.303	2.814	2.647	2.499
1.142	3.908	3.776	3.453	3.265	3.119	3.068
1.017	3.93	3.491	3.376	3.202	2.93	2.825
0.822	3.925	3.718	3.313	3.018	2.778	2.647
0.676	3.894	3.453	3.26	2.969	2.658	2.336
0.539	3.958	3.701	3.371	3.167	2.751	2.62

1.268	3.958	3.624	3.463	3.238	2.93	2.859
0.725	3.823	3.502	3.129	2.711	2.548	2.414
1.142	3.965	3.712	3.269	3.168	2.97	2.839
1.172	4.003	3.776	3.462	3.239	3.169	3.014
0	3.764	3.153	2.89	2.486	2.326	2.023
0.698	3.747	3.33	3.102	2.916	2.616	2.609
0.68	3.883	3.781	3.189	2.885	2.691	2.662
1.113	3.658	3.524	3.325	3.226	2.797	2.534
1.082	3.82	3.792	3.403	3.154	2.982	2.909
0.965	3.889	3.579	3.394	3.017	2.987	2.77
0.747	3.889	3.451	3.361	2.988	2.723	2.647
1.031	3.88	3.591	3.223	2.95	2.82	2.81
1.034	3.814	3.724	3.513	3.282	2.996	2.797
0.848	3.965	3.749	3.527	3.13	2.802	2.687
0.952	3.865	3.636	3.028	2.906	2.76	2.715
1.33	3.815	3.635	3.503	3.239	2.925	2.857
0.407	3.587	3.2	2.951	2.421	2.262	1.673
1.249	3.956	3.709	3.573	3.412	3.33	3.1
1.533	3.985	3.765	3.622	3.532	3.309	3.172
1.417	3.895	3.821	3.537	3.361	3.115	2.97
1.554	4.115	4	3.776	3.706	3.473	3.385
1.135	4.114	3.714	3.381	3.298	3.145	3.038
0.967	3.916	3.778	3.456	3.223	2.821	2.73
0.987	3.975	3.74	3.337	3.214	2.964	2.742
0.64	3.698	3.515	2.959	2.893	2.786	2.483
1.034	3.984	3.902	3.528	3.295	3.117	2.943
1.082	4.013	3.766	3.415	3.226	2.892	2.868
0.759	3.923	3.741	3.17	2.922	2.82	2.647

BEHv7	BEHv8	BELv1	BELv2	BELv3	BELv4	BELv5
2.155	1.731	1.8	1.798	1.577	1.423	1.207
2.648	2.528	1.978	1.825	1.744	1.616	1.504
2.647	2.645	2.044	1.961	1.744	1.538	1.453
1.247	1.069	1.94	1.585	1.12	0.996	0.784
1.937	1.709	1.887	1.723	1.555	1.293	1.035
1.409	1.409	1.91	1.633	1.391	1.181	0.831
2.808	2.797	2.016	1.88	1.722	1.683	1.631
0.557	0.43	1.825	1.497	1.066	0.83	0.213
2.647	2.531	2	1.892	1.677	1.559	1.231
2.442	2.414	1.962	1.821	1.621	1.571	1.189
3.009	2.917	1.999	1.926	1.889	1.779	1.545
2.955	2.877	1.974	1.903	1.828	1.785	1.554
2.676	2.539	1.994	1.934	1.774	1.478	1.445
2.818	2.687	2.023	1.823	1.694	1.648	1.515
2.605	2.414	1.972	1.895	1.745	1.547	1.341
2.577	2.414	1.968	1.893	1.744	1.539	1.34
1.898	1.51	1.934	1.702	1.499	1.192	0.996
2.88	2.731	2.01	1.962	1.79	1.6	1.512
2.435	2.414	1.959	1.823	1.636	1.54	1.182
2.647	2.528	1.965	1.887	1.663	1.603	1.405
2.586	2.414	2.014	1.801	1.707	1.46	1.448
2.114	2.093	1.933	1.797	1.695	1.572	1.078
2.595	2.489	1.986	1.923	1.618	1.402	1.149
2.575	2.414	1.959	1.893	1.744	1.487	1.328
2.68	2.647	1.994	1.89	1.828	1.731	1.645
2.83	2.767	2.074	1.865	1.772	1.523	1.508
2.647	2.493	1.961	1.824	1.66	1.597	1.38
3.119	3.054	1.874	1.835	1.833	1.756	1.718
2.647	2.572	2.003	1.933	1.795	1.634	1.369
2.647	2.501	1.955	1.823	1.768	1.625	1.547
3.099	2.862	2.063	1.98	1.91	1.808	1.725
3.145	3.111	2.075	1.943	1.875	1.809	1.722
2.414	2.414	1.982	1.869	1.693	1.514	1.262
2.799	2.586	1.996	1.925	1.712	1.452	1.4
2.96	2.855	2.145	1.94	1.824	1.697	1.448
2.825	2.797	1.977	1.939	1.81	1.669	1.645
2.763	2.607	2.125	1.942	1.803	1.629	1.433
1.935	1.355	1.903	1.682	1.429	1.231	1.044
3.027	2.904	1.995	1.926	1.863	1.756	1.538
2.729	2.684	2.077	1.983	1.648	1.562	1.436
3.003	2.865	2.044	1.979	1.835	1.697	1.526
2.52	2.271	1.97	1.929	1.498	1.384	1.04
3.189	3.056	2.136	2.014	1.848	1.738	1.651
2.497	2.123	2.016	1.947	1.632	1.264	1.149
2.9	2.855	2.019	1.945	1.938	1.766	1.537
2.572	2.31	1.992	1.932	1.613	1.455	1.236
2.62	2.546	2.004	1.943	1.661	1.501	1.296
2.689	2.647	2.001	1.935	1.719	1.631	1.457
2.768	2.512	1.995	1.777	1.599	1.561	1.366

2.788	2.749	2.015	1.862	1.732	1.645	1.51
2.778	2.48	1.997	1.77	1.634	1.552	1.473
3.078	2.856	2.01	1.957	1.901	1.721	1.666
2.754	2.562	1.998	1.764	1.653	1.518	1.482
2.71	2.509	2.007	1.739	1.628	1.502	1.461
2.647	2.599	2.007	1.945	1.71	1.556	1.424
2.826	2.801	2.048	2.006	1.93	1.746	1.599
2.835	2.72	2.036	1.937	1.819	1.68	1.542
2.471	2.281	1.985	1.747	1.54	1.482	1.384
2.833	2.761	1.996	1.843	1.752	1.579	1.482
2.753	2.513	1.998	1.772	1.641	1.522	1.482
2.647	2.638	2.043	1.948	1.7	1.601	1.409
2.6	2.536	2.003	1.939	1.658	1.494	1.296
2.717	2.647	2.036	1.936	1.704	1.543	1.459
2.56	2.34	1.997	1.736	1.633	1.517	1.456
2.611	2.551	2.001	1.925	1.66	1.505	1.296
2.797	2.749	1.958	1.823	1.768	1.665	1.468
2.797	2.647	2.026	1.874	1.839	1.671	1.453
1.727	1.353	2.095	1.814	1.435	1.233	0.828
2.582	2.425	2.002	1.829	1.749	1.414	1.117
2.601	2.457	2.025	1.789	1.783	1.499	1.139
2.289	1.609	2.014	1.768	1.614	1.32	0.996
2.601	2.457	2.031	1.787	1.776	1.477	1.131
2.428	2.195	2.028	1.9	1.673	1.454	1.151
2.336	1.988	1.969	1.872	1.431	1.342	0.996
2.344	1.937	1.795	1.733	1.583	1.47	1.302
2.647	2.642	1.976	1.913	1.712	1.483	1.355
2.776	2.681	2.1	1.965	1.743	1.562	1.448
2.149	2.092	2.01	1.627	1.537	1.432	1.123
2.717	2.401	2.038	1.854	1.597	1.33	1.272
2.755	2.736	1.94	1.813	1.765	1.628	1.502
1.491	1.251	1.924	1.73	1.158	1.117	0.996
2.667	2.647	1.999	1.93	1.77	1.498	1.385
1.529	1.349	1.906	1.575	1.537	0.979	0.909
2.722	2.66	2.083	1.939	1.846	1.424	1.272
1.788	1.526	1.928	1.671	1.403	1.217	1.116
2.358	1.802	2.072	1.874	1.533	1.14	1.009
2.676	2.539	1.987	1.934	1.743	1.445	1.444
2.83	2.767	2.074	1.865	1.772	1.523	1.508
2.577	2.488	1.991	1.874	1.671	1.363	1.107
2.514	1.865	1.987	1.848	1.512	1.199	0.971
2.75	2.721	1.936	1.892	1.752	1.66	1.444
2.118	1.918	1.876	1.715	1.69	1.339	1.115
2.253	1.413	2.011	1.803	1.324	1.09	0.987
2.849	2.707	2.082	1.867	1.751	1.541	1.5
3.25	3.165	2.084	1.968	1.919	1.868	1.834
2.78	2.712	1.958	1.942	1.77	1.688	1.499
2.647	2.526	1.999	1.848	1.77	1.43	1.178
2.647	2.626	2.028	1.867	1.836	1.579	1.576
3.344	3.244	2.106	1.974	1.95	1.926	1.804

2.77	2.647	2.004	1.99	1.841	1.789	1.713
2.797	2.732	1.968	1.916	1.866	1.708	1.518
2.816	2.646	2.098	1.942	1.682	1.635	1.487
2.529	2.285	2.019	1.824	1.603	1.459	1.455
2.041	1.874	1.873	1.786	1.541	1.369	1.342
2.567	2.413	2	1.943	1.591	1.488	1.463
3.005	2.906	2.066	1.958	1.891	1.65	1.594
2.085	1.923	1.905	1.703	1.641	1.518	1.209
2.627	2.268	2.015	1.831	1.669	1.277	0.996
2.518	2.465	1.986	1.879	1.592	1.149	1.098
2.797	2.647	2.004	1.943	1.848	1.668	1.485
2.647	2.518	1.938	1.912	1.648	1.316	1.167
2.914	2.736	2.072	1.876	1.795	1.676	1.505
2.684	2.636	1.974	1.91	1.607	1.569	1.494
2.453	2.099	1.987	1.756	1.626	1.427	1.426
2.217	2.158	1.94	1.91	1.507	1.118	0.996
2.532	2.452	1.969	1.8	1.661	1.47	1.43
0.004	0	1.832	1.117	1.05	0.261	0.192
2.797	2.797	2.037	1.934	1.817	1.677	1.519
2.498	2.252	2.018	1.836	1.564	1.149	0.996
0	0	1.704	1.018	0.6	0.348	0.123
3.199	3.103	2.085	1.872	1.835	1.808	1.726
2.279	2.052	2.025	1.74	1.591	1.521	1.28
3.135	3.018	2.099	1.897	1.844	1.746	1.595
1.57	1.559	1.97	1.589	1.416	1.272	1.062
2.626	2.547	1.974	1.901	1.706	1.446	1.311
2.188	1.723	1.892	1.767	1.605	1.294	1.228
2.466	2.414	1.97	1.861	1.549	1.42	1.159
2.906	2.826	1.994	1.932	1.882	1.557	1.493
3.001	2.843	2.037	1.946	1.784	1.78	1.669
2.885	2.824	2.037	1.941	1.926	1.741	1.557
2.823	2.578	2.026	1.976	1.73	1.556	1.398
2.649	2.647	1.974	1.893	1.758	1.543	1.377
1.729	1.423	2.096	1.821	1.453	1.325	0.85
2.654	2.355	2.086	1.848	1.645	1.4	1.308
2.414	2.041	1.987	1.748	1.561	1.149	1.117
2.949	2.837	2.022	1.987	1.936	1.863	1.736
2.674	2.647	2.007	1.918	1.764	1.515	1.382
2.545	2.476	1.975	1.912	1.649	1.416	1.149
2.924	2.752	2.03	1.857	1.792	1.697	1.645
1.649	1.138	1.801	1.798	1.498	1.494	1.346
1.278	0.929	1.8	1.671	1.496	1.369	0.761
2.825	2.645	2.027	1.831	1.722	1.548	1.411
2.63	2.416	2.009	1.83	1.698	1.285	1.042
2.518	2.466	2.003	1.882	1.6	1.224	1.149
1.917	1.58	1.892	1.605	1.484	1.128	1.04
2.713	2.445	1.968	1.956	1.684	1.552	1.301
2.647	2.49	2.026	1.87	1.608	1.426	1.197
1.603	1.348	1.832	1.636	1.453	1.374	0.863
2.676	2.645	2.077	1.886	1.638	1.611	1.336

2.355	2.089	1.981	1.819	1.476	1.459	1.4
2.652	2.563	1.987	1.794	1.752	1.515	1.422
2.674	2.469	2.046	1.882	1.63	1.541	1.407
2.545	2.302	1.982	1.858	1.662	1.238	1.017
2.868	2.647	2.032	1.888	1.777	1.669	1.526
2.586	2.404	2.087	1.713	1.597	1.573	1.315
2.323	2.129	1.984	1.744	1.598	1.403	1.135
2.543	2.507	2.018	1.807	1.639	1.405	1.272
2.523	2.485	1.996	1.965	1.74	1.341	1.269
2.548	2.25	1.992	1.911	1.454	1.4	1.391
0.539	0.399	1.795	1.459	1.076	0.808	0.168
0.91	0.298	1.801	1.552	1.132	1.117	0.931
2.647	2.647	2.037	1.848	1.795	1.657	1.42
2.734	2.45	1.978	1.882	1.738	1.45	1.381
2.769	2.754	2.075	1.966	1.812	1.644	1.511
2.802	2.713	2.082	1.86	1.737	1.591	1.477
2.557	2.369	1.957	1.835	1.571	1.459	1.411
2.533	2.414	1.96	1.824	1.645	1.574	1.282
1.357	1.127	1.91	1.672	1.478	1.111	0.959
2.652	2.533	1.999	1.891	1.685	1.584	1.261
3.255	3.051	2.079	2.002	1.944	1.705	1.638
2.646	2.351	2.087	1.852	1.646	1.391	1.296
2.754	2.728	2.032	1.9	1.706	1.492	1.392
2.591	2.414	1.996	1.767	1.651	1.543	1.388
2.566	2.414	2.029	1.862	1.681	1.597	1.291
2.892	2.618	2.1	1.904	1.743	1.654	1.487
2.296	1.891	2.022	1.681	1.543	1.289	1.081
2.837	2.62	2.093	1.861	1.69	1.427	1.411
2.708	2.551	2.091	1.844	1.711	1.497	1.392
2.419	2.219	2.098	1.734	1.486	1.299	1.149
2.414	2.414	2.018	1.748	1.65	1.147	1.117
2.923	2.781	2.087	1.906	1.872	1.71	1.503
2.799	2.519	2.058	1.876	1.592	1.508	1.469
2.541	2.322	2.04	1.896	1.831	1.483	1.093
2.789	2.627	2.052	1.892	1.651	1.534	1.504
1.735	1.719	1.824	1.729	1.56	1.468	1.122
1.478	1.317	1.96	1.609	1.144	1.064	0.875
2.671	2.401	2.045	1.86	1.619	1.54	1.272
2.676	2.539	1.993	1.934	1.729	1.445	1.427
3.018	2.947	2.032	1.974	1.934	1.745	1.636
2.676	2.645	2.077	1.886	1.638	1.611	1.336
2.742	2.611	1.982	1.968	1.688	1.424	1.413
2.518	2.386	1.983	1.95	1.706	1.467	1.445
2.746	2.647	1.95	1.904	1.752	1.66	1.445
2.54	2.291	1.988	1.864	1.663	1.238	1.018
2.685	2.587	2.09	1.857	1.648	1.438	1.382
2.647	2.647	1.969	1.901	1.807	1.55	1.41
3.146	3.112	2.111	2.025	1.99	1.818	1.685
2.311	1.838	1.962	1.7	1.522	1.416	1.122
1.867	1.328	1.949	1.63	1.534	1.065	0.996

2.647	2.538	2.041	1.849	1.783	1.299	1.272
2.405	1.813	2.002	1.763	1.516	1.122	1.098
2.647	2.647	1.966	1.898	1.806	1.549	1.4
2.526	2.399	2.005	1.848	1.533	1.444	0.996
2.414	2.213	1.979	1.823	1.646	1.588	1.172
2.892	2.803	2.072	1.955	1.91	1.704	1.66
2.797	2.633	1.983	1.913	1.903	1.549	1.364
2.647	2.516	2.051	1.892	1.695	1.645	1.524
2.385	2.091	1.965	1.645	1.592	1.405	1.148
2.444	2.414	1.952	1.822	1.635	1.561	1.191
2.85	2.811	2.04	1.843	1.8	1.692	1.589
2.797	2.709	2.097	1.899	1.632	1.539	1.52
2.793	2.704	2.07	1.853	1.736	1.508	1.458
2.806	2.691	2.078	1.857	1.737	1.515	1.483
2.399	2.093	1.956	1.835	1.572	1.459	1.282
2.343	2.09	1.949	1.83	1.526	1.459	1.279
2.742	2.647	1.979	1.919	1.808	1.63	1.55
2.505	2.414	2.087	1.828	1.634	1.516	1.217
2.414	1.548	1.995	1.69	1.51	1.117	1.117
2.505	2.414	2.087	1.828	1.634	1.516	1.217
2.32	1.755	2.015	1.789	1.522	1.264	1.017
2.826	2.797	1.98	1.908	1.858	1.687	1.476
2.72	2.696	2.107	2.011	1.625	1.525	1.4
2.72	2.696	2.107	2.011	1.625	1.525	1.4
2.461	2.414	1.89	1.79	1.676	1.551	1.465
2.988	2.797	2.022	1.97	1.846	1.744	1.62
1.463	0.905	1.94	1.523	1.362	0.979	0.905
1.675	1.409	1.966	1.786	1.391	1.188	0.919
2.84	2.797	2.01	1.978	1.888	1.7	1.509
2.477	2.414	2.006	1.806	1.675	1.588	1.427
3.157	3.116	2.088	1.948	1.908	1.781	1.645
2.71	2.486	1.995	1.922	1.649	1.451	1.4
0.9	0.839	1.935	1.332	1.076	0.827	0.461
2.647	2.528	1.966	1.896	1.605	1.455	1.406
2.988	2.797	2.07	1.917	1.807	1.77	1.632
2.414	2.146	2.006	1.833	1.746	1.471	1.117
2.797	2.764	2.015	1.879	1.706	1.637	1.518
1.832	1.777	2.025	1.713	1.628	1.342	1.066
2.647	2.54	2.036	1.948	1.699	1.465	1.402
2.694	2.647	1.966	1.941	1.893	1.737	1.4
2.427	1.852	2.027	1.75	1.636	1.422	1.154
2.647	2.621	1.972	1.901	1.748	1.509	1.424
2.752	2.415	2.031	1.808	1.695	1.655	1.473
2.616	2.414	2.031	1.837	1.797	1.601	1.357
2.281	2.141	1.975	1.656	1.513	1.384	1.149
2.844	2.645	2.038	1.941	1.793	1.625	1.581
2.549	2.514	1.937	1.835	1.712	1.57	1.398
2.578	2.488	1.991	1.876	1.686	1.373	1.124
2.188	2.083	1.97	1.81	1.67	1.449	1.12
2.531	2.38	2.013	1.896	1.544	1.356	1.339

2.797	2.71	2.097	1.901	1.668	1.597	1.53
2.414	1.993	1.967	1.823	1.506	1.124	1.117
2.699	2.523	2.073	1.833	1.687	1.508	1.445
2.804	2.636	2.099	1.943	1.68	1.634	1.502
1.656	0.577	1.89	1.592	1.412	1.23	0.916
2.312	1.861	1.957	1.692	1.501	1.414	1.122
2.269	2.03	1.96	1.822	1.468	1.31	1.099
2.414	2.414	1.887	1.858	1.678	1.414	1.272
2.701	2.647	1.953	1.946	1.784	1.572	1.405
2.647	2.589	2.029	1.804	1.768	1.669	1.327
2.535	2.024	2.029	1.803	1.683	1.446	1.224
2.525	2.436	2.025	1.838	1.675	1.41	1.289
2.777	2.722	1.985	1.852	1.831	1.685	1.384
2.667	2.537	1.995	1.884	1.675	1.559	1.23
2.557	2.45	2.005	1.875	1.45	1.441	1.378
2.851	2.797	1.998	1.923	1.83	1.636	1.421
1.458	1.26	1.864	1.619	1.424	1.069	0.958
2.901	2.854	2.033	1.907	1.794	1.751	1.516
3.146	3.106	2.09	2.025	1.93	1.811	1.685
2.847	2.832	2.007	1.97	1.809	1.72	1.516
3.301	3.17	2.057	2.023	1.974	1.908	1.776
2.834	2.713	2.081	1.979	1.689	1.651	1.571
2.647	2.536	2.027	1.918	1.693	1.52	1.186
2.642	2.63	2.062	1.871	1.654	1.564	1.447
2.264	2.077	1.905	1.794	1.563	1.432	1.307
2.797	2.754	1.986	1.937	1.85	1.563	1.515
2.647	2.608	2.031	1.957	1.759	1.529	1.38
2.463	2.311	1.943	1.799	1.683	1.421	1.162

BELv6	BELv7	BELv8	BEHe1	BEHe2	BEHe3	BEHe4
1.094	0.823	0.667	3.977	3.498	3.395	3.11
1.344	1.167	1.117	3.926	3.644	3.572	3.4
1.137	1.081	0.996	4.006	3.885	3.621	3.393
0.448	0.243	0.127	3.866	3.415	2.902	2.769
0.888	0.77	0.577	3.983	3.488	3.356	3.002
0.625	0.6	0.6	3.842	3.418	3.266	2.869
1.5	1.29	1.272	3.942	3.78	3.71	3.49
0.148	0	0	3.872	3.2	2.896	2.311
0.996	0.973	0.903	4.065	3.819	3.66	3.283
1.117	1.044	0.949	3.894	3.641	3.413	3.382
1.505	1.486	1.413	3.92	3.79	3.749	3.617
1.459	1.456	1.217	4.107	3.856	3.637	3.551
1.132	1.117	0.984	4.024	3.746	3.591	3.3
1.402	1.254	1.113	3.989	3.734	3.514	3.451
1.117	1.079	0.996	3.96	3.867	3.523	3.346
1.117	1.072	0.996	3.958	3.856	3.518	3.335
0.938	0.649	0.127	3.92	3.677	3.004	2.98
1.476	1.359	1.233	3.97	3.893	3.737	3.59
1.117	1.013	0.996	3.891	3.644	3.446	3.354
1.133	0.996	0.957	3.93	3.855	3.479	3.217
1.227	1.117	0.988	4.01	3.707	3.506	3.328
1.004	0.925	0.549	4.053	3.922	3.373	3.238
0.996	0.98	0.87	4.089	3.805	3.556	3.157
1.117	0.996	0.981	3.954	3.846	3.509	3.288
1.449	1.346	1.117	3.934	3.798	3.766	3.588
1.413	1.292	1.204	4.067	3.848	3.516	3.34
1.272	1.156	1.117	3.897	3.798	3.643	3.423
1.683	1.571	1.488	4.087	3.956	3.84	3.793
1.187	1.117	1.082	4.132	3.777	3.601	3.381
1.268	1.117	1.117	3.886	3.643	3.583	3.413
1.592	1.454	1.402	4.048	3.912	3.827	3.712
1.656	1.622	1.462	4.158	4	3.777	3.671
1.117	1.117	1.081	4.09	3.68	3.483	3.267
1.347	1.214	1.146	3.928	3.784	3.491	3.281
1.432	1.406	1.329	4.167	3.815	3.812	3.646
1.427	1.272	1.23	3.926	3.85	3.668	3.561
1.247	1.201	1.053	4.071	3.792	3.679	3.427
0.896	0.665	0	3.987	3.467	3.173	2.855
1.474	1.402	1.384	4	3.831	3.692	3.527
1.154	1.073	1.051	4.107	3.896	3.613	3.328
1.49	1.337	1.334	4.123	3.986	3.777	3.599
0.928	0.906	0.536	4.01	3.737	3.354	3.234
1.551	1.507	1.427	4.098	3.897	3.73	3.593
1.031	0.827	0.765	4.19	3.719	3.434	3.112
1.402	1.348	1.197	4.192	3.867	3.663	3.571
1.056	0.996	0.859	3.938	3.849	3.5	3.322
1.11	1.042	0.996	3.95	3.864	3.534	3.352
1.337	1.256	1.05	3.956	3.853	3.806	3.524
1.335	1.244	1.2	3.947	3.813	3.754	3.505

1.48	1.383	1.19	3.979	3.85	3.799	3.492
1.354	1.218	1.149	3.951	3.837	3.552	3.483
1.588	1.483	1.342	3.973	3.88	3.808	3.785
1.385	1.279	1.2	3.95	3.835	3.562	3.42
1.316	1.267	1.16	3.962	3.817	3.578	3.367
1.234	1.066	0.996	3.957	3.867	3.584	3.4
1.428	1.246	1.18	4.012	3.935	3.846	3.672
1.472	1.236	1.179	3.982	3.862	3.814	3.645
1.248	1.182	0.807	3.932	3.829	3.488	3.342
1.386	1.338	1.291	3.952	3.835	3.745	3.522
1.403	1.267	1.216	3.955	3.863	3.558	3.403
1.181	0.996	0.996	4.072	3.91	3.735	3.48
1.107	1.038	0.996	3.947	3.853	3.529	3.339
1.223	1.154	1.104	3.976	3.844	3.804	3.486
1.279	1.164	1.063	3.944	3.816	3.559	3.397
1.118	1.113	1.045	3.942	3.819	3.528	3.347
1.367	1.272	1.218	3.917	3.752	3.681	3.446
1.272	1.219	0.996	3.967	3.754	3.717	3.507
0.73	0.561	0.163	4.087	3.779	3.365	3.063
1.035	0.996	0.93	3.943	3.74	3.548	3.204
1.117	1.008	0.919	3.992	3.71	3.591	3.266
0.97	0.693	0.536	3.99	3.615	3.48	3.133
1.117	1.008	0.919	4.012	3.71	3.587	3.289
0.91	0.893	0.746	4.054	3.895	3.541	3.289
0.969	0.947	0.616	4	3.78	3.311	2.943
1.155	0.929	0.708	3.892	3.637	3.333	3.09
1.321	1.151	0.996	3.939	3.854	3.652	3.271
1.272	1.125	1.078	4.06	3.897	3.57	3.402
0.993	0.732	0.609	4.002	3.611	3.338	3.178
1.272	1.167	1.001	4.029	3.758	3.648	3.49
1.393	1.313	1.215	3.889	3.789	3.526	3.405
0.928	0.387	0.149	3.862	3.599	2.928	2.816
1.192	1.067	0.996	4.143	3.836	3.529	3.359
0.512	0.335	0.073	3.882	3.667	2.974	2.813
1.226	1.173	0.978	4.105	3.77	3.702	3.248
0.733	0.57	0.442	3.818	3.469	3.182	3.036
0.996	0.996	0.534	4.059	3.781	3.455	2.871
1.138	0.984	0.911	4.004	3.746	3.543	3.248
1.413	1.292	1.204	4.067	3.848	3.516	3.34
0.996	0.914	0.803	3.991	3.776	3.474	3.189
0.909	0.829	0.597	3.938	3.783	3.365	3.019
1.338	1.147	1.117	4.01	3.913	3.706	3.469
0.942	0.885	0.443	4.004	3.683	3.312	3.068
0.828	0.764	0.396	3.978	3.732	3.201	2.959
1.435	1.405	1.289	4.058	3.843	3.657	3.397
1.747	1.546	1.507	4.07	3.871	3.842	3.767
1.415	1.304	1.117	3.968	3.825	3.59	3.522
1.117	0.996	0.996	3.94	3.75	3.567	3.215
1.351	1.117	1.117	3.981	3.736	3.701	3.421
1.682	1.661	1.564	4.075	3.892	3.85	3.847

1.418	1.257	1.046	3.962	3.918	3.736	3.708
1.396	1.272	1.178	3.987	3.937	3.732	3.472
1.361	1.26	1.137	4.072	3.871	3.577	3.452
1.084	0.982	0.887	3.971	3.701	3.458	3.326
1.051	0.826	0.706	3.712	3.59	3.285	3.062
1.343	1.038	0.945	4.145	3.696	3.409	3.251
1.515	1.45	1.433	4.078	3.856	3.835	3.698
1.183	0.931	0.858	3.864	3.819	3.449	3.365
0.996	0.985	0.771	3.967	3.732	3.525	3.151
0.996	0.915	0.82	3.929	3.786	3.445	2.949
1.272	1.218	0.997	3.928	3.849	3.706	3.487
0.996	0.996	0.828	4	3.809	3.622	3.3
1.435	1.295	1.229	4.039	3.81	3.639	3.416
1.243	1.198	0.985	3.922	3.748	3.473	3.421
1.22	0.987	0.793	3.976	3.79	3.783	3.31
0.944	0.782	0.389	4.016	3.792	3.343	2.934
1.099	1.026	0.905	4.057	3.722	3.437	3.385
0	0	0	3.738	3.01	2.848	1.964
1.289	1.272	1.18	4.006	3.776	3.74	3.475
0.99	0.986	0.659	3.976	3.736	3.427	2.9
0	0	0	3.633	2.799	2.151	1.943
1.671	1.577	1.461	4.059	3.815	3.745	3.647
1.084	0.847	0.717	4.015	3.846	3.601	3.183
1.552	1.522	1.446	4.083	3.851	3.679	3.555
0.74	0.568	0.501	3.843	3.396	3.175	2.978
1.233	1.091	0.994	4.064	3.665	3.507	3.178
1.006	0.886	0.639	3.984	3.58	3.395	3.007
1.117	1.104	1.054	3.903	3.725	3.338	3.178
1.48	1.403	1.337	3.906	3.79	3.727	3.331
1.518	1.447	1.364	4.012	3.875	3.796	3.641
1.399	1.388	1.272	4.011	3.889	3.772	3.633
1.353	1.299	0.991	4.098	3.887	3.458	3.328
1.258	1.011	0.996	3.93	3.823	3.591	3.412
0.76	0.627	0.265	4.082	3.774	3.374	3.116
1.229	0.978	0.89	4.105	3.663	3.511	3.253
1.071	0.996	0.645	3.919	3.57	3.387	2.893
1.493	1.378	1.272	3.986	3.899	3.847	3.711
1.203	1.043	0.996	4.144	3.834	3.523	3.37
1.137	0.996	0.911	4.003	3.837	3.476	3.243
1.536	1.508	1.306	4.03	3.926	3.778	3.596
0.544	0.314	0.264	3.895	3.588	3.454	3.132
0.451	0.278	0.126	3.879	3.531	3.191	2.709
1.378	1.228	1.117	4.016	3.788	3.546	3.384
0.996	0.964	0.909	3.972	3.747	3.594	3.161
0.996	0.917	0.821	3.957	3.789	3.459	3.108
0.923	0.517	0.361	3.834	3.573	3.146	2.9
1.261	1.075	0.884	4.08	3.884	3.49	3.403
1.121	0.996	0.996	4.009	3.797	3.734	3.224
0.552	0.531	0.42	3.817	3.478	3.13	2.8
1.272	1.174	1.114	4.043	3.728	3.524	3.473

1.21	1.005	0.749	3.915	3.644	3.287	3.152
1.272	1.08	1.016	3.947	3.717	3.578	3.43
1.191	1.083	1	4.013	3.73	3.526	3.335
0.957	0.851	0.665	3.988	3.775	3.485	3.105
1.496	1.334	1.238	4.038	3.934	3.756	3.591
1.22	1.056	0.883	4.075	3.813	3.569	3.418
1.018	0.996	0.8	3.914	3.586	3.44	3.182
1.112	0.996	0.902	3.947	3.661	3.427	3.184
1.25	0.935	0.91	4.02	3.733	3.624	3.104
1.214	1.13	0.877	3.925	3.764	3.281	3.146
0	0	0	3.858	3.237	2.851	2.406
0.356	0	0	3.659	3.313	2.847	2.7
1.254	1.117	0.996	3.993	3.75	3.619	3.469
1.349	1.26	1.117	3.97	3.87	3.48	3.201
1.422	1.27	1.21	4.024	3.895	3.676	3.502
1.417	1.291	1.222	4.062	3.842	3.5	3.4
1.269	1.024	0.881	3.914	3.653	3.373	3.164
1.141	1.117	0.996	3.889	3.643	3.44	3.354
0.545	0.319	0	3.985	3.389	3.249	2.813
0.995	0.987	0.949	4.074	3.833	3.674	3.283
1.592	1.52	1.298	4.053	3.951	3.883	3.853
1.207	0.955	0.912	4.109	3.668	3.512	3.249
1.343	1.322	1.272	4.051	3.85	3.708	3.547
1.212	1.117	1.117	3.944	3.563	3.462	3.353
1.156	1.117	1.117	3.968	3.697	3.509	3.419
1.373	1.277	1.117	4.074	3.926	3.532	3.459
0.92	0.723	0.622	3.972	3.563	3.333	3.096
1.286	1.272	1.09	4.12	3.812	3.665	3.501
1.255	1.113	0.946	4.11	3.669	3.562	3.338
1.096	0.757	0.665	4.059	3.616	3.318	3.064
1.117	1.117	0.904	3.932	3.569	3.456	2.951
1.455	1.334	1.258	4.168	3.851	3.759	3.414
1.384	1.213	1.033	4.016	3.783	3.46	3.362
0.996	0.978	0.862	3.992	3.809	3.751	3.359
1.407	1.231	1.105	4.008	3.799	3.488	3.366
0.814	0.497	0.223	3.952	3.731	3.364	3.001
0.621	0.551	0.32	3.896	3.411	2.9	2.845
1.176	1.075	0.971	4.005	3.704	3.501	3.334
1.13	0.984	0.911	4.023	3.746	3.543	3.252
1.569	1.453	1.418	4.012	3.904	3.831	3.604
1.272	1.174	1.114	4.043	3.728	3.524	3.473
1.395	1.26	1.068	4.088	3.904	3.516	3.256
1.196	1.103	0.93	4.148	3.875	3.59	3.313
1.338	1.149	1.117	4.007	3.911	3.699	3.468
0.996	0.869	0.675	3.986	3.771	3.485	3.105
1.26	1.222	1.007	4.113	3.68	3.512	3.264
1.155	0.996	0.996	3.974	3.801	3.677	3.441
1.602	1.541	1.523	4.086	3.922	3.807	3.683
1.033	0.98	0.626	3.875	3.502	3.29	3.111
0.794	0.595	0.261	3.876	3.442	3.306	2.824

1.272	1.03	0.996	3.962	3.701	3.597	3.087
0.996	0.794	0.625	3.944	3.647	3.285	2.97
1.152	0.996	0.996	3.97	3.791	3.672	3.438
0.996	0.856	0.834	3.994	3.75	3.572	2.941
1.128	1.117	0.886	4.077	3.652	3.427	3.309
1.514	1.295	1.272	4.057	3.854	3.798	3.575
1.295	1.272	1.047	3.998	3.752	3.731	3.394
1.238	1.004	0.996	4.044	3.808	3.716	3.541
0.956	0.746	0.524	3.942	3.68	3.416	2.882
1.117	1.011	0.996	3.882	3.643	3.445	3.365
1.398	1.341	1.28	3.968	3.691	3.648	3.519
1.411	1.272	1.264	4.059	3.885	3.687	3.452
1.399	1.256	1.139	4.054	3.835	3.497	3.319
1.403	1.288	1.187	4.044	3.811	3.474	3.295
1.038	0.996	0.827	3.888	3.652	3.371	3.152
0.996	0.996	0.827	3.883	3.646	3.335	3.152
1.283	1.114	1.041	3.924	3.829	3.624	3.412
1.117	1.058	0.83	4.045	3.651	3.444	3.355
1.054	0.841	0.477	3.933	3.483	3.279	2.843
1.117	1.058	0.83	4.045	3.651	3.444	3.355
0.996	0.801	0.637	3.969	3.696	3.275	3.035
1.272	1.266	1.031	3.992	3.75	3.719	3.489
1.299	1.195	1.135	4.067	3.891	3.495	3.345
1.299	1.195	1.135	4.067	3.891	3.495	3.345
1.446	1.117	1.065	3.906	3.76	3.46	3.395
1.553	1.353	1.272	3.971	3.898	3.668	3.575
0.546	0.087	0	4.028	3.431	3.155	2.805
0.813	0.618	0.6	3.906	3.702	3.266	2.979
1.345	1.28	1.272	4.079	3.915	3.722	3.522
1.263	1.117	1.011	4.107	3.823	3.556	3.359
1.632	1.534	1.502	4.08	3.902	3.764	3.638
1.241	1.211	1.089	3.928	3.781	3.411	3.281
0.286	0.046	0	3.872	3.198	2.871	2.568
1.14	0.996	0.971	4.009	3.876	3.504	3.302
1.552	1.464	1.272	4.076	3.961	3.736	3.505
1.066	1.004	0.841	3.951	3.733	3.571	3.257
1.453	1.274	1.272	3.945	3.825	3.723	3.486
0.996	0.933	0.528	4.017	3.81	3.52	2.915
1.078	0.996	0.996	4.05	3.909	3.677	3.496
1.381	0.996	0.996	3.979	3.864	3.791	3.651
1.058	0.996	0.727	4.025	3.837	3.523	2.949
1.183	1.009	0.996	4.054	3.861	3.522	3.4
1.354	1.259	0.981	4.008	3.805	3.608	3.314
1.225	1.117	0.996	4.112	3.828	3.551	3.324
0.996	0.901	0.565	3.948	3.763	3.456	3.009
1.495	1.38	1.072	4.012	3.869	3.646	3.49
1.354	1.119	0.978	3.993	3.665	3.514	3.408
0.996	0.916	0.818	3.995	3.776	3.502	3.197
1.067	0.96	0.79	4.068	3.658	3.452	3.213
1.128	0.957	0.879	4.073	3.817	3.502	3.402

1.411	1.272	1.265	4.059	3.808	3.648	3.449
1.117	1.019	0.786	3.903	3.654	3.308	2.938
1.288	1.187	1.079	4.034	3.797	3.41	3.299
1.351	1.233	1.128	4.073	3.872	3.573	3.45
0.761	0.118	0	3.962	3.428	3.164	2.76
1.017	0.941	0.605	3.872	3.495	3.275	3.111
0.962	0.914	0.604	3.948	3.824	3.352	3.025
1.205	1.117	1.117	3.86	3.69	3.548	3.411
1.315	1.179	0.996	3.898	3.863	3.603	3.38
1.157	0.996	0.992	4	3.744	3.603	3.287
1.075	0.996	0.758	3.999	3.645	3.542	3.239
1.268	1.117	0.943	3.955	3.7	3.446	3.169
1.272	1.114	1.062	3.923	3.872	3.671	3.447
0.973	0.958	0.903	4.066	3.825	3.66	3.283
1.265	1.14	0.944	3.956	3.787	3.24	3.201
1.402	1.311	1.272	3.921	3.823	3.676	3.428
0.574	0.451	0.354	3.717	3.361	3.203	2.762
1.499	1.346	1.306	4.018	3.829	3.719	3.584
1.602	1.539	1.506	4.077	3.893	3.769	3.664
1.414	1.409	1.407	3.966	3.896	3.661	3.542
1.715	1.593	1.571	4.191	4.079	3.854	3.803
1.454	1.167	1.098	4.195	3.793	3.557	3.421
1.082	0.996	0.897	3.99	3.834	3.56	3.39
1.156	1.006	0.961	4.111	3.84	3.507	3.327
1.064	0.866	0.848	3.886	3.692	3.316	3.178
1.303	1.272	1.057	4.05	3.966	3.707	3.444
1.265	1.117	1.067	4.132	3.825	3.585	3.386
1.036	0.996	0.77	4.02	3.806	3.38	3.078

BEHe5	BEHe6	BEHe7	BEHe8	BELe1	BELe2	BELe3
2.981	2.774	2.514	2.098	1.682	1.594	1.355
3.317	3.12	2.897	2.704	1.873	1.595	1.542
3.328	2.941	2.875	2.769	1.943	1.85	1.565
2.427	2.082	1.798	1.515	1.832	1.325	0.831
2.788	2.511	2.376	2.07	1.66	1.438	1.272
2.463	2.151	2.151	2.129	1.742	1.371	1.021
3.367	3.228	3.085	2.978	1.878	1.735	1.484
2.26	1.505	1.109	0.983	1.653	1.156	0.8
2.942	2.78	2.769	2.66	1.928	1.793	1.496
2.939	2.804	2.7	2.676	1.867	1.594	1.421
3.332	3.291	3.263	3.189	1.8	1.701	1.678
3.351	3.222	3.152	3.033	1.915	1.821	1.632
3.239	2.894	2.778	2.7	1.94	1.857	1.555
3.314	3.153	3.024	2.885	1.938	1.706	1.43
3.176	2.873	2.769	2.7	1.864	1.706	1.594
3.176	2.833	2.769	2.7	1.865	1.706	1.594
2.769	2.705	2.219	1.93	1.832	1.533	1.226
3.376	3.294	3.032	3.019	1.901	1.868	1.588
2.929	2.769	2.702	2.7	1.861	1.595	1.394
2.992	2.981	2.87	2.769	1.871	1.706	1.493
3.101	2.881	2.827	2.7	1.899	1.627	1.469
2.831	2.763	2.333	2.233	1.773	1.512	1.373
2.872	2.797	2.769	2.698	1.9	1.843	1.409
3.175	2.776	2.769	2.7	1.859	1.706	1.593
3.407	3.161	2.866	2.769	1.887	1.805	1.621
3.3	3.16	2.998	2.975	1.98	1.737	1.622
3.312	2.941	2.769	2.769	1.862	1.596	1.414
3.482	3.465	3.238	3.208	1.758	1.746	1.732
3.17	2.875	2.806	2.769	1.938	1.811	1.572
3.319	3.043	2.799	2.769	1.851	1.595	1.517
3.61	3.387	3.307	3.135	1.942	1.842	1.774
3.59	3.387	3.337	3.288	2.006	1.782	1.713
3.089	2.725	2.7	2.7	1.907	1.668	1.508
3.146	3.062	2.948	2.824	1.894	1.755	1.516
3.486	3.28	3.147	3.112	2.058	1.825	1.674
3.369	3.054	3.041	2.978	1.786	1.757	1.62
3.221	3.034	2.956	2.777	2.03	1.799	1.674
2.743	2.655	2.157	1.746	1.685	1.403	1.126
3.333	3.271	3.194	3.064	1.898	1.801	1.709
3.138	3.024	2.894	2.856	2.005	1.858	1.46
3.474	3.276	3.201	2.973	1.973	1.899	1.737
2.825	2.741	2.601	2.455	1.876	1.866	1.255
3.488	3.355	3.319	3.23	2.052	1.902	1.708
2.914	2.816	2.69	2.249	1.967	1.84	1.431
3.333	3.165	3.135	3.016	1.969	1.825	1.821
3.069	2.823	2.769	2.575	1.868	1.802	1.408
3.118	2.874	2.781	2.769	1.87	1.825	1.465
3.323	3.126	2.881	2.81	1.866	1.827	1.5
3.233	3.133	2.936	2.743	1.838	1.538	1.42

3.349	3.117	3.081	2.995	1.853	1.63	1.537
3.299	3.186	2.957	2.829	1.845	1.586	1.452
3.551	3.443	3.285	3.126	1.886	1.829	1.678
3.333	3.105	3.085	2.802	1.837	1.576	1.427
3.254	3.096	3.025	2.768	1.84	1.574	1.437
3.264	3.037	2.864	2.769	1.873	1.837	1.546
3.488	3.276	3.124	3.038	1.904	1.883	1.784
3.489	3.295	3.079	2.924	1.884	1.773	1.608
3.095	3.051	2.784	2.573	1.816	1.563	1.34
3.362	3.16	3.102	3.088	1.84	1.596	1.555
3.313	3.099	3.077	2.769	1.837	1.58	1.428
3.147	2.937	2.769	2.769	1.932	1.861	1.452
3.118	2.835	2.778	2.769	1.87	1.825	1.465
3.252	3.079	2.903	2.769	1.884	1.772	1.564
3.247	3.102	2.797	2.613	1.837	1.573	1.435
3.118	2.891	2.818	2.78	1.848	1.807	1.466
3.235	3.149	2.981	2.978	1.824	1.591	1.59
3.265	3.048	2.978	2.769	1.932	1.761	1.663
2.617	2.52	2.277	1.797	1.965	1.535	1.176
2.797	2.769	2.71	2.7	1.913	1.726	1.512
2.959	2.874	2.712	2.7	1.945	1.714	1.558
2.769	2.673	2.548	2.052	1.886	1.533	1.363
2.947	2.871	2.712	2.7	1.988	1.714	1.559
2.981	2.69	2.603	2.453	1.934	1.813	1.402
2.836	2.769	2.66	2.25	1.887	1.767	1.199
2.954	2.891	2.61	2.237	1.597	1.505	1.329
3.192	3.008	2.849	2.769	1.882	1.826	1.466
3.268	3.142	2.899	2.84	2.047	1.881	1.552
2.886	2.774	2.49	2.304	1.897	1.421	1.3
3.031	2.978	2.909	2.736	1.934	1.638	1.432
3.183	3.144	2.992	2.99	1.743	1.65	1.535
2.769	2.7	2.004	1.867	1.818	1.538	0.799
3.073	3.063	2.832	2.769	1.921	1.835	1.616
2.551	2.442	1.947	1.647	1.821	1.331	1.262
3.056	2.978	2.946	2.779	1.997	1.835	1.642
2.821	2.405	2.119	1.964	1.701	1.445	1.171
2.769	2.769	2.714	2.175	1.969	1.779	1.293
3.193	2.886	2.778	2.635	1.891	1.857	1.519
3.3	3.16	2.998	2.975	1.98	1.737	1.622
2.917	2.769	2.709	2.597	1.921	1.779	1.447
2.813	2.761	2.648	2.252	1.898	1.776	1.262
3.179	3.076	2.935	2.813	1.858	1.714	1.6
2.851	2.53	2.365	2.318	1.665	1.515	1.426
2.81	2.582	2.568	1.999	1.942	1.695	1.055
3.303	3.142	3.025	2.911	1.977	1.747	1.606
3.696	3.647	3.407	3.303	1.977	1.757	1.738
3.313	3.198	3.032	2.854	1.877	1.836	1.549
2.943	2.769	2.769	2.7	1.916	1.764	1.506
3.314	3.092	2.808	2.769	1.944	1.729	1.665
3.649	3.554	3.523	3.422	1.984	1.87	1.839

3.553	3.2	2.985	2.776	1.885	1.85	1.579
3.325	3.214	2.978	2.931	1.837	1.825	1.723
3.312	3.199	3.055	2.93	2.012	1.822	1.527
3.152	2.925	2.812	2.647	1.897	1.611	1.39
3.039	2.712	2.423	2.242	1.651	1.573	1.294
3.21	3.148	2.763	2.658	1.932	1.782	1.399
3.456	3.27	3.201	3.196	1.989	1.79	1.719
3.013	2.721	2.467	2.356	1.635	1.483	1.421
2.797	2.769	2.769	2.507	1.952	1.73	1.475
2.872	2.769	2.682	2.604	1.889	1.791	1.386
3.267	2.992	2.978	2.769	1.875	1.852	1.697
2.843	2.769	2.769	2.653	1.865	1.845	1.49
3.296	3.122	3.092	2.951	1.965	1.717	1.624
3.328	3.034	2.923	2.865	1.857	1.727	1.313
2.814	2.703	2.703	2.389	1.872	1.541	1.319
2.778	2.769	2.515	2.335	1.859	1.84	1.346
3.006	2.909	2.734	2.689	1.872	1.605	1.381
1.79	1.548	0.692	0.416	1.592	0.815	0.665
3.297	3.193	2.978	2.978	1.954	1.736	1.71
2.809	2.794	2.769	2.416	1.952	1.738	1.368
1.797	0.516	0.311	0.116	1.436	0.452	0
3.56	3.459	3.349	3.268	2.004	1.723	1.705
3.06	2.624	2.502	2.342	1.914	1.516	1.375
3.43	3.346	3.321	3.189	2.002	1.779	1.704
2.748	2.317	2.039	1.959	1.831	1.356	1.063
3.129	3.006	2.808	2.759	1.906	1.75	1.466
2.921	2.773	2.511	2.205	1.663	1.505	1.339
2.927	2.823	2.72	2.7	1.877	1.702	1.302
3.28	3.21	3.164	3.087	1.794	1.723	1.687
3.425	3.303	3.194	3.057	1.918	1.792	1.663
3.388	3.235	3.142	3.016	1.919	1.844	1.763
3.153	3.118	3.051	2.764	1.903	1.854	1.478
3.21	3.126	2.815	2.769	1.874	1.815	1.491
2.627	2.556	2.282	1.897	1.961	1.533	1.184
3.066	2.929	2.845	2.597	1.997	1.71	1.419
2.772	2.769	2.7	2.29	1.907	1.575	1.352
3.626	3.334	3.143	3.005	1.911	1.874	1.834
3.072	3.06	2.823	2.769	1.916	1.84	1.62
2.902	2.871	2.769	2.631	1.886	1.843	1.441
3.559	3.375	3.04	2.944	1.968	1.749	1.626
2.64	2.473	2.133	1.671	1.497	1.429	1.074
2.477	2.322	1.831	1.487	1.487	1.411	1.061
3.177	3.162	3.014	2.895	1.942	1.712	1.487
2.836	2.791	2.769	2.703	1.95	1.734	1.558
2.875	2.769	2.684	2.605	1.93	1.796	1.404
2.853	2.365	2.305	2	1.684	1.349	1.257
3.115	3.023	2.846	2.716	1.868	1.839	1.472
3.038	2.769	2.769	2.692	1.947	1.8	1.389
2.67	2.343	2.112	1.83	1.621	1.441	1.183
3.108	2.978	2.945	2.888	1.958	1.666	1.458

3.146	2.937	2.659	2.39	1.891	1.584	1.214
3.026	2.978	2.829	2.806	1.863	1.56	1.51
3.144	3.022	2.889	2.778	1.933	1.675	1.459
2.867	2.792	2.668	2.556	1.917	1.771	1.414
3.489	3.108	3.034	2.846	1.969	1.765	1.622
3.015	2.944	2.814	2.623	1.963	1.537	1.456
2.94	2.769	2.71	2.465	1.886	1.525	1.373
2.978	2.825	2.769	2.679	1.918	1.644	1.355
3.053	3.007	2.667	2.657	1.906	1.828	1.552
3.106	2.948	2.806	2.519	1.893	1.736	1.212
1.905	1.665	1.022	0.995	1.607	1.197	0.832
2.467	1.97	1.386	0.941	1.519	1.219	0.761
3.158	2.985	2.769	2.769	1.966	1.764	1.596
3.184	3.146	3.05	2.7	1.859	1.695	1.59
3.367	3.246	3.035	3.032	1.913	1.857	1.609
3.281	3.14	2.999	2.925	1.994	1.735	1.569
3.152	3.042	2.748	2.654	1.856	1.6	1.307
3.016	2.817	2.769	2.7	1.861	1.595	1.397
2.512	1.907	1.76	1.655	1.636	1.391	1.165
2.959	2.811	2.789	2.685	1.925	1.79	1.524
3.512	3.433	3.366	3.271	2.005	1.926	1.876
3.06	2.909	2.835	2.599	2.002	1.72	1.426
3.295	3.153	2.989	2.98	1.93	1.727	1.528
3.128	2.92	2.751	2.7	1.901	1.502	1.453
3.072	2.861	2.772	2.7	1.935	1.657	1.46
3.273	3.185	3.159	2.82	2.042	1.706	1.594
2.84	2.714	2.515	2.208	1.899	1.515	1.305
3.259	3.048	3.002	2.828	2.003	1.737	1.507
3.253	2.958	2.919	2.749	1.998	1.713	1.523
2.924	2.835	2.536	2.389	2.042	1.58	1.248
2.7	2.7	2.7	2.681	1.877	1.472	1.387
3.377	3.2	3.126	3.026	2.003	1.784	1.713
3.241	3.105	2.969	2.757	1.955	1.724	1.362
2.934	2.769	2.66	2.641	1.893	1.79	1.541
3.327	3.153	2.99	2.867	1.951	1.732	1.395
2.81	2.474	2.195	1.98	1.532	1.461	1.294
2.552	2.222	2.027	1.852	1.857	1.343	0.812
3.025	2.978	2.867	2.733	1.937	1.642	1.443
3.216	2.868	2.778	2.635	1.939	1.857	1.521
3.533	3.389	3.27	3.188	1.926	1.886	1.735
3.108	2.978	2.945	2.888	1.958	1.666	1.458
3.127	3.075	2.968	2.816	1.871	1.862	1.485
3.022	2.936	2.696	2.632	1.91	1.797	1.493
3.179	3.076	2.933	2.769	1.868	1.715	1.6
2.867	2.769	2.666	2.549	1.919	1.773	1.414
3.164	3.023	2.892	2.815	1.997	1.714	1.421
3.014	2.909	2.769	2.769	1.857	1.807	1.642
3.499	3.392	3.32	3.308	1.989	1.821	1.814
2.97	2.827	2.593	2.211	1.805	1.475	1.233
2.769	2.419	2.231	1.862	1.837	1.359	1.282

2.978	2.978	2.769	2.736	1.932	1.703	1.62
2.92	2.769	2.614	2.181	1.904	1.578	1.23
2.995	2.882	2.769	2.769	1.857	1.807	1.642
2.769	2.769	2.741	2.626	1.933	1.764	1.266
2.976	2.721	2.7	2.433	1.888	1.594	1.424
3.474	3.281	3.089	2.991	1.973	1.786	1.773
3.226	3.051	2.978	2.803	1.871	1.782	1.684
3.178	2.842	2.769	2.731	1.932	1.797	1.452
2.791	2.713	2.608	2.327	1.882	1.45	1.437
2.943	2.769	2.705	2.7	1.838	1.594	1.387
3.408	3.207	3.097	3.013	1.936	1.652	1.615
3.153	3.103	2.994	2.978	1.981	1.684	1.447
3.276	3.112	2.976	2.901	1.982	1.729	1.569
3.255	3.13	2.979	2.883	1.985	1.727	1.568
3.063	2.769	2.725	2.451	1.857	1.6	1.308
3.063	2.769	2.7	2.444	1.852	1.591	1.245
3.378	3.008	2.943	2.786	1.886	1.831	1.576
3.028	2.783	2.7	2.605	2.027	1.598	1.427
2.7	2.7	2.597	2.06	1.913	1.511	1.306
3.028	2.783	2.7	2.605	2.027	1.598	1.427
2.791	2.769	2.561	2.212	1.919	1.649	1.265
3.271	3.085	2.978	2.901	1.866	1.839	1.665
3.187	3.038	2.93	2.872	2.019	1.87	1.467
3.187	3.038	2.93	2.872	2.019	1.87	1.467
3.219	2.968	2.729	2.7	1.615	1.565	1.496
3.451	3.33	3.188	2.998	1.914	1.878	1.668
2.66	1.947	1.744	1.463	1.86	1.301	0.986
2.69	2.523	2.201	2.151	1.843	1.61	1.021
3.315	3.145	3.068	2.978	1.901	1.837	1.713
3.046	2.805	2.7	2.684	1.928	1.604	1.408
3.439	3.376	3.331	3.288	1.979	1.75	1.727
3.146	2.951	2.935	2.764	1.894	1.751	1.436
2.038	1.893	1.396	1.309	1.839	1.048	0.811
3.085	2.948	2.879	2.769	1.871	1.714	1.387
3.439	3.319	3.226	2.978	1.938	1.715	1.638
2.817	2.726	2.7	2.509	1.884	1.578	1.517
3.344	3.137	2.978	2.963	1.879	1.74	1.555
2.783	2.769	2.308	2.163	1.898	1.527	1.352
2.814	2.784	2.769	2.769	1.904	1.844	1.491
3.354	2.945	2.882	2.769	1.857	1.854	1.806
2.868	2.777	2.769	2.257	1.899	1.601	1.367
3.251	2.958	2.779	2.769	1.91	1.831	1.646
3.223	3.066	2.918	2.62	1.911	1.588	1.472
3.046	2.948	2.772	2.7	1.939	1.618	1.554
2.847	2.769	2.432	2.291	1.9	1.511	1.372
3.337	3.273	3.111	2.814	1.922	1.823	1.564
3.151	3.028	2.839	2.707	1.866	1.627	1.502
2.958	2.769	2.709	2.6	1.921	1.779	1.448
2.927	2.649	2.603	2.262	1.785	1.578	1.424
2.966	2.879	2.715	2.626	1.84	1.752	1.39

3.162	3.125	2.994	2.978	1.981	1.685	1.476
2.754	2.7	2.7	2.453	1.86	1.631	1.268
3.128	3.008	2.897	2.768	1.979	1.705	1.5
3.344	3.187	3.027	2.905	2.012	1.822	1.527
2.656	2.292	2.034	1.203	1.666	1.295	1.108
2.97	2.795	2.592	2.203	1.807	1.478	1.246
2.81	2.789	2.442	2.24	1.861	1.703	1.351
2.978	2.847	2.7	2.7	1.663	1.633	1.437
3.218	3.053	2.93	2.769	1.85	1.848	1.544
3.149	2.991	2.865	2.769	1.91	1.59	1.541
2.937	2.822	2.769	2.347	1.91	1.585	1.436
3.033	3.002	2.7	2.684	1.92	1.693	1.41
3.204	2.978	2.931	2.893	1.854	1.713	1.65
2.943	2.792	2.78	2.66	1.926	1.79	1.496
3.079	2.995	2.852	2.657	1.893	1.699	1.16
3.169	3.135	3.098	2.978	1.861	1.711	1.633
2.542	2.08	1.916	1.882	1.695	1.397	1.132
3.552	3.286	3.145	3.028	1.969	1.767	1.658
3.499	3.392	3.309	3.303	1.981	1.821	1.762
3.283	3.182	3.128	3.128	1.917	1.866	1.635
3.606	3.566	3.434	3.364	1.973	1.911	1.881
3.341	3.21	3.005	2.854	1.952	1.907	1.489
2.962	2.94	2.769	2.69	1.936	1.834	1.542
3.174	2.96	2.805	2.767	1.942	1.715	1.465
3.071	2.81	2.571	2.377	1.708	1.556	1.295
3.258	3.091	2.978	2.86	1.892	1.872	1.664
3.047	3.027	2.818	2.769	1.905	1.871	1.548
3.061	2.769	2.727	2.501	1.86	1.614	1.563

BELe4	BELe5	BELe6	BELe7	BELe8	BEHp1	BEHp2
1.125	0.826	0.732	0.366	0.28	3.852	3.341
1.378	1.273	1.057	0.838	0.761	3.842	3.457
1.324	1.092	0.851	0.816	0.799	3.925	3.798
0.799	0.318	0	0	0	3.784	3.184
0.996	0.635	0.518	0.285	0.134	3.765	3.212
0.793	0.275	0.049	0	0	4.089	3.466
1.433	1.377	1.161	1.019	0.981	3.831	3.64
0.466	0	0	0	0	3.687	2.902
1.322	1.069	0.799	0.765	0.655	3.959	3.743
1.375	0.879	0.832	0.761	0.676	3.828	3.456
1.555	1.26	1.217	1.176	1.089	3.74	3.596
1.585	1.36	1.198	1.195	1.012	4.081	3.967
1.277	1.271	0.933	0.804	0.761	3.991	3.705
1.373	1.26	1.094	0.984	0.795	3.924	3.651
1.307	1.006	0.799	0.761	0.717	3.866	3.801
1.306	1.006	0.799	0.761	0.706	3.864	3.798
0.878	0.799	0.707	0.294	0	3.809	3.555
1.36	1.274	1.088	1.074	1.021	3.885	3.82
1.287	0.841	0.799	0.761	0.669	3.821	3.458
1.386	1.107	0.799	0.74	0.674	3.838	3.757
1.219	1.171	0.975	0.761	0.713	3.883	3.584
1.28	0.756	0.688	0.641	0.294	3.9	3.692
1.18	0.799	0.786	0.729	0.681	3.976	3.767
1.264	1.001	0.799	0.761	0.701	3.862	3.794
1.534	1.452	1.213	1.065	0.839	3.85	3.745
1.323	1.27	1.161	1.007	0.966	3.997	3.774
1.361	1.179	1.019	0.816	0.799	3.824	3.702
1.632	1.569	1.495	1.337	1.255	3.994	3.869
1.392	1.165	0.927	0.85	0.799	4.054	3.722
1.384	1.287	1.017	0.799	0.764	3.811	3.458
1.622	1.535	1.317	1.193	1.064	4.017	3.797
1.63	1.542	1.471	1.468	1.19	4.1	3.854
1.292	1.002	0.867	0.761	0.761	4	3.53
1.21	1.18	1.069	0.976	0.835	3.857	3.647
1.495	1.251	1.206	1.15	1.061	4.095	3.736
1.474	1.452	1.181	1.019	0.964	3.812	3.66
1.418	1.239	1.014	0.936	0.802	4.004	3.698
0.967	0.709	0.535	0.306	0	3.774	3.179
1.583	1.34	1.289	1.182	1.157	3.941	3.755
1.433	1.173	0.922	0.865	0.816	4.03	3.807
1.447	1.257	1.155	1.073	1.014	4.011	3.919
1.194	0.783	0.746	0.711	0.303	3.947	3.697
1.527	1.472	1.362	1.323	1.21	4.041	3.826
1.066	0.877	0.726	0.672	0.515	4.127	3.66
1.499	1.256	1.122	1.064	0.995	4.128	3.777
1.238	0.928	0.799	0.699	0.516	3.842	3.782
1.271	0.953	0.799	0.799	0.698	3.851	3.792
1.384	1.229	0.999	0.923	0.799	3.86	3.794
1.367	1.143	0.995	0.935	0.807	3.866	3.745

1.452	1.313	1.264	1.051	0.915	3.868	3.802
1.278	1.263	1.084	0.936	0.892	3.857	3.798
1.512	1.435	1.357	1.248	1.008	3.877	3.809
1.268	1.263	1.051	0.947	0.921	3.851	3.794
1.34	1.129	0.974	0.935	0.928	3.895	3.822
1.324	1.185	0.927	0.799	0.713	3.862	3.8
1.523	1.401	1.117	0.902	0.896	3.951	3.854
1.508	1.381	1.264	0.972	0.91	3.874	3.805
1.264	1.051	0.931	0.908	0.411	3.839	3.777
1.398	1.265	1.052	1.035	0.975	3.861	3.799
1.32	1.121	1.037	0.935	0.894	3.836	3.713
1.413	1.165	0.901	0.799	0.799	4.009	3.875
1.27	0.953	0.799	0.798	0.687	3.849	3.79
1.399	1.334	0.967	0.906	0.853	3.899	3.842
1.341	1.265	0.949	0.932	0.75	3.893	3.816
1.288	0.953	0.875	0.814	0.751	3.837	3.747
1.519	1.269	1.21	1.029	1.019	3.916	3.643
1.383	1.189	1.029	1.019	0.799	3.906	3.712
0.93	0.536	0.443	0.096	0	4.089	3.765
1.201	0.82	0.799	0.761	0.708	3.884	3.664
1.239	0.94	0.872	0.761	0.732	3.966	3.673
1	0.799	0.642	0.425	0.05	3.986	3.497
1.277	0.946	0.88	0.761	0.732	4.02	3.673
1.189	0.97	0.702	0.627	0.492	4.026	3.857
1.115	0.799	0.753	0.524	0.354	3.918	3.719
1.217	1	0.851	0.599	0.364	3.738	3.419
1.205	1.108	0.994	0.952	0.799	3.855	3.782
1.413	1.266	0.998	0.909	0.865	4.028	3.842
1.068	0.803	0.735	0.369	0.294	3.89	3.384
1.091	1.019	1.019	0.882	0.698	3.932	3.633
1.416	1.265	1.147	1.003	0.888	3.742	3.652
0.761	0.749	0.715	0	0	3.789	3.44
1.255	1.057	0.963	0.808	0.799	4.061	3.79
0.764	0.754	0.123	0	0	3.814	3.456
1.195	1.019	1.009	0.951	0.792	4.023	3.729
0.971	0.756	0.341	0.141	0	3.908	3.303
0.812	0.799	0.799	0.562	0.167	4.087	3.72
1.271	1.156	0.902	0.804	0.713	3.936	3.705
1.323	1.27	1.161	1.007	0.966	3.997	3.774
1.146	0.89	0.799	0.721	0.612	3.925	3.723
0.974	0.764	0.718	0.602	0.174	3.875	3.745
1.499	1.156	1.018	0.829	0.764	3.914	3.832
1.055	0.836	0.577	0.506	0	3.796	3.507
0.882	0.774	0.478	0.414	0	3.925	3.648
1.343	1.301	1.218	1.076	1.019	3.989	3.76
1.692	1.636	1.6	1.345	1.308	3.995	3.764
1.438	1.235	1.14	1.011	0.849	3.925	3.764
1.163	0.971	0.799	0.799	0.761	3.885	3.702
1.383	1.351	1.03	0.799	0.779	3.92	3.658
1.815	1.591	1.463	1.446	1.351	3.976	3.821

1.532	1.48	1.183	0.965	0.799	3.909	3.87
1.491	1.22	1.108	1.019	0.92	3.876	3.833
1.375	1.27	1.135	0.951	0.817	4.003	3.774
1.195	1.074	0.821	0.665	0.448	3.87	3.522
1.047	1.005	0.604	0.393	0.274	3.531	3.429
1.253	1.171	1.074	0.856	0.614	4.069	3.584
1.476	1.351	1.24	1.101	1.092	4.003	3.758
1.278	0.981	0.831	0.525	0.367	3.834	3.758
1.031	0.799	0.799	0.754	0.442	3.929	3.669
0.879	0.799	0.731	0.702	0.623	3.856	3.732
1.451	1.281	1.019	0.965	0.799	3.837	3.79
1.175	0.968	0.799	0.799	0.583	3.994	3.773
1.507	1.314	1.217	1.019	0.95	3.969	3.71
1.273	1.204	1	0.91	0.729	3.828	3.607
1.165	1.155	0.943	0.775	0.53	3.851	3.563
0.941	0.799	0.759	0.53	0	3.96	3.738
1.231	1.2	0.858	0.708	0.628	3.923	3.599
0	0	0	0	0	3.508	2.706
1.456	1.252	1.057	1.019	0.931	3.973	3.688
0.836	0.799	0.774	0.726	0.396	3.932	3.676
0	0	0	0	0	3.83	2.338
1.63	1.544	1.476	1.369	1.232	4.004	3.746
1.276	0.992	0.753	0.541	0.302	4.003	3.696
1.592	1.372	1.346	1.285	1.212	4.021	3.774
1.019	0.7	0.242	0.049	0.039	3.75	3.213
1.185	1.008	0.996	0.799	0.72	3.989	3.579
0.997	0.796	0.621	0.517	0.147	3.766	3.354
1.207	0.806	0.769	0.761	0.716	3.84	3.612
1.279	1.201	1.156	1.092	1.021	3.731	3.617
1.526	1.402	1.277	1.189	1.093	3.999	3.754
1.502	1.294	1.122	1.06	1.019	3.999	3.824
1.318	1.15	1.132	1.054	0.741	3.986	3.759
1.284	1.13	0.982	0.799	0.768	3.853	3.771
1.006	0.57	0.478	0.104	0	4.084	3.758
1.21	1.057	0.968	0.748	0.628	4.03	3.569
0.821	0.799	0.761	0.725	0.32	3.871	3.456
1.658	1.562	1.261	1.113	1.019	3.893	3.815
1.247	1.058	0.961	0.815	0.799	4.059	3.793
1.184	0.959	0.799	0.729	0.703	3.926	3.789
1.536	1.452	1.306	1.221	1.037	3.961	3.794
0.963	0.925	0.11	0	0	3.674	3.12
0.942	0.217	0	0	0	3.669	3.091
1.329	1.097	1.031	0.857	0.846	3.936	3.669
1.069	0.799	0.784	0.751	0.601	3.936	3.686
1.05	0.799	0.731	0.704	0.624	3.907	3.739
0.787	0.724	0.473	0.062	0	3.665	3.462
1.239	1.073	1.002	0.893	0.657	3.985	3.79
1.164	0.999	0.965	0.799	0.799	3.93	3.752
1.019	0.453	0.101	0.042	0	3.639	3.32
1.423	1.039	1.019	0.854	0.769	3.936	3.541

1.195	1.18	0.965	0.639	0.371	3.852	3.441
1.267	1.113	1.019	0.774	0.701	3.833	3.495
1.306	1.164	0.9	0.783	0.676	3.913	3.554
1.068	0.79	0.782	0.655	0.27	3.926	3.73
1.49	1.278	1.155	1.022	1.008	3.964	3.789
1.312	1.108	0.925	0.789	0.605	4.056	3.812
1.171	0.849	0.799	0.541	0.414	3.845	3.407
1.159	1.019	0.799	0.785	0.64	3.88	3.537
1.093	1.064	1.027	0.71	0.635	3.941	3.654
1.18	1.1	0.976	0.803	0.537	3.856	3.623
0.584	0	0	0	0	3.699	3.054
0.757	0.367	0	0	0	3.379	3.014
1.447	1.168	1.013	0.799	0.799	3.947	3.702
1.101	1.099	1.011	0.95	0.761	3.872	3.792
1.432	1.229	1.136	0.91	0.9	3.87	3.798
1.405	1.25	1.161	0.995	0.956	3.999	3.766
1.195	1.076	1.004	0.708	0.606	3.839	3.452
1.312	0.979	0.799	0.794	0.761	3.82	3.458
0.686	0.516	0.088	0	0	3.745	3.065
1.377	1.114	0.776	0.766	0.672	3.972	3.751
1.557	1.486	1.422	1.364	1.144	3.998	3.911
1.205	1.048	0.952	0.741	0.589	4.037	3.586
1.256	1.128	1.11	1.031	1.019	3.947	3.717
1.274	1.085	0.954	0.761	0.761	3.872	3.335
1.393	1.053	0.804	0.761	0.761	3.902	3.535
1.461	1.278	1.134	0.995	0.761	4.029	3.841
1.042	0.748	0.56	0.446	0.218	3.86	3.434
1.243	1.215	1.051	1.019	0.857	4.044	3.716
1.304	1.204	1.006	0.876	0.648	4.033	3.575
1.044	0.907	0.726	0.556	0.405	4.023	3.506
0.851	0.761	0.761	0.761	0.685	3.824	3.302
1.535	1.241	1.147	1.097	0.984	4.094	3.763
1.117	1.092	1.069	0.969	0.712	3.932	3.658
1.08	0.874	0.799	0.67	0.562	3.905	3.756
1.288	1.121	1.084	0.962	0.794	3.928	3.666
1.171	0.736	0.378	0.014	0	3.739	3.448
0.789	0.377	0	0	0	3.82	3.203
1.306	1.019	0.885	0.794	0.601	3.912	3.523
1.271	1.201	0.907	0.804	0.713	3.991	3.705
1.606	1.453	1.294	1.121	1.093	3.919	3.844
1.423	1.039	1.019	0.854	0.769	3.936	3.541
1.199	1.09	1.059	0.972	0.797	3.99	3.815
1.258	1.193	1.019	0.866	0.716	4.067	3.682
1.5	1.156	1.018	0.831	0.799	3.9	3.827
1.068	0.799	0.789	0.662	0.27	3.921	3.72
1.216	1.109	0.982	0.932	0.746	4.034	3.577
1.336	1.135	0.863	0.799	0.799	3.853	3.743
1.662	1.482	1.369	1.307	1.266	3.995	3.783
1.15	0.737	0.704	0.638	0.187	3.743	3.325
0.799	0.796	0.351	0.219	0	3.791	3.205

1.088	1.019	1.019	0.799	0.746	3.889	3.599
0.857	0.799	0.737	0.527	0.185	3.87	3.487
1.336	1.133	0.861	0.799	0.799	3.851	3.74
1.181	0.799	0.799	0.652	0.595	3.925	3.702
1.367	0.958	0.83	0.761	0.583	3.99	3.461
1.503	1.443	1.251	1.035	1.019	4.049	3.829
1.302	1.119	1.019	1.009	0.811	3.888	3.653
1.412	1.273	0.938	0.799	0.743	4.012	3.752
1.158	0.782	0.727	0.543	0.109	3.875	3.564
1.313	0.878	0.799	0.761	0.689	3.793	3.457
1.507	1.371	1.158	1.062	1.013	3.896	3.545
1.334	1.245	1.065	1.019	0.951	3.951	3.714
1.31	1.236	1.148	0.971	0.918	3.989	3.759
1.318	1.282	1.158	1.018	0.94	3.985	3.737
1.195	1.026	0.799	0.638	0.417	3.816	3.452
1.195	1.023	0.799	0.571	0.417	3.812	3.442
1.398	1.372	0.957	0.92	0.799	3.857	3.776
1.358	1.005	0.765	0.761	0.626	4.004	3.463
0.761	0.761	0.749	0.63	0	3.88	3.38
1.358	1.005	0.765	0.761	0.626	4.004	3.463
0.942	0.799	0.716	0.525	0.168	3.895	3.579
1.403	1.22	1.053	1.019	0.912	3.96	3.696
1.341	1.147	1.013	0.922	0.882	3.997	3.783
1.341	1.147	1.013	0.922	0.882	3.997	3.783
1.287	1.176	1.078	0.761	0.734	3.77	3.502
1.585	1.379	1.301	1.118	1.019	3.89	3.831
0.761	0.612	0.154	0	0	4.018	3.284
0.944	0.648	0.401	0.062	0	4.097	3.69
1.541	1.266	1.075	1.019	1.003	3.966	3.771
1.345	1.162	0.995	0.805	0.761	4.03	3.554
1.573	1.452	1.419	1.305	1.287	3.991	3.773
1.209	1.18	0.978	0.941	0.749	3.857	3.642
0.434	0	0	0	0	3.801	2.956
1.306	1.11	0.799	0.748	0.685	3.862	3.808
1.535	1.358	1.276	1.202	1.019	4.027	3.818
1.238	0.816	0.761	0.721	0.438	3.9	3.701
1.436	1.35	1.199	1.019	1.009	3.894	3.82
1.046	0.799	0.664	0.505	0.141	3.994	3.63
1.255	1.142	0.799	0.799	0.703	4.004	3.822
1.581	1.173	1.133	0.799	0.799	3.907	3.828
1.1	0.799	0.789	0.621	0.358	3.997	3.684
1.306	1.167	0.967	0.799	0.781	4.029	3.861
1.386	1.197	1.025	0.981	0.713	3.992	3.653
1.353	1.09	0.915	0.784	0.761	4.052	3.795
1.262	0.799	0.733	0.705	0.18	3.94	3.779
1.348	1.318	1.23	1.056	0.807	3.998	3.779
1.345	1.137	1.066	0.751	0.709	3.929	3.497
1.147	0.896	0.799	0.721	0.612	3.926	3.723
1.18	0.8	0.702	0.541	0.422	3.883	3.825
1.188	1.089	0.828	0.715	0.604	4.097	3.7

1.342	1.247	1.065	1.019	0.952	3.951	3.621
0.823	0.761	0.761	0.721	0.299	3.824	3.507
1.305	1.247	1.022	0.955	0.75	3.972	3.718
1.375	1.272	1.134	0.947	0.814	4.004	3.774
0.967	0.551	0.376	0	0	3.744	3.134
1.151	0.737	0.726	0.642	0.196	3.754	3.364
1.129	0.889	0.782	0.694	0.293	3.952	3.805
1.181	1.019	0.924	0.761	0.761	3.699	3.53
1.308	1.103	1.085	0.86	0.799	3.822	3.795
1.42	1.041	0.812	0.799	0.678	3.993	3.585
1.158	0.935	0.799	0.722	0.352	3.993	3.532
1.102	1.037	1.009	0.761	0.717	3.884	3.597
1.453	1.109	1.019	0.868	0.832	3.818	3.713
1.322	1.069	0.782	0.765	0.647	3.962	3.754
1.101	1.015	0.931	0.777	0.644	3.863	3.624
1.403	1.118	1.064	1.032	1.019	3.814	3.622
0.65	0.602	0.104	0	0	3.596	3.218
1.563	1.26	1.221	1.054	0.989	3.957	3.708
1.649	1.482	1.363	1.303	1.262	3.99	3.767
1.508	1.286	1.129	1.057	1.052	3.897	3.822
1.789	1.593	1.57	1.341	1.325	4.113	3.997
1.461	1.33	1.227	0.943	0.887	4.109	3.718
1.323	0.954	0.844	0.799	0.637	3.917	3.782
1.391	1.177	0.9	0.792	0.776	3.963	3.733
1.046	0.965	0.67	0.511	0.46	3.69	3.512
1.329	1.304	1.124	1.019	0.911	4.032	3.907
1.328	1.154	1.027	0.799	0.781	4.004	3.768
1.112	0.945	0.799	0.704	0.514	3.92	3.746

BEHp3	BEHp4	BEHp5	BEHp6	BEHp7	BEHp8	BELp1
3.223	2.815	2.681	2.517	2.166	1.756	1.794
3.414	3.223	3.146	2.889	2.655	2.543	1.977
3.484	3.225	2.967	2.742	2.66	2.644	2.046
2.699	2.66	2.029	1.511	1.257	1.121	1.938
3.082	2.706	2.424	2.179	1.953	1.708	1.909
3.285	2.583	1.946	1.627	1.416	1.416	1.784
3.432	3.306	3.168	2.968	2.817	2.813	2.014
2.604	2.022	1.839	0.966	0.568	0.459	1.843
3.518	3.126	2.813	2.69	2.66	2.542	1.998
3.302	3.221	2.728	2.69	2.455	2.447	1.955
3.58	3.435	3.101	3.064	3.006	2.917	1.998
3.564	3.457	3.439	3.298	3.079	3.009	1.967
3.436	3.152	3.132	2.757	2.687	2.549	1.986
3.316	3.205	3.107	2.9	2.827	2.716	2.022
3.35	3.16	2.909	2.66	2.602	2.447	1.971
3.349	3.154	2.909	2.66	2.58	2.447	1.967
2.807	2.728	2.66	2.547	1.905	1.495	1.931
3.514	3.334	3.189	3.015	2.889	2.724	2.009
3.25	3.158	2.662	2.66	2.447	2.444	1.953
3.297	3.036	2.849	2.759	2.66	2.538	1.957
3.299	3.117	2.942	2.697	2.583	2.447	2.024
3.008	2.881	2.739	2.446	2.131	2.11	1.94
3.385	2.997	2.692	2.66	2.603	2.499	1.983
3.343	3.115	2.909	2.66	2.579	2.447	1.957
3.617	3.443	3.263	2.987	2.688	2.66	1.99
3.372	3.207	3.101	3.014	2.842	2.781	2.066
3.458	3.243	3.136	2.687	2.66	2.507	1.953
3.75	3.701	3.286	3.274	3.126	3.061	1.874
3.388	3.212	2.99	2.744	2.66	2.596	2.002
3.373	3.231	3.118	2.876	2.66	2.512	1.949
3.706	3.625	3.473	3.21	3.134	2.867	2.036
3.651	3.535	3.48	3.325	3.15	3.113	2.072
3.365	3.13	2.915	2.562	2.447	2.447	1.977
3.364	3.077	3.025	2.857	2.813	2.603	1.992
3.714	3.555	3.322	3.156	2.975	2.86	2.138
3.506	3.382	3.209	2.84	2.827	2.817	1.984
3.59	3.271	3.101	2.872	2.773	2.623	2.119
2.897	2.615	2.465	2.331	1.942	1.326	1.923
3.579	3.421	3.214	3.14	3.036	2.917	1.982
3.493	3.199	2.959	2.832	2.733	2.685	2.078
3.681	3.293	3.269	3.087	2.997	2.868	2.042
3.168	3.063	2.649	2.628	2.529	2.28	1.971
3.631	3.419	3.362	3.235	3.2	3.067	2.126
3.301	2.951	2.776	2.605	2.502	2.138	2.01
3.595	3.347	3.107	2.994	2.908	2.862	2.013
3.333	3.148	2.917	2.66	2.569	2.397	1.989
3.369	3.169	2.919	2.66	2.66	2.574	2
3.559	3.346	3.14	2.922	2.827	2.66	1.996
3.497	3.351	2.995	2.939	2.876	2.578	1.99

3.638	3.343	3.159	2.904	2.865	2.76	2.011
3.417	3.23	3.097	2.945	2.894	2.466	1.993
3.573	3.56	3.379	3.282	3.084	2.923	2.006
3.384	3.189	3.113	2.919	2.764	2.627	1.994
3.39	3.086	2.971	2.917	2.724	2.563	2.006
3.456	3.218	3.069	2.916	2.66	2.591	2.003
3.738	3.62	3.342	2.996	2.916	2.862	2.045
3.702	3.466	3.348	3.131	2.903	2.767	2.035
3.316	3.139	2.917	2.76	2.461	2.343	1.982
3.542	3.362	3.173	2.975	2.914	2.798	1.99
3.371	3.137	2.97	2.915	2.761	2.569	1.994
3.621	3.39	2.953	2.66	2.66	2.636	2.014
3.368	3.163	2.919	2.66	2.656	2.554	1.999
3.702	3.346	3.072	2.902	2.763	2.66	2.035
3.378	3.151	3.058	2.916	2.626	2.342	1.992
3.368	3.181	2.92	2.726	2.651	2.596	1.997
3.6	3.246	3.096	3.005	2.817	2.757	1.942
3.529	3.246	3.066	2.913	2.817	2.66	2.021
3.286	2.829	2.503	2.369	1.755	1.41	2.02
3.363	3.063	2.662	2.66	2.599	2.447	1.995
3.409	3.061	2.985	2.752	2.617	2.473	2.01
3.345	2.869	2.66	2.495	2.331	1.641	1.94
3.435	3.142	2.987	2.754	2.617	2.473	2.01
3.477	3.117	2.843	2.6	2.462	2.19	1.984
3.147	2.798	2.692	2.66	2.35	2.012	1.967
3.219	2.834	2.779	2.67	2.357	2.003	1.798
3.447	3.069	2.939	2.835	2.66	2.653	1.972
3.436	3.304	3.14	2.906	2.785	2.695	2.093
3.143	2.909	2.704	2.471	2.144	2.081	2.019
3.491	3.34	2.817	2.779	2.71	2.39	2.044
3.403	3.259	3.125	2.917	2.773	2.756	1.934
2.725	2.66	2.521	2.447	1.548	1.31	1.917
3.38	3.154	2.892	2.816	2.676	2.66	2
2.803	2.764	2.4	2.074	1.579	1.484	1.9
3.509	3.081	2.914	2.817	2.731	2.669	2.086
3.074	3.055	2.515	2.167	1.863	1.585	1.833
3.39	2.66	2.66	2.657	2.411	1.811	2.004
3.371	3.142	2.959	2.72	2.687	2.549	1.985
3.372	3.207	3.101	3.014	2.842	2.781	2.066
3.296	3.014	2.771	2.66	2.596	2.508	1.987
3.15	2.855	2.764	2.6	2.521	1.885	1.983
3.586	3.303	2.913	2.822	2.764	2.745	1.94
3.047	2.824	2.541	2.235	2.117	1.928	1.895
2.982	2.797	2.658	2.357	2.247	1.424	2.009
3.515	3.253	3.179	2.992	2.862	2.728	2.069
3.671	3.585	3.529	3.503	3.258	3.179	2.071
3.407	3.319	3.109	2.998	2.809	2.76	1.948
3.34	3.031	2.777	2.66	2.66	2.536	1.991
3.536	3.273	3.171	2.845	2.66	2.625	2.024
3.788	3.744	3.472	3.37	3.343	3.245	2.102

3.703	3.67	3.365	3.039	2.778	2.66	1.973
3.598	3.327	3.101	3.001	2.817	2.734	1.964
3.462	3.235	3.157	3.019	2.818	2.647	2.097
3.266	2.977	2.938	2.729	2.512	2.262	2.03
3.107	2.816	2.777	2.306	2.063	1.907	1.866
3.277	3.085	2.999	2.946	2.578	2.419	1.995
3.635	3.53	3.209	3.044	3.002	2.91	2.062
3.398	3.108	2.929	2.5	2.096	1.929	1.812
3.369	2.951	2.66	2.66	2.629	2.276	2.006
3.278	2.773	2.66	2.579	2.519	2.482	1.98
3.585	3.312	3.132	2.817	2.81	2.66	2
3.599	3.071	2.81	2.66	2.66	2.565	1.93
3.495	3.289	3.169	2.93	2.923	2.749	2.062
3.181	3.136	3.081	2.874	2.695	2.633	1.974
3.484	3.126	2.658	2.512	2.458	2.106	1.99
3.225	2.777	2.66	2.65	2.208	2.163	1.934
3.192	3.141	2.781	2.733	2.532	2.469	1.973
2.492	1.316	1.187	0.897	0.009	0	1.851
3.579	3.304	3.093	3.054	2.817	2.809	2.024
3.269	2.739	2.66	2.658	2.503	2.261	2.011
1.416	1.246	1.133	0	0	0	1.525
3.718	3.545	3.417	3.334	3.223	3.134	2.074
3.514	2.95	2.872	2.45	2.294	2.065	1.977
3.596	3.442	3.238	3.195	3.14	3.033	2.087
2.88	2.817	2.445	1.921	1.615	1.587	1.955
3.302	3.012	2.874	2.839	2.643	2.568	1.968
3.109	2.712	2.539	2.418	2.179	1.72	1.912
3.147	3.056	2.674	2.522	2.494	2.447	1.962
3.576	3.111	3.046	2.943	2.909	2.83	1.992
3.689	3.559	3.197	3.105	3.024	2.847	1.981
3.654	3.556	3.179	3.005	2.908	2.839	1.983
3.282	3.148	2.997	2.953	2.831	2.584	2.02
3.353	3.191	2.978	2.903	2.66	2.658	1.97
3.29	2.872	2.515	2.389	1.756	1.467	2.02
3.322	3.125	2.89	2.726	2.664	2.369	2.079
3.229	2.66	2.656	2.501	2.447	2.067	1.977
3.779	3.52	3.466	3.148	2.952	2.834	2.027
3.391	3.135	2.896	2.816	2.685	2.66	2.01
3.314	3.072	2.774	2.66	2.539	2.49	1.97
3.679	3.448	3.299	3.2	2.931	2.763	2.024
2.792	2.711	2.277	2.251	1.652	1.168	1.837
2.748	2.299	2.273	1.79	1.299	0.98	1.836
3.354	3.226	2.911	2.873	2.832	2.645	2.028
3.487	2.995	2.721	2.66	2.635	2.415	2.001
3.304	2.965	2.66	2.581	2.52	2.484	1.996
2.908	2.649	2.568	2.099	1.926	1.596	1.908
3.472	3.115	2.978	2.867	2.717	2.491	1.962
3.485	3.104	2.893	2.66	2.66	2.477	2.024
2.856	2.549	2.35	2.008	1.619	1.386	1.832
3.375	3.312	2.86	2.817	2.682	2.644	2.084

3.064	3.025	2.977	2.795	2.367	2.116	1.976
3.38	3.178	2.817	2.81	2.658	2.558	1.99
3.385	3.143	2.98	2.782	2.671	2.471	2.053
3.284	2.951	2.729	2.716	2.557	2.299	1.977
3.646	3.362	3.287	2.962	2.881	2.66	2.027
3.481	3.308	2.814	2.73	2.606	2.444	2.03
3.264	3.055	2.742	2.66	2.329	2.162	1.978
3.177	2.998	2.817	2.66	2.563	2.507	2.01
3.448	2.979	2.894	2.825	2.536	2.488	1.996
3.077	3.025	2.879	2.812	2.562	2.271	1.989
2.763	2.198	1.773	1.211	0.648	0.442	1.799
2.518	2.447	1.92	1.469	0.926	0.38	1.819
3.476	3.303	3.001	2.808	2.66	2.66	2.029
3.319	2.962	2.909	2.903	2.747	2.469	1.981
3.504	3.317	3.11	2.995	2.763	2.733	2.095
3.359	3.275	3.083	2.999	2.815	2.728	2.072
3.153	2.977	2.926	2.838	2.583	2.368	1.956
3.248	3.157	2.772	2.66	2.547	2.447	1.953
2.909	2.466	2.105	1.563	1.338	1.165	1.938
3.581	3.127	2.843	2.701	2.658	2.542	1.998
3.841	3.795	3.462	3.336	3.272	3.065	2.078
3.328	3.132	2.889	2.698	2.654	2.366	2.081
3.611	3.407	3.127	2.884	2.761	2.718	2.04
3.321	3.131	2.883	2.76	2.602	2.447	1.991
3.344	3.26	2.915	2.609	2.574	2.447	2.022
3.401	3.304	3.101	3.025	2.908	2.621	2.096
3.139	2.912	2.585	2.384	2.294	1.904	2.03
3.591	3.363	3.128	2.881	2.845	2.627	2.088
3.413	3.203	3.124	2.815	2.709	2.556	2.086
3.138	2.912	2.711	2.505	2.432	2.234	2.092
3.218	2.718	2.542	2.447	2.447	2.447	2.019
3.621	3.305	3.191	2.988	2.934	2.794	2.084
3.277	2.948	2.923	2.848	2.807	2.533	2.064
3.677	2.956	2.761	2.66	2.55	2.315	2.033
3.292	3.122	2.977	2.884	2.796	2.632	2.055
2.968	2.63	2.49	2.113	1.728	1.709	1.865
2.687	2.623	2.073	1.598	1.505	1.34	1.956
3.352	3.143	2.817	2.777	2.672	2.39	2.05
3.411	3.143	3.051	2.703	2.687	2.549	1.985
3.654	3.514	3.386	3.151	3.015	2.934	2.035
3.375	3.312	2.86	2.817	2.682	2.644	2.084
3.495	3.086	2.952	2.804	2.764	2.626	1.976
3.486	3.146	2.89	2.766	2.536	2.389	1.979
3.576	3.303	2.913	2.816	2.738	2.66	1.949
3.284	2.95	2.727	2.66	2.549	2.284	1.984
3.324	3.128	2.935	2.785	2.698	2.602	2.086
3.565	3.271	2.821	2.707	2.66	2.66	1.971
3.658	3.559	3.316	3.188	3.148	3.117	2.103
3.056	2.937	2.591	2.539	2.342	1.862	1.963
3.109	2.66	2.656	2.04	1.895	1.327	1.946

3.487	2.951	2.817	2.817	2.66	2.547	2.033
3.051	2.724	2.66	2.591	2.403	1.842	2.001
3.564	3.27	2.814	2.693	2.66	2.66	1.967
3.353	2.773	2.66	2.66	2.536	2.38	1.999
3.246	3.184	2.788	2.476	2.447	2.223	1.979
3.67	3.502	3.332	3.077	2.951	2.818	2.032
3.548	3.171	3.014	2.817	2.809	2.644	1.989
3.598	3.433	2.942	2.662	2.66	2.54	2.014
3.267	2.715	2.589	2.498	2.41	2.11	1.958
3.237	3.169	2.721	2.66	2.456	2.447	1.95
3.507	3.39	3.244	3.032	2.861	2.826	2.03
3.495	3.227	2.907	2.832	2.817	2.718	2.108
3.35	3.19	3.077	2.974	2.805	2.718	2.061
3.339	3.182	3.084	2.987	2.822	2.712	2.065
3.147	2.977	2.886	2.66	2.394	2.107	1.953
3.093	2.977	2.885	2.66	2.336	2.106	1.945
3.435	3.261	3.234	2.831	2.747	2.66	1.971
3.33	3.216	2.909	2.536	2.515	2.447	2.081
3.152	2.621	2.474	2.447	2.447	1.566	1.987
3.33	3.216	2.909	2.536	2.515	2.447	2.081
3.088	2.842	2.66	2.546	2.331	1.771	2.02
3.542	3.247	3.07	2.97	2.891	2.817	1.982
3.386	3.218	3.004	2.82	2.73	2.704	2.107
3.386	3.218	3.004	2.82	2.73	2.704	2.107
3.328	3.168	2.842	2.812	2.495	2.447	1.913
3.536	3.469	3.263	3.123	2.991	2.817	2.018
2.818	2.636	2.364	1.802	1.487	0.949	1.933
3.466	2.864	2.479	2.217	1.685	1.416	1.927
3.581	3.424	3.128	2.949	2.842	2.817	2.012
3.376	3.185	2.879	2.65	2.48	2.447	2.004
3.623	3.458	3.269	3.244	3.171	3.12	2.076
3.269	3.076	3.025	2.813	2.722	2.497	1.992
2.687	2.259	1.465	1.238	0.956	0.883	1.93
3.31	3.118	2.819	2.811	2.66	2.538	1.958
3.595	3.354	3.284	3.111	2.995	2.817	2.025
3.403	3.1	2.679	2.547	2.447	2.209	1.975
3.628	3.31	3.15	2.892	2.817	2.769	2.014
3.428	2.704	2.66	2.52	1.837	1.76	1.962
3.571	3.403	2.673	2.66	2.66	2.556	1.988
3.74	3.547	3.294	2.816	2.693	2.66	1.967
3.444	2.754	2.66	2.583	2.457	1.847	1.964
3.445	3.359	3.088	2.813	2.66	2.635	1.951
3.512	3.098	3.04	2.784	2.769	2.433	1.983
3.377	3.164	2.859	2.708	2.638	2.447	1.999
3.309	2.813	2.66	2.504	2.321	2.29	1.966
3.555	3.335	3.133	3.085	2.854	2.654	1.989
3.375	3.193	2.932	2.833	2.547	2.516	1.932
3.307	3.014	2.789	2.66	2.597	2.508	1.987
3.314	2.962	2.641	2.355	2.249	2.084	1.988
3.481	3.301	2.754	2.661	2.534	2.378	1.964

3.461	3.226	2.924	2.861	2.817	2.719	2.108
3.135	2.711	2.555	2.447	2.447	1.996	1.963
3.277	3.182	2.984	2.852	2.72	2.539	2.061
3.461	3.234	3.169	3.015	2.808	2.64	2.097
2.887	2.49	2.334	2.02	1.637	0.567	1.91
3.177	2.939	2.694	2.591	2.345	1.919	1.951
3.219	2.9	2.736	2.705	2.288	2.127	1.951
3.349	3.253	2.817	2.564	2.447	2.447	1.888
3.404	3.156	2.976	2.923	2.706	2.66	1.948
3.523	3.014	3.005	2.784	2.66	2.636	1.967
3.422	2.993	2.757	2.66	2.572	2.049	1.966
3.225	2.956	2.837	2.821	2.543	2.454	2.016
3.513	3.284	2.987	2.817	2.78	2.74	1.983
3.518	3.126	2.815	2.717	2.69	2.55	1.993
3.024	2.909	2.767	2.72	2.549	2.469	2.009
3.5	3.244	2.922	2.863	2.851	2.817	1.998
2.959	2.427	2.294	1.711	1.509	1.282	1.846
3.574	3.424	3.298	3.106	2.91	2.854	2.028
3.626	3.539	3.314	3.185	3.148	3.111	2.078
3.54	3.362	3.128	2.975	2.855	2.841	2.001
3.777	3.709	3.474	3.381	3.306	3.173	2.054
3.38	3.304	3.155	3.053	2.841	2.719	2.083
3.461	3.225	2.829	2.732	2.66	2.54	2.024
3.334	3.215	2.98	2.733	2.642	2.619	2.074
2.954	2.863	2.77	2.466	2.279	2.082	1.918
3.525	3.298	3.168	2.976	2.817	2.797	1.982
3.415	3.221	2.904	2.877	2.66	2.614	2.039
3.171	2.934	2.829	2.66	2.479	2.333	1.939

BELp2	BELp3	BELp4	BELp5	BELp6	BELp7	BELp8
1.787	1.567	1.418	1.204	1.064	0.806	0.634
1.816	1.729	1.596	1.49	1.332	1.146	1.068
1.966	1.745	1.536	1.472	1.111	1.08	0.966
1.581	1.11	0.966	0.768	0.426	0.219	0.058
1.733	1.553	1.304	1.026	0.911	0.742	0.563
1.526	1.178	1.127	0.813	0.583	0.583	0.582
1.872	1.723	1.682	1.62	1.496	1.285	1.236
1.518	1.071	0.852	0.179	0.106	0	0
1.89	1.679	1.565	1.237	0.966	0.95	0.895
1.812	1.601	1.548	1.171	1.068	1.012	0.929
1.926	1.886	1.771	1.539	1.496	1.483	1.411
1.892	1.819	1.764	1.541	1.432	1.422	1.196
1.922	1.764	1.457	1.42	1.104	1.068	0.956
1.815	1.685	1.638	1.506	1.379	1.241	1.098
1.9	1.713	1.544	1.34	1.089	1.068	0.966
1.896	1.712	1.534	1.339	1.08	1.068	0.966
1.694	1.506	1.161	0.966	0.912	0.627	0.104
1.96	1.788	1.604	1.511	1.502	1.39	1.211
1.814	1.624	1.536	1.166	1.068	0.992	0.966
1.88	1.638	1.597	1.387	1.127	0.966	0.913
1.791	1.703	1.458	1.436	1.215	1.068	0.964
1.837	1.707	1.592	1.085	1.014	0.87	0.545
1.916	1.613	1.416	1.138	0.97	0.966	0.846
1.894	1.711	1.479	1.327	1.068	0.973	0.966
1.878	1.818	1.714	1.617	1.438	1.327	1.081
1.858	1.758	1.509	1.486	1.398	1.265	1.17
1.814	1.647	1.579	1.354	1.236	1.134	1.068
1.829	1.826	1.746	1.713	1.684	1.57	1.484
1.933	1.788	1.632	1.35	1.146	1.068	1.066
1.814	1.754	1.607	1.526	1.248	1.089	1.068
1.973	1.911	1.788	1.683	1.58	1.38	1.378
1.943	1.876	1.805	1.701	1.631	1.614	1.454
1.862	1.684	1.485	1.25	1.07	1.068	1.068
1.915	1.685	1.449	1.367	1.319	1.18	1.113
1.934	1.813	1.688	1.426	1.41	1.377	1.305
1.94	1.809	1.651	1.617	1.417	1.236	1.221
1.924	1.791	1.613	1.408	1.227	1.177	1.023
1.694	1.417	1.226	1.017	0.897	0.667	0
1.912	1.845	1.736	1.511	1.452	1.38	1.36
1.986	1.645	1.551	1.424	1.142	1.063	1.033
1.979	1.832	1.698	1.575	1.475	1.359	1.349
1.919	1.511	1.364	1.03	0.904	0.876	0.524
2.001	1.837	1.73	1.634	1.526	1.486	1.41
1.947	1.628	1.255	1.138	1.009	0.798	0.735
1.946	1.934	1.762	1.527	1.379	1.339	1.181
1.923	1.614	1.455	1.203	1.064	0.966	0.834
1.936	1.66	1.499	1.291	1.093	1	0.966
1.924	1.72	1.634	1.453	1.364	1.255	1.009
1.791	1.589	1.535	1.385	1.317	1.244	1.198

1.867	1.746	1.617	1.509	1.451	1.346	1.175
1.785	1.633	1.558	1.429	1.351	1.192	1.103
1.952	1.906	1.721	1.667	1.582	1.466	1.367
1.777	1.657	1.517	1.452	1.349	1.269	1.183
1.736	1.621	1.511	1.42	1.292	1.257	1.1
1.938	1.703	1.552	1.422	1.201	1.075	0.966
1.975	1.919	1.667	1.571	1.433	1.226	1.168
1.939	1.824	1.684	1.542	1.425	1.229	1.167
1.763	1.544	1.452	1.348	1.223	1.168	0.783
1.858	1.767	1.576	1.453	1.35	1.328	1.274
1.785	1.65	1.511	1.495	1.39	1.258	1.227
1.918	1.647	1.592	1.381	1.159	0.966	0.966
1.93	1.656	1.49	1.291	1.084	0.999	0.966
1.935	1.69	1.533	1.413	1.218	1.1	1.057
1.73	1.623	1.51	1.411	1.269	1.103	1.008
1.913	1.658	1.5	1.291	1.095	1.081	1
1.817	1.742	1.654	1.449	1.281	1.236	1.147
1.868	1.825	1.669	1.446	1.236	1.202	0.966
1.673	1.356	1.183	0.789	0.693	0.528	0.114
1.821	1.741	1.387	1.068	1.002	0.966	0.91
1.777	1.771	1.479	1.118	1.068	0.95	0.887
1.758	1.548	1.266	0.966	0.939	0.652	0.488
1.774	1.765	1.455	1.102	1.068	0.95	0.887
1.873	1.601	1.431	1.146	0.882	0.868	0.71
1.866	1.424	1.313	0.966	0.965	0.924	0.588
1.731	1.573	1.457	1.302	1.105	0.904	0.677
1.908	1.703	1.497	1.337	1.319	1.149	0.966
1.957	1.733	1.543	1.428	1.286	1.099	1.053
1.63	1.529	1.43	1.138	0.983	0.701	0.619
1.854	1.6	1.333	1.236	1.236	1.173	0.999
1.792	1.763	1.607	1.473	1.328	1.285	1.195
1.719	1.158	1.068	0.966	0.91	0.369	0.141
1.925	1.768	1.492	1.371	1.177	1.054	0.966
1.56	1.554	0.969	0.871	0.467	0.283	0.041
1.939	1.84	1.417	1.236	1.205	1.167	0.955
1.65	1.359	1.128	1.011	0.676	0.505	0.359
1.867	1.437	1.133	0.966	0.966	0.961	0.497
1.922	1.73	1.426	1.42	1.115	0.956	0.88
1.858	1.758	1.509	1.486	1.398	1.265	1.17
1.869	1.673	1.347	1.088	0.966	0.883	0.777
1.834	1.511	1.194	0.955	0.871	0.802	0.559
1.887	1.728	1.647	1.458	1.34	1.153	1.068
1.712	1.692	1.333	1.114	0.939	0.903	0.398
1.802	1.334	1.089	0.972	0.81	0.762	0.374
1.853	1.734	1.517	1.48	1.443	1.381	1.254
1.965	1.914	1.857	1.823	1.731	1.525	1.485
1.935	1.759	1.685	1.487	1.395	1.271	1.068
1.836	1.763	1.414	1.15	1.068	0.966	0.966
1.859	1.83	1.573	1.544	1.338	1.068	1.068
1.97	1.944	1.918	1.793	1.681	1.656	1.558

1.951	1.778	1.667	1.632	1.39	1.226	1.021
1.911	1.858	1.706	1.539	1.4	1.236	1.169
1.942	1.679	1.634	1.474	1.355	1.254	1.135
1.829	1.617	1.473	1.422	1.082	0.99	0.887
1.771	1.516	1.341	1.317	1.04	0.789	0.657
1.941	1.574	1.464	1.444	1.34	1.021	0.926
1.959	1.89	1.645	1.586	1.518	1.434	1.431
1.714	1.572	1.469	1.158	1.117	0.916	0.797
1.821	1.659	1.282	0.966	0.966	0.966	0.753
1.868	1.584	1.139	1.088	0.966	0.887	0.792
1.933	1.836	1.653	1.462	1.236	1.204	0.975
1.894	1.632	1.313	1.112	0.966	0.966	0.798
1.871	1.783	1.655	1.48	1.417	1.27	1.204
1.9	1.618	1.574	1.494	1.219	1.187	0.999
1.766	1.653	1.444	1.444	1.233	0.972	0.794
1.902	1.509	1.117	0.966	0.924	0.775	0.376
1.808	1.66	1.474	1.427	1.079	1.012	0.895
1.135	1.064	0.262	0.184	0	0	0
1.932	1.81	1.662	1.502	1.296	1.236	1.173
1.825	1.557	1.138	0.972	0.968	0.966	0.634
0.981	0.583	0.326	0.072	0	0	0
1.837	1.823	1.778	1.707	1.647	1.537	1.434
1.688	1.535	1.511	1.283	1.076	0.819	0.675
1.886	1.83	1.733	1.585	1.531	1.501	1.418
1.575	1.414	1.236	1.04	0.703	0.519	0.439
1.895	1.7	1.429	1.286	1.214	1.054	0.968
1.772	1.612	1.305	1.213	0.995	0.909	0.631
1.846	1.53	1.389	1.159	1.068	1.068	1.023
1.929	1.873	1.549	1.487	1.463	1.395	1.326
1.93	1.769	1.758	1.648	1.498	1.403	1.34
1.933	1.91	1.683	1.553	1.371	1.344	1.236
1.974	1.748	1.546	1.377	1.323	1.28	0.971
1.885	1.757	1.543	1.367	1.274	1	0.966
1.684	1.379	1.256	0.815	0.732	0.582	0.219
1.845	1.637	1.375	1.288	1.213	0.947	0.874
1.727	1.546	1.139	1.068	1.035	0.966	0.606
1.986	1.934	1.858	1.743	1.491	1.364	1.236
1.911	1.761	1.515	1.365	1.188	1.024	0.966
1.903	1.649	1.414	1.136	1.124	0.966	0.885
1.853	1.78	1.691	1.617	1.548	1.52	1.357
1.834	1.512	1.511	1.339	0.509	0.272	0.228
1.664	1.512	1.362	0.743	0.408	0.239	0.063
1.829	1.708	1.541	1.407	1.386	1.213	1.084
1.82	1.69	1.287	1.032	0.966	0.933	0.878
1.871	1.591	1.221	1.136	0.966	0.889	0.792
1.585	1.453	1.11	1.044	0.883	0.494	0.326
1.953	1.639	1.562	1.271	1.22	1.071	0.875
1.863	1.63	1.443	1.212	1.137	0.966	0.966
1.614	1.439	1.376	0.826	0.526	0.499	0.375
1.885	1.625	1.617	1.321	1.236	1.159	1.112

1.812	1.471	1.422	1.367	1.175	0.983	0.71
1.791	1.751	1.502	1.442	1.236	1.062	1.007
1.881	1.628	1.529	1.377	1.194	1.076	0.997
1.851	1.671	1.236	1.002	0.925	0.824	0.652
1.887	1.761	1.662	1.575	1.487	1.359	1.213
1.709	1.569	1.497	1.271	1.16	1.05	0.827
1.739	1.586	1.359	1.132	1.003	0.966	0.756
1.797	1.625	1.386	1.236	1.091	0.966	0.879
1.962	1.733	1.322	1.246	1.22	0.911	0.881
1.901	1.45	1.367	1.367	1.18	1.099	0.843
1.475	1.081	0.755	0.068	0	0	0
1.555	1.131	1.068	0.916	0.35	0	0
1.836	1.786	1.638	1.391	1.224	1.068	0.966
1.888	1.703	1.434	1.379	1.321	1.27	1.068
1.966	1.809	1.647	1.519	1.424	1.278	1.218
1.853	1.726	1.569	1.46	1.398	1.265	1.188
1.828	1.564	1.422	1.393	1.256	1.009	0.85
1.814	1.63	1.556	1.255	1.114	1.068	0.966
1.689	1.467	1.125	0.939	0.522	0.263	0
1.891	1.684	1.581	1.259	0.972	0.97	0.922
1.99	1.932	1.684	1.619	1.575	1.495	1.267
1.849	1.639	1.366	1.275	1.193	0.926	0.898
1.888	1.714	1.486	1.377	1.322	1.312	1.236
1.76	1.632	1.529	1.365	1.176	1.068	1.068
1.853	1.668	1.582	1.271	1.135	1.068	1.068
1.913	1.71	1.639	1.468	1.367	1.28	1.068
1.675	1.529	1.274	1.07	0.925	0.71	0.582
1.857	1.68	1.403	1.39	1.269	1.236	1.071
1.839	1.701	1.475	1.372	1.241	1.1	0.925
1.727	1.475	1.274	1.138	1.071	0.726	0.635
1.751	1.64	1.145	1.068	1.068	1.068	0.878
1.907	1.873	1.704	1.494	1.44	1.313	1.245
1.885	1.592	1.537	1.458	1.354	1.184	1.017
1.885	1.722	1.488	1.084	0.966	0.944	0.834
1.903	1.642	1.534	1.52	1.381	1.21	1.089
1.747	1.574	1.476	1.135	0.83	0.494	0.212
1.603	1.14	1.05	0.866	0.596	0.507	0.273
1.86	1.614	1.529	1.236	1.186	1.067	0.966
1.922	1.717	1.42	1.407	1.1	0.956	0.88
1.97	1.933	1.729	1.639	1.571	1.451	1.4
1.885	1.625	1.617	1.321	1.236	1.159	1.112
1.968	1.638	1.407	1.395	1.37	1.218	1.025
1.95	1.713	1.466	1.44	1.182	1.077	0.9
1.907	1.729	1.648	1.459	1.34	1.153	1.068
1.858	1.673	1.236	1.003	0.966	0.843	0.663
1.855	1.639	1.417	1.359	1.243	1.2	0.979
1.893	1.788	1.534	1.435	1.129	0.966	0.966
2.023	1.981	1.803	1.674	1.591	1.523	1.502
1.686	1.507	1.379	1.145	1.017	0.947	0.589
1.622	1.512	1.048	0.966	0.764	0.564	0.247

1.834	1.761	1.279	1.236	1.236	0.996	0.966
1.766	1.499	1.145	1.092	0.966	0.78	0.583
1.888	1.787	1.534	1.422	1.126	0.966	0.966
1.836	1.559	1.449	0.966	0.966	0.843	0.806
1.814	1.643	1.573	1.153	1.112	1.068	0.874
1.939	1.852	1.68	1.633	1.494	1.237	1.236
1.913	1.904	1.546	1.373	1.281	1.236	1.025
1.875	1.617	1.602	1.51	1.235	0.996	0.966
1.641	1.571	1.393	1.135	0.922	0.717	0.468
1.813	1.624	1.547	1.174	1.068	0.991	0.966
1.832	1.785	1.676	1.575	1.383	1.319	1.251
1.902	1.644	1.54	1.511	1.391	1.248	1.236
1.847	1.724	1.489	1.44	1.38	1.231	1.115
1.842	1.719	1.493	1.458	1.374	1.252	1.151
1.827	1.566	1.422	1.272	1.025	0.966	0.796
1.822	1.516	1.422	1.271	0.987	0.966	0.795
1.909	1.797	1.609	1.532	1.264	1.099	1.019
1.819	1.617	1.498	1.201	1.068	1.034	0.814
1.663	1.481	1.068	1.068	1.048	0.81	0.438
1.819	1.617	1.498	1.201	1.068	1.034	0.814
1.786	1.502	1.268	1	0.966	0.771	0.607
1.884	1.851	1.676	1.464	1.261	1.236	0.97
2.005	1.616	1.508	1.382	1.273	1.172	1.105
2.005	1.616	1.508	1.382	1.273	1.172	1.105
1.788	1.644	1.56	1.447	1.435	1.068	1.025
1.967	1.83	1.723	1.61	1.538	1.35	1.236
1.506	1.381	0.938	0.904	0.47	0.004	0
1.617	1.178	1.124	0.903	0.799	0.583	0.583
1.975	1.885	1.683	1.516	1.338	1.268	1.236
1.801	1.694	1.586	1.444	1.246	1.068	0.989
1.95	1.901	1.768	1.622	1.617	1.513	1.479
1.912	1.62	1.449	1.367	1.212	1.177	1.062
1.341	1.066	0.806	0.431	0.266	0	0
1.889	1.599	1.433	1.389	1.133	0.966	0.951
1.91	1.809	1.767	1.583	1.535	1.418	1.236
1.777	1.657	1.446	1.068	1.043	0.984	0.79
1.869	1.688	1.614	1.503	1.401	1.237	1.236
1.674	1.614	1.313	1.036	0.966	0.905	0.496
1.936	1.651	1.475	1.352	1.05	0.966	0.966
1.904	1.882	1.718	1.422	1.315	0.966	0.966
1.728	1.611	1.376	1.13	1.03	0.966	0.703
1.892	1.73	1.467	1.361	1.159	0.99	0.966
1.795	1.663	1.574	1.454	1.32	1.241	0.954
1.801	1.723	1.594	1.33	1.174	1.068	0.963
1.646	1.498	1.304	1.135	0.966	0.851	0.542
1.936	1.76	1.561	1.533	1.464	1.353	1.036
1.832	1.709	1.568	1.391	1.334	1.118	0.942
1.871	1.693	1.359	1.11	0.966	0.885	0.798
1.789	1.552	1.383	1.12	1.064	0.917	0.758
1.866	1.5	1.329	1.268	1.058	0.929	0.866

1.903	1.661	1.59	1.526	1.391	1.248	1.236
1.812	1.491	1.123	1.068	1.068	1.004	0.77
1.82	1.667	1.481	1.422	1.254	1.156	1.046
1.944	1.675	1.633	1.496	1.344	1.221	1.123
1.609	1.399	1.224	0.913	0.776	0.07	0
1.67	1.472	1.377	1.145	0.969	0.897	0.557
1.807	1.413	1.281	1.025	0.929	0.87	0.552
1.85	1.665	1.42	1.236	1.148	1.068	1.068
1.94	1.776	1.562	1.409	1.281	1.164	0.966
1.807	1.742	1.595	1.305	1.1	0.966	0.948
1.789	1.618	1.4	1.191	1.024	0.966	0.718
1.827	1.663	1.39	1.257	1.243	1.068	0.909
1.855	1.822	1.685	1.388	1.236	1.109	1.064
1.881	1.676	1.565	1.237	0.95	0.927	0.894
1.888	1.434	1.429	1.355	1.26	1.126	0.924
1.937	1.825	1.623	1.406	1.379	1.298	1.236
1.588	1.41	1.05	0.909	0.522	0.387	0.325
1.905	1.784	1.728	1.542	1.485	1.358	1.34
2.023	1.919	1.795	1.674	1.59	1.521	1.482
1.965	1.801	1.709	1.491	1.393	1.388	1.385
2.021	1.971	1.904	1.773	1.711	1.588	1.558
1.974	1.683	1.632	1.552	1.426	1.16	1.079
1.908	1.681	1.515	1.161	1.081	0.966	0.882
1.879	1.647	1.573	1.418	1.167	0.981	0.946
1.81	1.563	1.445	1.301	1.062	0.879	0.823
1.926	1.838	1.57	1.499	1.269	1.236	1.011
1.95	1.759	1.523	1.362	1.233	1.068	1.056
1.8	1.671	1.416	1.149	1.01	0.966	0.737

GGI1	GGI2	GGI3	GGI4	GGI5	GGI6	GGI7
4	2	1.917	1.14	0.493	0.519	0.062
6	2.444	1.438	1.293	0.778	0.589	0.365
5.5	3.333	2.313	2.16	1.437	0.689	0.528
3	0.889	0.5	0.502	0.174	0.162	0
3	1.778	1.194	0.929	0.361	0.198	0.147
6	3.556	2	0.56	0.333	0	0
6	4.222	2.917	1.814	0.944	0.816	0.457
1.5	1.333	0.563	0.333	0	0	0
3.5	2.667	1.847	1.309	0.834	0.597	0.215
3.5	1.778	0.625	0.702	0.458	0.204	0.165
9.5	6.667	4.188	3.547	1.826	1.665	1.351
5.5	3.556	2.917	1.992	0.987	0.945	0.729
3	2.222	1.375	0.989	0.427	0.327	0.311
4.5	3.111	2.688	2.418	2.007	0.978	0.597
7	4.222	3.722	2.314	0.955	0.7	0.446
6	3.778	3.66	2.123	0.844	0.618	0.422
2.5	1.778	1.729	0.601	0.285	0.141	0.028
11.5	4.889	3.194	2.364	2.427	0.989	0.749
5	1.556	0.625	0.942	0.431	0.242	0.227
4	2.444	1.882	0.818	0.524	0.424	0.231
4.5	2.889	2.736	2.379	1.431	0.574	0.324
3.5	2.667	1.382	0.978	0.427	0.354	0.233
5	2.889	1.354	1.161	0.743	0.394	0.353
6	2.889	3.41	2.083	0.643	0.578	0.319
4	2	0.903	0.832	0.688	0.445	0.318
10	8.667	5.799	3.819	2.039	1.145	0.596
4.5	1.833	0.472	0.827	0.372	0.208	0.259
10.5	6.222	5.681	2.05	2.238	1.535	1.237
3.5	2.444	1.701	1.188	0.629	0.394	0.302
5	1.778	0.5	0.862	0.458	0.182	0.302
9.5	5.333	2.688	2.4	2.062	1.385	1.092
11.5	7.556	6.146	5.491	3.057	1.75	1.296
5.5	2.444	1.257	1.263	0.698	0.3	0.288
3.5	2.667	1.563	1.116	0.639	0.469	0.207
11.5	8.5	4.59	5.053	3.104	1.99	1.188
5.5	3.111	1.944	1.279	0.747	0.336	0.299
6	4.556	2.813	2.347	1.035	0.584	0.264
3	2.667	1.75	1.392	0.278	0	0
9	5.111	3.194	1.899	0.959	0.688	0.731
6	4.667	2.764	2.533	1.362	0.72	0.469
5	3.556	2.507	1.818	1.463	0.665	0.444
2.5	2	1.5	1.379	0.563	0.229	0.015
16	10.667	6.625	6.173	3.326	2.073	1.398
4.5	2.667	1.764	0.977	0.737	0.399	0.266
4	2.889	1.979	1.173	0.58	0.35	0.333
4.5	3.333	3.063	1.716	1.104	0.506	0.396
5.5	3.778	3.125	1.907	1.215	0.588	0.42
5.5	4.222	3.382	1.961	1.42	0.811	0.704
6	2.889	3.167	1.884	1.229	0.67	0.638

7.5	4.222	3.917	2.284	1.837	1.016	0.762
6.5	3.778	3.472	2.089	1.532	0.848	0.561
9	6.667	4.694	3.276	2.163	1.401	1.211
6.5	3.778	3.347	2.169	1.671	0.949	0.644
7	3.667	3.785	2.311	1.653	0.94	0.607
5.5	3.778	3.125	2.107	1.5	0.729	0.448
8.5	4.667	4.563	2.658	1.896	1.318	0.939
9	5.111	3.66	2.321	2.15	1.286	1.023
5	3.333	3.035	1.658	1.212	0.565	0.411
8	5.111	3.972	2.734	1.761	0.989	0.887
5.5	3.556	3.028	2.044	1.504	0.894	0.625
8.5	4.222	1.778	1.919	1.424	0.782	0.587
4.5	3.333	3.063	1.716	1.104	0.506	0.396
5	2.444	2.91	1.831	1.264	0.695	0.613
5.5	2.667	2.847	1.839	1.285	0.696	0.535
4.5	3.333	3.063	1.716	1.104	0.506	0.396
2.5	1.778	1.465	0.626	0.414	0.28	0.246
4.5	2.444	1.563	1.076	0.354	0.704	0.502
6.5	3.333	2.125	1.409	1.146	0.184	0.083
3.5	2	0.938	0.573	0.528	0.485	0.267
4	2.222	1.375	1.391	0.521	0.504	0.325
5	2.444	1.375	0.822	0.604	0.203	0.134
4	2.222	1.375	1.391	0.521	0.504	0.325
7	4.444	3.299	1.361	1.195	0.517	0.575
3.5	2.889	1.347	1.117	0.511	0.349	0.23
2.5	1.556	0.819	0.365	0.191	0.096	0.103
5	2.222	1.444	1.259	0.747	0.289	0.258
5.5	3.111	1.813	1.316	0.66	0.344	0.224
4	2.667	1.632	1.795	0.875	0.379	0.181
4.5	2.944	1.875	1.866	1.243	0.845	0.315
6.5	4	2.882	2.331	1.295	0.855	0.522
5	2.222	0.75	0.822	0.313	0.283	0.118
5.5	3.111	2.674	2.046	1.431	0.736	0.478
2.5	1.556	0.91	0.728	0.18	0.18	0.015
4.5	2.667	1.313	1.638	0.625	0.538	0.38
3	2	1	0.542	0.236	0.121	0.056
5	3.111	1.5	0.844	0.597	0.242	0.174
2	2	1.25	0.989	0.427	0.245	0.142
10	8.667	5.799	3.819	2.039	1.145	0.596
3.5	2.667	1.625	1.309	0.788	0.49	0.15
4	2.222	1.188	1.236	0.576	0.306	0.335
7.5	4.889	4.91	2.836	1.445	0.713	0.583
3	2.222	1.257	0.873	0.309	0.232	0.16
4.5	2.667	1.625	0.987	0.736	0.506	0.153
8.5	6.889	3.951	2.957	1.479	0.889	0.455
16	10.222	6.951	5.33	2.466	2.41	1.286
6	3.333	2	2.234	1.525	1.091	0.589
2.5	1.556	1	0.382	0.194	0.362	0.26
5.5	3.778	2.875	0.978	0.875	1.176	0.431
18	10.778	8.625	7.218	5.583	3.928	2.675

9.5	2.444	1.5	1.484	0.75	0.667	0.286
5.5	3.556	3.118	1.503	0.848	0.591	0.43
7.5	5.778	5.563	5.396	2.743	1.36	0.859
5.5	3.111	2.375	1.364	0.743	0.509	0.458
2.5	1.111	0.625	0.24	0.111	0.163	0.125
5	3.111	2.132	1.823	0.948	0.505	0.26
8.5	5.778	3.819	4.034	2.077	1.858	1.5
6.5	1.333	1.569	0.605	0.462	0.3	0.156
3.5	2.222	0.813	0.493	0.514	0.384	0.168
3.5	1.556	1.063	0.653	0.361	0.322	0.196
3.5	2.222	1.375	1.084	0.542	0.442	0.278
4.5	2.222	1.257	1.073	0.98	0.489	0.268
9.5	5.778	3.694	2.646	1.455	1.01	0.5
8.5	2.889	1.25	1.604	1.201	0.757	0.701
5.5	2.667	2.257	1.083	1.014	0.295	0.332
3	2.444	2.361	1.129	0.552	0.256	0.147
4.5	3.333	2.035	1.848	1.081	0.633	0.234
2.5	1.333	0.563	0.413	0	0	0
6.5	3.556	1.75	1.813	1.111	0.789	0.561
3.5	2.444	1.375	0.844	0.569	0.362	0.172
3.5	1.333	0	0	0	0	0
10.5	6.667	3.354	2.439	1.563	1.169	0.652
6	2.889	1.847	1.369	1.156	0.367	0.265
11.5	7.778	6.069	4.775	2.977	2.037	1.12
3.5	1.111	0.875	0.622	0.181	0.08	0
5.5	3.333	2.514	2.012	0.973	0.505	0.285
4	2.222	1.319	1.009	0.448	0.253	0.273
5.5	2.889	1.063	0.853	0.313	0.264	0.293
8.5	5.333	3.313	2.644	1.375	1.017	0.958
9	4.889	3.389	1.819	1.17	0.828	0.471
8	4	2.5	1.707	1.194	0.71	0.474
4.5	2.889	2.451	1.241	0.524	0.496	0.219
7.5	3.333	1.875	1.582	0.847	0.632	0.663
6.5	3.333	2.125	1.409	1.146	0.184	0.083
5	4.222	2.667	2.441	0.906	0.474	0.087
4	1.778	1	0.742	0.41	0.282	0.125
7.5	3.556	1.875	1.791	0.938	0.726	0.668
5.5	3.111	2.674	2.046	1.431	0.736	0.478
4	3.111	2.014	1.169	0.643	0.296	0.241
5.5	4	2.639	1.614	1.112	0.623	0.315
4	1.778	1.069	0.81	0.598	0.345	0.062
3	1.778	0.944	0.57	0.327	0.193	0
5.5	3.333	2.75	2.079	1.757	1.003	0.763
4.5	3.111	1.375	0.924	0.653	0.444	0.259
3.5	2.444	1.438	0.733	0.542	0.403	0.196
3.5	1.778	1.16	0.743	0.476	0.167	0.115
6.5	3.111	1.729	1.641	0.819	0.611	0.377
3.5	2.222	1.278	0.914	0.473	0.542	0.328
2.5	1.778	1.125	0.56	0.24	0	0
5.5	3.778	2.625	2.778	1.479	1.051	0.45

4	2	1.438	1.013	0.229	0.345	0.191
5	2.667	1.819	1.299	0.757	0.656	0.278
5.5	3.333	3	2.613	1.514	0.769	0.345
4.5	2.889	1.563	1.736	0.868	0.686	0.174
4.5	3.333	2.292	1.454	1.007	0.582	0.332
6	3.778	3.257	2.178	1.031	0.489	0.418
4	2.222	0.875	0.822	0.576	0.323	0.236
4	2.889	1.5	0.742	0.632	0.362	0.149
3	2.222	1.438	1.448	0.549	0.385	0.089
3.5	2.667	1.563	0.996	0.493	0.368	0.116
1.5	1.333	0.563	0.333	0	0	0
3	1.111	0.5	0.32	0	0	0
5	3.556	2.125	1.458	1.153	0.689	0.292
6.5	4	4.285	2.506	0.997	0.682	0.512
8.5	4.222	2.313	2.28	1.208	0.75	0.523
9	7.111	4.667	3.548	1.771	1.097	0.536
4.5	2.667	1.5	1.204	0.604	0.305	0.2
4	1.556	0.375	0.782	0.403	0.182	0.224
2.5	1.778	1.194	0.775	0.191	0	0
4	3.111	2.16	1.581	0.889	0.756	0.297
11	5	2.625	2.294	1.306	0.797	0.839
5	4.222	2.667	2.441	0.906	0.474	0.087
5.5	3.389	2.917	2.603	1.896	1.11	0.556
5.5	2.222	1.5	1.404	0.833	0.549	0.309
6.5	2.889	1.188	1.578	0.681	0.386	0.318
6.5	4.222	4.222	2.759	1.052	0.801	0.575
4	2.444	1.625	1.649	0.84	0.202	0.118
6.5	5.611	3.41	3.259	1.215	0.695	0.217
5.5	5.111	3.444	2.863	0.962	0.518	0.187
4	2.222	1	1.107	0.521	0.344	0.177
5.5	2.222	1.125	0.88	0.528	0.367	0.243
7.5	4.667	3.542	2.746	1.473	0.762	0.536
5	3.333	2.813	2.298	1.875	1.144	0.41
6	2	1.25	1.444	0.674	0.669	0.49
5.5	3.333	2.813	2.298	1.569	0.859	0.76
4.5	2	1.389	0.74	0.278	0.248	0.197
3	2	0.688	0.653	0.319	0.081	0
4.5	3.111	2	2.031	1.257	0.627	0.255
2	2	1.25	0.989	0.427	0.245	0.142
12	7.111	4.5	4.48	2.542	1.379	1.115
5.5	3.778	2.625	2.778	1.479	1.051	0.45
5.5	3.333	2.104	1.743	0.681	0.603	0.334
3	2.444	2.458	1.257	0.636	0.333	0.224
6.5	4.222	4.16	2.543	0.952	0.713	0.583
4	2.444	1.25	1.544	0.726	0.527	0.146
6	5.556	3.507	3.179	1.187	0.616	0.171
3.5	2.667	2.674	1.998	1.094	0.462	0.357
12.5	8	5.076	4.766	3.164	1.962	1.155
5	3.111	2.563	1.076	0.549	0.101	0.059
3	0.889	0.5	0.542	0.264	0.202	0.062

2.5	2.222	1.25	0.24	0.292	0.235	0.049
4.5	2.889	2.375	1.084	0.514	0.28	0.047
2.5	2.222	2.611	1.807	0.983	0.333	0.191
4	2.667	2.194	0.998	0.528	0.281	0.104
3.5	2	0.688	0.693	0.337	0.19	0.184
7	4.444	2	1.893	1.167	0.951	0.655
4.5	2.889	2.125	2.092	0.948	0.634	0.221
6	2.889	1.59	1.893	1.871	0.715	0.513
3.5	2.444	1.215	0.813	0.601	0.268	0.147
5	1.556	0.625	0.942	0.431	0.242	0.227
8	3.556	2.75	1.769	1.222	1.04	0.608
5.5	3.778	1.903	2.031	1.084	0.634	0.416
8.5	6.889	4.354	3.157	1.483	0.961	0.474
7	5.333	3.194	2.406	0.979	0.7	0.356
3.5	2	0.875	0.462	0.375	0.264	0.137
3.5	2	0.875	0.462	0.375	0.264	0.137
3.5	2.222	1.063	0.813	0.667	0.429	0.231
3.5	2	0.813	0.796	0.347	0.224	0.168
3	2	1.438	0.653	0.472	0	0
3.5	2	0.813	0.796	0.347	0.224	0.168
4	2	1.75	1.187	0.479	0.424	0.122
3.5	2.444	1.125	1.487	0.671	0.38	0.299
4.5	3.111	1.625	1.671	0.931	0.487	0.307
4.5	3.111	1.625	1.671	0.931	0.487	0.307
5	1.111	1.319	0.685	0.462	0.178	0.197
6.5	3.111	2.625	1.627	1.514	0.793	0.648
5.5	1.333	1.479	0.728	0.473	0.309	0.216
6.5	3.556	2.063	1.111	0.632	0.327	0.167
5.5	4	2.319	1.938	0.976	0.57	0.4
4	1.778	1.076	0.688	0.448	0.441	0.215
10	7.111	4.389	4.238	2.632	1.616	0.99
3.5	2.667	1.563	1.116	0.639	0.408	0.179
2	1.333	0.875	0.302	0.125	0	0
4	2.556	2.264	1.049	0.552	0.455	0.281
8	5.111	3.479	2.205	1.865	1.172	0.814
6	1.778	1.25	0.902	0.618	0.466	0.191
5	3.111	2.097	1.609	0.809	0.653	0.379
5.5	2.444	1.569	0.777	0.709	0.151	0.168
5	3.111	1.563	1.058	0.941	0.399	0.234
3.5	3.111	2.924	1.838	1.136	0.531	0.464
5.5	3.333	1.792	0.937	0.82	0.233	0.184
6	3.111	2.424	1.681	1.404	0.477	0.385
6	3.556	1.757	1.303	1.101	0.509	0.222
6	2.444	1.354	0.973	0.594	0.665	0.341
3.5	2.667	1.278	0.782	0.41	0.312	0.202
6.5	3.111	2.063	1.573	0.771	0.568	0.306
3.5	2	1.104	0.733	0.479	0.32	0.188
4	2.889	1.75	1.754	0.955	0.608	0.178
5.5	2.444	1.382	1.093	0.417	0.342	0.28
5	3.556	2.556	2.299	0.715	0.518	0.303

5.5	3.778	1.903	2.031	1.084	0.634	0.416
6	1.778	1.188	1.053	0.486	0.224	0.3
6.5	5.111	2.792	2.076	0.868	0.58	0.231
9	6.667	5.938	5.173	2.91	1.401	0.891
2.5	2.444	1.306	1.192	0.16	0	0
5	3.111	2.563	1.076	0.549	0.101	0.059
3	1.778	0.882	0.751	0.205	0.247	0.161
5	2.222	0.938	0.886	0.399	0.318	0.24
4.5	2.667	1	0.844	0.639	0.224	0.24
5.5	2.444	1.625	0.867	0.746	0.396	0.28
5	2.444	1.375	0.822	0.66	0.203	0.196
5	2.667	1.688	1.173	0.743	0.322	0.172
4.5	2.889	1.889	0.907	0.674	0.352	0.287
4	3.111	2.16	1.581	0.889	0.756	0.297
5	2.444	1.813	1.538	0.59	0.507	0.326
5	3.778	2.063	1.52	0.632	0.504	0.286
2	1.111	0.625	0.4	0.056	0	0
5.5	3.556	2.528	1.844	1.212	0.742	0.498
11	7.111	4.639	4.375	2.823	1.819	1.08
8	4.889	2.625	2.027	1.361	0.996	0.458
15.5	9.556	9.083	7.717	5.801	3.53	2.792
4.5	3.556	3.236	2.466	1.792	0.728	0.398
4	2.444	2	1.889	1.201	0.531	0.347
4	3.111	2.007	0.916	0.792	0.521	0.269
4.5	2.667	1.667	1.424	0.778	0.366	0.284
5.5	3.333	1.986	1.329	0.851	0.402	0.31
5.5	3.111	1.576	1.441	1.063	0.757	0.518
5	2.667	2.292	1.352	0.889	0.354	0.273

GGI8	GGI9	GGI10	JGI1	JGI2	JGI3	JGI4
0.049	0	0	0.235	0.087	0.083	0.05
0.251	0.196	0.091	0.25	0.079	0.046	0.042
0.349	0.149	0.083	0.196	0.083	0.053	0.049
0	0	0	0.273	0.063	0.045	0.063
0.082	0.016	0	0.176	0.077	0.054	0.049
0	0	0	0.462	0.169	0.087	0.029
0.223	0.216	0.218	0.188	0.096	0.06	0.036
0	0	0	0.136	0.089	0.047	0.048
0.074	0.016	0	0.14	0.074	0.046	0.034
0.132	0.084	0.058	0.194	0.081	0.03	0.037
0.842	0.702	0.508	0.226	0.109	0.057	0.052
0.383	0.243	0.103	0.167	0.079	0.058	0.041
0.17	0.063	0	0.13	0.072	0.039	0.031
0.23	0.121	0.037	0.155	0.08	0.057	0.045
0.317	0.251	0.12	0.259	0.106	0.091	0.059
0.285	0.201	0.103	0.231	0.099	0.094	0.056
0	0	0	0.167	0.085	0.086	0.038
0.753	0.678	0.464	0.287	0.081	0.054	0.04
0.132	0.116	0.074	0.263	0.065	0.03	0.05
0.22	0.069	0.029	0.174	0.076	0.059	0.033
0.057	0.016	0	0.188	0.08	0.067	0.058
0.103	0	0	0.167	0.089	0.049	0.039
0.17	0.12	0.074	0.217	0.088	0.042	0.045
0.235	0.201	0.103	0.24	0.08	0.097	0.06
0.256	0.158	0.096	0.138	0.054	0.027	0.024
0.304	0.167	0.066	0.323	0.164	0.083	0.06
0.14	0.102	0.078	0.196	0.071	0.02	0.039
0.726	0.866	0.623	0.228	0.093	0.081	0.031
0.156	0.097	0.037	0.146	0.074	0.052	0.037
0.214	0.111	0.115	0.217	0.063	0.02	0.039
0.797	0.418	0.33	0.226	0.089	0.042	0.038
0.751	0.546	0.511	0.235	0.096	0.067	0.057
0.191	0.134	0	0.275	0.084	0.048	0.055
0.155	0.066	0.021	0.159	0.092	0.05	0.04
0.669	0.29	0.1	0.287	0.127	0.053	0.057
0.154	0.133	0.141	0.177	0.072	0.046	0.035
0.073	0.063	0.025	0.214	0.108	0.056	0.051
0	0	0	0.2	0.116	0.07	0.073
0.565	0.321	0.19	0.281	0.106	0.065	0.043
0.294	0.141	0.058	0.2	0.099	0.051	0.055
0.276	0.231	0.198	0.135	0.068	0.046	0.032
0	0	0	0.125	0.071	0.045	0.044
0.854	0.662	0.4	0.356	0.14	0.07	0.067
0.13	0.089	0	0.214	0.086	0.057	0.039
0.224	0.136	0.078	0.121	0.066	0.044	0.029
0.238	0.162	0.099	0.173	0.093	0.079	0.046
0.271	0.212	0.136	0.204	0.099	0.076	0.05
0.501	0.269	0.136	0.162	0.09	0.069	0.042
0.415	0.228	0.149	0.188	0.066	0.077	0.044

0.421	0.322	0.22	0.214	0.084	0.078	0.042
0.504	0.353	0.207	0.203	0.086	0.075	0.043
0.956	0.7	0.461	0.188	0.098	0.063	0.045
0.421	0.277	0.191	0.203	0.086	0.073	0.043
0.313	0.191	0.133	0.233	0.087	0.086	0.048
0.295	0.252	0.153	0.19	0.094	0.071	0.049
0.534	0.581	0.272	0.218	0.083	0.076	0.042
0.735	0.49	0.286	0.225	0.09	0.062	0.04
0.29	0.197	0.12	0.185	0.09	0.08	0.043
0.729	0.497	0.261	0.205	0.093	0.067	0.046
0.359	0.237	0.179	0.177	0.085	0.069	0.042
0.594	0.3	0.074	0.304	0.098	0.043	0.051
0.238	0.162	0.099	0.173	0.093	0.079	0.046
0.265	0.197	0.104	0.161	0.058	0.071	0.043
0.232	0.151	0.133	0.196	0.07	0.077	0.046
0.238	0.162	0.099	0.173	0.093	0.079	0.046
0.15	0.044	0.029	0.096	0.054	0.043	0.02
0.169	0.134	0.058	0.155	0.064	0.041	0.029
0	0	0	0.361	0.115	0.073	0.056
0.168	0.016	0	0.175	0.077	0.038	0.025
0.218	0.063	0	0.174	0.069	0.042	0.046
0.057	0.046	0.029	0.294	0.106	0.063	0.041
0.218	0.063	0	0.174	0.069	0.042	0.046
0.128	0.063	0	0.292	0.117	0.082	0.037
0.094	0.051	0	0.175	0.1	0.046	0.049
0.117	0.089	0.057	0.147	0.078	0.046	0.024
0.183	0.131	0.106	0.185	0.062	0.041	0.041
0.169	0.19	0.12	0.196	0.08	0.046	0.039
0.062	0	0	0.19	0.086	0.049	0.06
0.169	0.092	0.037	0.167	0.08	0.044	0.044
0.331	0.262	0.127	0.232	0.1	0.064	0.053
0	0	0	0.357	0.111	0.042	0.069
0.269	0.096	0.107	0.204	0.08	0.064	0.05
0	0	0	0.167	0.078	0.054	0.052
0.194	0.081	0.012	0.173	0.072	0.032	0.045
0	0	0	0.214	0.111	0.053	0.032
0.049	0.051	0	0.278	0.12	0.058	0.042
0.097	0.031	0	0.091	0.069	0.037	0.032
0.304	0.167	0.066	0.323	0.164	0.083	0.06
0.061	0	0	0.159	0.086	0.045	0.041
0.054	0.031	0	0.2	0.082	0.044	0.051
0.552	0.365	0.31	0.227	0.098	0.093	0.051
0.057	0.016	0	0.158	0.082	0.05	0.04
0.156	0	0	0.237	0.099	0.058	0.041
0.297	0.146	0.094	0.274	0.135	0.068	0.058
0.888	0.608	0.477	0.262	0.106	0.064	0.053
0.537	0.18	0.066	0.194	0.078	0.04	0.043
0.107	0.063	0	0.125	0.062	0.042	0.017
0.215	0.125	0	0.212	0.105	0.068	0.022
1.652	1.316	0.946	0.286	0.11	0.071	0.051

0.259	0.231	0.14	0.317	0.058	0.043	0.044
0.164	0.135	0.12	0.162	0.073	0.064	0.035
0.435	0.307	0.074	0.214	0.101	0.073	0.064
0.263	0.151	0	0.25	0.104	0.068	0.041
0.099	0	0	0.192	0.079	0.042	0.02
0.112	0	0	0.217	0.092	0.056	0.048
0.967	0.635	0.398	0.177	0.079	0.046	0.049
0.235	0.115	0.159	0.325	0.049	0.078	0.027
0.094	0.081	0.054	0.175	0.085	0.032	0.023
0.103	0.06	0.025	0.184	0.062	0.044	0.033
0.119	0.136	0.091	0.13	0.063	0.038	0.032
0.225	0.094	0.025	0.188	0.067	0.041	0.036
0.375	0.235	0.169	0.317	0.118	0.07	0.053
0.3	0.227	0.322	0.293	0.07	0.035	0.047
0.118	0.049	0	0.229	0.078	0.075	0.032
0.059	0.025	0	0.15	0.084	0.079	0.045
0.158	0.046	0.029	0.188	0.095	0.05	0.046
0	0	0	0.278	0.111	0.056	0.103
0.28	0.283	0.198	0.197	0.076	0.036	0.041
0.103	0.06	0.025	0.184	0.094	0.051	0.037
0	0	0	0.583	0.148	0	0
0.464	0.322	0.198	0.239	0.103	0.048	0.041
0.251	0.095	0.06	0.273	0.09	0.062	0.046
0.749	0.55	0.289	0.287	0.122	0.078	0.057
0	0	0	0.292	0.069	0.058	0.048
0	0	0	0.229	0.09	0.057	0.05
0.164	0.031	0	0.211	0.085	0.051	0.046
0.239	0.116	0.025	0.306	0.116	0.044	0.053
0.427	0.354	0.19	0.258	0.109	0.059	0.053
0.382	0.333	0.215	0.25	0.096	0.065	0.039
0.259	0.255	0.194	0.229	0.083	0.051	0.037
0.257	0.159	0.053	0.173	0.076	0.063	0.04
0.406	0.316	0.231	0.259	0.083	0.048	0.043
0	0	0	0.361	0.115	0.073	0.056
0	0	0	0.2	0.103	0.054	0.055
0.099	0	0	0.267	0.089	0.053	0.046
0.358	0.262	0.206	0.183	0.063	0.033	0.037
0.269	0.096	0.107	0.204	0.08	0.064	0.05
0.22	0.145	0.066	0.174	0.094	0.059	0.042
0.284	0.246	0.219	0.153	0.078	0.052	0.032
0.099	0	0	0.235	0.074	0.053	0.043
0	0	0	0.214	0.089	0.059	0.047
0.222	0.081	0	0.19	0.079	0.059	0.038
0.144	0.141	0.103	0.205	0.104	0.044	0.036
0.103	0.06	0.025	0.175	0.091	0.051	0.031
0.045	0	0	0.219	0.081	0.058	0.044
0.244	0.181	0.071	0.241	0.08	0.048	0.053
0.112	0.121	0.041	0.146	0.069	0.04	0.029
0	0	0	0.208	0.111	0.075	0.04
0.263	0.167	0.058	0.19	0.086	0.051	0.056

0.066	0.031	0	0.235	0.091	0.063	0.051
0.198	0.155	0.062	0.192	0.074	0.052	0.039
0.246	0.117	0.07	0.204	0.083	0.064	0.054
0.041	0	0	0.196	0.088	0.041	0.051
0.239	0.199	0.189	0.136	0.074	0.05	0.031
0.194	0.076	0.025	0.24	0.099	0.079	0.056
0.148	0.12	0.066	0.211	0.093	0.035	0.036
0.041	0	0	0.211	0.111	0.052	0.027
0.02	0	0	0.13	0.067	0.038	0.041
0.093	0	0	0.184	0.103	0.058	0.045
0	0	0	0.136	0.089	0.047	0.048
0	0	0	0.375	0.111	0.063	0.053
0.082	0	0	0.208	0.108	0.054	0.034
0.454	0.333	0.128	0.232	0.098	0.095	0.053
0.455	0.242	0.214	0.243	0.086	0.045	0.049
0.316	0.132	0.066	0.3	0.142	0.075	0.06
0.169	0.171	0.058	0.225	0.103	0.052	0.048
0.132	0.076	0.074	0.211	0.068	0.018	0.043
0	0	0	0.208	0.111	0.08	0.06
0.094	0.016	0	0.154	0.082	0.05	0.039
0.6	0.566	0.47	0.244	0.081	0.042	0.038
0	0	0	0.2	0.103	0.054	0.055
0.322	0.141	0.131	0.167	0.072	0.053	0.045
0.115	0.04	0.066	0.275	0.082	0.056	0.052
0.283	0.2	0.149	0.283	0.088	0.038	0.061
0.515	0.404	0.251	0.203	0.09	0.083	0.052
0	0	0	0.222	0.094	0.056	0.063
0.111	0.071	0.02	0.217	0.119	0.058	0.057
0.109	0.016	0	0.204	0.116	0.063	0.055
0.115	0.04	0	0.222	0.089	0.038	0.055
0	0	0	0.344	0.097	0.054	0.052
0.339	0.276	0.235	0.214	0.088	0.059	0.047
0.041	0	0	0.192	0.09	0.063	0.045
0.144	0.078	0	0.273	0.065	0.045	0.053
0.217	0.056	0	0.204	0.088	0.063	0.046
0.155	0.089	0	0.25	0.08	0.069	0.046
0	0	0	0.25	0.125	0.043	0.065
0.185	0.101	0.037	0.18	0.086	0.049	0.052
0.097	0.031	0	0.091	0.069	0.037	0.032
0.866	0.601	0.487	0.255	0.102	0.056	0.057
0.263	0.167	0.058	0.19	0.086	0.051	0.056
0.196	0.21	0.099	0.212	0.09	0.054	0.053
0.085	0.045	0.005	0.125	0.07	0.07	0.039
0.429	0.334	0.204	0.21	0.092	0.089	0.052
0.041	0	0	0.182	0.079	0.036	0.05
0.045	0	0	0.222	0.123	0.062	0.06
0.172	0.101	0.054	0.135	0.074	0.069	0.051
0.872	0.538	0.46	0.272	0.11	0.06	0.055
0	0	0	0.313	0.135	0.088	0.04
0.049	0	0	0.231	0.056	0.036	0.045

0	0	0	0.125	0.082	0.043	0.009
0.02	0	0	0.25	0.111	0.077	0.037
0.098	0.051	0.025	0.1	0.065	0.071	0.048
0	0	0	0.19	0.089	0.069	0.03
0.113	0.1	0.065	0.184	0.08	0.031	0.036
0.395	0.312	0.256	0.2	0.089	0.036	0.039
0.201	0.182	0.098	0.155	0.07	0.045	0.045
0.177	0.051	0.025	0.222	0.074	0.042	0.046
0.099	0.049	0	0.194	0.098	0.051	0.037
0.132	0.116	0.074	0.263	0.065	0.03	0.05
0.452	0.272	0.235	0.258	0.083	0.06	0.038
0.231	0.186	0.162	0.177	0.086	0.04	0.046
0.292	0.132	0.066	0.293	0.144	0.074	0.058
0.227	0.08	0.058	0.269	0.127	0.067	0.057
0.07	0.091	0.058	0.206	0.095	0.042	0.029
0.07	0.091	0.058	0.206	0.095	0.042	0.029
0.111	0.134	0.091	0.135	0.069	0.033	0.028
0.135	0.11	0.062	0.175	0.077	0.032	0.038
0	0	0	0.231	0.111	0.076	0.041
0.135	0.11	0.062	0.175	0.077	0.032	0.038
0.066	0	0	0.222	0.08	0.067	0.049
0.183	0.096	0.061	0.117	0.061	0.025	0.036
0.172	0.127	0.074	0.167	0.08	0.039	0.046
0.172	0.127	0.074	0.167	0.08	0.039	0.046
0.099	0.149	0.086	0.238	0.043	0.06	0.03
0.313	0.301	0.223	0.186	0.066	0.052	0.03
0	0	0	0.344	0.056	0.082	0.046
0	0	0	0.382	0.132	0.074	0.046
0.195	0.158	0.182	0.162	0.082	0.045	0.044
0.072	0.065	0.046	0.182	0.059	0.043	0.03
0.679	0.448	0.312	0.233	0.106	0.057	0.053
0.155	0.026	0.012	0.167	0.095	0.052	0.041
0	0	0	0.2	0.103	0.073	0.038
0.307	0.136	0.045	0.16	0.075	0.065	0.039
0.518	0.499	0.238	0.222	0.096	0.059	0.038
0.115	0.12	0	0.333	0.071	0.06	0.043
0.166	0.146	0.1	0.172	0.08	0.051	0.036
0.071	0.06	0	0.306	0.094	0.071	0.041
0.124	0.087	0.049	0.208	0.092	0.049	0.035
0.366	0.173	0.041	0.109	0.071	0.062	0.04
0.116	0.06	0	0.289	0.119	0.069	0.047
0.343	0.205	0.115	0.222	0.08	0.061	0.045
0.241	0.232	0.122	0.25	0.105	0.052	0.042
0.146	0.12	0.033	0.286	0.081	0.05	0.041
0.117	0.08	0.04	0.184	0.103	0.051	0.036
0.21	0.187	0.083	0.224	0.08	0.052	0.04
0.134	0.106	0.057	0.152	0.069	0.039	0.031
0.061	0	0	0.174	0.088	0.045	0.049
0.225	0.134	0.037	0.275	0.084	0.055	0.05
0.165	0.056	0.005	0.208	0.099	0.066	0.062

0.231	0.186	0.162	0.177	0.086	0.04	0.046
0.061	0	0	0.375	0.077	0.063	0.062
0.137	0.06	0	0.271	0.131	0.063	0.061
0.485	0.307	0.074	0.257	0.115	0.078	0.064
0	0	0	0.179	0.116	0.059	0.074
0	0	0	0.313	0.135	0.088	0.04
0.065	0.007	0	0.158	0.068	0.038	0.044
0.195	0.195	0.087	0.227	0.074	0.038	0.037
0.164	0.081	0.087	0.173	0.081	0.031	0.034
0.101	0.061	0.048	0.25	0.081	0.052	0.035
0.057	0.046	0.037	0.278	0.102	0.06	0.039
0.111	0	0	0.25	0.095	0.056	0.042
0.167	0.097	0.089	0.155	0.072	0.048	0.029
0.094	0.016	0	0.154	0.082	0.05	0.039
0.189	0.133	0.029	0.227	0.081	0.055	0.051
0.222	0.198	0.132	0.167	0.092	0.046	0.041
0	0	0	0.222	0.111	0.063	0.044
0.305	0.255	0.265	0.162	0.077	0.052	0.034
0.791	0.493	0.394	0.244	0.102	0.058	0.053
0.275	0.287	0.165	0.235	0.106	0.048	0.039
1.758	1.361	0.921	0.238	0.092	0.067	0.047
0.139	0.047	0	0.15	0.077	0.059	0.043
0.09	0.054	0	0.16	0.07	0.053	0.048
0.249	0.197	0.05	0.16	0.089	0.053	0.025
0.127	0.036	0	0.225	0.095	0.057	0.055
0.22	0.141	0.132	0.172	0.072	0.045	0.036
0.227	0.132	0	0.22	0.086	0.044	0.045
0.138	0.056	0.058	0.238	0.089	0.076	0.048

JGI5	JGI6	JGI7	JGI8	JGI9	JGI10	JGT
0.027	0.035	0.005	0.016	0	0	0.539
0.026	0.023	0.015	0.013	0.011	0.006	0.51
0.034	0.018	0.018	0.014	0.007	0.012	0.485
0.029	0.054	0	0	0	0	0.527
0.028	0.02	0.018	0.016	0.005	0	0.445
0.037	0	0	0	0	0	0.784
0.019	0.019	0.015	0.009	0.009	0.008	0.459
0	0	0	0	0	0	0.32
0.023	0.022	0.011	0.009	0.008	0	0.368
0.024	0.014	0.014	0.012	0.009	0.012	0.426
0.027	0.023	0.018	0.012	0.011	0.009	0.544
0.021	0.021	0.016	0.011	0.011	0.005	0.429
0.015	0.014	0.016	0.012	0.016	0	0.345
0.034	0.017	0.015	0.009	0.009	0.005	0.424
0.032	0.028	0.018	0.014	0.013	0.008	0.628
0.029	0.027	0.018	0.013	0.012	0.009	0.588
0.024	0.024	0.028	0	0	0	0.45
0.036	0.014	0.013	0.013	0.012	0.009	0.561
0.023	0.017	0.019	0.011	0.012	0.009	0.498
0.023	0.019	0.012	0.013	0.006	0.006	0.421
0.038	0.022	0.022	0.007	0.008	0	0.489
0.02	0.016	0.013	0.017	0	0	0.411
0.032	0.019	0.022	0.014	0.011	0.011	0.501
0.025	0.029	0.015	0.012	0.013	0.009	0.58
0.017	0.01	0.007	0.007	0.007	0.007	0.297
0.041	0.031	0.021	0.015	0.013	0.009	0.758
0.017	0.011	0.014	0.008	0.008	0.008	0.391
0.032	0.019	0.017	0.01	0.012	0.008	0.531
0.02	0.015	0.014	0.011	0.011	0.007	0.387
0.02	0.009	0.017	0.011	0.007	0.01	0.415
0.031	0.019	0.014	0.011	0.006	0.006	0.482
0.034	0.021	0.019	0.012	0.009	0.009	0.558
0.035	0.018	0.022	0.019	0.013	0	0.57
0.025	0.02	0.009	0.011	0.008	0.004	0.418
0.038	0.027	0.022	0.018	0.012	0.01	0.652
0.025	0.015	0.014	0.006	0.006	0.007	0.404
0.027	0.019	0.014	0.006	0.01	0.008	0.515
0.035	0	0	0	0	0	0.494
0.027	0.018	0.018	0.013	0.011	0.01	0.594
0.035	0.021	0.017	0.014	0.009	0.006	0.508
0.026	0.014	0.011	0.009	0.009	0.006	0.357
0.021	0.021	0.005	0	0	0	0.333
0.039	0.027	0.021	0.015	0.014	0.009	0.759
0.037	0.027	0.024	0.014	0.015	0	0.513
0.017	0.012	0.012	0.009	0.006	0.004	0.32
0.036	0.022	0.018	0.011	0.01	0.008	0.495
0.038	0.024	0.018	0.012	0.011	0.009	0.541
0.032	0.02	0.017	0.013	0.01	0.006	0.459
0.03	0.019	0.017	0.012	0.01	0.008	0.469

0.033	0.021	0.018	0.012	0.011	0.008	0.523
0.033	0.022	0.014	0.014	0.011	0.008	0.508
0.03	0.02	0.015	0.012	0.011	0.008	0.489
0.034	0.023	0.017	0.013	0.011	0.008	0.511
0.037	0.025	0.016	0.011	0.01	0.008	0.561
0.038	0.024	0.017	0.012	0.011	0.008	0.514
0.033	0.025	0.018	0.012	0.014	0.007	0.527
0.035	0.02	0.017	0.014	0.011	0.008	0.522
0.034	0.02	0.015	0.012	0.01	0.009	0.497
0.031	0.02	0.017	0.014	0.012	0.008	0.513
0.032	0.024	0.017	0.012	0.01	0.009	0.475
0.041	0.021	0.017	0.024	0.015	0.004	0.618
0.036	0.022	0.018	0.011	0.01	0.008	0.495
0.03	0.019	0.018	0.009	0.01	0.006	0.425
0.034	0.023	0.018	0.01	0.009	0.009	0.492
0.036	0.022	0.018	0.011	0.01	0.008	0.495
0.013	0.011	0.012	0.007	0.002	0.002	0.259
0.009	0.02	0.017	0.007	0.008	0.004	0.354
0.072	0.011	0.028	0	0	0	0.717
0.021	0.02	0.016	0.021	0.005	0	0.398
0.019	0.021	0.016	0.016	0.01	0	0.413
0.038	0.015	0.013	0.008	0.011	0.014	0.604
0.019	0.021	0.016	0.016	0.01	0	0.413
0.043	0.02	0.029	0.009	0.016	0	0.644
0.03	0.025	0.021	0.016	0.017	0	0.478
0.015	0.009	0.01	0.012	0.008	0.007	0.355
0.027	0.012	0.012	0.01	0.008	0.007	0.404
0.023	0.016	0.011	0.008	0.009	0.005	0.434
0.035	0.02	0.023	0.016	0	0	0.479
0.032	0.026	0.014	0.011	0.009	0.007	0.433
0.032	0.022	0.015	0.013	0.012	0.008	0.55
0.026	0.035	0.024	0	0	0	0.664
0.035	0.021	0.016	0.013	0.007	0.015	0.505
0.014	0.018	0.007	0	0	0	0.389
0.017	0.017	0.017	0.014	0.016	0.006	0.409
0.017	0.017	0.028	0	0	0	0.472
0.037	0.017	0.022	0.01	0.026	0	0.609
0.015	0.012	0.009	0.012	0.016	0	0.293
0.041	0.031	0.021	0.015	0.013	0.009	0.758
0.027	0.021	0.012	0.015	0	0	0.407
0.024	0.014	0.022	0.005	0.016	0	0.459
0.032	0.018	0.017	0.017	0.011	0.012	0.575
0.021	0.018	0.018	0.014	0.008	0	0.409
0.041	0.032	0.012	0.031	0	0	0.55
0.034	0.025	0.018	0.015	0.009	0.009	0.646
0.027	0.027	0.015	0.013	0.01	0.008	0.586
0.027	0.02	0.013	0.017	0.008	0.006	0.446
0.008	0.016	0.014	0.01	0.016	0	0.31
0.021	0.028	0.011	0.01	0.016	0	0.493
0.037	0.025	0.018	0.012	0.012	0.009	0.631

0.023	0.026	0.012	0.012	0.011	0.006	0.551
0.024	0.02	0.015	0.006	0.006	0.006	0.41
0.037	0.023	0.02	0.014	0.015	0.006	0.567
0.027	0.022	0.02	0.014	0.013	0	0.558
0.012	0.02	0.016	0.012	0	0	0.394
0.032	0.022	0.019	0.014	0	0	0.499
0.024	0.02	0.016	0.012	0.01	0.008	0.441
0.027	0.021	0.011	0.018	0.009	0.014	0.582
0.027	0.026	0.013	0.009	0.01	0.008	0.409
0.019	0.02	0.016	0.01	0.01	0.012	0.411
0.015	0.015	0.013	0.007	0.009	0.005	0.329
0.033	0.018	0.011	0.013	0.009	0.008	0.424
0.037	0.027	0.02	0.018	0.014	0.012	0.686
0.031	0.019	0.019	0.009	0.008	0.013	0.545
0.03	0.009	0.013	0.01	0.012	0	0.488
0.029	0.018	0.015	0.015	0.012	0	0.447
0.033	0.026	0.016	0.016	0.008	0.014	0.491
0	0	0	0	0	0	0.548
0.027	0.02	0.015	0.008	0.009	0.008	0.437
0.03	0.028	0.019	0.011	0.01	0.012	0.477
0	0	0	0	0	0	0.731
0.028	0.022	0.013	0.012	0.009	0.006	0.52
0.046	0.014	0.015	0.019	0.012	0.01	0.587
0.038	0.028	0.016	0.015	0.013	0.008	0.662
0.023	0.04	0	0	0	0	0.53
0.031	0.025	0.026	0	0	0	0.509
0.028	0.017	0.021	0.016	0.005	0	0.48
0.022	0.022	0.023	0.016	0.01	0.006	0.617
0.031	0.021	0.022	0.011	0.013	0.008	0.583
0.031	0.022	0.013	0.011	0.01	0.007	0.544
0.03	0.019	0.014	0.008	0.008	0.007	0.486
0.022	0.022	0.01	0.012	0.009	0.006	0.432
0.022	0.017	0.017	0.011	0.011	0.011	0.521
0.072	0.011	0.028	0	0	0	0.717
0.031	0.026	0.029	0	0	0	0.499
0.034	0.04	0.021	0.016	0	0	0.566
0.018	0.015	0.015	0.008	0.007	0.006	0.385
0.035	0.021	0.016	0.013	0.007	0.015	0.505
0.029	0.017	0.016	0.016	0.012	0.008	0.468
0.022	0.014	0.009	0.01	0.009	0.007	0.385
0.035	0.034	0.012	0.025	0	0	0.512
0.036	0.039	0	0	0	0	0.485
0.033	0.018	0.019	0.012	0.01	0	0.457
0.028	0.025	0.017	0.011	0.013	0.009	0.492
0.025	0.022	0.014	0.01	0.01	0.012	0.441
0.04	0.019	0.019	0.023	0	0	0.501
0.026	0.019	0.015	0.012	0.009	0.005	0.508
0.015	0.021	0.016	0.007	0.012	0.014	0.368
0.027	0	0	0	0	0	0.461
0.032	0.028	0.017	0.016	0.014	0.007	0.497

0.013	0.029	0.015	0.007	0.016	0	0.518
0.024	0.026	0.014	0.01	0.009	0.005	0.446
0.036	0.023	0.014	0.015	0.01	0.012	0.515
0.026	0.024	0.011	0.014	0	0	0.449
0.021	0.014	0.011	0.01	0.008	0.007	0.363
0.032	0.02	0.019	0.011	0.008	0.006	0.572
0.026	0.02	0.024	0.015	0.012	0.008	0.479
0.026	0.021	0.019	0.014	0	0	0.481
0.017	0.019	0.011	0.02	0	0	0.345
0.027	0.022	0.007	0.013	0	0	0.46
0	0	0	0	0	0	0.32
0	0	0	0	0	0	0.602
0.024	0.017	0.015	0.016	0	0	0.476
0.028	0.025	0.017	0.017	0.015	0.008	0.588
0.027	0.018	0.014	0.014	0.008	0.008	0.513
0.038	0.031	0.021	0.017	0.01	0.011	0.705
0.029	0.018	0.013	0.012	0.013	0.007	0.52
0.021	0.012	0.019	0.011	0.008	0.011	0.421
0.024	0	0	0	0	0	0.483
0.023	0.024	0.013	0.009	0.008	0	0.402
0.024	0.014	0.013	0.009	0.009	0.008	0.482
0.031	0.026	0.029	0	0	0	0.499
0.034	0.023	0.015	0.012	0.007	0.009	0.437
0.033	0.026	0.017	0.01	0.008	0.017	0.576
0.026	0.019	0.019	0.017	0.013	0.012	0.575
0.024	0.022	0.016	0.015	0.013	0.01	0.529
0.042	0.017	0.039	0	0	0	0.534
0.029	0.022	0.014	0.01	0.014	0.01	0.55
0.027	0.024	0.019	0.018	0.008	0	0.533
0.033	0.029	0.022	0.023	0.01	0	0.521
0.033	0.033	0.024	0	0	0	0.636
0.029	0.02	0.016	0.011	0.009	0.008	0.502
0.036	0.023	0.012	0.006	0	0	0.468
0.023	0.027	0.02	0.008	0.016	0	0.529
0.031	0.019	0.021	0.009	0.008	0	0.488
0.021	0.018	0.016	0.017	0.013	0	0.531
0.046	0.02	0	0	0	0	0.549
0.036	0.022	0.013	0.014	0.011	0.007	0.471
0.015	0.012	0.009	0.012	0.016	0	0.293
0.033	0.019	0.018	0.014	0.01	0.01	0.572
0.032	0.028	0.017	0.016	0.014	0.007	0.497
0.025	0.024	0.015	0.009	0.012	0.008	0.502
0.024	0.017	0.016	0.007	0.006	0.002	0.375
0.024	0.021	0.018	0.015	0.012	0.01	0.541
0.023	0.022	0.011	0.014	0	0	0.416
0.034	0.026	0.019	0.023	0	0	0.568
0.027	0.013	0.014	0.01	0.01	0.013	0.415
0.035	0.022	0.015	0.015	0.011	0.011	0.605
0.042	0.011	0.03	0	0	0	0.659
0.029	0.04	0.016	0.016	0	0	0.469

0.011	0.015	0.008	0	0	0	0.293
0.029	0.028	0.016	0.02	0	0	0.568
0.025	0.01	0.009	0.007	0.007	0.012	0.353
0.018	0.016	0.017	0	0	0	0.429
0.021	0.014	0.014	0.011	0.011	0.011	0.414
0.026	0.022	0.015	0.009	0.009	0.009	0.455
0.021	0.018	0.007	0.008	0.011	0.011	0.394
0.04	0.015	0.015	0.011	0.007	0.012	0.485
0.035	0.027	0.018	0.012	0.012	0	0.485
0.023	0.017	0.019	0.011	0.012	0.009	0.498
0.028	0.023	0.014	0.013	0.009	0.01	0.536
0.029	0.02	0.016	0.01	0.008	0.008	0.44
0.035	0.032	0.02	0.017	0.011	0.011	0.695
0.034	0.03	0.021	0.017	0.01	0.019	0.652
0.029	0.02	0.011	0.007	0.013	0.014	0.467
0.029	0.02	0.011	0.007	0.013	0.014	0.467
0.023	0.015	0.008	0.005	0.007	0.006	0.328
0.018	0.015	0.012	0.011	0.011	0.009	0.399
0.059	0	0	0	0	0	0.517
0.018	0.015	0.012	0.011	0.011	0.009	0.399
0.025	0.035	0.015	0.022	0	0	0.517
0.016	0.011	0.011	0.01	0.006	0.004	0.296
0.028	0.016	0.012	0.009	0.01	0.011	0.417
0.028	0.016	0.012	0.009	0.01	0.011	0.417
0.023	0.012	0.014	0.007	0.011	0.007	0.444
0.028	0.015	0.015	0.008	0.009	0.007	0.416
0.039	0.034	0.031	0	0	0	0.632
0.033	0.022	0.028	0	0	0	0.717
0.027	0.018	0.014	0.008	0.007	0.008	0.414
0.019	0.021	0.013	0.006	0.011	0.008	0.391
0.032	0.022	0.015	0.013	0.01	0.009	0.55
0.027	0.019	0.009	0.012	0.004	0.012	0.439
0.063	0	0	0	0	0	0.476
0.023	0.02	0.013	0.016	0.01	0.006	0.427
0.035	0.022	0.016	0.011	0.012	0.008	0.519
0.031	0.029	0.015	0.014	0.024	0	0.62
0.017	0.017	0.015	0.009	0.008	0.005	0.409
0.042	0.01	0.019	0.012	0.02	0	0.614
0.032	0.016	0.012	0.008	0.008	0.008	0.468
0.024	0.012	0.013	0.011	0.006	0.002	0.35
0.041	0.014	0.017	0.017	0.015	0	0.627
0.038	0.015	0.015	0.016	0.011	0.01	0.514
0.041	0.019	0.01	0.012	0.013	0.01	0.553
0.026	0.032	0.019	0.013	0.02	0.005	0.572
0.024	0.026	0.02	0.012	0.01	0.01	0.475
0.023	0.023	0.012	0.009	0.009	0.004	0.475
0.023	0.016	0.01	0.009	0.008	0.005	0.362
0.029	0.023	0.012	0.015	0	0	0.434
0.022	0.024	0.019	0.017	0.017	0.006	0.57
0.026	0.024	0.015	0.01	0.007	0.002	0.52

0.029	0.02	0.016	0.01	0.008	0.008	0.44
0.03	0.02	0.033	0.007	0	0	0.668
0.035	0.031	0.018	0.02	0.015	0	0.644
0.041	0.024	0.02	0.015	0.013	0.006	0.632
0.032	0	0	0	0	0	0.461
0.042	0.011	0.03	0	0	0	0.659
0.014	0.018	0.013	0.008	0.003	0	0.365
0.016	0.017	0.016	0.012	0.013	0.01	0.459
0.029	0.01	0.013	0.009	0.005	0.006	0.39
0.036	0.018	0.016	0.006	0.006	0.006	0.506
0.039	0.013	0.018	0.006	0.008	0.009	0.571
0.028	0.017	0.016	0.018	0	0	0.522
0.026	0.015	0.013	0.008	0.005	0.005	0.378
0.023	0.024	0.013	0.009	0.008	0	0.402
0.024	0.025	0.016	0.012	0.015	0.007	0.514
0.021	0.021	0.013	0.01	0.009	0.007	0.426
0.009	0	0	0	0	0	0.45
0.022	0.016	0.013	0.011	0.009	0.008	0.404
0.032	0.022	0.015	0.014	0.01	0.01	0.56
0.031	0.024	0.011	0.009	0.01	0.006	0.52
0.032	0.021	0.018	0.013	0.011	0.008	0.547
0.034	0.018	0.014	0.011	0.016	0	0.422
0.031	0.015	0.016	0.007	0.009	0	0.409
0.026	0.02	0.012	0.013	0.015	0.01	0.423
0.035	0.02	0.02	0.014	0.009	0	0.532
0.027	0.014	0.012	0.01	0.007	0.006	0.401
0.03	0.022	0.019	0.013	0.019	0	0.499
0.036	0.018	0.02	0.013	0.009	0.019	0.566

LP1	Eig1Z	Eig1m	Eig1v	Eig1e	Eig1p	SEigZ
2.436	54.128	54.135	67.722	54.165	70.568	1.25
2.354	112.829	112.849	162.151	113.071	173.644	1.286
2.485	99.644	99.655	119.106	99.738	123.728	1.643
2.22	23.085	23.088	32.362	23.142	34.681	0.643
2.449	44.702	44.716	78.649	44.888	86.584	1.464
2.481	26.615	26.42	46.891	38.625	38.306	3.093
2.482	154.978	154.995	223.937	155.49	243.031	1.607
2.37	15.469	15.47	23.026	15.525	25.198	0.821
2.511	64.251	64.224	77.203	65.284	80.071	1.218
2.275	68.386	68.399	98.98	68.513	105.819	0.643
2.482	234.283	234.363	386.353	234.86	418.422	3.964
2.504	155.448	155.412	202.383	158.517	211.92	2.666
2.448	71.077	71.078	77.855	71.133	79.792	0.143
2.518	112.13	112.127	158.273	113.14	167.399	2.183
2.631	105.03	105.036	142.001	109.94	147.986	2.304
2.631	96.692	96.696	129.789	101.449	135.032	2.054
2.431	31.693	31.697	47.823	31.809	52.847	0.536
2.457	192.25	191.749	295.4	192.921	324.353	3.655
2.252	80.306	80.321	114.57	80.452	122.249	1.036
2.436	80.735	80.75	112.845	80.848	119.755	0.893
2.586	70.227	70.233	96.933	70.429	104.624	1.071
2.441	40.825	40.82	69.485	51.217	69.262	2.125
2.451	69.463	69.436	85.273	70.568	88.173	1.54
2.63	92.084	92.087	122.927	96.668	127.663	1.911
2.35	123.483	123.5	171.484	123.773	183.343	0.536
2.724	114.867	114.813	129.064	114.932	132.958	1.583
2.259	113.55	113.576	167.2	113.745	178.763	0.893
2.499	254.669	254.681	296.302	254.951	307.269	2.071
2.502	75.305	75.31	105.43	75.528	115.076	0.429
2.244	122.973	123.01	198.465	123.23	214.505	1.143
2.469	174.616	174.639	288.985	192.194	297.844	2.839
2.684	242.802	242.778	314.345	244.832	331.643	2.65
2.443	65.326	65.334	85.515	65.431	90.365	0.643
2.368	81.609	81.614	104.339	81.804	110.512	0.536
2.828	147.31	147.324	181.247	147.467	189.242	1.143
2.463	164.139	164.151	216.608	164.571	231.013	1.214
2.67	89.004	89.009	105.021	89.112	109.14	0.643
2.505	28.068	28.072	43.849	28.188	48.074	1.071
2.543	169.652	169.657	178.301	169.676	180.163	0.75
2.601	91.371	91.378	109.029	91.488	113.311	1.286
2.51	154.642	154.655	197.62	154.893	208.869	1.607
2.462	44.195	44.198	56.978	44.293	60.517	0.536
2.719	252.505	252.529	298.382	252.632	307.974	1.75
2.502	53.731	53.703	62.122	54.742	63.373	1.29
2.505	164.622	164.667	259.765	164.99	280.614	1.286
2.568	95.661	95.638	130.109	100.001	134.798	2.701
2.568	105.383	105.388	142.385	108.952	148.191	2.304
2.574	144.341	144.331	206.906	162.219	205.346	3.357
2.572	124.395	124.383	192.832	145.087	192.105	4.018

2.581	156.08	156.085	229.359	163.054	240.364	3.357
2.573	136.916	136.932	217.64	142.26	234.096	3.714
2.575	277.296	277.291	385.241	299.078	391.308	4.393
2.574	139.332	139.347	216.895	145.615	228.896	3.714
2.646	120.517	120.528	184.669	127.748	191.388	3.429
2.573	120.551	120.554	159.97	124.938	164.742	2.304
2.579	189.17	189.167	260.378	201.802	261.657	3.821
2.577	196.894	196.912	307.63	204.658	327.65	3.857
2.553	99.421	99.431	151.495	103.575	162.424	3.214
2.575	182.767	182.758	281.103	206.925	283.678	4.768
2.574	131.193	131.21	207.9	136.403	220.251	3.196
2.489	89.928	89.703	128.417	96.307	134.698	2.554
2.568	96.981	96.985	130.109	100.452	135.17	2.054
2.574	125.345	125.344	172.149	132.807	175.342	2.679
2.572	112.354	112.361	166.18	119.198	171.1	3.036
2.568	100.09	100.094	133.063	103.543	138.102	2.054
2.38	111.082	111.099	152.241	112.018	161.058	1.268
2.411	133.012	133.001	189.649	134.287	202.754	1.683
2.494	27.052	27.014	48.433	38.877	40.961	3.326
2.35	63.184	63.156	73.071	64.22	74.977	0.933
2.485	67.922	67.894	91.484	72.443	94.335	1.558
2.359	44.724	44.689	73.578	54.569	70.647	2.308
2.485	66.337	66.306	78.14	71.035	77.652	1.415
2.564	61.277	61.246	79.269	67.894	76.93	2.558
2.482	50.713	50.685	60.56	51.778	61.902	1.29
2.257	57.847	57.843	87.142	67.072	87.791	1.482
2.401	114.372	114.397	170.383	114.607	182.909	1.286
2.397	127.637	127.407	155.021	127.903	164.381	1.143
2.51	51.691	51.701	76.261	51.828	81.993	1.536
2.541	84.603	84.548	115.128	84.862	123.359	1.512
2.451	125.132	125.122	171.168	129.514	178.585	2.808
2.315	34.063	34.044	55.524	35.064	59.264	1.397
2.544	88.439	88.414	111.925	89.616	116.742	1.79
2.336	31.725	31.677	46.932	33.457	49.786	1.723
2.467	73.358	73.333	101.763	74.563	108.843	1.218
2.368	29.119	28.922	55.698	39.236	50.579	2.68
2.42	36.668	36.66	50.559	46.166	45.622	1.411
2.447	64.981	64.984	81.707	65.121	86.385	0.286
2.724	114.867	114.813	129.064	114.932	132.958	1.583
2.457	56.229	56.203	67.289	57.235	69.569	1.183
2.364	57.185	57.137	74.535	58.98	77.582	1.937
2.633	130.761	130.708	174.045	138.184	179.94	3.598
2.475	47.167	47.179	77.726	47.334	84.901	1.321
2.4	50.257	50.114	64.945	50.363	69.751	1.417
2.675	120.013	120.018	130.175	120.04	132.361	1
2.676	483.55	483.714	714.78	484.425	758.155	3.25
2.483	117.43	117.443	169.75	123.117	178.172	1.911
2.336	64.638	64.649	90.702	64.745	96.619	0.393
2.495	97.327	97.331	113.927	97.469	118.402	0.679
2.727	390.04	390.128	568.188	390.637	606.375	3.643

2.285	160.13	160.144	246.445	172.475	251.811	2.929
2.491	165.214	165.194	222.471	166.694	237.323	1.861
2.679	128.463	128.473	152.295	128.571	157.931	2.286
2.386	77.2	77.207	101.298	77.343	107.903	1.679
2.091	51.821	51.827	72.167	51.964	77.456	0.786
2.571	65.795	65.801	78.313	65.857	81.252	0.893
2.607	218.73	218.797	335.026	219.093	358.352	3.25
2.279	67.529	67.518	108.623	84.567	102.84	3.375
2.331	70.704	70.706	76.079	70.718	77.22	0.75
2.321	55.73	55.743	79.61	55.797	84.528	0.75
2.383	119.212	119.219	149.991	119.466	158.451	0.536
2.445	71.406	71.374	84.429	75.42	84.459	1.915
2.621	124.794	124.797	139.213	124.9	143.07	0.786
2.372	135.16	134.739	221.866	135.72	245.724	3.036
2.452	72.332	72.192	104.216	72.596	114.624	1.774
2.516	51.058	50.977	57.778	51.113	60.214	0.833
2.52	68.378	68.312	101.607	68.582	110.266	1.512
2.246	13.587	13.523	23.65	13.671	26.755	1.119
2.502	150.485	150.272	211.438	154.252	226.859	2.304
2.382	55.478	55.421	63.188	55.526	65.689	0.833
2.175	5.119	5.045	14.988	9.668	11.764	1.85
2.599	340.949	340.523	388.291	360.647	387.284	3.042
2.435	63.216	63.193	92.211	69.569	92.982	2.808
2.714	196.546	196.56	224.077	196.619	230.103	1.5
2.334	31.263	31.265	36.062	31.283	37.16	0.643
2.628	62.543	62.554	85.56	62.63	90.761	0.893
2.451	55.887	55.904	98.54	56.107	108.363	1.714
2.289	67.817	67.83	92.806	67.887	97.945	0.75
2.472	155.108	155.168	261.168	155.492	282.998	2.464
2.421	209.763	209.763	296.128	226.973	301.501	2.446
2.373	189.018	188.986	265.171	206.445	267.163	2.951
2.474	89.361	89.366	113.538	89.554	120.516	0.821
2.378	127.552	127.332	208.204	128.886	227.207	2.79
2.494	27.875	27.838	49.575	39.646	42.429	3.326
2.707	59.563	59.57	74.247	59.63	77.666	0.893
2.298	47.017	47.019	50.855	47.028	51.679	0.5
2.372	232.182	232.198	305.285	232.807	325.335	1.25
2.544	87.596	87.572	114.294	88.796	119.906	1.79
2.475	74.763	74.768	92.052	74.879	96.486	0.893
2.54	164.885	164.891	194.131	165.115	202.633	1.107
2.47	44.051	44.072	99.601	44.213	115.158	2.286
2.455	29.923	29.937	62.42	30.016	71.121	1.643
2.535	101.272	101.29	139.8	101.42	148.893	1.679
2.364	80.14	80.115	86.842	81.036	87.594	1.397
2.361	62.516	62.519	67.702	62.53	68.817	0.75
2.383	39.312	39.322	68.427	40.938	74.212	1.804
2.419	88.735	88.489	148.182	102.248	153.617	2.554
2.418	74.842	74.844	86.324	74.928	89.97	0.714
2.342	28.302	28.304	38.619	28.376	41.354	0.786
2.579	94.169	94.117	132.28	94.468	142.227	1.762

2.321	56.171	56.175	75.761	56.33	81.139	0.536
2.434	103.488	103.441	149.526	103.827	162.042	1.762
2.539	89.719	89.608	123.591	90.017	133.19	1.845
2.459	59.138	59.084	73.462	61.056	75.72	2.08
2.443	142.539	142.517	169.774	143.6	176.851	1.754
2.548	63.772	63.769	98.975	74.254	99.475	2.679
2.306	71.18	71.123	90.673	73.248	94.126	2.08
2.468	56.087	56.095	73.179	56.158	77.055	0.643
2.5	56.837	56.84	71.144	56.961	75.139	0.429
2.358	58.261	58.265	74.961	58.407	79.451	0.536
2.37	15.489	15.49	22.705	16.017	24.202	1.196
2.117	16.532	16.534	27.385	16.624	30.387	0.714
2.51	80.611	80.612	88.107	80.666	90.2	0.393
2.632	103.253	103.265	153.17	108.353	161.772	2.411
2.417	188.137	188.153	251.191	188.617	267.975	2.393
2.678	111.07	111.075	121.835	111.099	124.154	1.25
2.374	76.756	76.738	108.144	77.797	115.608	1.576
2.243	82.44	82.458	122.238	82.593	130.952	0.893
2.37	24.034	24.038	40.101	24.131	44.65	1.179
2.517	69.026	68.925	84.976	70.096	89.33	1.409
2.46	248.191	248.15	278.496	264.377	272.659	2.165
2.707	59.122	59.129	75.421	59.193	79.182	0.893
2.582	115.769	115.721	159.947	116.109	171.357	1.762
2.388	71.039	71.059	113.611	71.193	122.888	0.893
2.419	93.036	93.055	135.396	93.208	144.776	1.143
2.633	122.283	122.294	171.891	127.763	180.308	2.161
2.47	43.659	43.664	57.401	43.771	60.528	1.036
2.751	87.028	87.035	107.685	87.144	112.704	0.893
2.744	72.732	72.738	89.672	72.828	93.792	0.893
2.397	48.42	48.426	60.927	48.454	63.588	0.75
2.342	44.293	44.306	76.047	44.457	83.518	0.786
2.633	142.457	142.432	193.929	144.326	204.367	2.007
2.518	85.041	85.053	114.156	85.161	121.189	1.786
2.378	59.136	59.147	104.208	64.546	108.392	2.161
2.505	95.857	95.872	129.808	95.992	137.81	1.786
2.342	47.954	47.966	87.608	48.173	99.044	1.821
2.302	26.918	26.922	35.452	26.949	37.335	0.893
2.504	79.498	79.443	108.175	79.743	115.873	1.512
2.447	64.035	64.036	68.474	64.071	69.75	0.143
2.514	283.477	283.542	414.062	284.003	442.397	3.036
2.579	94.169	94.117	132.28	94.468	142.227	1.762
2.438	77.364	77.364	119.129	90.625	118.209	1.804
2.555	67.843	67.846	80.979	67.947	84.594	0.679
2.633	119.987	119.992	158.807	125.317	165.195	2.304
2.446	55.804	55.778	68.651	56.819	71.254	1.433
2.749	68.625	68.636	92.191	68.715	97.48	1.143
2.532	88.479	88.486	119.357	88.689	128.565	1.036
2.661	244.327	244.352	310.868	244.673	326.73	1.286
2.493	46.71	46.713	55.914	46.771	58.284	1.036
2.242	33.781	33.789	51.878	33.867	56.041	0.643

2.478	55.566	55.568	64.753	55.644	67.314	0.143
2.511	45.145	45.148	54.887	45.209	57.388	1.036
2.53	81.893	81.899	108.478	82.085	116.644	0.786
2.55	52.382	52.384	61.022	52.438	63.359	0.786
2.39	63.475	63.489	95.256	63.612	102.394	0.786
2.51	158.724	158.724	217.356	171.424	220.344	2.429
2.494	99.661	99.67	140.368	100	151.436	1.214
2.487	78.576	78.589	123.419	83.808	128.276	2.161
2.388	51.02	51.001	64.288	51.836	67.071	1.29
2.252	78.852	78.868	119.01	79.048	128.31	1.036
2.475	167.268	167.288	206.113	167.375	214.232	1.75
2.467	122.735	122.755	186.506	123.147	202.551	1.714
2.671	104.913	104.917	114.319	104.938	116.332	1.25
2.618	90.19	90.192	94.6	90.202	95.549	0.5
2.261	63.072	63.077	83.883	63.242	89.594	0.679
2.261	61.325	61.338	92.318	61.47	99.43	0.786
2.326	126.823	126.841	165.948	126.98	174.68	0.893
2.373	69	69.014	101.143	69.134	108.311	0.643
2.358	31.123	31.124	33.437	31.129	33.963	0.25
2.373	69	69.014	101.143	69.134	108.311	0.643
2.413	44.341	44.311	52.308	45.393	53.715	1.218
2.461	121.472	121.488	180.959	124.872	193.499	1.554
2.495	90.989	90.996	108.584	91.122	112.361	0.786
2.495	90.989	90.996	108.584	91.122	112.361	0.786
2.278	86.637	86.639	138.738	98.163	143.311	1.946
2.402	177.341	177.357	226.039	177.648	238.311	1.286
2.406	32.547	32.333	65.033	35.08	72.592	2.161
2.497	35.833	35.616	57.59	49.111	48.035	3.093
2.459	162.216	162.14	200.263	162.53	211.437	1.405
2.43	70.255	70.259	88.443	70.401	93.829	0.714
2.661	215.65	215.668	268.609	215.957	281.698	1.286
2.367	72.403	72.408	93.416	72.584	99.109	0.536
2.267	16.928	16.931	22.822	16.944	24.06	0.75
2.63	86.986	87.004	123.016	87.109	130.723	1.143
2.525	137.537	137.538	202.558	151.813	204.507	2.482
2.315	57.722	57.722	82.085	62.814	81.862	1.661
2.482	123.575	123.583	164.532	126.134	173.436	1.411
2.383	38.658	38.653	63.844	47.621	60.881	1.804
2.425	62.129	62.123	99.329	73.331	97.261	1.696
2.531	117.963	117.957	159.865	131.066	161.144	1.661
2.41	42.657	42.653	69.74	52.098	66.643	1.804
2.522	91.482	91.403	106.374	96.783	105.984	1.708
2.402	86.77	86.774	124.372	92.465	128.248	2.054
2.434	64.163	64.159	84.092	71.241	81.761	1.554
2.357	57.429	57.43	64.482	59.343	64.54	1.375
2.396	140.837	140.863	217.755	146.805	229.258	2.161
2.421	97.626	97.635	136.101	97.905	147.86	0.964
2.475	60.099	60.073	74.878	61.127	78.037	1.433
2.436	55.766	55.649	100.457	63.682	102.345	2.314
2.558	59.99	59.99	92.769	67.748	94.302	2.679

2.467	126.705	126.727	193.097	127.123	209.705	1.714
2.313	50.678	50.684	65.796	50.765	69.505	0.893
2.609	74.138	74.14	78.435	74.149	79.363	0.5
2.676	129.324	129.334	153.35	129.433	159.02	2.286
2.476	24.545	24.549	38.285	24.649	41.949	1.071
2.493	46.353	46.355	54.815	46.941	56.576	1.411
2.382	50.129	50.103	62.238	52.102	63.993	1.415
2.367	87.359	87.379	139.105	88.17	150.527	1.946
2.26	135.189	135.223	208.457	135.484	224.6	1.286
2.364	69.502	69.499	118.201	80.307	120.747	1.804
2.359	55.158	55.153	84.869	64.482	82.462	1.661
2.463	63.018	63.024	79.483	63.097	83.434	0.643
2.437	131.645	131.625	187.554	133.075	202.772	1.611
2.517	68.009	67.953	82.572	69.973	85.091	1.866
2.395	70.105	70.118	100.256	70.228	107.048	1.321
2.384	154.774	154.791	222.294	155.283	240.261	1.464
2.136	25.795	25.797	28.993	25.804	29.681	0.5
2.418	164.538	164.546	198.166	164.783	207.62	1.464
2.658	237.525	237.55	301.91	237.854	317.179	1.286
2.454	196.982	197.003	250.154	197.239	262.65	1.286
2.796	329.888	329.916	396.44	330.175	411.815	2.571
2.673	86.814	86.824	112.854	86.969	118.927	0.786
2.482	79.21	79.219	89.704	79.257	91.513	1
2.475	76.504	76.51	99.574	76.691	106.106	0.964
2.439	60.594	60.606	95.962	60.797	105.124	1.714
2.446	139.519	139.5	177.758	141.445	186.563	2.093
2.503	78.782	78.786	93.745	78.903	97.85	0.679
2.456	65.471	65.444	78.772	66.554	81.303	1.54

SEigm	SEigv	SEige	SEigp	AEigZ	AEigm	AEigv
1.247	-4.769	1.233	-6.001	107.005	107.023	140.262
1.283	-4.694	1.262	-6.001	224.371	224.414	328.996
1.639	-6.162	1.617	-7.801	197.644	197.671	244.749
0.641	-2.347	0.631	-3	45.526	45.534	67.07
1.462	-5.057	1.429	-6.601	87.941	87.97	162.354
3.113	-6.809	1.682	-7.971	51.807	51.45	100.592
1.604	-5.496	1.567	-7.201	308.423	308.459	453.37
0.82	-2.71	0.798	-3.6	30.183	30.187	49.049
1.232	-1.756	0.762	-2.207	127.283	127.216	156.163
0.641	-2.347	0.631	-3	136.128	136.157	200.307
3.955	-14.596	3.895	-18.603	464.745	464.914	787.302
2.702	-2.458	0.899	-2.859	309.946	309.995	407.295
0.143	-0.439	0.138	-0.6	142.011	142.014	156.149
2.193	-5.648	1.719	-7.008	222.107	222.089	322.193
2.301	-6.006	1.718	-7.408	208.331	208.348	290.049
2.051	-5.052	1.471	-6.208	191.904	191.917	264.672
0.535	-1.832	0.522	-2.4	62.851	62.858	97.832
3.894	-14.699	3.479	-20.999	381.052	379.811	605.499
1.034	-3.74	1.016	-4.801	159.575	159.608	232.88
0.891	-3.301	0.878	-4.201	160.759	160.792	229.508
1.07	-3.664	1.045	-4.8	139.41	139.424	197.53
2.123	-4.9	1.53	-6.207	79.897	79.89	143.87
1.552	-3.301	1.088	-4.008	137.386	137.32	173.846
1.908	-4.613	1.333	-5.608	182.83	182.842	250.508
0.535	-1.832	0.522	-2.4	246.431	246.464	344.799
1.615	-6.21	1.545	-8.144	228.15	228.011	264.338
0.891	-3.301	0.878	-4.201	226.207	226.261	337.7
2.067	-7.48	2.031	-9.601	507.266	507.294	600.084
0.428	-1.317	0.414	-1.8	150.181	150.192	212.178
1.14	-4.255	1.124	-5.401	244.803	244.88	401.185
2.835	-7.838	2.24	-9.808	347.316	347.366	585.808
2.667	-6.248	1.931	-7.978	483.563	483.581	634.975
0.641	-2.347	0.631	-3	130.01	130.027	173.438
0.535	-1.832	0.522	-2.4	162.712	162.724	210.511
1.14	-4.255	1.124	-5.401	296.553	296.582	369.861
1.212	-4.103	1.183	-5.4	327.286	327.311	437.319
0.641	-2.347	0.631	-3	178.681	178.692	212.638
1.07	-3.664	1.045	-4.8	55.17	55.179	91.361
0.748	-2.862	0.74	-3.601	338.554	338.565	359.464
1.283	-4.694	1.262	-6.001	181.538	181.554	224.79
1.604	-5.496	1.567	-7.201	307.676	307.706	400.737
0.535	-1.832	0.522	-2.4	87.854	87.861	116.668
1.746	-6.677	1.726	-8.402	503.26	503.312	603.442
1.303	-2.347	0.841	-2.808	106.172	106.104	126.591
1.283	-4.694	1.262	-6.001	327.959	328.05	524.224
2.712	-5.052	1.681	-6.015	189.099	189.042	265.27
2.301	-6.006	1.718	-7.408	208.933	208.947	290.775
3.354	-7.242	2.202	-8.815	286.151	286.137	421.054
4.016	-7.085	2.303	-8.422	246.312	246.295	392.75

3.354	-7.242	2.202	-8.815	309.967	309.984	465.96
3.71	-8.71	2.558	-10.615	271.281	271.321	443.99
4.388	-10.981	3.218	-13.616	551.221	551.218	781.463
3.71	-8.71	2.558	-10.615	276.115	276.15	442.5
3.425	-7.832	2.282	-9.415	238.801	238.83	377.17
2.301	-6.006	1.718	-7.408	239.27	239.28	325.946
3.817	-9.225	2.666	-11.216	375.977	375.976	529.981
3.853	-9.149	2.696	-11.215	391.154	391.196	624.409
3.212	-6.803	2.065	-8.215	196.79	196.814	309.793
4.764	-9.947	3.043	-12.022	362.29	362.279	572.153
3.191	-9.306	2.595	-11.609	259.661	259.701	425.162
2.656	-7.465	1.915	-10.235	178.223	177.673	264.299
2.051	-5.052	1.471	-6.208	192.38	192.391	265.27
2.677	-4.971	1.542	-5.815	249.265	249.268	349.268
3.033	-6.439	1.897	-7.615	222.867	222.888	338.799
2.051	-5.052	1.471	-6.208	198.597	198.608	271.178
1.267	-2.266	0.702	-2.607	221.612	221.649	306.747
1.695	-3.74	1.225	-4.608	264.533	264.5	383.037
3.338	-4.971	1.752	-5.622	52.629	52.542	101.836
0.947	-0.878	0.486	-1.007	125.488	125.418	147.021
1.572	-0.797	0.557	-0.614	134.908	134.84	183.766
2.32	-3.659	1.296	-4.215	88.061	87.98	150.816
1.429	-0.358	0.419	-0.014	131.891	131.816	156.646
2.57	-4.613	1.543	-5.415	120.917	120.842	163.151
1.303	-2.347	0.841	-2.808	100.193	100.124	124.037
1.482	-2.553	0.899	-3.207	114.555	114.548	176.837
1.283	-4.694	1.262	-6.001	227.459	227.511	345.46
1.246	-4.76	1.075	-7.028	254.131	253.568	314.802
1.532	-5.648	1.509	-7.201	101.847	101.871	158.171
1.544	-5.619	1.466	-7.543	167.885	167.742	235.875
2.819	-5.567	1.79	-6.615	247.878	247.849	347.903
1.409	-2.862	0.95	-3.408	66.729	66.679	113.909
1.801	-4.255	1.334	-5.208	175.087	175.026	228.104
1.751	-1.317	0.833	-1.415	61.727	61.604	95.181
1.232	-1.756	0.762	-2.207	145.797	145.733	205.412
2.719	-2.632	1.097	-2.7	56.414	56.007	114.028
1.41	-2.705	0.84	-3.207	72.841	72.828	103.824
0.285	-0.878	0.276	-1.2	129.676	129.683	164.292
1.615	-6.21	1.545	-8.144	228.15	228.011	264.338
1.196	-1.832	0.732	-2.208	111.276	111.209	136.409
1.964	-2.347	1.051	-2.615	112.433	112.31	151.417
3.623	-6.006	2.137	-7.023	258.498	258.371	354.323
1.319	-4.618	1.291	-6.001	93.013	93.039	160.236
1.484	-5.742	1.365	-7.886	99.097	98.744	135.633
0.997	-3.816	0.986	-4.801	239.025	239.038	264.921
3.242	-12.401	3.206	-15.603	964.066	964.4	1442.733
1.908	-4.613	1.333	-5.608	234.252	234.284	344.363
0.392	-1.393	0.385	-1.8	128.883	128.907	182.797
0.678	-2.271	0.66	-3	194.028	194.037	230.126
3.634	-13.793	3.59	-17.403	777.698	777.882	1151.331

2.926	-5.925	1.789	-7.015	319.163	319.196	498.814
1.874	-4.103	1.393	-5.208	328.596	328.544	449.045
2.28	-8.509	2.249	-10.802	254.64	254.665	313.1
1.675	-6.087	1.647	-7.801	152.721	152.739	208.682
0.784	-2.786	0.769	-3.6	102.857	102.87	147.12
0.891	-3.301	0.878	-4.201	130.698	130.712	159.927
3.242	-12.401	3.206	-15.603	434.407	434.55	682.667
3.374	-4.738	1.672	-5.421	133.643	133.627	221.984
0.748	-2.862	0.74	-3.601	140.657	140.664	155.019
0.748	-2.862	0.74	-3.601	110.71	110.738	162.081
0.535	-1.832	0.522	-2.4	237.918	237.933	301.814
1.928	-2.266	0.912	-2.415	141.603	141.527	171.125
0.784	-2.786	0.769	-3.6	248.801	248.81	281.211
3.241	-12.381	2.891	-17.657	267.306	266.258	456.114
1.842	-6.469	1.699	-9.086	142.89	142.542	214.901
0.867	-3.348	0.806	-4.543	101.282	101.087	118.904
1.544	-5.619	1.466	-7.543	135.245	135.079	208.834
1.152	-4.226	1.082	-5.743	26.054	25.893	51.527
2.407	-6.511	1.669	-9.035	299.481	298.954	429.386
0.867	-3.348	0.806	-4.543	110.123	109.975	129.723
1.859	-4.616	1.142	-5.486	9.208	9.077	34.592
3.213	-9.982	2.373	-13.921	679.632	678.61	786.563
2.819	-5.567	1.79	-6.615	124.544	124.489	190.037
1.496	-5.723	1.48	-7.201	391.592	391.623	453.877
0.641	-2.347	0.631	-3	61.883	61.889	74.47
0.891	-3.301	0.878	-4.201	124.194	124.217	176.384
1.711	-6.011	1.676	-7.801	110.06	110.098	203.091
0.748	-2.862	0.74	-3.601	134.883	134.911	188.474
2.459	-8.872	2.416	-11.402	307.895	308.019	531.208
2.443	-6.445	1.856	-8.008	418.003	418.006	598.7
2.962	-6.006	1.928	-7.215	376.007	375.934	536.347
0.82	-2.71	0.798	-3.6	179.031	179.043	230.903
2.905	-8.575	2.272	-11.636	252.314	251.758	424.982
3.338	-4.971	1.752	-5.622	54.278	54.193	104.12
0.891	-3.301	0.878	-4.201	119.588	119.602	153.2
0.499	-1.908	0.493	-2.4	93.535	93.54	103.619
1.249	-4.027	1.212	-5.4	463.358	463.391	614.597
1.801	-4.255	1.334	-5.208	173.402	173.342	232.842
0.891	-3.301	0.878	-4.201	148.633	148.646	187.405
1.106	-3.588	1.074	-4.8	328.663	328.677	391.849
2.28	-8.509	2.249	-10.802	85.816	85.864	208.557
1.639	-6.162	1.617	-7.801	58.204	58.234	131.847
1.675	-6.087	1.647	-7.801	200.895	200.933	286.417
1.409	-2.862	0.95	-3.408	158.883	158.821	176.546
0.748	-2.862	0.74	-3.601	124.282	124.289	138.267
1.802	-4.098	1.225	-5.008	77.362	77.386	141.318
2.656	-7.465	1.915	-10.235	175.75	175.155	303.829
0.714	-2.195	0.69	-3	148.97	148.975	174.844
0.784	-2.786	0.769	-3.6	55.817	55.824	80.025
1.794	-6.573	1.713	-8.744	186.805	186.671	271.133

0.535	-1.832	0.522	-2.4	111.805	111.815	153.354
1.794	-6.573	1.713	-8.744	205.419	205.293	305.624
1.912	-7.059	1.779	-9.686	177.785	177.495	254.241
2.107	-2.786	1.189	-3.215	116.196	116.062	149.709
1.767	-3.588	1.284	-4.608	283.323	283.268	343.136
2.677	-4.971	1.542	-5.815	126.508	126.507	202.92
2.107	-2.786	1.189	-3.215	140.279	140.139	184.131
0.641	-2.347	0.631	-3	111.562	111.578	148.706
0.428	-1.317	0.414	-1.8	113.492	113.498	144.885
0.535	-1.832	0.522	-2.4	116.015	116.024	151.754
1.196	-1.675	0.623	-2.007	30.131	30.134	47.086
0.714	-2.195	0.69	-3	32.35	32.355	56.965
0.392	-1.393	0.385	-1.8	160.828	160.833	177.607
2.407	-6.52	1.826	-8.008	204.668	204.699	312.902
2.389	-8.282	2.336	-10.801	374.077	374.114	510.665
1.247	-4.769	1.233	-6.001	220.89	220.904	248.439
1.588	-3.225	1.117	-4.008	151.936	151.889	219.513
0.891	-3.301	0.878	-4.201	163.986	164.025	247.777
1.176	-4.179	1.154	-5.401	46.889	46.9	84.381
1.457	-2.757	0.936	-3.75	136.644	136.393	172.71
2.178	-3.22	1.158	-3.615	494.616	494.523	560.212
0.891	-3.301	0.878	-4.201	118.595	118.611	156.452
1.794	-6.573	1.713	-8.744	229.804	229.676	326.467
0.891	-3.301	0.878	-4.201	141.185	141.228	230.523
1.14	-4.255	1.124	-5.401	184.929	184.97	275.046
2.158	-5.567	1.58	-6.808	242.98	243.007	349.391
1.034	-3.74	1.016	-4.801	86.335	86.347	118.542
0.891	-3.301	0.878	-4.201	174.54	174.556	220.111
0.891	-3.301	0.878	-4.201	145.926	145.94	184.075
0.748	-2.862	0.74	-3.601	96.09	96.105	124.716
0.784	-2.786	0.769	-3.6	87.801	87.828	154.88
2.026	-3.902	1.3	-4.978	283.587	283.602	391.759
1.782	-6.601	1.755	-8.401	168.324	168.353	234.914
2.158	-5.567	1.58	-6.808	117.027	117.052	213.984
1.782	-6.601	1.755	-8.401	189.957	189.991	266.217
1.818	-6.526	1.785	-8.401	94.087	94.114	181.789
0.891	-3.301	0.878	-4.201	52.943	52.953	74.204
1.544	-5.619	1.466	-7.543	157.676	157.533	221.968
0.143	-0.439	0.138	-0.6	127.927	127.929	137.388
3.028	-11.371	2.988	-14.402	563.991	564.128	839.746
1.794	-6.573	1.713	-8.744	186.805	186.671	271.133
1.802	-4.098	1.225	-5.008	153.759	153.76	242.356
0.678	-2.271	0.66	-3	135.008	135.015	164.228
2.301	-6.006	1.718	-7.408	238.243	238.259	323.848
1.445	-2.786	0.979	-3.408	110.175	110.111	140.088
1.14	-4.255	1.124	-5.401	137.485	137.509	190.072
1.034	-3.74	1.016	-4.801	175.922	175.939	242.454
1.283	-4.694	1.262	-6.001	487.443	487.496	626.429
1.034	-3.74	1.016	-4.801	92.384	92.392	115.568
0.641	-2.347	0.631	-3	66.92	66.936	106.104

0.143	-0.439	0.138	-0.6	111.018	111.022	129.945
1.034	-3.74	1.016	-4.801	89.255	89.263	113.514
0.784	-2.786	0.769	-3.6	163.001	163.014	219.742
0.784	-2.786	0.769	-3.6	103.978	103.984	124.83
0.784	-2.786	0.769	-3.6	126.164	126.194	193.299
2.427	-4.017	1.296	-4.614	316.597	316.598	438.729
1.212	-4.103	1.183	-5.4	198.358	198.378	284.84
2.158	-5.567	1.58	-6.808	155.911	155.94	252.405
1.303	-2.347	0.841	-2.808	100.75	100.7	130.923
1.034	-3.74	1.016	-4.801	156.668	156.703	241.761
1.746	-6.677	1.726	-8.402	332.786	332.831	418.903
1.711	-6.011	1.676	-7.801	243.948	243.992	379.077
1.247	-4.769	1.233	-6.001	208.575	208.588	233.407
0.499	-1.908	0.493	-2.4	179.88	179.886	191.107
0.678	-2.271	0.66	-3	125.466	125.476	170.037
0.784	-2.786	0.769	-3.6	121.864	121.892	187.422
0.891	-3.301	0.878	-4.201	252.754	252.791	335.196
0.641	-2.347	0.631	-3	137.357	137.387	204.632
0.249	-0.954	0.247	-1.2	61.996	61.999	67.828
0.641	-2.347	0.631	-3	137.357	137.387	204.632
1.232	-1.756	0.762	-2.207	87.463	87.39	106.373
1.552	-3.144	0.978	-3.807	242.28	242.316	365.062
0.784	-2.786	0.769	-3.6	182.137	182.154	220.58
0.784	-2.786	0.769	-3.6	182.137	182.154	220.58
1.945	-4.537	1.362	-5.608	171.669	171.677	282.064
1.283	-4.694	1.262	-6.001	353.426	353.461	456.773
2.264	-6.072	1.531	-8.435	63.732	63.204	136.138
3.113	-6.809	1.682	-7.971	70.295	69.894	121.989
1.438	-5.104	1.357	-6.943	323.121	322.938	405.858
0.714	-2.195	0.69	-3	139.796	139.804	179.081
1.283	-4.694	1.262	-6.001	430.219	430.258	541.912
0.535	-1.832	0.522	-2.4	144.3	144.311	188.664
0.748	-2.862	0.74	-3.601	33.107	33.115	48.505
1.14	-4.255	1.124	-5.401	173.492	173.529	250.866
2.479	-6.369	1.885	-8.008	273.711	273.717	411.485
1.659	-3.659	1.087	-4.408	114.699	114.701	167.83
1.41	-2.705	0.84	-3.207	246.494	246.514	331.77
1.802	-4.098	1.225	-5.008	76.433	76.425	131.965
1.695	-3.583	1.116	-4.407	123.482	123.473	202.241
1.659	-3.659	1.087	-4.408	235.048	235.04	323.389
1.802	-4.098	1.225	-5.008	84.431	84.425	143.759
1.741	-4.221	1.123	-5.35	182.038	181.848	216.969
2.051	-5.052	1.471	-6.208	172.407	172.418	253.797
1.552	-3.144	0.978	-3.807	127.689	127.683	171.329
1.374	-2.781	0.811	-3.208	114.207	114.213	131.745
2.158	-5.567	1.58	-6.808	280.433	280.489	441.077
0.963	-3.149	0.936	-4.2	194.288	194.306	275.351
1.445	-2.786	0.979	-3.408	118.764	118.701	152.543
2.323	-5.857	1.585	-7.286	110.058	109.841	206.771
2.677	-4.971	1.542	-5.815	119.065	119.07	190.509

1.711	-6.011	1.676	-7.801	251.888	251.934	392.39
0.891	-3.301	0.878	-4.201	100.464	100.477	134.893
0.499	-1.908	0.493	-2.4	147.776	147.782	158.777
2.28	-8.509	2.249	-10.802	256.363	256.388	315.21
1.07	-3.664	1.045	-4.8	48.125	48.133	80.234
1.41	-2.705	0.84	-3.207	91.591	91.597	112.335
1.429	-0.358	0.419	-0.014	99.617	99.553	124.834
1.945	-4.537	1.362	-5.608	174.116	174.159	282.748
1.283	-4.694	1.262	-6.001	269.093	269.163	421.607
1.802	-4.098	1.225	-5.008	138.121	138.118	240.499
1.659	-3.659	1.087	-4.408	109.576	109.57	173.397
0.641	-2.347	0.631	-3	125.393	125.407	161.312
1.624	-3.149	1.146	-4.008	261.887	261.834	378.258
1.893	-1.756	0.971	-2.015	134.152	134.013	166.9
1.319	-4.618	1.291	-6.001	138.888	138.917	205.13
1.462	-5.057	1.429	-6.601	308.276	308.313	449.646
0.499	-1.908	0.493	-2.4	51.091	51.095	59.894
1.462	-5.057	1.429	-6.601	327.611	327.63	401.39
1.283	-4.694	1.262	-6.001	473.832	473.885	608.513
1.283	-4.694	1.262	-6.001	392.678	392.723	505.002
2.566	-9.387	2.524	-12.002	657.953	658.016	802.671
0.784	-2.786	0.769	-3.6	172.928	172.95	228.495
0.997	-3.816	0.986	-4.801	157.421	157.441	183.592
0.963	-3.149	0.936	-4.2	152.054	152.066	202.297
1.711	-6.011	1.676	-7.801	119.473	119.5	198.112
2.107	-2.629	1.079	-3.014	278.236	278.186	358.145
0.678	-2.271	0.66	-3	156.951	156.959	189.761
1.552	-3.301	1.088	-4.008	129.403	129.335	160.845

AEige	AEigp	VEA1	VEA2	VRA1	VRA2	VED1
107.097	147.484	3.389	0.199	16.442	0.967	4.042
224.88	353.289	3.797	0.158	23.453	0.977	4.804
197.859	256.397	4.288	0.165	27.414	1.054	5.012
45.653	72.362	3.096	0.281	10.491	0.954	3.267
88.346	179.769	3.414	0.213	16.434	1.027	3.928
75.568	84.666	3.178	0.227	12.44	0.889	3.676
309.468	493.263	4.223	0.141	31.422	1.047	5.387
30.305	54.808	3.026	0.303	10.459	1.046	3.136
129.806	162.35	4.169	0.19	24.407	1.109	4.621
136.395	214.638	3.473	0.193	17.478	0.971	4.174
465.965	855.446	5.326	0.133	41.403	1.035	6.218
316.136	427.192	4.265	0.138	32.411	1.046	5.463
142.127	160.184	3.942	0.188	22.425	1.068	4.506
224.588	341.806	4.25	0.152	28.409	1.015	5.186
218.163	305.253	3.651	0.146	26.391	1.056	4.927
201.427	278.145	3.641	0.152	25.391	1.058	4.829
63.095	109.097	3.368	0.241	14.441	1.031	3.691
382.563	669.705	5.397	0.146	39.406	1.065	5.992
159.888	249.298	3.633	0.191	18.477	0.972	4.284
161.003	244.459	3.746	0.178	22.433	1.068	4.523
139.835	214.594	4.025	0.183	23.392	1.063	4.607
100.904	144.731	4.073	0.214	20.421	1.075	4.306
140.048	180.353	3.995	0.19	22.423	1.068	4.508
192.003	262.806	3.574	0.155	24.392	1.061	4.725
247.024	369.087	4.483	0.166	28.443	1.053	5.109
228.318	274.06	4.552	0.163	30.352	1.084	5.204
226.613	361.726	4.07	0.185	22.465	1.021	4.605
507.871	624.482	5.497	0.128	45.397	1.056	6.449
150.641	231.951	3.844	0.175	23.418	1.064	4.606
245.336	434.41	3.711	0.161	22.479	0.977	4.708
382.148	605.496	5.187	0.133	41.409	1.062	6.134
487.77	671.511	4.587	0.107	48.366	1.125	6.473
130.231	184.17	3.629	0.191	19.433	1.023	4.293
163.114	223.425	4.111	0.196	21.44	1.021	4.512
296.88	387.91	4.752	0.14	39.327	1.157	5.729
328.173	467.427	3.617	0.129	30.428	1.087	5.222
178.904	221.281	4.12	0.172	27.374	1.141	4.811
55.422	100.948	3.456	0.247	14.418	1.03	3.695
338.611	363.926	3.86	0.129	31.411	1.047	5.386
181.791	236.363	4.534	0.174	29.379	1.13	5.028
308.22	424.938	4.286	0.13	36.413	1.103	5.664
88.064	125.207	3.892	0.216	19.422	1.079	4.19
503.538	624.349	5.032	0.123	44.35	1.082	6.271
108.643	129.554	3.813	0.201	20.414	1.074	4.29
328.718	567.229	3.673	0.122	32.418	1.081	5.411
198.322	276.004	3.72	0.155	25.407	1.059	4.829
216.187	304.181	3.738	0.15	26.407	1.056	4.926
322.236	419.899	3.928	0.127	33.403	1.078	5.474
287.871	393.803	3.826	0.132	31.405	1.083	5.287

323.905	490.385	4.164	0.13	34.398	1.075	5.568
281.961	479.65	3.879	0.129	31.404	1.047	5.387
595.127	796.625	4.142	0.094	47.402	1.077	6.522
288.671	469.25	3.932	0.131	31.403	1.047	5.382
253.214	392.584	3.814	0.136	29.389	1.05	5.19
248.158	337.283	3.817	0.141	28.404	1.052	5.11
400.994	534.921	4.203	0.117	38.4	1.067	5.899
406.675	667.359	4.092	0.111	39.402	1.065	5.984
205.085	333.895	3.757	0.15	26.412	1.056	4.927
410.808	580.222	4.057	0.113	38.403	1.067	5.903
270.21	452.845	3.899	0.134	30.403	1.048	5.292
190.698	279.632	4.351	0.167	27.413	1.054	5.009
199.433	276.938	3.72	0.155	25.407	1.059	4.829
264.128	356.891	3.855	0.138	30.404	1.086	5.198
236.498	350.206	3.753	0.144	27.405	1.054	5.005
205.615	282.801	3.72	0.155	25.407	1.059	4.829
223.334	324.927	3.856	0.161	25.448	1.06	4.831
267.533	410.115	4.225	0.156	28.434	1.053	5.093
76.001	87.583	3.788	0.223	17.415	1.024	4.072
128.005	150.961	3.842	0.202	19.452	1.024	4.283
144.439	189.993	3.882	0.185	22.418	1.068	4.503
107.842	145.508	3.496	0.206	16.457	0.968	4.049
141.658	155.549	3.882	0.185	22.418	1.068	4.503
134.245	159.274	4.04	0.184	23.4	1.064	4.628
102.769	127.69	3.765	0.209	19.42	1.079	4.178
133.245	178.789	3.055	0.18	16.491	0.97	4.068
227.951	371.818	3.884	0.155	26.44	1.058	4.919
254.731	335.79	3.772	0.145	27.444	1.056	5.042
102.148	171.288	3.956	0.208	20.408	1.074	4.295
168.444	254.262	4.254	0.177	26.398	1.1	4.819
257.309	363.786	4.466	0.165	27.418	1.015	5.115
69.178	121.935	3.448	0.246	13.464	0.962	3.689
177.898	238.693	4.047	0.162	26.406	1.056	4.906
66.082	100.987	3.497	0.25	14.458	1.033	3.697
148.654	220.138	4.325	0.188	25.415	1.105	4.721
77.411	103.859	3.241	0.232	13.462	0.962	3.675
91.492	94.451	3.683	0.217	17.44	1.026	4.057
129.966	174.408	3.911	0.196	21.426	1.071	4.393
228.318	274.06	4.552	0.163	30.352	1.084	5.204
113.737	141.346	4.014	0.201	21.423	1.071	4.408
116.908	157.78	3.95	0.208	19.444	1.023	4.298
274.23	369.486	3.916	0.131	32.388	1.08	5.407
93.376	176.379	3.727	0.219	18.422	1.084	4.061
99.362	147.388	3.895	0.216	18.435	1.024	4.181
239.094	270.756	4.421	0.164	30.367	1.125	5.106
965.853	1533.175	4.528	0.084	60.367	1.118	7.234
244.901	364.245	4.456	0.154	30.412	1.049	5.28
129.106	195.038	3.748	0.197	19.458	1.024	4.285
194.328	239.804	3.997	0.16	25.424	1.017	4.923
778.943	1231.31	5.138	0.089	62.347	1.075	7.489

343.162	510.636	5.043	0.174	29.448	1.015	5.305
332.023	479.853	4.061	0.135	33.419	1.114	5.407
254.892	326.664	4.786	0.15	34.361	1.074	5.577
153.039	223.606	3.97	0.18	21.44	0.975	4.623
103.159	158.513	3.487	0.249	12.512	0.894	3.685
130.837	166.712	3.958	0.188	22.397	1.067	4.509
435.174	732.977	5.12	0.122	47.378	1.128	6.375
167.462	211.542	3.485	0.174	19.478	0.974	4.409
140.697	158.04	3.823	0.201	19.453	1.024	4.285
110.855	172.657	3.84	0.213	18.456	1.025	4.184
238.438	319.303	4.09	0.164	26.443	1.058	4.92
149.928	171.784	4.017	0.183	23.428	1.065	4.607
249.032	289.74	4.418	0.164	29.377	1.088	5.106
268.569	509.104	4.244	0.152	28.444	1.016	5.195
143.492	238.334	4.092	0.186	23.427	1.065	4.615
101.421	124.971	3.697	0.205	19.416	1.079	4.179
135.699	228.074	4.111	0.187	23.405	1.064	4.609
26.26	59.253	2.863	0.318	8.49	0.943	2.964
307.13	463.462	4.219	0.141	32.409	1.08	5.382
110.246	135.92	3.821	0.212	18.442	1.025	4.175
18.194	29.04	2.454	0.351	5.527	0.79	2.607
718.921	788.489	4.226	0.103	43.385	1.058	6.321
137.349	192.906	3.85	0.183	21.431	1.021	4.511
391.758	467.407	4.673	0.126	39.356	1.064	5.963
61.935	77.321	3.114	0.259	11.472	0.956	3.41
124.382	189.199	3.995	0.19	23.385	1.114	4.508
110.537	224.527	3.506	0.195	18.433	1.024	4.177
135.034	199.492	3.876	0.215	17.463	0.97	4.192
308.706	577.397	4.83	0.156	32.407	1.045	5.488
452.09	611.223	3.759	0.111	35.444	1.042	5.744
410.962	541.541	4.587	0.139	34.443	1.044	5.662
179.449	245.738	4.249	0.185	25.417	1.105	4.738
255.501	466.051	4.486	0.16	28.433	1.015	5.195
77.609	90.48	3.788	0.223	17.415	1.024	4.072
119.73	161.707	4.115	0.196	24.363	1.16	4.524
93.562	105.759	3.489	0.233	14.466	0.964	3.807
464.637	656.07	5.264	0.142	40.428	1.093	5.985
176.257	245.021	4.047	0.162	26.406	1.056	4.906
148.879	197.2	3.881	0.185	22.422	1.068	4.513
329.157	410.067	3.938	0.123	35.411	1.107	5.574
86.178	242.816	3.407	0.213	16.43	1.027	3.927
58.415	151.739	3.215	0.247	13.437	1.034	3.55
201.22	307.171	4.437	0.164	28.4	1.052	5.114
161.122	178.597	4.017	0.191	21.443	1.021	4.512
124.32	141.234	3.951	0.208	19.445	1.023	4.297
80.651	155.135	3.568	0.238	15.445	1.03	3.811
202.631	317.469	4.216	0.169	26.429	1.057	4.928
149.167	182.939	4.133	0.188	23.432	1.065	4.6
55.984	86.308	3.148	0.262	11.466	0.955	3.413
187.443	293.198	4.359	0.168	28.389	1.092	5.011

112.138	164.679	3.506	0.206	16.465	0.969	4.065
206.139	332.827	4.211	0.175	25.424	1.059	4.811
178.44	276.065	4.273	0.171	26.4	1.056	4.918
120.923	154.654	4.149	0.198	22.419	1.068	4.52
285.916	358.309	4.784	0.159	32.417	1.081	5.394
146.967	205.208	3.942	0.171	24.404	1.061	4.726
145.306	191.467	3.782	0.199	18.461	0.972	4.28
111.712	157.111	3.654	0.203	18.432	1.024	4.164
113.747	154.399	4.111	0.206	22.409	1.12	4.414
116.32	161.303	3.95	0.219	18.444	1.025	4.192
31.5	50.411	3.026	0.303	10.459	1.046	3.136
32.558	63.774	2.775	0.308	7.527	0.836	2.955
160.947	182.201	3.962	0.172	23.421	1.018	4.717
214.88	333.427	3.753	0.144	27.39	1.053	5.03
375.088	546.751	4.238	0.128	34.431	1.043	5.657
220.965	254.309	4.508	0.167	29.363	1.088	5.111
154.477	235.223	3.552	0.178	19.451	0.973	4.402
164.309	266.104	3.593	0.189	18.48	0.973	4.279
47.108	94.702	3.081	0.257	11.461	0.955	3.403
139.256	182.41	4.282	0.186	25.404	1.105	4.728
527.646	548.933	3.887	0.095	44.446	1.084	6.305
118.744	165.795	4.115	0.196	24.363	1.16	4.524
230.531	351.458	4.665	0.161	32.384	1.117	5.299
141.509	249.977	3.667	0.183	19.445	0.972	4.386
185.291	294.953	3.701	0.168	22.436	1.02	4.624
253.947	369.298	3.867	0.133	31.389	1.082	5.31
86.577	126.177	3.697	0.217	17.423	1.025	4.057
174.781	231.86	4.29	0.172	29.351	1.174	4.914
146.127	194.017	4.157	0.181	26.354	1.146	4.715
96.168	130.776	3.724	0.219	17.438	1.026	4.052
88.145	170.636	3.537	0.221	15.457	0.966	3.945
287.443	413.727	4.248	0.137	34.378	1.109	5.486
168.593	250.78	4.24	0.17	25.408	1.016	4.923
127.511	223.591	4.025	0.192	21.442	1.021	4.508
190.256	284.021	4.223	0.162	26.412	1.016	5.012
94.561	206.814	3.876	0.228	17.446	1.026	4.067
53.02	78.871	3.196	0.266	11.47	0.956	3.411
158.205	239.29	4.149	0.18	24.409	1.061	4.717
128.004	140.1	3.911	0.196	21.426	1.071	4.393
565.087	900.16	5.3	0.12	46.393	1.054	6.523
187.443	293.198	4.359	0.168	28.389	1.092	5.011
180.075	241.425	4.317	0.18	25.422	1.059	4.827
135.235	172.189	3.862	0.184	23.403	1.114	4.515
248.916	340.38	3.837	0.137	30.389	1.085	5.219
112.66	145.916	4.037	0.202	21.424	1.071	4.408
137.679	202.599	4.202	0.183	26.352	1.146	4.724
176.362	262.119	3.989	0.166	25.41	1.059	4.802
488.153	659.46	5.13	0.125	45.362	1.106	6.296
92.525	121.369	3.443	0.215	15.429	0.964	3.928
67.102	115.082	3.261	0.251	12.483	0.96	3.538

111.178	135.229	3.727	0.207	19.429	1.079	4.179
89.403	119.577	3.609	0.212	17.422	1.025	4.06
163.401	237.077	3.925	0.171	24.411	1.061	4.704
104.107	130.319	3.742	0.197	20.412	1.074	4.291
126.456	208.389	3.37	0.187	18.45	1.025	4.183
341.69	446.014	4.375	0.137	34.405	1.075	5.557
199.057	308.272	4.281	0.165	28.412	1.093	5.021
166.036	263.361	4.236	0.169	26.415	1.057	4.92
102.832	137.157	3.759	0.221	17.44	1.026	4.059
157.08	261.421	3.633	0.191	18.477	0.972	4.284
333.025	436.865	4.065	0.135	30.421	1.014	5.381
244.804	413.243	4.433	0.158	30.415	1.086	5.207
208.643	238.665	4.418	0.17	28.366	1.091	5.014
179.91	193.498	4.29	0.187	25.379	1.103	4.717
125.824	182.188	3.452	0.203	16.478	0.969	4.061
122.172	202.461	3.452	0.203	16.478	0.969	4.061
253.082	353.56	3.921	0.157	25.461	1.018	4.91
137.636	219.623	3.511	0.185	19.452	1.024	4.295
62.011	69.127	3.285	0.253	12.458	0.958	3.554
137.636	219.623	3.511	0.185	19.452	1.024	4.295
90.025	109.638	3.708	0.218	17.438	1.026	4.057
248.951	392.325	4.328	0.16	29.417	1.09	5.092
182.419	228.936	4.428	0.184	26.405	1.1	4.824
182.419	228.936	4.428	0.184	26.405	1.1	4.824
194.964	292.567	3.337	0.159	20.479	0.975	4.516
354.061	482.623	4.752	0.144	34.432	1.043	5.661
68.63	154.147	3.508	0.234	15.44	1.029	3.819
96.589	104.125	3.459	0.203	16.431	0.967	4.068
323.793	430.693	4.798	0.16	33.415	1.114	5.405
140.112	190.658	3.736	0.187	21.435	1.072	4.398
430.849	569.398	4.975	0.131	42.364	1.115	6.061
144.674	200.619	4.079	0.204	20.44	1.022	4.41
33.149	51.72	2.965	0.296	9.486	0.949	3.122
173.756	267.642	3.294	0.15	24.397	1.109	4.629
301.927	417.022	4.791	0.145	35.397	1.073	5.648
124.542	168.131	3.718	0.207	17.459	0.97	4.175
251.456	350.079	4.078	0.151	28.423	1.053	5.102
94.017	127.45	3.773	0.222	17.444	1.026	4.062
145.547	198.93	4.206	0.191	23.427	1.065	4.622
261.045	326.885	4.073	0.14	31.41	1.083	5.295
102.971	138.974	3.885	0.216	18.434	1.024	4.182
192.443	217.317	3.928	0.157	26.413	1.057	4.911
183.46	262.705	4.066	0.177	23.436	1.019	4.728
141.503	167.329	3.751	0.188	20.433	1.022	4.397
117.876	132.288	3.912	0.217	18.444	1.025	4.178
292.03	465.324	3.669	0.131	28.446	1.016	5.223
194.873	299.92	3.465	0.157	22.441	1.02	4.61
121.275	159.483	4.093	0.195	22.416	1.067	4.517
125.779	212.019	3.518	0.185	19.438	1.023	4.299
134.004	194.419	3.817	0.174	23.402	1.064	4.627

252.756	427.831	4.433	0.158	30.415	1.086	5.207
100.652	143.211	3.578	0.224	15.462	0.966	3.947
147.806	161.127	4.183	0.199	23.382	1.113	4.516
256.617	328.842	4.857	0.152	34.36	1.074	5.579
48.342	88.698	3.364	0.259	13.426	1.033	3.564
93.042	116.359	3.443	0.215	15.429	0.964	3.928
103.785	128.001	3.76	0.221	18.442	1.085	4.075
175.176	306.662	3.65	0.174	21.453	1.022	4.518
269.706	455.201	4.027	0.161	25.474	1.019	4.935
159.39	246.707	3.783	0.18	21.453	1.022	4.519
127.877	169.332	3.512	0.195	17.457	0.97	4.165
125.562	169.869	3.739	0.197	19.431	1.023	4.288
265.201	409.551	3.574	0.137	28.439	1.094	5.033
138.975	172.196	4.282	0.186	25.404	1.105	4.728
139.165	220.096	4.06	0.193	21.436	1.021	4.512
309.322	487.123	3.99	0.142	29.447	1.052	5.226
51.115	61.763	2.822	0.282	8.525	0.852	3.103
328.136	421.84	4.781	0.149	33.424	1.045	5.572
474.508	640.36	4.993	0.125	44.365	1.109	6.215
393.216	531.302	3.932	0.119	33.431	1.013	5.662
658.579	836.752	5.158	0.09	64.334	1.129	7.444
173.252	241.455	4.373	0.168	29.366	1.129	5.016
157.528	188.957	4.121	0.179	24.416	1.062	4.723
152.454	216.514	4.076	0.177	24.418	1.062	4.726
119.918	218.668	3.877	0.204	19.427	1.022	4.284
281.996	376.172	4.719	0.169	31.419	1.122	5.223
157.206	198.7	4.003	0.174	24.415	1.062	4.712
132.021	166.613	3.784	0.189	20.429	1.021	4.396

VED2	VRD1	VRD2	VEZ1	VEZ2	VRZ1	VRZ2
0.238	16.588	0.976	4.031	0.237	16.587	0.976
0.2	23.561	0.982	4.786	0.199	23.564	0.982
0.193	27.522	1.059	5.026	0.193	27.515	1.058
0.297	10.581	0.962	3.262	0.297	10.583	0.962
0.245	16.546	1.034	3.917	0.245	16.546	1.034
0.263	12.643	0.903	3.633	0.259	12.623	0.902
0.18	31.517	1.051	5.393	0.18	31.514	1.05
0.314	10.527	1.053	3.145	0.314	10.515	1.051
0.21	24.491	1.113	4.627	0.21	24.487	1.113
0.232	17.563	0.976	4.167	0.232	17.564	0.976
0.155	41.531	1.038	6.216	0.155	41.529	1.038
0.176	32.523	1.049	5.438	0.175	32.523	1.049
0.215	22.514	1.072	4.505	0.215	22.514	1.072
0.185	28.555	1.02	5.186	0.185	28.551	1.02
0.197	26.523	1.061	4.93	0.197	26.517	1.061
0.201	25.521	1.063	4.831	0.201	25.515	1.063
0.264	14.543	1.039	3.693	0.264	14.542	1.039
0.162	39.51	1.068	6.001	0.162	39.506	1.068
0.225	18.56	0.977	4.275	0.225	18.561	0.977
0.215	22.503	1.072	4.525	0.215	22.499	1.071
0.209	23.538	1.07	4.586	0.208	23.54	1.07
0.227	20.512	1.08	4.298	0.226	20.514	1.08
0.215	22.516	1.072	4.501	0.214	22.517	1.072
0.205	24.517	1.066	4.727	0.206	24.511	1.066
0.189	28.511	1.056	5.112	0.189	28.511	1.056
0.186	30.523	1.09	5.219	0.186	30.515	1.09
0.209	22.516	1.023	4.592	0.209	22.518	1.024
0.15	45.517	1.059	6.447	0.15	45.518	1.059
0.209	23.518	1.069	4.594	0.209	23.521	1.069
0.205	22.55	0.98	4.696	0.204	22.554	0.981
0.157	41.51	1.064	6.104	0.157	41.515	1.064
0.151	48.474	1.127	6.474	0.151	48.472	1.127
0.226	19.545	1.029	4.278	0.225	19.553	1.029
0.215	21.537	1.026	4.504	0.214	21.538	1.026
0.169	39.482	1.161	5.732	0.169	39.48	1.161
0.186	30.475	1.088	5.228	0.187	30.47	1.088
0.2	27.476	1.145	4.812	0.201	27.474	1.145
0.264	14.564	1.04	3.698	0.264	14.558	1.04
0.18	31.523	1.051	5.383	0.179	31.522	1.051
0.193	29.482	1.134	5.03	0.193	29.481	1.134
0.172	36.483	1.106	5.667	0.172	36.482	1.106
0.233	19.51	1.084	4.196	0.233	19.507	1.084
0.153	44.512	1.086	6.282	0.153	44.507	1.086
0.226	20.519	1.08	4.284	0.225	20.52	1.08
0.18	32.48	1.083	5.421	0.181	32.479	1.083
0.201	25.517	1.063	4.833	0.201	25.507	1.063
0.197	26.52	1.061	4.93	0.197	26.513	1.061
0.177	33.496	1.081	5.483	0.177	33.493	1.08
0.182	31.493	1.086	5.3	0.183	31.49	1.086

0.174	34.504	1.078	5.569	0.174	34.499	1.078
0.18	31.528	1.051	5.394	0.18	31.52	1.051
0.148	47.495	1.079	6.536	0.149	47.491	1.079
0.179	31.528	1.051	5.39	0.18	31.521	1.051
0.185	29.524	1.054	5.195	0.186	29.517	1.054
0.189	28.522	1.056	5.116	0.189	28.517	1.056
0.164	38.507	1.07	5.903	0.164	38.498	1.069
0.162	39.507	1.068	5.993	0.162	39.504	1.068
0.197	26.523	1.061	4.932	0.197	26.513	1.061
0.164	38.506	1.07	5.912	0.164	38.502	1.069
0.182	30.526	1.053	5.299	0.183	30.518	1.052
0.193	27.528	1.059	5.015	0.193	27.521	1.058
0.201	25.517	1.063	4.831	0.201	25.51	1.063
0.186	30.491	1.089	5.204	0.186	30.486	1.089
0.192	27.519	1.058	5.009	0.193	27.513	1.058
0.201	25.517	1.063	4.824	0.201	25.51	1.063
0.201	25.495	1.062	4.825	0.201	25.493	1.062
0.189	28.515	1.056	5.105	0.189	28.509	1.056
0.24	17.558	1.033	4.088	0.24	17.526	1.031
0.225	19.537	1.028	4.292	0.226	19.534	1.028
0.214	22.519	1.072	4.488	0.214	22.521	1.072
0.238	16.569	0.975	4.008	0.236	16.565	0.974
0.214	22.519	1.072	4.489	0.214	22.521	1.072
0.21	23.527	1.069	4.649	0.211	23.506	1.068
0.232	19.513	1.084	4.189	0.233	19.51	1.084
0.239	16.553	0.974	4.069	0.239	16.553	0.974
0.197	26.509	1.06	4.914	0.197	26.507	1.06
0.194	27.502	1.058	5.052	0.194	27.499	1.058
0.226	20.522	1.08	4.288	0.226	20.521	1.08
0.201	26.499	1.104	4.81	0.2	26.497	1.104
0.189	27.546	1.02	5.11	0.189	27.539	1.02
0.263	13.58	0.97	3.693	0.264	13.58	0.97
0.196	26.528	1.061	4.91	0.196	26.524	1.061
0.264	14.529	1.038	3.695	0.264	14.52	1.037
0.205	25.491	1.108	4.712	0.205	25.49	1.108
0.262	13.582	0.97	3.64	0.26	13.581	0.97
0.239	17.537	1.032	4.02	0.236	17.539	1.032
0.22	21.513	1.076	4.388	0.219	21.514	1.076
0.186	30.523	1.09	5.219	0.186	30.515	1.09
0.22	21.514	1.076	4.418	0.221	21.506	1.075
0.226	19.54	1.028	4.299	0.226	19.533	1.028
0.18	32.511	1.084	5.408	0.18	32.504	1.083
0.239	18.512	1.089	4.055	0.239	18.512	1.089
0.232	18.546	1.03	4.183	0.232	18.544	1.03
0.189	30.486	1.129	5.118	0.19	30.481	1.129
0.134	60.465	1.12	7.238	0.134	60.464	1.12
0.182	30.53	1.053	5.263	0.181	30.531	1.053
0.226	19.532	1.028	4.287	0.226	19.532	1.028
0.197	25.544	1.022	4.93	0.197	25.545	1.022
0.129	62.503	1.078	7.5	0.129	62.498	1.078

0.183	29.524	1.018	5.336	0.184	29.512	1.018
0.18	33.468	1.116	5.425	0.181	33.461	1.115
0.174	34.526	1.079	5.591	0.175	34.517	1.079
0.21	21.573	0.981	4.617	0.21	21.57	0.98
0.263	12.605	0.9	3.683	0.263	12.606	0.9
0.215	22.533	1.073	4.497	0.214	22.538	1.073
0.152	47.48	1.13	6.383	0.152	47.478	1.13
0.22	19.557	0.978	4.414	0.221	19.553	0.978
0.226	19.529	1.028	4.282	0.225	19.525	1.028
0.232	18.531	1.03	4.181	0.232	18.532	1.03
0.197	26.501	1.06	4.93	0.197	26.499	1.06
0.209	23.515	1.069	4.605	0.209	23.512	1.069
0.189	29.512	1.093	5.119	0.19	29.507	1.093
0.186	28.534	1.019	5.186	0.185	28.53	1.019
0.21	23.509	1.069	4.633	0.211	23.503	1.068
0.232	19.511	1.084	4.193	0.233	19.499	1.083
0.209	23.53	1.07	4.603	0.209	23.529	1.069
0.329	8.597	0.955	2.979	0.331	8.581	0.953
0.179	32.502	1.083	5.374	0.179	32.501	1.083
0.232	18.537	1.03	4.173	0.232	18.537	1.03
0.372	5.709	0.816	2.587	0.37	5.702	0.815
0.154	43.5	1.061	6.33	0.154	43.496	1.061
0.215	21.539	1.026	4.517	0.215	21.523	1.025
0.161	39.527	1.068	5.972	0.161	39.522	1.068
0.284	11.588	0.966	3.418	0.285	11.58	0.965
0.215	23.506	1.119	4.51	0.215	23.504	1.119
0.232	18.546	1.03	4.168	0.232	18.547	1.03
0.233	17.562	0.976	4.193	0.233	17.563	0.976
0.177	32.524	1.049	5.488	0.177	32.524	1.049
0.169	35.506	1.044	5.717	0.168	35.505	1.044
0.172	34.503	1.046	5.646	0.171	34.497	1.045
0.206	25.484	1.108	4.738	0.206	25.48	1.108
0.186	28.538	1.019	5.194	0.185	28.534	1.019
0.24	17.558	1.033	4.082	0.24	17.527	1.031
0.215	24.482	1.166	4.533	0.216	24.475	1.165
0.254	14.579	0.972	3.795	0.253	14.582	0.972
0.162	40.475	1.094	5.978	0.162	40.475	1.094
0.196	26.528	1.061	4.905	0.196	26.526	1.061
0.215	22.513	1.072	4.511	0.215	22.509	1.072
0.174	35.479	1.109	5.566	0.174	35.479	1.109
0.245	16.555	1.035	3.956	0.247	16.534	1.033
0.273	13.55	1.042	3.567	0.274	13.534	1.041
0.189	28.534	1.057	5.112	0.189	28.533	1.057
0.215	21.534	1.025	4.512	0.215	21.527	1.025
0.226	19.536	1.028	4.297	0.226	19.532	1.028
0.254	15.543	1.036	3.81	0.254	15.541	1.036
0.197	26.509	1.06	4.912	0.196	26.513	1.061
0.209	23.516	1.069	4.613	0.21	23.51	1.069
0.284	11.597	0.966	3.413	0.284	11.588	0.966
0.193	28.511	1.097	5	0.192	28.51	1.097

0.239	16.57	0.975	4.056	0.239	16.573	0.975
0.2	25.513	1.063	4.801	0.2	25.511	1.063
0.197	26.53	1.061	4.906	0.196	26.529	1.061
0.215	22.519	1.072	4.533	0.216	22.508	1.072
0.18	32.497	1.083	5.385	0.179	32.499	1.083
0.205	24.516	1.066	4.722	0.205	24.512	1.066
0.225	18.571	0.977	4.278	0.225	18.567	0.977
0.231	18.546	1.03	4.169	0.232	18.542	1.03
0.221	22.484	1.124	4.42	0.221	22.482	1.124
0.233	18.533	1.03	4.187	0.233	18.532	1.03
0.314	10.527	1.053	3.151	0.315	10.509	1.051
0.328	7.66	0.851	2.948	0.328	7.663	0.851
0.205	23.548	1.024	4.721	0.205	23.548	1.024
0.193	27.532	1.059	5.03	0.193	27.526	1.059
0.171	34.513	1.046	5.654	0.171	34.513	1.046
0.189	29.52	1.093	5.127	0.19	29.513	1.093
0.22	19.567	0.978	4.396	0.22	19.565	0.978
0.225	18.558	0.977	4.269	0.225	18.561	0.977
0.284	11.601	0.967	3.392	0.283	11.6	0.967
0.206	25.497	1.109	4.734	0.206	25.493	1.108
0.154	44.488	1.085	6.308	0.154	44.488	1.085
0.215	24.482	1.166	4.532	0.216	24.478	1.166
0.183	32.488	1.12	5.288	0.182	32.485	1.12
0.219	19.575	0.979	4.375	0.219	19.581	0.979
0.21	22.538	1.024	4.613	0.21	22.54	1.025
0.183	31.51	1.087	5.308	0.183	31.507	1.086
0.239	17.558	1.033	4.053	0.238	17.558	1.033
0.197	29.463	1.179	4.917	0.197	29.458	1.178
0.205	26.492	1.152	4.729	0.206	26.484	1.151
0.238	17.546	1.032	4.045	0.238	17.549	1.032
0.247	15.581	0.974	3.934	0.246	15.584	0.974
0.177	34.483	1.112	5.507	0.178	34.476	1.112
0.197	25.561	1.022	4.93	0.197	25.553	1.022
0.215	21.541	1.026	4.52	0.215	21.526	1.025
0.193	26.558	1.021	5.017	0.193	26.552	1.021
0.239	17.536	1.032	4.076	0.24	17.525	1.031
0.284	11.591	0.966	3.4	0.283	11.596	0.966
0.205	24.523	1.066	4.707	0.205	24.521	1.066
0.22	21.513	1.076	4.392	0.22	21.513	1.076
0.148	46.513	1.057	6.515	0.148	46.514	1.057
0.193	28.511	1.097	5	0.192	28.51	1.097
0.201	25.516	1.063	4.804	0.2	25.523	1.063
0.215	23.482	1.118	4.525	0.215	23.477	1.118
0.186	30.505	1.089	5.219	0.186	30.501	1.089
0.22	21.514	1.076	4.42	0.221	21.505	1.075
0.205	26.496	1.152	4.736	0.206	26.488	1.152
0.2	25.524	1.064	4.789	0.2	25.523	1.063
0.154	45.493	1.11	6.31	0.154	45.488	1.109
0.245	15.596	0.975	3.927	0.245	15.587	0.974
0.272	12.578	0.968	3.527	0.271	12.581	0.968

0.232	19.502	1.083	4.179	0.232	19.502	1.083
0.239	17.55	1.032	4.071	0.239	17.539	1.032
0.205	24.521	1.066	4.691	0.204	24.52	1.066
0.226	20.515	1.08	4.308	0.227	20.505	1.079
0.232	18.529	1.029	4.175	0.232	18.533	1.03
0.174	34.505	1.078	5.541	0.173	34.501	1.078
0.193	28.494	1.096	5.017	0.193	28.489	1.096
0.197	26.522	1.061	4.902	0.196	26.512	1.06
0.239	17.539	1.032	4.048	0.238	17.536	1.032
0.225	18.56	0.977	4.276	0.225	18.562	0.977
0.179	30.545	1.018	5.383	0.179	30.54	1.018
0.186	30.494	1.089	5.208	0.186	30.491	1.089
0.193	28.516	1.097	5.029	0.193	28.507	1.096
0.205	25.507	1.109	4.726	0.205	25.503	1.109
0.239	16.561	0.974	4.058	0.239	16.56	0.974
0.239	16.561	0.974	4.055	0.239	16.561	0.974
0.196	25.525	1.021	4.902	0.196	25.523	1.021
0.226	19.53	1.028	4.286	0.226	19.534	1.028
0.273	12.597	0.969	3.534	0.272	12.608	0.97
0.226	19.53	1.028	4.286	0.226	19.534	1.028
0.239	17.546	1.032	4.055	0.239	17.55	1.032
0.189	29.496	1.092	5.087	0.188	29.496	1.092
0.201	26.499	1.104	4.828	0.201	26.494	1.104
0.201	26.499	1.104	4.828	0.201	26.494	1.104
0.215	20.554	0.979	4.51	0.215	20.554	0.979
0.172	34.517	1.046	5.659	0.171	34.516	1.046
0.255	15.544	1.036	3.817	0.254	15.549	1.037
0.239	16.58	0.975	4.073	0.24	16.552	0.974
0.18	33.468	1.116	5.415	0.181	33.465	1.115
0.22	21.507	1.075	4.389	0.219	21.51	1.075
0.16	42.488	1.118	6.074	0.16	42.483	1.118
0.22	20.537	1.027	4.403	0.22	20.538	1.027
0.312	9.593	0.959	3.122	0.312	9.592	0.959
0.21	24.48	1.113	4.633	0.211	24.475	1.112
0.171	35.511	1.076	5.62	0.17	35.514	1.076
0.232	17.572	0.976	4.171	0.232	17.565	0.976
0.189	28.509	1.056	5.114	0.189	28.506	1.056
0.239	17.534	1.031	4.026	0.237	17.536	1.032
0.21	23.506	1.068	4.601	0.209	23.503	1.068
0.183	31.491	1.086	5.31	0.183	31.484	1.086
0.232	18.539	1.03	4.143	0.23	18.545	1.03
0.196	26.526	1.061	4.927	0.197	26.519	1.061
0.206	23.54	1.023	4.726	0.205	23.528	1.023
0.22	20.548	1.027	4.387	0.219	20.544	1.027
0.232	18.534	1.03	4.177	0.232	18.531	1.03
0.187	28.527	1.019	5.234	0.187	28.521	1.019
0.21	22.531	1.024	4.594	0.209	22.535	1.024
0.215	22.519	1.072	4.527	0.216	22.512	1.072
0.226	19.539	1.028	4.314	0.227	19.531	1.028
0.21	23.511	1.069	4.626	0.21	23.502	1.068

0.186	30.494	1.089	5.198	0.186	30.492	1.089
0.247	15.575	0.973	3.944	0.246	15.577	0.974
0.215	23.499	1.119	4.53	0.216	23.493	1.119
0.174	34.526	1.079	5.592	0.175	34.517	1.079
0.274	13.557	1.043	3.572	0.275	13.547	1.042
0.245	15.596	0.975	3.928	0.245	15.586	0.974
0.24	18.49	1.088	4.091	0.241	18.482	1.087
0.215	21.53	1.025	4.504	0.214	21.533	1.025
0.197	25.515	1.021	4.943	0.198	25.514	1.021
0.215	21.522	1.025	4.489	0.214	21.518	1.025
0.231	17.564	0.976	4.118	0.229	17.565	0.976
0.226	19.548	1.029	4.292	0.226	19.546	1.029
0.194	28.476	1.095	5.048	0.194	28.471	1.095
0.206	25.497	1.109	4.736	0.206	25.49	1.108
0.215	21.544	1.026	4.517	0.215	21.545	1.026
0.187	29.505	1.054	5.237	0.187	29.5	1.054
0.31	8.647	0.865	3.102	0.31	8.641	0.864
0.174	33.516	1.047	5.564	0.174	33.518	1.047
0.155	44.491	1.112	6.229	0.156	44.486	1.112
0.172	33.53	1.016	5.679	0.172	33.527	1.016
0.131	64.484	1.131	7.444	0.131	64.482	1.131
0.193	29.495	1.134	5.006	0.193	29.498	1.135
0.205	24.516	1.066	4.729	0.206	24.511	1.066
0.205	24.524	1.066	4.718	0.205	24.526	1.066
0.225	19.552	1.029	4.299	0.226	19.543	1.029
0.187	31.462	1.124	5.234	0.187	31.456	1.123
0.205	24.525	1.066	4.7	0.204	24.53	1.067
0.22	20.546	1.027	4.4	0.22	20.541	1.027

VEm1	VEm2	VRm1	VRm2	VEv1	VEv2	VRv1
4.031	0.237	16.587	0.976	4.02	0.236	16.596
4.786	0.199	23.564	0.982	4.799	0.2	23.561
5.026	0.193	27.515	1.058	5	0.192	27.533
3.262	0.297	10.583	0.962	3.248	0.295	10.592
3.917	0.245	16.546	1.034	3.904	0.244	16.551
3.631	0.259	12.622	0.902	3.663	0.262	12.658
5.393	0.18	31.514	1.05	5.382	0.179	31.517
3.145	0.314	10.515	1.051	3.122	0.312	10.528
4.627	0.21	24.487	1.113	4.625	0.21	24.49
4.167	0.232	17.564	0.976	4.184	0.232	17.559
6.216	0.155	41.529	1.038	6.219	0.155	41.532
5.437	0.175	32.523	1.049	5.426	0.175	32.526
4.505	0.215	22.514	1.072	4.477	0.213	22.524
5.186	0.185	28.551	1.02	5.129	0.183	28.565
4.93	0.197	26.517	1.061	4.927	0.197	26.526
4.831	0.201	25.515	1.063	4.837	0.202	25.52
3.693	0.264	14.542	1.039	3.7	0.264	14.538
6.002	0.162	39.505	1.068	5.983	0.162	39.523
4.275	0.225	18.561	0.977	4.287	0.226	18.561
4.525	0.215	22.499	1.071	4.532	0.216	22.503
4.586	0.208	23.54	1.07	4.618	0.21	23.536
4.298	0.226	20.514	1.08	4.284	0.225	20.528
4.501	0.214	22.516	1.072	4.498	0.214	22.524
4.727	0.206	24.511	1.066	4.734	0.206	24.516
5.112	0.189	28.511	1.056	5.112	0.189	28.509
5.219	0.186	30.515	1.09	5.194	0.185	30.539
4.592	0.209	22.518	1.024	4.595	0.209	22.517
6.447	0.15	45.518	1.059	6.435	0.15	45.521
4.594	0.209	23.521	1.069	4.586	0.208	23.526
4.696	0.204	22.554	0.981	4.694	0.204	22.551
6.104	0.157	41.515	1.064	6.123	0.157	41.515
6.475	0.151	48.472	1.127	6.492	0.151	48.472
4.278	0.225	19.553	1.029	4.277	0.225	19.551
4.504	0.214	21.538	1.026	4.521	0.215	21.533
5.732	0.169	39.48	1.161	5.698	0.168	39.494
5.228	0.187	30.47	1.088	5.232	0.187	30.472
4.812	0.201	27.474	1.145	4.8	0.2	27.484
3.698	0.264	14.558	1.04	3.682	0.263	14.571
5.383	0.179	31.522	1.051	5.363	0.179	31.532
5.03	0.193	29.481	1.134	5.025	0.193	29.49
5.667	0.172	36.482	1.106	5.631	0.171	36.494
4.196	0.233	19.507	1.084	4.187	0.233	19.513
6.282	0.153	44.507	1.086	6.243	0.152	44.515
4.284	0.225	20.52	1.08	4.258	0.224	20.535
5.421	0.181	32.479	1.083	5.417	0.181	32.483
4.833	0.201	25.507	1.063	4.838	0.202	25.517
4.93	0.197	26.513	1.061	4.929	0.197	26.523
5.483	0.177	33.493	1.08	5.483	0.177	33.5
5.3	0.183	31.49	1.086	5.287	0.182	31.494

5.569	0.174	34.499	1.078	5.552	0.174	34.512
5.394	0.18	31.52	1.051	5.367	0.179	31.528
6.536	0.149	47.491	1.079	6.522	0.148	47.497
5.39	0.18	31.521	1.051	5.358	0.179	31.534
5.195	0.186	29.517	1.054	5.181	0.185	29.531
5.116	0.189	28.517	1.056	5.122	0.19	28.524
5.903	0.164	38.498	1.069	5.916	0.164	38.508
5.993	0.162	39.504	1.068	5.978	0.162	39.509
4.932	0.197	26.513	1.061	4.903	0.196	26.53
5.912	0.164	38.502	1.069	5.889	0.164	38.513
5.299	0.183	30.518	1.052	5.264	0.182	30.533
5.016	0.193	27.52	1.058	4.989	0.192	27.546
4.831	0.201	25.51	1.063	4.838	0.202	25.517
5.204	0.186	30.486	1.089	5.214	0.186	30.491
5.009	0.193	27.513	1.058	5.001	0.192	27.52
4.824	0.201	25.51	1.063	4.834	0.201	25.517
4.825	0.201	25.493	1.062	4.841	0.202	25.491
5.105	0.189	28.509	1.056	5.08	0.188	28.516
4.089	0.241	17.526	1.031	4.054	0.238	17.564
4.292	0.226	19.534	1.028	4.266	0.225	19.547
4.488	0.214	22.521	1.072	4.486	0.214	22.525
4.008	0.236	16.565	0.974	4.046	0.238	16.566
4.489	0.214	22.521	1.072	4.471	0.213	22.531
4.649	0.211	23.506	1.068	4.619	0.21	23.538
4.19	0.233	19.509	1.084	4.171	0.232	19.527
4.068	0.239	16.553	0.974	4.068	0.239	16.552
4.914	0.197	26.507	1.06	4.909	0.196	26.511
5.053	0.194	27.499	1.058	5.028	0.193	27.516
4.288	0.226	20.521	1.08	4.249	0.224	20.536
4.81	0.2	26.497	1.104	4.791	0.2	26.509
5.11	0.189	27.539	1.02	5.12	0.19	27.547
3.693	0.264	13.58	0.97	3.698	0.264	13.576
4.91	0.196	26.524	1.061	4.861	0.194	26.547
3.695	0.264	14.52	1.037	3.69	0.264	14.526
4.712	0.205	25.49	1.108	4.702	0.204	25.493
3.64	0.26	13.581	0.97	3.683	0.263	13.573
4.02	0.236	17.539	1.032	4.04	0.238	17.55
4.388	0.219	21.514	1.076	4.382	0.219	21.516
5.219	0.186	30.515	1.09	5.194	0.185	30.539
4.419	0.221	21.506	1.075	4.404	0.22	21.519
4.299	0.226	19.533	1.028	4.292	0.226	19.545
5.408	0.18	32.504	1.083	5.406	0.18	32.51
4.055	0.239	18.512	1.089	4.039	0.238	18.515
4.183	0.232	18.544	1.03	4.12	0.229	18.585
5.118	0.19	30.481	1.129	5.091	0.189	30.496
7.238	0.134	60.464	1.12	7.241	0.134	60.467
5.263	0.181	30.531	1.053	5.238	0.181	30.542
4.287	0.226	19.532	1.028	4.273	0.225	19.537
4.93	0.197	25.545	1.022	4.918	0.197	25.548
7.5	0.129	62.498	1.078	7.466	0.129	62.505

5.336	0.184	29.512	1.018	5.315	0.183	29.522
5.425	0.181	33.461	1.115	5.424	0.181	33.465
5.591	0.175	34.517	1.079	5.558	0.174	34.548
4.617	0.21	21.57	0.98	4.584	0.208	21.589
3.683	0.263	12.606	0.9	3.685	0.263	12.605
4.497	0.214	22.538	1.073	4.482	0.213	22.545
6.383	0.152	47.478	1.13	6.34	0.151	47.49
4.414	0.221	19.553	0.978	4.394	0.22	19.563
4.282	0.225	19.525	1.028	4.265	0.224	19.538
4.181	0.232	18.532	1.03	4.183	0.232	18.531
4.93	0.197	26.499	1.06	4.941	0.198	26.498
4.606	0.209	23.512	1.069	4.598	0.209	23.522
5.119	0.19	29.507	1.093	5.099	0.189	29.512
5.186	0.185	28.529	1.019	5.155	0.184	28.551
4.633	0.211	23.503	1.068	4.599	0.209	23.525
4.194	0.233	19.498	1.083	4.159	0.231	19.524
4.603	0.209	23.529	1.069	4.568	0.208	23.544
2.98	0.331	8.58	0.953	2.952	0.328	8.6
5.374	0.179	32.501	1.083	5.354	0.178	32.513
4.173	0.232	18.537	1.03	4.147	0.23	18.56
2.586	0.369	5.702	0.815	2.598	0.371	5.707
6.331	0.154	43.495	1.061	6.295	0.154	43.513
4.517	0.215	21.523	1.025	4.496	0.214	21.543
5.972	0.161	39.522	1.068	5.946	0.161	39.535
3.418	0.285	11.58	0.965	3.397	0.283	11.597
4.51	0.215	23.504	1.119	4.444	0.212	23.529
4.168	0.232	18.547	1.03	4.164	0.231	18.549
4.193	0.233	17.563	0.976	4.201	0.233	17.559
5.488	0.177	32.524	1.049	5.489	0.177	32.523
5.717	0.168	35.505	1.044	5.735	0.169	35.504
5.646	0.171	34.497	1.045	5.644	0.171	34.505
4.738	0.206	25.48	1.108	4.735	0.206	25.488
5.194	0.186	28.534	1.019	5.184	0.185	28.543
4.083	0.24	17.527	1.031	4.047	0.238	17.564
4.533	0.216	24.475	1.165	4.488	0.214	24.497
3.795	0.253	14.582	0.972	3.781	0.252	14.592
5.978	0.162	40.475	1.094	5.977	0.162	40.475
4.905	0.196	26.526	1.061	4.876	0.195	26.542
4.511	0.215	22.509	1.072	4.511	0.215	22.516
5.566	0.174	35.479	1.109	5.566	0.174	35.48
3.956	0.247	16.534	1.033	3.913	0.245	16.557
3.567	0.274	13.534	1.041	3.54	0.272	13.555
5.112	0.189	28.533	1.057	5.049	0.187	28.546
4.512	0.215	21.527	1.025	4.494	0.214	21.542
4.297	0.226	19.532	1.028	4.28	0.225	19.546
3.81	0.254	15.541	1.036	3.8	0.253	15.542
4.913	0.197	26.512	1.06	4.883	0.195	26.527
4.613	0.21	23.51	1.069	4.609	0.209	23.511
3.413	0.284	11.588	0.966	3.423	0.285	11.588
5	0.192	28.51	1.097	4.985	0.192	28.523

4.056	0.239	16.573	0.975	4.064	0.239	16.569
4.801	0.2	25.51	1.063	4.796	0.2	25.519
4.906	0.196	26.528	1.061	4.893	0.196	26.546
4.533	0.216	22.508	1.072	4.507	0.215	22.528
5.385	0.179	32.499	1.083	5.374	0.179	32.503
4.722	0.205	24.512	1.066	4.732	0.206	24.521
4.278	0.225	18.567	0.977	4.259	0.224	18.579
4.169	0.232	18.542	1.03	4.131	0.229	18.554
4.42	0.221	22.482	1.124	4.407	0.22	22.486
4.187	0.233	18.532	1.03	4.196	0.233	18.532
3.151	0.315	10.509	1.051	3.138	0.314	10.517
2.948	0.328	7.663	0.851	2.947	0.327	7.662
4.721	0.205	23.548	1.024	4.702	0.204	23.557
5.03	0.193	27.526	1.059	5.004	0.192	27.54
5.654	0.171	34.513	1.046	5.647	0.171	34.52
5.127	0.19	29.513	1.093	5.101	0.189	29.532
4.396	0.22	19.565	0.978	4.381	0.219	19.575
4.269	0.225	18.561	0.977	4.267	0.225	18.564
3.392	0.283	11.6	0.967	3.384	0.282	11.609
4.735	0.206	25.492	1.108	4.715	0.205	25.506
6.308	0.154	44.488	1.085	6.294	0.154	44.495
4.532	0.216	24.478	1.166	4.477	0.213	24.505
5.287	0.182	32.485	1.12	5.276	0.182	32.501
4.375	0.219	19.581	0.979	4.358	0.218	19.576
4.613	0.21	22.54	1.025	4.617	0.21	22.543
5.308	0.183	31.507	1.086	5.288	0.182	31.515
4.053	0.238	17.558	1.033	4.038	0.238	17.568
4.917	0.197	29.458	1.178	4.893	0.196	29.472
4.729	0.206	26.484	1.151	4.703	0.204	26.497
4.045	0.238	17.549	1.032	3.985	0.234	17.573
3.934	0.246	15.584	0.974	3.925	0.245	15.586
5.507	0.178	34.476	1.112	5.491	0.177	34.489
4.93	0.197	25.553	1.022	4.863	0.195	25.579
4.52	0.215	21.526	1.025	4.509	0.215	21.539
5.017	0.193	26.552	1.021	4.952	0.19	26.571
4.076	0.24	17.525	1.031	4.057	0.239	17.534
3.4	0.283	11.596	0.966	3.366	0.28	11.625
4.707	0.205	24.521	1.066	4.687	0.204	24.536
4.392	0.22	21.513	1.076	4.366	0.218	21.521
6.515	0.148	46.514	1.057	6.52	0.148	46.519
5	0.192	28.51	1.097	4.985	0.192	28.523
4.804	0.2	25.523	1.063	4.78	0.199	25.535
4.525	0.215	23.477	1.118	4.509	0.215	23.486
5.219	0.186	30.501	1.089	5.223	0.187	30.505
4.42	0.221	21.505	1.075	4.398	0.22	21.522
4.736	0.206	26.488	1.152	4.669	0.203	26.518
4.789	0.2	25.523	1.063	4.826	0.201	25.52
6.31	0.154	45.488	1.109	6.262	0.153	45.499
3.927	0.245	15.587	0.974	3.928	0.245	15.589
3.527	0.271	12.581	0.968	3.51	0.27	12.583

4.179	0.232	19.502	1.083	4.185	0.233	19.499
4.071	0.239	17.539	1.032	4.058	0.239	17.547
4.691	0.204	24.52	1.066	4.736	0.206	24.512
4.308	0.227	20.505	1.079	4.295	0.226	20.514
4.175	0.232	18.533	1.03	4.187	0.233	18.53
5.541	0.173	34.501	1.078	5.544	0.173	34.508
5.017	0.193	28.489	1.096	5.018	0.193	28.492
4.902	0.196	26.512	1.06	4.912	0.196	26.52
4.048	0.238	17.536	1.032	4.035	0.237	17.546
4.276	0.225	18.562	0.977	4.285	0.226	18.56
5.383	0.179	30.54	1.018	5.365	0.179	30.551
5.208	0.186	30.491	1.089	5.188	0.185	30.504
5.029	0.193	28.507	1.096	5.005	0.192	28.524
4.726	0.205	25.503	1.109	4.706	0.205	25.513
4.058	0.239	16.56	0.974	4.059	0.239	16.563
4.055	0.239	16.561	0.974	4.068	0.239	16.559
4.902	0.196	25.523	1.021	4.906	0.196	25.527
4.286	0.226	19.534	1.028	4.297	0.226	19.531
3.534	0.272	12.608	0.97	3.529	0.271	12.617
4.286	0.226	19.534	1.028	4.297	0.226	19.531
4.056	0.239	17.55	1.032	4.038	0.238	17.561
5.087	0.188	29.496	1.092	5.062	0.187	29.5
4.828	0.201	26.494	1.104	4.8	0.2	26.512
4.828	0.201	26.494	1.104	4.8	0.2	26.512
4.51	0.215	20.554	0.979	4.497	0.214	20.56
5.659	0.171	34.516	1.046	5.641	0.171	34.521
3.818	0.255	15.548	1.037	3.773	0.252	15.572
4.073	0.24	16.551	0.974	4.06	0.239	16.601
5.416	0.181	33.465	1.115	5.406	0.18	33.472
4.389	0.219	21.51	1.075	4.363	0.218	21.518
6.074	0.16	42.483	1.118	6.032	0.159	42.493
4.403	0.22	20.538	1.027	4.415	0.221	20.534
3.122	0.312	9.592	0.959	3.087	0.309	9.621
4.633	0.211	24.475	1.112	4.636	0.211	24.482
5.62	0.17	35.514	1.076	5.612	0.17	35.518
4.171	0.232	17.565	0.976	4.157	0.231	17.576
5.114	0.189	28.506	1.056	5.111	0.189	28.512
4.026	0.237	17.536	1.032	4.058	0.239	17.535
4.601	0.209	23.503	1.068	4.637	0.211	23.503
5.31	0.183	31.484	1.086	5.325	0.184	31.485
4.143	0.23	18.545	1.03	4.182	0.232	18.539
4.927	0.197	26.519	1.061	4.878	0.195	26.543
4.726	0.205	23.528	1.023	4.705	0.205	23.545
4.387	0.219	20.544	1.027	4.353	0.218	20.563
4.177	0.232	18.531	1.03	4.159	0.231	18.545
5.234	0.187	28.521	1.019	5.207	0.186	28.53
4.594	0.209	22.535	1.024	4.612	0.21	22.534
4.527	0.216	22.512	1.072	4.506	0.215	22.53
4.315	0.227	19.531	1.028	4.294	0.226	19.541
4.626	0.21	23.502	1.068	4.633	0.211	23.516

5.198	0.186	30.492	1.089	5.179	0.185	30.504
3.944	0.246	15.577	0.974	3.926	0.245	15.592
4.53	0.216	23.493	1.119	4.508	0.215	23.505
5.592	0.175	34.517	1.079	5.557	0.174	34.548
3.572	0.275	13.547	1.042	3.556	0.274	13.561
3.928	0.245	15.586	0.974	3.932	0.246	15.586
4.091	0.241	18.482	1.087	4.094	0.241	18.485
4.504	0.214	21.533	1.025	4.519	0.215	21.529
4.943	0.198	25.514	1.021	4.954	0.198	25.512
4.489	0.214	21.518	1.025	4.532	0.216	21.515
4.118	0.229	17.565	0.976	4.168	0.232	17.56
4.292	0.226	19.546	1.029	4.245	0.223	19.57
5.048	0.194	28.471	1.095	5.051	0.194	28.472
4.736	0.206	25.49	1.108	4.729	0.206	25.496
4.517	0.215	21.545	1.026	4.452	0.212	21.574
5.237	0.187	29.5	1.054	5.224	0.187	29.508
3.102	0.31	8.641	0.864	3.098	0.31	8.651
5.564	0.174	33.518	1.047	5.559	0.174	33.522
6.229	0.156	44.486	1.112	6.182	0.155	44.497
5.679	0.172	33.527	1.016	5.63	0.171	33.544
7.444	0.131	64.482	1.131	7.397	0.13	64.499
5.006	0.193	29.498	1.135	4.978	0.191	29.502
4.729	0.206	24.511	1.066	4.728	0.206	24.522
4.718	0.205	24.526	1.066	4.709	0.205	24.527
4.299	0.226	19.543	1.029	4.29	0.226	19.548
5.234	0.187	31.456	1.123	5.24	0.187	31.459
4.7	0.204	24.53	1.067	4.695	0.204	24.535
4.4	0.22	20.541	1.027	4.379	0.219	20.557

VRv2	VEe1	VEe2	VRe1	VRe2	VEp1	VEp2
0.976	4.031	0.237	16.587	0.976	4.017	0.236
0.982	4.786	0.199	23.564	0.982	4.799	0.2
1.059	5.026	0.193	27.515	1.058	4.989	0.192
0.963	3.262	0.297	10.583	0.962	3.246	0.295
1.034	3.918	0.245	16.546	1.034	3.902	0.244
0.904	3.677	0.263	12.631	0.902	3.641	0.26
1.051	5.393	0.18	31.514	1.05	5.379	0.179
1.053	3.144	0.314	10.515	1.051	3.114	0.311
1.113	4.624	0.21	24.491	1.113	4.626	0.21
0.975	4.167	0.232	17.564	0.976	4.185	0.233
1.038	6.216	0.155	41.529	1.038	6.218	0.155
1.049	5.446	0.176	32.528	1.049	5.419	0.175
1.073	4.505	0.215	22.514	1.072	4.469	0.213
1.02	5.186	0.185	28.553	1.02	5.119	0.183
1.061	4.934	0.197	26.517	1.061	4.92	0.197
1.063	4.836	0.201	25.514	1.063	4.831	0.201
1.038	3.693	0.264	14.542	1.039	3.7	0.264
1.068	6	0.162	39.506	1.068	5.971	0.161
0.977	4.275	0.225	18.561	0.977	4.288	0.226
1.072	4.525	0.215	22.499	1.071	4.531	0.216
1.07	4.587	0.208	23.54	1.07	4.621	0.21
1.08	4.318	0.227	20.504	1.079	4.262	0.224
1.073	4.496	0.214	22.52	1.072	4.501	0.214
1.066	4.732	0.206	24.51	1.066	4.728	0.206
1.056	5.112	0.189	28.511	1.056	5.113	0.189
1.091	5.219	0.186	30.515	1.09	5.184	0.185
1.024	4.592	0.209	22.518	1.024	4.595	0.209
1.059	6.447	0.15	45.518	1.059	6.431	0.15
1.069	4.594	0.209	23.521	1.069	4.584	0.208
0.98	4.696	0.204	22.554	0.981	4.693	0.204
1.064	6.12	0.157	41.512	1.064	6.112	0.157
1.127	6.472	0.151	48.474	1.127	6.494	0.151
1.029	4.278	0.225	19.553	1.029	4.274	0.225
1.025	4.504	0.214	21.538	1.026	4.523	0.215
1.162	5.732	0.169	39.48	1.161	5.688	0.167
1.088	5.228	0.187	30.47	1.088	5.232	0.187
1.145	4.812	0.2	27.474	1.145	4.796	0.2
1.041	3.698	0.264	14.558	1.04	3.679	0.263
1.051	5.383	0.179	31.522	1.051	5.358	0.179
1.134	5.03	0.193	29.481	1.134	5.022	0.193
1.106	5.667	0.172	36.482	1.106	5.622	0.17
1.084	4.196	0.233	19.507	1.084	4.184	0.232
1.086	6.282	0.153	44.507	1.086	6.235	0.152
1.081	4.279	0.225	20.523	1.08	4.255	0.224
1.083	5.421	0.181	32.479	1.083	5.416	0.181
1.063	4.838	0.202	25.509	1.063	4.832	0.201
1.061	4.935	0.197	26.513	1.061	4.921	0.197
1.081	5.483	0.177	33.491	1.08	5.479	0.177
1.086	5.298	0.183	31.488	1.086	5.275	0.182

1.078	5.573	0.174	34.498	1.078	5.541	0.173
1.051	5.398	0.18	31.52	1.051	5.356	0.179
1.079	6.53	0.148	47.49	1.079	6.52	0.148
1.051	5.394	0.18	31.52	1.051	5.348	0.178
1.055	5.199	0.186	29.516	1.054	5.176	0.185
1.056	5.121	0.19	28.516	1.056	5.117	0.19
1.07	5.91	0.164	38.5	1.069	5.916	0.164
1.068	5.997	0.162	39.503	1.068	5.97	0.161
1.061	4.936	0.197	26.513	1.061	4.888	0.196
1.07	5.915	0.164	38.499	1.069	5.875	0.163
1.053	5.305	0.183	30.517	1.052	5.253	0.181
1.059	5.005	0.192	27.527	1.059	4.982	0.192
1.063	4.837	0.202	25.51	1.063	4.832	0.201
1.089	5.206	0.186	30.485	1.089	5.212	0.186
1.058	5.012	0.193	27.511	1.058	4.997	0.192
1.063	4.83	0.201	25.51	1.063	4.828	0.201
1.062	4.825	0.201	25.493	1.062	4.843	0.202
1.056	5.103	0.189	28.511	1.056	5.078	0.188
1.033	4.076	0.24	17.546	1.032	4.058	0.239
1.029	4.288	0.226	19.537	1.028	4.261	0.224
1.073	4.499	0.214	22.52	1.072	4.476	0.213
0.974	4.034	0.237	16.564	0.974	4.034	0.237
1.073	4.5	0.214	22.52	1.072	4.456	0.212
1.07	4.638	0.211	23.518	1.069	4.621	0.21
1.085	4.183	0.232	19.514	1.084	4.169	0.232
0.974	4.08	0.24	16.548	0.973	4.059	0.239
1.06	4.914	0.197	26.507	1.06	4.908	0.196
1.058	5.052	0.194	27.5	1.058	5.016	0.193
1.081	4.288	0.226	20.521	1.08	4.242	0.223
1.105	4.81	0.2	26.497	1.104	4.786	0.199
1.02	5.108	0.189	27.544	1.02	5.123	0.19
0.97	3.687	0.263	13.584	0.97	3.7	0.264
1.062	4.905	0.196	26.527	1.061	4.854	0.194
1.038	3.692	0.264	14.53	1.038	3.69	0.264
1.108	4.708	0.205	25.493	1.108	4.702	0.204
0.97	3.671	0.262	13.578	0.97	3.669	0.262
1.032	4.054	0.238	17.532	1.031	4.015	0.236
1.076	4.388	0.219	21.514	1.076	4.38	0.219
1.091	5.219	0.186	30.515	1.09	5.184	0.185
1.076	4.415	0.221	21.51	1.076	4.402	0.22
1.029	4.298	0.226	19.539	1.028	4.293	0.226
1.084	5.407	0.18	32.507	1.084	5.4	0.18
1.089	4.055	0.239	18.512	1.089	4.036	0.237
1.033	4.182	0.232	18.545	1.03	4.101	0.228
1.129	5.118	0.19	30.481	1.129	5.084	0.188
1.12	7.238	0.134	60.464	1.12	7.241	0.134
1.053	5.283	0.182	30.526	1.053	5.219	0.18
1.028	4.287	0.226	19.532	1.028	4.268	0.225
1.022	4.929	0.197	25.545	1.022	4.914	0.197
1.078	7.5	0.129	62.498	1.078	7.459	0.129

1.018	5.317	0.183	29.52	1.018	5.328	0.184
1.115	5.422	0.181	33.463	1.115	5.425	0.181
1.08	5.591	0.175	34.517	1.079	5.549	0.173
0.981	4.617	0.21	21.57	0.98	4.576	0.208
0.9	3.684	0.263	12.606	0.9	3.685	0.263
1.074	4.497	0.214	22.538	1.073	4.477	0.213
1.131	6.383	0.152	47.478	1.13	6.333	0.151
0.978	4.422	0.221	19.552	0.978	4.384	0.219
1.028	4.282	0.225	19.525	1.028	4.261	0.224
1.029	4.181	0.232	18.532	1.03	4.183	0.232
1.06	4.93	0.197	26.498	1.06	4.943	0.198
1.069	4.606	0.209	23.515	1.069	4.595	0.209
1.093	5.118	0.19	29.507	1.093	5.094	0.189
1.02	5.185	0.185	28.53	1.019	5.144	0.184
1.069	4.632	0.211	23.504	1.068	4.587	0.208
1.085	4.193	0.233	19.499	1.083	4.143	0.23
1.07	4.603	0.209	23.529	1.07	4.561	0.207
0.956	2.979	0.331	8.581	0.953	2.945	0.327
1.084	5.38	0.179	32.5	1.083	5.343	0.178
1.031	4.173	0.232	18.537	1.03	4.136	0.23
0.815	2.608	0.373	5.699	0.814	2.59	0.37
1.061	6.327	0.154	43.496	1.061	6.281	0.153
1.026	4.514	0.215	21.533	1.025	4.494	0.214
1.069	5.972	0.161	39.522	1.068	5.938	0.16
0.966	3.418	0.285	11.58	0.965	3.393	0.283
1.12	4.51	0.215	23.504	1.119	4.431	0.211
1.031	4.168	0.232	18.547	1.03	4.164	0.231
0.976	4.193	0.233	17.563	0.976	4.201	0.233
1.049	5.488	0.177	32.524	1.049	5.487	0.177
1.044	5.728	0.168	35.504	1.044	5.73	0.169
1.046	5.654	0.171	34.498	1.045	5.636	0.171
1.108	4.738	0.206	25.48	1.108	4.731	0.206
1.019	5.191	0.185	28.536	1.019	5.177	0.185
1.033	4.073	0.24	17.546	1.032	4.047	0.238
1.167	4.533	0.216	24.475	1.165	4.477	0.213
0.973	3.795	0.253	14.582	0.972	3.777	0.252
1.094	5.978	0.162	40.475	1.094	5.977	0.162
1.062	4.9	0.196	26.528	1.061	4.874	0.195
1.072	4.511	0.215	22.509	1.072	4.511	0.215
1.109	5.566	0.174	35.479	1.109	5.564	0.174
1.035	3.956	0.247	16.534	1.033	3.905	0.244
1.043	3.567	0.274	13.535	1.041	3.534	0.272
1.057	5.112	0.189	28.533	1.057	5.035	0.186
1.026	4.511	0.215	21.529	1.025	4.491	0.214
1.029	4.297	0.226	19.532	1.028	4.275	0.225
1.036	3.812	0.254	15.541	1.036	3.793	0.253
1.061	4.929	0.197	26.508	1.06	4.858	0.194
1.069	4.613	0.21	23.51	1.069	4.605	0.209
0.966	3.413	0.284	11.588	0.966	3.424	0.285
1.097	5	0.192	28.51	1.097	4.98	0.192

0.975	4.057	0.239	16.573	0.975	4.064	0.239
1.063	4.801	0.2	25.511	1.063	4.793	0.2
1.062	4.906	0.196	26.529	1.061	4.888	0.196
1.073	4.527	0.216	22.515	1.072	4.504	0.214
1.083	5.385	0.179	32.5	1.083	5.371	0.179
1.066	4.729	0.206	24.51	1.066	4.729	0.206
0.978	4.271	0.225	18.571	0.977	4.26	0.224
1.031	4.169	0.232	18.542	1.03	4.123	0.229
1.124	4.42	0.221	22.482	1.124	4.402	0.22
1.03	4.187	0.233	18.532	1.03	4.196	0.233
1.052	3.149	0.315	10.514	1.051	3.134	0.313
0.851	2.948	0.328	7.663	0.851	2.947	0.327
1.024	4.721	0.205	23.548	1.024	4.695	0.204
1.059	5.034	0.194	27.525	1.059	4.993	0.192
1.046	5.654	0.171	34.513	1.046	5.646	0.171
1.094	5.127	0.19	29.513	1.093	5.094	0.189
0.979	4.395	0.22	19.567	0.978	4.378	0.219
0.977	4.269	0.225	18.561	0.977	4.266	0.225
0.967	3.393	0.283	11.6	0.967	3.382	0.282
1.109	4.731	0.206	25.496	1.109	4.707	0.205
1.085	6.309	0.154	44.488	1.085	6.289	0.153
1.167	4.531	0.216	24.478	1.166	4.464	0.213
1.121	5.288	0.182	32.485	1.12	5.271	0.182
0.979	4.375	0.219	19.581	0.979	4.354	0.218
1.025	4.613	0.21	22.54	1.025	4.616	0.21
1.087	5.311	0.183	31.506	1.086	5.279	0.182
1.033	4.053	0.238	17.558	1.033	4.034	0.237
1.179	4.917	0.197	29.458	1.178	4.886	0.195
1.152	4.729	0.206	26.484	1.151	4.696	0.204
1.034	4.045	0.238	17.549	1.032	3.974	0.234
0.974	3.934	0.246	15.584	0.974	3.923	0.245
1.113	5.502	0.177	34.479	1.112	5.488	0.177
1.023	4.93	0.197	25.553	1.022	4.849	0.194
1.026	4.513	0.215	21.534	1.025	4.509	0.215
1.022	5.017	0.193	26.552	1.021	4.94	0.19
1.031	4.076	0.24	17.525	1.031	4.054	0.238
0.969	3.4	0.283	11.596	0.966	3.359	0.28
1.067	4.707	0.205	24.521	1.066	4.682	0.204
1.076	4.392	0.22	21.513	1.076	4.358	0.218
1.057	6.515	0.148	46.514	1.057	6.519	0.148
1.097	5	0.192	28.51	1.097	4.98	0.192
1.064	4.828	0.201	25.517	1.063	4.757	0.198
1.118	4.525	0.215	23.477	1.118	4.504	0.214
1.089	5.222	0.186	30.5	1.089	5.217	0.186
1.076	4.416	0.221	21.509	1.075	4.395	0.22
1.153	4.736	0.206	26.489	1.152	4.655	0.202
1.063	4.79	0.2	25.523	1.063	4.832	0.201
1.11	6.31	0.154	45.488	1.109	6.253	0.153
0.974	3.927	0.245	15.587	0.974	3.926	0.245
0.968	3.527	0.271	12.581	0.968	3.508	0.27

1.083	4.179	0.232	19.502	1.083	4.186	0.233
1.032	4.071	0.239	17.539	1.032	4.054	0.238
1.066	4.691	0.204	24.519	1.066	4.743	0.206
1.08	4.308	0.227	20.505	1.079	4.29	0.226
1.029	4.175	0.232	18.533	1.03	4.187	0.233
1.078	5.547	0.173	34.503	1.078	5.543	0.173
1.096	5.017	0.193	28.489	1.096	5.017	0.193
1.061	4.915	0.197	26.518	1.061	4.905	0.196
1.032	4.051	0.238	17.539	1.032	4.032	0.237
0.977	4.276	0.225	18.562	0.977	4.285	0.226
1.018	5.383	0.179	30.54	1.018	5.361	0.179
1.089	5.208	0.186	30.491	1.089	5.187	0.185
1.097	5.029	0.193	28.507	1.096	4.999	0.192
1.109	4.726	0.205	25.503	1.109	4.702	0.204
0.974	4.058	0.239	16.56	0.974	4.059	0.239
0.974	4.055	0.239	16.561	0.974	4.068	0.239
1.021	4.902	0.196	25.523	1.021	4.905	0.196
1.028	4.286	0.226	19.534	1.028	4.297	0.226
0.971	3.534	0.272	12.608	0.97	3.526	0.271
1.028	4.286	0.226	19.534	1.028	4.297	0.226
1.033	4.049	0.238	17.554	1.033	4.037	0.237
1.093	5.091	0.189	29.495	1.092	5.057	0.187
1.105	4.828	0.201	26.494	1.104	4.793	0.2
1.105	4.828	0.201	26.494	1.104	4.793	0.2
0.979	4.524	0.215	20.55	0.979	4.484	0.214
1.046	5.659	0.171	34.516	1.046	5.638	0.171
1.038	3.814	0.254	15.548	1.037	3.76	0.251
0.977	4.08	0.24	16.573	0.975	4.059	0.239
1.116	5.415	0.181	33.465	1.115	5.4	0.18
1.076	4.389	0.219	21.51	1.075	4.356	0.218
1.118	6.074	0.16	42.483	1.118	6.023	0.158
1.027	4.403	0.22	20.538	1.027	4.417	0.221
0.962	3.122	0.312	9.592	0.959	3.081	0.308
1.113	4.633	0.211	24.475	1.112	4.635	0.211
1.076	5.629	0.171	35.511	1.076	5.601	0.17
0.976	4.168	0.232	17.571	0.976	4.157	0.231
1.056	5.111	0.189	28.506	1.056	5.112	0.189
1.031	4.056	0.239	17.531	1.031	4.039	0.238
1.068	4.621	0.21	23.501	1.068	4.63	0.21
1.086	5.306	0.183	31.484	1.086	5.328	0.184
1.03	4.173	0.232	18.538	1.03	4.164	0.231
1.062	4.912	0.196	26.525	1.061	4.872	0.195
1.024	4.724	0.205	23.533	1.023	4.701	0.204
1.028	4.383	0.219	20.547	1.027	4.347	0.217
1.03	4.176	0.232	18.53	1.029	4.154	0.231
1.019	5.232	0.187	28.524	1.019	5.203	0.186
1.024	4.594	0.209	22.535	1.024	4.616	0.21
1.073	4.523	0.215	22.515	1.072	4.503	0.214
1.028	4.296	0.226	19.541	1.028	4.307	0.227
1.069	4.635	0.211	23.502	1.068	4.628	0.21

1.089	5.198	0.186	30.492	1.089	5.177	0.185
0.974	3.943	0.246	15.577	0.974	3.923	0.245
1.119	4.53	0.216	23.493	1.119	4.503	0.214
1.08	5.592	0.175	34.517	1.079	5.547	0.173
1.043	3.571	0.275	13.547	1.042	3.553	0.273
0.974	3.927	0.245	15.587	0.974	3.933	0.246
1.087	4.086	0.24	18.487	1.087	4.099	0.241
1.025	4.506	0.215	21.533	1.025	4.52	0.215
1.02	4.943	0.198	25.514	1.021	4.955	0.198
1.025	4.508	0.215	21.516	1.025	4.529	0.216
0.976	4.147	0.23	17.561	0.976	4.158	0.231
1.03	4.291	0.226	19.546	1.029	4.234	0.223
1.095	5.045	0.194	28.473	1.095	5.052	0.194
1.109	4.731	0.206	25.497	1.109	4.73	0.206
1.027	4.517	0.215	21.545	1.026	4.44	0.211
1.054	5.237	0.187	29.5	1.054	5.222	0.186
0.865	3.102	0.31	8.641	0.864	3.097	0.31
1.048	5.564	0.174	33.518	1.047	5.556	0.174
1.112	6.229	0.156	44.486	1.112	6.172	0.154
1.016	5.679	0.172	33.527	1.016	5.622	0.17
1.132	7.444	0.131	64.482	1.131	7.385	0.13
1.135	5.007	0.193	29.498	1.135	4.972	0.191
1.066	4.729	0.206	24.511	1.066	4.725	0.205
1.066	4.718	0.205	24.526	1.066	4.706	0.205
1.029	4.299	0.226	19.543	1.029	4.287	0.226
1.124	5.234	0.187	31.458	1.124	5.241	0.187
1.067	4.7	0.204	24.53	1.067	4.693	0.204
1.028	4.395	0.22	20.544	1.027	4.377	0.219

VRp1	VRp2	DP01	DP02	DP03	DP04	DP05
16.598	0.976	4.25	5.221	5.938	6.474	6.863
23.56	0.982	4.949	6.331	7.482	8.457	9.286
27.538	1.059	4.819	5.91	6.729	7.345	7.8
10.593	0.963	3.516	4.2	4.648	4.925	5.064
16.551	1.034	4.164	5.149	5.9	6.471	6.888
12.653	0.904	3.869	4.596	5.045	5.303	5.412
31.518	1.051	5.094	6.346	7.343	8.152	8.805
10.531	1.053	3.22	3.63	3.748	3.654	3.392
24.488	1.113	4.506	5.429	6.064	6.489	6.745
17.558	0.975	4.462	5.629	6.55	7.279	7.846
41.533	1.038	5.431	6.667	7.611	8.352	8.934
32.525	1.049	5.213	6.541	7.605	8.469	9.171
22.527	1.073	4.485	5.443	6.122	6.599	6.916
28.566	1.02	4.893	5.962	6.754	7.351	7.796
26.528	1.061	4.694	5.663	6.333	6.783	7.057
25.522	1.063	4.779	5.943	6.849	7.556	8.096
14.537	1.038	3.83	4.552	5.01	5.279	5.398
39.528	1.068	5.355	6.611	7.586	8.361	8.98
18.561	0.977	4.629	5.952	7.048	7.962	8.718
22.504	1.072	4.572	5.671	6.52	7.177	7.673
23.535	1.07	4.521	5.478	6.169	6.67	7.021
20.539	1.081	4.327	5.229	5.85	6.264	6.51
22.523	1.073	4.657	5.894	6.91	7.752	8.445
24.517	1.066	4.745	5.945	6.903	7.671	8.28
28.509	1.056	4.94	6.102	6.978	7.646	8.15
30.545	1.091	4.858	5.937	6.781	7.458	7.998
22.517	1.024	4.976	6.515	7.832	8.971	9.959
45.522	1.059	5.666	7.105	8.283	9.279	10.132
23.527	1.069	4.648	5.757	6.601	7.248	7.737
22.551	0.98	5.003	6.5	7.761	8.834	9.75
41.516	1.065	5.434	6.711	7.705	8.497	9.129
48.472	1.127	5.601	6.997	8.145	9.111	9.929
19.55	1.029	4.455	5.54	6.372	7.01	7.487
21.532	1.025	4.542	5.57	6.321	6.863	7.234
39.496	1.162	5.088	6.158	6.954	7.553	7.994
30.473	1.088	5.237	6.783	8.093	9.207	10.155
27.485	1.145	4.621	5.594	6.311	6.844	7.231
14.573	1.041	3.753	4.368	4.701	4.832	4.802
31.535	1.051	5.259	6.738	8	9.094	10.043
29.492	1.134	4.859	6.033	6.959	7.695	8.272
36.495	1.106	5.178	6.391	7.33	8.069	8.645
19.514	1.084	4.134	4.87	5.314	5.548	5.618
44.516	1.086	5.627	7.161	8.487	9.649	10.671
20.536	1.081	4.416	5.48	6.312	6.969	7.476
32.483	1.083	5.208	6.578	7.685	8.594	9.343
25.518	1.063	4.772	5.922	6.811	7.502	8.03
26.525	1.061	4.848	6.036	6.962	7.69	8.252
33.505	1.081	5.282	6.733	7.951	8.989	9.873
31.497	1.086	5.247	6.764	8.072	9.215	10.218

34.516	1.079	5.295	6.723	7.914	8.92	9.772
31.53	1.051	5.202	6.575	7.689	8.603	9.353
47.5	1.08	5.855	7.536	8.988	10.263	11.39
31.537	1.051	5.227	6.656	7.85	8.863	9.726
29.534	1.055	4.89	5.948	6.713	7.268	7.658
28.527	1.057	5.017	6.318	7.371	8.234	8.939
38.508	1.07	5.464	6.947	8.201	9.281	10.212
39.511	1.068	5.504	6.964	8.162	9.159	9.992
26.533	1.061	4.818	5.951	6.81	7.467	7.96
38.519	1.07	5.563	7.16	8.529	9.716	10.749
30.536	1.053	5.163	6.563	7.732	8.723	9.564
27.549	1.06	4.87	6.018	6.891	7.56	8.064
25.518	1.063	4.75	5.87	6.728	7.389	7.889
30.493	1.089	5.082	6.443	7.583	8.552	9.376
27.523	1.059	4.985	6.312	7.407	8.326	9.098
25.518	1.063	4.767	5.915	6.806	7.504	8.043
25.491	1.062	4.721	5.795	6.605	7.231	7.709
28.515	1.056	5.009	6.286	7.308	8.141	8.819
17.554	1.033	4.15	4.988	5.552	5.916	6.114
19.548	1.029	4.389	5.363	6.061	6.561	6.905
22.527	1.073	4.531	5.541	6.269	6.789	7.14
16.566	0.974	4.272	5.273	6.022	6.582	6.987
22.534	1.073	4.531	5.541	6.269	6.791	7.146
23.535	1.07	4.551	5.543	6.267	6.795	7.161
19.528	1.085	4.278	5.224	5.924	6.443	6.815
16.555	0.974	4.502	5.793	6.828	7.652	8.298
26.512	1.06	5.046	6.513	7.753	8.809	9.709
27.522	1.059	5.105	6.574	7.796	8.815	9.663
20.539	1.081	4.311	5.215	5.858	6.314	6.62
26.512	1.105	4.71	5.784	6.6	7.231	7.716
27.546	1.02	4.977	6.226	7.23	8.054	8.729
13.574	0.97	3.837	4.57	5.052	5.356	5.518
26.549	1.062	4.773	5.855	6.662	7.27	7.72
14.522	1.037	3.845	4.564	5.001	5.233	5.302
25.492	1.108	4.636	5.666	6.42	6.968	7.351
13.575	0.97	3.928	4.735	5.267	5.599	5.771
17.556	1.033	4.214	5.139	5.799	6.266	6.575
21.516	1.076	4.41	5.345	6.001	6.453	6.742
30.545	1.091	4.854	5.921	6.746	7.4	7.914
21.52	1.076	4.369	5.251	5.844	6.225	6.436
19.543	1.029	4.241	5.019	5.497	5.762	5.858
32.51	1.084	4.981	6.048	6.813	7.358	7.728
18.515	1.089	4.203	5.147	5.853	6.379	6.755
18.595	1.033	4.289	5.247	5.957	6.484	6.86
30.499	1.13	4.848	5.966	6.853	7.575	8.16
60.468	1.12	6.26	8.187	9.894	11.422	12.796
30.546	1.053	4.959	6.055	6.864	7.468	7.909
19.538	1.028	4.379	5.331	5.991	6.436	6.709
25.549	1.022	4.697	5.672	6.356	6.832	7.147
62.506	1.078	5.915	7.26	8.31	9.148	9.817

29.517	1.018	5.251	6.712	7.906	8.894	9.714
33.464	1.115	5.276	6.759	7.992	9.026	9.893
34.554	1.08	5.048	6.168	7.035	7.72	8.255
21.593	0.981	4.633	5.744	6.607	7.283	7.803
12.605	0.9	4.121	5.195	6.013	6.624	7.06
22.546	1.074	4.421	5.302	5.897	6.283	6.501
47.491	1.131	5.54	6.874	7.95	8.849	9.612
19.564	0.978	4.744	6.108	7.216	8.118	8.844
19.541	1.028	4.55	5.782	6.789	7.617	8.293
18.531	1.029	4.281	5.199	5.833	6.251	6.492
26.498	1.06	4.958	6.307	7.421	8.347	9.112
23.523	1.069	4.649	5.763	6.62	7.286	7.797
29.513	1.093	4.931	6.156	7.153	7.979	8.659
28.557	1.02	5.098	6.41	7.438	8.25	8.886
23.53	1.07	4.506	5.433	6.084	6.541	6.847
19.532	1.085	4.24	5.134	5.775	6.233	6.544
23.547	1.07	4.573	5.621	6.44	7.099	7.63
8.604	0.956	3.08	3.468	3.576	3.492	3.262
32.518	1.084	5.153	6.455	7.487	8.319	8.988
18.567	1.032	4.322	5.332	6.106	6.7	7.144
5.701	0.814	2.791	3.107	3.103	2.874	2.479
43.519	1.061	6.019	7.997	9.731	11.257	12.605
21.538	1.026	4.588	5.699	6.562	7.242	7.771
39.537	1.069	5.363	6.668	7.736	8.637	9.407
11.6	0.967	3.55	4.096	4.343	4.378	4.247
23.533	1.121	4.384	5.224	5.779	6.131	6.324
18.55	1.031	4.35	5.391	6.182	6.78	7.213
17.559	0.975	4.472	5.654	6.584	7.308	7.86
32.524	1.049	5.121	6.349	7.314	8.081	8.686
35.504	1.044	5.521	7.122	8.472	9.627	10.621
34.505	1.046	5.449	6.979	8.24	9.29	10.169
25.489	1.108	4.734	5.916	6.845	7.573	8.132
28.546	1.02	5.008	6.21	7.133	7.851	8.406
17.555	1.033	4.153	4.993	5.559	5.924	6.125
24.502	1.167	4.301	5.032	5.468	5.697	5.768
14.594	0.973	4.061	4.995	5.699	6.232	6.62
40.475	1.094	5.653	7.309	8.722	9.947	11.018
26.543	1.062	4.761	5.824	6.609	7.19	7.611
22.517	1.072	4.611	5.783	6.727	7.492	8.104
35.48	1.109	5.157	6.373	7.311	8.047	8.623
16.561	1.035	4.142	5.104	5.844	6.419	6.854
13.558	1.043	3.745	4.48	4.973	5.292	5.47
28.548	1.057	4.76	5.7	6.336	6.757	7.013
21.544	1.026	4.66	5.884	6.877	7.685	8.335
19.549	1.029	4.367	5.322	5.998	6.465	6.762
15.543	1.036	3.988	4.816	5.389	5.774	6.006
26.535	1.061	4.987	6.373	7.525	8.492	9.304
23.511	1.069	4.602	5.655	6.443	7.039	7.479
11.588	0.966	3.547	4.092	4.34	4.378	4.255
28.526	1.097	4.818	5.939	6.821	7.536	8.117

16.568	0.975	4.229	5.179	5.87	6.368	6.703
25.521	1.063	4.89	6.224	7.337	8.277	9.069
26.551	1.062	4.768	5.877	6.742	7.432	7.98
22.527	1.073	4.425	5.3	5.879	6.243	6.435
32.504	1.083	5.074	6.274	7.2	7.924	8.488
24.521	1.066	4.685	5.811	6.7	7.414	7.982
18.579	0.978	4.506	5.647	6.542	7.249	7.8
18.556	1.031	4.211	5.04	5.588	5.934	6.118
22.487	1.124	4.339	5.186	5.745	6.093	6.271
18.532	1.03	4.277	5.212	5.891	6.381	6.714
10.516	1.052	3.229	3.641	3.753	3.652	3.381
7.662	0.851	3.176	3.671	3.891	3.918	3.794
23.56	1.024	4.509	5.359	5.91	6.247	6.419
27.543	1.059	4.818	5.897	6.695	7.286	7.709
34.52	1.046	5.403	6.908	8.17	9.243	10.159
29.535	1.094	4.831	5.916	6.758	7.426	7.953
19.575	0.979	4.603	5.799	6.745	7.494	8.079
18.565	0.977	4.672	6.043	7.194	8.168	8.992
11.611	0.968	3.588	4.197	4.518	4.631	4.578
25.508	1.109	4.547	5.457	6.076	6.487	6.732
44.496	1.085	5.717	7.272	8.552	9.625	10.528
24.509	1.167	4.274	4.973	5.375	5.571	5.609
32.504	1.121	4.962	6.1	6.979	7.667	8.2
19.576	0.979	4.493	5.545	6.349	6.978	7.47
22.544	1.025	4.754	6.019	7.041	7.87	8.534
31.516	1.087	4.992	6.133	6.987	7.628	8.097
17.57	1.034	4.127	4.946	5.498	5.862	6.078
29.474	1.179	4.615	5.522	6.166	6.631	6.957
26.5	1.152	4.453	5.262	5.791	6.13	6.323
17.577	1.034	4.232	5.218	5.976	6.566	7.013
15.587	0.974	4.115	5.014	5.657	6.11	6.405
34.49	1.113	5.242	6.668	7.869	8.896	9.774
25.584	1.023	4.606	5.464	6.016	6.354	6.526
21.536	1.026	4.515	5.506	6.213	6.71	7.038
26.575	1.022	4.753	5.738	6.428	6.906	7.22
17.536	1.032	4.322	5.412	6.258	6.915	7.413
11.63	0.969	3.652	4.37	4.839	5.127	5.266
24.539	1.067	4.649	5.716	6.534	7.175	7.674
21.523	1.076	4.41	5.344	6.001	6.458	6.756
46.52	1.057	5.875	7.596	9.089	10.397	11.546
28.526	1.097	4.818	5.938	6.821	7.535	8.116
25.541	1.064	4.899	6.239	7.349	8.275	9.046
23.488	1.118	4.495	5.494	6.229	6.769	7.146
30.505	1.089	4.91	5.987	6.765	7.324	7.706
21.523	1.076	4.356	5.222	5.799	6.163	6.357
26.523	1.153	4.448	5.25	5.77	6.097	6.275
25.519	1.063	4.696	5.753	6.555	7.18	7.669
45.501	1.11	5.565	6.996	8.183	9.18	10.017
15.588	0.974	3.97	4.654	5.047	5.238	5.274
12.583	0.968	3.854	4.741	5.407	5.91	6.276

19.499	1.083	4.162	4.921	5.384	5.632	5.708
17.549	1.032	4.048	4.755	5.176	5.397	5.464
24.51	1.066	4.621	5.64	6.402	6.982	7.42
20.516	1.08	4.238	5.022	5.504	5.767	5.855
18.53	1.029	4.471	5.671	6.638	7.417	8.035
34.506	1.078	5.247	6.568	7.618	8.47	9.164
28.492	1.096	4.813	5.908	6.739	7.378	7.86
26.516	1.061	4.694	5.662	6.335	6.796	7.093
17.546	1.032	4.211	5.156	5.86	6.387	6.768
18.56	0.977	4.624	5.938	7.022	7.921	8.665
30.553	1.018	5.121	6.39	7.404	8.233	8.914
30.505	1.089	5.085	6.442	7.562	8.497	9.275
28.528	1.097	4.771	5.832	6.645	7.28	7.769
25.515	1.109	4.629	5.688	6.513	7.167	7.679
16.563	0.974	4.4	5.58	6.521	7.272	7.862
16.559	0.974	4.303	5.333	6.1	6.664	7.063
25.528	1.021	5.046	6.486	7.694	8.724	9.61
19.531	1.028	4.546	5.767	6.756	7.558	8.201
12.619	0.971	3.736	4.429	4.856	5.098	5.192
19.531	1.028	4.546	5.767	6.756	7.558	8.201
17.561	1.033	4.158	5.022	5.636	6.079	6.39
29.501	1.093	4.988	6.255	7.279	8.121	8.81
26.515	1.105	4.658	5.649	6.352	6.841	7.16
26.515	1.105	4.688	5.726	6.485	7.035	7.416
20.564	0.979	4.906	6.421	7.696	8.772	9.68
34.521	1.046	5.286	6.628	7.709	8.59	9.307
15.579	1.039	4.024	4.925	5.596	6.096	6.45
16.588	0.976	4.045	4.721	5.093	5.25	5.239
33.474	1.116	5.309	6.856	8.172	9.302	10.272
21.519	1.076	4.497	5.534	6.284	6.815	7.17
42.494	1.118	5.404	6.726	7.791	8.66	9.366
20.533	1.027	4.45	5.438	6.153	6.664	7.007
9.625	0.962	3.273	3.744	3.928	3.907	3.722
24.484	1.113	4.55	5.57	6.35	6.949	7.399
35.521	1.076	5.27	6.598	7.665	8.54	9.259
17.574	0.976	4.412	5.512	6.362	7.022	7.524
28.512	1.056	4.921	6.102	7.034	7.781	8.377
17.537	1.032	4.18	5.057	5.66	6.061	6.301
23.503	1.068	4.587	5.614	6.364	6.913	7.301
31.485	1.086	5.1	6.409	7.469	8.339	9.052
18.542	1.03	4.262	5.158	5.778	6.195	6.449
26.546	1.062	4.814	5.962	6.861	7.58	8.155
23.543	1.024	4.756	5.93	6.835	7.537	8.074
20.563	1.028	4.52	5.589	6.388	6.985	7.421
18.549	1.03	4.354	5.397	6.2	6.818	7.281
28.529	1.019	4.859	5.855	6.54	7.004	7.297
22.533	1.024	4.864	6.264	7.434	8.425	9.266
22.531	1.073	4.42	5.298	5.885	6.261	6.466
19.533	1.028	4.251	5.041	5.532	5.812	5.929
23.516	1.069	4.567	5.587	6.348	6.919	7.332

30.505	1.089	5.087	6.443	7.564	8.5	9.28
15.593	0.975	4.179	5.147	5.854	6.364	6.709
23.507	1.119	4.444	5.389	6.088	6.609	6.983
34.554	1.08	5.057	6.184	7.056	7.742	8.277
13.563	1.043	3.629	4.192	4.467	4.536	4.442
15.585	0.974	4.007	4.754	5.246	5.569	5.765
18.483	1.087	4.204	5.113	5.748	6.179	6.443
21.528	1.025	4.678	5.899	6.87	7.645	8.255
25.512	1.02	5.157	6.743	8.09	9.24	10.224
21.513	1.024	4.581	5.678	6.521	7.174	7.673
17.561	0.976	4.405	5.503	6.36	7.032	7.55
19.575	1.03	4.329	5.235	5.868	6.306	6.589
28.471	1.095	5.086	6.53	7.723	8.713	9.532
25.493	1.108	4.548	5.457	6.076	6.485	6.728
21.578	1.028	4.501	5.479	6.176	6.663	6.981
29.509	1.054	5.252	6.809	8.122	9.232	10.17
8.652	0.865	3.425	4.094	4.514	4.76	4.869
33.523	1.048	5.143	6.353	7.293	8.036	8.62
44.498	1.112	5.495	6.852	7.948	8.849	9.588
33.547	1.017	5.469	7.054	8.392	9.53	10.502
64.502	1.132	5.903	7.289	8.406	9.327	10.089
29.503	1.135	4.722	5.687	6.368	6.846	7.164
24.525	1.066	4.565	5.498	6.142	6.576	6.842
24.527	1.066	4.709	5.856	6.76	7.484	8.062
19.549	1.029	4.368	5.343	6.059	6.583	6.948
31.459	1.124	5.203	6.707	7.977	9.057	9.975
24.536	1.067	4.641	5.677	6.443	7.017	7.441
20.558	1.028	4.476	5.525	6.337	6.978	7.481

DP06	DP07	DP08	DP09	DP10	DP11	DP12
7.13	7.288	7.348	7.32	7.211	7.026	6.771
9.992	10.588	11.087	11.498	11.827	12.08	12.262
8.116	8.313	8.404	8.399	8.308	8.138	7.894
5.083	4.994	4.81	4.54	4.19	3.768	3.282
7.17	7.33	7.38	7.329	7.186	6.957	6.65
5.397	5.271	5.047	4.733	4.338	3.868	3.333
9.328	9.737	10.046	10.268	10.41	10.48	10.484
2.992	2.481	1.895	1.289	0.749	0.36	0.145
6.859	6.851	6.734	6.521	6.219	5.836	5.38
8.271	8.571	8.759	8.845	8.838	8.747	8.577
9.389	9.733	9.982	10.143	10.224	10.231	10.169
9.734	10.175	10.509	10.745	10.894	10.961	10.954
7.101	7.171	7.139	7.016	6.809	6.525	6.17
8.111	8.314	8.415	8.424	8.346	8.189	7.957
7.182	7.181	7.069	6.858	6.559	6.18	5.728
8.491	8.76	8.915	8.968	8.93	8.807	8.606
5.391	5.274	5.059	4.756	4.373	3.916	3.394
9.47	9.85	10.132	10.325	10.437	10.473	10.439
9.34	9.841	10.237	10.536	10.748	10.88	10.937
8.034	8.276	8.413	8.457	8.417	8.299	8.11
7.246	7.361	7.38	7.309	7.157	6.929	6.63
6.616	6.6	6.476	6.255	5.947	5.558	5.095
9.008	9.456	9.803	10.057	10.227	10.321	10.343
8.749	9.094	9.329	9.464	9.509	9.47	9.355
8.517	8.767	8.914	8.968	8.937	8.829	8.649
8.419	8.734	8.955	9.088	9.142	9.123	9.035
10.815	11.554	12.187	12.725	13.175	13.543	13.836
10.865	11.494	12.027	12.474	12.84	13.132	13.354
8.092	8.333	8.472	8.52	8.486	8.376	8.196
10.528	11.184	11.732	12.181	12.54	12.817	13.017
9.63	10.015	10.299	10.49	10.597	10.626	10.581
10.621	11.202	11.683	12.075	12.383	12.615	12.777
7.824	8.039	8.145	8.152	8.071	7.908	7.67
7.461	7.563	7.553	7.445	7.246	6.965	6.609
8.302	8.494	8.583	8.578	8.487	8.317	8.075
10.962	11.644	12.217	12.691	13.076	13.38	13.609
7.491	7.64	7.688	7.645	7.518	7.313	7.036
4.637	4.356	3.973	3.501	2.954	2.35	1.723
10.865	11.572	12.173	12.679	13.096	13.432	13.693
8.713	9.034	9.247	9.365	9.394	9.343	9.216
9.082	9.396	9.602	9.711	9.73	9.668	9.531
5.549	5.362	5.071	4.686	4.217	3.674	3.068
11.569	12.355	13.038	13.627	14.131	14.554	14.902
7.853	8.116	8.275	8.342	8.324	8.228	8.06
9.957	10.453	10.844	11.14	11.35	11.48	11.536
8.417	8.681	8.836	8.891	8.857	8.742	8.551
8.673	8.971	9.16	9.251	9.254	9.175	9.022
10.621	11.248	11.765	12.182	12.506	12.745	12.905
11.097	11.863	12.528	13.098	13.582	13.986	14.315

10.491	11.094	11.592	11.997	12.317	12.558	12.727
9.963	10.45	10.829	11.11	11.304	11.417	11.457
12.389	13.276	14.061	14.754	15.363	15.894	16.353
10.458	11.073	11.584	12	12.329	12.579	12.754
7.914	8.057	8.105	8.069	7.959	7.781	7.54
9.507	9.956	10.299	10.546	10.706	10.786	10.792
11.013	11.697	12.276	12.76	13.156	13.472	13.713
10.684	11.255	11.717	12.082	12.359	12.556	12.679
8.317	8.556	8.691	8.733	8.692	8.574	8.386
11.646	12.423	13.091	13.661	14.141	14.539	14.862
10.276	10.873	11.365	11.764	12.075	12.307	12.464
8.429	8.674	8.812	8.855	8.81	8.685	8.485
8.253	8.497	8.635	8.678	8.634	8.51	8.313
10.071	10.651	11.127	11.507	11.8	12.011	12.147
9.743	10.275	10.706	11.044	11.297	11.471	11.572
8.447	8.732	8.911	8.995	8.992	8.91	8.754
8.062	8.303	8.443	8.489	8.45	8.33	8.136
9.368	9.804	10.141	10.389	10.558	10.653	10.682
6.17	6.101	5.92	5.637	5.263	4.804	4.271
7.121	7.23	7.247	7.181	7.041	6.832	6.56
7.349	7.436	7.414	7.295	7.087	6.798	6.434
7.258	7.411	7.46	7.414	7.283	7.073	6.79
7.36	7.454	7.44	7.33	7.133	6.855	6.503
7.391	7.5	7.501	7.405	7.221	6.954	6.612
7.061	7.196	7.232	7.179	7.044	6.834	6.552
8.789	9.147	9.387	9.521	9.559	9.511	9.383
10.475	11.12	11.659	12.102	12.456	12.729	12.928
10.366	10.942	11.407	11.773	12.049	12.243	12.362
6.799	6.869	6.841	6.723	6.523	6.246	5.897
8.076	8.329	8.484	8.549	8.531	8.436	8.267
9.276	9.711	10.045	10.287	10.446	10.526	10.535
5.557	5.488	5.321	5.066	4.73	4.32	3.844
8.039	8.244	8.349	8.365	8.3	8.16	7.951
5.234	5.049	4.759	4.377	3.912	3.376	2.78
7.592	7.713	7.725	7.641	7.47	7.218	6.892
5.807	5.726	5.54	5.259	4.893	4.448	3.933
6.751	6.814	6.775	6.647	6.437	6.153	5.8
6.893	6.924	6.85	6.681	6.425	6.091	5.683
8.306	8.592	8.781	8.883	8.904	8.851	8.73
6.502	6.443	6.273	6.004	5.646	5.206	4.692
5.814	5.649	5.377	5.01	4.557	4.027	3.429
7.954	8.056	8.05	7.949	7.762	7.496	7.158
7	7.128	7.15	7.076	6.913	6.669	6.348
7.108	7.243	7.277	7.219	7.078	6.86	6.57
8.625	8.983	9.243	9.416	9.507	9.524	9.472
14.037	15.162	16.184	17.111	17.954	18.717	19.408
8.217	8.41	8.505	8.51	8.436	8.289	8.075
6.837	6.842	6.738	6.539	6.253	5.889	5.451
7.329	7.398	7.369	7.251	7.054	6.784	6.446
10.345	10.752	11.053	11.258	11.376	11.416	11.382

10.392	10.949	11.399	11.752	12.019	12.206	12.319
10.618	11.22	11.712	12.107	12.414	12.641	12.794
8.662	8.954	9.144	9.241	9.252	9.185	9.045
8.189	8.456	8.617	8.682	8.661	8.56	8.385
7.345	7.495	7.528	7.454	7.285	7.028	6.69
6.577	6.529	6.371	6.116	5.772	5.348	4.851
10.262	10.814	11.275	11.654	11.955	12.185	12.347
9.422	9.87	10.203	10.435	10.575	10.632	10.612
8.835	9.26	9.578	9.801	9.935	9.989	9.968
6.583	6.543	6.388	6.131	5.78	5.345	4.834
9.736	10.237	10.625	10.914	11.111	11.224	11.261
8.177	8.444	8.611	8.688	8.684	8.604	8.455
9.213	9.655	9.995	10.244	10.41	10.498	10.514
9.375	9.737	9.986	10.136	10.195	10.173	10.075
7.03	7.108	7.094	6.997	6.824	6.58	6.269
6.728	6.799	6.77	6.649	6.444	6.16	5.804
8.05	8.371	8.602	8.748	8.816	8.811	8.738
2.914	2.475	1.97	1.436	0.931	0.521	0.25
9.522	9.936	10.246	10.462	10.591	10.64	10.616
7.456	7.652	7.743	7.74	7.649	7.479	7.236
1.967	1.398	0.854	0.433	0.181	0.065	0.021
13.799	14.86	15.803	16.64	17.383	18.04	18.618
8.17	8.453	8.632	8.714	8.708	8.619	8.454
10.067	10.628	11.098	11.485	11.794	12.03	12.197
3.977	3.587	3.097	2.527	1.907	1.288	0.75
6.383	6.328	6.173	5.928	5.603	5.204	4.737
7.503	7.666	7.715	7.661	7.515	7.282	6.971
8.264	8.539	8.701	8.762	8.733	8.621	8.433
9.155	9.503	9.745	9.891	9.951	9.931	9.837
11.479	12.218	12.853	13.395	13.852	14.233	14.542
10.901	11.507	12.002	12.398	12.704	12.929	13.079
8.543	8.827	8.996	9.064	9.039	8.93	8.743
8.823	9.122	9.316	9.415	9.428	9.362	9.222
6.184	6.117	5.939	5.66	5.289	4.835	4.305
5.71	5.545	5.287	4.948	4.535	4.056	3.519
6.881	7.029	7.076	7.03	6.9	6.693	6.414
11.957	12.781	13.503	14.132	14.676	15.143	15.538
7.895	8.064	8.129	8.103	7.992	7.806	7.548
8.582	8.942	9.197	9.356	9.427	9.419	9.336
9.066	9.394	9.62	9.755	9.806	9.78	9.683
7.163	7.359	7.451	7.447	7.355	7.181	6.931
5.523	5.466	5.309	5.061	4.73	4.323	3.846
7.133	7.139	7.045	6.862	6.598	6.26	5.852
8.848	9.238	9.519	9.703	9.796	9.809	9.746
6.914	6.941	6.857	6.675	6.403	6.049	5.62
6.105	6.088	5.967	5.751	5.45	5.07	4.617
9.981	10.539	10.992	11.348	11.617	11.806	11.92
7.79	7.989	8.09	8.102	8.034	7.893	7.684
4.002	3.641	3.189	2.665	2.091	1.505	0.961
8.583	8.947	9.218	9.403	9.509	9.54	9.501

6.899	6.972	6.935	6.799	6.572	6.262	5.876
9.732	10.281	10.727	11.079	11.345	11.532	11.646
8.405	8.72	8.935	9.059	9.098	9.058	8.944
6.484	6.409	6.225	5.944	5.576	5.128	4.609
8.917	9.23	9.44	9.558	9.592	9.551	9.439
8.422	8.749	8.974	9.107	9.156	9.127	9.026
8.215	8.511	8.7	8.792	8.796	8.718	8.566
6.168	6.104	5.938	5.682	5.345	4.935	4.456
6.308	6.223	6.03	5.742	5.368	4.917	4.396
6.912	6.992	6.965	6.843	6.634	6.344	5.98
2.973	2.455	1.865	1.259	0.724	0.344	0.137
3.547	3.195	2.758	2.255	1.713	1.178	0.712
6.452	6.368	6.181	5.902	5.54	5.102	4.596
7.99	8.15	8.203	8.16	8.03	7.821	7.539
10.939	11.601	12.157	12.617	12.99	13.284	13.504
8.358	8.656	8.856	8.968	9	8.956	8.844
8.521	8.839	9.046	9.155	9.175	9.114	8.978
9.686	10.265	10.739	11.118	11.41	11.622	11.759
4.387	4.077	3.664	3.162	2.59	1.972	1.355
6.839	6.826	6.709	6.497	6.199	5.823	5.373
11.288	11.923	12.448	12.874	13.21	13.464	13.643
5.52	5.323	5.032	4.66	4.213	3.701	3.134
8.601	8.885	9.065	9.15	9.149	9.067	8.912
7.846	8.12	8.302	8.399	8.416	8.358	8.231
9.053	9.445	9.724	9.899	9.982	9.979	9.898
8.423	8.626	8.72	8.718	8.629	8.461	8.219
6.173	6.163	6.06	5.872	5.607	5.269	4.862
7.166	7.272	7.284	7.211	7.057	6.829	6.532
6.394	6.36	6.232	6.018	5.724	5.357	4.921
7.335	7.547	7.658	7.679	7.615	7.474	7.261
6.565	6.606	6.542	6.383	6.138	5.814	5.417
10.523	11.158	11.691	12.133	12.493	12.776	12.99
6.563	6.485	6.309	6.046	5.704	5.291	4.812
7.225	7.29	7.247	7.108	6.88	6.572	6.189
7.399	7.463	7.427	7.3	7.091	6.807	6.453
7.775	8.021	8.163	8.211	8.175	8.062	7.877
5.278	5.177	4.975	4.682	4.308	3.859	3.347
8.049	8.316	8.483	8.559	8.549	8.46	8.296
6.923	6.977	6.931	6.796	6.58	6.289	5.929
12.557	13.446	14.226	14.908	15.501	16.011	16.447
8.581	8.945	9.215	9.4	9.506	9.537	9.498
9.683	10.203	10.618	10.939	11.176	11.334	11.42
7.385	7.502	7.511	7.422	7.244	6.984	6.648
7.942	8.052	8.052	7.956	7.772	7.508	7.171
6.406	6.33	6.144	5.858	5.484	5.028	4.5
6.331	6.283	6.141	5.915	5.611	5.235	4.792
8.045	8.324	8.515	8.626	8.663	8.63	8.531
10.715	11.291	11.758	12.127	12.407	12.605	12.729
5.183	4.985	4.692	4.318	3.869	3.358	2.794
6.523	6.664	6.708	6.664	6.539	6.338	6.066

5.64	5.447	5.145	4.746	4.259	3.695	3.067
5.403	5.232	4.966	4.612	4.181	3.68	3.119
7.738	7.951	8.069	8.099	8.048	7.921	7.723
5.797	5.612	5.318	4.926	4.446	3.889	3.266
8.512	8.864	9.104	9.244	9.293	9.258	9.146
9.727	10.175	10.523	10.78	10.954	11.05	11.075
8.208	8.44	8.567	8.599	8.545	8.412	8.205
7.254	7.3	7.247	7.105	6.882	6.585	6.22
7.021	7.161	7.2	7.145	7.005	6.788	6.498
9.273	9.764	10.15	10.442	10.648	10.777	10.833
9.47	9.918	10.267	10.527	10.706	10.808	10.84
9.918	10.439	10.853	11.168	11.394	11.536	11.603
8.13	8.38	8.529	8.588	8.564	8.464	8.294
8.068	8.347	8.527	8.617	8.623	8.553	8.412
8.314	8.644	8.867	8.993	9.032	8.991	8.877
7.32	7.453	7.477	7.401	7.237	6.99	6.668
10.372	11.025	11.58	12.045	12.427	12.732	12.964
8.705	9.085	9.354	9.523	9.601	9.596	9.513
5.159	5.014	4.767	4.427	4.003	3.502	2.937
8.705	9.084	9.354	9.523	9.601	9.596	9.513
6.591	6.697	6.716	6.655	6.52	6.315	6.043
9.367	9.807	10.141	10.38	10.532	10.604	10.602
7.335	7.386	7.327	7.172	6.93	6.607	6.212
7.653	7.767	7.771	7.679	7.499	7.24	6.908
10.444	11.084	11.615	12.048	12.393	12.658	12.85
9.882	10.331	10.668	10.904	11.048	11.108	11.09
6.677	6.791	6.804	6.725	6.562	6.321	6.009
5.088	4.815	4.435	3.961	3.404	2.781	2.115
11.105	11.818	12.425	12.936	13.361	13.708	13.981
7.376	7.454	7.421	7.289	7.069	6.768	6.394
9.931	10.373	10.706	10.94	11.085	11.149	11.138
7.208	7.285	7.251	7.118	6.896	6.591	6.211
3.399	2.959	2.428	1.837	1.24	0.718	0.347
7.719	7.923	8.025	8.034	7.96	7.808	7.585
9.844	10.312	10.677	10.947	11.132	11.239	11.274
7.888	8.133	8.271	8.313	8.267	8.14	7.937
8.841	9.19	9.434	9.586	9.651	9.638	9.551
6.404	6.39	6.272	6.062	5.768	5.398	4.957
7.555	7.693	7.73	7.674	7.536	7.321	7.035
9.629	10.087	10.438	10.693	10.862	10.95	10.966
6.566	6.564	6.457	6.258	5.973	5.612	5.179
8.609	8.955	9.205	9.368	9.45	9.457	9.395
8.471	8.747	8.918	8.995	8.988	8.903	8.748
7.721	7.906	7.99	7.984	7.897	7.737	7.51
7.609	7.819	7.922	7.929	7.849	7.688	7.453
7.451	7.487	7.421	7.262	7.021	6.703	6.316
9.98	10.581	11.082	11.492	11.818	12.068	12.245
6.527	6.464	6.289	6.016	5.653	5.209	4.691
5.912	5.782	5.554	5.241	4.852	4.394	3.877
7.608	7.765	7.813	7.763	7.624	7.402	7.103

9.925	10.45	10.867	11.188	11.419	11.568	11.642
6.915	6.998	6.973	6.849	6.638	6.345	5.977
7.229	7.363	7.395	7.336	7.192	6.97	6.676
8.684	8.976	9.166	9.263	9.274	9.207	9.067
4.21	3.86	3.41	2.876	2.28	1.657	1.066
5.857	5.855	5.77	5.605	5.366	5.057	4.681
6.564	6.56	6.446	6.233	5.931	5.548	5.09
8.722	9.063	9.293	9.421	9.457	9.409	9.283
11.066	11.784	12.392	12.901	13.321	13.658	13.919
8.04	8.294	8.446	8.508	8.486	8.387	8.218
7.933	8.199	8.358	8.421	8.396	8.291	8.112
6.74	6.778	6.715	6.559	6.319	6.001	5.61
10.206	10.752	11.185	11.519	11.761	11.92	12.004
6.834	6.82	6.702	6.489	6.19	5.812	5.361
7.157	7.209	7.152	6.998	6.756	6.433	6.037
10.962	11.626	12.177	12.628	12.988	13.266	13.468
4.86	4.746	4.538	4.243	3.867	3.42	2.91
9.07	9.401	9.628	9.761	9.808	9.777	9.672
10.188	10.667	11.037	11.31	11.494	11.597	11.626
11.331	12.037	12.635	13.138	13.554	13.892	14.159
10.717	11.226	11.632	11.945	12.173	12.326	12.408
7.349	7.421	7.392	7.274	7.074	6.799	6.454
6.967	6.97	6.865	6.663	6.374	6.006	5.563
8.515	8.86	9.107	9.266	9.345	9.348	9.283
7.177	7.286	7.29	7.198	7.02	6.763	6.433
10.754	11.411	11.96	12.412	12.777	13.061	13.272
7.745	7.948	8.063	8.1	8.064	7.962	7.797
7.864	8.142	8.323	8.418	8.431	8.369	8.236

DP13	DP14	DP15	DP16	DP17	DP18	DP19
6.449	6.065	5.622	5.123	4.574	3.977	3.34
12.378	12.432	12.426	12.365	12.25	12.084	11.87
7.581	7.204	6.767	6.273	5.726	5.128	4.485
2.742	2.166	1.583	1.04	0.599	0.299	0.132
6.27	5.822	5.311	4.74	4.116	3.447	2.744
2.745	2.124	1.505	0.945	0.513	0.24	0.099
10.426	10.312	10.143	9.924	9.655	9.34	8.98
0.051	0.016	0.005	0.001	0	0	0
4.855	4.268	3.627	2.942	2.234	1.543	0.934
8.335	8.026	7.653	7.221	6.733	6.192	5.602
10.041	9.851	9.603	9.299	8.943	8.535	8.08
10.878	10.738	10.536	10.277	9.965	9.6	9.187
5.748	5.263	4.721	4.126	3.484	2.807	2.117
7.656	7.29	6.862	6.376	5.835	5.243	4.603
5.21	4.632	4	3.323	2.616	1.907	1.245
8.334	7.996	7.595	7.136	6.622	6.056	5.443
2.819	2.207	1.591	1.023	0.571	0.274	0.116
10.337	10.173	9.949	9.669	9.334	8.949	8.514
10.926	10.85	10.714	10.52	10.273	9.974	9.626
7.855	7.538	7.162	6.731	6.248	5.716	5.137
6.265	5.837	5.35	4.808	4.215	3.578	2.906
4.565	3.974	3.33	2.648	1.953	1.294	0.742
10.299	10.193	10.029	9.809	9.537	9.215	8.846
9.168	8.914	8.597	8.222	7.79	7.305	6.77
8.401	8.09	7.72	7.294	6.814	6.284	5.706
8.883	8.671	8.402	8.08	7.707	7.285	6.816
14.06	14.217	14.313	14.351	14.333	14.264	14.145
13.51	13.605	13.641	13.623	13.552	13.431	13.264
7.951	7.646	7.282	6.864	6.394	5.875	5.309
13.146	13.209	13.21	13.152	13.039	12.874	12.659
10.469	10.293	10.057	9.764	9.417	9.018	8.571
12.872	12.905	12.879	12.797	12.663	12.479	12.246
7.362	6.989	6.555	6.064	5.52	4.925	4.285
6.182	5.691	5.139	4.532	3.875	3.178	2.457
7.764	7.39	6.956	6.466	5.923	5.33	4.69
13.769	13.863	13.897	13.873	13.794	13.664	13.486
6.692	6.285	5.82	5.299	4.728	4.11	3.452
1.125	0.633	0.302	0.125	0.046	0.016	0.005
13.884	14.009	14.074	14.081	14.035	13.937	13.79
9.021	8.76	8.438	8.059	7.625	7.139	6.605
9.325	9.053	8.721	8.331	7.887	7.392	6.848
2.416	1.751	1.128	0.624	0.292	0.119	0.044
15.181	15.395	15.548	15.642	15.682	15.67	15.609
7.825	7.528	7.172	6.761	6.298	5.786	5.228
11.524	11.447	11.31	11.117	10.869	10.57	10.222
8.29	7.963	7.576	7.131	6.631	6.081	5.483
8.799	8.513	8.166	7.763	7.307	6.801	6.247
12.991	13.01	12.964	12.857	12.694	12.477	12.209
14.576	14.772	14.907	14.985	15.009	14.982	14.906

12.828	12.865	12.843	12.765	12.632	12.449	12.217
11.427	11.334	11.181	10.971	10.708	10.394	10.032
16.745	17.073	17.342	17.554	17.713	17.82	17.88
12.859	12.9	12.88	12.803	12.67	12.487	12.254
7.241	6.887	6.48	6.022	5.517	4.965	4.371
10.73	10.603	10.417	10.173	9.876	9.529	9.133
13.884	13.991	14.036	14.023	13.956	13.837	13.669
12.733	12.722	12.652	12.525	12.344	12.112	11.832
8.132	7.818	7.447	7.022	6.546	6.023	5.455
15.115	15.302	15.429	15.499	15.514	15.479	15.396
12.552	12.575	12.537	12.442	12.291	12.089	11.837
8.216	7.882	7.486	7.033	6.525	5.966	5.358
8.047	7.717	7.327	6.88	6.379	5.828	5.23
12.213	12.213	12.151	12.03	11.854	11.626	11.347
11.603	11.57	11.476	11.324	11.117	10.858	10.55
8.529	8.24	7.89	7.484	7.023	6.512	5.952
7.873	7.545	7.156	6.71	6.21	5.659	5.06
10.649	10.557	10.41	10.212	9.964	9.668	9.327
3.67	3.014	2.323	1.634	1.01	0.531	0.237
6.228	5.84	5.397	4.904	4.362	3.778	3.156
6	5.502	4.944	4.33	3.669	2.969	2.251
6.44	6.027	5.555	5.029	4.452	3.829	3.169
6.081	5.595	5.049	4.448	3.797	3.105	2.391
6.201	5.724	5.186	4.593	3.949	3.263	2.551
6.205	5.796	5.328	4.806	4.233	3.615	2.961
9.182	8.912	8.578	8.184	7.734	7.23	6.676
13.056	13.119	13.12	13.063	12.951	12.787	12.573
12.412	12.398	12.323	12.191	12.005	11.769	11.485
5.481	5.002	4.464	3.874	3.238	2.571	1.898
8.03	7.728	7.364	6.942	6.464	5.934	5.355
10.477	10.355	10.174	9.937	9.647	9.307	8.918
3.308	2.724	2.112	1.504	0.955	0.527	0.252
7.678	7.345	6.955	6.511	6.016	5.473	4.885
2.148	1.515	0.943	0.503	0.23	0.092	0.034
6.497	6.038	5.518	4.943	4.317	3.645	2.939
3.355	2.729	2.075	1.434	0.871	0.453	0.203
5.383	4.907	4.377	3.797	3.176	2.526	1.872
5.209	4.672	4.079	3.437	2.759	2.067	1.403
8.544	8.297	7.994	7.637	7.229	6.772	6.269
4.111	3.471	2.787	2.082	1.402	0.819	0.406
2.779	2.1	1.434	0.853	0.43	0.185	0.071
6.753	6.286	5.761	5.18	4.549	3.873	3.161
5.957	5.5	4.981	4.406	3.781	3.114	2.422
6.214	5.795	5.318	4.787	4.205	3.579	2.918
9.355	9.178	8.943	8.655	8.316	7.928	7.494
20.031	20.591	21.091	21.535	21.926	22.266	22.557
7.797	7.46	7.067	6.621	6.124	5.579	4.989
4.948	4.383	3.763	3.099	2.407	1.719	1.091
6.044	5.582	5.064	4.493	3.875	3.217	2.532
11.281	11.116	10.892	10.612	10.279	9.895	9.463

12.364	12.344	12.265	12.129	11.939	11.698	11.409
12.878	12.899	12.859	12.763	12.614	12.414	12.165
8.836	8.565	8.233	7.845	7.404	6.913	6.374
8.141	7.834	7.466	7.042	6.565	6.037	5.461
6.278	5.797	5.252	4.649	3.992	3.292	2.563
4.288	3.666	2.998	2.302	1.615	0.999	0.529
12.445	12.483	12.464	12.39	12.265	12.09	11.868
10.521	10.365	10.148	9.873	9.544	9.164	8.736
9.878	9.723	9.507	9.233	8.904	8.525	8.096
4.253	3.612	2.922	2.207	1.509	0.897	0.451
11.226	11.124	10.96	10.737	10.459	10.129	9.749
8.241	7.966	7.633	7.245	6.805	6.316	5.779
10.463	10.35	10.178	9.95	9.669	9.339	8.96
9.908	9.676	9.384	9.035	8.633	8.181	7.681
5.896	5.464	4.975	4.434	3.846	3.217	2.56
5.38	4.894	4.349	3.753	3.113	2.445	1.777
8.6	8.401	8.144	7.831	7.467	7.052	6.59
0.104	0.039	0.014	0.004	0.001	0	0
10.523	10.365	10.147	9.871	9.541	9.158	8.727
6.924	6.548	6.112	5.621	5.076	4.484	3.847
0.006	0.002	0	0	0	0	0
19.123	19.562	19.937	20.255	20.517	20.728	20.89
8.217	7.913	7.545	7.118	6.634	6.097	5.51
12.3	12.341	12.324	12.252	12.128	11.954	11.733
0.366	0.152	0.056	0.019	0.006	0.002	0
4.209	3.626	2.998	2.342	1.687	1.086	0.607
6.587	6.135	5.619	5.045	4.418	3.743	3.032
8.176	7.854	7.471	7.032	6.54	5.997	5.406
9.676	9.451	9.166	8.824	8.429	7.984	7.49
14.786	14.968	15.093	15.163	15.181	15.15	15.073
13.161	13.178	13.135	13.036	12.884	12.683	12.433
8.485	8.16	7.772	7.326	6.825	6.272	5.67
9.015	8.743	8.412	8.025	7.584	7.092	6.553
3.708	3.055	2.365	1.675	1.045	0.555	0.25
2.933	2.314	1.69	1.109	0.635	0.314	0.137
6.069	5.662	5.198	4.68	4.112	3.501	2.857
15.865	16.128	16.332	16.48	16.574	16.617	16.612
7.224	6.838	6.394	5.895	5.345	4.746	4.104
9.185	8.969	8.693	8.36	7.974	7.536	7.05
9.519	9.293	9.007	8.666	8.271	7.825	7.332
6.611	6.225	5.777	5.271	4.713	4.105	3.457
3.309	2.722	2.106	1.495	0.944	0.517	0.245
5.381	4.849	4.262	3.627	2.953	2.26	1.581
9.613	9.415	9.157	8.841	8.472	8.051	7.582
5.122	4.561	3.944	3.279	2.582	1.88	1.224
4.098	3.522	2.897	2.243	1.594	1.005	0.547
11.965	11.945	11.864	11.726	11.533	11.29	10.997
7.411	7.08	6.692	6.251	5.76	5.221	4.638
0.529	0.249	0.103	0.038	0.013	0.004	0.001
9.396	9.229	9.004	8.722	8.387	8.001	7.567

5.419	4.897	4.315	3.681	3.004	2.304	1.615
11.69	11.67	11.589	11.452	11.259	11.016	10.723
8.761	8.511	8.2	7.83	7.405	6.927	6.399
4.025	3.386	2.705	2.009	1.342	0.779	0.384
9.262	9.024	8.729	8.379	7.979	7.529	7.034
8.858	8.626	8.335	7.988	7.588	7.137	6.638
8.344	8.057	7.708	7.302	6.842	6.33	5.77
3.917	3.325	2.693	2.041	1.409	0.858	0.45
3.814	3.179	2.509	1.832	1.198	0.679	0.329
5.547	5.049	4.492	3.882	3.227	2.542	1.853
0.048	0.015	0.004	0.001	0	0	0
0.37	0.167	0.067	0.025	0.008	0.003	0.001
4.028	3.408	2.746	2.067	1.412	0.846	0.434
7.189	6.775	6.301	5.771	5.188	4.556	3.881
13.655	13.743	13.77	13.741	13.658	13.525	13.343
8.667	8.429	8.134	7.785	7.385	6.935	6.44
8.774	8.506	8.179	7.797	7.361	6.877	6.346
11.826	11.828	11.767	11.648	11.474	11.247	10.97
0.809	0.409	0.176	0.067	0.023	0.007	0.002
4.857	4.279	3.646	2.97	2.269	1.581	0.969
13.753	13.797	13.78	13.707	13.579	13.4	13.172
2.524	1.897	1.294	0.776	0.402	0.181	0.073
8.687	8.398	8.048	7.64	7.177	6.663	6.1
8.038	7.783	7.47	7.101	6.68	6.208	5.69
9.743	9.52	9.234	8.888	8.485	8.03	7.523
7.91	7.536	7.104	6.615	6.073	5.482	4.844
4.392	3.863	3.283	2.662	2.023	1.402	0.859
6.169	5.744	5.262	4.726	4.141	3.513	2.851
4.421	3.865	3.259	2.616	1.961	1.334	0.798
6.982	6.639	6.237	5.779	5.269	4.71	4.107
4.953	4.428	3.847	3.219	2.558	1.889	1.258
13.14	13.229	13.261	13.239	13.167	13.047	12.881
4.273	3.681	3.045	2.38	1.716	1.106	0.618
5.737	5.221	4.645	4.016	3.342	2.635	1.925
6.034	5.554	5.016	4.427	3.79	3.116	2.419
7.625	7.311	6.937	6.508	6.026	5.495	4.917
2.781	2.18	1.575	1.019	0.574	0.279	0.12
8.063	7.763	7.401	6.981	6.504	5.975	5.396
5.504	5.02	4.479	3.888	3.253	2.588	1.916
16.812	17.113	17.354	17.537	17.667	17.747	17.778
9.393	9.226	9	8.718	8.382	7.996	7.561
11.44	11.396	11.294	11.136	10.925	10.664	10.356
6.242	5.77	5.237	4.648	4.007	3.324	2.611
6.766	6.298	5.77	5.188	4.554	3.875	3.159
3.906	3.256	2.567	1.869	1.213	0.678	0.321
4.287	3.726	3.118	2.476	1.827	1.216	0.708
8.371	8.152	7.877	7.548	7.169	6.74	6.265
12.783	12.772	12.701	12.573	12.391	12.159	11.878
2.196	1.594	1.038	0.59	0.29	0.126	0.05
5.728	5.327	4.868	4.354	3.791	3.186	2.55

2.393	1.708	1.075	0.575	0.259	0.102	0.036
2.512	1.883	1.278	0.759	0.387	0.172	0.068
7.458	7.131	6.744	6.301	5.805	5.259	4.666
2.593	1.9	1.24	0.694	0.326	0.132	0.048
8.962	8.711	8.398	8.025	7.598	7.117	6.588
11.033	10.927	10.762	10.54	10.265	9.938	9.563
7.93	7.59	7.19	6.733	6.223	5.661	5.053
5.79	5.299	4.752	4.153	3.508	2.829	2.135
6.14	5.719	5.24	4.705	4.12	3.492	2.829
10.823	10.75	10.618	10.43	10.19	9.9	9.563
10.806	10.71	10.555	10.344	10.081	9.768	9.407
11.598	11.526	11.392	11.199	10.95	10.649	10.297
8.058	7.761	7.407	6.998	6.538	6.029	5.474
8.203	7.932	7.601	7.213	6.772	6.28	5.739
8.694	8.448	8.143	7.782	7.368	6.904	6.393
6.276	5.819	5.301	4.726	4.101	3.432	2.732
13.129	13.229	13.269	13.252	13.181	13.059	12.887
9.36	9.139	8.856	8.513	8.115	7.665	7.164
2.324	1.695	1.103	0.62	0.297	0.124	0.047
9.359	9.139	8.855	8.513	8.115	7.664	7.164
5.708	5.314	4.863	4.359	3.806	3.212	2.587
10.532	10.399	10.205	9.956	9.653	9.301	8.9
5.749	5.224	4.643	4.009	3.333	2.627	1.918
6.509	6.047	5.526	4.952	4.328	3.661	2.961
12.972	13.031	13.031	12.974	12.864	12.704	12.495
11.001	10.844	10.625	10.346	10.012	9.626	9.19
5.63	5.19	4.692	4.141	3.543	2.908	2.254
1.452	0.864	0.433	0.184	0.068	0.023	0.007
14.187	14.329	14.413	14.44	14.415	14.34	14.217
5.953	5.45	4.889	4.277	3.619	2.926	2.218
11.058	10.914	10.71	10.45	10.136	9.772	9.36
5.76	5.245	4.67	4.041	3.366	2.659	1.947
0.142	0.051	0.017	0.005	0.001	0	0
7.295	6.944	6.535	6.07	5.554	4.99	4.38
11.241	11.145	10.99	10.78	10.517	10.204	9.844
7.666	7.328	6.93	6.473	5.962	5.399	4.788
9.397	9.18	8.903	8.57	8.184	7.748	7.264
4.453	3.891	3.28	2.632	1.971	1.339	0.8
6.683	6.27	5.798	5.271	4.694	4.07	3.407
10.912	10.795	10.618	10.384	10.097	9.758	9.371
4.682	4.125	3.515	2.865	2.192	1.534	0.949
9.267	9.077	8.83	8.527	8.172	7.767	7.315
8.528	8.248	7.91	7.519	7.078	6.588	6.054
7.219	6.87	6.465	6.008	5.502	4.949	4.353
7.149	6.781	6.353	5.868	5.33	4.742	4.11
5.863	5.349	4.778	4.157	3.49	2.791	2.082
12.356	12.403	12.392	12.324	12.202	12.03	11.81
4.105	3.462	2.775	2.068	1.388	0.807	0.397
3.308	2.698	2.069	1.454	0.909	0.494	0.234
6.734	6.298	5.801	5.245	4.636	3.979	3.282

11.645	11.582	11.457	11.273	11.035	10.744	10.403
5.54	5.039	4.479	3.866	3.209	2.522	1.834
6.315	5.89	5.407	4.868	4.278	3.644	2.973
8.859	8.588	8.257	7.869	7.429	6.937	6.398
0.585	0.271	0.109	0.039	0.013	0.004	0.001
4.244	3.749	3.204	2.619	2.014	1.422	0.896
4.564	3.978	3.339	2.662	1.972	1.315	0.763
9.085	8.819	8.491	8.102	7.658	7.161	6.614
14.11	14.235	14.299	14.303	14.253	14.151	13.998
7.982	7.683	7.327	6.914	6.449	5.935	5.373
7.864	7.552	7.18	6.752	6.27	5.738	5.159
5.151	4.63	4.051	3.422	2.755	2.072	1.415
12.016	11.963	11.848	11.675	11.448	11.169	10.84
4.843	4.264	3.63	2.953	2.253	1.566	0.957
5.574	5.048	4.465	3.831	3.156	2.455	1.759
13.599	13.666	13.672	13.621	13.516	13.36	13.156
2.353	1.773	1.212	0.73	0.38	0.173	0.071
9.5	9.265	8.97	8.619	8.214	7.759	7.256
11.585	11.48	11.314	11.092	10.815	10.487	10.111
14.36	14.498	14.58	14.606	14.582	14.509	14.389
12.426	12.384	12.285	12.133	11.931	11.681	11.386
6.044	5.574	5.047	4.467	3.84	3.173	2.483
5.054	4.482	3.855	3.182	2.48	1.778	1.134
9.151	8.959	8.708	8.401	8.042	7.633	7.176
6.036	5.576	5.06	4.49	3.874	3.218	2.537
13.413	13.491	13.508	13.468	13.376	13.232	13.04
7.572	7.289	6.951	6.561	6.12	5.63	5.095
8.037	7.776	7.456	7.081	6.652	6.173	5.646

DP20	SP01	SP02	SP03	SP04	SP05	SP06
2.675	3.946	5.035	5.849	6.461	6.912	7.227
11.609	4.673	6.114	7.317	8.339	9.211	9.954
3.8	4.438	5.624	6.521	7.205	7.718	8.085
0.053	3.257	3.987	4.473	4.789	4.969	5.029
2.032	3.893	4.968	5.786	6.409	6.869	7.186
0.038	3.711	4.51	5.013	5.313	5.454	5.464
8.577	4.835	6.125	7.159	8.006	8.703	9.269
0	2.984	3.53	3.757	3.745	3.541	3.181
0.481	4.175	5.214	5.943	6.443	6.762	6.928
4.965	4.252	5.447	6.39	7.141	7.73	8.178
7.578	4.963	6.282	7.312	8.137	8.802	9.333
8.728	4.934	6.357	7.495	8.418	9.168	9.771
1.452	4.236	5.271	6.007	6.528	6.883	7.099
3.921	4.649	5.848	6.743	7.418	7.922	8.283
0.704	4.167	5.177	5.877	6.356	6.659	6.817
4.784	4.357	5.637	6.642	7.434	8.048	8.508
0.045	3.508	4.36	4.93	5.292	5.486	5.54
8.032	4.983	6.286	7.301	8.113	8.767	9.292
9.232	4.343	5.672	6.777	7.704	8.477	9.116
4.514	4.224	5.37	6.255	6.946	7.479	7.877
2.218	4.101	5.237	6.074	6.693	7.137	7.438
0.362	3.96	4.945	5.634	6.109	6.415	6.578
8.431	4.256	5.558	6.629	7.52	8.258	8.863
6.188	4.356	5.661	6.711	7.558	8.234	8.76
5.084	4.752	5.968	6.886	7.587	8.118	8.508
6.304	4.467	5.637	6.556	7.297	7.895	8.367
13.979	4.728	6.27	7.59	8.732	9.725	10.587
13.051	5.196	6.696	7.929	8.975	9.877	10.658
4.7	4.376	5.585	6.506	7.212	7.748	8.143
12.398	4.767	6.268	7.534	8.613	9.533	10.317
8.078	5.178	6.519	7.565	8.399	9.067	9.598
11.968	5.046	6.492	7.683	8.695	9.566	10.313
3.605	4.168	5.332	6.228	6.919	7.442	7.82
1.744	4.302	5.415	6.232	6.828	7.246	7.514
4.01	4.675	5.852	6.739	7.418	7.932	8.305
13.26	4.991	6.571	7.915	9.067	10.054	10.898
2.766	4.328	5.403	6.204	6.807	7.251	7.561
0.002	3.277	4.111	4.623	4.898	4.983	4.911
13.598	4.907	6.425	7.717	8.836	9.81	10.656
6.024	4.449	5.757	6.795	7.623	8.276	8.781
6.258	4.835	6.098	7.082	7.864	8.483	8.964
0.015	3.87	4.747	5.306	5.631	5.772	5.759
15.501	5.249	6.84	8.208	9.405	10.456	11.38
4.626	3.977	5.133	6.042	6.766	7.336	7.772
9.828	4.935	6.336	7.473	8.418	9.205	9.858
4.84	4.345	5.607	6.593	7.37	7.974	8.429
5.649	4.371	5.654	6.662	7.463	8.092	8.574
11.892	4.902	6.452	7.75	8.851	9.786	10.576
14.783	4.882	6.504	7.895	9.102	10.155	11.073

11.939	4.938	6.471	7.746	8.821	9.729	10.495
9.624	4.785	6.242	7.43	8.412	9.222	9.886
17.892	5.413	7.165	8.678	10.007	11.183	12.226
11.974	4.827	6.353	7.626	8.702	9.614	10.386
3.738	4.542	5.681	6.513	7.126	7.567	7.871
8.69	4.617	6.031	7.177	8.118	8.887	9.511
13.454	5.072	6.648	7.979	9.119	10.098	10.939
11.506	5.121	6.666	7.937	8.996	9.882	10.621
4.845	4.425	5.665	6.613	7.348	7.91	8.328
15.266	5.107	6.77	8.191	9.421	10.49	11.42
11.539	4.807	6.32	7.579	8.642	9.54	10.297
4.704	4.522	5.735	6.663	7.38	7.929	8.334
4.587	4.316	5.542	6.494	7.24	7.818	8.251
11.021	4.775	6.272	7.525	8.584	9.478	10.229
10.193	4.621	6.065	7.259	8.257	9.094	9.791
5.347	4.337	5.593	6.579	7.361	7.977	8.448
4.419	4.557	5.698	6.566	7.239	7.756	8.14
8.943	4.765	6.081	7.129	7.979	8.671	9.231
0.093	3.838	4.791	5.452	5.895	6.159	6.271
2.511	4.147	5.175	5.917	6.452	6.826	7.071
1.553	4.217	5.31	6.103	6.678	7.077	7.33
2.486	4.075	5.136	5.93	6.526	6.96	7.256
1.688	4.225	5.327	6.129	6.712	7.12	7.381
1.841	4.2	5.275	6.065	6.651	7.072	7.352
2.288	3.886	4.918	5.686	6.264	6.692	6.991
6.074	4.32	5.61	6.65	7.488	8.153	8.668
12.312	4.779	6.299	7.588	8.691	9.636	10.442
11.154	4.821	6.304	7.539	8.578	9.45	10.18
1.265	3.912	4.954	5.71	6.258	6.639	6.883
4.729	4.307	5.514	6.443	7.167	7.728	8.152
8.483	4.569	5.915	6.998	7.889	8.622	9.219
0.107	3.57	4.357	4.882	5.229	5.435	5.519
4.255	4.39	5.586	6.487	7.172	7.687	8.062
0.012	3.616	4.442	4.966	5.27	5.396	5.376
2.219	4.308	5.427	6.251	6.857	7.289	7.574
0.081	3.848	4.722	5.303	5.671	5.868	5.922
1.257	3.927	4.899	5.595	6.092	6.43	6.634
0.834	4.237	5.272	6	6.504	6.831	7.011
5.722	4.462	5.616	6.511	7.223	7.789	8.229
0.172	4.055	5.048	5.732	6.187	6.458	6.575
0.025	3.966	4.846	5.413	5.754	5.915	5.926
2.43	4.459	5.619	6.464	7.084	7.525	7.82
1.735	3.879	4.924	5.707	6.296	6.727	7.019
2.241	3.893	4.931	5.706	6.29	6.722	7.022
7.015	4.457	5.626	6.551	7.307	7.924	8.421
22.803	5.758	7.696	9.415	10.957	12.348	13.607
4.357	4.639	5.827	6.713	7.381	7.88	8.24
0.596	4.21	5.222	5.928	6.408	6.709	6.861
1.848	4.436	5.47	6.203	6.723	7.077	7.295
8.985	5.525	6.942	8.055	8.948	9.669	10.244

11.073	4.988	6.451	7.649	8.643	9.472	10.162
11.87	4.851	6.349	7.598	8.654	9.549	10.305
5.789	4.466	5.75	6.754	7.554	8.189	8.682
4.841	4.258	5.433	6.353	7.084	7.66	8.098
1.836	3.992	5.084	5.918	6.549	7.007	7.313
0.239	4.137	5.132	5.819	6.281	6.561	6.687
11.601	5.047	6.476	7.632	8.602	9.429	10.138
8.262	4.465	5.822	6.927	7.834	8.572	9.165
7.62	4.306	5.592	6.645	7.513	8.223	8.796
0.194	4.023	5.008	5.699	6.166	6.449	6.577
9.321	4.763	6.18	7.349	8.322	9.128	9.79
5.197	4.366	5.577	6.514	7.246	7.811	8.237
8.537	4.553	5.845	6.895	7.767	8.488	9.079
7.136	4.873	6.238	7.309	8.157	8.823	9.338
1.899	4.267	5.267	5.978	6.484	6.832	7.05
1.159	3.88	4.911	5.664	6.211	6.593	6.835
6.082	4.202	5.394	6.328	7.079	7.683	8.163
0	2.793	3.295	3.497	3.491	3.327	3.035
8.248	4.862	6.216	7.291	8.16	8.864	9.43
3.175	4.003	5.096	5.938	6.593	7.091	7.453
0	2.589	3.026	3.113	2.952	2.607	2.127
21.005	5.681	7.638	9.353	10.87	12.216	13.415
4.875	4.283	5.488	6.431	7.177	7.761	8.206
11.467	4.939	6.323	7.451	8.402	9.215	9.911
0	3.421	4.015	4.302	4.372	4.269	4.022
0.292	4.024	4.977	5.626	6.057	6.315	6.431
2.303	4.024	5.133	5.983	6.632	7.114	7.448
4.771	4.164	5.347	6.28	7.02	7.596	8.029
6.951	4.725	6.037	7.075	7.909	8.575	9.099
14.95	5.142	6.748	8.112	9.291	10.315	11.207
12.139	5.161	6.71	7.988	9.06	9.962	10.72
5.022	4.32	5.574	6.564	7.349	7.961	8.422
5.968	4.73	5.979	6.938	7.687	8.268	8.709
0.099	3.842	4.797	5.459	5.904	6.171	6.285
0.054	3.8	4.67	5.219	5.544	5.697	5.713
2.197	3.714	4.691	5.431	6.003	6.435	6.743
16.561	5.349	7.046	8.497	9.757	10.859	11.827
3.424	4.377	5.554	6.43	7.088	7.571	7.911
6.518	4.22	5.472	6.483	7.309	7.978	8.511
6.792	4.898	6.172	7.161	7.946	8.57	9.059
2.777	3.761	4.802	5.605	6.234	6.717	7.07
0.103	3.357	4.182	4.747	5.129	5.365	5.474
0.979	4.367	5.426	6.162	6.669	6.997	7.181
7.067	4.316	5.587	6.62	7.469	8.16	8.714
0.689	4.072	5.108	5.852	6.376	6.722	6.918
0.256	3.672	4.578	5.213	5.651	5.929	6.071
10.658	4.682	6.139	7.352	8.371	9.228	9.944
4.014	4.374	5.51	6.365	7.015	7.502	7.853
0	3.278	3.956	4.309	4.431	4.374	4.174
7.086	4.332	5.572	6.552	7.346	7.993	8.517

1	3.976	5.008	5.766	6.322	6.711	6.954
10.383	4.576	5.995	7.179	8.178	9.021	9.726
5.824	4.324	5.553	6.513	7.277	7.886	8.362
0.163	4.072	5.067	5.745	6.192	6.454	6.562
6.494	4.791	6.054	7.034	7.81	8.424	8.901
6.093	4.313	5.547	6.521	7.303	7.929	8.421
5.164	4.258	5.439	6.366	7.104	7.685	8.131
0.205	4.04	4.939	5.54	5.925	6.142	6.219
0.139	4.043	5.024	5.692	6.125	6.371	6.462
1.212	3.982	5.023	5.791	6.357	6.757	7.013
0	2.987	3.531	3.753	3.733	3.521	3.152
0	2.895	3.497	3.799	3.895	3.833	3.641
0.193	4.307	5.251	5.877	6.273	6.49	6.56
3.171	4.377	5.554	6.437	7.105	7.599	7.948
13.115	4.984	6.509	7.789	8.883	9.822	10.628
5.9	4.427	5.617	6.546	7.287	7.877	8.337
5.77	4.321	5.572	6.562	7.352	7.975	8.454
10.646	4.473	5.877	7.057	8.059	8.909	9.627
0.001	3.312	4.043	4.46	4.649	4.659	4.519
0.508	4.182	5.214	5.934	6.429	6.745	6.913
12.898	5.395	6.967	8.264	9.357	10.284	11.07
0.028	3.759	4.584	5.088	5.368	5.478	5.454
5.491	4.493	5.749	6.723	7.487	8.083	8.535
5.126	4.156	5.287	6.154	6.836	7.374	7.79
6.969	4.412	5.696	6.726	7.561	8.232	8.76
4.163	4.561	5.793	6.724	7.434	7.969	8.356
0.455	3.741	4.693	5.349	5.796	6.08	6.23
2.176	4.168	5.191	5.927	6.468	6.862	7.133
0.412	4.099	5.038	5.673	6.095	6.354	6.48
3.465	3.888	4.955	5.782	6.433	6.938	7.313
0.732	3.805	4.75	5.432	5.923	6.258	6.459
12.672	4.816	6.281	7.512	8.568	9.479	10.263
0.297	4.242	5.238	5.908	6.344	6.598	6.705
1.259	4.254	5.318	6.087	6.639	7.014	7.24
1.731	4.388	5.48	6.257	6.811	7.192	7.431
4.296	3.931	5.049	5.922	6.611	7.147	7.551
0.047	3.36	4.181	4.733	5.091	5.293	5.359
4.77	4.274	5.453	6.366	7.086	7.651	8.084
1.283	4.233	5.267	5.999	6.513	6.854	7.055
17.764	5.423	7.203	8.743	10.092	11.277	12.32
7.08	4.332	5.572	6.551	7.345	7.992	8.516
10.002	4.519	5.938	7.118	8.109	8.939	9.63
1.898	4.149	5.28	6.122	6.745	7.19	7.485
2.424	4.443	5.605	6.457	7.083	7.528	7.824
0.132	4.034	5.005	5.669	6.105	6.358	6.457
0.355	4.026	4.965	5.598	6.017	6.272	6.392
5.745	4.423	5.609	6.515	7.218	7.764	8.181
11.551	5.239	6.756	8.018	9.081	9.976	10.725
0.018	3.722	4.52	5.005	5.271	5.367	5.327
1.908	3.652	4.589	5.297	5.838	6.24	6.521

0.012	4.061	4.894	5.414	5.703	5.809	5.763
0.025	3.808	4.616	5.118	5.402	5.519	5.498
4.032	4.399	5.562	6.44	7.109	7.613	7.979
0.016	4.033	4.912	5.471	5.795	5.932	5.912
6.011	4.193	5.423	6.411	7.212	7.853	8.354
9.141	4.939	6.316	7.413	8.307	9.04	9.638
4.4	4.491	5.669	6.558	7.238	7.754	8.132
1.466	4.409	5.471	6.222	6.747	7.095	7.299
2.153	3.875	4.911	5.692	6.286	6.728	7.037
9.18	4.338	5.659	6.752	7.665	8.426	9.055
9	4.725	6.036	7.077	7.928	8.63	9.207
9.897	4.734	6.169	7.358	8.356	9.193	9.888
4.877	4.398	5.553	6.442	7.138	7.678	8.084
5.153	4.223	5.353	6.23	6.928	7.481	7.908
5.837	4.177	5.389	6.357	7.134	7.749	8.226
2.024	4.083	5.15	5.945	6.537	6.961	7.243
12.669	4.901	6.411	7.675	8.75	9.672	10.463
6.616	4.29	5.544	6.558	7.385	8.053	8.583
0.016	3.383	4.199	4.722	5.04	5.201	5.228
6.615	4.29	5.544	6.558	7.385	8.053	8.583
1.953	3.827	4.779	5.459	5.956	6.316	6.564
8.453	4.786	6.138	7.224	8.113	8.84	9.429
1.257	4.297	5.385	6.167	6.724	7.101	7.328
2.247	4.328	5.465	6.3	6.912	7.344	7.624
12.241	4.627	6.119	7.379	8.45	9.36	10.132
8.706	4.989	6.381	7.501	8.419	9.171	9.78
1.61	3.691	4.654	5.381	5.933	6.338	6.612
0.002	3.851	4.6	5.035	5.247	5.283	5.171
14.048	4.9	6.481	7.833	9.004	10.02	10.899
1.533	4.212	5.312	6.114	6.693	7.09	7.336
8.903	5.067	6.46	7.585	8.506	9.259	9.868
1.278	4.195	5.274	6.063	6.636	7.033	7.281
0	3.029	3.624	3.911	3.968	3.842	3.562
3.73	4.095	5.183	6.018	6.67	7.173	7.547
9.439	4.898	6.293	7.418	8.344	9.108	9.735
4.134	4.139	5.279	6.164	6.855	7.385	7.777
6.735	4.745	5.987	6.966	7.755	8.387	8.885
0.411	3.884	4.801	5.437	5.872	6.145	6.283
2.716	4.341	5.43	6.23	6.82	7.244	7.528
8.937	4.845	6.265	7.416	8.361	9.134	9.761
0.506	3.918	4.864	5.526	5.985	6.281	6.439
6.816	4.383	5.596	6.545	7.308	7.922	8.413
5.476	4.467	5.702	6.66	7.409	7.989	8.426
3.718	4.241	5.377	6.235	6.887	7.372	7.718
3.44	4.022	5.131	5.989	6.661	7.176	7.555
1.406	4.627	5.672	6.4	6.904	7.235	7.423
11.543	4.668	6.119	7.334	8.364	9.242	9.988
0.168	4.063	5.06	5.746	6.203	6.475	6.593
0.099	3.95	4.806	5.351	5.68	5.842	5.868
2.56	4.246	5.395	6.261	6.915	7.395	7.726

10.015	4.735	6.169	7.358	8.356	9.193	9.89
1.194	3.872	4.893	5.645	6.194	6.578	6.819
2.285	4.059	5.079	5.832	6.394	6.803	7.082
5.814	4.519	5.783	6.768	7.552	8.175	8.66
0	3.212	3.991	4.446	4.657	4.674	4.529
0.493	3.76	4.622	5.202	5.594	5.845	5.982
0.377	3.945	4.933	5.633	6.118	6.429	6.593
6.019	4.461	5.724	6.726	7.529	8.165	8.658
13.799	4.88	6.453	7.792	8.943	9.935	10.79
4.768	4.368	5.517	6.403	7.092	7.622	8.016
4.537	4.221	5.371	6.265	6.965	7.504	7.905
0.847	4.105	5.083	5.774	6.259	6.581	6.766
10.464	4.76	6.236	7.461	8.487	9.345	10.058
0.499	4.183	5.215	5.934	6.426	6.741	6.907
1.123	4.183	5.232	5.984	6.519	6.88	7.095
12.906	4.869	6.416	7.729	8.854	9.818	10.642
0.027	3.299	4.064	4.561	4.863	5.012	5.031
6.708	4.843	6.117	7.114	7.91	8.544	9.041
9.688	5.145	6.57	7.722	8.671	9.452	10.09
14.225	5.181	6.782	8.135	9.294	10.291	11.148
11.048	5.488	6.957	8.145	9.129	9.949	10.629
1.796	4.392	5.506	6.304	6.873	7.261	7.502
0.622	4.271	5.333	6.084	6.603	6.937	7.117
6.673	4.373	5.592	6.554	7.33	7.957	8.456
1.857	4.018	5.091	5.886	6.477	6.902	7.185
12.802	4.792	6.313	7.602	8.709	9.66	10.475
4.517	4.253	5.386	6.234	6.879	7.365	7.723
5.074	4.086	5.24	6.135	6.844	7.405	7.838

SP07	SP08	SP09	SP10	SP11	SP12	SP13
7.424	7.517	7.515	7.428	7.261	7.022	6.715
10.585	11.114	11.55	11.903	12.176	12.377	12.51
8.328	8.459	8.492	8.433	8.293	8.075	7.786
4.983	4.839	4.605	4.29	3.898	3.437	2.917
7.378	7.455	7.43	7.31	7.102	6.815	6.452
5.36	5.154	4.855	4.473	4.014	3.487	2.903
9.721	10.071	10.33	10.506	10.606	10.636	10.602
2.694	2.116	1.495	0.912	0.461	0.193	0.069
6.964	6.885	6.704	6.43	6.072	5.636	5.129
8.501	8.711	8.82	8.835	8.764	8.613	8.389
9.749	10.06	10.277	10.408	10.458	10.433	10.338
10.247	10.611	10.875	11.047	11.136	11.149	11.09
7.197	7.19	7.09	6.904	6.64	6.303	5.897
8.52	8.649	8.678	8.618	8.475	8.256	7.965
6.849	6.772	6.597	6.334	5.992	5.577	5.095
8.833	9.037	9.135	9.135	9.047	8.877	8.633
5.474	5.301	5.032	4.677	4.242	3.735	3.167
9.704	10.016	10.239	10.378	10.44	10.43	10.352
9.638	10.055	10.377	10.612	10.767	10.847	10.858
8.159	8.337	8.421	8.421	8.341	8.189	7.968
7.614	7.682	7.651	7.532	7.331	7.055	6.709
6.617	6.543	6.369	6.102	5.75	5.321	4.819
9.352	9.737	10.028	10.234	10.362	10.417	10.404
9.156	9.435	9.609	9.687	9.678	9.588	9.423
8.778	8.941	9.009	8.992	8.895	8.725	8.487
8.73	8.993	9.166	9.256	9.27	9.214	9.091
11.334	11.977	12.526	12.988	13.369	13.677	13.915
11.334	11.915	12.407	12.819	13.153	13.416	13.612
8.418	8.587	8.663	8.654	8.568	8.409	8.184
10.978	11.531	11.985	12.35	12.633	12.84	12.976
10.011	10.319	10.533	10.66	10.707	10.68	10.584
10.952	11.49	11.937	12.299	12.582	12.792	12.932
8.072	8.211	8.249	8.195	8.057	7.842	7.555
7.65	7.671	7.589	7.413	7.152	6.812	6.401
8.555	8.696	8.737	8.688	8.556	8.346	8.065
11.616	12.222	12.726	13.139	13.468	13.719	13.898
7.751	7.834	7.82	7.718	7.535	7.277	6.949
4.704	4.38	3.952	3.436	2.846	2.206	1.555
11.385	12.01	12.539	12.98	13.339	13.623	13.836
9.155	9.415	9.571	9.634	9.611	9.51	9.336
9.321	9.569	9.716	9.773	9.746	9.641	9.464
5.616	5.359	5	4.551	4.021	3.42	2.764
12.188	12.892	13.502	14.024	14.465	14.831	15.128
8.089	8.298	8.411	8.435	8.379	8.248	8.047
10.392	10.819	11.149	11.389	11.548	11.63	11.641
8.753	8.96	9.063	9.071	8.992	8.833	8.601
8.927	9.165	9.301	9.343	9.3	9.18	8.987
11.239	11.786	12.231	12.58	12.843	13.025	13.133
11.873	12.565	13.161	13.669	14.096	14.447	14.728

11.136	11.669	12.103	12.448	12.712	12.902	13.021
10.423	10.846	11.168	11.398	11.545	11.614	11.613
13.154	13.977	14.706	15.349	15.912	16.402	16.822
11.035	11.575	12.016	12.367	12.637	12.831	12.955
8.059	8.149	8.151	8.076	7.93	7.719	7.446
10.006	10.388	10.668	10.857	10.962	10.99	10.947
11.656	12.265	12.775	13.196	13.534	13.797	13.988
11.232	11.73	12.128	12.435	12.659	12.807	12.884
8.622	8.806	8.892	8.89	8.807	8.649	8.423
12.225	12.922	13.518	14.025	14.45	14.798	15.076
10.93	11.453	11.877	12.211	12.463	12.638	12.743
8.616	8.788	8.862	8.845	8.747	8.571	8.324
8.556	8.748	8.838	8.835	8.748	8.582	8.343
10.853	11.365	11.775	12.092	12.324	12.477	12.558
10.365	10.83	11.197	11.474	11.669	11.788	11.836
8.793	9.024	9.154	9.191	9.143	9.017	8.819
8.407	8.567	8.63	8.603	8.495	8.311	8.056
9.678	10.027	10.288	10.471	10.581	10.626	10.61
6.247	6.104	5.853	5.504	5.068	4.552	3.965
7.206	7.248	7.206	7.09	6.905	6.655	6.345
7.456	7.472	7.387	7.21	6.95	6.612	6.202
7.431	7.499	7.47	7.355	7.16	6.892	6.555
7.515	7.538	7.461	7.293	7.041	6.712	6.311
7.509	7.554	7.498	7.35	7.117	6.805	6.42
7.177	7.262	7.254	7.162	6.991	6.746	6.432
9.05	9.314	9.471	9.532	9.505	9.397	9.213
11.124	11.696	12.167	12.547	12.843	13.061	13.207
10.784	11.276	11.668	11.97	12.189	12.331	12.403
7.008	7.027	6.951	6.787	6.542	6.221	5.829
8.457	8.655	8.758	8.772	8.703	8.559	8.342
9.698	10.07	10.346	10.535	10.642	10.676	10.64
5.493	5.368	5.151	4.85	4.471	4.021	3.506
8.316	8.466	8.521	8.492	8.384	8.205	7.959
5.229	4.97	4.613	4.167	3.644	3.054	2.417
7.732	7.779	7.727	7.584	7.359	7.057	6.685
5.853	5.675	5.4	5.037	4.595	4.08	3.502
6.723	6.711	6.608	6.423	6.163	5.834	5.441
7.066	7.011	6.857	6.615	6.292	5.894	5.428
8.559	8.789	8.929	8.986	8.968	8.878	8.723
6.559	6.426	6.19	5.86	5.444	4.951	4.387
5.807	5.574	5.238	4.811	4.3	3.717	3.073
7.987	8.042	7.997	7.862	7.643	7.347	6.981
7.189	7.248	7.207	7.073	6.854	6.556	6.185
7.207	7.289	7.276	7.178	6.999	6.746	6.423
8.811	9.103	9.307	9.429	9.476	9.453	9.365
14.75	15.79	16.736	17.596	18.378	19.088	19.729
8.482	8.62	8.665	8.628	8.513	8.328	8.076
6.886	6.802	6.62	6.349	5.999	5.574	5.082
7.398	7.4	7.311	7.141	6.896	6.581	6.199
10.695	11.037	11.279	11.433	11.504	11.5	11.425

10.73	11.193	11.56	11.84	12.041	12.169	12.228
10.94	11.465	11.893	12.232	12.489	12.672	12.784
9.051	9.308	9.464	9.527	9.507	9.408	9.237
8.416	8.624	8.733	8.751	8.686	8.543	8.329
7.485	7.537	7.481	7.328	7.084	6.759	6.358
6.682	6.561	6.336	6.017	5.615	5.136	4.587
10.744	11.255	11.68	12.025	12.295	12.494	12.627
9.631	9.985	10.237	10.399	10.477	10.478	10.409
9.248	9.59	9.835	9.99	10.064	10.061	9.988
6.569	6.443	6.209	5.88	5.464	4.969	4.403
10.322	10.738	11.051	11.268	11.4	11.451	11.429
8.543	8.744	8.851	8.873	8.817	8.689	8.493
9.554	9.926	10.204	10.396	10.51	10.551	10.524
9.722	9.993	10.161	10.239	10.233	10.152	10.001
7.159	7.172	7.099	6.949	6.725	6.434	6.078
6.956	6.969	6.884	6.71	6.454	6.122	5.719
8.534	8.805	8.986	9.084	9.105	9.055	8.937
2.64	2.164	1.641	1.117	0.662	0.334	0.145
9.876	10.216	10.459	10.615	10.689	10.689	10.618
7.694	7.825	7.858	7.8	7.66	7.443	7.154
1.568	1.004	0.536	0.235	0.087	0.028	0.008
14.485	15.439	16.291	17.049	17.722	18.318	18.842
8.527	8.738	8.848	8.865	8.797	8.649	8.428
10.505	11.006	11.423	11.76	12.025	12.22	12.35
3.652	3.177	2.618	2.002	1.378	0.821	0.411
6.425	6.313	6.107	5.816	5.447	5.008	4.503
7.652	7.739	7.72	7.605	7.402	7.117	6.757
8.337	8.533	8.628	8.632	8.552	8.396	8.168
9.498	9.786	9.975	10.074	10.089	10.028	9.896
11.981	12.651	13.227	13.717	14.129	14.469	14.741
11.353	11.876	12.299	12.632	12.883	13.058	13.162
8.752	8.965	9.072	9.083	9.008	8.852	8.622
9.03	9.245	9.364	9.396	9.349	9.229	9.04
6.264	6.124	5.876	5.531	5.098	4.586	4.002
5.615	5.419	5.137	4.776	4.345	3.848	3.294
6.939	7.032	7.03	6.942	6.773	6.531	6.218
12.678	13.423	14.074	14.638	15.122	15.533	15.874
8.127	8.235	8.247	8.172	8.016	7.787	7.49
8.923	9.225	9.428	9.54	9.568	9.52	9.399
9.431	9.697	9.868	9.951	9.954	9.881	9.738
7.306	7.434	7.464	7.404	7.259	7.038	6.744
5.47	5.364	5.162	4.873	4.504	4.061	3.551
7.242	7.195	7.053	6.823	6.515	6.132	5.682
9.144	9.463	9.682	9.809	9.853	9.819	9.714
6.982	6.932	6.779	6.532	6.202	5.794	5.314
6.093	6.007	5.825	5.553	5.2	4.772	4.275
10.537	11.02	11.404	11.698	11.909	12.043	12.107
8.086	8.216	8.254	8.209	8.087	7.895	7.637
3.854	3.435	2.933	2.368	1.77	1.188	0.692
8.932	9.248	9.475	9.619	9.685	9.679	9.605

7.068	7.068	6.963	6.763	6.477	6.111	5.671
10.312	10.79	11.17	11.462	11.672	11.807	11.871
8.722	8.978	9.138	9.209	9.199	9.113	8.954
6.538	6.398	6.156	5.821	5.402	4.907	4.344
9.26	9.512	9.668	9.737	9.726	9.641	9.488
8.795	9.064	9.237	9.322	9.327	9.256	9.116
8.456	8.673	8.791	8.819	8.763	8.631	8.427
6.177	6.032	5.795	5.475	5.079	4.615	4.087
6.42	6.263	6.004	5.655	5.224	4.72	4.15
7.144	7.161	7.075	6.896	6.631	6.288	5.872
2.658	2.074	1.453	0.875	0.437	0.18	0.064
3.337	2.939	2.464	1.935	1.39	0.884	0.485
6.504	6.34	6.08	5.734	5.309	4.814	4.255
8.169	8.278	8.285	8.2	8.031	7.784	7.465
11.315	11.897	12.383	12.781	13.1	13.344	13.519
8.683	8.926	9.077	9.144	9.134	9.051	8.901
8.807	9.048	9.188	9.237	9.203	9.093	8.912
10.226	10.719	11.115	11.422	11.648	11.798	11.877
4.252	3.874	3.4	2.845	2.232	1.598	1.006
6.952	6.88	6.706	6.441	6.092	5.666	5.169
11.732	12.284	12.738	13.101	13.383	13.588	13.722
5.316	5.082	4.762	4.365	3.899	3.371	2.793
8.863	9.08	9.198	9.227	9.173	9.043	8.842
8.1	8.316	8.445	8.492	8.463	8.363	8.196
9.162	9.451	9.637	9.731	9.739	9.668	9.523
8.615	8.762	8.807	8.762	8.632	8.426	8.147
6.267	6.205	6.055	5.823	5.515	5.136	4.69
7.296	7.363	7.341	7.235	7.051	6.793	6.466
6.493	6.404	6.225	5.961	5.621	5.209	4.73
7.573	7.728	7.788	7.761	7.653	7.469	7.216
6.54	6.516	6.394	6.184	5.892	5.525	5.088
10.934	11.505	11.984	12.38	12.7	12.949	13.132
6.688	6.563	6.343	6.037	5.654	5.2	4.681
7.338	7.323	7.208	7.001	6.711	6.344	5.906
7.549	7.561	7.477	7.307	7.058	6.734	6.341
7.839	8.023	8.113	8.117	8.042	7.893	7.675
5.306	5.146	4.89	4.545	4.122	3.628	3.073
8.4	8.61	8.723	8.745	8.683	8.544	8.331
7.136	7.112	6.996	6.795	6.517	6.167	5.751
13.238	14.045	14.753	15.371	15.907	16.367	16.757
8.93	9.246	9.473	9.616	9.682	9.676	9.602
10.199	10.659	11.021	11.294	11.485	11.602	11.648
7.651	7.701	7.649	7.502	7.27	6.959	6.574
7.99	8.043	7.995	7.855	7.632	7.332	6.961
6.424	6.274	6.021	5.675	5.245	4.738	4.162
6.397	6.302	6.116	5.847	5.502	5.086	4.605
8.488	8.701	8.828	8.878	8.857	8.77	8.62
11.345	11.852	12.256	12.567	12.793	12.941	13.017
5.17	4.912	4.566	4.141	3.646	3.091	2.492
6.693	6.766	6.747	6.645	6.464	6.21	5.888

5.586	5.296	4.904	4.423	3.862	3.233	2.554
5.36	5.121	4.79	4.379	3.894	3.344	2.742
8.225	8.367	8.414	8.375	8.257	8.066	7.806
5.759	5.49	5.119	4.656	4.112	3.497	2.826
8.732	8.998	9.163	9.236	9.225	9.135	8.972
10.12	10.498	10.782	10.981	11.101	11.148	11.126
8.392	8.547	8.606	8.579	8.471	8.289	8.037
7.381	7.358	7.241	7.04	6.763	6.414	5.999
7.227	7.31	7.296	7.192	7.006	6.744	6.41
9.568	9.978	10.295	10.527	10.682	10.764	10.779
9.676	10.047	10.329	10.53	10.655	10.708	10.696
10.457	10.913	11.267	11.526	11.7	11.794	11.814
8.374	8.56	8.652	8.66	8.589	8.447	8.237
8.224	8.439	8.562	8.6	8.561	8.449	8.269
8.581	8.827	8.976	9.037	9.017	8.923	8.759
7.399	7.445	7.392	7.248	7.021	6.718	6.344
11.139	11.713	12.193	12.587	12.901	13.141	13.313
8.989	9.283	9.477	9.579	9.597	9.536	9.402
5.136	4.937	4.639	4.25	3.778	3.234	2.632
8.989	9.283	9.477	9.579	9.597	9.536	9.402
6.714	6.776	6.757	6.66	6.49	6.25	5.944
9.895	10.252	10.511	10.681	10.769	10.782	10.726
7.426	7.409	7.292	7.084	6.794	6.427	5.99
7.775	7.814	7.751	7.599	7.365	7.055	6.677
10.784	11.328	11.777	12.139	12.421	12.631	12.772
10.261	10.628	10.892	11.062	11.146	11.151	11.081
6.77	6.823	6.781	6.652	6.444	6.161	5.809
4.931	4.578	4.125	3.584	2.97	2.305	1.628
11.658	12.309	12.862	13.326	13.708	14.015	14.252
7.45	7.45	7.349	7.157	6.882	6.532	6.112
10.349	10.718	10.986	11.162	11.255	11.272	11.217
7.4	7.402	7.301	7.105	6.823	6.462	6.028
3.153	2.64	2.053	1.439	0.873	0.443	0.188
7.807	7.964	8.027	8.003	7.9	7.722	7.474
10.243	10.643	10.947	11.164	11.3	11.362	11.355
8.048	8.21	8.275	8.252	8.146	7.965	7.713
9.265	9.537	9.712	9.798	9.802	9.731	9.589
6.302	6.216	6.036	5.77	5.427	5.012	4.531
7.694	7.755	7.723	7.606	7.411	7.144	6.81
10.26	10.645	10.929	11.121	11.229	11.261	11.221
6.477	6.41	6.247	5.997	5.669	5.266	4.797
8.795	9.079	9.275	9.388	9.425	9.391	9.29
8.74	8.945	9.053	9.074	9.016	8.885	8.686
7.943	8.063	8.09	8.032	7.897	7.691	7.421
7.813	7.96	8.008	7.964	7.837	7.631	7.354
7.491	7.452	7.319	7.1	6.803	6.432	5.994
10.618	11.144	11.576	11.921	12.187	12.38	12.503
6.578	6.447	6.21	5.88	5.464	4.97	4.405
5.78	5.592	5.315	4.961	4.535	4.046	3.5
7.928	8.014	7.996	7.884	7.685	7.407	7.055

10.462	10.923	11.282	11.548	11.728	11.83	11.859
6.937	6.944	6.853	6.672	6.407	6.067	5.655
7.245	7.306	7.273	7.155	6.959	6.69	6.352
9.023	9.276	9.43	9.492	9.471	9.371	9.2
4.245	3.843	3.34	2.755	2.115	1.466	0.881
6.018	5.964	5.827	5.612	5.323	4.964	4.54
6.627	6.547	6.365	6.09	5.73	5.293	4.785
9.024	9.276	9.426	9.482	9.453	9.345	9.162
11.524	12.15	12.679	13.119	13.478	13.76	13.972
8.293	8.467	8.548	8.544	8.463	8.309	8.089
8.185	8.356	8.431	8.417	8.322	8.153	7.915
6.833	6.794	6.66	6.439	6.137	5.76	5.314
10.644	11.116	11.486	11.764	11.956	12.07	12.11
6.945	6.871	6.695	6.429	6.079	5.652	5.154
7.183	7.16	7.038	6.825	6.531	6.161	5.721
11.343	11.933	12.423	12.821	13.137	13.375	13.542
4.935	4.739	4.45	4.079	3.632	3.12	2.556
9.416	9.683	9.853	9.932	9.93	9.851	9.702
10.603	11.004	11.306	11.517	11.645	11.696	11.676
11.883	12.51	13.042	13.487	13.853	14.146	14.371
11.188	11.64	11.995	12.263	12.452	12.568	12.616
7.617	7.623	7.534	7.357	7.102	6.773	6.377
7.166	7.098	6.928	6.666	6.319	5.895	5.4
8.844	9.131	9.327	9.439	9.473	9.435	9.328
7.344	7.392	7.341	7.199	6.974	6.673	6.302
11.169	11.755	12.243	12.642	12.959	13.201	13.372
7.973	8.129	8.204	8.203	8.132	7.997	7.799
8.159	8.38	8.51	8.556	8.524	8.42	8.248

SP14	SP15	SP16	SP17	SP18	SP19	SP20
6.343	5.912	5.424	4.883	4.294	3.661	2.993
12.579	12.587	12.538	12.434	12.279	12.075	11.824
7.431	7.014	6.538	6.006	5.423	4.791	4.115
2.351	1.766	1.203	0.721	0.374	0.17	0.069
6.02	5.523	4.965	4.352	3.69	2.99	2.272
2.28	1.65	1.066	0.596	0.286	0.121	0.046
10.508	10.357	10.152	9.896	9.592	9.241	8.845
0.022	0.007	0.002	0	0	0	0
4.557	3.927	3.247	2.535	1.82	1.16	0.634
8.096	7.739	7.321	6.846	6.318	5.738	5.111
10.177	9.955	9.673	9.336	8.946	8.506	8.018
10.965	10.778	10.532	10.231	9.878	9.474	9.023
5.427	4.898	4.315	3.682	3.01	2.317	1.636
7.607	7.187	6.707	6.171	5.584	4.947	4.265
4.552	3.954	3.31	2.634	1.949	1.303	0.761
8.319	7.941	7.502	7.006	6.456	5.856	5.209
2.552	1.914	1.297	0.769	0.39	0.171	0.067
10.21	10.007	9.746	9.431	9.063	8.646	8.181
10.805	10.69	10.517	10.29	10.01	9.681	9.305
7.682	7.336	6.931	6.472	5.961	5.4	4.793
6.297	5.823	5.292	4.707	4.074	3.4	2.697
4.252	3.626	2.953	2.254	1.566	0.954	0.495
10.328	10.191	9.998	9.75	9.451	9.103	8.708
9.19	8.891	8.531	8.113	7.641	7.118	6.545
8.185	7.823	7.405	6.932	6.409	5.837	5.22
8.905	8.661	8.361	8.008	7.605	7.153	6.656
14.088	14.199	14.253	14.251	14.198	14.094	13.943
13.743	13.814	13.827	13.786	13.692	13.55	13.36
7.895	7.547	7.143	6.685	6.177	5.621	5.019
13.047	13.056	13.007	12.904	12.748	12.543	12.292
10.423	10.201	9.921	9.586	9.199	8.762	8.279
13.007	13.021	12.976	12.876	12.723	12.52	12.269
7.201	6.785	6.31	5.779	5.197	4.567	3.894
5.922	5.381	4.783	4.132	3.438	2.713	1.983
7.718	7.307	6.838	6.313	5.735	5.109	4.438
14.01	14.059	14.049	13.983	13.864	13.695	13.478
6.557	6.104	5.594	5.032	4.421	3.768	3.08
0.961	0.503	0.223	0.086	0.03	0.01	0.003
13.984	14.07	14.098	14.072	13.994	13.866	13.692
9.095	8.79	8.425	8.005	7.531	7.007	6.436
9.219	8.911	8.544	8.121	7.644	7.116	6.541
2.078	1.407	0.826	0.409	0.173	0.065	0.022
15.358	15.528	15.639	15.695	15.7	15.654	15.562
7.781	7.454	7.07	6.631	6.141	5.601	5.017
11.585	11.467	11.29	11.058	10.772	10.437	10.053
8.3	7.935	7.51	7.028	6.494	5.909	5.277
8.728	8.406	8.026	7.59	7.103	6.567	5.984
13.171	13.144	13.055	12.908	12.707	12.454	12.151
14.944	15.098	15.194	15.235	15.224	15.164	15.056

13.075	13.069	13.004	12.885	12.714	12.494	12.226
11.544	11.414	11.225	10.98	10.684	10.337	9.943
17.177	17.471	17.707	17.887	18.016	18.095	18.126
13.013	13.009	12.948	12.831	12.662	12.444	12.178
7.115	6.728	6.288	5.799	5.261	4.677	4.053
10.838	10.667	10.437	10.153	9.816	9.431	8.999
14.114	14.177	14.182	14.131	14.027	13.873	13.672
12.894	12.843	12.734	12.569	12.353	12.087	11.774
8.134	7.784	7.379	6.92	6.412	5.857	5.257
15.288	15.439	15.532	15.57	15.557	15.495	15.386
12.781	12.757	12.675	12.537	12.347	12.106	11.819
8.01	7.634	7.198	6.706	6.161	5.566	4.925
8.037	7.668	7.24	6.756	6.219	5.633	5.001
12.571	12.519	12.408	12.241	12.019	11.747	11.427
11.818	11.737	11.598	11.402	11.154	10.855	10.508
8.553	8.224	7.836	7.392	6.895	6.347	5.753
7.734	7.351	6.91	6.414	5.867	5.272	4.632
10.536	10.408	10.227	9.997	9.72	9.396	9.028
3.317	2.625	1.916	1.247	0.695	0.327	0.132
5.976	5.552	5.076	4.549	3.977	3.364	2.722
5.725	5.185	4.588	3.938	3.245	2.525	1.808
6.154	5.694	5.178	4.61	3.995	3.34	2.658
5.843	5.312	4.724	4.083	3.397	2.68	1.958
5.967	5.451	4.876	4.247	3.572	2.862	2.139
6.052	5.612	5.113	4.56	3.958	3.314	2.64
8.959	8.641	8.26	7.822	7.33	6.785	6.192
13.285	13.3	13.256	13.155	13.001	12.796	12.542
12.41	12.355	12.242	12.074	11.854	11.586	11.27
5.371	4.852	4.275	3.648	2.981	2.291	1.614
8.058	7.711	7.303	6.839	6.321	5.751	5.134
10.539	10.377	10.158	9.885	9.561	9.187	8.767
2.937	2.33	1.712	1.131	0.653	0.325	0.142
7.65	7.282	6.857	6.38	5.852	5.277	4.658
1.763	1.146	0.641	0.305	0.126	0.047	0.016
6.246	5.745	5.186	4.574	3.914	3.214	2.491
2.872	2.21	1.553	0.963	0.511	0.233	0.093
4.987	4.477	3.917	3.312	2.674	2.024	1.399
4.898	4.311	3.672	2.992	2.29	1.602	0.989
8.505	8.228	7.895	7.509	7.073	6.589	6.059
3.761	3.083	2.373	1.667	1.03	0.54	0.24
2.387	1.696	1.062	0.565	0.254	0.1	0.036
6.548	6.053	5.5	4.892	4.236	3.537	2.808
5.746	5.243	4.681	4.066	3.405	2.711	2.009
6.035	5.586	5.079	4.518	3.909	3.258	2.579
9.216	9.009	8.748	8.434	8.07	7.659	7.202
20.307	20.825	21.287	21.696	22.053	22.363	22.626
7.761	7.388	6.958	6.475	5.942	5.361	4.735
4.527	3.916	3.257	2.565	1.869	1.218	0.687
5.756	5.254	4.697	4.09	3.439	2.756	2.063
11.285	11.084	10.824	10.509	10.143	9.726	9.263

12.223	12.159	12.037	11.861	11.634	11.358	11.036
12.831	12.817	12.745	12.618	12.44	12.211	11.936
8.998	8.696	8.334	7.917	7.446	6.926	6.358
8.049	7.705	7.302	6.844	6.334	5.773	5.166
5.886	5.349	4.752	4.101	3.404	2.675	1.942
3.977	3.313	2.613	1.904	1.239	0.694	0.33
12.696	12.707	12.661	12.561	12.41	12.21	11.964
10.274	10.077	9.822	9.512	9.151	8.74	8.283
9.848	9.647	9.387	9.072	8.704	8.286	7.82
3.773	3.091	2.377	1.667	1.027	0.536	0.237
11.338	11.183	10.969	10.697	10.373	9.997	9.574
8.235	7.916	7.541	7.113	6.633	6.105	5.53
10.434	10.283	10.076	9.816	9.505	9.145	8.738
9.784	9.506	9.171	8.782	8.342	7.854	7.32
5.661	5.186	4.658	4.079	3.456	2.799	2.128
5.25	4.72	4.135	3.501	2.829	2.141	1.475
8.756	8.515	8.218	7.867	7.464	7.013	6.515
0.056	0.02	0.006	0.002	0.001	0	0
10.48	10.281	10.023	9.708	9.341	8.923	8.457
6.8	6.382	5.907	5.377	4.796	4.168	3.5
0.002	0.001	0	0	0	0	0
19.299	19.693	20.03	20.311	20.541	20.721	20.856
8.137	7.782	7.366	6.892	6.363	5.784	5.156
12.418	12.427	12.381	12.282	12.132	11.934	11.689
0.174	0.064	0.022	0.007	0.002	0.001	0
3.939	3.324	2.67	2.001	1.358	0.808	0.412
6.326	5.83	5.274	4.661	3.999	3.294	2.564
7.874	7.518	7.103	6.633	6.111	5.539	4.921
9.697	9.436	9.116	8.741	8.313	7.835	7.31
14.95	15.099	15.193	15.234	15.225	15.167	15.063
13.202	13.18	13.101	12.968	12.783	12.55	12.271
8.322	7.959	7.534	7.053	6.519	5.933	5.301
8.786	8.472	8.101	7.677	7.201	6.676	6.105
3.357	2.667	1.958	1.284	0.724	0.344	0.14
2.693	2.066	1.449	0.899	0.482	0.223	0.092
5.841	5.403	4.908	4.36	3.765	3.13	2.47
16.151	16.368	16.527	16.632	16.686	16.691	16.649
7.128	6.706	6.227	5.694	5.111	4.481	3.809
9.211	8.96	8.65	8.284	7.865	7.395	6.878
9.529	9.258	8.929	8.545	8.109	7.623	7.09
6.383	5.958	5.475	4.936	4.347	3.713	3.043
2.985	2.378	1.757	1.17	0.68	0.341	0.149
5.168	4.596	3.972	3.302	2.602	1.899	1.243
9.541	9.307	9.013	8.664	8.262	7.81	7.31
4.77	4.166	3.511	2.817	2.107	1.425	0.84
3.716	3.104	2.455	1.798	1.181	0.673	0.329
12.105	12.04	11.917	11.739	11.509	11.229	10.901
7.319	6.942	6.511	6.028	5.496	4.918	4.298
0.343	0.147	0.056	0.02	0.006	0.002	0.001
9.467	9.268	9.011	8.699	8.335	7.92	7.458

5.163	4.593	3.966	3.291	2.585	1.874	1.213
11.868	11.804	11.682	11.504	11.273	10.992	10.664
8.729	8.439	8.09	7.684	7.224	6.713	6.153
3.72	3.046	2.342	1.644	1.016	0.535	0.239
9.27	8.992	8.657	8.269	7.831	7.344	6.812
8.91	8.642	8.316	7.934	7.5	7.016	6.485
8.156	7.823	7.431	6.984	6.484	5.934	5.338
3.503	2.875	2.22	1.572	0.988	0.536	0.251
3.522	2.851	2.157	1.482	0.891	0.457	0.202
5.388	4.841	4.238	3.584	2.893	2.184	1.498
0.02	0.006	0.002	0	0	0	0
0.229	0.094	0.035	0.012	0.004	0.001	0
3.64	2.979	2.293	1.615	1.006	0.538	0.247
7.079	6.629	6.12	5.555	4.939	4.275	3.571
13.629	13.679	13.671	13.609	13.496	13.334	13.125
8.689	8.417	8.089	7.709	7.278	6.798	6.274
8.666	8.358	7.993	7.574	7.105	6.587	6.023
11.89	11.84	11.731	11.566	11.348	11.079	10.763
0.538	0.242	0.094	0.033	0.011	0.003	0.001
4.607	3.987	3.316	2.61	1.898	1.232	0.688
13.791	13.797	13.745	13.637	13.477	13.268	13.01
2.182	1.57	1.009	0.563	0.271	0.116	0.045
8.574	8.245	7.857	7.414	6.918	6.372	5.78
7.966	7.675	7.328	6.926	6.473	5.97	5.421
9.311	9.034	8.697	8.304	7.856	7.358	6.811
7.802	7.395	6.928	6.406	5.832	5.209	4.541
4.181	3.617	3.005	2.361	1.714	1.115	0.63
6.074	5.62	5.109	4.545	3.932	3.278	2.597
4.191	3.598	2.961	2.297	1.64	1.042	0.574
6.896	6.515	6.076	5.582	5.037	4.444	3.808
4.587	4.026	3.415	2.764	2.094	1.445	0.879
13.254	13.317	13.326	13.283	13.19	13.05	12.865
4.102	3.471	2.801	2.112	1.446	0.868	0.445
5.402	4.837	4.217	3.549	2.844	2.125	1.436
5.884	5.367	4.793	4.169	3.5	2.799	2.089
7.392	7.048	6.646	6.19	5.681	5.124	4.522
2.473	1.853	1.256	0.747	0.382	0.17	0.068
8.05	7.705	7.299	6.836	6.318	5.75	5.133
5.273	4.737	4.149	3.514	2.843	2.156	1.492
17.081	17.345	17.552	17.704	17.805	17.858	17.865
9.463	9.264	9.007	8.695	8.33	7.916	7.453
11.629	11.549	11.412	11.219	10.976	10.683	10.342
6.12	5.603	5.027	4.396	3.717	3.001	2.268
6.523	6.023	5.465	4.852	4.191	3.487	2.754
3.526	2.844	2.138	1.452	0.858	0.429	0.184
4.063	3.468	2.832	2.172	1.524	0.948	0.508
8.412	8.147	7.829	7.459	7.039	6.573	6.061
13.026	12.973	12.86	12.693	12.473	12.203	11.886
1.872	1.275	0.763	0.393	0.177	0.071	0.027
5.501	5.053	4.549	3.993	3.392	2.756	2.104

1.856	1.197	0.658	0.304	0.121	0.043	0.014
2.109	1.482	0.919	0.49	0.225	0.091	0.034
7.482	7.098	6.657	6.162	5.616	5.023	4.387
2.123	1.436	0.839	0.412	0.172	0.063	0.022
8.742	8.448	8.094	7.683	7.219	6.705	6.142
11.039	10.891	10.685	10.424	10.112	9.749	9.339
7.72	7.342	6.905	6.414	5.87	5.277	4.639
5.521	4.986	4.396	3.759	3.082	2.382	1.691
6.011	5.55	5.031	4.458	3.838	3.177	2.491
10.73	10.622	10.457	10.238	9.968	9.65	9.285
10.62	10.486	10.295	10.051	9.756	9.413	9.024
11.765	11.651	11.476	11.244	10.957	10.618	10.23
7.965	7.634	7.247	6.807	6.317	5.779	5.197
8.024	7.718	7.354	6.935	6.464	5.943	5.375
8.532	8.244	7.899	7.5	7.049	6.551	6.006
5.903	5.4	4.84	4.228	3.569	2.876	2.168
13.419	13.464	13.451	13.383	13.264	13.095	12.878
9.2	8.935	8.609	8.227	7.79	7.303	6.767
1.995	1.369	0.82	0.418	0.183	0.071	0.025
9.2	8.935	8.609	8.226	7.79	7.303	6.767
5.575	5.146	4.661	4.124	3.539	2.917	2.273
10.604	10.421	10.181	9.887	9.541	9.147	8.707
5.488	4.927	4.311	3.646	2.945	2.227	1.532
6.235	5.732	5.175	4.566	3.911	3.218	2.504
12.85	12.868	12.83	12.739	12.597	12.407	12.17
10.944	10.742	10.48	10.161	9.788	9.365	8.893
5.392	4.915	4.383	3.801	3.176	2.522	1.864
1.005	0.524	0.229	0.087	0.03	0.01	0.003
14.424	14.534	14.586	14.584	14.529	14.426	14.276
5.628	5.085	4.487	3.841	3.156	2.446	1.745
11.096	10.913	10.673	10.377	10.03	9.634	9.19
5.527	4.962	4.341	3.67	2.96	2.233	1.528
0.069	0.023	0.007	0.002	0.001	0	0
7.161	6.786	6.352	5.863	5.322	4.733	4.099
11.284	11.152	10.963	10.721	10.427	10.085	9.696
7.395	7.014	6.575	6.08	5.532	4.935	4.293
9.383	9.114	8.789	8.409	7.977	7.497	6.971
3.99	3.397	2.764	2.11	1.472	0.91	0.485
6.412	5.955	5.443	4.878	4.264	3.609	2.921
11.114	10.946	10.72	10.439	10.105	9.722	9.292
4.265	3.678	3.045	2.382	1.719	1.108	0.619
9.126	8.904	8.624	8.292	7.908	7.475	6.996
8.425	8.105	7.73	7.303	6.827	6.304	5.737
7.089	6.699	6.256	5.762	5.219	4.631	4.002
7.009	6.602	6.135	5.613	5.039	4.418	3.754
5.494	4.935	4.322	3.663	2.967	2.253	1.561
12.562	12.561	12.502	12.389	12.224	12.01	11.749
3.778	3.099	2.387	1.679	1.038	0.546	0.243
2.908	2.286	1.663	1.087	0.621	0.307	0.134
6.635	6.15	5.606	5.006	4.355	3.659	2.931

11.818	11.714	11.549	11.327	11.051	10.724	10.347
5.177	4.638	4.044	3.401	2.723	2.032	1.372
5.95	5.488	4.97	4.399	3.781	3.125	2.444
8.961	8.659	8.298	7.88	7.41	6.889	6.321
0.444	0.189	0.07	0.024	0.007	0.002	0.001
4.055	3.516	2.93	2.314	1.694	1.118	0.647
4.213	3.585	2.912	2.215	1.533	0.931	0.483
8.911	8.596	8.221	7.788	7.301	6.763	6.176
14.117	14.2	14.224	14.192	14.106	13.971	13.787
7.805	7.461	7.061	6.608	6.104	5.552	4.954
7.612	7.249	6.83	6.357	5.834	5.263	4.649
4.803	4.232	3.609	2.944	2.256	1.581	0.979
12.083	11.992	11.841	11.633	11.371	11.059	10.698
4.59	3.969	3.297	2.591	1.88	1.216	0.676
5.218	4.655	4.039	3.377	2.683	1.981	1.318
13.642	13.679	13.657	13.58	13.45	13.27	13.043
1.961	1.373	0.851	0.456	0.212	0.088	0.033
9.487	9.209	8.874	8.483	8.041	7.548	7.009
11.59	11.443	11.237	10.976	10.662	10.3	9.89
14.534	14.638	14.685	14.681	14.626	14.523	14.375
12.602	12.529	12.401	12.22	11.991	11.714	11.393
5.918	5.401	4.829	4.207	3.541	2.844	2.135
4.84	4.221	3.551	2.842	2.117	1.423	0.83
9.158	8.927	8.639	8.296	7.902	7.458	6.968
5.865	5.368	4.815	4.211	3.563	2.882	2.187
13.477	13.521	13.506	13.437	13.315	13.144	12.926
7.542	7.228	6.859	6.438	5.967	5.447	4.883
8.012	7.715	7.36	6.951	6.49	5.979	5.422

SHP2	W3D	J3D	H3D	AGDD	DDI	ADDD
0.458	2580.828	3.721	145.201	156.414	531.068	32.186
0.36	9419.607	3.934	284.125	362.293	1300.274	50.011
0.351	4337.69	2.323	205.881	211.595	811.652	39.593
0.498	739.306	2.897	64.314	73.931	195.41	19.541
0.452	1663.319	2.256	102.546	123.209	361.591	26.785
0.41	1386.29	7.047	109.936	102.688	355.71	26.349
0.278	11468.496	2.943	424.434	369.951	1713.722	55.281
0.504	283.524	1.988	36.197	40.503	101.154	14.451
0.372	2832.307	1.886	161.375	161.846	600.949	34.34
0.389	4637.737	3.904	205.136	226.231	791.253	38.598
0.273	21348.346	3.665	700.634	514.418	2886.422	69.552
0.303	12430.61	2.42	361.378	414.354	1630.265	54.342
0.351	4749.264	2.583	243.92	215.876	900.388	40.927
0.308	7998.102	3.421	313.124	301.815	1305.794	49.275
0.37	4579.735	2.656	248.039	208.17	838.401	38.109
0.403	5151.851	2.224	220.28	239.621	896.458	41.696
0.484	1350.827	2.491	101.051	103.91	330.633	25.433
0.262	9641.005	2.352	370.382	332.448	1535.524	52.949
0.405	5100.201	3.564	198.845	248.79	809.603	39.493
0.384	5030.962	2.432	242.15	228.68	915.466	41.612
0.436	4571.83	2.362	216.73	217.706	852.193	40.581
0.412	1468.368	1.82	93.699	112.951	338.538	26.041
0.428	2970.301	1.811	136.083	180.018	560.281	33.956
0.404	4640.222	2.158	203.444	226.352	814.994	39.756
0.284	10666.624	2.982	415.101	349.725	1664.686	54.58
0.332	7963.183	2.746	408.62	279.41	1538.912	53.997
0.369	9622.729	2.501	270.818	377.362	1263.84	49.562
0.268	22503.487	2.493	581.27	569.709	2822.007	71.443
0.372	5552.593	2.362	240.744	246.782	968.232	43.033
0.348	10796.837	3.807	297.145	399.883	1358.21	50.304
0.233	15841.777	2.265	447.403	465.935	2042.639	60.078
0.282	23384.539	1.894	697.493	556.775	3174.745	75.589
0.41	4683.791	2.895	218.453	223.038	829.948	39.521
0.361	6190.637	3.458	292.892	252.679	1085.174	44.293
0.293	15243.066	2.141	644.428	406.482	2583.746	68.9
0.313	12070.398	1.967	368.469	409.166	1659.678	56.26
0.338	6836.743	2.234	355.371	257.99	1319.495	49.792
0.587	1100.955	2.431	88.778	91.746	286.904	23.909
0.321	18541.524	3.06	567.834	501.122	2544.277	68.764
0.384	4923.152	1.591	210.331	234.436	901.71	42.939
0.277	8274.338	1.816	307.616	312.239	1263.462	47.678
0.396	1866.656	2.192	128.141	124.444	443.357	29.557
0.263	28492.993	2.571	833.29	626.22	3905.897	85.844
0.467	2300.05	1.961	128.562	148.39	493.989	31.87
0.288	10231.098	1.976	326.223	365.396	1402.406	50.086
0.4	3849.204	2.081	175.707	202.59	706.917	37.206
0.404	5036.851	2.125	207.02	239.85	855.967	40.76
0.34	8086.578	1.729	251.897	330.064	1176.736	48.03
0.361	6374.743	1.527	197.058	296.5	966.398	44.949

0.324	10692.993	1.924	331.342	381.893	1497.773	53.492
0.347	6676.863	1.97	214.582	296.749	1012.447	44.998
0.276	21969.945	1.665	459.546	610.276	2538.634	70.518
0.353	7585.476	1.976	230.348	322.786	1105.678	47.05
0.316	5372.034	2.44	238.179	238.757	920.651	40.918
0.377	6627.401	2.116	233.919	288.148	1030.963	44.824
0.302	10987.602	1.877	319.44	392.414	1494.303	53.368
0.29	12328.173	1.92	344.23	417.904	1626.122	55.123
0.378	4001.671	2.013	169.806	210.614	702.879	36.994
0.322	11067.533	1.676	281.454	409.909	1459.412	54.052
0.351	6718.294	1.967	217.468	298.591	1014.833	45.104
0.337	4269.743	2.2	191.201	213.487	768.32	38.416
0.396	4502.247	2.215	203.295	219.622	794.942	38.778
0.348	6312.082	1.744	227.177	280.537	987.386	43.884
0.379	5428.645	1.995	198.98	258.507	875.411	41.686
0.399	5053.976	2.265	220.988	235.069	874.413	40.67
0.3	8080.508	2.875	361.527	293.837	1317.618	47.913
0.304	7657.681	2.634	310.089	294.526	1247.894	47.996
0.436	1059.866	2.231	77.44	92.162	281.677	24.494
0.37	3361.16	3.042	190.539	176.903	676.635	35.612
0.379	3941.864	2.361	204.054	197.093	762.32	38.116
0.395	2157.931	3.282	120.111	143.862	436.166	29.078
0.38	3822.347	2.263	191.534	196.018	733.4	37.61
0.377	2534.464	2.126	143.391	153.604	549.11	33.279
0.447	2011.706	2.037	123.932	134.114	447.7	29.847
0.401	3028.644	3.11	135.416	183.554	543.714	32.952
0.35	6131.951	1.881	204.483	285.207	917.761	42.687
0.332	7155.388	2.203	257.755	298.141	1130.63	47.11
0.45	1854.654	2.006	117.126	127.907	421.494	29.069
0.394	4526.498	1.988	219.19	215.548	849.877	40.47
0.37	10916.614	3.587	395.762	363.887	1681.179	56.039
0.436	1156.262	3.605	97.794	92.501	291.184	23.295
0.372	4397.41	2.29	202.725	214.508	812.356	39.627
0.444	1052.628	2.225	79.318	91.533	263.638	22.925
0.362	4424.301	2.035	221.353	210.681	858.187	40.866
0.393	1657.382	3.904	124.373	114.302	408.205	28.152
0.408	1945.847	2.365	113.843	134.196	419.299	28.917
0.351	4254.179	2.538	225.892	202.58	837.438	39.878
0.33	7887.17	2.77	409.501	276.743	1530.819	53.713
0.388	2434.426	2.202	147.332	147.541	535.599	32.461
0.373	1863.21	2.739	127.091	124.214	418.938	27.929
0.351	5868.846	2.09	256.968	249.738	1002.199	42.647
0.448	1791.929	2.071	121.321	123.581	417.126	28.767
0.448	1464.281	2.305	95.299	112.637	342.14	26.318
0.331	9037.209	2.514	469.345	296.302	1704.955	55.9
0.24	67492.766	1.593	1131.1	1143.945	6308.934	106.931
0.307	8574.493	2.741	336.681	311.8	1382.843	50.285
0.348	3914.023	3.017	203.041	195.701	736.546	36.827
0.304	7145.351	4.006	364.149	264.643	1279.767	47.399
0.193	42440.801	2.621	1139.998	764.699	5122.271	92.293

0.293	10276.779	3.072	325.737	367.028	1374.608	49.093
0.334	9322.742	1.705	317.599	345.287	1403.701	51.989
0.383	8449.243	2.458	369.133	301.759	1488.446	53.159
0.388	3499.379	3.812	174.034	189.156	642.901	34.751
0.424	3539.066	7.732	191.752	186.267	669.456	35.235
0.367	4153.586	2.59	228.472	197.79	838.805	39.943
0.282	17923.326	1.738	550.339	484.414	2486.209	67.195
0.388	3614.335	3.102	143.954	206.533	598.652	34.209
0.399	2931.399	2.308	138.072	177.661	545.671	33.071
0.385	2319.653	2.636	138.407	144.978	476.31	29.769
0.325	8463.663	2.509	317.153	319.384	1329.629	50.175
0.372	3016.574	1.933	143.599	177.446	583.271	34.31
0.344	10893.372	2.67	469.497	345.821	1850.814	58.756
0.297	7018.795	2.874	256.993	292.45	1100.881	45.87
0.329	2492.96	2.342	159.031	146.645	546.623	32.154
0.446	1556.242	1.945	103.605	115.277	371.947	27.552
0.415	3312.83	2.098	163.969	184.046	649.566	36.087
0.549	183.163	2.561	28.465	30.527	71.647	11.941
0.296	9462.041	2.162	345.97	337.93	1456.78	52.028
0.425	2292.194	2.453	128.9	147.883	473.742	30.564
0.605	129.724	3.383	18.831	25.945	52.175	10.435
0.263	35767.504	2.163	672.984	812.898	3886.944	88.34
0.392	2568.101	2.405	131.769	160.506	513.307	32.082
0.287	23060.067	3.04	749.828	542.59	3335.315	78.478
0.401	1425.045	4.587	138.121	98.279	385.825	26.609
0.383	4093.809	2.22	232.116	194.943	859.276	40.918
0.428	2272.838	2.461	130.29	146.635	466.721	30.111
0.411	4195.291	4.009	201.431	209.765	755.937	37.797
0.318	14716.479	3.221	531.617	420.471	2137.011	61.057
0.293	18963.765	2.282	429.188	557.758	2121.59	62.4
0.28	13838.803	2.261	360.643	453.731	1700.939	55.769
0.398	6136.265	2.013	266.517	261.118	1066.393	45.378
0.299	5760.415	2.9	230.04	256.018	935.228	41.566
0.436	1289.314	2.338	90.179	103.145	329.821	26.386
0.425	3258.41	2.117	227.491	162.921	773.999	38.7
0.469	2467.826	3.863	151.129	149.565	509.726	30.892
0.271	19484.555	1.572	419.345	573.075	2171.158	63.858
0.37	4354.132	2.314	203.602	212.397	806.94	39.363
0.421	3480.04	1.982	162.215	193.336	651.478	36.193
0.268	10994.476	1.947	384.081	366.483	1613.618	53.787
0.48	1073.404	2.539	90.313	89.45	287.756	23.98
0.523	824.923	2.552	80.231	74.993	240.689	21.881
0.339	6406.67	2.783	297.699	256.267	1137.489	45.5
0.399	3133.384	2.353	142.958	184.317	574.381	33.787
0.393	2525.578	2.657	145.484	153.065	517.435	31.36
0.458	1404.49	2.396	98.946	108.038	331.113	25.47
0.361	4601.298	1.882	175.356	235.964	774.662	39.726
0.344	2472.474	2.185	145.822	149.847	528.582	32.035
0.494	1190.786	3.931	108.635	91.599	315.902	24.3
0.398	5716.279	2.054	254.235	248.534	1027.45	44.672

0.417	3632.791	4.275	199.459	186.297	688.182	35.291
0.375	5989.906	2.057	223.349	272.268	948.8	43.127
0.397	5145.877	2.395	235.747	233.904	931.779	42.354
0.39	2124.815	2.111	129.947	137.085	480.977	31.031
0.288	8259.689	2.115	310.911	311.686	1256.097	47.4
0.396	3546.419	1.948	157.691	197.023	662.508	36.806
0.389	3367.806	3.999	177.561	182.044	642.281	34.718
0.353	3404.158	3.249	205.635	174.572	709.809	36.4
0.386	3434.974	2.13	204.4	176.153	747.373	38.327
0.419	3724.158	3.156	212.613	186.208	733.747	36.687
0.504	276.43	2.047	36.199	39.49	101.802	14.543
0.583	672.749	5.689	67.623	67.275	178.037	17.804
0.309	5793.743	3.955	320.986	231.75	1112.382	44.495
0.37	5726.352	2.428	248.642	248.972	973.628	42.332
0.31	10615.389	2.213	308.467	386.014	1397.647	50.824
0.351	7962.87	2.746	407.668	279.399	1539.871	54.031
0.398	5058.486	3.849	211.07	240.88	841.863	40.089
0.392	6226.612	3.605	220.636	283.028	929.576	42.253
0.505	742.905	3.337	73.125	70.753	209.82	19.983
0.372	3034.254	1.91	168.249	168.57	637.633	35.424
0.24	22195.823	1.985	551.448	576.515	2719.576	70.638
0.417	3267.191	2.11	226.66	163.36	769.345	38.467
0.359	7726.402	1.956	330.419	291.562	1309.411	49.412
0.407	5392.075	4.469	244.385	239.648	922.255	40.989
0.38	7160.876	2.977	278.144	292.281	1140.296	46.543
0.341	7458.148	2.089	291.241	292.476	1202.844	47.17
0.469	1814.103	2.567	118.264	125.111	414.373	28.577
0.371	5835.6	1.973	322.899	233.424	1184.988	47.4
0.36	4136.518	2.18	265.203	188.024	920.35	41.834
0.45	2281.87	2.48	130.282	147.217	473.289	30.535
0.432	2876.842	3.933	166.073	164.391	564.611	32.264
0.331	10032.368	1.888	357.285	352.013	1601.62	56.197
0.349	4405.06	3.347	233.178	204.887	854.747	39.756
0.355	2557.509	2.628	142.486	155.001	516.729	31.317
0.342	5405.683	3.327	254.073	235.03	983.166	42.746
0.459	1187.528	1.974	75.496	103.263	269.582	23.442
0.523	980.504	3.314	83.701	85.261	253.978	22.085
0.39	4167.254	2.405	214.049	203.281	793.69	38.717
0.351	4064.149	2.47	214.791	198.251	799.901	39.02
0.277	29021.675	1.993	553.921	725.542	3019.007	75.475
0.398	5716.195	2.055	254.257	248.53	1027.363	44.668
0.396	5914.947	1.941	207.479	275.114	921.181	42.846
0.406	3502.844	1.93	187.08	184.36	713.948	37.576
0.35	5890.702	2.081	258.416	250.668	999.694	42.54
0.385	2088.175	2.145	132.333	134.721	473.517	30.549
0.382	4284.424	2.115	261.287	194.747	932.239	42.374
0.351	5202.967	2.374	232.332	236.499	923.032	41.956
0.25	34884.501	2.442	1016.371	690.782	4664.746	92.371
0.411	2249.481	4.656	172.532	132.322	532.351	31.315
0.459	1527.044	3.074	96.867	117.465	325.651	25.05

0.326	3837.604	3.002	261.246	178.493	837.906	38.972
0.385	1616.223	2.872	126.614	111.464	401.827	27.712
0.348	4880.847	2.364	224.891	227.016	883.427	41.09
0.351	1996.652	2.251	136.385	128.816	467.305	30.149
0.417	3971.602	2.545	179.791	209.032	705.066	37.109
0.287	13332.418	2.355	441.84	410.228	1899.526	58.447
0.333	6048.71	2.044	261.286	257.392	1054.295	44.864
0.322	4530.57	2.545	229.692	210.724	855.905	39.81
0.446	2166.094	2.593	133.5	139.748	464.646	29.977
0.404	5136.019	3.539	198.037	250.538	807.215	39.376
0.318	12646.549	4.111	482.495	383.229	1925.14	58.338
0.343	7563.457	1.852	264.542	308.713	1142.111	46.617
0.347	6508.859	2.573	345.082	250.341	1292.151	49.698
0.382	7351.441	2.674	386.294	267.325	1429.623	51.986
0.415	3911.028	3.695	182.198	205.844	687.613	36.19
0.396	3419.644	3.877	177.091	184.846	620.851	33.559
0.321	9522.348	2.686	281.039	366.244	1319.86	50.764
0.396	4532.782	2.596	194.518	226.639	779.549	38.977
0.525	1972.436	4	140.466	127.254	454.395	29.316
0.396	4532.762	2.596	194.517	226.638	779.549	38.977
0.434	1886.536	2.712	127.613	125.769	426.007	28.4
0.307	8132.693	2.049	300.322	312.796	1272.538	48.944
0.359	5683.745	2.326	289.68	236.823	1040.904	43.371
0.364	5882.564	2.249	286.446	245.107	1059.729	44.155
0.382	6219.711	3.343	203.992	289.289	915.878	42.599
0.277	15130.577	3.008	494.456	438.567	2086.677	60.483
0.465	801.5	1.978	60.748	80.15	209.443	20.944
0.354	1429.879	4.064	119.129	102.134	365.93	26.138
0.341	11029.949	1.687	344.794	387.016	1585.452	55.63
0.379	3843.838	2.23	191.139	197.12	716.385	36.738
0.258	25760.178	2.438	873.637	566.158	3713.299	81.611
0.377	5239.462	3.389	266.535	227.803	958.841	41.689
0.518	402.419	2.748	44.611	50.302	127.592	15.949
0.399	4470.41	2.149	240.472	207.926	842.215	39.173
0.3	13542.541	2.119	399.806	429.922	1860.234	59.055
0.406	3632.027	3.968	184.685	191.159	676.983	35.631
0.285	8815.222	2.986	381.179	309.306	1457.808	51.151
0.4	1786.695	2.322	107.222	127.621	381.218	27.23
0.339	3260.717	2.095	163.107	181.151	612.284	34.016
0.313	7422.86	1.884	262.672	302.974	1176.569	48.023
0.405	2253.91	2.474	128.115	145.414	461.329	29.763
0.373	4818.297	2.245	208.97	229.443	854.779	40.704
0.356	5976.99	2.975	249.082	259.869	1001.165	43.529
0.384	4515.882	2.811	204.434	220.287	804.381	39.238
0.428	2187.637	2.338	119.733	145.842	447.858	29.857
0.27	8122.785	3.897	365.607	290.099	1246.435	44.516
0.36	6839.699	2.433	221.079	303.987	984.256	43.745
0.389	2595.735	2.252	155.07	152.69	564.728	33.219
0.37	2338.191	2.867	150.242	141.709	493.697	29.921
0.385	2643.705	2.019	141.349	160.225	539.576	32.702

0.343	8977.755	1.948	306.522	338.783	1309.911	49.431
0.445	2785.319	4.064	168.792	159.161	570.622	32.607
0.391	5329.153	2.639	325.099	217.516	1141.008	46.572
0.361	8490.736	2.445	367.825	303.241	1492.407	53.3
0.57	781.879	2.323	71.625	74.465	221.199	21.067
0.42	2274.715	4.615	171.557	133.807	536.702	31.571
0.411	2061.122	2.166	134.935	132.976	471.094	30.393
0.358	5755.611	2.868	243.154	255.805	934.047	41.513
0.34	8507.126	2.516	252.781	347.23	1156.197	47.192
0.345	4739.534	2.86	217.646	225.692	812.351	38.683
0.384	3524.377	3.464	160.45	195.799	619.968	34.443
0.363	4515.516	3.495	255.145	205.251	890.252	40.466
0.346	7206.742	1.826	259.806	300.281	1121.782	46.741
0.373	2839.502	1.882	160.048	162.257	603.827	34.504
0.374	3703.866	2.954	193.477	189.942	681.367	34.942
0.338	11295.593	2.311	340.419	396.337	1550.602	54.407
0.508	1264.338	6.877	107.163	97.257	318.23	24.479
0.278	11140.471	2.843	407.793	365.261	1623.99	53.246
0.253	30533.991	2.483	958.384	629.567	4192.807	86.45
0.283	18679.052	3.446	490.913	526.17	2333.662	65.737
0.205	43789.267	1.88	1128.214	788.996	5546.075	99.929
0.344	6977.824	2.07	326.643	268.378	1299.149	49.967
0.356	3698.333	2.347	192.779	189.658	726.809	37.272
0.373	3766.023	2.133	178.815	198.212	705.845	37.15
0.424	2504.659	2.72	147.702	151.798	531.274	32.198
0.351	7683.487	1.574	266.259	313.612	1189.495	48.551
0.385	5239.027	2.35	230.085	238.138	915.114	41.596
0.437	2710.462	2.731	152.092	159.439	558.211	32.836

G1	G2	RGyr	SPAN	SPAM	MEcc	SPH
22.901	11.063	3.409	6.461	0.442	0.998	0.832
33.423	15.26	4.933	9.235	0.421	1	0.932
41.137	17.708	3.84	6.852	0.409	0.992	0.704
13.089	7.153	2.564	4.627	0.481	1	0.927
22.565	11.512	3.384	6.301	0.483	1	0.946
27.756	13.681	2.614	5.651	0.457	0.989	0.673
50.02	20.414	4.431	8.855	0.378	0.99	0.623
12.811	7.344	1.998	3.444	0.496	1	1
34.255	15.246	3.375	5.924	0.411	0.99	0.655
22.717	11.124	3.976	7.317	0.422	1	0.881
78.35	26.488	4.509	7.964	0.31	0.983	0.617
75.287	23.399	4.455	8.44	0.375	0.989	0.792
28.654	13.189	3.412	6.398	0.381	0.989	0.636
48.649	18.803	3.834	7.483	0.376	0.983	0.663
44.245	17.32	3.448	6.227	0.376	0.98	0.65
39.061	16.566	4.003	6.753	0.396	0.998	0.795
18.468	9.476	2.66	4.831	0.431	0.999	0.851
68.477	25.462	4.611	7.325	0.355	0.982	0.594
24.484	12.081	4.587	8.136	0.445	1	0.887
30.604	13.622	3.743	6.615	0.388	0.998	0.764
34.315	15.133	3.381	6.473	0.393	0.998	0.844
30.963	14.446	3.097	5.53	0.461	0.987	0.617
30.326	14.316	4.531	7.534	0.478	1	0.901
37.066	15.918	4.04	7.926	0.44	0.998	0.779
39.561	17.122	4.115	7.399	0.348	0.972	0.533
49.917	17.869	3.755	7.053	0.352	0.997	0.742
27.682	13.964	5.615	10.085	0.445	1	0.929
71.655	28.383	5.388	10.053	0.357	0.996	0.775
30.361	14.438	3.877	7.148	0.399	0.998	0.798
29.998	14.556	5.478	9.531	0.42	0.999	0.889
73.288	29.367	4.612	8.027	0.344	0.98	0.679
86.973	29.943	5.514	8.977	0.327	0.997	0.681
25.659	12.07	3.724	6.654	0.398	0.997	0.808
29.811	13.227	3.586	6.478	0.364	0.994	0.685
64.621	22.527	3.815	7.31	0.312	0.991	0.732
41.816	18.837	5.607	10.111	0.414	1	0.914
38.451	15.569	3.438	6.935	0.362	0.993	0.647
20.734	10.286	2.455	4.281	0.422	1	0.934
44.336	18.088	5.436	10.212	0.371	1	0.873
41.016	17.941	4.071	7.748	0.43	1	0.926
55.347	22.968	4.296	7.613	0.379	0.998	0.796
25.975	12.158	2.754	4.905	0.404	0.979	0.586
73.95	26.191	5.736	12.029	0.364	0.999	0.821
27.167	12.782	3.871	6.614	0.462	0.999	0.859
44.718	20.074	4.891	8.557	0.391	0.999	0.89
41.081	17.009	4.081	6.807	0.423	0.999	0.809
40.771	17.51	4.15	7.355	0.418	0.999	0.848
54.321	22.79	5.114	8.523	0.417	0.999	0.809
52.436	22.755	5.448	9.944	0.481	1	0.935

58.121	23.424	5.212	9.188	0.405	0.999	0.872
52.164	22.008	4.983	8.963	0.446	0.998	0.84
81.469	31.943	6.55	12.142	0.411	0.999	0.89
52.707	22.155	5.212	9.688	0.454	0.998	0.832
53.854	20.604	3.748	7.032	0.395	0.97	0.676
43.424	18.648	4.526	8.298	0.425	0.999	0.891
69.331	28.394	5.312	10.272	0.428	0.999	0.862
67.757	26.821	5.407	9.001	0.391	0.998	0.86
43.518	18.677	4.048	6.766	0.422	0.991	0.766
67.099	27.336	6.044	10.004	0.43	0.999	0.908
49.377	21.078	4.937	9.063	0.449	0.998	0.83
44.535	20.247	4.195	6.672	0.408	0.999	0.881
39.421	16.75	3.825	6.61	0.402	0.998	0.81
48.501	20.439	4.764	9.013	0.448	1	0.882
44.538	19.041	4.662	8.666	0.454	0.999	0.854
39.314	16.651	3.911	7.059	0.405	0.999	0.826
38.399	15.96	3.73	6.913	0.355	0.998	0.792
41.117	17.89	4.711	8.547	0.405	0.997	0.681
35.215	17.618	3.043	4.965	0.465	0.999	0.86
26.304	12.259	3.638	6.244	0.405	0.998	0.699
32.96	14.521	3.627	6.676	0.409	1	0.886
28.69	14.213	3.524	7.318	0.494	0.998	0.786
32.136	14.185	3.62	6.312	0.402	0.996	0.807
39.671	17.779	3.562	6.069	0.429	0.994	0.696
25.976	12.214	3.526	5.793	0.439	0.999	0.726
22.664	11.855	4.273	6.722	0.451	0.999	0.839
34.994	16.946	5.251	9.542	0.471	1	0.889
36.53	16.557	5.335	8.867	0.43	0.999	0.813
28.838	13.576	3.225	5.734	0.445	1	0.913
37.308	16.468	3.796	6.673	0.399	1	0.84
48.445	17.965	4.357	9.376	0.395	0.996	0.746
20.259	9.266	2.91	4.54	0.426	0.998	0.697
38.945	16.92	4.025	7.16	0.418	0.989	0.713
21.783	9.827	2.703	4.64	0.449	0.991	0.67
35.432	15.729	3.772	6.519	0.394	1	0.853
24.962	11.865	3.095	4.944	0.413	0.997	0.759
26.382	13.695	3.158	5.708	0.444	0.992	0.66
27.551	12.87	3.326	6.675	0.399	0.98	0.59
49.743	17.862	3.722	6.654	0.342	0.996	0.712
30.069	13.47	3.261	5.705	0.416	0.993	0.708
32.388	12.815	2.895	4.976	0.407	0.983	0.657
59.572	21.738	3.817	6.026	0.358	0.964	0.616
24.683	12.151	3.264	6.037	0.456	0.999	0.818
25.303	11.9	3.382	5.475	0.459	0.999	0.875
45.411	17.369	3.89	6.982	0.338	0.997	0.609
100.173	35.816	8.22	14.381	0.349	1	0.923
50.489	19.918	3.848	7.347	0.365	0.961	0.573
25.098	12.017	3.366	5.767	0.38	0.991	0.645
38.923	15.755	3.473	6.054	0.335	0.983	0.645
127.045	38.465	4.98	9.05	0.286	0.959	0.476

49.58	24.669	5.587	8.203	0.383	0.999	0.811
47.298	21.05	5.581	8.686	0.401	0.999	0.821
59.926	21.38	3.929	7.311	0.361	0.997	0.755
32.907	14.667	3.889	7.196	0.441	1	0.91
16.511	8.305	3.667	5.642	0.385	0.999	0.85
32.028	13.788	3.179	5.441	0.36	0.967	0.575
79.409	29.645	4.892	9.64	0.361	0.99	0.653
34.63	17.56	4.775	7.853	0.474	0.999	0.785
24.008	12.072	4.239	7.504	0.477	1	0.985
24.256	11.72	3.246	5.375	0.41	0.993	0.663
35.28	16.02	4.674	8.899	0.41	0.999	0.847
34.212	15.21	3.982	7.357	0.465	1	0.925
43.473	17.219	4.261	7.865	0.353	0.999	0.785
45.361	18.803	4.812	8.035	0.409	1	0.931
35.653	15.526	3.276	5.711	0.41	0.989	0.571
24.551	11.802	3.169	5.389	0.447	0.993	0.663
34.135	15.398	3.67	7.679	0.462	1	0.908
11.718	6.543	1.956	2.784	0.482	1	1
49.9	20.508	4.712	9.072	0.402	0.999	0.849
24.167	11.51	3.475	6.301	0.451	0.998	0.841
10.832	7.015	1.702	3.049	0.552	0.96	0.605
66.856	27.328	8.709	13.94	0.398	1	0.923
37.087	17.311	3.845	7.337	0.479	1	0.881
65.633	23.287	4.7	9.325	0.331	0.996	0.758
15.577	7.355	2.332	3.922	0.368	0.978	0.488
32.963	14.309	3.088	5.731	0.369	0.993	0.746
26.498	12.782	3.584	5.875	0.435	0.999	0.835
23.147	11.227	4.046	6.738	0.41	1	0.888
55.177	20.386	4.348	7.496	0.327	0.995	0.721
57.08	25.35	6.045	10.838	0.399	1	0.926
56.007	24.925	5.885	9.291	0.39	1	0.895
34.335	15.61	4.051	7.073	0.388	0.999	0.832
46.029	18.86	4.369	7.761	0.415	0.976	0.592
35.278	17.512	3.06	4.994	0.447	0.999	0.842
34.455	14.337	2.805	5.061	0.356	0.973	0.444
18.635	9.152	3.271	6.007	0.427	0.999	0.769
55.904	24.858	6.426	11.536	0.412	1	0.928
39.31	16.992	3.962	6.975	0.412	0.985	0.701
29.435	13.921	4.015	7.094	0.444	1	0.898
51.263	21.471	4.336	7.736	0.359	0.99	0.68
24.973	12.274	3.277	5.23	0.467	0.999	0.744
18.93	9.51	2.674	4.763	0.465	0.999	0.695
45.936	18.515	3.427	5.753	0.339	0.965	0.578
29.838	13.563	4.221	7.14	0.458	1	0.856
25.526	12.071	3.37	5.746	0.417	0.998	0.802
23.119	10.957	2.962	5.476	0.459	0.999	0.839
40.848	18.68	4.838	8.71	0.473	1	0.95
30.33	14.601	3.638	6.255	0.435	0.997	0.733
15.946	7.81	2.307	3.931	0.389	0.973	0.602
43.12	18.037	3.929	7.465	0.403	1	0.84

22.76	10.666	3.287	5.73	0.383	0.995	0.663
36.46	16.703	4.676	8.976	0.452	1	0.876
41.12	16.974	3.872	6.998	0.399	0.999	0.781
35.016	14.426	3.237	5.498	0.421	0.958	0.645
48.92	20.566	4.357	7.713	0.381	0.986	0.758
42.411	19.242	3.849	7.612	0.46	1	0.944
27.36	12.367	4.242	7.281	0.444	1	0.821
25.725	11.558	3.001	5.359	0.371	0.989	0.711
29.383	13.424	3.062	5.358	0.371	0.997	0.814
25.17	11.52	3.236	5.721	0.378	0.996	0.732
13.586	7.25	2.078	3.468	0.498	0.999	0.781
10.603	5.885	2.165	3.746	0.433	0.995	0.748
35.382	14.212	3.119	5.66	0.336	0.927	0.344
45.258	18.364	3.761	6.177	0.366	0.986	0.666
51.818	22.67	5.619	9.451	0.415	0.999	0.864
45.237	17.066	3.801	6.914	0.348	0.998	0.75
28.894	13.171	4.185	7.259	0.416	1	0.891
23.794	11.972	4.813	8.895	0.45	1	0.886
16.219	8.526	2.431	4.284	0.452	0.999	0.794
36.995	15.795	3.338	5.817	0.402	0.981	0.588
67.182	26.885	6.038	10.32	0.366	0.993	0.708
35.274	14.315	2.726	4.997	0.353	0.967	0.434
50.172	19.957	4.006	6.902	0.361	0.999	0.807
28.161	12.87	3.686	6.646	0.384	0.999	0.829
31.082	13.932	4.396	8.038	0.405	1	0.878
49.912	20.094	4.01	6.84	0.366	0.989	0.72
24.032	11.416	2.983	5.3	0.427	1	0.91
42.499	16.916	3.249	6.584	0.363	0.989	0.593
38.694	15.473	2.968	5.806	0.363	0.98	0.47
22.562	11.088	3.443	6.681	0.464	0.999	0.847
22.325	10.79	3.16	5.556	0.398	0.997	0.756
54.268	22.144	5.716	9.706	0.413	1	0.876
43.25	17.209	3.165	5.076	0.344	0.952	0.553
35.09	17.288	3.519	6.189	0.433	0.998	0.758
42.598	17.775	3.567	6.264	0.369	0.978	0.65
24.009	12.479	3.701	5.628	0.495	1	0.984
14.726	7.609	2.695	4.866	0.46	0.998	0.798
35.59	15.613	3.744	6.468	0.397	0.999	0.822
26.738	12.532	3.336	6.575	0.4	0.986	0.608
77.232	29.821	6.805	12.966	0.403	0.999	0.868
43.14	18.046	3.928	7.472	0.403	1	0.839
38.117	17.985	4.582	8.996	0.457	1	0.944
30.891	14.361	3.453	6.101	0.401	1	0.883
48.772	19.927	3.8	5.786	0.351	0.985	0.636
30.655	13.517	3.212	5.682	0.428	0.992	0.746
39.467	15.788	2.987	6.047	0.371	0.983	0.57
36.44	16.025	3.718	7.682	0.418	0.998	0.833
77.219	26.814	5.238	8.815	0.295	0.999	0.819
23.83	10.5	2.645	5.397	0.398	0.946	0.613
15.943	8.466	3.077	5.837	0.474	1	0.944

25.629	11.252	2.829	5.055	0.343	0.98	0.585
25.357	11.528	2.676	5.045	0.417	0.967	0.669
34.375	15.264	3.567	7.458	0.416	0.998	0.837
27.84	12.814	2.882	5.113	0.406	0.947	0.465
23.409	11.708	4.068	7.549	0.446	1	0.899
56.651	24.408	4.801	10.065	0.394	0.998	0.816
41.637	17.966	3.83	7.586	0.402	0.995	0.781
44.74	19.614	3.447	5.865	0.369	0.975	0.544
24.938	11.235	3.244	5.787	0.432	0.998	0.798
24.719	12.194	4.544	8.155	0.446	1	0.928
48.253	18.875	4.535	8.891	0.367	0.999	0.85
44.424	19.845	4.798	8.624	0.42	1	0.937
42.94	16.6	3.72	6.634	0.357	0.997	0.728
35.125	14.342	3.695	6.78	0.351	0.999	0.778
21.222	10.757	4.023	6.942	0.427	1	0.893
21.999	10.841	3.558	6.327	0.414	0.997	0.73
33.45	15.745	5.163	10.253	0.444	1	0.925
24.652	12.094	4.149	7.72	0.439	1	0.912
15.778	7.772	2.65	4.506	0.381	0.993	0.651
24.652	12.094	4.149	7.72	0.439	1	0.912
24.283	11.381	3.3	5.7	0.436	0.999	0.717
42.937	18.581	4.469	8.54	0.405	0.998	0.796
36.893	15.811	3.509	6.155	0.358	0.984	0.604
36.772	15.811	3.645	6.453	0.367	0.989	0.651
29.761	14.749	5.334	9.228	0.463	1	0.878
52.4	21.293	4.856	8.077	0.342	0.991	0.588
23.626	11.004	3.178	5.756	0.536	1	0.963
35.362	15.967	2.533	4.757	0.412	0.969	0.494
45.682	20.462	5.695	9.692	0.412	1	0.905
27.375	13.595	3.643	6.337	0.403	0.998	0.804
70.37	24.761	4.761	8.044	0.297	0.996	0.737
28.41	12.658	3.438	5.928	0.359	0.994	0.684
11.936	6.462	2.133	3.707	0.481	1	0.983
34.968	14.725	3.466	6.785	0.397	0.997	0.706
59.185	25.437	4.775	8.664	0.371	0.997	0.849
27.548	14.184	3.835	7.01	0.429	0.999	0.774
44.465	18.122	4.217	8.134	0.378	0.992	0.577
28.404	14.705	3.034	5.759	0.454	0.995	0.775
36.667	17.736	3.5	6.353	0.42	0.98	0.609
47.248	20.991	4.554	8.421	0.415	0.999	0.878
30.379	15.292	3.1	5.671	0.428	0.99	0.736
38.86	17.693	4.192	7.553	0.424	0.999	0.831
37.655	18.007	4.133	7.257	0.397	1	0.897
31.328	15.879	3.739	6.669	0.403	0.999	0.886
25.806	12.216	3.575	6.249	0.456	0.999	0.839
50.304	21.214	3.63	6.047	0.329	0.955	0.376
28.574	14.321	4.995	9.537	0.46	1	0.943
32.909	14.169	3.253	5.808	0.413	0.985	0.644
33.712	15.713	2.896	5.111	0.394	0.972	0.542
40.978	18.651	3.497	6.848	0.456	0.998	0.834

44.414	19.573	4.834	8.508	0.401	1	0.927
20.721	9.973	3.366	5.897	0.41	0.997	0.767
31.876	13.207	3.33	5.554	0.337	0.997	0.65
59.065	21.38	3.969	7.174	0.358	0.998	0.776
18.945	9.636	2.326	4.127	0.443	1	0.942
24.576	10.509	2.893	6.062	0.422	0.988	0.622
25.665	11.834	3.271	5.398	0.417	0.994	0.691
32.456	14.898	4.215	7.807	0.417	1	0.864
33.591	16.306	5.812	9.661	0.444	1	0.926
35.419	16.849	3.679	6.728	0.4	0.996	0.77
28.498	14.458	3.665	7.892	0.468	0.998	0.836
27.092	12.036	3.241	6.348	0.38	0.994	0.712
39.135	18.099	5.239	8.58	0.423	1	0.897
39.387	16.151	3.305	5.768	0.406	0.97	0.56
31.325	14.439	3.473	6.047	0.394	0.993	0.632
42.026	19.234	5.715	9.318	0.404	1	0.885
11.204	5.838	2.527	4.705	0.425	0.999	0.813
53.373	21.103	4.288	7.785	0.357	0.984	0.666
74.308	26.076	4.941	8.543	0.297	0.994	0.698
49.247	21.302	6.001	10.802	0.39	0.999	0.847
123.523	38.722	5.139	9.053	0.286	0.991	0.653
42.439	17.484	3.465	6.793	0.361	0.994	0.796
35.209	15.154	3.3	5.97	0.391	0.965	0.52
33.136	15.586	3.968	6.915	0.427	0.996	0.668
28.847	13.295	3.388	5.939	0.424	0.996	0.698
44.8	19.909	5.438	9.079	0.43	0.999	0.883
33.68	15.294	3.644	6.633	0.388	0.999	0.869
29.228	13.317	3.861	6.916	0.451	0.998	0.717

ASP	FDI	PJI3	L/Bw	SEig	HOMA	RCI
0.398	0.982	0.821	3.42	67.877	0.086	1.471
0.585	0.967	0.952	5.66	106.299	0.724	1.404
0.255	0.971	0.644	2.44	86.759	0.82	1.403
0.63	0.982	0.878	6.67	40.091	0.996	1.389
0.595	0.998	0.94	5.59	54.696	0.756	1.39
0.231	0.988	0.736	2.37	56.367	0	0
0.439	0.898	0.901	6.61	129.5	0.994	1.389
0.311	1	0.792	1.8	28.972	0.711	0
0.166	0.987	0.74	1.38	75.679	0.765	1.403
0.591	0.949	0.842	6.38	82.001	0.994	1.388
0.206	0.846	0.806	2.39	173.819	0	0
0.227	0.912	0.728	2.35	127.675	0.554	1.477
0.225	0.939	0.889	2.27	91.375	0.654	1.403
0.173	0.935	0.707	1.91	111.962	0.824	1.384
0.186	0.878	0.686	2.25	91.713	0.994	1.387
0.512	0.976	0.947	5.42	89.002	0.991	1.382
0.383	0.985	0.819	3.05	53.377	0.892	1.389
0.299	0.919	0.878	4.06	124.436	0.964	1.385
0.794	0.969	0.896	18.9	82.642	0.994	1.387
0.557	0.952	0.781	6.78	90.522	0.991	1.382
0.27	0.973	0.817	1.94	86.685	0.967	1.401
0.276	1	0.83	3.19	55.889	0.787	1.484
0.825	1	0.85	23.22	70.156	0.727	1.404
0.594	0.976	0.826	8.73	85.159	0.991	1.382
0.204	0.9	0.751	2.86	128.779	0.992	1.389
0.517	0.956	0.907	6.51	121.311	-0.209	0
0.814	0.978	0.901	19.19	103.666	0.994	1.387
0.362	0.909	0.871	3.4	173.588	0.588	1.397
0.352	0.963	0.794	2.94	92.75	0.714	1.472
0.715	0.937	0.814	12.27	108.735	0.994	1.387
0.19	0.889	0.826	2.29	146.777	0.898	1.398
0.601	0.909	0.856	9.47	181.536	0.524	1.458
0.535	0.949	0.721	6.87	84.326	0.547	1.468
0.273	0.912	0.72	2.55	100.654	0.994	1.389
0.165	0.926	0.774	1.17	163.869	0.982	1.402
0.813	0.959	0.993	22.34	122.141	0.993	1.386
0.279	0.945	0.899	2.74	111.729	0.99	1.39
0.281	1	0.699	1.57	48.322	0.889	1.389
0.752	0.937	0.958	13.7	156.526	-0.113	0
0.54	1	0.761	4.92	88.908	0.753	1.419
0.36	0.908	0.838	3.11	115.109	0.747	1.404
0.176	0.99	0.712	2.14	62.762	-0.703	1.476
0.705	0.95	0.946	12.36	198.561	0.085	0
0.685	1	0.849	10.17	65.706	0.827	1.474
0.5	0.902	0.876	4.49	118.867	0.873	1.475
0.535	0.986	0.861	5.41	79.821	0.645	1.382
0.472	0.977	0.817	4.28	88.2	0.624	1.387
0.669	0.986	0.882	11.14	104.626	0.654	1.406
0.813	1	0.898	19.76	93.812	0.57	1.765

0.676	0.963	0.982	12.23	120.098	0.177	1.762
0.487	1	0.815	5.28	97.938	0.188	1.762
0.684	0.985	0.904	10.36	155.203	0.488	1.406
0.669	1	0.883	14.1	102.025	0.153	1.762
0.117	0.918	0.791	1.49	97.394	0.23	1.759
0.538	0.979	0.852	5.43	96.985	0.581	1.387
0.671	0.957	0.945	11	120.336	0.653	1.398
0.482	0.942	0.836	4.83	127.167	0.446	1.762
0.313	0.981	0.845	3.43	82.146	0.152	1.762
0.749	1	0.908	15.08	117.494	0.176	1.762
0.661	1	0.931	12.79	96.814	0.111	1.407
0.291	0.967	0.724	1.96	87.199	0.787	1.401
0.446	0.951	0.895	4.16	86.165	0.621	1.382
0.707	0.981	0.917	10.7	96.355	0.577	1.759
0.617	0.998	0.803	7.84	89.862	0.234	1.759
0.492	0.951	0.983	4.68	89.72	-0.107	0
0.37	0.879	0.944	3.23	113.899	0.446	1.759
0.42	0.928	0.877	4.2	108.73	0.992	1.384
0.39	1	0.801	3.26	50.157	0.991	1.391
0.343	0.945	0.839	2.91	77.841	0.991	1.387
0.236	0.963	0.8	1.25	84.726	0.995	1.392
0.382	0.977	0.7	3.21	61.687	0.992	1.384
0.204	0.973	0.859	1.51	83.419	0.691	1.398
0.427	1	0.834	5.26	71.315	0.823	1.397
0.571	1	0.756	7.01	62.827	0.99	1.39
0.716	1	0.777	12.68	68.563	0.868	1.369
0.799	0.997	0.863	20.94	89.703	0.991	1.397
0.757	0.987	0.983	19.42	99.764	0.92	1.418
0.389	1	0.943	2.7	60.97	0.447	1.41
0.428	0.97	0.853	3.38	88.325	0.473	1.407
0.474	0.941	0.895	5.77	126.183	0	0
0.539	0.941	0.856	6.58	51.456	0.994	1.387
0.272	0.974	0.64	3.01	87.862	0.648	1.475
0.187	1	0.687	1.66	48.514	0.995	1.389
0.288	0.979	0.795	1.88	88.233	0.831	1.466
0.205	0.98	0.64	1.3	61.327	0	0
0.337	1	0.617	3.69	60.052	0.994	1.389
0.137	0.956	0.745	1.44	85.977	0.979	1.4
0.469	0.951	0.952	5.43	121.031	-0.209	0
0.171	0.989	0.797	1.11	70.516	0.704	1.403
0.137	0.937	0.731	1.24	64.445	0.994	1.39
0.18	0.915	0.724	2.76	101.366	0.449	1.478
0.604	0.998	0.736	7.36	59.788	0.758	1.392
0.541	1	0.815	5.42	54.393	0.678	1.402
0.59	0.924	0.924	9.67	130.026	0.08	0
0.875	0.91	0.98	34.29	254.817	0.08	0
0.118	0.923	0.844	1.78	115.715	0.995	1.393
0.186	0.927	0.737	1.66	80.851	0.991	1.382
0.193	0.887	0.926	2.19	113.242	0.99	1.387
0.138	0.84	0.704	2.18	245.568	0.817	1.396

0.511	0.884	0.612	4.83	117.408	0.995	1.389
0.683	0.968	0.838	10.46	113.636	0.993	1.397
0.401	0.957	0.846	3.8	119.028	0.77	1.401
0.586	0.949	0.901	5.88	76.741	0.616	1.403
0.611	0.934	0.813	7.71	74.74	0	0
0.192	0.959	0.828	2.88	87.669	0.829	1.445
0.371	0.916	0.986	4.71	158.797	0.974	1.404
0.694	0.988	0.877	11.63	74.633	0.667	1.762
0.782	1	0.963	11.78	69.018	0.683	1.4
0.316	0.936	0.68	3.17	65.717	0.994	1.388
0.659	0.952	0.92	8.9	110.021	0.992	1.389
0.419	1	0.853	3.15	75.084	0.658	1.762
0.658	0.941	0.921	10.07	132.087	-0.003	0
0.231	0.961	0.664	1.11	100.587	0.698	1.401
0.243	0.951	0.897	2.51	71.68	0.833	1.404
0.463	1	0.787	6.4	57.348	0.693	1.408
0.541	1	0.908	5.08	75.258	0.684	1.405
0.358	0.998	0.435	2.23	23.956	0.694	0
0.363	0.937	0.881	2.7	119.259	0.995	1.391
0.61	0.991	0.949	8.57	64.229	0.808	1.396
0.143	1	0.556	2.24	21.109	-0.056	0
0.81	1	0.95	17.36	192.751	0.969	1.389
0.531	1	0.9	4.85	68.527	0.585	1.414
0.454	0.929	0.932	5.07	184.864	-0.008	0
0.169	0.925	0.686	2.06	57.797	0	0
0.174	0.98	0.784	1.2	86.695	0.881	1.403
0.586	0.978	0.947	6.44	62.472	0.756	1.39
0.653	0.953	0.844	7.48	80.466	0.994	1.388
0.437	0.879	0.938	5.12	145.106	0	0
0.601	0.923	0.857	6.09	141.676	0.681	1.382
0.5	0.921	0.71	4.34	127.299	0.823	1.401
0.614	0.972	0.939	7.57	97.788	0.62	1.469
0.182	0.93	0.735	2.33	94.344	0.993	1.388
0.394	1	0.7	3.32	54.135	0.99	1.394
0.135	0.974	0.805	1.72	84.142	0.986	1.399
0.659	0.943	0.861	11.11	66.381	0.991	1.382
0.648	0.944	0.861	7.43	143.664	0.813	1.398
0.228	0.967	0.576	2.57	87.672	0.723	1.467
0.731	1	0.94	12.91	75.905	0.824	1.397
0.295	0.904	0.764	3.23	127.304	0.685	1.406
0.716	1	0.834	12.53	48.7	0	0
0.587	1	0.855	6.71	44.382	0	0
0.103	0.917	0.862	1.31	105.516	0.825	1.393
0.695	0.999	0.837	10.45	71.551	0.573	1.4
0.35	0.958	0.903	2.99	69.047	0.69	1.397
0.468	0.987	0.879	4.19	53.6	0.851	0
0.732	1	0.906	11.62	83.884	0.916	1.405
0.254	0.976	0.633	1.96	70.922	0.913	1.404
0.147	0.943	0.673	1.9	51.746	0	0
0.524	0.977	0.896	4.82	95.968	0.478	1.406

0.474	0.913	0.911	6.22	79.098	0.994	1.389
0.737	0.985	0.923	13.22	90.563	0.996	1.391
0.519	0.969	0.816	5.22	91.593	0.475	1.407
0.094	1	0.732	1.06	66.567	0.704	1.403
0.262	0.902	0.884	3.02	113.284	0.765	1.404
0.551	1	0.837	5.09	78.34	0.729	1.762
0.441	0.945	0.756	3.54	77.005	0.994	1.389
0.175	0.94	0.692	1.59	80.261	0.991	1.382
0.197	0.988	0.683	1.12	81.275	0.967	1.401
0.487	0.926	0.966	5.88	81.63	0.994	1.389
0.256	1	0.6	1.63	29.198	0.908	0
0.356	0.899	0.713	3.6	37.924	0.97	0
0.084	0.898	0.882	1.82	104.561	0.991	1.382
0.304	0.927	0.872	3.77	95.975	0.709	1.403
0.641	0.928	0.925	8.51	115.034	0.775	1.464
0.488	0.956	0.939	5.28	121.442	-0.098	0
0.535	0.961	0.925	4.83	86.511	0.706	1.404
0.807	0.966	0.936	22.37	88.257	0.994	1.387
0.23	0.96	0.632	1.34	42.309	0.869	1.369
0.139	0.989	0.746	1.42	77.777	0.746	1.404
0.368	0.927	0.851	4.1	167.617	0.79	1.418
0.138	0.968	0.742	1.96	84.078	0.983	1.398
0.419	0.94	0.827	3.65	113.696	0.475	1.406
0.468	0.917	0.884	4.47	91.575	0.996	1.387
0.647	0.957	0.89	8.18	99.423	0.987	1.394
0.263	0.931	0.917	2.83	108.152	0.655	1.462
0.363	0.991	0.831	2.47	59.733	0.473	1.406
0.26	0.954	0.773	2.76	106.601	0.963	1.399
0.189	0.957	0.69	2.26	92.935	0.92	1.395
0.676	0.99	0.864	11.95	63.931	0.884	1.418
0.493	0.928	0.839	5.9	69.281	0.994	1.387
0.813	0.993	0.988	18.64	122.762	0.677	1.463
0.092	0.931	0.835	1.47	90.652	0.822	1.383
0.268	0.955	0.765	1.97	70.358	0.994	1.393
0.126	0.935	0.713	1.09	96.762	0.823	1.383
0.677	1	0.878	7.21	48.313	0.388	1.477
0.509	0.968	0.845	5.66	45.302	0.995	1.392
0.485	0.953	0.926	4.23	84.521	0.47	1.407
0.187	0.959	0.885	1.96	85.107	0.654	1.403
0.757	0.948	0.954	18.39	171.843	0.729	1.406
0.523	0.977	0.894	4.81	95.95	0.469	1.406
0.755	1	0.858	13.63	91.84	0.915	1.405
0.429	0.994	0.734	3.29	79.765	0.813	1.465
0.22	0.913	0.657	2.5	99.679	0.449	1.478
0.165	0.991	0.754	1.07	66.528	0.701	1.403
0.164	0.969	0.838	1.78	92.945	0.985	1.399
0.332	0.959	0.799	2.62	92.867	0.995	1.389
0.607	0.921	0.966	8	221.727	0	0
0.106	0.928	0.838	1.9	68.638	0	0
0.697	0.969	0.775	8.61	52.515	0.996	1.389

0.133	0.914	0.702	1.3	89.551	0.991	1.382
0.19	0.962	0.672	2.84	60.087	0.991	1.382
0.302	0.961	0.737	2.34	90.713	0.994	1.389
0.081	0.978	0.644	1.24	63.876	0.991	1.382
0.732	0.984	0.962	11.35	77.266	0.747	1.465
0.295	0.909	0.895	2.2	138.296	0.995	1.392
0.349	0.96	0.77	3.43	98.011	0.843	1.402
0.119	0.933	0.895	1.16	91.764	0.823	1.398
0.478	0.973	0.86	4.99	64.596	0.995	1.39
0.764	0.967	0.878	12.38	82.694	0.996	1.388
0.408	0.895	0.908	3.25	139.002	-0.514	0
0.682	0.956	0.781	8.26	102.919	0.71	1.416
0.481	0.963	0.824	5.37	111.039	-0.264	0
0.582	0.954	0.89	6.89	115.723	0	0
0.718	0.959	0.931	10.91	76.89	0.692	1.398
0.488	0.914	0.752	5.51	74.77	0.685	1.399
0.603	0.981	0.799	6.08	107.406	0.837	1.404
0.721	0.981	0.884	10.46	81.462	0.886	1.417
0.368	0.953	0.997	4.07	61.315	0.994	1.387
0.721	0.981	0.884	10.46	81.461	0.885	1.417
0.522	0.954	0.767	5.33	62.159	0.806	1.408
0.504	0.946	0.958	5.45	109.891	0.859	1.4
0.214	0.911	0.875	2.45	100.328	0.91	1.417
0.298	0.926	0.772	3.34	100.796	0.908	1.417
0.846	0.998	0.937	30.74	90.065	0.392	1.414
0.462	0.886	0.909	7.14	144.119	0.825	1.402
0.688	1	0.82	9.32	43.744	0.825	1.762
0.121	0.945	0.716	1.59	60.477	0.99	1.387
0.832	0.982	0.98	22.12	119.456	0.657	1.465
0.305	0.949	0.704	2.34	79.891	0.737	1.465
0.492	0.906	0.959	6.46	197.335	0	0
0.343	0.914	0.948	3.46	94.67	0.994	1.389
0.331	1	0.831	2	32.087	0.699	1.402
0.556	0.919	0.879	7.46	90.447	0.991	1.382
0.418	0.944	0.777	4.18	135.371	0.741	1.408
0.593	0.947	0.631	7.54	76.775	0.994	1.389
0.419	0.905	0.812	5.36	121.415	0.744	1.759
0.242	0.979	0.572	2.09	58.444	0.844	1.421
0.285	0.95	0.641	3.98	76.734	0.892	1.401
0.518	0.986	0.954	4.88	105.91	0.957	1.389
0.239	0.967	0.528	2.39	64.238	0.875	1.409
0.471	0.975	0.847	4.45	91.968	0.55	1.411
0.431	0.953	0.907	3.4	96.096	0.696	1.401
0.379	0.964	0.882	2.95	84.599	0.738	1.465
0.607	1	0.843	8.06	63.709	0.606	1.758
0.111	0.804	0.701	1.81	117.893	0.994	1.392
0.728	0.977	0.961	10.3	92.41	0.766	1.411
0.143	0.983	0.797	1.14	72.844	0.705	1.403
0.154	0.914	0.641	2.05	69.282	0.944	1.405
0.404	1	0.791	3.51	71.608	0.757	1.752

0.677	0.939	0.775	8.29	110.777	0.934	1.417
0.5	0.94	0.801	6.09	68.959	0.997	1.388
0.513	0.958	0.91	6.39	102.904	0.196	0
0.398	0.959	0.846	3.58	120.019	0.77	1.401
0.267	1	0.677	1.41	42.37	0.89	1.389
0.367	0.936	0.781	4.96	69.235	0	0
0.382	0.987	0.84	4.21	65.684	0.77	1.758
0.537	0.931	0.806	4.99	91.263	0.955	1.696
0.818	0.968	0.921	19.42	100.674	0.82	1.397
0.453	0.926	0.795	5.33	86.283	0.991	1.382
0.399	0.964	0.758	3.47	72.929	0.991	1.382
0.268	0.928	0.809	2.49	89.728	0.994	1.388
0.643	0.979	0.894	7.72	100.621	0.892	1.389
0.115	0.991	0.751	1.41	76.272	0.765	1.403
0.291	0.904	0.553	2.88	79.473	0.993	1.397
0.789	0.96	0.947	18.28	118.279	0.939	1.394
0.38	0.949	0.995	3.01	50.907	0	0
0.356	0.881	0.7	5.25	129.32	0.685	1.406
0.441	0.899	0.895	5.62	209.718	0	0
0.665	0.933	0.941	10.38	149.284	0.995	1.392
0.371	0.906	0.911	4.44	247.924	0.867	1.458
0.184	0.967	0.851	1.35	111.59	0.6	1.447
0.14	0.961	0.725	2.06	81.235	0.82	1.403
0.584	0.984	0.885	9.83	79.781	0.782	1.408
0.403	0.981	0.792	4.19	67.168	0.693	0
0.751	0.996	0.88	17.4	105.558	0.829	1.765
0.279	0.953	0.927	1.97	92.025	0.78	1.41
0.585	0.973	0.861	8.43	71.661	0.674	1.397

AROM	HOMT	DISPm	QXXm	QYYm	QZZm	DISPv
0.894	0.605	15.429	64.102	192.587	236.054	4.153
0.982	5.795	7.722	111.957	584.177	675.468	7.641
0.986	13.12	8.15	169.334	306.288	401.506	5.234
0.993	5.974	4.697	12.123	73.345	82.879	4.344
0.939	6.808	10.147	38.27	190.909	225.254	10.82
0	0	13.438	57.441	116.151	142.722	3.874
0.993	5.961	4.971	155.313	603.368	608.974	3.342
0	5.687	5.822	18.416	28.224	46.64	5.549
0.929	16.071	5.995	143.809	182.382	264.971	3.844
0.994	5.962	9.548	53.098	289.11	324.713	14.813
0	0	2.257	375.634	732.919	875.712	1.634
0.94	10.531	11.322	415.373	871.496	889.118	9.539
0.984	9.813	6.991	101.243	196.746	247.681	10.346
0.998	9.884	9.609	210.771	352.981	453.948	9.546
0.995	5.961	6.719	145.471	265.653	319.034	3.134
1	5.944	2.312	90.615	397.198	445.6	5.746
0.993	8.027	7.705	31	86.85	109.425	7.307
0.998	14.463	14.961	297.281	811.106	846.811	5.219
0.995	5.965	15.227	35.546	457.019	467.171	12.445
1	5.944	7.147	58.458	299.671	324.617	9.126
0.985	7.737	5.414	105.125	193.697	277.605	9.163
0.903	11.801	6.678	79.48	173.515	200.792	5.73
0.951	12.361	16.755	33.41	515.11	525.495	16.633
1	5.944	6.029	71.031	416.022	427.788	7.531
0.994	11.906	9.27	193.8	410.371	443.254	12.909
0	-1.046	7.008	88.949	406.789	433.454	8.996
0.995	5.961	5.782	53.189	770.607	796.442	9.502
0.967	15.298	3.656	392.603	1111.449	1327.57	0.652
0.95	12.137	10.511	108.225	284.017	357.918	15.324
0.995	5.961	5.041	84.952	757.328	790.082	4.771
0.94	22.44	7.703	385.142	726.427	863.002	5.792
0.936	6.284	24.145	274.621	1585.761	1621.895	14.199
0.926	5.472	8.377	56.24	269.732	288.167	10.624
0.993	5.961	8.32	105.672	230.919	289.812	9.523
0.983	5.893	7.898	286.158	327.504	537.088	7.125
0.938	5.96	8.074	67.622	973.105	991.613	5.955
0.99	5.939	5.688	109.033	242.656	298.136	4.737
0.938	6.223	1.519	38.363	59.612	97.176	1.922
0	-0.566	10.407	93.508	966.207	1012.329	7.417
0.935	12.795	5.309	90.762	401.728	473.511	8.301
0.924	23.171	1.988	191.455	519.583	643.558	4.891
0.835	-10.541	2.426	60.565	109.711	129.461	3.24
0	0.255	9.903	169.501	1464.745	1518.02	2.007
0.953	12.4	15.103	45.936	321.479	344.901	16.949
0.959	18.331	4.287	165.102	667.478	790.51	10.018
1	5.801	21.392	100.964	459.871	524.52	7.303
0.996	5.613	3.666	113.769	426.712	504.724	1.748
0.915	9.158	8.761	125.881	938.73	973.975	0.919
0.613	7.41	0.763	78.207	1068.998	1099.625	2.432

0.592	2.128	18.155	136.503	1063.634	1089.203	11.588
0.592	2.639	10.203	194.757	805.381	896.367	4.16
0.914	8.301	17.939	274.339	2151.071	2277.875	10.077
0.592	1.839	9.631	129.22	975.596	978.187	8.916
0.621	1.839	3.783	242.881	323.353	437.017	5.06
0.995	6.386	3.65	124.962	562.55	644.39	1.893
0.947	11.095	7.495	154.539	1159.767	1203.548	2.233
0.676	8.026	12.389	282.396	1127.835	1287.91	7.099
0.592	1.826	2.151	167.602	406.174	473.45	2.636
0.592	2.996	2.32	158.399	1580.012	1637.425	1.84
0.95	1.337	6.091	108.58	805.711	815.433	6.896
0.959	14.957	10.896	258.149	332.502	562.879	4.046
1	5.589	7.787	100.268	347.133	409.678	3.111
0.687	8.079	7.869	88.034	724.181	773.348	3.886
0.621	1.873	5.411	108.802	640.624	695.732	11.006
0	-0.322	10.558	94.48	376.677	438.533	1.928
0.621	2.228	4.181	111.395	316.778	384.098	3.749
0.998	11.899	9.495	185.929	588.443	683.702	15.591
0.99	5.945	5.352	69.711	171.655	219.943	3.24
0.95	11.89	19.611	110.226	236.282	311.224	15.901
0.992	11.944	12.842	171.028	211.457	370.387	14.772
0.998	5.955	26.822	93.352	249.279	307.039	22
0.984	10.361	18.343	161.626	226.775	344.546	17.317
0.988	11.521	12.259	93.583	314.476	331.186	4.136
0.95	11.886	15.496	51.172	247.416	268.796	13.118
0.955	4.342	3.982	42.592	354.758	371.693	4.252
0.988	11.889	9.091	60.709	743.134	760.731	21.399
0.964	15.647	12.196	78.458	829.706	831.409	5.891
0.979	5.367	11.533	68.205	169.205	232.136	7.26
0.973	5.677	13.224	103.796	321.677	406.27	8.356
0	0	14.535	149.173	604.357	647.024	4.57
0.995	5.966	16.592	30.792	144.614	155.792	13.856
0.948	12.308	12.108	175.954	373.246	436.639	10.339
0.994	5.968	7.777	61.524	94.434	132.994	10.468
0.948	10.803	14.234	182.633	236.815	402.85	14.408
0	0	12.733	103.428	123.445	208.71	6.966
0.993	11.923	9.516	60.001	170.193	192.205	5.153
0.983	11.748	6.352	117.671	157.424	222.4	9.535
0	-1.046	5.296	99.253	389.042	421.808	7.2
0.983	10.567	7.372	129.98	145.961	236.365	5.766
0.993	11.928	6.054	106.498	125.47	186.387	4.957
0.866	6.292	11.25	228.849	453.07	487.19	12.203
0.938	6.824	14.205	36.052	195.842	215.117	15.163
0.986	10.168	4.781	43.788	203.222	231.803	3.934
0	0.241	10.72	74.953	440.166	448.91	5.808
0	0.241	0.668	186.27	4184.063	4237.323	10.489
0.993	11.934	7.923	237.615	358.579	439.624	9.442
1	11.889	7.07	103.902	159.647	225.414	11.271
0.95	11.882	7.67	137.314	253.375	311.583	9.5
0.989	11.431	3.896	718.566	1225.815	1399.229	2.725

0.993	11.935	9.593	228.342	965.069	1122.99	2.918
0.99	11.911	11.761	137.714	1022.412	1085.061	7.234
0.982	11.553	6.834	146.394	464.439	545.387	7.178
0.979	6.155	21.863	68.584	355.903	408.702	10.686
0	0	3.562	34.533	204.004	220.197	0.678
0.946	8.289	6.491	94.254	192.447	203.635	6.484
0.98	11.689	4.334	308.953	1000.077	1045.969	8.176
0.585	4.67	3.537	77.495	588.159	619.342	12.208
0.979	10.244	5.842	30.247	353.001	382.715	8.674
0.994	11.927	4.758	66.112	165.467	195.73	8.143
0.994	11.906	0.612	77.967	551.844	594.547	3.885
0.743	12.499	7.883	123.427	327.762	435.676	4.232
0	-0.008	8.232	73.133	521.564	548.165	7.225
0.984	5.58	23.325	406.852	449.752	831.91	6.981
0.925	13.332	2.875	97.33	202.967	248.363	1.593
0.983	11.095	6.064	45.278	178.244	182.278	0.91
0.944	10.938	12.962	64.134	294.35	342.925	13.009
0	2.083	4.16	13.679	28.779	42.458	1.87
0.992	11.939	22.415	252.712	625.996	833.526	18.916
0.988	10.505	4.402	37.247	221.8	234.542	8.181
0	-0.111	3.509	13.585	23.832	24.272	3.255
0.981	5.815	36.73	270.647	3800.882	3964.193	5.606
0.947	7.601	10.922	83.705	352.152	414.242	3.367
0	-0.023	3.615	210.104	806.393	887.953	2.537
0	0	7.946	35.62	54.716	70.461	1.646
0.956	5.284	6.652	113.772	131.56	217.243	2.592
0.939	6.808	10.152	47.594	247.154	277.597	12.142
0.994	5.962	2.03	46.617	300.303	336.756	7.104
0	0	3.3	162.134	601.796	653.895	0.415
1	6.132	11.231	241.138	1290.955	1483.69	5.528
0.987	11.517	18.409	305.801	1186.783	1426.123	14.395
0.901	7.445	10.265	63.662	385.567	420.229	11.001
0.996	11.92	11.448	274.424	507.083	576.273	13.021
0.99	5.943	9.484	69.044	182.012	229.469	4.248
0.985	5.919	10.258	88.255	132.092	168.933	5.268
1	5.944	11.184	25.215	178.009	182.67	3.846
0.941	21.961	13.523	233.615	1522.086	1697.684	15.197
0.95	13.74	9.483	188.254	346.363	419.569	8.63
0.987	11.529	4.207	37.782	352.575	367.352	7.482
0.924	13.012	10.932	214.646	537.559	609.841	13.292
0	0	0.765	22.885	195.277	204.155	0.62
0	0	8.208	20.717	103.485	115.877	2.215
0.991	9.897	3.172	186.305	228.288	318.347	2.913
0.979	10.319	5.308	53.317	408.593	435.99	9.703
0.984	10.356	4.468	71.548	182.593	229.64	9.077
0	4.254	6.227	37.625	138.373	164.194	4.778
0.938	15.566	10.19	75.684	669.05	716.006	8.48
0.939	15.521	1.88	116.939	213.288	299.163	3.693
0	0	7.06	34.867	54.576	67.734	3.996
0.982	5.734	13.3	95.664	403.157	475.432	8.441

0.993	5.961	7.762	44.904	187.3	197.138	10.098
0.993	5.979	6.089	58.783	574.871	600.029	3.186
0.975	5.699	16.45	89.952	385.64	441.585	11.25
0.983	10.561	6.36	162.492	163.678	240.127	4.998
0.922	18.352	12.286	252.589	510.235	599.642	9.957
0.705	10.93	4.439	81.369	362.405	426.764	6.045
0.993	5.961	7.825	110.101	363.236	451.014	5.924
1	5.944	4.337	82.863	119.278	171.133	7.51
0.951	11.6	3.914	104.118	113.085	200.941	7.726
0.993	5.961	5.628	44.122	187.62	202.359	6.614
0	5.449	8.534	23.827	37.237	57.66	6.043
0	2.909	5.291	14.162	41.014	47.364	2.247
1	11.889	6.458	122.855	180.815	202.764	8.668
0.982	5.673	7.305	137.173	370.373	399.903	4.875
0.912	14.726	4.69	161.443	1075.558	1157.907	8.553
0	-0.489	2.774	95.552	381.253	426.059	5.563
0.98	5.65	16.986	94.238	384.293	457.654	11.718
0.995	5.961	7.754	35.826	498.667	505.215	6.019
0.955	4.345	9.904	43.961	49.15	88.257	9.41
0.95	15.664	6.086	148.998	195.445	266.405	3.712
0.954	20.529	13.896	496.641	1488.958	1643.286	15.943
0.984	5.901	8.827	80.745	127.038	155.122	3.351
0.975	5.701	11.588	144.581	432.561	531.208	6.112
0.994	5.974	8.428	69.882	264.338	307.226	6.117
0.988	5.923	13.728	67.881	456.627	497.322	9.337
0.943	8.513	8.186	188.504	428.347	504.457	6.898
0.928	5.671	13.179	55.737	129.412	180.571	6.978
0.977	5.778	7.431	104.564	229.802	274.394	5.356
0.969	5.523	9.527	91.631	171.483	206.365	6.024
0.956	9.721	4.509	28.387	211.73	217.138	1.909
0.995	5.962	4.783	37.736	162.615	175.882	5.215
0.925	12.181	34.424	103.174	1394.081	1440.977	12.641
0.999	9.858	5.634	147.887	189.449	246.233	2.894
0.993	11.931	7.835	123.942	216.821	306.045	4.475
0.999	9.881	7.626	206.349	220.206	349.406	3.082
0.897	2.718	7.745	35.371	241.4	276.387	1.982
0.993	5.969	8.988	20.549	90.622	100.209	7.751
0.974	5.642	15.185	83.66	318.958	381.683	9.503
0.984	9.816	6.118	100.542	170.995	222.319	9.633
0.978	13.125	5.74	209.639	2246.469	2278.642	9.246
0.974	5.632	13.73	95.746	402.836	475.165	8.477
0.937	15.551	5.779	53.827	565.678	593.133	5.411
0.927	13.826	5.926	69.687	215.9	275.339	5.831
0.867	6.291	4.736	179.482	361.218	428.416	7.774
0.983	10.519	7.3	129.107	143.48	228.279	4.885
0.984	5.909	8.648	102.9	162.355	213.457	4.196
0.993	11.935	9.436	118.069	280.079	366.58	10.056
0	0	1.991	197.546	1168.271	1253.222	1.14
0	0	8.416	68.418	93.966	113.792	4.155
0.993	5.974	3.794	16.531	126.957	140.598	2.825

1	5.944	4.383	80.667	99.099	147.057	5.539
1	5.944	7.17	56.98	106.769	118.688	3.891
0.993	11.923	7.337	109.204	236.84	317.73	11.739
1	11.889	7.281	93.986	111.358	149.665	0.601
0.938	7.473	16.185	34.858	328.819	350.602	18.304
0.992	11.938	20.76	323.724	668.96	913.521	19.227
0.947	11.809	11.952	125.442	351.196	410.969	13.387
0.986	11.518	12.203	196.988	218.068	332.108	4.849
0.994	5.968	10.11	48.214	192.692	210.675	5.26
0.994	5.974	12.747	40.301	433.798	463.433	11.803
0	-1.543	2.434	187.417	561.065	706.624	5.148
0.932	12.773	3.171	87.524	657.364	726.837	4.686
0	-1.322	4.287	86.571	352.826	389.971	6.108
0	0	1.45	58.008	316.008	345.854	2.167
0.981	5.538	15.325	33.877	301.718	321.441	13.825
0.975	5.481	13.621	52.54	221.066	241.722	12.155
0.984	11.721	10.144	118.966	661.073	757.051	16.978
0.957	9.749	14.547	39.271	351.698	378.069	21.785
0.995	5.961	0.454	27.707	85.593	95.155	2.486
0.957	9.733	12.488	39.273	351.688	378.06	17.465
0.935	10.475	11.025	44.185	197.659	224.431	9.018
0.984	12.891	12.807	129.083	555.285	619.03	21.308
0.934	10.005	7.898	126.147	249.749	299.274	6.869
0.933	9.988	8.312	113.017	287.652	326.751	7.696
0.941	1.958	14.987	43.981	738.494	743.18	2.186
0.986	11.55	10.755	200.571	801.782	805.46	7.235
0.688	8.249	8.312	23.596	183.422	198.761	4.705
0.935	5.938	11.557	78.049	105.948	134.104	3.287
0.915	9.861	15.203	66.909	1086.917	1113.13	10.303
0.917	11.061	9.577	104.542	215.65	295.022	11.317
0	0	2.441	200.223	877.57	924.444	1.504
0.993	5.961	6.679	77.605	217	252.678	7.382
0.982	5.588	6.298	18.361	36.143	54.172	3.309
1	5.944	2.723	52.051	269.916	285.817	6.683
0.944	11.863	8.922	225.798	740.635	855.407	5.995
0.993	5.961	24.394	58.186	323.847	350.493	12.201
0.703	8.189	6.066	139.238	496.813	521.399	6.763
0.884	9.287	11.474	74.472	143.071	186.304	5.118
0.959	16.061	7.582	103.126	267.239	279.353	3.801
0.994	18.187	5.379	136.834	584.927	680.812	0.989
0.886	9.63	11.017	80.213	167.492	197.866	4.374
0.979	10.443	6.915	114.471	434.787	504.879	4.437
0.983	5.566	21.616	134.359	396.152	502.325	10.236
0.936	7.383	10.359	100.415	264.964	341.405	7.766
0.727	8.491	7.585	42.245	252.013	268.158	11.377
0.994	11.931	11.549	231.837	304.875	387.692	3.877
0.975	8.424	15.36	65.75	591.712	641.77	16.024
0.983	10.568	7.124	139.306	157.972	240.137	5.732
0.927	9.443	7.626	90.323	143.246	177.614	9.821
0.714	11.358	12.64	91.179	282.668	341.836	8.723

0.926	10.275	4.881	92.408	675.246	745.407	5.046
0.995	5.979	13.316	44.571	194.927	209.525	9.239
0	0.588	5.16	52.245	233.502	250.838	3.953
0.982	11.553	6.438	152.022	468.616	560.911	7.513
0.938	6.231	3.071	34.903	47.061	81.431	3.758
0	0	17.492	57.213	158.792	164.407	10.077
0.705	8.47	8.202	65.948	197.199	216.57	5.095
0.652	2.864	18.563	98.049	413.609	489.253	11.906
0.987	11.482	8.762	62.478	927.587	959.226	1.73
1	5.944	12.648	82.912	311.537	337.145	10.096
1	5.944	16.129	87.591	254.158	311.104	11.183
0.994	5.962	5.033	79.114	171.039	215.113	4.039
0.993	9.812	7.71	124.998	751.56	836.29	5.479
0.929	16.073	3.552	158.725	201.15	266.562	3.211
0.939	11.92	1.546	93.413	222.473	267.709	3.563
0.992	8.453	7.331	79.13	1006.245	1033.348	2.218
0	0	7.76	21.542	59.864	75.923	0.95
0.924	13.014	10.596	184.233	587.714	592.292	13.682
0	0	4.379	257.798	979.326	1037.288	2.699
0.993	11.935	5.403	169.433	1251.659	1311.926	1.738
0.954	13.876	3.61	451.195	1430.816	1550.489	4.969
0.94	8.403	7.156	163.002	212.105	332.874	8.027
0.986	13.12	5.612	120.589	204.861	237.633	4.27
0.938	13.295	17.817	69.438	384.682	387.999	19.687
0	2.078	4.228	67.19	209.178	239.13	4.943
0.762	12.435	4.48	96.017	964.435	977.601	3.762
0.954	13.266	6.131	123.026	231.414	332.162	7.639
0.972	9.435	10.834	62.957	334.148	340.898	12.506

QXXv	QYYv	QZZv	DISPe	QXXe	QYYe	QZZe
58.393	186.214	223.339	0.675	129.321	380.27	456.026
131.929	739.974	842.32	0.178	264.597	1415.173	1605.036
136.944	296.204	368.149	0.372	272.263	497.063	630.131
15.191	74.715	86.618	0.194	33.396	149.514	174.377
32.532	166.527	194.506	0.32	65.756	325.334	377.73
38.129	81.504	99.159	0.504	97.685	183.24	224.396
194.581	644.768	656.445	0.185	368.816	1196.207	1220.373
13.416	26.341	39.758	0.22	27.216	50.345	77.561
109.469	203.01	253.309	0.15	175.015	333.268	404.489
73.274	374.82	423.371	0.074	144.247	672.265	753.444
367.971	686.296	834.524	0.081	768.554	1427.808	1697.4
285.687	817.39	939.848	0.151	467.402	1424.283	1642.696
136.27	254.356	315.447	0.021	243.067	454.424	547.874
239.304	364.883	478.002	0.107	467.486	684.952	921.322
123.888	243.344	294.825	0.269	247.488	432.895	534.89
84.309	425.887	459.067	0.235	173.857	738.896	797.345
33.088	113.507	135.192	0.172	67.966	198.751	241.551
214.311	542.319	585.186	0.782	432.679	1028.418	1109.677
40.763	514.144	523.256	0.345	92.509	967.109	982.71
72.976	373.105	393.35	0.14	157.541	639.083	682.316
129.391	251.328	349.535	0.089	259.415	455.313	644.368
55.308	151.228	162.391	0.416	111.148	248.484	280.617
37.443	396.789	414.468	0.168	78.073	624.715	657.182
64.994	441.774	446.573	0.365	132.473	745.873	756.183
260.252	523.904	573.348	0.017	468.373	895.189	996.526
106.87	382.609	428.746	0.178	232.706	700.187	784.36
70.353	959.488	993.128	0.128	144.247	1786.471	1840.91
385.891	1199.231	1396.979	0.209	751.614	2004.631	2370.059
141.645	359.915	449.991	0.028	247.89	629.648	767.623
113.72	926.595	973.873	0.117	230.727	1796.794	1881.961
380.333	820.155	981.178	0.363	691.608	1457.635	1719.669
323.447	1224.883	1299.612	0.019	625.992	1878.693	2027.18
75.495	347.984	379.701	0.11	153.653	626.052	683.487
155.845	301.912	385.011	0.077	312.415	532.222	684.526
324.481	424.345	643.961	0.207	612.645	802.35	1184.657
78.725	1080.294	1109.982	0.173	166.81	1799.457	1853.748
129.093	311.823	359.786	0.137	257.557	580.239	664.287
43.105	65.275	105.539	0.119	88.854	132.196	212.098
129.192	1085.379	1133.789	0.217	278.796	2040.545	2137.642
94.64	414.687	491.773	0.514	185.703	681.533	824.541
206.232	530.985	656.773	0.143	389.644	823.181	1061.154
66.584	132.449	154.228	0.198	128.579	221.015	263.598
205.108	1408.374	1462.383	0.415	433.533	2635.802	2737.607
40.681	254.651	275.687	0.187	81.135	416.546	452.395
194.281	752.076	891.353	0.099	373.984	1162.483	1429.389
86.853	364.062	411.163	0.631	171.943	578.388	662.832
91.301	427.117	478.387	0.267	185.548	754.091	850.035
107.296	839.314	858.603	0.456	217.467	1390.561	1417.372
53.488	858.847	879.876	0.553	105.454	1395.522	1437.391

124.31	940.726	984.935	0.421	255.353	1581.053	1679.598
148.164	648.994	725.272	0.445	297.943	1099.72	1252.175
245.533	1882.993	2003.255	0.37	471.541	3228.996	3436.061
112.208	794.268	818.557	0.234	235.217	1371.982	1428.931
204.271	271.787	386.645	0.393	337.998	540.762	703.316
102.581	586.911	649.234	0.233	210.04	1027.033	1149.684
117.215	1057.594	1086.958	0.32	235.88	1738.628	1796.49
241.546	974.267	1120.993	0.511	456.801	1579.536	1851.575
123.187	339.064	396.934	0.295	224.388	596.526	697.398
137.665	1192.32	1261.032	0.364	283.081	1968.93	2116.283
89.77	705.151	715.626	0.375	184.969	1270.945	1293.374
175.912	237.822	388.906	0.59	351.377	447.507	740.889
92.617	352.01	400.223	0.507	180.323	617.703	705.67
70.105	713.628	739.329	0.247	136.222	1081.546	1125.955
85.644	584.559	617.272	0.445	165.22	962.059	1024.601
91.423	384.675	433.407	0.489	185.982	682.42	776.704
121.568	423.021	485.132	0.068	229.815	780.023	879.676
183.691	498.612	578.388	0.41	319.831	863.29	961.666
36.6	105.039	130.973	0.366	70.595	196.956	241.945
122.169	203.3	278.658	0.224	212.529	338.163	452.745
150.069	225.462	356.323	0.18	234.438	380.104	570.76
56.981	223.963	251.077	0.568	107.756	374.791	417.169
141.81	245.953	347.126	0.165	224.963	413.817	561.671
70.672	224.677	244.978	0.372	134.484	381.974	419.96
50.496	198.475	215.362	0.128	98.923	319.562	351.115
46.373	360.312	372.905	0.118	91.375	628.616	648.818
59.613	755.839	770.391	0.231	113.274	1242.39	1259.041
87.049	717.144	729.091	0.826	174.078	1180.662	1197.556
62.989	133.195	187.909	0.489	124.552	260.531	365.24
99.828	294.332	362.318	0.493	194.399	556.825	680.952
146.357	635.005	668.949	0.515	306.606	1206.986	1277.672
30.204	105.171	116.217	0.23	66.639	173.155	195.994
152.718	301.154	376.825	0.193	292.536	485.49	637.336
35.904	109.237	121.566	0.146	60.974	192.525	203.464
158.021	253.546	383.874	0.171	250.024	428.983	618.11
62.015	103.661	148.203	0.207	110.501	191.198	257.043
49.271	189.667	190.145	0.268	102.076	342.138	345.449
153.906	206.722	283.45	0.007	265.305	388.487	497.82
119.724	360.939	412.547	0.129	259.986	655.35	755.689
94.856	168.081	222.262	0.17	157.404	284.834	365.229
73.567	132.316	168.268	0.271	136.042	214.113	278.624
198.47	350.766	424.773	0.237	343.732	584.426	709.096
34.963	165.132	182.663	0.39	73.271	299.802	331.661
38.592	144.276	170.7	0.355	82.919	268.184	323.762
110.995	418.508	427.557	0.31	237.82	768.052	785.783
245.19	4110.494	4183.063	0.33	521.696	7449.154	7585.469
272.83	393.692	502.386	0.184	509.885	689.307	900.175
138.04	211.177	287.722	0.018	240.462	388.307	497.245
165.585	307.644	386.855	0.145	305.755	548.703	669.657
779.569	1298.068	1491.217	0.169	1372.737	2317.064	2591.085

225.527	704.92	838.097	0.267	444.398	1231.392	1490.358
134.981	882.061	934.404	0.224	249.145	1313.684	1389.865
132.523	464.87	525.216	0.332	290.87	823.188	948.319
59.623	287.158	330.264	0.929	128.822	550.394	641.695
46.462	242.583	267.919	0.145	111.914	496.352	549.302
107.948	220.644	255.157	0.348	220.905	415.135	470.229
273.132	940.888	973.991	0.131	529.543	1748.264	1803.338
67.523	419.75	436.7	0.354	131.904	771.142	788.91
33.187	340.369	371.662	0.611	69.41	592.136	655.578
72.213	176.784	207.235	0.242	140.404	309.301	359.52
107.118	673.576	723.968	0.098	211.695	1070.391	1160.002
92.92	296.665	375.337	0.458	161.429	452.983	584.431
107.147	578.307	611.499	0.167	225.918	1052.806	1112.765
198.618	415.631	594.771	1.169	460.566	800.541	1205.95
86.836	169.478	205.527	0.162	160.836	298.04	346.959
38.431	168.773	169.359	0.402	76.645	282.28	283.402
70.455	271.787	326.119	0.343	139.728	497.271	599.571
11.088	13.777	24.865	0.244	24.579	31.386	55.964
201.863	589.108	733.307	0.545	380.712	1003.924	1257.91
35.776	232.06	245.482	0.287	78.809	407.674	436.124
6.58	14.575	15.57	0.117	17.309	37.646	40.828
275.39	2755.846	2909.11	1.719	536.271	5296.134	5563.097
53.335	266.861	301.779	0.48	106.958	466.454	530.521
237.558	980.408	1059.614	0.09	501.337	1828.923	1980.878
41.057	62.201	75.372	0.349	95.503	134.466	164.361
138.765	155.314	253.893	0.257	270.251	315.913	496.283
43.28	208.525	232.075	0.317	91.007	403.057	444.703
63.671	326.646	370.136	0.3	138.304	592.049	680.192
191.591	653.387	719.302	0.165	414.641	1289.047	1414.133
228.266	1492.52	1656.506	0.287	416.68	2677.646	2946.933
286.381	1073.071	1288.943	0.34	516.226	1751.021	2116.799
88.872	464.861	507.261	0.155	184.34	779.223	863.425
186.546	419.98	469.615	0.473	321.787	762.296	816.342
38.594	115.809	141.49	0.43	76.386	228.49	273.362
104.295	133.935	169.653	0.377	197.866	266.855	323.578
34.811	194.679	200.348	0.411	77.716	388.214	400.938
271.34	1700.704	1894.206	0.207	467.748	2788.485	3106.887
165.634	276.194	364.096	0.155	315.886	446.156	618.575
41.692	368.5	389.53	0.348	89.08	625.351	665.971
254.952	622.59	720.205	0.127	480.611	1012.039	1201.839
19.757	100.249	105.438	0.065	46.475	224.809	234.392
18.558	66.064	72.124	0.425	43.572	150.5	162.406
191.789	265.252	344.731	0.18	366.987	519.498	669.894
44.122	379.26	394.832	0.447	87.734	621.284	645.411
73.508	191.844	238.675	0.205	140.298	333.298	414.949
32.705	124.832	145.002	0.116	68.977	237.317	275.511
57.77	531.061	572.873	0.807	118.038	923.582	1005.896
110.717	200.518	273.139	0.076	180.785	286.41	394.008
36.007	66.923	80.283	0.258	79.61	138.956	166.388
96.155	383.23	440.622	0.483	198.046	719.017	830.166

69.764	246.844	262.435	0.048	149.147	442.338	472.57
50.218	584.075	592.492	0.251	110.991	1097.483	1114.931
90.704	359.444	399.043	0.594	191.176	674.075	754.862
100.517	155.696	203.094	0.366	169.682	255.873	333.908
213.648	519.898	619.016	0.288	395.255	834.27	1038.666
69.437	358.388	416.017	0.301	136.422	582.218	690.372
72.613	269.776	313.188	0.196	130.897	500.456	564.934
110.138	154.043	223.815	0.087	217.428	286.913	415.027
129.152	149.077	252.243	0.085	220.947	276.223	439.908
66.562	241.158	266.429	0.059	143.287	420.902	471.267
21.305	24.695	41.644	0.074	29.617	45.905	65.767
18.225	48.851	57.608	0.169	42.915	106.661	126.354
165.337	238.527	267.968	0.068	310.096	435.768	477.363
137.079	361.876	401.463	0.35	278.18	651.384	739.51
147.385	920.888	987.087	0.339	302.08	1642.198	1781.123
119.914	359.496	421.152	0.208	251.364	670.897	782.763
80.718	418.069	470.639	0.385	158.941	768.163	863.634
47.027	603.512	611.006	0.18	103.776	1167.139	1178.489
35.793	40.677	68.832	0.45	78.107	85.742	144.516
116.233	215.033	260.028	0.078	189.083	355.983	411.66
470.354	1472.796	1627.144	0.212	824.295	2175.104	2468.607
100.103	135.343	165.283	0.358	190.33	273.771	319.794
141.068	440.004	521.204	0.446	284.114	796.935	954.85
103.611	309.931	375.842	0.216	215.882	608.453	736.143
78.253	536.221	579.377	0.377	169.091	1004.437	1081.688
209.55	420.522	528.566	0.397	380.849	744.396	927.747
63.413	115.606	171.147	0.533	125.717	226.686	333.084
124.768	268.289	309.077	0.243	244.157	491.365	563.241
113.268	186.586	220.477	0.304	217.036	337.537	392.004
33.805	210.679	223.842	0.276	72.007	414.235	437.794
52.941	203.809	222.674	0.221	109.13	408.282	439.597
118.32	884.196	948.546	0.401	234.587	1263.305	1380.591
155.886	188.811	255.303	0.327	291.857	381.405	497.106
85.868	191.699	243.124	0.414	169.898	336.989	433.644
187.127	229.264	332.779	0.325	360.423	457.202	649.904
23.809	161.572	184.339	0.469	51.779	296.368	344.968
22.365	77.641	88.793	0.263	51.768	168.21	192.792
81.589	280.445	329.573	0.554	167.864	526.156	621.965
134.431	220.477	281.426	0.02	237.169	401.147	492.163
214.181	2375.014	2400.606	0.333	440.752	4272.875	4316.125
96.203	383.208	440.42	0.506	198.18	719.039	829.838
59.109	619.69	656.431	0.121	117.822	1090.977	1163.654
89.232	242.519	313.964	0.167	167.171	402.073	527.706
183.601	336.647	417.906	0.173	337.331	579.617	715.239
88.165	146.544	203.354	0.372	150.76	245.308	332.509
115.156	183.231	228.436	0.321	226.375	363.956	445.915
130.413	327.978	417.309	0.237	229.399	603.555	746.234
257.763	1408.19	1504.326	0.063	530.587	2611.059	2788.968
58.155	110.937	132.251	0.288	126.216	226.227	266.803
20.915	155.629	171.972	0.126	45.155	308.467	340.8

119.59	135.025	201.101	0.019	236.51	243.473	364.164
51.027	104.328	126.569	0.389	106.701	188.459	231.036
124.515	313.232	399.204	0.106	214.273	555.736	690.715
83.287	131.787	158.465	0.407	160.105	221.508	265.088
46.667	389.091	416.21	0.212	97.485	675.75	723.328
325.403	647.768	879.964	0.196	573.935	1125.997	1506.802
145.571	388.714	470.017	0.266	257.759	677.362	804.395
173.855	213.572	296.906	0.54	338.979	386.721	539.127
39.32	193.86	207.363	0.324	77.466	358.411	379.876
45.91	510.833	538.189	0.252	100.434	952.446	1004.6
242.793	513.39	701.808	0.26	474.828	985.666	1325.031
104.234	645.629	726.752	0.038	218.753	1094.777	1261.363
101.869	330.263	374.247	0.236	213.398	609.701	689.638
90.249	347.414	390.884	0.041	193.094	662.532	741.011
46.095	359.436	383.977	0.274	101.023	651.556	701.565
66.342	265.181	284.272	0.267	141.072	485.648	524.917
151.56	837.368	954.057	0.042	262.836	1446.712	1626.126
53.89	439.596	474.459	0.087	109.059	742.726	802.768
39.258	128.98	136.904	0.089	86.133	259.149	273.848
53.892	439.583	474.449	0.095	109.063	742.704	802.753
56.985	145.887	175.432	0.157	117.777	239.101	298.809
161.539	569.601	653.502	0.207	285.415	981.327	1108.811
145.042	294.887	347.088	0.188	277.279	516.424	608.552
132.184	341.784	383.837	0.179	250.986	601.904	675.028
56.761	680.027	687.607	0.658	122.255	1292.721	1308.96
250.016	843.705	855.791	0.437	473.236	1497.19	1498.765
16.332	101.082	115.275	0.786	31.965	215.572	239.945
52.343	78.645	96.496	0.57	120.387	152.653	193.723
79.51	1036.133	1065.722	0.47	167.249	1700.678	1757.383
116.487	242.982	323.324	0.196	208.495	439.235	570.129
248.163	1007.793	1070.148	0.075	502.608	1862.406	1984.388
116.936	281.438	334.911	0.075	240.905	492.53	593.816
16.879	31.762	48.214	0.439	38.009	65.678	102.552
62.448	324.535	330.857	0.112	134.589	549.73	564.937
235.878	808.219	945.554	0.259	454.891	1483.597	1747.695
66.111	276.94	302.697	0.598	137.362	529.944	576.864
177.967	510.497	541.704	0.105	340.278	822.493	876.397
53.684	157.764	185.168	0.364	112.026	286.915	341.601
90.073	287.46	297.915	0.273	169.946	476.808	488.423
145.042	645.457	742.168	0.155	252.291	984.502	1141.192
61.902	185.843	210.467	0.365	128.553	338.108	387.285
99.933	374.079	430.931	0.384	194.731	669.347	772.616
118.611	393.125	484.132	0.577	250.156	719.422	904.763
108.475	286.952	371.047	0.338	204.007	564.743	711.356
37.792	243.208	258.13	0.246	77.478	418.155	443.723
274.278	284.46	367.676	0.345	508.23	550.562	703.222
75.477	691.337	743.933	0.357	151.842	1242.251	1343.507
99.927	173.725	223.882	0.243	171.517	298.624	369.659
74.225	144.025	162.618	0.291	154.534	275.358	316.477
70.863	253.094	296.66	0.361	129.088	401.419	473.652

111.306	677.822	759.678	0.121	234.792	1228.47	1395.6
45.228	206.675	220.423	0.386	106.271	391.632	425.892
77.858	255.144	276.553	0.106	169.831	478.179	516.439
135.6	462.042	531.281	0.389	297.249	823.023	967.653
33.735	49.925	81.766	0.1	71.537	97.076	162.645
54.761	137.014	153.426	0.295	113.059	247.035	274.429
63.796	178.845	203.914	0.156	117.196	265.381	310.588
77.944	453.104	497.799	0.424	145.299	841.835	903.406
82.374	948.531	995.352	0.368	174.468	1545.953	1637.978
71.275	384.097	400.284	0.324	149.533	678.975	706.408
68.734	317.971	353.879	0.472	143.137	575.874	643.046
109.175	206.554	267.97	0.165	226.754	401.999	518.664
104.908	735.943	800.36	0.179	185.661	1112.766	1211.766
113.333	213.069	248.465	0.124	177.071	348.858	391.731
114.987	210.456	261.639	0.084	242.924	391.896	500.798
98.319	1010.75	1045.419	0.378	209.815	1727.946	1797.721
21.399	88.613	100.316	0.289	54.597	183.451	210.387
212.309	652.622	699.655	0.222	410.11	1089.692	1184.496
333.315	1132.058	1216.591	0.122	660.689	2100.251	2257
201.552	1311.445	1384.248	0.199	410.692	2293.335	2445.174
552.248	1566.191	1711.002	0.252	1034.682	2740.169	3000.774
211.02	259.3	418.036	0.178	374.706	499.398	761.602
134.196	232.918	282.254	0.216	257.453	386.456	460.386
82.689	370.766	374.575	0.313	151.689	583.756	593.051
58.95	191.988	212.149	0.216	131.618	367.822	410.044
78.204	866.908	884.173	0.335	140.01	1288.863	1320.993
150.795	301.661	422.463	0.108	281.229	528.763	741.526
64.494	244.199	263.026	0.198	127.441	404.064	440.324

DISPp	QXXp	QYYp	QZZp	G(N..N)	G(N..O)	G(N..S)
0.069	63.084	200.411	239.614	0	0	0
0.469	145.194	813.703	924.513	10.103	43.918	0
0.46	142.139	309.879	382.647	0	27.602	0
0.289	17.049	80.682	93.938	0	5.529	0
0.605	33.897	173.63	202.198	21.627	75.078	0
0.534	44.065	92.291	114.278	0	43.301	0
0.247	213.527	689.056	702.399	55.412	106.527	0
0.346	13.609	27.453	41.063	12.201	12.88	0
0.346	119.508	217.187	270.763	10.818	0	0
1.004	81.768	410.21	463.199	0	7.709	0
0.088	396.265	733.843	891.102	77.924	420.972	0
0.371	336.149	913.379	1038.341	0	22.161	0
0.708	149.723	277.91	342.984	0	0	0
0.741	261.919	398.566	523.368	4.827	55.936	0
0.308	133.307	260.896	315.356	13.657	70.74	9.113
0.318	92.949	460.436	495.61	13.782	55.142	11.118
0.45	36.098	124.025	147.211	0	3.404	0
0.133	218.61	537.241	584.356	11.091	48.243	0
0.705	45.483	555.022	564.458	12.064	35.972	0
0.62	81.692	404.064	425.427	0	16.02	0
0.617	142.601	275.607	382.524	6.418	24.773	0
0.259	57.074	154.736	163.468	85.108	63.856	20.11
1.374	41.305	429.025	449.021	0	12.948	0
0.535	71.731	472.507	479.209	3.12	25.868	2.905
0.852	286.137	569.356	625.273	3.733	7.09	0
0.579	119.324	403.814	455.788	0	0	0
0.657	78.643	1050.37	1086.784	0	19.908	0
0.106	413.361	1272.842	1480.98	34.044	156.023	0
1.041	154.735	391.306	487.513	8.517	0	0
0.299	127.057	1017.946	1070.306	0	31.21	0
0.469	409.434	887.37	1058.012	27.766	153.755	19.101
1.147	359.903	1317.753	1402.715	41.206	109.507	0
0.701	84.403	380.838	416.545	0	7.443	0
0.606	175.402	330.087	422.139	3.63	2.702	0
0.444	352.566	467.889	701.633	0	23.936	0
0.292	87.032	1146.265	1179.365	49.394	75.612	0
0.285	142.063	342.882	392.545	0	9.65	0
0.13	47.11	70.992	114.484	11.314	21.439	0
0.419	146.119	1177.441	1229.986	0	0	0
0.693	102.15	437.205	520.052	3.625	42.474	0
0.386	221.732	552.804	686.733	53.73	111.95	0
0.301	71.993	142.54	166.134	0	0	0
0.148	228.172	1495.801	1553.647	0	0	0
1.398	45.551	275.256	299.585	0	12.975	0
0.701	212.1	795.479	946.614	8.579	45.375	0
0.775	95.746	396.434	448.2	13.591	53.917	11.015
0.231	99.603	459.739	514.815	13.734	75.754	10.975
0.182	115.604	877.344	896.7	76.15	224.23	55.106
0.939	61.524	909.948	932.379	207.302	211.525	197.806

0.999	136.934	1034.192	1081.726	95.488	170.664	66.404
0.45	159.409	701.392	781.009	48.743	211.845	47.351
0.909	262.62	1991.523	2116.287	257.64	588.793	106.622
0.926	122.613	863.876	891.225	48.644	241.609	47.654
0.69	226.561	291.17	415.427	18.989	92.829	28.954
0.194	109.73	626.586	690.688	13.636	75.55	11.208
0.355	126.651	1112.518	1144.093	39.43	215.974	50.177
0.537	261.086	1044.96	1200.447	94.988	268.393	66.44
0.477	139.95	369.724	435.179	41.275	129.581	41.062
0.363	151.653	1269.624	1343.749	234.293	433.084	170.456
0.538	97.748	735.567	746.849	41.212	189.781	16.904
0.192	177.45	257.825	408.321	13.941	27.816	12.78
0.349	100.159	374.212	424.132	13.086	52.057	9.296
0.586	77.727	779.369	807.784	14.454	48.548	32.278
1.249	93.994	640.863	674.767	2.949	52.288	15.597
0.279	100.156	410.098	461.742	13.188	52.099	9.261
0.44	140.431	467.662	537.818	0	7.665	5.69
1.315	202.07	532.846	619.833	2.847	34.97	0
0.233	40.419	115.372	144.932	14.237	48.109	21.983
1.203	133.971	226.014	306.901	5.26	0	0
1.141	165.695	253.322	396.638	4.894	0	10.93
1.683	64.501	247.647	278.878	0	9.013	3.858
1.387	158.546	273.678	383.948	0	0	7.114
0.411	76.436	245.312	266.396	7.099	28.979	6.372
1.152	55.912	218.183	235.75	0	8.22	0
0.44	50.05	378.494	390.663	85.049	0	31.071
1.499	63.138	793.039	806.815	11.557	57.703	0
0.654	95.093	736.379	750.73	0	0	0
0.351	66.993	137.59	194.979	0	45.317	0
0.443	106.547	309.507	379.355	14.508	67.713	0
0.529	165.817	708.36	746.527	3.704	52.017	14.104
1.088	33.548	117.277	129.76	0	0	0
0.919	165.15	326.606	407.871	0	15.602	0
0.824	41.894	119.016	134.358	0	0	0
1.027	174.946	274.767	417.796	22.27	0	0
0.617	72.301	117.98	170.112	0	0	0
0.452	54.839	202.548	203.53	9.538	25.888	11.852
0.653	167.877	227.496	308.549	4.893	0	0
0.469	133.68	379.759	437.946	0	0	0
0.529	104.893	181.452	240.327	2.993	3.312	0
0.446	80.92	143.755	181.936	0	7.814	0
0.787	217.143	373.328	455.097	14.86	67.287	9.431
0.872	37.546	169.745	187.931	11.324	55.116	0
0.327	41.282	145.243	173.621	0	0	0
0.287	126.198	442.58	452.351	0	0	0
0.809	275.44	4360.054	4440.442	0	0	0
0.756	300.583	425.299	542.545	3.734	42.537	8.528
0.775	151.059	232.157	312.818	0	2.896	0
0.616	180.066	333.463	419.029	15.676	10.553	0
0.198	834.76	1387.48	1589.829	0	84.775	0

0.299	252.175	777.03	925.533	27.98	108.778	42.91
0.575	147.164	931.013	987.883	63.185	49.029	0
0.506	142.492	491.471	553.73	5.008	83.445	0
0.432	63.403	296.146	341.352	18.854	79.173	0
0.071	53.301	267.811	296.647	3.738	18.434	0
0.452	118.762	239.956	279.498	0	11.509	0
0.561	287.852	995.137	1028.069	0	0	0
1.034	78.512	457.837	478.905	132.59	96.705	123.636
0.769	36.445	358.145	392.177	0	0	0
0.596	78.431	188.787	220.808	0	0	0
0.295	119.555	722.108	776.699	4.213	4.084	0
0.339	103.138	320.431	407.557	0	9.877	0
0.439	121.395	621.788	658.051	10.022	20.627	0
0.091	188.255	440.125	606.928	2.814	27.319	0
0.116	90.127	172.726	207.708	51.327	20.946	0
0.174	40.402	175.627	176.935	0	0	0
0.784	76.458	285.898	344.536	7.434	34.415	0
0.069	11.658	12.9	24.558	0	3.936	0
1.538	209.485	640.904	785.497	15.144	18.303	21.179
0.604	38.913	247.268	261.962	0	0	0
0.33	6.531	15.586	16.687	0	0	0
0.674	297.035	2855.359	3015.416	0	0	0
0.517	57.355	290.475	327.596	6.607	45.842	5.929
0.14	262.178	1072.234	1156.768	0	0	0
0.177	45.985	68.765	82.742	0	7.74	0
0.107	152.621	171.095	277.878	0	18.291	0
0.709	45.96	215.818	239.369	21.627	111.157	0
0.569	72.005	351.229	399.239	0	0	0
0.081	211.868	707.173	779.066	72.913	206.994	0
0.415	251.368	1622.021	1800.204	38.79	108.986	22.359
1.064	315.098	1160.907	1396.032	17.473	78.287	11.996
0.689	99.831	499.622	546.245	26.559	12.929	0
0.977	200.408	437.389	493.824	0	14.008	0
0.394	42.814	129.484	158.735	14.237	48.252	22.053
0.276	113.948	144.328	181.524	0	18.256	0
0.166	39.572	212.231	218.598	0	0	0
0.957	291.267	1801.151	2006.19	175.862	49.058	0
0.801	179.484	299.247	394.283	0	15.362	0
0.607	45.964	390.811	414.29	0	13.552	0
0.846	277.2	663.139	771.959	50.605	33.305	0
0.071	21.366	94.733	99.708	8.221	66.861	0
0.076	20.173	66.66	72.224	0	15.71	0
0.191	205.144	290.272	372.728	13.129	65.98	0
0.908	49.829	399.638	417.723	0	0	0
0.635	79.006	204.241	253.755	0	0	0
0.443	36.988	137.026	159.539	4.079	34.738	17.81
0.854	60.836	542.109	585.454	11.37	30.06	3.446
0.292	113.532	202.909	274.847	59.831	0	0
0.195	39.481	74.012	88.412	3.69	6.766	0
0.434	104.136	405.232	464.589	15.325	79.009	0

0.664	79.583	270.568	288.182	3.691	2.628	0
0.202	53.666	623.509	630.15	20.263	62.756	0
0.601	98.587	378.846	419.229	15.311	68.056	0
0.582	110.305	167.494	216.926	2.993	6.962	0
0.659	231.922	555.524	663.001	51.351	44.179	0
0.348	77.902	394.073	458.935	8.743	41.296	17.939
0.768	84.501	293.652	344.74	6.288	23.342	0
0.528	122.492	169.218	246.16	0	8.141	0
0.557	139.671	163.919	273.774	7.397	0	0
0.429	75.791	262.79	291.616	3.637	2.694	0
0.813	26.279	27.171	48.289	10.978	0	12.299
0.159	20.505	53.844	63.736	15.27	0	0
0.578	182.884	262.209	293.7	0	3.374	0
0.294	150.566	385.447	432.962	3.042	44.435	2.894
0.505	156.19	944.182	1012.978	144.06	440.692	0
0.415	133.758	380.578	448.634	0	0	0
0.75	91.902	456.859	515.987	20.253	27.885	0
0.357	53.094	662.064	670.001	0	19.907	0
0.515	37.64	42.678	71.379	3.614	29.35	0
0.369	127.734	229.937	279.78	7.417	0	0
1.069	508.976	1576.745	1735.126	0	29.381	8.687
0.176	109.983	147.104	178.577	0	17.558	0
0.308	151.576	467.91	552.467	15.111	74.995	0
0.359	117.335	339.283	413.403	0	19.796	0
0.577	86.838	583.909	630.06	0	26.38	0
0.428	228.446	447.921	568.488	3.12	35.696	2.903
0.325	69.362	122.115	182.241	0	30.401	0
0.306	137.006	291.204	333.904	0	14.745	0
0.325	124.505	200.431	235.817	0	14.183	0
0.157	37.584	226.401	241.517	0	0	0
0.339	59.551	224.973	245.622	6.737	10.282	0
1.031	131.598	953.203	1023.938	26.428	56.197	0
0.202	167.172	203.584	274.99	5.505	46.158	0
0.223	88.212	210.438	260.699	5.65	34.694	6.634
0.132	194.686	250.386	353.989	7.421	58.04	0
0.099	24.153	158.774	181.624	24.1	86.556	0
0.475	24.904	82.302	94.725	0	17.519	0
0.493	87.929	292.712	343.407	14.508	67.713	0
0.668	147.413	241.225	305.912	0	0	0
0.74	231.75	2539.529	2564.765	12.162	161.332	0
0.432	104.186	405.257	464.385	15.327	78.987	0
0.381	65.383	664.707	704.576	11.373	51.659	3.446
0.354	97.94	257.428	334.629	14.151	10.542	0
0.414	198.898	354.789	445.046	14.904	67.308	9.471
0.536	96.441	156.347	218.035	2.993	6.244	0
0.199	125.582	200.142	247.309	0	21.053	0
0.626	139.901	356.496	450.387	0	18.823	0
0.059	287.829	1535.289	1639.399	10.813	56.476	0
0.169	61.594	122.193	145.082	0	8.056	0
0.18	23.44	171.489	189.472	0	5.529	0

0.364	134.437	148.76	221.758	0	0	0
0.419	54.248	110.156	135.467	0	8.056	0
0.792	133.72	343.364	433.881	0	6.878	0
0.151	87.195	141.698	167.67	0	3.533	0
1.147	52.333	418.552	448.02	9.471	19.577	0
1.562	358.848	713.441	967.708	17.152	53.383	29.442
0.872	156.951	414.961	503.015	44.737	42.021	0
0.398	191.81	227.288	318.714	5.522	42.11	5.151
0.501	44.993	207.524	224.044	0	13.552	0
0.7	50.982	554.724	583.671	10.092	31.457	0
0.429	269.052	543.2	749.708	0	0	0
0.309	115.095	678.179	767.208	28.291	124.636	0
0.402	112.822	348.733	396.529	0	0	0
0.156	103.022	376.62	424.829	0	0	0
0.814	52.155	388.586	415.808	20.244	11.405	0
0.713	74.134	287.313	307.701	8.662	20.338	0
1.164	165.009	909.955	1034.554	0	14.242	0
1.469	60.331	475.708	513.772	0	7.755	0
0.192	44.616	145.526	153.738	0	0	0
1.192	60.334	475.694	513.762	0	7.755	0
0.823	63.238	159.525	191.45	8.776	0	0
1.705	178.183	619.753	707.817	7.531	38.256	13.678
0.428	157.723	318.143	373.641	6.41	19.667	0
0.46	143.84	369.493	414.259	6.407	19.282	0
0.312	63.809	713.794	722.471	43.433	75.321	23.906
0.437	274.585	899.725	913.224	4.213	61.083	0
0.836	18.676	101.666	118.681	0	12.971	0
0.145	59.917	86.248	107.34	0	34.172	0
0.569	87.921	1077.608	1109.672	37.38	46.959	0
0.723	124.324	260.08	343.577	48.918	0	0
0.123	275.024	1091.955	1161.212	9.649	53.432	0
0.47	132.369	307.145	366.987	3.638	2.692	0
0.358	18.308	33.761	51.574	0	0	0
0.487	69.594	349.591	355.188	0	16.581	0
0.283	256.277	879.822	1027.07	91.665	135.711	34.763
0.949	73.76	308.673	336.993	6.906	26.366	8.032
0.697	197.739	569.863	605.226	3.483	11.489	13.186
0.458	59.517	169.707	200.378	12.871	30.487	8.955
0.199	97.917	306.759	316.458	25.354	33.001	14.694
0.157	156.914	693.268	795.222	0	20.021	11.822
0.373	68.574	200.812	228.456	16.887	28.794	10.163
0.585	105.644	413.669	472.64	0	0	0
0.797	132.428	432.028	532.96	18.624	59.481	13.909
0.551	117.743	320.332	410.296	22.23	27.29	13.697
1.105	41.868	263.921	281.009	0	0	0
0.258	301.074	314.32	405.142	6.763	43.823	5.365
1.006	82.045	748.203	804.258	30.206	42.905	0
0.549	110.08	187.133	241.536	2.994	6.249	0
0.323	83.448	153.612	177.544	23.004	97.247	0
0.998	80.91	275.135	326.564	9.548	43.654	29.195

0.306	123.151	720.413	810.171	28.29	131.356	0
0.51	50.059	222.679	237.929	0	16.511	0
0.241	88.807	275.729	299.053	0	0	0
0.532	145.462	487.751	559.379	5.004	88.89	0
0.223	36.325	53.562	87.474	11.314	21.439	0
0.869	62.832	158.7	180.035	0	8.056	0
0.57	73.168	202.822	230.141	0	0	4.478
0.888	91.581	496.898	549.5	25.887	53.125	11.947
0.137	92.242	995.339	1047.696	9.082	50.183	0
0.702	79.444	420.104	438.42	2.707	22.331	8.219
0.787	77.016	350.143	390.344	0	9.013	3.858
0.245	122.621	226.832	294.967	0	11.556	0
0.585	117.554	776.936	849.965	44.745	16.815	0
0.361	124.392	228.874	265.302	10.818	0	0
0.251	127.655	220.465	276.943	8.774	87.186	0
0.278	109.697	1062.94	1101.405	56.761	95.72	0
0.044	23.967	100.247	112.497	0	0	0
0.872	230.53	692.456	752.057	24.864	80.962	0
0.145	369.879	1228.476	1322.469	9.582	56.31	0
0.095	222.134	1396.014	1477.192	5.601	69.626	0
0.345	603.96	1675.165	1832.69	41.225	212.277	0
0.509	230.83	284.6	456.995	3.676	19.468	0
0.312	145.815	249.029	302.723	0	0	0
1.186	89.706	381.365	385.577	46.771	35.152	0
0.384	62.641	201.458	221.268	37.907	93.443	0
0.532	90.297	921.38	942.305	38.278	35.106	24.23
0.51	164.415	329.562	459.867	10.294	7.362	0
1.128	72.002	262.876	284.639	0	11.25	0

G(N..P)	G(N..F)	G(N..Cl)	G(N..Br)	G(N..I)	G(O..O)	G(O..S)
0	0	0	0	0	42.639	0
0	0	0	0	0	30.941	0
0	0	0	0	0	84.636	0
0	0	0	0	0	6.023	0
0	0	0	0	0	18.844	0
11.795	0	0	0	0	59.291	0
0	0	0	0	0	20.493	0
0	0	0	0	0	0	0
0	0	28.122	0	0	0	0
0	0	0	0	0	2.874	0
0	0	0	0	0	479.783	0
0	0	0	0	9.593	18.685	0
0	0	0	0	0	0	0
0	0	16.153	0	0	48.166	0
0	0	0	0	0	51.304	27.099
0	0	0	0	0	27.724	18.98
0	0	0	0	0	0	0
0	275.596	0	0	0	11.247	0
0	0	0	0	0	17.399	0
0	0	0	0	0	6.805	0
0	0	0	0	0	4.723	0
0	0	0	0	0	0	7.85
0	0	6.819	0	0	13.51	0
0	0	0	0	0	27.165	18.944
0	0	0	0	0	0	0
0	0	0	0	0	66.128	0
0	0	0	0	0	20.729	0
0	0	0	0	0	120.271	0
0	0	0	0	0	0	0
0	0	0	0	0	40.706	0
0	0	0	0	0	80.625	25.969
0	0	0	28.124	0	37.775	0
0	0	0	0	0	2.859	0
0	0	0	0	0	0	0
0	0	0	0	0	27.932	0
0	0	0	0	0	4.41	0
0	0	0	0	0	7.252	0
0	0	0	0	0	4.437	0
0	0	0	0	0	36.498	0
0	0	0	0	0	22.001	0
0	0	0	0	0	11.988	0
0	0	0	0	0	0	0
0	0	0	0	0	197.262	0
0	0	5.878	0	0	0	0
0	0	0	0	0	26.113	0
0	0	21.598	0	0	25.796	20.384
0	0	0	0	0	58.796	30.665
0	0	0	0	0	53.494	61.973
0	0	0	0	0	26.257	79.074

0	0	0	0	0	56.476	69.59
0	0	0	0	0	145.671	102.152
0	0	0	0	0	212.055	133.523
0	0	0	0	0	135.244	110.088
0	0	0	0	0	101.352	78.508
0	0	0	0	0	67.438	28.429
0	0	0	0	0	179.254	86.661
0	0	0	0	0	143.277	102.109
0	0	0	0	0	52.928	66.575
0	0	0	0	0	167.718	170.701
0	0	0	0	0	191.932	46.792
0	53.321	0	0	0	0	0
0	0	0	0	0	26.068	20.248
0	0	0	0	0	25.81	51.927
0	0	0	0	0	79.379	87.366
0	0	0	0	0	25.563	20.265
0	0	0	0	0	0	10.155
0	0	15.428	0	0	6.258	0
0	0	15.228	0	0	24.229	23.153
0	0	16.209	0	0	0	0
0	0	11.531	0	0	0	0
0	0	14.8	0	0	6.865	2.81
0	0	5.63	0	0	0	0
0	0	9.897	0	0	33.245	13.692
0	0	7.769	0	0	4.427	0
0	0	0	0	0	0	0
0	0	0	0	0	31.887	0
0	24.758	0	0	0	0	0
0	0	0	0	0	51.979	0
0	13.633	0	0	0	6.674	0
0	0	9.261	0	0	44.459	19.242
0	0	0	0	0	6.258	0
0	0	8.359	0	0	33.986	0
0	0	22.477	0	0	0	0
0	0	26.817	0	0	0	0
0	0	19.688	0	0	0	0
0	0	0	0	0	0	0
0	0	0	0	0	0	0
0	0	0	0	0	66.269	0
0	0	12.159	0	0	0	0
0	0	5.948	0	0	0	0
0	0	29.536	0	0	57.868	25.373
0	0	0	0	0	19.266	0
0	0	0	0	0	6.669	0
0	0	0	0	0	42.208	0
0	0	0	0	0	856.083	0
0	0	0	0	0	26.333	19.338
0	0	0	0	0	0	0
0	0	0	0	0	0	0
0	0	0	0	0	556.942	0

0	0	0	0	0	95.617	73.564
0	0	50.777	0	0	8.833	0
0	0	0	0	0	146.415	0
0	0	0	0	0	52.09	0
0	0	0	0	0	8.768	0
0	0	0	0	0	6.731	0
0	0	0	0	0	553.548	0
0	0	0	0	0	0	36.148
0	0	0	0	0	9.826	0
0	0	0	0	0	12.738	0
0	0	0	0	0	0	0
0	0	6.786	0	0	0	8.427
0	0	0	0	0	10.325	0
0	97.069	0	0	0	13.938	0
0	73.127	0	0	0	0	0
0	0	0	0	0	4.733	0
0	19.097	0	0	0	13.249	0
0	7.215	0	0	0	4.439	0
0	62.718	0	0	0	0	12.73
0	0	0	0	0	0	0
0	0	0	0	0	20.906	0
0	0	0	0	0	42.604	32.061
0	0	8.339	0	0	55.314	20.668
0	0	0	0	0	98.276	0
0	0	0	0	0	0	0
0	0	0	0	0	10.93	0
0	0	0	0	0	33.13	0
0	0	0	0	0	13.73	0
0	0	0	0	0	100.376	0
0	0	0	0	0	70.405	22.121
0	0	33.306	0	0	71.115	23.028
0	0	0	0	0	0	0
0	27.34	8.704	0	0	26.123	0
0	0	15.217	0	0	24.138	23.088
0	0	0	0	0	10.376	0
0	0	0	0	0	0	0
0	0	0	0	0	0	0
0	0	6.758	0	0	33.986	0
0	0	0	0	0	14.341	0
0	0	0	0	0	0	0
0	0	0	0	0	130.237	0
0	0	0	0	0	57.426	0
0	0	0	0	0	47.075	0
0	0	0	0	0	7.557	0
0	0	0	0	0	14.972	0
0	0	0	0	0	13.137	7.588
0	84.666	0	0	0	6.312	5.687
0	0	0	0	0	0	0
0	0	0	0	0	2.739	0
0	12.453	0	0	0	26.947	0

0	0	0	0	0	0	0
0	16.692	0	0	0	44.415	0
0	23.313	0	0	0	6.674	0
0	0	19.898	0	0	2.59	0
0	0	37.261	0	0	0	0
0	0	0	0	0	23.485	34.049
0	0	25.724	0	0	0	0
0	0	0	0	0	0	0
0	0	0	0	0	0	0
0	0	0	0	0	0	0
0	0	0	0	0	0	0
0	0	0	0	0	0	0
0	0	0	0	0	0	0
0	0	0	0	0	82.317	29.814
0	0	0	0	0	45.69	0
0	0	0	0	0	66.344	0
0	0	16.826	0	0	2.499	0
0	0	0	0	0	20.728	0
0	0	0	0	0	9.986	0
0	12.959	20.167	0	0	0	0
0	0	5.067	0	0	20.256	14.697
0	0	0	0	0	8.845	0
0	10.547	0	0	0	26.949	0
0	0	0	0	0	11.362	0
0	0	0	0	0	30.153	0
0	0	0	0	0	55.201	24.097
0	0	0	0	0	6.683	0
0	0	0	0	0	14.514	0
0	0	0	0	0	11.675	0
0	0	0	0	0	17.895	0
0	0	0	0	0	0	0
0	0	0	30.597	0	11.994	0
0	0	0	0	0	60.473	0
0	0	0	0	0	52.265	18.893
0	0	0	0	0	77.654	0
0	0	0	0	0	60.411	0
0	0	0	0	0	14.914	0
0	13.633	0	0	0	6.674	0
0	0	0	0	0	0	0
0	0	0	0	0	423.19	0
0	12.449	0	0	0	26.947	0
0	0	0	0	0	25.448	13.242
0	0	0	0	0	0	0
0	0	0	0	0	57.818	25.337
0	0	12.16	0	0	3.338	0
0	0	0	0	0	26.441	0
0	0	0	0	0	19.741	0
0	0	0	0	0	45.392	0
0	0	0	0	0	13.575	0
0	0	0	0	0	6.022	0

0	0	0	0	0	0	0
0	0	0	0	0	13.575	0
0	0	0	0	0	4.728	0
0	0	0	0	0	4.497	0
0	0	0	0	0	2.874	0
0	0	0	0	0	23.96	39.398
0	0	0	0	0	5.028	0
0	0	0	0	0	44.49	14.612
0	0	2.975	0	0	0	0
0	0	0	0	0	17.549	0
0	0	0	0	0	139.168	0
0	0	0	0	0	53.481	0
0	0	0	0	0	65.817	0
0	0	0	0	0	11.483	0
0	0	0	0	0	0	0
0	0	0	0	0	0	0
0	0	0	0	0	9.542	0
0	0	0	0	0	2.874	0
0	0	0	0	0	0	0
0	0	0	0	0	2.874	0
0	0	32.804	0	0	0	0
0	0	0	0	0	2.874	21.033
0	0	0	0	0	6.302	0
0	0	0	0	0	6.425	0
0	0	0	0	0	23.108	19.971
0	0	0	0	0	25.136	0
0	46.867	0	0	0	0	5.25
8.96	0	0	0	0	59.312	0
0	45.761	0	0	0	10.133	0
0	0	0	0	0	0	0
0	0	0	0	0	48.887	0
0	0	0	0	0	0	0
0	0	0	0	0	6.678	0
0	0	0	0	0	18.638	0
0	0	0	0	0	33.84	12.966
0	0	0	0	0	15.899	7.321
0	0	0	0	0	3.505	17.821
0	0	0	0	0	9.586	4.23
0	0	0	0	0	0	0
0	0	0	0	0	16.043	10.247
0	0	0	0	0	7.281	3.038
0	0	0	0	0	21.816	22.179
0	0	0	0	0	28.985	12.2
0	0	0	0	0	0	0
0	0	0	0	0	14.985	18.871
0	0	0	0	0	48.668	16.165
0	0	0	0	0	0	0
0	0	12.158	0	0	3.337	0
24.166	0	0	0	0	10.327	0
0	0	0	0	0	23.758	28.152

0	0	0	0	0	55.08	0
0	0	0	0	0	15.654	0
0	0	0	0	0	9.871	0
0	0	0	0	0	155.568	0
0	0	0	0	0	4.437	0
0	0	0	0	0	4.739	9.69
0	0	3.355	0	0	0	0
0	0	0	0	0	14.837	16.481
0	0	0	0	0	46.457	0
0	0	0	0	0	6.902	2.812
0	0	0	0	0	6.865	2.81
0	0	0	0	0	4.937	0
0	0	46.702	0	0	0	0
0	0	46.079	0	0	0	0
0	0	0	0	0	10.27	0
0	0	0	0	0	30.606	0
0	0	0	0	0	0	0
0	0	0	0	0	8.875	0
0	0	0	0	0	45.072	0
0	0	0	0	0	65.694	0
0	0	0	0	0	165.839	0
0	0	0	0	0	0	0
0	0	0	0	0	19.802	0
0	0	0	0	0	0	0
0	0	0	0	0	29.833	0
0	0	25.549	0	0	0	15.452
0	0	0	0	0	0	0
0	0	7.419	0	0	13.035	0

G(O..P)	G(O..F)	G(O..Cl)	G(O..Br)	G(O..I)	G(S..S)	G(S..P)
0	0	0	0	0	0	0
0	0	0	0	0	0	0
0	0	0	0	0	0	0
0	0	0	0	0	0	0
0	0	0	0	0	0	0
22.2	0	0	0	0	0	0
0	0	0	0	0	0	0
0	0	0	0	0	0	0
0	0	0	0	0	0	0
0	0	0	0	0	0	0
0	0	0	0	0	0	0
0	0	0	0	33.786	0	0
0	0	0	0	0	0	0
0	0	24.984	0	0	0	0
0	0	0	0	0	0	0
0	0	0	0	0	0	0
0	0	0	0	0	0	0
0	168.697	0	0	0	0	0
0	0	0	0	0	0	0
0	0	0	0	0	0	0
0	0	0	0	0	0	0
0	0	0	0	0	0	0
0	0	33.013	0	0	0	0
0	0	0	0	0	0	0
0	0	0	0	0	0	0
0	27.957	0	0	0	0	0
0	0	0	0	0	0	0
0	0	0	0	0	0	0
0	0	0	0	0	0	0
0	0	0	0	0	0	0
0	0	0	0	0	0	0
0	0	0	60.785	0	0	0
0	0	0	0	0	0	0
0	0	0	0	0	0	0
0	0	0	0	0	0	0
0	0	0	0	0	0	0
0	0	0	0	0	0	0
0	0	0	0	0	0	0
0	0	0	0	0	0	0
0	0	0	0	0	0	0
0	0	0	0	0	0	0
0	0	0	0	0	0	0
0	0	0	0	0	0	0
0	0	20.969	0	0	0	0
0	0	0	0	0	0	0
0	0	20.493	0	0	0	0
0	0	0	0	0	0	0
0	0	0	0	0	4.677	0
0	0	0	0	0	13.228	0

0	0	0	0	0	9.678	0
0	0	0	0	0	9.742	0
0	0	0	0	0	4.705	0
0	0	0	0	0	9.688	0
0	0	0	0	0	5.856	0
0	0	0	0	0	0	0
0	0	0	0	0	5.991	0
0	0	0	0	0	9.771	0
0	0	0	0	0	8.374	0
0	0	0	0	0	28.098	0
0	0	0	0	0	0	0
0	62.45	0	0	0	0	0
0	0	0	0	0	0	0
0	0	0	0	0	7.144	0
0	0	0	0	0	7.169	0
0	0	0	0	0	0	0
0	0	0	0	0	0	0
0	0	38.434	0	0	0	0
0	0	21.011	0	0	5.46	0
0	0	0	0	0	0	0
0	0	0	0	0	0	0
0	0	20.106	0	0	0	0
0	0	0	0	0	0	0
0	0	22.375	0	0	0	0
0	0	16.517	0	0	0	0
0	0	0	0	0	0	0
0	0	0	0	0	0	0
0	0	0	0	0	0	0
0	0	0	0	0	0	0
0	21.094	0	0	0	0	0
0	0	26.777	0	0	0	0
0	0	19.333	0	0	0	0
0	0	35.646	0	0	0	0
0	0	0	0	0	0	0
0	0	0	0	0	0	0
0	0	21.739	0	0	0	0
0	0	0	0	0	0	0
0	0	0	0	0	0	0
0	27.967	0	0	0	0	0
0	0	7.959	0	0	0	0
0	0	19.162	0	0	0	0
0	0	60.712	0	0	0	0
0	0	0	0	0	0	0
0	44.962	0	0	0	0	0
0	0	0	0	0	0	0
0	0	0	0	0	0	0
0	0	0	0	0	0	0
0	0	0	0	0	0	0
0	0	0	0	0	0	0
0	0	0	0	0	0	0

0	0	0	0	0	13.472	0
0	0	22.562	0	0	0	0
0	0	0	0	0	0	0
0	0	0	0	0	0	0
0	0	0	0	0	0	0
0	0	0	0	0	0	0
0	0	0	0	0	0	0
0	0	0	0	0	21.808	0
0	0	0	0	0	0	0
0	0	0	0	0	0	0
0	0	0	0	0	0	0
0	0	14.173	0	0	0	0
0	0	0	0	0	0	0
0	108.501	0	0	0	0	0
0	10.468	0	0	0	0	0
0	12.836	0	0	0	0	0
0	24.186	0	0	0	0	0
0	8.01	0	0	0	0	0
0	35.212	0	0	0	0	0
0	12.706	0	0	0	0	0
0	0	0	0	0	0	0
0	252.087	0	0	0	0	0
0	0	28.479	0	0	0	0
0	0	0	0	0	0	0
0	0	0	0	0	0	0
0	0	0	0	0	0	0
0	0	0	0	0	0	0
0	0	0	0	0	0	0
0	0	0	0	0	0	0
0	0	0	0	0	0	0
0	0	52.348	0	0	0	0
0	0	0	0	0	0	0
0	93.669	30.178	0	0	0	0
0	0	21.022	0	0	5.448	0
0	0	0	0	0	0	0
0	0	0	0	0	0	0
0	0	0	0	0	0	0
0	0	35.348	0	0	0	0
0	0	0	0	0	0	0
0	0	0	0	0	0	0
0	0	0	0	0	0	0
0	0	0	0	0	0	0
0	0	0	0	0	0	0
0	0	26.427	0	0	0	0
0	0	0	0	0	0	0
0	0	0	0	0	0	0
0	36.487	0	0	0	0	0
0	0	0	0	0	0	0
0	0	0	0	0	0	0
0	25.873	0	0	0	0	0

0	0	0	0	0	0	0
0	26.593	0	0	0	0	0
0	41.339	0	0	0	0	0
0	0	27.005	0	0	0	0
0	0	3.797	0	0	0	0
0	0	0	0	0	7.722	0
0	0	43.828	0	0	0	0
0	0	0	0	0	0	0
0	0	0	0	0	0	0
0	0	0	0	0	0	0
0	0	0	0	0	0	0
0	0	0	0	0	0	0
0	0	0	0	0	0	0
0	0	0	0	0	0	0
0	0	0	0	0	0	0
0	0	0	0	0	0	0
0	0	12.155	0	0	0	0
0	0	0	0	0	0	0
0	0	0	0	0	0	0
0	0	0	0	0	0	0
0	0	37.866	0	0	0	0
0	0	0	0	0	0	0
0	25.853	0	0	0	0	0
0	0	0	0	0	0	0
0	0	0	0	0	0	0
0	0	0	0	0	0	0
0	0	0	0	0	0	0
0	0	0	0	0	0	0
0	0	0	0	0	0	0
0	0	0	0	0	0	0
0	0	0	0	0	0	0
0	0	0	23.628	0	0	0
0	0	0	0	0	0	0
0	0	0	0	0	0	0
0	0	0	0	0	0	0
0	0	0	0	0	0	0
0	0	0	0	0	0	0
0	21.094	0	0	0	0	0
0	0	0	0	0	0	0
0	0	0	0	0	0	0
0	25.846	0	0	0	0	0
0	0	0	0	0	0	0
0	0	0	0	0	0	0
0	0	0	0	0	0	0
0	0	13.926	0	0	0	0
0	0	0	0	0	0	0
0	0	0	0	0	0	0
0	0	0	0	0	0	0
0	0	0	0	0	0	0
0	0	0	0	0	0	0

0	0	0	0	0	0	0
0	0	0	0	0	0	0
0	0	0	0	0	0	0
0	0	0	0	0	0	0
0	0	0	0	0	0	0
0	0	0	0	0	6.293	0
0	0	0	0	0	0	0
0	0	0	0	0	0	0
0	0	11.584	0	0	0	0
0	0	0	0	0	0	0
0	0	0	0	0	0	0
0	0	0	0	0	0	0
0	0	0	0	0	0	0
0	0	0	0	0	0	0
0	0	0	0	0	0	0
0	0	0	0	0	0	0
0	0	0	0	0	0	0
0	0	0	0	0	0	0
0	0	0	0	0	0	0
0	0	0	0	0	0	0
0	0	0	0	0	0	0
0	0	0	0	0	0	0
0	0	0	0	0	0	0
0	0	0	0	0	0	0
0	0	0	0	0	0	0
0	0	0	0	0	0	0
0	0	0	0	0	0	0
22.193	0	0	0	0	0	0
0	18.866	0	0	0	0	0
0	0	0	0	0	0	0
0	0	0	0	0	0	0
0	0	0	0	0	0	0
0	0	0	0	0	0	0
0	0	0	0	0	0	0
0	0	0	0	0	0	0
0	0	0	0	0	0	0
0	0	0	0	0	0	0
0	0	0	0	0	0	0
0	0	0	0	0	0	0
0	0	0	0	0	0	0
0	0	0	0	0	0	0
0	20.391	0	0	0	0	0
0	0	0	0	0	0	0
0	0	0	0	0	0	0
0	0	0	0	0	0	0
0	0	0	0	0	0	0
0	0	0	0	0	0	0
0	0	13.919	0	0	0	0
0	0	0	0	0	0	0
0	0	0	0	0	4.273	0

0	0	0	0	0	0	0
0	0	0	0	0	0	0
0	0	0	0	0	0	0
0	0	0	0	0	0	0
0	0	0	0	0	0	0
0	0	0	0	0	0	0
0	0	0	0	0	0	0
0	0	0	0	0	0	0
0	0	0	0	0	0	0
0	0	0	0	0	0	0
0	0	0	0	0	0	0
0	0	0	0	0	0	0
0	0	11.217	0	0	0	0
0	0	0	0	0	0	0
0	0	0	0	0	0	0
0	0	0	0	0	0	0
0	0	0	0	0	0	0
0	0	0	0	0	0	0
0	0	0	0	0	0	0
0	0	0	0	0	0	0
0	0	0	0	0	0	0
0	0	0	0	0	0	0
0	0	0	0	0	0	0
0	0	0	0	0	0	0
0	0	0	0	0	0	0
0	0	7.437	0	0	0	0
0	0	0	0	0	0	0
0	0	29.199	0	0	0	0

G(S..F)	G(S..Cl)	G(S..Br)	G(S..I)	G(P..P)	G(P..F)	G(P..Cl)
0	0	0	0	0	0	0
0	0	0	0	0	0	0
0	0	0	0	0	0	0
0	0	0	0	0	0	0
0	0	0	0	0	0	0
0	0	0	0	0	0	0
0	0	0	0	0	0	0
0	0	0	0	0	0	0
0	0	0	0	0	0	0
0	0	0	0	0	0	0
0	0	0	0	0	0	0
0	0	0	0	0	0	0
0	0	0	0	0	0	0
0	0	0	0	0	0	0
0	0	0	0	0	0	0
0	0	0	0	0	0	0
0	0	0	0	0	0	0
0	0	0	0	0	0	0
0	0	0	0	0	0	0
0	0	0	0	0	0	0
0	0	0	0	0	0	0
0	0	0	0	0	0	0
0	0	0	0	0	0	0
0	0	0	0	0	0	0
0	0	0	0	0	0	0
0	0	0	0	0	0	0
0	0	0	0	0	0	0
0	0	0	0	0	0	0
0	0	0	0	0	0	0
0	0	0	0	0	0	0
0	0	0	0	0	0	0
0	0	0	0	0	0	0
0	0	0	0	0	0	0
0	0	0	0	0	0	0
0	0	0	0	0	0	0
0	0	0	0	0	0	0
0	0	0	0	0	0	0
0	0	0	0	0	0	0
0	0	0	0	0	0	0
0	0	0	0	0	0	0
0	0	0	0	0	0	0
0	0	0	0	0	0	0
0	0	0	0	0	0	0
0	0	0	0	0	0	0
0	0	0	0	0	0	0
0	4.34	0	0	0	0	0
0	0	0	0	0	0	0
0	0	0	0	0	0	0
0	0	0	0	0	0	0

0	0	0	0	0	0	0
0	0	0	0	0	0	0
0	0	0	0	0	0	0
0	0	0	0	0	0	0
0	0	0	0	0	0	0
0	0	0	0	0	0	0
0	0	0	0	0	0	0
0	0	0	0	0	0	0
0	0	0	0	0	0	0
0	0	0	0	0	0	0
0	0	0	0	0	0	0
29.568	0	0	0	0	0	0
0	0	0	0	0	0	0
0	0	0	0	0	0	0
0	0	0	0	0	0	0
0	0	0	0	0	0	0
0	0	0	0	0	0	0
0	0	0	0	0	0	0
0	9.389	0	0	0	0	0
0	0	0	0	0	0	0
0	6.268	0	0	0	0	0
0	6.263	0	0	0	0	0
0	6.274	0	0	0	0	0
0	3.133	0	0	0	0	0
0	0	0	0	0	0	0
0	0	0	0	0	0	0
0	0	0	0	0	0	0
0	0	0	0	0	0	0
0	0	0	0	0	0	0
0	0	0	0	0	0	0
0	4.237	0	0	0	0	0
0	0	0	0	0	0	0
0	0	0	0	0	0	0
0	0	0	0	0	0	0
0	0	0	0	0	0	0
0	0	0	0	0	0	9.998
0	0	0	0	0	0	0
0	0	0	0	0	0	0
0	0	0	0	0	0	0
0	0	0	0	0	0	0
0	0	0	0	0	0	0
0	13.175	0	0	0	0	0
0	0	0	0	0	0	0
0	0	0	0	0	0	0
0	0	0	0	0	0	0
0	0	0	0	0	0	0
0	0	0	0	0	0	0
0	0	0	0	0	0	0
0	0	0	0	0	0	0
0	0	0	0	0	0	0

0	0	0	0	0	0	0
0	0	0	0	0	0	0
0	0	0	0	0	0	0
0	0	0	0	0	0	0
0	0	0	0	0	0	0
0	0	0	0	0	0	0
0	0	0	0	0	0	0
0	0	0	0	0	0	0
0	0	0	0	0	0	0
0	0	0	0	0	0	0
0	0	0	0	0	0	0
0	8.448	0	0	0	0	0
0	0	0	0	0	0	0
0	0	0	0	0	0	0
0	0	0	0	0	0	0
0	0	0	0	0	0	0
0	0	0	0	0	0	0
0	0	0	0	0	0	0
19.91	0	0	0	0	0	0
0	0	0	0	0	0	0
0	0	0	0	0	0	0
32.076	0	0	0	0	0	0
0	3.126	0	0	0	0	0
0	0	0	0	0	0	0
0	0	0	0	0	0	0
0	0	0	0	0	0	0
0	0	0	0	0	0	0
0	0	0	0	0	0	0
0	0	0	0	0	0	0
0	0	0	0	0	0	0
0	13.112	0	0	0	0	0
0	0	0	0	0	0	0
0	0	0	0	0	0	0
0	9.398	0	0	0	0	0
0	0	0	0	0	0	0
0	0	0	0	0	0	0
0	0	0	0	0	0	0
0	0	0	0	0	0	0
0	0	0	0	0	0	0
0	0	0	0	0	0	0
0	0	0	0	0	0	0
0	0	0	0	0	0	0
0	0	0	0	0	0	0
0	0	0	0	0	0	0
0	0	0	0	0	0	0
0	0	0	0	0	0	0
25.387	0	0	0	0	0	0
0	0	0	0	0	0	0
0	0	0	0	0	0	0
0	0	0	0	0	0	0

0	0	0	0	0	0	0
0	0	0	0	0	0	0
0	0	0	0	0	0	0
0	0	0	0	0	0	0
0	0	0	0	0	0	0
0	0	0	0	0	0	0
0	0	0	0	0	0	0
0	0	0	0	0	0	0
0	0	0	0	0	0	0
0	0	0	0	0	0	0
0	0	0	0	0	0	0
0	0	0	0	0	0	0
0	0	0	0	0	0	0
0	0	0	0	0	0	0
0	0	0	0	0	0	0
0	0	0	0	0	0	0
0	0	0	0	0	0	0
0	0	0	0	0	0	0
0	0	0	0	0	0	0
0	0	0	0	0	0	0
0	12.459	0	0	0	0	0
0	0	0	0	0	0	0
0	0	0	0	0	0	0
0	0	0	0	0	0	0
0	0	0	0	0	0	0
0	0	0	0	0	0	0
0	0	0	0	0	0	0
0	0	0	0	0	0	0
0	0	0	0	0	0	0
0	0	0	0	0	0	0
0	0	0	0	0	0	0
0	0	0	0	0	0	0
0	0	0	0	0	0	0
0	0	0	0	0	0	0
0	0	0	0	0	0	0
0	0	0	0	0	0	0
0	0	0	0	0	0	0
0	0	0	0	0	0	0
0	0	0	0	0	0	0
0	0	0	0	0	0	0
0	0	0	0	0	0	0
0	0	0	0	0	0	0
0	0	0	0	0	0	0
0	0	0	0	0	0	0
0	0	0	0	0	0	0
0	0	0	0	0	0	0
0	0	0	0	0	0	0
0	0	0	0	0	0	0
0	0	0	0	0	0	0
0	0	0	0	0	0	0

0	0	0	0	0	0	0
0	0	0	0	0	0	0
0	0	0	0	0	0	0
0	0	0	0	0	0	0
0	0	0	0	0	0	0
0	0	0	0	0	0	0
0	0	0	0	0	0	0
0	0	0	0	0	0	0
0	0	0	0	0	0	0
0	0	0	0	0	0	0
0	0	0	0	0	0	0
0	0	0	0	0	0	0
0	0	0	0	0	0	0
0	0	0	0	0	0	0
0	0	0	0	0	0	0
0	0	0	0	0	0	0
0	0	0	0	0	0	0
0	0	0	0	0	0	0
0	0	0	0	0	0	0
0	0	0	0	0	0	0
0	0	0	0	0	0	0
0	0	0	0	0	0	0
0	0	0	0	0	0	0
0	0	0	0	0	0	0
0	0	0	0	0	0	0
0	0	0	0	0	0	0
21.043	0	0	0	0	0	0
0	0	0	0	0	0	0
0	0	0	0	0	0	0
0	0	0	0	0	0	0
0	0	0	0	0	0	0
0	0	0	0	0	0	0
0	0	0	0	0	0	0
0	0	0	0	0	0	0
0	0	0	0	0	0	0
0	0	0	0	0	0	0
0	0	0	0	0	0	0
0	0	0	0	0	0	0
0	0	0	0	0	0	0
0	0	0	0	0	0	0
0	0	0	0	0	0	0
8.55	0	0	0	0	0	0
0	0	0	0	0	0	0
0	0	0	0	0	0	0
0	0	0	0	0	0	0
0	0	0	0	0	0	0
0	0	0	0	0	0	0
0	0	0	0	0	0	0
0	0	0	0	0	0	0
0	0	0	0	0	0	0

0	0	0	0	0	0	0
0	0	0	0	0	0	0
0	0	0	0	0	0	0
0	0	0	0	0	0	0
0	0	0	0	0	0	0
0	0	0	0	0	0	0
0	6.946	0	0	0	0	0
0	0	0	0	0	0	0
0	0	0	0	0	0	0
0	0	0	0	0	0	0
0	0	0	0	0	0	0
0	0	0	0	0	0	0
0	0	0	0	0	0	0
0	0	0	0	0	0	0
0	0	0	0	0	0	0
0	0	0	0	0	0	0
0	0	0	0	0	0	0
0	0	0	0	0	0	0
0	0	0	0	0	0	0
0	0	0	0	0	0	0
0	0	0	0	0	0	0
0	0	0	0	0	0	0
0	0	0	0	0	0	0
0	0	0	0	0	0	0
0	0	0	0	0	0	0
0	10.358	0	0	0	0	0
0	0	0	0	0	0	0
0	0	0	0	0	0	0

G(P..Br)	G(P..I)	G(F..F)	G(F..Cl)	G(F..Br)	G(F..I)	G(Cl..Cl)
0	0	0	0	0	0	0
0	0	0	0	0	0	0
0	0	0	0	0	0	0
0	0	0	0	0	0	0
0	0	0	0	0	0	0
0	0	0	0	0	0	0
0	0	0	0	0	0	0
0	0	0	0	0	0	0
0	0	0	0	0	0	0
0	0	0	0	0	0	0
0	0	0	0	0	0	0
0	0	0	0	0	0	0
0	0	0	0	0	0	0
0	0	0	0	0	0	0
0	0	0	0	0	0	0
0	0	0	0	0	0	0
0	0	0	0	0	0	0
0	0	102.679	0	0	0	0
0	0	0	0	0	0	0
0	0	0	0	0	0	0
0	0	0	0	0	0	0
0	0	0	0	0	0	0
0	0	0	0	0	0	0
0	0	0	0	0	0	0
0	0	0	0	0	0	0
0	0	0	0	0	0	0
0	0	0	0	0	0	0
0	0	0	0	0	0	0
0	0	0	0	0	0	0
0	0	0	0	0	0	0
0	0	0	0	0	0	0
0	0	0	0	0	0	0
0	0	0	0	0	0	0
0	0	0	0	0	0	0
0	0	0	0	0	0	0
0	0	0	0	0	0	0
0	0	0	0	0	0	0
0	0	0	0	0	0	0
0	0	0	0	0	0	0
0	0	0	0	0	0	0
0	0	0	0	0	0	0
0	0	0	0	0	0	0
0	0	0	0	0	0	0
0	0	0	0	0	0	0
0	0	0	0	0	0	0
0	0	0	0	0	0	0
0	0	0	0	0	0	0
0	0	0	0	0	0	0
0	0	0	0	0	0	0

0	0	0	0	0	0	0
0	0	0	0	0	0	0
0	0	0	0	0	0	0
0	0	0	0	0	0	0
0	0	0	0	0	0	0
0	0	0	0	0	0	0
0	0	0	0	0	0	0
0	0	0	0	0	0	0
0	0	0	0	0	0	0
0	0	0	0	0	0	0
0	0	0	0	0	0	0
0	0	0	0	0	0	0
0	0	0	0	0	0	0
0	0	0	0	0	0	0
0	0	0	0	0	0	0
0	0	0	0	0	0	0
0	0	0	0	0	0	0
0	0	0	0	0	0	0
0	0	0	0	0	0	0
0	0	0	0	0	0	0
0	0	0	0	0	0	0
0	0	0	0	0	0	0
0	0	0	0	0	0	0
0	0	0	0	0	0	0
0	0	0	0	0	0	0
0	0	0	0	0	0	0
0	0	0	0	0	0	0
0	0	0	0	0	0	0
0	0	0	0	0	0	0
0	0	0	0	0	0	0
0	0	0	0	0	0	0
0	0	0	0	0	0	0
0	0	0	0	0	0	0
0	0	0	0	0	0	5.415
0	0	0	0	0	0	0
0	0	0	0	0	0	7.174
0	0	0	0	0	0	0
0	0	0	0	0	0	0
0	0	0	0	0	0	0
0	0	0	0	0	0	0
0	0	0	0	0	0	5.416
0	0	0	0	0	0	5.426
0	0	0	0	0	0	0
0	0	4.736	0	0	0	0
0	0	0	0	0	0	0
0	0	0	0	0	0	0
0	0	0	0	0	0	0
0	0	0	0	0	0	0
0	0	0	0	0	0	0
0	0	0	0	0	0	0

0	0	0	0	0	0	0
0	0	0	0	0	0	0
0	0	0	0	0	0	0
0	0	0	0	0	0	0
0	0	0	0	0	0	0
0	0	0	0	0	0	0
0	0	0	0	0	0	0
0	0	0	0	0	0	0
0	0	0	0	0	0	0
0	0	0	0	0	0	0
0	0	0	0	0	0	0
0	0	0	0	0	0	0
0	0	0	0	0	0	0
0	0	98.118	0	0	0	0
0	0	4.738	0	0	0	0
0	0	0	0	0	0	0
0	0	0	0	0	0	0
0	0	0	0	0	0	0
0	0	0	0	0	0	0
0	0	0	0	0	0	0
0	0	0	0	0	0	0
0	0	18.394	0	0	0	0
0	0	0	0	0	0	0
0	0	0	0	0	0	0
0	0	0	0	0	0	0
0	0	0	0	0	0	0
0	0	0	0	0	0	0
0	0	0	0	0	0	0
0	0	0	0	0	0	0
0	0	0	0	0	0	0
0	0	0	0	0	0	0
0	0	0	0	0	0	0
0	0	0	23.212	0	0	0
0	0	0	0	0	0	0
0	0	0	0	0	0	0
0	0	0	0	0	0	0
0	0	0	0	0	0	0
0	0	0	0	0	0	0
0	0	0	0	0	0	0
0	0	0	0	0	0	0
0	0	0	0	0	0	0
0	0	0	0	0	0	0
0	0	0	0	0	0	0
0	0	0	0	0	0	0
0	0	0	0	0	0	0
0	0	0	0	0	0	0
0	0	0	0	0	0	0
0	0	0	0	0	0	0
0	0	0	0	0	0	0
0	0	0	0	0	0	0

0	0	0	0	0	0	0
0	0	0	0	0	0	0
0	0	4.75	0	0	0	0
0	0	0	0	0	0	5.838
0	0	0	0	0	0	0
0	0	0	0	0	0	0
0	0	0	0	0	0	7.151
0	0	0	0	0	0	0
0	0	0	0	0	0	0
0	0	0	0	0	0	0
0	0	0	0	0	0	0
0	0	0	0	0	0	0
0	0	0	0	0	0	0
0	0	0	0	0	0	0
0	0	0	0	0	0	0
0	0	0	0	0	0	0
0	0	0	0	0	0	0
0	0	0	0	0	0	0
0	0	0	0	0	0	0
0	0	0	5.738	0	0	0
0	0	0	0	0	0	0
0	0	0	0	0	0	0
0	0	0	0	0	0	0
0	0	0	0	0	0	0
0	0	0	0	0	0	0
0	0	0	0	0	0	0
0	0	0	0	0	0	0
0	0	0	0	0	0	0
0	0	0	0	0	0	0
0	0	0	0	0	0	0
0	0	0	0	0	0	0
0	0	0	0	0	0	0
0	0	0	0	0	0	0
0	0	0	0	0	0	0
0	0	0	0	0	0	0
0	0	0	0	0	0	0
0	0	0	0	0	0	0
0	0	0	0	0	0	0
0	0	0	0	0	0	0
0	0	0	0	0	0	0
0	0	0	0	0	0	0
0	0	0	0	0	0	0
0	0	0	0	0	0	0
0	0	0	0	0	0	0
0	0	0	0	0	0	0
0	0	0	0	0	0	0
0	0	0	0	0	0	0
0	0	0	0	0	0	0
0	0	0	0	0	0	0
0	0	0	0	0	0	0

0	0	0	0	0	0	0
0	0	0	0	0	0	0
0	0	0	0	0	0	0
0	0	0	0	0	0	0
0	0	0	0	0	0	0
0	0	0	0	0	0	0
0	0	0	0	0	0	0
0	0	0	0	0	0	0
0	0	0	0	0	0	0
0	0	0	0	0	0	0
0	0	0	0	0	0	0
0	0	0	0	0	0	0
0	0	0	0	0	0	0
0	0	0	0	0	0	0
0	0	0	0	0	0	0
0	0	0	0	0	0	0
0	0	0	0	0	0	0
0	0	0	0	0	0	0
0	0	0	0	0	0	0
0	0	0	0	0	0	0
0	0	0	0	0	0	0
0	0	0	0	0	0	0
0	0	0	0	0	0	0
0	0	0	0	0	0	0
0	0	0	0	0	0	0
0	0	0	0	0	0	0
0	0	0	0	0	0	0
0	0	0	0	0	0	0
0	0	0	0	0	0	0
0	0	0	0	0	0	0
0	0	0	0	0	0	0
0	0	0	0	0	0	0
0	0	0	0	0	0	0
0	0	0	0	0	0	0
0	0	0	0	0	0	0
0	0	0	0	0	0	0
0	0	0	0	0	0	0
0	0	0	0	0	0	0
0	0	0	0	0	0	0
0	0	0	0	0	0	0
0	0	0	0	0	0	0
0	0	0	0	0	0	0
0	0	0	0	0	0	0
0	0	0	0	0	0	0
0	0	0	0	0	0	0
0	0	0	0	0	0	0
0	0	0	0	0	0	0
0	0	0	0	0	0	0
0	0	0	0	0	0	0
0	0	0	0	0	0	0

0	0	0	0	0	0	0
0	0	0	0	0	0	0
0	0	0	0	0	0	0
0	0	0	0	0	0	0
0	0	0	0	0	0	0
0	0	0	0	0	0	0
0	0	0	0	0	0	0
0	0	0	0	0	0	0
0	0	0	0	0	0	0
0	0	0	0	0	0	0
0	0	0	0	0	0	0
0	0	0	0	0	0	0
0	0	0	0	0	0	0
0	0	0	0	0	0	5.845
0	0	0	0	0	0	0
0	0	0	0	0	0	0
0	0	0	0	0	0	0
0	0	0	0	0	0	0
0	0	0	0	0	0	0
0	0	0	0	0	0	0
0	0	0	0	0	0	0
0	0	0	0	0	0	0
0	0	0	0	0	0	0
0	0	0	0	0	0	0
0	0	0	0	0	0	0
0	0	0	0	0	0	0
0	0	0	0	0	0	0
0	0	0	0	0	0	0

G(Cl..Br)	G(Cl..I)	G(Br..Br)	G(Br..I)	G(I..I)	RDF010u	RDF015u
0	0	0	0	0	8.045	9.147
0	0	0	0	0	14.193	13.86
0	0	0	0	0	7.823	10.909
0	0	0	0	0	5.265	3.232
0	0	0	0	0	6.843	5.9
0	0	0	0	0	9.137	7.44
0	0	0	0	0	14.671	17.876
0	0	0	0	0	2.89	2.555
0	0	0	0	0	6.443	8.829
0	0	0	0	0	11.808	10.396
0	0	0	0	0	27.601	31.944
0	0	0	0	6.034	13.405	16.548
0	0	0	0	0	10.974	12.827
0	0	0	0	0	13.032	13.432
0	0	0	0	0	11.284	13.198
0	0	0	0	0	10.914	13.031
0	0	0	0	0	5.842	5.041
0	0	0	0	0	10.866	18.911
0	0	0	0	0	12.065	10.508
0	0	0	0	0	11.109	14.764
0	0	0	0	0	9.166	10.823
0	0	0	0	0	3.831	4.858
0	0	0	0	0	6.381	7.735
0	0	0	0	0	9.375	11.986
0	0	0	0	0	15.95	17.151
0	0	0	0	0	14.526	23.996
0	0	0	0	0	14.23	15.147
0	0	0	0	0	19.497	22.348
0	0	0	0	0	11.056	11.436
0	0	0	0	0	15.119	14.529
0	0	0	0	0	14.729	14.164
0	0	0	0	0	19.994	31.803
0	0	0	0	0	11.561	11.856
0	0	0	0	0	13.183	14.129
0	0	0	0	0	19.308	30.447
0	0	0	0	0	14.06	19.142
0	0	0	0	0	14.052	20.476
0	0	0	0	0	4.549	4.398
0	0	0	0	0	21.259	25.938
0	0	0	0	0	8.085	13.697
0	0	0	0	0	10.736	12.529
0	0	0	0	0	7.112	8.075
0	0	0	0	0	22.453	35.348
0	0	0	0	0	6.767	7.954
0	0	0	0	0	13.941	14.486
0	0	0	0	0	8.686	11.049
0	0	0	0	0	10.391	12.136
0	0	0	0	0	9.825	13.023
0	0	0	0	0	7.265	10.538

0	0	0	0	0	12.201	17.186
0	0	0	0	0	9.357	12.01
0	0	0	0	0	14.223	20.413
0	0	0	0	0	9.548	12.864
0	0	0	0	0	9.71	12.138
0	0	0	0	0	11.445	13.053
0	0	0	0	0	11.543	16.997
0	0	0	0	0	12.188	16.082
0	0	0	0	0	7.838	10.056
0	0	0	0	0	10.468	13.634
0	0	0	0	0	9.265	12.427
0	0	0	0	0	7.908	9.911
0	0	0	0	0	10.021	11.926
0	0	0	0	0	8.727	12.625
0	0	0	0	0	8.331	11.719
0	0	0	0	0	10.746	14.453
0	0	0	0	0	14.051	17.554
0	0	0	0	0	12.329	15.497
0	0	0	0	0	4.324	4.391
0	0	0	0	0	9.112	9.183
0	0	0	0	0	9.029	9.401
0	0	0	0	0	7.06	5.592
0	0	0	0	0	8.667	10.082
0	0	0	0	0	7.339	8.887
0	0	0	0	0	6.203	8.182
0	0	0	0	0	8.765	5.752
0	0	0	0	0	8.834	10.904
0	0	0	0	0	11.241	13.5
0	0	0	0	0	5.084	8.586
0	0	0	0	0	9.271	14.82
0	0	0	0	0	16.516	19.46
0	0	0	0	0	5.688	5.242
0	0	0	0	0	8.169	10.456
0	0	0	0	0	5.189	5.045
0	0	0	0	0	9.498	11.823
0	0	0	0	0	7.22	8.353
0	0	0	0	0	7.874	4.999
0	0	0	0	0	10.993	11.276
0	0	0	0	0	14.526	24.243
0	0	0	0	0	6.454	8.398
0	0	0	0	0	6.405	5.967
0	0	0	0	0	8.778	14.314
0	0	0	0	0	6.432	8.249
0	0	0	0	0	4.967	5.887
0	0	0	0	0	16.121	24.895
0	0	0	0	0	30.823	47.488
0	0	0	0	0	12.25	13.662
0	0	0	0	0	10.166	9.822
0	0	0	0	0	14.592	14.917
0	0	0	0	0	26.212	40.557

0	0	0	0	0	13.609	12.527
0	0	0	0	0	12.21	14.929
0	0	0	0	0	14.152	19.526
0	0	0	0	0	8.008	9.444
0	0	0	0	0	12.675	11.244
0	0	0	0	0	10.524	12.96
0	0	0	0	0	15.934	26.703
0	0	0	0	0	10.508	5.184
0	0	0	0	0	7.446	8.295
0	0	0	0	0	7.435	6.39
0	0	0	0	0	13.291	14.604
0	0	0	0	0	6.546	7.481
0	0	0	0	0	17.138	23.261
0	0	0	0	0	10.17	15.544
0	0	0	0	0	6.368	8.147
0	0	0	0	0	4.686	8.359
0	0	0	0	0	6.577	9.274
0	0	0	0	0	2.373	1.192
0	0	0	0	0	12.595	17.522
0	0	0	0	0	6.908	7.342
0	0	0	0	0	2.704	1.64
0	0	0	0	0	22.06	29.788
0	0	0	0	0	7.175	7.014
0	0	0	0	0	21.979	31.979
0	0	0	0	0	9.122	9.37
0	0	0	0	0	10.124	14.934
0	0	0	0	0	8.184	7.418
0	0	0	0	0	10.499	9.943
0	0	0	0	0	23.629	26.935
0	0	0	0	0	16.89	18.972
0	0	0	0	0	14.459	15.539
0	0	0	0	0	11.297	15.371
0	0	0	0	0	8.715	11.663
0	0	0	0	0	5.6	6.047
0	0	0	0	0	9.082	16.945
0	0	0	0	0	8.802	8.218
0	0	0	0	0	15.81	17.666
0	0	0	0	0	8.17	9.81
0	0	0	0	0	7.807	9.495
0	0	0	0	0	13.597	17.888
0	0	0	0	0	3.593	8.879
0	0	0	0	0	4.473	8.515
0	0	0	0	0	11.982	13.509
0	0	0	0	0	7.08	8.387
0	0	0	0	0	7.445	8.238
0	0	0	0	0	6.456	5.661
0	0	0	0	0	7.192	8.699
0	0	0	0	0	6.033	8.057
0	0	0	0	0	7.188	7.448
0	0	0	0	0	9.532	15.722

0	0	0	0	0	10.514	9.583
0	0	0	0	0	9.634	13.05
0	0	0	0	0	9.634	14.015
0	0	0	0	0	5.958	8.127
0	0	0	0	0	11.813	13.205
0	0	0	0	0	7.124	7.463
0	0	0	0	0	9.897	9.489
0	0	0	0	0	9.767	11.634
0	0	0	0	0	9.029	12.534
0	0	0	0	0	10.514	11.392
0	0	0	0	0	2.414	3.419
0	0	0	0	0	7.239	2.594
0	0	0	0	0	12.849	13.415
0	0	0	0	0	10.099	12.678
0	0	0	0	0	13.484	13.393
0	0	0	0	0	14.971	23.266
0	0	0	0	0	11.368	10.314
0	0	0	0	0	12.45	11.492
0	0	0	0	0	4.555	4.845
0	0	0	0	0	6.443	9.15
0	0	0	0	0	18.169	21.632
0	0	0	0	0	9.686	16.604
0	0	0	0	0	11.858	20.251
0	0	0	0	0	11.3	11.029
0	0	0	0	0	14.161	15.283
0	0	0	0	0	11.235	15.028
0	0	0	0	0	6.055	7.571
0	0	0	0	0	12.355	20.833
0	0	0	0	0	10.302	18.11
0	0	0	0	0	7.188	7.146
0	0	0	0	0	8.792	6.738
0	0	0	0	0	12.153	18.551
0	0	0	0	0	8.842	10.354
0	0	0	0	0	6.484	5.946
0	0	0	0	0	9.731	11.272
0	0	0	0	0	3.407	5.179
0	0	0	0	0	7.591	5.382
0	0	0	0	0	9.271	12.971
0	0	0	0	0	10.631	11.923
0	0	0	0	0	19.3	22.254
0	0	0	0	0	9.532	15.682
0	0	0	0	0	9.251	8.503
0	0	0	0	0	8.15	10.699
0	0	0	0	0	9.833	14.281
0	0	0	0	0	6.485	8.128
0	0	0	0	0	9.972	18.111
0	0	0	0	0	10.112	10.186
0	0	0	0	0	26.718	39.451
0	0	0	0	0	8.978	8.702
0	0	0	0	0	6.593	4.752

0	0	0	0	0	11.534	14.928
0	0	0	0	0	6.721	6.615
0	0	0	0	0	9.743	9.988
0	0	0	0	0	7.122	7.139
0	0	0	0	0	10.777	10.028
0	0	0	0	0	15.709	18.374
0	0	0	0	0	10.408	13.284
0	0	0	0	0	9.984	11.426
0	0	0	0	0	7.105	9.066
0	0	0	0	0	11.595	9.909
0	0	0	0	0	18.097	23.313
0	0	0	0	0	10.701	12.108
0	0	0	0	0	12.753	22.597
0	0	0	0	0	14.783	21.699
0	0	0	0	0	11.088	9.492
0	0	0	0	0	10.175	9.36
0	0	0	0	0	13.763	13.888
0	0	0	0	0	10.918	10.215
0	0	0	0	0	8.707	7.34
0	0	0	0	0	10.918	10.218
0	0	0	0	0	7.989	6.054
0	0	0	0	0	12.234	14.694
0	0	0	0	0	11.788	15.386
0	0	0	0	0	11.788	15.403
0	0	0	0	0	10.99	9.851
0	0	0	0	0	17.534	19.45
0	0	0	0	0	3.41	4.085
0	0	0	0	0	7.483	6.752
0	0	0	0	0	12.267	19.101
0	0	0	0	0	9.416	10.211
0	0	0	0	0	24.29	36.26
0	0	0	0	0	12.293	13.224
0	0	0	0	0	3.913	2.903
0	0	0	0	0	10.219	16
0	0	0	0	0	14.071	17.807
0	0	0	0	0	10.695	9.244
0	0	0	0	0	14.016	16.405
0	0	0	0	0	6.726	5.177
0	0	0	0	0	8.542	6.432
0	0	0	0	0	10.155	10.187
0	0	0	0	0	7.533	6.081
0	0	0	0	0	8.687	11.211
0	0	0	0	0	11.896	12.604
0	0	0	0	0	10.61	10.782
0	0	0	0	0	6.39	6.957
0	0	0	0	0	14.518	13.063
0	0	0	0	0	12.972	11.364
0	0	0	0	0	6.906	9.006
0	0	0	0	0	8.235	7.261
0	0	0	0	0	6.317	6.337

0	0	0	0	0	12.235	15.232
0	0	0	0	0	10.603	8.962
0	0	0	0	0	12.355	20.354
0	0	0	0	0	14.152	20.306
0	0	0	0	0	4.129	3.5
0	0	0	0	0	8.968	8.726
0	0	0	0	0	6.723	8.521
0	0	0	0	0	11.676	13.339
0	0	0	0	0	13.283	12.005
0	0	0	0	0	10.619	9.828
0	0	0	0	0	9.285	7.475
0	0	0	0	0	11.903	13.027
0	0	0	0	0	10.455	13.29
0	0	0	0	0	5.916	8.852
0	0	0	0	0	10.131	7.272
0	0	0	0	0	13.8	14.856
0	0	0	0	0	7.583	6.565
0	0	0	0	0	14.506	16.708
0	0	0	0	0	25.383	38.575
0	0	0	0	0	17.557	18.678
0	0	0	0	0	25.248	41.234
0	0	0	0	0	11.988	18.556
0	0	0	0	0	8.333	9.948
0	0	0	0	0	7.418	8.877
0	0	0	0	0	7.719	9.051
0	0	0	0	0	10.317	15.314
0	0	0	0	0	9.93	10.341
0	0	0	0	0	7.114	8.533

RDF020u	RDF025u	RDF030u	RDF035u	RDF040u	RDF045u	RDF050u
4.812	23.428	12.213	7.121	11.727	17.573	7.515
12.879	48.912	16.049	10.935	21.809	25.584	32.87
5.317	32.616	11.495	14.762	14.092	21.164	29.331
4.995	10.445	4.34	4.037	5.02	6.554	5.052
10.05	16.128	5.59	8.243	11.714	13.77	8.217
4.564	18.336	18.096	8.297	11.171	12.174	12.774
15.011	60.512	29.884	30.961	43.396	45.245	51.26
3.092	4.589	2.388	3.238	2.095	6.631	1.041
4.717	27.553	12.662	13.552	18.878	11.233	17.148
11.398	35.273	11.573	9.98	17.897	21.595	24.248
33.441	103.757	34.963	40.123	66.128	76.13	81.619
9.928	55.411	28.325	18.757	21.771	38.386	25.372
10.131	38.236	14.073	19.25	20.798	28.099	28.964
13.712	38.813	15.221	14.04	33.65	33.594	33.102
10.419	35.873	22.813	23.328	26.73	29.081	28.611
9.064	34.789	15.933	18.506	21.1	21.271	21.508
4.181	14.648	7.997	7.831	9.824	8.205	10.479
11.077	44.858	15.927	18.167	34.438	38.813	47.812
11.376	33.463	9.552	9.893	15.93	24.524	22.517
9.378	41.031	15.421	24.095	28.477	26.766	21.283
5.139	32.262	23.329	13.095	13.723	21.135	27.839
4.19	7.951	5.519	8.277	11.216	10.52	12.81
3.872	22.762	6.787	13.688	15.071	13.189	17.672
7.086	30.214	18.696	17.589	15.18	23.67	20.806
14.031	59.952	25.281	26.525	38.463	46.219	39.3
13.315	74.159	33.664	50.35	53.554	58.364	47.709
12.44	42.545	16.803	11.135	23.704	26.555	26.361
15.815	39.977	28.897	31.515	47.038	48.846	47.915
8.371	32.626	18.839	16.054	22.626	20.623	20.25
14.797	46.768	14.623	11.996	22.633	35.216	26.071
14.049	46.186	11.606	29.467	31.67	38.376	52.371
16.771	81.431	58.844	49.306	47.086	69.566	91.362
7.141	39.23	15.828	14.679	16.284	17.951	18.069
9.379	53.816	25.959	15.508	31.088	30.469	28.243
17.028	86.254	45.633	61.156	73.38	78.435	73.826
9.563	62.356	28.442	23.265	37.187	37.482	33.019
9.756	71.487	26.965	30.035	39.57	32.023	57.657
4.104	11.491	9.217	9.364	8.459	8.737	5.838
15.366	90.998	43.17	52.406	48.092	57.641	66.56
6.853	33.25	19.439	15.642	11.909	14.841	19.988
11.132	38.837	17.605	22.514	28.377	31.551	40.579
8.325	17.498	9.578	11.85	14.055	21.83	11.146
16.441	120.148	72.501	89.023	83.18	89.869	111.357
4.261	17.919	7.626	11.332	10.42	15.086	13.76
16.42	38.057	13.156	12.072	24.652	31.666	28.396
8.391	27.527	9.416	12.88	12.383	16.246	19.905
11.573	28.604	13.53	14.648	15.323	21.312	26.695
10.792	32.237	15.259	15.268	21.808	21.576	27.79
8.543	23.803	15.447	13.392	14.584	17.748	22.8

13.242	41.47	26.362	26.457	24.167	32.481	37.85
10.371	30.325	13.519	13.33	14.679	17.905	26.715
13.353	51.635	31.644	24.133	33.969	36.317	48.547
12.069	29.597	13.313	13.916	15.516	19.954	28.38
12.677	31.964	14.52	15.913	25.758	30.567	30.437
10.799	32.984	16.245	14.198	17.855	24.386	29.833
11.417	42.306	23.608	23.501	23.846	36.579	37.718
10.994	44.73	17.323	21.859	22.94	23.66	39.917
8.892	24.4	10.736	10.255	11.904	19.216	22.359
11.646	33.266	14.19	19.666	20.247	21.104	33.173
11.61	24.988	12.255	10.361	16.687	20.614	26.162
2.955	28.271	13.958	16.126	13.951	19.78	18.219
11.041	30.253	12.74	13.288	15.665	19.538	24.924
8.673	34.041	14.146	17.766	11.518	24.681	24.445
9.733	27.194	14.773	13.256	15.328	19.542	23.345
11.645	33.375	21.341	18.897	18.542	23.937	26.586
12.433	72.043	31.395	36.857	38.511	33.511	41.646
12.337	47.765	13.244	15.199	44.103	26.151	36.261
3.947	14.924	5.906	4.325	13.573	9.754	7.902
7.128	30.339	13.06	11.581	19.635	20.9	27.672
7.554	35.69	14.589	9.511	20.102	18.66	20.94
5.033	26.251	8.298	6.516	13.553	10.613	14.033
8.252	30.56	11.23	11.304	22.435	15.879	21.845
3.709	21.042	7.824	10.133	13.144	16.33	19.96
5.3	14.065	9.307	10.514	11.056	12.582	10.387
7.977	15.975	9.444	7.725	14.104	13.01	12.562
9.62	29.831	11.217	12.525	15.191	22.202	18.215
8.702	44.683	13.649	11.617	20.514	24.028	29.676
4.627	21.66	5.644	9.66	9.121	11.991	16.142
7.584	39.339	5.583	14.796	19.377	16.907	21.684
12.559	65.034	38.579	20.272	34.806	42.901	46.863
4.31	15.001	4.496	13.381	12.092	9.124	9.008
8.641	21.983	9.151	16.91	19.789	19.728	23.474
4.129	7.922	10.177	5.192	7.862	10.563	10.377
9.606	38.315	11.999	21.768	21.095	34.893	18.474
7.088	24.857	15.914	10.573	16.661	12.567	16.031
5.104	20.972	3.51	6.917	10.863	8.977	9.321
9.049	40.604	16.282	13.441	19.808	24.259	28.005
12.329	78.413	34.947	52.707	49.339	54.636	47.163
5.336	26.554	9.806	10.233	13.743	15.589	22.312
5.741	17.305	9.029	14.119	12.653	21.913	12.431
7.125	29.235	21.387	23.593	22.14	29.355	27.167
7.756	15.141	11.964	13.632	11.511	13.805	11.816
3.504	20.026	3.844	5.926	6.534	9.228	11.648
13.016	95.617	49.185	59.635	61.343	56.355	62.203
30.372	159.951	76.089	86.421	97.885	106.132	116.579
11.181	38.321	14.905	17.995	38.249	38.569	39.73
8.597	33.133	8.141	16.024	12.632	25.928	16.897
13.641	56.17	32.461	39.732	43.127	38.175	39.571
26.436	126.132	63.605	84.672	96.09	122.842	119.053

12.068	39.971	21.199	18.633	30.427	36.395	22.045
9.365	48.154	21.128	21.564	32.515	35.029	34.031
14.876	60.618	27.151	36.492	33.014	37.53	51.89
6.549	34.941	12.739	12.677	20.005	15.835	26.445
13.073	40.256	15.674	14.28	17.348	23.035	16.331
7.907	35.289	20.494	19.331	18.123	25.201	19.322
21.821	57.245	24.203	25.482	57.16	52.478	81.196
7.012	27.175	11.576	7.177	11.4	18.99	18.372
4.707	29.474	6.197	5.45	7.876	5.932	23.237
4.339	20.525	4.963	14.538	9.996	15.709	12.902
8.821	53.272	19.048	21.335	36.009	37.473	33.522
5.509	23.146	8.069	6.917	12.572	13.822	13.275
10.927	84.813	37.875	47.845	50.8	48.058	57.136
11.825	45.506	9.708	8.436	26.568	20.222	35.935
6.701	21.269	7.182	23.205	16.86	22.448	28.233
2.693	18.487	8.886	14.437	5.993	11.175	11.14
5.65	25.538	14.545	15.103	15.213	17.333	18.909
4.347	4.428	0.029	4.293	2.956	1.707	0.424
12.248	59.664	17.389	13.722	34.448	28.642	40.448
4.042	24.194	7.048	10.381	12.175	13.827	14.12
1.057	2.582	2.43	1.516	1.82	0.091	0.1
13	110.455	62.111	31.935	40.585	56.417	83.176
4.158	21.443	4.723	5.769	15.848	10.066	18.256
18.589	100.779	59.819	70.62	83.529	90.667	88.348
9.036	41.019	15.953	22.465	19.854	8.526	10.035
14.867	35.819	13.32	27.78	22.389	29.676	32.517
11.823	19.483	4.382	10.205	11.261	15.589	10.148
6.945	36.023	15.12	12.048	24.434	17.206	24.282
25.284	88.608	39.925	31.782	59.749	62.057	62.344
12.012	71.708	32.868	25.883	40.383	34.395	30.208
12.293	65.101	18.068	17.44	33.234	30.323	32.96
11.268	43.894	20.372	32.427	24.392	28.672	21.831
7.207	30.194	6.881	10.744	16.895	23.467	29.852
3.323	15.596	7.396	4.536	14.45	11.741	8.958
7.507	47.025	23.084	19.742	27.931	24.935	34.827
4.309	31.108	13.403	13.069	13.593	19.191	12.471
16.419	61.003	12.019	15.678	35.449	30.196	43.638
7.604	21.445	9.811	20.857	20.287	21.027	25.437
5.623	23.702	17.187	11.81	12.419	15.749	16.721
7.529	55.782	28.156	18.632	28.467	27.026	41.341
4.514	15.881	11.571	9.478	7.291	10.665	3.902
4.931	14.98	11.073	8.59	7.618	6.548	2.329
10.239	36.907	15.276	17.973	29.426	34.182	38.183
5.076	25.26	6.852	11.538	11.837	10.939	15.853
4.549	25.682	6.976	13.754	13.617	14.409	20.874
6.603	15.266	6.263	7.639	10.545	10.367	4.514
5.936	21.314	8.431	12.573	13.641	18.409	17.47
3.966	23.331	6.771	19.961	10.174	11.956	14.916
4.433	16.763	20.588	8.176	16.415	16.433	5.868
9.164	43.898	14.095	14.194	22.583	22.855	29.714

8.715	36.641	16.849	11.841	19.317	27.676	23.52
9.723	34.542	15.504	10.171	18.122	21.455	17.973
8.558	43.279	11.025	13.403	20.715	19.562	25.211
6.121	20.93	10.808	9.837	10.231	16.691	13.969
11.858	44.106	18.771	16.845	27.139	25.19	29.224
5.899	25.055	11.281	11.657	15.758	14.032	18.469
7.093	34.782	16.622	9.054	16.143	25.858	18.946
9.211	38.604	14.495	17.309	26.054	27.5	27.883
8.768	35.925	12.539	11.136	22.747	24.616	21.466
7.827	40.618	18.409	12.785	23.349	28.787	18.589
2.984	5.278	3.366	3.197	3.296	2.713	2.196
6.946	9.766	3.933	7.179	8.091	8.899	2.086
8.913	48.552	24.519	33.855	37.536	37.447	53.16
11.848	29.511	17.214	19.032	22.039	27.06	25.842
12.359	45.571	15.224	18.597	22.808	28.369	32.762
12.208	68.899	36.987	42.58	46.451	52.564	54.367
11.134	40.112	11.052	8.857	25.436	22.884	21.979
12.724	36.868	14.168	7.544	20.219	27.616	23.392
5.15	9.672	5.859	6.355	7.589	8.006	5.041
4.375	23.424	16.595	13.882	15.455	21.724	14.862
10.923	62.247	23.537	39.072	41.695	51.099	60.734
11.06	38.614	23.549	19.819	31.934	28.896	25.081
10.461	49.506	20.813	25.78	25.861	32.301	38.737
8.15	33.466	15.581	16.102	21.179	25.249	26.171
13.136	53.097	17.799	16.775	24.83	21.114	32.267
10.137	40.055	21.621	22.081	21.139	28.998	34.26
3.903	23.14	6.819	7.122	11.53	10.814	16.338
11.253	55.768	28.101	27.614	37.605	35.727	46.886
9.78	49.964	23.827	26.592	33.517	28.975	40.004
6.81	21.449	4.767	10.791	15.724	15.641	15.088
8.564	23.373	14.016	11.777	6.735	16.415	7.758
11.637	53.924	27.754	22.2	28.663	37.328	52.892
9.247	30.203	8.659	16.719	20.253	26.316	31.124
4.03	21.568	11.911	13.383	11.428	13.323	16.991
9.079	33.307	10.643	14.119	25.962	27.882	34.768
2.862	9.579	7.722	6.929	3.756	4.14	6.501
7.634	15.873	7.398	5.564	6.467	11.409	9.887
8.609	40.095	10.095	12.066	20.134	19.919	26.574
10.315	35.403	13.946	15.013	18.9	25.206	23.902
19.807	67.462	22.635	41.728	49.753	52.627	59.12
9.23	43.78	14.033	14.216	22.685	23.393	28.968
8.904	23.486	15.381	15.537	18.552	24.325	14.217
5.97	27.989	17.44	14.419	13.86	15.787	12.354
7.655	31.96	20.047	23.989	20.584	26.422	26.983
6.39	25.848	8.813	9.045	13.728	12.435	18.194
9.795	49.26	20.597	20.645	33.27	30.526	39.006
5.209	36.804	28.294	15.3	13.382	29.875	32.396
20.292	161.196	69.121	78.236	109.295	107.548	123.286
7.457	40.883	23.791	28.534	17.254	20.111	24.987
6.75	15.452	2.265	4.516	10.739	11.139	8.046

8.478	61.652	20.604	27.176	40.643	27.405	37.725
8.097	25.181	10.175	22.943	13.85	12.604	19.356
4.645	37.101	25.861	16.522	12.126	27.449	32.106
7.66	21.929	6.296	10.493	12.546	13.317	20.188
9.671	28.922	10.708	7.661	12.717	20.281	23.636
13.934	70.324	26.874	23.248	37.528	39.329	53.463
10.079	39.083	17.142	16.511	24.586	28.156	19.86
4.528	28.751	13.896	16.039	22.022	20.787	27.025
4.397	17.55	12.096	12.264	14.784	17.276	8.809
11.226	32.174	10.3	8.526	16.461	21.843	21.519
16.473	76.513	42.166	32.934	50.156	44.126	63.423
12.499	32.432	5.269	19.597	29.927	24.213	29.774
9.227	54.456	35.868	45.557	41.944	43.467	38.829
8.748	65.301	41.251	43.582	44.552	50.248	48.066
9.38	39.035	9.352	9.431	20.316	20.437	20.377
8.203	36.958	7.808	12.306	17.649	20.041	20.132
11.741	48.089	19.324	6.937	23.438	19.474	40.123
10.357	35.688	7.763	9.538	16.303	18.845	22.222
3.714	28.826	10.846	17.869	16.808	24.739	20.362
10.356	35.694	7.767	9.544	16.33	18.846	22.202
5.173	22.593	8.457	10.387	14.392	13.514	17.925
14.28	44.726	14.991	18.824	30.347	34.337	26.857
14.342	49.526	12.35	29.262	26.998	30.597	34.327
15.328	51.312	13.53	31.907	25.127	31.165	30.562
8.945	26.164	15.088	11.056	20.223	17.367	15.077
13.542	72.749	27.029	26.288	36.782	50.21	64.961
2.933	8.805	1.237	4.111	7.433	6.076	6.744
4.698	16.638	17.366	10.594	14.75	18.032	11.938
8.734	58.286	21.764	20.594	32.391	29.968	37.23
8.039	23.193	11.488	12.397	13.888	23.129	15.113
23.899	132.227	64.22	72.848	99.252	97.799	106.987
8.59	50.547	21.616	13.988	31.771	29.806	29.424
3.026	10.886	1.656	3.413	1.769	4.418	4.998
8.501	40.94	15.224	26.894	28.319	28.731	23.558
12.37	54.091	21.443	22.878	39.718	34.828	41.841
8.6	32.131	14.654	11.613	14.594	20.898	16.567
13.097	62.467	25.757	31.861	41.311	49.033	44.615
4.784	17.302	7.706	9.439	12.073	10.77	9.997
5.178	24.489	7.589	12.296	13.446	18.758	13.127
5.305	33.53	23.882	21.002	14.09	29.685	34.608
5.339	17.978	7.265	13.094	15.042	12.084	10.204
5.59	25.044	15.283	16.145	15.266	24.117	22.878
8.647	40.144	17.037	16.438	23.663	29.595	25.277
7.767	24.261	16.034	16.919	12.021	22.769	11.92
3.445	22.969	6.572	10.467	10.69	13.188	15.873
14.43	47.093	15.673	22.33	41.769	51.639	53.591
11.65	38.858	15.269	11.009	16.243	18.552	17.477
6.316	27.676	10.772	10.435	14.336	17.272	23.997
7.074	20.721	11.698	13.566	22.314	20.013	17.918
5.513	22.851	10.769	11.797	13.616	14.188	15.311

14.141	35.286	14.695	21.759	32.882	28.956	28.864
8.179	33.23	15.325	11.634	15.527	14.042	17.697
10.703	55.924	35.679	44.855	44.397	41.976	34.2
14.345	61.655	22.988	32.894	32.011	39.047	59.238
3.793	8.802	6.117	9.523	6.964	7.465	5.906
8.157	38.907	22.011	23.162	19.796	20.875	22.066
4.936	24.218	9.804	9.319	13.737	15.804	17.069
11.639	46.774	13.597	13.521	26.257	13.87	27.171
14.584	40.368	11.588	7.209	23.931	23.242	31.231
10.246	33.9	18.507	24.817	24.685	16.379	16.071
5.943	34.723	13.429	6.766	14.242	11.726	19.348
10.138	52.903	17.811	18.286	31.651	25.876	34.778
10.962	42.206	11.033	13.662	29.023	22.964	27.316
4.452	22.71	16.047	14.279	15.884	17.318	16.197
13.29	20.414	4.489	13.088	18.36	24.776	22.583
14.394	47.781	12.439	16.1	41.082	36.65	36.158
4.846	30.469	17.293	4.579	8.929	8.655	16.257
12.44	62.222	27.584	30.865	38.469	36.342	43.171
22.151	153.851	63.445	86.189	99.323	105.71	110.433
22.434	54.023	32.777	30.895	49.57	43.623	59.301
32.066	109.553	67.413	87.324	118.779	137.32	125.152
9.002	54.087	28.114	25.56	24.454	36.621	36.59
6.291	30.539	10.914	12.645	16.175	20.103	25.87
4.046	26.636	10.595	16.24	10.585	17.896	17.357
8.321	19.301	11.097	12.838	10.885	14.257	9.87
8.697	39.861	18.682	13.41	27.899	26.569	19.952
6.604	28.586	11.75	17.743	19.841	20.968	16.866
4.602	17.533	9.776	14.832	16.995	14.972	15.438

RDF055u	RDF060u	RDF065u	RDF070u	RDF075u	RDF080u	RDF085u
11.775	14.369	7.884	5.788	7.236	9.183	5.23
8.663	21.666	22.28	15.499	8.121	10.991	27.5
14.577	20.229	23.171	17.109	17.543	15.116	6.372
1.177	3.699	2.3	3.023	0.022	0.498	0.791
5.904	7.023	2.077	7.119	4.069	0.84	3.756
6.609	2.8	6.109	1.795	2.08	0.36	1.312
36.098	38.676	39.426	35.857	27.379	27.284	19.386
3.639	0.845	0.706	0	0	0	0
8.756	17.532	9.688	11.89	4.816	10.912	2.28
9.052	19.475	12.664	9.717	7.326	7.29	12.221
56.642	82.827	98.89	94.253	72.557	69.589	63.197
30.498	34.908	28.58	23.869	25.652	22.719	22.561
25.462	23.463	20.52	14.719	14.825	10.33	10.488
26.619	38.744	35.966	39.261	13.739	24.06	21.549
22.306	22.882	25.326	19.13	15.161	9.804	10.585
20.796	14.908	14.635	14.239	10.776	16.112	6.363
6.323	5.571	4.26	1.716	3.293	1.605	2.147
36.7	47.367	43.458	40.907	24.066	29.257	32.807
7.567	9.031	14.458	11.717	3.714	7.049	16.926
17.202	20.445	17.549	12.763	14.56	14.71	19.214
21.911	26.157	21.03	17.528	20.738	12.147	8.847
9.079	9.864	4.925	14.456	0.944	7.375	0
7.196	14.094	3.793	4.702	4.363	7.829	3.319
20.243	8.614	13.285	11.882	11.31	10.097	7.477
40.101	39.215	46.758	40.642	49.465	26.674	33.214
36.968	37.737	29.999	24.759	22.771	17.139	11.844
12.631	11.278	14.564	16.195	7.582	9.413	20.213
61.281	49.916	63.842	38.212	65.405	57.012	65.58
16.342	24.242	22.072	18.948	20.338	20.027	10.157
14.288	19.526	27.788	18.305	14.091	12.015	23.909
32.653	57.559	47.158	45.848	50.627	41.318	43.009
66.578	72.821	76.284	85.153	72.595	63.608	62.338
17.758	15.966	13.351	14.322	13.253	10.663	14.045
23.818	27.434	27.417	27.465	19.668	20.151	16.577
66.385	57.217	71.288	53.012	51.025	46.418	34.185
19.385	18.936	33.363	21.324	19.609	22.662	26.771
27.349	31.729	28.922	21.071	21.219	13.127	13.665
10.855	7.228	6.817	2.366	2.011	0	0
35.704	40.773	51.232	51.34	41.288	35.205	36.453
14.511	14.761	17.241	15.015	16.535	13.571	12.495
31.71	34.017	26.791	31.844	17.608	23.101	24.565
15.659	13.086	5.321	6.562	1.109	4.691	0.027
65.075	64.727	84.567	61.608	51.024	45.208	51.523
8.622	14.065	7.066	6.808	10.461	4.067	7.407
20.74	40.488	36.852	32.634	25.247	25.391	31.505
17.987	16.433	13.228	8.14	6.716	10.715	12.093
19.603	14.581	16.334	10.396	12.763	13.656	12.591
19.148	21.386	17.825	19.3	13.302	16.551	18.03
10.34	17.08	11.379	13.396	4.686	14.382	14.985

20.96	23.834	20.81	21.585	17.705	16.949	13.614
15.519	19.705	13.742	13.209	10.211	12.779	15.824
31.454	41.235	45.714	30.871	22.113	30.846	26.897
16.007	20.19	19.449	12.622	16.111	18.1	12.947
24.188	28.581	22.298	23.389	24.012	20.585	8.157
16.236	18.59	22.962	13.229	12.29	11.223	12.391
16.824	21.336	25.165	26.638	18.562	15.267	21.578
26.775	29.921	29.103	23.211	20.343	24.841	23.417
14.351	18.129	13.958	11.837	11.696	9.828	10.096
20.882	27.221	21.236	19.215	16.556	22.175	20.193
14.203	19.158	15.186	14.379	14.954	14.808	11.473
14.306	12.535	17.297	16.03	15.132	14.328	9.702
11.599	21.479	21.402	11.191	10.516	17.374	12.461
15.927	12.465	20.34	17.634	17.344	12.506	9.972
8.841	12.342	16.099	12.384	12.832	19.914	10.328
18.984	13.625	25.69	15.098	11.858	13.109	12.221
28.449	34.035	30.704	28.717	25.823	20.244	17.904
25.216	31.774	22.221	24.398	24.598	19.277	21.024
3.581	3.244	5.633	3.427	2.921	0.718	1.831
18.796	26.056	19.955	17.313	9.534	11.23	8.174
20.278	21.974	17.921	19.141	16.701	7.863	12.827
7.931	3.706	12.338	9.972	9.103	6.359	7.154
8.401	20.602	15.522	20.483	16.689	10.339	12.793
9.861	11.866	10.412	8.641	5.638	8.889	4.369
7.82	8.961	9.066	6.267	4.662	2.661	4.315
7.689	3.939	7.091	4.804	8.72	4.052	7.501
12.183	12.308	17.406	13.033	13.413	7.852	13.776
14.426	30.076	17.989	16.717	14.585	11.88	10.928
10.499	13.941	9.151	4.93	5.078	1.368	1.109
15.669	23.437	20.97	16.128	10.616	9.686	14.688
31.456	30.088	43.13	31.334	31.002	19.817	21.657
4.168	8.426	2.41	4.945	1.143	0.453	0.529
19.965	19.944	17.875	17.722	20.49	14.364	10.852
5.687	3.793	6.657	2.922	3.462	0.804	0.001
18.723	16.917	24.323	13.932	15.242	18.092	15.188
6.979	14.641	4.317	5.55	0.897	0.543	0.854
3.411	3.276	11.815	6.041	9.486	3.482	4.899
25.586	22.613	17.943	13.147	17.913	9.672	9.064
37.332	44.755	28.47	21.699	23.661	14.574	9.858
6.128	15.613	11.103	9.802	7.135	7.828	2.592
9.735	5.818	5.716	7.007	1.648	4.559	0
26.994	21.269	35.02	26.926	21.031	22.057	18.447
12.909	8.89	5.264	2.602	5.208	3.61	1.973
1.547	7.316	9.863	5.884	2.248	0.718	4.477
39.705	47.757	33.209	25.136	25.519	14.284	19.968
86.405	103.726	81.971	81.297	70.444	72.973	61.023
33.026	44.249	36.534	38.538	31.528	36.664	24.899
17.994	18.984	22.623	19.443	11.988	13.645	7.757
33.036	38.613	30.215	26.402	28.878	17.795	18.16
110.331	132.087	139.006	125.006	129.327	106.844	107.973

25.525	22.608	22.989	21.551	27.769	23.059	38.605
12.819	20.558	25.982	26.793	24.005	25.725	13.611
29.453	35.212	33.32	31.704	19.766	18.073	16.713
7.45	15.056	10.301	7.772	12.216	6.374	9.206
8.194	12.478	14.655	7.599	6.272	8.719	12.923
26.615	26.012	20.159	16.131	22.423	9.674	7.098
36.252	80.737	59.617	65.435	44.137	43.183	51.208
10.775	11.658	7.676	12.003	12.915	7.075	7.791
0.504	7.958	13.87	10.475	9.295	4.512	6.688
15.581	16.457	13.087	9.57	11.102	5.775	3.938
26.07	25.662	31.482	18.337	14.415	22.647	20.484
6.501	13.563	10.139	11.985	10.335	10.222	9.101
33.571	37.663	33.835	35.541	26.814	19.449	22.669
5.176	16.631	26.506	15.368	12.176	11.075	29.072
9.64	16.612	9.755	8.85	5.815	2.937	5.106
7.022	5.827	5.681	5.84	1.808	6.317	3.101
8.546	11.5	15.201	16.249	11.254	8.529	8.135
0.013	0	0	0	0	0	0
23.229	30.013	35.89	26.863	30.676	23.877	28.769
4.178	6.139	9.399	10.091	3.952	3.48	5.242
0.129	0	0	0	0	0	0
42.891	40.45	66.702	54.987	50.405	48.411	28.249
5.297	10.275	13.504	10.342	8.052	3.242	4.449
69.486	72.684	69.842	83.829	49.189	49.852	60.396
4.92	4.886	1.455	0.946	0	0	0
15.667	22.703	23.868	21.91	11.936	12.582	5.543
10.729	11.576	7.571	4.783	7.438	3.032	3.177
7.134	13.023	19.127	13.059	15.753	9.665	12.023
36.563	55.929	55.318	42.534	34.929	44.179	41.243
31.646	17.362	36.406	30.98	19.225	26.03	27.189
15.744	16.753	34.727	31.773	22.02	19.579	22.98
13.126	11.627	11.536	18.316	12.911	14.598	15.433
15.615	31.754	21.699	25.124	18.622	10.483	17.122
4.378	4.735	5.877	3.791	6.545	1.584	3.431
21.322	24.158	12.316	10.55	3.707	2.425	2.438
10.67	9.094	13.067	6.47	5.53	5.17	5.898
17.683	28.244	37.482	27.243	12.614	19.316	39.083
20.337	14.704	20.271	22.527	17.101	9.316	9.802
8.388	8.576	8.179	10.577	7.293	6.542	7.212
30.236	35.556	41.82	38.946	30.22	37.878	27.459
6.146	3.878	4.577	3.519	1.623	1.697	0.843
4.08	2.57	2.596	1.427	0.064	0.828	0.009
30.011	41.305	35.367	22.08	15.947	15.144	14.595
4.716	5.229	15.425	10.034	5.151	3.791	8.598
8.958	17.087	10.475	13.035	9.561	8.427	6.465
4.726	10.897	7.339	3.772	2.703	3.211	4.144
9.839	13.758	6.839	8.624	7.726	10.26	9.719
15.349	11.626	7.675	10.249	6.917	3.961	3.767
10.351	4.211	3.33	1.483	0.824	0	0
21.293	33.122	22.22	22.958	14.955	13.073	15.779

22.424	15.839	16.126	15.77	12.418	11.041	6.308
9.866	9.28	16.916	19.114	4.985	5.84	13.391
14.766	28.045	19.277	22.151	13.503	8.413	13.796
9.032	14.672	12.143	13.137	6.472	7.314	1.105
24.324	35.04	25.497	34.305	19.46	24.164	17.966
6.182	9.943	14.665	9.789	5.881	7.111	10.653
14.245	10.547	14.553	9.121	7.314	12.629	7.76
14.686	25.287	19.11	16.332	10.927	11.293	4.608
12.217	16.751	16.066	19.563	8.357	12.933	3.416
19.107	15.547	16.642	18.136	8.057	7.319	4.975
0.881	0.001	0.002	0	0	0	0
4.75	1.362	3.103	0.327	0.604	0	0
32.063	37.524	28.527	25.845	12.767	6.406	6.372
23.264	22.016	19.214	18.234	29.185	24.232	19.136
22.994	25.539	24.263	17.548	17.895	17.549	29.174
34.436	44.465	32.716	20.183	22.42	18.66	11.81
5.252	12.873	20.406	12.782	4.222	5.027	15.643
12.288	9.992	12.427	10.593	5.461	4.288	20.111
5.091	1.576	3.347	0.001	0.004	0	0
14.966	18.395	11.853	11.403	6.465	10.416	5.151
37.868	61.569	58.249	42.488	51.587	43.084	47.132
22.853	21.393	11.696	10.345	4.542	1.404	1.726
25.644	29.614	25.353	28.071	20.206	18.897	20.494
21.165	26.263	31.216	24.173	19.308	18.585	11.542
6.834	15.12	19.877	13.12	16.276	11.182	20.259
26.414	21.732	29.835	22.512	24.525	30.511	26.198
8.297	16.77	9.28	6.402	3.073	1.482	2.276
35.888	31.539	26.338	23.564	12.14	12.099	9.728
25.98	26.688	18.85	16.925	7.056	7.675	4.096
6.262	11.441	5.666	8.393	1.314	5.278	4.156
14.485	10.495	7.943	12.719	13.363	10.845	7.689
24.424	32.977	38.43	22.316	28.755	19.388	19.42
26.089	34.726	22.367	23.299	8.78	7.491	8.523
7.058	12.571	11.174	13.241	7.547	8.233	5.162
21.743	46.418	24.969	32.657	11.115	10.483	10.917
3.074	1.468	5.991	2.892	2.853	4.934	2.474
1.53	3.87	5.196	2.133	1.947	1.271	1.908
18.7	28.476	20.126	11.592	8.974	5.896	11.969
20.555	18.845	17.449	14.629	16.404	11.584	7.552
31.638	46.878	39.888	35.788	27.161	25.62	29.364
21.453	33.931	21.015	21.55	15.366	12.855	16.001
14.212	14.962	11.454	10.193	7.957	12.085	6.237
21.903	14.164	17.105	10.223	10.783	7.308	4.636
26.376	16.664	26.179	16.206	34.043	17.964	15.794
6.516	14.735	8.605	9.738	6.764	7.101	1.15
20.813	27.282	17.517	19.205	6.9	8.742	4.999
18.611	23.186	20.87	23.245	13.575	14.776	10.223
80.39	96.223	108.141	62.713	85.366	62.115	64.678
12.383	10.806	12.691	2.363	6.077	0.003	4.468
2.061	8.399	3.184	9.005	0.186	2.86	5.936

18.18	29.206	11.272	17.176	5.565	5.036	0.891
7.998	12.245	6.92	3.353	4.295	1.527	0.399
16.892	22.25	18.4	18.829	14.217	17.065	9.814
8.729	14.149	4.876	7.114	5.257	2.267	1.413
9.412	11.18	9.841	12.895	7.755	6.133	12.63
33.314	41.532	51.758	42.128	44.03	32.167	41.628
15.74	20.831	19.159	22.182	16.765	17.611	14.142
29.664	28.445	27.413	21.102	15.589	14.592	6.146
9.24	7.432	8.786	6.617	4.972	4.1	6.844
8.062	12.536	10.918	12.027	4.695	5.282	15.668
35.847	38.83	56.654	41.531	59.401	27.265	32.056
13.343	36.844	21.586	13.89	10.917	17.422	24.184
27.91	30.54	25.013	22.169	8.779	14.235	12.504
32.212	28.888	37.931	19.453	19.729	18.403	13.296
4.215	6.024	14.883	8.261	3.866	4.542	12.413
11.253	10.833	6.976	12.62	9.765	9.91	9.546
4.735	15.458	24.929	24.376	21.38	9.945	22.637
7.91	15.362	12.945	6.77	7.449	6.501	14.812
6.327	20.717	6.99	14.076	3.802	6.24	2.099
7.933	15.38	12.933	6.775	7.424	6.51	14.82
12.066	9.369	14.473	3.037	1.209	0.587	1.669
22.565	27.895	23.533	26.943	23.446	29.209	11.333
21.126	29.131	20.677	28.332	12.181	18.896	12.826
20.317	25.854	21.91	20.941	15.281	15.98	18.599
8.67	9.03	9.678	5.361	15.568	8.866	10.072
50.419	59.498	48.455	56.123	41.242	35.914	33.348
2.563	4.852	2.007	1.253	1.834	1.941	3.006
8.508	8.19	3.463	1.117	0.269	0	0
18.337	23.894	29.292	21.686	20.633	24.28	18.302
9.414	15.271	17.734	16.702	13.84	17.116	13.603
86.418	86.322	78.692	58.494	57.193	61.034	58.731
16.883	23.865	24.96	29.346	12.376	15.338	12.171
0.153	2.271	0.325	0.739	0	0	0
23.556	20.953	12.537	11.312	14.062	10.63	11.327
32.048	45.359	33.075	39.239	28.832	30.395	20.635
15.763	17.414	12.448	16.252	11.072	7.9	8.728
31.224	39.179	34.532	42.981	26.174	22.374	23.359
8.804	8.024	10.093	9.321	4.522	6.642	1.424
7.633	13.058	14.751	8.122	11.808	5.986	7.386
18.384	24.084	16.853	24.081	19.022	11.85	14.826
9.765	13.356	11.439	12.767	6.439	7.157	5.12
18.983	13.676	25.389	13.886	16.311	11.332	9.081
7.525	22.353	25.179	21.003	12.231	15.717	23.24
11.898	13.545	19.602	13.942	13.826	16.583	9.552
5.531	7.832	10.385	7.808	6.655	3.558	4.944
32.603	59.23	46.654	38.656	30.256	26.78	18.8
9.352	19.116	17.737	12.474	11.413	10.92	12.309
7.784	17.877	11.848	10.931	8.502	9.083	2.584
15.033	16.461	10.969	10.288	7.634	1.135	2.253
10.192	13.774	10.097	6.351	6.394	8.485	5.828

14.708	40.18	27.336	15.419	18.77	16.632	28.371
10.423	12.763	10.415	8.738	7.505	5.875	4.733
30.217	18.4	25.237	20.448	16.666	9.107	10.283
31.427	41.41	32.213	35.148	22.639	20.868	17.848
8.158	7.167	3.008	1.246	1.901	0	0
7.659	13.165	8.177	5.671	3.947	1.048	0.067
7.538	10.365	9.601	7.241	4.986	5.728	4.017
9.253	12.959	18.207	16.043	17.692	7.285	14.982
5.977	20.812	16.118	13.203	9.564	7.197	20.474
15.659	14.363	17.246	14.036	11.839	15.336	12.606
9.185	5.282	17.277	10.446	10.348	8.346	7.93
17.809	27.735	19.591	22.17	12.406	11.239	7.02
12.493	17.96	17.921	15.928	15.896	16.203	9.703
13.559	16.322	9.591	11.774	6.595	9.489	4.048
27.835	18.888	14.052	16.244	11.748	9.74	8.481
12.945	29.704	38.306	27.165	17.369	14.494	28.894
1.032	4.947	8.829	0.791	4.392	0	3.838
31.708	36.168	36.114	35.938	30.438	24.629	26.696
86.22	101.28	96.999	75.705	79.342	70.426	64.693
31.389	53.786	48.73	34.12	29.461	30.724	29.182
115.795	132.479	139.333	121.394	110.114	100.211	86.57
33.783	30.537	34.669	33.385	28.679	18.475	13.944
16.914	20.418	15.963	21.494	16.876	11.931	7.494
9.485	15.198	13.744	18.521	12.325	11.64	6.616
11.762	13.989	6.094	6.242	4.739	9.306	6.106
16.979	17.911	17.544	15.262	13.165	14.533	14.38
16.412	22.288	23.526	20.238	21.932	21.281	13.581
12.461	15.83	6.647	8.073	5.299	7.441	2.764

RDF090u	RDF095u	RDF100u	RDF105u	RDF110u	RDF115u	RDF120u
4.259	2.711	1.841	0.035	1.786	0.119	0.153
11.433	12.421	11.199	21.354	18.973	5.271	7.232
11.682	3.445	5.212	0.99	2.24	0.613	0.021
0	0	0	0	0	0	0
4.139	2.762	1.117	1.915	0.096	0.215	0
0	0	0	0	0	0	0
19.335	20.075	16.426	13.38	19.63	11.525	8.807
0	0	0	0	0	0	0
2.3	0.349	0.124	0	0	0	0
8.972	9.41	9.076	10.465	6.84	4.466	3.415
44.224	35.695	33.702	26.441	18.137	11.423	8.53
28.254	21.382	26.475	24.33	30.874	21.896	16.303
8.112	6.749	2.671	3.054	0.824	0.944	0
22.794	8.772	11.785	8.377	10.506	4.575	3.153
6.598	3.684	0.684	0.18	0	0	0
13.798	16.045	11.606	12.553	7.576	2.502	2.104
0.829	0.859	0	0	0	0	0
23.426	13.726	14.507	9.064	7.002	7.167	5.365
5.858	10.434	5.094	10.004	8.904	3.07	2.651
10.172	12.479	8.713	4.82	3.333	2.211	1.857
9.626	7.056	5.844	5.185	1.843	1.002	0.004
2.915	0	0.872	0	0	0	0
4.885	4.17	5.886	9.036	3.317	2.257	2.529
10.515	9.765	4.761	5.425	8.58	7.18	3.639
32.233	17.94	19.969	10.283	10.307	3.993	7.146
8.32	12.518	3.403	5.528	4.346	1.48	0.372
10.97	16.68	13.449	14.094	15.129	7.17	7.37
58.073	41.973	38.704	41.173	33.229	13.556	18.319
12.757	11.12	14.191	4.039	4.457	2.691	1.358
13.014	16.21	9.273	19.861	15.633	13.48	7.849
30.159	33.266	29.939	26.908	24.646	14.562	16.654
42.16	38.537	41.602	28.526	29.51	24.221	26.899
13.158	11.161	9.291	7.618	5.592	2.384	1.209
16.007	6.636	6.386	1.569	0.766	0.021	0
29.486	23.173	17.15	15.822	9.348	3.851	1.032
16.666	20.827	16.69	15.741	17.051	13.78	12.234
9.837	6.543	7.301	4.974	0.32	0.396	0.199
0	0	0	0	0	0	0
44.447	30.584	27.305	16.271	21.595	16.315	17.126
7.699	11.216	9.921	5.847	7.618	2.674	6.692
18.84	16.193	18.62	15.001	7.841	10.244	7.138
0.69	0	0	0	0	0	0
55.963	33.381	36.052	36.291	35.722	21.059	17.459
3.037	3.351	4.565	1.55	0.485	1.598	1.005
23.035	19.264	19.249	15.432	10.907	9.662	8.036
10.837	10.515	5.724	6.804	3.463	0.064	0.817
7.944	13.964	8.439	11.701	9.843	2.195	2.598
13.082	13.501	11.584	12.821	11.721	7.036	3.518
14.843	8.337	8.922	8.67	9.598	6.014	6.044

18.129	12.126	17.218	15.683	12.18	15.203	13.603
13.017	12.62	9.959	13.136	12.367	15.397	8.743
31.637	38.03	22.253	26.511	24.252	15.783	11.888
11.973	8.917	9.789	11.717	10.751	11.473	10.033
10.412	5.808	2.192	2.419	3.587	0.237	2.63
14.958	11.415	10.428	14.138	10.929	4.966	8.506
16.651	18.631	10.795	13.585	10.143	9.135	6.205
21.579	23.271	17.718	26.464	21.787	20.607	15.917
12.02	4.062	6.498	6.054	5.757	2.913	1.177
10.047	14.323	12.204	16.946	13.256	9.977	13.522
15.405	13.154	7.836	14.983	8.563	8.039	8.654
9.444	9.741	5.765	3.125	1.874	0.247	0
8.232	5.591	8.644	4.29	4.678	0.201	1.791
13.014	9.256	7.292	5.051	11.444	10.686	7.038
9.232	9.399	7.609	6.204	6.475	5.486	5.808
10.661	10.602	8.157	5.794	4.494	5.184	1.894
13.035	20.012	15.029	8.397	8.943	4.509	2.249
13.903	13.776	10.385	14.124	8.606	7.894	3.061
0	0.672	0	0	0	0	0
6.328	1.624	0.845	0.65	0.81	0	0
6.994	7.005	4.475	1.481	0.354	0	0
3.958	6.409	2.399	1.495	0.743	0	0
5.544	6.025	5.255	0.076	0.05	0.024	0
3.74	3.916	0.075	1.318	0.06	0	0
4.868	2.049	0.987	1.06	0	0	0
7.46	6.22	6.56	6.74	2.588	3.184	3.622
9.523	12.267	7.259	11.539	6.239	12.382	4.128
16.292	11.966	11.562	12.359	5.629	13.616	6.712
0.979	1.788	0	1.139	0.001	0.003	0
12.257	2.326	3.152	2.606	4.927	1.04	3.12
10.5	23.004	21.439	17.902	11.049	7.741	8.87
0.004	0	0	0	0	0	0
13.523	5.027	6.383	2.868	1.839	0.18	0.852
0.124	0	0	0	0	0	0
4.512	4.224	3.721	3.192	1.895	0.227	0
0	0	0	0	0	0	0
2.435	0.875	3.604	1.682	0	0	0
6.277	5.23	3.091	1.456	0	0	0
9.822	9.494	7.724	3.684	2.705	2.183	0.454
1.276	0.882	0	0	0	0	0
2.906	0	0	0	0	0	0
17.88	9.025	2.247	1.019	0.062	0.023	0
3.377	4.312	0.399	0.126	0	0	0
0.903	0.547	0.934	0	0	0	0
13.614	10.544	9.139	4.85	3.444	1.938	2.441
78.337	64.315	64.545	60.746	46.963	72.845	45.278
20.337	12.158	6.503	6.11	2.711	2.008	2.063
4.256	4.027	1.661	0.522	0	0	0
5.359	10.393	2.136	5.105	1.181	2.07	0
102.404	102.038	82.596	76.447	57.96	52.158	43.83

20.74	29.059	23.186	15.806	13.731	16.807	14.042
14.933	13.576	13.925	10.027	14.087	10.263	11.1
15.483	12.757	7.284	11.6	8.21	2.866	3.591
6.641	9.528	1.858	4.87	3.6	1.185	0.636
6.386	12.204	3.917	3.973	0.043	0	0
2.183	2.123	0.032	0	0	0	0
29.605	31.635	24.874	26.648	19.022	9.722	19.184
6.079	10.165	5.544	4.107	4.568	6.03	0.787
8.784	4.575	3.743	3.954	5.375	1.13	2.568
0.669	0.098	0	0	0	0	0
11.333	11.874	14.453	11.359	8.646	6.68	10.88
6.495	2.112	4.301	6.442	2.155	1.861	0.02
21.175	20.659	16.825	15.267	13.353	8.197	7.752
14.392	7.95	9.021	22.044	15.919	2.322	6.239
0.995	1.163	0.021	0.05	0	0	0
3.16	0	2.746	0	0	0	0
6.856	4.203	5.664	3.826	1.01	2.766	1.459
0	0	0	0	0	0	0
20.012	21.762	13.982	10.583	10.392	11.637	5.703
4.827	2.63	4.396	2.581	0.924	1.776	0.986
0	0	0	0	0	0	0
42.561	33.369	32.049	35.743	23.006	27.609	27.266
5.578	5.729	0.102	2.849	1.786	3.719	0.288
39.601	35.662	24.129	31.019	34.689	18.224	8.932
0	0	0	0	0	0	0
2.989	1.766	0.088	0	0	0	0
6.173	3.723	4.095	1.717	0	0.99	0
9.182	8.244	6.615	4.573	2.042	0.323	0.468
29.776	26.924	25.668	25.461	21.716	15.839	9.286
37.27	20.075	21.167	16.829	26.942	23.027	20.526
30.545	25.175	25.846	21.119	12.818	26.063	21.982
17.859	15.551	12.119	9.397	7.448	5.45	2.335
12.751	9.387	8.489	10.392	5.69	3.329	3.37
1.001	0	0	0	0	0	0
0.698	0	0	0	0	0	0
2.797	3.273	1.723	0.994	1.003	0.166	0
28.311	17.446	17.574	28.885	32.415	13.808	21.421
10.157	4.67	2.88	2.752	0.001	1.092	0
4.804	6.588	5.255	2.544	5.23	2.735	3.76
30.889	21.463	18.919	9.862	14.042	12.653	8.589
0.234	0.409	0	0.008	0	0	0
0	0	0	0	0	0	0
13.842	4.421	5.682	1.19	0.63	0	0
3.751	6.822	2.705	4.601	4.028	4.863	2.322
1.383	2.902	0.629	0.672	0	0	0
0.464	0.355	0.413	0	0	0	0
7.867	7.341	8.107	5.078	4.804	5.524	6.021
1.794	2.521	0.997	0.322	0.045	0	0
0	0	0	0	0	0	0
15.163	7.664	6.086	2.96	4.593	3.512	1.15

10.737	1.577	3.179	0.207	0.99	0	0
15.044	4.019	4.477	12.727	10.149	5.763	1.811
14.651	7.022	5.125	1.108	5.364	4.698	2.191
1.91	0.001	0	0	0	0	0
22.205	12.443	17.654	10.011	12.631	6.033	5.944
5.156	5.933	6.935	8.664	2.204	4.978	4.25
3.25	5.224	4.59	2.958	3.418	1.896	0
3.447	0.927	0.469	0	0	0	0
4.238	1.037	0	0	0	0	0
5.337	2.477	2.936	0.11	0	0.07	0
0	0	0	0	0	0	0
0	0	0	0	0	0	0
5.34	2.798	1.572	0	0	0	0
9.906	11.608	6.6	4.654	4.242	0.08	0.375
18.547	19.854	13.883	22.344	9.981	10.61	5.961
12.635	10.55	5.458	3.12	2.831	3.203	0.019
12.551	7.808	7.777	9.866	13.812	6.636	4.941
7.69	12.791	6.323	9.518	10.607	5.276	5.261
0	0	0	0	0	0	0
4.646	2.356	0.455	0	0	0	0
29.616	43.192	28.494	30.107	22.711	25.977	22.688
0	0	0	0	0	0	0
23.541	13.354	8.152	11.462	6.719	6.393	1.519
7.502	2.336	5.421	6.542	4.399	1.037	1.881
15.071	8.862	9.409	14.338	14.784	9.64	8.579
26.832	12.418	15.574	9.554	5.895	2.539	1.121
3.082	0.961	2.057	0	0	0	0
8.495	5.791	1.814	1.568	2.807	0.039	0
1.54	0.275	0	0	0	0	0
3.415	5.485	1.539	4.83	0.433	1.92	0.007
5.915	2.025	0.872	1.72	0	0	0
20.471	7.635	19.057	6.122	14.165	5.906	6.524
5.848	1.271	0.184	0	0	0	0
3.655	2.626	1.383	0.966	0	0	0
9.049	6.735	2.015	3.01	1.147	0	0
3.045	0.846	2.883	0.005	0.02	0	0
0	0	0	0	0	0	0
10.342	0.987	2.694	2.662	5.724	0.947	3.129
6.522	3.132	0.849	1.721	0.016	0.787	0
30.602	29.133	25.226	26.734	28.034	24.659	23.555
15.774	7.17	6.088	3.05	4.629	3.474	1.174
12.623	8.403	8.865	6.914	6.997	8.873	8.316
5.75	3.417	2.708	0.191	0.72	0	0
17.34	9.305	5.04	1.043	0.986	0	0
1.039	0.106	0	0	0	0	0
2.539	1.62	0.375	0.479	0	0	0
12.526	5.299	8.827	4.87	6.258	3.838	0.098
66.809	50.975	57.682	40.743	40.794	46.617	37.884
0.001	0.003	0	0	0	0	0
1.438	0	0.441	3.115	0.875	0	0

0.398	0	0	0	0	0	0
0	0	0	0	0	0	0
10.244	6.643	6.649	3.369	2.746	4.693	0.48
0.016	0	0	0	0	0	0
6.801	9.938	5.137	8.644	6.009	3.862	5.952
34.152	35.772	20.667	27.952	19.113	12.863	9.998
12.318	13.521	11.236	8.056	3.543	2.236	0.789
5.635	1.739	2.462	0.685	0.054	0.133	0
5.129	0.99	0.373	0.606	0.003	0.132	0
5.652	5.841	5.236	11.407	12.954	2.977	3.319
25.011	22.999	18.449	11.96	8.276	4.561	6.34
10.103	11.461	9.287	13.065	5.764	6.469	10.518
12.961	6.26	5.492	2.617	3.772	0.664	0.04
12.94	10.149	6.446	6.889	4.239	3.874	1.942
7.853	8.123	4.876	6.282	7.14	3.699	3.209
7.516	6.646	4.829	3.621	0.793	0	0
20.461	4.198	11.165	17.018	23.23	3.717	5.493
5.099	9.252	6.589	7.06	9.056	1.489	4.66
0.337	0	0	0	0	0	0
5.11	9.269	6.567	7.053	9.061	1.489	4.662
2.248	0	0.714	1.114	0	0	0
13.375	12.541	12.306	9.809	13.511	9.02	8.544
10.26	7.041	2.739	0.891	0	0	0
13.477	7.687	11.591	4.438	2.785	0.375	0
11.918	7.737	7.588	6.75	14.913	8.368	11.571
33.594	22.205	26.55	22.605	15.56	18.054	15.53
1.305	0.006	0.011	0.007	0	0	0
0	0	0	0	0	0	0
15.196	10.561	16.732	16.46	16.892	13.091	11.171
8.291	4.266	2.068	0.369	0.009	0	0
52.471	49.443	42.632	44.361	37.196	28.085	33.288
8.739	4.833	3.333	0.344	0.008	0.014	0
0	0	0	0	0	0	0
8.269	8.585	5.889	2.012	0.511	1.372	0.985
27.734	29.262	18.453	17.926	21.18	23.841	15.247
10.029	7.514	2.419	6.159	0.092	0.428	0
17.649	10.31	11.522	5.536	7.85	5.889	3.163
0.268	1.567	0.035	0.166	0	0	0
2.873	3.273	4.043	3.14	0.818	0.007	0.001
13.625	9.216	11.388	9.67	13.355	11.119	5.304
3.964	3.155	1.055	0	0	0	0
5.794	6.02	8.12	5.085	4.357	3.998	2.037
12.267	13.473	9.587	12.546	8.772	6.182	3.249
10.84	8.92	3.686	4.485	4.245	4.382	0.619
4.484	1.254	3.073	4.367	2.566	0.042	0.109
12.506	6.986	4.533	0.003	0.146	0	0
15.836	3.674	8.219	13.25	8.44	9.691	8.846
1.275	0.882	0	0	0	0	0
1.537	1.708	0	0	0	0	0
3.685	3.795	1.057	1.225	1.022	0.368	0

11.697	15.452	10.208	16.099	13.266	7.418	8.887
8.74	5.45	3.242	1.991	0	0	0
4.927	4.605	1.333	1.204	0.025	0	0
13.735	15.225	6.761	7.693	7.048	2.125	3.173
0	0	0	0	0	0	0
1.384	0.009	0.057	0	0	0	0
0.227	0.088	0	0	0	0	0
20.429	9.404	6.008	9.332	9.05	2.66	3.375
15.736	5.888	11.135	12.348	13.411	6.916	9.832
8.18	7.298	7.775	10.331	5.079	2.665	0.809
10.695	6.501	7.312	5.735	3.922	1.871	1.898
5.84	5.127	3.222	0.97	0.01	0	0
14.926	8.795	10.543	10.073	10.052	9.399	7.19
3.884	1.272	0.391	0.001	0	0	0
7.091	2.543	1.48	1.578	0	0	0
22.053	12.733	14.19	17.839	15.034	9.338	11.58
0.001	0.004	0	0	0	0	0
28.609	26.432	24.326	15.605	15.216	13.928	7.383
61.621	63.019	49.275	46.266	45.914	37.356	35.438
26.152	25.225	22.281	24.685	30.572	22.761	23.077
91.975	80.681	80.528	72.023	75.489	50.658	58.433
17.528	7.658	10.005	1.52	1.459	0.003	0.001
5.977	4.653	0.779	0.859	0	0	0
9.256	7.108	2.661	4.496	3.39	1.328	1.82
7.089	1.653	1.692	0.266	0	0	0
15.935	7.939	11.355	7.898	8.895	9.315	7.598
7.957	10.117	5.932	2.331	1.189	1.682	3.56
3.182	0.791	2.108	0.196	2.001	0.061	0.842

RDF125u	RDF130u	RDF135u	RDF140u	RDF145u	RDF150u	RDF155u
0	0	0	0	0	0	0
12.155	11.497	3.186	4.322	9.231	7.756	2.64
0	0	0	0	0	0	0
0	0	0	0	0	0	0
0	0	0	0	0	0	0
0	0	0	0	0	0	0
6.703	3.345	2.305	2.465	4.169	0.691	0.142
0	0	0	0	0	0	0
0	0	0	0	0	0	0
1.958	0.266	0	0	0	0	0
6.569	7.711	6.606	1.201	0.316	0	0
16.764	11.219	8.046	10.682	2.251	0.83	0
0	0	0	0	0	0	0
0	0	0	0	0	0	0
0	0	0	0	0	0	0
0.044	0	0	0	0	0	0
0	0	0	0	0	0	0
1.779	1.522	1.261	0.193	0	0	0
6.797	5.913	4.01	1.901	1.866	1.797	0.824
1.652	0.587	0	0	0	0	0
0	0	0	0	0	0	0
0	0	0	0	0	0	0
1.943	1.012	0.137	0.453	0.787	0	0
3.934	0.352	1.451	0	0	0	0
0.108	1.158	0.216	0	0	0	0
0.487	0	0.398	0	0	0	0
9.62	13.841	7.236	9.342	6.289	4.891	8.178
15.001	12.506	9.562	8.116	6.179	6.975	9.103
0.163	0.027	0	0	0	0	0
16.105	10.995	8.647	9.107	12.15	10.144	7.079
14.374	17.512	5.779	4.668	0.732	0.607	0
11.378	11.53	11.382	7.655	6.525	6.48	6.417
0	0	0	0	0	0	0
0	0	0	0	0	0	0
0.171	0	0	0	0	0	0
14.045	13.162	8.257	11.995	8.637	6.686	5.53
0	0	0	0	0	0	0
0	0	0	0	0	0	0
12.612	11.96	8.119	13.273	8.766	14.299	9.067
1.333	1.035	0	0	0	0	0
3.025	2.854	0	0.03	0	0	0
0	0	0	0	0	0	0
25.08	21.616	18.412	10.582	10.374	19.161	12.165
0	0	0	0	0	0	0
10.151	7.717	6.346	2.273	4.596	2.167	0.894
0	0	0	0	0	0	0
0.897	1.47	0.085	0	0	0	0
4.995	6.352	5.549	11.642	6.879	9.518	2.892
5.284	2.92	5.384	1.106	3.884	2.447	1.54

15.159	13.008	9.314	7.621	4.247	4.876	6.442
12.684	5.127	4.076	5.299	1.89	3.045	0.266
16.197	17.87	18.648	18.607	18.475	18.793	17.22
6.948	4.741	6.498	4.581	5.879	4.357	1.987
0	0.137	0	0	0	0	0
7.125	7.765	6.577	1.985	0.163	1.382	0.008
10.54	14.568	11.643	5.496	8.357	6.974	3.101
11.513	15.817	11.712	6.266	7.616	1.696	2.481
0.166	0.175	0	0	0	0	0
10.459	7.886	5.07	5.422	14.022	6.978	7.662
6.294	9.005	6.027	3.968	3.534	3.134	1.113
0	0	0	0	0	0	0
0.166	0.082	0	0	0	0	0
5.335	6.58	4.485	2.776	3.346	3.013	0.953
4.631	4.54	1.529	2.362	1.182	0.617	1.993
1.13	0.052	0	0	0	0	0
0.916	0.421	0	0	0	0	0
3.428	2.834	1.847	0	0.733	0.002	0
0	0	0	0	0	0	0
0	0	0	0	0	0	0
0	0	0	0	0	0	0
0	0	0	0	0	0	0
0	0	0	0	0	0	0
0	0	0	0	0	0	0
0	0	0	0	0	0	0
3.187	0	0	0	0	0	0
5.882	3.805	4.287	4.07	2.792	3.8	1.929
8.208	2.435	5.011	3.187	4.531	1.459	1.763
0	0	0	0	0	0	0
0	0.51	0	0	0	0	0
8.669	4.352	4.706	3.175	0.35	0.735	0.03
0	0	0	0	0	0	0
0	0	0	0	0	0	0
0	0	0	0	0	0	0
0	0	0	0	0	0	0
0	0	0	0	0	0	0
0	0	0	0	0	0	0
0	0	0	0	0	0	0
0.007	0.002	0	0	0	0	0
0	0	0	0	0	0	0
0	0	0	0	0	0	0
0	0	0	0	0	0	0
0	0	0	0	0	0	0
0	0	0	0	0	0	0
0.456	0.311	0.002	0.002	0	0	0
63.304	43.918	38.52	51.723	35.806	31.408	46.075
0.003	0.596	0.011	0	0	0	0
0	0	0	0	0	0	0
0	0	0	0	0	0	0
33.493	24.117	18.757	10.025	4.238	0.513	0.249

8.482	4.111	3.808	1.267	1.91	0.413	0
7.38	9.021	5.696	7.981	2.91	2.298	1.954
0.355	0.198	0.233	0.023	0	0	0
0.261	0	0	0	0	0	0
0	0	0	0	0	0	0
0	0	0	0	0	0	0
14.503	10.937	4.021	8.415	7.519	2.112	2.16
7.228	0.794	2.544	0.355	0	0	0
0.837	0.813	0.99	0.301	0.011	0	0
0	0	0	0	0	0	0
7.316	5.92	3.623	4.055	2.477	1.083	1.573
1.539	0	0	0	0	0	0
3.274	2.525	2.091	1.761	0.372	0	0
1.953	0.005	1.489	0	0	0	0
0	0	0	0	0	0	0
0	0	0	0	0	0	0
2.625	0	0.527	0	0	0	0
0	0	0	0	0	0	0
4.972	6.052	2.576	2.391	1.564	0.266	0
0	0	0	0	0	0	0
0	0	0	0	0	0	0
25.635	29.368	20.126	21.755	18.524	16.907	24.654
0.186	0.001	0	0	0	0	0
17.922	20.204	10.008	8.629	6.617	13.402	6.208
0	0	0	0	0	0	0
0	0	0	0	0	0	0
0	0	0	0	0	0	0
0.062	0.052	0	0	0	0	0
9.096	4.699	1.926	1.93	0.771	0	0
16.731	19.023	15.694	23.365	15.646	18.976	21.029
19.74	20.725	13.195	12.961	6.681	6.009	4.816
1.088	0.001	0.003	0	0	0	0
1.786	0.897	0	0	0	0	0
0	0	0	0	0	0	0
0	0	0	0	0	0	0
0	0	0	0	0	0	0
22.718	21.71	18.923	19.302	16.964	16.461	11.111
0	0	0	0	0	0	0
0.517	1.027	1.015	0.009	0	0	0
5.694	5.286	2.298	0.047	0.021	0	0
0	0	0	0	0	0	0
0	0	0	0	0	0	0
0	0	0	0	0	0	0
4.022	0.828	0.28	0.006	0	0	0
0	0	0	0	0	0	0
0	0	0	0	0	0	0
2.607	5.182	5.319	2.687	0.208	0.789	0.021
0	0	0	0	0	0	0
0	0	0	0	0	0	0
0.751	2.492	1.324	1.413	0	0	0

0	0	0	0	0	0	0
6.294	7.418	2.995	3.11	1.29	4.892	2.59
2.358	0.095	0.992	0	0	0	0
0	0	0	0	0	0	0
2.959	1.502	1.972	0.446	0	0	0
2.252	1.392	0.234	0.217	0	0	0
0.085	0	0	0	0	0	0
0	0	0	0	0	0	0
0	0	0	0	0	0	0
0	0	0	0	0	0	0
0	0	0	0	0	0	0
0	0	0	0	0	0	0
0	0	0	0	0	0	0
0	0	0	0	0	0	0
11.965	6.976	9.673	10.422	8.403	6.331	3.618
0.159	0.298	0	0	0	0	0
2.851	1.531	0.066	0	0	0	0
7.252	7.542	5.143	5.009	4.104	3.314	3.341
0	0	0	0	0	0	0
0	0	0	0	0	0	0
31.545	22.012	27.398	18.804	18.345	10.496	8.628
0	0	0	0	0	0	0
1.854	0.129	0	0	0	0	0
2.456	1.705	0	0	0	0	0
6.59	5.954	2.141	0.561	0	0	0
0.004	0.001	0	0	0	0	0
0	0	0	0	0	0	0
0	0	0	0	0	0	0
0	0	0	0	0	0	0
0.012	0	0	0	0	0	0
0	0	0	0	0	0	0
7.801	7.873	7.244	4.04	3.935	6.19	0.2
0	0	0	0	0	0	0
0	0	0	0	0	0	0
0	0	0	0	0	0	0
0	0	0	0	0	0	0
0	0	0	0	0	0	0
0	0	0	0	0	0	0
0	0	0	0	0	0	0
19.626	18.499	15.511	23.607	24.161	21.971	24.837
0.821	2.536	1.323	1.483	0	0	0
5.82	5.673	6.047	3.532	1.947	2.376	3.188
0	0	0	0	0	0	0
0	0	0	0	0	0	0
0	0	0	0	0	0	0
0	0	0	0	0	0	0
0.545	1.463	0.017	0	0	0	0
31.432	34.579	33.969	23.31	25.467	13.043	11.194
0	0	0	0	0	0	0
0	0	0	0	0	0	0

0	0	0	0	0	0	0
0	0	0	0	0	0	0
0.809	0.08	0	0	0	0	0
0	0	0	0	0	0	0
1.378	2.534	0.001	0.004	0	0	0
4.522	6.364	3.204	4.775	0.545	0.282	0.091
0.488	0	0	0	0	0	0
0	0	0	0	0	0	0
0	0	0	0	0	0	0
5.098	4.836	3.287	3.038	2.336	4.106	0.065
5.342	2.931	1.904	0.031	0.779	1.487	0.007
5.465	6.369	8.457	2.878	3.595	4.22	0.08
0	0	0	0	0	0	0
0.095	0.845	0	0	0	0	0
3.151	2.009	0.19	0	0	0	0
0	0	0	0	0	0	0
9.762	12.275	1.996	4.051	7.251	7.239	3.357
2.527	3.625	0.191	1.039	0	0	0
0	0	0	0	0	0	0
2.527	3.617	0.191	1.031	0	0	0
0	0	0	0	0	0	0
7.302	0.835	2.22	1.599	0.96	0.001	0.005
0	0	0	0	0	0	0
0	0	0	0	0	0	0
7.789	7.099	4.871	3.619	2.509	3.669	3.186
15.002	14.172	8.79	4.118	0.856	0.998	0
0	0	0	0	0	0	0
0	0	0	0	0	0	0
9.597	7.472	7.119	6.33	6.448	5.149	5.852
0	0	0	0	0	0	0
20.236	14.809	10.418	6.244	1.952	1.005	0.595
0	0	0	0	0	0	0
0	0	0	0	0	0	0
0.03	0	0	0	0	0	0
11.628	15.677	14.256	9.104	6.287	0.659	1.51
0	0	0	0	0	0	0
3.129	3.216	1.657	0.851	0	0	0
0	0	0	0	0	0	0
0	0	0	0	0	0	0
5.831	5.04	3.085	1.302	2.028	1.479	0
0	0	0	0	0	0	0
2.801	1.004	0.001	0.546	0	0	0
1.069	0	0	0	0	0	0
0.005	0	0	0	0	0	0
0	0	0	0	0	0	0
0	0	0	0	0	0	0
8.779	5.79	7.041	5.746	6.222	2.703	3.266
0	0	0	0	0	0	0
0	0	0	0	0	0	0
0	0	0	0	0	0	0

9.621	10.335	5.427	5.258	5.748	2.823	0.518
0	0	0	0	0	0	0
0	0	0	0	0	0	0
0.337	0.228	0.167	0.027	0	0	0
0	0	0	0	0	0	0
0	0	0	0	0	0	0
0	0	0	0	0	0	0
2.762	0.016	1.098	0.008	0	0	0
13.818	10.424	8.216	7.674	5.953	4.812	4.782
1.94	1.141	0	0	0	0	0
0.16	0.009	0	0	0	0	0
0	0	0	0	0	0	0
7.626	3.633	6.633	4.04	3.843	2.04	1.552
0	0	0	0	0	0	0
0	0	0	0	0	0	0
9.464	11.5	9.688	6.913	4.628	7.682	6.085
0	0	0	0	0	0	0
7.138	3.022	1.302	1.504	0	0	0
30.044	16.647	15.942	13.631	6.706	2.993	0.841
16.426	19.385	25.952	10.002	13.168	11.336	11.271
43.974	32.802	18.69	25.073	11.14	9.879	6.862
0	0	0	0	0	0	0
0	0	0	0	0	0	0
1.996	0.903	0.383	0	0	0	0
0	0	0	0	0	0	0
5.791	7.249	4.676	4.284	4.495	1.952	3.967
0.32	0.029	0	0	0	0	0
0.838	0	0	0	0	0	0

RDF010m	RDF015m	RDF020m	RDF025m	RDF030m	RDF035m	RDF040m
0.755	9.253	0.519	12.545	0.962	3.028	1.658
1.272	14.679	1.095	22.009	1.875	2.318	4.339
0.735	11.163	0.927	20.516	4.908	10.846	7.011
0.494	3.357	0.453	6.707	0.406	1.264	1.627
0.649	6.786	1.141	8.265	1.873	6.77	5.078
0.921	15.306	0.634	11.736	12.994	3.097	8.165
1.263	19.544	1.694	27.133	12.532	16.116	6.654
0.278	2.772	0.316	3.344	0.903	4.033	2.593
0.543	9.099	1.283	19.157	9.434	7.132	10.405
1.032	11.142	0.943	14.532	0.957	2.187	3.219
2.659	35.643	3.052	50.257	16.757	13.795	15.495
1.139	17.294	9.355	22.036	45.57	11.412	17.456
0.923	13.276	0.798	19.478	3.382	3.674	3.548
1.159	14.449	1.356	21.686	8.353	10.372	23.229
1.083	13.845	2.76	25.196	7.017	8.497	10.751
1.027	13.63	2.181	23.021	2.673	7.399	12.029
0.502	5.426	0.335	8.948	4.386	3.816	1.679
0.951	21.697	1.274	28.622	6.545	7.807	11.517
1.093	11.231	0.969	15.116	1.307	1.174	2.357
0.976	15.732	0.717	22.258	7.465	4.04	5.94
0.798	11.454	0.46	18.911	9.319	8.978	2.965
0.335	5.407	0.625	8.364	4.411	9.958	14.261
0.578	7.864	0.525	18.116	5.335	7.504	8.038
0.87	12.493	2.106	21.175	4.687	5.194	6.428
1.341	18.36	1.078	23.801	4.187	5.226	5.112
1.325	24.189	1.111	43.878	14.095	19.881	11.973
1.236	16.293	1.104	16.141	0.925	2.165	2.882
1.794	21.694	1.322	30.597	9.708	11.735	6.009
0.93	12.312	0.742	15.016	4.392	6.256	2.27
1.31	16.075	1.366	17.799	1.655	1.252	2.964
1.277	17.981	1.11	29.072	6.843	12.122	21.074
1.775	33.395	3.342	48.797	29.981	23.624	18.414
1.011	12.416	0.654	18.745	1.879	4.013	1.76
1.135	14.651	0.73	20.756	4.891	2.43	6.216
1.674	31.599	1.429	52.198	22.039	17.264	12.279
1.211	20.145	0.899	27.157	4.473	9.03	7.063
1.232	21.153	1.221	32.842	11.518	6.346	6.762
0.405	5	0.625	8.675	3.583	9.386	4.415
1.863	26.245	1.039	41.359	10.982	6.962	6.019
0.731	14.411	0.552	22.437	4.571	9.227	4.206
0.954	13.071	2.906	27.38	13.307	15.469	10.266
0.638	8.104	0.725	12.729	8.164	5.581	4.123
2	35.022	1.129	66.982	30.847	13.341	14.304
0.623	8.073	0.383	13.877	3.723	5.301	7.204
1.224	15.969	1.436	19.305	1.991	3.902	5.041
0.835	11.6	2.168	19.359	5.384	8.252	11.558
1.004	12.696	2.861	20.269	5.844	9.323	8.599
0.929	13.988	3.661	21.314	6.86	12.041	14.284
0.691	11.163	4.576	24.303	9.024	11.777	9.244

1.108	18.534	3.506	30.478	9.734	12.855	13.526
0.93	12.967	2.856	28.617	5.71	9.223	13.205
1.339	21.732	3.58	34.452	10.73	22.753	19.527
0.921	14.087	3.13	28.484	6.969	10.074	12.643
0.931	13.264	2.777	28.174	11.626	12.273	12.129
1.092	13.612	2.791	21.944	4.42	8.42	9.311
1.09	21.944	3.439	26.955	14.701	15.158	13.598
1.148	16.996	2.911	34.988	10.817	13.504	15.812
0.761	11.004	2.326	24.03	6.593	7.77	10.419
1.019	14.93	3.111	31.631	9.205	19.822	22.471
0.893	13.686	2.737	20.259	7.35	8.836	11.588
0.691	13.657	0.327	23.072	7.476	6.968	6.907
0.947	12.441	2.815	19.668	8.684	8.842	8.151
0.791	12.421	3.104	23.656	9.411	11.955	8.256
0.794	12.448	2.806	23.687	9.986	10.58	9.779
1.009	14.977	2.391	22.21	10.438	8.342	4.87
1.198	18.258	1.116	31.688	10.695	7.383	8.353
1.078	16.841	1.154	22.297	2.823	3.157	10.655
0.402	10.277	0.374	18.988	7.694	3.437	19.685
0.766	9.654	0.601	13.929	3.6	2.327	8.876
0.759	10.116	0.622	14.758	5.409	0.774	18.489
0.631	8.705	0.465	12.842	3.591	2.902	13.696
0.729	10.542	0.729	15.831	4.195	1.424	18.774
0.692	12.148	0.325	17.498	4.531	5.496	12.866
0.547	8.71	0.495	10.275	1.387	3.87	9.09
0.946	6.207	1.132	8.156	1.055	8.257	5.3
0.926	12.081	0.904	16.839	2.685	7.509	3.407
0.959	14.429	0.685	22.483	0.939	2.509	3.272
0.476	9.372	0.476	15.387	1.054	8.529	3.885
0.841	15.743	0.673	21.663	3.399	4.674	5.764
1.49	21.1	1.398	28.573	17.795	4.813	10.622
0.52	5.495	0.384	11.457	3.29	3.625	9.467
0.74	10.942	0.67	17.261	2.342	6.066	10.345
0.462	5.494	0.371	5.084	7.131	7.736	12.46
0.812	12.749	0.816	21.921	2.957	6.42	10.299
0.621	12.048	0.759	12.129	7.825	1.034	11.713
0.713	7.78	0.447	12.728	2.423	1.556	12.59
0.938	11.823	0.782	17.483	4.418	4.903	3.159
1.325	24.112	0.918	42.805	14.259	20.801	9.436
0.556	8.753	0.485	16.475	7.905	3.99	10.312
0.593	6.127	0.508	11.863	12.024	11.017	17.273
0.838	14.862	3.01	23.908	11.442	11.842	26.203
0.588	9.204	0.906	11.466	3.177	8.183	6.097
0.48	6.087	0.308	16.163	2.231	2.866	4.109
1.418	25.593	0.882	41.72	18.497	12.579	6.209
2.73	51.191	2.497	74.077	31.457	18.91	10.956
1.06	14.875	1.129	21.46	8.705	7.565	21.212
0.855	10.674	0.811	13.962	1.591	3.461	1.822
1.266	14.399	0.894	25.278	11.737	9.725	8.371
2.383	41.798	3.4	74.299	29.491	21.373	36.907

1.17	19.193	1.181	24.13	7.45	1.979	7.948
1.075	15.945	0.921	23.267	5.978	11.032	8.897
1.378	20.466	1.273	46.3	11.866	16.433	12.57
0.931	10.004	1.09	21.5	2.715	7.965	4.754
1.144	12.245	1.11	12.736	3.695	1.826	2.4
0.939	13.454	0.653	20.712	6.653	6.445	5.863
1.432	30.17	2.028	39.576	8.681	11.862	16.781
0.987	8.277	0.782	24.17	5.86	7.587	10.567
0.681	8.322	0.412	15.836	1.481	1.102	1.463
0.667	6.508	0.383	12.084	3.515	4.383	2.126
1.131	15.265	0.795	22.192	5.36	5.937	6.136
0.592	7.529	0.47	21.438	7.355	4.49	11.907
1.492	23.176	0.845	41.059	10.491	7.905	6.927
0.894	17.756	0.963	23.138	1.655	2.711	10.22
0.561	9.121	1.376	15.586	6.279	12.508	6.274
0.422	8.422	0.226	14.106	5.775	10.769	1.288
0.577	10.068	0.859	18.487	9.082	9.799	5.964
0.248	1.334	0.407	2.179	0.005	5.642	2.873
1.084	19.514	1.073	24.851	6.514	2.441	15.064
0.623	7.431	0.358	15.792	5.431	3.878	4.198
0.319	5.46	0.195	4.315	1.937	0.86	1.632
1.905	32.436	1.102	45.861	9.107	5.828	6.601
0.679	10.193	0.511	19.029	4.12	1.524	12.501
1.952	31.808	1.382	53.649	22.679	17.695	12.973
0.836	9.477	0.732	15.172	5.518	4.543	2.001
0.877	16.022	1.325	22.737	9.14	8.316	6.233
0.786	8.505	1.308	11.127	2.483	6.997	5.428
0.925	10.21	0.54	17.744	4.355	2.142	2.801
2.187	29.767	2.289	39.642	11.624	8.499	17.411
1.494	22.42	1.088	31.132	9.238	9.763	12.576
1.278	19.058	1.075	31.899	6.062	4.694	16.2
0.975	16.118	1	24.232	7.23	10.428	2.912
0.776	12.999	0.696	18.901	5.098	5.86	12.04
0.522	12.163	0.322	18.207	8.796	2.009	19.579
0.805	17.745	0.671	25.705	11.564	7.679	7.591
0.782	8.242	0.376	14.623	4.663	3.184	2.247
1.367	18.795	1.42	31.342	2.652	4.425	7.147
0.741	10.184	0.624	16.458	2.926	9.585	11.667
0.71	9.744	0.519	14.791	5.893	5.855	2.82
1.171	18.412	1.42	32.391	9.613	10.714	5.741
0.353	10.732	1.488	11.374	2.4	9.31	2.864
0.427	10.137	1.011	10.84	2.096	7.776	2.195
1.07	14.929	0.985	23.05	4.378	11.091	9.425
0.646	8.427	0.427	16.202	7.91	4.424	11.268
0.679	8.263	0.401	16.173	5.303	4.902	3.211
0.604	6.514	1.027	9.91	4.161	4.432	7.415
0.618	11.033	0.722	14.807	1.787	11.691	6.534
0.848	8.381	0.77	17.463	2.848	10.205	6.126
0.657	7.736	0.394	8.875	7.478	3.94	3.105
0.849	17.104	0.892	26.053	4.501	5.729	7.087

0.91	10.106	0.68	15.56	5.665	1.8	5.32
0.849	14.58	0.958	17.147	4.88	6.328	3.182
0.871	15.03	0.796	24.078	3.465	4.709	7.133
0.553	8.545	0.578	16.166	10.786	7.449	15.295
1.032	13.655	3.118	24.944	6.674	7.029	7.057
0.647	10.65	0.546	26.042	9.021	8.044	14.198
0.902	9.948	0.758	15.885	2.387	2.125	8.039
0.838	12.373	0.752	18.668	7.677	6.825	3.613
0.759	12.917	0.787	19.311	5.214	3.192	4.703
0.91	11.916	0.663	18.241	3.775	2.144	3.399
0.216	3.691	0.348	5.458	1.037	3.151	4.623
0.673	2.932	0.613	3.976	1.41	1.377	0.56
1.097	13.798	0.653	22.437	7.991	8.817	9.917
0.929	13.662	2.841	22.447	5.137	10.293	10.52
1.293	14.021	1.11	26.475	4.096	9.612	7.239
1.362	23.217	0.934	39.639	12.065	13.536	8.704
1.006	11.189	0.912	16.547	6.127	1.558	8.856
1.086	12.637	1.158	14.724	0.886	1.006	2.593
0.434	5.416	1.243	8.021	2.198	3.328	1.752
0.542	9.496	0.634	19.245	11.389	8.873	9.623
1.595	21.363	1.011	34.205	9.408	7.224	12.29
0.865	17.635	0.987	25.472	11.88	6.844	8.318
1.058	20.264	0.886	27.79	6.4	9.983	7.4
0.967	11.797	0.698	16.675	4.229	4.251	4.072
1.28	16.428	1.112	24.699	5.534	1.617	3.349
1.024	15.776	2.306	27.452	5.54	9.481	7.829
0.556	7.812	0.354	14.361	1.294	3.561	4.672
1.089	21.785	0.888	29.607	12.449	10.248	8.77
0.908	19.072	0.844	27.61	11.165	11.88	8.84
0.646	7.417	0.557	14.311	3.475	3.925	2.462
0.752	7.634	0.689	9.513	3.168	4.809	2.971
1.034	19.927	3.142	30.078	10.243	6.867	8.281
0.804	11.038	1.418	21.722	3.185	12.554	8.497
0.583	9.183	0.941	14.227	6.291	4.906	4.831
0.879	11.957	1.384	21.611	4.227	11.307	6.889
0.327	5.659	0.301	8.555	2.674	8.072	0.833
0.741	5.757	0.605	8.895	0.868	1.898	1.09
0.841	13.919	0.781	20.959	2.811	4.633	4.739
0.908	12.222	0.809	18.217	2.706	3.515	3.566
1.771	23.997	1.696	42.767	10.022	14.307	16.616
0.849	17.063	0.898	25.918	4.512	5.804	7.141
0.791	10.452	0.991	15.683	2.804	13.524	5.514
0.698	11.111	0.861	17.729	4.269	6.413	3.592
0.927	14.814	3.049	23.851	5.544	10.594	8.35
0.597	8.538	0.596	15.701	9.782	3.969	10.283
0.88	19.313	0.877	27.831	12.387	8.828	8.653
0.902	10.497	0.438	18.041	14.868	9.072	5.379
2.28	39.809	1.494	65.225	17.782	16.503	17.888
0.821	8.757	0.701	15.22	7.264	17.774	5.967
0.581	5.075	0.569	7.675	0.231	1.366	2.558

0.97	14.784	0.64	21.692	6.785	4.323	6.567
0.631	6.673	0.644	13.31	5.642	17.862	6.805
0.846	10.25	0.39	17.243	14.727	8.825	5.102
0.651	7.338	0.467	14.042	6.065	5.736	4.014
0.958	10.841	0.828	12.42	1.746	2.116	2.137
1.346	22.489	1.179	31.028	8.426	4.54	20.117
0.913	14.396	1.02	21.011	9.698	9.414	7.483
0.903	13.921	0.414	20.342	6.222	9.308	13.547
0.639	9.281	0.402	11.147	7.591	5.571	8.769
1.04	10.735	0.96	14.28	1.424	1.569	3.181
1.655	23.527	1.318	37.152	10.601	5.406	10.052
0.937	13.304	1.11	20.035	2.742	9.47	9.852
1.167	22.289	0.716	33.699	8.73	16.62	6.839
1.285	21.881	0.664	34.329	11.283	6.522	7.783
0.983	10.116	0.739	13.469	2.771	1.315	1.906
0.894	10.117	0.677	13.249	3.32	1.497	4.093
1.21	14.63	0.993	20.968	2.03	1.572	3.65
0.957	10.96	0.853	15.771	1.198	1.027	2.349
0.757	7.38	0.303	12.747	4.131	4.439	4.025
0.957	10.963	0.852	15.771	1.194	1.027	2.35
0.723	6.098	0.432	11.591	1.663	5.514	10.279
1.055	16.114	1.288	21.27	9.223	6.148	15.233
1.017	16.315	1.044	27.945	5.16	9.491	4.532
1.017	16.328	1.052	29.026	7.007	10.936	4.55
0.975	10.912	1.613	13.235	2.738	8.587	6.076
1.538	20.217	1.16	32.506	6.387	5.515	6.805
0.313	4.986	0.287	10.887	0.942	3.16	7.986
0.755	14.549	0.556	15.059	17.383	4.905	11.443
1.042	20.106	0.766	28.654	5.194	7.364	4.507
0.805	11.05	1.145	12.675	1.914	4.68	3.259
2.084	37.084	1.924	57.727	17.402	20.829	13.498
1.06	13.738	0.698	20.202	3.358	2.303	6.684
0.391	2.977	0.268	8.151	0.296	1.133	0.52
0.901	17.479	0.805	25.57	11.917	7.371	9.015
1.207	22.115	1.124	30.947	5.578	10.598	14.122
0.951	12.727	0.825	16.728	2.979	1.39	3.645
1.192	16.707	1.25	30.333	8.294	14.186	8.287
0.623	8.203	1.53	14.145	6.647	5.598	11.87
0.757	9.656	0.517	17.969	8.765	5.526	8.569
0.881	11.821	0.538	18.945	15.179	10.407	10.922
0.691	9.099	1.57	14.874	7.4	6.603	11.413
0.772	12.671	0.562	17.472	5.338	5.042	8.473
1.051	16.361	0.735	24.684	3.236	5.538	9.427
0.918	14.37	0.776	14.42	2.876	9.968	2.053
0.59	7.01	0.319	18.479	6.218	4.058	6.888
1.26	16.99	1.106	22.97	8.313	3.47	15.244
1.167	11.353	1.054	14.371	0.968	4.152	2.43
0.62	9.562	0.579	17.21	10.087	4.134	10.433
0.769	11.716	0.928	13.913	5.087	8.834	17.052
0.579	9.593	0.509	21.521	8.067	8.338	10.15

1.068	16.888	1.174	21.684	3.565	8.946	10.361
0.981	9.552	0.67	15.567	0.831	1.92	1.671
1.074	20.529	0.699	29.01	11.583	8.193	5.75
1.378	20.471	1.198	44.911	9.588	13.505	11.174
0.383	3.955	0.585	6.688	2.815	8.823	4.285
0.808	8.786	0.639	16.582	7.248	13.914	6.803
0.565	8.986	0.459	17.322	6.257	1.31	10.712
1.021	14.956	1.125	21.472	2.288	3.402	3.089
1.193	12.973	1.206	18.194	1.933	2.114	4.041
0.93	13.06	0.864	18.446	6.381	6.725	9.341
0.818	10.579	0.537	15.799	3.731	2.916	8.147
1.026	13.922	0.852	20.451	5.654	3.175	4.929
0.89	14.511	0.949	18.959	3.27	7.646	10.296
0.498	9.129	1.264	18.966	13.126	11.016	14.727
0.903	8.054	1.094	12.052	0.631	5.788	4.562
1.195	16.472	1.265	19.785	3.36	6.747	7.942
0.679	6.589	0.461	8.811	3.412	0.857	2.774
1.288	16.892	1.983	30.864	9.552	11.584	11.221
2.168	38.828	1.595	64.378	16.764	20.207	13.717
1.647	19.668	1.772	28.149	7.284	8.291	7.914
2.211	43.784	2.865	67.228	32.122	41.477	38.356
1.021	19.409	0.783	30.447	8.327	8.42	5.928
0.753	10.041	0.504	17.887	6.393	6.646	8.418
0.808	9.276	0.409	17.871	3.238	11.157	5.88
0.723	10.069	0.83	13.326	4.524	9.455	5.079
0.894	16.23	0.779	24.876	7.488	4.923	10.725
0.849	10.756	0.749	20.461	2.211	8	4.476
0.651	8.77	0.461	14.506	2.275	5.742	8.401

RDF045m	RDF050m	RDF055m	RDF060m	RDF065m	RDF070m	RDF075m
7.275	5.095	4.256	2.012	1.813	1.352	1.194
2.34	13.701	3.063	8.212	8.979	2.956	2.103
9.485	20.541	4.384	7.972	13.848	10.316	9.102
0.747	1.237	1.455	2.452	0.941	0.136	0
8.431	4.669	3.929	3.353	1.292	3.207	0.479
7.516	2.429	3.813	1.42	4.549	0.433	0.405
12.797	11.128	15.239	6.089	9.679	12.407	8.262
3.401	0.057	0.36	0.006	0.005	0	0
7.288	10.928	4.669	6.923	5.747	6.15	1.736
5.697	8.085	3.232	4.522	4.145	0.55	2.509
24.919	27.95	22.508	36.672	41.08	27.395	25.934
41.479	14.661	46.488	135.471	55.416	14.722	30.695
9.437	8.819	6.491	5.306	5.412	2.83	2.869
11.814	16.911	8.429	16.372	16.907	11.858	2.496
10.3	15.33	10.169	10.498	7.616	5.404	8.053
14.692	14.252	7.636	5.92	3.024	5.591	7.469
2.657	3.996	1.061	1.622	0.364	0.625	0.28
17.037	31.52	22.551	24.957	22.642	22.332	12.927
7.349	5.771	1.884	2.481	3.793	1.381	1.507
5.987	5.978	2.696	6.422	7.434	4.24	2.535
9.233	12.243	11.084	6.276	6.184	3.797	2.779
10.534	11.519	3.536	6.73	1.238	10.936	0.063
7.29	6.682	3.057	11.794	1.593	1.723	1.065
15.984	12.168	7.735	4.667	2.485	6.93	3.072
13.121	10.771	12.777	8.976	14.613	10.191	12.481
10.769	17.825	7.963	10.699	11.823	6.566	8.845
7.177	7.354	3.048	2.805	3.287	2.22	2.604
16.45	23.953	21.897	25.176	25.623	18.843	24.748
9.527	4.439	6.852	8.247	5.831	6.56	5.716
9.053	6.931	5.269	3.991	6.444	4.36	2.8
15.168	40.76	22.532	29.056	18.99	25.775	23.311
26.257	38.833	22.182	24.482	33.175	27.734	18.687
8.65	5.821	4.731	5.697	2.592	5.213	1.667
5.614	6.907	4.54	5.473	9.273	6.309	2.735
21.876	21.077	16.079	13.025	17.246	10.448	11.291
10.035	6.472	4.892	6.158	9.533	3.743	4.824
7.311	17.963	6.383	5.189	7.923	1.631	5.011
5.877	1.887	3.219	1.206	1.299	0.159	0.015
9.809	14.117	5.63	6.223	9.606	9.951	11.095
4.703	10.84	5.219	10.075	8.357	8.616	5.46
15.171	17.941	15.537	14.391	17.109	11.395	5.829
8.158	4.164	5.319	2.723	2.149	1.608	0.041
17.382	30.371	11.275	11.777	22.488	14.516	10.642
5.977	6.228	1.801	13.467	3.399	2.106	4.247
4.954	15.316	9.711	14.167	11.086	11.01	10.143
10.23	13.609	7.861	7.867	6.528	7.521	5.63
11.435	14.794	9.172	6.843	6.808	6.605	8.326
14.825	17.642	10.673	12.237	14.563	15.363	7.315
16.363	17.482	4.158	15.628	12.549	7.938	3.412

18.422	22.61	10.016	9.454	15.779	8.878	11.231
15.628	18.481	10.296	11.22	12.947	7.43	10.852
18.32	27.675	16.184	22.836	25.601	18.359	16.703
12.64	20.36	9.253	11.437	21.23	5.292	10.693
19.016	14.739	14.241	19.447	26.049	15.261	9.616
15.737	13.328	6.252	10.094	9.637	5.666	5.303
24.936	24.467	10.977	18.891	19.695	15.978	10.484
16.26	26.342	14.654	16.947	21.884	16.162	13.332
11.628	15.78	7.553	14.881	13.172	9.287	9.02
17.906	22.256	16.935	12.1	15.434	11.685	11.151
12.371	15.621	7.722	10.739	17.213	4.927	8.901
12.085	7.866	7.003	9.146	11.165	7.97	10.985
13.602	14.406	4.315	11.268	9.183	4.041	5.614
16.948	14.091	6.164	8.43	11.268	14.47	8.934
12.145	13.085	3.047	10.305	14.284	8.207	7.988
12.885	17.402	8.284	3.838	12.996	3.676	5.933
13.842	15.837	7.473	11.282	7.611	10.76	3.87
8.574	12.1	7.298	13.651	11.89	6.435	11.46
14.271	5.575	8.189	2.769	7.457	3.461	2.446
8.258	9.99	4.479	9.395	8.85	5.061	1.013
14.927	7.028	12.61	7.495	6.851	6.574	9.832
7.051	8.299	2.031	0.921	6.584	7.602	5.04
13.163	9.732	5.429	6.591	9.288	10.862	7.348
19.395	11.539	3.489	6.744	5.975	7.301	6.357
6.885	5.478	1.099	6.898	3.269	2.452	0.405
4.924	3.47	1.74	0.363	2.9	2.143	2.52
7.338	10.211	6.368	4.855	7.818	4.709	2.648
7.85	15.452	5.305	6.491	3.9	4.523	2.117
3.607	12.517	7.4	3.868	5.573	2.489	1.988
3.684	15.786	4.807	7.375	14.775	4.648	4.528
21.017	13.778	7.379	12.048	10.412	14.047	7.222
5.397	4.485	2.052	6.001	5.082	0.31	3.787
12.456	11.562	9.665	10.968	5.247	7.943	10.127
15.784	6.653	6.31	0.571	1.156	0.223	0.164
20.44	6.424	9.168	5.953	7.09	8.864	4.775
3.895	4.355	4.594	4.118	1.124	1.97	1.32
6.039	5.762	0.573	4.685	7.513	1.238	0.795
6.028	9.731	8.288	4.061	4.721	3.485	1.774
11.911	17.911	8.296	11.148	9.138	6.299	8.304
6.037	12.388	3.623	5.166	7.922	4.41	1.032
17.984	4.155	11.082	3.349	7.254	1.943	0.159
31.693	25.405	20.67	21.674	22.8	23.878	12.462
8.851	2.154	3.377	4.479	3.476	0.806	2.364
2.491	6.198	1.153	5.099	9.304	1.849	1.365
16.516	9.211	9.376	7.442	11.328	6.521	5.552
26.003	22.936	24.597	27.737	30.648	16.626	15.598
16.283	16.187	14.576	15.544	18.959	16.289	9.624
7.207	7.771	7.701	5.513	6.925	3.83	3.636
9.917	18.091	10.594	9.708	9.016	7.237	7.847
45.993	59.837	36.269	50.778	54.858	46.184	42.575

9.996	10.765	6.533	6.132	12.477	5.662	8.605
13.924	7.075	5.398	6.944	5.878	9.968	9.058
14.16	30.525	11.031	13.908	17.27	14.455	8.024
4.298	15.482	1.922	6.233	6.283	2.397	9.172
3.524	2.77	2.146	3.268	1.143	0.655	1.379
10.202	8.02	6.033	11.061	5.101	2.624	5.453
16.716	37.191	18.462	42.069	22.755	33.357	26.491
11.132	10.617	2.539	4.255	4.726	2.636	7.937
0.903	10.92	0.407	0.814	8.268	2.906	5.264
5.228	4.731	6.886	8.075	3.145	5.386	4.811
10.45	7.368	8.336	7.177	6.564	3.537	5.022
8.627	8.552	4.92	12.97	5.959	5.669	6.563
8.025	13.254	4.292	9.477	11.888	7.58	5.352
1.361	24.868	4.324	9.043	12.812	3.754	5.538
11.584	12.154	5.645	7.994	6.683	2.889	2.671
3.779	5.131	1.369	5.394	2.474	4.541	0.154
9.91	7.451	6.043	8.204	5.973	3.846	2.859
1.351	0.047	0.027	0	0	0	0
14.982	16.261	14.853	9.626	14.697	9.954	15.957
3.37	5.349	0.768	3.909	5.69	4.804	1.112
0.001	0.011	0.001	0	0	0	0
10.013	22.503	9.991	6.593	18.527	10.468	15.308
11.819	9.987	4.991	6.352	10.022	2.537	3.921
25.562	27.434	15.572	19.875	24.485	22.434	12.961
1.319	2.776	0.348	0.121	0.107	0.007	0
7.917	12.398	5.287	8.952	5.644	3.742	0.663
8.982	5.425	4.975	5.784	3.436	2.286	2.536
1.391	9.281	0.48	1.72	6.128	2.936	4.835
14.088	18.717	7.988	11.528	20.306	9.305	10.351
13.895	15.107	7.434	10.493	12.633	10.454	6.279
12.939	17.053	4.792	5.689	14.728	11.565	9.151
12.217	6.377	3.37	6.093	3.813	5.847	5.076
12.645	18.252	9.534	18.405	15.528	18.239	13.552
14.647	5.697	8.095	2.401	8.431	3.349	3.895
7.77	13.792	5.607	4.592	3.037	1.112	0.208
5.027	4.888	0.979	3.672	4.021	1.533	1.67
6.468	20.128	6.263	13.213	15.578	8.998	4.478
10.507	13.079	10.374	10.419	4.943	11.299	10.402
4.172	6.887	2.369	5.07	5.24	6.009	1.667
8.979	16.366	7.864	14.866	19.725	15.317	9.51
10.611	2.555	5.203	2.237	0.511	4.054	0.284
4.987	1.23	1.545	0.961	0.302	1.103	0.113
15.145	19.877	15.009	14.877	11.517	6.55	2.686
6.584	9.732	1.602	2.091	12.773	4.11	1.471
2.695	10.48	4.031	4.663	4.725	4.093	4.084
6.552	2.249	1.719	8.661	2.471	1.998	0.516
5.991	14.778	3.407	8.673	1.884	5.174	4.275
5.597	7.584	5.302	4.351	3.549	5.648	7.159
3.423	0.472	0.621	1.014	0.163	0.012	0.006
5.091	18.129	9.572	13.201	9.912	9.818	4.74

4.678	3.5	2.888	4.223	4.281	3.522	1.803
7.188	7.378	2.39	7.231	7.855	5.652	0.632
5.776	15.817	6.529	11.314	8.841	9.434	3.245
18.038	9.129	6.872	9.217	9.747	5.139	1.933
8.242	12.622	6.125	19.998	12.591	15.202	7.22
13.836	13.463	2.857	3.365	11.701	2.026	3.666
9.15	9.302	5.417	5.408	5.006	10.329	1.419
6.346	7.954	5.91	2.377	5.322	2.255	1.067
7.742	6.687	5.147	5.739	5.011	3.111	0.531
6.307	4.853	2.487	3.508	4.812	4.582	1.121
3.473	0.256	0.196	0	0	0	0
2.216	0.111	0.285	0.447	0.046	0.002	0.004
11.852	17.449	6.562	8.253	5.513	4.852	1.11
18.081	13.943	9.412	11.437	9.003	10.438	14.658
9.11	15.368	11.925	6.918	11.461	8.394	9.756
11.811	19.161	6.786	9.759	8.566	6.034	7.08
4.302	8.571	1.02	2.196	8.848	3.551	3.336
6.068	6.993	2.962	2.486	3.219	1.386	1.556
4.775	1.356	1.266	2.158	0.283	0	0
17.099	8.738	6.36	9.864	5.123	4.467	1.974
17.349	25.981	12.59	18.464	19.994	17.387	19.136
10.003	8.947	4.861	4.298	2.138	1.864	0.217
7.95	20.544	9.949	9.138	10.716	10.458	6.708
5.421	9.849	6.286	5.968	6.041	5.376	1.616
2.656	14.81	1.345	3.091	7.437	1.614	8.062
18.426	18.476	10.217	11.145	13.271	16.238	10.325
3.018	10.728	5.581	3.116	4.526	1.179	0.207
11.062	17.518	7.883	7.973	6.826	6.507	1.559
10.983	16.889	7.078	4.207	4.164	4.061	1.251
2.461	5.932	1.985	4.48	1.268	3.143	0.112
4.53	3.761	4.865	2.506	2.275	1.484	1.654
13.079	21.45	14.85	5.418	19.601	6.539	11.607
10.766	19.824	12.909	13.56	9.475	4.377	1.891
6.236	16.233	3.089	4.346	9.696	5.365	8.062
6.782	21.328	6.294	22.862	7.348	13.371	4.345
4.039	3.696	1.923	1.75	6.023	2.089	2.707
3.389	2.548	0.469	1.444	0.951	0.486	0.163
5.179	15.854	8.053	6.139	10.512	3.88	1.939
8.793	7.806	5.673	4.503	5.211	3.107	5.281
12.109	30.428	9.249	18.368	19.573	16.937	9.588
5.104	17.58	9.677	12.674	10.052	9.472	5.015
7.723	8.755	5.707	8.971	2.634	4.333	5.544
7.2	5.517	7.24	6.591	3.659	2.711	2.741
20.425	17.313	16.368	11.464	11.047	12.107	18.154
8.383	11.264	3.404	8.598	7.172	5.2	0.743
11.855	14.947	5.863	8.159	4.178	4.974	0.782
10.579	16.817	7.646	7.472	9.889	7.756	3.576
24.373	29.015	16.493	19.584	27.932	7.376	19.666
4.745	9.197	3.855	1.468	1.691	0.093	1.297
0.811	2.27	1.856	3.474	1.675	0.736	0.002

4.826	9.43	4.32	4.552	2.767	1.613	0.25
5.444	12.895	1.186	2.062	1.309	0.302	0.68
10.325	17.686	5.934	6.351	6.863	6.351	2.641
8.81	8.696	6.488	1.654	1.669	1.324	0.325
3.826	6.062	1.645	2.537	2.902	1.819	1.782
16.646	17.205	14.342	9.942	17.832	14.949	20.593
8.843	12.442	5.787	6.817	9.149	6.839	7.428
12.64	16.699	15.209	9.673	12.929	8.228	4.479
8.06	7.177	7.382	2.215	1.314	1.379	0.642
1.842	7.189	2.806	4.505	3.687	1.013	1.357
12.967	20.449	10.49	11.681	19.012	4.534	16.298
6.458	13.88	6.24	12.271	2.787	5.533	3.404
8.376	13.255	7.145	13.41	7.231	5.505	4.451
8.819	9.615	3.004	6.406	10.009	1.587	2.912
1.152	6.741	1.061	1.18	5.676	0.984	0.801
1.339	6.494	1.9	3.633	2.778	2.635	4.178
4.354	16.684	0.589	3.955	11.84	7.775	5.684
2.745	9.799	1.978	2.82	4.017	0.495	3.303
1.841	5.962	0.134	4.311	0.557	1.64	0.206
2.745	9.78	1.979	2.828	4.017	0.495	3.293
8.073	6.529	3.077	5.113	9.156	1.055	0.148
16.09	13.235	8.054	7.375	9.577	8.28	7.217
6.558	14.023	6.373	10.343	9.082	13.034	3.611
7.752	11.255	8.468	8.684	7.107	6.669	6.091
6.062	5.347	2.783	4.314	3.781	2.092	4.61
15.324	19.923	13.149	14.331	15.254	12.026	7.937
3.231	7.146	3.083	2.963	2.675	0.7	4.756
13.013	8.797	6.142	2.405	0.346	0.125	0.029
11.708	9.687	8.599	4.873	8.106	3.597	4.931
8.726	6.709	2.298	6.022	3.357	8.012	5.482
23.868	22.5	20.757	18.113	20.306	8.743	10.691
5.645	5.84	4.015	5.227	4.869	7.71	1.595
0.457	2.376	0.011	0.222	0.002	0.005	0
7.717	6.43	6.389	5.419	4.314	3.464	3.493
14.488	23.307	9.125	16.311	13.558	17.568	12.452
7.957	7.026	2.757	5.584	3.113	3.062	3.774
9.43	14.646	6.555	7.455	9.764	9.124	4.977
5.285	6.895	3.663	4.521	5.832	1.864	1.958
12.103	6.313	2.801	8.804	9.618	3.1	7.887
13.9	17.303	6.184	12.539	8.632	10.075	10.137
6.124	6.46	4.91	6.175	6.035	3.435	2.827
9.31	12.445	9.508	7.44	9.867	7.916	4.373
5.139	12.09	2.259	6.208	11.726	7.407	6.404
6.942	4.296	4.356	5.822	11.221	6.006	4.669
5.548	7.072	0.988	4.008	7.59	4.901	3.544
16.64	22.473	12.386	22.443	14.065	9.274	8.351
5.456	6.851	2.901	8.142	5.721	6.085	3.238
8.809	12.712	3.772	8.593	8.833	5.473	1.084
10.012	10.932	6.275	7.602	2.806	3.315	0.411
5.826	13.982	6.553	8.673	7.532	5.311	3.907

7.447	13.293	7.208	12.614	4.499	6.972	5.144
5.193	7.009	4.433	3.639	1.729	3.22	1.404
5.858	6.518	2.865	5.905	5.057	4.139	4.726
13.056	33.333	14.24	17.163	17.503	17.302	9.132
5.243	1.6	2.659	0.894	0.159	0.076	0.013
4.188	6.68	6.371	1.73	4.118	0.444	0.574
12.028	5.972	4.339	4.915	5.405	9.242	1.178
4.132	8.52	3.213	5.934	10.175	4.161	8.254
1.307	11.604	3.432	3.712	6.229	2.136	3.958
6.406	8.186	10.603	6.527	7.197	5.319	4.057
4.264	9.326	2.053	3.301	6.037	2.558	4.873
4.603	12.624	2.746	7.391	4.37	3.246	1.919
7.283	11.765	3.856	5.858	8.027	4.329	3.957
19.739	10.766	6.517	10.149	4.823	5.19	2.081
7.181	7.744	13.238	3.986	9.376	3.093	2.915
5.244	9.802	3.41	4.604	13.423	5.982	5.598
0.876	3.736	0.587	0.296	0.601	0.032	1.2
12.241	16.312	9.848	11.02	14.726	15.526	11.765
23.522	22.638	20.364	23.671	23.872	9.077	14.635
8.129	21.336	8.926	5.987	10.64	6.083	4.887
44.668	49.39	35.321	47.7	44.182	36.9	24.023
17.068	10.614	12.879	13.225	9.69	6.901	4.355
8.975	17.322	6.137	4.909	6.953	6.636	4.749
9.146	6.651	1.719	7.651	6.405	6.755	4.771
5.012	4.161	4.041	7.932	2.886	3.414	2.319
13.005	6.272	7.654	8.903	8.946	4.274	5.591
9.063	7.846	6.919	9.096	5.67	6.191	3.509
9.433	6.809	2.566	5.781	0.954	4.094	7.185

RDF080m	RDF085m	RDF090m	RDF095m	RDF100m	RDF105m	RDF110m
4.387	0.46	1.356	0.561	0.115	0.045	0.093
3.236	9.084	2.765	1.956	4.036	2.97	2.679
5.998	2.393	4.572	1.628	3.142	0.219	0.075
0.072	0.023	0	0	0	0	0
0.057	1.808	2.054	1.445	0.109	0.184	0.001
0.003	0.114	0	0	0	0	0
10.9	8.082	6.525	9.188	4.198	5.069	5.372
0	0	0	0	0	0	0
8.574	0.736	0.12	0.031	0.001	0	0
0.998	3.994	1.194	1.549	1.73	0.53	1.175
19.789	18.981	11.959	9.799	7.083	7.684	6.489
22.539	5.677	17.737	8.876	8.74	7.825	8.594
2.253	1.626	1.612	1.107	0.222	0.153	0.006
5.713	5.001	7.193	1.586	3.568	1.899	0.59
2.081	1.84	1.951	0.215	0.005	0.001	0
8.766	4.255	4.425	3.512	2.121	2.898	1.018
0.134	0.084	0.006	0.006	0	0	0
13.652	26.945	18.664	6.62	5.702	5.117	7.006
2.401	4.948	1.391	1.531	1.402	1.701	1.631
4.06	4.756	1.773	2.118	1.386	1.225	0.937
1.791	1.535	1.926	0.453	1.046	0.118	0.086
3.913	0	0.235	0	0.006	0	0
7.328	1.241	0.983	2.494	3.478	8.84	1.309
4.854	5.706	4.67	3.381	1.453	0.632	3.29
8.691	6.817	6.416	3.965	3.472	1.38	2.365
3.068	4.763	2.603	4.547	1.599	1.913	2.468
2.07	6.48	2.174	2.678	3.763	0.774	2.779
22.863	23.99	17.69	13.922	14.154	14.295	16.173
4.477	2.808	3.6	3.47	1.467	0.152	0.976
3.597	7.154	4.212	2.685	2.782	3.887	2.581
11.613	16.301	18.783	7.412	7.179	9.138	5.87
10.215	15.333	19.05	8.626	13.257	10.254	15.803
3.164	1.531	4.334	2.616	0.863	1.593	0.176
4.584	2.038	1.998	0.485	0.138	0.012	0.005
8.724	8.625	5.499	4.176	2.808	1.582	0.343
5.828	7.156	6.147	3.723	5.323	6.763	6.839
3.127	2.918	1.896	1.007	0.36	0.62	0.005
0	0	0	0	0	0	0
9.908	6.125	8.461	5.777	2.554	6.232	2.534
5.798	4.068	3.361	3.512	3.845	3.297	2.459
11.371	9.775	11.579	5.859	8.393	4.174	5.16
0.208	0	0.005	0	0	0	0
11.819	15.579	15.458	8.427	6.165	8.429	11.023
2.538	1.1	1.327	6.581	2.081	0.493	0.038
5.273	10.597	5.785	7.144	5.826	4.852	3.921
5.591	7.906	6.622	5.771	3.315	7.172	2.805
10.703	9.725	4.673	4.676	1.829	3.246	2.259
7.306	10.932	7.128	5.416	4.043	6.533	9.205
11.373	7.583	11.312	8.079	3.595	7.324	5.63

9.191	7.412	8.019	8.1	5.415	8.984	7.528
7.839	10.042	8.569	10.282	3.856	7.408	6.762
18.427	18.005	18.206	16.552	10.103	19.149	11.535
11.282	7.293	6.723	8.457	4.134	7.476	4.532
9.421	4.026	3.075	0.895	0.878	1.37	0.773
6.903	4.693	8.312	3.55	2.273	8.599	4.939
7.581	13.897	11.942	9.159	5.713	10.12	6.281
13.872	12.161	16.137	12.519	3.386	13.96	16
5.415	6.319	8.035	2.697	1.056	5.242	0.475
13.304	15.147	7.892	10.126	6.348	8.757	5.025
11.515	7.243	11.474	8.207	2.976	7.362	2.482
15.511	5.088	4.071	4.199	3.189	1.436	1.206
9.857	3.004	2.454	3.692	2.736	0.647	1.704
6.985	7.024	9.514	5.188	5.858	4	6.075
10.165	8.929	7.99	3.086	3.853	2.457	1.817
6.424	5.605	0.932	4.263	1.871	4.32	0.496
4.029	2.114	1.689	2.409	2.09	2.349	0.365
4.795	8.591	5.315	4.979	6.373	5.401	4.273
0.016	0.17	0	0.005	0	0	0
5.75	2.912	5.236	0.072	0.006	1.156	0.201
3.262	2.117	4.666	0.593	0.465	0.01	0.003
1.031	1.529	0.825	3.22	0.461	0.248	0.183
5.643	3.191	3.136	0.373	0.237	0.002	0
7.56	9.499	0.508	1.205	0.004	0.044	0
1.33	1.255	0.354	0.015	3.039	0.263	0
0.95	3.687	1.803	1.514	2.88	0.552	2.56
4.863	7.704	3.031	3.77	2.682	4.997	3.188
2.751	3.729	8.691	3.102	5.693	4.792	3.971
0.261	1.275	0.119	0.82	0	0.033	0
1.701	8.175	2.966	0.104	1.216	1.74	1.936
10.677	8.59	2.138	10.348	4.865	3.821	3.005
1.108	0.131	0.001	0	0	0	0
6.617	4.976	4.689	4.871	4.01	0.286	0.378
0.022	0	0.001	0	0	0	0
9.708	3.98	1.38	3.348	0.567	0.249	0.013
0.091	0.212	0	0	0	0	0
3.937	2.198	0.166	1.178	0.354	0.012	0
2.32	1.347	1.151	0.203	0.034	0.01	0
4.869	2.698	1.917	4.356	2.633	0.864	0.937
3.949	0.214	0.024	0.006	0	0	0
0.36	0	0.021	0	0	0	0
5.246	4.332	8.514	4.526	0.128	0.098	0
1.499	0.234	1.234	2.063	0.04	0.001	0
0.273	2.429	0.041	0.078	1.874	0	0
4.812	5.35	5.341	2.065	4.929	0.994	2.343
19.809	16.239	29.436	20.116	20.187	18.783	9.562
9.185	8.879	4.695	0.855	0.38	0.564	1.003
2.272	0.913	0.153	0.233	0.012	0.004	0
1.241	4.711	0.198	1.493	0.112	0.257	0.008
40.684	40.788	36.555	30.178	27.831	28.337	16.403

6.671	14.016	10.793	15.947	10.726	9.739	4.954
13.189	2.504	5.275	5.248	5.474	3.775	8.112
4.475	9.115	5.384	6.399	3.177	1.67	2.474
1.259	6.202	2.142	4.214	0.611	0.495	1.053
2.663	2.517	2.49	0.54	0.236	0.028	0
0.588	0.422	0.028	0.015	0	0	0
19.593	23.383	14.228	14.171	6.394	12.54	6.35
0.758	4.708	6.653	4.219	2.484	1.584	5.272
2.756	2.873	3.835	1.41	2.458	1.289	0.614
1.703	0.251	0.005	0.001	0	0	0
2.534	4.874	4.067	3.229	4.346	4.41	2.954
5.785	8.298	2.701	0.304	2.028	4.606	0.122
5.855	4.762	5.249	3.243	5.254	3.369	2.843
6.097	15.57	8.537	2.662	3.952	13.254	10.833
0.584	3.083	0.098	0.014	0.038	0.007	0
3.194	0.27	0.338	0	0.19	0	0
1.89	1.026	1.991	2.045	2.531	1.78	0.107
0	0	0	0	0	0	0
9.786	9.439	10.58	6.582	5.81	3.051	4.57
1.342	3.36	0.482	0.26	0.856	1.324	0.085
0	0	0	0	0	0	0
13.366	4.358	10.215	5.206	8.219	11.617	3.193
1.967	2.522	8.193	2.552	0.041	0.396	0.681
15.361	14.886	9.466	7.541	5.581	5.157	4.752
0	0	0	0	0	0	0
0.507	0.905	0.045	0.057	0.001	0	0
1.034	1.509	4.734	0.304	0.345	0.053	0
0.909	4.573	2.055	0.628	0.957	0.235	1.411
13.83	15.651	8.194	6.506	6.263	7.045	4.512
13.42	9.2	11.792	6.392	6.593	7.626	16.778
8.166	10.502	11.587	11.056	7.159	10.533	7.289
5.034	5.592	3.304	2.845	3.381	1.3	0.452
7.179	12.753	5.001	6.287	0.871	5.23	4.217
0.14	0.281	0.007	0	0	0	0
0.17	0.254	0.005	0	0	0	0
1.085	0.632	0.102	0.3	0.668	0.104	0.007
5.215	17.966	6.216	8.22	6.999	12.377	9.921
3.502	3.024	3.317	3.909	0.365	2.248	0
3.021	3.114	1.77	1.484	3.23	0.837	3.741
11.983	7.977	7.912	7.23	5.662	3.301	3.482
3.009	1.321	0.402	0.719	0.001	0.014	0
1.225	0.001	0	0	0	0	0
4.297	1.818	0.679	0.135	0.076	0.009	0.004
2.935	7.33	3.14	6.694	1.866	5.092	2.552
1.912	1.307	0.178	0.089	0.012	0.005	0
2.213	0.538	0.037	0.025	0.003	0	0
5.572	7.238	6.028	4.409	4.657	5.129	2.744
2.859	2.193	0.641	0.872	0.099	0.042	0.061
0	0	0	0	0	0	0
2.721	6.515	3.778	2.551	1.138	0.414	0.776

1.599	0.885	1.041	0.031	0.161	0.001	0.007
3.86	7.478	5.18	1.295	3.728	5.093	3.664
1.291	6.416	1.802	2.688	1.142	0.272	1.72
7.671	0.012	0.026	0	0	0	0
10.709	6.869	8.026	5.294	6.907	2.238	5.106
2.909	7.343	3.502	2.514	4.336	2.589	0.371
1.238	4.221	3.509	2.645	2.704	2.53	6.514
1.047	0.33	0.034	0.058	0.003	0	0
0.819	0.201	0.032	0.007	0	0	0
1.351	1.094	0.54	0.102	0.038	0.002	0
0	0	0	0	0	0	0
0	0	0	0	0	0	0
0.511	0.449	0.155	0.081	0.011	0	0
8.215	2.421	4.76	4.081	2.698	0.193	0.278
7.625	14.182	7.129	5.803	3.532	9.798	4.168
6.213	4.16	2.245	3.836	2.068	0.47	0.29
1.941	6.425	6.444	0.746	1.08	1.396	2.916
0.653	6.493	1.521	1.796	1.711	0.631	1.355
0	0	0	0	0	0	0
7.651	1.62	0.347	0.129	0.003	0	0
11.571	19.433	12.341	12.369	12.713	10.488	12.295
0.025	0.087	0	0	0	0	0
7.076	7.702	6.319	3.08	1.271	3.729	0.296
2.129	1.739	2.056	1.2	2.023	1.541	0.316
0.86	5.901	2.635	2.141	1.516	3.065	5.527
13.749	7.688	6.694	3.813	4.34	1.656	0.477
0.585	0.931	0.345	0.083	0.015	0	0
3.685	1.541	1.584	0.385	1.119	0.112	0.125
1.631	0.156	0.207	0.027	0	0	0
1.798	3.818	0.934	2.201	0.297	0.288	0.032
1.61	1.122	1.047	0.102	0.007	0.012	0
2.493	6.817	13.55	2.318	6.995	7.384	12.341
1.707	1.062	0.233	0.01	0.001	0	0
2.023	5.275	0.641	0.361	0.102	0.007	0
5.362	2.228	3.335	0.507	0.152	0.099	0.008
1.875	2.914	2.268	0.083	2.856	0.001	0.035
0.132	0.018	0	0	0	0	0
1.2	7.871	2.95	0.093	1.167	1.746	2.025
2.165	0.946	0.977	0.791	0.061	0.115	0
9.533	16.42	11.204	9.565	14.221	14.664	16.629
2.658	6.307	3.721	2.471	1.196	0.434	0.79
3.769	2.53	7.3	2.849	3.252	4.695	1.63
2.12	1.514	1.205	0.36	0.136	0.016	0.005
4.674	2.268	8.086	4.153	0.373	0.067	0.007
4.031	0.02	0.02	0.001	0	0	0
1.246	0.512	0.112	0.048	0.003	0.003	0
2.671	1.902	2.191	0.695	1.711	0.78	2.411
15.068	9.036	17.009	10.688	13.724	8.679	9.924
0	0.088	0	0	0	0	0
0.739	0.91	0.089	0	0.395	0.098	0.006

0.17	0.006	0.003	0	0	0	0
0.011	0.045	0	0	0	0	0
2.893	1.479	3.547	0.433	0.301	0.536	0.86
0.114	0.01	0	0	0	0	0
0.924	3.872	3.212	2.561	1.479	0.916	1.34
10.982	11.558	13.532	6.105	5.909	4.095	5.202
5.198	5.764	2.062	3.719	2.123	0.643	0.488
1.091	3.371	1.673	0.455	0.197	0.076	0
1.858	4.192	0.332	0.084	0.069	0.051	0
1.085	5.217	2.863	0.502	3.566	2.052	1.82
6.995	7.067	8.05	4.114	9.606	3.359	2.588
7.132	10.344	2.444	7.505	3.009	1.233	2.256
1.656	4.607	2.076	3.874	1.77	1.982	0.237
6.15	3.65	3.952	1.456	1.59	0.441	1.432
1.388	5.283	1.867	0.856	0.992	0.523	1.79
2.29	1.479	0.578	2.715	0.121	0.119	0.006
3.366	5.269	3.488	0.766	4.084	5.418	3.478
1.286	4.328	0.677	1.327	1.397	0.449	3.366
0.343	0.015	0.002	0	0	0	0
1.287	4.328	0.679	1.335	1.397	0.449	3.354
1.874	0.346	0.031	0	2.46	0.277	0
4.172	4.409	3.383	6.036	4.204	5.713	4.838
5.414	2.704	0.606	0.386	0.027	0.006	0
5.189	4.046	2.31	1.1	1.196	0.1	0.02
1.496	3.094	3.157	2.071	2.149	1.247	6.692
7.02	9.448	9.876	8.737	8.977	7.72	5.28
0.217	4.891	0.126	0.011	0.002	0.001	0
0	0	0	0	0	0	0
5.845	4.46	5.672	2.536	4.979	8.498	7.25
4.997	2.67	1.177	0.904	0.088	0.003	0
15.497	11.84	9.363	12.03	11.284	12.282	9.232
3.056	2.375	1.059	0.199	0.072	0.003	0
0	0	0	0	0	0	0
2.636	6.156	1.871	1.967	0.339	0.405	0.07
8.124	7.957	9.851	12.426	4.075	6.462	5.503
4.004	2.696	3.397	2.839	1.458	2.288	0.011
6.222	8.963	4.89	5.576	6.459	5.005	2.513
1.42	0.031	0.016	0.046	0	0.001	0
1.454	2.252	0.788	0.13	0.362	0.24	0.053
3.237	8.277	9.002	3.181	4.338	4.054	5.637
1.418	1.107	0.23	0.185	0.007	0	0
6.976	9.132	3.58	3.145	4.201	3.252	0.366
3.509	10.014	5.342	2.476	2.666	3.495	0.705
3.282	2.989	0.953	1.314	0.226	0.175	1.108
1.744	2.16	0.32	0.308	2.329	0.661	0.057
7.622	3.373	1.564	0.622	0.215	0	0.001
2.814	3.644	2.776	0.832	3.929	4.911	1.321
4.032	0.213	0.024	0.006	0	0	0
0.033	0.125	0.086	0.012	0	0	0
2.348	3.696	1.933	1.782	0.099	0.071	0.007

5.178	11.063	3.041	8.113	3.677	4.648	2.817
1.75	0.332	2.153	0.332	0.034	0.014	0
2.782	2.651	0.345	1.459	0.454	0.135	0.003
5.116	10.2	4.595	5.849	1.319	2.044	2.331
0	0	0	0	0	0	0
0.112	0.001	2.334	0.002	0.013	0	0
4.32	2.874	0.002	0.012	0	0	0
1.497	3.917	5.075	1.626	0.662	2.993	1.755
0.655	7.514	4.567	1.79	1.21	5.144	8.523
4.687	4.736	0.752	2.014	0.606	1.491	1.19
0.872	1.409	2.453	0.377	1.456	1.231	0.238
0.702	1.453	1.19	0.168	0.101	0.007	0
7.446	1.909	10.544	2.149	2.777	5.856	7.853
7.424	1.492	0.175	0.11	0.003	0	0
2.626	1.989	1.276	0.231	0.041	0.011	0
2.294	9.646	5.173	6.057	3.765	5.179	5.177
0	0.085	0	0	0	0	0
6.489	9.157	8.25	9.07	4.076	3.372	4.049
15.278	12.801	12.235	13.798	10.599	12.217	7.617
5.493	6.03	6.102	6.923	6.08	5.32	14.472
31.775	28.401	24.568	29.983	29.519	22.807	19.448
3.907	1.519	2.516	0.37	0.491	0.013	0.011
1.399	1.321	0.4	0.187	0.006	0.006	0
3.674	2.792	2.27	2.039	1.216	1.962	1.074
2.097	3.205	1.197	0.13	0.269	0.03	0
4.898	7.728	4.542	2.759	6.046	9.032	6.67
4.889	0.72	0.658	0.781	0.507	0.148	0.019
4.911	2.74	2.579	1.567	0.158	0.43	0.181

RDF115m	RDF120m	RDF125m	RDF130m	RDF135m	RDF140m	RDF145m
0.001	0.001	0	0	0	0	0
2.219	2.338	2.11	1.553	1.147	0.351	0.728
0.063	0	0	0	0	0	0
0	0	0	0	0	0	0
0.002	0	0	0	0	0	0
0	0	0	0	0	0	0
0.701	3.039	0.514	0.238	0.161	0.183	0.179
0	0	0	0	0	0	0
0	0	0	0	0	0	0
0.183	0.088	0.014	0.002	0	0	0
2.805	1.459	2.204	1.848	0.323	0.009	0.002
4.457	3.463	2.305	3.231	1.279	0.316	0.019
0.007	0	0	0	0	0	0
0.171	0.022	0	0	0	0	0
0	0	0	0	0	0	0
0.138	0.035	0	0	0	0	0
0	0	0	0	0	0	0
6.181	5.405	2.078	2.575	0.168	0.026	0
0.103	1.268	1.38	1.807	0.305	0.123	0.098
0.081	0.102	0.012	0.004	0	0	0
0.007	0	0	0	0	0	0
0	0	0	0	0	0	0
0.9	0.437	0.838	0.012	0.034	1.781	0.195
2.598	0.236	0.221	0.002	0.01	0	0
0.124	0.231	0.009	0.008	0.002	0	0
1.279	0.04	0.132	0	0.045	0	0
1.566	1.543	1.517	3.62	0.966	2.078	2.283
5.092	5.657	7.332	5.821	2.766	1.631	3.136
0.483	0.027	0.013	0	0	0	0
3.894	0.866	1.701	1.757	2.125	0.637	3.219
2.728	4.185	1.173	1.741	0.13	0.131	0.005
20.746	30.774	9.798	4.609	5.259	5.016	5.82
0.083	0.009	0	0	0	0	0
0	0	0	0	0	0	0
0.176	0.008	0.001	0	0	0	0
2.196	3.067	7.683	5.291	2.352	4.133	3.317
0.013	0.001	0	0	0	0	0
0	0	0	0	0	0	0
2.992	3.956	0.547	4.109	0.787	5.094	3.574
0.75	1.328	0.107	0.105	0	0	0
3.527	1.275	0.231	0.097	0	0	0
0	0	0	0	0	0	0
6.023	4.551	7.855	8.19	5.414	3.689	1.613
3.649	0.062	0	0	0	0	0
3.433	3.111	2.462	1.413	1.826	0.196	0.288
0.007	0.006	0	0	0	0	0
0.101	0.375	0.007	0.011	0.001	0	0
3.81	0.47	3.907	2.059	2.55	4.627	4.71
3.758	3.564	2.82	4.989	2.105	1.037	6.222

6.531	2.467	4.013	4.196	2.468	1.951	0.946
9.289	7.993	3.602	3.134	1.409	3.667	0.116
12.477	6.99	15.922	7.207	11.545	10.469	7.275
6.618	3.534	3.587	4.991	2.061	2.556	2.031
0.027	0.161	0	0.001	0	0	0
1.982	4.484	2.924	0.91	0.468	0.128	0.014
3.347	1.75	7.098	7.906	7.286	6.064	1.796
13.652	5.173	6.3	10.406	5.084	1.973	3.034
0.404	0.055	0.015	0.001	0	0	0
9.145	6.768	8.399	7.63	4.315	2.077	8.543
5.515	2.547	2.476	4.126	3.012	1.397	0.571
0.032	0	0	0	0	0	0
0.004	0.152	0.001	0.001	0	0	0
2.534	4.342	3.739	2.277	2.452	2.398	1.229
2.603	2.212	4.225	1.001	1.06	1.097	1.026
2.461	0.055	0.123	0	0	0	0
0.223	0.114	0.006	0.003	0	0	0
5.616	0.423	4.378	0.706	0.224	0	2.874
0	0	0	0	0	0	0
0	0	0	0	0	0	0
0	0	0	0	0	0	0
0	0	0	0	0	0	0
0	0	0	0	0	0	0
0	0	0	0	0	0	0
0	0	0	0	0	0	0
0.858	0.232	0.024	0	0	0	0
3.958	2.492	2.729	2.798	1.645	0.624	0.491
8.97	4.315	5.065	0.665	1.97	1.676	1.678
0	0	0	0	0	0	0
0.087	0.173	0	0.004	0	0	0
2.43	1.558	1.343	1.597	0.374	0.188	0.007
0	0	0	0	0	0	0
0.045	0.013	0	0	0	0	0
0	0	0	0	0	0	0
0.002	0	0	0	0	0	0
0	0	0	0	0	0	0
0	0	0	0	0	0	0
0	0	0	0	0	0	0
0.229	0.004	0.009	0	0	0	0
0	0	0	0	0	0	0
0	0	0	0	0	0	0
0	0	0	0	0	0	0
0	0	0	0	0	0	0
0	0	0	0	0	0	0
1.824	2.213	0.042	0.553	0	0	0
21.592	14.254	15.474	16.607	6.232	14.859	10.479
0.016	0.094	0	0.004	0	0	0
0	0	0	0	0	0	0
0.015	0	0	0	0	0	0
13.898	6.75	10.038	3.605	2.638	0.308	0.135

8.844	9.382	3.204	4.181	7.754	3.709	3.371
6.07	7.659	3.863	4.228	3.389	6.088	2.753
0.204	0.385	0.015	0.019	0.002	0	0
0.358	0.071	0.029	0	0	0	0
0	0	0	0	0	0	0
0	0	0	0	0	0	0
2.946	5.895	2.867	5.814	0.273	1.431	2.102
6.579	0.154	3.61	0.072	0.574	0.02	0
0.918	0.171	0.198	1.008	0.107	0.029	0
0	0	0	0	0	0	0
4.507	2.697	1.585	0.826	0.827	0.157	0.208
3.617	0	0.382	0	0	0	0
0.716	1.075	0.684	0.606	0.047	1.1	0.042
0.147	1.115	1.752	0.001	0.198	0	0
0	0	0	0	0	0	0
0	0	0	0	0	0	0
0.337	0.046	1.797	0	0.07	0	0
0	0	0	0	0	0	0
6.254	2.002	0.576	3.531	0.344	0.123	0.011
0.093	0.007	0	0	0	0	0
0	0	0	0	0	0	0
9.552	4.859	4.809	10.543	6.299	14.144	12.569
0.306	0.028	0.001	0	0	0	0
5.533	1.81	2.701	4.362	2.031	3.464	1.818
0	0	0	0	0	0	0
0	0	0	0	0	0	0
0.007	0	0	0	0	0	0
0.024	0.038	0	0	0	0	0
2.915	2.459	1.303	0.196	0.069	0.014	0.005
11.014	7.731	4.378	9.301	7.881	7.847	4.249
13.38	8.629	5.08	12.725	10.768	2.032	1.612
0.201	0.023	0.009	0	0	0	0
1.807	1.663	0.14	0.006	0	0	0
0	0	0	0	0	0	0
0	0	0	0	0	0	0
0.001	0	0	0	0	0	0
2.59	8.548	6.946	9.133	6.054	7.28	5.848
0.234	0	0	0	0	0	0
0.163	0.324	0.102	0.043	0.014	0	0
2.678	1.503	0.794	0.272	0.053	0	0
0	0	0	0	0	0	0
0	0	0	0	0	0	0
0	0	0	0	0	0	0
2.199	1.093	0.303	0.092	0.002	0.001	0
0	0	0	0	0	0	0
0	0	0	0	0	0	0
3.924	2.55	1.315	3.459	2.864	0.396	0.027
0	0	0	0	0	0	0
0	0	0	0	0	0	0
1.796	0.372	0.166	0.659	0.143	0.01	0

0	0	0	0	0	0	0
1.216	1.368	1.836	1.183	1.635	0.232	0.223
3.145	0.178	0.238	0.003	0.007	0	0
0	0	0	0	0	0	0
2.699	0.261	0.919	0.105	0.067	0.003	0
1.072	0.337	0.095	0.011	0.003	0.002	0
3.535	0	0.021	0	0	0	0
0	0	0	0	0	0	0
0	0	0	0	0	0	0
0	0	0	0	0	0	0
0	0	0	0	0	0	0
0	0	0	0	0	0	0
0	0	0	0	0	0	0
0.001	0.003	0	0	0	0	0
5.916	4.447	4.408	3.209	4.364	5.54	2.419
0.313	0	0.279	0.033	0	0	0
0.111	1.272	0.152	0.011	0	0	0
0.885	0.307	1.429	2.206	0.302	1.067	1.735
0	0	0	0	0	0	0
0	0	0	0	0	0	0
8.125	15.869	24.392	14.974	8.749	9.016	5.73
0	0	0	0	0	0	0
1.84	0.104	0.1	0.001	0	0	0
0.012	0.135	0.103	0.012	0	0	0
2.698	1.108	0.399	0.174	0.015	0.004	0
0.084	0.011	0	0	0	0	0
0	0	0	0	0	0	0
0	0	0	0	0	0	0
0	0	0	0	0	0	0
0.017	0.001	0	0	0	0	0
0	0	0	0	0	0	0
13.871	3.152	3.939	15.16	3.693	6.717	1.754
0	0	0	0	0	0	0
0	0	0	0	0	0	0
0	0	0	0	0	0	0
0	0	0	0	0	0	0
0	0	0	0	0	0	0
0.08	0.074	0	0	0	0	0
0.006	0	0	0	0	0	0
8.764	12.806	10.163	4.071	4.201	7.901	8.308
1.785	0.403	0.193	0.656	0.141	0.01	0
5.042	1.373	1.711	0.5	1.314	1.117	0.131
0	0	0	0	0	0	0
0	0	0	0	0	0	0
0	0	0	0	0	0	0
0	0	0	0	0	0	0
0.073	0.001	0.207	0.062	0	0	0
11.043	12.633	5.779	11.091	3.411	4.816	4.202
0	0	0	0	0	0	0
0	0	0	0	0	0	0

0	0	0	0	0	0	0
0	0	0	0	0	0	0
0.085	0.027	0.006	0.001	0	0	0
0	0	0	0	0	0	0
0.67	0.615	0.044	0.027	0	0	0
6.955	1.869	0.462	2.107	0.503	3.358	0.057
0.078	0.007	0.003	0	0	0	0
0.001	0	0	0	0	0	0
0.001	0	0	0	0	0	0
1.682	0.749	1.103	1.602	0.224	0.033	0.215
1.982	0.677	1.826	0.826	1.36	0.033	0.084
2.382	8.512	2.179	1.583	1.173	2.134	0.08
0.144	0.004	0	0	0	0	0
3.05	0.216	0.003	0.006	0	0	0
0.049	0.763	0.116	0.014	0.001	0	0
0	0	0	0	0	0	0
0.156	2.842	1.624	0.736	1.316	1.102	0.555
0.997	1.667	0.141	0.278	0.001	0.007	0
0	0	0	0	0	0	0
0.997	1.667	0.141	0.277	0.001	0.007	0
0	0	0	0	0	0	0
3.278	2.269	1.429	0.1	0.186	0.073	0.007
0	0	0	0	0	0	0
0.003	0	0	0	0	0	0
2.281	5.601	2.402	1.661	0.36	0.698	0.501
7.395	4.418	5.328	1.901	0.458	0.22	0.01
0	0	0	0	0	0	0
0	0	0	0	0	0	0
4.382	4.704	3.886	1.558	3.714	5.782	2.228
0	0	0	0	0	0	0
7.738	13.214	1.932	0.943	1.531	0.289	0.077
0	0	0	0	0	0	0
0	0	0	0	0	0	0
0.107	0.014	0	0	0	0	0
7.191	1.786	2.843	2.381	2.787	0.457	1.051
0.048	0	0	0	0	0	0
0.846	0.437	0.779	0.196	0.054	0.006	0
0	0	0	0	0	0	0
0	0	0	0	0	0	0
2.653	1.514	0.935	2.146	0.727	0.165	0.092
0	0	0	0	0	0	0
1.143	2.373	0.279	0.141	0	0.009	0
0.287	1.204	0.12	0	0	0	0
0.198	0.004	0	0	0	0	0
0.003	0.001	0	0	0	0	0
0	0	0	0	0	0	0
2.529	2.274	1.137	1.761	1.192	0.95	1.227
0	0	0	0	0	0	0
0	0	0	0	0	0	0
0.003	0	0	0	0	0	0

1.551	5.868	1.925	3.764	2.162	2.783	0.288
0	0	0	0	0	0	0
0	0	0	0	0	0	0
0.196	0.411	0.013	0.022	0.001	0	0
0	0	0	0	0	0	0
0	0	0	0	0	0	0
0	0	0	0	0	0	0
0.375	0.077	0.09	0	0.008	0	0
0.734	1.601	7.783	5.179	1.717	1.352	2.579
0.85	0.056	0.022	0.008	0	0	0
0.027	0.085	0.001	0	0	0	0
0	0	0	0	0	0	0
7.047	6.683	5.49	0.782	2.717	0.539	1.773
0	0	0	0	0	0	0
0	0	0	0	0	0	0
3.849	5.992	2.865	6.117	4.012	3.987	0.672
0	0	0	0	0	0	0
2.683	0.838	0.666	0.174	0.011	0.011	0
7.814	11.987	5.634	2.125	3.227	2.433	0.398
7.951	7.748	5.754	6.666	8.953	2.755	5.003
21.576	14.275	12.052	6.173	2.329	5.16	1.038
0	0	0	0	0	0	0
0	0	0	0	0	0	0
0.656	0.388	0.187	0.089	0.038	0	0
0	0	0	0	0	0	0
8.226	7.624	0.979	2.509	1.462	5.146	3.906
0.363	0.15	0.014	0	0	0	0
0.011	0.02	0.208	0	0	0	0

RDF150m	RDF155m	RDF010v	RDF015v	RDF020v	RDF025v	RDF030v
0	0	2.148	8.981	1.446	10.464	2.125
0.846	0.253	3.942	12.501	3.09	19.006	2.651
0	0	2.213	10.474	1.5	19.548	4.745
0	0	1.362	3.017	1.369	5.112	0.718
0	0	1.666	4.385	2.415	6.505	1.07
0	0	1.896	5.572	1.327	5.17	5.098
0.056	0.001	4.39	15.052	3.337	25.738	11.257
0	0	0.699	2.213	0.755	2.251	0.72
0	0	1.926	8.347	1.592	17.598	8.819
0	0	3.308	9.182	2.794	14.172	1.47
0	0	6.398	25.559	7.854	38.501	11.521
0.006	0	4.009	15.265	3.53	22.4	13.574
0	0	3.28	11.992	2.508	21.604	4.809
0	0	3.635	11.694	3.101	19.715	5.8
0	0	2.854	11.841	3.696	19.951	7.126
0	0	2.875	11.745	3.354	19.056	3.728
0	0	1.748	4.382	0.963	7.814	3.839
0	0	3.083	15.235	2.627	24.58	4.798
0.087	0.006	3.22	9.227	2.829	13.815	1.274
0	0	3.192	13.162	1.995	21.199	7.246
0	0	2.744	9.704	1.293	17.672	7.687
0	0	1.062	3.913	1.093	5.013	2.086
0	0	1.779	7.545	1.225	15.225	3.756
0	0	2.595	11.058	2.934	17.441	4.753
0	0	4.767	15.054	2.897	24.98	5.907
0	0	3.954	22.33	3.074	42.311	13.098
0.361	2.761	4.032	13.342	2.731	15.389	2.131
4.094	1	5.242	20.925	4.352	26.797	10.115
0	0	3.305	9.842	1.965	15.464	6.483
0.636	0.885	4.298	12.133	3.178	16.326	1.946
0.004	0	4.188	12.306	2.857	21.668	4.698
1.861	2.424	5.686	29.081	4.323	45.035	20.19
0	0	3.234	10.911	1.938	17.583	2.709
0	0	3.859	13.155	2.373	21.537	6.736
0	0	5.509	28.092	3.794	51.145	20.405
2.726	0.4	4.207	17.292	2.321	27.563	7.331
0	0	3.938	19.305	2.856	34.219	12.077
0	0	1.364	3.283	0.953	7.661	2.878
2.393	2.642	5.962	24.783	3.62	45.003	14.713
0	0	2.29	12.565	1.628	20.054	5.666
0	0	2.997	11.636	3.266	24.928	9.05
0	0	1.962	7.913	2.489	12.319	7.694
6.308	4.874	6.46	34.199	3.784	70.721	33.643
0	0	1.811	7.755	1.269	12.526	3.573
0.093	0.009	3.862	12.16	3.572	17.23	2.431
0	0	2.208	9.873	2.935	15.262	3.804
0	0	2.586	10.941	4.066	16.704	4.549
3.531	1.748	2.598	11.4	4.108	17.675	5.828
1.554	3.01	1.964	9.414	3.901	15.177	6.297

0.646	0.819	3.404	14.391	4.124	23.261	9.633
0.421	0.002	2.294	10.424	3.88	18.435	4.844
9.694	7.347	3.92	18.078	4.339	29.099	11.19
2.369	1.005	2.482	10.888	3.913	18.088	5.152
0	0	2.53	10.234	3.667	18.287	7.072
0.01	0	2.901	11.861	4.071	18.777	4.481
1.889	2.169	3.079	14.267	4.219	22.792	9.75
0.272	1.237	3.272	14.037	3.532	24.792	7.673
0	0	1.968	8.481	2.941	15.22	4.728
7.53	3.213	2.675	11.508	4.041	20.61	6.071
0.653	0.136	2.397	10.404	3.382	14.397	4.992
0	0	2.197	8.372	0.908	16.418	7.08
0	0	2.607	10.798	3.903	16.688	5.233
1.162	0.029	2.533	10.856	3.424	18.53	5.969
0.027	0.108	2.285	10.504	3.502	16.406	5.916
0	0	2.824	13.305	3.958	19.26	8.422
0	0	4.203	16.314	3.039	30.95	11.751
0.001	0	3.557	13.195	2.862	21.034	3.141
0	0	1.043	3.228	1.052	7.811	2.879
0	0	2.724	8.326	1.684	15.298	4.646
0	0	2.699	8.103	1.701	16.256	4.63
0	0	1.946	4.764	1.429	8.406	1.966
0	0	2.591	9.245	2.149	17.574	4.38
0	0	1.815	7.76	1.112	11.467	2.607
0	0	1.723	7.409	1.313	9.12	2.642
0	0	2.432	4.911	2.269	6.98	2.551
2.251	1.168	2.705	9.087	1.88	15.62	3.283
0.34	1.24	3.269	12.305	2.327	23.118	2.461
0	0	1.393	7.352	1.045	11.966	1.43
0	0	2.554	13.099	1.895	19.019	3.095
0.015	0	4.463	16.804	3.134	26.509	11.964
0	0	1.572	4.815	1.269	8.296	1.896
0	0	2.316	9.672	1.807	15.196	3.497
0	0	1.384	4.249	1.136	4.162	3.435
0	0	2.756	10.133	2.354	20.562	3.968
0	0	2.068	6.697	1.526	8.132	5.316
0	0	2.02	4.242	1.531	8.867	1.24
0	0	3.195	10.262	2.428	18.609	5.486
0	0	3.954	22.281	2.858	42.143	14.051
0	0	1.931	7.757	1.294	16.042	6.676
0	0	1.703	5.711	1.715	10.489	5.506
0	0	2.417	13.345	3.146	18.888	7.11
0	0	1.71	6.741	1.891	8.353	2.635
0	0	1.225	5.65	1.047	12.934	2.096
0	0	4.559	23.855	2.824	46.006	21.27
5.845	20.219	8.561	42.021	6.345	71.389	29.165
0	0	3.667	11.621	2.416	19.829	6.688
0	0	3.039	8.398	2.005	14.867	1.973
0	0	4.197	13.114	3.024	26.487	13.509
0.004	0.002	7.239	35.339	6.702	61.763	27.524

0.732	0	3.901	10.12	2.635	17.658	6.283
2.662	3.413	3.487	13.045	2.514	21.941	6.304
0	0	3.415	17.874	3.821	38.307	10.881
0	0	2.197	8.444	1.741	18.146	3.63
0	0	3.346	9.541	3.083	12.979	3.453
0	0	2.934	12.202	1.959	18.99	7.498
0.455	0.885	4.373	21.597	4.089	32.351	8.005
0	0	2.474	4.275	2.102	12.221	3.644
0	0	2.1	8.254	1.395	16.421	1.881
0	0	2.094	6.215	1.29	11.186	2.86
0.011	0.011	3.975	13.352	2.321	22.463	6.968
0	0	1.828	7.423	1.549	16.413	5.61
0	0	4.96	22.289	2.709	44.018	13.483
0	0	2.868	12.692	2.59	18.355	1.88
0	0	1.773	6.653	1.769	12.962	4.058
0	0	1.405	8.295	0.805	12.498	4.342
0	0	1.97	7.97	1.349	15.368	7.439
0	0	0.547	0.984	1.284	1.433	0.008
0.002	0	3.634	14.451	2.792	24.738	5.594
0	0	1.937	7.236	1.201	15.098	4.507
0	0	0.418	0.984	0.346	0.703	0.634
2.946	12.318	6.332	28.109	3.385	47.67	14.388
0	0	1.766	5.993	1.18	11.921	1.555
3.37	0.262	6.182	30.103	4.468	55.551	25.68
0	0	2.418	8.987	2.052	16.81	6.113
0	0	2.895	13.181	3.446	21.604	6.439
0	0	1.936	5.608	2.851	8.331	1.294
0	0	3.01	9.535	1.613	18.19	4.178
0	0	5.856	21.769	5.927	33.733	9.777
2.584	5.098	4.805	17.503	3.241	28.352	9.582
2.429	3.05	4.076	14.05	3.085	26.603	5.336
0	0	3.295	13.989	2.623	23.158	8.073
0	0	2.527	9.932	1.84	15.48	2.231
0	0	1.34	4.38	0.869	7.805	3.778
0	0	2.587	15.573	1.969	25.504	11.593
0	0	2.503	8.175	1.27	15.383	4.765
3.695	3.131	4.561	15.585	3.935	30.513	3.486
0	0	2.316	9.231	1.677	14.485	3.89
0	0	2.207	9.045	1.518	14.018	6.916
0	0	3.983	16.98	2.239	31.366	9.436
0	0	1.076	6.323	0.803	6.781	2.336
0	0	1.207	6.214	0.972	6.391	2.176
0	0	3.328	11.577	2.471	20.778	5.393
0	0	1.99	8.338	1.476	16.214	4.598
0	0	2.099	8.195	1.352	16.101	4.572
0	0	1.634	4.225	1.689	6.449	2.594
0.428	0.003	2.066	7.267	1.53	11.587	2.465
0	0	1.928	7.522	1.214	16.888	3.303
0	0	1.986	6.808	1.217	7.534	6.804
0	0	2.722	13.337	2.058	22.321	4.351

0	0	3.062	8.605	2.07	15.283	6.226
1.499	0.094	2.801	10.547	2.157	14.507	5.464
0	0	2.662	12.249	2.137	20.66	3.242
0	0	1.569	7.462	1.703	13.884	7.301
0	0	3.317	12.463	3.534	24.234	6.859
0	0	1.917	6.286	1.631	14.949	6.064
0	0	2.649	8.46	1.962	15.358	4.227
0	0	2.922	10.375	1.885	18.376	6.3
0	0	2.699	10.716	2.165	18.844	5.555
0	0	3.062	10.417	2.025	18.035	5.139
0	0	0.638	2.955	0.789	3.166	0.933
0	0	1.747	1.753	1.852	4.369	1.246
0	0	3.843	12.518	2.066	23.918	9.324
0	0	2.811	11.048	3.522	18.052	4.764
3.376	0.39	3.36	12.039	3.365	23.463	3.964
0	0	4.087	21.784	2.9	39.744	14.105
0	0	3.15	8.69	2.414	15.78	4.135
0.3	0.113	3.5	9.686	2.753	14.034	1.797
0	0	1.232	3.866	1.274	5.346	1.676
0	0	1.926	8.578	1.267	16.933	10.065
3.033	1.086	5.172	20.784	3.06	34.975	10.307
0	0	2.634	14.87	2.958	24.288	10.937
0	0	3.327	17.043	2.347	25.123	7.391
0	0	3.38	9.718	1.753	15.828	4.978
0	0	3.75	13.477	3.146	23.137	4.239
0	0	3.151	13.745	3.408	23.609	5.848
0	0	1.683	7.135	1.042	12.963	1.725
0	0	3.431	19.269	2.807	28.641	12.707
0	0	2.951	16.523	2.644	26.688	12.405
0	0	2.02	6.742	1.506	13.846	2.793
0	0	2.63	5.11	1.686	10.636	3.769
2.176	0.881	3.635	16.269	2.888	29.101	10.004
0	0	2.564	9.257	2.108	19.071	2.741
0	0	1.856	4.909	1.118	9.598	5.227
0	0	2.83	10.174	2.152	19.253	4.173
0	0	0.94	4.349	0.801	4.538	2.264
0	0	1.695	4.608	1.944	7.805	1.204
0	0	2.554	11.274	2.166	18.729	3.327
0	0	3.087	11.366	2.692	19.883	4.178
6.444	7.044	5.132	19.54	4.573	36.991	8.095
0	0	2.722	13.299	2.075	22.195	4.335
0.734	0.046	2.682	7.342	1.903	13.78	4.388
0	0	2.438	9.938	1.601	17.128	5.442
0	0	2.733	13.313	3.303	19.131	5.552
0	0	1.727	7.462	1.782	14.837	6.774
0	0	2.853	16.166	2.277	26.512	11.422
0	0	2.896	9.673	1.546	16.371	12.095
1.31	1.378	7.992	35.072	4.403	67.816	23.445
0	0	2.524	8.589	2.023	16.885	7.169
0	0	1.889	4.24	1.523	6.131	0.388

0	0	3.447	13.554	2.145	23.865	8.111
0	0	1.849	6.505	2.028	13.322	4.868
0	0	2.917	9.548	1.379	16.057	11.628
0	0	1.967	6.775	1.763	13.288	3.799
0	0	2.917	8.692	2.319	11.131	1.706
0.006	0.001	4.565	15.737	3.148	28.133	7.948
0	0	3.032	11.24	2.519	18.67	8.647
0	0	2.689	9.208	1.249	14.234	4.003
0	0	1.996	8.146	1.221	10.315	5.353
0.165	0	3.163	8.548	2.729	12.229	1.455
0.111	0	4.891	21.305	4.058	35.434	11.518
0.302	0.001	3.034	9.84	2.39	17.389	2.111
0	0	3.558	21.33	2.305	33.434	11.908
0	0	4.291	21.387	2.276	37.311	15.08
0	0	3.066	8.229	2.259	14.696	3.255
0	0	2.877	7.961	1.89	13.378	3.145
1.453	0.803	3.895	12.679	2.907	19.539	2.972
0	0	3.042	9.003	2.475	15.122	1.053
0	0	2.472	7.268	1.086	13.908	4.797
0	0	3.042	9.006	2.475	15.122	1.051
0	0	2.055	5.768	1.53	11.998	2.674
0	0	3.526	12.307	3.13	20.14	6.035
0	0	3.393	13.837	2.993	27.772	6.063
0	0	3.393	13.851	3.074	28.266	6.895
0.209	2.611	3.119	7.916	2.295	11.01	4.008
0.007	0	5.031	18.06	3.307	31.218	7.941
0	0	0.853	2.937	0.809	6.522	0.593
0	0	1.582	5.202	1.149	6.969	7.083
1.339	1.777	3.668	17.485	2.209	27.358	6.641
0	0	2.731	8.72	2.055	12.712	2.744
0.007	0.004	7.132	31.921	5.268	58.937	23.027
0	0	3.593	12.242	2.225	20.398	5.235
0	0	0.91	2.793	0.904	5.837	0.337
0	0	2.927	13.646	1.912	21.937	8.153
0.01	0.081	4.124	14.902	2.856	22.888	4.768
0	0	2.892	7.79	2.159	13.211	3.007
0	0	4.192	13.863	3.208	28.175	8.927
0	0	1.761	4.399	1.704	7.661	2.959
0	0	2.303	5.335	1.542	11.37	5.06
0.046	0	3.04	9.51	1.611	16.422	12.17
0	0	2.002	5.317	1.869	8.163	2.908
0	0	2.469	10.867	1.647	16.214	7.046
0	0	3.308	10.559	1.831	18.496	3.789
0	0	3.005	9.06	1.893	11.971	3.819
0	0	1.784	6.917	1.026	15.516	4.439
0	0	4.082	10.883	2.829	18.319	4.294
0.079	0.689	3.413	9.839	3.006	15.337	2.193
0	0	1.936	8.07	1.533	16.364	7.302
0	0	2.033	5.464	1.669	8.795	2.794
0	0	1.676	5.193	1.491	12.476	4.977

0.199	0.028	3.493	12.369	2.637	19.157	4.618
0	0	2.686	7.976	2.158	14.947	2.169
0	0	3.564	20.056	2.535	31.644	14.28
0	0	3.415	17.943	3.728	38.251	9.562
0	0	1.155	2.66	0.971	5.592	1.852
0	0	2.519	8.602	2.089	16.559	7.14
0	0	2.009	7.687	1.28	15.077	4.423
0	0	3.269	10.825	2.532	17.202	2.502
2.435	1.428	3.584	10.367	3.368	16.985	1.818
0	0	3.009	8.785	2.563	14.656	6.051
0	0	2.611	6.645	1.694	11.926	2.799
0	0	3.427	11.533	2.224	22.357	6.095
0.1	0.131	3.127	11.107	2.429	17.843	2.778
0	0	1.769	8.37	1.513	16.661	10.397
0	0	2.695	5.884	2.557	11.624	1.095
3.1	1.174	4.045	11.966	3.046	19.897	3.477
0	0	2.138	6.521	1.228	9.812	3.725
0	0	4.127	15.508	3.404	30.816	9.381
0.122	0.006	7.593	34.515	4.77	65.176	21.9
4.409	5.409	5.31	15.976	4.344	29.766	10.646
2.163	0.272	7.342	36.854	7.177	62.871	27.585
0	0	3.585	17.067	2.194	29.402	10.469
0	0	2.365	9.806	1.643	18.218	4.634
0	0	2.282	8.097	1.073	15.677	3.972
0	0	2.014	7.418	1.981	10.547	3.792
0.145	5.009	3.003	13.658	2.234	20.818	6.677
0	0	2.97	9.568	1.788	19.532	3.517
0	0	2	8.116	1.278	13.001	3.342

RDF035v	RDF040v	RDF045v	RDF050v	RDF055v	RDF060v	RDF065v
2.493	2.853	6.793	2.889	2.959	3.366	1.759
3.121	6.56	4.792	14.387	3.059	7.158	8.59
7.597	5.077	7.188	17.213	4.554	8.25	9.708
1.487	1.726	1.437	1.661	0.398	1.267	1.164
3.764	3.718	5.391	3.195	2.601	2.587	0.669
2.005	3.188	3.988	2.657	1.833	0.727	1.99
12.998	12.241	15.541	17.033	14.954	10.214	12.264
1.892	1.237	2.276	0.19	0.769	0.076	0.063
6.037	8.657	5.092	10.856	4.196	6.983	4.853
3.259	5.626	6.74	9.549	3.379	6.579	5.2
10.454	17.283	21.474	24.938	17.774	27.583	32.116
7.851	8.635	19.583	12.338	15.56	22.355	17.902
6.321	7.198	13.173	12.41	9.922	8.22	7.509
7.978	13.715	11.435	15.581	8.259	16.508	12.966
8.145	10.229	9.15	11.351	7.939	9.827	8.544
7.186	7.539	9.105	9.713	6.88	5.496	4.562
3.494	2.688	3.385	3.573	1.592	2.145	1.068
5.796	10.452	14.583	20.786	16.044	18.413	16.301
2.2	4.813	7.745	7.664	2.432	2.778	4.486
7.326	7.007	7.274	7.589	4.254	7.23	6.477
6.532	4.253	9.06	12.15	11.087	9.032	7.453
5.23	7.348	5.994	6.954	2.71	4.643	1.127
7.31	5.428	5.663	7.275	3.229	9.1	1.535
5.818	5.401	9.988	8.103	6.869	3.619	3.612
8.911	10.608	16.167	13.413	16.271	12.858	19.177
18.391	15.483	15.513	18.12	10.344	11.74	11.726
2.952	6.579	7.128	10.103	4.546	3.404	4.477
13.268	11.753	19.807	21.154	24.044	22.072	24.883
6.874	5.85	10.7	6.581	8.242	9.971	8.414
2.663	6.812	9.771	8.228	6.066	5.483	8.963
11.409	15.26	13.763	24.512	18.971	23.04	17.958
17.824	16.816	24.927	37.074	21.932	26.249	30.093
4.842	3.987	8.707	7.275	5.462	5.81	3.636
4.668	9.847	8.702	9.684	7.301	9	12.007
20.497	21.044	28.181	26.191	20.393	18.516	22.81
8.074	9.984	13.639	8.725	6.505	7.782	10.416
9.008	12.532	11.097	21.955	7.847	9.038	9.385
4.819	2.834	3.642	1.77	3.356	2.059	1.689
15.41	13.642	18.081	21.735	9.353	11.409	15.079
6.774	4.124	5.666	9.476	5.467	8.67	8.097
11.191	11.568	14.798	15.101	13.163	14.857	15.555
5.408	4.838	8.59	5.474	6.293	4.585	2.459
24.146	23.857	27.464	36.871	16.548	18.204	28.611
5.687	4.234	5.17	6.988	2.651	9.253	3.421
4.373	7.994	8.397	12.312	9.799	15.43	11.462
5.549	5.29	7.114	8.752	6.02	6.258	6.074
6.514	5.561	9.248	10.958	6.836	5.175	5.947
7.626	8.295	10.238	11.437	6.964	8.819	9.905
7.65	5.988	8.957	9.524	3.254	7.112	7.671

10.087	8.653	13.617	14.459	7.383	7.379	9.805
6.314	6.916	8.84	11.508	6.451	6.922	7.56
12.768	11.856	13.522	19.945	12.688	17.35	19.224
6.403	7.301	8.027	12.658	5.571	6.893	10.594
6.364	10.256	12.679	12.709	10.024	12.215	14.318
6.552	6.793	11.122	11.133	5.163	7.679	9.394
10.15	8.555	15.401	15.026	7.562	9.692	12.124
9.363	9.792	10.314	18.975	11.18	11.746	14.267
5	5.676	7.471	9.656	5.017	7.338	7.835
9.43	10.506	9.954	14.14	9.37	8.445	9.525
5.053	7.37	8.032	11.15	4.722	6.586	8.125
6.876	5.619	9.516	7.717	5.924	6.355	7.421
6.236	5.704	9.882	10.841	3.544	9.629	8.387
7.395	5.44	12.534	10.063	6.608	6.241	8.784
6.054	7.01	8.609	9.729	2.542	6.054	8.087
7.414	5.176	9.702	11.847	6.44	4.207	10.083
11.239	11.227	13.829	16.054	9.692	13.535	9.502
4.508	13.235	9.869	14.192	8.637	13.346	10.582
1.835	8.752	5.35	3.349	1.759	1.013	2.003
3.93	7.051	8.63	12.852	6.414	9.838	10.171
2.091	10.764	10.735	8.45	11.049	9.054	7.404
2.928	6.923	3.915	5.227	3.027	1.052	3.646
3.355	11.906	8.283	11.583	3.95	7.962	8.276
4.829	6.639	10.289	9.359	3.798	4.538	4.117
4.286	5.927	5.249	4.869	2.157	4.506	3.607
3.939	4.82	4.063	4.181	2.322	1.033	2.255
5.334	4.949	6.798	9.176	5.279	4.804	6.631
3.578	5.476	9.603	15.022	5.82	8.909	6.115
4.69	3.059	4.611	8.492	4.853	3.609	3.806
4.75	5.708	5.334	12.498	4.325	7.803	11.592
5.753	9.984	15.221	15.146	9.309	11.146	11.593
3.535	6.346	3.15	4.932	1.563	3.312	1.973
7.145	8.368	8.647	11.592	8.292	9.478	6.594
3.603	4.808	7.47	4.353	2.833	1.26	2.058
8.787	7.575	18.778	5.835	10.68	6.858	9.089
2.204	6.586	4.1	3.601	3.173	3.926	0.819
2.552	7.331	4.173	3.559	1.04	2.048	4.22
6.457	6.284	9.29	12.802	10.291	7.19	6.212
20.008	14.314	16.24	17.752	10.142	11.786	10.972
4.053	7.156	5.014	12.63	2.965	5.296	6.231
7.951	8.217	11.859	5.535	4.583	3.12	2.498
9.876	11.21	13.84	14.841	11.666	12.757	15.316
5.325	4.872	6.213	3.508	4.271	3.609	2.172
2.228	2.186	2.444	4.871	0.593	3.653	5.823
19.18	13.998	21.071	15.959	11.545	11.263	13.564
25.428	23.644	34.556	31.192	25.838	29.667	31.897
7.024	16.355	14.205	16.992	13.424	16.391	15.362
4.94	3.725	10.6	7.528	9.356	7.655	9.029
12.55	14.235	12.758	19.381	11.216	12.224	12.069
27.416	32.76	42.789	48.789	39.628	45.507	49.837

4.187	9.962	10.852	9.979	8.541	6.715	8.511
10.064	9.125	15.184	11.076	6.383	6.635	8.058
13.077	11.286	13.586	21.151	10.033	13.614	13.979
5.323	5.5	5.52	11.317	2.447	4.138	4.736
2.862	5.186	5.904	4.458	2.122	3.127	2.787
7.193	5.803	11.523	7.586	9.156	11.074	6.055
9.416	19.166	18.529	34.605	14.132	34.348	20.48
3.639	5.989	6.5	6.677	3.46	3.759	3.123
1.722	2.324	1.649	10.308	0.422	1.545	7.777
5.799	3.01	5.713	6.006	8.166	8.281	5.1
8.225	8.989	13.72	12.115	9.687	9.371	10.336
2.833	7.441	5.683	6.571	3.366	8.692	5.001
12.991	13.343	14.541	19.443	8.274	12.286	12.631
2.652	8.075	2.993	15.713	2.472	5.946	10.449
10.879	5.195	8.656	11.524	4.367	5.555	4.801
7.595	1.724	3.127	5.676	2.188	5.392	1.235
7.14	4.894	7.487	6.558	3.665	4.663	5.253
1.981	0.922	0.317	0.064	0.003	0	0
3.052	12.343	12.393	15.293	10.925	10.654	12.823
4.577	3.382	3.792	6.258	0.795	2.943	6.355
0.323	0.372	0.008	0.015	0.012	0	0
8.286	11.653	16.475	30.504	11.312	10.611	24.976
1.614	7.18	4.899	6.944	2.335	3.509	7.059
23.761	24.255	30.846	30.571	18.327	25.361	28.019
6.396	4.575	2.263	2.407	0.689	0.54	0.241
10.197	8.36	10.787	12.878	5.7	9.693	6.533
4.34	3.387	5.908	4.077	4.04	4.589	2.756
3.169	5.981	3.953	9.651	1.082	3.302	8.278
9.017	16.577	17.553	17.966	8.191	14.171	19.599
9.831	11.002	12.911	12.174	9.129	6.893	12.203
6.232	11.888	10.717	13.767	5.604	4.697	13.441
12.486	6.183	10.255	7.154	4.167	5.069	3.991
4.307	7.282	9.318	13.223	7.695	13.005	9.489
1.64	9	5.975	3.353	1.834	1.386	2.463
7.692	10.17	10.154	14.947	6.207	6.071	4.056
4.952	4.037	6.689	6.104	2.175	3.098	5.355
5.297	11.022	9.904	19.52	6.934	13.603	17.008
10.235	9.258	9.499	11.521	8.444	7.716	6.541
5.292	3.579	4.349	7.833	3.065	4.792	4.337
8.59	8.624	11.237	17.868	9.602	15.949	21.713
3.823	1.777	3.088	1.229	2.055	1.422	0.806
3.295	1.621	1.831	0.613	1.3	0.696	0.4
9.199	8.718	12.513	17.008	10.55	15.036	11.384
3.588	5.438	4.619	7.842	1.55	2.497	9.412
5.115	4.848	3.647	10.811	3.743	5.565	5.246
3.372	4.106	3.081	1.578	1.727	5.56	1.837
6.782	5.109	6.367	7.637	3.228	6.478	1.499
9.511	5.616	6.946	7.155	6.573	5.334	4.023
3.021	3.59	4.828	1.152	2.08	0.955	0.52
5.555	6.064	7.789	15.285	7.847	12.631	8.568

3.343	6.721	6.581	6.831	5.28	5.746	5.943
4.467	4.534	6.107	7.351	2.744	4.848	6.243
4.214	5.546	7.555	11.888	5.372	9.516	7.837
5.649	6.457	9.661	8.393	4.432	7.534	6.337
7.588	9.334	10.007	13.414	8.534	15.311	12.725
4.472	8.393	5.162	8.15	2.531	3.01	7.914
3.023	5.375	10.412	8.004	5.355	3.657	5.466
6.545	6.69	7.638	11.295	6.086	5.726	6.97
4.282	6.884	9.397	8.937	5.373	7.029	6.086
3.96	6.275	8.702	6.715	4.509	5.695	6.479
1.779	2.281	1.143	0.463	0.286	0	0
1.39	1.482	2.219	0.417	1.016	0.367	0.33
11.341	13.656	15.129	23.343	10.675	12.746	9.507
7.206	8.652	9.335	9.425	7.044	8.956	8.075
7.42	8.081	9.625	14.492	11.936	7.01	10.445
15.219	14.973	16.864	20.801	9.386	11.599	12.308
2.204	8.661	5.047	8.366	0.96	2.922	8.073
1.911	5.728	7.176	8.846	4.298	2.886	4.034
2.604	1.924	2.567	1.078	1.051	0.648	0.479
7.748	6.105	10.654	8.775	6.318	9.505	5.574
13.198	14.196	17.946	26.72	15.156	23.369	24.344
7.105	11.515	11.381	10.53	6.007	6.017	2.88
9.565	7.232	11.534	18.184	9.272	9.112	10.227
5.136	6.556	8.473	11.515	6.879	9.584	8.899
4.057	6.527	4.355	12.428	1.83	4.18	6.512
7.966	7.769	10.779	14.622	8.657	10.269	12.595
2.64	3.499	4.278	9.222	4.659	4.658	3.857
11.929	13.313	13.116	21.179	10.966	9.446	9.32
11.272	12.021	11.082	18.561	8.362	6.642	6.254
4.676	4.838	3.605	7.582	1.437	4.269	1.642
4.102	2.888	5.457	3.737	5.227	3.012	2.477
7.458	8.665	14.465	22.185	8.678	8.711	15.198
6.278	6.592	11.252	15.603	9.271	12.821	7.248
5.1	4.545	5.639	8.951	2.648	4.118	5.285
8.269	7.388	9.081	15.826	6.602	19.217	7.07
4.445	0.938	1.734	1.91	1.123	1.031	3.056
1.899	1.77	3.964	2.828	0.295	1.028	1.351
4.313	4.863	7.102	13.258	6.953	7.774	8.924
5.599	7.106	12.069	10.656	8.489	6.768	6.819
13.612	15.717	14.216	26.352	10.095	17.108	16.605
5.574	6.067	7.843	14.462	7.797	12.244	8.636
8.803	5.88	8.966	7.125	4.578	6.76	2.684
6.335	4.725	6.972	6.233	8.012	7.333	5.243
9.071	6.955	11.672	12.482	12.08	9.87	9.95
3.854	7.005	4.987	10.841	2.915	5.342	4.837
8.041	12.17	11.624	16.698	6.271	8.107	5.784
6.908	5.162	12.19	15.613	7.329	9.275	10.354
23.664	29.255	35.236	40.548	24.335	28.961	38.372
11.733	4.788	4.636	9.383	3.467	1.991	3.083
1.612	3.773	1.932	3.335	0.904	2.83	1.706

8.46	12.148	8.877	14.409	6.46	8.51	3.964
11.272	5.035	3.411	9.385	2.418	3.138	2.283
7.107	4.923	11.913	16.03	6.98	8.874	8.42
4.801	4.583	6.366	9.668	5.683	3.645	1.932
2.633	3.935	5.641	7.818	2.43	3.051	3.545
6.992	15.309	16.064	18.965	14.447	13.732	17.341
7.701	7.839	9.419	10.297	5.137	8.583	9.365
8.288	9.45	9.823	11.449	14.458	10.576	12.554
6.504	6.015	6.114	4.966	4.32	2.284	2.36
2.422	5.168	4.077	8.293	2.375	3.673	3.973
9.578	13.735	13.619	23.386	11.23	13.488	19.654
8.221	11.465	7.427	12.919	4.989	12.598	4.789
15.427	11.619	12.612	14.405	7.421	11.62	8.158
13.404	13.329	15.626	15.218	7.33	8.769	13.148
2.27	5.353	3.584	6.786	0.976	1.693	5.334
2.548	5.341	3.989	6.537	3.046	3.97	2.632
2.165	6.743	6.221	16.82	1.178	5.124	10.418
2.395	4.929	4.215	10.194	2.786	4.594	4.814
6.856	5.481	5.269	8.729	0.807	7.078	1.105
2.397	4.933	4.215	10.187	2.791	4.603	4.813
5.64	7.314	7.072	7.182	3.82	3.24	4.469
7.071	13.235	14.943	12.591	9.155	9.106	9.617
12.489	8.327	9.278	14.954	6.398	11.209	9.294
13.278	8.09	10.263	13.143	8.007	9.385	7.258
4.53	5.858	6.064	5.133	2.975	2.794	3.364
8.694	10.869	20.032	23.67	17.165	20.206	17.024
2.04	3.911	2.244	2.901	0.934	1.56	1.186
2.93	5.075	6.553	5.076	2.562	2.121	0.497
7.206	7.659	10.916	13.882	8.126	7.38	9.491
5.3	4.875	9.934	6.416	3.44	6.895	5.594
25.52	25.277	32.499	32.966	27.983	25.932	27.151
4.225	9.57	8.343	9.747	5.6	7.805	7.87
1.032	0.514	0.914	1.205	0.031	0.489	0.029
9.236	9.086	7.976	7.785	6.758	6.336	5.105
8.782	13.577	12.149	21.834	11.654	16.385	10.469
2.819	4.732	7.325	6.759	4.702	5.326	3.549
11.929	13.181	14.961	16.825	8.622	11.957	12.292
4.294	5.998	4.2	4.338	2.602	2.923	4.124
5.612	5.384	9.447	4.625	3.486	6.005	5.889
9.145	7.248	13.112	15.772	6.674	12.424	8.518
5.249	6.565	4.778	4.03	4.185	5.411	4.462
6.87	6.768	10.448	11.892	8.4	5.492	10.169
5.858	8.562	7.176	10.029	2.204	5.391	8.495
7.314	3.35	7.873	4.483	4.249	5.994	6.923
4.413	4.024	4.585	7.722	1.286	3.537	5.099
5.932	15.549	18.96	21.922	13.106	20.17	15.48
4.589	4.364	5.713	6.128	3.346	7.829	5.994
4.111	7.325	6.127	13.041	3.335	5.898	6.514
5.386	8.966	7.457	8.62	5.177	4.927	2.265
5.039	6.363	4.068	7.464	3.465	6.518	4.148

8.472	11.754	9.38	12.734	5.515	13.861	6.791
3.033	3.602	5.433	5.907	4.185	3.741	2.77
13.142	12.632	12.316	10.345	6.588	6.164	7.877
11.246	10.916	13.526	23.313	9.54	15.329	13.551
4.446	2.412	3.166	1.814	2.517	1.518	0.632
9.665	5.594	5.288	8.01	3.591	2.779	3.277
2.591	6.872	7.463	7.124	3.279	4.963	4.958
3.417	5.662	4.411	8.636	3.159	4.332	6.266
2.229	7.582	4.026	11.867	2.488	5.823	6.071
7.14	8.345	5.547	4.171	7.016	4.759	6.216
2.982	5.817	3.559	6.414	3.149	2.087	5.276
4.919	8.733	7.91	13.557	4.544	8.486	6.84
6.48	9.694	7.128	10.351	4.83	6.942	7.323
8.143	7.834	10.996	9.488	6.213	8.921	4.601
4.877	6.163	9.035	10.215	12.221	4.817	6.277
4.927	11.647	8.437	12.906	4.092	8.119	13.948
1.296	2.559	1.805	5.87	0.28	0.787	2.183
10.438	13.055	13.524	16.139	10.893	12.56	15.264
26.794	25.924	33.13	35.11	27.367	31.632	31.815
10.147	15.584	12.433	26.931	9.967	13.956	15.053
40.592	43.778	52.597	48.6	40.815	51.607	53.037
9.267	9.088	19.073	13.519	14.124	14.925	12.527
5.957	5.863	8.576	14.645	6.282	5.649	7.629
9.294	4.689	9.653	6.647	3.008	7.825	7.381
5.836	3.286	3.878	3.174	3.457	5.306	2.07
4.891	9.139	11.553	7.558	7.057	7.119	8.255
7.21	5.839	9.84	8.384	7.463	10.186	7.974
6.304	6.512	7.151	7.437	3.76	5.562	1.375

RDF070v	RDF075v	RDF080v	RDF085v	RDF090v	RDF095v	RDF100v
1.674	1.589	3.752	0.843	1.049	0.454	0.423
3.83	2.943	3.659	9.775	4.365	3.196	3.53
6.978	7.342	4.655	2.391	3.57	1.425	2.123
0.582	0.002	0.097	0.081	0	0	0
2.703	1.103	0.12	1.059	1.199	0.709	0.244
0.269	0.344	0.033	0.18	0	0	0
11.926	7.971	10.17	6.74	6.946	8.178	5.283
0	0	0	0	0	0	0
5.44	2.1	4.047	0.901	0.384	0.083	0.011
1.8	3.027	2.025	4.868	2.406	3.205	2.786
27.78	22.084	19.614	16.101	10.568	8.644	8.297
12.727	12.874	11.008	6.843	10.514	8.225	8.402
4.653	4.849	3.087	2.874	2.561	1.842	0.791
11.248	3.638	6.92	6.311	6.803	2.209	3.928
6.552	4.828	2.708	2.249	1.225	0.477	0.061
4.834	5.91	6.42	3.266	4.01	4.467	3.55
0.862	0.896	0.476	0.378	0.074	0.077	0
14.551	8.2	9.174	12.397	10.067	4.92	4.83
2.673	1.748	2.479	6.151	1.77	3.234	1.81
5.682	4.266	4.798	6.623	3.162	3.664	2.308
5.686	5.566	3.373	2.461	3.184	1.71	1.902
7.393	0.238	3.028	0	0.695	0	0.078
1.955	1.537	4.946	1.118	1.897	1.357	2.145
4.546	4.003	4.354	4.776	4.69	3.461	1.816
13.276	17.757	11.755	10.389	10.921	6.159	6.358
6.918	8.644	3.982	4.644	2.756	4.006	1.811
4.552	3.533	3.229	7.025	3.696	5.426	4.23
17.201	25.502	24.681	25.124	19.524	15.905	14.513
8.795	7.687	6.805	3.984	5.218	4.218	3.265
5.015	4.638	4.795	7.901	4.648	4.434	3.305
20.72	19.041	14.904	16.858	14.231	10.804	9.121
29.806	23.966	17.427	19.041	17.358	12.067	15.036
5.854	3.038	4.252	3.653	5.703	3.973	2.44
10.034	5.698	7.045	4.26	4.476	1.272	0.823
15.457	14.912	14.809	11.818	8.198	6.052	4.496
6.193	6.072	7.189	9.245	5.769	5.97	6.802
4.577	6.592	4.758	4.556	3.032	1.805	1.312
0.587	0.18	0	0	0	0	0
16.104	14.803	13.13	8.235	12.704	8.551	5.964
7.447	5.816	5.37	4.739	3.403	4.027	3.652
12.465	6.235	11.652	10.52	7.824	6.201	8.793
2.632	0.175	0.894	0.002	0.062	0	0
18.82	15.813	13.179	16.093	19.355	11.161	8.485
2.544	4.708	1.762	1.872	1.539	1.597	1.476
11.642	10.416	7.8	11.477	7	7.486	7.127
3.662	3.487	4.379	6.578	5.205	4.213	2.466
3.927	5.953	7.922	6.377	3.642	4.495	2.22
8.688	6.329	7.777	8.69	5.437	4.919	3.483
5.886	1.915	7.577	5.71	6.389	3.084	3.308

8.478	6.733	7.178	4.993	6.373	5.478	5.768
5.942	4.738	5.983	6.978	5.49	5.647	2.45
13.704	10.685	14.189	12.778	14.823	14.544	8.313
5.113	6.292	7.42	5.465	4.752	4.758	3.476
8.86	7.675	6.755	3.176	2.27	1.272	0.725
4.613	4.223	5.435	4.276	6.827	3.81	2.281
9.975	7.119	5.613	8.428	6.512	7.122	4.897
12.104	8.06	10.17	9.015	10.12	9.69	4.367
6.524	4.317	4.358	2.9	4.817	1.759	1.407
8.62	7.09	8.75	7.851	5.491	7.17	3.682
5.068	5.118	6.946	4.922	7.253	6.749	2.3
6.068	6.842	7.29	3.184	2.776	2.623	1.144
4.159	5.432	8.696	3.972	2.544	2.166	3.229
10.005	6.442	5.571	5.048	7.011	3.877	4.591
5.792	5.189	7.399	5.426	5.66	3.36	2.985
5.045	5.455	5.14	5.299	2.361	2.985	2.075
10.712	7.12	6.701	3.955	3.285	5.154	4.492
6.875	10.543	5.501	7.827	4.38	5.469	4.795
0.983	1.025	0.072	0.442	0	0.06	0
5.442	2.264	4.095	2.69	1.984	0.3	0.076
7.039	8.962	2.585	3.293	3.514	1.631	0.937
4.482	4.09	1.55	1.936	1.527	1.988	0.779
8.792	7.497	4.063	4.472	3.087	1.061	0.97
4.275	2.782	3.946	2.611	0.793	1.208	0.01
2.614	1.065	0.93	1.642	1.077	0.183	0.868
1.646	2.629	1.166	3.359	2.07	2.223	2.618
6.282	4.353	4.029	7.029	3.759	4.972	2.077
5.87	4.079	3.804	4.875	7.419	4.742	5.871
2.119	1.297	0.361	0.566	0.152	0.473	0
5.908	3.127	2.953	5.878	2.695	0.324	1.569
10.15	7.288	7.896	7.584	2.116	9.188	6.414
0.884	0.522	0.382	0.158	0.001	0	0
7.611	8.362	6.001	3.733	4.072	2.448	2.358
0.551	0.631	0.115	0	0.011	0	0
7.964	6.284	7.924	4.152	1.97	2.361	1.03
1.44	0.542	0.114	0.255	0	0	0
1.527	2.806	2.36	1.996	0.621	0.419	0.749
4.621	4.526	3.598	2.498	1.905	0.92	0.31
6.882	8.438	4.197	3.151	2.86	3.869	2.617
4.209	1.926	1.789	0.763	0.126	0.079	0
2.703	0.272	1.299	0	0.26	0	0
13.162	9.766	7.17	5.948	5.605	2.431	0.254
1.034	1.768	1.247	0.36	1.012	0.971	0.079
2.062	1.23	0.245	0.913	0.101	0.067	0.192
8.5	8.516	5.208	5.6	5.805	2.566	3.046
23.531	21.35	21.068	16.567	27.604	20.383	20.016
17.419	12.949	11.6	9.812	5.554	2.764	1.342
6.871	4.516	4.258	1.89	0.714	0.915	0.148
9.601	11.342	3.604	6.549	0.719	2.888	0.456
45.281	42.634	41.761	43.31	36.429	34.289	30.794

7.068	10.23	7.704	13.273	8.138	12.142	8.54
9.954	9.969	11.103	4.355	5.935	5.346	5.268
9.251	7.928	4.563	8.463	4.916	5.124	3.385
2.337	5.375	1.869	3.641	1.889	2.715	0.439
1.524	1.743	2.351	4.352	2.409	2.103	0.695
4.331	6.757	1.951	1.513	0.23	0.19	0.003
25.948	17.836	17.228	17.564	9.222	10.79	7.356
2.894	5.05	1.668	3.346	2.887	3.326	1.268
3.891	3.978	2.21	2.952	3.937	1.854	2.053
3.497	3.198	1.79	0.541	0.06	0.009	0
6.099	5.221	5.815	6.367	5.428	3.944	5.631
3.889	5.931	4.512	4.128	3.077	0.546	2.189
11.361	7.763	6.641	7.168	6.879	5.836	5.338
4.301	5.249	3.338	8.4	5.697	1.822	2.612
3.234	2.428	0.984	1.965	0.207	0.115	0.006
4.831	0.541	3.889	0.341	0.95	0	0.29
4.263	3.31	2.396	2.128	1.419	1.924	1.885
0	0	0	0	0	0	0
9.803	13.22	9.566	8.314	8.303	6.404	5.344
3.873	1.677	1.577	3.241	1.224	0.682	0.822
0	0	0	0	0	0	0
12.969	19.15	13.621	7.094	12.177	8.2	11.39
2.579	2.58	2.099	2.604	2.819	1.305	0.036
26.891	15.829	16.258	20.646	11.642	10.892	6.767
0.085	0	0	0	0	0	0
6.486	2.155	2.175	1.424	0.311	0.279	0.008
1.623	2.571	1.161	0.838	2.056	0.668	0.781
4.47	6.261	2.511	4.539	2.716	2.106	1.325
12.082	9.418	13.422	12.982	8.121	7.086	6.795
10.752	7.416	11.431	9.487	13.25	6.434	8.659
11.741	8.824	8.282	8.954	11.64	10.346	8.706
7.188	6.202	5.762	6.452	5.734	4.738	4.405
10.826	7.252	3.903	8.424	4.473	4.636	1.81
1.172	2.107	0.351	0.571	0.089	0	0
1.823	0.484	0.293	0.475	0.062	0	0
1.572	1.918	1.696	1.05	0.426	0.843	0.363
10.137	4.916	6.623	18.084	9.859	7.782	7.644
10.238	7.132	3.534	3.639	2.695	2.378	0.524
6.103	2.492	3.201	3.431	1.523	1.849	2.634
16.135	10.629	14.381	10.187	9.734	9.372	7.19
1.167	0.255	0.446	0.296	0.067	0.11	0
0.354	0.017	0.201	0.001	0	0	0
5.982	3.836	4.118	3.437	2.329	0.677	0.606
4.043	2.09	2.308	4.393	1.791	4.028	1.251
4.259	4.625	2.781	1.811	0.393	0.444	0.061
1.72	0.641	1.078	0.914	0.133	0.061	0.037
4.113	2.663	4.673	4.207	3.68	3.753	3.692
5.376	4.198	2.356	2.038	0.679	0.909	0.208
0.134	0.074	0	0	0	0	0
8.972	4.088	4.108	4.388	4.155	2.047	1.842

5.289	3.733	3.176	1.94	2.418	0.194	0.661
6.907	1.104	2.618	6.126	4.522	1.61	2.875
8.879	2.674	2.252	4.142	2.847	2.111	1.563
5.803	1.962	2.25	0.102	0.178	0	0
13.438	7.645	10.696	7.359	7.766	5.863	6.878
2.51	2.158	3.249	5.584	2.464	2.519	3.625
5.436	2.266	2.725	2.323	1.255	1.656	1.539
4.616	2.442	2.564	0.825	0.335	0.224	0.042
6.064	1.853	2.68	0.787	0.383	0.093	0
6.8	2.339	2.162	1.759	1.372	0.45	0.309
0	0	0	0	0	0	0
0.029	0.054	0	0	0	0	0
8.149	2.852	1.595	1.255	0.797	0.416	0.14
8.049	13.789	8.924	4.932	4.164	3.885	1.686
6.325	8.131	6.032	11.759	6.743	6.861	3.875
6.596	7.796	5.424	4.744	2.844	3.584	1.579
3.964	2.315	2.449	5.505	5.541	2.092	1.675
2.972	2.254	1.266	6.936	2.608	3.881	1.938
0	0	0	0	0	0	0
5.551	2.408	4.446	2.041	0.945	0.47	0.041
18.119	20.818	15.093	22.002	11.722	16.205	11.162
2.058	0.635	0.165	0.199	0	0	0
11.076	5.726	8.197	6.728	8.001	3.811	2.64
8.804	4.466	3.263	2.775	2.285	0.735	1.636
2.19	6.773	2.385	6.651	4.693	2.181	2.691
12.751	10.567	14.294	9.638	8.142	3.896	4.459
1.229	0.615	0.793	0.691	0.472	0.275	0.184
8.16	2.802	4.269	2.859	2.186	1.037	0.528
5.977	2.108	1.867	0.656	0.254	0.04	0
3.436	0.28	1.32	1.657	0.764	2.73	0.219
3.138	3.359	2.392	2.06	1.634	0.419	0.079
7.106	11.472	4.322	7.101	8.903	2.238	7.985
4.979	2.12	2.084	2.035	0.827	0.115	0.016
4.15	3.184	2.422	2.254	0.718	0.763	0.18
9.08	2.927	3.669	2.683	2.385	1.038	0.356
1.594	1.128	1.758	0.749	1.319	0.176	0.81
0.527	0.263	0.246	0.173	0	0	0
4.201	1.582	1.855	5.098	2.516	0.201	1.474
4.822	7.425	3.257	1.822	1.802	1.116	0.224
15.498	9.879	9.03	12.608	9.253	9.663	9.994
8.671	4.325	3.984	4.356	4.188	1.954	1.909
4.543	3.352	4.464	3.074	5.703	3.025	3.594
4.228	3.084	2.575	1.855	1.837	0.654	0.558
8.772	15.597	5.465	3.75	6.237	2.426	0.66
4.165	1.557	1.635	0.135	0.1	0.009	0
5.512	1.666	1.835	0.864	0.407	0.245	0.034
8.172	4.096	4.473	3.11	3.68	1.605	1.9
15.337	27.286	22.222	16.246	22.309	13.926	18.755
0.343	1.955	0	0.553	0	0	0
2.076	0.017	0.686	1.366	0.343	0	0.405

3.926	1.071	0.817	0.08	0.036	0	0
0.869	0.786	0.136	0.061	0	0	0
7.009	4.3	5.009	2.959	4.45	1.546	1.179
2.285	0.892	0.469	0.126	0.001	0	0
3.069	2.806	1.792	4.786	3.519	3.066	2.143
14.397	18.865	12.471	13.383	13.478	9.797	6.826
8.481	7.774	6.121	6.157	3.787	4.251	3.231
7.202	4.26	2.544	1.992	1.086	0.424	0.486
2.116	1.092	1.864	3.374	1.004	0.296	0.055
2.86	1.833	1.454	5.581	2.777	1.498	2.416
9.135	21.382	6.679	9.484	8.362	4.839	7.994
5.086	3.583	6.504	9.689	2.727	6.177	3.536
6.768	3.75	3.267	3.73	3.197	2.222	1.349
3.919	5.59	6.466	3.412	4.555	2.242	1.335
1.927	1.253	1.932	6.184	3.019	2.091	1.26
4.44	4.794	3.557	2.459	2.076	2.559	0.554
9.76	8.296	4.015	7.149	5.145	1.187	5.386
1.252	3.319	2.154	5.654	1.559	3.002	2.133
3.362	0.827	1.372	0.188	0.03	0	0
1.253	3.309	2.157	5.654	1.564	3.011	2.131
1.427	0.224	0.391	0.409	0.241	0	0.496
9.194	8.449	8.176	4.113	4.108	5.964	5.661
13.574	4.433	7.441	4.167	2.05	1.431	0.265
8.256	6.869	6.825	6.364	3.977	1.975	2.985
2.021	4.821	2.476	3.379	4.185	2.297	2.251
18.832	13.114	10.338	11.112	12.391	9.343	9.82
0.576	0.7	0.297	1.249	0.331	0.002	0.001
0.171	0.041	0	0	0	0	0
6.184	6.805	7.258	5.438	5.613	3.572	5.715
7.755	5.934	5.996	3.621	2.004	1.242	0.354
15.422	15.337	21.19	18.069	13.275	16.02	12.965
11.293	3.163	5.121	4.173	2.476	0.879	0.429
0.066	0	0	0	0	0	0
5.029	4.993	3.698	6.989	2.354	2.754	0.933
15.399	11.885	9.666	6.875	9.638	11.376	4.785
4.563	3.245	2.655	2.958	3.174	2.111	0.831
13.872	7.52	7.552	10.133	6.542	5.067	4.32
2.837	1.726	2.315	0.171	0.06	0.232	0.003
3.069	6.628	2.061	3.037	0.93	0.544	0.923
9.709	8.126	3.169	6.835	6.852	3.609	5.042
4.986	2.726	2.332	1.299	0.825	0.514	0.094
6.738	4.954	4.016	4.118	3.686	2.262	2.206
6.851	4.369	4.245	7.848	5.188	4.224	3.159
5.888	4.762	4.416	2.622	2.255	1.6	0.672
3.472	2.925	1.73	1.767	0.823	0.529	1.176
10.945	8.343	7.261	5.093	2.291	1.278	0.652
5.759	3.125	3.798	4.544	4.62	0.942	4.595
4.69	2.075	1.961	0.761	0.126	0.079	0
2.868	1.427	0.123	0.351	0.34	0.153	0
3.387	3.001	2.884	2.815	1.791	1.704	0.294

6.564	5.498	5.286	10.522	2.972	7.038	3.722
3.915	2.038	1.465	0.976	2.167	0.925	0.299
6.051	5.004	2.757	3.41	0.985	1.11	0.19
11.022	8.605	4.991	8.743	3.704	5.345	1.891
0.295	0.17	0	0	0	0	0
1.079	1.085	0.197	0.007	0.997	0.003	0.019
4.062	2.024	2.841	1.77	0.02	0.021	0
4.854	7.065	2.059	5.365	6.036	2.466	1.39
3.576	3.731	1.986	7.74	4.546	1.598	2.514
5.06	4.267	6.389	5.458	1.889	2.894	1.597
3.497	4.208	2.034	2.187	3.289	1.371	2.413
5.66	2.813	2.18	2.219	1.84	0.817	0.501
6.176	5.078	6.906	3.297	7.509	2.769	2.875
5.86	2.443	3.505	1.668	0.629	0.362	0.035
4.126	3.093	2.884	1.925	1.519	0.592	0.171
7.265	6.249	3.871	10.338	6.786	4.823	5.172
0.142	1.803	0	0.5	0	0	0
14.716	11.486	8.614	10.062	10.222	10.539	6.82
18.037	22.664	23.036	19.665	18.079	18.785	14.462
9.048	8.8	8.312	8.813	8.401	8.418	6.898
42.233	34.906	35.249	30.034	31.358	30.197	31.71
10.061	8.035	5.617	3.403	4.17	1.343	1.925
9.049	7.031	2.907	2.451	1.505	0.834	0.07
7.092	5.133	4.474	2.259	2.745	2.17	1.486
2.388	1.568	2.672	1.854	1.46	0.22	0.195
5.553	4.483	5.99	6.804	6.016	2.874	5.746
8.166	6.803	7.653	2.83	1.824	2.139	1.222
3.057	2.555	4.022	1.246	1.487	0.562	0.465

RDF105v	RDF110v	RDF115v	RDF120v	RDF125v	RDF130v	RDF135v
0.017	0.225	0.011	0.014	0	0	0
5.311	4.727	2.197	2.586	2.9	2.967	1.544
0.166	0.295	0.091	0.002	0	0	0
0	0	0	0	0	0	0
0.332	0.009	0.019	0	0	0	0
0	0	0	0	0	0	0
5.752	6.71	2.15	3.045	1.466	0.438	0.447
0	0	0	0	0	0	0
0	0	0	0	0	0	0
2.101	2.013	0.811	0.478	0.175	0.024	0
5.124	5.4	2.73	1.773	1.495	1.635	1.033
8.547	10.657	7.288	5.542	4.179	4.668	2.424
0.631	0.074	0.084	0	0	0	0
2.409	1.738	0.785	0.282	0	0	0
0.016	0	0	0	0	0	0
4.316	2.044	0.551	0.242	0.004	0	0
0	0	0	0	0	0	0
3.668	2.622	2.47	1.355	0.447	0.435	0.155
2.236	2.293	0.44	0.757	2.039	1.778	0.781
1.948	1.32	0.374	0.409	0.148	0.052	0
0.684	0.364	0.089	0	0	0	0
0	0	0	0	0	0	0
5.105	1.692	0.766	0.681	0.457	0.095	0.041
1.7	4.194	2.447	0.775	0.661	0.031	0.13
2.659	3.969	0.603	1.129	0.032	0.103	0.019
2.231	1.28	0.552	0.06	0.056	0	0.061
2.841	3.689	2.47	2.475	2.601	4.442	1.598
13.152	14.681	4.311	6.423	6.85	4.186	2.81
0.695	1.416	0.857	0.168	0.048	0.002	0
5.216	4.588	4.444	1.706	3.306	2.768	2.273
10.072	8.412	3.742	5.61	3.523	4.003	0.758
11.239	13.238	10.925	13.027	4.813	4.797	5.282
2.442	0.87	0.393	0.108	0	0	0
0.141	0.068	0.002	0	0	0	0
3.765	1.485	0.527	0.093	0.015	0	0
6.046	7.32	3.232	3.873	7.118	4.935	2.868
0.996	0.036	0.042	0.018	0	0	0
0	0	0	0	0	0	0
7.486	5.195	4.525	4.899	2.202	4.652	1.633
3.334	2.865	0.692	1.172	0.191	0.152	0
5.359	3.831	4.597	2.057	0.795	0.463	0
0	0	0	0	0	0	0
10.132	12.828	6.56	4.29	7.5	6.381	6.03
0.438	0.134	0.571	0.137	0	0	0
6.551	4.232	3.357	3.774	3.615	2.626	2.754
3.091	1.559	0.011	0.073	0	0	0
4.052	3.298	0.316	0.628	0.08	0.132	0.008
4.289	4.304	2.605	0.934	2.089	1.945	2.356
4.924	3.498	2.306	2.723	1.792	2.125	1.548

6.54	5.198	4.917	4.405	4.442	4.009	2.703
5.606	5.534	5.734	4.181	3.619	2.081	1.49
11.96	8.021	7.332	4.967	8.807	7.224	8.215
5.424	3.626	4.144	3.466	2.601	2.027	1.611
0.658	0.993	0.036	0.529	0	0.012	0
7.729	4.516	1.882	3.341	2.619	2.032	1.542
6.67	4.42	3.397	2.088	4.094	6.535	5.595
11.695	10.435	8.379	5.818	3.776	7.418	4.394
2.835	1.399	0.567	0.206	0.023	0.016	0
6.695	4.218	4.11	6.076	3.89	4.365	2.177
6.033	3.014	3.088	2.569	1.75	3.127	1.629
0.617	0.239	0.03	0	0	0	0
0.803	1.573	0.02	0.342	0.015	0.007	0
2.574	4.431	3.614	3.757	2.519	2.858	2.049
1.949	2.094	2.13	2.31	2.011	1.14	1.095
3.34	0.911	1.638	0.196	0.171	0.005	0
3.25	1.528	0.923	0.467	0.082	0.038	0
4.346	2.889	2.174	0.91	1.028	0.576	0.348
0	0	0	0	0	0	0
0.452	0.242	0	0	0	0	0
0.132	0.032	0	0	0	0	0
0.34	0.221	0	0	0	0	0
0.011	0.004	0.002	0	0	0	0
0.163	0.005	0	0	0	0	0
0.317	0	0	0	0	0	0
1.67	1.529	0.712	0.882	0.287	0	0
4.071	2.804	5.121	1.308	1.906	2.386	1.147
5.102	4.221	5.756	3.491	3.221	0.83	1.609
0.12	0	0	0	0	0	0
1.054	0.948	0.311	0.406	0	0.046	0
5.903	2.427	2.457	2.38	2.163	1.34	0.765
0	0	0	0	0	0	0
0.479	0.527	0.054	0.082	0	0	0
0	0	0	0	0	0	0
0.543	0.169	0.02	0	0	0	0
0	0	0	0	0	0	0
0.15	0	0	0	0	0	0
0.13	0	0	0	0	0	0
0.788	0.533	0.425	0.041	0.002	0	0
0	0	0	0	0	0	0
0	0	0	0	0	0	0
0.239	0.006	0.002	0	0	0	0
0.011	0	0	0	0	0	0
0	0	0	0	0	0	0
1.188	1.275	0.54	0.944	0.117	0.082	0
22.553	13.342	22.132	15.216	17.493	14.561	9.956
1.063	1.244	0.182	0.4	0.001	0.053	0.001
0.047	0	0	0	0	0	0
1.056	0.106	0.185	0	0	0	0
29.815	19.237	16.624	11.629	11.653	5.819	4.855

6.409	4.609	5.789	5.359	2.604	1.526	1.952
3.519	6.168	5.496	6.166	4.018	4.702	2.817
2.656	2.556	0.544	0.661	0.043	0.043	0.021
0.973	0.712	0.248	0.097	0.04	0	0
0.355	0.004	0	0	0	0	0
0	0	0	0	0	0	0
9.594	6.572	2.629	5.781	3.094	3.886	0.79
1.229	2.011	2.672	0.174	1.841	0.156	0.477
1.328	1.367	0.592	0.485	0.159	0.395	0.173
0	0	0	0	0	0	0
4.774	4.257	4.971	4.312	2.752	1.686	1.349
2.549	0.467	1.281	0.002	0.46	0	0
4.32	4.194	1.721	1.856	1.063	0.731	0.223
7.469	4.312	0.468	0.99	0.535	0.001	0.182
0.006	0	0	0	0	0	0
0	0	0	0	0	0	0
2.196	0.206	0.555	0.229	0.609	0	0.065
0	0	0	0	0	0	0
2.972	4.389	4.838	2.226	1.454	1.517	0.682
0.681	0.238	0.208	0.088	0	0	0
0	0	0	0	0	0	0
11.367	5.63	10.167	6.476	7.528	10.449	6.051
0.696	0.515	0.703	0.06	0.017	0	0
8.594	8.386	6.219	2.856	4.858	6.674	3.594
0	0	0	0	0	0	0
0	0	0	0	0	0	0
0.2	0	0.088	0	0	0	0
0.639	0.679	0.043	0.07	0.005	0.005	0
8.572	4.861	3.613	2.585	2.402	0.775	0.322
5.668	11.385	8.696	8.173	5.869	8.15	6.238
9.099	5.399	10.985	6.418	6.68	8.8	5.82
2.69	1.718	0.921	0.226	0.1	0	0
3.021	2.388	1.105	0.754	0.237	0.08	0
0	0	0	0	0	0	0
0	0	0	0	0	0	0
0.171	0.09	0.015	0	0	0	0
13.312	12.46	4.809	9.039	8.571	9.682	7.953
1.15	0	0.294	0	0	0	0
0.934	3.17	0.383	1.044	0.164	0.114	0.095
4.604	5.006	4.852	2.612	1.509	1.11	0.305
0.002	0	0	0	0	0	0
0	0	0	0	0	0	0
0.107	0.056	0	0	0	0	0
1.719	2.074	2.689	0.788	0.821	0.126	0.025
0.06	0	0	0	0	0	0
0	0	0	0	0	0	0
3.554	2.023	2.603	2.045	0.57	2.449	2.023
0.072	0.022	0	0	0	0	0
0	0	0	0	0	0	0
0.471	0.877	0.769	0.438	0.242	0.493	0.233

0.019	0.088	0	0	0	0	0
4.691	3.582	1.444	0.946	1.82	2.01	1.039
0.276	1.849	1.348	0.451	0.459	0.01	0.089
0	0	0	0	0	0	0
2.798	4.681	2.518	0.882	1.188	0.392	0.321
3.501	0.667	1.514	1.195	0.417	0.129	0.024
1.095	1.205	0.55	0	0.025	0	0
0	0	0	0	0	0	0
0	0	0	0	0	0	0
0.014	0	0.006	0	0	0	0
0	0	0	0	0	0	0
0	0	0	0	0	0	0
0	0	0	0	0	0	0
0.736	0.651	0.007	0.034	0	0	0
8.26	4.013	4.152	2.59	4.409	2.267	3.991
0.894	0.351	0.653	0.002	0.041	0.046	0
2.483	3.579	0.758	1.209	0.472	0.137	0.006
2.15	2.672	1.594	0.965	2.311	2.394	0.974
0	0	0	0	0	0	0
0	0	0	0	0	0	0
12.536	9.991	8.962	12.809	14.138	12.999	10.262
0	0	0	0	0	0	0
2.852	0.987	1.691	0.211	0.218	0.012	0
1.675	0.912	0.106	0.362	0.452	0.152	0
4.205	5.853	3.296	2.179	1.271	0.886	0.191
1.943	0.851	0.274	0.103	0	0	0
0	0	0	0	0	0	0
0.414	0.314	0.003	0	0	0	0
0	0	0	0	0	0	0
1.029	0.052	0.182	0.001	0.001	0	0
0.154	0	0	0	0	0	0
2.553	7.736	4.484	2.625	3.999	5.174	4.344
0	0	0	0	0	0	0
0.086	0	0	0	0	0	0
0.476	0.103	0	0	0	0	0
0.001	0.005	0	0	0	0	0
0	0	0	0	0	0	0
1.062	1.07	0.283	0.347	0	0	0
0.433	0.001	0.07	0	0	0	0
11.463	11.954	8.024	9.853	7.963	6.331	5.387
0.494	0.881	0.771	0.469	0.266	0.497	0.238
4.022	2.327	4.545	2.499	1.843	1.709	1.732
0.057	0.064	0	0	0	0	0
0.236	0.088	0	0	0	0	0
0	0	0	0	0	0	0
0.043	0	0	0	0	0	0
1.368	2.224	0.466	0.01	0.158	0.162	0.002
12.252	12.98	13.822	12.721	8.833	11.935	7.666
0	0	0	0	0	0	0
0.486	0.078	0	0	0	0	0

0	0	0	0	0	0	0
0	0	0	0	0	0	0
0.88	1.237	0.561	0.107	0.072	0.007	0
0	0	0	0	0	0	0
2.25	2.221	1.132	1.218	0.216	0.247	0
7.412	5.977	5.678	2.644	1.01	1.609	0.646
1.878	0.903	0.242	0.076	0.044	0	0
0.108	0.005	0.012	0	0	0	0
0.178	0	0.012	0	0	0	0
2.731	3.335	1.62	0.869	1.254	2.005	0.84
2.836	2.554	1.16	1.188	1.392	0.736	0.629
2.692	2.218	2.583	4.826	2.127	1.999	1.893
0.755	0.676	0.106	0.006	0	0	0
1.395	1.01	1.106	0.307	0.01	0.076	0
1.536	1.977	0.387	0.782	0.491	0.18	0.017
0.445	0.071	0	0	0	0	0
6.406	6.345	0.654	3.119	2.742	2.855	1.392
1.624	4.258	1.11	2.241	0.56	1.009	0.017
0	0	0	0	0	0	0
1.624	4.248	1.111	2.24	0.56	1.007	0.017
0.333	0	0	0	0	0	0
4.499	4.818	3.092	2.668	2.553	0.228	0.442
0.08	0	0	0	0	0	0
0.582	0.25	0.034	0	0	0	0
1.479	5.403	3.388	3.39	3.049	1.862	0.978
8.586	5.117	6.514	6.197	4.922	2.747	1.605
0.001	0	0	0	0	0	0
0	0	0	0	0	0	0
6.052	7.114	5.34	4.193	3.488	2.363	3.796
0.033	0.001	0	0	0	0	0
15.206	8.66	8.666	12.927	4.156	2.925	2.274
0.031	0.001	0.001	0	0	0	0
0	0	0	0	0	0	0
0.607	0.162	0.386	0.108	0.003	0	0
6.368	6.773	7.812	3.12	3.979	4.464	4.068
1.617	0.014	0.065	0	0	0	0
4.017	2.578	1.692	0.859	1.317	0.758	0.262
0.015	0	0	0	0	0	0
0.754	0.142	0.001	0	0	0	0
5.26	7.026	4.256	2.267	1.875	2.714	1.057
0	0	0	0	0	0	0
1.803	1.001	1.409	0.453	0.648	0.23	0
3.935	1.631	0.942	0.667	0.163	0	0
0.773	1.546	0.847	0.055	0	0	0
0.966	0.336	0.005	0.01	0	0	0
0	0.013	0	0	0	0	0
5.582	2.204	3.76	3.115	2.668	2.451	1.972
0	0	0	0	0	0	0
0	0	0	0	0	0	0
0.217	0.091	0.033	0	0	0	0

5.745	3.626	1.88	4.176	2.015	3.209	1.28
0.178	0	0	0	0	0	0
0.184	0.004	0	0	0	0	0
2.454	2.163	0.494	0.639	0.039	0.049	0.015
0	0	0	0	0	0	0
0	0	0	0	0	0	0
0	0	0	0	0	0	0
2.572	2.134	0.856	0.389	0.437	0.002	0.098
4.742	5.939	1.461	3.178	7.013	5.552	2.627
3.099	1.815	1.147	0.209	0.196	0.102	0
1.68	0.922	0.182	0.365	0.014	0.001	0
0.087	0.001	0	0	0	0	0
4.422	5.28	5.072	4.321	4.051	1.227	3.245
0	0	0	0	0	0	0
0.141	0	0	0	0	0	0
6.236	4.737	3.809	5.256	3.072	4.502	3.509
0	0	0	0	0	0	0
4.927	5.788	4.688	1.78	1.558	0.686	0.12
16.123	12.199	10.714	12.452	9.787	3.719	4.003
7.043	13.7	10.329	8.961	5.553	7.474	8.83
27.055	24.65	21.762	17.28	13.916	9.268	4.728
0.141	0.131	0	0	0	0	0
0.077	0	0	0	0	0	0
2.039	1.117	0.635	0.487	0.508	0.188	0.079
0.041	0	0	0	0	0	0
5.199	6.447	5.314	5.512	1.553	2.632	2.09
0.562	0.136	0.472	0.659	0.06	0.003	0
0.15	0.599	0.01	0.077	0.251	0	0

RDF140v	RDF145v	RDF150v	RDF155v	RDF010e	RDF015e	RDF020e
0	0	0	0	8.138	9.255	4.567
0.846	2.23	1.551	0.642	13.939	14.663	13.09
0	0	0	0	7.662	11.157	5.247
0	0	0	0	5.377	3.354	4.959
0	0	0	0	7.137	6.8	10.854
0	0	0	0	10.287	8.449	4.497
0.63	0.78	0.201	0.013	13.826	19.493	15.991
0	0	0	0	3	2.763	3.213
0	0	0	0	6.069	9.083	4.749
0	0	0	0	11.547	11.127	11.436
0.107	0.028	0	0	29.544	35.814	35.178
1.608	0.21	0.074	0	12.628	17.28	10.081
0	0	0	0	10.335	13.263	9.908
0	0	0	0	12.718	14.487	14.953
0	0	0	0	11.63	13.882	10.35
0	0	0	0	11.006	13.669	8.761
0	0	0	0	5.505	5.412	4.071
0.024	0	0	0	10.513	21.371	11.918
0.343	0.33	0.26	0.074	12.068	11.25	11.431
0	0	0	0	10.744	15.708	9.466
0	0	0	0	8.641	11.433	5.203
0	0	0	0	3.746	5.368	4.442
0.232	0.235	0	0	6.291	7.856	3.695
0	0	0	0	9.257	12.476	6.886
0	0	0	0	15.021	18.331	14.384
0	0	0	0	14.519	24.683	13.681
3.054	1.987	0.985	3.293	13.827	16.272	13.215
2.311	3.6	3.417	2.615	19.482	22.535	14.895
0	0	0	0	10.413	12.281	8.571
2.141	3.79	2.153	1.685	14.665	16.05	16.04
0.683	0.065	0.054	0	14.286	15.524	14.61
2.872	3.975	2.805	2.253	19.395	33.354	16.626
0	0	0	0	11.314	12.405	7.031
0	0	0	0	12.558	14.636	9.046
0	0	0	0	18.737	31.751	17.644
4.774	4.007	2.535	1.382	13.251	20.11	9.797
0	0	0	0	13.787	21.138	9.49
0	0	0	0	4.291	4.98	4.49
5.665	2.527	3.145	2.555	20.851	26.498	14.659
0	0	0	0	7.9	14.397	6.809
0.003	0	0	0	10.529	13.058	11.908
0	0	0	0	6.978	8.136	7.851
2.446	2.368	6.121	4.483	21.719	35.621	15.643
0	0	0	0	6.793	8.064	4.022
0.483	1.083	0.406	0.087	13.693	15.94	17.384
0	0	0	0	8.906	11.633	8.202
0	0	0	0	10.788	12.737	11.702
4.454	3.291	2.983	1.351	9.956	13.956	11.014
1.053	2.761	0.886	1.386	7.269	11.136	8.976

2.365	1.554	1.174	1.617	11.917	18.732	14.103
1.899	0.38	0.778	0.024	9.795	12.973	10.437
7.524	6.557	6.503	5.966	14.11	21.684	13.769
2.11	1.484	1.504	0.579	9.699	14.088	13.131
0	0	0	0	9.851	13.269	13.636
0.238	0.023	0.124	0.001	11.78	13.653	10.457
3.01	2.454	1.67	1.133	11.578	18.634	11.534
1.935	2.718	0.424	0.95	12.187	17.234	11.414
0	0	0	0	8.083	11.007	9.28
2.007	6.21	4.014	2.929	10.705	14.903	12.168
1.297	0.73	1.038	0.243	9.431	13.69	12.726
0	0	0	0	7.723	11.099	2.797
0	0	0	0	10.163	12.482	11.2
1.412	1.524	1.583	0.138	8.37	13.384	8.914
0.906	0.812	0.116	0.274	8.277	12.425	10.28
0	0	0	0	10.846	15.02	11.221
0	0	0	0	13.238	18.253	12.681
0	0.375	0.001	0	11.893	16.796	12.992
0	0	0	0	4.486	5.42	3.94
0	0	0	0	8.582	9.632	7.197
0	0	0	0	8.504	10.081	7.608
0	0	0	0	6.929	6.247	4.865
0	0	0	0	8.163	10.518	8.192
0	0	0	0	7.605	9.721	3.495
0	0	0	0	6.118	8.697	5.528
0	0	0	0	8.702	6.192	7.892
1.174	0.653	1.756	0.897	8.358	12.06	10.699
1.039	1.142	0.205	0.408	10.736	14.282	8.314
0	0	0	0	5.072	9.359	5.041
0	0	0	0	9.165	15.725	7.636
0.516	0.045	0.072	0.003	16.526	21.065	13.14
0	0	0	0	5.639	5.509	4.087
0	0	0	0	7.979	10.926	8.754
0	0	0	0	5.163	5.465	4.046
0	0	0	0	9.083	12.709	9.626
0	0	0	0	6.949	9.277	7.627
0	0	0	0	7.967	5.561	4.808
0	0	0	0	10.503	11.805	8.901
0	0	0	0	14.519	24.893	12.181
0	0	0	0	6.082	8.735	5.378
0	0	0	0	6.451	6.109	5.417
0	0	0	0	8.703	14.832	7.174
0	0	0	0	6.475	9.184	8.309
0	0	0	0	5.235	6.059	3.307
0	0	0	0	15.74	25.588	12.73
16.637	11.05	9.114	17.853	30.415	51.146	31.903
0	0	0	0	11.546	14.844	11.793
0	0	0	0	9.575	10.653	9.125
0	0	0	0	14.022	15.267	13.024
1.355	0.665	0.046	0.022	25.951	42.993	27.719

0.616	0.605	0.108	0	13.092	14.282	12.646
4.314	1.284	1.119	1.167	11.782	15.91	9.342
0.002	0	0	0	14.997	20.477	14.837
0	0	0	0	8.143	9.987	6.731
0	0	0	0	12.789	12.22	13.371
0	0	0	0	10.33	13.449	7.794
1.653	2.396	0.826	0.581	15.841	30.123	24.466
0.042	0	0	0	10.999	5.799	6.693
0.043	0.001	0	0	7.298	8.322	4.44
0	0	0	0	7.284	6.507	4.095
0.661	0.74	0.106	0.141	12.522	15.252	8.829
0	0	0	0	6.446	7.518	5.172
0.523	0.057	0	0	16.424	23.527	10.612
0	0	0	0	9.869	17.457	12.122
0	0	0	0	6.274	9.065	6.921
0	0	0	0	4.421	8.409	2.536
0	0	0	0	6.202	10.035	6.199
0	0	0	0	2.518	1.318	4.139
0.317	0.14	0.024	0	12.139	19.329	12.638
0	0	0	0	6.787	7.418	3.815
0	0	0	0	3.381	2.004	1
9.676	6.064	4.19	9.613	21.329	31.078	12.304
0	0	0	0	7.45	7.767	4.092
3.516	2.653	3.92	0.966	21.538	32.587	18.026
0	0	0	0	9.172	9.535	9.138
0	0	0	0	9.811	16.001	15.513
0	0	0	0	8.677	8.516	12.724
0	0	0	0	10.17	10.212	6.878
0.172	0.069	0	0	24.428	29.95	26.534
8.278	4.961	4.526	7.638	16.331	19.959	11.839
3.435	2.157	2.595	1.697	14.038	16.592	12.313
0	0	0	0	10.781	16.094	11.147
0	0	0	0	8.355	12.842	7.413
0	0	0	0	5.825	7.338	3.344
0	0	0	0	8.835	17.734	7.518
0	0	0	0	8.572	8.245	4.063
7.379	7.076	5.018	4.09	15.169	18.754	16.668
0	0	0	0	7.98	10.172	7.694
0.001	0	0	0	7.638	9.736	5.602
0.004	0.002	0	0	12.948	18.394	7.505
0	0	0	0	3.405	10.699	5.838
0	0	0	0	4.499	10.111	5.914
0	0	0	0	11.717	14.9	10.841
0.001	0	0	0	6.952	8.421	4.776
0	0	0	0	7.296	8.265	4.292
0	0	0	0	6.636	6.53	7.055
0.49	0.04	0.229	0.003	6.911	9.757	6.077
0	0	0	0	5.75	8.368	3.99
0	0	0	0	7.053	7.76	4.327
0.126	0	0	0	9.26	17.049	9.85

0	0	0	0	10.044	10.092	8.575
0.707	0.473	1.299	0.431	9.219	14.529	10.562
0	0	0	0	9.506	14.977	8.779
0	0	0	0	6.029	8.521	6.036
0.04	0	0	0	11.54	13.638	12.563
0.019	0	0	0	7.127	8.294	5.778
0	0	0	0	9.901	9.992	6.99
0	0	0	0	9.204	12.355	9.56
0	0	0	0	8.504	13.246	8.935
0	0	0	0	10.044	11.9	7.694
0	0	0	0	2.411	3.664	3.063
0	0	0	0	7.506	2.986	6.892
0	0	0	0	12.106	13.843	8.692
0	0	0	0	9.938	13.64	12.552
3.124	3.027	1.606	0.8	13.955	14.067	12.086
0	0	0	0	14.938	23.785	12.077
0	0	0	0	11.124	11.192	11.437
1.554	1.379	0.818	0.543	12.152	12.617	13.69
0	0	0	0	4.577	5.4	5.654
0	0	0	0	6.068	9.466	4.266
9.564	7.211	3.766	2.447	17.669	21.816	10.239
0	0	0	0	9.675	17.628	11.031
0	0	0	0	11.601	21.761	10.768
0	0	0	0	10.647	11.781	8.458
0.05	0	0	0	14.316	16.409	13.396
0	0	0	0	11.008	15.756	10.093
0	0	0	0	5.986	7.805	3.866
0	0	0	0	12.189	21.768	10.984
0	0	0	0	9.984	19.051	9.596
0	0	0	0	7.051	7.415	6.957
0	0	0	0	8.284	7.606	8.823
2.52	1.798	1.713	0.236	11.45	19.894	12.607
0	0	0	0	8.482	11.027	10.278
0	0	0	0	6.255	6.73	4.245
0	0	0	0	9.32	11.946	9.917
0	0	0	0	3.355	5.642	2.832
0	0	0	0	8.277	5.809	7.451
0	0	0	0	9.165	13.878	8.717
0	0	0	0	10.162	12.213	9.927
9.296	10.01	5.872	7.351	19.43	24.011	20.309
0.132	0	0	0	9.26	17.008	9.921
1.278	0.385	0.929	0.348	8.851	9.313	9.633
0	0	0	0	7.68	11.097	6.228
0	0	0	0	9.696	14.797	7.672
0	0	0	0	6.526	8.521	6.288
0	0	0	0	9.673	19.291	10.217
0	0	0	0	9.809	10.488	4.908
7.095	7.642	3.199	2.528	25.173	41.033	20.056
0	0	0	0	8.743	8.759	7.192
0	0	0	0	6.351	5.071	6.959

0	0	0	0	10.862	15.294	8.14
0	0	0	0	6.618	6.672	7.566
0	0	0	0	9.184	10.241	4.373
0	0	0	0	6.991	7.33	7.079
0	0	0	0	10.714	10.823	9.828
1.013	0.107	0.029	0.008	15.071	20.012	14.232
0	0	0	0	9.948	14.355	10.335
0	0	0	0	9.959	12.283	4.428
0	0	0	0	6.973	9.437	4.284
0.304	0.49	0.736	0.006	11.488	10.717	11.38
0.008	0.139	0.194	0.001	18.159	24.214	16.154
1.642	0.468	1.005	0.007	10.358	13.377	13.299
0	0	0	0	12.577	23.191	9.021
0	0	0	0	14.204	21.895	8.376
0	0	0	0	10.86	10.128	9.21
0	0	0	0	9.862	10.131	8.294
1.347	1.99	2.21	1.384	13.392	14.615	11.824
0.093	0	0	0	10.709	10.944	10.453
0	0	0	0	8.476	7.384	3.491
0.092	0	0	0	10.709	10.947	10.452
0	0	0	0	8.075	6.158	4.868
0.213	0.086	0	0	11.798	16.073	15.113
0	0	0	0	11.379	16.292	14.439
0	0	0	0	11.379	16.308	15.33
0.885	0.533	0.609	1.385	10.636	10.876	9.433
0.72	0.079	0.089	0	16.935	20.22	13.659
0	0	0	0	3.487	4.873	2.942
0	0	0	0	8.43	7.635	4.518
2.972	2.92	1.654	1.905	11.556	20.064	8.662
0	0	0	0	9.006	11.02	8.307
1.102	0.347	0.09	0.053	23.158	38.046	24.389
0	0	0	0	11.72	13.73	8.336
0	0	0	0	4.242	2.976	2.856
0	0	0	0	9.906	17.448	9.093
1.678	1.704	0.074	0.325	13.393	19.627	12.761
0	0	0	0	10.636	10.269	8.728
0.076	0	0	0	13.205	17.39	13.667
0	0	0	0	6.755	5.788	4.806
0	0	0	0	8.458	7.232	4.917
0.419	0.392	0.229	0	9.572	10.669	5.001
0	0	0	0	7.515	6.685	5.324
0.052	0	0	0	8.463	11.517	5.266
0	0	0	0	11.62	13.924	9.019
0	0	0	0	10.268	11.904	7.917
0	0	0	0	6.303	6.985	3.255
0	0	0	0	14.098	14.56	14.997
1.632	1.71	0.405	1.114	13.045	11.873	11.757
0	0	0	0	6.784	9.54	6.436
0	0	0	0	8.584	8.279	7.695
0	0	0	0	6.366	7.163	5.457

1.958	0.821	0.542	0.113	11.803	16.904	15.195
0	0	0	0	10.964	9.54	7.978
0	0	0	0	11.915	20.541	10.089
0.002	0	0	0	14.997	21.169	14.123
0	0	0	0	4.033	3.939	4.024
0	0	0	0	8.73	8.782	7.887
0	0	0	0	6.331	8.959	4.966
0.001	0	0	0	11.422	14.842	12.751
2.46	2.846	1.729	1.374	13.215	12.987	14.852
0	0	0	0	10.28	10.605	10.098
0	0	0	0	9.023	8.129	5.72
0	0	0	0	11.487	13.902	10.512
1.208	2.072	0.415	0.464	9.849	14.461	10.92
0	0	0	0	5.572	9.107	4.499
0	0	0	0	10.092	8.111	14.241
2.726	1.171	2.802	1.659	13.141	16.42	15.086
0	0	0	0	7.423	6.592	4.998
0.134	0	0	0	14.085	17.209	12.638
3.044	1.126	0.542	0.075	23.916	40.016	21.821
3.416	4.477	4.209	3.036	16.569	20.038	23.64
6.68	2.357	2.68	1.016	24.208	43.774	33.585
0	0	0	0	11.294	19.393	9.065
0	0	0	0	8.133	10.041	6.124
0	0	0	0	7.025	9.291	4.051
0	0	0	0	7.829	10.046	8.834
3.035	3.927	0.516	1.964	9.858	16.169	8.715
0	0	0	0	9.356	10.744	6.636
0	0	0	0	6.985	8.758	4.523

RDF025e	RDF030e	RDF035e	RDF040e	RDF045e	RDF050e	RDF055e
23.828	12.113	7.824	12.153	18.704	8.344	12.754
49.244	15.492	10.609	22.509	25.6	33.263	8.752
33.993	12.084	16.15	15.86	23.301	31.377	15.075
11.257	4.558	3.934	5.091	7.312	4.697	1.655
17.167	5.797	9.222	12.706	15.332	9.093	6.655
21.006	19.276	9.17	14.256	12.917	13.099	7.981
59.606	29.967	32.61	42.93	46.238	49.104	36.76
4.864	2.684	4.05	2.686	7.513	1.005	4.017
27.856	13.209	14.066	18.987	11.806	16.822	8.801
34.981	10.955	9.639	17.69	21.408	24.111	9.047
109.059	36.721	41.939	70.085	81.512	88.052	60.623
53.639	27.023	19.36	21.481	39.089	24.865	29.566
36.526	13.036	17.926	19.577	26.73	27.478	24.067
39.338	15.731	15.03	36.112	34.557	34.607	27.518
38.19	23.348	23.63	28.066	30.105	29.226	23.834
36.262	16.55	19.04	23.122	22.397	22.007	22.01
14.605	8.337	8.052	9.561	8.431	10.98	6.454
46.887	16.508	19.576	36.614	41.383	53.47	39.707
33.337	9.586	9.939	15.772	24.736	22.563	7.435
40.717	15.675	23.19	29.163	26.301	20.888	16.674
31.772	24.178	14.46	14.238	22.139	28.752	22.175
9.165	6.247	9.677	13.019	11.851	14.708	10.164
23.745	7.648	13.853	16.16	13.457	17.782	7.077
31.191	19.604	17.903	15.868	25.083	21.778	21.503
57.803	23.899	25.001	37.113	45.189	38.485	38.315
75.395	34.383	52.545	53.941	58.584	48.089	36.995
41.871	15.283	10.904	23.065	26.89	25.952	12.077
41.55	29.229	31.565	48.169	49.362	48.781	62.794
31.713	17.757	15.56	21.305	20.509	19.417	15.946
45.93	13.664	11.495	22.105	34.966	26.005	14.194
49.202	11.901	30.343	33.373	38.849	57.917	34.497
83.396	61.131	50.985	47.305	71.89	95.304	66.105
38.467	15.141	14.557	15.54	18.281	18.09	17.689
51.595	24.645	15.265	29.825	29.747	27.255	22.934
86.798	45.521	60.283	71.358	77.117	71.242	65.417
61.211	26.712	23.61	36.454	36.702	33.839	19.071
69.899	26.017	29.225	37.785	31.185	56.692	26.518
12.242	10.171	11.539	9.59	9.915	6.039	11.171
87.801	39.985	48.924	45.361	54.25	63.053	33.423
34.369	19.822	16.784	12.655	15.188	20.891	14.259
39.16	19.761	24.421	29.172	32.744	42.852	33.257
17.705	9.917	11.974	14.287	22.118	10.616	15.593
118.274	71.599	85.408	82.024	85.698	108.949	63.304
18.276	7.951	11.246	11.23	15.147	13.423	8.274
38.783	12.57	11.999	23.985	32.349	29.913	20.86
29.144	10.091	13.887	13.98	17.018	21.253	19.449
30.454	13.825	15.898	16.688	22.673	27.723	21.217
34.288	15.828	16.725	24.844	22.885	29.641	20.229
26.289	16.488	14.281	16.139	19.22	24.652	10.894

43.211	26.526	27.602	24.845	34.523	38.984	22.1
33.789	14.226	14.513	16.448	20.01	28.393	16.636
54.121	32.147	27.805	38.37	38.579	52.164	32.928
33.521	14.478	15.322	17.178	21.427	30.408	17.592
36.036	16.458	17.459	27.535	32.667	32.493	26.092
34.717	15.871	15.16	18.883	26.063	30.386	16.939
44.637	26.038	24.904	27.088	40.23	41.423	18.177
47.89	18.628	23.495	25.184	26.229	40.725	28.746
27.065	11.513	11.17	13.579	20.589	23.895	15.441
37.024	15.347	23.394	23.223	23.567	35.208	23.109
28.276	13.337	11.642	18.681	22.536	28.169	16.098
31.194	14.643	16.736	13.85	20.659	18.547	15.013
31.563	13.663	14.132	17.228	20.582	25.585	11.882
34.682	15.581	19.144	12.709	26.343	25.205	16.047
29.567	16.666	14.324	16.687	20.934	24.453	9.327
34.893	22.011	19.529	18.377	24.765	27.671	19.821
69.907	30.358	35.3	37.464	33.248	41.822	28.162
46.99	12.903	14.78	42.253	25.982	36.036	24.764
19.677	7.165	5.055	15.348	11.435	9.129	4.245
29.122	12.128	10.893	19.794	20.063	26.56	17.635
34.398	13.715	8.764	20.39	18.843	20.309	20.39
28.242	8.874	6.69	14.725	11.087	15.423	7.968
29.318	10.509	10.549	22.41	15.855	21.272	8.433
23.95	8.72	10.741	14.373	18.582	20.982	10.217
14.877	9.336	10.441	11.749	12.851	10.636	7.565
16.374	9.109	8.275	14.452	12.511	12.519	7.695
29.768	11.207	13.766	15.35	23.077	18.858	12.639
43.613	12.383	11.245	20.534	23.172	29.888	14.395
23.017	5.59	11.382	9.817	12.25	17.836	11.602
40.095	5.541	15.58	19.747	18	23.163	16.669
65.822	38.829	19.949	35.867	44.91	47.006	30.198
16.698	5.04	13.911	13.078	10.297	8.992	4.776
23.069	9.46	17.115	19.894	21.276	23.511	20.527
8.002	10.779	5.924	8.67	12.251	10.716	6.226
38.365	11.327	21.417	21.164	35.297	18.374	18.224
25.401	16.678	10.332	18.304	12.835	16.669	7.126
23.224	3.921	6.545	11.109	9.214	10.348	3.165
38.884	15.136	12.866	19.101	23.513	26.799	24.331
79.307	36.13	54.046	48.884	54.697	48.836	37.352
26.77	10.433	10.357	14.122	15.984	21.869	6.156
17.65	10.723	15.219	13.806	24.279	12.48	11.005
30.925	22.79	24.537	25.28	34.104	28.933	29.737
16.279	12.404	14.386	12.544	15.401	11.38	12.932
22.375	3.839	6.21	7.497	10.967	11.997	1.992
93.166	47.797	56.656	58.383	55.521	58.883	38.055
160.56	76.414	84.114	94.938	106.437	114.362	85.661
38.939	14.456	18.318	38.655	39.677	39.261	33.935
32.333	7.671	15.443	12.416	24.74	16.525	17.269
54.539	31.679	38.693	41.262	36.646	38.728	33.51
135.019	65.812	85.151	100.836	127.535	127.133	112.706

42.913	22.587	18.39	29.966	36.59	21.85	24.477
46.976	20.725	22.326	32.164	34.392	32.483	12.809
64.364	28.481	39.268	34.979	39.63	58.11	31.606
36.404	12.338	13.862	20.935	16.388	28.252	7.508
39.037	15.421	13.792	17.031	21.878	15.585	8.078
35.393	20.352	19.19	18.371	25.683	19.432	25.888
60.435	24.816	26.632	58.974	53.401	84.39	39.013
31.444	12.666	7.473	11.934	19.602	19.623	10.511
29.622	6.685	5.324	8.3	5.473	23.519	0.513
20.731	5.36	14.198	10.322	16.302	12.335	15.482
51.198	18.296	20.969	34.782	37.34	31.44	25.373
23.623	8.79	6.995	13.206	14.63	13.553	6.526
81.874	35.996	46.11	48.365	46.134	54.257	31.563
46.786	9.203	8.841	28.066	21.334	40.154	5.968
22.497	8.269	24.608	18.021	24.144	29.153	10.334
19.009	9.985	16.267	6.345	12.141	10.814	6.786
26.938	15.406	16.38	16.402	19.431	20.243	10.035
5.42	0.031	5.804	3.692	2.563	0.526	0.024
58.492	16.557	13.39	34.614	28.812	40.832	24.977
24.463	7.565	10.223	13.202	14.514	13.755	4.409
4.286	3.211	2.009	2.714	0.081	0.125	0.115
107.695	57.484	32.197	38.799	55.112	80.113	41.217
25.1	5.818	6.025	16.701	11.735	20.015	6.266
100.24	58.6	69.203	79.673	89.012	88.026	69.93
39.154	15.46	22.361	19.071	8.309	10.444	4.698
36.329	14.586	27.137	22.284	28.929	32.936	16.067
20.965	5.028	11.14	12.205	17.318	10.871	11.438
35.623	14.676	11.653	24.652	17.162	24.79	6.542
90.994	39.036	32.031	63.281	62.954	65.192	36.726
71.899	32.721	26.303	41.269	35.288	31.363	31.691
66.657	18.15	17.202	33.582	31.608	34.716	15.624
43.545	20.159	31.395	23.164	29.578	21.584	13.206
31.921	8.668	11.789	18.346	25.924	32.898	17.129
20.512	8.557	4.813	16.113	13.408	9.86	5.352
46.476	23.017	20.164	27.212	24.834	35.664	21.16
29.868	13.02	12.557	13.519	18.632	11.809	10.171
60.632	11.32	15.438	34.341	29.698	43.609	17.213
22.556	10.136	21.235	21.019	21.322	25.824	21.382
23.604	17.335	12.155	12.946	15.988	16.167	8.345
54.833	27.774	19.963	28.233	26.464	42.177	30.136
18.669	12.891	12.241	8.292	14.873	4.819	8.159
17.533	11.999	10.797	8.617	8.139	2.881	4.502
37.962	15.356	19.184	31.9	36.742	39.946	32.151
25.694	7.718	12.314	13.591	11.733	16.049	5.083
25.619	7.419	13.66	13.842	14.306	20.705	9.378
16.15	6.55	8.411	11.735	11.348	5.162	4.999
22.903	8.391	13.815	14.413	18.814	20.523	10.219
23.396	6.564	20.859	10.524	11.524	14.853	15.053
16.998	20.964	8.804	17.113	16.524	5.981	9.95
45.04	13.697	15.469	23.307	23.291	31.489	22.41

36.261	16.724	11.456	19.33	27.776	22.039	21.81
35.032	15.765	11.322	17.893	23.649	18.263	10.039
44.132	10.672	14.807	21.808	20.494	27.331	15.674
21.62	11.999	10.645	11.911	18.434	14.103	10.124
42.851	18.686	16.98	26.682	25.038	29.235	24.483
28.884	11.839	13.809	16.873	16.605	20.055	7.133
34.709	16.172	8.749	16.691	26.055	18.696	14.683
37.839	14.929	17.784	25.537	27.983	26.917	14.459
35.378	12.094	10.846	21.979	24.508	20.714	11.766
39.589	17.771	12.672	22.48	28.623	18.054	18.169
5.987	3.618	3.656	3.434	3.101	2.127	0.892
10.276	3.979	6.727	7.931	9.264	1.951	4.493
46.948	23.918	32.893	36.539	36.306	50.595	30.302
31.059	18.101	20.78	23.281	30.522	27.421	25.345
47.697	15.995	19.344	23.538	29.057	34.008	23.312
68.451	36.816	43.057	44.522	51.515	54.579	34.509
40.023	10.875	8.562	25.293	23.087	22.325	4.973
35.969	12.984	7.286	19.78	27.335	23.372	11.63
10.486	6.256	6.615	8.304	9.2	4.815	5.652
24.091	17.564	14.389	16.355	24.576	14.812	15.228
61.134	22.772	37.21	40.416	49.55	59.575	36.225
38.781	24.073	20.312	31.341	29.205	24.885	22.603
50.245	20.526	26.54	26.446	32.235	40.479	26.144
33.638	15.075	16.103	20.508	25.379	25.77	20.903
53.215	17.931	15.965	24.427	21.193	34.167	6.925
41.014	22.321	23.38	21.555	31.638	35.393	27.664
23.909	6.734	7.767	12.404	10.782	17.255	8.681
56.098	27.75	27.015	36.246	35.619	46.132	34.713
50.22	23.335	27.106	32.841	29.856	40.16	25.519
21.604	5.176	10.59	15.643	15.344	14.622	6.23
23.06	13.768	11.807	7.198	16.817	7.909	14.562
54.15	27.223	21.905	28.496	36.967	52.985	24.779
31.984	9.605	19.961	22.284	26.595	33.209	28.799
24.292	12.787	13.68	11.9	14.586	19.541	7.292
34.908	11.022	15.51	27.197	28.188	37.995	21.825
11.53	8.437	8.497	3.838	5.342	7.206	3.352
16.625	7.165	5.639	6.373	12.09	9.522	1.511
40.607	9.604	12.811	20.234	20.845	27.797	19.794
33.763	12.846	14.08	17.914	24.002	22.744	19.535
72.452	23.434	42.411	52.098	55.249	61.629	31.884
44.951	13.646	15.516	23.412	23.784	30.748	22.556
25.055	15.108	16.53	18.253	24.572	14.927	14.806
27.358	17.03	14.692	13.55	16.826	12.384	21.591
33.685	20.846	25.162	21.883	29.23	27.625	27.893
26.202	10.046	9.259	14.188	14.141	17.929	6.722
49.878	21.064	21.21	32.532	31.69	39.992	20.822
36.117	28.9	16.252	13.676	30.927	33.278	18.839
157.123	65.839	74.517	104.376	105.704	118.958	77.404
39.793	24.232	30.895	18.616	21.108	25.641	12.549
15.861	2.222	4.386	10.498	11.399	7.627	2.506

58.18	19.382	25.842	38.016	25.565	35.634	16.931
25.342	10.934	25.843	15.23	13.646	21.474	8.162
35.975	26.729	17.526	12.53	27.924	33.132	16.611
22.031	7.257	11.034	13.061	14.623	19.581	9.051
28.568	10.407	7.543	12.738	19.513	23.076	9.184
70.191	25.714	22.449	37.515	38.657	53.015	33.104
38.719	17.353	17.521	24.755	29.544	21.112	16.031
31.968	14.512	16.301	22.766	22.389	29.154	30.258
17.671	12.65	12.114	15.257	17.305	9.262	9.827
32.466	10.261	8.238	16.501	21.383	21.56	8.266
76.222	41.499	31.605	49.73	45.237	64.353	36.643
33.69	5.499	20.269	29.603	25.79	30.792	14.605
55.022	36.085	46.965	41.061	42.207	38.296	28.562
63.089	39.374	40.654	42.794	47.022	44.889	29.765
37.919	8.784	9.014	19.523	19.318	20.18	4.049
36.66	7.526	12.04	17.7	19.168	20.038	10.919
47.325	18.252	6.867	23.51	19.534	41.049	4.429
35.228	7.346	9.081	16.077	18.43	22.272	7.732
27.224	10.17	16.94	16.63	24.492	19.25	5.675
35.234	7.349	9.086	16.101	18.431	22.248	7.754
22.356	8.315	10.361	14.603	13.457	17.665	12.11
43.87	14.755	18.607	29.15	34.374	27.113	22.267
49.104	11.876	28.288	26.142	30.143	34.503	21.353
51.282	13.513	31.451	24.043	30.805	30.297	20.546
27.294	14.631	11.893	20.132	16.914	15.438	8.808
71.637	26.035	25.709	35.823	49.632	64.093	48.594
9.651	1.3	4.575	8.622	6.712	8.376	3.498
20.458	18.699	11.636	17.65	21.424	12.417	10.593
57.545	20.873	21.007	31.712	31.251	36.426	18.842
22.56	10.765	12.081	13.495	22.955	14.946	8.843
129.577	61.299	70.802	93.749	94.842	104.072	82.857
48.769	20.63	13.878	30.884	29.764	28.015	16.281
12.201	1.572	3.454	1.924	5.022	5.301	0.153
41.646	17.289	27.265	29.513	28.982	23.971	24.209
57.059	21.156	24.41	40.475	37.421	42.7	31.624
33.718	14.807	11.492	14.563	20.87	16.444	14.874
61.214	24.656	33.731	39.951	48.575	43.656	30.13
20.577	8.52	10.165	13.173	11.07	11.589	9.35
27.349	8.601	12.427	13.611	19.355	13.979	7.468
33.711	25.379	22.218	14.809	29.947	35.603	18.14
21.47	8.639	13.901	16.579	12.619	11.804	10.412
25.93	15.409	15.877	14.949	24.07	23.042	19.554
43.14	17.171	16.154	23.532	28.837	26.624	7.42
25.189	15.674	17.279	11.87	23.432	11.587	12.125
23.109	7.105	10.482	11.423	13.02	15.886	5.532
48.448	16.974	21.786	41.155	52.726	55.269	32.937
37.393	13.862	10.903	15.869	18.737	17.366	9.107
28.087	11.896	10.609	14.739	18.816	23.787	7.884
22.665	13.724	15.445	24.936	21.133	18.338	15.824
25.883	11.695	13.677	15.231	15.783	16.759	11.946

36.507	14.406	21.932	32.44	30.48	29.367	16.373
33.532	13.813	11.315	14.81	14.358	18.745	10.52
55.307	33.913	42.645	42.041	39.588	31.672	28.55
65.628	23.223	34.081	33.435	40.286	66.548	35.676
9.227	7.105	11.906	8.119	8.768	5.901	8.363
38.046	22.278	24.688	20.525	20.19	22.306	7.899
23.809	9.599	9.027	13.988	16.229	16.424	7.462
46.888	12.629	13.968	25.808	13.836	28.214	9.571
41.186	11.243	7.319	23.822	22.859	31.542	6.481
36.543	18.55	24.504	24.871	16.445	17.36	16.683
36.104	13.472	6.92	14.733	11.737	20.593	9.083
51.631	16.863	17.796	30.146	24.839	35.024	16.966
41.005	10.936	14.361	28.566	22.828	27.649	12.582
23.256	17.041	15.072	17.031	18.808	16.681	14.142
21.232	4.642	13.249	18.606	25.203	21.888	28.509
46.761	11.713	17.608	40.672	36.46	36.124	12.772
28.828	16.356	4.725	9.453	9.123	15.334	1.139
60.835	27.386	31.922	38.89	36.714	44.462	32.043
150.748	60.851	83.584	95.449	101.826	106.325	83.033
52.911	30.432	30.099	46.934	43.778	57.204	31.09
111.597	70.417	88.644	120.413	136.562	128.334	116.117
53.305	27.016	25.614	23.437	36.749	35.401	33.655
30.687	12.037	13.073	17.802	20.855	27.462	17.642
27.227	10.209	16.995	11.185	18.124	17.144	9.202
20.971	11.469	14.749	12.399	15.644	10.28	12.536
39.835	18.197	13.62	27.125	26.628	19.706	16.947
28.776	11.694	18.019	19.8	20.745	16.588	16.119
18.316	10.246	15.07	17.461	15.905	15.057	12.373

RDF060e	RDF065e	RDF070e	RDF075e	RDF080e	RDF085e	RDF090e
13.506	8.301	5.656	7.152	9.845	5.944	4.557
22.871	21.997	15.123	7.924	11.42	27.831	10.944
21.195	25.026	19.437	19.268	16.153	6.442	12.982
4.647	2.202	2.763	0.019	0.591	0.76	0
7.798	2.413	7.446	3.839	0.777	4.559	4.895
3.167	6.84	2.016	2.627	0.32	1.524	0
36.957	39.128	36.576	27.944	27.58	20	19.567
0.75	0.626	0	0	0	0	0
17.891	9.784	11.96	4.822	11.875	2.16	2.226
18.451	12.128	9.279	7.367	6.85	11.763	8.336
89.197	106.441	97.073	77.366	71.531	67.573	47.576
34.527	28.943	24.521	24.888	22.744	22.518	27.806
22.096	19.347	13.942	13.918	9.779	9.88	7.528
39.763	37.879	41.964	13.387	24.655	21.553	23.777
23.617	25.469	19.154	17.919	9.939	10.678	7.038
15.572	14.936	15.271	10.906	17.272	6.321	13.463
5.667	3.943	1.64	3.072	1.51	1.953	0.736
54.299	48.822	46.349	26.842	33.79	40.298	28.531
8.962	14.17	11.278	3.87	7.309	16.614	5.84
20.789	18.554	12.541	14.211	14.354	18.691	9.454
26.145	21.36	16.916	19.482	11.317	8.299	9.021
11.236	5.464	16.34	0.878	7.971	0.001	2.915
14.999	3.878	4.619	4.289	8.199	3.408	4.637
9.222	13.316	12.347	11.648	10.331	7.829	10.631
36.739	44.76	39.315	46.933	25.66	31.381	30.129
37.649	31.015	25.506	23.481	16.986	11.918	8.612
11.024	14.161	15.435	7.507	8.82	20.724	10.256
51.231	65.311	40.162	66.82	56.199	67.591	60.169
23.349	21.206	18.01	19.738	19.23	9.584	12.012
19.097	26.434	18.601	13.414	11.949	23.914	13.035
60.123	49.166	48.73	54.023	42.113	43.979	32.261
72.298	75.763	84.306	70.374	60.874	61.011	42.172
15.937	13.651	14.408	12.569	10.182	13.056	12.474
25.926	26.224	25.801	18.293	18.839	15.264	14.849
54.758	69.03	51.152	50.449	44.487	32.164	28.556
18.846	33.375	19.985	19.092	22.379	26.067	17.214
30.767	28.168	19.855	21.021	12.651	12.74	9.133
7.431	6.604	2.215	1.787	0	0	0
38.318	48.474	48.575	39.557	33.965	34.928	43.373
15.616	18.043	15.795	16.724	14.105	12.685	7.791
34.494	27.89	32.027	17.676	23.166	24.45	20.745
12.397	5.218	6.205	1.024	4.285	0.024	0.612
62.143	82.294	59.821	49.199	45.498	51.215	54.472
14.893	7.163	6.773	10.285	4.352	7.461	2.894
41.097	37.446	33.821	25.439	25.028	31.034	23.253
17.988	13.867	8.718	7.351	11.148	12.367	11.62
16.238	17.039	11.287	14.149	14.836	14.079	8.11
23.444	19.637	21.745	13.565	17.03	18.733	14.13
20.192	12.348	14.002	5.523	15.433	15.608	15.51

26.494	23.34	21.414	19.887	18.285	14.161	19.33
22.374	15.622	13.816	12.338	13.889	17.232	15.021
44.308	48.378	33.076	24.419	33.321	28.451	34.025
23.159	22.41	12.972	17.728	20.374	12.953	13.143
31.23	24.684	25.305	25.614	22.421	8.461	10.969
19.78	23.415	13.948	13.304	12.089	13.231	16.06
22.976	28.67	28.93	21.284	16.489	24.297	18.705
32.965	31.68	24.381	22.67	26.586	25.027	24.295
20.698	15.691	12.635	12.857	10.648	11.31	13.305
30.356	23.654	21.183	17.936	24.32	22.487	11.162
22.852	17.345	14.461	16.784	17.26	12.718	17.423
13.398	19.493	17.076	16.832	16.264	10.937	10.597
21.959	22.809	11.713	10.651	18.013	12.308	8.355
13.844	22.031	18.131	18.441	13.955	10.496	13.594
14.577	17.752	12.815	13.657	22.389	11.494	9.737
14.152	26.995	15.013	11.937	13.712	12.623	10.718
32.888	30.428	27.429	24.505	18.971	16.706	12.453
31.373	22.575	24.021	25.462	18.768	21.514	14.589
3.466	7.58	4.736	3.762	0.667	1.902	0
25.591	19.689	16.988	8.79	11.265	8.387	6.483
21.113	17.417	18.603	16.597	7.627	12.006	7.045
4.021	12.971	10.329	9.619	5.942	7.205	3.75
19.875	15.616	20.133	16.386	9.82	12.307	5.602
13.02	11.45	9.15	6.283	9.325	6.027	3.972
9.431	9.253	6.384	4.334	2.687	4.07	4.832
3.704	7.048	4.89	8.831	3.863	7.577	7.186
12.731	18.726	12.535	12.55	8.503	14.799	9.44
30.362	17.665	16.549	14.642	11.444	10.337	17.271
15.962	10.201	5.229	5.815	1.33	1.465	1.225
24.707	22.044	16.398	11.258	9.553	16.539	13.167
30.09	44.301	31.002	30.79	21.508	21.903	10.571
9.099	2.805	4.673	1.838	0.543	0.63	0.005
20.366	18.112	18.389	21.664	14.658	11.174	13.103
3.671	6.482	2.649	3.231	0.724	0.001	0.11
16.468	23.996	13.654	15.382	17.765	14.603	4.544
14.507	4.166	5.685	1.071	0.572	1.018	0
3.776	13.652	6.331	8.925	4.01	5.161	2.265
21.343	16.998	12.516	16.811	9.154	8.363	5.803
44.369	28.095	21.539	24.275	14.707	9.724	9.601
15.868	11.521	9.699	6.97	8.365	2.438	1.18
5.848	6.916	6.645	1.689	4.277	0	2.577
23.276	36.756	28.739	22.503	21.785	18.342	19.137
9.866	5.958	2.675	5.595	3.914	2.325	3.448
8.502	11.514	5.968	2.227	0.748	6.126	0.84
46.846	32.479	24.354	25.017	14.215	19.972	14.23
103.809	82.692	80.121	70.744	73.016	60.816	81.959
44.599	37.361	38.509	30.379	36	24.966	20.112
18.819	21.31	18.402	11.603	12.697	7.292	3.863
38.051	29.065	25.866	27.581	16.43	17.65	4.803
139.517	144.91	131.656	132.726	109.572	109.625	103.389

22.106	24.018	21.531	27.139	22.984	38.835	20.512
20.618	25.285	26.38	24.269	26.624	13.075	15.36
36.13	35.886	35.902	20.107	18.595	17.18	16.605
16.182	10.922	7.922	14.209	5.999	10.54	7.037
12.579	13.667	7.357	6.358	8.599	12.483	6.838
25.742	20.144	15.589	22.182	9.259	6.733	1.945
87.693	60.963	70.398	49.189	45.057	55.986	32.984
11.405	7.66	11.578	14.349	6.953	8.29	6.891
7.858	14.183	10.273	10.081	4.601	7.166	8.986
16.39	12.354	10.749	11.598	5.804	4.195	0.593
24.838	30.24	17.119	14.142	21.282	19.71	10.838
14.399	10.612	12.195	11.355	10.198	9.272	6.147
36.129	32.991	33.955	25.461	19.302	22.204	20.603
18.31	28.63	16.924	12.759	13.495	35.456	16.147
18.079	10.909	9.312	6.139	2.936	5.706	1.086
6.003	6.286	5.763	1.703	6.107	3.627	3.001
13.406	16.003	15.955	11.592	9.143	8.31	7.058
0	0	0	0	0	0	0
30.368	36.718	26.748	32.615	24.718	29.536	22.209
6.558	9.126	10.56	3.834	3.365	5.4	4.462
0	0	0	0	0	0	0
38.712	63.176	55.258	48.596	49.757	27.267	40.865
11.112	14.774	10.923	9.279	3.152	4.687	6.493
70.736	70.103	82.931	49.324	51.102	58.583	38.789
4.562	1.555	0.839	0	0	0	0
22.767	23.766	21.538	11.309	11.45	5.228	2.697
12.644	7.899	5.144	7.921	3.044	3.507	7.748
12.061	18.484	12.138	14.99	9.294	12.058	9.225
55.772	56.47	42.339	36.845	44.654	43.054	30.452
18.908	38.42	31.613	19.501	26.867	27.335	37.001
17.659	36.133	31.851	22.54	20.199	23.781	31.239
12.165	12.182	18.242	12.593	14.116	15.258	16.702
34.794	23.846	28.125	20.609	11.386	18.956	14.266
4.771	8.105	4.632	7.644	1.594	3.741	0.888
23.931	11.99	10.53	3.636	2.506	2.833	0.619
9.336	12.398	6.357	5.263	5.191	5.655	2.581
28.246	36.601	27.133	12.459	19.423	38.843	27.365
15.534	19.909	23.52	18.172	9.767	10.125	10.334
8.659	8.543	10.653	6.929	6.634	7.514	5.144
35.819	41.407	38.577	30.184	37.34	26.789	30.279
4.563	5.438	5.071	2.06	2.987	1.309	0.398
2.884	3.243	1.97	0.112	1.381	0.011	0
42.035	35.995	23.91	16.028	16.143	13.892	13.26
5.166	16.456	10.247	5.042	4.006	9.801	3.918
17.306	10.666	12.919	9.384	8.054	6.603	1.351
11.487	7.22	4.239	2.707	3.616	4.149	0.436
15.167	7.03	8.926	8.557	10.25	11.381	9.115
11.586	7.62	10.387	8.03	4.171	3.963	1.781
4.129	3.225	1.318	0.731	0	0	0
35.307	22.936	23.683	15.339	12.486	16.963	16.539

14.985	15.289	15.19	11.561	10.239	5.896	9.823
10.525	18.217	18.646	4.795	6.833	14.79	15.2
30.802	20.48	22.808	14.045	8.101	15.315	15.83
15.246	13.189	13.444	7.018	8.304	0.995	1.739
36.773	25.772	34.741	19.943	24.779	18.218	21.979
10.482	15.893	9.772	6.556	7.172	11.04	5.337
10.308	14.61	9.813	7.12	11.88	8.693	4.087
24.35	18.217	15.381	10.12	10.516	4.168	3.064
16.418	16.001	18.401	7.925	11.839	3.156	3.76
14.757	15.694	16.97	7.55	6.745	4.647	4.935
0.001	0.002	0	0	0	0	0
1.348	2.784	0.29	0.536	0	0	0
35.448	26.803	24.585	11.835	5.984	5.802	4.82
22.928	20.024	19.185	30.036	24.758	19.088	10.301
27.221	25.622	18.811	19.035	19.092	30.456	18.742
43.976	31.963	19.995	22.78	18.934	11.893	13.359
12.808	20.793	12.787	4.633	4.884	15.487	13.266
10.1	12.209	10.039	5.482	4.03	20.607	7.204
2.282	3.834	0.001	0.004	0	0	0
19.23	11.647	11.264	6.35	10.721	4.876	4.445
59.582	56.574	42.804	50.07	42.817	46.15	29.966
20.448	11.389	10.632	4.335	1.258	1.789	0
30.709	26.198	28.578	21.747	18.825	21.908	23.693
25.157	29.341	22.918	17.829	18.466	10.895	7.662
15.185	20.508	13.453	17.685	10.668	19.809	14.78
21.957	30.691	23.283	25.138	31.279	26.137	26.743
17.358	9.768	6.797	3.051	1.43	2.354	3.853
30.687	25.196	23.029	11.578	11.62	9.591	8.586
25.996	18.057	16.179	6.82	7.641	3.794	1.761
11.769	5.214	8.292	1.225	5.775	5.253	3.496
10.588	8.114	12.13	12.69	10.452	7.298	5.425
32.72	38.416	22.746	29.1	18.661	19.555	20.59
36.546	23.906	25.595	9.19	8.131	7.874	5.607
13.549	12.703	14.647	9.451	8.224	6.358	3.549
49.332	25.573	36.529	12.415	12.017	11.075	9.681
1.755	7.288	3.314	3.762	5.471	3.559	3.511
4.6	5.257	2.241	2.233	1.44	1.707	0
29.713	20.792	11.953	9.318	5.533	13.641	11.465
17.802	16.52	13.867	15.668	10.929	7.14	6.031
49.755	42.746	37.771	27.902	26.701	31.996	32.865
36.008	21.916	22.381	15.745	12.263	17.078	17.107
15.758	10.95	10.522	8.215	12.318	6.129	13.562
13.976	16.967	9.7	10.425	7.159	4.471	5.626
17.529	26.371	17.832	36.298	18.076	15.752	18.176
15.742	9.053	10.009	6.734	8.07	1.028	0.965
27.932	16.958	19.279	6.633	8.589	5.679	2.41
23.492	20.992	23.634	13.635	13.816	9.86	11.567
92.887	103.97	58.379	82.219	60.117	61.05	64.289
10.82	12.631	2.251	5.975	0.002	4.003	0.001
9.147	3.186	8.281	0.166	3.259	5.804	1.332

27.22	10.467	15.744	5.086	4.563	0.79	0.353
12.213	6.76	3.376	4.056	1.355	0.499	0
22.486	17.781	18.938	13.767	15.898	9.539	9.597
13.959	4.806	6.68	5.337	2.081	1.253	0.014
11.052	9.503	12.308	7.722	5.874	12.253	6.657
40.3	51.297	41.163	43.279	32.264	41.723	33.383
20.925	19.402	22.224	16.49	17.331	14.686	11.934
29.032	28.126	22.307	15.936	14.027	7.003	6.212
7.658	8.735	6.719	4.634	4.366	7.173	5.098
13.52	10.608	11.249	4.713	5.449	15.395	5.759
38.376	57.755	39.504	58.07	27.289	31.584	25.011
37.328	20.705	13.979	11.187	18.085	24.581	10.539
32.581	25.413	22.329	9.131	13.995	13.213	12.822
27.269	37.433	18.309	18.539	18.297	13.236	12.647
6.196	15.127	8.346	3.798	4.331	12.131	7.39
10.891	7.213	12.357	9.454	9.303	9.221	7.063
14.93	25.674	24.406	20.305	10.077	21.485	19.361
14.677	12.465	6.662	7.747	6.226	14.326	4.95
19.316	6.263	12.89	3.5	5.748	1.862	0.299
14.694	12.458	6.666	7.721	6.235	14.332	4.962
9.686	14.82	2.921	1.234	0.839	1.829	2.004
27.884	23.691	26.145	22.348	27.853	11.332	13.488
28.807	21.002	28.088	11.88	19.111	12.523	9.468
25.825	21.565	21.859	15.405	15.692	18.194	12.935
8.943	9.841	5.384	15.343	8.25	9.671	11.695
57.871	47.774	54.059	40.501	34.909	32.504	33.266
5.409	1.991	1.389	2.855	2.426	4.559	1.371
8.266	3.918	1.396	0.333	0	0	0
23.149	29.291	20.321	19.959	25.378	18.03	15.347
14.998	16.922	17.413	13.645	17.03	13.159	8.013
83.091	76.025	55.361	55.698	59.48	56.766	51.072
22.673	23.285	27.8	11.377	14.287	11.39	8.124
2.515	0.288	0.655	0	0	0	0
20.546	12.003	11.018	13.685	9.942	11.017	8.07
46.623	34.733	40.064	29.506	30.146	21.246	28.489
17.851	12.309	16.051	11.918	8.06	8.695	10.475
37.396	33.083	40.702	25.254	21.161	22.457	16.878
8.46	11.203	9.715	4.587	6.536	1.281	0.249
13.997	16.6	8.345	12.464	5.805	7.756	2.723
24.528	17.058	24.308	18.463	11.794	15.134	13.91
13.538	12.898	12.872	6.489	7.031	4.956	3.747
14.149	25.64	14.406	16.528	12.653	9.803	5.809
23.583	27.06	21.882	13.953	15.986	24.331	13.052
13.938	20.312	14.644	14.339	16.477	9.458	10.18
8.016	10.948	8.177	6.367	3.86	5.304	4.414
61.064	47.17	38.537	31.957	27.001	18.653	11.841
19.685	17.565	13.004	11.681	11.264	11.956	15.351
18.592	12.46	11.101	8.346	9.827	2.431	1.179
18.025	11.924	10.697	7.32	1.035	2.236	1.417
14.545	11.27	6.901	6.631	8.201	6.016	3.73

40.287	26.548	15.729	19.306	16.934	29.002	12.055
13.221	10.055	8.426	7.652	6.068	4.743	9.252
18.421	24.382	19.64	16.421	9.277	10.049	4.849
44.161	34.402	39.947	23.753	21.702	19.11	14.474
7.565	2.781	1.154	1.686	0	0	0
12.473	8.258	5.566	4.065	0.994	0.06	1.394
10.034	9.395	7.236	4.734	5.51	3.91	0.201
13.137	19.761	15.76	19.029	6.988	14.697	20.315
19.931	16.374	13.049	10.123	6.807	20.868	16.303
15.267	18.352	14.899	12.017	15.294	12.227	7.696
5.539	18.147	10.616	10.435	7.693	7.621	10.628
27.477	18.607	21.224	12.449	10.937	6.799	5.421
17.379	18.174	15.731	15.387	16.387	9.776	15.584
16.925	9.745	11.802	6.525	10.04	3.931	3.679
18.912	15.685	15.657	12.062	9.578	9.08	7.271
28.879	37.512	27.553	17.399	13.645	28.638	21.873
5.048	8.191	0.72	4.141	0	3.445	0.001
35.668	36.179	36.834	31.455	24.043	26.937	28.38
97.61	93.513	70.757	76.797	67.184	61.305	58.308
50.634	46.092	33.363	28.118	29.804	28.364	25.138
132.74	139.08	121.863	106.332	100.103	87.007	89.436
29.863	34.31	32.579	27.493	17.892	12.938	16.224
21.207	15.626	21.232	16.182	11.28	6.996	5.555
15.195	13.491	18.204	12.322	11.44	7.183	9.156
16.137	6.312	6.862	5.273	9.22	6.98	7.403
17.9	18.422	14.739	13.712	14.591	14.871	15.115
22.564	22.853	19.904	20.826	20.704	12.5	7.312
15.594	6.577	8.855	6.491	8.061	3.29	3.572

RDF095e	RDF100e	RDF105e	RDF110e	RDF115e	RDF120e	RDF125e
2.802	1.733	0.046	1.842	0.105	0.136	0
12.407	11.347	20.747	17.956	5.355	7.589	11.868
3.534	5.747	1.045	2.11	0.748	0.019	0
0	0	0	0	0	0	0
3.265	1.217	2.186	0.085	0.191	0	0
0	0	0	0	0	0	0
20.556	16.383	13.399	20.237	11.094	8.626	6.758
0	0	0	0	0	0	0
0.358	0.11	0	0	0	0	0
8.926	8.389	9.582	6.317	4.069	3.075	1.737
37.328	34.817	29.659	18.708	11.533	8.704	7.533
20.617	26.718	23.822	30.217	21.314	15.335	15.49
6.247	2.514	2.803	0.731	0.837	0	0
8.66	11.632	8.073	9.986	4.156	2.797	0
3.869	0.607	0.159	0	0	0	0
16.184	11.508	11.931	7.126	2.305	1.88	0.039
0.762	0	0	0	0	0	0
15.82	15.635	10.655	9.502	9.429	7.549	2.719
9.773	5.171	9.963	8.654	2.763	2.894	6.651
11.616	8.142	4.587	3.157	2.007	1.711	1.466
6.54	5.45	4.657	1.687	0.888	0.004	0
0	0.774	0	0	0	0	0
4.892	6.336	9.912	3.16	2.496	2.67	2.159
9.725	4.801	5.144	8.512	7.318	3.534	3.907
16.936	18.595	9.449	9.717	3.605	6.467	0.102
12.897	3.324	5.714	4.782	1.832	0.452	0.496
15.65	13.441	13.259	14.541	6.68	7.055	9.252
41.371	39.763	42.315	33.726	14.36	18.917	15.404
10.605	13.229	3.67	4.174	2.503	1.217	0.154
16.209	9.088	19.641	14.925	13.402	7.383	15.241
33.075	29.933	27.365	24.89	14.046	16.173	13.496
36.449	40.354	28.261	30.433	24.131	27.779	11.668
10.404	8.624	7.048	5.057	2.161	1.072	0
6.028	5.731	1.392	0.679	0.018	0	0
22.616	16.297	15.5	8.596	3.808	0.916	0.151
19.981	16.313	16.005	16.907	13.425	12.024	14.451
6.229	6.93	4.944	0.286	0.387	0.177	0
0	0	0	0	0	0	0
29.863	25.232	16.121	20.571	15.354	17.265	11.707
11.401	10.628	5.702	7.623	2.647	7.016	1.505
15.765	18.747	14.633	8.553	10.016	6.98	2.89
0	0	0	0	0	0	0
32.451	35.183	36.178	35.6	20.894	17.535	27.018
4.403	5.227	1.558	0.455	2.388	0.96	0
19.426	18.739	14.831	10.613	9.816	7.902	9.763
11.292	5.965	7.848	3.564	0.065	0.725	0
14.819	8.359	11.623	9.511	2.148	2.768	0.796
14.135	12.445	13.556	13.21	7.513	3.405	5.654
9.868	9.184	9.454	11.175	6.439	6.327	5.478

12.945	17.712	17.347	13.009	15.927	13.032	14.845
14.982	11.377	13.663	13.374	17.13	9.714	12.911
39.95	23.59	28.854	25.848	17.78	12.821	17.797
10.208	10.48	12.711	11.149	12.804	10.438	7.231
6.087	2.472	2.694	3.442	0.297	2.535	0
11.859	10.617	14.788	11.119	5.038	9.064	7.367
20.852	10.67	14.832	11.335	9.546	6.022	12.003
24.521	18.057	27.357	23.937	22.744	15.79	12.853
4.144	6.727	6.627	5.445	2.74	1.112	0.194
15.73	13.56	17.796	14.313	10.73	14.413	11.093
13.709	9.012	15.844	9.196	9.353	8.875	6.767
11.271	7.442	3.857	2.291	0.336	0	0
6.355	9.33	4.153	4.856	0.188	1.943	0.147
9.953	7.346	5.688	11.979	10.408	7.599	5.081
9.687	7.895	6.454	6.664	5.815	5.922	4.831
11.269	8.56	6.159	4.506	5.611	1.822	1.399
18.645	13.997	7.852	8.116	4.135	2.064	0.812
13.443	11.331	14.895	9.568	8.765	3.143	4.001
0.596	0	0	0	0	0	0
1.487	0.75	0.802	0.966	0	0	0
6.631	4.342	1.313	0.314	0	0	0
6.47	2.288	1.626	0.884	0	0	0
5.788	4.816	0.069	0.044	0.021	0	0
4.274	0.077	1.246	0.054	0	0	0
1.817	1.331	1.263	0	0	0	0
6.029	6.467	6.666	3.025	3.098	3.364	2.83
11.644	7.726	12.257	6.528	12.183	4.975	6.356
11.397	12.028	12.581	5.611	15.425	7.095	9.456
2.239	0	1.091	0.001	0.003	0	0
2.26	3.083	2.916	6.437	0.98	3.228	0
23.309	20.905	17.295	11.798	7.902	8.554	8.038
0	0	0	0	0	0	0
5.529	6.695	2.961	1.764	0.215	0.764	0
0	0	0	0	0	0	0
4.242	3.915	3.099	1.681	0.201	0	0
0	0	0	0	0	0	0
1.173	3.937	1.492	0	0	0	0
4.757	2.75	1.292	0	0	0	0
9.689	7.835	3.778	2.707	2.537	0.403	0.012
0.782	0	0	0	0	0	0
0	0	0	0	0	0	0
9.991	2.163	1.098	0.055	0.021	0	0
5.237	0.449	0.112	0	0	0	0
0.751	1.777	0	0	0	0	0
10.903	10.683	5.416	4.09	2.637	3.174	0.47
65.494	66.457	60.376	47.11	73.681	46.546	63.288
11.5	6.053	5.649	2.552	1.786	1.886	0.003
3.717	1.474	0.463	0	0	0	0
9.954	1.964	4.685	1.048	1.836	0	0
101.597	83.771	78.056	57.927	52.207	42.596	33.212

30.606	24.019	16.666	14.084	18.477	15.774	9.298
13.754	14.388	10.214	15.33	10.715	11.392	7.284
13.854	7.296	11.595	8.603	2.881	3.364	0.352
10.873	2.278	5.553	4.096	1.496	0.792	0.326
11.433	3.909	3.524	0.038	0	0	0
1.883	0.028	0	0	0	0	0
33.994	25.886	28.639	20.409	10.114	20.374	14.423
10.556	5.577	4.481	5.377	6.993	0.844	8.178
4.919	4.158	4.011	5.037	1.271	2.528	1.053
0.087	0	0	0	0	0	0
11.441	13.891	11.082	8.276	6.609	10.26	6.893
1.972	4.283	6.912	2.009	2.114	0.018	1.834
19.352	16.946	15.033	12.631	7.528	8.019	3.256
8.499	10.733	27.044	19.631	2.237	6.848	2.762
1.035	0.035	0.069	0	0	0	0
0	3.089	0	0	0	0	0
4.56	6.178	3.703	0.915	3.183	1.32	3.679
0	0	0	0	0	0	0
22.521	14.238	10.857	10.768	12.129	5.872	4.66
2.56	4.202	2.729	0.951	1.855	0.874	0
0	0	0	0	0	0	0
33.094	30.408	38.002	21.669	27.511	27.804	24.713
6.504	0.119	2.695	1.779	3.979	0.314	0.165
34.552	24.443	29.81	33.578	17.703	8.283	16.795
0	0	0	0	0	0	0
1.598	0.078	0	0	0	0	0
3.995	4.4	1.628	0	0.878	0	0
7.685	6.976	4.185	2.33	0.359	0.526	0.055
27.783	25.787	25.181	21.788	16.046	9.252	8.373
20.476	20.657	16.732	29.389	24.44	20.403	16.456
25.832	25.973	21.404	13.305	26.923	23.462	18.749
14.878	11.549	8.716	6.889	4.958	2.076	0.966
10.544	8.61	12.129	6.632	3.66	3.845	2.026
0	0	0	0	0	0	0
0	0	0	0	0	0	0
3.362	1.749	1.187	0.891	0.147	0	0
17.475	17.527	28.905	31.38	13.138	21.335	22.613
4.886	3.105	3.127	0.001	1.255	0	0
6.413	5.366	2.555	5.747	2.787	3.531	0.505
21.076	18.246	9.438	13.416	11.972	7.955	5.239
0.713	0.001	0.014	0	0	0	0
0	0	0	0	0	0	0
3.995	5.066	1.056	0.559	0	0	0
7.372	3.264	5.375	4.186	4.899	2.573	4.04
2.626	0.583	0.596	0	0	0	0
0.366	0.367	0	0	0	0	0
7.96	8.848	5.792	5.352	6.102	6.631	2.996
2.656	1.09	0.352	0.061	0	0	0
0	0	0	0	0	0	0
8.414	5.621	2.73	5.125	4.493	1.073	0.739

1.413	2.919	0.184	0.878	0	0	0
4.153	4.97	13.579	10.549	6.185	2.09	6.555
7.605	4.773	1.077	5.854	6.079	2.188	2.711
0.001	0	0	0	0	0	0
12.148	17.226	10.065	12.436	5.834	5.699	2.862
5.854	6.957	8.86	2.126	5.387	3.981	2.054
5.92	4.993	3.654	4.382	2.395	0	0.101
0.859	0.416	0	0	0	0	0
0.92	0	0	0	0	0	0
2.257	2.617	0.099	0	0.062	0	0
0	0	0	0	0	0	0
0	0	0	0	0	0	0
2.525	1.394	0	0	0	0	0
11.613	7.333	4.391	4.462	0.071	0.333	0
19.67	14.042	23.212	10.568	11.964	7.155	12.321
10.887	5.622	3.143	2.945	3.577	0.017	0.279
7.792	7.816	9.2	13.27	5.94	4.981	2.777
12.064	6.439	9.112	9.829	4.89	4.924	7.083
0	0	0	0	0	0	0
2.22	0.404	0	0	0	0	0
42.234	28.441	29.435	23.3	25.741	23.12	32.431
0	0	0	0	0	0	0
13.347	7.838	11.631	6.165	7.023	1.646	1.946
2.554	5.98	6.406	4.383	0.923	1.709	2.239
8.791	8.688	13.775	14.895	9.688	8.579	6.454
12.367	15.731	9.949	6.169	2.474	1.004	0.003
0.93	1.825	0	0	0	0	0
5.924	1.984	1.463	2.852	0.035	0	0
0.332	0	0	0	0	0	0
5.38	1.817	4.584	0.433	1.706	0.009	0.01
1.858	0.774	1.526	0	0	0	0
7.936	18.855	6.313	14.303	6.37	6.592	8.165
1.128	0.163	0	0	0	0	0
2.631	1.546	0.858	0	0	0	0
6.824	2.146	2.729	1.018	0	0	0
0.925	3.725	0.007	0.036	0	0	0
0	0	0	0	0	0	0
1.071	2.58	2.985	7.433	0.892	2.892	0
2.961	0.792	1.6	0.014	0.698	0	0
30.374	28.156	29.333	31.279	27.017	25.612	20.834
7.968	5.634	2.814	5.153	4.433	1.101	0.811
8.639	9.015	7.33	6.771	9.124	7.859	6.271
3.255	2.485	0.18	0.638	0	0	0
9.982	5.539	0.991	0.874	0	0	0
0.094	0	0	0	0	0	0
1.463	0.333	0.425	0	0	0	0
4.951	8.773	4.797	6.265	3.436	0.087	0.619
49.571	55.462	39.158	40.914	45.597	38.109	30.848
0.003	0	0	0	0	0	0
0	0.438	2.818	0.776	0	0	0

0	0	0	0	0	0	0
0	0	0	0	0	0	0
6.135	6.043	3.083	2.605	4.199	0.443	0.718
0	0	0	0	0	0	0
9.716	4.844	8.275	5.67	3.566	5.686	1.247
34.797	20.274	27.509	19.263	13.112	9.918	4.536
13.603	10.676	8.012	3.251	2.194	0.701	0.433
1.71	2.6	0.849	0.048	0.118	0	0
0.933	0.353	0.576	0.004	0.117	0	0
5.449	5.863	11.367	12.636	3.099	3.158	5.182
23.05	19.47	12.239	8.692	4.872	6.498	5.528
11.965	9.641	13.186	5.759	6.461	12.797	5.82
6.982	6.01	3.48	3.748	0.851	0.049	0
9.93	6.566	6.752	4.58	4.747	2.406	0.094
7.685	4.81	5.868	6.873	3.304	3.302	2.913
6.821	4.47	3.422	0.704	0	0	0
4.288	10.771	16.859	21.87	3.382	5.463	9.107
8.696	6.102	6.536	8.548	1.457	4.428	2.328
0	0	0	0	0	0	0
8.712	6.083	6.529	8.551	1.457	4.429	2.329
0	1.049	1.328	0	0	0	0
12.692	11.754	9.805	13.573	9.12	8.365	6.904
6.505	2.435	0.79	0	0	0	0
7.231	10.716	3.985	2.471	0.333	0	0
8.01	7.95	6.645	15.67	8.144	12.843	7.519
21.897	26.855	22.898	16.058	18.575	15.105	15.713
0.01	0.015	0.01	0	0	0	0
0	0	0	0	0	0	0
10.351	16.416	16.968	17.326	13.291	11.721	10.068
4.178	1.92	0.327	0.008	0	0	0
48.587	41.797	43.843	36.88	27.969	33.799	19.125
4.404	2.99	0.306	0.007	0.012	0	0
0	0	0	0	0	0	0
8.562	5.613	1.894	0.479	1.286	0.879	0.026
29.689	18.257	18.218	21.005	24.138	15.283	11.162
7.345	2.703	7.16	0.115	0.535	0	0
9.965	11.32	5.475	7.4	5.582	3.057	2.976
1.417	0.031	0.147	0	0	0	0
2.971	3.751	3.066	0.822	0.006	0.001	0
9.524	11.439	9.438	12.919	10.457	5.019	5.451
3.145	0.936	0	0	0	0	0
6.598	8.82	5.998	4.304	4.028	2.581	2.648
13.418	9.653	12.3	8.298	5.987	3.276	1.336
8.735	3.395	4.098	3.947	4.006	0.549	0.004
1.194	3.59	4.677	2.304	0.045	0.097	0
7.181	4.429	0.003	0.13	0	0	0
3.661	8.057	13.197	8.137	9.525	8.619	8.272
0.782	0	0	0	0	0	0
1.515	0	0	0	0	0	0
4.109	1.049	1.209	0.907	0.326	0	0

15.637	10.394	16.445	13.289	7.419	9.858	9.848
5.509	2.912	1.766	0	0	0	0
4.597	1.419	1.505	0.031	0	0	0
16.027	7.208	7.771	7.476	2.203	2.999	0.331
0	0	0	0	0	0	0
0.01	0.058	0	0	0	0	0
0.085	0	0	0	0	0	0
9.024	5.709	8.981	9.349	2.505	3.107	2.5
6.207	10.336	12.728	14.799	6.874	9.467	14.307
6.843	7.091	9.615	4.737	2.507	0.753	1.727
5.978	6.804	5.273	3.628	1.662	1.734	0.142
4.641	2.914	0.861	0.009	0	0	0
8.404	10.702	10.179	10.758	9.902	7.643	8.051
1.228	0.347	0.001	0	0	0	0
2.687	1.381	1.399	0	0	0	0
13.269	14.016	17.949	15.918	9.9	11.884	9.317
0.004	0	0	0	0	0	0
26.869	23.519	15.228	14.27	13.458	6.77	6.52
61.986	47.031	45.067	44.441	37.123	35.3	28.372
25.228	22.122	24.154	30.765	22.532	22.808	16.928
82.72	80.248	71.268	74.241	51.376	58.104	43.654
6.951	9.279	1.349	1.294	0.003	0.001	0
4.236	0.691	0.762	0	0	0	0
7.46	2.547	4.697	3.486	1.347	1.914	2.03
1.875	1.61	0.333	0	0	0	0
8.243	11.303	8.589	8.835	10.285	8.187	5.835
9.248	5.402	2.16	1.063	1.532	3.247	0.292
0.997	2.109	0.255	1.887	0.076	0.749	0.999

RDF130e	RDF135e	RDF140e	RDF145e	RDF150e	RDF155e	RDF010p
0	0	0	0	0	0	2.692
10.836	3.03	3.946	8.861	7.013	2.432	4.946
0	0	0	0	0	0	2.792
0	0	0	0	0	0	1.689
0	0	0	0	0	0	2.028
0	0	0	0	0	0	2.264
3.063	2.098	2.292	3.804	0.65	0.126	5.587
0	0	0	0	0	0	0.84
0	0	0	0	0	0	2.453
0.236	0	0	0	0	0	4.167
7.893	6.397	1.065	0.28	0	0	7.755
10.599	7.47	9.645	1.999	0.736	0	5.104
0	0	0	0	0	0	4.178
0	0	0	0	0	0	4.549
0	0	0	0	0	0	3.512
0	0	0	0	0	0	3.558
0	0	0	0	0	0	2.225
2.469	1.728	0.264	0	0	0	3.877
6.067	3.682	1.853	1.79	1.759	0.731	4.005
0.521	0	0	0	0	0	4.044
0	0	0	0	0	0	3.491
0	0	0	0	0	0	1.328
0.904	0.163	0.761	0.937	0	0	2.244
0.313	1.287	0	0	0	0	3.255
1.027	0.192	0	0	0	0	6.072
0	0.497	0	0	0	0	4.973
13.78	6.82	9.006	6.645	4.568	7.864	5.089
13.941	9.836	7.894	5.934	7.554	8.943	6.531
0.024	0	0	0	0	0	4.209
10.679	8.362	8.767	11.437	9.709	6.791	5.428
16.034	5.19	4.21	0.65	0.538	0	5.291
11.368	11.36	7.695	7.005	6.144	6.494	7.167
0	0	0	0	0	0	4.073
0	0	0	0	0	0	4.889
0	0	0	0	0	0	6.978
13.265	8.36	12.216	8.389	6.908	5.248	5.354
0	0	0	0	0	0	4.977
0	0	0	0	0	0	1.733
11.923	7.521	13.367	9.191	14.467	9.601	7.535
1.256	0	0	0	0	0	2.893
2.586	0	0.027	0	0	0	3.771
0	0	0	0	0	0	2.448
22.933	18.688	12.029	10.53	19.258	12.536	8.179
0	0	0	0	0	0	2.261
7.412	6.015	2.074	4.251	1.978	0.794	4.849
0	0	0	0	0	0	2.709
1.306	0.075	0	0	0	0	3.172
6.686	5.923	11.973	7.478	10.137	3.114	3.24
3.341	5.766	1.101	4.612	2.835	1.65	2.451

12.942	9.337	7.483	4.162	4.566	6.133	4.256
5.338	4.466	5.698	1.822	3.26	0.236	2.802
18.581	19.35	20.318	19.021	20.765	18.57	4.916
5.673	7.25	4.805	6.398	4.671	2.091	3.061
0.121	0	0	0	0	0	3.122
7.48	6.358	2.058	0.188	1.226	0.007	3.573
15.624	12.027	6.071	8.032	7.384	3.64	3.82
17.102	12.393	6.817	7.749	1.618	2.505	4.066
0.155	0	0	0	0	0	2.409
8.977	5.897	5.856	15.657	8.431	8.259	3.281
9.781	7.328	3.859	3.752	3.142	1.061	2.952
0	0	0	0	0	0	2.75
0.073	0	0	0	0	0	3.217
6.639	4.402	2.796	3.145	2.905	0.858	3.194
4.525	1.607	2.482	1.303	0.563	2.043	2.858
0.046	0	0	0	0	0	3.494
0.374	0	0	0	0	0	5.35
3.175	1.904	0	1.23	0.003	0	4.508
0	0	0	0	0	0	1.255
0	0	0	0	0	0	3.469
0	0	0	0	0	0	3.437
0	0	0	0	0	0	2.428
0	0	0	0	0	0	3.3
0	0	0	0	0	0	2.217
0	0	0	0	0	0	2.175
0	0	0	0	0	0	2.984
4.008	4.606	3.797	2.88	4.191	2.093	3.402
2.645	5.694	3.652	5.416	1.828	2.008	4.138
0	0	0	0	0	0	1.751
0.453	0	0	0	0	0	3.203
4.52	4.787	2.994	0.314	0.687	0.027	5.599
0	0	0	0	0	0	1.98
0	0	0	0	0	0	2.925
0	0	0	0	0	0	1.715
0	0	0	0	0	0	3.486
0	0	0	0	0	0	2.607
0	0	0	0	0	0	2.476
0	0	0	0	0	0	4.044
0.002	0	0	0	0	0	4.973
0	0	0	0	0	0	2.458
0	0	0	0	0	0	2.123
0	0	0	0	0	0	3.026
0	0	0	0	0	0	2.133
0	0	0	0	0	0	1.519
0.549	0.002	0.003	0	0	0	5.766
45.992	37.515	52.204	35.363	30.929	47.966	10.804
0.528	0.009	0	0	0	0	4.665
0	0	0	0	0	0	3.87
0	0	0	0	0	0	5.295
23.209	18.228	9.3	3.834	0.455	0.221	9.108

4.962	4.174	1.795	2.834	0.727	0	4.92
8.966	6.188	8.12	3.047	2.429	2.188	4.389
0.212	0.206	0.021	0	0	0	4.198
0	0	0	0	0	0	2.714
0	0	0	0	0	0	4.17
0	0	0	0	0	0	3.691
12.7	3.867	9.276	7.63	2.003	2.619	5.508
0.85	3.04	0.376	0	0	0	2.957
1.054	1.193	0.359	0.01	0	0	2.65
0	0	0	0	0	0	2.645
5.486	3.401	3.671	2.332	0.963	1.395	5.061
0	0	0	0	0	0	2.307
2.789	1.945	2.13	0.465	0	0	6.265
0.007	2.039	0	0	0	0	3.6
0	0	0	0	0	0	2.238
0	0	0	0	0	0	1.785
0	0.722	0	0	0	0	2.505
0	0	0	0	0	0	0.644
7.363	2.493	2.436	1.388	0.236	0	4.609
0	0	0	0	0	0	2.445
0	0	0	0	0	0	0.472
30.769	19.606	23.224	20.233	16.77	26.681	8.026
0.001	0	0	0	0	0	2.154
18.732	9.399	8.463	6.218	12.978	5.601	7.81
0	0	0	0	0	0	3.004
0	0	0	0	0	0	3.668
0	0	0	0	0	0	2.353
0.046	0	0	0	0	0	3.812
4.38	1.747	1.712	0.684	0	0	7.162
19.502	16.614	23.752	15.667	19.824	19.974	6.041
21.327	14.508	12.51	6.449	6.01	4.941	5.115
0.001	0.003	0	0	0	0	4.171
0.795	0	0	0	0	0	3.189
0	0	0	0	0	0	1.61
0	0	0	0	0	0	3.272
0	0	0	0	0	0	3.166
21.602	18.228	19.324	16.583	15.83	10.985	5.759
0	0	0	0	0	0	2.926
1.032	0.922	0.008	0	0	0	2.788
4.855	2.064	0.041	0.018	0	0	5.047
0	0	0	0	0	0	1.365
0	0	0	0	0	0	1.515
0	0	0	0	0	0	4.16
1.034	0.248	0.007	0	0	0	2.511
0	0	0	0	0	0	2.65
0	0	0	0	0	0	2.011
5.583	6.387	2.845	0.252	0.943	0.029	2.608
0	0	0	0	0	0	2.374
0	0	0	0	0	0	2.477
3.139	1.591	1.253	0	0	0	3.444

0	0	0	0	0	0	3.873
7.35	3.331	3.095	1.222	4.903	2.358	3.539
0.091	0.88	0	0	0	0	3.341
0	0	0	0	0	0	1.952
1.4	1.787	0.395	0	0	0	4.181
1.236	0.209	0.192	0	0	0	2.396
0	0	0	0	0	0	3.299
0	0	0	0	0	0	3.719
0	0	0	0	0	0	3.438
0	0	0	0	0	0	3.873
0	0	0	0	0	0	0.789
0	0	0	0	0	0	2.104
0	0	0	0	0	0	4.892
0	0	0	0	0	0	3.53
7.545	10.25	11.82	8.573	7.12	3.588	4.111
0.373	0	0	0	0	0	5.142
1.358	0.058	0	0	0	0	3.937
7.458	4.718	4.871	4.332	3.137	3.027	4.412
0	0	0	0	0	0	1.546
0	0	0	0	0	0	2.453
22.402	27.083	18.421	17.821	10.284	8.105	6.545
0	0	0	0	0	0	3.315
0.115	0	0	0	0	0	4.188
1.513	0	0	0	0	0	4.303
5.381	1.899	0.498	0	0	0	4.691
0.001	0	0	0	0	0	3.963
0	0	0	0	0	0	2.12
0	0	0	0	0	0	4.331
0	0	0	0	0	0	3.737
0	0	0	0	0	0	2.551
0	0	0	0	0	0	3.348
8.304	6.959	4.174	3.876	6.143	0.219	4.628
0	0	0	0	0	0	3.235
0	0	0	0	0	0	2.336
0	0	0	0	0	0	3.574
0	0	0	0	0	0	1.168
0	0	0	0	0	0	2.048
0	0	0	0	0	0	3.203
0	0	0	0	0	0	3.906
18.611	15.382	23.431	24.365	22.558	25.6	6.4
3.193	1.579	1.315	0	0	0	3.444
5.343	6.059	3.418	1.951	2.209	2.844	3.392
0	0	0	0	0	0	3.104
0	0	0	0	0	0	3.428
0	0	0	0	0	0	2.153
0	0	0	0	0	0	3.611
1.477	0.015	0	0	0	0	3.665
34.279	32.376	22.357	24.324	12.034	10.192	10.173
0	0	0	0	0	0	3.159
0	0	0	0	0	0	2.38

0	0	0	0	0	0	4.391
0	0	0	0	0	0	2.3
0.071	0	0	0	0	0	3.711
0	0	0	0	0	0	2.452
2.261	0.001	0.003	0	0	0	3.645
6.728	3.042	5.125	0.631	0.259	0.08	5.794
0	0	0	0	0	0	3.833
0	0	0	0	0	0	3.355
0	0	0	0	0	0	2.52
4.625	3.058	2.703	2.127	3.739	0.057	3.957
3.073	2.45	0.044	0.932	1.668	0.006	6.146
6.214	8.802	3.198	3.227	4.082	0.071	3.814
0	0	0	0	0	0	4.485
0.75	0	0	0	0	0	5.443
1.782	0.168	0	0	0	0	3.831
0	0	0	0	0	0	3.613
11.348	1.934	3.715	6.794	6.673	3.189	4.912
3.394	0.17	0.922	0	0	0	3.829
0	0	0	0	0	0	3.128
3.387	0.17	0.914	0	0	0	3.829
0	0	0	0	0	0	2.52
0.804	2.058	1.59	0.853	0.001	0.004	4.471
0	0	0	0	0	0	4.302
0	0	0	0	0	0	4.302
7.495	4.8	3.835	2.828	3.659	3.939	3.921
13.99	8.378	4.006	0.772	0.886	0	6.36
0	0	0	0	0	0	1.037
0	0	0	0	0	0	1.918
7.699	7.557	7.819	6.362	5.049	5.745	4.671
0	0	0	0	0	0	3.454
13.854	9.889	5.871	1.776	0.891	0.528	9.062
0	0	0	0	0	0	4.55
0	0	0	0	0	0	1.118
0	0	0	0	0	0	3.705
14.744	13.591	8.576	5.764	0.588	1.389	5.227
0	0	0	0	0	0	3.613
2.976	1.499	0.755	0	0	0	5.337
0	0	0	0	0	0	2.168
0	0	0	0	0	0	2.861
4.82	2.862	1.225	1.854	1.337	0	3.868
0	0	0	0	0	0	2.475
1.118	0.001	0.503	0	0	0	3.122
0	0	0	0	0	0	4.138
0	0	0	0	0	0	3.778
0	0	0	0	0	0	2.248
0	0	0	0	0	0	5.125
5.45	6.473	5.408	6.205	2.44	3.043	4.23
0	0	0	0	0	0	2.444
0	0	0	0	0	0	2.502
0	0	0	0	0	0	2.089

10.856	6.409	5.523	5.604	2.887	0.477	4.398
0	0	0	0	0	0	3.336
0	0	0	0	0	0	4.518
0.244	0.148	0.024	0	0	0	4.198
0	0	0	0	0	0	1.443
0	0	0	0	0	0	3.155
0	0	0	0	0	0	2.559
0.014	0.974	0.007	0	0	0	4.117
10.653	7.605	7.584	5.968	5.034	4.923	4.469
1.012	0	0	0	0	0	3.783
0.008	0	0	0	0	0	3.274
0	0	0	0	0	0	4.345
3.739	6.639	4.107	3.65	1.87	1.462	3.981
0	0	0	0	0	0	2.252
0	0	0	0	0	0	3.335
12.241	10.313	7.208	5.022	8.053	6.345	5.124
0	0	0	0	0	0	2.702
2.789	1.156	1.334	0	0	0	5.207
15.993	15.154	13.4	6.75	2.727	0.746	9.665
19.709	26.794	9.931	13.245	11.632	12.321	6.722
32.348	18.01	24.666	10.698	9.597	6.325	9.299
0	0	0	0	0	0	4.565
0	0	0	0	0	0	2.988
0.987	0.418	0	0	0	0	2.863
0	0	0	0	0	0	2.493
7.061	4.682	4.556	4.481	1.822	4.619	3.798
0.026	0	0	0	0	0	3.781
0	0	0	0	0	0	2.524

RDF015p	RDF020p	RDF025p	RDF030p	RDF035p	RDF040p	RDF045p
8.962	1.825	10.886	2.737	2.717	3.491	7.489
12.265	3.883	20.204	3.394	3.704	7.743	6.037
10.391	1.882	19.935	5.054	7.768	5.492	7.733
2.976	1.726	5.12	0.893	1.681	2.001	1.698
4.148	2.922	6.661	1.278	3.553	4.06	5.561
6.514	1.751	5.599	9.387	3.125	3.387	5.963
14.493	4.038	27.087	12.111	13.7	14.673	17.434
2.144	0.904	2.209	0.79	1.669	1.148	2.31
8.238	1.819	17.794	8.93	6.277	10.013	5.553
8.978	3.522	15.172	2.049	3.817	6.694	7.637
24.698	9.741	40.295	12.217	11.872	20.124	24.123
15.022	5.117	24.208	21.159	9.081	11.068	25.561
11.801	3.156	22.809	5.526	7.491	8.651	14.697
11.459	3.795	20.417	6.492	8.078	16.264	13.204
11.561	4.385	20.68	9.162	9.1	11.742	10.832
11.474	3.998	19.858	5.326	7.999	9.037	9.863
4.242	1.215	7.927	4.01	3.708	3.298	3.703
14.635	3.154	25.06	5.239	6.296	11.576	15.698
9.019	3.559	14.608	1.67	2.739	5.859	8.599
12.876	2.531	22.171	7.672	8.818	8.533	8.57
9.459	1.586	18.238	8.365	6.656	4.837	9.612
3.709	1.254	4.743	2.526	5.082	8.01	6.245
7.518	1.521	15.261	3.733	7.727	6.58	6.394
10.851	3.443	18.276	6.833	6.756	6.55	10.909
14.642	3.639	26.878	7.235	10.496	13.009	18.233
22.228	3.876	43.937	14.183	20.407	18.136	18.613
13.069	3.385	16.654	3.077	3.582	8.171	8.099
20.863	5.569	26.82	11.245	14.663	14.261	21.808
9.482	2.39	16.324	7.47	7.474	7.364	11.337
11.79	3.907	17.73	2.698	3.386	8.398	11.253
12.354	3.606	22.18	5.158	12.405	17.728	16.15
28.558	5.46	46.203	21.98	20.15	19.567	27.392
10.739	2.434	18.588	3.507	5.59	5.074	9.375
12.934	3.011	23.136	7.97	5.623	11.685	10.45
27.755	4.748	53.024	21.863	23.644	25.357	32.106
16.869	2.821	29.238	8.8	8.83	11.931	15.551
19.079	3.477	36.318	13.035	10.728	14.993	12.825
3.029	1.063	7.705	3.163	4.44	2.967	3.697
24.696	4.699	48.107	16.78	18.976	16.867	21.67
12.389	2.066	20.362	6.587	7.107	4.566	6.323
11.477	3.671	25.267	8.869	11.236	12.616	15.67
7.886	3.168	12.454	7.738	5.828	5.541	9.332
34.175	4.929	74.174	36.161	29.837	28.714	32.626
7.716	1.615	12.62	3.824	6.104	5.163	6.16
11.808	4.436	17.917	3.115	4.878	9.529	10.034
9.628	3.449	15.914	4.259	6.337	5.978	7.962
10.699	4.703	17.177	5.28	7.345	6.378	10.278
11.08	4.611	18.522	6.873	9.193	9.406	11.337
9.165	4.134	16.087	7.685	9.225	6.795	10.515

13.874	4.682	25.204	11.068	11.356	10.306	15.412
10.153	4.482	19.636	5.636	7.09	8.398	10.199
17.569	4.979	30.051	12.85	13.985	13.249	15.134
10.572	4.44	19.178	5.845	7.102	8.486	9.242
9.929	4.289	19.798	7.9	7.967	11.671	14.573
11.621	4.757	19.5	5.406	7.466	7.999	12.496
14.432	4.93	24.167	10.68	11.978	9.499	17.07
13.702	4.101	26.429	8.301	10.406	11.406	11.269
8.208	3.401	16.475	5.293	5.668	6.622	8.534
11.125	4.621	21.966	7.221	10.468	12.457	11.732
10.085	3.882	14.592	5.398	5.688	8.045	9.133
8.431	1.141	16.294	7.489	7.401	7.479	10.62
10.564	4.497	17.563	5.944	7.123	6.276	11.121
10.639	3.843	20.954	7.03	8.982	5.862	13.418
10.281	3.956	18.165	7.057	7.502	7.559	9.508
13.067	4.753	20.187	9.462	8.676	6.097	10.774
16.085	3.783	34.743	13.006	13.547	14.059	16.314
12.739	3.497	22.267	3.694	5.319	16.652	11.507
3.659	1.303	7.684	3.509	2.032	11.716	6.761
8.131	2.073	16.287	5.261	4.633	8.5	9.903
7.808	2.104	17.535	5.616	2.717	14.174	13.037
4.919	1.799	9.099	2.229	3.107	8.975	4.983
9.058	2.67	18.582	5.141	4.153	15.421	10.518
7.89	1.417	11.375	3.233	5.133	8.682	11.198
7.317	1.627	9.188	3.243	4.768	6.821	6.091
4.719	2.789	7.656	3.376	4.691	6.014	5.491
8.827	2.294	16.223	3.888	5.42	5.842	7.568
12.079	2.961	24.263	3.282	4.269	6.667	10.806
7.169	1.268	12.03	1.722	4.586	3.422	5.133
12.724	2.334	19.603	3.197	5.244	6.651	6.052
16.362	3.886	28.932	14.989	6.933	11.925	18.287
4.77	1.615	8.338	1.932	4.129	7.222	3.662
9.539	2.298	15.253	3.968	7.904	9.89	9.205
4.068	1.42	4.212	4.175	4.004	6.015	7.896
9.748	2.911	21.125	4.555	9.888	8.921	19.867
6.89	1.857	10.429	7.282	3.673	7.841	4.631
4.405	1.952	9.03	1.398	2.981	9.881	5.571
10.031	3.03	19.818	6.193	7.133	7.528	10.581
22.197	3.664	44.059	15.291	22.229	17.041	19.115
7.611	1.601	16.48	6.662	4.473	8.173	5.936
5.659	2.183	10.627	6.198	8.728	9.903	13.168
13.135	3.55	19.463	9.747	10.611	13.339	15.126
6.506	2.271	8.265	3.202	5.32	5.12	6.311
5.618	1.333	12.904	2.193	2.432	2.288	2.671
23.734	3.661	49.313	23.427	22.72	17.908	23.967
41.381	7.983	75.896	31.769	30.394	29.743	39.75
11.244	2.975	20.772	8.13	7.771	19.797	16.115
8.139	2.437	15.938	2.382	5.878	4.573	12.011
12.967	3.904	28.193	14.684	14.529	16.759	14.549
34.922	8.192	63.865	29.497	31.764	36.43	47.313

10.297	3.285	19.06	7.337	5.275	11.92	13.164
12.618	3.095	22.887	7.21	10.642	11.389	17.005
17.59	4.789	38.842	11.763	14.156	12.588	14.97
8.23	2.125	18.497	4.242	5.403	6.488	6.243
9.218	3.85	14.341	4.004	3.636	6.273	7.121
12.102	2.496	19.64	8.284	8.054	6.646	12.586
20.999	4.996	32.978	8.859	10.106	22.102	20.874
4.482	2.68	14.095	4.21	4.598	8.338	8.523
8.249	1.777	17.19	2.085	2.092	2.746	2.005
6.195	1.643	11.559	2.892	6.633	3.531	6.276
13.07	2.88	23.928	7.837	9.405	11.007	15.463
7.416	1.987	17.346	6.274	3.455	8.942	7.026
22.175	3.465	46.557	15.163	15.826	16.423	17.496
12.214	3.249	19.179	2.281	3.075	9.181	3.861
6.373	2.035	12.974	3.947	11.182	5.91	8.795
8.285	1.025	12.632	4.449	7.682	2.034	3.503
7.725	1.568	15.397	7.64	7.213	5.496	7.596
0.941	1.626	1.413	0.009	1.762	0.839	0.285
13.831	3.428	26.469	6.672	3.741	16.1	14.933
7.222	1.529	15.454	4.544	5.092	3.844	4.336
1.497	0.476	0.648	0.67	0.41	0.344	0.013
28.005	4.385	51.525	18.138	10.232	14.523	20.206
6.129	1.458	11.832	1.838	1.972	9.43	5.839
30.029	5.751	58.404	28.109	27.832	29.383	35.363
8.922	2.596	18.289	6.643	7.649	5.811	2.75
12.895	4.249	22.294	6.659	11.592	9.486	12.24
5.336	3.463	8.495	1.425	4.266	3.639	6.119
9.488	2.065	19.209	4.823	3.902	7.405	5.03
21.023	7.337	35.562	11.164	10.413	18.807	20.419
17.512	4.069	30.457	11.098	10.911	14.015	14.554
14.095	3.883	28.287	6.074	7.183	15.209	12.422
13.674	3.26	23.922	8.925	13.996	7.78	10.973
9.673	2.286	15.849	2.14	4.561	8.274	10.112
4.687	1.071	7.367	4.474	1.896	12.075	7.467
15.322	2.438	26.616	12.372	8.565	11.741	11.397
8.17	1.618	16.321	5.238	5.772	4.834	7.724
15.109	4.85	31.787	4.041	6.008	13.035	11.496
9.146	2.135	14.511	4.373	11.043	10.603	10.45
8.947	1.902	14.421	7.725	5.711	4.211	5.028
16.784	2.672	32.324	10.397	8.908	10.021	12.488
6.027	0.87	6.642	2.764	3.676	1.911	2.854
5.946	1.129	6.272	2.621	3.234	1.81	1.832
11.319	3.065	21.191	6.016	9.419	9.804	13.67
8.333	1.885	16.738	5.124	4.101	6.598	5.324
8.19	1.722	16.586	4.621	5.747	5.574	4.408
4.013	2.119	7.459	3.386	3.465	4.668	3.672
7.187	1.899	11.903	3.192	8.037	6.07	7.473
7.409	1.433	17.079	3.577	9.843	5.732	7.485
6.672	1.525	7.749	7.428	3.212	4.294	5.563
12.845	2.46	22.819	4.924	5.941	7.073	8.93

8.382	2.612	16.324	6.872	4.028	7.83	7.957
10.095	2.607	15.121	6.121	4.527	5.531	6.749
11.861	2.611	21.279	3.631	4.561	6.403	8.324
7.345	2.14	13.935	7.478	5.851	7.871	10.85
12.328	3.981	25.05	7.43	8.113	10.797	11.279
6.376	2.038	15.65	7.453	4.953	10.405	6.434
8.248	2.446	16.42	5.395	3.479	6.493	11.725
10.135	2.344	19.369	6.546	7.192	8.289	8.946
10.407	2.642	19.593	5.955	4.816	8.054	10.42
10.195	2.534	19.041	5.991	4.728	7.757	10.215
2.855	0.942	2.943	1.118	1.629	3.074	1.491
1.582	2.299	4.597	1.374	1.667	1.891	2.457
12.332	2.617	25.404	10.3	13.059	15.71	16.942
10.761	4.082	18.873	6.609	7.996	10.095	10.031
11.76	4.213	23.986	4.511	7.666	8.984	10.703
21.716	3.707	41.42	15.638	17.353	17.77	19.778
8.371	3.007	17.176	4.595	2.702	10.81	6.478
9.413	3.394	15.118	2.576	2.375	7.072	8.373
3.676	1.479	5.137	1.804	2.743	2.306	2.637
8.453	1.54	16.865	10.223	7.937	7.281	10.888
20.767	3.95	37.144	12.024	15.967	18.216	22.485
14.577	3.687	24.881	11.693	8.136	13.293	12.619
16.641	2.918	25.977	8.312	10.479	8.324	13.139
9.473	2.161	16.665	5.658	5.906	7.944	9.792
13.204	3.956	24.439	4.891	5.178	8.049	5.473
13.496	4.055	24.65	8.021	8.825	9.317	11.859
7.041	1.294	13.265	2.077	2.748	3.882	4.819
19.003	3.543	29.936	13.804	13.197	15.547	14.846
16.246	3.313	27.77	13.284	12.243	13.895	12.358
6.695	1.911	14.195	2.813	5.213	5.844	4.476
4.751	2.051	11.564	4.413	4.483	3.107	6.085
15.862	3.487	30.202	11.736	8.458	10.452	16.27
9.085	2.555	19.15	2.914	6.32	7.248	12.281
5.031	1.381	10.05	6.068	5.66	5.037	6.088
10.001	2.626	19.497	4.498	8.272	8.606	10.527
4.175	0.986	4.315	2.563	4.134	1.121	1.615
4.498	2.469	8.054	1.585	2.17	2.175	4.34
10.897	2.655	19.431	3.802	4.66	5.916	7.937
11.239	3.415	20.928	4.926	6.491	8.463	13.406
19.209	5.771	37.823	8.821	15.41	17.741	16.499
12.808	2.481	22.688	4.901	5.951	7.072	9.01
7.318	2.338	14.2	5.589	10.096	7.292	10.383
9.764	1.891	17.555	6.325	6.909	5.406	7.405
13.1	3.751	19.82	7.648	9.921	8.168	12.503
7.344	2.242	15.208	6.525	4.22	7.992	5.434
15.848	2.806	27.54	11.948	8.815	14.107	12.961
9.572	1.97	17.099	12.607	7.221	5.689	13.232
34.544	5.575	73.375	26.648	27.975	35.662	41.049
8.564	2.573	18.314	7.858	11.87	5.396	5.53
4.165	1.92	6.366	0.485	1.826	4.473	2.458

13.397	2.731	25.946	8.964	10.238	14.653	10.619
6.48	2.63	13.867	5.013	11.078	5.374	3.764
9.456	1.758	16.881	11.998	7.514	5.384	12.91
6.692	2.32	13.556	3.725	5.143	5.001	6.559
8.461	2.906	11.847	2.196	2.985	4.715	6.678
15.515	3.895	30.114	9.531	8.575	20.251	19.991
10.772	3.059	19.233	8.85	8.012	9.005	10.494
9.312	1.564	14.342	4.815	9.115	11.507	11.262
7.994	1.531	10.602	5.97	7	7.092	7.385
8.31	3.426	12.916	1.889	2.89	6.177	5.198
21.18	5.187	37.439	13.287	11.636	16.398	15.539
9.436	2.942	17.727	2.251	8.734	12.792	8.431
21.277	2.947	34.615	13.57	17.33	14.066	15.081
21.354	2.921	39.277	17.15	16.315	16.004	18.698
7.952	2.838	16.062	3.605	2.838	6.746	4.775
7.701	2.361	14.573	3.378	3.208	6.344	5.219
12.476	3.649	20.891	3.944	2.593	8.152	7.109
8.799	3.117	16.047	1.41	3.019	6.003	5.202
7.261	1.386	14.832	5.21	7.934	6.564	6.773
8.802	3.116	16.047	1.407	3.021	6.009	5.202
5.725	1.951	12.542	3.14	5.931	8.371	7.829
11.861	3.847	21.196	7.246	7.84	16.959	17.344
13.557	3.806	28.875	6.508	14.001	10.016	10.745
13.571	3.938	29.348	7.335	14.753	9.688	11.749
7.483	2.75	11.952	5.044	5.483	7.177	7.488
17.795	4.15	33.132	9.07	10.227	13.038	22.311
2.774	1.018	7.067	0.981	2.045	4.334	3.009
6.199	1.555	6.945	12.263	3.82	6.241	6.964
17.162	2.746	28.599	7.591	8.115	9.513	11.71
8.39	2.425	13.229	3.298	5.77	5.632	10.844
31.38	6.616	63.045	26.096	29.02	31.336	37.508
12.021	2.809	21.811	6.332	5.039	11.313	9.957
2.78	1.151	5.879	0.42	1.194	0.595	1.103
13.273	2.348	22.541	8.188	10.642	10.382	9.273
14.633	3.498	23.456	5.667	9.405	16.5	13.698
7.834	2.711	14.209	3.831	3.511	5.692	8.394
13.553	3.961	31.045	10.409	13.36	15.797	17.557
4.579	1.962	7.545	3.358	4.355	7.987	5.286
5.448	1.944	11.373	5.391	5.996	7.361	11.378
9.522	2.06	17.111	12.761	9.734	8.928	14.932
5.502	2.172	8.04	3.203	5.456	8.457	5.987
10.956	2.113	16.879	7.821	7.74	8.628	12.182
10.486	2.268	19.097	4.558	6.456	10.771	9.341
9.01	2.319	12.669	4.974	9.533	4	8.915
6.921	1.311	17.073	4.774	5.148	5.104	5.729
10.861	3.58	19.484	4.876	7.286	18.754	21.964
9.694	3.733	16.576	3.02	4.99	5.25	6.391
7.89	1.902	16.797	7.177	4.536	8.373	6.882
5.788	2.03	9.338	2.972	5.32	10.418	8.936
5.28	1.855	13.586	5.831	5.386	7.193	4.509

11.892	3.256	19.586	5.245	9.143	13.242	10.539
7.799	2.734	15.769	3.031	3.732	4.609	5.941
20.023	3.304	33.372	15.892	15.94	15.554	15.125
17.703	4.69	38.84	10.46	12.361	12.316	15.268
2.468	1.117	5.617	1.931	4.095	2.424	3.187
8.584	2.675	17.958	7.783	9.837	6.836	7.216
7.499	1.576	16.755	5.506	3.371	8.749	9.226
10.405	3.071	18.861	3.116	3.971	7.156	5.641
10.117	4.237	17.908	2.366	2.58	9.104	5.334
8.896	3.206	15.499	6.869	8.143	10.769	6.808
6.8	2.137	12.932	3.376	3.178	7.603	4.58
11.266	2.752	24.18	6.774	6.029	10.647	9.378
10.619	3.016	18.805	3.211	6.699	11.677	8.439
8.262	1.719	16.561	10.818	8.42	9.61	12.079
5.71	3.196	11.978	1.324	5.186	7.117	9.985
11.347	3.709	21.293	3.966	5.286	13.927	10.445
6.517	1.535	10.976	4.434	1.562	2.891	2.225
15.337	4.111	32.325	10.179	11.547	14.73	14.994
34.048	6.067	70.004	24.815	31.251	32.038	38.754
15.64	5.445	31.414	12.329	11.554	18.933	14.531
36.086	8.876	64.909	29.603	43.335	48.893	58.769
16.778	2.734	30.508	11.67	10.429	10.549	20.353
9.79	2.095	18.873	4.818	6.391	6.449	9.237
7.938	1.317	15.774	4.419	9.419	4.88	10.091
7.15	2.423	10.531	4.065	5.754	3.413	4.252
13.295	2.759	22.387	8.008	5.545	11.745	13.83
9.392	2.173	19.821	4.136	7.687	6.838	10.549
8.027	1.586	13.018	3.818	6.928	7.931	7.779

RDF050p	RDF055p	RDF060p	RDF065p	RDF070p	RDF075p	RDF080p
2.951	3.461	4.142	2.144	1.976	1.971	3.961
15.749	3.442	7.887	9.377	4.711	3.343	4.091
17.67	5.143	9.015	10.138	7.243	7.774	5.103
1.909	0.35	1.302	1.269	0.789	0.003	0.112
3.298	2.503	2.708	0.719	2.91	1.382	0.166
3.622	1.901	0.755	2.58	0.314	0.38	0.052
19.873	16.115	12.309	14.084	13.183	8.887	10.926
0.253	0.907	0.123	0.102	0	0	0
11.455	4.487	7.748	5.337	6.505	2.339	4.644
10.726	3.753	7.628	5.798	2.4	3.337	2.484
27.419	19.432	29.828	35.092	31.302	24.302	22.403
14.157	21.461	33.631	24.11	14.44	18.135	14.179
13.983	11.397	9.579	8.576	5.471	5.717	3.618
16.669	9.671	18.114	14.926	12.843	4.408	7.949
12.953	9.084	10.54	9.87	7.647	5.218	3.19
10.956	7.603	5.845	5.246	5.443	6.749	7.504
3.85	1.863	2.379	1.37	0.955	1.145	0.607
21.188	16.706	18.893	17.094	15.407	8.746	9.941
8.78	2.768	3.132	5.126	3.318	1.894	2.731
8.627	5.194	8.137	7.113	6.303	5.052	5.502
13.013	11.681	10.276	8.372	6.673	6.817	4.096
7.16	3.213	4.543	1.251	7.182	0.309	3.09
7.899	3.518	9.508	1.707	2.233	1.804	5.416
9.637	7.489	3.876	4.494	5.498	4.502	4.662
15.27	18.091	14.837	21.488	15.318	20.335	13.072
20.071	12.144	13.351	12.802	7.984	9.487	4.977
11.523	5.247	3.885	5.22	5.571	3.91	3.759
22.296	26.145	23.302	27.12	18.208	28.114	27.081
7.614	8.92	11.014	9.624	9.756	8.683	7.899
9.532	6.611	6.459	10.5	5.85	5.458	5.333
27.448	19.243	26.107	20.908	21.976	22.02	16.999
40.792	25.165	29.653	33.455	33.826	28.222	21.318
8.114	6.376	6.42	4.35	6.448	3.757	4.801
11.098	8.549	10.57	13.285	11.664	6.985	8.163
29.662	23.776	21.465	26.522	18.191	17.566	17.474
10.157	7.467	8.582	11.887	7.381	6.899	8.183
24.582	9.12	10.971	10.713	5.926	7.622	5.469
1.882	3.736	2.382	2.078	0.763	0.292	0
25.493	11.443	13.887	17.977	19.119	17.003	14.812
9.808	6.11	8.835	8.578	7.665	6.592	5.693
16.039	14.049	15.87	16.089	13.7	7.029	12.341
6.026	6.969	5.404	2.632	3.044	0.241	1.214
42.376	20.084	21.799	32.923	22.011	18.58	15.105
7.46	3.227	9.57	3.751	2.902	5.334	2.166
13.037	10.662	17.094	12.964	13.061	11.444	9.131
9.748	6.545	6.641	6.509	4.614	3.826	5.319
12.236	7.321	5.448	6.54	4.94	6.273	8.701
12.773	8.199	9.248	10.674	9.985	7.162	8.645
11.551	3.618	8.222	8.742	6.621	2.164	8.645

16.925	8.637	8.37	10.804	10.225	7.84	7.993
13.313	7.593	7.433	7.886	6.339	5.499	6.249
21.723	14.383	18.823	20.68	15.078	11.312	15.489
14.283	6.671	7.379	11.761	5.531	7.705	7.82
14.434	11.403	14.277	15.943	9.85	9.074	7.599
12.837	5.654	8.678	11.243	5.537	4.618	5.704
17.074	8.163	12.722	13.005	12.206	7.914	6.249
21.281	12.468	13.034	16.005	13.84	9.278	11.475
11.538	5.225	7.793	8.602	7.183	4.976	4.98
16.118	11.108	9.292	10.416	8.961	8.08	9.79
12.283	4.924	6.713	8.837	5.573	5.538	7.242
8.559	6.25	6.778	7.542	6.533	7.639	8.628
12.934	3.961	10.752	9.27	4.769	5.723	9.169
12.093	7.23	6.797	9.396	12.045	7.148	5.964
11.332	2.949	6.119	9.341	7.149	6.061	7.759
14.409	7.405	4.725	11.73	6.065	6.088	5.513
17.911	11.494	15.716	11.909	13.139	8.857	8.198
15.798	9.941	14.787	11.684	8.235	11.944	6.618
3.486	3.239	1.356	2.163	1.015	0.943	0.112
14.221	7.38	11.301	11.269	6.556	2.867	5.146
9.93	13.002	10.549	8.553	8.412	9.769	3.6
6.137	3.393	1.197	4.634	5.108	5.242	1.988
13.025	4.727	9.408	9.263	10.343	8.926	5.637
10.089	4.283	5.248	4.911	5.129	3.466	5.11
5.07	2.622	5.033	4.125	2.913	1.385	1.108
4.902	3.084	1.311	2.723	2.118	2.993	1.354
9.767	5.636	5.213	7.167	6.936	5.158	4.133
15.984	6.352	10.346	7.094	6.709	4.889	4.39
8.52	4.996	4.007	3.937	2.226	1.39	0.443
12.819	5.013	8.593	11.656	6.64	3.454	3.505
18.028	11.058	12.285	14.8	12.381	9.33	9.161
5.222	1.751	3.755	2.39	1.276	0.579	0.476
12.377	8.95	10.373	7.454	8.3	9.338	6.759
5.416	3.624	1.568	2.446	0.725	0.847	0.165
6.97	11.381	7.628	10.256	8.521	7.231	9.522
4.656	3.723	5.389	1.129	2.069	0.713	0.18
3.797	1.25	2.404	5.344	1.701	3.576	2.131
14.178	11.456	8.556	7.074	5.229	5.729	4.143
19.593	11.999	13.633	12.316	7.873	9.299	4.768
13.54	3.177	6.185	7.01	4.986	2.463	2.343
6.184	5.616	3.388	2.981	3.054	0.419	1.664
16.698	13.383	13.999	17.373	15.513	10.835	8.9
4.125	4.787	3.72	2.225	1.146	1.891	1.344
5.158	0.56	3.76	5.675	2.311	1.27	0.278
19.553	13.501	13.731	15.109	9.773	9.952	5.788
37.219	29.719	34.112	35.067	27.728	24.883	24.208
19.922	14.949	18.905	17.599	20.237	14.5	13.749
8.029	10.121	8.627	10.087	8.107	5.071	5.124
20.659	12.487	13.968	13.533	10.94	12.851	4.719
51.849	44.307	50.042	54.733	50.007	47.868	45.831

11.986	9.892	7.811	9.463	8.888	12.229	8.76
13.037	6.977	7.367	9.309	11.38	11.177	12.237
21.925	11.003	14.907	14.581	10.06	8.603	5.387
11.709	2.777	4.523	4.914	2.672	5.421	2.198
5.386	2.384	3.612	3.711	1.953	1.979	2.717
8.26	10.764	11.908	7.018	5.267	7.801	2.568
37.417	15.271	36.298	22.73	27.725	18.567	18.694
7.629	4.333	5.073	3.749	4.124	5.752	2.056
11.2	0.427	1.934	8.099	4.516	4.18	2.283
6.626	8.797	8.89	5.911	3.721	3.529	2.1
14.144	10.905	10.64	12.036	7.224	5.753	7.31
7.623	4.251	9.647	5.475	4.797	6.276	5.096
22.457	10.325	14.083	13.928	13.189	9.246	7.448
15.947	2.49	6.185	11.104	4.892	5.713	3.5
12.49	4.48	5.8	4.789	3.466	2.535	1.142
6.104	2.615	5.393	1.494	4.947	0.688	4.16
7.257	3.693	4.757	5.719	4.991	3.869	2.752
0.073	0.002	0	0	0	0	0
17.055	12.309	12.629	14.243	11.371	13.851	10.629
6.923	0.959	3.029	6.648	4.287	1.922	1.703
0.017	0.019	0	0	0	0	0
34.49	13.758	12.868	29.639	15.852	21.795	15.65
7.617	2.263	4.412	7.508	3.012	3.013	2.19
34.46	21.862	28.842	30.925	30.989	18.05	18.411
2.701	0.928	0.797	0.307	0.137	0	0
14.297	6.38	10.568	7.694	7.7	2.872	3.001
4.252	4.117	4.835	3.015	1.709	2.816	1.324
10.605	1.457	4.074	9.268	5.189	7.066	3.173
20.076	9.917	16.774	21.356	14.151	10.651	14.946
13.969	10.764	8.158	14.51	12.048	8.304	12.554
15.683	6.518	5.952	15.939	13.963	10.735	9.258
7.96	4.786	5.249	4.334	8.005	6.739	6.337
13.681	7.842	13.997	10.197	11.375	8.108	4.689
3.648	3.153	1.836	2.738	1.608	2.355	0.469
16.197	7.211	7.397	4.646	2.368	0.648	0.39
6.657	2.831	3.391	5.986	1.884	2.166	1.962
20.641	7.562	14.385	18.365	11.279	5.37	7.621
12.246	9.051	8.383	7.719	11.23	8.186	3.897
8.536	3.471	5.006	4.501	6.442	2.879	3.47
19.146	10.906	17.063	23.2	17.489	11.757	15.992
1.198	1.918	1.381	0.915	1.023	0.283	0.352
0.617	1.371	0.727	0.452	0.323	0.013	0.167
18.025	11.358	16.631	12.954	6.68	4.636	4.541
8.813	1.791	2.779	9.758	4.434	2.399	2.334
11.508	3.995	6.379	5.632	4.895	5.032	3.238
1.624	1.83	6.67	2.611	1.737	0.737	1.281
7.406	3.731	6.616	1.754	4.648	2.729	5.664
7.463	7.231	5.828	4.282	5.537	3.873	2.365
1.431	2.739	1.069	0.684	0.217	0.119	0
16.008	8.505	13.655	9.315	9.639	4.684	4.853

8.301	6.463	6.547	6.795	6.136	4.558	3.878
8.086	3.233	4.837	6.633	7.814	1.364	2.538
12.355	5.833	10.233	8.39	9.524	3.31	2.725
8.956	4.585	8.283	6.864	6.503	2.34	2.717
14.321	9.965	16.639	13.345	14.989	8.649	11.513
8.927	2.744	3.373	8.411	2.946	2.269	3.646
9.013	6.046	4.532	6.337	6.242	2.689	3.652
12.849	6.639	7.251	7.87	5.671	3.097	3.258
9.982	5.751	7.74	6.752	7.306	2.403	3.547
7.647	5.551	6.602	7.313	7.801	2.859	2.565
0.825	0.549	0	0	0	0	0
0.553	1.326	0.393	0.505	0.047	0.088	0
26.021	12.52	14.946	11.249	9.658	3.654	2.048
10.744	7.615	9.927	8.673	9.297	15.059	10.174
15.338	12.449	7.852	11.067	6.756	8.35	6.384
23.12	11.119	13.643	13.97	7.584	8.721	6.154
9.209	1.162	3.568	9.01	4.812	2.337	2.714
10.055	4.96	3.269	4.62	3.66	2.54	1.537
1.223	1.175	0.568	0.584	0	0.001	0
9.186	6.828	10.261	6.338	6.252	2.708	5.334
30.731	17.295	26.752	27.474	20.265	23.788	17.09
11.65	7.144	7.182	3.385	2.492	0.881	0.247
19.284	10.221	10.237	11.08	12.093	6.448	9.032
12.602	7.957	11.175	10.545	10.244	5.759	4.165
13.543	2.163	4.887	7.183	2.761	7.207	3.085
16.367	10.013	11.804	13.992	14.803	11.575	15.479
9.513	4.78	5.509	4.168	1.472	0.794	0.872
23.227	12.899	11.083	10.631	9.417	3.444	4.828
20.017	9.657	8.17	7.233	6.857	2.505	2.259
8.276	1.663	4.738	1.9	3.765	0.366	1.584
4.009	5.94	3.516	2.841	3.91	4.127	2.896
24.219	9.951	10.392	16.842	8.015	12.537	5.447
16.119	9.926	14.035	7.931	5.882	2.502	2.363
9.629	3.006	5.021	6.019	4.452	3.375	2.981
16.375	7.653	20.395	8.13	9.903	3.234	3.782
2.002	1.135	0.98	2.95	1.553	1.056	1.872
3.289	0.343	1.121	1.591	0.623	0.322	0.281
13.889	7.608	8.984	9.313	4.637	1.963	2.188
11.914	9.675	7.839	7.67	5.603	8.298	3.895
27.837	11.464	18.749	17.37	16.607	10.946	10.006
15.145	8.438	13.356	9.312	9.297	4.936	4.71
7.455	5.244	7.67	3.674	5.155	3.719	4.892
6.652	8.996	7.841	6.095	4.84	3.489	2.882
13.811	13.762	10.196	11.531	9.268	16.737	6.456
11.518	3.167	6.144	5.293	4.878	2.056	2.095
18.151	7.232	9.297	6.649	6.415	2.082	2.249
16.373	8.014	10.273	11.102	9.177	4.716	5.312
47.069	28.771	34.002	44.013	19.119	31.806	25.681
10.24	3.927	2.508	3.838	0.476	2.259	0
3.798	0.899	3.132	1.816	2.683	0.027	0.748

16.607	7.454	10.282	4.548	5.022	1.452	1.143
9.472	2.873	3.759	2.701	1.068	1.005	0.221
16.734	7.723	9.9	9.275	7.888	5.108	5.992
10.394	5.803	4.472	2.176	2.705	1.166	0.628
9.11	2.883	3.513	4.028	3.783	3.223	2.17
21.716	16.923	16.56	20.212	17.846	22.236	14.425
10.685	5.677	9.527	9.921	9.345	8.342	6.806
12.504	16.259	11.794	13.728	7.937	5.334	3.49
5.719	5.24	2.777	3.011	2.549	1.351	1.989
9.323	2.657	4.063	4.424	3.678	2.044	1.722
26.045	12.679	15.217	21.991	11.67	24.362	7.871
13.762	5.223	14.079	5.955	5.569	3.957	7.006
16.008	8.679	12.499	9.259	7.878	3.981	4.108
17.877	9.43	10.299	15.003	5.146	6.797	7.215
7.602	1.141	1.932	5.892	2.351	1.448	2.177
7.438	3.624	4.426	2.855	5.18	5.174	4.121
18.375	1.459	5.836	11.17	10.795	9.466	4.471
11.096	3.164	5.489	5.381	1.637	3.6	2.534
9.88	1.189	8.306	1.468	4.211	1.1	1.82
11.089	3.171	5.497	5.378	1.639	3.589	2.537
7.799	4.417	3.789	5.335	1.628	0.351	0.437
14.331	10.634	10.503	10.818	10.374	10.321	10.173
16.248	7.241	12.485	9.988	14.374	5.025	8.265
14.463	8.754	10.577	8.155	9.097	7.399	7.551
5.613	4.058	3.7	4.347	2.292	5.794	3.065
26.69	19.68	23.305	19.154	21.943	15.471	12.265
2.665	0.927	1.535	1.78	0.584	0.674	0.336
7.058	2.493	3.091	0.606	0.193	0.046	0
15.822	8.638	8.7	10.674	7.419	7.887	8.105
6.776	3.966	7.425	6.617	8.084	6.404	6.661
38.785	32.487	30.32	31.13	18.66	18.47	24.288
11.497	6.381	9.071	9.311	12.899	3.915	6.038
1.277	0.04	0.598	0.047	0.107	0	0
8.857	7.823	7.321	5.647	5.653	5.747	4.255
22.94	13.084	18.232	12.189	16.795	13.602	11.121
8.023	5.621	6.104	4.165	5.396	3.876	3.285
19.201	10.257	14.41	13.906	17.224	8.974	9.621
4.327	2.842	3.338	4.775	3.274	1.858	2.688
5.038	3.802	6.749	6.551	3.336	6.815	2.329
16.699	7.714	13.535	9.13	10.791	10.413	3.631
4.128	4.388	6.09	5.195	5.608	2.931	2.725
12.609	9.022	6.817	11.483	7.137	5.81	4.758
10.808	2.496	6.375	9.559	8.163	4.486	4.914
4.918	4.95	6.837	8.432	6.429	5.858	5.282
8.51	1.604	3.785	6.047	4.214	3.859	1.824
24.246	14.552	23.682	17.806	13.167	9.559	8.887
6.561	3.776	8.268	6.585	5.98	3.531	4.264
14.022	3.64	6.919	7.245	5.441	2.676	2.549
11.281	5.922	5.93	2.775	3.184	1.899	0.181
8.481	3.919	7.623	4.732	3.484	3.279	3.7

13.534	5.725	15.578	8.201	7.046	6.183	5.908
6.564	4.547	4.265	3.326	4.322	2.419	1.685
12.225	8.459	6.913	9.194	7.167	5.763	3.188
24.203	10.248	16.84	14.097	11.71	9.313	5.928
2.03	2.803	1.78	0.842	0.388	0.276	0
9.252	4.886	3.562	3.743	1.616	1.287	0.27
8.183	3.879	5.788	5.841	5.407	2.361	3.82
9.739	3.45	5.253	6.859	5.615	7.273	2.446
13.091	2.622	6.944	6.638	4.283	4.157	2.522
4.861	8.025	5.891	7.48	5.882	4.839	7.209
7.493	3.583	2.768	6.993	3.919	5.22	2.569
14.986	5.613	9.745	7.94	6.898	3.482	2.85
11.59	5.368	7.978	8.253	7.178	5.962	7.318
10.3	6.899	9.585	5.109	6.557	2.736	4.141
11.381	12.978	5.766	6.349	4.878	3.496	3.319
14.687	4.636	9.762	15.237	8.507	6.944	4.697
6.803	0.285	1.034	2.842	0.195	2.056	0
17.414	12.228	13.921	16.473	15.786	12.385	9.812
41.264	31.978	36.562	36.78	22.441	27.149	27.018
29.712	11.281	17.532	17.496	10.837	10.562	9.821
53.304	46.007	56.812	59.257	47.743	40.857	39.729
15.203	15.653	16.111	14.221	11.918	9.753	6.543
15.09	6.897	6.618	8.284	10.135	7.975	3.615
7.136	3.581	8.333	7.885	7.773	5.665	4.917
3.505	3.803	5.479	2.142	2.495	1.667	3.087
8.933	8.333	8.626	9.474	6.58	5.27	6.824
8.862	8.078	10.93	9.111	9.08	8.187	8.806
8.043	4.54	6.59	1.744	3.277	3.102	4.132

RDF085p	RDF090p	RDF095p	RDF100p	RDF105p	RDF110p	RDF115p
1.029	1.187	0.541	0.553	0.016	0.302	0.017
10.816	5.027	3.908	3.999	6.458	5.844	2.337
2.635	3.953	1.508	2.158	0.196	0.417	0.105
0.119	0	0	0	0	0	0
1.079	1.28	0.755	0.289	0.386	0.014	0.031
0.218	0	0	0	0	0	0
7.107	7.611	8.646	5.932	6.194	7.464	2.818
0	0	0	0	0	0	0
1.002	0.502	0.101	0.018	0	0	0
5.419	2.956	3.854	3.295	2.822	2.411	1.111
18.212	12.017	9.966	9.705	5.868	5.882	3.171
8.282	14.037	9.724	10.062	9.68	12.46	8.689
3.488	3.012	2.209	1.008	0.846	0.119	0.137
7.275	8.031	2.628	4.521	2.853	2.467	1.087
2.755	1.664	0.622	0.099	0.026	0	0
4.036	5.283	5.246	4.284	4.98	2.497	0.731
0.521	0.12	0.124	0	0	0	0
11.965	10.031	5.111	5.21	3.673	2.337	2.259
6.878	2.067	3.94	2.048	2.765	2.719	0.626
7.563	3.786	4.402	2.809	2.237	1.489	0.519
2.951	3.748	2.236	2.272	1	0.492	0.145
0	0.874	0	0.126	0	0	0
1.255	2.245	1.605	2.245	5.757	1.852	0.953
5.239	5.737	3.882	2.083	2.126	4.569	2.792
12.278	12.883	7.154	7.615	3.277	4.622	0.855
5.088	3.12	4.449	1.951	2.473	1.326	0.52
7.762	4.361	6.553	4.89	3.764	4.48	2.88
27.514	21.94	17.677	16.002	14.539	15.576	4.81
4.526	5.917	4.711	4.159	0.961	1.634	1.023
9.001	5.303	5.292	3.793	6.279	5.494	5.056
18.533	14.994	12.743	10.627	11.176	9.706	4.498
22.032	18.981	14.327	17.027	12.436	14.48	13.164
4.581	6.321	4.594	3.105	2.856	1.227	0.549
5.296	5.549	1.678	1.21	0.229	0.111	0.003
13.501	9.902	7.356	5.445	4.785	2.068	0.721
10.487	6.28	7.141	7.563	6.512	7.982	4.038
5.334	3.579	2.186	1.765	1.26	0.055	0.06
0	0	0	0	0	0	0
10.241	15.014	10.246	7.663	8.187	6.452	5.466
5.301	3.699	4.506	4.032	3.487	3.277	0.817
11.407	8.145	6.751	9.317	6.04	3.958	5.047
0.004	0.1	0	0	0	0	0
18.339	22.043	12.79	10.349	11.879	14.582	7.505
2.382	1.656	1.76	1.88	0.509	0.173	0.631
12.788	8.057	8.301	8.037	7.298	4.708	3.708
7.578	5.926	5.109	2.881	3.848	1.959	0.018
7.172	4.38	5.042	2.644	4.719	3.827	0.432
9.585	5.657	5.402	3.896	5.47	5.077	3.032
6.783	8.123	3.428	3.813	5.23	3.678	2.458

5.931	7.015	6.333	6.367	6.953	5.539	6.071
7.835	5.658	5.75	2.722	6.013	5.741	7.222
14.068	15.599	15.704	9.189	13.374	10.27	8.196
6.5	5.267	5.235	3.695	5.66	4.165	5.048
3.514	2.651	1.646	0.773	0.67	1.165	0.041
4.68	7.307	4.239	2.708	8.048	4.892	2.093
9.603	7.218	7.924	5.849	7.434	4.587	3.756
10.636	10.309	10.81	5.335	12.865	10.719	9.565
3.455	5.196	2.475	1.73	3.365	1.979	0.73
8.725	6.075	7.47	4.061	7.518	4.576	5.481
5.368	7.971	8.079	2.403	6.778	3.315	3.162
3.33	3.488	2.936	1.173	0.686	0.27	0.03
4.588	2.875	2.222	3.527	1.018	1.796	0.03
6.052	8.137	4.847	5.124	2.6	5.428	4.195
6.033	6.593	4.502	3.634	2.19	2.486	2.505
5.692	2.98	3.217	2.451	3.385	1.155	1.776
4.962	4.037	6.398	5.508	3.675	2.107	1.239
9.169	5.099	6.065	5.117	4.797	3.052	2.514
0.533	0	0.097	0	0	0	0
3.555	2.501	0.412	0.123	0.589	0.382	0
4.209	4.337	2.361	1.521	0.215	0.051	0
2.47	1.8	2.646	0.99	0.538	0.349	0
5.551	3.407	1.47	1.334	0.016	0.007	0.004
2.93	1.288	1.434	0.014	0.226	0.009	0
1.883	1.385	0.297	1.063	0.5	0	0
3.503	2.362	2.533	2.769	2.084	1.421	0.82
7.178	4.177	5.582	2.419	4.342	2.989	5.749
5.393	7.82	5.434	6.161	5.572	4.343	5.818
0.555	0.171	0.477	0	0.176	0	0
6.24	3.122	0.43	1.715	1.095	0.959	0.396
8.79	2.795	10.692	8.472	6.866	2.817	2.847
0.249	0.002	0	0	0	0	0
4.532	5.214	2.956	2.817	0.736	0.672	0.085
0	0.018	0	0	0	0	0
5.232	2.334	2.722	1.481	0.825	0.275	0.033
0.403	0	0	0	0	0	0
2.083	0.807	0.339	0.857	0.244	0	0
3.041	2.264	1.267	0.486	0.211	0	0
3.605	3.375	4.214	2.936	0.935	0.606	0.507
0.974	0.193	0.128	0	0	0	0
0	0.421	0	0	0	0	0
7.07	6.451	2.719	0.365	0.295	0.009	0.003
0.407	1.155	1.08	0.09	0.018	0	0
0.879	0.148	0.066	0.134	0	0	0
6.48	6.283	3.008	3.126	1.384	1.286	0.562
19.34	30.391	22.9	22.48	25.309	15.805	25.222
10.846	6.507	3.579	1.78	1.421	1.362	0.293
2.337	0.992	1.209	0.241	0.076	0	0
7.495	1.029	3.476	0.607	1.415	0.171	0.3
47.403	40.424	38.849	34.284	32.975	21.96	19.046

15.325	9.893	13.814	10.552	7.615	5.127	6.776
5.196	6.743	5.958	6.015	3.806	6.222	5.784
8.916	5.387	5.434	3.63	3.295	2.87	0.705
3.699	2.066	2.857	0.459	1.162	0.782	0.257
5.123	2.586	2.865	0.912	0.576	0.006	0
1.992	0.356	0.308	0.005	0	0	0
19.155	9.938	11.685	8.515	10.465	7.248	2.968
3.393	3.671	3.758	1.816	1.45	2.616	2.918
3.145	4.251	2.031	2.095	1.54	1.723	0.596
0.701	0.097	0.014	0	0	0	0
7.41	5.99	4.563	6.322	5.122	4.759	5.144
5.356	3.377	0.676	2.349	3.258	0.618	1.608
8.39	7.876	7.053	6.012	5.057	4.875	2.222
8.685	5.857	2.22	2.725	7.56	4.76	0.615
2.001	0.237	0.181	0.005	0.006	0	0
0.409	1.2	0	0.366	0	0	0
2.577	1.687	2.041	2.083	2.363	0.261	0.659
0	0	0	0	0	0	0
9.518	8.845	7.449	5.785	3.493	4.788	5.962
3.376	1.552	0.864	1.025	0.72	0.298	0.279
0	0	0	0	0	0	0
8.601	14.982	9.912	13.148	12.739	7.023	12.1
2.716	3.888	1.497	0.055	0.881	0.626	0.864
23.729	13.635	12.742	7.805	10.404	10.338	7.121
0	0	0	0	0	0	0
1.702	0.479	0.392	0.013	0	0	0
0.88	2.011	0.821	0.96	0.284	0	0.143
4.993	3.151	2.724	1.607	0.901	0.678	0.054
14.239	9.486	8.051	7.731	9.718	5.783	4.298
11.036	15.303	7.47	9.789	6.555	12.361	9.886
10.1	13.613	11.379	9.918	10.55	6.239	12.464
7.039	6.803	5.602	4.988	3.301	2.259	1.275
8.928	4.961	4.863	2.296	3.237	2.52	1.173
0.684	0.145	0	0	0	0	0
0.567	0.101	0	0	0	0	0
1.359	0.596	1.055	0.403	0.201	0.145	0.024
19.259	11.482	8.261	8.196	14.293	13.911	5.694
4.283	3.142	2.671	0.786	1.422	0	0.465
3.682	1.697	2.226	2.719	1.074	3.213	0.51
11.512	11.214	10.358	8.04	5.135	5.685	5.705
0.236	0.053	0.087	0	0.002	0	0
0.001	0	0	0	0	0	0
4.219	3.143	0.958	0.934	0.173	0.091	0
4.862	2.13	4.423	1.317	1.959	2.154	2.882
2.179	0.491	0.628	0.093	0.097	0	0
1.362	0.17	0.075	0.06	0	0	0
4.255	3.64	3.837	3.877	3.524	2.115	2.679
2.084	0.75	0.974	0.238	0.08	0.018	0
0	0	0	0	0	0	0
4.961	4.608	2.252	2.184	0.604	1.035	0.777

2.376	3.087	0.288	0.885	0.03	0.144	0
6.321	5.08	1.774	2.857	5.089	3.977	1.655
4.547	3.458	2.289	1.802	0.327	2.007	1.306
0.163	0.28	0	0	0	0	0
8.121	9.072	6.589	7.747	3.339	5.424	2.963
6.672	3.204	3.025	4.183	4.694	0.822	1.708
2.73	1.5	1.78	1.837	1.323	1.414	0.652
1.096	0.53	0.293	0.068	0	0	0
1.037	0.62	0.15	0	0	0	0
2.052	1.736	0.616	0.478	0.021	0	0.01
0	0	0	0	0	0	0
0	0	0	0	0	0	0
1.656	1.133	0.593	0.228	0	0	0
6.055	4.462	4.4	1.817	1.013	0.842	0.012
12.378	7.441	7.789	4.492	8.81	4.31	4.183
5.235	3.412	4.008	1.736	1.098	0.46	0.796
6.306	6.082	2.604	2.043	3.074	4.297	1.146
7.647	3.078	4.743	2.229	2.785	3.326	1.916
0	0	0	0	0	0	0
2.278	1.332	0.619	0.066	0	0	0
24.835	13.088	18.478	13.206	14.046	11.634	10.415
0.27	0	0	0	0	0	0
7.387	9.014	4.385	3.194	3.287	1.386	1.925
3.352	2.604	0.784	1.772	1.918	1.142	0.165
7.559	5.607	2.658	3.248	4.925	6.452	3.779
10.991	9.573	4.431	5.139	2.358	1.098	0.394
0.775	0.533	0.349	0.298	0	0	0
3.423	2.629	1.342	0.534	0.536	0.435	0.006
0.91	0.301	0.047	0	0	0	0
1.548	0.892	2.959	0.258	1.354	0.07	0.29
2.48	1.946	0.561	0.128	0.249	0	0
7.993	10.205	2.56	8.728	2.939	8.624	5.145
2.526	1.147	0.186	0.027	0	0	0
2.63	0.896	1.169	0.225	0.14	0	0
3.186	2.597	1.358	0.45	0.668	0.166	0
0.705	1.297	0.201	0.737	0.001	0.004	0
0.278	0	0	0	0	0	0
5.289	2.836	0.231	1.6	1.105	1.097	0.36
2.269	2.189	1.269	0.29	0.563	0.002	0.114
13.385	10.146	10.894	10.374	12.101	12.344	8.862
4.944	4.677	2.133	2.251	0.631	1.04	0.781
3.499	5.883	3.48	3.955	4.188	2.665	4.754
2.044	2.135	0.808	0.748	0.072	0.104	0
4.863	6.733	2.729	0.837	0.308	0.143	0
0.203	0.154	0.015	0	0	0	0
1.044	0.553	0.348	0.054	0.069	0	0
3.71	4.398	1.979	2.324	1.632	2.453	0.695
20.175	25.534	16.437	21.758	14.345	14.96	16.06
0.821	0	0	0	0	0	0
1.618	0.45	0	0.409	0.685	0.127	0

0.129	0.058	0	0	0	0	0
0.069	0	0	0	0	0	0
3.573	4.901	2.026	1.611	1.063	1.392	0.84
0.205	0.002	0	0	0	0	0
5.331	3.757	3.544	2.426	2.793	2.595	1.354
16.051	15.986	12.244	7.895	9.417	6.881	6.717
6.573	4.53	4.906	3.817	2.349	1.11	0.347
2.04	1.206	0.506	0.613	0.123	0.008	0.019
3.506	1.306	0.377	0.07	0.226	0.001	0.019
6.187	2.993	1.913	2.465	3.297	4.069	1.661
11.095	9.377	6.058	8.452	3.379	2.83	1.251
10.424	3.063	6.326	3.95	3.367	2.467	2.872
4.133	3.874	2.251	1.622	0.721	0.888	0.116
3.983	5.127	2.777	1.58	1.82	1.126	1.134
6.645	3.498	2.61	1.489	1.97	2.267	0.598
2.929	2.663	2.726	0.802	0.62	0.115	0
8.326	6.29	1.373	5.982	7.103	7.713	0.899
6.364	1.892	3.67	2.491	2.129	4.669	1.154
0.304	0.049	0	0	0	0	0
6.365	1.898	3.68	2.488	2.128	4.66	1.155
0.467	0.371	0	0.553	0.525	0	0
4.788	4.732	6.502	6.273	5.138	5.424	3.457
4.851	2.727	1.904	0.419	0.129	0	0
7.385	4.744	2.395	3.771	0.852	0.405	0.054
4.037	4.814	2.594	2.576	1.804	5.797	3.825
12.749	13.86	10.191	10.903	9.543	5.828	7.173
1.005	0.411	0.001	0.001	0.001	0	0
0	0	0	0	0	0	0
6.289	6.17	4.184	6.526	6.336	7.587	5.813
4.208	2.439	1.401	0.48	0.053	0.001	0
21.291	15.957	18.423	15.047	17.268	10.237	9.897
4.937	3.077	1.204	0.631	0.05	0.001	0.002
0	0	0	0	0	0	0
7.383	2.829	3.195	1.254	0.706	0.199	0.496
7.755	11.804	13.045	6.024	7.357	7.689	8.735
3.415	3.56	3.011	1.2	2.131	0.016	0.074
11.788	8.034	5.839	5.406	4.727	3.295	2.177
0.253	0.078	0.33	0.005	0.024	0	0
3.336	1.053	0.754	1.19	0.952	0.178	0.001
8.239	7.446	3.968	5.705	5.871	7.741	4.928
1.507	1.08	0.652	0.153	0	0	0
4.863	4.012	2.665	2.72	1.861	1.298	1.554
8.818	5.987	5.124	3.788	4.574	2.545	1.273
3.282	3.125	2.012	0.893	1.064	1.761	1.147
2.214	1.077	0.614	1.172	1.168	0.492	0.007
6.09	2.968	1.734	0.86	0	0.021	0
5.058	5.429	1.075	4.931	6.22	2.646	4.261
0.971	0.193	0.128	0	0	0	0
0.449	0.451	0.247	0	0	0	0
3.866	1.91	1.895	0.368	0.287	0.148	0.053

11.484	3.386	7.409	4.168	6.345	4.279	2.262
1.258	2.533	1.213	0.476	0.289	0	0
3.881	1.281	1.287	0.231	0.208	0.004	0
9.049	4.159	5.889	2.179	2.818	2.383	0.619
0	0	0	0	0	0	0
0.011	1.516	0.006	0.036	0	0	0
2.697	0.033	0.039	0	0	0	0
6.166	7.012	3.109	2.113	3.44	2.547	1.059
8.484	5.203	1.803	3.15	5.17	6.139	1.829
5.923	2.398	3.287	2.086	3.786	2.1	1.281
2.669	4.025	1.839	2.845	1.947	1.211	0.285
2.565	2.156	1.147	0.707	0.141	0.001	0
3.969	7.959	3.203	3.242	5.096	5.718	5.261
1.787	0.843	0.456	0.057	0	0	0
2.24	1.775	0.737	0.245	0.229	0	0
11.407	7.823	5.126	5.82	7.026	5.203	4.041
0.733	0	0.001	0	0	0	0
10.975	11.503	11.417	8.091	5.823	6.584	5.515
23.255	21.296	21.975	17.043	18.312	14.706	12.567
10.328	9.692	9.585	7.946	8.27	14.67	11.373
33.869	35.757	33.272	34.94	30.218	28.519	23.464
4.279	5.117	1.832	2.577	0.226	0.212	0
2.932	1.959	1.145	0.113	0.124	0	0
2.417	3.146	2.368	1.6	2.161	1.215	0.672
1.934	1.759	0.272	0.268	0.046	0	0
7.994	6.865	3.181	6.639	5.758	6.541	5.933
3.774	2.356	2.79	1.584	0.736	0.205	0.546
1.58	1.484	0.685	0.596	0.169	0.762	0.011

RDF120p	RDF125p	RDF130p	RDF135p	RDF140p	RDF145p	RDF150p
0.022	0	0	0	0	0	0
2.803	3.491	3.635	1.709	1.108	2.831	1.967
0.003	0	0	0	0	0	0
0	0	0	0	0	0	0
0	0	0	0	0	0	0
0	0	0	0	0	0	0
3.401	1.846	0.617	0.591	0.814	1.063	0.257
0	0	0	0	0	0	0
0	0	0	0	0	0	0
0.69	0.284	0.039	0	0	0	0
2.194	1.589	1.98	1.364	0.174	0.046	0
6.46	5.14	5.282	2.931	2.284	0.336	0.12
0	0	0	0	0	0	0
0.457	0	0	0	0	0	0
0	0	0	0	0	0	0
0.366	0.006	0	0	0	0	0
0	0	0	0	0	0	0
1.186	0.357	0.327	0.153	0.023	0	0
0.788	2.339	1.926	1.031	0.439	0.432	0.34
0.542	0.239	0.085	0	0	0	0
0.001	0	0	0	0	0	0
0	0	0	0	0	0	0
0.847	0.576	0.154	0.065	0.255	0.371	0
1.01	0.876	0.051	0.21	0	0	0
1.588	0.041	0.168	0.031	0	0	0
0.07	0.075	0	0.069	0	0	0
2.893	3.129	5	2.037	3.52	2.192	1.292
7.072	7.343	4.478	3.235	2.681	3.816	3.533
0.249	0.061	0.004	0	0	0	0
2.148	4.175	3.368	2.566	2.769	4.332	2.806
6.488	4.525	5.096	1.109	0.976	0.106	0.088
15.056	5.616	5.483	5.875	3.275	4.345	3.192
0.175	0	0	0	0	0	0
0	0	0	0	0	0	0
0.15	0.025	0	0	0	0	0
4.36	7.431	5.314	3.203	5.154	4.398	2.658
0.029	0	0	0	0	0	0
0	0	0	0	0	0	0
5.748	3.006	5.174	2.133	6.174	2.808	3.958
1.402	0.235	0.176	0	0	0	0
2.421	1.02	0.648	0	0.004	0	0
0	0	0	0	0	0	0
5.059	8.382	7.079	6.836	2.768	2.944	6.837
0.219	0	0	0	0	0	0
4.128	4.125	3.103	3.128	0.624	1.42	0.553
0.119	0	0	0	0	0	0
0.763	0.13	0.214	0.012	0	0	0
1.147	2.02	2.078	2.429	4.779	3.31	3.16
3.249	2.535	2.368	1.619	1.059	2.835	0.984

5.503	5.078	4.962	3.513	2.894	1.785	1.545
4.633	4.413	2.794	1.683	2.46	0.491	1.177
5.611	9.803	7.825	9.885	8.057	7.1	7.95
4.032	2.723	2.506	2.206	2.255	1.634	1.674
0.683	0	0.02	0	0	0	0
3.598	2.958	2.491	1.978	0.318	0.028	0.2
2.532	4.415	6.906	6.571	3.293	3.049	1.958
6.652	4.06	8.237	4.992	2.125	3.552	0.563
0.279	0.028	0.025	0	0	0	0
6.666	5.027	4.855	2.267	2.146	6.285	3.815
3.035	1.91	3.33	1.667	1.435	0.88	1.191
0	0	0	0	0	0	0
0.425	0.024	0.012	0	0	0	0
3.886	3.295	3.156	2.548	2.01	1.669	1.784
2.548	2.654	1.429	1.147	1.001	0.814	0.158
0.288	0.194	0.008	0	0	0	0
0.625	0.133	0.061	0	0	0	0
1.249	1.207	0.736	0.553	0	0.412	0.001
0	0	0	0	0	0	0
0	0	0	0	0	0	0
0	0	0	0	0	0	0
0	0	0	0	0	0	0
0	0	0	0	0	0	0
0	0	0	0	0	0	0
0	0	0	0	0	0	0
1.152	0.463	0	0	0	0	0
1.339	1.997	2.451	1.292	1.432	0.764	1.819
3.646	3.276	0.929	1.668	1.081	1.23	0.213
0	0	0	0	0	0	0
0.532	0	0.074	0	0	0	0
3.239	2.902	1.501	1.181	0.701	0.066	0.109
0	0	0	0	0	0	0
0.133	0	0	0	0	0	0
0	0	0	0	0	0	0
0	0	0	0	0	0	0
0	0	0	0	0	0	0
0	0	0	0	0	0	0
0	0	0	0	0	0	0
0.066	0.001	0	0	0	0	0
0	0	0	0	0	0	0
0	0	0	0	0	0	0
0	0	0	0	0	0	0
0	0	0	0	0	0	0
0	0	0	0	0	0	0
0.87	0.146	0.064	0	0	0	0
17.075	20.456	16.116	12.171	18.935	12.58	10.795
0.542	0.001	0.086	0.002	0	0	0
0	0	0	0	0	0	0
0	0	0	0	0	0	0
14.048	13.218	7.112	5.958	1.933	0.937	0.074

7.087	3.254	1.926	3.818	0.8	0.696	0.085
6.552	4.218	5.039	2.92	4.732	1.652	1.413
0.849	0.06	0.05	0.034	0.003	0	0
0.11	0.045	0	0	0	0	0
0	0	0	0	0	0	0
0	0	0	0	0	0	0
6.526	3.765	3.991	1.035	2.008	2.72	0.97
0.201	2.376	0.184	0.523	0.056	0	0
0.634	0.169	0.356	0.201	0.051	0.002	0
0	0	0	0	0	0	0
4.976	3.225	2.068	1.57	0.919	0.943	0.168
0.003	0.726	0	0	0	0	0
2.206	1.223	0.812	0.334	0.512	0.064	0
1.178	0.44	0.001	0.18	0	0	0
0	0	0	0	0	0	0
0	0	0	0	0	0	0
0.322	0.515	0	0.064	0	0	0
0	0	0	0	0	0	0
2.437	1.847	1.639	1.142	0.427	0.227	0.039
0.143	0	0	0	0	0	0
0	0	0	0	0	0	0
7.909	8.993	11.679	8.052	11.623	7.625	5.698
0.069	0.027	0	0	0	0	0
3.355	5.975	7.781	4.239	3.85	3.018	4.619
0	0	0	0	0	0	0
0	0	0	0	0	0	0
0	0	0	0	0	0	0
0.087	0.009	0.008	0	0	0	0
2.901	2.937	1.053	0.448	0.28	0.112	0
8.941	6.904	8.832	7.129	9.218	5.902	5.378
7.309	8.181	9.879	6.459	4.166	2.605	3.075
0.358	0.161	0	0.001	0	0	0
0.899	0.293	0.13	0	0	0	0
0	0	0	0	0	0	0
0	0	0	0	0	0	0
0	0	0	0	0	0	0
9.747	9.528	10.442	8.805	7.95	7.794	5.764
0	0	0	0	0	0	0
1.337	0.2	0.159	0.149	0.001	0	0
3.123	1.851	1.484	0.446	0.007	0.003	0
0	0	0	0	0	0	0
0	0	0	0	0	0	0
0	0	0	0	0	0	0
0.855	1.056	0.143	0.041	0.001	0	0
0	0	0	0	0	0	0
0	0	0	0	0	0	0
2.22	0.578	2.534	2.052	0.585	0.046	0.237
0	0	0	0	0	0	0
0	0	0	0	0	0	0
0.481	0.292	0.519	0.273	0.205	0	0

0	0	0	0	0	0	0
0.909	2.029	2.391	1.144	0.905	0.568	1.515
0.578	0.551	0.014	0.144	0	0	0
0	0	0	0	0	0	0
1.227	1.373	0.508	0.448	0.065	0	0
1.532	0.569	0.206	0.037	0.031	0	0
0	0.04	0	0	0	0	0
0	0	0	0	0	0	0
0	0	0	0	0	0	0
0	0	0	0	0	0	0
0	0	0	0	0	0	0
0	0	0	0	0	0	0
0	0	0	0	0	0	0
0.054	0	0	0	0	0	0
2.572	4.783	2.479	4.193	3.225	3.353	1.654
0.003	0.033	0.052	0	0	0	0
1.325	0.614	0.222	0.01	0	0	0
1.293	2.702	2.646	1.313	1.779	1.46	1.04
0	0	0	0	0	0	0
0	0	0	0	0	0	0
13.468	16.812	14.484	11.748	10.804	8.362	4.675
0	0	0	0	0	0	0
0.269	0.292	0.019	0	0	0	0
0.477	0.618	0.247	0	0	0	0
2.664	1.668	1.26	0.31	0.081	0	0
0.165	0.001	0	0	0	0	0
0	0	0	0	0	0	0
0	0	0	0	0	0	0
0	0	0	0	0	0	0
0.001	0.002	0	0	0	0	0
0	0	0	0	0	0	0
3.178	4.311	6.093	4.621	2.991	2.067	2.152
0	0	0	0	0	0	0
0	0	0	0	0	0	0
0	0	0	0	0	0	0
0	0	0	0	0	0	0
0	0	0	0	0	0	0
0.506	0	0	0	0	0	0
0	0	0	0	0	0	0
10.459	8.357	7.304	6.159	10.444	11.049	6.807
0.511	0.32	0.525	0.279	0.215	0	0
3.001	2.044	2.17	2.015	1.407	0.498	1.034
0	0	0	0	0	0	0
0	0	0	0	0	0	0
0	0	0	0	0	0	0
0	0	0	0	0	0	0
0.016	0.173	0.226	0.002	0	0	0
14.171	10.424	13.298	9.683	8.398	9.206	4.056
0	0	0	0	0	0	0
0	0	0	0	0	0	0

0	0	0	0	0	0	0
0	0	0	0	0	0	0
0.142	0.117	0.012	0	0	0	0
0	0	0	0	0	0	0
1.532	0.305	0.388	0	0.001	0	0
3.13	1.442	1.956	0.819	1.374	0.128	0.046
0.12	0.071	0	0	0	0	0
0	0	0	0	0	0	0
0	0	0	0	0	0	0
1.015	1.422	2.208	1.091	0.477	0.63	1.01
1.501	1.53	0.851	0.595	0.008	0.162	0.242
4.824	2.339	2.351	2.314	1.658	0.687	1.296
0.007	0	0	0	0	0	0
0.35	0.015	0.123	0	0	0	0
0.857	0.678	0.291	0.027	0	0	0
0	0	0	0	0	0	0
3.265	3.312	3.757	1.433	1.512	2.556	2.595
2.48	0.742	1.297	0.028	0.151	0	0
0	0	0	0	0	0	0
2.48	0.742	1.293	0.028	0.149	0	0
0	0	0	0	0	0	0
3.222	3.073	0.285	0.584	0.291	0.139	0
0	0	0	0	0	0	0
0	0	0	0	0	0	0
3.534	3.376	2.063	1.242	1.03	0.606	0.807
6.96	5.431	3.457	2.149	0.96	0.125	0.145
0	0	0	0	0	0	0
0	0	0	0	0	0	0
4.445	3.751	2.705	3.956	2.938	3.203	1.953
0	0	0	0	0	0	0
14.142	5.364	3.862	2.772	1.493	0.478	0.146
0	0	0	0	0	0	0
0	0	0	0	0	0	0
0.165	0.004	0	0	0	0	0
3.841	4.562	5.418	4.787	2.229	2.045	0.112
0	0	0	0	0	0	0
1.437	1.524	0.996	0.368	0.123	0	0
0	0	0	0	0	0	0
0	0	0	0	0	0	0
2.576	2.266	2.949	1.218	0.517	0.531	0.324
0	0	0	0	0	0	0
0.535	1.04	0.275	0	0.081	0	0
0.747	0.185	0	0	0	0	0
0.09	0.001	0	0	0	0	0
0.016	0	0	0	0	0	0
0	0	0	0	0	0	0
3.522	3.309	2.755	2.364	1.987	2.005	0.576
0	0	0	0	0	0	0
0	0	0	0	0	0	0
0	0	0	0	0	0	0

4.3	2.417	3.628	1.472	2.048	1.116	0.697
0	0	0	0	0	0	0
0	0	0	0	0	0	0
0.8	0.055	0.058	0.024	0.004	0	0
0	0	0	0	0	0	0
0	0	0	0	0	0	0
0	0	0	0	0	0	0
0.576	0.615	0.003	0.159	0.001	0	0
3.818	7.323	5.897	3.073	2.909	3.078	1.816
0.271	0.307	0.165	0	0	0	0
0.495	0.023	0.001	0	0	0	0
0	0	0	0	0	0	0
4.634	4.314	1.531	3.581	1.648	2.216	0.557
0	0	0	0	0	0	0
0	0	0	0	0	0	0
5.541	3.501	4.694	3.662	2.824	1.389	3.011
0	0	0	0	0	0	0
2.234	1.999	0.906	0.193	0.218	0	0
13.834	11.607	4.618	4.827	3.696	1.482	0.743
9.914	6.229	8.362	9.89	3.833	4.989	4.577
20.03	15.908	11.063	5.873	7.893	2.994	3.186
0	0	0	0	0	0	0
0	0	0	0	0	0	0
0.575	0.619	0.215	0.091	0	0	0
0	0	0	0	0	0	0
6.171	1.97	3.667	2.335	3.295	4.011	0.667
0.899	0.081	0.004	0	0	0	0
0.123	0.395	0	0	0	0	0

RDF155p	Mor01u	Mor02u	Mor03u	Mor04u	Mor05u	Mor06u
0	528	30.198	-4.007	-1.914	-4.929	-2.327
0.818	1326	57.885	-11.266	12.543	-8.136	-4.772
0	820	33.661	-4.773	3.194	-6.225	-3.638
0	190	20.183	-1.375	1.429	-2.802	-1.96
0	351	26.752	-2.736	0.218	-3.463	-2.664
0	351	16.484	-4.705	4.639	-4.735	0.642
0.021	1891	34.58	-8.665	5.282	-9.743	-4.001
0	91	13.983	-0.593	-0.374	-1.63	-1.554
0	595	25.203	-3.079	-0.658	-5.784	-1.206
0	820	38.287	-7.657	5.831	-6.21	-4.894
0	3403	79.345	-18.314	18.783	-14.009	-6.649
0	1770	46.577	-8.29	0.626	-9.318	-4.46
0	946	34.029	-7.988	2.129	-5.857	-5.008
0	1378	37.908	-6.761	6.805	-7.568	-3.687
0	946	30.953	-5.571	1.859	-5.45	-3.01
0	903	31.06	-6.2	1.368	-5.918	-2.372
0	325	20.985	-1.485	-1.115	-3.449	-1.945
0	1653	40.762	-9.428	5.003	-7.888	-4.094
0.119	820	42.799	-7.085	7.541	-5.204	-5.229
0	946	46.097	-6.818	3.592	-6.378	-5.347
0	861	42.49	-5.859	1.888	-5.801	-3.273
0	325	17.562	-1.566	-2.417	-2.44	-1.431
0	528	28.758	-2.158	0.175	-4.597	-2.476
0	820	31.236	-4.981	0.851	-5.327	-2.547
0	1830	59.459	-12.189	4.061	-9.187	-6.85
0	1596	22.639	-11.936	7.792	-10.568	-2.568
3.65	1275	57.251	-11.438	10.016	-7.518	-4.94
3.272	3081	65.891	-9.749	-0.394	-11.547	-1.677
0	990	44.294	-3.622	-0.606	-5.744	-3.012
2.075	1431	63.544	-10.229	8.649	-7.547	-6.321
0	2278	69.873	-4.85	1.579	-8.534	-6.551
2.579	3486	71.555	-19.92	9.174	-13.138	-5.687
0	861	43.303	-6.557	1.485	-7.405	-2.205
0	1176	52.552	-10.532	6.002	-8.85	-4.057
0	2775	38.353	-14.543	9.595	-12.737	-1.747
1.773	1711	56.578	-11.345	8.379	-8.81	-4.972
0	1378	30.938	-13.054	10.616	-9.868	-2.418
0	276	21.014	-1.525	-1.581	-2.892	-1.456
2.895	2701	71.058	-15.805	14.855	-11.349	-4.859
0	861	37.81	-6.155	2.095	-6.457	-2.131
0	1378	30.539	-6.028	0.659	-6.991	-5.284
0	435	17.229	-1.762	0.8	-4.986	-3.69
4.848	4095	34.46	-17.738	22.183	-16.833	-0.339
0	465	27.151	-2.358	0.235	-4.308	-2.28
0.138	1540	58.877	-8.931	5.767	-8.406	-6.098
0	703	28.594	-7.057	2.066	-4.941	-2.904
0	861	30.84	-7.152	2.597	-5.085	-3.283
1.346	1176	42.116	-7.701	2.008	-6.322	-3.687
1.963	903	35.741	-5.679	0.826	-4.74	-2.643

2.298	1540	46.81	-9.191	5.091	-6.617	-4.136
0.039	990	34.965	-4.995	2.217	-5.783	-2.115
6.511	2556	61.097	-8.797	7.273	-10.493	-4.777
0.685	1081	39.608	-5.356	2.491	-6.805	-1.86
0	990	39.677	-4.615	1.539	-6.712	-2.165
0.001	1035	34.772	-6.908	4.188	-5.836	-2.999
1.106	1540	42.729	-7.588	5.014	-6.822	-5.236
0.981	1711	43.835	-6.519	3.863	-7.392	-4.562
0	703	30.208	-3.399	2.212	-5.326	-1.589
3.354	1431	42.859	-5.657	2.455	-7.289	-2.792
0.31	990	37.085	-4.8	2.641	-7.161	-2.962
0	780	32.533	-2.749	1.455	-5.631	-2.058
0	820	35.305	-7.049	2.866	-4.329	-3.48
0.197	990	35.996	-7.718	2.361	-5.15	-4.065
0.365	861	34.805	-6.075	2.443	-5.438	-2.197
0	903	35.534	-6.206	1.98	-4.431	-3.442
0	1485	41.289	-13.303	4.368	-7.074	-5.71
0	1326	37.079	-7.477	4.839	-7.502	-4.938
0	253	16.275	-1.487	1.52	-3.349	0.552
0	703	33.125	-6.357	2.651	-5.306	-3.597
0	780	39.449	-7.482	2.27	-5.419	-2.252
0	435	32.955	-5.873	4.036	-4.127	-1.041
0	741	41.708	-5.164	2.246	-5.417	-1.798
0	528	20.716	-3.835	2.616	-4.577	-1.563
0	435	23.739	-1.46	0.166	-3.63	-2.013
0	528	29.388	-3.55	2.081	-4.159	-2.299
0.955	903	42.024	-5.876	3.029	-5.821	-4.183
0.391	1128	41.032	-9.236	5.531	-8.54	-4.733
0	406	25.298	-3.315	0.217	-4.559	-2.166
0	861	41.938	-6.704	3.866	-5.972	-3.972
0.004	1770	45.915	-13.204	10.903	-9.71	-4.499
0	300	21.609	-1.67	0.745	-3.058	-2.177
0	820	32.595	-1.557	-2.976	-4.967	-3.18
0	253	20.554	-3.107	0.74	-2.512	-1.797
0	861	38.755	-5.154	1.72	-4.556	-4.272
0	406	21.648	-7.334	2.853	-3.464	-1.228
0	406	26.092	-1.354	1.593	-3.359	-2.933
0	861	34.618	-8.262	2.726	-6.719	-4.991
0	1596	22.708	-13.294	7.811	-10.226	-2.872
0	528	23.743	-3.148	1.545	-5.272	-1.968
0	435	13.858	-1.369	0.233	-3.438	-2.934
0	1081	42.138	-2.793	-0.851	-5.166	-0.646
0	406	26.822	-4.178	-0.044	-2.731	-2.951
0	325	21.219	-2.223	2.136	-4.455	-1.825
0	1830	21.464	-14.845	10.596	-11.356	-2.141
19.337	6903	53.355	-28.563	19.385	-19.212	-3.219
0	1485	55.322	-7.834	0.277	-7.703	-4.973
0	780	39.126	-4.625	1.078	-4.145	-5.152
0	1431	33.88	-6.434	1.844	-7.544	-6.224
0.036	6105	58.414	-14.623	11.439	-17.376	-10.432

0	1540	49.195	-5.471	3.159	-7.96	-3.637
1.462	1431	47.491	-8.767	5.612	-8.082	-3.764
0	1540	26.722	-7.546	6.262	-7.831	-5.641
0	666	31.845	-5.251	2.37	-5.907	-2.263
0	703	46.884	-8.877	5.601	-4.572	-5.421
0	861	34.847	-5.352	1.615	-6.214	-2.403
0.608	2701	55.47	-14.878	8.449	-12.401	-6.147
0	595	30.686	-3.33	4.401	-5.222	-2.229
0	528	32.352	-4.422	4.825	-5.335	-2.703
0	496	29.933	-1.718	0.156	-4.01	-4.193
0.228	1378	44.738	-8.427	5.709	-8.224	-5.718
0	561	30.683	-2.522	1.329	-4.205	-2.64
0	1953	39.891	-13.625	10.825	-12.434	-1.343
0	1128	53.51	-12.291	11.336	-7.86	-3.875
0	561	15.778	-2.663	0.405	-3.545	-3.855
0	351	20.979	-2.713	0.349	-2.706	-2.817
0	630	30.043	-4.224	1.071	-4.742	-2.252
0	66	13.638	-0.486	-0.373	-1.11	-1.571
0	1540	52.707	-11.17	7.247	-8.125	-3.891
0	465	27.08	-1.814	2.066	-4.453	-3.282
0	45	7.667	0.392	-0.011	-1.106	-0.107
11.464	3828	79.373	-27.257	24.562	-18.543	7.461
0	496	26.04	-3.01	2.93	-4.279	-1.289
1.345	3570	48.158	-14.047	16.477	-14.456	-6.66
0	406	25.966	-5.619	2.37	-4.468	-2.124
0	861	37.398	-4.435	1.727	-4.237	-4.832
0	465	29.882	-2.839	0.416	-3.297	-3.746
0	780	42.602	-4.222	4.106	-6.091	-2.335
0	2415	46.31	-16.235	12.614	-13.788	-3.315
8.765	2278	70.759	-11.616	10.03	-10.927	-2.415
2.263	1830	67.561	-9.427	10.949	-8.949	-4.271
0	1081	42.555	-6.278	2.364	-5.449	-4.574
0	990	37.867	-7.496	4.954	-6.35	-4.782
0	300	19.734	-2.472	2.243	-3.563	0.81
0	780	15.628	-8.36	4.924	-7.388	-2.363
0	528	34.237	-3.789	3.565	-3.659	-3.113
4.578	2278	66.405	-7.966	9.304	-10.186	-8.588
0	820	32.867	-1.674	-2.533	-4.865	-2.865
0	630	31.278	-2.596	2.25	-4.814	-3.209
0	1770	59.451	-11.361	4.723	-7.881	-5.251
0	276	23.04	-3.508	0.481	-2.372	-2.916
0	231	23.009	-3.567	0.728	-2.303	-2.47
0	1225	26.014	-8.224	3.132	-6.879	-4.054
0	561	26.507	-2.715	2.858	-5.39	-2.812
0	528	30.196	-2.673	1.007	-4.685	-3.709
0	325	23.084	-2.338	1.949	-3.189	-2.891
0.003	741	31.997	-3.577	0.665	-5.095	-3.605
0	528	20.702	-1.909	-0.221	-4.923	-3.279
0	325	20.243	-3.989	2.632	-4.213	-1.888
0	1035	43.123	-6.935	4.59	-7.83	-3.606

0	741	36.176	-6.362	2.435	-6.73	-3.987
0.599	946	46.307	-7.206	9.751	-6.291	-3.276
0	946	43.245	-7.884	4.123	-7.094	-3.754
0	465	20.139	-1.936	1.025	-4.28	-1.843
0	1378	45.009	-6.604	0.559	-7.888	-4.429
0	630	28.465	-2.441	1.307	-5.269	-0.366
0	666	34.584	-4.668	3.41	-5.078	-3.488
0	741	30.29	-5.944	4.037	-6.167	-3.464
0	741	31.582	-5.4	2.426	-6.471	-4.377
0	780	33.081	-6.592	3.595	-6.981	-4.133
0	91	13.919	-0.776	-0.726	-1.227	-0.963
0	190	17.17	1.185	-0.169	-1.975	-2.161
0	1225	17.316	-5.346	2.888	-6.951	-4.882
0	1035	46.689	-5.491	-0.232	-4.848	-3.28
0.979	1485	46.323	-6.668	4.13	-8.105	-5.223
0	1596	24.754	-12.987	9.181	-11.076	-2.398
0	861	37.705	-5.625	6.862	-6.485	-3.264
0.759	946	48.366	-9.158	7.651	-6.318	-4.645
0	210	16.395	-0.698	-0.799	-2.383	-1.298
0	630	23.723	-2.887	-0.652	-5.234	-1.2
3.074	2926	66.525	-10.711	4.091	-12.38	-8.18
0	780	13.234	-6.963	3.263	-6.127	-2.985
0	1378	47.014	-8.426	4.271	-8.377	-3.609
0	990	46.902	-4.197	2.183	-4.595	-3.599
0	1176	44.737	-9.949	8.916	-8.409	-3.123
0	1275	52.081	-7.531	1.255	-5.686	-3.205
0	406	22.573	-3.487	0.806	-4.428	-2.479
0	1225	20.039	-10.488	7.178	-8.422	-2.974
0	946	14.563	-8.95	4.673	-8.177	-2.958
0	465	28.866	-1.919	1.455	-4.127	-3.722
0	595	42.017	-4.127	-1.049	-4.009	-4.371
0.28	1596	42.188	-10.537	6.767	-9.879	-1.125
0	903	18.418	-5.252	2.292	-5.192	-4.185
0	528	26.286	-2.487	1.578	-5.06	-2.156
0	1035	26.291	-7.248	3.349	-6.82	-4.328
0	253	25.704	-2.886	0.709	-2.99	-0.962
0	253	21.77	-2.734	2.636	-3.3	-1.757
0	820	34.947	-7.207	3.933	-6.269	-3.927
0	820	33.983	-7.169	3.375	-6.044	-4.756
8.46	3160	54.436	-7.349	5.579	-12.169	-6.839
0	1035	43.229	-6.856	4.528	-7.854	-3.688
0.533	903	34.377	-1.809	-1.112	-6.091	-1.833
0	703	35.816	-5.794	0.95	-5.769	-1.472
0	1081	47.847	-1.004	-1.857	-6.401	-2.051
0	465	20.652	-1.962	1.281	-4.921	-2.184
0	946	24.064	-7.626	5.782	-7.596	-2.41
0	946	37.483	-6.838	5.818	-8.092	-3.291
3.152	5050	51.976	-25.991	19.379	-18.035	-2.766
0	561	22.157	-6.526	3.118	-5.774	-1.21
0	325	28.238	-2.935	2.369	-3.164	-3.471

0	903	24.191	-8.787	4.297	-6.999	-4.243
0	406	18.401	-0.836	-0.452	-3.352	-3.511
0	903	36.018	-6.825	4.83	-7.637	-3.957
0	465	18.8	-3.415	1.041	-4.343	-3.775
0	703	38.909	-7.701	5.574	-5.186	-4.127
0.013	2080	62.075	-11.997	8.471	-9.745	-2.719
0	1081	42.874	-5.728	4.505	-8.177	-4.465
0	903	31.985	-3.844	1.844	-6.357	-2.859
0	465	30.918	-3.535	2.029	-4.321	-2.222
0.009	820	44.463	-7.839	7.453	-6.133	-4.485
0.001	2145	63.105	-15.389	13.204	-11.717	-3.323
0.012	1176	48.528	-5.674	0.128	-5.6	-6.993
0	1326	26.368	-8.396	7.401	-9.245	-3.459
0	1485	32.74	-11.76	11.319	-10.098	-1.323
0	703	38.35	-5.13	6.123	-5.627	-4.154
0	666	37.828	-4.915	3.303	-4.839	-4.039
1.609	1326	55.87	-10.649	13.78	-8.048	-5.525
0	780	39.698	-7.088	5.5	-5.973	-5.395
0	465	28.861	-3.002	1.385	-4.487	-4.113
0	780	39.696	-7.088	5.499	-5.973	-5.396
0	435	14.068	-1.998	1.31	-4.12	-2.619
0.001	1326	45.142	-8.148	2.228	-7.542	-5.474
0	1128	43.684	-7.116	1.91	-5.893	-6.548
0	1128	47.583	-7.63	2.761	-5.547	-6.889
1.285	903	42.703	-6.13	1.929	-5.873	-2.865
0	2346	47.152	-13.029	7.678	-10.672	-7.246
0	190	18.258	-1.995	0.107	-2.552	-0.98
0	378	8.68	-2.343	2.384	-5.271	-1.024
2.199	1596	53.777	-8.018	5.734	-8.983	-3.374
0	741	45.773	-3.937	-0.306	-4.517	-3.239
0.086	4095	28.156	-24.32	17.55	-16.76	-3.542
0	1035	45.478	-8.734	5.551	-8.17	-4.058
0	120	13.797	-1.036	0.555	-2.373	-1.633
0	903	31.977	-6.961	3.666	-6.992	-4.348
0.433	1953	53.136	-7.887	2.11	-8.799	-4.706
0	703	37.902	-6.1	3.718	-6.415	-2.662
0	1596	36.034	-9.71	5.972	-9.317	-5.721
0	378	26.831	-0.905	0.374	-3.376	-1.557
0	630	31.855	-2.245	1.894	-4.738	-3.382
0	1176	33.529	-5.534	3.204	-7.801	-5.355
0	465	29.581	0.15	-1.156	-3.716	-2.164
0	861	26.802	-2.275	-1.241	-6.177	-1.429
0	1035	48.102	-7.219	5.922	-6.161	-4.176
0	820	43.077	-4.792	0.782	-5.048	-2.674
0	435	25.799	-2.233	2.11	-4.092	-2.455
0	1540	28.536	-11.639	5.541	-6.096	-6.101
1.305	990	49.117	-7.265	4.62	-6.877	-4.453
0	561	22.05	-3.258	1.539	-5.361	-2.143
0	528	16.174	-0.939	0.731	-4.611	-1.912
0	528	24.991	-3.579	0.284	-4.321	-2.42

0.15	1378	53.421	-8.092	1.852	-5.576	-6.971
0	595	35.988	-6.541	3.138	-6.458	-0.851
0	1176	29.664	-9.218	7.448	-7.619	-3.296
0	1540	28.766	-7.368	5.853	-9.204	-5.122
0	210	18.095	-2.62	-1.273	-3.082	-2.146
0	561	23.259	-4.039	4.741	-6.198	-1.056
0	465	25.848	-4.821	2.523	-4.036	-2.384
0	990	45.304	-9.851	7.021	-7.73	-2.183
1.581	1176	48.079	-8.184	7.623	-7.699	-6.206
0	861	44.854	-5.911	1.472	-4.688	-4.201
0	630	40.231	-5.69	5.733	-5.292	-1.848
0	946	36.279	-7.18	4.545	-7.473	-3.287
0.591	1128	47.649	-8.049	3.478	-6.97	-3.911
0	595	21.948	-2.436	-1.01	-5.334	-0.664
0	741	31.341	-2.227	0.969	-4.335	-4.177
1.936	1596	53.197	-11.043	10.364	-8.766	-6.512
0	325	29.96	-6.78	4.916	-4.19	-1.272
0	1830	43.898	-10.6	5.381	-9.195	-4.815
0.122	4656	49.027	-25.499	17.118	-17.938	-4.835
3.335	2485	63.84	-11.528	8.127	-8.827	-6.728
1.42	6105	37.399	-11.375	8.168	-16.164	-8.753
0	1326	47.908	-9.561	4.493	-6.303	-3.138
0	741	33.029	-4.548	2.008	-5.907	-4.092
0	703	32.624	-1.686	0.136	-4.803	-4.123
0	528	30.812	-5.502	0.194	-4.206	-3.657
2.168	1176	45.323	-7.366	4.98	-7.58	-2.707
0	946	44.92	-3.984	-1.731	-6.209	-3.107
0	561	19.181	-1.508	-3.311	-4.441	-1.806

Mor07u	Mor08u	Mor09u	Mor10u	Mor11u	Mor12u	Mor13u
2.4	1.344	-0.088	0.027	0.072	-1.316	1.027
2.049	1.625	2.197	-1.225	-1.373	-2.991	0.427
4.265	1.451	-0.457	-0.374	0.215	-0.869	0.117
1.487	0.738	0.138	-0.52	-0.065	-0.486	-0.116
2.532	0.477	-0.931	-0.233	0.412	-0.164	-0.858
-0.558	1.02	0.843	0.844	-0.392	-1.594	-0.014
4.892	0.939	2.018	-1.587	-0.307	-2.486	0.893
2.37	0.306	-1.016	0.218	0.318	-0.195	-0.12
3.116	-0.375	-0.503	-0.652	-0.301	-0.187	-0.439
2.753	1.311	1.385	-1.02	-0.581	-2.623	0.549
4.827	-0.423	3.64	0.625	0.81	-4.421	-2.152
5.951	2.302	0.09	-0.881	-1.252	-1.428	0.346
4.816	1.55	0.683	-1.309	0.099	-2.307	0.221
3.278	2.205	1.214	-2.109	-0.396	-1.406	-0.006
2.708	2.026	0.392	-0.566	-0.162	-1.728	0.062
2.728	1.24	1.474	-0.765	-0.191	-1.788	0.405
2.686	0.74	-0.165	-0.064	-0.375	-0.625	0.114
5.888	0.163	-1.007	-0.578	0.521	-2.226	0.502
3.172	0.824	1.919	-0.719	0.003	-2.836	0.467
4.838	1.104	0.825	-0.378	-0.417	-2.634	1.023
4.348	1.26	0.148	-1.035	-1.715	-0.592	0.358
1.747	0.381	-1.219	-0.141	0.925	-0.044	0.499
3.45	0.359	-0.683	-0.48	0.039	-1.372	0.176
3.734	1.214	0.758	-0.523	-0.264	-1.682	0.545
7.496	1.613	1.81	-1.184	-0.159	-3.252	1.736
4.258	-0.508	1.346	0.764	-1.568	-2.508	-0.145
3.07	1.958	1.585	-0.706	-1.138	-3.317	0.796
7.797	5.276	-1.528	-0.022	-0.444	-3.716	1.881
4.443	1.023	0.396	-1.194	-0.303	-1.384	-0.064
4.246	1.925	2.545	-1.102	-0.522	-3.1	0.621
7.944	0.404	0.396	-2.19	-0.308	-2.473	0.98
6.402	0.824	0.913	1.608	-1.284	-3.874	0.068
3.197	1.057	1.623	-0.733	-0.976	-1.911	0.653
2.493	2.794	1.683	-1.141	-0.849	-1.692	0.798
5.204	1.43	3.324	-1.218	-0.837	-3.925	1.346
7.284	0.637	2.017	-0.706	-0.54	-2.791	1.57
2.763	-0.765	3.727	-0.666	-1.288	-3.129	1.136
2.866	1.091	-0.216	-0.569	-0.756	0.084	0.287
2.591	2.145	4.091	-0.297	-1.89	-4.758	1.784
4.706	0.157	-0.884	-0.031	-0.828	-0.133	-0.291
5.606	0.608	-1.555	-0.223	1.137	-0.881	-0.4
4.177	0.33	-1.257	-0.138	-0.341	-0.367	-0.261
4.138	1.124	5.607	-0.034	-2.849	-4.671	1.449
3.474	0.701	-1.148	-0.007	0.106	-1.264	0.565
5.546	1.772	-0.765	-0.557	0.852	-1.789	-0.04
3.617	0.361	0.536	-0.73	0.268	-1.599	0.276
3.652	1.203	0.989	-0.92	-0.107	-1.645	0.088
3.429	0.733	0.115	-1.611	0.649	-1.178	0.033
1.654	1.266	-0.283	-0.848	-0.266	-0.864	0.344

4.193	0.974	0.72	0.194	-1.669	-2.257	0.926
2.695	0.464	0.573	-0.519	-0.515	-0.736	-0.789
4.318	3.291	1.06	-1.322	-0.711	-1.725	0.679
2.15	1.075	1.191	-0.795	-0.542	-1.128	0.116
2.18	0.949	1.047	-1.424	0.484	-1.551	-0.679
3.498	0.987	1.124	-1.007	-0.057	-1.446	-0.411
6.149	0.288	-0.34	0.017	0.984	-1.75	-0.596
4.735	-0.042	2.263	-1.127	-0.929	-1.894	0.748
2.333	0.432	-0.068	-0.318	-0.444	-0.681	-0.152
2.757	0.614	0.98	-1.166	-0.819	-0.573	-0.099
3.007	1.513	-0.226	-0.27	0.138	-1.602	0.393
4.864	-0.572	-0.966	0.793	-0.089	-0.147	-0.419
3.33	0.294	0.863	-0.773	0.119	-1.288	0.009
6.457	0.716	0.224	-0.856	0.332	-1.233	0.525
2.59	1.113	0.855	-0.909	-0.18	-0.826	0.414
3.606	1.385	1.291	-0.318	-0.403	-1.144	-0.164
5.134	0.314	2.574	-1.198	-1.284	-2.207	0.378
3.516	1.031	1.81	-2.459	0.203	-2.812	0.577
1.038	-0.308	0.075	-0.05	0.455	-0.164	-0.478
2.208	0.859	1.032	-1.359	-0.113	-2.239	0.916
2.546	0.849	0.779	-1.258	-0.176	-1.271	0.32
1.639	0.854	0.825	-0.462	-0.074	-0.877	0.304
1.018	0.603	0.824	-1.566	-0.221	-1.108	0.094
2.912	-0.663	0.216	0.061	0.473	-1.146	-0.275
3.165	0.627	-0.983	-0.139	-0.096	-0.52	0.081
1.573	2.24	0.373	-0.836	-0.235	-1.328	0.436
6.399	0.58	-0.143	-0.934	0.299	-1.069	0.414
5.004	-0.477	0.663	-0.855	0.046	-1.804	0.145
3.021	0.355	-0.079	0.09	-0.793	0.13	-0.176
4.789	-0.554	1.045	0.128	-0.897	-1.344	0.249
3.047	2.142	3.414	0.439	-1.929	-1.965	-0.001
1.922	0.071	0.914	-0.776	-0.261	-1.128	0.563
4.151	1.138	-0.181	-0.337	0.393	-1.1	0.472
2.202	0.717	-0.458	0.592	-0.034	-0.517	0.062
5.914	0.361	0.166	-0.677	0.257	-0.751	-0.457
0.257	0.421	0.862	-0.258	-1.026	-1.219	-0.014
2.532	0.662	0.754	0.235	0.394	-0.447	-0.392
4.732	1.396	0.504	-0.581	-0.549	-1.522	-0.574
4.473	-1.162	1.596	0.607	-1.656	-2.292	-0.161
2.927	-0.227	-0.007	-0.119	-0.109	-0.71	-0.357
3.623	0.259	-0.981	-0.12	0.743	-0.709	-0.319
3.768	1.1	0.307	0.259	0.072	-1.28	0.395
3.716	1.062	-1.456	0.603	-0.184	-0.957	0.044
2.742	0.009	-0.564	0.206	0.527	-0.002	-0.737
3.019	-0.325	3.191	-1.654	-0.968	-3.643	0.685
6.012	-0.249	3.333	-2.192	-1.178	-8.274	1.966
3.756	1.226	1.221	-2.137	0.435	-2.164	0.618
6.046	0.012	0.912	-1.067	0.022	-1.572	0.147
4.661	1.526	2.452	-1.444	-0.949	-1.799	0.925
11.848	0.386	2.519	-0.373	-1.23	-4.032	-0.407

4.91	1.423	1.091	-1.727	-0.107	-2.006	0.332
4.927	0.71	0.04	0.314	0.269	-1.833	0.179
4.051	-0.672	3.157	0.056	-1.121	-0.726	-1.493
2.578	-0.235	0.788	-0.544	-0.864	-0.557	-0.539
1.669	2.107	2.174	-0.368	-1.5	-1.645	-0.27
4.296	1.024	-0.011	-1.005	-0.81	-1.142	0.289
1.859	0.056	0.162	0.408	0.359	-2.983	0.179
1.695	-0.355	1.218	-0.206	-0.582	-0.952	-0.714
2.427	0.283	0.884	-0.162	0.242	-1.037	0.247
5.16	-0.012	-0.323	-0.781	0.198	-1.553	0.486
6.307	1.489	2.044	-1.561	0.27	-3.029	0.802
3.516	0.451	-1.123	-0.355	0.257	-1.031	0.272
1.137	0.854	3.672	-1.247	-1.385	-4.263	2.507
3.366	0.177	1.279	-1.15	0.319	-2.408	-0.136
4.136	0.521	-1.316	0.055	0.624	-0.982	-0.252
4.121	0.588	-1.004	0.542	-0.009	-0.115	-0.384
3.211	0.652	-0.462	0.051	-0.077	0.215	-0.399
2.035	-0.043	-0.406	-0.125	0.221	0.081	-0.1
4.619	0.153	2.39	-1.494	-0.059	-2.365	0.072
3.165	0.805	0.028	0.058	0.644	-1.216	0.28
-0.215	0.267	0.265	0.281	-0.096	-0.334	-0.039
3.717	0.613	2.808	0.703	-2.853	-3.68	1.681
1.978	0.113	0.229	-0.379	0.801	-1.08	0.219
4.83	-0.036	3.469	-1.009	-2.444	-3.997	0.379
0.84	0.921	1.964	-0.86	-0.549	-1.335	0.06
4.612	0.174	1.006	-0.035	0.16	-2.093	-0.249
3.322	1.097	-0.945	-0.167	0.115	-0.727	-0.507
1.64	1.644	2.488	-1.65	-0.545	-2.195	1.405
2.002	1.135	3.533	0.733	0.413	-3.739	-1.397
4.351	2.998	2.692	-1.085	-0.961	-2.721	0.873
4.493	1.558	2.19	-1.408	0.237	-2.814	0.333
4.537	1.07	0.53	-0.436	0.121	-2.276	-0.119
4.664	0.162	0.152	-1.108	0.625	-1.463	0.585
0.927	-0.454	0.069	0.258	0.53	-0.736	-0.359
2.748	-0.35	1.114	-0.133	-0.835	-1.499	0.071
3.163	1.253	0.867	-0.884	-0.5	-1.925	0.783
6.715	1.277	2.729	-1.811	0.74	-2.441	0.735
4.872	0.884	-0.322	0.018	0.513	-1.147	-0.017
4.056	0.608	-0.991	0.144	-0.6	-0.953	-0.083
5.139	1.687	-0.136	0.019	0.035	-2.471	0.783
4.103	0.092	-0.902	0.514	-0.967	-0.405	0.247
3.124	-0.082	-0.38	0.536	-0.911	-0.578	0.101
4.476	1.76	0.904	-1.357	-0.54	-1.058	0.237
3.944	0.637	0.433	-0.038	0.673	-0.798	-0.257
3.384	0.222	-0.076	-0.356	0.176	-1.605	0.635
2.487	0.865	-0.171	-0.482	-0.159	-0.212	-0.326
4.195	0.518	-0.762	-0.535	0.07	-0.942	0.415
5.001	0.158	-0.688	-0.786	0.51	-0.941	0.207
1.616	1.952	-0.11	0.375	-1.339	-0.847	0.23
4.031	0.002	1.08	-1.289	-1.036	-1.454	-0.074

2.423	1.954	1.992	-0.907	-1.095	-1.002	0.181
3.916	1.11	0.3	-0.615	-0.272	-1.374	0.294
3.789	-0.396	0.921	-0.584	-0.7	-0.826	0.163
3.083	-0.11	-1.228	0.195	0.635	-0.292	-0.512
3.958	1.462	-0.503	-0.735	1.338	-1.362	0.534
2.223	0.281	-0.294	0.562	-0.511	-0.284	-0.178
2.649	1.164	1.349	-0.36	-0.623	-1.372	-0.114
2.355	0.567	1.597	-1.674	-0.261	-1.85	0.548
3.917	0.447	0.694	-0.307	0.486	-1.281	0.3
2.931	1.83	0.948	-0.998	-0.43	-1.385	0.631
1.801	0.096	-0.785	0.182	0.255	-0.338	0.1
1.115	1.093	0.794	-0.499	-0.245	-0.524	-0.045
2.908	1.211	3.179	-1.135	-0.822	-2.382	0.922
3.498	1.737	0.94	-0.868	-0.154	-1.292	0.423
5.625	-0.295	1.45	-1.083	-0.213	-0.9	-0.604
3.355	-0.698	2.061	0.699	-1.541	-3.756	0.856
1.558	1.714	2.745	-1.563	-0.192	-1.6	-0.143
3.163	1.91	1.472	-1.024	-0.527	-3.023	0.268
2.089	1.139	-0.415	-0.076	-0.196	-0.527	0.609
4.189	-0.079	-2.058	-0.096	0.075	-0.133	-0.514
6.884	1.645	1.693	0.111	-0.739	-3.401	1.655
3.26	0.91	0.081	-0.483	-0.274	-1.248	-0.667
4.627	-0.761	0.36	1.361	-2.064	-1.66	0.781
3.061	1.769	1.7	-1.937	-1.614	-1.556	1.155
2.582	0.548	2.724	-0.981	-0.771	-2.744	0.545
4.43	1.941	1.005	-0.234	-0.693	-1.781	0.157
2.284	0.077	0.119	-0.618	-0.845	-0.039	-0.072
3.422	-0.274	1.543	0.036	-1.099	-1.853	0.136
3.644	-0.487	2.046	-0.155	-0.548	-1.506	-0.119
2.647	0.583	0.469	-0.985	0.078	-1.505	0.099
3.567	1.139	1.313	-0.799	-1.375	-0.487	0.865
4.058	0.079	1.204	-0.575	-0.293	-2.179	0.138
4.308	0.734	0.564	-1.815	-0.273	-0.714	1.017
3.437	-0.19	-0.167	0.072	-0.428	0.197	-0.225
2.416	1.568	0.11	-1.911	-0.15	-0.923	1.067
3.425	0.475	-1.539	0.807	-0.211	0.405	0.099
1.433	0.57	0.096	0.018	0.318	-0.78	-0.829
3.937	0.027	1.12	-0.711	-0.605	-1.199	0.076
4.464	1.524	0.212	-1.013	0.074	-1.975	-0.153
5.631	1.605	2.22	-0.946	-0.813	-1.781	-0.374
3.96	0.038	0.991	-1.418	-1.096	-1.467	-0.061
3.466	0.971	0.294	-1.637	-0.517	-0.524	0.089
4.946	1.346	-1.123	-0.68	-0.051	-1.234	0.237
4.607	1.534	0.312	-0.371	-0.194	-1.677	0.422
3.025	-0.819	-0.299	-0.064	0.099	-0.464	-0.705
2.441	-0.518	1.906	-0.758	-0.799	-1.865	0.162
4.732	0.725	-1.026	1.076	-0.616	-0.97	-0.331
5.485	-0.512	9.589	-2.616	-2.997	-5.618	4.028
2.4	0.151	2.091	-0.061	-1.287	-0.568	-0.322
2.129	0.769	0.54	-1.293	-0.083	-1.201	0.488

2.357	-0.542	1.908	-1.304	-0.034	-2.722	1.015
3.42	0.166	0.292	-0.158	-0.214	-0.621	-0.175
5.091	0.625	-0.77	0.932	-0.974	-1.283	-0.03
4.565	-1.052	-0.824	-0.453	0.705	-1.18	0.218
2.785	1.053	0.287	0.001	-0.314	-2.568	0.139
3.537	1.121	2.827	-1.225	-0.463	-2.898	0.716
5.838	0.168	-0.166	-1.108	-0.309	-0.697	-0.305
5.355	-0.289	-0.07	0.664	-1.171	-1.276	-0.073
3.067	1.361	-0.682	0.318	-0.729	-1.485	0.692
2.328	1.346	1.322	-0.858	-0.581	-2.514	0.479
4.645	1.635	3.594	-0.293	-1.485	-3.66	0.141
4.419	1.175	0.485	-1.559	-0.153	-1.763	-0.346
3.769	0.011	1.869	0.408	-1.721	-2.054	0.42
3.095	1.91	2.24	0.344	-1.915	-3.721	1.591
2.602	1.097	2.663	-1.093	-0.397	-1.664	-0.173
2.788	0.577	1.806	-1.596	-0.688	-1.14	-0.004
3.48	1.105	1.326	-0.807	-0.549	-2.011	0.073
3.957	0.787	1.49	-0.848	-0.534	-2.235	0.184
1.368	0.9	0.568	-0.195	-0.383	-1.92	0.811
3.956	0.786	1.492	-0.847	-0.533	-2.238	0.184
2.846	0.906	1.408	-0.089	0.004	-0.497	-0.254
4.406	1.466	1.205	-1.254	0.395	-1.87	-0.142
4.733	-0.241	1.538	-0.856	0.196	-2.919	0.402
5.93	0.253	1.243	-0.867	0.209	-2.338	0.232
2.685	2.003	1.132	-1.296	-0.171	-1.435	0.589
6.106	1.43	2.343	-1.396	-1.238	-4.054	1.726
2.103	-0.125	-0.851	0.258	0.34	-0.155	-0.5
1.01	0.463	0.603	0.415	0.278	-1.469	0.387
5.614	1.073	1.032	-0.562	-1.085	-2.143	0.166
4.473	0.896	-0.584	-0.509	0.745	-1.85	0.338
6.087	-1.061	5.963	-1.483	-2.594	-6.84	2.459
2.143	1.774	1.85	-0.892	-0.756	-1.514	0.996
1.749	0.109	-0.09	-0.008	0.161	-0.144	-0.346
5.128	0.892	0.631	-1	-0.164	-3.044	1.478
2.954	2.017	1.506	-0.915	-1.165	-1.516	0.338
1.82	0.692	1.129	-0.533	-0.72	-1.693	0.707
3.384	0.803	1.985	-1.117	-0.62	-1.934	0.852
2.512	0.027	0.351	-0.356	0.406	-0.514	-0.309
4.65	0.175	-0.624	0.106	0.835	-0.753	-0.333
6.391	0.268	-1.572	0.567	-0.603	-1.272	0.034
2.184	0.029	0.374	-0.471	0.041	-0.679	-0.109
5.027	0.934	-0.005	0.401	0.055	-0.783	0.398
1.931	1.211	1.048	0.373	-0.978	-1.84	0.474
3.661	1.188	0.6	-0.696	-0.337	-1.381	0.083
3.194	0.675	0.146	-0.15	0.031	-1.127	0.572
3.451	0.217	0.8	-2.131	-0.384	-1.94	0.471
3.516	1.201	0.351	-0.088	-0.115	-1.624	-0.771
3.3	-0.601	-0.081	0.043	-0.143	-0.603	-0.516
2.206	0.467	-0.178	-0.109	0.199	-1.022	-0.332
3.088	0.029	-0.564	0.238	-0.122	-0.13	-0.791

5.076	1.293	0.335	-1.163	-1.113	-1.928	0.008
0.745	0.913	1.871	-0.935	-0.326	-1.439	0.15
4.498	0.469	2.245	-0.088	-1.681	-2.611	0.661
2.871	-0.592	3.438	-0.197	-1.331	-1.448	-0.928
2.545	1.025	-0.444	-0.244	-0.152	-0.074	0.329
0.811	-0.127	2.076	-0.831	-1.48	-1.257	0.007
2.809	0.303	0.783	-0.381	0.254	-1.323	0.497
1.357	0.743	3.237	-1.833	-0.457	-2.1	0.874
3.903	1.082	1.708	-0.992	0.237	-2.485	-0.49
3.66	0.865	1.283	-0.693	-0.942	-1.274	0.14
1.86	1.394	1.912	-0.568	-0.634	-1.05	0.209
1.401	0.18	3.171	-1.916	-0.552	-2.186	0.659
4.402	0.764	1.517	-1.147	0.508	-1.9	0.548
3.437	0.037	-1.471	-0.271	0.009	-0.244	-0.355
3.862	1.283	1.163	-2.34	-0.108	-1.503	-0.086
4.928	2.455	1.871	-1.323	0.702	-2.417	1.144
0.541	1.043	1.364	-0.224	-1.032	-1.165	0.282
5.03	1.634	1.231	-0.631	0.089	-1.892	0.539
5.755	-0.327	7.287	-2.506	-2.746	-6.985	2.745
3.012	3.473	3.458	-2.468	-1.245	-2.731	0.359
10.037	2.692	1.223	-1.555	-1.831	-3.053	-0.081
5.767	0.308	0.027	-0.727	-1.096	-1.737	0.396
4.266	1.096	-0.638	-0.833	0.313	-0.93	-0.157
4.491	0.218	-0.463	-0.196	-0.201	-0.383	0.314
3.295	0.985	-0.654	0.48	-0.404	-1.036	0.334
4.081	0.493	0.084	-1.172	-0.205	-1.79	0.532
4.611	1.132	0.387	-1.03	0.355	-1.044	0.961
2.618	0.685	-0.589	-0.231	0.139	-1.148	0.977

Mor14u	Mor15u	Mor16u	Mor17u	Mor18u	Mor19u	Mor20u
0.781	0.143	0.386	-0.802	-1.076	0.573	1.524
2.521	0.388	0.133	-1.406	-1.766	1.04	2.831
0.404	0.677	0.371	-0.887	-1.273	0.671	1.715
0.22	0.283	0.246	-0.231	-0.602	-0.118	1.065
0.389	0.906	-0.092	0.377	-1.298	0.072	0.634
0.754	0.416	-0.02	-0.228	-0.91	0.36	0.493
2.778	-0.247	0.859	-1.879	-2.473	1.906	3.696
-0.263	0.331	0.402	0.078	-0.39	-0.249	0.105
0.534	-0.121	0.557	-0.568	-1.643	0.769	2.007
2.077	0.459	-0.093	-1.242	-1.459	0.742	2.627
5.561	2.474	-2.693	0.205	-3.506	0.586	3.59
2.52	0.397	0.996	-1.65	-2.342	1.813	3.49
1.621	-0.043	0.572	-1.323	-1.892	0.937	3.193
1.267	0.346	0.089	-0.455	-2.158	1.3	3.213
1.066	0.52	-0.209	-0.64	-1.304	1.007	1.127
1.393	0.642	-0.176	-0.458	-1.494	1.044	1.253
0.125	0.301	0.541	-0.536	-1.321	0.611	1.254
-0.036	2.018	0.074	-1.558	-2.104	0.819	2.866
1.8	0.528	-0.072	-0.91	-1.203	0.153	2.396
1.981	0.895	0.194	-1.497	-1.048	1.696	1.677
0.966	0.632	0.171	-1.323	-1.532	1.097	2.83
-0.311	0.107	0.721	-0.04	-1.06	-0.091	0.487
0.675	0.602	0.767	-0.475	-1.123	0.52	1.728
0.878	0.529	-0.031	-0.799	-1.173	0.818	1.39
2.464	0.05	-0.595	-2.046	-2.279	1.54	3.871
2.941	0.281	-0.964	-0.668	-2.181	2.178	1.543
2.469	0.661	-0.181	-1.998	-1.391	1.121	2.565
0.212	1.009	1.064	-2.087	-3.289	1.373	3.287
1.365	0.351	0.794	-1.604	-1.959	1.052	2.93
2.726	0.405	-0.016	-1.622	-1.718	0.909	3.789
0.67	2.035	0.203	-1.36	-1.747	-0.051	4.545
3.264	0.514	0.372	-3.948	-1.849	2.343	3.765
1.018	0.478	0.439	-1.61	-1.601	1.228	2.845
2.447	0.229	0.148	-1.698	-2.148	1.565	3.026
2.205	0.85	0.077	-2.956	-3.363	3.974	2.378
2.767	0.332	0.051	-1.907	-2.066	2.15	2.7
3.163	0.076	0.411	-2.272	-1.591	2.295	1.793
0.107	0.106	0.74	-0.412	-0.918	0.346	1.152
4.941	-0.177	0.075	-2.875	-2.216	3.18	3.719
0.136	1.009	0.2	-0.933	-1.485	0.53	1.993
0.834	0.52	1.345	-0.857	-2.256	0.574	2.64
0.038	1.088	-0.282	-0.357	-0.491	-0.592	1.149
6.308	-1.621	1.49	-5.093	-2.071	4.585	3.189
0.109	0.909	0.314	-0.637	-1.03	0.459	1.486
1.358	1.362	0.229	-0.786	-2.277	0.233	3.856
0.803	0.679	0.251	-0.535	-0.899	0.449	1.138
1.047	0.912	0.018	-0.721	-0.799	0.175	1.312
0.522	0.202	0.153	-0.788	-1.55	0.667	1.428
0.288	-0.167	0.392	-0.587	-0.975	0.691	0.749

0.905	0.558	-0.008	-1.149	-1.205	1.445	1.664
0.33	0.686	0.444	-0.472	-0.864	0.383	0.8
1.601	0.985	0.132	-1.493	-2.095	1.383	2.426
0.471	0.177	0.538	-0.587	-1.023	0.528	0.993
0.967	0.61	0.074	0.211	-1.052	0.705	1.091
1.018	0.795	0.042	-0.917	-0.952	0.384	1.381
1.001	0.509	0.039	-1.358	-1.27	0.783	1.986
0.437	0.692	0.583	-0.739	-1.439	0.678	1.588
0.567	0.807	0.41	-0.153	-0.863	0.54	0.704
0.363	0.777	0.795	-0.857	-1.128	0.454	1.299
0.1	0.656	0.449	-0.575	-0.907	0.826	0.954
-0.13	1.067	1.003	-0.778	-1.989	0.004	1.627
0.723	0.837	0.07	-0.593	-1.169	0.429	1.37
0.591	-0.001	-0.19	-0.832	-1.052	1.079	1.169
0.692	0.17	0.115	-0.619	-0.971	0.683	1.185
2.199	-0.002	0.052	-0.931	-1.11	0.839	1.22
4.515	-1.284	0.015	-1.831	-2.198	3.008	2.509
2.258	0.501	0.127	-0.916	-2.138	1.142	3.302
-0.137	0.725	0.169	0.619	-1.007	-0.345	0.773
1.21	0.36	0.332	-1.151	-1.274	0.671	2.319
1.077	0.649	0.367	-0.566	-1.858	0.588	2.78
0.925	0.074	0.357	-0.169	-1.364	0.175	1.217
1.054	0.262	0.067	-0.76	-1.412	0.779	2.71
-0.753	1.184	0.101	0.004	-1.161	-0.247	1.575
-0.256	0.994	0.13	-0.54	-1.167	0.248	1.428
0.263	0.275	0.265	-0.363	-1.509	0.465	1.491
1.18	0.497	0.256	-1.09	-1.305	0.414	2.569
1.061	1.659	-0.141	-0.807	-1.574	0.232	3.473
0.275	1.118	-0.164	-0.145	-1.032	0.374	1.151
0.567	1.429	-0.271	-0.467	-1.085	0.922	1.615
2.831	1.246	-0.874	-1.183	-1.706	1.282	2.447
0.236	0.056	0.316	-0.522	-0.655	0.065	1.537
0.446	0.835	0.373	-0.737	-1.25	0.33	2.402
0.294	0.354	0.242	-0.509	-0.786	0.005	0.96
1.892	0.405	-0.024	-0.505	-2.01	0.71	2.179
1.943	-0.441	0.073	-0.495	-1.072	1.367	1.23
0.208	0.534	0.158	0.403	-1.506	-0.049	1.164
1.321	0.804	0.478	-0.993	-2.175	0.743	3.443
3.499	0.049	-0.5	-0.597	-2.168	2.617	0.981
0.425	0.524	0.238	-0.524	-1.348	0.619	2.099
-0.282	0.769	0.063	-0.069	-1.386	-0.141	1.764
0.668	0.403	0.192	-0.676	-1.116	1.197	1.267
0.226	0.76	0.257	-0.103	-0.889	0.42	0.637
-0.318	1.044	0.099	0.007	-1.01	-0.095	1.249
4.265	-0.661	0.536	-2.322	-2.229	4.304	0.88
7.38	0.226	-0.055	-4.051	-3.303	5.285	4.391
1.942	0.576	-0.038	-1.47	-1.892	0.952	3.652
1.165	0.468	0.091	-1.02	-1.644	0.952	2.806
2.164	0.06	1.315	-1.697	-1.671	1.087	3.263
4.858	0.278	0.522	-2.244	-3.923	3.385	4.414

1.397	0.877	0.46	-1.126	-1.849	0.479	3.359
1.583	-0.031	0.298	-0.771	-1.98	0.978	2.719
1.235	2.082	-0.461	-0.44	-1.383	0.511	1.772
1.09	0.469	-0.294	-0.38	-1.439	0.946	1.697
2.469	0.474	-0.425	-0.838	-1.401	1.08	1.662
0.966	0.632	0.516	-1.251	-1.367	1.298	1.861
1.978	2.197	-0.76	-1.619	-1.879	0.415	3.991
0.588	0.499	0.319	0.48	-1.149	-0.353	0.958
0.372	0.603	0.318	-1.165	-1.045	0.613	1.432
0.253	0.553	0.468	-0.635	-1.023	0.113	1.846
2.565	0.168	0.209	-1.424	-2.012	1.214	3.661
-0.063	0.208	0.509	-0.317	-1.393	0.464	1.847
2.93	0.168	0.508	-3.024	-1.763	3.062	1.838
1.946	0.872	-0.129	-0.771	-1.826	0.598	2.532
0.613	0.042	0.476	-0.074	-1.697	1.02	1.05
0.319	0.408	0.49	-0.328	-0.775	0.373	1.751
0.744	-0.15	0.604	-0.686	-1.681	0.927	1.273
-0.354	0.255	0.055	0.33	-0.214	-0.514	0.373
1.858	1.235	-0.195	-0.794	-2.342	1.184	3.792
0.483	0.46	0.398	-0.64	-1.127	0.216	1.933
-0.117	0.19	0.082	0.186	-0.14	-0.109	-0.164
4.522	0.635	0.034	-3.914	-2.74	2.965	3.904
-0.586	0.893	0.513	0.222	-1.763	0.445	0.97
6.001	-0.566	0.41	-3.323	-4.081	4.236	2.32
2.095	-0.516	0.163	-0.91	-0.905	1.119	1.071
1.765	0.299	-0.026	-1.002	-1.518	1.036	2.646
0.091	1.513	-0.33	0.645	-1.47	0.064	0.76
1.797	0.038	0.582	-1.778	-1.346	1.104	2.622
4.244	1.37	-1.297	-0.798	-2.781	0.974	3.62
3.07	-0.091	0.648	-1.772	-2.531	1.475	3.359
2.132	0.205	-0.102	-1.167	-2.681	1.025	2.891
2.249	-0.557	0.375	-1.173	-2.066	2.438	1.39
0.381	0.95	0.365	-0.736	-1.031	0.368	3.308
-0.054	0.904	0.285	0.406	-1.019	-0.148	0.875
1.907	0.806	-0.037	-0.672	-1.621	1.428	1.79
1.445	0.235	0.409	-1.12	-0.969	0.523	1.976
0.926	2.064	0.3	-1.14	-2.798	0.336	4.245
0.601	0.513	0.273	-0.967	-1.479	0.101	2.485
0.594	0.455	0.744	-1.024	-1.073	0.127	2.117
1.111	0.233	1.373	-1.618	-2.807	1.612	2.883
0.459	0.495	-0.573	-0.267	-0.413	0.697	0.573
0.624	0.444	-0.497	-0.363	-0.519	0.638	0.688
0.259	1.087	0.271	-1.105	-1.759	1.343	2.748
0.377	0.601	0.338	-0.71	-1.341	0.493	1.428
0.883	0.334	0.431	-1.048	-1.073	0.531	1.998
-0.056	0.857	0.123	-0.141	-0.587	-0.038	1.145
-0.043	0.694	0.837	-0.632	-1.627	0.301	1.917
0.202	0.405	0.716	-0.837	-1.224	0.769	1.456
1.292	-0.079	0.188	-0.532	-0.476	0.402	0.998
1.018	1.582	-0.811	-0.612	-1.585	0.721	2.822

1.912	0.318	0.082	-1.21	-2.052	0.774	2.577
0.891	0.591	0.051	-0.855	-1.402	0.954	2.124
0.712	1.483	-0.508	-0.246	-1.698	0.538	2.59
0.117	0.91	0.286	-0.236	-1.126	-0.149	1.669
0.99	0.629	1.571	-0.75	-2.506	0.961	3.229
-0.314	0.524	0.238	0.129	-1.817	0.205	1.779
1.615	0.623	-0.115	-0.583	-1.286	0.411	1.87
2.247	-0.142	0.23	-1.581	-0.853	1.149	2.264
0.842	0.622	-0.119	-0.947	-1.593	0.568	2.909
1.721	0.149	-0.218	-1.031	-2.276	1.102	2.391
-0.127	0.112	0.143	0.22	-0.629	0.11	0.155
-0.044	0.689	0.055	0.165	-0.883	-0.361	0.612
2.445	0.272	0.89	-2.494	-0.934	1.344	3.402
0.766	0.748	-0.09	-0.36	-1.423	0.592	1.936
0.687	1.51	0.62	-0.427	-1.574	-0.473	1.894
2.774	0.149	-0.075	-1.302	-1.834	2.341	1.362
1.701	0.151	0.238	-0.949	-1.836	0.791	2.456
2.386	0.666	-0.248	-1.212	-1.53	0.667	3.097
0.375	0.1	-0.058	-0.027	-1.062	0.711	0.669
0.316	0.325	0.794	-0.577	-1.591	0.461	1.727
1.909	1.923	0.604	-2.359	-2.453	1.539	3.886
1.337	0.943	-0.203	-0.782	-1.091	0.786	1.625
0.712	2.325	-1.054	-0.891	-1.425	1.081	1.801
1.532	0.005	0.395	-1.796	-1.632	1.364	2.591
2.093	0.621	-0.036	-1.715	-1.9	1.248	2.99
1.314	0.684	-0.255	-1.109	-1.517	1.119	2.154
0.225	0.862	-0.134	-0.085	-1.045	0.59	1.164
2.339	1.382	-0.25	-1.311	-1.771	1.184	1.593
1.898	0.689	-0.402	-1.102	-1.446	1.027	2.049
0.65	0.521	0.118	-0.626	-0.944	-0.062	2.03
0.833	0.174	0.523	-0.721	-1.372	0.335	2.229
1.665	0.897	0.389	-1.887	-1.548	1.101	2.885
-0.119	0.812	0.384	-0.247	-1.601	1.008	2.777
0.019	0.803	0.19	-0.453	-0.993	-0.029	1.874
0.76	0.286	0.272	-0.695	-1.591	1.584	2.555
-0.661	0.411	0.525	-0.154	-0.582	0.361	0.06
0.523	0.768	-0.138	-0.056	-0.751	-0.126	0.845
1.043	1.518	-0.264	-0.089	-1.782	0.948	2.09
1.459	-0.09	0.37	-1.203	-1.865	0.621	2.868
1.209	2.24	-0.233	-1.118	-2.551	0.642	4.096
1.027	1.618	-0.788	-0.584	-1.487	0.707	2.844
0.33	0.482	0.418	-0.769	-1.732	0.329	2.249
0.56	0.229	0.851	-1.123	-1.755	1.439	1.316
1.212	0.528	0.423	-1.051	-1.201	1.014	1.069
0.117	0.786	0.049	-0.203	-1.264	0.199	1.625
2.077	0.866	-0.504	-0.644	-1.584	1.208	2.301
1.76	0.779	1.046	-1.587	-1.208	0.537	2.967
6.783	-0.444	-0.13	-3.678	-4.071	5.32	3.703
1.884	-0.74	0.954	-1.171	-0.592	1.173	0.79
0.893	-0.2	0.225	-0.546	-0.985	0.391	1.53

3.168	-0.582	0.324	-1.567	-2.081	2.418	2.336
0.898	0.178	0.527	-0.755	-0.735	0.492	1.067
1.642	0.282	1.002	-1.746	-1.279	0.67	2.939
0.014	0.704	0.445	-0.594	-1.063	-0.268	1.723
1.099	0.54	0.067	-0.973	-1.393	0.656	1.969
2.906	0.894	-0.059	-1.157	-2.648	1.707	4.002
0.662	0.914	-0.121	-0.967	-1.626	0.257	3.209
-0.312	1.775	0.266	-0.423	-1.626	0.298	2.418
0.558	0.373	0.1	-0.765	-0.525	0.688	1.393
1.695	0.383	0.1	-0.971	-1.293	0.413	2.432
4.138	-0.161	-0.32	-1.417	-1.89	2.219	2.41
0.549	1.021	0.19	-0.522	-2.007	0.682	2.154
3.293	0.414	-0.034	-2.153	-0.645	1.497	1.374
3.354	-0.188	0.595	-2.917	-1.667	2.949	1.649
1.864	0.2	0.164	-1.012	-1.791	0.789	2.227
2.035	0.224	0.249	-1.042	-1.695	1.068	2.274
2.564	0.464	-0.177	-1.461	-1.83	0.949	3.529
1.485	0.771	-0.127	-0.809	-1.446	0.304	2.763
1.869	0.166	0.506	-1.157	-0.808	1.035	2.044
1.487	0.768	-0.125	-0.805	-1.447	0.304	2.761
0.184	0.632	0.993	-0.213	-1.231	0.038	1.152
1.965	0.144	0.212	-0.828	-1.899	1.185	3.09
1.645	0.742	-0.509	-0.626	-2.387	1.748	2.142
2.041	0.763	-0.361	-0.634	-2.283	1.722	1.853
0.676	0.349	-0.024	-0.542	-1.768	1.122	2.034
2.845	0.508	-0.062	-2.27	-2.561	2.271	4.013
-0.635	0.665	0.526	0.145	-0.841	-0.191	0.931
0.111	0.74	0.101	-0.266	-0.66	-0.332	0.94
1.41	0.362	0.013	-1.744	-2.269	2.121	2.845
0.655	-0.009	0.247	-1.01	-1.876	1.161	1.698
5.653	0.271	0.036	-2.681	-3.416	4.11	3.901
2.438	-0.086	-0.43	-1.564	-2.152	1.334	3.037
-0.158	0.59	-0.033	-0.048	-0.486	-0.05	0.634
1.993	0.469	0.13	-1.889	-1.083	1.323	1.976
1.877	0.77	0.041	-1.494	-2.443	1.715	3.326
1.267	0.848	-0.129	-1.08	-1.36	0.423	2.272
3.075	-0.574	0.27	-1.391	-2.24	1.518	2.924
0.576	0.138	0.447	0.264	-1.471	0.355	0.833
-0.181	0.914	1.046	0.006	-1.666	0.315	1.505
1.016	0.828	0.66	-1.602	-1.77	0.665	3.241
0.406	0.181	0.521	-0.062	-1.527	0.282	1.177
0.249	1.012	0.115	-1.237	-1.441	0.427	2.265
1.699	0.581	0.015	-0.894	-1.683	0.913	2.127
0.732	0.31	0.114	-1.26	-1.787	0.965	2.024
0.686	0.186	0.474	-0.746	-1.001	0.841	1.183
1.747	0.117	-0.38	-0.725	-1.745	0.692	3.253
1.204	0.222	0.69	-0.434	-1.951	0.584	2.344
0.361	0.674	0.182	-0.431	-1.408	0.524	1.994
0.326	0.342	0.374	0.061	-1.5	0.426	0.788
-0.569	0.92	0.635	-0.102	-1.226	-0.154	1.435

1.015	1.149	-0.149	-1.108	-1.642	0.849	2.61
0.787	1.128	0.081	-1.044	-1.033	0.384	2.146
3.341	-0.782	0.081	-1.799	-1.558	2.569	1.206
1.375	2.425	-0.932	-0.322	-1.666	0.859	1.755
-0.007	-0.105	0.945	-0.265	-0.767	0.028	1.028
1.962	-0.747	0.752	-1.166	-0.788	0.733	1.008
0.965	0.169	0.204	-0.833	-1.254	0.834	1.727
1.672	0.464	-0.063	-1.217	-1.709	0.889	2.923
2.229	0.629	0.189	-0.878	-1.769	0.256	3.089
2.008	-0.292	0.805	-1.008	-1.61	0.887	1.807
1.474	0.142	0.747	-0.728	-1.561	0.554	1.787
2.463	0.3	-0.077	-1.541	-1.7	1.344	2.678
1.423	0.772	0.173	-0.867	-1.804	0.671	3.368
0.413	0.048	0.77	-0.634	-1.454	0.371	1.896
-0.005	1.334	0.566	-0.244	-1.511	-0.337	2.522
1.9	0.594	0.192	-1.517	-2.042	0.995	3.865
1.78	-0.456	0.413	-1.195	-0.713	0.781	1.455
1.616	0.145	0.81	-0.988	-2.779	1.469	2.974
6.769	-1.41	0.068	-3.124	-3.833	5.59	2.818
3.354	0.335	0.869	-2.337	-1.979	1.635	4.759
7.024	1.417	-1.863	-1.77	-4.305	3.421	6.258
2.596	0.426	0.158	-1.725	-2.33	1.812	2.234
0.357	0.743	0.261	-0.928	-1.463	0.918	2.154
-0.397	0.478	0.881	-0.762	-1.442	0.737	1.909
-0.044	0.666	0.271	-0.358	-0.671	-0.059	1.235
1.46	0.549	0.475	-1.12	-1.855	0.525	2.575
0.158	0.282	0.555	-0.906	-1.793	0.947	2.605
0.309	0.277	0.35	-0.826	-1.165	0.79	1.676

Mor21u	Mor22u	Mor23u	Mor24u	Mor25u	Mor26u	Mor27u
-1.288	0.947	-0.704	0.037	0.408	0.385	0.52
-2.148	0.475	-0.632	-0.885	1.165	0.478	0.09
-1.1	0.098	-0.809	-0.405	0.691	0.26	-0.562
-0.306	0.05	-0.242	-0.256	0.414	0.237	-0.432
0.073	-0.283	-0.08	-0.325	0.234	0.256	-0.229
-0.505	0.621	-0.07	-0.262	0.1	-0.112	0.127
-3.193	0.744	-1.355	-0.751	1.589	0.441	0.209
0.279	0.085	-0.18	-0.086	0.002	0.15	-0.1
-0.985	0.47	-1.042	-0.605	1.085	0.519	-0.467
-1.661	0.361	-0.584	-0.971	1.033	0.384	-0.21
-2.331	1.172	0.426	-1.783	1.378	-0.128	0.433
-2.703	0.982	-2.108	0.092	1.116	0.561	0.047
-1.886	-0.201	-0.59	-0.618	1.429	0.696	-0.323
-1.299	-0.287	-0.222	-0.804	0.882	0.524	-0.379
-0.819	0.298	-0.428	-0.325	0.794	-0.135	0.069
-0.995	0.452	-0.543	-0.691	0.825	0.011	0.116
-0.466	-0.061	-0.784	-0.112	0.707	0.53	-0.456
-1.5	0.007	-0.939	-0.425	2.038	0.115	-0.692
-1.53	0.5	-0.366	-0.673	1.12	0.074	-0.1
-1.754	0.558	-0.564	-0.714	1.424	0.411	-0.166
-1.627	0.19	-1.054	0.113	0.741	0.39	0.045
0.177	0.065	-0.412	-0.398	0.335	0.453	0.063
-1.044	0.454	-0.937	-0.319	1.058	-0.035	-0.233
-1.141	0.451	-0.744	-0.273	1.183	-0.111	-0.003
-3.166	1.112	-1.388	-1.114	2.593	0.365	-0.34
-2.085	0.656	-0.496	-0.871	1.157	-0.005	0.566
-1.909	0.547	-0.881	-0.79	1.506	0.498	0.139
-1.856	1.014	-0.987	-0.255	0.613	1.433	-0.137
-1.389	-0.01	-0.98	-0.582	1.168	0.496	-0.503
-2.307	0.151	-0.52	-1.217	1.853	0.198	-0.172
-1.927	-0.229	-1.301	-0.797	1.716	0.248	-0.353
-2.679	0.02	-0.89	-1.52	1.718	0.153	0.376
-1.937	0.914	-1.158	-0.533	1.43	0.186	0.55
-2.623	0.798	-1.069	-0.615	1.364	0.211	0.286
-2.565	0.256	-1.049	-0.63	1.132	0.803	1.131
[truncated: 437,876 more chars]
